# A *COLQ* Missense Mutation in Labrador Retrievers Having Congenital Myasthenic Syndrome

**DOI:** 10.1371/journal.pone.0106425

**Published:** 2014-08-28

**Authors:** Caitlin J. Rinz, Jonathan Levine, Katie M. Minor, Hammon D. Humphries, Renee Lara, Alison N. Starr-Moss, Ling T. Guo, D. Colette Williams, G. Diane Shelton, Leigh Anne Clark

**Affiliations:** 1 Department of Genetics and Biochemistry, College of Agriculture, Forestry, and Life Sciences, Clemson University, Clemson, South Carolina, United States of America; 2 Department of Small Animal Clinical Sciences, College of Veterinary Medicine and Biomedical Sciences, Texas A&M University, College Station, Texas, United States of America; 3 Department of Veterinary and Biomedical Sciences, College of Veterinary Medicine, University of Minnesota, St. Paul, Minnesota, United States of America; 4 Department of Pathology, School of Medicine, University of California San Diego, La Jolla, California, United States of America; 5 Kingdom Animal Hospital, Bryan, Texas, United States of America; 6 R. Prichard Veterinary Medical Teaching Hospital, University of California Davis, Davis, California, United States of America; University of Sydney, Australia

## Abstract

Congenital myasthenic syndromes (CMSs) are heterogeneous neuromuscular disorders characterized by skeletal muscle weakness caused by disruption of signal transmission across the neuromuscular junction (NMJ). CMSs are rarely encountered in veterinary medicine, and causative mutations have only been identified in Old Danish Pointing Dogs and Brahman cattle to date. Herein, we characterize a novel CMS in 2 Labrador Retriever littermates with an early onset of marked generalized muscle weakness. Because the sire and dam share 2 recent common ancestors, CMS is likely the result of recessive alleles inherited identical by descent (IBD). Genome-wide SNP profiles generated from the Illumina HD array for 9 nuclear family members were used to determine genomic inheritance patterns in chromosomal regions encompassing 18 functional candidate genes. SNP haplotypes spanning 3 genes were consistent with autosomal recessive transmission, and microsatellite data showed that only the segment encompassing *COLQ* was inherited IBD. *COLQ* encodes the collagenous tail of acetylcholinesterase, the enzyme responsible for termination of signal transduction in the NMJ. Sequences from *COLQ* revealed a variant in exon 14 (c.1010T>C) that results in the substitution of a conserved amino acid (I337T) within the C-terminal domain. Both affected puppies were homozygous for this variant, and 16 relatives were heterozygous, while 288 unrelated Labrador Retrievers and 112 dogs of other breeds were wild-type. A recent study in which 2 human CMS patients were found to be homozygous for an identical *COLQ* mutation (c.1010T>C; I337T) provides further evidence that this mutation is pathogenic. This report describes the first *COLQ* mutation in canine CMS and demonstrates the utility of SNP profiles from nuclear family members for the identification of private mutations.

## Introduction

Skeletal muscle contraction is stimulated by the emission of the neurotransmitter acetylcholine (ACh) by the motor neuron, and terminated by acetylcholinesterase (AChE) in the neuromuscular junction (NMJ). Disruption of signal transmission within the NMJ resulting from presynaptic, synaptic, or post-synaptic defects causes congenital myasthenic syndromes (CMSs), heterogeneous neuromuscular disorders characterized by skeletal muscle weakness and fatigue. Mutations causing CMSs in humans have been identified in 18 genes to date, with a majority of cases attributed to *CHRNE*, *COLQ*, *RAPSN*, and *DOK7* [1]. Mutations are predominantly autosomal recessive, and often act in compound heterozygosity [2]–[5].

Naturally-occurring CMSs are rarely described in veterinary medicine; when they do occur, they are usually in animals between 6 to 12 weeks of age, appear to be familial, and are characterized by severe generalized skeletal muscle weakness. The first report of canine CMS was in the Jack Russell Terrier in 1974 [6]. Since that time, acetylcholine receptor (AChR) deficiency has been confirmed in Jack Russell Terriers [7], as well as Smooth Fox Terriers [8]. CMS has also been clinically described in families of Springer Spaniels [9], Miniature Smooth-Haired Dachshunds [10], and Old Danish Pointing Dogs [11]. Characterization of CMS at the molecular level has only been achieved in Old Danish Pointing Dogs (missense mutation in *CHAT)* [12], and in young Brahman cows (deletion in *CHRNE)* [13].

Diagnosis of CMS is challenging and relies on clinical evaluation, morphological studies of muscle and peripheral nerves, electrodiagnostic studies, the absence of serum antibodies against muscle AChRs, demonstration of AChR deficiency, and most recently, molecular genetic studies. We have identified a novel canine CMS in a family of Labrador Retrievers. Affected littermates exhibited signs clinically distinct from neuromuscular disorders previously characterized in the breed: exercise-induced collapse (EIC) [14], centronuclear myopathy (CNM) [15], and myotubular myopathy [16].

While genome-wide association studies (GWAS) are an efficient approach for the identification of recessive alleles, they require several unrelated affected individuals [17]–[19]. In the absence of a population suitable for GWAS, we utilized genome-wide SNP profiles from a nuclear family to evaluate inheritance patterns in chromosomal regions harboring all 18 candidate genes. Described herein is the clinical characterization of CMS in a Labrador Retriever family and identification of a missense mutation in *COLQ*.

## Materials and Methods

### Animals

The dogs evaluated in this study were members of a Labrador Retriever family. The dam and sire were clinically normal and both tested clear for the EIC [14] and CNM [15] mutations affecting the Labrador Retriever breed. At 6 weeks of age, 2 female puppies from a litter of 9 were presented to the Texas A&M University (TAMU) Veterinary Teaching Hospital with a 3- to 4-week history of exercise-induced tetraparesis. One puppy was euthanized for progression of clinical signs at 7 weeks of age. Further evaluation including electrophysiology and blood and tissue collection was performed prior to euthanasia of the second puppy. No clinical signs of weakness were observed in 6 littermates (1 puppy died at birth), and a neuromuscular disease had not been previously identified in this family.

### Sample Collection and DNA Isolation

Whole blood was drawn from family members by their primary care veterinarians. Buccal swabs were collected from additional Labrador Retrievers, both related and unrelated to the affected dogs. DNAs from Labrador Retrievers and other breeds previously collected for unrelated studies were also available. Informed owner consent and all samples were obtained in accordance with protocols approved by the Clemson University Institutional Review Board (IBC2008-17). Owner consent was obtained for post-mortem tissue collection from both affected puppies at TAMU. Genomic DNA was isolated using Gentra Puregene Blood Kit (Qiagen).

### Electrophysiology

Electrodiagnostics on the affected second puppy, including electromyography (EMG), measurement of motor nerve conduction velocity (MNCV), and measurement of the decrement in the compound muscle action potential (CMAP) following repetitive nerve stimulation, were performed on the left side under general inhalational anesthesia using a Nicolet Viking Select EMG/evoked potential system (Nicolet, Biomedical Inc.). Insulated stainless steel needle electrodes were used for both nerve stimulation and recording from muscle, while a platinum subdermal electrode (Grass-Telefactor) was employed as a ground. MNCV of the peroneal and ulnar nerves was determined by dividing the distance between proximal and distal stimulation sites by the difference in latency of the corresponding CMAP recorded from the extensor digitorum brevis muscle and palmar interosseous muscles, respectively, after supramaximal stimulation (2 Hz stimulus rate, 0.2 ms stimulus duration). Amplitude (peak to peak) was measured from CMAPs derived from stimulation at the proximal and distal stimulation sites. Repetitive nerve stimulation parameters included stimulation frequencies of 1, 2, 3, 5, 10, 20, or 50 Hz.

### Histopathology, Histochemistry, and Immunohistochemistry

Specimens collected post-mortem from the second puppy included the infraspinatus, extensor carpi radialis, triceps brachii, biceps femoris, quadriceps, and cranial tibial muscles on the right side. The muscles were frozen in isopentane pre-cooled in liquid nitrogen and stored at –80°C until further processing. Light microscopic evaluation of histological and histochemical stains and reactions was performed according to standard protocols [20] and included hematoxylin and eosin, modified Gomori trichrome, periodic acid Schiff, phosphorylase, esterase, myofibrillar ATPase reaction at preincubation pH of 9.8, 4.5, and 4.3, reduced nicotinamide adenine dinucleotide-tetrazolium reductase, succinic dehydrogenase, acid phosphatase, and oil red O.

Specimens from the radial and peroneal nerves were immersion fixed in 2.5% glutaraldehyde in 0.1 M phosphate buffer before shipment to the laboratory. Upon receipt, nerves were post-fixed in 1% aqueous osmium tetroxide for 3 h to 4 h and then dehydrated in a graded alcohol series and propylene oxide. After infiltration with a 1:1 mixture of propylene oxide and araldite resin for 4 h, nerves were placed in 100% araldite resin overnight and then embedded in fresh araldite resin. Thick sections (1 µm) were cut and stained with paraphenylediamine prior to light microscopic evaluation.

For immunohistochemical localization of motor end-plates, serial cryosections (8 µm) were obtained from the external intercostal muscle of 1 affected Labrador Retriever, archived frozen muscle of a previously diagnosed Jack Russell Terrier with CMS due to AChR deficiency (neuromuscular disease control), and a normal dog (wild-type control). Sections from each dog were incubated with the esterase reaction for identification of presumptive motor end-plates or Alexa Fluor 594 α-bungarotoxin (1:1000, Molecular Probes) for localization of AChRs at the motor end-plate. Serial sections were evaluated with light microscopy (esterase reaction) and fluorescent microscopy (red fluorescence) and localization of stainings compared.

### AChR Quantification and Antibody-Bound AChR

AChR was extracted from external intercostal muscle specimens of both affected puppies by a procedure modified from that of Lindstrom and Lambert [21]. The muscle specimens were stored at –70°C prior to homogenization and extraction of AChR in 2% Triton X-100. Solubilized AChRs were labeled by incubation with an excess of ^125^I-α-bungarotoxin (^125^I-αbgt) followed by sequential addition of high titer rat-anti-AChR antibody and precipitation with goat anti-rat IgG. The precipitate was pelleted, washed, and quantitated in a gamma counter. The amount of AChR complexed with ^125^I-αbgt was quantified and expressed in terms of moles of ^125^I-αbgt precipitated per gram of tissue. The concentration of *in-situ* antibody-AChR complexes was determined by precipitation with goat anti-rat serum in the presence of normal rat serum. Quantitative serum AChR antibody concentrations were determined as previously described using an immunoprecipitation radioimmunoassay procedure [22].

### SNP Profiling

**Figure 1:**
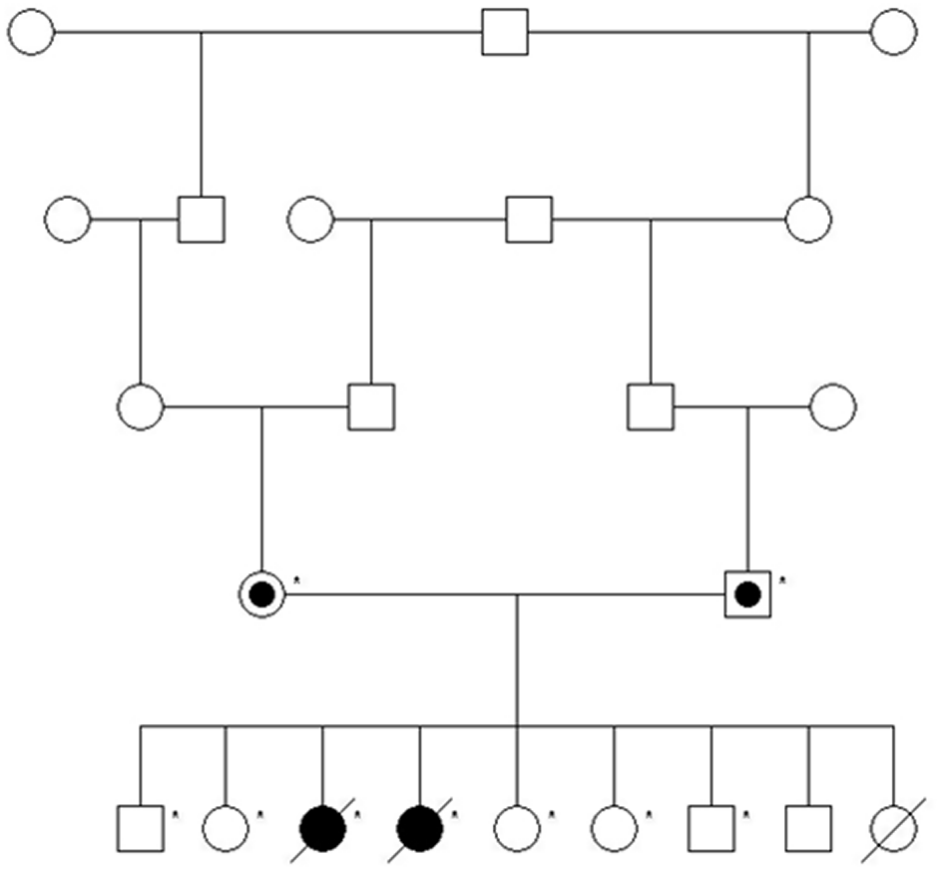
Multigenerational pedigree of Labrador Retrievers. Filled individual icons denote affected dogs and semi-filled icons denote obligate carriers. Asterisks mark individuals selected for whole-genome SNP profiling.

Eighteen candidate genes were selected based on their involvement in human CMSs. Candidate genes were distributed across 13 canine chromosomes: 3 (*DOK7*), 5 (*AGRN*, *CHRNB1*, *CHRNE, DPAGT1*), 6 (*ALG14*), 9 (*SCN4A*), 10 (*GFPT1*), 11 (*ALG2*, *MUSK*), 13 (*PLEC1*), 18 (*RAPSN*), 20 (*LAMB2*), 23 (*COLQ*), 25 (*CHRNG*, *CHRND*), 28 (*CHAT*), and 36 (*CHRNA1*) [1]. The Illumina CanineHD Infinium BeadChip was used to profile 173,662 SNPs representing all chromosomes for 9 nuclear family members: 7 littermates and both parents (Figure 1). Arrays were processed according to manufacturer’s protocols. Allele frequencies were calculated using a case/control analysis conducted with PLINK [23]. Chromosomal regions harboring candidate genes were evaluated for SNP haplotypes with frequencies of 1.0 in the CMS cases and between 0.14 and 0.50 in the controls. Individual genotypes in regions fitting these parameters were then manually examined to ensure that no unaffected littermates were homozygous for the allele present in the affected dogs.

### Microsatellite Analysis

PCR for amplification of individual microsatellite markers was performed using materials and thermal cycling parameters previously described [24], [25]. Products were resolved with GeneScan 600 LIZ size standard (Applied Biosystems) on an ABI 3730XL DNA Analyzer (Applied Biosystems). GeneMapper Software version 4.0 (Applied Biosystems) was used to visualize the microsatellite signals.

### Sanger Sequencing

Primers were designed to amplify all coding regions and splice sites of *CHRNG*, *CHRND*, and *COLQ*. Products were amplified using ReddyMix Master Mix (Thermo Scientific) with 0.4 µM of each primer and 50 ng of DNA. Products were purified using the Qiax II Gel Extraction Kit (Qiagen) or an ExoSAP master mix consisting of 0.5 units of Exonuclease I (New England BioLabs) and 0.25 units of SAP (Promega). Primer sequences and purification methods for each amplicon are given in Table S1. Sequencing products were resolved on an ABI 3730XL DNA Analyzer (Applied Biosystems).

### Restriction Enzyme Digest

Detection of the *COLQ* variant was accomplished using the endonuclease BtsI, which recognizes the following sequence and cutsite (∧): 5′ …NN∧CACTGC…3′. The digestion was performed using BtsI CutSmart (New England BioLabs) with 2.5 µL 10X buffer, 0.40 µL BtsI, 1 µg DNA, and water adjusted accordingly for a total reaction volume of 25 µL. Products were resolved on a 2% agarose gel.

## Results

### Clinical Description

Neurological examination was consistent with a generalized neuromuscular disease with marked short-strided tetraparesis that worsened with exercise. Postural reactions were preserved with the exception of hopping which was diminished in all limbs when the puppies were made to bear full weight. Spinal reflexes including the patellar, cranial tibial, and flexor withdrawals were reduced in all limbs. A pyridostigmine bromide challenge resulted in worsening of muscle weakness.

### Electrophysiology

**Figure 2:**
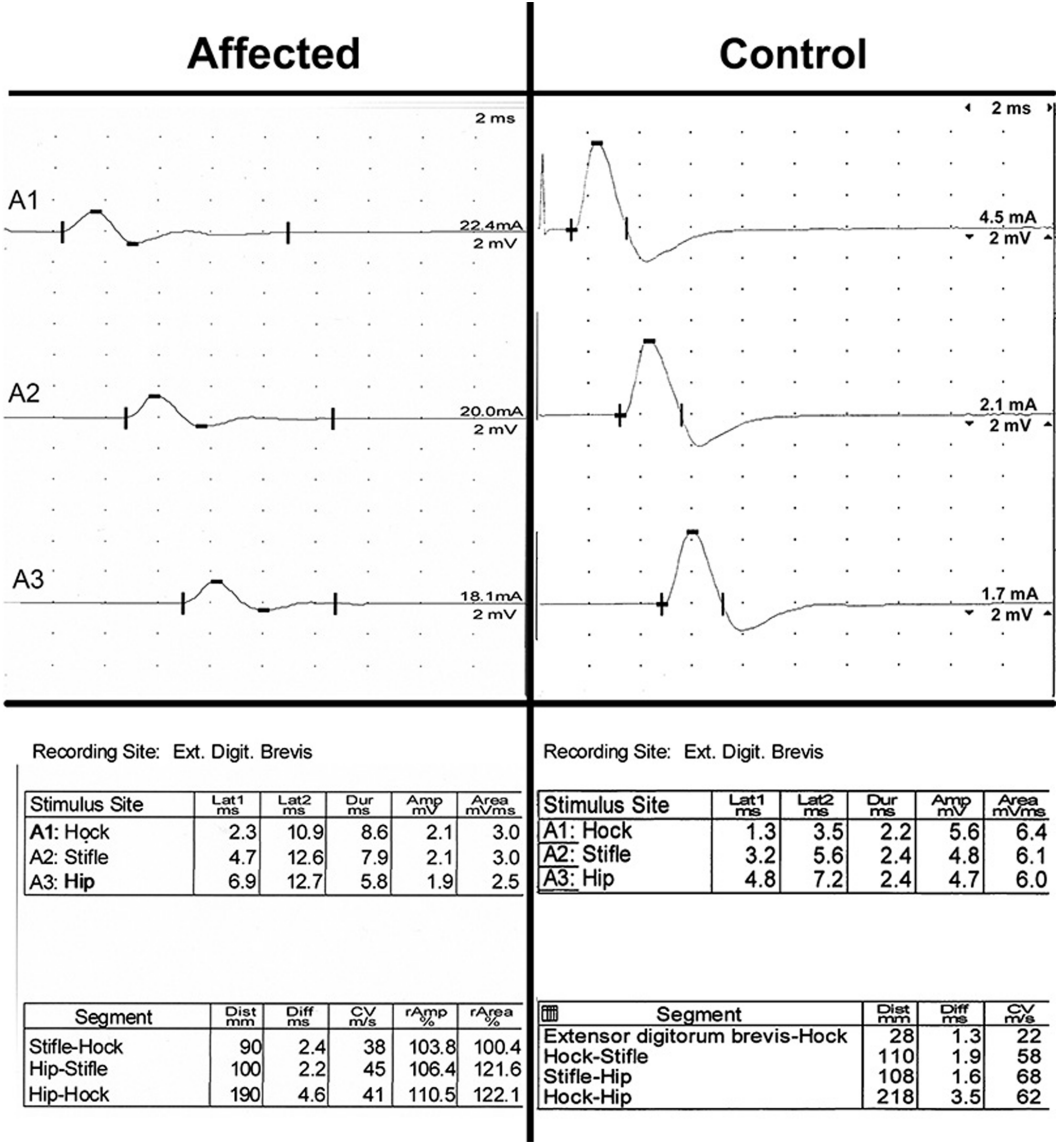
Peroneal MNCV of an affected Labrador Retriever recorded at the extensor digitorum brevis muscle with stimulation at the level of the hock, stifle, and hip. Although the CV was normal considering the dog’s age, the amplitude of the CMAP was uniformly diminished. The timebase between vertical columns on the x-axis is 2 msec and the voltage measured between adjacent rows on the y-axis is 2 mV. Control tracings are from the peroneal nerve of a healthy 5 month old Beagle. Note: labeling varies with the control amplitudes measured from baseline to peak, peak-to-peak amplitudes are within normal limits (≥8 mV). Latency 2 markings also vary.

**Figure 3:**
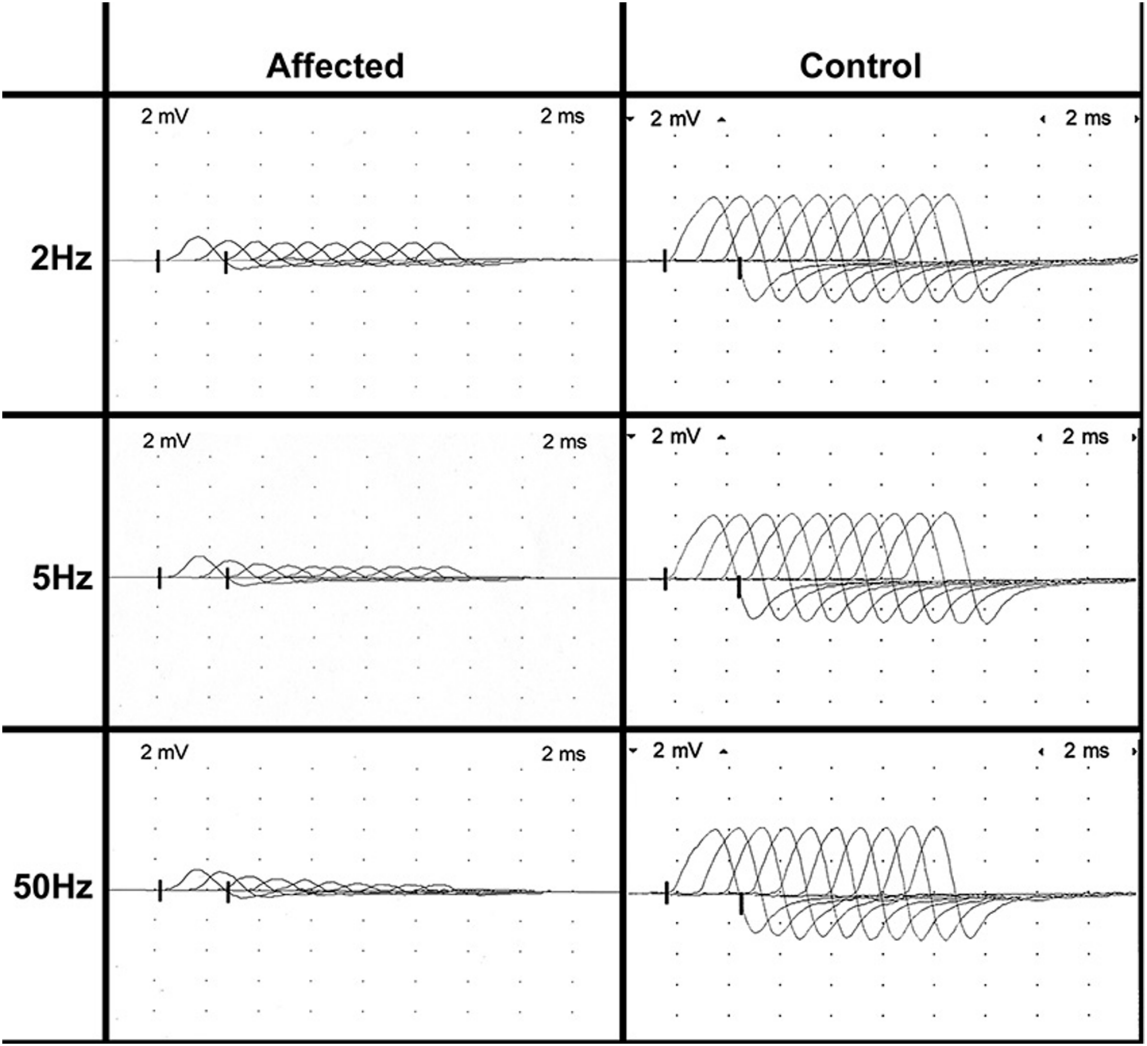
Repetitive stimulation of the peroneal motor nerve of an affected Labrador Retriever at 2 Hz (A), 5 Hz (B), and 50 Hz (C). Decrement of the CMAP was observed at all tested frequencies. Sweep speed and sensitivity settings are identical to those in Figure 2. Control tracings are from the peroneal nerve of a healthy 5 month old Beagle with no decrement seen at low frequency stimulation and normal pseudofaciliation (CMAP gets taller and narrower) with tetanic stimulation.

EMG was performed on the left side of the body and included the supraspinatus, infraspinatus, triceps, biceps, extensor carpi radialis, superficial and deep digital flexor, interosseous, biceps femoris, rectus femoris, cranial tibial, and gastrocnemius muscles. Abnormal EMG activity was not identified in any muscle group. Peroneal and ulnar MNCV was likely age appropriate (41 m/sec and 31 m/sec, respectively; reported values for normal dogs 1 to 6 months of age 32 m/sec to 55 m/sec and 25 m/sec to 48 m/sec) [26], [27]. The peroneal and ulnar nerve CMAP amplitudes were reduced at all stimulus sites (1.9 mV to 2.1 mV and 2.6 mV to 2.9 mV, respectively, reference ≥8 mV, peak-to-peak) (Figure 2) [27]. F-waves were not recordable upon stimulation of either the peroneal or ulnar nerve. Repetitive stimulation of the peroneal nerve revealed a decrement in the CMAP amplitude of 22% at 2 Hz, 33% at 5 Hz, and 35% at 50 Hz when the first and third CMAPs were compared (Figure 3). Decrements greater than 10% are considered significant.

### Histopathology, Histochemistry, and Immunohistochemistry

**Figure 4:**
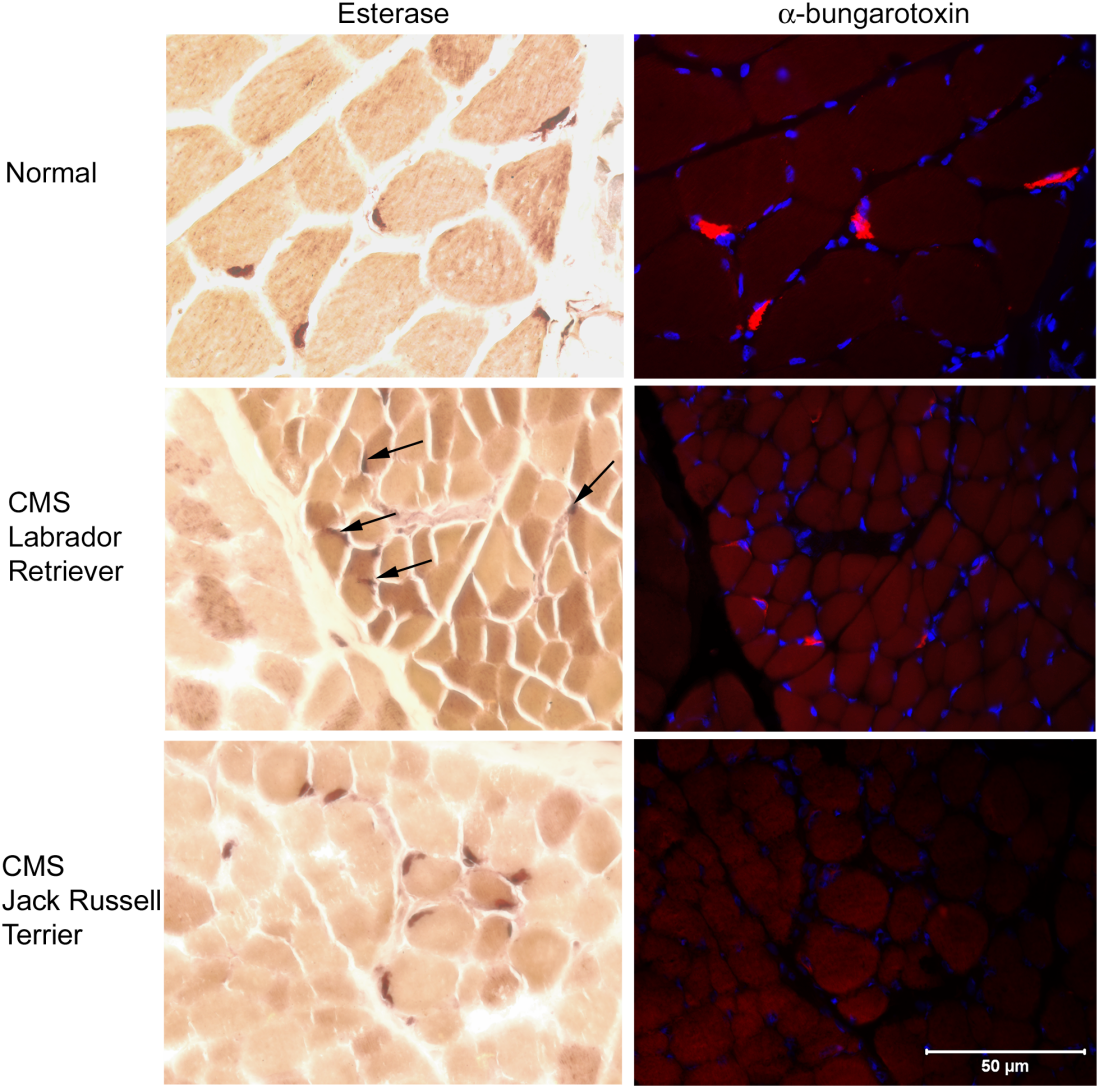
Cryosections (8 µm) of intercostal muscle from a normal dog, a Labrador Retriever with CMS (end-plate AChE deficiency), and a Jack Russell Terrier with CMS due to AChR deficiency (neuromuscular disease control) are illustrated. For each dog, histochemical staining for esterase activity (brown stain) is shown along with a serial section demonstrating immunofluorescent localization of α-bungarotoxin for AChR and end-plate localization (red color). Muscle nuclei are blue (Dapi stain). There is a good correlation between esterase staining (brown) and α-bungarotoxin localization (red) in the control dog muscle. Although esterase staining is present in the Labrador Retriever muscle (arrows), the localization correlates poorly with that of AChRs. In the CMS Jack Russell Terrier esterase staining was present; however, staining for AChR was markedly decreased or absent, consistent with a markedly decreased AChR content. Bar = 50 µm for all images.

Myofiber size was appropriate in all muscles evaluated and no specific abnormalities were identified, making a congenital myopathy unlikely. Multifocal areas of esterase reactivity were identified, but it could not be determined from this reaction if staining correlated with localization of motor end-plates. In the Labrador Retriever with CMS, esterase staining was evident in multiple locations but did not fully correlate with AChR labeling (Figure 4). There was a good correlation with esterase staining and AChR labeling in the normal dog. In the Jack Russell Terrier with CMS (neuromuscular disease control), several end-plates were stained with esterase; however, no AChRs were labeled in the serial muscle section, consistent with the marked decrease in muscle AChRs described in this breed. There was no evidence of axonal degeneration, demyelination, or abnormalities of supporting structures in the peripheral nerve evaluations, excluding a congenital polyneuropathy.

### AChR Quantification and Antibody-Bound AChR

The concentration of AChR was determined from external intercostal muscle samples collected origin to insertion following euthanasia of both affected Labrador Retriever puppies. The AChR concentration was decreased in both puppies (0.07 pmol/gm and 0.10 pmol/gm tissue, reference 0.2 pmol/gm to 0.4 pmol/gm). AChR antibodies were not detected bound to muscle AChRs or in the serum.

### Analysis of Family Genomic Inheritance Patterns

Over 172,000 SNPs across 40 canine chromosomes were genotyped for each of 9 nuclear family members. Allele frequencies in regions on the 13 chromosomes harboring candidate genes were evaluated for a pattern consistent with a recessive trait (Table S2). SNP haplotypes on both chromosomes 23 and 25 were homozygous in the cases and had frequencies of 0.36 and 0.29, respectively, in the unaffected individuals. Chromosome 23 harbors *COLQ*, while the region on chromosome 25 includes both *CHRNG* and *CHRND*.

**Figure 5:**
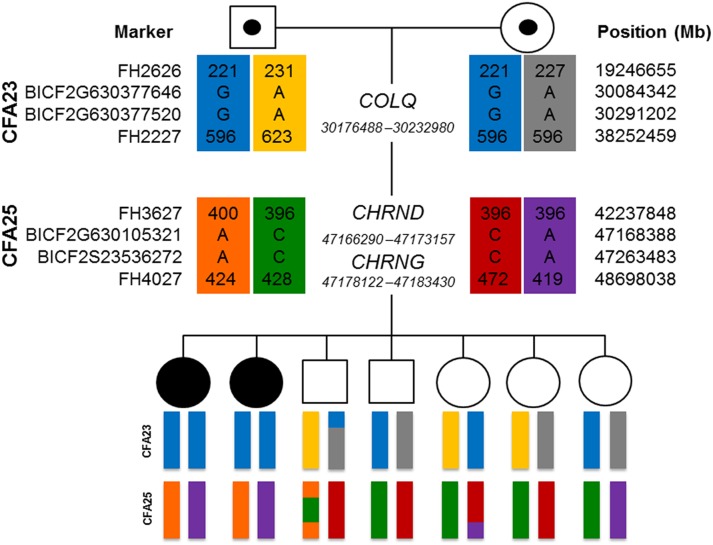
Microsatellite and SNP haplotypes (color-coded bars below individuals) are shown for 3 candidate genes. Positions (in Mb) are according to CanFam 2. Filled individual icons denote affected dogs and semi-filled icons denote obligate carriers. Chromosome 23 haplotypes (blue) are inherited IBD in both affected dogs.

Because the sire and dam share 2 recent common ancestors, we hypothesized that the causative mutation was inherited identical by descent (IBD). To determine whether the aforementioned segments of chromosomes 23 and 25 were inherited IBD, we genotyped polymorphic microsatellite markers in each region. Genotypes revealed homozygosity in the affected dogs for markers flanking *COLQ,* providing evidence that the segment on chromosome 23 is IBD (Figure 5). Affected dogs were heterozygous for haplotypes encompassing *CHRNG* and *CHRND,* suggesting that the chromosome 25 segment was not inherited from a recent common ancestor.

### Sequencing of CHRNG, CHRND, and COLQ

**Figure 6:**
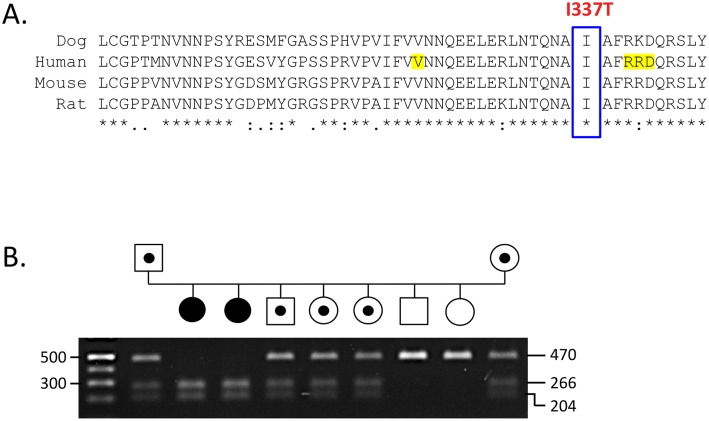
(A) Sequence from the 5′ end of the C-terminal domain of ColQ in mammals. Identical residues are denoted by an asterisk, conserved substitutions by a colon, and semi-conserved substitutions by a period. Residues altered in human CMS cases are highlighted in yellow [4], [30], [32], [37]. (B) BtsI digest results for the Labrador Retriever family. PCR amplicons from *COLQ* exon 14 are 470 bp in size and cleaved into 204 and 266 bp fragments in the presence of c.1010T>C. Three clinically normal littermates were identified as carriers, denoted by semi-filled icons.

No nonsynonymous variants were identified in *CHRNG*. A single nonsynonymous variant in exon 4 of *CHRND* did not segregate with a recessive phenotype. Sequence data for *COLQ* revealed 3 nonsynonymous variants, only 1 of which segregated with a recessive trait. The exon 14 variant, c.1010T>C, predicts the substitution of isoleucine with threonine at residue 337 (Figure 6A).

### Screening of c.1010T>C Variant

PCR amplicons from the *COLQ* exon 14 primer set are 470 bp in size. BtsI cleaves the amplicon only in individuals having the c.1010T>C allele, yielding fragments of 204 bp and 266 bp. Heterozygotes for the variant have all 3 fragments sizes (Figure 6B).

Digestion with Bts1 was used to genotype the variant for 49 additional members of this Labrador Retriever family, 288 unrelated Labrador Retrievers, and 112 dogs representing 65 other breeds (Table S3). Of the 58 family members, 40 were homozygous wild-type, 16 were heterozygous, and only the 2 affected dogs described herein were homozygous for the variant. The variant was not present in any unrelated Labrador Retrievers or dogs from other breeds.

## Discussion

The probands in this study presented with an early onset neuromuscular disorder characterized by severe exercise-induced weakness. The lack of specific morphological changes in muscle and peripheral nerve biopsies excluded an underlying congenital myopathy or neuropathy. Electrodiagnostic findings and decreased AChR concentration in the muscle indicated a disorder of neuromuscular transmission. The autoimmune disease myasthenia gravis was eliminated based on the early age of onset and an absence of AChR antibodies in serum and AChR-bound antibodies in the muscle. The clinical diagnosis in the Labrador Retrievers was CMS.

While clinical signs and electrophysiological findings are generally similar between presynaptic, synaptic, and postsynaptic forms of CMS, a notable observation in the affected puppies was a worsening of the phenotype upon administration of an AChE inhibitor. This response indicates desensitization of the AChRs from overexposure to ACh and is consistent with a synaptic form of CMS referred to as end-plate AChE deficiency (EAD) [5]. EAD accounts for 10% to 15% of all human cases of CMSs and is always caused by mutations in *COLQ* [5]. *COLQ* encodes a collagen strand that homotrimerizes to form the tail subunit of asymmetric AChE. ColQ anchors AChE to the basal lamina where the enzyme hydrolyzes ACh, thereby limiting the length of the synaptic response [28]. In the absence of ColQ, ACh accumulates, causing prolonged muscle contraction and eventually the desensitization of AChR [29].

Through the examination of SNP allele frequencies in the Labrador Retriever family, we identified 2 chromosomes harboring CMS candidate genes that showed an inheritance pattern consistent with autosomal recessive transmission. Whereas human forms of CMS are often caused by compound heterozygosity, low levels of genetic diversity within purebred dog populations make simple recessive alleles more common. Linebreeding in this Labrador Retriever family makes it likely that the sire and dam inherited the mutation from a common ancestor and that the affected puppies are homozygous for a chromosome segment transmitted IBD. Analysis of polymorphic microsatellites showed that the regions flanking *COLQ* are IBD, whereas those flanking the other 2 identified candidate genes are not.

Sequencing of *COLQ* in the Labrador Retrievers revealed a missense mutation that predicts the replacement of a conserved hydrophobic isoleucine with a hydrophilic threonine in the C-terminal domain. ColQ has 3 domains: an N-terminal proline-rich attachment domain (PRAD), a collagenic central domain, and a C-terminal domain. The PRAD serves to attach the ColQ strand to an AChE tetramer. The collagen domain assembles the triple helix, while the C-terminal domain is involved in both the formation of the triple helix [30] and anchoring of the structure to the basal lamina [30], [31].

In humans, mutations responsible for EAD have been identified in each domain of *COLQ* and have different functional consequences depending on their location [4]. In the C-terminus, missense mutations in residues ranging from positions 342 to 452 are thought to inhibit the attachment of ColQ to the basal lamina of the muscle cell [30]–[33]. Some C-terminus mutations (e.g., V322D) may prevent the formation of the ColQ triple helix [32]. In the affected Labrador Retrievers, localization of the esterase reaction showed a poor correlation between AChE and AChR. This finding suggests improper anchoring of ColQ to the basal lamina, or mislocalization. Insufficient muscle samples prevented us from conducting a sedimentation profile of AChE to determine the exact consequence of the I337T mutation identified.

Linebreeding practices expedite the appearance of recessive diseases in purebred dog populations. The availability of genetic tests for the detection of carrier dogs allows for selective breeding to prevent widespread dissemination of the deleterious allele to the breed while maintaining genetic diversity. Because only 2 affected littermates were available for study herein, GWAS techniques could not be applied. The analysis of chromosomal inheritance patterns indicated a single functional and positional candidate gene and led to the discovery of the *COLQ* c.1010T>C mutation; however, our approach does not exclude the possibility that another mutation exists in a novel CMS gene.

While this manuscript was in revision, Matlik et al. reported that an identical mutation (c.1010T>C; I337T) was homozygous in 2 human CMS patients with EAD [34]. The affected children were first cousins from consanguineous relationships; both sets of parents were heterozygous for the mutation [34]. The substitution was the only variation identified in *COLQ* and was determined to be pathogenic through a prediction program [34]. Although uncommon, identical changes at the DNA level between humans and dogs with similar phenotypes have been previously identified [35], [36]. The identification of c.1010T>C in humans and dogs diagnosed with CMS strongly supports the causality of the mutation and shows that conservation of residue 337 is critical for proper function of ColQ.

## Supporting Information

Table S1 Primers (5′-3′) for amplification of *CHRNG, CHRND*, and *COLQ.* Primers were designed to amplify exons and splice sites. ExoSAP indicates the use of the ExoSAP protocol and Gel X indicates the use of the gel extraction protocol for post-PCR clean-up.
(PDF)

Table S2 Candidate regions based on allele frequencies. Genomic regions known to harbor CMS candidate genes were screened for case allele frequencies of 1.0 and control allele frequencies of between 0.14 and 0.50. CHR = chromosome number; SNP = SNP name; BP = chromosome position; A1 =  allele 1; F_A = frequency of allele 1 in affected dogs (cases); F_U = frequency of allele 1 in unaffected dogs (controls); A2 =  allele 2.
(PDF)

Table S3 Dogs screened for the *COLQ* 14 variant. Digestion with Bts1 was used to genotype the 2 affected dogs, 56 other members of the Labrador Retriever pedigree, 288 unrelated Labrador Retrievers, and 112 dogs representing 65 other breeds.
(PDF)
