# Supplementary material for: A COLQ Missense Mutation in Labrador Retrievers Having Congenital Myasthenic Syndrome
Source: PLoS One. 2014 Aug 28;9(8):e106425. doi: 10.1371/journal.pone.0106425 (PMC4148433; doi:10.1371/journal.pone.0106425)
Supplement: Table S1 — Primers (5′-3′) for amplification of CHRNG, CHRND, and COLQ. Primers were designed to amplify exons and splice sites. ExoSAP indicates the use of the ExoSAP protocol and Gel X indicates the use of the gel extraction protocol for post-PCR clean-up. (PDF) [file pone.0106425.s001.pdf]

Table S1. Primers (5'-3') for amplification of *CHRNG, CHRND*, and *COLQ*

Gene	Exon(s)	Forward Primer Sequence (5′-3′)	Reverse Primer Sequence (5′-3′)	Cleanup Protocol
CHRNG	1	CCTGGCATTTTGTCCATCTT	TCAGGGGACAGAGGGTCT	ExoSAP
	2	GTTGTCCCACCCGTCACT	AAGAAGGGGCTGGCTCTA	ExoSAP
	3	GACAGTGGGGGACACAGAG	GTGCTAAGGGTGCCGTCC	ExoSAP
	4	CCCTCCCTGCCTCTTCTG	GGAAAAGGGACAAGTGGACA	ExoSAP
	5	GCAGGTGGGGAGTAGCG	TCCTTCCCTGCCTCAGTTTC	ExoSAP
	6	GGCAGCAGAGGGGAGGAA	CCTCCCTGTGCCCACCCT	Gel X
	7	GTCAGGCATCCAGTCTCC	CACACACGAGACCACCTG	Gel X
	8	GGGCATCACACACGAGAC	CTACCCTCCTGCCACTCC	ExoSAP
	9	CACTACCCTCCTGCCACTC	TGTGGCAGGTGTATTATGC	ExoSAP
	10	CAACCTTCACCTTCTGCTCT	CAGGTTGAGGAGAGAGAAGC	ExoSAP
	11	CTTCTTCTCCACTCTATCCCCA	TGAGAAGGACCGAAGGAC	ExoSAP
	12	GAGAAGGACCGAAGGACAC	TGAGTGTGCTCTTTGGGAA	ExoSAP
CHRND	1	TACTGGTCGTGTGTGGTAAGAG	CTTCCCCTCCAGCCTCAGTT	ExoSAP
	2	TTCCCCTCCAGCCTCAGT	CTGGTAGATTGGGATTCTTCATC	ExoSAP
	3&4	ATAGGCTCCCTTGGTTGTG	CTGAAGCAGGAGGAAGAAG	ExoSAP
	5	CCCCTGCTCTGTCCTGTTTAG	GGACATACCTACCGACACAAATCT	ExoSAP
	6	GGAGACACCCACAAACAGAA	CCTCTCCTCACTTCCTCCTG	Gel X
	7&8	ACTCACTCTCTCACTTGGTCCTCA	CCAGGCACAAATGAGCAGAAG	ExoSAP
	9	AATAGGGGAGAGTTGGAAGCA	GACCAGAACGATTACAACGAGG	ExoSAP
	10	CTGTAAAATAGCCTCCTTGTGTCC	GGAGACACACACCCACTTAGATG	ExoSAP
	11	TTACCCTCCTTAGACCCCT	GAGAGGAAGGGAGAAACC	ExoSAP
COLQ	1	GGGAAAGGAGAGAGAGAGACA	TTCACCATTCTGCTCTCCG	Gel X
	2	CTGGGGGAAGGTTACATCAAG	TGTGTCTGTTCTTCCACTGTC	Gel X
	3	TCCATTCAAGCCAAGTATGTCTC	GCTGCTGCCTAGATGGGTG	Gel X
	4&5	AGTCCTGCCTTCCTCACCA	GTTGGGGGAGGGATGTGTAT	Gel X
	6	TGAACCCCCAAAACAAACC	CTTTCTCCTTCGGTTGGACTAC	Gel X
	7	TGTCCCTGGTTCATCCTAATCT	ATTGAAAGCCCAGTGCCG	Gel X
	8	TTACAGCCCCCACTAAAACTTG	CAATGGTGAGCAGAAGACA	Gel X
	9&10	TGGGCAAGTCCCTAATCTCTG	TTGATGAGAGGGTTCCAGCA	Gel X
	11	CAGTTGGAAAAAGTCTGGAATGAT	GTGTAGGTGGGTGGTGTAGA	Gel X
	12	GGCTCCTGCTCCAAAGTG	GGAGAGGGCACAGGAGATG	Gel X
	13	CGTGGACTTTGACCTTCTTTCT	CGCAAAATCAGCCATCCAAC	Gel X
	14	CTTCAGGAGGGATGACAGAGTG	GAACCTCCTCTTTGAGCC	Gel X
	15	AGAAGGTAGCCCCCACAGAC	CCTGTCCCTCCCTTTTCCT	Gel X
	16	GGCAGTCCCTGGATACAT	TTTCTTAGTGCTCCTTATGTTCC	Gel X
	17a	AGTGGCTGGTGTCTGATG	CCAAATAGCCAACCTTCAGT	Gel X
	17b	GTCCAGTTTCTACCACCTTCATA	TGGGTAGGCTTTTGGTGTTC	Gel X
