# Supplementary material for: A COLQ Missense Mutation in Labrador Retrievers Having Congenital Myasthenic Syndrome
Source: PLoS One. 2014 Aug 28;9(8):e106425. doi: 10.1371/journal.pone.0106425 (PMC4148433; doi:10.1371/journal.pone.0106425)
Supplement: Table S3 — Dogs screened for the COLQ 14 variant. Digestion with Bts1 was used to genotype the 2 affected dogs, 56 other members of the Labrador Retriever pedigree, 288 unrelated Labrador Retrievers, and 112 dogs representing 65 other breeds. (PDF) [file pone.0106425.s003.pdf]

Table S3. Dogs screened for the *COLQ* 14 Variant

Breed	Sample Number (*n*)	*COLQ* Exon 14 Variant Genotype		
		T/T	T/C	C/C
Labrador Retriever (related)	58	40	16	2
Labrador Retriever (unrelated)	288	288		
Afghan Hound	1	1		
Airedale Terrier	2	2		
Akita	3	3		
American Eskimo Dog	2	2		
Australian Shepherd	2	2		
Basenji	2	2		
Bichon Frise	1	1		
Blue Tick Hound	1	1		
Border Collie	2	2		
Boston Terrier	2	2		
Boxer	2	2		
Briard	2	2		
Bull Terrier	1	1		
Cairn Terrier	2	2		
Cardigan Welsh Corgi	2	2		
Catahoula Leopard Dog	2	2		
Cavalier King Charles Spaniel	2	2		
Chihuahua	2	2		
Chinese Shar-Pei	1	1		
Chow Chow	1	1		
Cocker Spaniel	2	2		
Collie	3	3		
Dachshund	2	2		
Doberman Pinscher	1	1		
English Bulldog	2	2		
English Setter	2	2		
English Springer Spaniel	2	2		
Flat-Coated Retriever	1	1		
German Shepherd Dog	2	2		
Golden Retriever	3	3		
Great Dane	2	2		
Havanese	1	1		
Irish Setter	2	2		
Lhasa Apso	1	1		
Maltese	1	1		
Miniature Poodle	2	2		
Miniature Schnauzer	2	2		
New Guinea Singing Dog	2	2		
Newfoundland	2	2		
Norwegian Elkhound	2	2		
Papillon	1	1		

Pekingese	1	1		
Pembroke Welsh Corgi	2	2		
Petit Basset Griffon Vendéen	2	2		
Podengo	1	1		
Polish Lowland Sheepdog	1	1		
Portuguese Water Dog	1	1		
Pyrenean Shepherd	2	2		
Rhodesian Ridgeback	2	2		
Rottweiler	1	1		
Saint Bernard	2	2		
Shetland Sheepdog	2	2		
Shih Tzu	1	1		
Shiloh Shepherd	1	1		
Siberian Husky	2	2		
Silken Windhound	2	2		
Silky Terrier	1	1		
Swedish Vallhund	2	2		
Tibetan Terrier	2	2		
Vizsla	1	1		
Weimaraner	2	2		
Welsh Fox Terrier	2	2		
West Highland White Terrier	2	2		
Wire Fox Terrier	2	2		
Yorkshire Terrier	2	2		
