# Supplementary material for: A COLQ Missense Mutation in Labrador Retrievers Having Congenital Myasthenic Syndrome
Source: PLoS One. 2014 Aug 28;9(8):e106425. doi: 10.1371/journal.pone.0106425 (PMC4148433; doi:10.1371/journal.pone.0106425)
Supplement: Table S2 — Candidate regions based on allele frequencies. Genomic regions known to harbor CMS candidate genes were screened for case allele frequencies of 1.0 and control allele frequencies of between 0.14 and 0.50. CHR = chromosome number; SNP = SNP name; BP = chromosome position; A1 = allele 1; F_A = frequency of allele 1 in affected dogs (cases); F_U = frequency of allele 1 in unaffected dogs (controls); A2 = allele 2. (PDF) [file pone.0106425.s002.pdf]

Table S2. Candidate regions based on allele frequencies.

CHR	SNP	BP	A1	F_A	F_U	A2
3	BICF2P187550	3039065	0	0	0	C
3	BICF2P686633	3069417	A	0	0	G
3	BICF2P1191190	3092524	G	0	0	A
3	BICF2P970802	3093508	G	0	0	A
3	BICF2P446597	3114178	G	0	0	A
3	BICF2P292856	3117110	0	0	0	C
3	TIGRP2P36343_rs8854696	3130036	0	0	0	A
3	BICF2P769773	3148115	A	0	0	G
3	BICF2P892164	3153404	0	0	0	A
3	BICF2P273232	3168517	G	0	0	A
3	BICF2P979854	3193623	A	0	0	G
3	BICF2P957704	3198320	C	0	0	A
3	BICF2P957980	3263471	A	0	0	G
3	BICF2P1473165	3273029	G	0	0	A
3	BICF2S22919855	3284779	G	0.5	0.5	C
3	BICF2P260085	3300471	A	0	0	C
3	BICF2P60226	3304950	A	0.5	0.5	G
3	BICF2P1417361	3333218	G	0.5	0.5	A
3	BICF2P1385313	3338634	A	0	0	G
3	BICF2P1385315	3338821	C	0.5	0.5	A
3	BICF2P1161316	3350089	C	0.5	0.5	A
3	BICF2P483614	3351403	A	0	0	G
3	BICF2P1250829	3368640	A	0	0	C
3	BICF2P294175	3383005	A	0.5	0.5	G
3	BICF2P698064	3395298	A	0	0	G
3	BICF2P708935	3408351	G	0.5	0.5	A
3	BICF2P826816	3421421	G	0.5	0.5	A
3	BICF2P1361109	3434927	G	0	0	A
3	BICF2P1385584	3448218	A	0	0	G
3	BICF2P1226230	3467283	A	0	0	G
3	BICF2P726132	3475890	A	0	0	G
3	BICF2P321581	3487991	G	0.5	0.5	A
3	BICF2P1157253	3501613	A	0.5	0.5	G
3	BICF2P843988	3512438	G	0.5	0.5	A
3	BICF2P350608	3521618	A	0.5	0.5	G
3	BICF2P590308	3537778	0	0	0	G
3	BICF2P1496475	3547159	A	0	0	G
3	BICF2P343750	3563420	G	0.5	0.5	A
3	BICF2S23643602	3568490	A	0.5	0.5	G
3	BICF2P216187	3593045	A	0.5	0.5	G
3	BICF2P1342089	3620587	A	0.5	0.5	C
3	BICF2S23734876	3624489	G	1	0.7143	A
3	TIGRP2P36385_rs8799946	3665093	G	0.5	0.5	A
3	TIGRP2P36389_rs9013240	3677104	0	0	0	G

3	BICF2P388873	3680666	0	0	0	G
3	TIGRP2P36393_rs9000896	3693974	G	0.5	0.5	A
3	BICF2P211817	3712280	A	0.5	0.5	C
3	BICF2P893560	3715842	A	0.5	0.5	G
3	BICF2S23246428	3737394	G	0.5	0.5	A
3	BICF2P211151	3745121	A	0.5	0.5	G
3	BICF2P770078	3756501	C	0.5	0.5	A
3	TIGRP2P36404_rs8872386	3763186	0	0	0	A
3	BICF2P559486	3780109	A	0.5	0.5	G
3	TIGRP2P36420_rs9214670	3798474	A	0	0	G
3	TIGRP2P36424_rs9086194	3809151	0	0	0	C
3	BICF2P386747	3819124	G	0.5	0.5	A
3	BICF2S23234865	3870869	A	0	0	G
3	BICF2S23230666	3875619	A	0	0	G
3	BICF2S23735196	3903071	G	0	0	A
3	BICF2P789209	3911149	G	0	0	A
3	BICF2P229642	3922440	A	0	0	G
3	TIGRP2P36451_rs8780839	3938760	0	0	0	G
3	TIGRP2P36458_rs8587647	3946047	G	0	0	A
3	BICF2S23328309	3956041	C	0	0	A
3	BICF2P455712	3975513	0	0	0	A
3	BICF2P948430	3983276	T	0	0	A
3	BICF2S23019708	3997069	A	0	0	G
3	BICF2P374175	4003152	T	0	0	A
3	BICF2P1039509	4016616	A	1	1	G
3	BICF2S23720562	4029146	T	0.5	0.5	A
3	BICF2P1173228	4041855	A	1	1	G
3	BICF2P579821	4056460	A	0	0	G
3	BICF2P42954	4067754	A	1	1	G
3	TIGRP2P36489_rs9234704	4069151	C	1	1	A
3	TIGRP2P36492_rs8648834	4078544	A	0	0	C
3	BICF2P174272	4083246	C	0	0	A
3	BICF2P1239217	4112009	G	1	1	A
3	BICF2S23118602	4119845	0	0	0	G
3	TIGRP2P36496_rs9179969	4138894	A	1	1	C
3	TIGRP2P36497_rs9146112	4147576	G	1	1	A
3	BICF2S23327686	4156158	G	0	0	C
3	BICF2P131234	4163973	G	0	0	A
3	TIGRP2P36499_rs8778118	4179182	A	1	1	T
3	BICF2P608440	4196413	A	0	0	G
3	TIGRP2P36501_rs9086277	4218941	A	1	1	G
3	BICF2P929337	4229407	0	0	0	A
3	BICF2P173670	4235656	0	0	0	T
3	BICF2S23536383	4246963	0	0	0	C
3	BICF2P267803	4256968	G	1	1	A
3	BICF2P132279	4274768	G	0	0	A
3	BICF2S23522972	4309405	G	1	1	C

3	BICF2S2331851	4312101	G	0	0	A
3	BICF2P1300188	4316110	A	0	0	G
3	BICF2P882229	4333324	A	0.5	0.5	C
3	BICF2S23141168	4346531	0	0	0	G
3	BICF2S23347900	4352336	A	1	1	G
3	BICF2P131240	4375256	G	0	0	A
3	TIGRP2P36524_rs8789378	4387480	A	1	1	G
3	BICF2S2315274	4389257	C	0	0	A
3	BICF2S22942369	4410116	T	0	0	A
3	BICF2P51740	4421103	G	1	1	A
3	BICF2S2321203	4437180	G	0	0	A
3	BICF2P646107	4441536	G	1	1	A
3	BICF2S22942857	4468832	A	0	0	C
3	TIGRP2P36564_rs8512496	4474123	0	0	0	A
3	TIGRP2P36570_rs8784408	4489224	A	1	1	C
3	BICF2P768261	4503704	0	0	0	T
3	BICF2S22955566	4512472	A	1	1	G
3	BICF2P675173	4527335	G	0	0	A
3	TIGRP2P36576_rs9086577	4532615	0	0	0	G
3	BICF2P770008	4553401	G	1	1	A
3	BICF2P506590	4555779	G	0	0	A
3	BICF2P8793	4593671	A	0	0	G
3	BICF2S24112683	4625311	A	0	0	G
3	BICF2P911821	4656514	0	0	0	G
3	TIGRP2P36578_rs9180233	4668151	0	0	0	G
3	BICF2P1446089	4671003	0	0	0	G
3	BICF2P910977	4685799	0	0	0	G
3	BICF2P863023	4695416	G	0	0	A
3	BICF2S23051267	4711551	A	0.5	0.5	G
3	BICF2P405194	4733612	0	0	0	G
3	G213f32S347	4754957	0	0	0	C
3	BICF2P817678	4776946	T	0	0	A
3	BICF2S23119909	4800345	A	0	0	G
3	BICF2P1149827	4807249	0	0	0	A
3	BICF2S2454847	4820610	G	0	0	A
3	BICF2S23354924	4834254	A	0	0	G
3	BICF2S23655793	4843316	A	0	0	C
3	TIGRP2P36588_rs9221704	4858035	A	0	0	C
3	TIGRP2P36590_rs8840854	4871621	G	0	0	A
3	BICF2P323237	4877956	G	0	0	A
3	BICF2P763435	4888748	0	0	0	T
3	BICF2P448218	4903560	A	0	0	G
3	TIGRP2P36592_rs8960698	4914879	G	0	0	A
3	BICF2P64170	4928180	A	0	0	G
3	BICF2P1176410	4933849	A	1	1	G
3	BICF2P597829	4954601	A	0	0	G
3	BICF2P1080714	4959855	G	0	0	A

3	TIGRP2P36601_rs9162510	4973704	A	1	1	G
3	BICF2S23030965	4980149	A	0	0	G
3	TIGRP2P36602_rs8695031	4998453	A	0.5	0.5	G
3	BICF2P373707	5002569	A	0	0	G
3	BICF2P183465	5017690	A	0	0	G
3	BICF2P123336	5026158	A	0	0	G
3	TIGRP2P36605_rs8503054	5027243	A	0	0	G
3	BICF2P1269887	5031928	G	0	0	A
3	BICF2S23453643	5063126	0	0	0	G
3	BICF2S23239386	5082620	G	0	0	A
3	BICF2P502510	5084816	T	0.5	0.5	A
3	BICF2S23452615	5095744	G	0	0	A
3	BICF2S23214550	5106228	A	0	0	G
3	BICF2P770946	5114261	0	0	0	A
3	TIGRP2P45085_rs9080684	5121092	A	0	0	G
3	BICF2P608989	5137673	0	0	0	A
3	BICF2P399448	5151785	0	0	0	G
3	BICF2P1117346	5162057	A	0	0	C
3	BICF2P774726	5170412	G	0	0	A
3	BICF2S23239200	5176486	A	0	0	G
3	BICF2P39835	5187817	0	0	0	A
3	BICF2P1436036	5201070	A	0	0	C
3	BICF2P879101	5211310	A	0	0	G
3	BICF2P113112	5221225	A	0.5	0.5	G
3	BICF2P379530	5233667	C	0	0	A
3	BICF2P475099	5245090	A	0	0	G
3	TIGRP2P45104_rs8838788	5260931	G	0	0	A
3	BICF2P640419	5268379	0	0	0	A
3	BICF2P614195	5279507	0	0	0	G
3	BICF2P150216	5299580	A	0	0	G
3	BICF2P627931	5303385	0	0	0	G
3	BICF2P360769	5317509	0	0	0	G
3	BICF2P529456	5328933	A	0	0	G
3	TIGRP2P45112_rs9001383	5338620	C	0	0	A
3	BICF2S23246002	5360078	0	0	0	G
3	BICF2P194703	5371137	G	0	0	A
3	BICF2P209319	5384473	G	0	0	A
3	BICF2P22627	5401853	A	0	0	G
3	TIGRP2P45129_rs9029329	5410018	A	0	0	G
3	BICF2P1038896	5426478	A	0	0	G
3	BICF2S2337294	5436506	A	0	0	G
3	BICF2P785126	5441183	A	0	0	G
3	BICF2P1209289	5456793	A	0	0	G
3	TIGRP2P45140_rs9126689	5471514	G	0	0	A
3	TIGRP2P45143_rs8858705	5475615	G	0	0	A
3	TIGRP2P45151_rs9083376	5487857	G	0	0	A
3	TIGRP2P45157_rs8516350	5508995	T	1	1	A

3	BICF2P112727	5521110	A	0	0	G
3	BICF2P148547	5534827	A	0	0	G
3	TIGRP2P45171_rs8861245	5564882	A	1	1	G
3	BICF2P761881	5573179	C	0.5	0.5	A
3	BICF2P363714	5581700	G	1	1	A
3	BICF2P1010972	5592544	A	1	1	G
3	TIGRP2P45176_rs9105743	5638043	A	0	0	G
3	TIGRP2P45178_rs9042445	5647032	C	0	0	G
3	TIGRP2P45180_rs9114922	5663002	0	0	0	G
3	BICF2P578362	5678533	0	0	0	G
3	BICF2P1026069	5700685	A	0	0	G
3	BICF2P327737	5709075	A	0	0	G
3	TIGRP2P45197_rs8692610	5719522	G	0	0	A
3	BICF2S22960989	5736343	C	0.5	0.5	A
3	TIGRP2P45218_rs8912453	5742823	G	0.5	0.5	A
3	BICF2P1190297	5752510	G	0.5	0.5	A
3	BICF2S2328630	5770706	A	0	0	G
3	BICF2P840628	5806192	A	0	0	G
3	BICF2S23347294	5808087	0	0	0	G
3	TIGRP2P45242_rs8804141	5834019	G	0	0	A
3	BICF2P1294537	5849603	0	0	0	A
3	TIGRP2P45246_rs8859379	5864745	A	0	0	G
3	TIGRP2P45247_rs9138202	5865947	A	0.5	0.5	G
3	TIGRP2P45249_rs9090647	5876747	G	0	0	A
3	BICF2P721123	5899710	G	0	0	A
3	BICF2P121216	5916047	0	0	0	A
3	BICF2P1381494	5931456	0	0	0	T
3	TIGRP2P45259_rs8833312	5942967	A	0	0	C
3	TIGRP2P45264_rs8762304	5960454	A	0	0	G
3	BICF2P1018229	5970041	A	0	0	G
3	TIGRP2P45267_rs9126590	5988284	G	0	0	C
3	BICF2P569426	6000198	G	0	0	A
3	BICF2P238213	6009648	C	0	0	A
3	BICF2P374874	6017539	0	0	0	G
3	BICF2P370702	6036115	0	0	0	G
3	TIGRP2P45276_rs9120264	6048543	A	0	0	G
3	BICF2P432580	6063402	0	0	0	A
3	BICF2P87500	6082996	0	0	0	G
3	BICF2P1023047	6148587	G	0	0	A
3	BICF2P926075	6174110	0	0	0	G
3	BICF2P927164	6177284	0	0	0	T
3	BICF2P133199	6216098	A	0	0	G
3	BICF2P1340534	6230628	0	0	0	G
3	BICF2P556026	6241715	C	0	0	A
3	BICF2P35114	6250178	G	0.5	0.5	A
3	BICF2P510160	6257542	G	0.5	0.5	A
3	TIGRP2P45301_rs9104411	6272593	A	0	0	G

3	BICF2P412371	6280200	G	0.5	0.5	A
3	BICF2P152439	6305511	A	0.5	0.5	C
3	TIGRP2P45306_rs8965847	6319525	A	0	0	G
3	G215f44S98	6340031	A	0.5	0.5	G
3	BICF2P844916	6359765	0	0	0	G
3	BICF2P623216	6379817	A	0.5	0.5	G
3	TIGRP2P45309_rs9154617	6386168	0	0	0	C
3	BICF2P1182555	6401686	A	0.5	0.5	G
3	BICF2P64060	6411068	A	0.5	0.5	G
3	BICF2P1458832	6417921	A	0.5	0.5	C
3	BICF2P158838	6433958	G	0.5	0.5	A
3	BICF2P255248	6448198	0	0	0	A
3	BICF2S23717124	6453902	G	0	0	C
3	BICF2P120908	6465754	0	0	0	G
3	BICF2S23122481	6479523	0	0	0	G
3	BICF2P778286	6488105	A	0.5	0.5	C
3	BICF2P1072765	6535448	G	0	0	A
3	BICF2P218960	6545410	0	0	0	A
3	BICF2P764002	6565695	A	0.5	0.5	G
3	BICF2S22911832	6585336	0	0	0	A
3	BICF2P257223	6595265	0	0	0	A
3	BICF2P775487	6608661	0	0	0	A
3	BICF2P1038060	6617219	A	0.5	0.5	G
3	BICF2P1389575	6643811	A	0.5	0.5	G
3	BICF2P718727	6654087	0	0	0	A
3	BICF2P1324543	6669598	0	0	0	G
3	BICF2P1126290	6677696	0	0	0	A
3	BICF2P625748	6684247	A	0.5	0.5	C
3	BICF2P1475251	6693476	T	1	1	A
3	BICF2P750243	6705845	0	0	0	G
3	BICF2P818194	6717409	G	0	0	A
3	BICF2P160065	6730555	A	0	0	G
3	BICF2P425876	6751338	0	0	0	A
3	BICF2S244930	6770801	G	0	0	A
3	BICF2P226095	6784469	0	0	0	A
3	BICF2P1275437	6814927	0	0	0	A
3	BICF2S23218457	6829635	A	0	0	G
3	BICF2S2444071	6838009	0	0	0	A
3	TIGRP2P45350_rs8998629	6859497	0	0	0	A
3	TIGRP2P45351_rs8778463	6902396	0	0	0	C
3	TIGRP2P45352_rs9214992	6936062	0	0	0	G
3	BICF2P1049811	6956567	A	0.5	0.5	G
3	BICF2P1315430	6959902	0	0	0	G
3	BICF2P1109615	6974365	A	0.5	0.5	C
3	TIGRP2P45362_rs8911608	7003060	A	1	1	C
3	TIGRP2P45363_rs9170628	7005739	G	0.5	0.5	A
3	BICF2P963720	7015262	0	0	0	A

3	TIGRP2P45368_rs9056918	7031996	T	0.5	0.5	A
3	BICF2P1100375	7044323	A	0.5	0.5	G
3	BICF2P1191460	7053155	0	0	0	G
3	BICF2P100171	7085123	0	0	0	A
3	BICF2P1210047	7099529	C	1	1	A
3	BICF2P324741	7108488	A	1	1	G
3	BICF2P944098	7128965	A	1	1	C
3	BICF2P1281325	7142656	G	1	1	A
3	TIGRP2P45392_rs8912615	7157590	C	0	0	G
3	BICF2P765781	7159271	A	1	1	G
3	BICF2P1467435	7165460	G	1	1	A
3	BICF2P548951	7183766	A	1	1	G
3	BICF2P746545	7187572	A	1	1	C
3	BICF2P321758	7200983	0	0	0	C
3	BICF2P652168	7216242	A	1	1	G
3	BICF2S23415855	7233462	A	0	0	G
3	BICF2S23417246	7237134	A	0	0	G
3	BICF2S23250633	7247855	A	0	0	C
3	BICF2S2373463	7261663	C	0	0	A
3	BICF2P721893	7271683	A	1	1	G
3	BICF2P639558	7281263	0	0	0	G
3	BICF2P1305404	7291000	A	1	1	G
3	BICF2P1438676	7304494	0	0	0	G
3	BICF2P334455	7318331	A	1	1	G
3	BICF2P1235966	7324890	0	0	0	G
3	BICF2S23343243	7330607	C	0	0	A
3	BICF2S23622462	7338908	G	1	1	A
3	BICF2S23442646	7360247	A	0	0	G
3	BICF2S23144152	7374435	G	0	0	A
3	TIGRP2P45421_rs9134255	7394172	0	0	0	G
3	BICF2P402373	7400987	0	0	0	C
3	BICF2P135713	7424979	0	0	0	G
3	BICF2P1141444	7439976	0	0	0	C
3	BICF2S2293065	7473058	A	0	0	G
3	BICF2S2371566	7480480	A	0	0	G
3	TIGRP2P45424_rs9030361	7499946	0	0	0	G
3	BICF2S2296470	7512729	A	0	0	C
3	BICF2P540583	7532293	A	1	1	G
3	BICF2P545236	7546602	0	0	0	G
3	BICF2P1196237	7585270	G	1	1	A
3	BICF2P1189099	7591169	0	0	0	G
3	BICF2S23637832	7607940	C	1	1	A
3	BICF2P1248631	7621958	A	1	1	G
3	TIGRP2P45434_rs8643526	7633836	0	0	0	A
3	BICF2P1371995	7658828	0	0	0	C
3	BICF2P1011641	7676703	A	1	1	G
3	BICF2P191993	7686724	0	0	0	A

3	BICF2P384729	7694686	G	1	1	A
3	BICF2P668685	7706187	C	1	1	A
3	BICF2P113222	7719077	0	0	0	G
3	BICF2P92993	7745945	A	1	1	G
3	BICF2P1230901	7751495	A	1	1	C
3	BICF2P59387	7771458	A	1	1	G
3	TIGRP2P45454_rs8811853	7782466	A	1	1	G
3	BICF2P877719	7791517	G	1	1	A
3	TIGRP2P45462_rs8969936	7800966	A	0	0	C
3	TIGRP2P45470_rs8903125	7809289	A	0	0	G
3	BICF2S2339067	7821823	A	0.5	0.5	G
3	TIGRP2P45492_rs9034213	7832484	C	0	0	A
3	BICF2P1331559	7843594	A	0.5	0.5	G
3	BICF2P365041	7845498	G	0.5	0.5	A
3	BICF2P1339644	7869260	G	0	0	A
3	BICF2P1093678	7898116	0	0	0	A
3	BICF2P910224	7901603	A	0	0	T
3	TIGRP2P45498_rs8907234	7913198	0	0	0	G
3	BICF2S23148795	7941706	T	0	0	A
3	BICF2P8038	7963004	A	NA	0.5	G
3	BICF2S23138077	7980952	A	0.5	0.5	G
3	BICF2S23137813	7985001	0	0	0	A
3	BICF2P121017	8005271	0	0	0	A
3	BICF2P96074	8022952	0	0	0	A
3	BICF2S23715582	8046860	A	0.5	0.5	C
3	BICF2P239474	8052016	0	0	0	A
3	BICF2P105885	8068793	0	0	0	A
3	G216f18S108	8094673	0	0	0	A
3	G216f22S363	8097273	0	0	0	C
3	BICF2P1174301	8122987	0	0	0	A
3	BICF2P1196757	8133607	0	0	0	A
3	BICF2P249198	8165688	A	0	0	G
3	TIGRP2P45503_rs9133910	8196101	0	0	0	A
3	BICF2P1192849	8211178	G	0	0	A
3	G217f42S297	8241731	0	0	0	C
3	BICF2P852292	8269294	A	0.5	0.5	G
3	BICF2P968439	8285812	0	0	0	G
3	BICF2S2316598	8296160	A	0.5	0.5	G
3	BICF2S22936690	8304460	0	0	0	G
3	BICF2S2304070	8321106	C	0.5	0.5	A
3	BICF2S24115352	8332047	G	0.5	0.5	A
3	BICF2P1058423	8346513	0	0	0	C
3	BICF2P223058	8370253	0	0	0	G
3	TIGRP2P45505_rs9136545	8379921	0	0	0	A
3	BICF2P1402035	8391807	0	0	0	A
3	BICF2P384548	8400182	A	0.5	0.5	C
3	BICF2P1061008	8440653	A	0.5	0.5	C

3	BICF2P394877	8443087	A	0.5	0.5	G
3	BICF2P394880	8443237	A	0.5	0.5	C
3	BICF2P1011805	8474530	G	0.5	0.5	A
3	BICF2S23210027	8483449	A	0	0	C
3	BICF2S23345528	8487745	G	0.5	0.5	A
3	G218f46S257	8510901	A	0	0	G
3	TIGRP2P45528_rs9141525	8525501	A	0	0	G
3	BICF2P351298	8566768	A	0.5	0.5	G
3	BICF2P1173106	8577197	G	0.5	0.5	A
3	BICF2P906317	8582474	A	0.5	0.5	G
3	BICF2S23413877	8596944	C	0	0	A
3	BICF2P1361596	8610095	G	0	0	A
3	BICF2P568320	8624923	G	0.5	0.5	A
3	TIGRP2P45589_rs8592830	8637459	G	0.5	0.5	A
3	BICF2P1207838	8657487	G	0.5	0.5	A
3	BICF2P248036	8667425	A	0.5	0.5	G
3	BICF2S23420591	8672885	G	0.5	0.5	A
3	BICF2P856382	8691934	G	0	0	A
3	BICF2P258336	8721170	C	0.5	0.5	A
3	BICF2P295557	8737205	A	0.5	0.5	C
3	TIGRP2P45615_rs9044961	8777165	0	0	0	A
3	BICF2P1183213	8802690	0	0	0	A
3	TIGRP2P45620_rs8593068	8813812	0	0	0	A
3	BICF2P806256	8831011	0	0	0	C
3	BICF2P274451	8851662	0	0	0	A
3	BICF2P512031	8856941	C	1	1	A
3	BICF2P732834	8892632	0	0	0	G
3	TIGRP2P45621_rs8904167	8903718	C	0.5	0.5	A
3	BICF2S23512103	8913698	A	0	0	C
3	BICF2P1314301	8944849	0	0	0	G
3	BICF2S23320254	8954795	A	0	0	G
3	BICF2S23241911	8968621	G	0.5	0.5	A
3	BICF2P290894	8978705	C	1	1	A
3	BICF2P951218	8982612	A	1	1	G
3	BICF2P1007222	8995523	G	1	1	A
3	BICF2P569255	9004559	A	1	1	G
3	BICF2P588481	9019454	A	1	1	G
3	BICF2S23427319	9028398	G	0	0	A
3	BICF2P5008	9032658	G	0.5	0.5	A
3	BICF2P822346	9040798	G	0.5	0.5	A
3	BICF2P834433	9058347	A	0.5	0.5	G
3	BICF2P546585	9064208	A	0.5	0.5	C
3	BICF2P937173	9074502	A	0.5	0.5	G
3	BICF2P422772	9096468	G	0.5	0.5	A
3	BICF2P1104313	9112930	G	0.5	0.5	A
3	BICF2S23532115	9153378	0	0	0	A
3	TIGRP2P45681_rs8909428	9160101	0	0	0	C

3	BICF2P992541	9167188	A	0.5	0.5	G
3	TIGRP2P45691_rs8865526	9179326	G	0	0	A
3	BICF2P258919	9190590	G	0	0	A
3	BICF2P715385	9205664	A	0	0	G
3	TIGRP2P45706_rs8599500	9218569	G	0	0	A
3	BICF2S23333174	9229621	G	0	0	A
3	BICF2S23148241	9241904	A	0.5	0.5	G
3	BICF2P466284	9247011	G	0.5	0.5	A
3	BICF2P457198	9260655	G	0.5	0.5	A
3	BICF2P628008	9271118	A	0.5	0.5	G
3	BICF2P483772	9289756	C	0	0	A
3	BICF2P289429	9295671	G	0.5	0.5	A
3	BICF2P966238	9306122	A	0.5	0.5	G
3	BICF2P3611	9322095	G	0.5	0.5	A
3	BICF2P1284028	9335997	A	0.5	0.5	G
3	BICF2P552772	9346034	C	0.5	0.5	G
3	TIGRP2P45754_rs9123858	9352098	0	0	0	A
3	BICF2S24411064	9362773	A	0	0	G
3	BICF2S23314596	9374543	A	0	0	G
3	BICF2P173727	9388094	A	0.5	0.5	C
3	TIGRP2P45779_rs9107776	9403160	A	0.5	0.5	G
3	BICF2P755008	9410150	0	0	0	A
3	TIGRP2P45797_rs9041842	9424776	C	0.5	0.5	A
3	BICF2P601292	9434108	A	0.5	0.5	G
3	TIGRP2P45824_rs8859874	9443380	C	0.5	0.5	A
3	TIGRP2P45829_rs8770184	9459700	A	1	1	G
3	BICF2S23612703	9472808	A	0.5	0.5	G
3	BICF2S23535224	9483495	C	0.5	0.5	A
3	BICF2P1177132	9498050	0	0	0	G
3	BICF2P618328	9508000	C	1	1	A
3	BICF2P597287	9520707	G	0.5	0.5	A
3	BICF2P568205	9524677	C	0.5	0.5	A
3	BICF2S23415543	9533656	A	0.5	0.5	G
3	BICF2P321638	9548768	A	0.5	0.5	G
3	TIGRP2P45860_rs8748956	9565006	A	0.5	0.5	G
3	TIGRP2P45864_rs9117122	9570681	G	0	0	A
3	TIGRP2P45870_rs8723359	9587629	A	0	0	G
3	TIGRP2P45883_rs8651134	9598231	G	0.5	0.5	A
3	TIGRP2P45888_rs8495342	9610915	A	0.5	0.5	G
3	BICF2P1276741	9614971	A	0.5	0.5	C
3	TIGRP2P45895_rs9120278	9636904	G	0.5	0.5	A
3	BICF2P421687	9639531	G	0	0	A
3	TIGRP2P45906_rs8571680	9649939	C	0.5	0.5	A
3	BICF2P544891	9667374	0	0	0	G
3	BICF2P243279	9683118	A	0.5	0.7	G
3	BICF2P411956	9697479	0	0	0	G
3	BICF2S23653857	9713463	G	0	0	A

3	BICF2S23452713	9718589	G	0	0	A
3	TIGRP2P45913_rs8785988	9732824	A	0	0	G
3	BICF2S23724973	9765040	C	0.5	0.5	G
3	BICF2P48333	9780868	G	0	0	A
3	BICF2P1436660	9801015	G	0	0	A
3	BICF2S23312223	9818208	A	0	0	G
3	BICF2P1216659	9836417	A	1	1	T
3	BICF2P712305	9846089	G	0	0	A
3	BICF2S23525101	9856191	G	0	0	A
3	BICF2P874673	9869094	A	0	0	G
3	BICF2S2302543	9882752	C	0	0	A
3	BICF2P964521	9897612	A	0	0	G
3	BICF2P322642	9910120	A	0	0	G
3	BICF2P132784	9913272	A	0.5	0.5	G
3	BICF2S23428747	9924517	T	0	0	A
3	BICF2S23551404	9944981	A	0	0	G
3	TIGRP2P45978_rs9156962	9954059	G	0.5	0.5	A
3	BICF2S23335974	9963481	A	0	0	G
3	TIGRP2P45985_rs8854856	9972611	A	1	1	G
3	BICF2P638648	9995007	C	0	0	A
3	BICF2P232647	10012277	A	1	1	C
3	BICF2P205732	10020239	A	0	0	C
3	BICF2S23144027	10031804	A	1	1	G
3	BICF2P55003	10043907	0	0	0	C
3	BICF2P245502	10055971	C	1	1	A
3	BICF2P715712	10067941	0	0	0	A
3	BICF2S23238701	10091291	A	0	0	C
3	BICF2P1140981	10101122	C	1	1	G
3	BICF2P533355	10115131	C	0.5	0.5	A
3	BICF2S22943822	10120347	G	0	0	A
3	BICF2P736471	10135700	A	0	0	C
3	BICF2P610812	10160589	0	0	0	A
3	BICF2P344170	10172367	A	0	0	G
3	TIGRP2P36622_rs9183410	10188118	A	0	0	G
3	BICF2P144764	10192064	A	1	1	C
3	TIGRP2P36631_rs8783832	10221293	G	0	0	A
3	BICF2P723714	10227835	A	1	1	T
3	BICF2P761067	10236519	A	1	1	C
3	BICF2S23319098	10250420	A	0	0	G
3	BICF2S23020622	10264073	A	0	0	G
3	BICF2P1216654	10275855	G	0	0	A
3	BICF2P1162849	10285254	G	0	0	A
3	BICF2P14296	10296328	C	0	0	A
3	BICF2P1510622	10306292	A	0	0	G
3	BICF2S231822	10326335	G	0	0	A
3	BICF2P1330386	10328241	G	0	0	A
3	TIGRP2P36683_rs8626297	10341071	A	0	0	C

3	BICF2P395505	10355292	A	0.5	0.5	G
3	BICF2P901484	10372715	0	0	0	A
3	BICF2P901485	10373289	A	0.5	0.5	C
3	BICF2P999252	10384532	G	0.5	0.5	A
3	BICF2P107402	10399716	A	0.5	0.5	G
3	BICF2P594494	10430389	C	0.5	0.5	A
3	BICF2P476249	10436969	A	0.5	0.5	C
3	BICF2P822927	10452511	G	0.5	0.5	A
3	TIGRP2P36715_rs8711082	10455881	G	0.5	0.5	A
3	BICF2P370003	10468470	G	0.5	0.5	A
3	BICF2P604268	10484925	G	0	0	A
3	TIGRP2P36733_rs9090895	10488060	G	0.5	0.5	A
3	BICF2P629147	10499521	G	0.5	0.5	A
3	TIGRP2P36746_rs8772126	10512466	A	0	0	G
3	BICF2P926937	10531115	G	0	0	A
3	TIGRP2P36751_rs9193634	10543325	A	1	1	G
3	BICF2P533876	10547662	0	0	0	G
3	BICF2P403811	10566141	G	0	0	A
3	BICF2P982386	10574372	C	1	1	A
3	BICF2P1151110	10579925	A	1	1	G
3	BICF2P919299	10583711	G	0	0	A
3	BICF2P759842	10592323	A	1	1	G
3	BICF2P819090	10598980	A	1	1	G
3	BICF2P459711	10609988	A	0	0	C
3	TIGRP2P36777_rs8579883	10618637	G	0	0	A
3	BICF2P839933	10630359	A	0.5	0.5	G
3	BICF2S22960543	10646996	A	0.5	0.5	G
3	BICF2P512547	10655446	0	0	0	G
3	TIGRP2P36804_rs8667668	10667871	G	0.5	0.5	A
3	BICF2P180647	10683231	G	0.5	0.5	A
3	BICF2P124146	10695322	A	0	0	G
3	BICF2P738656	10717081	0	0	0	G
3	BICF2P1028059	10727602	G	0	0	A
3	BICF2P41627	10731878	G	0	0	A
3	BICF2P674750	10751107	G	1	1	A
3	BICF2P178694	10753798	G	1	1	A
3	BICF2P736757	10767061	G	1	1	A
3	BICF2P819554	10785940	G	0.5	0.5	A
3	TIGRP2P36862_rs9078300	10788038	G	1	1	A
3	BICF2P823912	10807515	G	0	0	A
3	BICF2P1120851	10819591	A	0	0	G
3	TIGRP2P36875_rs8966525	10829776	G	0	0	A
3	BICF2P1125699	10833728	G	0.5	0.5	A
3	BICF2S23121259	10848833	G	0.5	0.5	A
3	BICF2P303472	10858968	A	0	0	C
3	BICF2S2349428	10872213	C	1	1	A
3	TIGRP2P36885_rs8763792	10886340	A	0.5	0.5	G

3	BICF2P1069225	10894273	G	0.5	0.5	A
3	BICF2P192626	10902363	G	0	0	A
3	BICF2P407904	10934470	A	0	0	G
3	BICF2P786548	10953876	A	0	0	G
3	BICF2S23026541	10967906	C	0	0	A
3	BICF2P716758	10978842	G	0	0	A
3	BICF2P648968	10999819	G	0	0	A
3	BICF2P355091	11024892	G	0	0	A
3	TIGRP2P36913_rs9074295	11031253	A	0	0	G
3	BICF2P795341	11053726	A	0	0	G
3	TIGRP2P36922_rs9028836	11063859	G	0	0	A
3	TIGRP2P36926_rs8786180	11075791	G	0	0	A
3	TIGRP2P36936_rs9137495	11093809	A	0	0	T
3	BICF2P968794	11108039	A	0	0	G
3	BICF2P968797	11109548	C	0	0	A
3	BICF2S2337168	11111795	G	0	0	A
3	TIGRP2P36944_rs8598010	11123567	A	0.5	0.5	G
3	TIGRP2P36953_rs8559586	11132074	A	0	0	C
3	BICF2P448370	11152865	A	0	0	G
3	BICF2P503364	11159840	G	0	0	A
3	BICF2S23158229	11171634	A	0.5	0.5	C
3	BICF2P724585	11187415	A	0	0	G
3	BICF2S22932515	11191297	G	0	0	A
3	BICF2S23443419	11202424	G	0	0	A
3	BICF2P893044	11212683	A	0	0	G
3	BICF2P93678	11241077	A	0	0	G
3	BICF2P696947	11256097	A	0	0	C
3	BICF2P728680	11264446	A	0	0	G
3	BICF2P785681	11270993	A	0	0	G
3	BICF2P1040888	11285173	G	0	0	A
3	BICF2P1063268	11295498	A	0	0	C
3	BICF2P1277561	11305564	G	1	1	A
3	BICF2P77485	11326527	A	0	0	G
3	BICF2P77488	11327223	G	1	1	A
3	BICF2P1295599	11337008	A	1	1	G
3	BICF2P7036	11343553	A	0	0	G
3	TIGRP2P37007_rs9095959	11360445	G	0	0	A
3	BICF2P165491	11363967	A	0	0	G
3	BICF2P423580	11383393	0	0	0	A
3	BICF2S23326710	11386247	A	0.5	0.5	G
3	BICF2P459172	11398372	A	1	1	C
3	TIGRP2P37018_rs9229091	11409267	G	1	1	A
3	BICF2P771589	11420033	C	1	1	A
3	BICF2P1085025	11431074	A	0	0	G
3	TIGRP2P37032_rs8579372	11448625	0	0	0	G
3	BICF2P940908	11458192	A	0.5	0.5	G
3	BICF2P210365	11467133	A	1	1	G

3	BICF2P574442	11480535	A	0	0	G
3	BICF2P215968	11519852	A	0	0	G
3	BICF2P366448	11534378	G	0.5	0.5	A
3	BICF2P671174	11540377	A	0.5	0.5	G
3	BICF2P233044	11546378	0	0	0	A
3	BICF2P724577	11562857	G	0.5	0.5	A
3	BICF2P428965	11580496	G	0.5	0.5	A
3	BICF2P397686	11587215	0	0	0	A
3	BICF2P1353167	11599231	A	0.5	0.5	G
3	BICF2S23240631	11610468	G	0.5	0.5	A
3	BICF2P462679	11637336	G	0.5	0.5	A
3	BICF2P1234362	11642809	G	0.5	0.5	A
3	BICF2P905533	11685513	C	0.5	0.5	A
3	TIGRP2P37062_rs8620323	11697877	G	0	0	A
3	BICF2P734604	11713985	A	0	0	G
3	BICF2P211305	11741054	0	0	0	A
3	BICF2P958827	11753329	0	0	0	A
3	BICF2S23643570	11765779	G	0	0	A
3	TIGRP2P37064_rs9027663	11781464	G	0.5	0.5	A
3	TIGRP2P37066_rs8864371	11792182	G	0	0	A
3	BICF2S23316437	11802184	A	0.5	0.5	G
3	BICF2P451307	11817158	0	0	0	A
3	BICF2P772232	11826877	T	0.5	0.5	A
3	TIGRP2P37070_rs8801353	11839998	0	0	0	C
3	BICF2P991961	11845844	0	0	0	G
3	BICF2P1190915	11857309	A	0	0	C
3	TIGRP2P37071_rs9237152	11874423	0	0	0	A
3	BICF2S23424630	11890805	A	0	0	T
3	BICF2P341630	11894226	G	0	0	A
3	BICF2S23526145	11902752	A	0	0	C
3	BICF2P44650	11917834	A	0	0	G
3	BICF2S23329502	11960343	A	0	0	C
3	BICF2P215327	11993227	0	0	0	A
3	BICF2P1301193	12019245	0	0	0	A
3	BICF2P1023944	12043963	0	0	0	G
3	BICF2S2415265	12070746	A	0	0	G
3	BICF2P132093	12078369	A	0	0	G
3	BICF2P497244	12087796	0	0	0	A
3	BICF2P97318	12100284	A	0	0	C
3	BICF2P1170098	12111971	A	0	0	T
3	BICF2P1108458	12122767	G	0	0	A
3	BICF2S24512053	12142919	0	0	0	A
3	BICF2P1356718	12152790	0	0	0	A
3	BICF2P178933	12161552	0	0	0	A
3	BICF2S23247040	12182362	0	0	0	G
3	BICF2P1273390	12199344	G	0.5	0.5	A
3	TIGRP2P37095_rs8781034	12207069	A	0.5	0.5	C

3	BICF2S23225857	12217142	A	0.5	0.5	G
3	BICF2P943716	12224700	0	0	0	T
3	BICF2S24110538	12238968	C	0	0	A
3	BICF2P106465	12255919	A	0.5	0.5	C
3	BICF2P1169041	12262363	G	1	1	A
3	BICF2P344705	12270940	A	0.5	0.5	G
3	TIGRP2P37102_rs8669940	12290222	G	0	0	A
3	BICF2P1059276	12308017	G	0	0	A
3	BICF2S23030474	12317340	G	1	1	A
3	BICF2S2297571	12345445	C	0.5	0.5	A
3	BICF2P172528	12354622	G	0.5	0.5	A
3	BICF2P785100	12369838	A	0	0	C
3	BICF2P326243	12382111	A	0.5	0.5	G
3	BICF2S23524979	12388266	A	0.5	0.5	C
3	BICF2P1250405	12400201	C	0	0	A
3	BICF2P751259	12409828	G	1	1	A
3	BICF2P1339840	12423171	A	1	1	G
3	BICF2P460206	12439348	A	0	0	G
3	BICF2P616721	12449359	A	1	1	G
3	BICF2S23426432	12456791	A	0	0	G
3	BICF2P18052	12471917	G	1	1	A
3	BICF2P1105887	12482219	G	0	0	A
3	BICF2P403216	12496007	G	0.5	0.5	A
3	BICF2P699633	12501694	G	0.5	0.5	A
3	BICF2S23347332	12521142	A	0	0	G
3	BICF2S24410955	12527343	G	0	0	A
3	BICF2P748769	12551373	C	0	0	A
3	TIGRP2P37166_rs8932843	12564262	A	0	0	G
3	TIGRP2P37176_rs9016831	12577361	A	0	0	G
3	BICF2P846462	12582638	G	0	0	A
3	BICF2P686189	12594004	G	0	0	A
3	BICF2P1110909	12601092	A	0.5	0.5	C
3	BICF2P1424985	12609145	A	0	0	G
3	TIGRP2P37188_rs8901932	12615634	A	0	0	G
3	BICF2S23322147	12631338	A	0.5	0.5	G
3	BICF2S23238427	12640495	A	0.5	0.5	G
3	BICF2P219067	12653764	C	0	0	A
3	TIGRP2P37194_rs9077372	12664215	A	0	0	T
3	BICF2P713806	12674721	A	0	0	G
3	BICF2S23328242	12693619	A	0.5	0.5	G
3	BICF2P459546	12698199	0	0	0	G
3	BICF2S23436266	12733324	G	0	0	A
3	BICF2P921776	12744983	A	0	0	G
3	BICF2P662839	12755989	0	0	0	A
3	BICF2S23519256	12780796	A	0.5	0.5	G
3	BICF2S23318729	12853720	G	1	1	C
3	BICF2P1113376	12859465	A	1	1	G

3	BICF2P372137	12879035	0	0	0	G
3	BICF2P973463	12881665	A	1	1	G
3	TIGRP2P37212_rs9195774	12897223	G	1	1	A
3	TIGRP2P37215_rs9023022	12912100	G	1	1	A
3	BICF2S23528652	12914872	G	0	0	A
3	TIGRP2P37218_rs8525632	12935358	A	1	1	G
3	BICF2S23551091	12947793	G	0.5	0.5	C
3	BICF2S23551310	12951458	A	0.5	0.5	G
3	BICF2P840255	12960968	G	1	1	A
3	TIGRP2P37232_rs9114409	12982300	A	1	1	G
3	BICF2P149851	12984151	A	0.5	0.5	G
3	TIGRP2P37246_rs8856738	13001529	A	0.5	0.5	G
3	TIGRP2P37258_rs8559316	13038213	A	0	0	G
3	BICF2S23524855	13043288	G	0.5	0.5	A
3	BICF2P424454	13077394	C	0	0	A
3	TIGRP2P37271_rs9149830	13085445	C	0	0	A
3	BICF2P1319182	13091739	A	0	0	G
3	BICF2P536548	13117102	0	0	0	A
3	BICF2S2399705	13137570	G	0.5	0.5	A
3	BICF2S23327505	13147375	G	0.5	0.5	A
3	BICF2S23214697	13185909	A	0	0	G
3	BICF2S23424050	13193117	G	0	0	A
3	TIGRP2P37283_rs8526227	13204082	A	0	0	G
3	BICF2P1062023	13221751	A	0	0	G
3	TIGRP2P37294_rs8555020	13228173	G	0	0	A
3	BICF2P1188267	13237535	A	0.5	0.5	C
3	BICF2P1174418	13253218	A	0.5	0.5	G
3	TIGRP2P37299_rs9096243	13261835	A	0.5	0.5	G
3	BICF2P520575	13272443	0	0	0	G
3	BICF2P234820	13286037	C	0.5	0.5	A
3	BICF2P455088	13300166	G	0.5	0.5	A
3	BICF2P725253	13316914	A	0.5	0.5	G
3	BICF2P1003142	13328828	0	0	0	G
3	BICF2P426873	13345865	G	0.5	0.5	A
3	BICF2P322969	13354710	A	0.5	0.5	G
3	TIGRP2P37320_rs8620438	13372831	G	0	0	A
3	BICF2P855574	13380299	A	0	0	G
3	BICF2P460115	13395568	G	0.5	0.5	A
3	BICF2P1405260	13409084	C	0	0	G
3	BICF2S2338262	13439674	C	0.5	0.5	A
3	BICF2P511129	13449536	A	0.5	0.5	G
3	BICF2P414180	13456753	A	0.5	0.2857	T
3	BICF2S23119273	13469170	C	0.5	0.2857	A
3	BICF2P285817	13480409	A	0	0	G
3	BICF2P1022802	13491070	A	0	0.2143	G
3	TIGRP2P37369_rs8977768	13506748	G	0	0.2143	A
3	BICF2P184748	13519752	A	0	0	G

3	TIGRP2P37379_rs9097265	13527575	A	0.5	0.5	G
3	BICF2P30406	13546592	A	0	0	G
3	BICF2S2306288	13549313	A	0.5	0.5	G
3	TIGRP2P37392_rs9160368	13565145	A	0	0	G
3	BICF2P216447	13565285	A	0	0	C
3	BICF2P1241810	13569459	A	1	0.7857	G
3	BICF2P1026277	13576155	G	0	0	A
3	BICF2P1441940	13581887	G	0	0	A
3	BICF2P820082	13604439	A	0.5	0.7143	G
3	BICF2P122967	13608948	A	0	0	G
3	BICF2P808674	13618358	A	0.5	0.7143	G
3	BICF2P229390	13631883	A	0.5	0.2857	C
3	TIGRP2P37403_rs8806468	13646981	A	0.5	0.2857	G
3	BICF2P450187	13658271	A	0	0	C
3	BICF2P450189	13658340	G	0.5	0.2857	A
3	BICF2S23619751	13658602	A	0	0	C
3	BICF2S2315014	13671386	A	1	0.7857	G
3	BICF2S23349562	13682845	A	0.5	0.2857	C
3	BICF2P410181	13685564	G	0.5	0.5	A
3	BICF2P780674	13697080	A	0.5	0.5	G
3	BICF2S23523926	13711408	C	0.5	0.5	A
3	BICF2P353177	13721819	A	0	0	G
3	BICF2P9189	13739068	A	0	0	G
3	TIGRP2P37429_rs8511689	13742697	A	0	0.2143	G
3	BICF2P1469511	13757471	A	0	0	T
3	BICF2P384752	13771505	A	0	0	G
3	TIGRP2P37451_rs8795953	13778843	A	0	0	G
3	BICF2P929809	13790079	A	0.5	0.5	G
3	BICF2P179192	13801482	C	0.5	0.5	A
3	BICF2P823113	13811859	A	0.5	0.7143	G
3	TIGRP2P37485_rs8664065	13824411	G	0	0	A
3	BICF2P552674	13841897	C	0.5	0.7143	A
3	BICF2P309148	13854567	G	0	0	A
3	BICF2P698836	13860062	A	0.5	0.7143	C
3	BICF2P1194771	13879127	A	0.5	0.7143	G
3	BICF2P740885	13889231	G	0.5	0.7143	A
3	BICF2P889516	13894319	T	0	0.2143	A
3	BICF2P177046	13914621	0	0	0	A
3	BICF2S23746636	13922790	G	0.5	0.7143	A
3	BICF2P737878	13928418	A	0	0	G
3	BICF2S22959231	13941421	G	0	0	A
3	BICF2P604997	13949858	A	0.5	0.2857	G
3	TIGRP2P37527_rs8792455	13962757	0	0	0	G
3	BICF2S23434865	13975174	A	0	0	G
3	BICF2S23419459	13985920	A	NA	0.5	C
3	BICF2S23435114	13999480	C	0	0	A
3	BICF2P1368174	14011930	G	1	0.7857	A

3	TIGRP2P37531_rs8980563	14025263	A	0	0	T
3	BICF2P174751	14039542	G	0	0.2143	A
3	BICF2S22923775	14043537	A	0	0	G
3	BICF2P38302	14069150	T	0.5	0.5	A
3	BICF2P305584	14080267	C	0.5	0.5	A
3	BICF2P774739	14091049	A	0.5	0.5	G
3	BICF2P965349	14102906	A	0	0	G
3	BICF2P496677	14124304	A	0	0.2143	C
3	BICF2P385811	14134060	A	0.5	0.2857	G
3	BICF2P568033	14140274	A	0.5	0.5	G
3	BICF2P1177107	14151945	A	0	0	G
3	BICF2P445195	14167357	A	0.5	0.2857	G
3	BICF2P928962	14174447	G	0.5	0.2857	A
3	BICF2P309787	14191817	G	0.5	0.5	A
3	BICF2P67682	14206524	A	0.5	0.5	C
3	BICF2P926118	14216112	0	0	0	A
3	BICF2P859855	14233838	A	0.5	0.5	G
3	BICF2P1170805	14265391	A	1	0.7857	G
3	BICF2P1464897	14274245	A	1	0.7857	G
3	TIGRP2P37620_rs8875131	14291149	A	0	0	G
3	BICF2P1122319	14295057	A	0.5	0.5	G
3	BICF2P2166	14307665	G	0.5	0.2857	A
3	BICF2P963113	14325254	0	0	0	A
3	BICF2P1400155	14330281	G	0.5	0.5	A
3	BICF2S23425352	14357509	A	0.5	0.7143	G
3	BICF2P1116208	14366542	G	0.5	0.5	A
3	BICF2P261911	14386278	G	0	0	A
3	BICF2S23211846	14399060	G	0	0.2143	A
3	BICF2S2356607	14413388	A	1	0.7857	G
3	BICF2P1142695	14422933	0	0	0	A
3	TIGRP2P37633_rs8898064	14432992	G	0.5	0.25	A
3	BICF2S2358620	14445552	A	0.5	0.2857	G
3	BICF2S23546324	14456853	G	0.5	0.2857	A
3	BICF2S23340205	14467151	G	0.5	0.25	A
3	BICF2P327677	14487665	G	0.5	0.5	A
3	BICF2P1004368	14495103	G	0.5	0.5	A
3	BICF2S23055617	14505900	A	0	0	G
3	BICF2P226096	14517798	0	0	0	A
3	BICF2P1461292	14539916	A	0	0	G
3	BICF2S23710973	14551192	A	1	0.7857	G
3	BICF2P447550	14563241	G	0	0	A
3	BICF2P283440	14578210	G	0	0	A
3	BICF2P996183	14585741	A	0	0	G
3	BICF2S23210201	14596954	C	0	0	A
3	BICF2P467774	14607123	A	0.5	0.2857	G
3	BICF2S2322937	14625123	G	0	0.2143	A
3	TIGRP2P37703_rs8543293	14631607	A	0	0.2143	G

3	BICF2P1335336	14642087	0	0	0	A
3	TIGRP2P37711_rs8831890	14662642	0	0	0	G
3	BICF2P940625	14680231	G	0	0	A
3	BICF2P685806	14687467	G	0	0	A
3	BICF2P529279	14705033	A	0	0	C
3	TIGRP2P37732_rs9039278	14714478	G	0.5	0.5	A
3	BICF2P958553	14726803	G	0	0	A
3	TIGRP2P37738_rs8866173	14735828	A	0	0.2143	G
3	TIGRP2P37743_rs9243592	14747609	0	0	0	A
3	BICF2P331050	14761741	G	0	0.2143	A
3	BICF2P1218685	14767196	A	0.5	0.7143	G
3	TIGRP2P37758_rs8588906	14778714	A	0	0.2143	T
3	BICF2P1332205	14779102	G	0.5	0.7143	A
3	BICF2P840661	14789546	G	0.5	0.5	A
3	TIGRP2P37772_rs8887964	14803833	A	0.5	0.75	G
3	TIGRP2P37775_rs9185133	14813424	A	0.5	0.7143	G
3	BICF2P1124139	14834366	A	0	0	G
3	TIGRP2P37784_rs8915307	14837412	A	0.5	0.7143	C
3	BICF2P729215	14848270	G	0.5	0.2857	A
3	TIGRP2P37803_rs9175644	14861433	A	0.5	0.5	C
3	BICF2P621814	14875236	G	0.5	0.5	A
3	TIGRP2P37817_rs8563529	14882121	A	0.5	0.2857	G
3	TIGRP2P37821_rs9054568	14896097	A	1	0.7857	G
3	TIGRP2P37822_rs8693004	14904525	C	0.5	0.2857	A
3	BICF2P1257029	14925319	G	0	0	A
3	BICF2P1468579	14930724	A	0.5	0.2857	G
3	TIGRP2P37847_rs9214771	14941944	G	0.5	0.2857	A
3	BICF2P1197951	14954251	A	0	0	C
3	BICF2P550345	14962091	A	0	0	G
3	BICF2P1350589	14980066	A	0	0	G
3	TIGRP2P37867_rs9085019	14995054	A	0	0	G
3	BICF2P443733	15006230	G	0	0	A
3	BICF2P983169	15013438	C	0.5	0.5	A
3	BICF2P1002948	15024671	0	0	0	A
3	TIGRP2P37876_rs8678983	15038866	0	0	0	T
3	BICF2S23528641	15044346	G	0	0	A
3	BICF2P834479	15070221	G	1	1	A
3	BICF2S22937557	15085973	G	0	0	A
3	BICF2P367069	15099144	A	1	1	G
3	TIGRP2P37888_rs9232957	15102813	A	0	0	C
3	BICF2P575140	15118998	C	0	0	A
3	TIGRP2P37911_rs9133602	15151502	G	0.5	0.5	A
3	TIGRP2P37920_rs8865094	15164324	A	0	0	G
3	BICF2P319805	15179109	0	0	0	G
3	TIGRP2P37923_rs9170596	15188459	G	0	0.2143	A
3	BICF2P245913	15196378	A	1	1	T
3	BICF2S23013284	15206454	A	0	0	G

3	BICF2P482686	15214741	G	1	1	A
3	BICF2P786284	15227152	G	0.5	0.5	A
3	BICF2P706828	15247041	A	0	0.2143	G
3	BICF2P1029008	15251459	A	0.5	0.7143	C
3	BICF2P243703	15268765	G	1	1	A
3	TIGRP2P37956_rs9082632	15275111	G	1	1	A
3	BICF2P1092722	15291114	A	0	0.2143	G
3	BICF2P336524	15295187	A	0	0.2143	G
3	BICF2P673257	15312564	0	0	0	A
3	TIGRP2P37971_rs8641504	15333472	0	0	0	A
3	TIGRP2P37982_rs8795704	15346326	G	0.5	0.5	A
3	BICF2P447670	15371954	0	0	0	A
3	BICF2P957135	15375907	T	0.5	0.5	A
3	BICF2P436367	15387074	G	0.5	0.5	A
3	BICF2P491037	15400209	A	0	0	G
3	TIGRP2P37995_rs8755294	15413657	0	0	0	G
3	BICF2S2377050	15422937	G	0	0	A
3	BICF2S23423057	15433607	A	0	0	G
3	TIGRP2P38008_rs8705495	15439503	G	0	0.2143	A
3	BICF2P424489	15446344	A	0	0.1667	G
3	BICF2P424490	15446494	G	0	0.2143	A
3	BICF2P789355	15460152	A	0	0.2143	G
3	BICF2P464724	15473084	A	0	0	C
3	BICF2P1349347	15482758	G	0	0.2143	A
3	TIGRP2P38036_rs9146894	15494909	A	0	0.2143	G
3	BICF2G630700972	15504843	0	0	0	C
3	BICF2S2355060	15520955	G	0	0.2143	A
3	BICF2P744681	15526315	G	0	0	A
3	BICF2S23128637	15549531	G	0	0.1667	A
3	BICF2S23128636	15549789	A	0	0	G
3	BICF2G630700980	15560062	C	0.5	0.5	A
3	BICF2G630700990	15573392	A	1	1	G
3	BICF2G630701005	15586044	G	1	1	A
3	BICF2G630701008	15595353	C	0	0.2143	A
3	BICF2G630701024	15609259	C	0	0.2143	A
3	BICF2G630701035	15618558	A	0	0.2143	G
3	BICF2G630701052	15630142	G	0	0.2143	A
3	BICF2G630701057	15643612	A	0	0.2143	G
3	TIGRP2P38047_rs8889799	15657166	G	0.5	0.3333	A
3	BICF2G630701064	15669428	G	0	0	A
3	BICF2G630701077	15678826	A	0	0	G
3	BICF2G630701099	15690259	T	0	0	A
3	BICF2G630701116	15704246	G	0	0	C
3	BICF2G630701126	15710578	G	1	1	A
3	BICF2P402801	15721015	A	0	0	G
3	BICF2G630701144	15732294	A	0	0	G
3	BICF2G630701178	15762607	G	0.5	0.2857	A

3	BICF2P97074	15766977	G	0	0	A
3	TIGRP2P38081_rs9190946	15784064	A	0.5	0.5	G
3	BICF2P598598	15791297	A	1	1	C
3	TIGRP2P38086_rs8767234	15805838	A	0	0.2143	G
3	BICF2G630701265	15814891	A	0	0.2143	G
3	BICF2G630701271	15824147	A	0	0	C
3	BICF2G630701275	15836679	A	0.5	0.5	C
3	BICF2G630701280	15854437	A	0.5	0.2857	C
3	BICF2S23619860	15868283	A	0	0	C
3	BICF2P1016953	15874174	G	0.5	0.2857	A
3	BICF2P1297261	15879595	A	0	0	T
3	TIGRP2P38098_rs8496083	15882608	A	0	0	G
3	BICF2S23242270	15899066	A	0.5	0.5	G
3	BICF2S23314277	15906753	A	0	0	C
3	BICF2P710687	15918810	A	0	0	G
3	BICF2P595641	15928869	0	0	0	A
3	TIGRP2P38112_rs9067919	15947008	G	0	0	A
3	TIGRP2P38118_rs8667303	15955148	A	0	0	G
3	BICF2S23042954	15969274	A	1	1	G
3	TIGRP2P38131_rs9101351	15984851	A	0	0	G
3	BICF2S23444456	15991418	A	0.5	0.2143	G
3	BICF2P869625	15999864	C	0	0.5	A
3	TIGRP2P38139_rs9021354	16008385	G	0	0	A
3	TIGRP2P38144_rs9105483	16019566	A	0	0	G
3	BICF2P357969	16037988	G	0.5	0.7857	A
3	BICF2P864287	16043247	A	0	0	G
3	BICF2P606956	16056402	G	0	0.5	A
3	BICF2P643809	16066704	G	0	0.5	A
3	BICF2P350462	16083428	A	0	0	G
3	BICF2P740970	16088549	G	0	0	A
3	BICF2P674534	16105975	G	0.5	0.7143	A
3	BICF2P455696	16111760	G	0	0	A
3	BICF2P868828	16130996	A	0	0	G
3	TIGRP2P38179_rs9093463	16137102	A	0	0	G
3	TIGRP2P38184_rs9022398	16152015	A	NA	0.875	G
3	BICF2P1165882	16163393	G	0.5	0.7143	A
3	TIGRP2P38197_rs8902626	16173747	A	1	0.5	G
3	TIGRP2P38200_rs9020103	16181606	A	1	0.5	G
3	BICF2S23120382	16198306	A	0	0.5	G
3	BICF2P256997	16209690	A	0	0	G
3	BICF2P120703	16224441	A	0.5	0.2143	C
3	BICF2P1510439	16228061	A	0.5	0.2143	G
3	BICF2P72855	16240165	A	NA	0.1	G
3	BICF2G630701313	16252554	G	0.5	0.2143	A
3	BICF2P342472	16264048	A	0.5	0.2143	G
3	BICF2G630701332	16275404	G	0.5	0.2143	A
3	TIGRP2P38224_rs8700342	16285670	0	0	0	A

3	BICF2G630701350	16297901	G	0	0	A
3	BICF2G630701358	16307641	G	0	0	A
3	BICF2G630701366	16322301	G	0	0	A
3	BICF2G630701373	16336059	A	0	0	G
3	BICF2S23240830	16352815	0	0	0	C
3	BICF2P1121302	16355254	G	1	0.5	A
3	BICF2S23025632	16382670	A	0	0	G
3	BICF2G630701430	16395011	A	0	0	G
3	BICF2G630701448	16407852	G	0	0	A
3	BICF2G630701454	16412476	G	0	0	A
3	BICF2G630701475	16432023	A	0	0	T
3	BICF2G630701477	16443933	C	0	0	A
3	BICF2G630701481	16448993	G	0	0	A
3	BICF2G630701495	16456914	A	1	0.5	C
3	BICF2G630701514	16494323	G	0	0	A
3	BICF2G630701527	16510274	G	1	0.5	A
3	BICF2G630701538	16515631	A	0	0	G
3	BICF2G630701556	16525965	G	1	0.5	A
3	BICF2G630701589	16538375	G	1	0.5	A
3	BICF2S22915658	16558921	A	1	1	G
3	BICF2P628433	16575267	0	0	0	C
3	BICF2P90664	16593361	0	0	0	G
3	TIGRP2P38234_rs8917540	16609219	0	0	0	A
3	BICF2G630701597	16628007	G	0	0	A
3	BICF2P1388375	16651590	C	0.5	0.2857	A
3	BICF2G630701602	16652435	0	0	0	A
3	BICF2G630701627	16665544	0	0	0	G
3	BICF2S23220688	16680382	A	0.5	0.7857	G
3	BICF2G630701658	16686887	C	0.5	0.7857	A
3	TIGRP2P38248_rs8968268	16701836	G	0.5	0.7857	A
3	BICF2P612973	16710369	0	0	0	G
3	BICF2G630701677	16721840	G	0	0	A
3	BICF2S22947691	16734435	G	0.5	0.7857	A
3	BICF2G630701687	16747091	A	0	0	G
3	BICF2G630701695	16789517	G	0	0	A
3	TIGRP2P38258_rs8817286	16790441	A	0.5	0.2143	G
3	BICF2G630701706	16802889	G	0.5	0.2143	A
3	BICF2S23736176	16821205	G	0.5	0.2143	A
3	BICF2S24114087	16841237	G	0.5	0.2143	A
3	BICF2G630701711	16854573	G	0.5	0.2143	A
3	BICF2G630701714	16860523	C	0	0	A
3	BICF2P1310360	16885023	G	0.5	0.1667	A
3	BICF2P905908	16901027	A	0.5	0.2143	G
3	BICF2P320638	16906165	0	0	0	C
3	BICF2G630701715	16930588	0	0	0	G
3	TIGRP2P38281_rs8816629	16946976	0	0	0	A
3	BICF2P1337044	16969949	0	0	0	C

3	TIGRP2P38282_rs9010377	16992536	0	0	0	A
3	TIGRP2P38283_rs8897540	17029727	0	0	0	A
3	BICF2P216233	17037264	A	0	0	G
3	BICF2P145991	17054184	T	0.5	0.2857	A
3	BICF2G630701724	17061906	A	1	0.5	G
3	BICF2P1395755	17070803	G	1	0.5	A
3	BICF2G630701743	17079867	A	0	0.5	C
3	BICF2G630701744	17089857	G	0	0	A
3	BICF2P1244232	17101493	C	0.5	0.2143	A
3	BICF2P40461	17117918	0	0	0	A
3	BICF2S2439627	17127453	A	0.5	0.2857	G
3	BICF2G630701747	17138300	C	0.5	0.2857	A
3	BICF2P990365	17153177	G	1	0.5	A
3	BICF2P276148	17159780	A	0	0	G
3	BICF2P421915	17173176	G	0	0	A
3	BICF2P611780	17176510	A	0	0	G
3	BICF2G630701750	17184292	A	0.5	0.7857	G
3	BICF2G630701765	17197794	G	0.5	0.2143	A
3	BICF2G630701768	17210369	G	0	0	A
3	BICF2G630701790	17222007	C	0	0	A
3	BICF2G630701806	17231092	G	0	0	A
3	BICF2G630701840	17245761	G	0	0	A
3	BICF2G630701844	17256259	C	0	0	A
3	BICF2G630701858	17265880	0	0	0	A
3	BICF2G630701877	17273888	C	1	0.5	A
3	BICF2S23453477	17285656	0	0	0	G
3	BICF2G630701953	17296351	A	0.5	0.2143	G
3	BICF2S23745471	17307748	A	0.5	0.2143	G
3	BICF2G630702025	17330829	A	0	0	G
3	BICF2G630702063	17348248	A	0	0.5	G
3	BICF2S2342009	17355972	A	0	0	G
3	BICF2P1101075	17368907	A	0	0	G
3	BICF2S22950223	17383472	A	0	0.5	G
3	BICF2P776868	17398083	A	0	0	C
3	BICF2P180728	17405705	G	0	0	A
3	BICF2P1281581	17416714	G	0.5	0.2857	A
3	BICF2P1115233	17425357	G	1	0.5	A
3	BICF2P1375792	17436361	A	1	0.5	G
3	TIGRP2P38386_rs9192128	17451518	G	1	0.5	A
3	BICF2P98993	17460549	A	0	0	G
3	BICF2P997750	17479547	A	0	0	G
3	BICF2P997752	17482017	G	0	0	A
3	BICF2G630702079	17492054	A	0	0	G
3	BICF2G630702084	17509067	A	0	0	G
3	BICF2G630702090	17515666	A	0	0	G
3	BICF2G630702114	17533787	G	0.5	0.2857	A
3	BICF2G630702118	17539242	A	0.5	0.2857	G

3	BICF2G630702119	17539378	A	0.5	0.2857	C
3	BICF2P555321	17545790	A	0	0	T
3	BICF2G630702131	17552546	C	0.5	0.2857	A
3	BICF2G630702150	17575057	0	0	0	G
3	BICF2G630702156	17585963	G	0	0	A
3	BICF2G630702157	17603451	A	0	0	G
3	BICF2P821332	17612185	G	0	0	A
3	BICF2G630702165	17621701	C	0	0	A
3	BICF2G630702179	17634897	A	0	0	G
3	BICF2P196662	17635661	G	0	0	A
3	BICF2G630702190	17647483	0	0	0	G
3	BICF2P1444739	17662566	A	1	1	G
3	BICF2P475608	17667242	G	0.5	0.2857	A
3	BICF2G630702222	17680894	A	0	0	C
3	BICF2G630702225	17689693	G	0	0	A
3	BICF2G630702234	17699781	G	0	0	A
3	BICF2G630702244	17715101	G	0	0	A
3	BICF2G630702251	17729554	A	0	0	G
3	BICF2G630702257	17737465	G	0	0	A
3	BICF2G630702265	17747067	C	0	0	A
3	BICF2P1410710	17777453	A	0	0.5	G
3	BICF2P35462	17783692	G	0	0.5	A
3	BICF2P1303390	17792179	G	0	0.5	A
3	BICF2G630702314	17804733	A	0	0.5	G
3	BICF2G630702316	17816096	A	0	0	G
3	BICF2G630702321	17829098	A	0	0.5	C
3	BICF2P557227	17841704	0	0	0	A
3	BICF2G630702329	17856464	A	0	0.5	T
3	BICF2P165111	17865317	0	0	0	G
3	BICF2G630702337	17867401	A	0.5	0.7857	G
3	BICF2G630702344	17876514	A	0	0	G
3	BICF2S23017032	17885099	C	0.5	0.7857	A
3	BICF2S23136763	17895783	A	0.5	0.2143	G
3	BICF2S2412425	17915305	A	0.5	0.2143	C
3	BICF2G630702365	17918227	G	0.5	0.7857	A
3	BICF2G630702397	17930158	A	0	0.5	G
3	BICF2G630702413	17942926	G	0.5	0.7857	A
3	BICF2G630702427	17951755	G	0.5	0.7857	A
3	BICF2P881868	17963647	A	0.5	0.7143	C
3	BICF2G630702453	17979085	A	0.5	0.7143	C
3	BICF2G630702467	17988892	0	0	0	T
3	BICF2P1116681	17998098	C	1	0.5	A
3	BICF2G630702480	18012399	0	0	0	G
3	BICF2P155967	18033212	A	0.5	0.2857	G
3	BICF2G630702501	18054602	G	0.5	0.2857	A
3	BICF2G630702504	18060114	G	0.5	0.7857	A
3	BICF2G630702516	18071770	0	0	0	A

3	BICF2G630702521	18078445	0	0	0	A
3	BICF2G630702531	18093604	G	0	0.5	A
3	BICF2G630702558	18116540	G	0	0.5	A
3	TIGRP2P38460_rs9189734	18124789	0	0	0	G
3	BICF2G630702562	18128825	G	0	0.5	A
3	BICF2G630702576	18143520	G	0	0.5	C
3	BICF2G630702582	18151730	0	0	0	A
3	BICF2G630702604	18164856	0	0	0	A
3	BICF2G630702605	18170238	0	0	0	A
3	BICF2G630702608	18181987	A	0	0	C
3	BICF2G630702622	18194758	A	0	0	G
3	BICF2G630702632	18215016	A	0	0	C
3	BICF2G630702634	18216692	A	0	0	G
3	BICF2G630702643	18234840	A	0	0	C
3	BICF2G630702649	18241874	A	0	0	G
3	BICF2G630702662	18257541	C	0	0	A
3	BICF2S232346	18272310	A	0	0	C
3	BICF2S23761366	18283572	A	0	0	G
3	BICF2G630702677	18285390	G	0	0	C
3	BICF2G630702679	18299368	0	0	0	A
3	BICF2G630702683	18314344	G	0	0	A
3	BICF2G630702685	18323618	0	0	0	A
3	BICF2P1189187	18337862	0	0	0	G
3	BICF2G630702689	18347913	0	0	0	G
3	BICF2G630702700	18374016	C	0	0	A
3	BICF2G630702703	18376467	0	0	0	A
3	BICF2P1202236	18377270	0	0	0	A
3	BICF2G630702709	18390288	A	0	0	G
3	BICF2G630702716	18410324	G	0	0	A
3	BICF2G630702723	18422733	0	0	0	G
3	BICF2G630702725	18424972	0	0	0	G
3	BICF2G630702729	18437521	0	0	0	A
3	BICF2P958229	18446407	A	0	0	G
3	BICF2P1108766	18465601	0	0	0	A
3	BICF2G630702733	18475314	G	0	0	A
3	BICF2P663455	18491402	C	0	0	A
3	BICF2G630702741	18500212	A	0	0	C
3	TIGRP2P38475_rs9037161	18512021	G	0	0	C
3	BICF2P964513	18531517	A	0	0	G
3	BICF2S2415971	18544107	0	0	0	A
3	BICF2P1088595	18552274	0	0	0	G
3	BICF2G630702755	18571735	A	0	0	G
3	TIGRP2P38479_rs8501840	18575035	A	0	0	G
3	BICF2P708685	18587977	A	0.5	0.2143	G
3	BICF2P538525	18597140	A	0	0	G
3	BICF2G630702757	18608252	A	0	0	G
3	BICF2G630702764	18620631	A	0.5	0.2143	G

3	BICF2G630702769	18634070	C	0.5	0.2857	A
3	BICF2G630702772	18653701	C	0.5	0.2143	G
3	BICF2G630702777	18674782	A	0	0	G
3	BICF2G630702779	18676158	0	0	0	A
3	BICF2G630702783	18688997	A	0.5	0.1667	C
3	BICF2G630702793	18700802	A	0.5	0.2143	G
3	BICF2P508653	18711568	A	1	0.5	G
3	BICF2G630702804	18733170	A	0.5	0.25	C
3	TIGRP2P38501_rs9194254	18734967	A	0	0.5	G
3	BICF2S23216307	18755070	G	0.5	0.2857	A
3	BICF2G630702826	18767090	A	0.5	0.2857	G
3	BICF2G630702827	18767185	A	0.5	0.2143	T
3	BICF2P544779	18772675	C	0	0.5	G
3	BICF2G630702838	18793308	A	0.5	0.2143	G
3	BICF2G630702845	18803992	A	0	0	G
3	TIGRP2P38508_rs9198208	18817367	A	0.5	0.2857	G
3	BICF2G630702869	18848381	G	0.5	0.2857	A
3	BICF2P1198038	18855030	A	0.5	0.7857	T
3	BICF2G630702882	18860517	A	0.5	0.7857	G
3	BICF2P232735	18875454	C	0	0	A
3	BICF2P323649	18887708	G	0.5	0.2143	A
3	TIGRP2P38522_rs8652558	18897187	0	0	0	G
3	BICF2S23430670	18909666	A	1	1	C
3	BICF2S2298506	18928811	A	0	0	G
3	BICF2G630702902	18939550	A	1	1	G
3	BICF2G630702903	18943264	G	1	1	A
3	BICF2G630702914	18961026	A	0.5	0.2143	C
3	BICF2G630702939	18989966	0	0	0	A
3	BICF2S22947400	19001033	G	0	0	A
3	BICF2P1331535	19031079	0	0	0	G
3	BICF2S22926697	19035805	A	0	0	G
3	BICF2P120117	19050898	A	0	0	G
3	BICF2S2316728	19060344	0	0	0	G
3	BICF2G630702944	19070601	G	0	0	A
3	BICF2P732779	19079044	G	0.5	0.7143	A
3	BICF2G630702970	19093318	A	0	0	G
3	BICF2G630702985	19109056	G	0	0	A
3	BICF2G630702988	19114662	A	0	0	G
3	BICF2P1365344	19130129	A	0	0	G
3	BICF2S23138900	19146081	A	0	0	C
3	BICF2P603794	19147996	G	0	0	A
3	TIGRP2P38558_rs9053658	19162081	0	0	0	A
3	TIGRP2P38560_rs9237046	19171730	A	0	0	G
3	BICF2G630703005	19184563	0	0	0	A
3	BICF2G630703011	19200927	0	0	0	G
3	BICF2G630703020	19206533	A	0	0	G
3	BICF2G630703030	19216517	G	0	0	A

3	BICF2G630703056	19237173	0	0	0	G
3	BICF2G630703061	19239561	0	0	0	A
3	BICF2G630703095	19256245	0	0	0	C
3	BICF2G630703124	19276605	0	0	0	A
3	BICF2G630703137	19285614	0	0	0	G
3	BICF2G630703151	19303013	C	0.5	0.25	A
3	BICF2P841378	19312341	G	1	0.5	A
3	BICF2G630703160	19317603	A	0	0.5	C
3	BICF2G630703163	19320397	G	0	0	A
3	BICF2P624502	19338973	C	0.5	0.2857	A
3	BICF2S23057764	19345993	A	0.5	0.7143	G
3	BICF2P131093	19354772	A	0	0	G
3	BICF2G630703165	19376268	A	0	0	G
3	TIGRP2P38581_rs9060853	19378023	G	0.5	0.7143	A
3	BICF2G630703186	19398025	A	0.5	0.2143	G
3	BICF2G630703200	19405029	G	0	0	A
3	BICF2G630703208	19418101	A	0.5	0.2143	G
3	BICF2G630703215	19426646	G	0	0	A
3	BICF2G630703225	19437944	A	0	0	G
3	BICF2G630703238	19447081	A	1	1	G
3	BICF2G630703240	19461083	A	0	0	C
3	BICF2G630703242	19470024	G	0.5	0.2857	A
3	BICF2G630703245	19482991	G	0	0	A
3	BICF2G630703259	19496345	A	0.5	0.2143	G
3	BICF2G630703266	19506869	A	0	0	G
3	BICF2P795472	19518958	G	0	0	A
3	BICF2P866954	19538428	G	0	0	A
3	BICF2P777955	19542668	G	0	0	A
3	BICF2G630703308	19559403	A	0.5	0.2857	G
3	BICF2P1431252	19568917	A	0	0	G
3	BICF2G630703343	19585617	G	0.5	0.2857	A
3	BICF2P152793	19586308	A	0	0	C
3	BICF2G630703354	19596475	A	0	0	G
3	BICF2G630703376	19611889	G	0	0	A
3	BICF2G630703383	19629578	C	0.5	0.2857	A
3	BICF2P731705	19635495	A	0.5	0.2857	G
3	BICF2G630703403	19646292	G	0.5	0.2857	A
3	BICF2G630703436	19679898	G	0	0	A
3	BICF2P676761	19689532	G	0.5	0.2857	A
3	BICF2G630703452	19722496	A	1	0.5	G
3	G219f30S242	19743758	A	0	0	G
3	BICF2G630703453	19763578	A	0.5	0.7857	G
3	BICF2G630703457	19769884	C	NA	1	A
3	BICF2G630703480	19782527	A	0.5	0.7857	G
3	BICF2G630703499	19802024	C	0.5	0.2857	A
3	BICF2G630703507	19809004	A	1	0.5	G
3	BICF2G630703528	19815216	A	0.5	0.2143	G

3	BICF2G630703540	19833615	G	0.5	0.2857	A
3	BICF2P819277	19848981	A	0	0	G
3	BICF2P425048	19849663	A	0	0	G
3	TIGRP2P38645_rs8733900	19870660	A	0.5	0.2857	G
3	TIGRP2P38654_rs9030381	19879973	A	0.5	0.2143	G
3	BICF2P563532	19884385	G	0.5	0.2857	A
3	BICF2P912159	19895823	A	0	0	C
3	TIGRP2P38668_rs9181658	19914811	G	0	0	A
3	BICF2S23432488	19919299	G	0	0.5	A
3	BICF2S23024424	19933754	G	1	0.5	A
3	BICF2S23228062	19942977	A	0	0	G
3	BICF2G630703558	19953151	A	0	0	G
3	TIGRP2P38679_rs8571535	19971122	0	0	0	C
3	BICF2P896362	19976901	A	0	0	G
3	BICF2G630703591	19995818	0	0	0	G
3	BICF2P1021592	20000524	A	0	0	G
3	BICF2G630703598	20017067	A	0	0	G
3	BICF2G630703620	20026519	G	0.5	0.2143	A
3	BICF2P1413618	20033803	A	0.5	0.2857	G
3	BICF2G630703633	20050549	A	0	0	G
3	BICF2G630703638	20056675	G	0	0	A
3	BICF2G630703641	20069096	G	0	0.5	A
3	BICF2G630703657	20089275	C	0.5	0.25	A
3	TIGRP2P38714_rs8963275	20097715	A	0.5	0.2857	G
3	BICF2P728993	20112571	A	0.5	0.2857	G
3	BICF2P728990	20113630	G	0.5	0.2143	A
3	BICF2P403384	20132409	0	0	0	A
3	BICF2P1385868	20137858	G	0	0	A
3	BICF2G630703694	20149828	G	0.5	0.2143	A
3	BICF2G630703710	20160185	G	0	0	A
3	BICF2P177556	20174664	A	0.5	0.7857	C
3	BICF2P535618	20185982	A	0	0	C
3	BICF2P685831	20197492	A	0.5	0.7857	C
3	BICF2P68391	20209435	A	0.5	0.7857	G
3	BICF2P916407	20220143	0	0	0	A
3	BICF2P769583	20228640	0	0	0	A
3	BICF2P252925	20247172	G	0	0	A
3	BICF2P542982	20262267	0	0	0	G
3	BICF2G630703735	20295114	A	0.5	0.2143	T
3	BICF2G630703738	20297665	G	0.5	0.2143	A
3	BICF2G630703741	20318499	G	0	0	A
3	BICF2G630703743	20322930	C	0	0	A
3	BICF2G630703754	20335945	G	0.5	0.2143	A
3	BICF2G630703760	20344707	G	0	0	A
3	BICF2G630703764	20356280	A	0.5	0.2143	G
3	BICF2G630703765	20359373	G	0.5	0.2143	A
3	TIGRP2P38769_rs8773291	20375206	G	0	0	A

3	BICF2G630703792	20385990	C	0	0	A
3	BICF2G630703795	20400908	A	0	0	G
3	BICF2G630703800	20406716	A	0	0	G
3	BICF2G630703813	20423886	A	0	0	G
3	BICF2G630703815	20431385	G	0.5	0.2143	A
3	BICF2G630703820	20438830	A	0.5	0.2143	G
3	BICF2G630703826	20451249	G	0.5	0.2143	A
3	BICF2G630703828	20464761	G	0.5	0.2143	A
3	BICF2G630703833	20471442	G	0.5	0.2143	A
3	BICF2P1156514	20487166	0	0	0	G
3	BICF2P243389	20501382	0	0	0	A
3	BICF2G630703836	20505717	C	0.5	0.2143	A
3	BICF2G630703840	20525151	A	0	0	G
3	BICF2G630703842	20529992	A	0.5	0.2143	G
3	BICF2G630703872	20556036	0	0	0	C
3	BICF2P392704	20571421	A	0.5	0.2143	C
3	BICF2G630703881	20581297	A	0.5	0.2143	G
3	BICF2G630703888	20590954	A	0.5	0.2143	G
3	BICF2G630703896	20607438	0	0	0	C
3	BICF2G630703897	20610522	0	0	0	G
3	BICF2P696038	20623665	A	0.5	0.7143	G
3	BICF2P820316	20635937	G	0.5	0.7143	A
3	BICF2P107454	20646227	G	0.5	0.7143	A
3	BICF2P1425768	20655511	A	0	0.5	G
3	BICF2G630703914	20665913	0	0	0	A
3	BICF2G630703916	20680798	A	0	0.5	G
3	BICF2P1140878	20698806	G	0.5	0.7143	A
3	BICF2G630703924	20715001	C	0	0.5	A
3	BICF2P1377213	20749552	A	0	0	G
3	BICF2S24413706	20760778	A	0.5	0.2143	G
3	BICF2G630703937	20782197	G	0	0	A
3	BICF2S23634771	20792649	0	0	0	G
3	BICF2P862831	20813300	0	0	0	A
3	BICF2P1024676	20822329	G	0.5	0.7143	A
3	BICF2P1171512	20828633	0	0	0	C
3	BICF2G630703940	20843210	A	0.5	0.7143	G
3	BICF2G630703951	20851767	A	0.5	0.7143	C
3	BICF2P435096	20866372	A	0	0	G
3	BICF2S23155225	20882541	A	0.5	0.7143	G
3	BICF2P100686	20885173	G	0	0.5	A
3	BICF2P785425	20901638	A	1	0.5	G
3	BICF2P823068	20916763	G	1	0.5	A
3	TIGRP2P38827_rs8662461	20924483	G	0	0.5	A
3	BICF2G630703970	20933738	0	0	0	G
3	BICF2S23661029	20948299	A	0	0.5	G
3	BICF2S2373643	20959505	A	0.5	0.7143	G
3	BICF2P674253	20966022	0	0	0	A

3	BICF2P1311415	20974420	G	0.5	0.2143	A
3	BICF2P926553	20985086	G	1	0.5	A
3	BICF2G630703974	20992168	A	0	0	C
3	BICF2G630703986	21007121	G	0	0	A
3	BICF2G630703990	21015972	G	0	0	A
3	BICF2G630703997	21025756	G	0	0	A
3	BICF2G630704003	21034016	C	0.5	0.2143	A
3	BICF2G630704005	21046428	0	0	0	A
3	BICF2G630704022	21072440	C	0	0	A
3	BICF2G630704025	21083034	G	0.5	0.2143	A
3	BICF2G630704031	21093302	G	0	0	A
3	BICF2G630704040	21105681	G	0	0	A
3	BICF2G630704051	21123015	C	0.5	0.2143	A
3	BICF2P445420	21134775	G	0	0.5	A
3	BICF2G630704069	21140138	G	0.5	0.7143	A
3	BICF2G630704070	21141328	G	0.5	0.2143	A
3	BICF2G630704084	21156688	G	0.5	0.2143	A
3	BICF2G630704088	21160115	C	0.5	0.7143	A
3	BICF2S23161534	21180618	A	0.5	0.2143	T
3	BICF2G630704092	21184276	C	0.5	0.2143	A
3	BICF2G630704093	21197733	G	0.5	0.2143	A
3	BICF2G630704099	21209308	G	0.5	0.2143	A
3	BICF2G630704115	21220106	G	0.5	0.2143	A
3	BICF2G630704126	21230171	G	0.5	0.2143	A
3	BICF2G630704131	21269377	G	0	0.5	A
3	BICF2G630704143	21275535	0	0	0	T
3	BICF2P1104599	21281629	G	0.5	0.7143	A
3	TIGRP2P38864_rs8590289	21296387	G	0	0	A
3	TIGRP2P38865_rs8711012	21298503	0	0	0	G
3	BICF2P594612	21317307	A	0	0	G
3	TIGRP2P38874_rs8636589	21325130	A	0	0	T
3	BICF2P1386054	21334310	0	0	0	G
3	TIGRP2P38882_rs9075166	21344514	0	0	0	A
3	BICF2S2337717	21386068	0	0	0	A
3	BICF2P1076691	21423953	0	0	0	A
3	BICF2P1003709	21425680	0	0	0	A
3	BICF2P1027258	21437655	A	0	0	T
3	BICF2P614287	21475341	0	0	0	T
3	BICF2S23218931	21520061	0	0	0	A
3	BICF2P118782	21532796	0	0	0	A
3	BICF2P1087899	21545394	0	0	0	A
3	BICF2P812543	21554735	0	0	0	C
3	BICF2P874126	21577483	0	0	0	A
3	BICF2P1067466	21595904	0	0	0	A
3	BICF2P1438162	21620707	0	0	0	G
3	BICF2S23547902	21650840	0	0	0	G
3	BICF2P1307535	21663645	G	0.5	0.2857	A

3	TIGRP2P38902_rs8823003	21751044	0	0	0	A
3	BICF2P1006434	21785268	0	0	0	A
3	G223f34S128	21805856	0	0	0	C
3	BICF2P1472450	21830184	0	0	0	A
3	BICF2P318427	21839851	0	0	0	G
3	BICF2P797664	21882272	G	0	0.5	A
3	BICF2P1213922	21886247	0	0	0	A
3	BICF2P147784	21952562	0	0	0	A
3	BICF2P117121	21956046	0	0	0	A
3	TIGRP2P38908_rs9091489	21982181	0	0	0	A
3	BICF2P571131	21997907	A	0	0.5	G
3	BICF2P843931	22004586	C	0	0.5	A
3	BICF2P1172002	22020263	A	0	0	G
3	BICF2P1172003	22020354	G	0	0	A
3	BICF2P1506703	22022910	0	0	0	C
3	BICF2S23753620	22035624	A	0.5	0.7143	G
3	BICF2P695830	22047890	C	0.5	0.7143	A
3	TIGRP2P38910_rs8697050	22059384	0	0	0	A
3	BICF2P1398376	22078534	G	0	0.5	A
3	BICF2P1470119	22089159	A	0	0	G
3	TIGRP2P38921_rs8642210	22100011	A	0	0.5	G
3	TIGRP2P38932_rs8676347	22111274	A	0	0.5	G
3	BICF2S23641595	22115038	A	0	0.5	G
3	TIGRP2P38936_rs8732510	22127553	C	0.5	0.7143	A
3	TIGRP2P38939_rs8780953	22138508	A	0	0	G
3	BICF2P136332	22154425	G	0	0	A
3	BICF2S2292282	22169108	A	0	0	C
3	BICF2P856574	22203131	0	0	0	A
3	BICF2S23646139	22215865	G	0	0	A
3	TIGRP2P38948_rs8717770	22227209	A	0	0	G
3	BICF2S23353083	22233338	C	0	0	A
3	BICF2P941736	22252200	A	0	0	G
3	BICF2P526597	22266555	G	0	0	A
3	BICF2S23318678	22278940	C	0.5	0.2143	A
3	BICF2P1189059	22287397	0	0	0	G
3	BICF2P1271138	22304781	A	0.5	0.2143	G
3	BICF2P875275	22310878	A	0.5	0.2143	G
3	BICF2S23634710	22324424	G	0.5	0.2143	A
3	BICF2P1080217	22338139	G	0.5	0.2143	A
3	BICF2P842711	22350904	0	0	0	C
3	BICF2P589379	22399723	0	0	0	A
3	BICF2S23351315	22409332	G	0.5	0.2143	A
3	BICF2P991877	22415198	0	0	0	C
3	BICF2P144179	22452113	0	0	0	G
3	BICF2S2311484	22468897	G	0.5	0.7143	A
3	BICF2P1157776	22475279	G	0.5	0.7143	A
3	BICF2P353546	22482943	A	0.5	0.1667	G

3	BICF2P885942	22497674	G	0.5	0.2143	A
3	BICF2S22916431	22509566	G	0.5	0.7857	A
3	BICF2S23244437	22521287	G	0.5	0.7857	A
3	BICF2P453997	22534410	0	0	0	C
3	BICF2P1283972	22548586	G	0	0.5	A
3	BICF2S23117369	22556229	A	0	0	G
3	BICF2P1312271	22570040	0	0	0	G
3	BICF2S23251848	22576872	G	0	0	A
3	BICF2P388477	22595205	A	0	0	G
3	BICF2P268805	22601694	0	0	0	A
3	TIGRP2P38992_rs9084574	22613040	0	0	0	A
3	TIGRP2P38993_rs8657891	22629829	0	0	0	C
3	BICF2S23539164	22634605	A	1	0.5	G
3	BICF2P368655	22653761	A	1	0.5	G
3	BICF2P1110565	22665388	G	0	0	A
3	BICF2P493449	22667324	G	1	0.5	A
3	BICF2P446530	22687977	G	0	0	A
3	BICF2P739271	22691584	A	0	0	G
3	BICF2P492922	22712913	A	0	0	G
3	BICF2P828459	22728672	C	0	0.5	A
3	BICF2P119003	22736566	0	0	0	A
3	BICF2S2334492	22752574	A	0	0.5	G
3	BICF2P458690	22769546	C	0	0.5	A
3	BICF2S23721624	22779312	G	0	0	A
3	BICF2P421448	22799244	A	0	0.5	C
3	BICF2P1163892	22809511	G	0	0.5	A
3	BICF2P1098056	22819966	0	0	0	C
3	BICF2S22946718	22833598	G	0	0.5	A
3	TIGRP2P38997_rs8659554	22839513	G	0.5	0.2143	A
3	BICF2P331235	22860679	A	0	0	G
3	BICF2P264333	22867119	A	0	0	G
3	TIGRP2P39005_rs8875213	22874783	A	0.5	0.25	G
3	BICF2P1134009	22895752	A	0.5	0.7857	G
3	TIGRP2P39017_rs9159504	22902597	A	0.5	0.7857	G
3	TIGRP2P39020_rs8933801	22912231	G	0.5	0.2143	A
3	TIGRP2P39022_rs8924216	22920062	0	0	0	C
3	TIGRP2P39029_rs8914036	22932700	A	0	0	C
3	BICF2P961290	22944739	C	0	0	A
3	BICF2P508378	22958540	A	0.5	0.2143	G
3	BICF2P1057376	22964006	A	0	0	G
3	BICF2P1118886	22975275	C	0.5	0.7857	A
3	BICF2S23912901	22987520	C	0	0	A
3	BICF2P349656	22989086	A	0	0	G
3	BICF2P591392	23000723	A	0.5	0.7857	G
3	TIGRP2P39064_rs8862137	23013823	G	0	0	A
3	BICF2P928359	23028486	0	0	0	G
3	BICF2P875580	23039291	A	0.5	0.7857	C

3	BICF2S23136277	23046664	A	0	0	C
3	BICF2P580740	23075598	C	0	0	A
3	BICF2P1243785	23089527	A	0	0	G
3	BICF2P222513	23095289	G	0	0	A
3	BICF2P865031	23104667	A	0	0	G
3	BICF2P521685	23118440	A	0	0	G
3	BICF2P468650	23136488	G	0	0	A
3	BICF2P351775	23145729	0	0	0	G
3	BICF2P351776	23146400	A	0.5	0.2857	G
3	BICF2S2398047	23158204	G	0	0	A
3	BICF2P246825	23164254	T	1	0.5	A
3	BICF2P1385718	23176116	A	0.5	0.1667	C
3	BICF2P623268	23186337	A	0.5	0.2143	G
3	TIGRP2P39115_rs8718367	23218241	C	0.5	0.2857	A
3	BICF2P701091	23221275	C	0.5	0.2143	A
3	BICF2P1126860	23230988	A	0.5	0.2143	T
3	BICF2P1033453	23253145	A	0.5	0.2143	C
3	BICF2P173125	23268139	G	0.5	0.2143	A
3	BICF2P270580	23280595	G	0.5	0.2143	A
3	BICF2P169169	23297707	C	0.5	0.2143	A
3	BICF2S23229652	23303439	G	0.5	0.2143	A
3	TIGRP2P39125_rs9060488	23328391	G	0.5	0.2857	A
3	BICF2S23146185	23332280	A	0.5	0.25	C
3	BICF2S23146186	23332370	A	0.5	0.2857	T
3	BICF2S23146626	23336179	C	0.5	0.2857	A
3	BICF2P888889	23351158	G	0	0	A
3	BICF2P856017	23358440	0	0	0	A
3	BICF2P1030690	23368786	G	0.5	0.7857	C
3	TIGRP2P39148_rs8905390	23379691	0	0	0	G
3	BICF2P122888	23391906	A	0.5	0.2143	G
3	BICF2P465473	23416078	0	0	0	C
3	TIGRP2P39152_rs9235522	23435786	A	0	0	G
3	BICF2S23162032	23447664	C	0	0	G
3	BICF2S23162439	23450903	C	0	0	A
3	BICF2P867663	23461780	A	0.5	0.7857	G
3	BICF2P1035475	23476220	G	0.5	0.7857	A
3	TIGRP2P39158_rs8540981	23483305	A	0.5	0.9	C
3	BICF2S23221577	23501494	G	0.5	0.2143	C
3	BICF2S23617513	23510970	G	0.5	0.2143	A
3	TIGRP2P39169_rs8775070	23521461	0	0	0	G
3	BICF2P1410994	23542679	G	0	0	A
3	BICF2S23232383	23557152	A	0	0.5	G
3	BICF2P200162	23564544	A	0	0	G
3	BICF2P329448	23579446	G	0	0	A
3	BICF2P1031182	23589110	C	0	0	A
3	TIGRP2P39195_rs9227814	23610377	A	0	0	G
3	BICF2P845619	23634880	0	0	0	A

3	BICF2P129403	23647980	0	0	0	A
3	BICF2P123477	23665006	G	0.5	0.2143	A
3	BICF2S2453627	23682958	0	0	0	G
3	BICF2P886939	23694168	A	0.5	0.1667	C
3	BICF2P307209	23703493	C	0.5	0.2143	G
3	BICF2P1161338	23723692	A	0.5	0.1667	G
3	TIGRP2P39240_rs9184695	23724971	G	0.5	0.2143	C
3	BICF2P167222	23754738	C	0	0	A
3	BICF2P1196530	23774455	0	0	0	G
3	G226f18S241	23801585	0	0	0	A
3	BICF2P663267	23832907	A	1	0.5	G
3	G227f19S258	23855145	0	0	0	C
3	TIGRP2P39268_rs8817594	23878651	C	0	0	A
3	BICF2P419928	23902242	0	0	0	A
3	BICF2S23050028	23914226	A	0	0	C
3	BICF2S23354206	23934516	A	0	0	G
3	BICF2S2417245	23949758	A	0	0.5	G
3	TIGRP2P39281_rs9092970	23962165	C	1	1	A
3	BICF2S23035138	24006708	A	0.5	0.2857	G
3	G228f27S159	24030289	0	0	0	G
3	BICF2S23027842	24055553	T	0.5	0.2857	A
3	BICF2S23441320	24063446	G	0	0	A
3	BICF2S23619319	24070901	0	0	0	G
3	BICF2P360795	24098583	A	0	0	G
3	BICF2P712561	24112831	C	0	0	A
3	BICF2S23513533	24120137	A	1	1	G
3	BICF2S23437075	24134024	A	0	0	G
3	BICF2P1146146	24148101	G	0.5	0.25	A
3	BICF2S22938778	24150654	A	0	0	C
3	BICF2S23529877	24163528	G	0.5	0.2143	A
3	TIGRP2P39300_rs8860106	24175230	A	0	0	G
3	TIGRP2P39302_rs8633338	24185742	G	1	1	A
3	BICF2P409071	24202023	G	0.5	0.8	A
3	TIGRP2P39318_rs8715142	24213375	A	0.5	0.2143	C
3	TIGRP2P39320_rs8747089	24230305	0	0	0	G
3	BICF2S23634134	24251866	A	0	0	G
3	BICF2S23631893	24256563	G	0.5	0.2143	A
3	TIGRP2P39336_rs9111755	24275325	G	0.5	0.7143	A
3	TIGRP2P39343_rs9086022	24287342	A	0	0	G
3	BICF2S23650397	24302364	0	0	0	G
3	BICF2P880355	24315938	A	0.5	0.7143	G
3	BICF2S23439285	24332542	A	0.5	0.7143	G
3	BICF2P1047463	24335637	A	0.5	0.7143	G
3	BICF2P957161	24348731	A	0.5	0.2857	G
3	BICF2S2444899	24359096	A	0.5	0.7143	G
3	BICF2P643688	24361777	A	0.5	0.7143	G
3	BICF2P1250319	24373394	G	0	0	A

3	BICF2P276691	24399421	G	0.5	0.7143	C
3	BICF2P1017324	24419595	A	0.5	0.75	G
3	BICF2P400723	24433978	A	0.5	0.7143	C
3	BICF2P525257	24437877	G	0.5	0.7143	C
3	TIGRP2P39383_rs8689514	24449994	C	0.5	0.7143	A
3	BICF2S23515454	24466694	A	0.5	0.7143	C
3	TIGRP2P39394_rs9029032	24478755	A	0.5	0.2857	G
3	TIGRP2P39403_rs8797790	24486280	G	0.5	0.7143	A
3	BICF2S24110294	24509461	C	0	0.5	A
3	TIGRP2P39421_rs9214502	24525024	0	0	0	G
3	BICF2P359531	24552875	0	0	0	G
3	BICF2P1308230	24589115	A	0.5	0.2857	G
3	TIGRP2P39480_rs8971143	24598864	G	0.5	0.2857	A
3	BICF2P675091	24631026	A	0.5	0.2857	G
3	BICF2S2329711	24653969	G	0	0.5	A
3	BICF2S23414921	24662231	A	0	0.5	G
3	TIGRP2P39492_rs8526946	24695270	0	0	0	A
3	TIGRP2P39493_rs8596531	24702582	A	0	0.5	G
3	BICF2S23621295	24717744	G	0	0.5	A
3	BICF2S2318793	24728840	A	0	0.5	G
3	BICF2S23132141	24748340	G	0	0.5	A
3	BICF2P50041	24781908	A	0	0	G
3	BICF2P655153	24816289	G	0	0.5	C
3	BICF2P1369888	24847947	A	0	0.5	G
3	BICF2S2359279	24852592	G	0	0.5	A
3	BICF2P220833	24868090	0	0	0	A
3	BICF2S23129534	24919255	G	0	0.5	C
3	BICF2P258371	24962616	0	0	0	A
3	BICF2P263159	24975784	0	0	0	C
3	BICF2S23640689	25012387	0	0	0	G
3	BICF2S23640225	25016620	0	0	0	A
3	BICF2P422973	25105023	0	0	0	A
3	G229f51S198	25134153	G	0	0	A
3	BICF2P968932	25161507	0	0	0	C
3	G230f36S128	25186657	G	0	0	A
3	BICF2S23256653	25205032	0	0	0	G
3	BICF2S23256268	25209199	0	0	0	C
3	BICF2P500949	25241271	0	0	0	A
3	BICF2P360758	25263247	G	0	0	A
3	BICF2S23636912	25288291	A	0	0	G
3	BICF2P701856	25290132	C	0	0.5	G
3	G231f40S141	25310956	A	0	0	G
3	G231f42S85	25312108	T	0	0	A
3	BICF2S2458889	25325038	G	0	0	A
3	BICF2P407379	25346499	G	0	0	A
3	G232f29S243	25376520	G	0	0	A
3	BICF2P202105	25398744	0	0	0	A

3	BICF2P1471305	25406842	0	0	0	A
3	G233f29S171	25427121	A	0.5	0.2857	C
3	BICF2P1362697	25465539	0	0	0	A
3	BICF2S23751151	25477939	A	NA	0.25	G
3	TIGRP2P39533_rs8704484	25492353	0	0	0	G
3	BICF2S23046425	25503305	G	0	0	A
3	BICF2S2414722	25508635	G	0	0	A
3	BICF2P553191	25537161	C	0	0	A
3	G234f22S297	25567037	0	0	0	A
3	BICF2P1414674	25596301	G	0	0	A
3	BICF2S23911804	25620349	G	0	0.5	A
3	BICF2S23324938	25630966	A	0	0.5	G
3	BICF2P230811	25647436	0	0	0	G
3	BICF2S23259979	25656748	C	0	0.5	A
3	BICF2P389416	25679327	G	0	0.5	A
3	BICF2P328536	25682735	G	0	0.5	A
3	BICF2P1464927	25698880	A	0	0.5	G
3	BICF2P184676	25709203	G	0	0.5	A
3	BICF2P262945	25722340	A	0	0.5	C
3	BICF2P1121278	25727109	A	0	0.5	G
3	BICF2S23032154	25747407	G	0	0.5	A
3	BICF2P1153944	25754784	A	0	0	G
3	BICF2P131960	25790634	0	0	0	G
3	BICF2P1097594	25809495	0	0	0	A
3	BICF2S23655675	25825838	G	0	0	A
3	BICF2P1017532	25861389	G	0	0	A
3	TIGRP2P39575_rs8909431	25882960	0	0	0	G
3	BICF2S2416461	25891108	0	0	0	C
3	BICF2P1468746	25915104	G	0	0	A
3	BICF2P954138	25921719	0	0	0	C
3	BICF2P969157	25941336	0	0	0	A
3	BICF2P31847	25958904	A	0	0	G
3	BICF2S23130561	25996364	A	0.5	0.2857	G
3	BICF2P1130908	26040200	0	0	0	A
3	BICF2S23545557	26048569	G	0	0	C
3	BICF2P949469	26091733	A	0	0	G
3	BICF2S23535906	26101214	A	0	0	G
3	BICF2P992327	26135807	G	0	0	A
3	BICF2S2357429	26140385	G	0	0	A
3	BICF2S23738401	26159968	C	1	1	A
3	BICF2P973502	26166373	A	0	0	G
3	BICF2P1257926	26180810	A	0	0.5	G
3	BICF2P1506502	26191120	A	0	0	G
3	BICF2P618822	26212315	C	0	0	A
3	BICF2P1013068	26220527	A	0	0	G
3	BICF2P525869	26245187	A	0	0	G
3	BICF2P1393990	26257943	G	1	1	A

3	BICF2P506304	26269363	G	0	0.5	A
3	BICF2S2293842	26280693	A	0	0	G
3	BICF2P386807	26290081	C	0	0.5	A
3	BICF2S23111278	26309064	A	0.5	0.7143	G
3	BICF2S23447985	26316810	A	0.5	0.2857	G
3	BICF2P19481	26336975	G	0	0.5	A
3	BICF2P634314	26363858	A	NA	0.8	G
3	BICF2P926140	26369859	A	0	0	C
3	TIGRP2P39585_rs8570590	26389159	0	0	0	C
3	BICF2P557863	26399191	A	0.5	0.2143	G
3	BICF2S23049495	26404558	G	0.5	0.7857	A
3	BICF2P1056346	26420133	A	0	0	T
3	BICF2P1414108	26427382	A	0	0	G
3	BICF2P815348	26444974	A	0	0	G
3	BICF2P904305	26454924	G	0	0	A
3	BICF2P928905	26466190	A	0	0	C
3	BICF2P1109448	26473163	A	0	0	G
3	BICF2P162683	26485574	G	0	0	A
3	BICF2P247820	26543188	0	0	0	A
3	BICF2S23661479	26557956	C	0	0	G
3	BICF2P839951	26565799	G	0	0	A
3	BICF2P230345	26580009	0	0	0	A
3	BICF2P892935	26590717	G	0	0	A
3	BICF2P323914	26604337	G	0.5	0.7143	A
3	BICF2P1272068	26617696	0	0	0	A
3	BICF2P305031	26629323	G	0.5	0.7143	A
3	BICF2P906090	26634672	A	0.5	0.7143	G
3	BICF2P487494	26654672	A	0.5	0.7143	G
3	BICF2S23020974	26662634	A	0.5	0.7143	C
3	BICF2P795793	26676334	0	0	0	A
3	BICF2P838944	26687529	0	0	0	G
3	BICF2P71679	26700198	G	0.5	0.7143	A
3	BICF2P1222117	26723056	G	0.5	0.7857	A
3	BICF2P1026265	26735267	0	0	0	G
3	BICF2P104847	26738656	A	0.5	0.2143	G
3	BICF2P1336383	26749713	G	1	0.5	A
3	BICF2S23450252	26760791	G	1	0.5	A
3	BICF2P1387536	26779452	A	1	0.5	G
3	BICF2S23144247	26793760	G	1	1	A
3	BICF2P804534	26796016	A	1	1	G
3	BICF2P213239	26819038	0	0	0	T
3	BICF2P170026	26839490	G	0	0	C
3	BICF2P786294	26854647	C	0.5	0.2857	G
3	BICF2S23639760	26877172	0	0	0	G
3	BICF2P433362	26911997	A	0	0.5	C
3	BICF2P1093928	26927473	G	0	0.5	C
3	BICF2P484725	26929046	T	0	0.5	A

3	BICF2P707393	26937767	G	0	0.5	A
3	BICF2P398233	26940248	G	1	1	A
3	BICF2P882496	26952327	G	0	0.5	A
3	BICF2P522592	26957127	A	0	0.5	G
3	BICF2P1190	26969440	0	0	0	A
3	BICF2S23734564	27000437	A	0	0.5	T
3	BICF2S23537285	27002632	A	1	0.5	G
3	BICF2P2488	27019426	A	0	0	G
3	BICF2P1299857	27031306	C	0	0.5	A
3	BICF2P522372	27037426	G	1	0.5	A
3	BICF2P1188893	27049642	A	1	0.5	G
3	BICF2S23143268	27062291	G	0	0	A
3	BICF2P801025	27072503	A	0	0.5	G
3	BICF2S23251194	27104661	A	0	0.5	G
3	BICF2P965703	27107341	0	0	0	A
3	BICF2P289076	27124402	C	0	0.5	A
3	BICF2P1431987	27131195	A	0	0	G
3	BICF2P548607	27141226	0	0	0	A
3	BICF2S22943694	27152587	C	0	0.5	G
3	BICF2P455281	27169426	A	0	0	G
3	BICF2S23445983	27185563	G	0	0.5	A
3	BICF2S23434272	27203016	0	0	0	G
3	BICF2P879160	27229187	0	0	0	A
3	BICF2S23729794	27237519	A	0	0.5	G
3	BICF2P762254	27246859	0	0	0	A
3	BICF2P904963	27263263	0	0	0	G
3	BICF2P1420556	27266969	0	0	0	A
3	BICF2P1410153	27282736	0	0	0	A
3	BICF2P606301	27303479	0	0	0	C
3	BICF2P1217355	27321334	0	0	0	G
3	BICF2P1294181	27348322	0	0	0	A
3	BICF2P343919	27409442	0	0	0	A
3	BICF2P1129447	27430930	0	0	0	G
3	BICF2P502138	27440789	0	0	0	A
3	BICF2P243808	27455289	0	0	0	G
3	BICF2S23625268	27465689	G	0	0	A
3	BICF2S2394791	27476104	G	0	0.5	A
3	BICF2P673244	27492245	A	0	0	G
3	BICF2S22961979	27496567	G	0	0.5	A
3	BICF2S2357596	27522282	A	1	0.5	G
3	BICF2S23222621	27537681	A	0	0	T
3	BICF2S2398146	27553186	0	0	0	A
3	BICF2P104790	27564416	A	0	0	T
3	BICF2P259318	27576863	G	1	0.5	A
3	BICF2P390967	27586885	C	1	0.5	A
3	BICF2P390970	27587058	A	1	0.5	G
3	BICF2P366133	27589192	A	1	0.5	G

3	BICF2P46038	27602064	0	0	0	A
3	BICF2P923955	27621820	G	0	0	A
3	BICF2S23715427	27649262	A	0	0	G
3	BICF2S2301082	27659324	G	0	0.5714	A
3	BICF2P876619	27706926	C	1	0.4286	A
3	BICF2S23325523	27715423	A	0	0	G
3	BICF2P72852	27729965	C	0	0	A
3	BICF2P792851	27742581	A	0	0	G
3	BICF2P286736	27749630	G	0	0	A
3	BICF2S23025088	27769218	A	0	0	T
3	BICF2P833680	27778332	G	0.5	0.1429	A
3	BICF2S23040767	27788552	A	0	0	G
3	BICF2P901689	27804609	A	0	0	G
3	BICF2P449184	27808018	G	0.5	0.1429	A
3	BICF2P386907	27833506	A	0.5	0.1429	G
3	BICF2P1182396	27846683	0	0	0	G
3	BICF2P206483	27861785	G	1	0.4286	A
3	BICF2S23225359	27869091	C	0	0	G
3	BICF2S23316944	27883232	A	0	0	T
3	BICF2P826176	27895457	0	0	0	A
3	BICF2S23650428	27905563	0	0	0	A
3	BICF2S23014760	27917659	A	0	0.5714	G
3	BICF2P322726	27923254	0	0	0	G
3	BICF2P496541	27942286	G	0	0	A
3	BICF2P143014	27952916	A	0	0	G
3	BICF2S23752096	27958271	A	0	0	G
3	BICF2P830671	27972664	C	0	0.5714	A
3	BICF2P1020933	27987015	A	0	0.5714	G
3	BICF2S23523257	27996340	A	0	0.5714	G
3	BICF2P370771	28005305	A	0	0.5714	G
3	BICF2P638308	28014995	G	0.5	0.1429	A
3	BICF2S23615024	28031123	0	0	0	A
3	BICF2S23348565	28041026	A	0	0.6	G
3	BICF2P1241730	28054319	G	0.5	0.7143	A
3	BICF2P75457	28064273	0	0	0	A
3	BICF2P1228706	28073814	A	0	0	G
3	BICF2S23735831	28084913	A	0.5	0.1429	G
3	BICF2S23428713	28104821	G	0.5	0.7143	A
3	BICF2S23342014	28106511	G	0.5	0.7143	A
3	BICF2S23644249	28119067	G	0.5	0.2857	A
3	BICF2S2414730	28130150	G	0.5	0.2857	A
3	BICF2P436531	28156450	0	0	0	A
3	BICF2P159737	28167855	G	0.5	0.2857	A
3	BICF2P161297	28182839	G	0.5	0.2857	A
3	BICF2P1312074	28196051	G	0	0	A
3	BICF2P770296	28200500	A	0	0	G
3	BICF2P1128773	28217541	0	0	0	A

3	BICF2P1160610	28231024	G	0.5	0.2857	A
3	BICF2P1277472	28242675	C	0	0	A
3	BICF2P996731	28245451	0	0	0	A
3	BICF2P800481	28257464	G	0.5	0.2857	A
3	BICF2P418798	28273265	0	0	0	C
3	BICF2P1375464	28289542	A	0	0	C
3	BICF2P742446	28291082	G	0.5	0.2857	A
3	BICF2P345904	28306859	G	0.5	0.125	A
3	BICF2P1053435	28316632	G	0.5	0.2857	A
3	BICF2P942722	28327765	A	1	1	T
3	TIGRP2P39613_rs8521746	28344835	0	0	0	G
3	BICF2P602810	28354775	C	0	0	A
3	BICF2P1068506	28359706	A	0	0	G
3	BICF2S23524749	28376427	G	0	0	A
3	BICF2S23764041	28394120	A	0	0	G
3	BICF2P686400	28412132	A	0.5	0.2857	G
3	BICF2S23643545	28427238	0	0	0	A
3	BICF2P888162	28444222	A	0	0	T
3	BICF2P207058	28456599	G	0	0	A
3	BICF2S23214831	28487970	0	0	0	A
3	BICF2S23749747	28501151	A	0	0.5	G
3	BICF2P214109	28514166	G	0.5	0.7143	A
3	BICF2P602688	28520681	G	0	0	C
3	BICF2P1275554	28538166	G	0	0	A
3	BICF2P823712	28545513	C	0	0	A
3	BICF2P345066	28557092	G	0	0	A
3	TIGRP2P39619_rs8890737	28574281	0	0	0	G
3	BICF2P335768	28583159	A	0	0	C
3	BICF2P864385	28593142	A	0.5	0.2857	T
3	BICF2P613099	28607094	G	0	0	A
3	BICF2P968772	28627590	A	0	0	C
3	BICF2P240284	28643478	A	0	0	T
3	TIGRP2P39622_rs8875287	28672799	0	0	0	C
3	BICF2P782360	28704455	A	0.5	0.2857	G
3	BICF2P574188	28735452	G	0	0	A
3	BICF2P1130462	28741419	A	0	0	G
3	BICF2S23097	28758077	C	0.5	0.7143	A
3	BICF2P1366454	28772873	A	0	0	G
3	BICF2S23243416	28777614	G	0.5	0.2857	A
3	BICF2S23258915	28789949	A	0	0	G
3	BICF2P791792	28792461	C	0	0	G
3	BICF2S23532408	28806139	0	0	0	A
3	BICF2S2323387	28809330	0	0	0	A
3	BICF2P1123096	28822700	C	0	0	A
3	BICF2P71957	28831605	0	0	0	A
3	BICF2S2355183	28849658	G	0.5	0.7143	A
3	BICF2S23430651	28872678	G	0	0	A

3	BICF2P411143	28877013	G	0.5	0.7857	A
3	BICF2P516268	28892832	C	0	0	A
3	BICF2S23258887	28902757	A	0.5	0.2143	G
3	BICF2S2337858	28913759	A	0	0	G
3	BICF2P645291	28922948	G	0	0	A
3	BICF2P110650	28940991	G	0.5	0.7857	A
3	BICF2P224540	28945514	C	0.5	0.7857	A
3	BICF2P77534	28957812	0	0	0	G
3	BICF2P200069	28973331	0	0	0	C
3	BICF2P237401	29002208	T	0.5	0.2857	A
3	BICF2P101135	29004240	C	0	0	A
3	BICF2P727795	29014860	C	0.5	0.2857	A
3	BICF2P740499	29055962	G	0	0.5	A
3	BICF2P449883	29062269	G	1	1	A
3	BICF2S23057910	29081821	A	0.5	0.2857	C
3	BICF2S23610777	29094728	0	0	0	G
3	BICF2S2363826	29098746	0	0	0	T
3	BICF2P760589	29110089	A	0.5	0.2857	G
3	BICF2P448398	29123403	C	0	0	A
3	BICF2P689243	29136015	A	0.5	0.2857	G
3	BICF2S23761662	29149439	G	0	0	A
3	BICF2P1125787	29154291	A	1	1	C
3	BICF2P780704	29195208	0	0	0	G
3	BICF2P449336	29210965	A	0.5	0.7143	G
3	BICF2P1337760	29225806	0	0	0	G
3	BICF2P184224	29236850	0	0	0	A
3	BICF2P806955	29275088	0	0	0	G
3	BICF2S2348799	29280939	A	1	0.5	G
3	BICF2S23444014	29293918	G	1	0.5	A
3	BICF2S23548777	29313767	A	1	0.5	T
3	BICF2P1444593	29335571	0	0	0	C
3	BICF2P191639	29346566	A	1	0.5	C
3	BICF2P1436832	29351955	0	0	0	G
3	BICF2S23731886	29370602	G	1	0.5	A
3	BICF2P460793	29393488	0	0	0	G
3	BICF2P1307080	29420007	A	1	1	G
3	BICF2P817458	29429128	A	1	1	G
3	BICF2P1089829	29440983	A	0.5	0.7857	G
3	BICF2P741495	29459535	A	0	0	G
3	BICF2S23732794	29465502	G	1	1	A
3	BICF2P460360	29480798	A	1	0.5	G
3	BICF2P430169	29499395	G	1	0.5	A
3	BICF2P1143290	29526853	A	1	0.5	G
3	BICF2S23024979	29545695	A	0	0	T
3	BICF2P340942	29619350	0	0	0	A
3	BICF2S2297262	29655385	G	0.5	0.2143	A
3	BICF2S23425783	29660471	G	1	0.5	A

3	BICF2S23763170	29675904	A	0.5	0.2857	G
3	BICF2S2299918	29688077	G	0	0	A
3	BICF2P856855	29714061	A	0.5	0.2857	T
3	BICF2P263446	29723351	C	0.5	0.2857	A
3	BICF2S23048225	29728566	C	0.5	0.7857	G
3	BICF2P790287	29749785	A	0	0.5	G
3	BICF2P81531	29753289	G	0	0.5	A
3	BICF2S23651049	29763908	A	1	0.5	G
3	BICF2P1288047	29780756	A	0	0	G
3	BICF2P309004	29783736	G	0	0	A
3	BICF2P309003	29784015	G	0	0	A
3	BICF2P781073	29790943	A	0.5	0.2143	G
3	BICF2S23648843	29796684	A	0.5	0.7857	G
3	BICF2P1271157	29827992	A	0	0	G
3	BICF2S22925934	29840342	A	0	0	C
3	BICF2S2327436	29847427	C	0	0	A
3	BICF2P1452462	29855599	A	0	0	G
3	BICF2S2346956	29869180	C	0	0	A
3	BICF2P367760	29889879	C	1	0.5714	A
3	BICF2P270615	29900815	A	1	0.5714	G
3	BICF2P1011366	29903841	A	1	0.5714	G
3	BICF2S2413225	29922674	0	0	0	C
3	BICF2S23647639	29945170	0	0	0	A
3	BICF2S23644295	29949728	G	0	0	A
3	BICF2P415349	29965076	G	0	0.4286	A
3	BICF2P357166	29984124	A	0	0.4286	G
3	BICF2S23329646	30002398	C	0.5	0.7143	G
3	BICF2S23329647	30002474	A	0.5	0.2857	G
3	BICF2P724336	30010206	C	0	0	G
3	BICF2P926172	30019018	A	0.5	0.2857	G
3	BICF2P881821	30032848	A	0	0	C
3	BICF2S23252486	30046076	A	0	0	C
3	BICF2S23445287	30059175	A	0.5	0.2857	G
3	BICF2S23658664	30063590	G	1	0.5714	A
3	BICF2P1194662	30082394	G	0	0.4286	A
3	BICF2P453152	30086301	0	0	0	C
3	BICF2P1071875	30098076	G	0	0.4286	A
3	BICF2P1095750	30114576	A	0	0	G
3	BICF2S23554724	30140191	A	1	0.5714	C
3	BICF2S23244025	30145544	A	1	0.5714	G
3	BICF2P94176	30157239	0	0	0	A
3	BICF2G630704173	30164905	G	0	0	A
3	BICF2G630704177	30178909	A	0	0	G
3	BICF2G630704182	30196894	A	0	0	G
3	BICF2P353885	30200899	T	1	1	A
3	BICF2P276702	30215138	A	0	0.4286	G
3	BICF2P560963	30229810	G	0	0	A

3	BICF2P1303290	30241896	A	0.5	0.7143	G
3	BICF2P770273	30252773	G	0	0	A
3	BICF2S23035822	30263752	G	0	0	A
3	BICF2P1360705	30268826	C	0	0	A
3	BICF2P1109038	30287124	A	0	0	G
3	BICF2G630704234	30294194	A	0	0	G
3	BICF2G630704243	30304187	G	0.5	0.7143	A
3	BICF2G630704249	30314493	A	0.5	0.7143	G
3	BICF2G630704261	30326469	G	0	0	A
3	BICF2G630704278	30346668	G	0	0	A
3	BICF2G630704284	30352582	G	1	1	A
3	BICF2P930463	30364362	T	1	1	A
3	BICF2P495845	30382865	C	0	0	G
3	BICF2P197385	30404020	A	0	0	G
3	BICF2S23762981	30416931	G	1	1	A
3	BICF2G630704292	30428922	A	0	0	G
3	BICF2S23240902	30430108	G	0	0	A
3	BICF2G630704294	30451610	A	0	0	G
3	BICF2S23452927	30457794	G	0.5	0.7143	A
3	BICF2P456217	30472754	0	0	0	C
3	BICF2S23225389	30503827	C	0.5	0.2857	A
3	BICF2G630704298	30530775	C	1	0.5714	A
3	BICF2P1166071	30540146	0	0	0	A
3	BICF2G630704313	30550559	A	1	1	G
3	BICF2G630704329	30568341	A	0.5	0.2857	G
3	BICF2P400976	30581602	G	0	0	A
3	BICF2G630704343	30597963	G	0	0	A
3	BICF2G630704365	30606257	A	0.5	0.2857	G
3	BICF2P1176933	30613475	G	0.5	0.2857	A
3	BICF2G630704404	30629414	G	0	0	A
3	BICF2G630704414	30636006	A	0.5	0.2857	G
3	BICF2G630704432	30648725	G	1	1	A
3	BICF2P660895	30653466	G	0	0	A
3	BICF2G630704438	30659663	G	0.5	0.2857	A
3	BICF2G630704445	30674008	G	0.5	0.2857	A
3	BICF2P58672	30684680	A	0.5	0.7143	G
3	BICF2P58673	30684786	A	0	0	G
3	BICF2S23540887	30695678	A	0.5	0.2857	G
3	BICF2S23723565	30716444	A	1	0.5714	G
3	BICF2P1125729	30727645	0	0	0	A
3	BICF2P1259920	30756492	G	0	0.4286	A
3	BICF2P664113	30774041	G	0.5	0.7143	A
3	BICF2G630704471	30782110	A	0.5	0.7143	G
3	BICF2G630704480	30792459	0	0	0	G
3	BICF2G630704490	30799265	G	0	0.4286	A
3	BICF2G630704511	30812031	0	0	0	G
3	BICF2G630704526	30828281	0	0	0	G

3	BICF2G630704534	30832167	A	0.5	0.2857	G
3	BICF2G630704559	30845264	A	0	0	G
3	BICF2S23126266	30854820	G	0	0	A
3	BICF2P716862	30874316	G	0	0	A
3	BICF2G630704573	30878182	0	0	0	G
3	BICF2P1034072	30899994	G	1	1	A
3	BICF2P1034065	30900841	G	1	1	A
3	BICF2P1049492	30919589	A	0.5	0.7143	C
3	BICF2G630704599	30927673	A	1	1	G
3	BICF2P967997	30939044	A	0	0	G
3	BICF2G630704611	30942338	A	1	1	G
3	BICF2G630704618	30949459	A	0	0	C
3	BICF2S22931067	30958746	A	0	0	G
3	BICF2G630704630	30969875	A	1	1	G
3	BICF2G630704655	30986655	C	0	0	A
3	BICF2G630704675	30994201	A	0	0	G
3	BICF2G630704681	30995913	A	0	0	G
3	BICF2G630704697	31010174	C	0	0	A
3	BICF2G630704706	31018587	G	0	0	A
3	BICF2G630704717	31028777	A	0	0	C
3	BICF2G630704731	31046254	G	0.5	0.2857	A
3	BICF2P752461	31058687	A	0	0	G
3	BICF2G630704753	31059268	G	0	0	A
3	BICF2G630704758	31063890	A	0	0	G
3	BICF2G630704771	31076651	G	1	1	A
3	BICF2G630704786	31082395	G	0	0	A
3	BICF2G630704796	31086846	0	0	0	A
3	BICF2G630704818	31103574	G	0	0	A
3	BICF2G630704825	31108824	G	0.5	0.2857	A
3	BICF2G630704841	31119989	A	0.5	0.7143	G
3	BICF2G630704846	31132471	A	1	1	G
3	BICF2P37982	31153482	0	0	0	C
3	BICF2G630704855	31163217	A	1	1	G
3	BICF2G630704858	31165850	A	0	0	G
3	BICF2G630704866	31187342	A	0	0	G
3	BICF2G630704870	31199538	A	0	0	T
3	BICF2G630704876	31221809	A	0	0	G
3	BICF2G630704880	31235088	A	1	1	G
3	BICF2G630704885	31248499	A	1	1	G
3	BICF2S23642809	31278767	0	0	0	A
3	BICF2G630704902	31286025	G	0	0	A
3	BICF2G630704907	31303177	A	1	1	G
3	BICF2G630704922	31315152	G	NA	0	A
3	BICF2S22964837	31328839	A	0.5	0.7143	G
3	BICF2G630704954	31341807	G	1	1	A
3	BICF2G630704959	31351957	C	0.5	0.2857	A
3	BICF2G630704969	31365788	G	1	1	A

3	BICF2G630704980	31383210	A	1	1	C
3	BICF2G630704981	31390024	A	0	0	G
3	BICF2G630704986	31404003	A	1	1	G
3	BICF2G630704988	31412314	A	0	0	G
3	BICF2G630704999	31430685	A	0	0	C
3	BICF2G630705003	31449574	A	0	0	G
3	BICF2P445265	31462933	0	0	0	A
3	BICF2G630705006	31465329	G	1	1	A
3	BICF2G630705012	31475837	A	1	1	G
3	BICF2G630705021	31490103	T	0	0	A
3	BICF2P130112	31508547	A	1	1	G
3	BICF2G630705026	31514418	G	0	0	A
3	BICF2G630705031	31527000	A	1	1	G
3	BICF2G630705033	31536128	G	1	1	A
3	BICF2P994292	31570799	A	1	1	G
3	BICF2G630705040	31587223	C	1	1	A
3	BICF2P1236507	31613155	A	0	0	G
3	BICF2G630705049	31621990	A	1	1	T
3	BICF2G630705050	31636088	G	1	1	A
3	BICF2G630705053	31643473	C	0	0	A
3	BICF2G630705055	31648935	G	0.5	0.7143	A
3	BICF2G630705065	31663879	A	0	0	G
3	BICF2G630705075	31677319	G	0.5	0.2857	A
3	BICF2G630705081	31684376	G	0	0	A
3	BICF2P1178293	31698752	C	0	0	A
3	BICF2S2398896	31735251	A	0	0	G
3	BICF2G630705098	31747696	A	0	0	G
3	BICF2P1078994	31759214	0	0	0	A
3	BICF2G630705111	31766592	G	0	0	C
3	BICF2G630705112	31767817	A	0	0	C
3	BICF2G630705117	31776343	G	0	0	A
3	BICF2G630705122	31786693	G	0	0	A
3	BICF2G630705143	31797925	G	0	0	A
3	BICF2G630705163	31808118	A	0	0	G
3	BICF2G630705183	31815866	G	0	0	A
3	BICF2G630705201	31838535	A	0.5	0.7143	G
3	BICF2S229611	31848617	A	0.5	0.7143	G
3	BICF2G630705205	31859418	A	0	0	G
3	BICF2S2296215	31871611	T	0.5	0.7143	A
3	TIGRP2P39723_rs9214006	31885867	0	0	0	G
3	BICF2P1357679	31907677	0	0	0	G
3	BICF2P776848	31917926	0	0	0	A
3	BICF2S23016713	31924305	0	0	0	C
3	BICF2S23250152	31949049	G	0	0	A
3	BICF2G630705208	31965120	G	0.5	0.7143	A
3	BICF2G630705216	31974944	G	0.5	0.7143	A
3	BICF2G630705224	32006992	0	0	0	A

3	BICF2S2375078	32049502	G	0	0	A
3	BICF2G630705240	32052421	A	0.5	0.7143	G
3	BICF2G630705251	32075009	A	0	0	G
3	BICF2G630705257	32100451	C	0.5	0.7143	A
3	BICF2G630705263	32118134	G	0.5	0.7143	A
3	BICF2G630705266	32130229	C	0.5	0.75	A
3	BICF2G630705273	32140551	A	NA	0.75	G
3	BICF2P902519	32143064	A	0.5	0.7143	G
3	BICF2G630705279	32155318	G	0.5	0.7143	A
3	BICF2G630705282	32173118	G	1	1	A
3	BICF2G630705287	32181622	G	0.5	0.7143	A
3	BICF2G630705306	32188869	G	1	1	A
3	BICF2P487670	32206022	0	0	0	T
3	BICF2P659009	32212094	A	1	1	G
3	BICF2G630705323	32259408	A	1	1	G
3	BICF2S22957004	32280342	G	0	0	A
3	BICF2G630705335	32288273	C	1	1	G
3	BICF2G630705339	32292208	G	1	1	C
3	BICF2G630705340	32292529	G	0	0	A
3	BICF2G630705371	32310426	G	0	0	A
3	BICF2P861454	32324218	A	0	0	G
3	BICF2G630705409	32342708	A	1	1	G
3	BICF2G630705420	32353173	G	1	1	A
3	BICF2G630705432	32362648	G	0.5	0.7143	A
3	BICF2G630705449	32378037	A	0.5	0.7143	G
3	BICF2P23073	32384818	A	0.5	0.7143	C
3	BICF2P377786	32396152	A	0	0	G
3	BICF2P1198425	32422534	0	0	0	G
3	BICF2P248854	32423725	A	0	0	G
3	BICF2G630705502	32433719	C	1	1	A
3	BICF2P1281057	32447848	G	1	1	A
3	BICF2P239967	32452666	G	1	1	A
3	BICF2P285460	32455613	A	1	1	C
3	BICF2P903929	32471455	0	0	0	G
3	BICF2P967684	32480197	G	0	0	A
3	BICF2G630705512	32496591	A	0	0	G
3	BICF2G630705517	32501214	G	0	0	A
3	BICF2S23752143	32512991	C	0	0	A
3	BICF2G630705527	32524889	G	0	0	A
3	BICF2G630705534	32534135	G	0	0	A
3	BICF2G630705546	32545547	T	0	0	A
3	BICF2G630705568	32561687	A	0	0	G
3	BICF2G630705581	32570844	C	0	0	A
3	BICF2G630705602	32584223	C	0	0	G
3	BICF2G630705641	32610115	G	1	1	A
3	BICF2G630705644	32614257	A	0	0	G
3	BICF2G630705661	32630689	A	0	0	G

3	BICF2S23414795	32640922	A	0	0	G
3	BICF2S2349746	32650063	A	0	0	G
3	BICF2S23429852	32662716	G	0	0	A
3	BICF2P1265387	32677764	G	0	0	A
3	BICF2P442410	32684962	G	0	0	A
3	BICF2P629029	32695675	A	0	0	G
3	BICF2P1107935	32710231	G	0	0	A
3	BICF2P1421518	32728138	A	0	0	G
3	BICF2G630705690	32736491	A	0.5	0.7143	G
3	BICF2P764513	32756555	G	0	0	C
3	BICF2P193367	32768775	A	0.5	0.7143	C
3	BICF2G630705724	32786259	0	0	0	A
3	BICF2S23231085	32795114	G	0.5	0.7143	A
3	BICF2G630705733	32825332	A	0	0	C
3	BICF2G630705734	32845906	0	0	0	A
3	BICF2S23713941	32856098	T	0	0	A
3	BICF2S23339717	32888077	C	0	0	A
3	BICF2S23659169	32898928	A	0	0	C
3	BICF2G630705740	32909640	0	0	0	C
3	BICF2G630705745	32913190	A	1	1	C
3	BICF2P613112	32927987	G	0	0	A
3	BICF2S23742266	32937335	G	0	0	A
3	BICF2S2367305	32960201	0	0	0	G
3	BICF2G630705758	32974334	A	0.5	0.2857	T
3	BICF2G630705760	32988952	A	0.5	0.7143	G
3	TIGRP2P39739_rs8684241	32996855	0	0	0	A
3	BICF2S23545014	33008129	G	0	0	A
3	BICF2G630705768	33019606	A	0.5	0.7143	G
3	BICF2G630705781	33029540	G	0	0	A
3	BICF2G630705795	33047875	A	0.5	0.7143	G
3	BICF2G630705808	33058973	A	0	0	G
3	BICF2G630705819	33061677	A	0	0	G
3	BICF2P1092111	33063475	A	0	0	G
3	BICF2G630705826	33078978	A	0.5	0.2857	G
3	BICF2G630705838	33092025	G	0.5	0.2857	A
3	BICF2P1278218	33103918	0	0	0	G
3	BICF2S23643528	33117632	A	0	0	C
3	BICF2G630705846	33129258	A	0.5	0.2857	C
3	BICF2G630705853	33135447	G	0.5	0.7143	A
3	BICF2G630705869	33149098	C	0.5	0.7143	G
3	BICF2G630705902	33157327	A	0.5	0.2857	G
3	BICF2G630705915	33167800	A	NA	NA	G
3	BICF2G630705928	33180856	G	0.5	0.2857	A
3	BICF2G630705941	33186081	A	0.5	0.2857	G
3	BICF2G630705955	33191286	G	0.5	0.2857	A
3	BICF2G630705959	33202953	A	0.5	0.2857	G
3	BICF2G630705972	33212987	G	0.5	0.7143	A

3	BICF2G630705993	33230289	A	0.5	0.2857	G
3	BICF2P991639	33242179	A	1	1	G
3	BICF2G630706010	33257555	A	0.5	0.2857	G
3	BICF2G630706014	33259058	G	0.5	0.2857	A
3	BICF2G630706024	33269536	A	0.5	0.2857	G
3	BICF2S22916507	33289605	A	0	0	G
3	BICF2G630706034	33294225	0	0	0	A
3	BICF2S23727801	33307583	A	1	1	C
3	BICF2G630706036	33319356	0	0	0	G
3	BICF2P1122760	33332930	0	0	0	A
3	BICF2P802963	33355812	0	0	0	A
3	BICF2P497923	33401415	0	0	0	G
3	BICF2G630706040	33409702	A	0	0	C
3	BICF2G630706066	33426570	A	0.5	0.2857	T
3	BICF2G630706092	33431598	G	0.5	0.2857	A
3	BICF2G630706144	33448055	G	0	0	A
3	BICF2G630706155	33453932	A	0.5	0.25	C
3	BICF2G630706178	33472405	A	0	0	G
3	BICF2G630706180	33481012	G	0.5	0.2857	A
3	BICF2G630706186	33488468	C	1	1	A
3	BICF2G630706195	33501404	A	1	1	G
3	BICF2G630706227	33518965	G	1	1	A
3	BICF2G630706242	33524946	C	1	1	A
3	BICF2P95479	33538034	A	0	0	G
3	BICF2P697415	33547058	G	0	0	A
3	BICF2P1299715	33558213	C	0	0	A
3	BICF2P612073	33574710	C	0	0	A
3	BICF2G630706333	33591366	C	1	1	A
3	BICF2G630706335	33592851	A	0.5	0.7143	C
3	BICF2G630706365	33605155	A	1	1	G
3	BICF2G630706394	33618785	A	1	1	G
3	BICF2G630706414	33629238	A	1	1	G
3	BICF2G630706437	33640691	G	1	1	A
3	BICF2G630706466	33657014	G	1	1	A
3	BICF2G630706475	33661746	A	0	0	G
3	BICF2S23437266	33676674	A	0.5	0.2857	G
3	BICF2G630706494	33689223	G	0.5	0.7143	A
3	BICF2G630706502	33701236	G	1	1	A
3	BICF2P73373	33710931	A	0	0	G
3	BICF2G630706525	33725374	G	1	1	A
3	BICF2G630706539	33731118	A	1	1	G
3	BICF2G630706558	33745101	A	0.5	0.7143	C
3	BICF2P1027575	33753076	0	0	0	C
3	BICF2G630706563	33767315	A	0.5	0.7143	G
3	BICF2G630706571	33793939	A	0.5	0.7143	G
3	BICF2G630706574	33805116	A	0	0	G
3	BICF2G630706577	33818106	A	0.5	0.7143	G

3	BICF2G630706588	33836297	G	0.5	0.7143	A
3	BICF2G630706597	33855550	G	0.5	0.75	A
3	BICF2G630706598	33856364	G	0.5	0.7143	A
3	BICF2G630706607	33870369	A	0.5	0.7143	G
3	BICF2S23155837	33881474	G	0.5	0.7143	A
3	BICF2G630706642	33908473	G	0.5	0.7143	A
3	BICF2G630706643	33914503	A	0.5	0.7143	G
3	BICF2P765488	33936454	0	0	0	G
3	BICF2P1499671	33938353	A	1	1	G
3	BICF2G630706670	33953622	G	1	1	A
3	BICF2P466165	33978007	0	0	0	G
3	BICF2S23440746	33984799	A	0	0	G
3	BICF2G630706686	34102925	C	0	0	A
3	BICF2G630706693	34117330	A	0.5	0.2857	C
3	BICF2G630706701	34122261	A	0.5	0.7143	C
3	BICF2G630706724	34154652	G	0.5	0.7	A
3	BICF2P94158	34204620	G	0.5	0.7143	A
3	BICF2G630707076	34281738	A	0.5	0.75	G
3	BICF2P751515	34304944	0	0	0	A
3	BICF2G630707194	34358044	A	NA	0.75	G
3	BICF2G630707245	34396539	G	0.5	0.7143	A
3	BICF2G630707288	34409175	A	0.5	0.7143	G
3	BICF2G630707421	34471250	A	0.5	0.2857	G
3	BICF2G630707455	34483695	G	0.5	0.2	A
3	BICF2G630707484	34488618	G	NA	0.25	A
3	TIGRP2P41019_rs8572873	34508399	0	0	0	G
3	G236f20S298	34524018	0	0	0	A
3	BICF2G630337470	34560969	G	0.5	0.7143	A
3	BICF2G630337477	34568242	0	0	0	G
3	BICF2G630337478	34570718	G	0.5	0.7143	A
3	BICF2G630337484	34580593	0	0	0	G
3	BICF2G630337494	34600733	G	0.5	0.7143	A
3	BICF2G630337498	34604177	G	0.5	0.75	A
3	BICF2G630337517	34620335	0	0	0	G
3	BICF2P756511	34630563	G	0.5	0.7143	C
3	BICF2G630337535	34643976	A	0.5	0.7143	G
3	BICF2G630337540	34650557	A	0.5	0.75	G
3	BICF2G630337555	34668969	G	0.5	0.7143	A
3	BICF2G630337562	34672648	A	0.5	0.7143	G
3	BICF2G630337567	34690200	0	0	0	C
3	BICF2G630337573	34707206	0	0	0	G
3	BICF2G630337587	34725254	G	0.5	0.7143	C
3	BICF2G630337590	34731190	0	0	0	A
3	BICF2P331952	34756985	0	0	0	A
3	BICF2G630337624	34786449	G	0.5	0.7143	A
3	BICF2S22954524	34787576	A	0.5	0.7143	G
3	BICF2S22958963	34806577	G	0.5	0.7143	A

3	BICF2G630337644	34817750	0	0	0	A
3	BICF2G630337648	34848173	G	0.5	0.2857	A
3	G237f18S270	34871640	G	0	0	A
3	BICF2G630337653	34900126	A	0	0	C
3	BICF2P876861	34935041	0	0	0	A
3	BICF2P386701	34953626	0	0	0	T
3	BICF2G630337661	34976633	0	0	0	A
3	BICF2G630337662	34988865	A	0	0	C
3	BICF2G630337665	35027351	A	0	0	G
3	BICF2G630337669	35058381	A	0.5	0.2857	C
3	G238f9S294	35093114	0	0	0	C
3	BICF2P1287964	35101211	0	0	0	A
3	BICF2G630337705	35127520	C	0.5	0.2857	A
3	BICF2G630337711	35136498	G	0.5	0.2857	A
3	BICF2G630337726	35146536	G	0.5	0.2857	A
3	BICF2G630337736	35155934	0	0	0	A
3	BICF2G630337747	35172960	A	0.5	0.2857	G
3	G239f21S306	35210414	0	0	0	G
3	TIGRP2P41030_rs8566520	35232279	0	0	0	C
3	BICF2P1305090	35236625	A	0	0	G
3	TIGRP2P41031_rs8831671	35251192	0	0	0	C
3	BICF2S23036294	35264780	G	0.5	0.2857	A
3	BICF2G630106037	35288824	G	0.5	0.2857	A
3	BICF2G630106039	35304384	0	0	0	A
3	G240f23S204	35331753	0	0	0	A
3	BICF2S22931832	35365665	C	0.5	0.2857	A
3	BICF2S22929076	35381994	G	0.5	0.2857	A
3	BICF2G630106043	35396083	A	0	0	G
3	BICF2P1271063	35412405	0	0	0	G
3	BICF2P352268	35427831	0	0	0	A
3	BICF2P1047890	35463391	0	0	0	G
3	BICF2P191589	35488035	0	0	0	A
3	BICF2S23212956	35490383	A	0.5	0.2857	G
3	G241f17S21	35519445	A	0.5	0.2857	G
3	G241f18S277	35525943	A	0	0	C
3	BICF2G630106061	35554593	C	0.5	0.2857	A
3	BICF2G630106077	35561727	A	0.5	0.2857	G
3	BICF2G630106088	35565691	A	0.5	0.2857	T
3	BICF2S23423362	35590913	A	0.5	0.2857	G
3	BICF2P988675	35594223	A	0	0.4286	G
3	BICF2P134520	35605537	G	0	0	A
3	BICF2P662888	35617909	G	0	0	A
3	BICF2G630106151	35633609	A	0.5	0.2857	G
3	BICF2G630106181	35645653	G	0.5	0.2857	C
3	BICF2G630106202	35656108	C	0	0	G
3	BICF2G630106206	35663990	G	0	0	A
3	BICF2S23131953	35701884	G	0.5	0.2	A

3	BICF2G630106235	35727806	G	0	0	A
3	BICF2G630106237	35739739	A	0	0	G
3	BICF2G630106241	35742496	G	0	0	A
3	BICF2G630106257	35762139	C	0.5	0.2857	A
3	BICF2G630106274	35774486	A	0.5	0.2857	C
3	BICF2G630106292	35785642	T	0	0.4286	A
3	BICF2G630106304	35790057	G	0.5	0.75	A
3	BICF2G630106341	35799839	C	0	0.4286	A
3	BICF2G630106356	35821899	C	0.5	0.7143	A
3	BICF2G630106360	35823798	A	0.5	0.7143	G
3	BICF2G630106385	35837137	A	0.5	0.7143	C
3	BICF2G630106390	35866782	G	1	1	A
3	BICF2P222705	35930933	A	0	0	G
3	G242f20S94	35955395	A	0	0	G
3	BICF2G630106397	35995620	A	0	0	T
3	BICF2P1496235	36040540	0	NA	0	A
3	BICF2P617130	36055823	0	0	0	G
3	BICF2G630106399	36071787	A	0	0	G
3	BICF2S23327759	36076169	A	0.5	0.2857	G
3	BICF2G630106402	36123640	A	0.5	0.2857	G
3	BICF2G630106463	36150674	A	0	0.4286	G
3	BICF2G630106472	36158339	G	0.5	0.2857	A
3	BICF2P523797	36175246	A	0	0	G
3	BICF2P1363915	36207050	A	0.5	0.2857	C
3	BICF2S23043164	36224089	G	0	0	A
3	TIGRP2P41173_rs9234444	36236428	G	0	0	A
3	BICF2P46127	36236612	G	0.5	0.2857	C
3	TIGRP2P41180_rs9120523	36248011	G	0.5	0.2857	A
3	TIGRP2P41189_rs8742795	36262561	G	0	0	A
3	BICF2G630106482	36282419	A	0	0	G
3	TIGRP2P41195_rs8796158	36282971	A	0	0	G
3	BICF2P778347	36298018	C	0	0	A
3	BICF2P621551	36306450	C	0	0	A
3	BICF2G630106489	36328333	G	0	0	A
3	BICF2P889439	36330130	A	0	0	C
3	TIGRP2P41218_rs9231423	36355057	G	0.5	0.7143	A
3	BICF2G630106551	36395966	A	0.5	0.7143	T
3	BICF2G630106552	36398534	G	0.5	0.2857	A
3	BICF2G630106563	36410575	C	0.5	0.2857	G
3	BICF2G630106591	36432197	G	0.5	0.2857	A
3	BICF2G630106597	36448642	G	0.5	0.7143	A
3	BICF2P1103001	36457203	0	0	0	A
3	BICF2G630106615	36476262	A	0.5	0.75	C
3	BICF2G630106625	36482789	A	0.5	0.2857	G
3	TIGRP2P41233_rs9009061	36490051	A	0.5	0.2857	G
3	BICF2P531199	36509190	A	0	0	G
3	BICF2P321547	36518028	G	0	0	A

3	BICF2P1255508	36525745	A	0	0	G
3	BICF2G630106677	36530531	G	0.5	0.2857	A
3	BICF2G630106693	36542297	A	0.5	0.25	C
3	BICF2P424215	36548895	G	0.5	0.2857	A
3	BICF2P153503	36565993	C	0.5	0.2857	A
3	TIGRP2P41275_rs8985855	36575897	0	0	0	A
3	BICF2P362029	36584899	G	0	0.4286	A
3	BICF2G630106718	36633140	C	0	0	A
3	BICF2G630106741	36649317	C	0	0	A
3	BICF2P523410	36656068	A	0.5	0.2857	G
3	BICF2G630106760	36671485	G	0.5	0.7143	A
3	BICF2P572663	36684651	A	0	0.4167	G
3	TIGRP2P41285_rs8544465	36685002	G	0	0.4286	A
3	BICF2P448616	36709946	A	0	0.4286	T
3	BICF2G630106782	36721494	A	0	0	G
3	BICF2G630106786	36738612	A	0.5	0.2857	G
3	BICF2G630106787	36748479	A	0.5	0.2857	C
3	BICF2G630106790	36754360	A	0	0	G
3	BICF2G630106808	36788461	G	0.5	0.2857	A
3	TIGRP2P41307_rs8807822	36796650	0	0	0	A
3	BICF2G630106811	36803951	A	0.5	0.2857	G
3	BICF2G630106815	36819286	A	0	0	G
3	BICF2G630106817	36824525	A	0	0	G
3	BICF2G630106838	36848434	A	1	0.5714	G
3	BICF2G630106857	36875341	A	0	0	C
3	BICF2P100992	36899859	G	0	0	A
3	BICF2G630106876	36906261	G	0	0	A
3	BICF2G630106879	36924413	A	0	0	G
3	BICF2G630106895	36936375	A	0.5	0.75	C
3	BICF2G630106901	36943998	A	0.5	0.7143	G
3	BICF2G630106912	36956577	G	0	0	A
3	BICF2G630106926	36967027	A	0	0	G
3	BICF2P1266151	36992234	G	0.5	0.7143	A
3	BICF2G630106957	37000220	A	0.5	0.2857	G
3	BICF2G630106970	37007850	A	0.5	0.2857	G
3	BICF2S23719407	37018649	G	0.5	0.2857	A
3	BICF2P798483	37050939	A	0	0	G
3	BICF2P678049	37074377	0	0	0	C
3	BICF2P1211828	37089347	G	0.5	0.7143	A
3	BICF2G630106991	37100163	A	0	0.4286	G
3	BICF2S22912703	37127801	G	0	0.4286	A
3	BICF2P1398021	37138163	A	0	0.4286	G
3	BICF2G630107005	37157251	A	0	0	G
3	BICF2G630107019	37169571	A	0	0	G
3	BICF2P610095	37185647	G	0	0	A
3	BICF2G630107056	37192535	A	0	0	G
3	BICF2G630107076	37211443	A	0	0	G

3	BICF2G630107090	37215049	A	0	0	G
3	BICF2G630107107	37228125	A	0	0	G
3	BICF2S23117827	37243570	A	0	0	G
3	BICF2G630107136	37252772	A	0	0.4286	G
3	BICF2G630107149	37262769	A	0	0.4286	G
3	BICF2G630107155	37274474	G	0	0	A
3	BICF2G630107179	37289259	A	0	0	G
3	BICF2G630107204	37297566	A	1	0.5714	G
3	BICF2G630107229	37324228	A	1	0.5714	C
3	BICF2P401184	37342704	T	1	0.5714	A
3	BICF2G630107237	37342973	A	0	0	G
3	TIGRP2P41408_rs9028596	37362124	G	0	0.4286	A
3	BICF2G630107255	37380260	G	0	0	A
3	BICF2G630107271	37397250	G	0.5	0.2857	A
3	BICF2G630107292	37409209	A	1	1	T
3	BICF2G630107308	37420245	A	0	0	G
3	BICF2G630107310	37422499	A	0	0	G
3	BICF2G630107335	37433085	A	0.5	0.7143	G
3	BICF2G630107381	37446376	G	0.5	0.2857	A
3	BICF2G630107404	37459889	C	0.5	0.2857	A
3	BICF2G630107451	37478740	G	0.5	0.2857	A
3	BICF2G630107463	37500543	G	0.5	0.2857	A
3	BICF2G630107465	37504146	A	0.5	0.2857	G
3	BICF2G630107478	37535201	G	0.5	0.2857	A
3	BICF2G630107483	37539732	G	0.5	0.25	A
3	BICF2G630107488	37553938	A	0.5	0.2857	G
3	BICF2G630107505	37559875	A	0.5	0.25	G
3	BICF2G630107546	37578720	G	0.5	0.2857	A
3	BICF2G630107565	37591557	T	0	0	A
3	BICF2G630107566	37591793	G	0	0	A
3	BICF2G630107567	37591987	A	0	0	G
3	TIGRP2P41458_rs9205281	37596153	A	0	0	G
3	BICF2G630107585	37607964	G	0	0	A
3	TIGRP2P41466_rs8931554	37631618	A	0	0	G
3	BICF2G630107620	37640940	G	0.5	0.2857	A
3	BICF2G630107632	37655486	0	0	0	A
3	BICF2P1393287	37675170	G	0.5	0.2857	A
3	BICF2P1283031	37685920	C	0.5	0.2857	G
3	BICF2G630107645	37691155	G	0.5	0.7143	A
3	BICF2P1401845	37701483	A	0.5	0.7143	G
3	TIGRP2P41485_rs9211195	37712424	G	1	1	A
3	BICF2P970499	37729860	G	1	0.5714	A
3	BICF2P783048	37733511	A	1	0.5714	C
3	BICF2G630107672	37743285	A	0.5	0.2857	G
3	BICF2G630107676	37757211	G	0.5	0.2857	A
3	TIGRP2P41503_rs8599393	37766813	G	0	0	A
3	BICF2G630107696	37790414	G	0	0	A

3	BICF2G630107714	37810019	0	0	0	A
3	BICF2G630107717	37814785	G	0.5	0.7143	A
3	BICF2G630107726	37830447	0	0	0	G
3	BICF2G630107747	37853748	G	0.5	0.7143	A
3	BICF2G630107767	37867683	G	0.5	0.7143	A
3	BICF2G630107782	37874247	0	0	0	G
3	BICF2S23240792	37904421	A	0	0	G
3	BICF2G630107794	37915954	A	1	0.5833	G
3	BICF2G630107839	37939953	G	0	0	A
3	BICF2G630107853	37951617	C	1	1	A
3	BICF2S23415334	37957183	A	0	0	C
3	BICF2S23415333	37957261	A	0	0	T
3	BICF2G630107873	37969837	0	0	0	A
3	BICF2G630107882	37975988	0	0	0	G
3	BICF2G630107902	37986469	A	0	0	G
3	BICF2G630107925	38005218	A	0	0.4286	G
3	BICF2G630107939	38010157	G	0	0.4286	A
3	BICF2P627977	38048658	C	0	0	G
3	BICF2G630107948	38056896	G	1	0.5714	A
3	BICF2G630107949	38065848	C	0	0.4286	A
3	BICF2G630107973	38095570	0	0	0	C
3	BICF2P299730	38107353	A	1	0.5714	C
3	TIGRP2P41556_rs9065281	38114688	C	0.5	0.2857	G
3	G243f10S262	38126953	G	0.5	0.2857	A
3	G244f4S92	38182726	C	0	0	A
3	BICF2S23250460	38201963	C	0	0	A
3	BICF2S23410538	38206087	A	0.5	0.25	G
3	BICF2G630107989	38225516	0	0	0	A
3	BICF2P1393860	38229297	A	0.5	0.2857	G
3	TIGRP2P41573_rs8939936	38247132	G	0.5	0.2857	A
3	BICF2P82045	38262610	0	0	0	G
3	BICF2P602877	38272321	A	0	0.4286	G
3	BICF2G630107990	38293172	0	0	0	G
3	BICF2P297290	38304250	A	0.5	0.25	G
3	BICF2P1141135	38311389	0	NA	0	G
3	BICF2P577483	38335649	A	0.5	0.2857	G
3	BICF2P841998	38369439	A	0.5	0.2857	G
3	BICF2P290367	38394225	G	0	0.4286	A
3	BICF2P72203	38417816	G	0	0.4286	A
3	TIGRP2P41596_rs8991090	38430822	A	0	0	G
3	BICF2P956418	38440620	G	0	0.4286	A
3	BICF2P742793	38450206	A	0	0.4286	C
3	BICF2G630107993	38475275	G	0	0	A
3	BICF2P451434	38504219	A	0	0	G
3	BICF2P590342	38514990	G	0	0.4286	A
3	BICF2P1136357	38548634	A	0	0	G
3	BICF2G630107998	38566665	0	0	0	G

3	BICF2P1195596	38571176	A	0	0	C
3	BICF2P523258	38594972	A	0	0.4167	G
3	BICF2P1275525	38607091	A	0	0.4286	C
3	BICF2P422395	38622849	A	0	0.4167	C
3	BICF2P838358	38638661	C	0	0.4167	A
3	BICF2P124476	38644961	C	0	0	G
3	BICF2P513550	38659699	G	0	0.4286	A
3	TIGRP2P41786_rs8734585	38664186	C	0	0	A
3	BICF2P1065968	38676703	A	0	0	C
3	BICF2P846328	38694017	C	0	0.4286	A
3	BICF2G630108159	38699826	G	0	0.4286	A
3	BICF2S23114976	38712512	C	0	0	A
3	TIGRP2P41826_rs9173489	38727830	C	1	0.5714	A
3	BICF2S23130331	38734987	A	1	0.5714	G
3	TIGRP2P41844_rs9040786	38747508	G	1	0.5714	A
3	BICF2S23215515	38758694	A	1	0.5714	G
3	BICF2S23023683	38779091	G	1	0.5714	A
3	BICF2P1236304	38796678	A	1	0.5714	C
3	TIGRP2P41862_rs8911839	38804687	G	1	0.5714	A
3	G245f4S235	38826693	0	0	0	A
3	G246f1S175	38883774	0	0	0	C
3	BICF2P198292	38945183	A	0	0	G
3	BICF2P372011	38960583	0	0	0	A
3	BICF2G630108169	38971137	A	0	0	G
3	BICF2P188686	38981318	A	0.5	0.2857	C
3	BICF2G630108170	38988675	A	0	0	G
3	BICF2G630108171	39001207	C	0	0	A
3	TIGRP2P41929_rs9031905	39022566	A	0	0	G
3	BICF2P1501362	39044801	0	0	0	A
3	BICF2G630108179	39080205	T	0	0	A
3	BICF2S23034309	39093205	A	0.75	0.5714	G
3	TIGRP2P41932_rs8971398	39108430	0	0	0	C
3	BICF2G630108182	39112556	G	0	0	A
3	TIGRP2P41941_rs9189419	39185489	C	0	0	G
3	BICF2P579846	39202443	G	0	0	A
3	BICF2S2306486	39224190	A	1	1	G
3	BICF2P767079	39237810	G	1	1	A
3	BICF2G630108204	39238509	A	1	1	G
3	BICF2G630108228	39270137	A	0	0	G
3	TIGRP2P41949_rs9161169	39278037	A	1	1	G
3	BICF2G630108239	39286424	G	0.25	0.4286	A
3	BICF2P409381	39306656	A	0	0	G
3	TIGRP2P41970_rs8639930	39330182	G	0	0	A
3	BICF2G630108333	39352127	A	0	0	G
3	BICF2P490864	39368647	G	0.25	0.4286	A
3	BICF2S23622684	39395861	G	0	0	A
3	BICF2G630108404	39424250	G	0	0	A

3	BICF2G630108432	39443093	A	0	0	G
3	BICF2P865064	39456397	0	0	0	G
3	BICF2G630108456	39457918	A	0.25	0.4286	G
3	BICF2S23148483	39469011	G	0	0	A
3	BICF2G630108494	39485393	G	0.25	0.4286	A
3	BICF2G630108499	39498529	A	0.25	0.4286	G
3	BICF2S23042301	39510990	A	0	0	G
3	BICF2P866702	39517119	A	0	0	G
3	BICF2P1403833	39537009	A	0.25	0.4286	G
3	BICF2G630108543	39551089	A	0	0	G
3	BICF2G630108567	39572061	A	0	0	G
3	BICF2G630108595	39606538	C	0.25	0.4286	A
3	TIGRP2P42039_rs9057010	39626589	A	0	0.4286	G
3	TIGRP2P42043_rs9217994	39636103	G	0.25	0.4286	A
3	BICF2G630108626	39660974	0	0	0	T
3	BICF2P1014669	39673434	0	0	0	A
3	BICF2S23125394	39713807	A	0.75	0.7143	C
3	BICF2G630108643	39726018	0	0	0	A
3	BICF2P1208903	39745108	C	0.25	0.4286	A
3	BICF2S22935083	39773224	A	0.5	0.2857	G
3	BICF2S23040183	39783385	A	0	0	G
3	BICF2G630108652	39794979	0	0	0	C
3	BICF2P1435332	39823849	0	0	0	A
3	BICF2G630108655	39832658	C	0.5	0.2857	A
3	TIGRP2P42055_rs8921712	39836160	C	0.25	0.2857	A
3	BICF2S23452236	39880576	A	0.5	0.2857	G
3	BICF2G630108662	39899480	C	0.25	0.4286	A
3	BICF2P1002549	39912032	A	0.25	0.2857	G
3	BICF2P986838	39925309	A	0.5	0.5	G
3	BICF2P205703	39956081	A	0.25	0.4286	G
3	BICF2P246215	39984687	A	0.25	0.4286	G
3	BICF2P516822	39988184	A	0	0	G
3	BICF2G630108671	40029208	A	0.75	0.5714	G
3	BICF2P390808	40039186	A	0	0	G
3	BICF2G630108676	40042604	A	0.25	0.4286	G
3	TIGRP2P42106_rs9061369	40045737	G	0.75	0.5714	A
3	BICF2G630108688	40057269	A	0.25	0.4286	G
3	TIGRP2P42111_rs9169155	40067345	G	0.25	0.4286	C
3	BICF2P186653	40073821	G	0.75	0.5714	A
3	TIGRP2P42115_rs8725922	40082298	A	0.75	0.5714	G
3	BICF2G630337926	40093065	C	0.25	0.4286	A
3	BICF2G630337942	40108436	A	0.25	0.4286	G
3	BICF2G630337950	40116064	A	0	0.4167	G
3	BICF2P933266	40141730	0	0	0	A
3	BICF2G630337984	40162031	A	0	0.4286	G
3	BICF2G630337991	40184776	A	0	0	C
3	BICF2G630338000	40197981	A	0.75	0.5714	G

3	BICF2G630338007	40206565	G	0.25	0.4286	A
3	BICF2G630338024	40215817	A	0.25	0.4286	G
3	BICF2G630338066	40231650	A	0.25	0.4286	G
3	BICF2G630338087	40246132	G	0.25	0.4286	A
3	BICF2G630338113	40254259	A	0.25	0.4286	G
3	BICF2P156516	40270774	A	0	0	G
3	BICF2G630338153	40283467	A	0.25	0.4286	G
3	BICF2G630338168	40292838	C	0.25	0.4286	A
3	TIGRP2P42156_rs8720215	40292943	A	0	0	G
3	BICF2G630338174	40300142	G	0	0	A
3	BICF2G630338208	40317985	C	0	0	G
3	BICF2G630338211	40321019	A	0	0	G
3	BICF2G630338219	40332959	A	0.5	0.7143	C
3	BICF2G630338255	40351817	A	0.25	0.2857	G
3	BICF2G630338258	40354792	G	0.5	0.7143	A
3	BICF2G630338265	40364793	G	0.5	0.7143	A
3	TIGRP2P42176_rs9091206	40370979	A	0.5	0.7143	G
3	BICF2G630338273	40374442	G	0.5	0.7143	C
3	TIGRP2P42177_rs8411128	40380180	G	0.25	0.2857	A
3	BICF2P960472	40388090	G	0.5	0.7143	A
3	BICF2G630338286	40401187	0	0	0	C
3	TIGRP2P42186_rs8880518	40421396	0	0	0	C
3	G248f45S296	40431499	0	0	0	G
3	G248f50S177	40440818	T	0	0	A
3	BICF2S23336288	40466410	G	0	0	A
3	BICF2P1223825	40471308	A	0.25	0.4286	G
3	TIGRP2P42195_rs9194739	40491743	A	0.25	0.4286	G
3	BICF2P434416	40505005	0	0	0	G
3	BICF2G630338333	40519829	A	0.25	0.4286	G
3	BICF2S23728055	40533804	A	0.25	0.2857	G
3	BICF2G630338353	40535225	A	0.25	0.4286	C
3	BICF2G630338354	40540844	A	0	0	G
3	BICF2P146473	40550214	0	0	0	G
3	BICF2P1256816	40560941	A	0.25	0.4286	G
3	BICF2G630338366	40580180	A	0	0	G
3	BICF2P1303594	40592066	A	0.5	0.2857	G
3	BICF2G630338372	40592980	A	0.25	0.4286	G
3	BICF2P367279	40595622	G	0.5	0.2857	A
3	BICF2G630338376	40595785	G	0.25	0.4286	A
3	BICF2P1262655	40608564	A	0.5	0.2857	G
3	BICF2P293107	40614346	G	0.25	0.2857	A
3	BICF2G630338405	40623195	A	0.75	0.7143	G
3	BICF2G630338428	40633388	A	0.25	0.4286	G
3	BICF2G630338450	40647962	A	0.75	0.7143	G
3	BICF2G630338456	40652831	G	0.25	0.4286	A
3	BICF2G630338498	40671896	G	0	0	C
3	BICF2G630338503	40678815	A	0	0	G

3	BICF2G630338529	40690909	A	0.25	0.4286	G
3	BICF2P643134	40704476	G	0.25	0.4286	A
3	BICF2P695312	40714135	G	0.75	0.5714	A
3	BICF2G630338548	40734830	A	0.25	0.2857	G
3	BICF2G630338565	40748674	G	0	0	A
3	BICF2S23754815	40762370	A	0.25	0.2857	C
3	BICF2G630338573	40769567	G	0	0	A
3	BICF2G630338589	40782544	G	0.25	0.2857	A
3	BICF2G630338603	40790580	A	0.25	0.2857	G
3	BICF2G630338622	40804943	A	0	0	C
3	BICF2G630338650	40818313	C	0	0	A
3	BICF2P1225475	40834327	0	0	0	G
3	BICF2G630338653	40844380	A	0	0	G
3	TIGRP2P42263_rs9208556	40858976	G	0	0	A
3	BICF2G630338654	40864626	G	0	0	A
3	BICF2P156497	40873735	G	0	0	A
3	BICF2G630338666	40892957	A	0	0	G
3	BICF2S23445965	40901963	A	0.25	0.4286	C
3	BICF2G630338674	40914488	A	0.5	0.2857	G
3	BICF2P1149544	40921015	A	0.5	0.2857	G
3	BICF2P484635	40934388	0	0	0	G
3	BICF2S22913970	40961557	G	0	0	A
3	BICF2G630338700	40967788	A	0.5	0.7143	G
3	BICF2G630338704	40988917	C	0.5	0.7143	G
3	BICF2P1496229	41013156	A	0	0	C
3	BICF2P275415	41028069	C	0	0	G
3	BICF2G630338718	41040267	C	0.25	0.4286	A
3	BICF2G630338738	41045170	G	0.25	0.4286	A
3	BICF2P684982	41048247	A	0.25	0.4286	G
3	BICF2G630338747	41063353	G	0.25	0.4286	A
3	BICF2G630338778	41078493	G	0.25	0.4286	A
3	BICF2G630338844	41112873	A	0.25	0.4286	G
3	BICF2G630338855	41132703	C	0.25	0.4286	A
3	BICF2S23226602	41157915	A	0	0	G
3	BICF2G630338866	41166759	G	0.75	0.5714	A
3	BICF2P1279830	41180995	0	0	0	A
3	BICF2S23418256	41191394	A	0	0	G
3	BICF2P320286	41206830	0	0	0	G
3	BICF2G630338875	41219385	A	0.25	0.4286	G
3	BICF2P1050747	41227703	G	0	0	A
3	BICF2S23429322	41240470	G	1	1	A
3	BICF2G630338879	41251838	A	0	0	C
3	BICF2G630338891	41265309	G	0	0	A
3	BICF2G630338894	41273930	G	0	0	A
3	BICF2G630338918	41285363	A	0	0	G
3	BICF2G630338927	41303025	A	0	0	C
3	BICF2G630338928	41309053	A	0.25	0.4286	G

3	BICF2P555557	41313663	A	0.25	0.4286	G
3	BICF2G630338935	41324341	A	0.25	0.4286	G
3	BICF2G630338965	41341678	A	0.25	0.4286	C
3	BICF2G630338966	41342635	G	0	0.4167	A
3	BICF2G630338996	41362935	A	0	0	C
3	TIGRP2P42328_rs8767171	41367554	C	0	0	A
3	TIGRP2P42333_rs9149913	41377483	A	0	0	G
3	BICF2G630339022	41393709	0	0	0	A
3	BICF2P496778	41406123	0	0	0	A
3	BICF2P731550	41425748	0	0	0	G
3	BICF2G630339024	41460029	0	0	0	A
3	BICF2S23012614	41508558	A	0	0	G
3	BICF2G630339037	41529556	G	1	1	A
3	BICF2S23162322	41545262	C	0.5	0.2857	G
3	BICF2G630339042	41550594	G	1	1	A
3	BICF2G630339050	41561567	G	0	0	A
3	TIGRP2P42352_rs8560797	41573909	G	0.5	0.2857	A
3	BICF2G630339062	41593312	C	0	0	A
3	BICF2G630339064	41598400	G	0	0	A
3	BICF2G630339071	41608370	G	0.5	0.7143	A
3	BICF2P231278	41619978	0	0	0	A
3	BICF2G630339085	41630484	G	0	0	A
3	BICF2G630339094	41653986	A	0	0	G
3	BICF2S23134459	41655316	G	0.5	0.2857	A
3	BICF2G630339101	41661203	C	0.5	0.7143	G
3	BICF2G630339108	41668800	A	0	0	G
3	BICF2G630339135	41693486	A	0.5	0.25	C
3	TIGRP2P42363_rs9061832	41706030	C	NA	0.2	A
3	BICF2P1260907	41725230	A	0.5	0.7143	G
3	BICF2S236866	41741366	0	0	0	A
3	BICF2G630339144	41750695	A	0.5	0.7143	C
3	BICF2G630339151	41757369	A	0	0	G
3	BICF2G630339162	41768055	A	0.5	0.7143	G
3	BICF2P628739	41780324	A	0	0	G
3	BICF2P1398956	41808103	G	0.5	0.2857	A
3	TIGRP2P42395_rs8869559	41815676	A	0.5	0.2857	G
3	TIGRP2P42402_rs8997601	41831647	A	0	0	G
3	BICF2G630339210	41838029	A	0	0	T
3	BICF2P994362	41853572	G	0	0	A
3	BICF2S23754010	41870926	A	0	0	G
3	BICF2P1216097	41886981	A	0	0	G
3	BICF2G630339226	41912134	A	0	0	G
3	BICF2S22946127	41918728	0	0	0	G
3	BICF2S23054523	41929703	G	0	0	A
3	BICF2P154097	41941924	0	0	0	C
3	BICF2G630339236	41961282	A	0.5	0.2857	G
3	BICF2G630339239	41964494	T	0.75	0.5714	A

3	BICF2S23721391	41975782	G	0.25	0.2857	A
3	BICF2G630339250	42000675	G	0	0	A
3	BICF2G630339255	42010056	A	0.75	0.5714	G
3	BICF2G630339261	42028323	G	0.75	0.5714	A
3	BICF2G630339270	42039748	G	0	0	A
3	BICF2G630339274	42044033	A	1	1	G
3	BICF2G630339296	42088959	G	1	1	A
3	BICF2G630339298	42092803	A	0.5	0.2857	G
3	TIGRP2P42432_rs8463557	42115002	C	0.25	0.2857	G
3	TIGRP2P42442_rs9189133	42138759	A	0.5	0.2857	G
3	TIGRP2P42449_rs9216481	42158410	C	0.5	0.2857	A
3	BICF2P1247015	42169931	A	0.75	0.5714	G
3	BICF2P713044	42177325	A	0.75	0.5714	G
3	TIGRP2P42463_rs9073274	42181548	A	0.75	0.5714	C
3	BICF2P429071	42182140	G	0.75	0.5714	A
3	BICF2P1453717	42194378	0	0	0	A
3	BICF2G630339307	42207073	A	0.75	0.5714	G
3	BICF2G630339317	42219839	A	0.25	0.4286	C
3	BICF2P69735	42229118	G	0	0	A
3	TIGRP2P42480_rs9075195	42242921	G	0	0	A
3	TIGRP2P42481_rs9021241	42253414	G	0	0	A
3	BICF2P1239215	42269704	A	0	0	G
3	BICF2G630339329	42282053	A	0	0	G
3	BICF2G630339373	42307680	A	0.25	0.4286	C
3	BICF2G630339376	42316963	C	0.25	0.4286	A
3	BICF2P555818	42339876	A	0.75	0.5714	G
3	TIGRP2P42493_rs8890264	42348458	G	0	0	A
3	TIGRP2P42494_rs8890947	42358288	A	0	0	G
3	BICF2S22936448	42368395	C	0.75	0.5714	G
3	BICF2S2369266	42385522	G	0	0	A
3	BICF2G630339389	42395352	A	0.5	0.2857	C
3	BICF2G630339399	42404899	A	0.25	0.4286	G
3	BICF2G630339410	42424902	C	0	0	A
3	BICF2G630339421	42438275	A	0	0	G
3	BICF2G630339425	42449579	A	0.25	0.2857	C
3	BICF2P157577	42458005	A	0.25	0.2857	G
3	BICF2P560851	42479513	A	0.25	0.2857	G
3	BICF2G630339433	42492623	G	0.25	0.4286	A
3	BICF2G630339440	42507351	A	0	0	G
3	BICF2P1335812	42516120	A	0	0	G
3	BICF2P689707	42531555	C	0.5	0.7143	A
3	BICF2P265130	42548827	A	1	1	G
3	BICF2G630339473	42560493	G	1	1	A
3	BICF2G630339493	42565168	A	1	1	G
3	BICF2G630339499	42577812	A	0.75	0.7143	C
3	BICF2P165412	42586522	A	0.25	0.2857	G
3	TIGRP2P42524_rs8603007	42600219	G	0.25	0.2857	A

3	TIGRP2P42528_rs8728661	42611622	G	0	0	A
3	BICF2G630339516	42626358	A	0.25	0.2857	C
3	BICF2G630339525	42637201	G	0.25	0.4286	A
3	BICF2P171546	42653266	C	0.25	0.4286	A
3	BICF2G630339542	42659118	A	0.25	0.4286	G
3	BICF2G630339547	42666802	G	0.25	0.4286	A
3	BICF2G630339574	42685261	A	0.25	0.4286	G
3	BICF2G630339584	42694477	G	0.25	0.4286	A
3	BICF2P178696	42710085	G	0.75	0.7143	A
3	TIGRP2P42536_rs9209225	42714862	A	0.75	0.7143	G
3	BICF2S23541261	42722922	A	0.75	0.7143	C
3	TIGRP2P42545_rs8835456	42749134	A	0.75	0.7143	G
3	BICF2P897067	42761487	A	0	0	G
3	BICF2G630339599	42773874	0	0	0	A
3	TIGRP2P42557_rs8860550	42784668	A	0.25	0.2857	G
3	TIGRP2P42559_rs8963357	42793607	G	0.25	0.2857	A
3	BICF2G630339616	42824809	G	0.75	0.5714	A
3	BICF2G630339621	42829136	G	0.5	0.2857	A
3	TIGRP2P42564_rs8919420	42847774	G	0.5	0.2857	A
3	BICF2P914100	42862621	G	0.25	0.25	A
3	TIGRP2P42585_rs9169895	42877793	A	0.25	0.2857	G
3	BICF2P188107	42878112	G	0.25	0.2857	A
3	TIGRP2P42594_rs8943446	42884280	A	0	0	G
3	BICF2P1302336	42896967	A	0	0	G
3	BICF2P32874	42923585	A	0.25	0.4286	G
3	BICF2P559541	42932098	G	0.25	0.4286	A
3	BICF2G630339669	42943284	A	0	0	G
3	BICF2G630339684	42953287	A	0	0	T
3	BICF2G630339707	42969912	G	0	0	A
3	BICF2G630339713	42976711	G	0	0	A
3	BICF2P801184	42987507	0	0	0	G
3	BICF2S23319506	43006057	A	0	0	G
3	BICF2G630339728	43015006	0	0	0	A
3	BICF2P794022	43022894	A	0.25	0.2857	G
3	BICF2P387754	43067111	0	0	0	A
3	BICF2P387756	43067697	A	0	0	G
3	BICF2G630339732	43079314	G	0.25	0.2857	A
3	BICF2G630339738	43092398	C	0.25	0.2857	A
3	BICF2P937567	43094389	A	0.25	0.2857	C
3	BICF2S23213482	43111383	A	0.25	0.2857	G
3	BICF2G630339764	43114029	A	0	0	G
3	BICF2G630339776	43128915	G	0.25	0.2857	A
3	BICF2G630339788	43142694	G	0.25	0.2857	A
3	BICF2G630339794	43145641	T	0.25	0.2857	A
3	BICF2G630339796	43145699	A	0.25	0.2857	G
3	BICF2G630339800	43150128	A	0.25	0.2857	G
3	BICF2G630339806	43159521	A	0.25	0.2857	G

3	G249f24S70	43195229	G	0	0	A
3	BICF2P646966	43216511	0	0	0	A
3	BICF2G630339813	43220000	0	0	0	G
3	BICF2P1334017	43239116	0	0	0	G
3	BICF2P657914	43257377	0	0	0	G
3	BICF2S22937971	43265238	G	0	0	A
3	BICF2G630339821	43274788	0	0	0	A
3	BICF2G630339826	43292397	0	0	0	G
3	BICF2P1146176	43305999	0	0	0	A
3	BICF2G630339828	43313899	0	0	0	T
3	TIGRP2P42614_rs9211918	43340039	A	0.25	0.2857	G
3	BICF2P329270	43355717	A	0	0	G
3	BICF2P909751	43368377	A	0	0	G
3	TIGRP2P42618_rs8993407	43384704	0	0	0	A
3	BICF2P247110	43397621	A	0.75	0.5833	G
3	BICF2G630339832	43409020	G	0	0	A
3	BICF2G630339833	43414411	A	0	0	G
3	TIGRP2P42626_rs8800295	43427329	C	0.75	0.5714	A
3	BICF2P58843	43445996	A	1	1	C
3	BICF2P122621	43449278	0	0	0	G
3	BICF2P1040272	43464965	G	0	0	A
3	BICF2G630339843	43472851	0	0	0	G
3	BICF2G630339850	43489192	G	0	0	A
3	BICF2P898384	43499533	A	0.5	0.2857	G
3	BICF2S23230642	43522701	A	0.25	0.4286	G
3	BICF2S23622321	43552387	G	0	0	A
3	BICF2S23312930	43562629	0	0	0	G
3	BICF2S23415185	43576160	A	0	0	G
3	BICF2P1175015	43587434	0	0	0	A
3	BICF2G630339863	43597477	A	0.5	0.2857	G
3	BICF2G630339865	43599336	A	0.5	0.7143	C
3	BICF2G630339869	43605612	G	0.5	0.7143	A
3	BICF2P1349863	43635120	0	0	0	A
3	BICF2P864209	43651650	G	0.25	0.2857	A
3	BICF2G630339874	43665475	0	0	0	G
3	BICF2P144834	43670112	0	0	0	C
3	BICF2P1352759	43688434	A	0.75	0.7143	G
3	TIGRP2P42645_rs8875823	43694486	A	0.75	0.7143	G
3	BICF2P873434	43702781	G	0.75	0.7143	A
3	BICF2P201136	43715937	A	1	1	G
3	BICF2G630339876	43723923	0	0	0	G
3	TIGRP2P42660_rs8690947	43729382	G	0	0	A
3	BICF2P992772	43754418	A	0	0	G
3	BICF2S23628037	43765344	A	0.5	0.2857	G
3	BICF2P963958	43781636	A	0.25	0.2857	C
3	BICF2G630339885	43793652	G	0.25	0.2857	A
3	TIGRP2P42684_rs9188380	43803902	A	0.5	0.2857	C

3	BICF2G630339923	43816982	A	0.5	0.2857	G
3	BICF2G630339930	43845408	0	0	0	G
3	BICF2G630339931	43871645	0	0	0	A
3	BICF2G630339932	43890044	0	0	0	A
3	BICF2S22943910	43918353	0	0	0	A
3	BICF2P547807	43929331	0	0	0	A
3	G250f27S115	43950009	A	0	0	C
3	BICF2P996261	43977219	0	0	0	G
3	BICF2P127492	43991179	0	0	0	G
3	BICF2G630339936	44003971	A	0.75	0.5714	G
3	BICF2P720168	44021698	0	0	0	A
3	BICF2P1103664	44032340	0	0	0	A
3	BICF2G630339939	44033819	C	0	0	A
3	BICF2P228369	44045545	A	0	0	G
3	TIGRP2P42722_rs9206774	44061336	A	0.5	0.2857	G
3	TIGRP2P42726_rs9145375	44074040	A	0.5	0.2857	C
3	BICF2P1378441	44085302	A	0	0	G
3	BICF2P279364	44099822	A	0.5	0.2857	G
3	BICF2P936560	44103178	G	0.5	0.2857	A
3	BICF2P233888	44114250	G	0.5	0.2857	A
3	BICF2S22941908	44126215	G	0	0	A
3	BICF2S22938870	44147699	G	0.25	0.2857	A
3	BICF2G630339954	44154320	A	0.25	0.2857	G
3	BICF2P1018678	44170507	A	0.5	0.2857	G
3	BICF2P1018675	44171004	A	0.25	0.2857	G
3	BICF2P1192466	44175898	G	0.25	0.2857	A
3	BICF2G630339989	44191164	G	0.5	0.2857	A
3	BICF2G630339993	44194848	A	0.5	0.2857	G
3	BICF2G630340008	44210640	A	0.25	0.2857	G
3	BICF2P393510	44239362	G	0.5	0.2857	A
3	BICF2G630340017	44242160	C	0	0	G
3	BICF2P841996	44254817	G	0.5	0.2857	A
3	TIGRP2P42785_rs9076130	44277025	A	0.5	0.2857	G
3	BICF2G630340031	44292469	A	0.25	0.2857	C
3	BICF2G630340036	44304812	C	0.25	0.4286	A
3	BICF2G630340037	44317100	G	0	0	A
3	BICF2G630340041	44332123	A	0.25	0.2857	C
3	BICF2S229813	44340149	A	0	0	G
3	BICF2P161192	44358425	A	0.75	0.5714	G
3	BICF2P800307	44367451	A	0.5	0.2857	G
3	TIGRP2P42808_rs8796316	44382053	A	0.25	0.2857	G
3	BICF2P716362	44398830	C	0	0	A
3	BICF2S23240123	44405470	A	0.5	0.2857	G
3	TIGRP2P42817_rs8967382	44423299	A	0.5	0.2857	C
3	TIGRP2P42818_rs8618871	44428428	0	0	0	G
3	TIGRP2P42825_rs8720568	44445705	A	0.5	0.2857	C
3	TIGRP2P42827_rs9154080	44448182	A	0.25	0.4286	G

3	TIGRP2P42832_rs8905541	44469087	0	0	0	C
3	BICF2P1417609	44481999	G	0.75	0.5714	A
3	BICF2P1417611	44483632	G	0.75	0.5714	A
3	TIGRP2P42849_rs9217030	44495910	A	0.75	0.5714	G
3	BICF2P1036426	44505514	C	0.75	0.5714	G
3	BICF2G630340058	44519852	G	0.25	0.4286	A
3	BICF2G630340077	44543893	A	0.25	0.2857	G
3	BICF2G630340083	44551778	A	0	0	T
3	BICF2G630340096	44563408	A	0	0	G
3	BICF2G630340121	44592607	C	0	0	A
3	BICF2P183331	44606365	0	0	0	G
3	BICF2G630340127	44615782	A	0	0	G
3	BICF2G630340128	44622942	A	0	0	G
3	BICF2G630340133	44639029	A	0	0	G
3	BICF2S23726155	44648858	G	0.25	0.2857	A
3	TIGRP2P42865_rs9138451	44660125	A	0	0	C
3	BICF2G630340139	44667313	A	0	0	G
3	BICF2G630340148	44683097	G	0	0	A
3	BICF2G630340151	44689464	A	0	0	G
3	TIGRP2P42867_rs8516972	44708539	G	0	0	A
3	BICF2G630340164	44713043	G	0	0	A
3	BICF2G630340171	44721125	A	0	0	G
3	BICF2G630340175	44733126	A	0	0	C
3	BICF2G630340177	44735078	C	0	0	A
3	BICF2G630340182	44750048	C	0	0	A
3	BICF2P889260	44768592	C	0	0	A
3	BICF2G630340191	44780948	G	0	0	A
3	BICF2G630340192	44782059	G	0	0	A
3	BICF2G630340196	44797967	G	0	0	A
3	BICF2G630340201	44821173	A	0	0	G
3	BICF2G630340206	44827161	C	0	0	A
3	BICF2G630340217	44846605	A	0	0	G
3	TIGRP2P42872_rs8820868	44857590	C	0	0	A
3	BICF2G630340220	44864340	A	0	0	C
3	TIGRP2P42876_rs8565984	44884277	G	0.75	0.7143	A
3	BICF2P618831	44886722	0	0	0	G
3	TIGRP2P42879_rs9048996	44899291	G	0.75	0.7143	A
3	BICF2G630340222	44915687	A	0.25	0.2857	G
3	BICF2S23144494	44929188	G	0.25	0.2857	A
3	TIGRP2P42888_rs9083332	44939293	0	0	0	G
3	BICF2P1130044	44952918	0	0	0	G
3	BICF2G630340224	44959747	A	0	0	G
3	BICF2G630340234	44966808	G	0.25	0.2857	A
3	BICF2P1197185	44984911	A	0	0	G
3	BICF2P415419	44994501	A	0.25	0.2857	G
3	BICF2G630340256	45007105	A	0	0	G
3	BICF2P1415923	45026840	A	0.75	0.7143	G

3	BICF2G630340269	45026946	0	0	0	G
3	BICF2G630340273	45038064	G	0.75	0.7143	A
3	TIGRP2P42917_rs8640918	45053192	0	0	0	G
3	BICF2P524350	45064899	G	0.75	0.7143	A
3	BICF2P1213979	45075558	G	0.75	0.7143	A
3	BICF2P24040	45088775	A	1	0.7143	G
3	TIGRP2P42919_rs8861158	45108681	G	0	0	A
3	BICF2P65875	45117124	G	0	0	A
3	BICF2G630340305	45134667	A	0.25	0.2857	G
3	BICF2G630340312	45147303	A	0.25	0.2857	G
3	BICF2G630340316	45150218	A	0.25	0.2	G
3	BICF2G630340334	45161257	G	0	0	A
3	BICF2G630340347	45177848	G	0.75	0.7143	A
3	BICF2S23437438	45185965	0	0	0	G
3	BICF2G630340360	45201605	A	0.75	0.7143	C
3	BICF2G630340367	45209848	A	0	0	C
3	BICF2G630340375	45219756	A	0	0	G
3	BICF2G630340390	45231951	A	0	0	G
3	BICF2G630340420	45251647	A	0	0	G
3	BICF2G630340423	45252005	A	0	0	C
3	BICF2G630340424	45252198	G	0	0	C
3	BICF2G630340425	45255398	G	0	0	A
3	BICF2G630340429	45286264	A	0	0	G
3	BICF2G630340454	45308695	A	0	0	G
3	BICF2G630340458	45315009	A	0	0	G
3	BICF2G630340488	45341118	G	0	0	C
3	BICF2G630340501	45344845	A	0	0	G
3	BICF2G630340542	45356122	G	0	0	A
3	BICF2G630340566	45368222	G	0	0	A
3	BICF2G630340591	45383843	C	0	0	A
3	BICF2G630340595	45391606	G	1	0.7143	A
3	BICF2G630340601	45406561	A	0.75	0.75	G
3	BICF2P469832	45410993	A	0	0	G
3	BICF2G630340609	45422732	C	0	0	A
3	BICF2G630340624	45435818	C	0	0	A
3	BICF2G630340647	45441892	A	0	0	G
3	BICF2G630340654	45455674	G	0	0	A
3	BICF2G630340666	45461295	A	1	1	C
3	BICF2P1003371	45481237	A	0	0	G
3	BICF2G630340679	45483408	G	0.75	0.7143	A
3	BICF2G630340701	45487806	A	0.75	0.7143	G
3	BICF2G630340720	45497515	G	0.75	0.7143	A
3	BICF2G630340731	45507463	T	0.75	0.7143	A
3	BICF2G630340744	45523141	A	NA	0.5	C
3	BICF2S23324122	45536639	A	0	0	G
3	BICF2S2338165	45545376	A	0	0	C
3	BICF2P1182264	45574148	C	0	0	G

3	BICF2S23016977	45594832	A	0.75	0.7143	G
3	TIGRP2P42942_rs8664578	45643432	0	0	0	C
3	BICF2S23635003	45657202	G	0.25	0.2857	A
3	BICF2G630340753	45676480	A	0.5	0.2857	G
3	BICF2P299391	45683637	0	0	0	A
3	BICF2S23540043	45696135	A	0.25	0.2857	G
3	BICF2G630340757	45705417	C	0.25	0.2857	A
3	BICF2G630340773	45718344	A	0	0	G
3	BICF2S2359143	45725878	G	0	0	A
3	BICF2G630340813	45746954	G	0.25	0.4286	A
3	BICF2G630340827	45750809	A	0.25	0.4286	G
3	BICF2P1150459	45768351	A	0	0	G
3	BICF2G630340850	45774525	G	0	0	A
3	BICF2P207885	45784224	0	0	0	A
3	BICF2G630340864	45793545	G	0	0	A
3	BICF2G630340881	45808638	C	0.25	0.3333	G
3	BICF2G630340902	45820255	A	0	0	G
3	BICF2G630340909	45831376	G	0.5	0.2857	A
3	BICF2G630340913	45834497	G	0.5	0.2857	A
3	BICF2G630340916	45844780	G	0	0	A
3	BICF2G630340922	45852748	G	0.75	0.7143	A
3	BICF2G630340927	45862403	A	0	0	G
3	BICF2G630340930	45883843	A	0.25	0.4286	G
3	BICF2G630340931	45885643	G	0	0	A
3	BICF2G630340934	45898121	G	0	0	A
3	BICF2G630340940	45912570	G	0	0	A
3	BICF2G630340944	45920551	G	0.25	0.4286	A
3	BICF2P1013202	45932586	G	0	0	A
3	BICF2G630340989	45960132	A	1	1	C
3	BICF2G630341007	45968752	G	0.25	0.4286	A
3	BICF2G630341029	45981406	A	0.75	0.5714	G
3	TIGRP2P42952_rs8811198	45995672	0	0	0	C
3	BICF2S23113325	46008771	0	0	0	G
3	BICF2G630341058	46013313	G	0.75	0.5714	A
3	BICF2S2359785	46041527	A	0.25	0.2857	G
3	BICF2G630341088	46046826	G	0	0	A
3	BICF2G630341108	46058345	A	0.25	0.2857	G
3	BICF2P554775	46072451	A	0.5	0.7143	G
3	BICF2G630341140	46082745	G	0	0	A
3	BICF2G630341141	46082799	G	0	0	A
3	BICF2P226583	46092902	C	0.5	0.7143	A
3	BICF2P705880	46109101	G	1	1	A
3	BICF2P225883	46119146	G	0.75	0.7143	A
3	BICF2P210686	46126626	0	0	0	A
3	BICF2S24113788	46129799	G	0.75	0.7143	A
3	BICF2G630341177	46147426	G	0.75	0.7143	A
3	BICF2G630341181	46150382	A	0.75	0.7143	G

3	BICF2G630341190	46169521	A	0.5	0.2857	G
3	BICF2G630341202	46186173	A	0	0	C
3	G251f39S186	46208036	0	0	0	G
3	BICF2P1003568	46229417	0	0	0	G
3	BICF2S23410777	46235344	A	0	0	G
3	BICF2G630341206	46243521	A	0.5	0.2857	G
3	BICF2G630341221	46255879	A	0.5	0.25	C
3	BICF2G630341226	46268865	G	0.5	0.2857	A
3	BICF2G630341238	46282868	A	0.5	0.2857	G
3	BICF2S23610689	46289220	A	0.75	0.7143	G
3	BICF2G630341243	46312671	A	0	0	G
3	BICF2P352000	46343201	G	0.25	0.4286	A
3	BICF2P511785	46346796	A	0.25	0.4286	G
3	BICF2G630341245	46364683	A	0.75	0.5714	G
3	BICF2G630341258	46372543	G	0	0	A
3	TIGRP2P42954_rs9149511	46390942	0	0	0	G
3	BICF2G630341259	46399999	G	0.75	0.7143	A
3	BICF2G630341260	46409354	0	0	0	G
3	BICF2G630341264	46458625	G	0	0	A
3	BICF2G630341268	46467337	A	0	0	G
3	BICF2G630341271	46498642	A	0.5	0.25	C
3	BICF2G630341284	46506458	A	0.5	0.2857	G
3	BICF2G630341298	46523619	A	0.5	0.2857	C
3	BICF2G630341305	46530984	C	0	0	A
3	BICF2G630341314	46542275	C	0	0	A
3	BICF2S23160949	46564837	A	0	0	G
3	BICF2S23649456	46616382	G	0.5	0.2857	A
3	BICF2G630341331	46631875	G	0.5	0.2857	A
3	BICF2G630341336	46639400	C	0.25	0.4286	A
3	BICF2G630341356	46646251	A	0.25	0.4286	G
3	BICF2P703683	46683073	0	0	0	A
3	BICF2G630341360	46691807	A	0	0	G
3	BICF2P669696	46702233	A	0	0	G
3	BICF2G630341363	46739684	0	0	0	T
3	BICF2G630341366	46748610	G	0	0	A
3	TIGRP2P42963_rs9092855	46760455	0	0	0	C
3	BICF2S23153423	46772012	A	0.25	0.4286	G
3	BICF2P304511	46792256	0	0	0	T
3	BICF2G630341376	46825437	A	0.5	0.2857	C
3	BICF2G630341391	46832927	A	0.5	0.2857	G
3	BICF2G630341403	46843957	A	0.75	0.7143	C
3	BICF2G630341410	46851899	G	0.75	0.7143	A
3	BICF2G630341422	46856261	G	0.75	0.7143	C
3	BICF2P1410028	46864213	A	0.75	0.5714	G
3	BICF2G630341438	46874044	G	0	0	A
3	BICF2G630341451	46886951	G	0	0	A
3	BICF2P1018831	46908459	G	0	0	C

3	BICF2G630341479	46917064	G	0.25	0.2857	A
3	BICF2G630341483	46924332	A	0.25	0.2857	G
3	BICF2S22935424	46950934	A	0.5	0.2857	G
3	BICF2G630341491	46977052	A	0.5	0.7143	G
3	BICF2G630341492	46984347	G	0.5	0.2857	C
3	BICF2G630341493	46992550	A	0.5	0.2857	G
3	BICF2P567380	47012346	0	0	0	C
3	BICF2G630341496	47033045	A	0	0	G
3	BICF2P1426685	47046274	A	0	0	C
3	BICF2P804475	47052807	A	0	0	G
3	BICF2P1453798	47064332	0	0	0	C
3	BICF2P227781	47073418	A	0	0	G
3	BICF2P932403	47078606	A	0	0	G
3	BICF2P368071	47086715	A	0	0	C
3	BICF2P489172	47107220	G	0	0	A
3	BICF2P1127703	47117124	0	0	0	A
3	BICF2P1025882	47135124	0	0	0	G
3	BICF2P645938	47147533	0	0	0	A
3	TIGRP2P42971_rs8860098	47151340	0	0	0	G
3	BICF2G630341507	47164178	A	0.75	0.7143	G
3	BICF2P1004673	47175240	G	0.75	0.7143	A
3	BICF2P1041506	47187165	A	0.75	0.7143	T
3	BICF2P1058326	47194514	G	0.75	0.7143	A
3	BICF2P1058327	47194645	G	0.75	0.7143	A
3	BICF2G630341528	47198379	C	0.5	0.7143	A
3	BICF2P841598	47213014	A	0.75	0.6	C
3	BICF2S23034296	47229398	C	0.5	0.2857	A
3	BICF2P286079	47237379	A	0.5	0.2857	C
3	BICF2G630341544	47260773	A	0.75	0.7143	C
3	BICF2P64775	47266457	G	0.25	0.4286	A
3	BICF2G630341548	47282285	A	0	0	G
3	BICF2S23551674	47299463	A	0	0	T
3	BICF2P1075243	47304911	T	0	0	A
3	BICF2G630341550	47319614	G	0	0	A
3	BICF2S23625698	47326704	A	0.75	0.5714	G
3	BICF2S23433417	47331901	A	0.5	0.7143	G
3	BICF2S23550005	47338479	G	0	0	A
3	BICF2S2359863	47343020	G	0	0	A
3	BICF2S23417238	47348754	A	0.25	0.2857	G
3	BICF2G630341593	47360896	G	0	0	A
3	BICF2G630341597	47368642	A	0	0	G
3	BICF2G630341612	47382183	G	0.5	0.2857	A
3	BICF2G630341618	47392700	G	0.75	0.5714	A
3	BICF2G630341654	47408410	A	0	0	G
3	BICF2S22947478	47420606	A	0	0	G
3	TIGRP2P107604_rs8885113	47426493	0	0	0	G
3	BICF2G630341660	47441910	G	0	0	A

3	BICF2G630341661	47462027	G	0.5	0.2857	A
3	BICF2G630341669	47474157	G	0.5	0.2857	C
3	BICF2P327833	47484646	A	0	0	G
3	BICF2S2366790	47487148	G	0.5	0.2857	A
3	TIGRP2P43002_rs8613954	47499394	A	0.5	0.2857	C
3	TIGRP2P43006_rs8763585	47508529	A	0.5	0.2857	G
3	BICF2P94700	47518643	A	0	0	G
3	TIGRP2P43009_rs9194154	47531082	A	0	0	G
3	BICF2G630341680	47543276	G	0	0	A
3	BICF2G630341701	47554070	G	1	0.75	A
3	BICF2G630341713	47574578	G	0.5	0.7143	A
3	BICF2G630341724	47585499	C	0.25	0.4286	G
3	BICF2G630341726	47590969	C	0	0	A
3	BICF2G630341756	47606009	A	0.5	0.2857	G
3	BICF2G630341767	47611665	G	0.75	0.7143	A
3	BICF2G630341786	47627442	A	0.5	0.2857	G
3	BICF2G630341790	47633164	A	0	0	G
3	BICF2G630341794	47649379	A	0.25	0.2857	G
3	BICF2G630341809	47657671	A	0	0	G
3	BICF2G630341819	47662119	G	0	0	A
3	BICF2G630341820	47677479	0	0	0	A
3	BICF2P321592	47692430	G	0.25	0.2857	A
3	BICF2P701892	47710735	A	0.5	0.7143	G
3	BICF2P111391	47716066	A	0.25	0.4286	G
3	BICF2P270861	47732652	A	0.5	0.2857	G
3	BICF2P632856	47742699	A	0.25	0.2857	G
3	BICF2G630341822	47764251	0	0	0	G
3	BICF2P457992	47778623	A	0.25	0.4286	G
3	TIGRP2P43067_rs8950841	47786975	A	0	0	G
3	TIGRP2P43073_rs8796758	47797799	A	0	0	G
3	BICF2P1506515	47810382	A	0	0	G
3	BICF2G630341829	47822039	C	0.5	0.7143	A
3	BICF2P1097910	47848325	A	0.5	0.2857	G
3	BICF2G630341867	47856255	A	0.5	0.2857	C
3	BICF2S2313861	47864535	A	0	0	G
3	BICF2G630341893	47889869	G	0	0	A
3	BICF2G630341905	47899656	G	0.25	0.4286	A
3	BICF2G630341922	47910549	C	0.25	0.4286	A
3	BICF2G630341924	47915261	A	0.25	0.4286	G
3	BICF2G630341930	47924396	A	0.25	0.2857	G
3	BICF2P626775	47938817	G	0	0	C
3	BICF2P881714	47944625	A	0	0	G
3	BICF2G630341953	47944786	A	0.25	0.2857	G
3	BICF2P1259613	47963041	G	0	0	A
3	BICF2P727856	47973222	A	0.5	0.2857	G
3	TIGRP2P43123_rs9078122	47983585	A	0.25	0.4286	G
3	BICF2P544095	47991544	A	0	0	C

3	TIGRP2P43128_rs9154743	48003038	C	0	0	A
3	BICF2P1460684	48013824	G	0	0	A
3	TIGRP2P43139_rs8747445	48026036	A	0	0	G
3	BICF2P620981	48042273	C	0.25	0.3571	G
3	TIGRP2P43141_rs8556949	48054854	G	0	0	A
3	BICF2P66852	48059810	G	0	0	A
3	TIGRP2P43176_rs8829751	48141454	0	0	0	A
3	BICF2P46955	48180068	A	0.25	0.2857	G
3	BICF2P170932	48195150	G	0.5	0.6429	A
3	BICF2P1116462	48202767	C	0.25	0.2857	A
3	BICF2P416619	48217782	G	0.25	0.2857	A
3	BICF2P245087	48230071	A	0.25	0.2857	C
3	BICF2P790800	48233470	G	0	0	A
3	TIGRP2P43195_rs8711997	48266577	A	0.25	0.2857	G
3	BICF2P646035	48314597	G	0.25	0.25	A
3	BICF2P1041612	48326158	A	0.25	0.2857	G
3	BICF2P952644	48340282	G	0.5	0.3571	C
3	BICF2P411942	48356813	G	0.25	0.2857	A
3	BICF2S2357706	48366720	A	0.25	0.3571	C
3	BICF2P1414521	48387997	G	0.25	0.3571	A
3	TIGRP2P43242_rs9142432	48392545	G	0.25	0.3571	A
3	BICF2P509355	48406122	A	0.25	0.3571	G
3	BICF2P69764	48423251	A	0.25	0.3571	C
3	BICF2S23211360	48456575	C	0	0	A
3	BICF2P622583	48468443	0	0	0	G
3	BICF2P508979	48475515	A	0	0	G
3	BICF2P617639	48491565	A	0.5	0.3571	G
3	BICF2P1012045	48500539	G	0.5	0.3571	C
3	BICF2P957732	48515755	A	0	0	C
3	BICF2P429917	48518810	G	0	0	A
3	TIGRP2P43277_rs9112867	48534330	0	0	0	A
3	BICF2P775828	48544454	0	0	0	G
3	BICF2S23419889	48557713	G	0.5	0.3333	A
3	TIGRP2P43282_rs8951545	48582174	G	0.5	0.3571	A
3	BICF2S23434837	48602309	0	0	0	G
3	TIGRP2P43290_rs8904658	48619926	G	0.25	0.2857	A
3	BICF2S23763526	48622291	G	0.25	0.2857	A
3	TIGRP2P43296_rs9251047	48636076	A	0	0	C
3	BICF2P1059287	48652307	C	0.5	0.3571	A
3	BICF2P1409573	48656627	0	0	0	G
3	BICF2P81078	48674051	G	0.25	0.2857	A
3	BICF2P386725	48680965	G	0.25	0.2857	A
3	BICF2S23343347	48706968	C	0.5	0.3571	A
3	TIGRP2P43310_rs8884436	48715280	0	0	0	G
3	TIGRP2P43320_rs8941222	48734163	C	0.25	0.3571	A
3	BICF2P710537	48737054	G	0	0	A
3	BICF2P905084	48753066	T	0	0	A

3	TIGRP2P43338_rs8711347	48800145	A	0.5	0.3571	G
3	BICF2S2345469	48806975	A	0.5	0.3571	G
3	BICF2P408958	48819877	A	0.5	0.6429	T
3	BICF2P1382682	48837097	G	0	0	A
3	BICF2P269531	48843117	A	0	0	G
3	BICF2S23637784	48853189	A	0	0	G
3	BICF2P1109077	48873558	A	1	1	G
3	BICF2P1431921	48878860	A	1	1	G
3	BICF2P241884	48880579	A	1	1	G
3	BICF2P690735	48886741	A	0	0	G
3	BICF2P818390	48903088	A	0	0	C
3	BICF2P1010563	48914729	C	0	0	A
3	BICF2P771998	48921928	A	0.5	0.3571	G
3	BICF2S23331916	48932783	G	0	0	A
3	BICF2P460650	48961588	A	0.5	0.6429	G
3	BICF2P497253	48971248	G	0.5	0.6429	A
3	BICF2P575277	48983968	A	0.5	0.6429	G
3	BICF2S23114851	49000545	A	0.5	0.6429	G
3	TIGRP2P43409_rs9238745	49002268	G	0.5	0.6429	A
3	BICF2P1143318	49027809	A	0	0	G
3	BICF2P360179	49039696	G	0	0	A
3	BICF2S23054481	49070024	0	0	0	A
3	BICF2S23210420	49080479	A	0.5	0.6429	G
3	BICF2P199404	49085376	A	0	0	C
3	BICF2P573237	49099344	0	0	0	G
3	BICF2P794721	49105243	0	0	0	G
3	BICF2P1213834	49119013	0	0	0	G
3	BICF2S23252915	49133642	A	0	0	G
3	BICF2P939368	49176126	A	0	0	G
3	TIGRP2P43446_rs8921649	49198264	A	0	0	C
3	BICF2P357072	49225143	0	0	0	A
3	BICF2P685913	49247737	T	0	0	A
3	BICF2P801170	49261686	G	0	0	A
3	BICF2P740677	49278072	C	1	1	A
3	BICF2P452121	49294235	G	0	0	A
3	BICF2S23431990	49310104	A	0	0	G
3	BICF2S23344894	49321425	A	1	1	C
3	BICF2S23344617	49325263	A	1	1	G
3	BICF2S23545799	49367005	A	0.5	0.6429	G
3	BICF2S23322476	49378670	A	0.5	0.6429	G
3	BICF2S23313295	49390061	A	1	1	T
3	BICF2P552173	49411858	G	0.75	0.6429	A
3	BICF2P118183	49423415	A	0	0	G
3	TIGRP2P43454_rs8930478	49433529	G	1	1	A
3	BICF2P977875	49446545	A	0	0	G
3	BICF2P416762	49454281	A	0	0	G
3	TIGRP2P43467_rs9191118	49468675	0	0	0	G

3	BICF2P1462174	49480516	G	0.25	0.2857	A
3	BICF2S23634659	49490953	G	0	0	A
3	BICF2P1244112	49504025	G	0	0	A
3	TIGRP2P43480_rs8711497	49508273	T	0	0	A
3	BICF2S22935902	49524907	0	0	0	G
3	BICF2P1371167	49538829	A	0	0	G
3	BICF2S2444296	49550147	0	0	0	A
3	TIGRP2P43485_rs9072900	49562707	0	0	0	G
3	BICF2P104696	49569663	0	0	0	A
3	BICF2P1081431	49579647	G	0	0	A
3	BICF2P336849	49591455	A	0	0	G
3	BICF2P689516	49600756	A	0.75	0.7143	G
3	BICF2P411522	49602835	A	0	0	G
3	BICF2P216217	49619879	G	0	0	A
3	BICF2P1109826	49628484	G	0	0	A
3	BICF2P992312	49638463	0	0	0	C
3	TIGRP2P43520_rs8566114	49646752	C	0	0	A
3	BICF2P777990	49660497	G	0	0	A
3	BICF2P6182	49680612	G	1	1	A
3	TIGRP2P43535_rs8522305	49703366	A	0.25	0.2857	G
3	TIGRP2P43536_rs8983965	49721051	0	0	0	C
3	BICF2S23913703	49743106	0	0	0	G
3	BICF2P124425	49759222	0	0	0	A
3	TIGRP2P43539_rs9129306	49763180	A	0.25	0.3571	G
3	BICF2P1280201	49776468	A	0.25	0.3571	G
3	BICF2P920675	49807406	0	0	0	A
3	BICF2P904947	49843003	0	0	0	C
3	BICF2P180878	49852688	A	0.5	0.3571	C
3	BICF2S23716406	49864849	G	0	0	C
3	BICF2P799126	49905504	C	0.75	0.7143	A
3	BICF2P119710	49916642	0	0	0	A
3	BICF2P71635	49941323	0	0	0	C
3	BICF2P288751	49952729	A	0.5	0.3571	G
3	BICF2S23049367	49965293	A	0	0	G
3	BICF2S23427095	49996694	A	0	0	G
3	BICF2P599622	50069770	0	0	0	G
3	BICF2S23052135	50074261	A	0	0	G
3	BICF2P1237424	50101305	G	0	0	A
3	BICF2P279463	50107992	G	0.25	0.2857	C
3	BICF2S23058351	50133368	A	0.25	0.2857	G
3	TIGRP2P43568_rs8991953	50145224	G	0.25	0.2857	A
3	BICF2P1040112	50157317	G	0.25	0.3571	A
3	BICF2P1081093	50174116	0	0	0	A
3	BICF2S23524990	50178660	A	0	0	G
3	BICF2P136191	50193904	0	0	0	A
3	BICF2P140578	50209351	A	0	0	G
3	BICF2P1418437	50226414	A	0.5	0.3571	G

3	TIGRP2P43587_rs8708977	50247389	0	0	0	G
3	BICF2P1223807	50255938	G	0.25	0.3571	A
3	TIGRP2P43599_rs9064856	50267225	0	0	0	G
3	BICF2P905416	50290207	C	0.5	0.6667	A
3	BICF2P146329	50304137	A	0	0	G
3	TIGRP2P43611_rs8764675	50318315	0	0	0	G
3	BICF2P699800	50330095	C	0.25	0.2857	A
3	BICF2P1367028	50336106	G	0.25	0.2857	A
3	BICF2P1349104	50359092	C	0.25	0.2857	A
3	BICF2S23326476	50370738	0	0	0	G
3	BICF2P649296	50389940	A	0	0	G
3	BICF2P257821	50398822	A	0	0	G
3	BICF2P257820	50398905	A	0	0	C
3	BICF2P235641	50403783	A	0	0	G
3	BICF2P235640	50403922	G	0	0	A
3	TIGRP2P43642_rs8665397	50404660	G	0	0	A
3	BICF2S23150313	50425248	A	0	0	G
3	TIGRP2P43677_rs8680257	50427964	A	0	0	G
3	BICF2P968952	50447243	A	NA	0.3571	G
3	TIGRP2P43686_rs8535575	50455954	A	0	0	G
3	BICF2P576935	50473364	A	0	0	G
3	BICF2P888225	50480269	G	0	0	A
3	BICF2P1121544	50508105	A	0	0	G
3	BICF2P902317	50509722	A	0	0	G
3	BICF2P901297	50516162	G	0	0	A
3	TIGRP2P43703_rs8619113	50521165	G	0	0	A
3	TIGRP2P43707_rs8784101	50539646	A	0	0	G
3	TIGRP2P43712_rs8544229	50548541	A	0	0	G
3	BICF2P196697	50554700	A	0.25	0.2857	G
3	BICF2P66339	50571877	A	0.75	0.6429	G
3	BICF2P970603	50577823	A	0.75	0.6429	G
3	BICF2P548052	50580673	A	0.75	0.6429	G
3	BICF2P261287	50608403	A	1	1	G
3	TIGRP2P43732_rs9088588	50632464	C	1	1	A
3	TIGRP2P43736_rs8654288	50653808	C	0.5	0.4167	A
3	BICF2S23313783	50664319	G	1	1	A
3	TIGRP2P43747_rs9174560	50674011	A	1	1	G
3	BICF2P934094	50690325	G	0.75	0.7143	A
3	BICF2P379254	50697288	A	0.75	0.7143	C
3	TIGRP2P43764_rs8649845	50712595	A	0	0	G
3	TIGRP2P43766_rs8649844	50715130	A	0	0	G
3	BICF2P471365	50737830	A	0	0	G
3	BICF2P69681	50740095	A	0	0	G
3	BICF2S23431126	50767096	A	0	0	G
3	BICF2P908788	50783161	A	0.25	0.2857	G
3	BICF2P915945	50788221	G	0.5	0.3571	C
3	BICF2P681112	50797287	G	0.25	0.2857	A

3	TIGRP2P43786_rs8652092	50823311	A	0.75	0.6429	C
3	BICF2P466157	50825738	A	0.5	0.3571	C
3	BICF2S23325013	50838957	A	0.75	0.6429	G
3	BICF2P716851	50842299	A	0	0	G
3	BICF2P1141190	50853483	A	0	0	G
3	BICF2P601694	50867172	G	0	0	A
3	BICF2P1221529	50879889	A	0.75	0.6429	G
3	BICF2S2412246	50914556	A	0	0	G
3	TIGRP2P43832_rs8800673	50924601	A	0	0	C
3	BICF2P409204	50940931	A	0	0	G
3	TIGRP2P43842_rs8817770	50945638	A	0	0	G
3	BICF2P856624	50961131	G	0.25	0.2857	A
3	BICF2S23325948	50962554	A	0.25	0.2857	C
3	BICF2S2338207	50972108	C	0	0	A
3	BICF2P555991	50983719	A	0	0	G
3	BICF2P28182	51010226	C	0	0	G
3	TIGRP2P43888_rs8831376	51018465	A	0	0	G
3	BICF2P923746	51018487	G	0	0	A
3	BICF2P17016	51043706	A	0.25	0.2857	G
3	BICF2P920304	51055740	G	0.25	0.2857	A
3	BICF2S23715254	51075543	A	0.5	0.3571	C
3	TIGRP2P43926_rs9027764	51083690	G	0.5	0.3571	A
3	BICF2P179731	51094337	A	0	0	G
3	BICF2P179733	51094956	A	0	0	G
3	BICF2P299996	51114246	A	0.75	0.6429	G
3	TIGRP2P43932_rs9158460	51127898	A	0	0	G
3	BICF2P572576	51134367	G	0.25	0.2857	A
3	TIGRP2P43956_rs8976778	51141845	0	0	0	G
3	BICF2P65435	51155555	G	0.5	0.3571	A
3	TIGRP2P43980_rs9084836	51165073	G	0.25	0.2857	A
3	BICF2P1093594	51179049	A	0.25	0.3571	C
3	TIGRP2P43987_rs8645164	51194893	A	0.75	0.6429	G
3	BICF2P95716	51207726	A	0.5	0.3571	G
3	BICF2P1385988	51210397	G	0	0	A
3	BICF2P170040	51221027	G	0.5	0.3571	A
3	BICF2P1230346	51222231	G	0.5	0.3571	A
3	BICF2S22937296	51241822	G	0.5	0.3571	A
3	BICF2P1243654	51247299	C	0.25	0.3571	A
3	BICF2P591088	51275771	A	0.25	0.3333	C
3	BICF2P1010983	51279055	A	0	0.2857	G
3	TIGRP2P44021_rs8775299	51290232	A	0	0	G
3	BICF2P1110357	51307598	A	0	0	G
3	BICF2P1315838	51315230	C	0	0	A
3	BICF2P235961	51343538	A	0.25	0.3571	G
3	BICF2P707145	51350669	G	0	0	A
3	BICF2P408573	51377296	G	0.25	0.2857	A
3	BICF2P1108386	51378652	A	0.25	0.2857	G

3	BICF2P422004	51386480	A	0	0	C
3	BICF2P117647	51399957	G	0	0	A
3	TIGRP2P44070_rs8966334	51405959	G	0	0	A
3	TIGRP2P44084_rs8735993	51418812	A	0.25	0.2857	G
3	BICF2P83480	51428513	A	0.75	0.6429	G
3	BICF2P590394	51439957	C	0.75	0.6429	A
3	BICF2P792339	51457098	A	0.75	0.7143	G
3	BICF2P736243	51466307	G	0.25	0.2857	A
3	BICF2P823623	51474541	C	0.25	0.3571	A
3	BICF2P861827	51496222	A	0	0	G
3	BICF2P950127	51517073	A	0	0	G
3	TIGRP2P44134_rs8816289	51517240	A	0	0	G
3	BICF2P820977	51524876	G	0.25	0.3571	A
3	BICF2P874397	51541371	G	0	0	A
3	BICF2S23658443	51546152	A	0.25	0.3571	G
3	BICF2P1121178	51555795	A	0	0	G
3	TIGRP2P44162_rs9076906	51568325	A	0	0	G
3	BICF2P193711	51582025	G	0.25	0.2857	A
3	BICF2S22940000	51597793	G	0.5	0.3571	A
3	TIGRP2P44186_rs8812771	51621549	A	0.5	0.3333	G
3	TIGRP2P44188_rs8717237	51630672	A	0.5	0.6429	G
3	BICF2P1027127	51641125	G	0	0	A
3	TIGRP2P44206_rs8905582	51651301	G	1	1	C
3	BICF2P52992	51663319	G	0.75	0.6429	A
3	BICF2S2451509	51672879	G	0	0	A
3	BICF2P663151	51681296	G	0.5	0.3571	A
3	TIGRP2P44217_rs8524162	51696939	A	0.5	0.3571	G
3	BICF2P111009	51704709	G	0.5	0.6429	A
3	BICF2S23453029	51711327	A	0.5	0.6429	G
3	BICF2P893059	51723615	A	0.5	0.3571	G
3	BICF2P991573	51763781	G	0.25	0.2857	A
3	BICF2P1212481	51773534	A	0.75	0.7143	G
3	BICF2P229582	51798802	A	0.25	0.3571	C
3	BICF2P921155	51813605	G	0.5	0.3571	A
3	BICF2P1308013	51819602	C	0.5	0.3571	A
3	BICF2P412106	51836152	A	0.25	0.3571	G
3	BICF2S23334142	51849664	G	0	0.3333	A
3	BICF2P284629	51855646	G	0.25	0.2857	A
3	TIGRP2P44253_rs8686903	51863124	C	0.5	0.3571	G
3	TIGRP2P44260_rs8695140	51869204	G	0.5	0.3571	A
3	BICF2P266385	51877782	A	0.25	0.2857	G
3	BICF2P1082896	51893285	C	0.5	0.3571	A
3	BICF2P554275	51913312	G	0.5	0.3571	A
3	BICF2P300063	51924565	A	0	0	G
3	BICF2P274947	51937230	A	0.25	0.3571	G
3	TIGRP2P44272_rs9137246	51953845	T	0	0	A
3	BICF2P1146917	51958700	G	0	0	A

3	BICF2P1263809	51971193	G	0.5	0.3571	A
3	TIGRP2P44276_rs8613795	51988373	0	0	0	G
3	TIGRP2P44278_rs8764029	52017595	A	0.5	0.3571	G
3	BICF2P114641	52026619	A	0.5	0.3333	G
3	BICF2P601431	52028635	G	0.75	0.6429	A
3	TIGRP2P44281_rs9103118	52040593	G	0.5	0.3571	A
3	TIGRP2P44283_rs8868505	52058570	G	0.5	0.3333	A
3	BICF2P1260010	52063515	G	0.5	0.3571	A
3	BICF2P573109	52072336	G	0.5	0.3571	A
3	BICF2S23435447	52087102	C	0.5	0.3571	G
3	TIGRP2P44286_rs9169939	52095041	C	0.5	0.3571	A
3	BICF2P876669	52113353	A	0.5	0.3333	G
3	TIGRP2P44290_rs8625527	52124026	A	0.5	0.3571	G
3	BICF2P944359	52138319	A	0.5	0.3571	G
3	TIGRP2P44296_rs9147030	52142664	A	0.5	0.3571	G
3	TIGRP2P44300_rs8748342	52162947	G	0.5	0.3571	A
3	TIGRP2P44301_rs8895405	52170087	A	0.5	0.3571	G
3	BICF2P687613	52184775	G	0.25	0.25	A
3	BICF2P528674	52196205	C	0	0	A
3	BICF2P46818	52200995	A	0.75	0.7143	C
3	BICF2S23649907	52220465	A	0.25	0.3571	G
3	BICF2P1332217	52231894	A	0.25	0.3571	G
3	BICF2P178716	52235252	A	0	0	G
3	BICF2S2334503	52254560	G	0.75	0.6429	A
3	BICF2P244978	52264153	0	0	0	G
3	BICF2S23442987	52269077	G	0.25	0.3571	A
3	BICF2S23430879	52294303	C	0	0	A
3	BICF2P885986	52313018	A	0.75	0.7143	G
3	BICF2P1390236	52315046	A	0.75	0.7143	C
3	BICF2P1101079	52335942	G	0	0	A
3	BICF2P745285	52338496	C	0	0	A
3	BICF2P1284188	52355369	T	0	0	A
3	BICF2P1284189	52355386	0	0	0	G
3	TIGRP2P44401_rs8623038	52367323	G	0.75	0.6429	A
3	TIGRP2P44407_rs8710523	52373627	A	0	0	C
3	BICF2P889119	52387462	0	0	0	A
3	TIGRP2P44416_rs8663241	52395675	G	0	0	A
3	BICF2S23014752	52431157	G	0.5	0.3571	A
3	BICF2P1125015	52448049	0	0	0	G
3	TIGRP2P44421_rs8819250	52451735	A	0	0	G
3	BICF2P50199	52466631	0	0	0	A
3	BICF2P944575	52485526	A	0.5	0.6429	G
3	BICF2P944569	52486561	A	0.5	0.6429	C
3	BICF2P1422190	52498099	G	0.5	0.6429	A
3	BICF2P1057404	52515137	A	0	0	G
3	BICF2P234063	52529658	A	0.75	0.6429	C
3	TIGRP2P44447_rs9028694	52537221	A	0.75	0.6429	G

3	BICF2P63522	52548350	G	1	1	A
3	BICF2P743032	52564401	G	0.75	0.7143	A
3	BICF2P1108719	52572108	G	0.75	0.7143	A
3	BICF2P1038875	52586945	A	0.25	0.2857	G
3	BICF2S23520724	52598578	A	0	0	C
3	BICF2S23715583	52605332	G	0.25	0.3571	A
3	BICF2P10837	52623524	0	0	0	G
3	TIGRP2P44466_rs8793696	52631521	A	0	0	G
3	BICF2P992370	52631763	A	0	0	G
3	BICF2P643845	52638422	A	0.5	0.6429	G
3	BICF2P984407	52648283	A	0.5	0.3571	G
3	BICF2S2335582	52661140	C	0	0	A
3	BICF2P560507	52673002	G	0	0	A
3	BICF2S23659404	52683025	G	0	0	A
3	BICF2S2326411	52695933	C	0.5	0.3571	A
3	BICF2P722675	52705136	C	0.5	0.3571	A
3	BICF2P1064847	52720884	A	0	0	G
3	BICF2P265831	52734099	A	0.5	0.6429	G
3	BICF2P912945	52739680	A	0.5	0.6429	G
3	BICF2P312995	52760551	G	0	0	A
3	BICF2P899090	52765315	A	0	0	G
3	BICF2S23137549	52795650	0	0	0	C
3	BICF2P742960	52804066	G	0.5	0.3571	A
3	BICF2S229432	52811539	C	0	0	G
3	BICF2P54257	52820643	A	0.5	0.3571	G
3	BICF2P1416443	52845712	G	0	0	A
3	BICF2S23020373	52864469	C	0	0	A
3	BICF2P663112	52868607	A	0.75	0.6429	C
3	BICF2P739380	52886174	T	0.5	0.3571	A
3	BICF2P771807	52894396	G	0	0	A
3	BICF2S22938607	52906696	A	0	0	C
3	BICF2P880059	52910478	A	0	0	T
3	BICF2P97590	52912216	A	0	0	G
3	BICF2P993641	52928063	A	0.25	0.2857	G
3	BICF2P475301	52944082	G	0.25	0.2857	A
3	BICF2P1135353	52947122	A	0	0	C
3	TIGRP2P44515_rs9191790	52961389	A	0.25	0.2857	G
3	TIGRP2P44529_rs8887331	52973030	A	0.25	0.2857	G
3	BICF2P1322570	52984514	G	0	0	A
3	BICF2P693545	52993270	A	0.5	0.6429	C
3	BICF2S23514203	53007306	G	0	0	A
3	BICF2P263938	53025697	G	1	1	A
3	BICF2S22955571	53037990	T	0	0	A
3	BICF2P52557	53039085	A	0	0	G
3	BICF2S23736202	53059698	G	0.5	0.6429	A
3	BICF2S23158270	53068455	G	0	0	A
3	BICF2S22944430	53091856	A	0	0.2857	G

3	BICF2S2319554	53096655	G	0.25	0.2857	A
3	BICF2P340993	53108429	G	0	0	A
3	BICF2P689890	53129004	A	0.25	0.2857	G
3	BICF2P1293197	53131074	C	0.75	0.7143	A
3	BICF2P273204	53151334	A	0	0	G
3	BICF2P6915	53159147	G	1	1	A
3	BICF2P1070866	53167043	A	0.25	0.2857	C
3	TIGRP2P44559_rs9212998	53181806	G	0	0	A
3	BICF2P893699	53193767	G	0.25	0.2857	A
3	BICF2S23518923	53203952	A	0	0	G
3	BICF2P68877	53213774	C	0	0	A
3	BICF2P1235551	53230138	C	0	0	A
3	BICF2P332059	53239314	G	0	0	A
3	BICF2P1408651	53260264	A	0	0	G
3	BICF2P1059412	53274357	G	0	0	A
3	BICF2P423203	53291074	G	0	0	A
3	TIGRP2P44616_rs9187212	53306554	0	0	0	G
3	BICF2P363011	53316722	A	0.5	0.3571	G
3	BICF2P958474	53326168	G	0	0	C
3	BICF2S23454295	53339867	C	0	0	A
3	TIGRP2P44648_rs8941449	53358362	A	0	0	G
3	G254f58S5	53378603	G	0	0	A
3	BICF2P1365820	53416869	0	0	0	A
3	BICF2S23212208	53427695	A	0	0	G
3	BICF2P1038569	53431294	G	0	0	A
3	BICF2P314623	53442796	C	0	0	A
3	BICF2P1324021	53458497	0	0	0	A
3	BICF2P884099	53465040	0	0	0	A
3	BICF2P512470	53483301	C	0	0	A
3	TIGRP2P44653_rs8998553	53506504	A	0	0	G
3	TIGRP2P44655_rs8933932	53516242	A	0	0	G
3	BICF2P872446	53528305	A	0	0	G
3	BICF2P674653	53538925	C	0	0	A
3	BICF2P1028986	53545467	A	0.25	0.2857	G
3	BICF2P1426828	53564068	A	0	0	T
3	TIGRP2P44692_rs8898988	53570326	0	0	0	G
3	BICF2S23431697	53581087	A	0	0	C
3	BICF2P223830	53591510	0	0	0	A
3	BICF2S23915468	53606030	A	0	0	G
3	BICF2P704841	53609120	G	1	1	A
3	BICF2P618708	53626857	C	0	0	A
3	TIGRP2P44721_rs9021873	53636717	0	0	0	A
3	BICF2P290854	53648456	0	0	0	G
3	BICF2S23239266	53665761	G	0.25	0.2857	A
3	TIGRP2P44734_rs8965412	53677126	A	0.25	0.2857	C
3	BICF2P633314	53686410	A	0	0	G
3	BICF2P809883	53710832	A	0.25	0.2857	C

3	BICF2P749436	53717819	A	0	0	C
3	BICF2P638865	53735777	0	0	0	A
3	BICF2P236945	53740437	0	0	0	A
3	BICF2P856127	53758292	A	0	0	G
3	BICF2P546627	53763219	G	0	0	A
3	BICF2S23915658	53782384	A	0.75	0.7143	C
3	BICF2P639571	53786283	G	1	1	A
3	BICF2P404880	53798499	G	0	0	A
3	BICF2P1330533	53806734	T	0	0	A
3	BICF2P1330530	53809232	A	0.75	0.7143	G
3	BICF2P451172	53824359	A	0	0	T
3	BICF2P773672	53840474	G	0	0	A
3	BICF2P173134	53844020	A	0	0	G
3	BICF2S23419852	53875193	A	0	0	G
3	BICF2S23513086	53882908	G	0.25	0.2857	A
3	BICF2S2305914	53889874	A	0	0	G
3	BICF2P1448707	53901580	A	0	0	T
3	BICF2S22910036	53912183	A	1	1	G
3	BICF2S23312245	53929785	A	1	1	C
3	BICF2P625943	53940622	A	0	0	G
3	BICF2S2362588	53951656	A	0.25	0.2857	C
3	BICF2S23631021	53963519	A	1	1	G
3	BICF2P1106056	53978961	A	0.75	0.7143	G
3	BICF2P249986	53983379	A	1	1	G
3	BICF2S23744551	53997714	G	0.25	0.2857	A
3	BICF2S23448000	54019637	A	0	0	G
3	BICF2S23447998	54019904	A	1	1	G
3	BICF2S23618401	54034161	G	0	0	A
3	BICF2P1276947	54047298	G	1	1	A
3	TIGRP2P44863_rs9127806	54051694	G	1	1	A
3	TIGRP2P44868_rs9085285	54063241	A	0.25	0.2857	G
3	BICF2S23746185	54065452	G	0	0	C
3	BICF2P1428463	54071460	A	0.25	0.2857	C
3	BICF2P798582	54073108	A	0	0	G
3	BICF2P463368	54086045	G	0	0	A
3	BICF2P74349	54101477	G	0	0	A
3	BICF2S23059076	54117714	A	0.75	0.7143	G
3	BICF2P602661	54125429	A	0.75	0.7143	G
3	BICF2P797821	54138989	G	0.25	0.2857	C
3	BICF2S23447407	54142311	G	0.75	0.7143	A
3	BICF2S23152168	54160905	G	0	0	A
3	BICF2P386012	54178180	G	0	0	A
3	BICF2P1185597	54192969	G	1	1	A
3	BICF2P236884	54202813	A	0.25	0.2857	G
3	BICF2S23550151	54203311	G	0	0	A
3	BICF2P966382	54221303	G	0	0	C
3	BICF2P1320211	54222577	G	0	0	A

3	BICF2P1098349	54238168	A	0	0	G
3	BICF2S23145338	54253010	0	0	0	G
3	BICF2P150141	54258373	A	0	0	C
3	BICF2P150142	54258488	G	0	0	A
3	BICF2P1330114	54263618	G	0	0	A
3	BICF2P490547	54274430	A	0	0	G
3	BICF2P354228	54287564	A	0.25	0.2857	C
3	BICF2S22947643	54300967	T	1	1	A
3	BICF2S22935897	54305243	A	0	0	G
3	BICF2P801933	54318404	C	0	0	A
3	BICF2S23121736	54328008	0	0	0	G
3	BICF2S2317646	54337965	G	0	0	A
3	TIGRP2P44949_rs9095011	54351354	A	0	0	C
3	BICF2P469289	54364353	A	1	1	G
3	BICF2P125449	54379452	0	0	0	C
3	BICF2S23145664	54393433	T	0	0	A
3	BICF2S23317753	54414543	A	0	0	G
3	TIGRP2P44951_rs8570923	54418694	0	0	0	C
3	BICF2P423212	54431043	G	0	0	A
3	BICF2P1053453	54455902	A	0	0	G
3	BICF2P888363	54477636	0	0	0	A
3	BICF2S23355550	54493689	A	0.25	0.2857	G
3	BICF2S22940311	54521549	A	0.25	0.2857	G
3	BICF2P1250963	54540089	0	0	0	A
3	BICF2P1374449	54562372	0	0	0	A
3	TIGRP2P44955_rs8657221	54574333	0	0	0	G
3	BICF2P361611	54589685	G	0.25	0.2857	A
3	BICF2P589675	54591630	G	0.25	0.2857	A
3	BICF2S22925601	54619135	A	0	0	G
3	BICF2S23153080	54635232	G	1	1	A
3	BICF2S2291106	54637127	A	1	1	G
3	BICF2P995123	54666903	T	0	0	A
3	BICF2P1066948	54681203	A	0	0	G
3	BICF2P1066951	54682780	C	0.75	0.7143	G
3	TIGRP2P44992_rs8579777	54694616	C	0	0	A
3	TIGRP2P45011_rs9208280	54712535	A	0.75	0.7143	G
3	BICF2S23711086	54718911	T	0	0	A
3	TIGRP2P45019_rs9104661	54729458	A	0.25	0.2857	G
3	BICF2P1143865	54731201	A	0.25	0.2857	G
3	BICF2S23356370	54767312	G	0.75	0.7143	A
3	BICF2P905650	54775434	G	1	1	A
3	BICF2P638856	54793396	0	0	0	A
3	BICF2P88321	54797908	A	0	0	C
3	BICF2P1370151	54826270	A	0.25	0.2857	G
3	BICF2P843390	54831916	A	0.25	0.2857	G
3	BICF2S2326663	54837339	G	1	1	A
3	BICF2P913573	54845641	A	0	0	G

3	BICF2P525801	54859347	A	0	0	G
3	BICF2P487224	54867980	G	0.75	0.7143	A
3	BICF2P1368727	54882411	G	0.75	0.7143	A
3	BICF2S23911371	54890331	A	0.25	0.2857	G
3	BICF2P378975	54910110	G	0	0	A
3	BICF2P804036	54923295	G	0	0	A
3	BICF2P126124	54927291	A	0	0	G
3	BICF2P776971	54941361	A	0	0	C
3	TIGRP2P46011_rs8639372	54954538	A	0	0	C
3	BICF2P1391272	54967906	G	0	0	A
3	BICF2P1391277	54971748	G	0	0	A
3	BICF2P1137753	54989650	A	0.25	0.2857	G
3	BICF2P1282690	54997381	A	0.25	0.2857	G
3	BICF2S2356168	55011878	A	0	0	G
3	BICF2S23643916	55018170	A	0	0	C
3	BICF2P311666	55033159	A	0.25	0.2857	G
3	BICF2P445387	55047093	C	0	0	G
3	BICF2P579292	55057390	0	0	0	A
3	BICF2P1367838	55071459	0	0	0	A
3	TIGRP2P46028_rs9195993	55073754	A	1	1	G
3	G255f49S50	55085017	G	0	0	A
3	BICF2P1330621	55114205	A	0	0	G
3	BICF2S23138463	55128931	A	0	0	G
3	BICF2S23442092	55136194	C	0	0	A
3	BICF2S23035503	55147296	C	0	0	A
3	BICF2P456629	55157929	G	0.75	0.7143	A
3	BICF2S23118209	55168117	A	0	0	G
3	TIGRP2P46039_rs8660428	55183399	A	0	0	G
3	BICF2P777556	55232129	T	0.75	0.7143	A
3	BICF2P356709	55242126	A	0	0	G
3	BICF2P564273	55250188	A	0.25	0.2857	C
3	BICF2S23619253	55260355	G	1	1	C
3	BICF2P1426885	55270728	A	0.25	0.2857	G
3	BICF2S23711940	55284784	0	0	0	A
3	BICF2S23148129	55287596	A	0	0	T
3	BICF2S2357894	55289371	A	0	0	G
3	BICF2P1159620	55292221	A	0.25	0.2857	G
3	BICF2P515330	55300786	A	0	0	G
3	BICF2P351574	55307553	0	0	0	G
3	BICF2S23613080	55319424	0	0	0	G
3	BICF2P387760	55335308	G	0	0	A
3	BICF2S22936695	55342144	A	0.25	0.2857	G
3	BICF2S23549343	55360264	G	0	0	A
3	BICF2P638432	55366221	A	0.25	0.2857	G
3	BICF2P679587	55375823	G	0.25	0.2857	A
3	BICF2S23047550	55385795	A	0	0	G
3	TIGRP2P46116_rs8756252	55403128	A	0.25	0.2857	G

3	BICF2P1353429	55416892	G	0	0	A
3	BICF2S23615375	55423607	C	0.25	0.2857	A
3	TIGRP2P46133_rs8968403	55431543	A	0	0	G
3	BICF2P1350820	55457601	A	0	0	G
3	BICF2P1153163	55467376	G	0	0	A
3	TIGRP2P46150_rs8784424	55484738	G	0	0	A
3	BICF2S23763081	55490426	0	0	0	A
3	BICF2P463061	55510437	G	0	0	A
3	BICF2S233314	55518288	C	0	0	G
3	BICF2P474501	55531762	G	1	1	A
3	TIGRP2P46163_rs9021452	55554924	A	1	1	G
3	BICF2S23752024	55561389	A	1	1	G
3	TIGRP2P46169_rs8906864	55576425	A	1	1	G
3	BICF2P403503	55578124	G	1	1	A
3	BICF2P288112	55586976	A	1	1	G
3	BICF2P1300512	55597276	A	0	0	G
3	TIGRP2P46185_rs8721322	55606271	A	1	1	G
3	TIGRP2P46192_rs8646740	55615364	A	0	0	G
3	BICF2P716666	55619056	A	1	1	G
3	BICF2P871170	55623487	A	1	1	G
3	BICF2P702506	55635154	A	0	0	G
3	BICF2P1387550	55639110	A	0	0	G
3	BICF2P1048394	55643579	A	0	0	G
3	BICF2P1031402	55653365	G	1	1	A
3	TIGRP2P46216_rs8578059	55660225	A	0	0	G
3	BICF2P258826	55700004	0	0	0	G
3	BICF2P1256187	55727655	0	0	0	A
3	BICF2P522834	55736109	G	1	1	A
3	BICF2P1451351	55755973	G	1	1	A
3	BICF2S23035325	55772900	A	1	1	G
3	BICF2P354567	55787413	G	1	1	A
3	TIGRP2P46258_rs8984567	55820441	A	1	1	G
3	BICF2P622783	55821310	A	1	1	G
3	TIGRP2P46272_rs8866951	55850602	A	1	1	C
3	BICF2P162832	55868631	G	1	1	A
3	BICF2P1421165	55883084	G	0.25	0	A
3	BICF2P302065	55902728	0	0	0	G
3	BICF2G630342021	55917898	0	0	0	G
3	BICF2S23160800	55929551	A	0	0	C
3	BICF2S23910594	55945329	0	0	0	A
3	BICF2P688425	55952453	C	0.75	0.7143	A
3	TIGRP2P46284_rs8838138	55981354	A	0	0	T
3	BICF2P939679	55985032	G	0.25	0.2857	A
3	BICF2P39442	55993521	A	0.25	0.2857	G
3	BICF2S2332226	56005866	A	0.25	0.2857	G
3	TIGRP2P46301_rs8960321	56022284	A	0	0	G
3	BICF2P463721	56033699	A	0	0	G

3	BICF2P322996	56043692	A	0	0	G
3	TIGRP2P46309_rs8861647	56060376	G	0.25	0.2857	C
3	TIGRP2P46311_rs8786028	56065303	G	0.25	0.2857	A
3	TIGRP2P46315_rs9029249	56083779	A	0.25	0.2857	C
3	TIGRP2P46324_rs8685794	56095589	C	0.25	0.2857	A
3	BICF2P1126097	56098529	G	0.25	0.2857	A
3	BICF2P195763	56114909	A	0.25	0.2857	C
3	TIGRP2P46332_rs8567961	56134051	A	0.25	0.2857	G
3	BICF2S23061083	56154248	G	0	0	A
3	BICF2P948021	56156029	G	0.75	0.7143	A
3	BICF2P948022	56156335	A	0.75	0.7143	G
3	BICF2P1043804	56161311	G	0.75	0.7143	A
3	BICF2P1371206	56170412	A	0	0	G
3	TIGRP2P46350_rs9087864	56188542	A	0	0	G
3	BICF2P43046	56192526	C	0	0	A
3	BICF2P861945	56209231	0	0	0	G
3	BICF2S2292455	56222665	A	0.25	0.2857	C
3	BICF2S23420033	56233790	A	0	0	G
3	BICF2S23152312	56245215	G	0.75	0.7143	A
3	BICF2S23122083	56248415	G	0.75	0.7143	A
3	BICF2S2398108	56265218	C	0.75	0.7143	A
3	TIGRP2P46358_rs9083132	56270102	0	0	0	A
3	BICF2S23111383	56303651	C	0.75	0.7143	A
3	BICF2S23139400	56392703	G	0.75	0.7143	A
3	BICF2P1459020	56412039	0	0	0	C
3	BICF2G630342023	56423596	A	0.5	0.5	G
3	BICF2S23139601	56446713	G	0.75	0.7143	C
3	BICF2S23135235	56455534	G	0.5	0.5	A
3	BICF2P1199134	56468276	A	1	1	G
3	BICF2P1107829	56484982	0	0	0	A
3	BICF2P1292085	56490759	0	0	0	G
3	BICF2P1317713	56504090	G	0	0	C
3	BICF2P1143742	56522827	A	1	1	G
3	BICF2P731226	56544884	A	0.25	0.2857	G
3	BICF2P1296465	56556413	A	0	0	C
3	BICF2P1296466	56556653	A	0.25	0.2857	G
3	TIGRP2P46387_rs8705305	56561903	0	0	0	A
3	BICF2P157564	56573250	A	0.25	0.2857	G
3	BICF2P1285815	56587122	0	0	0	G
3	BICF2P1028290	56602528	A	0.25	0.2857	G
3	BICF2P1028292	56603378	G	0.25	0.2857	A
3	BICF2P812355	56623830	A	0	0	G
3	BICF2P527992	56639179	A	0	0	G
3	BICF2S23340184	56649556	G	0.25	0.2857	A
3	BICF2P752720	56663579	G	1	1	A
3	BICF2P1356640	56682293	A	0.25	0.2857	G
3	BICF2P906599	56691492	A	0.25	0.2857	G

3	BICF2S23356321	56697128	C	0.25	0.2857	A
3	BICF2S23761113	56707855	A	0.25	0.2857	C
3	BICF2P1378482	56730586	A	1	0.8	G
3	BICF2P631541	56733617	A	0.75	0.7143	T
3	BICF2P264970	56741149	A	0.75	0.7143	G
3	BICF2P539248	56758127	C	0.75	0.7143	A
3	BICF2P161096	56764379	A	0.75	0.7143	G
3	BICF2S23244869	56777831	A	0.25	0.2857	G
3	BICF2S23056688	56804659	A	0.25	0.2857	G
3	BICF2P219474	56813514	A	0.5	0.3571	C
3	BICF2S23013305	56840375	A	0.25	0.3571	G
3	BICF2P596472	56851954	G	0.5	0.3571	A
3	BICF2P345400	56876770	A	0.75	0.7143	G
3	BICF2P978508	56884190	C	0.75	0.7143	A
3	BICF2P1153515	56896699	A	0.75	0.7143	G
3	BICF2P310206	56912174	G	0.75	0.7143	A
3	BICF2P910151	56914137	G	0.75	0.7143	A
3	BICF2S22951931	56925222	A	0.75	0.7143	C
3	BICF2P193033	56936966	A	0.25	0.2857	G
3	TIGRP2P46395_rs8740992	56954200	C	0.5	0.5	A
3	BICF2P1364239	56965210	A	0	0	G
3	BICF2P590783	56972702	A	0.25	0.2857	C
3	BICF2P1337240	56985805	G	0.25	0.2857	A
3	BICF2P317914	56995051	G	0.25	0.2857	A
3	BICF2P1323306	57008087	A	0.25	0.3571	G
3	BICF2P779616	57021552	G	0	0	A
3	BICF2P154842	57034238	0	0	0	G
3	BICF2P935449	57047258	A	0.5	0.3571	G
3	BICF2P985389	57060019	A	0.5	0.3571	G
3	BICF2P1359456	57072376	G	0.25	0.2857	A
3	BICF2P576393	57080840	A	0.75	0.7143	G
3	BICF2P1317716	57087733	G	0	0	A
3	BICF2P1058409	57101425	A	1	0.75	G
3	BICF2P225679	57110010	G	0	0	A
3	BICF2P206965	57125969	0	0	0	A
3	TIGRP2P46442_rs8769072	57141407	A	0.25	0.2857	G
3	TIGRP2P46443_rs8697444	57149997	0	0	0	C
3	BICF2P345423	57161195	G	0.75	0.7143	C
3	BICF2P543344	57178980	A	0.25	0.2857	G
3	BICF2P1216525	57180109	A	0.75	0.7143	G
3	BICF2P1381745	57200871	A	0.25	0.2857	G
3	TIGRP2P46456_rs8554843	57203362	A	0.25	0.2857	G
3	BICF2S23039528	57226928	A	0.75	0.7143	G
3	BICF2P1291007	57241368	C	0.75	0.7143	A
3	BICF2S23510470	57263998	A	0	0	G
3	BICF2P562243	57276207	G	0.25	0.2857	A
3	BICF2P1342834	57289365	T	0.25	0.2857	A

3	BICF2P805006	57300053	G	0.25	0.2857	A
3	BICF2P1021727	57308756	A	0.25	0.2857	G
3	TIGRP2P46459_rs8776472	57317093	0	0	0	C
3	BICF2P361688	57344170	C	0.25	0.2857	A
3	BICF2P777989	57355000	A	0.25	0.2857	C
3	BICF2P1417321	57364905	0	0	0	A
3	BICF2P35707	57382494	A	0.25	0.3571	G
3	BICF2S22916819	57386010	A	0.25	0.3571	G
3	BICF2S22951824	57396505	A	0	0	C
3	BICF2P662576	57409524	C	0.75	0.6429	A
3	BICF2P1340094	57427279	G	0.75	0.6429	A
3	TIGRP2P46461_rs8541124	57440651	A	0.5	0.3571	G
3	BICF2P713565	57449719	G	0.25	0.2857	C
3	BICF2P326886	57456374	A	0.25	0.2857	G
3	TIGRP2P46481_rs8815653	57466015	C	0.25	0.2857	A
3	BICF2S23740841	57479877	C	0	0.2	A
3	BICF2P64002	57495652	G	0.75	0.6429	A
3	TIGRP2P46486_rs8889937	57500181	G	0.25	0.2857	A
3	BICF2P861110	57505722	A	0	0.2	G
3	BICF2P1155019	57522939	A	0	0	G
3	BICF2P975550	57525613	A	0	0	G
3	BICF2P723850	57538269	A	0	0	G
3	BICF2P560104	57542327	G	0	0	A
3	BICF2P560110	57543179	C	0	0	G
3	BICF2P1056437	57556755	G	0	0	A
3	BICF2P259449	57565870	A	0	0	G
3	BICF2P1018628	57574407	T	0.5	0.3571	A
3	BICF2P72059	57580692	A	0.5	0.3571	G
3	BICF2P1014029	57592947	A	0	0	G
3	TIGRP2P46511_rs8488293	57619212	0	0	0	G
3	BICF2S22924381	57635546	G	0.5	0.3571	A
3	BICF2P1024513	57646931	C	0	0	A
3	BICF2P708229	57660110	G	0.75	0.7143	A
3	BICF2P958059	57663178	G	0.75	0.7143	A
3	BICF2P233194	57679896	0	0	0	A
3	TIGRP2P46520_rs9200949	57694118	A	0.5	0.3571	G
3	TIGRP2P46522_rs9184580	57698908	A	0.5	0.3571	C
3	TIGRP2P46531_rs8655673	57714433	G	0	0	A
3	BICF2P1326842	57724382	A	0	0	G
3	BICF2P1417439	57732551	G	0	0	A
3	BICF2S23051663	57762949	A	0	0	G
3	BICF2P163789	57842562	0	0	0	A
3	BICF2P1046163	57873830	G	0	0	A
3	BICF2P797490	57889602	A	0	0	G
3	BICF2P446253	57894658	G	0	0	A
3	BICF2S23615791	57901338	A	0.5	0.6429	G
3	BICF2S23611102	57905632	A	0.5	0.3571	C

3	BICF2P1301559	57916874	A	0.25	0.3571	G
3	BICF2S23018890	57936120	G	0.25	0.2857	A
3	BICF2P1226516	57959197	G	0.25	0.3571	C
3	BICF2P17124	57962088	A	0.25	0.3571	G
3	TIGRP2P46568_rs9002672	57973552	A	0.75	0.6429	G
3	BICF2P1028009	57983293	A	0.25	0.25	G
3	BICF2P121699	57995232	G	0.5	0.3571	A
3	BICF2P278666	58012067	A	0.25	0.2857	G
3	BICF2P278665	58012250	G	0.25	0.2857	A
3	BICF2P277878	58020190	A	0	0	G
3	TIGRP2P46582_rs8739278	58036898	G	0.25	0.2857	A
3	BICF2P1087702	58037821	G	0.25	0.2857	A
3	BICF2P938427	58042150	G	0	0	A
3	BICF2P30081	58056951	T	0	0	A
3	BICF2P30088	58059094	T	0	0	A
3	BICF2P908905	58065298	A	0	0	G
3	BICF2P315515	58085421	A	0.25	0.2857	G
3	BICF2S23525130	58085457	A	0.25	0.2857	G
3	BICF2P459166	58085809	A	0.25	0.2857	G
3	TIGRP2P46607_rs9218681	58089420	A	0.75	0.7143	G
3	BICF2P940514	58100165	G	0.25	0.2857	A
3	BICF2P240528	58119584	A	0.5	0.3571	G
3	BICF2P648237	58132433	G	0.5	0.6429	A
3	TIGRP2P46634_rs9067170	58149643	A	0.5	0.3571	C
3	TIGRP2P46636_rs9180353	58164266	A	0.25	0.2857	C
3	BICF2P1291281	58171553	A	0.5	0.6429	G
3	BICF2S23010495	58184886	A	0.5	0.3571	G
3	TIGRP2P46645_rs8576557	58196157	A	0	0	G
3	BICF2S2365882	58210466	A	0	0	G
3	BICF2P265483	58223247	A	0.5	0.3571	G
3	BICF2S23328993	58258912	A	1	1	G
3	TIGRP2P46656_rs9079808	58270801	0	0	0	G
3	BICF2S23216141	58293204	A	0.25	0.2857	G
3	TIGRP2P46658_rs8716954	58307845	A	0.75	0.7143	G
3	BICF2P1209036	58316859	0	0	0	G
3	BICF2S23056517	58338037	C	0.75	0.6429	G
3	BICF2P336175	58340653	0	0	0	C
3	BICF2P787395	58383063	G	0.5	0.3571	A
3	BICF2P666894	58395936	G	0	0	A
3	TIGRP2P46681_rs9107480	58404885	G	0	0	A
3	BICF2P352274	58415007	0	0	0	C
3	BICF2P623061	58432433	G	0.25	0.3571	A
3	TIGRP2P46685_rs9017831	58447539	0	0	0	G
3	BICF2S2412808	58487083	C	0.25	0.3571	A
3	BICF2P701755	58489468	A	0	0	G
3	BICF2P450864	58506050	C	0	0	A
3	BICF2S2378091	58513008	A	0	0	G

3	BICF2P527246	58515391	G	0	0	A
3	BICF2P1423477	58533073	G	1	1	A
3	BICF2P1423478	58533583	A	1	1	G
3	TIGRP2P46709_rs8672244	58541916	0	0	0	C
3	BICF2P600703	58553691	A	0	0	G
3	TIGRP2P46718_rs8761151	58571772	0	0	0	A
3	TIGRP2P46724_rs8570087	58586918	A	0	0	C
3	BICF2P819372	58601172	C	0	0	A
3	BICF2P129927	58610986	G	0	0	A
3	BICF2P62222	58626431	A	0	0	G
3	TIGRP2P46734_rs8928293	58632821	0	0	0	A
3	BICF2S23027784	58641206	A	0	0	G
3	TIGRP2P46743_rs8647384	58653198	C	0	0	A
3	BICF2P1403991	58668741	A	0	0	C
3	BICF2S2323938	58687388	A	0	0	G
3	TIGRP2P46766_rs8736150	58697770	A	0	0	G
3	TIGRP2P46773_rs9006821	58708428	G	1	1	A
3	BICF2P981669	58721335	A	0	0	G
3	TIGRP2P46789_rs8952227	58731798	G	0	0	A
3	TIGRP2P46798_rs9165444	58744477	C	1	1	A
3	TIGRP2P46803_rs8649370	58762413	G	0.75	0.6429	C
3	TIGRP2P46811_rs9062999	58770543	A	0	0	G
3	BICF2P986395	58778231	0	0	0	A
3	BICF2S23735412	58790438	A	0.75	0.6429	G
3	BICF2P1133363	58810862	A	0	0	G
3	TIGRP2P46816_rs8721640	58821391	0	0	0	G
3	BICF2P436626	58823466	0	0	0	A
3	TIGRP2P46827_rs9140943	58850826	G	0.25	0.3571	A
3	TIGRP2P46844_rs9108296	58858407	G	0	0	A
3	BICF2P1228370	58889827	G	1	1	A
3	BICF2P356938	58900022	A	0	0	G
3	TIGRP2P46860_rs9117118	58909774	A	1	0.75	G
3	BICF2P773829	58932471	0	0	0	A
3	BICF2S22937646	58941454	0	0	0	G
3	BICF2S22921233	58950510	A	0	0	G
3	BICF2P189710	58966724	C	0.25	0.3571	G
3	BICF2S23451528	58979408	G	0.75	0.6429	A
3	BICF2P556667	58989085	A	0.25	0.3571	G
3	BICF2P777999	59003457	G	0.25	0.3571	A
3	BICF2S231757	59006732	0	0	0	G
3	BICF2P634515	59023611	A	0	0	C
3	BICF2P601900	59029115	G	0.75	0.6429	A
3	TIGRP2P46884_rs8783818	59050415	C	0	0	A
3	BICF2P196021	59056457	A	0.25	0.3571	C
3	BICF2P461715	59059577	A	0.75	0.6429	G
3	BICF2S23443302	59080239	A	0.75	0.6429	G
3	TIGRP2P46892_rs8970576	59087097	G	1	1	A

3	BICF2P651494	59088051	G	1	1	A
3	TIGRP2P46905_rs8400984	59103745	G	0	0	A
3	TIGRP2P46910_rs9000203	59112095	G	1	1	A
3	BICF2P1408506	59125981	0	0	0	C
3	TIGRP2P46917_rs8997867	59143441	A	0	0	C
3	BICF2P765435	59147759	C	0	0	A
3	BICF2S23514192	59157082	A	0	0	G
3	BICF2P785723	59167431	C	0	0	G
3	TIGRP2P46923_rs8759098	59178518	A	1	1	C
3	BICF2P940901	59181893	A	0	0	G
3	BICF2P193899	59194257	0	NA	NA	G
3	TIGRP2P46929_rs8954995	59208474	0	0	0	G
3	BICF2P125760	59217199	A	0	0	G
3	BICF2P733852	59243330	A	1	1	G
3	TIGRP2P46946_rs8985656	59250889	G	1	1	A
3	BICF2P787031	59260229	G	1	1	A
3	BICF2P684926	59275200	G	0	0	A
3	BICF2P821692	59286924	A	0	0	G
3	BICF2P850771	59309684	G	1	1	A
3	TIGRP2P46983_rs8491385	59314616	A	0	0	C
3	TIGRP2P46995_rs8491372	59317142	A	0	0	G
3	BICF2S23742412	59336756	A	1	1	T
3	BICF2S22941239	59349290	A	0	0	G
3	BICF2S22941992	59353168	A	0	0	G
3	BICF2P421085	59373975	0	0	0	G
3	TIGRP2P47006_rs9026135	59378598	A	0	0	G
3	BICF2P769122	59390065	A	1	1	C
3	TIGRP2P47014_rs9092365	59399260	G	0	0	C
3	BICF2P162635	59409601	A	0	0	G
3	TIGRP2P47035_rs8530261	59428338	A	0	0	G
3	BICF2P1126601	59435837	A	0	0	G
3	BICF2S23150351	59444529	A	0.25	0.25	G
3	BICF2P1369136	59455414	G	0	0	A
3	BICF2P1394756	59472814	A	0	0	G
3	BICF2S23126274	59481536	A	0.25	0.2857	C
3	BICF2S23139063	59490371	G	0.25	0.2857	A
3	TIGRP2P47078_rs9243047	59492660	C	0	0	A
3	BICF2P638159	59502464	A	0	0	G
3	BICF2P514286	59520794	A	0	0	G
3	TIGRP2P47114_rs8854693	59530227	A	0	0	G
3	BICF2P911926	59535500	G	0	0	A
3	BICF2S23618562	59537372	A	0	0	G
3	BICF2P560719	59541140	A	0	0	C
3	BICF2P335633	59541960	A	0	0	G
3	BICF2P399512	59547401	A	0	0	C
3	BICF2P547573	59559495	G	0	0	A
3	BICF2P1189190	59570345	A	0	0	G

3	TIGRP2P47159_rs9182961	59582169	A	0	0	C
3	BICF2P25895	59594734	A	0.75	0.7143	G
3	TIGRP2P47184_rs8650949	59619158	G	0.75	0.7143	A
3	BICF2P614740	59631071	A	0	0	G
3	BICF2P934354	59644944	A	0	0	G
3	BICF2P576162	59651227	G	0.75	0.7143	A
3	BICF2P662354	59672947	C	0.25	0.2857	A
3	TIGRP2P47208_rs9018581	59680429	A	0.75	0.7143	G
3	TIGRP2P47213_rs8835960	59686810	0	0	0	A
3	TIGRP2P47215_rs9082321	59699010	A	0	0	G
3	BICF2S23435950	59708155	A	0	0	C
3	BICF2S22928638	59741389	A	0	0	C
3	BICF2S23746058	59762534	G	0.75	0.7143	A
3	BICF2P1431115	59783388	A	0	0	G
3	BICF2P971579	59797380	0	0	0	G
3	BICF2P1272708	59803931	A	0.25	0.25	G
3	BICF2S23759490	59823094	A	0.25	0.2857	C
3	BICF2P496493	59844575	G	0	0	A
3	BICF2S23543323	59846839	A	0	0	G
3	TIGRP2P47247_rs9018900	59857899	C	0	0.2857	A
3	BICF2P1339336	59876105	A	0	0	G
3	BICF2P348925	59894803	A	0	0	G
3	BICF2S23617348	59903982	A	0	0	G
3	BICF2P933112	59942762	A	0	0	G
3	BICF2P126084	59951421	G	0.25	0.2857	A
3	BICF2P840702	59964791	A	0.25	0.2857	G
3	BICF2P761632	59981619	C	0.25	0.2857	A
3	BICF2S24319510	59985773	G	0.25	0.2857	A
3	BICF2P1390607	59988696	A	0.25	0.2857	G
3	BICF2P498967	60023127	A	1	1	G
3	BICF2S2394890	60037359	G	0	0	A
3	BICF2P816673	60042969	0	0	0	C
3	BICF2S23257459	60060890	A	0.5	0.6429	C
3	BICF2P136222	60070811	G	0.25	0.3571	A
3	BICF2S2363134	60083608	A	0.25	0.2857	G
3	BICF2S23146792	60089029	A	0.75	0.6667	G
3	TIGRP2P47272_rs9158027	60103103	G	0.25	0.2857	A
3	BICF2P1200174	60118737	A	0.75	0.6429	G
3	BICF2S23318290	60125716	C	0.75	0.6429	A
3	BICF2S23235862	60139595	A	0	0	G
3	BICF2P493969	60150677	A	0	0	G
3	BICF2P1132727	60156223	A	NA	0	T
3	BICF2P949897	60167287	A	0.75	0.7143	G
3	BICF2S23025102	60171151	A	0.75	0.7143	G
3	BICF2S23710268	60183356	A	0.75	0.7143	G
3	TIGRP2P47329_rs9158777	60195187	A	0	0.3	G
3	TIGRP2P47339_rs8853618	60208301	A	0	0	G

3	BICF2P1071075	60214565	A	1	1	G
3	TIGRP2P47346_rs8741608	60233742	0	0	0	A
3	BICF2S23118161	60241529	A	0	0	G
3	BICF2P750212	60257942	G	0	0	A
3	BICF2P535866	60260695	G	0	0	A
3	BICF2P1497831	60275491	A	0	0	G
3	BICF2P584984	60293870	A	0	0	G
3	BICF2P391848	60298984	A	0.25	0.2857	G
3	BICF2P967467	60325835	G	0	0	A
3	BICF2G630342052	60345668	G	0.25	0.2857	A
3	BICF2G630342090	60370566	A	0.75	0.7143	C
3	TIGRP2P47392_rs8934440	60373918	G	0.75	0.75	A
3	BICF2P771848	60384820	A	0	0	G
3	BICF2G630342165	60398515	C	0	0	G
3	BICF2G630342183	60420517	A	0.5	0.6429	G
3	BICF2G630342188	60421975	A	0	0	G
3	BICF2G630342219	60440275	C	0	0	G
3	BICF2S23530561	60454547	G	0	0	A
3	BICF2G630342233	60456877	A	0	0	G
3	BICF2P569275	60471309	A	0	0	G
3	BICF2G630342252	60485857	G	0.5	0.3571	A
3	BICF2S23730892	60491423	G	0.5	0.3571	A
3	BICF2S23044035	60502892	A	0	0	G
3	BICF2G630342289	60515050	G	0	0	A
3	BICF2G630342295	60523425	A	0.5	0.3333	C
3	BICF2G630342302	60529664	A	0	0	G
3	BICF2G630342305	60546469	G	0	0	C
3	BICF2G630342306	60548700	C	0.5	0.3571	A
3	BICF2S23014069	60576604	G	0.5	0.6429	A
3	BICF2G630342317	60585949	A	0.25	0.3333	C
3	BICF2G630342336	60604161	G	0.25	0.2857	A
3	BICF2S23220772	60605108	A	0.25	0.3571	G
3	BICF2G630342348	60622432	A	0	0	G
3	BICF2G630342351	60631260	A	0	0	G
3	BICF2G630342358	60640066	A	0	0	G
3	BICF2G630342412	60667409	0	0	0	A
3	BICF2S2296229	60676983	G	0.75	0.6429	A
3	BICF2G630342454	60690584	A	0	0	G
3	BICF2G630342477	60703323	A	0.25	0.3571	G
3	BICF2P1181576	60713511	A	0.25	0.3571	G
3	TIGRP2P47472_rs8732868	60730652	A	0.75	0.6429	G
3	BICF2S2323000	60737421	G	0.25	0.3571	A
3	BICF2G630342485	60753407	G	0.5	0.6429	A
3	BICF2G630342493	60756920	G	0.5	0.6429	A
3	BICF2G630342532	60770724	A	0	0	G
3	TIGRP2P47485_rs9021276	60777791	A	0.25	0.3571	G
3	BICF2P902281	60793181	T	0.25	0.2857	A

3	BICF2S23638610	60820280	A	0.5	0.3571	C
3	BICF2S23640598	60825061	C	0.5	0.3571	G
3	BICF2S23424250	60835993	0	0	0	G
3	TIGRP2P47572_rs8743751	60855498	0	0	0	G
3	BICF2P692658	60860668	A	0	0	G
3	TIGRP2P47591_rs8580523	60870855	A	0.75	0.7143	G
3	BICF2P522022	60888002	A	0	0	C
3	BICF2G630342581	60907813	A	0.25	0.2857	G
3	BICF2S2356938	60918350	A	0	0	G
3	BICF2P980477	60931147	A	0	0	G
3	BICF2P994346	60938941	A	0.75	0.6429	G
3	BICF2G630342603	60959181	A	0.75	0.7143	G
3	BICF2G630342623	60978779	G	0	0	C
3	BICF2P465702	60993461	G	0.25	0.2857	A
3	BICF2P635067	61006592	A	0.25	0.2857	G
3	BICF2P805788	61008633	G	0.25	0.2857	A
3	BICF2G630342638	61019969	C	0.25	0.3571	A
3	BICF2S23622583	61035357	A	0	0	G
3	TIGRP2P47676_rs9005554	61043109	C	0.25	0.2857	A
3	BICF2P307990	61056570	A	0.5	0.3333	G
3	BICF2P1043648	61069111	A	0.5	0.3571	G
3	BICF2P1350378	61081502	A	0.5	0.3571	G
3	BICF2P1184966	61094841	A	0.5	0.3571	G
3	BICF2G630342723	61098781	G	0.75	0.7143	A
3	BICF2G630342725	61100529	G	0.75	0.7143	A
3	BICF2G630342748	61114920	A	0.25	0.3333	G
3	BICF2P563518	61129840	A	0.25	0.2857	C
3	BICF2G630342760	61144242	A	0.25	0.3571	G
3	BICF2G630342761	61145506	A	0.25	0.2857	G
3	BICF2P314519	61157377	G	0.5	0.3571	A
3	TIGRP2P47728_rs8789067	61169833	G	0.25	0.3571	A
3	TIGRP2P47734_rs8806656	61189901	G	0.5	0.6429	A
3	TIGRP2P47735_rs8576948	61191986	G	0.5	0.6429	A
3	BICF2S23433128	61210371	A	0	0	G
3	BICF2P92856	61216493	0	0	0	C
3	BICF2P998351	61244168	G	0.75	0.7143	A
3	BICF2S2347901	61244760	G	0.25	0.2857	A
3	BICF2S23644773	61251198	A	0	0	C
3	TIGRP2P47760_rs8836179	61270159	A	0	0	G
3	TIGRP2P47768_rs8503847	61280513	A	0	0	G
3	TIGRP2P47772_rs9100052	61291200	0	0	0	A
3	BICF2G630342799	61291248	A	0	0	G
3	TIGRP2P47781_rs8936167	61309396	C	0	0	A
3	BICF2S23233804	61335961	A	0	0	G
3	BICF2S23614175	61342394	C	0	0	A
3	BICF2P1299301	61364783	0	0	0	G
3	TIGRP2P47807_rs8917856	61376409	G	0.75	0.7143	A

3	BICF2P265077	61387207	G	0.75	0.7143	A
3	BICF2G630342818	61407825	G	0.75	0.7143	A
3	BICF2G630342831	61411604	G	0	0	A
3	BICF2G630342862	61442748	G	0	0	A
3	BICF2G630342901	61465893	A	0	0	C
3	BICF2G630342913	61469967	G	0.5	0.3	A
3	BICF2S23129486	61495568	0	0	0	G
3	BICF2G630342935	61513470	G	0.5	0.3571	A
3	BICF2G630342937	61516442	A	0.5	0.3571	G
3	BICF2G630342954	61525785	G	0.5	0.3571	A
3	TIGRP2P47893_rs8483655	61540437	0	0	0	G
3	BICF2G630343024	61554384	A	0	0	G
3	BICF2G630343035	61560534	G	0.5	0.4	A
3	BICF2G630343046	61582117	A	0	0	G
3	BICF2G630343050	61586653	A	0	0	G
3	BICF2G630343081	61602001	C	0.5	0.3571	G
3	BICF2P994384	61622980	C	0	0	A
3	BICF2G630343094	61643221	A	0	0	G
3	BICF2G630343100	61656590	A	0	0	G
3	BICF2G630343111	61669551	A	0	0	G
3	TIGRP2P47930_rs8546918	61692080	A	0.5	0.3571	G
3	BICF2G630343137	61698622	A	0	0	G
3	BICF2G630343144	61714946	A	0	0	G
3	BICF2P956917	61722723	0	0	0	G
3	BICF2P795021	61736406	A	0	0	G
3	BICF2G630343157	61746899	G	0	0	A
3	BICF2G630343177	61764408	A	0	0	G
3	BICF2G630343195	61776444	C	0	0	G
3	BICF2G630343199	61778381	A	0	0	G
3	BICF2G630343201	61782194	A	0	0	G
3	BICF2G630343209	61796167	A	0	0	G
3	BICF2G630343218	61811149	G	0	0	A
3	BICF2G630343227	61821320	T	0.25	0.3571	A
3	BICF2G630343236	61822884	A	0	0	T
3	BICF2G630343238	61823388	G	0	0	A
3	BICF2P828618	61827150	A	0	0	G
3	BICF2S22947559	61835833	C	0.25	0.3571	A
3	BICF2G630343268	61850471	G	0	0	A
3	BICF2G630343282	61860018	A	0.25	0.3571	G
3	BICF2G630343289	61872666	G	0	0	A
3	BICF2G630343296	61890193	A	0	0	G
3	BICF2G630343300	61897429	A	0	0	G
3	BICF2G630343306	61911022	A	0	0	G
3	BICF2S2294724	61919106	A	1	1	G
3	BICF2G630343315	61934362	A	0.75	0.6429	G
3	TIGRP2P47976_rs9096075	61940175	G	0.25	0.3571	A
3	BICF2P1408957	61950120	A	0.25	0.3571	C

3	BICF2G630343318	61950224	A	0.5	0.3571	G
3	BICF2G630343320	61953173	G	0	0	A
3	BICF2G630343347	61963797	G	0.5	0.3571	A
3	BICF2G630343376	61974633	A	0	0	G
3	BICF2G630343398	61993311	A	0	0	G
3	BICF2G630343405	62004113	A	0.5	0.3571	G
3	BICF2G630343442	62019762	A	0.5	0.3571	C
3	BICF2P257151	62039179	G	0	0	A
3	BICF2G630343507	62044082	G	0	0	A
3	BICF2G630343520	62055114	G	0.25	0.3571	A
3	BICF2S23535584	62073789	G	0.25	0.3571	A
3	BICF2P675684	62096654	A	0.5	0.6429	G
3	BICF2P629597	62100238	G	0.5	0.6429	A
3	BICF2P603949	62113880	A	0.5	0.6429	C
3	BICF2P753151	62123479	A	0.5	0.3571	G
3	BICF2G630343527	62136587	A	0.25	0.3571	T
3	BICF2S23435649	62155441	A	0	0	G
3	BICF2S22951698	62163778	C	0.25	0.2857	A
3	BICF2G630343593	62174589	A	0.25	0.2857	C
3	BICF2G630343615	62190638	G	0.25	0.2857	A
3	BICF2G630343620	62199806	A	0.5	0.3571	G
3	BICF2G630343629	62211510	G	0.5	0.3571	A
3	BICF2G630343631	62215007	G	0.5	0.3571	A
3	BICF2G630343656	62231357	A	0.5	0.6429	T
3	BICF2G630343663	62242138	G	1	1	C
3	BICF2G630343682	62256541	A	0	0	G
3	BICF2G630343705	62261524	A	1	1	G
3	BICF2G630343752	62273572	C	0.5	0.3571	A
3	BICF2S23411121	62288923	A	0.75	0.6429	G
3	BICF2P1379972	62296064	0	0	0	A
3	BICF2P664615	62314635	A	0	0	G
3	BICF2P772349	62323843	A	0	0	G
3	BICF2S23539139	62330170	G	0.25	0.3571	A
3	BICF2P756351	62344818	A	0.5	0.3571	G
3	BICF2G630343787	62347679	A	0.75	0.6429	G
3	BICF2S2344954	62355008	A	0.5	0.3571	G
3	BICF2P778641	62365653	G	0.5	0.3571	A
3	BICF2G630343819	62371768	A	0.25	0.2857	G
3	BICF2G630343824	62380582	C	0	0	A
3	BICF2G630343860	62391789	A	0	0	G
3	BICF2G630343874	62399676	A	0.25	0.2857	T
3	BICF2G630343901	62417619	A	0.25	0.3571	G
3	BICF2S23019370	62422987	G	0.5	0.6429	A
3	BICF2S2297194	62441438	G	0.25	0.2857	A
3	BICF2P572151	62449519	A	0.25	0.2857	G
3	BICF2P331789	62457327	G	1	1	A
3	BICF2P275180	62472245	A	1	1	G

3	BICF2P33688	62481096	A	0.25	0.2857	G
3	BICF2P797226	62492861	A	0.25	0.2857	G
3	TIGRP2P48207_rs8761162	62507510	A	0.75	0.7143	G
3	BICF2P690343	62516337	A	0	0	G
3	BICF2P202126	62526346	G	0.25	0.2857	A
3	BICF2G630343911	62539350	G	0	0	A
3	BICF2P716326	62551278	A	0.75	0.6429	G
3	BICF2G630343957	62566321	A	0.25	0.3571	C
3	BICF2P1137642	62577854	G	0	0	A
3	BICF2P751285	62592273	A	0	0	G
3	TIGRP2P48249_rs9077671	62599963	G	0	0	A
3	BICF2G630344005	62612669	A	0.25	0.3571	G
3	BICF2G630344018	62624404	A	0	0	G
3	BICF2G630344019	62625087	A	0	0	G
3	BICF2P1138157	62634378	A	0.5	0.6429	G
3	TIGRP2P48255_rs9059761	62651265	0	0	0	G
3	BICF2G630344050	62661742	A	0.5	0.6429	C
3	BICF2S23659655	62670368	A	0.75	0.7143	G
3	BICF2G630344069	62679327	A	0	0	G
3	BICF2G630344078	62688040	A	0.25	0.2857	G
3	BICF2P439860	62704095	A	0.75	0.7143	C
3	BICF2G630344104	62705734	A	0.25	0.3571	G
3	TIGRP2P48277_rs8579528	62711121	A	0.5	0.3571	C
3	BICF2P1352497	62725738	A	0	0	G
3	BICF2P423242	62733600	G	0	0	A
3	TIGRP2P48312_rs8703374	62751020	0	0	0	C
3	BICF2G630344171	62766706	G	0.5	0.3571	A
3	BICF2P394142	62775318	A	0	0	G
3	BICF2P724879	62778400	G	0	0	A
3	BICF2P724891	62780161	A	0	0	G
3	TIGRP2P48361_rs8664040	62792396	A	0	0	G
3	BICF2P1285989	62804560	G	0.5	0.3571	A
3	BICF2G630344210	62815972	G	0.75	0.7857	A
3	BICF2G630344249	62830323	A	0	0	G
3	BICF2P36500	62850765	C	0	0	A
3	BICF2S23528080	62864057	A	0.75	0.5714	G
3	BICF2S23025030	62876620	G	0	0	A
3	BICF2P1284604	62877415	G	0	0	A
3	BICF2P310822	62893465	0	0	0	G
3	BICF2P310823	62894273	A	0.25	0.2143	G
3	BICF2P852798	62904883	A	0	0	G
3	BICF2G630344296	62925469	A	0	0	C
3	BICF2G630344299	62928679	G	0.25	0.4286	A
3	BICF2G630344324	62938727	A	0	0	G
3	BICF2G630344330	62945847	A	0	0	C
3	BICF2G630344343	62951807	A	0	0	G
3	BICF2G630344371	62968609	A	0	0	G

3	BICF2G630344389	62981499	G	0	0	A
3	BICF2G630344410	62985536	G	0	0	A
3	BICF2G630344441	63000129	G	0.75	0.7857	A
3	BICF2P874613	63014606	0	0	0	C
3	BICF2G630344491	63026180	A	0.25	0.4286	T
3	BICF2S23160787	63042676	A	0.5	0.6429	G
3	BICF2P920025	63044344	A	0.25	0.2143	G
3	BICF2P467364	63058894	A	0.25	0.2143	G
3	BICF2S23251871	63065087	A	0	0	G
3	BICF2P980846	63074953	A	0.25	0.4286	G
3	BICF2S23245766	63082816	A	0	0	G
3	BICF2S23758385	63087817	A	0.25	0.1667	G
3	BICF2P1458660	63090355	A	0.25	0.4286	C
3	BICF2P133062	63101353	A	0	0	G
3	BICF2P625063	63117855	G	0.25	0.2143	A
3	TIGRP2P48552_rs8535624	63134021	A	0	0	G
3	TIGRP2P48556_rs8535625	63135741	A	0	0	C
3	BICF2G630344508	63148652	A	0	0	G
3	BICF2G630344522	63168062	A	0.75	0.7857	G
3	BICF2G630344525	63169634	G	0.75	0.7857	A
3	BICF2G630344562	63182153	A	0	0	G
3	BICF2G630344575	63196234	G	0	0	A
3	BICF2S23320076	63202177	G	0	0	A
3	BICF2P913696	63209002	A	1	1	C
3	BICF2G630344606	63223013	A	0	0	G
3	BICF2G630344617	63228310	G	0	0	A
3	BICF2G630344656	63245413	G	0.5	0.3571	A
3	BICF2G630344667	63250714	A	0.25	0.2143	G
3	BICF2P953182	63255964	A	0.5	0.3571	G
3	BICF2S2303353	63258859	A	0.25	0.2143	C
3	BICF2P767422	63268838	A	0.25	0.2143	G
3	BICF2G630344670	63274295	A	0	0	G
3	TIGRP2P48590_rs9099015	63289377	A	0.25	0.2143	G
3	BICF2G630344694	63304512	G	0	0	A
3	BICF2G630344701	63311225	A	0.25	0.2143	G
3	BICF2G630344715	63322119	G	0	0	A
3	BICF2S23333400	63341213	A	0	0	G
3	BICF2P664975	63345328	A	0.75	0.5714	G
3	BICF2G630344724	63356045	A	0	0	G
3	BICF2P1322888	63368010	A	0	0	G
3	BICF2G630344739	63382691	G	0	0	A
3	BICF2G630344751	63401319	0	0	0	G
3	BICF2G630344768	63419783	0	0	0	G
3	TIGRP2P48652_rs8688145	63425495	A	0.5	0.3571	G
3	BICF2P705264	63435649	C	0	0	A
3	BICF2P1103910	63448348	A	0	0	G
3	BICF2P306560	63449859	A	0	0	G

3	BICF2G630344776	63459071	A	0	0	G
3	BICF2S23352016	63471330	A	0	0	G
3	BICF2S23114047	63484442	A	0	0	G
3	BICF2S23147827	63491641	G	0.5	0.3571	A
3	BICF2P1395947	63505875	A	0	0	C
3	TIGRP2P48699_rs8545474	63515105	G	0.75	0.7857	A
3	BICF2S2327843	63527033	A	0	0.1429	G
3	BICF2P589335	63533946	A	0	0	G
3	BICF2P310837	63540221	A	0.25	0.4286	G
3	BICF2S23212481	63558969	A	0	0.1429	G
3	BICF2P1079764	63578412	G	0.75	0.7857	A
3	BICF2P764626	63584918	A	0	0	G
3	TIGRP2P48745_rs8936838	63600690	A	0	0.1429	G
3	BICF2G630344816	63614805	G	0.25	0.2143	A
3	BICF2P1231012	63618387	A	0	0	G
3	TIGRP2P48762_rs9049599	63637131	A	0	0	G
3	TIGRP2P48765_rs8876448	63641191	G	0.25	0.2857	A
3	BICF2P584373	63660589	0	0	0	C
3	TIGRP2P48774_rs8910077	63667614	A	0	0	G
3	TIGRP2P48779_rs9090811	63680486	C	0	0	A
3	BICF2P964958	63693565	A	0.5	0.3571	G
3	BICF2P1069954	63704137	0	0	0	T
3	BICF2P648863	63712849	A	0.25	0.2857	G
3	BICF2P1239561	63725438	A	0	0	C
3	BICF2P1140868	63740054	G	0.25	0.4286	A
3	BICF2P1275566	63747674	0	0	0	C
3	BICF2P1364100	63757754	A	0.25	0.4286	G
3	BICF2P1393158	63776598	G	0.25	0.4286	A
3	TIGRP2P48834_rs8776478	63791062	A	0.25	0.4	G
3	BICF2S23658886	63805753	A	0.25	0.2857	G
3	BICF2S23647186	63823062	A	0	0	G
3	TIGRP2P48843_rs9241977	63829220	A	0	0	C
3	BICF2P751737	63841073	A	0	0	G
3	BICF2P936646	63852457	G	0.25	0.2857	A
3	TIGRP2P48854_rs9248837	63858405	A	0	0	G
3	BICF2P654531	63860805	G	0.75	0.5714	C
3	TIGRP2P48876_rs8791684	63875363	A	0	0	G
3	TIGRP2P48879_rs9084938	63882531	C	0	0	A
3	BICF2P562884	63907288	G	0.75	0.5714	A
3	BICF2P1347925	63918740	G	0	0	A
3	BICF2P28563	63941417	A	0	0	G
3	BICF2P621451	63952085	A	0.25	0.4286	G
3	BICF2S23628019	63965444	G	0.25	0.2857	C
3	TIGRP2P48892_rs9062439	63975320	A	0	0	T
3	BICF2P787746	63989974	0	0	0	G
3	BICF2P1468862	64006757	G	0	0	A
3	TIGRP2P48895_rs9035989	64013828	A	0	0	G

3	BICF2S23423111	64029682	0	0	0	G
3	BICF2P1417016	64039970	A	0	0	G
3	BICF2P435692	64051789	G	0.25	0.2857	C
3	BICF2P565225	64053888	G	0.75	0.7857	A
3	BICF2P256279	64058087	A	0.75	0.7857	G
3	BICF2P100021	64071242	0	0	0	G
3	BICF2P88342	64082134	G	0.75	0.7857	A
3	BICF2S23147347	64100410	A	0.5	0.3571	G
3	BICF2S23019603	64121948	A	0.5	0.3571	G
3	BICF2P247653	64127047	A	0.25	0.2857	G
3	BICF2P1024218	64135951	G	0.75	0.7857	A
3	TIGRP2P48916_rs8937700	64148415	G	0.25	0.2857	A
3	BICF2P174683	64161002	C	NA	NA	A
3	BICF2S23037111	64170680	A	0	0.1429	G
3	TIGRP2P48923_rs9082981	64181840	C	0	0.1429	A
3	BICF2P515	64195515	A	0	0	C
3	BICF2P301639	64205016	A	0	0	G
3	TIGRP2P48928_rs9231399	64218794	G	0	0	A
3	BICF2P395307	64227935	A	0	0	G
3	TIGRP2P48933_rs8545710	64240590	C	0	0	A
3	TIGRP2P48939_rs8938291	64258079	A	0	0	G
3	TIGRP2P48942_rs9214541	64267241	G	0	0	A
3	BICF2P775482	64279010	G	0	0	A
3	TIGRP2P48949_rs8984522	64289056	G	0.25	0.2857	A
3	TIGRP2P48951_rs8882532	64297322	G	0.25	0.2857	A
3	BICF2S2444942	64311064	A	0.25	0.2	G
3	BICF2P773345	64320192	A	0.25	0.25	G
3	TIGRP2P48970_rs9050658	64335071	0	0	0	C
3	BICF2S23453461	64350980	G	0.5	0.5	A
3	TIGRP2P48980_rs9032474	64354729	G	0.5	0.5	A
3	BICF2P411106	64365635	A	0.5	0.3571	G
3	BICF2S23538058	64386124	A	0	0	G
3	BICF2P1109281	64394072	C	0.5	0.5	A
3	BICF2P423517	64411319	A	0.5	0.3571	G
3	BICF2P264341	64421993	A	0.5	0.3571	G
3	BICF2S23643404	64433666	G	0.5	0.3571	A
3	BICF2P541067	64439304	A	0.5	0.3571	G
3	BICF2P1052534	64452764	C	0	0	G
3	BICF2S239724	64458956	0	0	0	A
3	BICF2P1365720	64472930	A	0.5	0.3571	G
3	BICF2P1358359	64503166	G	0.5	0.3571	A
3	BICF2S23037417	64508829	0	0	0	A
3	BICF2P242101	64525419	G	0.5	0.5	A
3	BICF2P1467932	64535682	C	0.25	0.2143	A
3	BICF2S23641880	64545361	A	0.25	0.2143	G
3	TIGRP2P49028_rs8602859	64558746	C	0.25	0.2143	A
3	BICF2S2367608	64561624	A	0.25	0.2143	G

3	BICF2S23237800	64566686	A	0.25	0.2143	G
3	BICF2P430946	64573123	G	0.25	0.2143	A
3	BICF2P1225657	64603358	G	0	0	A
3	BICF2S23635879	64610858	0	0	0	G
3	BICF2S23221435	64640241	A	0.25	0.2143	G
3	BICF2S23154903	64642649	G	0.75	0.5714	A
3	BICF2S23653614	64658336	A	0.75	0.5714	G
3	BICF2S2324339	64669920	G	0.5	0.3571	A
3	TIGRP2P49036_rs8805651	64710816	0	0	0	G
3	BICF2S23040613	64723341	C	0.25	0.2143	A
3	BICF2P490876	64737796	0	0	0	G
3	BICF2S2392712	64749333	G	0.5	0.3571	A
3	BICF2P513996	64769568	C	0.5	0.5	A
3	BICF2P1264870	64800557	0	0	0	A
3	BICF2P804811	64826403	C	0.5	0.5	A
3	BICF2P739402	64861390	G	0	0	A
3	BICF2P43095	64879649	G	0.5	0.5	A
3	BICF2P1352501	64902960	A	0.5	0.5	G
3	BICF2P1153028	64909200	A	0.25	0.2857	G
3	BICF2P1206807	64935199	0	0	0	A
3	BICF2P639074	64945617	0	0	0	C
3	BICF2S2331215	64966522	C	0.75	0.7143	A
3	BICF2P1331666	64978020	A	0.5	0.6429	G
3	BICF2P1438357	65005845	A	0.25	0.3571	G
3	BICF2S2329727	65018201	A	0.25	0.2857	G
3	TIGRP2P49043_rs8626439	65028420	0	0	0	C
3	BICF2P1178263	65040857	0	0	0	C
3	BICF2P1080329	65078184	G	0.25	0.3571	A
3	BICF2P669450	65096689	A	0	0	G
3	BICF2P66930	65101888	0	0	0	G
3	BICF2P288807	65118122	0	0	0	A
3	BICF2P1316750	65131354	G	0	0	A
3	BICF2P1334644	65140462	G	0	0	A
3	BICF2P729782	65151281	G	0.5	0.3333	A
3	BICF2P843852	65161813	A	0	0	G
3	BICF2P866800	65178515	A	0	0	C
3	BICF2P558595	65184078	G	0	0	A
3	BICF2P27635	65194743	G	0	0	A
3	BICF2S2344808	65209415	G	0.75	0.6429	A
3	BICF2S2374775	65226916	G	0.75	0.6429	A
3	TIGRP2P49066_rs8525028	65235594	G	0.5	0.3571	A
3	BICF2P178120	65243659	G	0.5	0.3571	A
3	BICF2P1172482	65246590	G	0.5	0.3571	A
3	BICF2P1117410	65253431	G	0.5	0.3571	A
3	BICF2P662321	65280297	A	0.5	0.3571	C
3	TIGRP2P49082_rs9178526	65285982	G	0.5	0.3571	A
3	BICF2P74058	65302811	A	0	0	G

3	TIGRP2P49103_rs9146336	65316634	A	0	0	G
3	BICF2P837418	65320722	G	0	0	C
3	TIGRP2P49111_rs8957607	65339786	A	0	0	G
3	TIGRP2P49119_rs8846167	65353514	G	0.25	0.3571	A
3	BICF2P894545	65356384	G	0	0	A
3	BICF2P162060	65374962	0	0	0	G
3	BICF2P606342	65387457	A	0	0	G
3	BICF2P1126258	65398104	A	0.25	0.3571	G
3	TIGRP2P49153_rs8613185	65402031	A	0.25	0.3571	C
3	BICF2P321923	65420284	A	0.5	0.3571	G
3	TIGRP2P49171_rs8870704	65432281	G	0.25	0.3571	A
3	BICF2S233196	65435606	0	0	0	G
3	TIGRP2P49191_rs8805750	65448134	A	0	0	G
3	BICF2P520230	65464435	G	0.5	0.6429	A
3	BICF2P769649	65471660	C	0.25	0.3571	G
3	BICF2P1307374	65491350	A	0.25	0.3571	G
3	BICF2S23022381	65492218	A	0.25	0.3571	G
3	BICF2P453832	65495867	G	0.25	0.3571	A
3	BICF2P719471	65508171	A	0.5	0.7	C
3	BICF2P326606	65521032	A	0.5	0.6429	T
3	BICF2P300958	65532774	G	0	0	A
3	TIGRP2P49234_rs8488140	65539565	C	0.25	0.3571	A
3	TIGRP2P49239_rs9250235	65559164	G	0	0	A
3	BICF2P658394	65566630	G	0	0	A
3	BICF2P490086	65577908	G	0.25	0.3571	A
3	BICF2P502811	65585619	C	0.25	0.3571	A
3	TIGRP2P49255_rs8824353	65599045	A	0.25	0.3571	T
3	BICF2P934755	65608113	C	0.25	0.3571	A
3	BICF2P1250546	65624121	A	0.25	0.3571	G
3	BICF2P301717	65641386	G	1	1	A
3	BICF2P709341	65647100	G	0.5	0.5	A
3	TIGRP2P49281_rs8893465	65656763	A	0	0.1429	G
3	BICF2P569615	65671605	A	0	0.1429	G
3	TIGRP2P49316_rs8806912	65679389	A	NA	0	G
3	BICF2P35664	65692398	A	0	0	C
3	BICF2S23519714	65701180	0	0	0	T
3	BICF2S23618444	65719704	0	0	0	G
3	BICF2P21544	65733033	A	0	0	G
3	BICF2P642457	65741110	A	0.5	0.5	G
3	BICF2S2332988	65754103	A	1	1	G
3	BICF2S23427474	65768058	A	0	0	G
3	TIGRP2P49323_rs8616963	65772273	G	0.25	0.2857	A
3	BICF2S23716361	65782820	A	0	0	T
3	TIGRP2P49338_rs8816394	65798932	A	0.25	0.2857	G
3	BICF2P542628	65805602	C	0.25	0.2857	A
3	BICF2P819375	65819677	A	0.5	0.5	G
3	BICF2P193250	65828321	G	0.5	0.5	A

3	BICF2S2355918	65878368	A	0.5	0.3571	G
3	BICF2P694343	65887720	A	0.5	0.5	C
3	TIGRP2P49370_rs8999964	65902596	A	0.5	0.5	G
3	BICF2P1103227	65909090	A	0.25	0.3571	G
3	TIGRP2P49382_rs9098498	65918354	A	0	0.1429	G
3	BICF2P1090399	65936595	A	0	0.1429	G
3	BICF2P1468754	65946368	G	0	0.1429	A
3	BICF2P555691	65960857	0	0	0	A
3	BICF2P764473	65973039	A	0.25	0.3571	G
3	TIGRP2P49402_rs9012777	65982789	A	0.25	0.2143	C
3	BICF2S23152827	65987276	A	0.25	0.4286	G
3	BICF2P592635	65998877	A	0.75	0.5714	G
3	BICF2S23130617	66013421	G	0.25	0.4286	A
3	BICF2S23638859	66024768	A	0.25	0.4286	G
3	BICF2P1366510	66041506	0	0	0	G
3	BICF2S22951222	66065045	C	0.25	0.2143	G
3	TIGRP2P49443_rs8907435	66069853	G	0.25	0.2143	A
3	BICF2S23736695	66089780	G	0.25	0.2143	A
3	BICF2S22915070	66096931	A	0.25	0.2143	C
3	BICF2P322435	66108564	0	0	0	G
3	TIGRP2P49457_rs8894721	66122325	G	0.25	0.2857	A
3	TIGRP2P49463_rs8782464	66131428	C	0	0	A
3	TIGRP2P49465_rs8603847	66139117	G	0.5	0.5	A
3	BICF2P1097241	66149547	A	0.25	0.2143	C
3	BICF2P240201	66163367	0	0	0	G
3	BICF2P856918	66202083	0	0	0	C
3	TIGRP2P49485_rs8740704	66243437	A	0.25	0.2143	G
3	BICF2P997216	66267153	0	0	0	C
3	BICF2P1014101	66274565	G	0.25	0.2857	A
3	TIGRP2P49493_rs8923196	66286706	G	0.5	0.5	A
3	BICF2P321009	66297524	A	0	0	T
3	TIGRP2P49503_rs8588300	66313308	A	0	0	G
3	BICF2P1317936	66323543	A	0.25	0.2143	G
3	BICF2P640768	66336117	G	0	0	A
3	BICF2P787994	66348280	A	0	0	G
3	BICF2P251730	66377205	A	0	0	C
3	BICF2P298595	66384297	G	0.5	0.3333	A
3	BICF2P416288	66384942	A	0.5	0.3571	G
3	BICF2P1368437	66393222	A	0.5	0.3571	G
3	BICF2P617840	66402781	0	0	0	G
3	BICF2P1294407	66416172	G	0.5	0.3571	A
3	BICF2P312117	66429336	0	0	0	A
3	TIGRP2P49515_rs9087005	66438974	G	0	0	A
3	BICF2P1075017	66451777	A	0	0.1429	C
3	BICF2P734859	66468931	A	0.25	0.3571	G
3	BICF2P1163811	66471791	A	0.25	0.3571	G
3	BICF2P1154239	66491608	A	0.5	0.3571	G

3	BICF2P994544	66494958	G	0	0	C
3	BICF2P542436	66513676	A	0	0	G
3	BICF2P58639	66521123	G	0.5	0.5	A
3	BICF2P48815	66529334	G	0.75	0.7143	A
3	BICF2P959145	66550016	0	0	0	T
3	BICF2P959146	66551971	A	0	0	C
3	BICF2S2392617	66564883	A	0.75	0.7857	G
3	BICF2P1163375	66574331	A	0.75	0.6429	G
3	BICF2P848619	66586691	A	0.5	0.3571	G
3	BICF2P1382935	66598466	A	0.25	0.2143	G
3	TIGRP2P49555_rs9082920	66611026	0	0	0	C
3	BICF2P468740	66625495	0	0	0	G
3	BICF2S23342385	66635649	C	0	0	A
3	BICF2P368516	66663886	G	0.25	0.2857	A
3	BICF2P1396444	66683189	0	0	0	A
3	BICF2P931287	66696910	0	0	0	A
3	BICF2P891498	66701847	G	0.5	0.3571	A
3	BICF2P168507	66717151	A	0	0	G
3	BICF2P610913	66726168	G	0.75	0.7857	A
3	BICF2S23056422	66745235	A	0	0	C
3	BICF2P1073231	66750386	G	0	0	A
3	BICF2P338360	66767746	G	0.25	0.2857	A
3	BICF2S23020868	66776073	G	0.75	0.6429	A
3	BICF2S23116437	66788808	G	0.75	0.6429	A
3	BICF2P1438438	66794802	A	0.25	0.2857	G
3	BICF2P46639	66797351	A	0.25	0.2857	G
3	BICF2P46642	66797875	A	0.25	0.2857	G
3	BICF2P169178	66803028	A	0	0	G
3	BICF2P447527	66810286	G	0.25	0.2857	C
3	BICF2P447529	66811189	A	0	0	G
3	TIGRP2P49576_rs9100268	66824733	A	0	0	G
3	BICF2P773595	66830938	C	0	0	G
3	BICF2S22940151	66840067	A	0	0	G
3	BICF2S22940150	66840260	A	0	0	G
3	BICF2P1384848	66843894	A	0	0	G
3	BICF2S23332321	66867572	A	0.5	0.3571	C
3	BICF2P714427	66873406	G	0	0	A
3	BICF2P1026251	66888730	A	0.25	0.2857	C
3	TIGRP2P49634_rs8688959	66897187	A	0.25	0.3571	G
3	TIGRP2P49655_rs8812824	66910500	G	0.25	0.2857	A
3	BICF2S23415368	66924426	A	0.5	0.3571	C
3	BICF2P44586	66931307	A	0.5	0.5	G
3	BICF2P363864	66944740	A	0.5	0.6429	C
3	TIGRP2P49662_rs8852356	66958772	A	0	0	C
3	BICF2P1293295	66977621	A	0.25	0.3571	G
3	TIGRP2P49666_rs8695194	67012961	G	0.25	0.3571	A
3	BICF2S23727063	67028911	A	0	0	G

3	BICF2P512949	67036555	A	0	0	G
3	BICF2P535851	67055333	A	0	0	G
3	BICF2P309055	67056782	G	0.5	0.6429	A
3	BICF2P688082	67063890	0	0	0	G
3	BICF2P492132	67068552	A	0.25	0.2143	G
3	BICF2S23626216	67074930	G	0	0	A
3	TIGRP2P49699_rs9116058	67082489	0	0	0	G
3	BICF2P530359	67092919	G	0.25	0.2143	A
3	BICF2P683939	67102843	A	0	0	G
3	BICF2S22942140	67114726	G	0.5	0.5	A
3	BICF2P1386441	67125807	C	0	0	G
3	BICF2P65473	67131513	A	0.25	0.2857	G
3	TIGRP2P49720_rs8614640	67138798	G	0.75	0.6429	A
3	BICF2P1073727	67154892	G	0.75	0.6429	A
3	BICF2S23751029	67161315	G	0	0	A
3	BICF2P567431	67171666	A	0.75	0.6429	G
3	BICF2P26328	67190435	A	0	0	G
3	BICF2P1424969	67202324	G	0	0	A
3	TIGRP2P49740_rs8959733	67208156	A	0.5	0.3571	G
3	BICF2P240270	67223110	0	0	0	C
3	BICF2P712052	67234725	0	0	0	A
3	BICF2P1424914	67239116	0	0	0	A
3	BICF2P317973	67245773	A	0.25	0.2143	G
3	TIGRP2P49760_rs8958538	67261480	A	0.25	0.2857	G
3	BICF2S23142243	67268191	G	0.25	0.2857	A
3	BICF2P1043342	67278561	A	0.25	0.2143	G
3	BICF2P894852	67287393	A	0.75	0.7857	G
3	BICF2P78723	67298418	0	0	0	G
3	BICF2P358456	67314634	G	0	0	A
3	BICF2P1443597	67331430	G	0.5	0.3571	A
3	BICF2P7008	67335818	A	0.5	0.3571	G
3	BICF2P552620	67350592	A	0.75	0.7857	G
3	TIGRP2P49812_rs9051671	67358831	T	0.75	0.7857	A
3	BICF2P869841	67367856	G	0	0	A
3	BICF2S22917354	67387649	G	0.25	0.4286	A
3	BICF2S2299139	67391711	T	0.5	0.6429	A
3	BICF2S22932414	67411536	A	0	0.1429	C
3	BICF2P180050	67419174	A	0.25	0.3571	G
3	BICF2S2457463	67430638	A	1	0.8571	G
3	BICF2S23428312	67445082	G	0.25	0.3571	A
3	BICF2S23539070	67452634	G	0.5	0.3571	A
3	BICF2S22947988	67459698	G	0.75	0.6429	A
3	BICF2G630344836	67480795	G	0.75	0.6429	A
3	BICF2P995134	67490324	C	0.5	0.5	A
3	TIGRP2P49822_rs8875284	67498577	G	0.25	0.2143	A
3	BICF2P816097	67507471	A	0	0	G
3	TIGRP2P49838_rs8998603	67533451	A	0	0.1429	G

3	BICF2P661179	67548649	A	0	0.1429	G
3	BICF2P635501	67551095	G	0.5	0.5	A
3	BICF2P519601	67574906	A	0	0.1429	G
3	BICF2S23153107	67578621	G	0.5	0.5	A
3	BICF2S23131014	67591489	G	0.25	0.2857	A
3	BICF2G630344838	67601686	0	0	0	G
3	BICF2G630344853	67612212	0	0	0	A
3	BICF2S23433396	67629000	A	1	0.8571	G
3	BICF2G630344873	67635310	A	0	0.08333	G
3	BICF2G630344887	67643449	0	0	0	G
3	BICF2G630344897	67658709	A	0	0	G
3	BICF2P1471820	67666262	G	0	0.08333	A
3	BICF2P1264821	67685912	A	0.25	0.4286	G
3	BICF2G630344913	67686137	A	0.25	0.2857	G
3	BICF2G630344915	67686711	A	0.5	0.3571	G
3	BICF2G630344927	67691251	A	0	0	G
3	BICF2G630344936	67705849	A	0	0	G
3	BICF2G630344945	67712095	A	0.75	0.5714	G
3	BICF2G630344967	67724932	G	0.75	0.7143	A
3	BICF2P557415	67725714	G	0.75	0.7143	A
3	BICF2G630344981	67735724	A	0.75	0.5714	T
3	TIGRP2P49866_rs9008125	67740234	G	0	0	A
3	BICF2G630345011	67755303	A	0	0	G
3	BICF2G630345026	67763483	G	0.25	0.2857	A
3	BICF2P309665	67779501	0	0	0	A
3	BICF2G630345065	67786972	A	0	0	G
3	BICF2G630345066	67787519	A	0.75	0.7143	G
3	BICF2G630345080	67794889	A	0	0	G
3	BICF2S23331471	67810190	G	0.75	0.7143	A
3	BICF2G630345101	67820032	G	0.25	0.2857	A
3	BICF2G630345121	67831403	0	0	0	A
3	BICF2G630345144	67844629	0	0	0	A
3	BICF2G630345156	67852801	C	0.5	0.6429	A
3	BICF2G630345196	67872772	C	0	0	G
3	BICF2G630345201	67874772	0	0	0	G
3	BICF2G630345220	67888202	C	0	0	G
3	BICF2S23218201	67897603	A	0.25	0.2143	G
3	BICF2P1283067	67914760	G	0.75	0.6429	A
3	BICF2G630345260	67923630	0	0	0	G
3	BICF2P285312	67932091	A	1	0.8571	T
3	BICF2P473628	67937731	A	1	0.8571	G
3	BICF2P1185439	67944661	A	0.5	0.3571	G
3	BICF2P709396	67962554	0	0	0	T
3	BICF2P1123361	67975617	A	1	0.8571	G
3	BICF2P1123362	67977288	A	0	0	G
3	BICF2P1251459	67990743	A	0.5	0.3571	G
3	BICF2P1132914	68023120	A	0	0	G

3	TIGRP2P49917_rs9117548	68044070	0	0	0	G
3	BICF2P959251	68053465	G	0	0	A
3	BICF2P1145840	68069386	A	0	0	C
3	BICF2P761855	68085992	G	0	0	A
3	BICF2P274285	68099807	A	0.5	0.5	G
3	BICF2P138501	68103223	A	0	0.08333	C
3	BICF2P228853	68124649	A	0	0.1429	C
3	BICF2P609956	68126903	A	0	0.1429	G
3	BICF2P1213451	68139813	A	0	0	C
3	BICF2S23640431	68165617	G	0.5	0.5	A
3	BICF2S23344482	68205869	A	0.75	0.5714	G
3	BICF2P439249	68209925	A	0.25	0.2857	G
3	BICF2P7565	68219772	A	0.25	0.2857	G
3	BICF2P245896	68229669	A	0	0	C
3	TIGRP2P49928_rs8790711	68242993	A	0.25	0.2857	G
3	BICF2S23143373	68260652	G	0	0.1429	A
3	BICF2S23345176	68267363	A	0	0	G
3	BICF2S23014631	68282170	A	0	0	G
3	BICF2P846585	68288751	G	1	0.75	A
3	BICF2P1103033	68307060	A	0.5	0.3571	C
3	TIGRP2P49942_rs8703629	68310410	A	0	0	C
3	BICF2P973388	68331032	A	0	0.1429	G
3	BICF2S2316059	68333394	G	0.75	0.6667	A
3	BICF2S23132463	68339839	A	0.25	0.4286	G
3	BICF2P467898	68345210	A	0.25	0.2857	G
3	BICF2S2322715	68366130	G	0.75	0.7857	A
3	BICF2P1451136	68377590	A	0	0	G
3	BICF2S23730494	68389918	G	0.25	0.2857	A
3	BICF2P414412	68398777	G	0.75	0.8333	A
3	BICF2P1372577	68403630	A	NA	1	G
3	BICF2P457326	68414265	A	0.75	0.7143	G
3	BICF2P654341	68428527	A	0.25	0.2857	G
3	BICF2S23617521	68441724	G	0	0	A
3	TIGRP2P49969_rs8871623	68454861	A	0	0	G
3	BICF2P1237477	68466584	A	0.5	0.3571	G
3	BICF2P489823	68479078	G	0.5	0.5	A
3	BICF2P1386329	68491366	G	0.25	0.2143	C
3	BICF2P1186035	68504949	G	0.5	0.5	A
3	BICF2P132838	68506087	A	0.5	0.5	G
3	TIGRP2P49979_rs8990898	68527985	G	0	0	A
3	BICF2P609698	68529551	A	0	0	G
3	TIGRP2P49999_rs8581867	68553145	A	0.75	0.7857	G
3	BICF2P177393	68574297	A	0	0	C
3	TIGRP2P50007_rs9061085	68579798	A	0.5	0.5	G
3	BICF2S23043248	68589318	G	0.5	0.5	A
3	BICF2P187049	68593030	A	0.5	0.5	G
3	BICF2P1324033	68602798	A	0.5	0.5	G

3	BICF2P1324491	68616598	A	0.5	0.5	G
3	BICF2S23427334	68628755	A	0.5	0.5	G
3	TIGRP2P50020_rs8998002	68640323	C	0.5	0.3571	A
3	BICF2P51950	68646738	A	0	0.1429	G
3	BICF2P502761	68659250	G	0	0.1429	A
3	BICF2S23758753	68667118	G	0.5	0.3571	A
3	BICF2P929916	68678428	0	0	0	C
3	BICF2P72727	68734456	A	0	0	T
3	TIGRP2P50046_rs9033895	68745255	A	0.25	0.2857	C
3	BICF2P894266	68765812	G	0.25	0.2143	A
3	TIGRP2P50052_rs8620719	68777949	0	0	0	A
3	BICF2P1364518	68791918	0	0	0	G
3	TIGRP2P50055_rs8669528	68795364	A	0	0	G
3	BICF2P593682	68813286	G	0.5	0.6429	A
3	BICF2P18746	68819182	C	0	0	G
3	BICF2P779130	68822743	C	0	0	A
3	BICF2P710229	68828416	0	0	0	A
3	BICF2P500977	68854204	G	0	0	A
3	TIGRP2P50073_rs8498840	68862653	A	0	0	G
3	TIGRP2P50082_rs9191723	68874216	G	0.25	0.2857	A
3	BICF2P1126281	68886788	A	0.75	0.6429	G
3	BICF2S2315924	68900707	A	0	0	G
3	BICF2P565216	68910570	A	0.75	0.6667	C
3	BICF2P723911	68928478	G	1	0.8571	A
3	BICF2S23738452	68933915	G	0.25	0.2857	A
3	BICF2P648372	68945567	A	0.25	0.3571	C
3	BICF2S22957793	68960830	A	0.25	0.2143	G
3	BICF2P527136	68975843	A	0	0	C
3	BICF2P1186716	68986874	A	0.5	0.5	C
3	BICF2P1186710	68990291	G	0	0.1429	A
3	BICF2P303544	69006427	0	0	0	A
3	BICF2P732794	69021070	A	0	0.1429	G
3	BICF2S2329505	69031025	A	0.5	0.6429	G
3	BICF2P1097648	69035536	G	0	0	A
3	BICF2P243298	69051615	A	0	0	G
3	BICF2P524402	69062854	A	0	0	G
3	BICF2P379989	69076414	A	0	0	C
3	BICF2P1467922	69088664	0	0	0	A
3	BICF2P261621	69111456	A	0	0.2857	G
3	BICF2P96219	69117997	G	0.75	0.5714	C
3	BICF2P648682	69127226	G	0	0	A
3	BICF2P1129696	69149359	0	0	0	A
3	BICF2P849209	69154338	A	0.75	0.7857	C
3	BICF2S23448872	69161697	A	0	0.1429	G
3	BICF2P328746	69172980	G	0.25	0.2143	A
3	BICF2P1156154	69185359	0	0	0	G
3	BICF2P992809	69203064	G	0.75	0.7857	A

3	BICF2S23520686	69218276	A	0	0	C
3	BICF2P688220	69223699	G	0	0.1429	A
3	TIGRP2P50215_rs8728468	69234436	0	0	0	A
3	BICF2P1083868	69243978	0	0	0	A
3	BICF2P916420	69258507	0	0	0	G
3	BICF2P350158	69272190	C	0	0.1429	G
3	BICF2S23714291	69280578	C	1	0.8571	G
3	BICF2P886901	69288749	G	1	0.8571	C
3	BICF2P249565	69299286	A	1	0.8571	C
3	BICF2P935862	69313456	A	0	0	G
3	BICF2P399980	69323758	G	0	0.1429	A
3	TIGRP2P50242_rs8861480	69338780	A	0.25	0.2143	G
3	BICF2S23526990	69355627	A	0	0	G
3	TIGRP2P50246_rs8547899	69359254	A	0	0	G
3	BICF2P54999	69368988	A	0.25	0.2143	C
3	BICF2P969447	69393345	G	0	0	A
3	BICF2P642590	69408881	G	0.75	0.6429	A
3	TIGRP2P50258_rs8798843	69415426	0	0	0	A
3	BICF2P835144	69434513	C	0.25	0.3571	G
3	BICF2G630345263	69435096	A	0	0	T
3	BICF2G630345270	69444577	C	0.25	0.3571	A
3	BICF2G630345286	69459185	G	0.25	0.3571	A
3	BICF2G630345289	69466824	G	0.25	0.3571	A
3	BICF2G630345293	69471784	G	0	0	A
3	BICF2S23136281	69490762	G	0	0	C
3	BICF2G630345318	69503924	A	0.25	0.3571	C
3	BICF2S2296258	69539934	A	0	0	G
3	BICF2G630345330	69544125	A	0	0	G
3	BICF2G630345336	69569274	A	0	0	C
3	BICF2G630345352	69581680	G	0	0	A
3	BICF2G630345367	69589789	A	0.75	0.6429	G
3	BICF2G630345396	69597139	A	0.75	0.6429	G
3	BICF2G630345410	69604454	A	0.25	0.3571	G
3	BICF2G630345426	69610135	C	0.25	0.3571	A
3	BICF2S23250158	69621013	A	1	1	G
3	BICF2G630345466	69634623	A	0	0	G
3	BICF2G630345490	69648339	A	0	0	G
3	BICF2G630345504	69657743	A	0	0	G
3	BICF2G630345507	69669598	G	0.75	0.6429	A
3	BICF2G630345527	69679660	A	0.75	0.6429	G
3	BICF2G630345576	69693921	C	0.75	0.6429	G
3	BICF2G630345596	69709314	C	0.75	0.6429	A
3	TIGRP2P50308_rs8927007	69718879	A	0.25	0.2143	C
3	BICF2G630345618	69725979	A	0.75	0.6429	C
3	BICF2G630345625	69730837	A	0	0.1429	G
3	BICF2G630345635	69745232	A	0.75	0.6429	G
3	BICF2G630345647	69754125	G	0.75	0.6429	A

3	BICF2G630345649	69760998	A	0	0.1429	G
3	BICF2P10506	69778761	0	0	0	G
3	BICF2G630345673	69790926	T	0.75	0.6429	A
3	BICF2G630345674	69791354	A	0.75	0.6429	G
3	BICF2G630345690	69802257	C	0.75	0.7857	A
3	TIGRP2P50320_rs8755840	69808184	0	0	0	A
3	BICF2P1081142	69816728	C	0	0	A
3	BICF2P459498	69833015	A	0	0	G
3	BICF2G630345711	69840368	A	0	0.1429	G
3	BICF2G630345740	69857166	A	0	0	T
3	BICF2G630345754	69862892	G	0	0	A
3	BICF2G630345774	69879927	C	0.5	0.3571	G
3	BICF2G630345786	69890009	G	0.5	0.3571	A
3	BICF2G630345802	69905649	A	0.75	0.6429	C
3	BICF2G630345808	69911168	T	0.5	0.5	A
3	BICF2G630345826	69920556	A	0	0	G
3	BICF2G630345828	69933007	T	0	0	A
3	BICF2P1214528	69946507	A	0.25	0.4286	T
3	BICF2G630345839	69960441	A	0	0	G
3	BICF2P17991	69975205	C	0	0.1429	A
3	BICF2S23526539	69977749	A	0.75	0.5714	G
3	BICF2S23355278	69995706	G	0	0.1429	A
3	BICF2P948881	70009692	A	0	0.1429	G
3	BICF2P79149	70014068	G	0	0.1429	A
3	TIGRP2P50394_rs9036798	70028209	G	0.75	0.7857	A
3	BICF2P758703	70035165	G	0.25	0.4286	A
3	BICF2G630345852	70049657	A	0	0	G
3	BICF2G630345858	70054032	A	0	0	G
3	BICF2G630345875	70064843	A	0.75	0.6429	T
3	BICF2S2309863	70070862	A	0	0.1429	G
3	TIGRP2P50432_rs9000420	70082400	A	0.25	0.2857	G
3	BICF2G630345921	70097063	C	0	0	A
3	BICF2P518476	70113548	G	0.75	0.6429	A
3	BICF2P518474	70113864	G	0.75	0.6429	A
3	BICF2G630345967	70117499	C	0	0	G
3	BICF2G630345981	70126870	C	0	0.1429	G
3	BICF2G630345989	70130362	A	0	0	G
3	BICF2G630346015	70139605	A	0.5	0.3571	G
3	BICF2G630346055	70152534	A	0	0	C
3	BICF2G630346070	70158591	A	0.75	0.6429	G
3	BICF2G630346076	70164800	G	0	0.1429	A
3	BICF2G630346114	70179319	G	0	0.1429	A
3	BICF2G630346124	70185797	G	0	0.1429	A
3	BICF2G630346139	70203326	A	0.75	0.7143	G
3	BICF2G630346140	70203556	G	0.75	0.7143	A
3	BICF2S23018412	70204740	C	0	0	A
3	BICF2P206870	70210381	A	0	0	C

3	BICF2S2333853	70237721	G	0.25	0.2143	A
3	BICF2G630346148	70243707	A	0	0.1429	G
3	BICF2G630346177	70257239	A	0.5	0.5	G
3	BICF2G630346192	70267418	G	0.5	0.5	A
3	BICF2P687530	70292683	0	0	0	G
3	BICF2G630346205	70304250	C	0	0	A
3	BICF2G630346215	70313892	A	0.25	0.2143	G
3	BICF2G630346253	70328498	G	0	0	A
3	BICF2G630346277	70337891	G	0	0	A
3	BICF2G630346294	70352777	G	0.25	0.2143	A
3	BICF2G630346295	70353088	A	0	0.2143	T
3	BICF2G630346312	70362867	G	0	0	A
3	BICF2G630346337	70369295	A	0	0	G
3	BICF2G630346378	70385175	A	0.25	0.2143	G
3	BICF2P984507	70398896	G	0.25	0.2143	A
3	BICF2G630346400	70405977	A	0.75	0.6429	G
3	BICF2P801735	70419734	A	0.75	0.6429	G
3	TIGRP2P50503_rs8880756	70430956	A	0.75	0.6429	C
3	BICF2G630346420	70442914	G	0	0	A
3	BICF2G630346442	70457519	A	0.75	0.6429	G
3	BICF2P1376419	70466313	A	0	0.1429	C
3	BICF2G630346449	70474108	G	0.75	0.6429	A
3	BICF2G630346466	70495378	A	0	0	G
3	BICF2P534809	70513154	G	0	0.1429	A
3	BICF2G630346470	70518642	C	0	0.08333	A
3	BICF2P530052	70529662	A	0	0	G
3	BICF2G630346481	70542063	G	0	0	A
3	BICF2G630346504	70553341	C	0.25	0.1	A
3	BICF2G630346524	70566153	A	0.25	0.2143	G
3	TIGRP2P50542_rs9199122	70577496	0	0	0	G
3	BICF2G630346570	70592503	A	0.25	0.4286	G
3	BICF2G630346592	70600677	A	0.25	0.4286	C
3	BICF2G630346621	70612887	G	1	1	A
3	BICF2S23741132	70621871	G	0	0	A
3	BICF2G630346633	70635732	A	0	0.1429	G
3	BICF2G630346639	70646946	A	0	0	G
3	BICF2G630346654	70659010	A	0	0	G
3	BICF2G630346685	70673530	A	0	0	G
3	BICF2G630346705	70683990	A	0.25	0.2857	G
3	BICF2G630346722	70696592	G	0	0.2143	A
3	BICF2G630346748	70705296	G	0	0	A
3	BICF2G630346757	70715809	T	0	0	A
3	BICF2G630346806	70726005	A	0	0	G
3	BICF2P1305514	70746883	A	0	0.1429	G
3	BICF2G630346821	70748090	A	0	0.1429	G
3	BICF2G630346846	70765878	A	0	0	T
3	BICF2G630346856	70774716	A	0	0	G

3	BICF2P441522	70791155	A	0	0	C
3	BICF2P441524	70791458	A	0	0	T
3	BICF2P1069152	70798514	A	0	0.1429	C
3	BICF2P219616	70815775	A	0	0.1429	G
3	BICF2G630346906	70836391	A	0	0	G
3	BICF2G630346917	70840759	G	1	1	A
3	BICF2S22918373	70853907	A	0	0	G
3	BICF2G630346944	70865137	A	0.5	0.6429	G
3	BICF2S23338900	70876425	G	0.5	0.5	A
3	BICF2G630346977	70903599	A	0.5	0.6429	G
3	TIGRP2P50581_rs9033606	70914958	C	0.5	0.3571	A
3	BICF2G630346994	70922649	G	0.5	0.6429	A
3	TIGRP2P50592_rs8753807	70935976	A	0	0	G
3	BICF2P738728	70939763	G	0.5	0.6429	A
3	BICF2G630347017	70943959	A	0	0	G
3	BICF2G630347036	70955174	A	0.25	0.3571	G
3	BICF2S22918699	70973257	C	0.5	0.6429	A
3	BICF2G630347062	70981310	A	0	0	G
3	BICF2G630347110	70990639	A	0	0	G
3	BICF2G630347144	71003477	A	0	0	G
3	BICF2P269213	71019627	G	0.25	0.2143	A
3	BICF2G630347165	71028495	G	0.25	0.2143	A
3	BICF2P353800	71045878	A	0.75	0.5714	C
3	BICF2G630347168	71051486	G	0	0	A
3	BICF2S23120885	71062605	G	0.75	0.5714	A
3	BICF2G630347177	71070587	A	0	0	G
3	BICF2G630347196	71082011	G	0.75	0.7143	A
3	BICF2G630347220	71101561	G	0.75	0.7143	A
3	BICF2S23719507	71108118	A	0.75	0.7143	G
3	BICF2G630347233	71116588	C	0.75	0.7143	A
3	BICF2S22949005	71129633	G	0	0	A
3	BICF2G630347243	71145537	A	0.25	0.4286	C
3	BICF2G630347268	71151315	G	0.75	0.7857	A
3	BICF2G630347272	71155871	A	0.75	0.7857	G
3	BICF2G630347317	71182647	A	0	0	G
3	BICF2G630347325	71188032	A	0	0	C
3	BICF2P1319289	71197980	A	0	0	C
3	BICF2P1461417	71214348	G	0	0	A
3	BICF2P1007558	71221439	A	0.75	0.7857	G
3	BICF2G630347341	71232685	T	0	0	A
3	BICF2S23424012	71253392	G	0	0	A
3	BICF2G630347342	71255987	A	0	0	G
3	TIGRP2P50626_rs8682637	71272924	G	0	0	A
3	BICF2P1124383	71281919	A	0	0	T
3	BICF2G630347351	71293182	A	0.75	0.6429	C
3	BICF2S23021807	71301841	G	0.25	0.2857	A
3	BICF2G630347383	71322263	A	0.25	0.2143	G

3	BICF2G630347384	71323591	A	0	0.1429	G
3	BICF2S23238260	71341132	G	0.25	0.2857	A
3	BICF2S23338505	71353837	G	0.75	0.5714	A
3	BICF2P493832	71368891	T	0	0	A
3	BICF2G630347399	71378585	G	0	0	A
3	BICF2G630347417	71388170	C	0	0.1429	A
3	BICF2G630347439	71395797	G	0	0.1429	A
3	BICF2G630347463	71404996	G	0	0.1429	A
3	BICF2G630347491	71415537	A	0	0.1429	G
3	BICF2G630347520	71427205	A	0	0	G
3	BICF2P72337	71445387	G	1	0.8571	A
3	BICF2P432596	71453898	A	0.25	0.2857	T
3	BICF2G630347578	71468137	A	0.25	0.2857	G
3	BICF2P221716	71475304	A	0.25	0.4167	G
3	BICF2G630347622	71487729	A	0.25	0.4286	G
3	BICF2P365699	71496977	G	0.25	0.4286	A
3	BICF2S2303708	71527181	A	0	0.1429	G
3	BICF2G630347627	71538386	A	1	0.8571	C
3	TIGRP2P50724_rs8961586	71541987	G	0	0.1429	A
3	BICF2P992012	71555943	G	0	0.1429	A
3	BICF2G630347652	71568191	A	0	0	G
3	BICF2G630347666	71573298	A	0.5	0.5	G
3	BICF2G630347692	71583344	G	0.5	0.5	A
3	BICF2G630347699	71587787	A	0.5	0.5	G
3	BICF2G630347737	71600779	A	0.5	0.5	G
3	BICF2P217260	71613837	0	0	0	A
3	BICF2G630347746	71624503	G	0.5	0.5	A
3	BICF2G630347756	71641175	G	0	0.1429	A
3	BICF2G630347769	71646796	A	0	0.08333	G
3	BICF2P121267	71657451	C	0	0	A
3	BICF2G630347779	71668200	C	0.25	0.3571	A
3	BICF2G630347788	71688922	A	0	0.1429	G
3	BICF2G630347794	71702440	A	0	0.1429	G
3	BICF2G630347798	71707174	G	0.75	0.5714	A
3	BICF2P444394	71709260	C	0.75	0.5714	A
3	BICF2G630347814	71719914	G	0	0	A
3	BICF2G630347836	71736803	G	0	0.1429	A
3	BICF2G630347841	71737753	A	1	0.8571	G
3	BICF2G630347859	71751778	G	0.5	0.5	A
3	BICF2G630347872	71760916	A	0.5	0.5	G
3	BICF2S23548534	71772975	C	0.25	0.2143	G
3	BICF2S23548535	71772992	A	0.25	0.2143	G
3	BICF2S23548540	71773212	A	0.25	0.2143	G
3	BICF2P770452	71787096	0	0	0	C
3	TIGRP2P50780_rs8719116	71800066	0	0	0	G
3	BICF2G630347886	71815398	A	0.5	0.5	G
3	BICF2G630347890	71821018	A	0.5	0.5	G

3	BICF2G630347902	71829153	G	0.25	0.2857	A
3	BICF2G630347930	71856931	G	0.75	0.5714	A
3	BICF2G630347951	71870691	A	0	0	G
3	BICF2G630347953	71870968	G	0	0	A
3	BICF2P1468025	71879154	G	0	0	A
3	BICF2P557472	71896408	G	0.75	0.7857	A
3	BICF2P869960	71898732	A	0.75	0.7857	G
3	BICF2P1109292	71912261	A	0	0	T
3	BICF2P60654	71923771	A	0	0	G
3	BICF2P615665	71932535	G	0	0	A
3	BICF2P1272848	71948543	G	0	0	A
3	BICF2P330663	71959362	0	0	0	G
3	TIGRP2P50831_rs8956749	71973132	G	0	0	A
3	TIGRP2P50833_rs8932257	71982066	C	0	0	G
3	BICF2P178053	71996218	A	0.5	0.5	G
3	TIGRP2P50846_rs8702602	72005779	A	0	0	C
3	BICF2P1127874	72015450	A	0	0	G
3	BICF2P9173	72034203	A	0	0	G
3	BICF2P625852	72038826	0	0	0	A
3	BICF2P566461	72051166	0	0	0	G
3	BICF2S22954802	72064496	A	0.5	0.5	C
3	BICF2P228774	72073318	A	0	0	G
3	BICF2P1192071	72093129	G	0.5	0.5	A
3	BICF2S23732589	72098850	G	0.5	0.5	A
3	BICF2S2321653	72113424	A	0.5	0.5	T
3	TIGRP2P50882_rs8551791	72119854	G	0.5	0.5	A
3	BICF2P242241	72129624	A	0.5	0.5	G
3	BICF2P1392927	72148949	A	0.75	0.6429	G
3	BICF2P667799	72153585	A	0.25	0.2143	G
3	TIGRP2P50911_rs8594995	72164164	A	0	0	G
3	TIGRP2P50914_rs9094815	72183994	C	0.5	0.3571	A
3	BICF2P1072780	72193443	A	0.25	0.2857	G
3	BICF2S23318793	72203466	A	0.25	0.2143	G
3	BICF2P395650	72208959	G	0.5	0.5	A
3	BICF2P923795	72223819	G	0.25	0.2857	A
3	BICF2S2366662	72225068	A	0.25	0.2857	G
3	BICF2S235409	72231761	A	0	0	G
3	BICF2S23145647	72246471	A	0.25	0.2857	C
3	BICF2P961864	72274592	0	0	0	A
3	TIGRP2P50941_rs9148102	72284943	G	0	0	A
3	BICF2P1016747	72298148	0	0	0	G
3	BICF2P1468191	72307188	A	0.25	0.2857	G
3	BICF2P708664	72313899	0	0	0	G
3	BICF2S23060671	72332273	G	0.75	0.5714	A
3	BICF2S2445407	72340915	0	0	0	G
3	BICF2P1135229	72353255	G	0.75	0.5714	A
3	BICF2P957428	72369230	A	0.5	0.3571	C

3	BICF2P1333491	72370758	A	0.5	0.3571	G
3	BICF2P243267	72381996	A	0.5	0.3571	G
3	BICF2P360283	72402830	A	0	0	G
3	BICF2P1052561	72404255	A	0	0	G
3	BICF2S23454836	72425599	0	0	0	A
3	BICF2P1136548	72432601	A	0	0	G
3	BICF2P1301995	72441308	A	0	0	T
3	BICF2S23248707	72453034	T	0	0	A
3	BICF2P1406464	72466455	G	0	0	C
3	BICF2S23660412	72494744	A	0	0	G
3	BICF2S2325069	72501056	A	0	0	G
3	BICF2P378404	72525542	A	0	0	G
3	BICF2P340157	72532661	0	0	0	A
3	BICF2P1068900	72544096	A	0	0	G
3	BICF2P930535	72559605	A	0	0	G
3	BICF2P788663	72575868	G	0	0	A
3	BICF2P1379863	72596815	A	0	0.1429	G
3	BICF2S22916658	72608124	G	0.25	0.2143	A
3	BICF2P610015	72611617	0	0	0	A
3	BICF2P1238123	72631735	C	0.25	0.3571	G
3	TIGRP2P51004_rs8687942	72636084	C	0.25	0.3571	A
3	BICF2P1392344	72646112	A	0.75	0.6429	G
3	BICF2S23010369	72654000	A	0.75	0.6429	T
3	BICF2P873500	72663570	A	0.25	0.2143	G
3	BICF2P461716	72666187	G	0.75	0.7857	A
3	BICF2S23516817	72690768	G	0.75	0.7857	A
3	BICF2P96174	72695282	G	0.75	0.6429	A
3	BICF2P825759	72704274	A	0.75	0.7857	C
3	BICF2S23248106	72722088	G	0.75	0.7857	A
3	BICF2P521131	72727082	A	0	0.1429	G
3	BICF2P183632	72746555	G	0	0.1429	A
3	BICF2P170895	72754703	0	0	0	A
3	BICF2P420058	72763866	G	0.75	0.7857	A
3	BICF2P894119	72772508	G	0	0.1429	A
3	BICF2P1100109	72784438	C	0	0.1429	A
3	BICF2P261633	72806259	A	0	0	G
3	BICF2P261632	72807411	A	0	0	G
3	BICF2P605847	72826897	G	0.75	0.6429	C
3	TIGRP2P51037_rs9166790	72832353	C	0	0.1429	A
3	BICF2P470927	72841282	A	0	0	G
3	BICF2P545282	72857689	G	0	0	C
3	BICF2S23147554	72868505	0	0	0	G
3	BICF2P80520	72875831	A	0.5	0.5	C
3	BICF2S2374352	72887814	A	0.5	0.5	G
3	BICF2P947946	72892536	G	0.5	0.5	C
3	BICF2P1119585	72905638	C	0.25	0.2143	A
3	BICF2P713236	72931950	A	0.25	0.2143	G

3	BICF2P1438424	72941299	G	0.75	0.5714	A
3	TIGRP2P51100_rs9163137	72945605	G	0	0	A
3	BICF2P709410	72960253	A	0.75	0.5714	G
3	BICF2P312351	72967639	A	0.5	0.3571	G
3	TIGRP2P51108_rs9153785	72990414	A	0	0	C
3	BICF2P1401786	73000579	A	0.75	0.7143	G
3	BICF2S22927352	73001936	A	0.75	0.7143	G
3	TIGRP2P51122_rs8719003	73020643	G	0.75	0.7143	A
3	TIGRP2P51126_rs8638818	73025435	A	0.75	0.6429	G
3	BICF2P1327535	73037104	T	0.75	0.6429	A
3	BICF2P1252199	73058693	A	0.75	0.7857	G
3	BICF2P1377845	73062129	G	0.5	0.5833	A
3	BICF2P896521	73071513	A	0	0	C
3	BICF2P808591	73085096	A	0	0	C
3	BICF2P196588	73093046	A	0.75	0.6429	C
3	TIGRP2P51172_rs8992730	73097368	A	0.25	0.2143	G
3	BICF2S23014059	73106086	A	0	0	G
3	BICF2S2328793	73117143	C	0	0.1429	A
3	BICF2P1448320	73141562	A	0	0.1429	G
3	BICF2P1363449	73159735	G	1	1	A
3	BICF2P1174412	73164522	A	1	1	G
3	BICF2P946015	73177829	G	1	0.9167	A
3	TIGRP2P51213_rs9048321	73192459	T	0.25	0.2857	A
3	BICF2P426848	73204747	A	0.25	0.2857	C
3	BICF2P1050238	73223479	A	0	0	C
3	G258f9S223	73264264	G	0	0.1429	A
3	BICF2S23138930	73308714	A	0	0	G
3	BICF2P1180402	73315471	G	0	0	A
3	TIGRP2P51273_rs8750177	73338962	A	0	0	T
3	BICF2P781101	73347280	0	0	0	A
3	BICF2P354818	73376035	A	0.5	0.5	C
3	BICF2P1047844	73397081	G	0	0	A
3	BICF2P979666	73446002	0	0	0	A
3	BICF2P794473	73471353	A	0	0.1429	G
3	TIGRP2P51291_rs9194575	73480519	0	0	0	G
3	BICF2P1133744	73495967	G	1	0.8571	A
3	BICF2P3479	73502785	G	1	0.8571	A
3	BICF2S2413222	73515498	G	0	0.1429	A
3	BICF2P834603	73515695	A	0	0.1429	G
3	BICF2P1023666	73522218	A	0	0.1429	G
3	BICF2P1167144	73525040	A	0	0.1429	G
3	BICF2P982426	73544442	A	0.75	0.6429	G
3	BICF2S23731276	73560324	A	0.25	0.3571	G
3	BICF2P873286	73571000	G	0.25	0.3571	A
3	BICF2P939079	73578399	A	0	0.1429	G
3	BICF2P659016	73596196	A	0.25	0.4286	G
3	TIGRP2P51334_rs8862149	73597780	G	0.25	0.4286	A

3	TIGRP2P51338_rs9018373	73607697	G	0.25	0.4286	A
3	BICF2P632475	73618543	A	0.25	0.4286	G
3	TIGRP2P51350_rs8849029	73626967	G	0	0.1429	A
3	BICF2P890578	73627212	A	0	0.1429	G
3	BICF2P11678	73636617	A	0	0	G
3	BICF2P1445689	73649181	A	0.75	0.7143	T
3	BICF2P6967	73662071	A	0.25	0.2857	G
3	BICF2S23221558	73673799	A	0	0	G
3	BICF2P281036	73688175	A	0.5	0.3571	G
3	BICF2S23146789	73698381	G	0.25	0.3571	A
3	BICF2P954856	73703936	G	0.5	0.5	A
3	G259f43S141	73729311	A	0.5	0.5	G
3	BICF2P90273	73755990	A	0.5	0.5	G
3	BICF2P1037985	73769188	C	0	0	A
3	BICF2P1119519	73780207	0	0	0	G
3	BICF2S23255725	73784960	G	0.5	0.5	A
3	BICF2P728232	73807753	C	0	0	G
3	BICF2P728233	73807904	C	0	0	A
3	BICF2P180998	73828246	G	0	0	A
3	BICF2P1370573	73833402	A	0	0	G
3	BICF2P581198	73839482	G	0	0	A
3	BICF2P1172461	73843085	0	0	0	G
3	BICF2P69130	73863730	A	0	0	G
3	BICF2P296071	73869145	A	0.5	0.5	C
3	BICF2S23143105	73883322	A	0	0	G
3	BICF2S23018094	73889266	T	0.5	0.5	A
3	BICF2P778817	73902672	C	0.25	0.2857	A
3	TIGRP2P51430_rs8695788	73920450	A	0	0	C
3	TIGRP2P51431_rs8842193	73923070	0	0	0	A
3	BICF2P1066162	73944748	0	0	0	G
3	BICF2S23341988	73946266	G	0.75	0.6429	C
3	BICF2S23027829	73962149	G	1	0.8571	A
3	BICF2P81375	73972394	A	0.5	0.5	G
3	BICF2P29133	73981180	G	0.25	0.2857	A
3	BICF2P257902	73998075	A	0.25	0.3571	G
3	BICF2P1304854	74003993	A	0.5	0.5	G
3	BICF2P916246	74016375	A	0.5	0.5	G
3	BICF2P124436	74023926	A	0.5	0.4167	G
3	BICF2P734792	74026387	G	0.5	0.5	A
3	BICF2S2443102	74047624	G	0.25	0.2143	A
3	BICF2P8218	74055408	0	0	0	C
3	BICF2S23713529	74074557	A	0	0	G
3	BICF2S23055151	74085609	G	0.25	0.2857	A
3	BICF2S22951811	74104472	A	0.5	0.375	G
3	BICF2S239917	74108087	C	0.25	0.4286	A
3	BICF2S23037351	74127585	A	0.25	0.2143	G
3	TIGRP2P51453_rs8489444	74136356	C	NA	0.3333	A

3	BICF2P855213	74161596	A	0	0	C
3	BICF2P358326	74174311	G	0	0	A
3	BICF2S23236572	74189324	G	0	0	A
3	BICF2S23319791	74220247	C	0	0	G
3	BICF2G630347978	74230710	A	0.5	0.3571	C
3	BICF2G630347979	74233163	C	0.5	0.6429	A
3	BICF2G630348010	74259093	G	0	0	A
3	BICF2G630348051	74273641	A	0	0	G
3	TIGRP2P51459_rs9041828	74285157	0	0	0	A
3	BICF2G630348067	74290766	A	0	0	G
3	BICF2G630348101	74305180	G	0	0	A
3	BICF2G630348129	74313818	A	0	0	G
3	BICF2G630348153	74325989	A	0	0	G
3	TIGRP2P51461_rs8628566	74338531	A	0	0	G
3	BICF2G630348194	74351297	G	1	1	A
3	BICF2G630348211	74360719	A	0.5	0.6429	G
3	BICF2G630348227	74376285	G	1	1	A
3	BICF2G630348235	74382533	A	0.75	0.6429	G
3	BICF2S22958901	74397432	A	0.75	0.6429	G
3	BICF2S23034985	74416816	G	0.75	0.7143	A
3	BICF2P528832	74435389	A	0.25	0.2857	G
3	BICF2P245826	74444030	G	0.25	0.3571	A
3	TIGRP2P51482_rs9049112	74459311	A	0.75	0.6429	G
3	BICF2G630348282	74464384	A	0	0	C
3	BICF2G630348283	74465961	A	0	0	G
3	BICF2P335747	74476842	G	0.5	0.3571	A
3	BICF2G630348291	74494813	A	1	0.8571	G
3	BICF2G630348304	74505349	G	0.25	0.4286	A
3	BICF2P417767	74517809	G	0	0.1429	A
3	BICF2G630348308	74523478	A	0.25	0.4286	G
3	BICF2G630348339	74535183	A	0	0	G
3	BICF2P705542	74536246	G	0.5	0.3571	A
3	BICF2G630348347	74538486	G	0.25	0.2857	A
3	BICF2G630348359	74547958	C	0	0	G
3	BICF2G630348375	74556961	A	0.75	0.6429	G
3	BICF2P1433760	74570209	A	0.75	0.6429	T
3	BICF2P1435022	74575003	A	0.5	0.7	G
3	BICF2G630348400	74580325	A	0.75	0.7857	G
3	BICF2S22913713	74597055	G	0	0	A
3	BICF2S232997	74601227	A	0.5	0.5	G
3	BICF2G630348432	74612235	A	0.75	0.5714	G
3	BICF2G630348441	74624197	A	0	0.25	C
3	BICF2P1232035	74646455	A	0.25	0.4286	G
3	BICF2P712567	74655067	G	1	0.8571	A
3	BICF2G630348457	74655694	A	0	0	G
3	BICF2G630348459	74657432	A	0	0	C
3	BICF2G630348466	74665486	A	0	0	G

3	BICF2G630348474	74679563	A	1	0.8571	G
3	BICF2G630348488	74686683	C	0	0	A
3	BICF2G630348501	74696125	G	1	0.8571	A
3	BICF2G630348518	74708966	A	1	0.8571	G
3	BICF2S23549679	74724867	G	1	0.9167	A
3	BICF2P1080484	74743010	G	0	0	A
3	BICF2P39201	74764009	G	0	0	A
3	BICF2P1158700	74787893	G	0	0	A
3	BICF2G630348527	74803723	0	0	0	G
3	BICF2G630348528	74808188	A	0.25	0.2857	G
3	BICF2P337164	74821246	A	0.75	0.7857	G
3	BICF2G630348552	74834524	G	0.25	0.4286	A
3	TIGRP2P51612_rs8726259	74850629	G	0	0	A
3	BICF2P1446224	74863193	G	0.5	0.6429	A
3	BICF2P1016209	74877912	A	0.25	0.2143	G
3	BICF2S2331039	74892123	C	0.25	0.4286	A
3	BICF2G630348560	74901970	G	0.25	0.2143	A
3	BICF2P1291068	74916812	T	0.5	0.3571	A
3	TIGRP2P51626_rs8771887	74923533	0	0	0	G
3	BICF2P420002	74943960	0	0	0	A
3	BICF2G630348561	74947509	0	0	0	C
3	BICF2G630348576	74957735	A	0.5	0.5	G
3	BICF2S23332395	74976281	G	1	0.8571	A
3	BICF2G630348578	74982720	0	0	0	G
3	BICF2P272681	75001794	T	0	0	A
3	BICF2S23335442	75021255	T	0.25	0.3333	A
3	BICF2S23748055	75029902	A	0.5	0.3571	G
3	BICF2S23055790	75044834	C	0.25	0.2857	A
3	BICF2P961084	75058536	G	1	0.8571	A
3	BICF2P332981	75074539	A	0	0.1429	G
3	BICF2S23027120	75084547	A	0	0.1429	G
3	BICF2S23342718	75096037	A	0.5	0.3571	C
3	BICF2P66747	75119943	C	0	0	A
3	BICF2P1215949	75135006	G	0.25	0.2143	A
3	BICF2P1260092	75147101	A	0.25	0.3571	C
3	BICF2G630348586	75163574	A	0.25	0.2857	G
3	BICF2P830792	75165239	A	0.25	0.3571	T
3	BICF2G630348588	75176143	G	0.5	0.3571	A
3	BICF2P30779	75202718	A	0	0.1667	C
3	TIGRP2P51664_rs9040587	75232997	0	0	0	G
3	BICF2G630348608	75251825	A	0	0	G
3	BICF2G630348613	75264196	A	0.5	0.5	G
3	BICF2G630348620	75271134	A	0.25	0.2857	C
3	BICF2P281021	75279795	0	0	0	C
3	BICF2S23142730	75308206	A	0.5	0.5	G
3	BICF2P511640	75327306	0	0	0	A
3	BICF2P830303	75339207	0	0	0	C

3	BICF2G630348651	75363304	G	0.75	0.7857	A
3	BICF2G630348662	75382092	A	0.75	0.7857	G
3	BICF2P1029559	75385573	G	0	0	A
3	TIGRP2P51665_rs8909172	75399202	0	0	0	G
3	BICF2G630348671	75415141	A	0.25	0.4286	G
3	BICF2G630348680	75424473	G	0.25	0.4286	A
3	BICF2G630348681	75429157	A	0.25	0.4286	G
3	BICF2G630348693	75453798	G	0.25	0.4286	A
3	BICF2G630348696	75465521	0	0	0	G
3	BICF2G630348703	75476379	0	0	0	A
3	BICF2G630348705	75486320	A	0.25	0.4286	G
3	BICF2G630348712	75501321	G	0.25	0.4286	A
3	BICF2G630348726	75518355	A	0.25	0.4286	G
3	BICF2G630348740	75528018	0	0	0	A
3	BICF2G630348753	75534860	A	1	1	C
3	BICF2P159353	75548207	0	0	0	A
3	BICF2G630348780	75557345	A	0.25	0.4286	G
3	BICF2G630348802	75594176	A	0.75	0.7857	C
3	BICF2G630348809	75608168	A	1	0.9167	C
3	BICF2G630348813	75612783	A	0.5	0.5	G
3	BICF2G630348832	75625821	G	0.5	0.5	A
3	BICF2G630348853	75639428	A	0.5	0.5	G
3	BICF2G630348856	75645309	0	0	0	G
3	BICF2G630348862	75664237	A	0.5	0.5	G
3	TIGRP2P51678_rs9103452	75670037	0	0	0	A
3	BICF2P496561	75691772	0	0	0	G
3	BICF2P1177787	75703008	0	0	0	A
3	BICF2P758757	75713382	A	0.75	0.5714	T
3	BICF2P69899	75722321	0	0	0	A
3	BICF2P10422	75736804	0	0	0	G
3	TIGRP2P51697_rs9130909	75741694	C	0.75	0.5714	A
3	BICF2G630348870	75760819	0	0	0	C
3	BICF2G630348872	75763252	A	0	0.1429	C
3	BICF2G630348887	75774686	A	0	0.1429	G
3	BICF2P1285271	75790936	0	0	0	G
3	BICF2G630348906	75803421	A	0	0	G
3	BICF2G630348909	75814564	G	0	0	A
3	BICF2S23039999	75837390	A	0	0	G
3	BICF2P622973	75850042	G	0.5	0.5	A
3	TIGRP2P51704_rs8494220	75861173	0	0	0	G
3	BICF2G630348931	75869864	G	0.25	0.3571	A
3	TIGRP2P51712_rs9152073	75888387	A	0	0	G
3	BICF2P60427	75900261	G	0	0	A
3	TIGRP2P51720_rs9084682	75921529	A	0	0	T
3	TIGRP2P51722_rs8677777	75924666	0	0	0	G
3	BICF2P208763	75938480	A	0	0	G
3	BICF2G630348954	75946371	A	0	0	G

3	TIGRP2P51730_rs9081380	75963298	A	0	0	G
3	BICF2P879946	75974378	G	0	0	A
3	BICF2S23055054	75983366	A	0	0	G
3	BICF2S23423331	75994661	A	0.5	0.3333	G
3	BICF2P1160261	76009002	A	0.5	0.3333	G
3	TIGRP2P51747_rs8880370	76018571	A	0	0	C
3	BICF2P971557	76028632	G	0.5	0.3571	A
3	BICF2P508493	76038030	G	0.5	0.3571	A
3	BICF2P158199	76064227	0	0	0	G
3	BICF2G630348959	76088384	A	0.5	0.5	C
3	BICF2P668082	76108602	A	0	0	T
3	BICF2P783171	76145366	0	0	0	T
3	BICF2G630348964	76153739	C	0	0.1429	A
3	BICF2G630348969	76170598	C	0.75	0.5714	A
3	BICF2P432339	76190131	G	0	0	A
3	TIGRP2P51796_rs9160734	76216782	A	0	0	G
3	BICF2G630348974	76224598	A	0.5	0.5	G
3	BICF2G630349009	76236837	G	0.75	0.7143	A
3	TIGRP2P51809_rs9131941	76245472	A	0	0	G
3	BICF2G630349050	76264351	A	1	0.8571	G
3	BICF2G630349065	76268380	G	1	0.8571	A
3	TIGRP2P51811_rs8899455	76280705	A	0.5	0.3571	G
3	BICF2G630349110	76314243	G	0.5	0.3571	A
3	BICF2S23350455	76330010	A	0.5	0.3571	G
3	BICF2G630349119	76358704	C	0	0	G
3	BICF2P535165	76363428	A	0	0	G
3	BICF2P684422	76372063	A	0.75	0.7857	G
3	BICF2P1164341	76398500	A	0.5	0.3571	G
3	TIGRP2P51864_rs8694831	76398656	A	0	0	G
3	BICF2G630349127	76409636	A	0	0	G
3	BICF2G630349145	76421633	A	0	0	G
3	BICF2P628966	76432944	G	0.5	0.5	A
3	BICF2S23156690	76464697	G	0.75	0.7857	A
3	BICF2G630349156	76477343	A	0	0	G
3	BICF2G630349158	76491101	A	0	0	G
3	BICF2P1256783	76501278	G	0.25	0.4286	A
3	BICF2G630349187	76511018	A	0	0	G
3	TIGRP2P51897_rs8761598	76528237	G	1	0.8571	A
3	BICF2P629516	76534139	A	0	0.1429	G
3	BICF2S23635067	76549026	G	0.75	0.5714	A
3	BICF2G630349208	76556394	0	0	0	G
3	BICF2P1303810	76569069	A	0	0	G
3	BICF2G630349242	76585862	A	0	0	G
3	BICF2G630349258	76591546	G	0	0	A
3	BICF2G630349288	76603385	A	0	0	G
3	BICF2G630349313	76616736	C	0.75	0.5714	A
3	BICF2G630349321	76627986	A	0.75	0.5714	G

3	BICF2G630349344	76647136	G	0.75	0.5714	A
3	BICF2G630349388	76670427	G	0.5	0.6429	A
3	BICF2G630349414	76686949	G	0	0.1429	C
3	BICF2S23021560	76687591	A	0.5	0.5	C
3	BICF2G630349419	76695044	G	0	0	A
3	BICF2G630349422	76712664	A	0.75	0.5714	G
3	BICF2G630349427	76718717	A	0.25	0.2857	G
3	BICF2G630349435	76736276	0	0	0	A
3	BICF2G630349439	76742406	G	0.25	0.2857	C
3	BICF2G630349453	76754889	G	0.25	0.2143	A
3	BICF2P1345210	76771428	A	0.5	0.3571	C
3	BICF2G630349487	76775110	A	0.25	0.2857	G
3	BICF2P799197	76779236	A	0	0	G
3	BICF2G630349494	76780451	A	0.25	0.2857	G
3	BICF2P1363352	76787207	A	0	0	G
3	BICF2P767878	76815454	A	0.25	0.2857	G
3	BICF2P1172332	76823135	A	0	0	G
3	BICF2P1128420	76840426	A	0.75	0.7143	C
3	BICF2P1351343	76853434	G	0	0	A
3	BICF2P194303	76858933	A	0	0.1429	G
3	BICF2S23726902	76866712	A	0.25	0.2143	G
3	BICF2P1221611	76868039	A	0.75	0.6429	G
3	BICF2P1010828	76873849	A	0	0.1429	G
3	BICF2S23026850	76882343	C	0.25	0.4286	A
3	BICF2P421182	76889015	G	0.75	0.5714	A
3	TIGRP2P51989_rs8879126	76905021	A	0.25	0.2	G
3	TIGRP2P51992_rs8717996	76915735	G	0.25	0.2143	A
3	BICF2S23314011	76928912	0	0	0	G
3	BICF2S22945795	76931700	A	0	0	G
3	BICF2S22957553	76939044	A	0.5	0.3571	G
3	BICF2P597143	76948680	A	0	0	G
3	TIGRP2P52015_rs9000772	76965152	A	0	0	C
3	BICF2P588803	76965621	G	0.5	0.3571	A
3	BICF2P303600	76972709	A	0	0	G
3	BICF2S2363772	76982570	A	0.5	0.5	G
3	BICF2P447707	76993944	A	1	0.8571	G
3	BICF2P581718	77008938	G	0.5	0.5	A
3	TIGRP2P52037_rs8836449	77017567	G	0.5	0.5	A
3	BICF2P480331	77027506	G	0.75	0.6429	A
3	BICF2P1329771	77044070	A	0	0	T
3	BICF2P1329770	77044398	A	0.5	0.6429	G
3	TIGRP2P52054_rs8894580	77051936	G	0.5	0.3571	A
3	BICF2P198634	77064729	0	0	0	A
3	BICF2P935759	77082250	G	0	0	A
3	TIGRP2P52083_rs8917637	77091309	G	0.25	0.4286	A
3	BICF2S23436526	77100589	A	0.75	0.5714	G
3	BICF2P211600	77114189	G	0.75	0.5714	A

3	BICF2P370559	77123436	G	0.75	0.5714	A
3	BICF2P1034830	77132463	A	0	0	G
3	BICF2P461272	77145667	G	0	0	A
3	BICF2P612776	77163507	G	0.25	0.2857	A
3	TIGRP2P52123_rs8945903	77167296	G	0.25	0.2143	A
3	BICF2P1458746	77186343	0	0	0	G
3	BICF2P357425	77192506	0	0	0	G
3	TIGRP2P52139_rs8921562	77199959	G	0	0	A
3	BICF2S23629281	77211742	A	0	0	G
3	TIGRP2P52152_rs8706382	77232016	A	0.5	0.3571	G
3	TIGRP2P52164_rs9233667	77235984	A	0	0.1429	C
3	BICF2S23120139	77246576	C	0	0.1429	A
3	BICF2P899066	77259768	A	0	0.1429	G
3	BICF2S23054595	77274253	G	0.5	0.6429	A
3	BICF2P254094	77284846	A	0.25	0.4286	G
3	BICF2P780826	77292153	A	0.25	0.3571	G
3	BICF2P174582	77312759	A	0.5	0.3571	G
3	TIGRP2P52204_rs8728111	77319808	A	0.75	0.6429	G
3	TIGRP2P52208_rs8998680	77329699	G	0	0	A
3	BICF2P1331463	77340454	A	0	0.1429	G
3	BICF2P493602	77350118	A	0.5	0.5	C
3	BICF2S23040234	77371515	A	0.25	0.2143	G
3	BICF2S23042961	77375714	A	0.25	0.2143	G
3	BICF2S22911397	77388107	A	0.75	0.6429	G
3	BICF2G630349507	77398683	A	0.5	0.3571	G
3	BICF2G630349514	77403017	A	1	1	G
3	BICF2S23657288	77409440	G	0	0	A
3	BICF2G630349570	77420427	A	0	0	G
3	BICF2G630349577	77421906	G	0	0	A
3	BICF2G630349582	77434496	A	0.5	0.3571	C
3	BICF2P785980	77434528	G	0.25	0.2857	A
3	BICF2G630349611	77450054	A	0.5	0.5	G
3	BICF2P601460	77462040	A	0.75	0.7857	G
3	BICF2G630349638	77473272	C	0	0	G
3	BICF2G630349656	77484653	G	0	0	A
3	BICF2P742001	77492832	C	0.5	0.5	A
3	BICF2P832035	77495231	G	0	0	A
3	BICF2P832034	77495352	G	0.25	0.2857	A
3	TIGRP2P52228_rs8981579	77499353	G	0.75	0.7143	A
3	BICF2P155225	77513136	A	0.25	0.2857	C
3	BICF2P1297019	77523199	G	0.25	0.2857	C
3	BICF2P468395	77535819	A	0	0.1429	G
3	BICF2G630349680	77545893	C	0.5	0.5	A
3	BICF2G630349685	77550729	A	0	0.1429	G
3	BICF2G630349703	77559364	A	0	0.1429	G
3	TIGRP2P52267_rs9018117	77572364	A	1	0.8571	G
3	BICF2G630349736	77598589	G	0	0	A

3	BICF2P98220	77602060	C	0	0	A
3	BICF2G630349762	77617467	G	0	0	A
3	BICF2G630349775	77625147	A	0	0	G
3	BICF2G630349793	77638581	A	0	0	G
3	BICF2S23912836	77650260	0	0	0	A
3	BICF2S23037810	77662474	A	0.25	0.2143	G
3	BICF2G630349802	77676446	A	0.75	0.5714	C
3	TIGRP2P52286_rs8742992	77687076	G	0	0	A
3	G260f57S271	77719513	0	0	0	C
3	BICF2P1251492	77757407	0	0	0	G
3	BICF2P203299	77777415	A	0.5	0.5	G
3	BICF2P511457	77787903	A	0	0.1429	G
3	BICF2P1371258	77799453	0	0	0	A
3	BICF2P367290	77812046	A	0	0.1429	G
3	BICF2S23743883	77839601	A	0	0	G
3	BICF2G630349815	77845643	G	0	0	A
3	TIGRP2P52300_rs8780585	77858838	A	0	0	G
3	BICF2G630349827	77876070	G	0	0	A
3	BICF2P701334	77882539	G	0	0	A
3	BICF2G630349864	77902349	G	0	0	A
3	TIGRP2P52322_rs9163918	77919316	C	0	0	A
3	BICF2P804602	77934564	A	0	0	C
3	BICF2G630349877	77954740	A	0	0	G
3	BICF2G630349906	77969447	C	0	0	A
3	BICF2G630349908	77970949	C	0	0	A
3	BICF2G630349937	77987698	G	0	0	A
3	BICF2G630349943	77995876	G	0	0	A
3	BICF2G630349947	78006496	A	0	0	G
3	BICF2P438218	78019204	G	0	0	A
3	TIGRP2P52342_rs8868715	78044628	0	0	0	A
3	TIGRP2P52344_rs9147391	78053304	C	0	0.1429	A
3	BICF2P928261	78069320	G	0	0.1429	A
3	BICF2P1015040	78083966	0	0	0	A
3	BICF2G630349985	78102542	0	0	0	G
3	BICF2S23764213	78121971	C	0	0	G
3	BICF2P136769	78142294	0	0	0	G
3	BICF2S23111583	78153961	A	1	1	G
3	BICF2P19789	78156052	0	0	0	A
3	BICF2G630350010	78170662	A	0.25	0.3571	G
3	BICF2G630350031	78180593	A	0	0	G
3	TIGRP2P52378_rs8550921	78190148	0	0	0	A
3	TIGRP2P52392_rs9010331	78203243	T	0	0	A
3	BICF2P1079711	78213482	G	0.25	0.2857	A
3	BICF2P731342	78228787	0	0	0	A
3	BICF2G630350055	78245370	C	0	0	A
3	BICF2G630350056	78246123	G	0	0	A
3	BICF2G630350091	78268086	A	0	0	G

3	BICF2G630350096	78270333	A	0	0	G
3	BICF2G630350122	78283283	G	0.25	0.25	A
3	BICF2G630350140	78294250	0	0	0	G
3	BICF2G630350157	78306101	G	0.75	0.6429	A
3	BICF2G630350178	78318394	A	0.5	0.3571	G
3	BICF2G630350205	78337812	A	0	0	G
3	BICF2G630350208	78338645	0	0	0	G
3	TIGRP2P52407_rs8676594	78349582	A	0	0	G
3	BICF2P467023	78365777	G	0.25	0.2857	A
3	BICF2G630350266	78375599	C	0.25	0.2143	A
3	BICF2G630350278	78390514	G	0	0.1429	A
3	BICF2P938917	78401594	G	0.25	0.2857	A
3	BICF2S23613021	78410733	A	0	0	G
3	BICF2P981921	78428265	0	0	0	A
3	BICF2P1394511	78451311	G	0	0	A
3	TIGRP2P52418_rs9005402	78458918	G	0	0	A
3	BICF2P826538	78465543	A	0.25	0.2143	G
3	BICF2G630350280	78481536	0	0	0	A
3	TIGRP2P52420_rs8817908	78487527	A	0.5	0.3571	C
3	BICF2S23053881	78507473	C	0.5	0.5	A
3	BICF2G630350314	78514818	A	0	0	G
3	TIGRP2P52432_rs8898465	78523773	A	0.25	0.4286	G
3	BICF2G630350331	78533723	G	0	0	A
3	BICF2G630350342	78547465	A	0.25	0.4167	G
3	BICF2P220016	78565160	A	0	0	G
3	BICF2G630350370	78568398	G	0.25	0.4286	A
3	BICF2P817449	78585526	A	0	0.4286	C
3	BICF2P88103	78597075	G	0.25	0.4286	A
3	BICF2P285254	78611845	A	0	0.4167	G
3	BICF2G630350421	78619209	A	0	0	G
3	BICF2G630350444	78636039	G	1	0.8571	A
3	BICF2G630350447	78637087	A	0	0.1429	G
3	BICF2G630350457	78649779	A	0	0.1429	G
3	BICF2G630350469	78661044	G	0.25	0.3571	A
3	TIGRP2P52457_rs8886612	78674756	A	0.75	0.6429	G
3	TIGRP2P52467_rs8761491	78694396	0	0	0	A
3	BICF2P515586	78707952	A	0	0.1429	G
3	BICF2P271022	78720491	A	0	0.1429	C
3	BICF2G630350484	78731522	C	0	0.1429	G
3	BICF2G630350495	78744858	C	0	0	A
3	TIGRP2P52493_rs8933866	78754516	G	0.5	0.5	A
3	BICF2P867441	78766026	A	0	0	G
3	BICF2G630350530	78775036	G	0	0.1429	A
3	BICF2P1047609	78794982	A	0.25	0.4286	G
3	BICF2P176587	78808483	T	0.5	0.6429	A
3	BICF2P31893	78810511	A	0.25	0.4286	G
3	TIGRP2P52529_rs9110366	78823092	A	0.25	0.4286	C

3	BICF2P1235979	78836678	A	0.5	0.6429	G
3	BICF2G630350549	78849091	G	0.5	0.6429	A
3	BICF2G630350568	78858764	G	0.5	0.6429	A
3	BICF2G630350585	78871088	G	0.5	0.6429	A
3	BICF2G630350598	78882371	A	0.5	0.6429	G
3	BICF2S23723021	78890598	A	0.25	0.4286	G
3	BICF2G630350625	78905878	G	0.25	0.4286	A
3	TIGRP2P52562_rs8912466	78913355	A	0.5	0.6429	G
3	BICF2G630350655	78925104	A	0.25	0.2143	C
3	BICF2G630350673	78938267	A	0.75	0.5714	G
3	BICF2P158944	78984961	A	0.5	0.3571	G
3	BICF2G630350680	79008271	A	0	0	G
3	BICF2S235788	79032165	A	0	0	G
3	BICF2G630350733	79057111	A	0.75	0.5714	G
3	BICF2G630350750	79066740	A	0	0.1429	G
3	BICF2P350476	79084338	A	0	0.1429	T
3	BICF2G630350797	79096289	G	0.25	0.2857	A
3	BICF2G630350798	79098334	G	0.25	0.2857	A
3	BICF2G630350817	79110313	C	0	0	A
3	BICF2G630350829	79134016	G	0	0	A
3	BICF2G630350854	79148088	G	0.75	0.6429	A
3	BICF2S22923022	79158126	0	0	0	G
3	BICF2G630350875	79166004	G	0	0.1429	A
3	BICF2G630350909	79180723	A	0	0.1429	G
3	BICF2G630350926	79189529	G	0.25	0.2143	A
3	TIGRP2P52627_rs8594309	79208003	A	0	0	G
3	BICF2P910940	79213365	A	0.25	0.4167	G
3	BICF2G630350937	79229821	A	0	0	G
3	BICF2G630350951	79237982	G	1	0.8571	A
3	BICF2G630350988	79250592	G	0	0	A
3	BICF2G630351019	79264559	A	0	0	G
3	BICF2G630351037	79279744	G	0	0	A
3	BICF2G630351044	79282793	G	0.75	0.6429	A
3	BICF2G630351048	79283989	G	0.75	0.6429	A
3	BICF2G630351066	79302715	A	0	0	G
3	BICF2P1318782	79305920	G	0.75	0.6429	A
3	BICF2P636424	79306893	G	0.75	0.6429	A
3	BICF2P1209669	79317191	G	0.25	0.3571	A
3	BICF2G630351083	79328247	G	0.25	0.3571	A
3	BICF2G630351085	79347494	A	0	0	T
3	BICF2P738381	79353205	G	0	0	A
3	BICF2G630351108	79366709	G	0	0	A
3	BICF2G630351132	79376145	A	0	0	G
3	BICF2G630351160	79387950	G	0	0	A
3	BICF2G630351179	79397412	A	0.25	0.2143	C
3	BICF2G630351193	79413315	G	0.25	0.2143	A
3	BICF2S23519935	79419697	G	0	0	A

3	BICF2G630351236	79434470	A	0	0	C
3	BICF2G630351247	79445813	G	0	0	A
3	BICF2G630351248	79445904	A	0	0	T
3	BICF2G630351268	79453670	G	0	0	A
3	BICF2G630351281	79471478	G	0	0	A
3	BICF2G630351287	79477511	G	0	0	A
3	BICF2G630351304	79488468	A	0	0	C
3	BICF2G630351314	79500669	A	0	0	T
3	BICF2G630351326	79513219	C	0	0	A
3	BICF2G630351334	79523561	A	0.5	0.3571	G
3	BICF2G630351361	79547129	A	0.5	0.3571	G
3	BICF2P619667	79557135	0	0	0	T
3	BICF2P655413	79570235	A	0	0	G
3	BICF2G630351386	79581043	A	1	0.5833	C
3	BICF2G630351415	79595089	C	0	0	A
3	BICF2G630351438	79611303	A	1	0.8571	G
3	BICF2G630351455	79628868	A	0.75	0.5714	G
3	BICF2G630351466	79643568	A	1	0.9167	C
3	BICF2P1375355	79653276	A	1	0.8571	C
3	BICF2G630351527	79670918	A	0	0	G
3	BICF2G630351530	79672651	C	1	0.8571	A
3	BICF2S22929895	79689102	A	0.25	0.4286	G
3	BICF2S23127335	79702898	G	0.75	0.7857	A
3	BICF2S2311919	79712939	G	0	0.1429	A
3	BICF2P356174	79722257	C	1	1	A
3	BICF2G630351548	79742288	G	0	0	A
3	BICF2G630351560	79753070	G	0	0.1429	A
3	BICF2P336665	79766411	A	0	0.1429	G
3	BICF2S2377445	79785881	A	0	0	G
3	BICF2G630351596	79794503	A	0.75	0.7143	G
3	BICF2G630351615	79806468	G	0	0	C
3	BICF2G630351638	79828801	A	0	0.1429	G
3	BICF2P985366	79838408	G	0.5	0.3571	A
3	BICF2G630351670	79854953	A	0	0	G
3	TIGRP2P52740_rs8736588	79860774	A	0	0	G
3	BICF2G630351682	79867991	G	1	1	A
3	BICF2S23335038	79887712	A	0.5	0.3571	G
3	BICF2S2396612	79897238	G	0	0	A
3	TIGRP2P52745_rs8756117	79910793	0	0	0	A
3	BICF2P914922	79920914	G	0.5	0.3571	A
3	BICF2P819052	79934161	G	0	0	A
3	BICF2P819051	79934358	G	0	0	A
3	BICF2S22963906	79945520	A	0	0	T
3	BICF2P1232976	79954709	G	0.75	0.7143	A
3	BICF2G630351698	79962138	A	0	0	G
3	BICF2G630351712	79964546	C	0.25	0.2143	G
3	BICF2G630351727	79977654	C	0	0	A

3	BICF2G630351734	79984511	G	0.25	0.2143	A
3	BICF2G630351748	79996299	A	0	0	G
3	TIGRP2P52773_rs8951371	80015718	G	0.75	0.6429	A
3	BICF2G630351779	80027023	A	0	0.1429	T
3	BICF2S2367381	80035245	0	0	0	A
3	BICF2S23040483	80047451	A	0	0	G
3	BICF2G630351787	80054839	0	0	0	C
3	TIGRP2P52803_rs9198335	80070562	A	0.25	0.2143	G
3	BICF2P718663	80082492	C	0.25	0.4286	A
3	BICF2P414826	80086095	C	0.25	0.4286	A
3	TIGRP2P52822_rs9003030	80105817	A	0	0	G
3	BICF2G630351801	80114990	G	0.5	0.6429	A
3	BICF2P330097	80128364	0	0	0	A
3	BICF2G630351828	80140623	C	0	0	A
3	BICF2G630351846	80152528	G	0.5	0.3571	A
3	BICF2G630351852	80156101	G	0	0	A
3	BICF2P606587	80164745	A	0	0	G
3	BICF2G630351861	80166712	A	0.5	0.3571	G
3	BICF2G630351882	80178241	A	0.25	0.2857	G
3	BICF2S23060697	80189746	C	0	0	G
3	BICF2S23758115	80203644	A	0	0	G
3	BICF2G630351916	80214528	G	0.25	0.2857	A
3	BICF2G630351931	80228000	A	0	0.2857	G
3	BICF2G630351936	80235939	A	0.25	0.25	G
3	TIGRP2P52854_rs8971349	80248522	C	0	0.1429	A
3	BICF2G630351961	80265404	G	0.25	0.4286	A
3	BICF2G630351969	80270038	0	0	0	G
3	BICF2G630351991	80287340	G	1	0.8571	A
3	BICF2G630352002	80302832	G	0.75	0.6429	A
3	BICF2G630352027	80311390	G	0	0	C
3	BICF2G630352039	80321200	G	0	0	A
3	BICF2G630352059	80329880	G	0	0	A
3	TIGRP2P52881_rs8913452	80343076	C	0	0.1429	A
3	BICF2G630352088	80345225	A	0	0	G
3	BICF2G630352098	80353173	G	0.25	0.3571	A
3	BICF2G630352123	80364443	G	0	0.1429	A
3	BICF2G630352144	80374146	A	0.75	0.7857	G
3	BICF2G630352171	80386420	G	0	0	A
3	BICF2P338286	80396664	G	0	0	A
3	TIGRP2P52906_rs8752263	80418801	A	0	0	G
3	BICF2G630352218	80423419	A	0	0	G
3	BICF2G630352228	80431713	G	0.25	0.2857	A
3	BICF2P286852	80443429	C	0	0	A
3	BICF2G630352257	80464707	A	0.25	0	C
3	BICF2G630352263	80467514	G	0.25	0.2857	A
3	BICF2G630352285	80477329	A	0	0	G
3	TIGRP2P52947_rs8934715	80493595	A	0	0	G

3	BICF2G630352302	80505564	G	0.75	0.6429	A
3	BICF2G630352322	80520903	A	0	0	G
3	BICF2S2444900	80528686	G	0.25	0.3571	A
3	BICF2G630352353	80548984	A	0.75	0.7143	G
3	BICF2G630352358	80563660	G	0.75	0.7143	A
3	BICF2G630352374	80576248	A	0	0	G
3	BICF2G630352375	80576378	G	0.75	0.7143	A
3	BICF2P1027365	80582847	A	0.25	0.3333	G
3	BICF2G630352395	80597432	A	0	0	G
3	BICF2P820596	80604924	A	0	0	G
3	BICF2G630352412	80619443	A	0	0	G
3	BICF2P398015	80626708	G	0.5	0.3571	C
3	BICF2G630352430	80638623	A	0	0	C
3	BICF2P378883	80649840	A	0.5	0.6429	C
3	BICF2G630352476	80661364	C	0.75	0.6429	A
3	BICF2P1130015	80675511	C	0	0	G
3	BICF2P1307987	80684065	A	0.25	0.2857	G
3	BICF2G630352488	80701051	G	0.5	0.6429	A
3	BICF2G630352516	80707135	G	0.25	0.2857	A
3	BICF2G630352537	80718567	A	0.75	0.7143	G
3	BICF2S23019521	80735258	A	0.5	0.3571	C
3	TIGRP2P53013_rs9161504	80746593	0	0	0	A
3	BICF2G630352541	80759499	C	0.5	0.3571	A
3	BICF2G630352562	80768212	G	0	0	A
3	BICF2G630352570	80779965	T	0	0	A
3	BICF2G630352589	80790798	A	0.5	0.6429	C
3	BICF2G630352603	80801257	G	0	0	A
3	BICF2S23646130	80815435	A	0	0	G
3	BICF2G630352623	80826994	A	0.5	0.6667	G
3	BICF2G630352631	80834869	A	1	0.8	C
3	BICF2G630352636	80859355	A	0.5	0.3571	G
3	BICF2G630352655	80868905	A	0.5	0.5	G
3	BICF2G630352670	80880200	A	0	0	G
3	BICF2S23431583	80901087	G	0.75	0.7857	A
3	BICF2S23447659	80904651	G	0.25	0.2857	A
3	BICF2S23647860	80915084	A	0.5	0.5	C
3	BICF2G630352679	80934759	A	0.5	0.5	G
3	BICF2G630352683	80937583	G	0.75	0.7857	A
3	BICF2G630352700	80949037	A	0	0	G
3	BICF2G630352723	80962990	G	0.75	0.7857	A
3	BICF2P596527	80978069	G	1	0.8571	A
3	BICF2G630352736	80989827	A	0	0.1429	G
3	BICF2G630352743	80997714	A	0	0.1429	G
3	BICF2G630352753	81011766	A	0.5	0.5	T
3	BICF2G630352772	81022130	G	0.25	0.2857	A
3	BICF2S2299618	81034529	G	0.75	0.5714	A
3	BICF2G630352830	81043795	G	0.75	0.6429	A

3	BICF2G630352847	81054600	A	0.25	0.3571	G
3	BICF2G630352898	81078376	A	0	0	C
3	BICF2P535731	81088665	G	0.75	0.6429	A
3	TIGRP2P53083_rs8731448	81098611	A	0	0	G
3	BICF2S23331003	81111559	A	0.25	0.2857	G
3	BICF2G630352919	81122789	A	0.5	0.6429	G
3	BICF2P720809	81141706	G	0.75	0.7143	A
3	BICF2G630352940	81144023	G	0.75	0.7143	A
3	BICF2P918968	81158706	A	0.5	0.5	C
3	BICF2P1419798	81168419	G	0	0	A
3	BICF2S23713111	81180675	A	0.25	0.3571	G
3	BICF2G630352964	81197048	G	0.5	0.6667	A
3	BICF2G630352972	81204994	G	0	0	A
3	BICF2G630352992	81217941	A	0	0	G
3	BICF2G630353014	81226612	A	0.5	0.5	C
3	BICF2G630353026	81236278	A	0.5	0.5	G
3	BICF2G630353072	81251679	G	0.5	0.5	A
3	BICF2G630353085	81260069	A	0.5	0.5	C
3	BICF2S23356466	81272685	G	0	0	A
3	BICF2P203547	81283151	A	0.5	0.5	G
3	BICF2P1377301	81295319	G	0	0	A
3	TIGRP2P53115_rs9188435	81307080	0	0	0	G
3	BICF2G630353110	81321706	A	0.5	0.6429	G
3	BICF2G630353115	81332061	A	0.25	0.3571	G
3	BICF2P744392	81349164	G	0	0	A
3	BICF2G630353124	81352688	G	0.5	0.5	A
3	BICF2G630353159	81368575	A	0	0.1429	G
3	BICF2G630353164	81374898	C	0	0	A
3	BICF2G630353188	81392541	C	0.25	0.2857	A
3	BICF2G630353199	81397454	G	0	0.1429	A
3	BICF2P492879	81409974	A	0	0	G
3	BICF2P1396993	81426723	G	0.25	0.3571	A
3	BICF2S23541639	81442657	A	0.25	0.2857	C
3	BICF2G630353235	81446119	A	0	0	C
3	BICF2G630353261	81457673	C	0	0.1429	A
3	BICF2G630353280	81470961	A	0	0.1429	G
3	BICF2G630353298	81481760	A	0	0.1429	G
3	BICF2G630353318	81494304	A	0	0.1429	G
3	BICF2G630353329	81505801	A	0.25	0.25	G
3	BICF2G630353343	81513678	G	0.25	0.3571	A
3	BICF2G630353361	81526188	G	0.25	0.2857	A
3	BICF2G630353380	81547005	G	0.25	0.3571	A
3	BICF2G630353404	81562205	G	0.5	0.5	C
3	TIGRP2P53119_rs8582261	81570129	A	0	0.1429	G
3	BICF2G630353408	81570257	C	0	0.1429	G
3	BICF2P968238	81581529	A	0	0	T
3	BICF2G630353451	81593625	A	0.75	0.6429	G

3	BICF2P1268518	81608905	G	0	0	A
3	BICF2S2305298	81622482	G	0.25	0.2143	A
3	BICF2P323806	81629679	A	0	0.1429	G
3	BICF2P147859	81649558	C	0	0.1429	A
3	BICF2P887071	81656307	C	0.25	0.3571	G
3	BICF2P176337	81670800	A	0	0.1429	G
3	TIGRP2P53164_rs9084471	81677134	A	0.5	0.3571	G
3	BICF2P188465	81684553	A	0.25	0.25	C
3	BICF2P330098	81696737	A	0.5	0.3571	G
3	BICF2S23656996	81718821	A	0.5	0.3571	G
3	BICF2P357872	81719430	A	0.5	0.3571	G
3	TIGRP2P53179_rs8876441	81731529	A	0.25	0.3333	G
3	BICF2S23339371	81747509	G	0.5	0.3571	A
3	BICF2P308830	81759916	G	0.25	0.2857	A
3	BICF2P534725	81766348	A	0	0.2857	T
3	TIGRP2P53204_rs8953266	81782018	G	0.25	0.3571	A
3	BICF2G630353474	81783730	A	0.25	0.25	G
3	BICF2S23338036	81794955	A	0.5	0.3	G
3	BICF2G630353489	81809378	G	1	1	A
3	BICF2P327754	81831663	A	0.5	0.5	C
3	BICF2S23752543	81835300	G	0.5	0.5	A
3	BICF2G630353565	81854536	0	0	0	A
3	BICF2G630353572	81859673	A	0.75	0.7857	G
3	BICF2G630353589	81869511	A	0.75	0.7857	G
3	BICF2P619275	81873170	G	0.25	0.4286	A
3	BICF2G630353629	81883092	A	0.5	0.6429	G
3	BICF2P498905	81894488	G	0.25	0.4286	A
3	BICF2P872459	81912681	G	0	0	A
3	BICF2G630353682	81923682	G	0.25	0.1667	A
3	BICF2G630353692	81936089	C	0.25	0.2143	A
3	TIGRP2P53247_rs9011208	81943014	A	0.25	0.25	G
3	BICF2P384316	81953535	G	0.5	0.6429	A
3	BICF2G630353710	81963285	G	0.5	0.6429	A
3	BICF2G630353736	81983499	T	1	1	A
3	BICF2G630353762	81992067	G	0.75	0.75	A
3	BICF2G630353776	82006134	G	0.5	0.5	A
3	BICF2P908017	82010803	A	0	0	G
3	BICF2G630353795	82020214	A	0.5	0.5	G
3	BICF2S23429490	82034758	A	0	0	C
3	BICF2G630353804	82041467	0	0	0	G
3	BICF2G630353823	82059075	A	0.75	0.7857	G
3	BICF2G630353829	82066003	A	0.5	0.3571	G
3	BICF2G630353842	82075688	A	0.5	0.5	G
3	BICF2P125810	82094802	A	0	0.4167	G
3	BICF2P1332991	82117525	C	0	0	A
3	BICF2P683381	82122204	G	0	0.1429	A
3	BICF2P1097877	82133864	G	0	0	A

3	BICF2P885972	82143098	A	0	0	G
3	BICF2P1342564	82148878	G	0.5	0.5	A
3	BICF2P980426	82164001	0	0	0	G
3	TIGRP2P53292_rs8747144	82171660	0	0	0	T
3	BICF2P774372	82187294	G	0	0.1429	A
3	BICF2P315087	82192325	G	1	1	A
3	BICF2P450388	82204122	G	1	1	A
3	BICF2P349336	82214620	A	0.75	0.7143	G
3	BICF2P274497	82225893	A	1	0.75	G
3	BICF2P1144541	82240338	G	0.25	0.2143	A
3	TIGRP2P53312_rs8723956	82249450	A	0	0	G
3	BICF2P1116169	82261144	A	0	0	G
3	BICF2P518314	82275318	A	0	0	G
3	BICF2S23645482	82294464	G	1	0.8571	A
3	BICF2P1105366	82314805	A	0.5	0.5	G
3	BICF2S23719887	82317800	A	0.5	0.3571	G
3	BICF2P1156194	82333403	G	0.5	0.3571	A
3	BICF2P1125677	82342011	G	0.5	0.3571	A
3	BICF2P1011005	82362238	A	0.5	0.6429	C
3	TIGRP2P53355_rs8579202	82368181	A	0.5	0.6429	G
3	TIGRP2P53357_rs9187653	82374867	A	0.25	0.2143	G
3	BICF2S23210287	82388755	G	0.5	0.6429	C
3	BICF2P211876	82392735	A	0.5	0.6429	G
3	BICF2P351423	82407649	A	0.5	0.5	G
3	BICF2S23720670	82415331	A	0	0.4167	C
3	BICF2S23543305	82441139	A	0.75	0.6429	C
3	BICF2S22954619	82466086	G	0.25	0.2143	A
3	TIGRP2P53391_rs8707920	82476362	G	0	0.1429	A
3	TIGRP2P53394_rs9146936	82478172	A	0.25	0.3571	G
3	TIGRP2P53410_rs9155465	82497821	G	0	0.1429	A
3	BICF2P807595	82508242	C	0	0.1429	A
3	BICF2P525932	82520023	A	1	1	T
3	BICF2S23726110	82527438	T	0.5	0.5	A
3	BICF2S23737781	82541720	C	0	0	A
3	BICF2P347939	82551354	G	0.5	0.5	A
3	BICF2P1271500	82562206	G	0.5	0.6429	A
3	BICF2P485843	82573784	G	0.5	0.5	A
3	BICF2P998082	82583186	G	0	0	A
3	BICF2P1096140	82597380	G	1	0.8571	A
3	TIGRP2P53476_rs8736981	82606973	0	0	0	C
3	TIGRP2P53477_rs8992924	82627358	A	1	0.8571	G
3	BICF2P828798	82630385	G	0.75	0.6429	A
3	BICF2P1320078	82650187	G	0.75	0.6429	C
3	TIGRP2P53487_rs8847032	82654564	G	0	0	A
3	TIGRP2P53514_rs8977688	82669887	G	1	1	A
3	BICF2S23127058	82684665	A	0	0.1429	G
3	BICF2S23351425	82693577	A	0	0.1429	G

3	BICF2P905769	82704209	0	0	0	G
3	BICF2P25756	82708959	C	1	1	A
3	BICF2P573494	82726483	0	0	0	A
3	BICF2S23522632	82733187	A	0.25	0.4286	G
3	BICF2P290493	82744999	C	0	0	A
3	BICF2P1233261	82761195	T	1	1	A
3	BICF2P397912	82766251	A	0.5	0.3571	G
3	BICF2P397907	82767564	G	0.25	0.2143	A
3	TIGRP2P53545_rs8910179	82783785	A	0.25	0.2143	G
3	BICF2P109049	82790410	A	0.5	0.3571	C
3	BICF2P516591	82804094	G	0	0	C
3	BICF2P525577	82812087	C	0	0	A
3	BICF2S23222335	82843821	A	0.25	0.2857	G
3	BICF2P1021741	82847568	A	1	1	G
3	BICF2P337683	82865569	A	0.75	0.5714	G
3	BICF2S23342553	82869195	A	1	1	G
3	BICF2P1249342	82870031	G	0	0.1429	A
3	TIGRP2P53579_rs8974870	82883006	A	0.5	0.5	C
3	BICF2P894936	82894917	G	0.25	0.4286	A
3	BICF2P858838	82904198	A	0	0	G
3	BICF2S23320911	82917515	A	0.25	0.3571	G
3	BICF2P461845	82928581	A	0	0	G
3	BICF2P263082	82940459	A	0.25	0.3571	C
3	BICF2P942586	82964671	A	0.5	0.3571	C
3	BICF2P1049730	82975253	0	0	0	A
3	BICF2P99511	82993804	A	0.25	0.2857	C
3	TIGRP2P53619_rs9234379	83006482	A	0.75	0.6429	G
3	BICF2P921462	83012438	C	0.75	0.6429	A
3	TIGRP2P53620_rs8828953	83018546	G	0.75	0.6429	A
3	BICF2P1298241	83030135	A	1	0.8571	C
3	BICF2S23024737	83044375	G	1	0.8571	A
3	TIGRP2P53639_rs9161436	83058149	C	0	0	A
3	TIGRP2P53643_rs8983141	83068152	C	0	0	A
3	BICF2S23319317	83080708	A	0.75	0.7143	G
3	TIGRP2P53660_rs8817748	83088508	A	0.25	0.3571	C
3	TIGRP2P53672_rs8805193	83099450	0	0	0	A
3	BICF2S23038061	83111606	C	0.25	0.2857	A
3	BICF2P1189195	83123639	A	0.5	0.3571	G
3	BICF2P207217	83133691	C	0.25	0.2857	G
3	BICF2P658659	83149347	G	0.25	0.2857	A
3	TIGRP2P53689_rs8704547	83158462	0	0	0	G
3	BICF2P665409	83168613	A	0	0.1429	G
3	BICF2P1347927	83191741	G	0.75	0.5714	A
3	TIGRP2P53709_rs8603572	83207487	G	0.75	0.6429	A
3	BICF2P1313603	83221323	A	0	0.1429	G
3	BICF2P289699	83226640	A	0.25	0.4286	G
3	BICF2S23724823	83247930	A	0	0	G

3	BICF2P376613	83249341	G	0.25	0.2857	A
3	BICF2P327951	83250031	A	0	0.1429	G
3	BICF2P418337	83263121	G	0.5	0.5	A
3	TIGRP2P53724_rs9069573	83272139	A	0.25	0.2143	T
3	BICF2P1206866	83288530	0	0	0	A
3	BICF2P106451	83299111	G	0.75	0.6429	A
3	BICF2P520660	83312546	A	0	0.1429	G
3	BICF2P490997	83325788	A	0.25	0.4286	G
3	BICF2P783430	83331530	G	0.75	0.5714	C
3	BICF2S23317675	83345121	A	0.75	0.5714	G
3	BICF2P1340843	83361269	G	0.25	0.2143	A
3	TIGRP2P53768_rs9238519	83366529	G	0.25	0.2857	A
3	BICF2P821092	83376818	A	0.25	0.2857	G
3	BICF2S23544955	83390731	G	1	0.8571	A
3	BICF2P1329311	83410135	A	0	0	G
3	TIGRP2P53783_rs9118797	83431877	A	0.5	0.3571	G
3	TIGRP2P53788_rs9081929	83441858	A	0	0	G
3	TIGRP2P53789_rs8883486	83444407	C	1	0.8571	A
3	BICF2P1281888	83456602	A	0	0	G
3	BICF2P332154	83470734	T	0	0	A
3	BICF2P863731	83484302	G	1	0.8571	A
3	BICF2P1209608	83499102	G	1	0.8571	A
3	BICF2P573477	83520091	A	1	0.8571	T
3	BICF2S2363742	83526124	0	0	0	G
3	BICF2P53494	83562496	0	0	0	A
3	TIGRP2P53829_rs8846258	83574104	A	0.5	0.3571	G
3	TIGRP2P53839_rs8678033	83592405	G	0	0	A
3	BICF2P156090	83604309	G	0.5	0.3571	A
3	BICF2P406943	83606491	G	0.5	0.3571	A
3	TIGRP2P53851_rs8800016	83625440	C	0.5	0.3571	G
3	TIGRP2P53857_rs8497336	83646911	A	0.5	0.3571	G
3	BICF2P494821	83654337	A	0.5	0.3571	G
3	BICF2P93316	83669119	G	0	0	A
3	TIGRP2P53881_rs9104135	83676657	A	0.25	0.2143	G
3	BICF2P628275	83685995	C	1	0.8571	G
3	BICF2P589754	83706518	G	0.75	0.6429	A
3	TIGRP2P53889_rs8904484	83715369	0	0	0	A
3	BICF2P280384	83726659	0	0	0	G
3	TIGRP2P53912_rs9075478	83733096	A	0.25	0.2143	C
3	TIGRP2P53926_rs8521009	83745309	A	1	0.8571	T
3	BICF2P293673	83756106	0	0	0	A
3	G261f38S125	83775932	A	1	0.8571	T
3	TIGRP2P53931_rs8741851	83783206	0	0	0	G
3	BICF2P987955	83798854	G	0	0.2143	A
3	BICF2S23549123	83801163	G	0	0	A
3	BICF2P1158180	83819394	A	0.25	0.2143	G
3	BICF2S2351140	83828618	A	0	0	G

3	BICF2P974752	83843926	A	0.25	0.1	C
3	BICF2P1327554	83858045	A	0.25	0.2143	G
3	BICF2P1319407	83880428	A	0.25	0.2143	G
3	BICF2P213131	83884204	C	0.25	0.2143	A
3	BICF2P801483	83898298	G	0	0	A
3	BICF2P726412	83902791	G	0	0	A
3	BICF2P1070803	83920177	A	0	0	G
3	BICF2S2366714	83955551	G	0	0	A
3	BICF2P972151	83962101	A	0	0	G
3	BICF2P667331	83978862	C	0	0	A
3	BICF2P1296708	83987349	0	0	0	A
3	TIGRP2P53943_rs8908278	83997919	C	0	0	A
3	BICF2P1147704	84011701	G	0.75	0.6429	A
3	BICF2P1050373	84024987	G	0.75	0.6429	A
3	BICF2S23620236	84034450	G	0	0	A
3	BICF2S23516729	84044485	A	0	0	G
3	BICF2P137158	84063248	0	0	0	A
3	BICF2P157556	84069547	C	0	0	A
3	BICF2P104190	84078976	A	0.75	0.6667	G
3	BICF2P167169	84089480	A	0	0	G
3	BICF2P375093	84104124	C	0	0	G
3	BICF2P294431	84121710	A	0	0	G
3	TIGRP2P53960_rs8998545	84133182	G	0.75	0.6429	A
3	BICF2P1314918	84139326	A	0.75	0.6429	G
3	BICF2P1245917	84162993	0	0	0	A
3	BICF2P1270358	84187273	A	0.75	0.6429	G
3	BICF2P966653	84195436	A	0	0	G
3	BICF2S2326180	84224129	A	0	0	G
3	BICF2S23538758	84252628	G	0	0	A
3	BICF2P761094	84265414	0	0	0	C
3	BICF2P323251	84284506	0	0	0	A
3	BICF2P25233	84297068	0	0	0	G
3	BICF2P592907	84328797	0	0	0	A
3	BICF2S2339009	84344867	A	0	0	G
3	BICF2S23621071	84357731	G	1	1	A
3	BICF2P590503	84366842	C	0	0	A
3	BICF2P824891	84378086	A	0	0	G
3	BICF2S23645832	84392498	A	0	0	G
3	BICF2P246579	84401380	C	1	1	A
3	BICF2P534349	84411029	A	1	1	C
3	BICF2P1205774	84423314	C	1	1	A
3	BICF2P406679	84433666	A	1	1	G
3	BICF2P531383	84447755	G	0	0	A
3	BICF2P532792	84457801	A	1	1	G
3	BICF2P1190149	84471700	G	0	0	A
3	BICF2P690691	84490100	G	0.25	0.3571	A
3	BICF2S23155841	84524508	A	0.25	0.3571	G

3	BICF2S23218996	84530710	A	0	0	G
3	BICF2P884615	84545969	G	0	0	A
3	BICF2P29565	84552727	G	0	0	A
3	BICF2S2342348	84569446	A	0	0	G
3	BICF2S22917428	84571107	A	0	0	C
3	BICF2P1091176	84587016	A	0	0	C
3	BICF2S23535199	84595052	A	0	0	G
3	BICF2P1339623	84612353	A	0	0	G
3	BICF2P1088116	84622084	A	0	0	G
3	BICF2S23429361	84635942	G	0	0	A
3	BICF2P666599	84648887	A	0	0	G
3	BICF2S2342085	84661795	C	0	0	A
3	TIGRP2P54019_rs8610151	84665259	C	0.25	0.3571	A
3	TIGRP2P54021_rs9106848	84681767	A	0	0	G
3	BICF2S23733490	84699707	G	0.25	0.3571	A
3	BICF2S23327029	84712846	G	0.75	0.6429	A
3	BICF2P704014	84739032	A	0.25	0.2143	G
3	BICF2P1406832	84754945	G	1	1	A
3	BICF2P1406826	84755286	G	1	1	A
3	BICF2P380031	84777759	G	0.25	0.3571	A
3	BICF2P1213717	84790422	G	0.75	0.6429	A
3	TIGRP2P54057_rs9051236	84805686	A	1	0.8571	G
3	BICF2P268833	84830491	G	1	1	A
3	BICF2P1362134	84836043	G	0	0.1429	A
3	BICF2P791308	84849711	G	0	0	A
3	BICF2P1076740	84860287	0	0	0	G
3	BICF2P131107	84871295	A	0	0	G
3	TIGRP2P54086_rs9040865	84885587	0	0	0	T
3	BICF2P785166	84917918	0	0	0	A
3	BICF2S23746026	84934559	A	0.75	0.7857	G
3	BICF2S23316367	84944693	A	0.75	0.7857	C
3	BICF2P644431	84950240	A	0	0	G
3	BICF2S23335587	84959306	A	0.25	0.2143	C
3	BICF2S23330853	84962549	G	0.25	0.2143	A
3	BICF2P1447101	84976213	A	0.25	0.3571	G
3	TIGRP2P54095_rs9036325	84980180	G	0.25	0.3571	A
3	TIGRP2P54102_rs8649896	85000432	A	0	0	G
3	TIGRP2P54115_rs9123739	85008634	A	0.75	0.7857	G
3	TIGRP2P54233_rs8779655	85028067	C	0.75	0.7857	G
3	BICF2P461602	85032660	A	0.75	0.7857	G
3	BICF2S23120607	85041746	G	0.75	0.7857	A
3	BICF2S23340573	85049214	A	0	0	G
3	BICF2P385903	85055394	A	0.75	0.6429	G
3	BICF2P1254163	85067723	0	0	0	C
3	BICF2S24117207	85086506	G	0	0	A
3	BICF2P423011	85098144	A	0	0	C
3	BICF2P805182	85117617	0	0	0	A

3	BICF2P721731	85132407	0	0	0	A
3	BICF2P40213	85144716	G	0.75	0.6429	A
3	TIGRP2P54246_rs8657228	85164426	0	0	0	G
3	BICF2S23737307	85169998	A	0	0	G
3	BICF2P1379990	85185018	C	1	1	G
3	BICF2S23639513	85195031	A	1	1	G
3	BICF2P1230210	85214288	0	0	0	A
3	BICF2P1172537	85217406	A	1	1	G
3	TIGRP2P54278_rs8814964	85232374	G	1	1	C
3	BICF2P604465	85249224	A	0	0	C
3	TIGRP2P54285_rs8993335	85250215	A	1	0.8571	G
3	BICF2P233704	85271705	A	0.75	0.6429	G
3	BICF2P1283946	85275343	G	0	0	A
3	BICF2P1100147	85293152	A	0	0.1429	G
3	TIGRP2P54297_rs8830346	85304339	A	0.25	0.2143	G
3	BICF2P459737	85312835	G	0.25	0.2143	C
3	BICF2S22934159	85325315	C	0.25	0.2143	G
3	BICF2P872873	85333250	A	0.25	0.2143	G
3	TIGRP2P54318_rs8993068	85348386	A	0	0	G
3	BICF2P305308	85354181	C	0.75	0.6429	A
3	BICF2S23429590	85371914	0	0	0	G
3	BICF2P602949	85376840	0	0	0	G
3	BICF2P340336	85396030	0	0	0	A
3	BICF2P341502	85408186	0	0	0	A
3	TIGRP2P54320_rs8750063	85419225	A	0.25	0.3571	G
3	TIGRP2P54326_rs9119631	85425658	0	0	0	G
3	BICF2P680033	85439712	A	0.25	0.3571	G
3	TIGRP2P54329_rs9153128	85445971	G	0.25	0.3571	A
3	BICF2P485381	85473616	A	0.25	0.3571	G
3	TIGRP2P54362_rs9232807	85487544	A	0	0	G
3	TIGRP2P54368_rs9023687	85491248	A	0	0	G
3	BICF2P73594	85508096	G	0.75	0.6429	A
3	BICF2P825508	85517063	A	0.75	0.6429	G
3	BICF2P1184786	85533280	0	0	0	G
3	TIGRP2P54391_rs9008388	85557597	A	0	0	G
3	BICF2S2374373	85577997	A	1	1	G
3	BICF2S2374372	85578269	C	1	1	A
3	BICF2S2336388	85589719	0	0	0	T
3	BICF2S23231050	85594990	A	0	0	C
3	BICF2S229186	85608697	G	0	0.1429	A
3	TIGRP2P54395_rs8895672	85640400	C	0	0.1429	A
3	BICF2P1212167	85647926	C	0	0.1429	A
3	BICF2S23541438	85656214	A	0	0	G
3	TIGRP2P54421_rs8803573	85668105	G	0	0.1429	A
3	TIGRP2P54428_rs8680040	85684974	A	0	0.1429	G
3	BICF2P512037	85689826	G	1	1	A
3	BICF2S23421003	85702073	G	1	1	A

3	BICF2P978779	85715602	A	1	1	G
3	BICF2P659903	85732393	A	0	0	G
3	TIGRP2P54443_rs8932828	85736134	A	1	1	G
3	BICF2P1501195	85747824	G	1	1	A
3	TIGRP2P54447_rs8784158	85756854	G	1	1	A
3	BICF2S23326188	85771925	A	0	0.1429	G
3	BICF2P98099	85788553	A	1	1	G
3	BICF2P98100	85788686	A	1	0.8571	C
3	BICF2P186550	85790043	A	1	1	G
3	BICF2P357365	85804819	C	0	0	G
3	BICF2P104002	85814856	A	0	0.1429	G
3	BICF2P445309	85824704	G	0	0.1429	A
3	TIGRP2P54459_rs8554281	85864893	G	0.25	0.3571	A
3	BICF2S2353371	85874493	A	0.25	0.3571	G
3	TIGRP2P54471_rs8694649	85887162	A	0.25	0.3571	G
3	TIGRP2P54476_rs9139863	85898534	G	0.25	0.3571	A
3	TIGRP2P54491_rs8967010	85910193	C	0.25	0.3333	A
3	BICF2P658181	85910634	G	0.25	0.3571	A
3	BICF2P1402829	85925485	G	0.25	0.3571	A
3	BICF2P1363900	85932776	T	0.25	0.3571	A
3	BICF2S23115040	85939679	G	0.25	0.2143	A
3	BICF2P945593	85951618	G	0.25	0.2143	A
3	BICF2P996639	85963233	G	1	1	A
3	BICF2S22937367	85983036	A	0.75	0.6429	G
3	BICF2P284759	85987286	0	0	0	A
3	BICF2P1504745	86001737	A	0	0	G
3	BICF2P240147	86012264	0	0	0	A
3	BICF2S23013072	86035993	G	NA	0.5	A
3	BICF2P118538	86052252	G	0.25	0.3571	A
3	BICF2P1248122	86072424	A	0	0	G
3	BICF2S23255121	86088077	A	0.75	0.6429	G
3	BICF2S23254797	86091708	G	0.75	0.6667	A
3	G263f41S60	86112699	G	0	0.1429	A
3	BICF2S23050492	86140007	A	0	0	G
3	BICF2P595766	86148223	G	1	0.8571	A
3	BICF2P1220445	86160759	A	0.25	0.2143	G
3	BICF2P1258895	86169673	G	0.25	0.2143	A
3	BICF2S23236388	86182534	G	0	0	A
3	BICF2S23648437	86198841	G	0	0	A
3	BICF2S23734615	86212021	G	1	1	A
3	BICF2P479234	86217478	G	1	1	A
3	BICF2P143331	86229784	A	0	0	G
3	TIGRP2P54533_rs8609660	86241578	C	0	0	A
3	BICF2P988491	86251480	A	0	0	C
3	TIGRP2P54553_rs9019590	86270985	C	0	0	A
3	TIGRP2P54568_rs8872042	86278497	C	0	0	A
3	BICF2P678427	86284939	A	0	0	G

3	TIGRP2P54579_rs8631111	86300409	G	0	0	A
3	BICF2S23725924	86311306	G	0	0	A
3	BICF2S2335282	86322701	G	0	0	A
3	BICF2P171964	86331041	0	0	0	G
3	BICF2P972570	86347221	C	0.25	0.2143	A
3	BICF2S22915988	86349925	G	0	0	A
3	BICF2P997826	86354628	A	0.25	0.3571	G
3	BICF2S23646854	86372185	T	0.25	0.3571	A
3	BICF2S2371005	86378924	G	0	0.1429	A
3	TIGRP2P54622_rs8529807	86397551	C	0.75	0.7857	A
3	BICF2P872327	86403839	G	0.75	0.7857	A
3	BICF2P845255	86412804	G	0.75	0.7857	A
3	BICF2P477119	86432265	G	0	0	A
3	BICF2P692472	86437117	G	0.75	0.7857	A
3	BICF2P1046949	86445497	0	0	0	A
3	BICF2P1046953	86446985	C	0.75	0.7857	A
3	BICF2P1269599	86468147	G	0.75	0.7857	A
3	TIGRP2P54641_rs8837605	86470336	A	0.75	0.7857	G
3	BICF2P866743	86481742	A	0.75	0.7857	G
3	BICF2P677825	86494938	A	0.75	0.7857	G
3	BICF2P1322449	86525684	G	0.75	0.7857	A
3	BICF2P495288	86531507	A	0.75	0.7857	G
3	BICF2P1157267	86546302	A	0	0.08333	C
3	TIGRP2P54664_rs9035952	86550672	A	0.25	0.2143	G
3	BICF2P161091	86562584	A	0	0.1429	G
3	TIGRP2P54686_rs9006094	86575198	A	0	0	G
3	BICF2P55526	86591178	G	0	0	A
3	BICF2S2295729	86596620	A	0.75	0.7857	G
3	BICF2S23136096	86612659	G	0	0.1429	A
3	BICF2S23125423	86619301	G	0	0.1429	A
3	BICF2P1430054	86639713	G	0.75	0.7857	A
3	BICF2P453459	86644273	A	0	0	G
3	TIGRP2P54734_rs9099263	86663806	G	0	0	A
3	BICF2P264030	86665477	C	0.75	0.7857	A
3	TIGRP2P54743_rs8694610	86676611	A	0	0.1429	T
3	TIGRP2P54751_rs8715177	86689744	G	0.75	0.7857	A
3	TIGRP2P54760_rs9235980	86704402	G	0	0	A
3	BICF2P869845	86732079	G	0.75	0.6429	A
3	BICF2P152485	86733643	G	0.75	0.6429	A
3	BICF2P850481	86745035	0	0	0	A
3	BICF2P471295	86763411	A	0.25	0.3571	G
3	TIGRP2P54788_rs8976376	86782192	G	0.75	0.7857	A
3	BICF2S23750064	86801163	G	0.25	0.3571	A
3	BICF2S23753732	86803219	A	0	0	G
3	BICF2P1343705	86814586	C	0	0	A
3	BICF2G630353868	86823281	A	0	0	C
3	BICF2G630353890	86835275	C	0.75	0.7857	G

3	BICF2G630353893	86836550	A	0.75	0.7857	G
3	BICF2P688357	86849529	A	1	1	C
3	BICF2G630353909	86858401	A	0	0	G
3	BICF2G630353913	86861508	A	1	1	G
3	BICF2G630353933	86875054	C	0	0	A
3	BICF2S22926677	86887809	A	0.25	0.2143	C
3	BICF2G630353937	86897716	A	0.25	0.3571	G
3	BICF2G630353938	86915512	G	0.25	0.3571	A
3	TIGRP2P54812_rs8614417	86928489	0	0	0	A
3	BICF2S2342150	86948527	A	0.25	0.2143	G
3	BICF2S23624701	86961225	G	0.25	0.2143	A
3	BICF2S24415473	86974042	G	0.25	0.2143	A
3	BICF2P1171201	86989506	G	0.25	0.2143	A
3	BICF2G630353950	86998520	A	0	0	G
3	BICF2G630353960	87009772	A	0	0	G
3	BICF2P714471	87024213	A	0.25	0.2143	G
3	BICF2G630353969	87040724	G	0.75	0.6429	A
3	BICF2G630353970	87043914	G	0	0	A
3	BICF2P357975	87055519	A	0.25	0.2143	G
3	BICF2G630353978	87069600	A	0	0	G
3	TIGRP2P54845_rs8745137	87080529	G	0.75	0.7857	A
3	BICF2G630354004	87098004	G	0.75	0.6429	A
3	BICF2G630354028	87110416	A	0.25	0.3571	G
3	BICF2G630354054	87122431	A	0.75	0.6429	C
3	BICF2S23044057	87127127	A	0	0	G
3	BICF2G630354070	87137693	A	0.75	0.6429	C
3	BICF2G630354090	87148877	A	0.75	0.7857	G
3	BICF2G630354100	87158880	G	0.75	0.6429	A
3	BICF2G630354113	87175709	G	1	0.8571	A
3	BICF2G630354117	87180410	G	1	0.8571	A
3	BICF2G630354118	87181098	G	1	0.8571	A
3	BICF2G630354121	87183671	G	1	1	A
3	BICF2P25746	87193941	A	1	1	G
3	BICF2G630354161	87208542	G	0	0.1429	A
3	BICF2P233426	87227061	A	0	0.1429	G
3	BICF2G630354176	87238147	A	1	0.9167	G
3	BICF2G630354178	87238784	A	1	0.8571	G
3	BICF2G630354185	87240247	A	0	0	G
3	BICF2G630354211	87260088	A	1	0.8571	G
3	BICF2S2372445	87264628	A	1	1	G
3	BICF2S23322019	87281824	G	0	0	A
3	BICF2G630354227	87287907	A	0	0	G
3	BICF2G630354247	87293739	G	0	0	A
3	BICF2P540899	87298569	A	0.25	0.3571	G
3	BICF2S23331953	87314488	0	0	0	A
3	BICF2G630354291	87328476	G	0.75	0.6429	A
3	BICF2G630354295	87335463	A	0	0	G

3	BICF2G630354311	87345610	A	0	0	G
3	BICF2S23428498	87363835	A	0.25	0.2857	C
3	BICF2G630354353	87384657	A	0	0	G
3	BICF2P86187	87389771	0	0	0	G
3	BICF2S23524859	87409491	G	0	0	A
3	BICF2P1218152	87419347	G	0.75	0.7143	A
3	BICF2G630354380	87430807	C	0.75	0.7143	A
3	BICF2G630354395	87435544	G	0.75	0.7143	A
3	BICF2G630354399	87448468	A	0.75	0.7143	G
3	BICF2S23159175	87456447	G	0	0	A
3	BICF2G630354407	87464393	T	0	0	A
3	BICF2P83915	87478706	C	0	0	A
3	BICF2P83911	87480167	A	0	0	G
3	BICF2S2314217	87491770	T	0	0	A
3	BICF2P527602	87498668	0	0	0	A
3	TIGRP2P54976_rs8765673	87513623	T	0	0	A
3	BICF2G630354410	87517616	A	0	0	C
3	BICF2P1342955	87528392	C	0	0	A
3	BICF2P786531	87540447	A	0	0	G
3	BICF2P1410883	87556948	A	0.5	0.3571	G
3	BICF2P639445	87565740	A	0.5	0.5	G
3	BICF2P529273	87572866	G	0.25	0.2857	A
3	BICF2P349	87587606	A	0.25	0.3571	C
3	BICF2P718095	87597329	C	0	0.1429	A
3	BICF2P1439630	87612795	G	0.25	0.2143	A
3	TIGRP2P55093_rs8873590	87619090	A	0	0	G
3	TIGRP2P55102_rs8659844	87646010	G	0.5	0.5	A
3	TIGRP2P55107_rs8536869	87662664	G	0	0	A
3	BICF2P1383213	87664894	A	0	0	G
3	BICF2P296559	87677563	A	0.5	0.6429	C
3	BICF2P467821	87687756	A	0	0	G
3	TIGRP2P55124_rs8698359	87699246	A	0	0.1429	C
3	BICF2S23115974	87718312	A	0	0	G
3	BICF2P965687	87724489	A	0	0.1429	G
3	TIGRP2P55135_rs9182432	87740825	G	0.5	0.5	A
3	TIGRP2P55142_rs8633645	87750605	G	0	0.1429	A
3	TIGRP2P55145_rs9069918	87763014	G	0.5	0.5	A
3	TIGRP2P55155_rs9168928	87790773	A	0.5	0.3571	C
3	BICF2G630354481	87802430	C	0.5	0.3571	A
3	BICF2G630354486	87804060	G	0	0	A
3	TIGRP2P55162_rs9014022	87817240	0	0	0	A
3	BICF2S23716752	87830585	A	0	0.1429	C
3	BICF2G630354516	87838490	A	1	0.8571	G
3	BICF2P785990	87849460	0	0	0	C
3	BICF2G630354549	87861393	A	0	0	G
3	BICF2G630354573	87872088	G	0	0.1429	A
3	BICF2G630354615	87890888	A	1	1	G

3	BICF2G630354623	87894691	0	0	0	G
3	BICF2G630354651	87913266	G	0	0	A
3	BICF2G630354660	87917531	A	0.5	0.6429	G
3	BICF2G630354679	87929093	A	0.25	0.3571	G
3	BICF2G630354725	87948518	G	0.25	0.3571	A
3	BICF2P754440	87961120	A	0.5	0.3571	G
3	BICF2G630354733	87967409	A	0.25	0.2143	G
3	TIGRP2P55183_rs8574209	87976703	A	0	0	G
3	BICF2G630354781	87987914	A	0	0	G
3	BICF2P1008034	88007430	G	0	0	C
3	BICF2P1116832	88010533	G	0.5	0.3571	A
3	BICF2P244762	88039909	A	0.25	0.2143	G
3	BICF2P504292	88051536	G	0.25	0.2857	A
3	BICF2P733834	88055521	A	0.75	0.7857	C
3	BICF2G630354843	88071328	A	0.25	0.2143	G
3	BICF2G630354859	88080183	A	0	0	C
3	BICF2S23536469	88096201	A	0.25	0.2143	G
3	TIGRP2P55255_rs8563210	88103432	A	0	0	G
3	BICF2S23336675	88103633	G	0.75	0.6429	A
3	BICF2S2342753	88109868	A	0.75	0.6429	G
3	BICF2G630354922	88116331	A	0.75	0.5714	C
3	BICF2G630354937	88130429	G	0.25	0.2143	A
3	BICF2G630354944	88137822	A	0	0	G
3	TIGRP2P55281_rs8884009	88146237	G	0.75	0.5714	A
3	BICF2G630354947	88156919	G	0.25	0.4286	A
3	BICF2G630354955	88159126	G	0.25	0.4286	A
3	BICF2G630354969	88172792	A	0.25	0.4286	G
3	BICF2P548970	88210401	A	0	0	G
3	BICF2G630355069	88222437	G	0.25	0.4286	A
3	BICF2P1453147	88230573	A	0	0	T
3	BICF2G630355103	88240942	A	0.25	0.4286	G
3	BICF2G630355149	88256788	A	0	0	G
3	BICF2G630355158	88264568	C	0	0	A
3	BICF2G630355176	88275058	G	0.25	0.4286	A
3	BICF2P759838	88291044	A	0.25	0.4286	T
3	BICF2G630355187	88302348	G	0.25	0.2857	A
3	BICF2P160533	88318463	G	0.75	0.7857	A
3	BICF2G630355218	88329354	G	0	0	A
3	BICF2G630355223	88331575	A	0	0	G
3	BICF2P1385640	88334903	A	0	0	G
3	BICF2P846449	88350962	G	0.25	0.2857	A
3	BICF2P846438	88354629	A	0.25	0.2857	G
3	BICF2S2415484	88407530	A	0.25	0.2857	G
3	BICF2P1269894	88421670	0	0	0	A
3	BICF2S23723485	88430732	G	0.25	0.2857	A
3	BICF2S23135712	88440500	A	0	0	G
3	BICF2G630355258	88467411	A	0	0	C

3	BICF2G630355260	88470055	A	0.25	0.2857	C
3	BICF2P527426	88492158	0	0	0	G
3	BICF2S23048528	88494320	A	0	0	G
3	BICF2S2297778	88506450	A	0	0	G
3	BICF2P321713	88512406	0	0	0	A
3	BICF2G630355275	88524973	G	0	0	A
3	BICF2S23416517	88533180	A	0.75	0.7143	G
3	BICF2G630355286	88543508	A	0	0	G
3	BICF2G630355296	88553757	A	0	0	G
3	BICF2G630355317	88562463	A	0	0	G
3	BICF2G630355367	88578968	A	0	0	G
3	BICF2G630355378	88585595	G	0.25	0.2857	A
3	TIGRP2P55389_rs8902261	88600745	A	0.25	0.2857	G
3	BICF2G630355422	88607867	A	0.25	0.2857	G
3	BICF2G630355434	88621090	G	0.25	0.2857	A
3	BICF2G630355441	88631803	A	0	0	G
3	BICF2G630355445	88636523	G	0.25	0.2857	A
3	BICF2P1316274	88643658	G	0.25	0.2857	A
3	BICF2G630355451	88657763	A	0.25	0.2857	T
3	BICF2P298966	88661363	A	0	0	G
3	BICF2P1188903	88684315	G	0.25	0.2857	A
3	BICF2G630355467	88688943	A	0.75	0.7143	G
3	BICF2P1400938	88709676	A	0	0	G
3	BICF2G630355477	88712844	A	0	0	C
3	BICF2P326103	88722536	0	0	0	G
3	BICF2G630355506	88730294	A	0.75	0.7143	G
3	BICF2G630355520	88737074	A	1	1	G
3	BICF2G630355529	88746254	G	0.75	0.7143	A
3	BICF2G630355542	88759196	G	0.25	0.2857	A
3	BICF2G630355559	88776538	A	0	0	G
3	BICF2G630355567	88782868	C	0	0	A
3	BICF2G630355588	88800079	A	0.5	0.5	G
3	TIGRP2P55427_rs8618538	88808988	A	0	0	G
3	BICF2G630355615	88819842	A	0.5	0.5	G
3	BICF2G630355625	88826870	A	0.5	0.5	G
3	BICF2P394014	88843548	G	0.25	0.2857	A
3	BICF2G630355646	88855677	G	0.25	0.2143	A
3	BICF2G630355670	88864725	A	0.25	0.2857	T
3	BICF2G630355701	88879079	A	0	0	G
3	TIGRP2P55431_rs9116380	88893816	A	0	0	G
3	BICF2G630355714	88897174	G	0.75	0.7857	A
3	TIGRP2P55432_rs8807909	88926600	G	0	0	A
3	BICF2G630355732	88939019	A	0	0	C
3	BICF2G630355738	88941999	G	0.25	0.2857	A
3	BICF2G630355758	88953365	G	0	0	A
3	BICF2P853105	88965070	A	0.5	0.5	G
3	BICF2S24318709	88985716	G	0.5	0.5	A

3	BICF2G630355828	88989230	G	0.25	0.2857	A
3	BICF2G630355851	89004408	A	0.25	0.2857	G
3	BICF2P1404126	89013551	A	0.75	0.7857	G
3	BICF2P1326614	89023174	0	0	0	T
3	BICF2P778113	89031642	A	0.25	0.2857	G
3	BICF2G630355875	89040117	G	0	0	A
3	BICF2G630355876	89040160	G	0.25	0.2143	A
3	BICF2G630355893	89050253	G	0.25	0.2143	A
3	BICF2G630355909	89056035	A	0.25	0.2143	C
3	BICF2G630355937	89070950	A	0	0	G
3	BICF2G630355949	89082340	A	0	0	G
3	BICF2G630355956	89092267	A	0.25	0.2143	G
3	BICF2S23445034	89102109	A	0	0	G
3	BICF2G630355965	89115451	G	0	0	A
3	BICF2G630355982	89135642	G	0	0	A
3	BICF2S23347031	89146316	A	0	0	G
3	BICF2G630356013	89151306	G	0.25	0.2143	A
3	BICF2G630356026	89159078	A	0	0	G
3	BICF2G630356027	89159216	A	0	0	G
3	BICF2S2346012	89159812	A	0	0	G
3	BICF2G630356044	89172826	A	0.5	0.5	G
3	BICF2G630356073	89186028	A	0.25	0.2857	G
3	BICF2P514014	89195373	C	0	0	A
3	BICF2G630356099	89216567	G	0.25	0.2143	A
3	BICF2P228434	89218315	A	0.25	0.2143	G
3	BICF2G630356112	89224916	A	0	0	T
3	BICF2G630356115	89229582	G	NA	NA	A
3	BICF2P1253474	89243765	G	0.5	0.5	A
3	BICF2G630356171	89261683	A	0	0	G
3	BICF2G630356178	89267065	A	0	0	G
3	BICF2G630356195	89284056	G	1	1	A
3	BICF2G630356200	89285807	A	1	1	C
3	BICF2G630356205	89287012	A	0.5	0.5	G
3	BICF2G630356221	89299322	A	0.25	0.2143	G
3	BICF2P1416281	89309010	A	0	0	G
3	BICF2G630356255	89321536	G	0.25	0.2143	A
3	BICF2P881093	89335949	A	0.25	0.2857	G
3	BICF2P605518	89343330	A	0	0	T
3	BICF2P544534	89352825	A	0.75	0.7857	G
3	BICF2P297559	89355248	G	0	0	A
3	BICF2G630356326	89388259	A	0.25	0.2857	G
3	TIGRP2P55490_rs8868634	89392461	A	0.25	0.2143	G
3	BICF2P857116	89402283	G	0.25	0.2143	A
3	BICF2P1343469	89419819	C	0.25	0.2143	A
3	BICF2P977797	89431260	A	0.5	0.5	G
3	BICF2P1176918	89444236	G	0.5	0.5	A
3	BICF2P346845	89449609	A	0	0	C

3	BICF2P329146	89461817	G	0	0	A
3	BICF2P101048	89472215	A	0	0	G
3	BICF2P1418480	89490312	0	0	0	G
3	TIGRP2P55525_rs8735190	89495882	G	0.5	0.5	A
3	TIGRP2P55530_rs9107251	89510966	A	0	0	C
3	BICF2P1141118	89517733	A	0	0	C
3	BICF2P1133207	89531836	G	1	1	A
3	BICF2P1295882	89541710	G	0	0	A
3	BICF2G630356365	89551297	A	0	0	C
3	BICF2G630356396	89563948	G	0	0	C
3	BICF2G630356417	89573640	A	0	0	G
3	BICF2P1426993	89592661	0	0	0	A
3	BICF2G630356442	89598786	A	0.5	0.5	G
3	BICF2G630356452	89618611	A	0	0	G
3	BICF2G630356455	89623082	A	0	0	G
3	BICF2G630356463	89631192	A	0	0	G
3	BICF2G630356484	89642627	C	0	0	A
3	BICF2G630356516	89654003	C	0.75	0.7143	A
3	BICF2G630356604	89678792	G	0.25	0.2857	A
3	BICF2G630356628	89689045	A	0.25	0.2857	C
3	BICF2S2318439	89702266	G	0.25	0.2857	A
3	TIGRP2P55579_rs9187865	89714506	A	0.5	0.5	G
3	BICF2S22913163	89729794	A	0.25	0.2143	G
3	BICF2S23216860	89752511	G	0	0	A
3	BICF2S22917719	89765429	A	0	0	G
3	BICF2G630356647	89775440	A	0	0	G
3	BICF2G630356655	89781992	T	0.25	0.2857	A
3	BICF2G630356669	89793145	A	0	0	G
3	BICF2G630356689	89803710	A	0.5	0.5	G
3	BICF2G630356714	89820656	A	0.5	0.5	G
3	BICF2G630356729	89827681	A	0.5	0.5	G
3	BICF2P1164525	89838155	C	0	0	A
3	BICF2G630356766	89850126	G	0.5	0.5	A
3	TIGRP2P55601_rs8863097	89877535	A	0.25	0.2857	C
3	BICF2P151582	89878445	G	0.25	0.2857	A
3	BICF2P457751	89882180	A	0.25	0.2857	G
3	BICF2P267040	89892953	A	0.25	0.2857	G
3	BICF2G630356780	89904012	G	0	0	A
3	BICF2G630356788	89910136	A	0.25	0.2143	G
3	BICF2G630356817	89922792	A	0.25	0.2143	G
3	BICF2G630356837	89935922	A	0.25	0.2857	G
3	BICF2G630356866	89948783	A	0.5	0.5	C
3	BICF2P1021583	89953056	A	0.75	0.7143	G
3	BICF2G630356932	89967365	G	0.25	0.2857	A
3	BICF2G630356957	89977511	G	0.5	0.5	A
3	BICF2G630356983	89988327	G	0.75	0.7857	A
3	BICF2G630357032	90003245	A	0.25	0.2143	C

3	BICF2G630357072	90019424	G	0.75	0.7857	A
3	BICF2G630357081	90022114	A	0	0	G
3	BICF2P1149848	90033614	G	0.25	0.2143	A
3	BICF2G630357140	90047032	G	0	0	A
3	BICF2G630357215	90067970	A	0.25	0.2857	G
3	BICF2P736644	90070178	A	0.25	0.2857	G
3	BICF2G630357248	90101416	G	0.5	0.5	A
3	BICF2G630357260	90110643	C	0	0	A
3	BICF2G630357274	90117965	A	0	0	G
3	BICF2P1260487	90134431	G	0	0	A
3	BICF2G630357299	90139314	C	0.25	0.2143	A
3	BICF2S23032490	90148614	A	0	0	C
3	BICF2G630357319	90160750	G	0.25	0.2857	A
3	BICF2G630357334	90174919	G	0.75	0.7143	A
3	BICF2G630357341	90180731	A	0.75	0.75	G
3	BICF2P435419	90193262	C	0.25	0.2857	A
3	BICF2S2309707	90194722	A	0.25	0.2857	G
3	BICF2S23315277	90207859	G	0.75	0.7143	A
3	BICF2S23315274	90208111	A	0	0	G
3	BICF2G630357407	90219273	G	0.25	0.2143	A
3	BICF2G630357414	90221599	G	0	0	A
3	BICF2G630357432	90231702	A	0	0	G
3	BICF2G630357442	90246482	A	0	0	G
3	BICF2G630357452	90256404	A	0	0	G
3	BICF2G630357458	90261236	A	0	0	G
3	BICF2G630357472	90269479	A	0	0	G
3	BICF2G630357488	90278825	A	0	0	G
3	BICF2G630357515	90293285	A	0.25	0.2857	G
3	BICF2G630357534	90298614	A	0.25	0.2857	C
3	BICF2P1251270	90312715	G	0.25	0.2143	A
3	BICF2G630357616	90328149	G	0.25	0.2143	A
3	BICF2G630357625	90338160	G	0	0	A
3	BICF2G630357647	90347514	A	0	0	G
3	BICF2G630357710	90368989	G	0.75	0.7143	A
3	BICF2G630357728	90381075	G	0	0	A
3	BICF2G630357748	90397417	A	0.25	0.2857	G
3	BICF2G630357768	90414435	G	0	0	A
3	BICF2G630357780	90430513	C	0	0	A
3	BICF2P725076	90445365	0	0	0	C
3	BICF2P427640	90454862	0	0	0	A
3	BICF2S2352275	90466189	A	0.25	0.2857	G
3	BICF2P965985	90474124	G	0.25	0.2857	A
3	BICF2S23720272	90489157	A	1	1	G
3	BICF2P874738	90498112	G	0.25	0.2857	A
3	BICF2P874739	90498565	A	0.25	0.2857	G
3	BICF2S2337940	90525676	A	0	0	G
3	BICF2S23336259	90530673	A	0	0	G

3	BICF2G630357835	90563150	A	0.25	0.2857	G
3	BICF2G630357849	90575284	A	0	0	G
3	BICF2P856988	90587115	G	1	1	A
3	BICF2P856987	90587396	G	1	1	A
3	BICF2P263449	90588369	G	1	1	A
3	BICF2G630357873	90601528	A	0	0	G
3	TIGRP2P55710_rs9174112	90611300	G	0	0	A
3	BICF2G630357890	90626083	G	0.25	0.2857	A
3	TIGRP2P55718_rs8893256	90634191	A	0	0	G
3	BICF2G630357943	90656425	C	0.25	0.2857	A
3	BICF2P401637	90675531	A	0	0	G
3	BICF2G630357966	90677333	G	0	0.1429	A
3	BICF2G630357968	90679530	A	0	0	G
3	BICF2G630357985	90693648	C	0.25	0.4286	A
3	BICF2G630358035	90718802	C	0	0	A
3	BICF2G630358046	90724165	G	1	0.8571	A
3	BICF2G630358064	90742553	C	1	0.9167	A
3	BICF2P866204	90757469	G	0	0	A
3	BICF2G630358096	90767797	A	0	0.1429	G
3	BICF2G630358118	90773840	G	0.75	0.6429	A
3	BICF2S22912748	90787957	C	0	0.1429	A
3	BICF2P793882	90799028	C	0	0	A
3	BICF2G630358124	90807324	A	0	0	C
3	BICF2G630358126	90817113	G	1	1	A
3	BICF2P781352	90829889	G	0.25	0.3571	A
3	BICF2S23220241	90832872	A	0	0	G
3	BICF2G630358129	90842529	A	0.25	0.2143	G
3	BICF2G630358140	90850009	G	0.75	0.7143	A
3	BICF2G630358155	90861550	G	0.5	0.5	A
3	BICF2G630358205	90876433	A	0	0	G
3	BICF2G630358221	90885261	A	0.25	0.4286	G
3	BICF2G630358225	90889366	A	0	0	C
3	BICF2G630358244	90896109	A	0.25	0.2143	G
3	BICF2G630358257	90906836	A	0.25	0.2143	G
3	BICF2S23242114	90907197	A	0.25	0.2143	C
3	BICF2G630358279	90915155	A	0	0	G
3	BICF2G630358287	90919121	C	0.25	0.2143	A
3	BICF2P217896	90922391	G	0.75	0.625	A
3	BICF2G630358305	90933142	A	0	0	G
3	BICF2S23221091	90953280	A	1	1	C
3	BICF2G630358346	90954735	A	1	1	G
3	TIGRP2P55830_rs9237785	90965971	G	0.25	0.2857	A
3	BICF2P854814	90985609	G	1	0.8571	A
3	BICF2G630358393	90987595	A	0	0	C
3	BICF2G630358395	90988310	G	0	0.1429	A
3	BICF2P552322	91006987	G	0.25	0.2857	A
3	BICF2S23649843	91018500	A	0.25	0.4286	G

3	BICF2P1298736	91031497	G	0.75	0.7857	A
3	TIGRP2P55842_rs8846762	91037189	A	0	0	G
3	TIGRP2P55856_rs9224139	91048832	0	0	0	A
3	BICF2G630358448	91077800	A	0.5	0.5	G
3	BICF2G630358465	91084659	A	0	0.1429	G
3	BICF2G630358478	91087300	A	0	0.1429	C
3	BICF2G630358491	91093319	A	0	0.1429	G
3	BICF2P726237	91108125	0	0	0	A
3	BICF2G630358500	91118478	T	0.25	0.2857	A
3	BICF2S23348076	91133389	G	0	0.1429	A
3	BICF2P1152859	91143858	A	0	0.1429	G
3	TIGRP2P55888_rs9211220	91147591	G	0.5	0.5	A
3	TIGRP2P55895_rs8565358	91159415	T	0	0.1429	A
3	BICF2P213912	91161736	C	0	0.1429	A
3	BICF2G630358507	91180492	G	0.25	0.2143	A
3	BICF2G630358512	91185426	A	0.75	0.7857	G
3	TIGRP2P55899_rs8955313	91198889	C	0.5	0.3571	G
3	BICF2G630358530	91201876	A	0.25	0.3571	C
3	BICF2G630358534	91203501	A	0.25	0.2857	T
3	BICF2P192508	91209284	G	0.5	0.3571	A
3	BICF2P1329392	91219743	G	0.5	0.3571	A
3	BICF2G630358589	91233444	A	0.25	0.3571	T
3	BICF2G630358611	91247771	A	0	0	G
3	BICF2G630358638	91255099	A	1	1	C
3	TIGRP2P55933_rs8767797	91273897	A	0.25	0.3	G
3	BICF2S23120051	91284844	A	0.5	0.3571	G
3	BICF2P391939	91289839	0	0	0	A
3	BICF2S23520428	91304682	G	0	0	A
3	BICF2G630358640	91314456	G	0.25	0.3571	C
3	BICF2S23142281	91324432	0	0	0	G
3	BICF2P461483	91336346	0	0	0	A
3	BICF2G630358684	91365888	G	0.5	0.3571	A
3	BICF2G630358687	91369267	C	0.5	0.3333	A
3	BICF2G630358690	91369800	G	0.5	0.3571	C
3	BICF2G630358717	91380967	C	0.5	0.3571	A
3	BICF2G630358738	91391177	G	0.5	0.5	A
3	BICF2P903299	91402786	A	0.5	0.3571	G
3	BICF2G630358788	91406506	G	0	0.1429	A
3	BICF2G630358801	91418066	A	0	0	G
3	BICF2G630358827	91433824	A	0.5	0.3571	G
3	BICF2G630358846	91438673	A	1	0.8571	G
3	BICF2G630358864	91456583	G	0	0	A
3	BICF2G630358871	91468680	A	0	0	G
3	BICF2P528180	91471138	A	0	0.1429	G
3	BICF2P1141218	91487767	G	0	0.1429	A
3	BICF2P1282453	91493748	A	0	0.1429	G
3	BICF2G630358907	91494413	A	0	0	G

3	BICF2P235747	91511643	A	0	0.1429	G
3	BICF2G630358959	91518210	A	0	0	C
3	BICF2P1145732	91537696	T	0	0.2857	A
3	BICF2G630359000	91539418	G	0.5	0.3571	A
3	TIGRP2P56000_rs8602529	91549042	A	0	0.1429	C
3	BICF2G630359015	91553711	G	0	0	A
3	BICF2S23159307	91557582	G	0.5	0.3571	A
3	BICF2G630359046	91571695	A	0.5	0.3571	G
3	BICF2G630359047	91571825	G	0	0	A
3	BICF2G630359066	91582955	A	0	0	C
3	BICF2P1156603	91594342	A	0.5	0.3571	G
3	BICF2G630359092	91595106	G	0.5	0.3571	A
3	BICF2G630359107	91600495	A	0.5	0.3571	G
3	BICF2S23646464	91609883	A	0.5	0.2143	C
3	BICF2P1282800	91629817	A	0	0.2857	G
3	TIGRP2P56017_rs8890666	91632249	0	0	0	A
3	BICF2P1435943	91647652	C	1	0.8571	G
3	TIGRP2P56046_rs9039529	91661684	0	0	0	A
3	BICF2G630359132	91666827	A	0	0	G
3	BICF2G630359134	91678586	A	1	0.5714	G
3	BICF2G630359182	91692951	A	0.5	0.5	G
3	BICF2S23653217	91701153	A	0.5	0.3571	G
3	BICF2G630359186	91721437	C	0	0.1429	A
3	BICF2P1097236	91721974	A	0.5	0.2143	G
3	BICF2S23539373	91724378	A	0	0	C
3	BICF2P310706	91740607	A	0	0.1429	C
3	BICF2S2346160	91747119	A	0	0	G
3	BICF2G630359233	91764521	G	0	0	A
3	BICF2G630359242	91770049	A	0.5	0.2143	G
3	BICF2P280649	91785257	A	0	0.4286	T
3	BICF2P280650	91785665	A	0	0.4286	G
3	BICF2G630359277	91802222	A	0	0	G
3	BICF2S22920827	91804817	C	0	0	A
3	BICF2G630359334	91816766	G	0.5	0.2143	A
3	BICF2G630359356	91829514	G	0.5	0.3571	A
3	BICF2S23657159	91849499	G	1	0.5714	A
3	BICF2G630359393	91849783	G	1	0.8571	A
3	BICF2G630359424	91867282	G	0	0	A
3	BICF2G630359433	91875286	A	0.5	0.6429	G
3	BICF2G630359448	91890729	G	1	0.8571	A
3	BICF2G630359454	91900153	C	1	0.8571	A
3	BICF2P12045	91909053	G	0	0	A
3	BICF2G630359478	91923091	G	0.5	0.2143	A
3	BICF2G630359488	91933300	G	0.5	0.3571	C
3	BICF2S22956632	91939525	A	1	0.5714	G
3	BICF2G630359517	91944314	A	0	0	C
3	BICF2G630359539	91954471	G	1	0.8571	A

3	BICF2P1405503	91965542	A	0	0.1429	G
3	BICF2G630359593	91972805	A	1	0.8571	G
3	BICF2G630359611	91979851	G	0	0.1429	A
3	BICF2G630359629	91995750	C	1	0.5714	A
3	BICF2P204702	92007286	A	1	0.9167	G
3	BICF2G630359661	92016374	T	0	0	A
3	BICF2G630359688	92023901	A	0	0.2857	G
3	TIGRP2P56189_rs9051831	92038947	A	0.5	0.2143	C
3	BICF2G630359740	92046482	A	0	0	G
3	BICF2G630359785	92060036	G	0.5	0.2143	A
3	BICF2G630359806	92076801	A	0.5	0.2143	G
3	BICF2G630359825	92083794	A	0	0.1429	C
3	BICF2G630359853	92093703	A	0.5	0.2143	G
3	TIGRP2P56199_rs8868169	92105769	0	0	0	C
3	BICF2P1331792	92118498	G	0.5	0.2143	A
3	BICF2G630359866	92129218	A	0	0.2857	C
3	BICF2G630359877	92142778	G	1	0.8571	A
3	BICF2P300068	92157207	G	0	0	A
3	BICF2G630359898	92163860	A	1	0.9167	C
3	BICF2G630359917	92173779	A	0	0.1429	G
3	BICF2G630359938	92186454	G	0	0	A
3	BICF2G630359951	92196460	A	0	0	C
3	BICF2G630359981	92207326	G	0	0	A
3	BICF2G630360010	92223569	G	0.5	0.5	A
3	BICF2G630360016	92225694	A	0.5	0.3571	G
3	BICF2G630360036	92230594	A	0.5	0.5	C
3	BICF2G630360054	92247429	A	0	0	C
3	BICF2G630360062	92259782	G	0.5	0.3571	A
3	BICF2G630360067	92266056	G	0.5	0.3571	A
3	BICF2G630360106	92279131	A	0	0	C
3	BICF2G630360159	92295134	G	0	0	A
3	BICF2G630360179	92304010	A	0	0	G
3	TIGRP2P56266_rs9151482	92314560	A	0	0	G
3	BICF2G630360211	92325351	C	0	0.2857	A
3	TIGRP2P56271_rs8992400	92337946	A	0.5	0.7857	G
3	BICF2S2341832	92351729	A	0	0.08333	C
3	BICF2P606039	92357499	A	0.5	0.2143	G
3	BICF2S23033256	92370635	G	0	0	A
3	BICF2G630360225	92386749	A	0	0	G
3	BICF2P862964	92402043	G	0	0.4286	A
3	BICF2P964095	92411287	C	0.5	0.2143	G
3	BICF2G630360248	92417378	G	0	0	A
3	BICF2G630360272	92429580	G	0	0	A
3	BICF2G630360278	92434850	A	0	0	G
3	BICF2P1444829	92444609	0	0	0	A
3	BICF2G630360282	92454874	G	0	0	A
3	BICF2P238409	92463582	A	0.5	0.3571	G

3	BICF2G630360306	92476123	A	0.5	0.3571	C
3	BICF2G630360326	92487972	A	1	0.5714	G
3	TIGRP2P56296_rs8626443	92497811	A	1	0.7143	G
3	BICF2S2337748	92527005	G	0	0	A
3	BICF2G630360337	92529071	0	0	0	G
3	BICF2P94972	92551164	G	0	0	A
3	TIGRP2P56300_rs8682212	92555190	A	0	0	G
3	BICF2P577230	92569486	G	0.5	0.3571	A
3	BICF2P573054	92584140	G	1	0.5714	A
3	BICF2P559214	92592289	0	0	0	G
3	TIGRP2P56325_rs8972094	92600209	G	1	0.5714	A
3	BICF2P305365	92611988	A	0	0	C
3	BICF2P462755	92618508	A	0.5	0.3571	G
3	BICF2P178641	92632104	G	1	0.8571	A
3	BICF2S23310557	92639431	A	0	0.2857	G
3	BICF2S2326451	92656899	A	0.5	0.5	G
3	BICF2G630360341	92669263	G	0	0.1429	A
3	BICF2G630360352	92679280	A	0	0.4286	C
3	BICF2G630360392	92696042	G	1	0.7143	A
3	TIGRP2P56360_rs8991444	92704755	A	0	0	G
3	BICF2S22958394	92714412	G	0.5	0.3571	A
3	BICF2G630360421	92730902	A	0.5	0.3571	T
3	BICF2P736282	92737264	0	0	0	G
3	BICF2G630360437	92758002	A	1	0.8571	G
3	BICF2G630360440	92762689	A	0.5	0.8333	C
3	BICF2P1085907	92771518	A	0.5	0.7857	G
3	BICF2G630360460	92782126	G	0.5	0.6429	A
3	BICF2P440167	92801379	T	0	0.2857	A
3	BICF2G630360498	92814113	A	0.5	0.6429	C
3	BICF2P965800	92818162	A	0	0.2857	C
3	BICF2P1433471	92844742	0	0	0	G
3	BICF2G630360527	92861353	G	0.5	0.3571	A
3	BICF2G630360540	92864088	A	0.5	0.3571	G
3	BICF2G630360558	92876102	A	0.5	0.3571	G
3	BICF2G630360569	92888906	A	0.5	0.2143	G
3	BICF2G630360596	92898131	G	0.5	0.3571	A
3	BICF2G630360626	92918288	A	0.5	0.3571	G
3	BICF2G630360632	92920698	A	0.5	0.2143	G
3	BICF2S22910940	92934377	A	1	0.5714	G
3	BICF2G630360637	92948610	A	0	0	G
3	BICF2P971218	92959822	A	1	0.8571	G
3	BICF2P1000952	92968134	A	0	0	G
3	BICF2S23434560	92979889	A	0	0.1429	G
3	BICF2S2299695	92997196	A	0	0.4286	G
3	BICF2G630360651	93002840	G	1	0.8571	A
3	BICF2G630360662	93015509	A	0	0.2857	C
3	BICF2G630360692	93026056	0	0	0	G

3	BICF2G630360725	93036963	G	0	0	C
3	BICF2G630360750	93049536	A	1	0.8571	T
3	TIGRP2P56461_rs8795035	93068421	A	0	0	C
3	BICF2P1383106	93069666	T	0	0	A
3	BICF2G630360758	93083109	A	0	0	G
3	BICF2G630360771	93106600	G	0.5	0.3571	A
3	TIGRP2P56495_rs9206866	93106827	G	0	0	A
3	BICF2P285489	93118976	A	0	0	G
3	BICF2S2334899	93136680	G	0	0.1429	A
3	BICF2G630360777	93141486	A	0	0	T
3	BICF2G630360807	93158328	0	0	0	G
3	BICF2S23020837	93175173	0	0	0	A
3	BICF2G630360814	93190144	G	0	0	A
3	BICF2P1334353	93207105	C	0	0.2857	A
3	TIGRP2P56508_rs9191466	93209970	G	0	0.2857	A
3	TIGRP2P56519_rs8959077	93226945	C	0	0	A
3	BICF2G630360841	93232560	G	0	0	A
3	BICF2G630360899	93252748	A	0	0.1429	G
3	BICF2G630360917	93261593	A	0.5	0.7857	G
3	BICF2G630360921	93266933	A	0.5	0.3571	G
3	BICF2P547826	93287324	G	0	0.1429	A
3	BICF2G630360965	93288970	G	0	0	A
3	BICF2P985903	93300498	C	0	0.1429	G
3	BICF2G630360986	93302632	T	1	0.8571	A
3	TIGRP2P56526_rs8841319	93315941	0	0	0	G
3	BICF2P1442524	93323292	A	0	0	C
3	BICF2G630361025	93334344	G	0	0	A
3	BICF2G630361060	93351298	C	0	0	A
3	BICF2P1316299	93359777	G	0	0	A
3	BICF2P1400721	93364570	A	0	0	C
3	BICF2G630361118	93373458	A	0	0	G
3	BICF2G630361156	93388160	G	0	0	C
3	BICF2G630361183	93397800	G	0.5	0.2143	A
3	BICF2P1386355	93408534	A	0	0	G
3	BICF2G630361219	93415696	G	0	0	A
3	BICF2P486742	93432486	A	0.5	0.7857	G
3	BICF2P616077	93435448	A	0.5	0.6429	C
3	BICF2G630361240	93443600	G	0.5	0.3571	A
3	TIGRP2P56552_rs8783692	93449255	A	0.5	0.3571	C
3	BICF2G630361250	93453951	A	0.5	0.2143	G
3	BICF2S23739949	93458125	G	0	0	A
3	BICF2P736579	93468384	G	0.5	0.3571	A
3	BICF2P1034616	93478160	G	1	1	A
3	BICF2S23140592	93490378	A	0	0	G
3	BICF2P1336605	93496113	A	0	0	G
3	BICF2S23320812	93520425	C	0	0.4286	A
3	TIGRP2P56604_rs8738045	93539230	G	0	0	A

3	BICF2P1090735	93542118	A	0	0	G
3	BICF2S23630742	93554654	A	0.5	0.3571	C
3	BICF2S23658912	93572732	A	0	0	G
3	TIGRP2P56631_rs9248872	93576449	C	0	0	A
3	TIGRP2P56632_rs8586189	93594808	A	0.5	0.7857	G
3	BICF2P186901	93609499	0	0	0	G
3	BICF2P186904	93610097	G	0.5	0.2143	C
3	BICF2P16210	93618479	C	0.5	0.7857	G
3	TIGRP2P56654_rs9051017	93622231	A	0	0	G
3	BICF2P593795	93639445	A	0	0	G
3	TIGRP2P56667_rs9213518	93654803	A	0	0	G
3	BICF2P69321	93656112	A	0	0	G
3	TIGRP2P56697_rs8554529	93668058	G	0	0	A
3	BICF2G630361279	93684805	A	0	0	G
3	BICF2G630361292	93697691	A	0.5	0.6429	G
3	BICF2S23344409	93700678	A	0	0	G
3	BICF2S23021180	93702157	G	0	0	A
3	BICF2G630361358	93715699	G	0	0	A
3	BICF2G630361390	93731523	A	0	0	G
3	BICF2G630361402	93737251	A	0	0	G
3	TIGRP2P56753_rs8687916	93757480	A	0.75	0.7857	C
3	BICF2G630361429	93759842	G	0.75	0.7857	A
3	BICF2G630361447	93771750	A	1	1	G
3	TIGRP2P56757_rs8845596	93782961	C	0	0	A
3	BICF2G630361486	93799182	C	0	0	A
3	TIGRP2P56768_rs9180220	93815429	A	0.75	0.7143	G
3	BICF2P157363	93815630	T	0	0	A
3	BICF2G630361536	93826885	T	0	0	A
3	BICF2G630361552	93848039	A	0	0	G
3	BICF2P635494	93851186	A	0.5	0.6429	G
3	BICF2G630361560	93855729	A	0	0	G
3	BICF2G630361583	93864300	G	0.5	0.5	A
3	BICF2G630361616	93878015	G	0.25	0.2857	A
3	BICF2S2377250	93890772	G	0	0	A
3	BICF2G630361659	93897764	A	0	0	G
3	BICF2G630361674	93914980	G	0.5	0.5	A
3	BICF2G630361693	93927492	A	0.25	0.3571	G
3	TIGRP2P56799_rs8666557	93933450	A	0.25	0.2857	C
3	BICF2G630361702	93944095	A	0	0	G
3	TIGRP2P56825_rs9063147	93955329	G	0.25	0.2857	A
3	BICF2G630361725	93970062	A	0.25	0.2857	C
3	BICF2G630361729	93988514	T	0.25	0.2857	A
3	BICF2G630361732	93996489	T	0.25	0.2857	A
3	BICF2G630361734	94008502	C	0.25	0.4286	A
3	BICF2G630361736	94014134	G	0	0	A
3	BICF2G630361738	94025739	A	0.25	0.2857	G
3	BICF2G630361742	94035855	C	0.25	0.2857	A

3	BICF2G630361752	94075783	G	0.25	0.2857	A
3	BICF2G630361761	94088224	A	0.25	0.2857	G
3	BICF2S23417660	94098516	A	0	0	G
3	BICF2G630361774	94112168	A	0	0	G
3	BICF2P120209	94119812	0	0	0	G
3	BICF2G630361777	94129590	G	0	0	A
3	BICF2G630361781	94145159	A	0	0	T
3	BICF2G630361787	94159014	G	0.25	0.3571	A
3	BICF2G630361800	94163416	C	0.25	0.3571	A
3	BICF2G630361819	94183739	A	0	0.1429	G
3	BICF2S2302098	94186764	G	0	0.1429	A
3	BICF2G630361830	94202201	C	0	0	A
3	BICF2P1456918	94211980	A	0	0.1429	G
3	BICF2P981487	94221985	A	0	0	G
3	BICF2G630361854	94234613	C	0	0	G
3	BICF2P124515	94246515	G	1	1	A
3	BICF2G630361863	94254918	G	0	0	A
3	BICF2P1251013	94268480	0	0	0	G
3	BICF2G630361892	94271352	G	0	0	A
3	BICF2G630361903	94287447	A	0.25	0.2143	G
3	BICF2P12847	94296622	A	0	0	G
3	BICF2P1008504	94306027	G	0.75	0.7857	A
3	BICF2P1240644	94321355	A	0	0	G
3	BICF2P888349	94323625	G	0.25	0.3571	A
3	BICF2P440396	94355737	A	0	0	G
3	BICF2S23326367	94365026	0	0	0	G
3	BICF2G630361929	94372069	A	0	0	G
3	BICF2P570239	94389426	A	0.25	0.3571	G
3	BICF2G630361953	94397805	A	0.75	0.6667	G
3	BICF2G630361960	94410234	G	0.25	0.3571	A
3	BICF2G630361962	94415960	G	1	1	A
3	BICF2G630361968	94444210	A	0.75	0.6429	G
3	BICF2G630361978	94451861	A	0	0	G
3	BICF2S2323991	94469321	A	0	0	G
3	BICF2G630362013	94476356	G	0.75	0.6429	A
3	BICF2G630362025	94484373	A	0	0	G
3	TIGRP2P56940_rs9188947	94503865	0	0	0	G
3	TIGRP2P56941_rs9070076	94507527	0	0	0	G
3	BICF2G630362033	94526955	C	0	0	A
3	BICF2G630362037	94545445	G	0	0	A
3	BICF2G630362040	94560609	A	0	0	G
3	BICF2S23735867	94574810	A	0.25	0.2143	G
3	BICF2G630362044	94578249	A	0	0	G
3	BICF2G630362047	94588307	A	0	0	G
3	BICF2G630362054	94607062	A	0	0	G
3	BICF2G630362055	94622240	A	0.25	0.2143	G
3	TIGRP2P56952_rs9077464	94641142	0	0	0	C

3	BICF2G630362059	94659212	G	0	0	A
3	BICF2G630362060	94668519	A	0.25	0.2143	C
3	BICF2S22917226	94684238	C	0	0	A
3	BICF2P1491488	94693816	0	0	0	G
5	BICF2P1500181	3110350	A	0	0	G
5	BICF2P56652	3125894	A	0	0	G
5	BICF2P114876	3141134	A	0	0	G
5	BICF2P1288014	3154438	A	0	0	G
5	BICF2P475735	3167473	A	0	0	G
5	BICF2P677323	3192752	C	0	0	A
5	BICF2P675052	3206571	G	0	0	A
5	TIGRP2P69198_rs8753444	3210199	0	0	0	A
5	BICF2P778734	3222808	G	0	0.07143	A
5	BICF2P1201924	3236960	G	0	0	A
5	BICF2S23351408	3246631	A	0	0	G
5	BICF2S2363087	3267089	G	1	0.7857	A
5	BICF2S23145124	3288561	A	0	0	G
5	BICF2S23144867	3292352	A	0	0.2857	C
5	BICF2S23656250	3309063	A	0	0	G
5	BICF2P1302176	3324711	0	0	0	A
5	BICF2P255244	3349182	0	0	0	A
5	BICF2P762227	3376802	0	0	0	A
5	BICF2P1470603	3398537	0	0	0	A
5	BICF2S23347568	3412417	A	0	0	G
5	BICF2P1230843	3423976	0	0	0	T
5	BICF2S23246910	3440799	A	0	0	G
5	BICF2S23111744	3468623	A	0	0	C
5	BICF2P1004508	3482952	0	0	0	A
5	BICF2P878791	3489634	0	0	0	A
5	TIGRP2P69200_rs8912367	3517064	0	0	0	C
5	BICF2S23145981	3529758	G	0	0	A
5	BICF2P781083	3532699	0	0	0	A
5	BICF2P1299264	3551586	A	0	0	G
5	BICF2S232682	3551748	A	0	0	G
5	BICF2P1168178	3555046	0	0	0	C
5	BICF2S23043641	3576014	G	0	0	A
5	BICF2P68740	3582721	A	0	0	G
5	BICF2P11220	3596243	0	0	0	A
5	BICF2P1370280	3601062	0	0	0	G
5	BICF2S23630240	3629824	A	0	0.2143	G
5	BICF2S2378885	3639193	A	1	0.7857	G
5	BICF2S23019493	3653290	G	1	0.75	A
5	BICF2P1268945	3662168	0	0	0	C
5	BICF2P399740	3690698	0	0	0	G
5	BICF2P1334765	3722027	A	0	0	G
5	TIGRP2P69203_rs8645766	3735899	0	0	0	C
5	BICF2S23713946	3741194	G	0	0.2143	A

5	TIGRP2P69205_rs8688773	3764172	0	0	0	C
5	BICF2S23331034	3789372	A	0	0	G
5	G320f26S259	3814438	G	0	0.2143	A
5	BICF2S23654148	3838565	A	1	0.7857	G
5	BICF2S23312738	3841187	A	0	0	C
5	BICF2S23229412	3856781	A	0	0	G
5	BICF2S23763724	3874176	A	0	0	G
5	BICF2S23630569	3888762	G	0	0	A
5	BICF2S23024940	3921483	A	0	0	G
5	BICF2S23022533	3940307	A	0	0	C
5	BICF2S23027795	3956713	G	0	0	A
5	BICF2S23316443	3970728	A	0	0	G
5	BICF2S23723731	3984647	A	0	0	G
5	TIGRP2P69207_rs9190391	3993303	0	0	0	C
5	BICF2S23018252	4005343	A	0	0	G
5	BICF2P1368718	4020276	G	0	0	A
5	BICF2P792591	4032314	0	0	0	G
5	BICF2S23341594	4047398	G	0	0	A
5	BICF2P392853	4062985	0	0	0	A
5	BICF2S23225878	4073074	A	0	0.2143	G
5	BICF2S23615788	4087919	G	0	0	A
5	BICF2S23438379	4109201	A	0	0	G
5	BICF2S2308288	4122702	G	0	0	A
5	BICF2S23333242	4136210	A	0	0	G
5	BICF2S23422195	4159669	A	0	0	G
5	BICF2P117765	4168368	A	0	0	C
5	BICF2S2459722	4195975	G	0	0	A
5	TIGRP2P69209_rs8802587	4198535	0	0	0	A
5	BICF2S2362618	4219765	G	0	0	A
5	BICF2P524908	4221133	0	0	0	C
5	BICF2S23325646	4242068	A	0	0	G
5	BICF2P500511	4260995	0	0	0	G
5	BICF2S2397964	4289863	A	0	0.2143	G
5	BICF2S23313764	4304623	G	0	0	A
5	BICF2S23127889	4314822	A	0	0	G
5	BICF2P374835	4340964	A	0	0	G
5	TIGRP2P69210_rs8950597	4355547	0	0	0	G
5	G321f18S109	4359281	A	0	0	G
5	BICF2P862384	4388522	A	0	0	G
5	BICF2S23251062	4394285	A	0	0	G
5	BICF2P656448	4423748	A	0	0	G
5	BICF2P656450	4423865	0	0	0	C
5	BICF2S23618216	4445761	A	0	0	G
5	BICF2P1170793	4451358	0	0	0	G
5	BICF2P886802	4477088	A	0	0	G
5	BICF2P856824	4485926	0	0	0	C
5	BICF2S23527029	4498273	A	0	0	G

5	BICF2P196552	4518164	0	0	0	A
5	BICF2S23140940	4528783	A	0	0	G
5	BICF2S2379205	4558780	C	0	0.2143	A
5	BICF2P742243	4571811	A	0	0	G
5	BICF2P1266529	4593592	0	0	0	C
5	BICF2P1368700	4600369	0	0	0	G
5	BICF2P334147	4620374	0	0	0	A
5	BICF2P1041087	4649433	0	0	0	A
5	BICF2S23347253	4664452	A	0	0.5	G
5	BICF2P179815	4685577	0	0	0	A
5	BICF2S23056544	4697408	G	0	0.5	A
5	G322f34S191	4728376	C	0	0.5	A
5	BICF2S23418659	4781523	G	0	0.2143	A
5	BICF2S2345424	4790781	G	0	0.2143	A
5	BICF2S23537022	4796153	G	0	0.1667	A
5	BICF2S23714765	4812737	A	0	0	C
5	BICF2S2441187	4830522	A	0	0.1667	G
5	BICF2P1195895	4850514	0	0	0	G
5	BICF2S23061033	4874678	A	0	0	C
5	BICF2S23617519	4890275	A	0	0	G
5	BICF2S23531185	4910141	A	0	0.2143	G
5	TIGRP2P69220_rs9087523	4912769	0	0	0	C
5	BICF2P768596	4927198	0	0	0	A
5	BICF2S23233354	4943609	A	0	0	G
5	BICF2S23427325	4959605	G	0	0	A
5	BICF2S23752195	4979116	A	0	0	G
5	BICF2S23736883	4995639	A	0	0.2857	G
5	BICF2P1298577	5003186	A	0	0	C
5	BICF2P954875	5022265	A	0	0	G
5	BICF2S23121225	5036566	A	1	0.5	G
5	BICF2P1219699	5053170	A	0	0	G
5	BICF2P287339	5061258	G	0	0	A
5	BICF2P1140415	5080128	C	0	0	G
5	BICF2S23323013	5081598	A	0	0	G
5	BICF2P1114742	5092933	A	0	0	C
5	BICF2P1238072	5096384	G	0	0.2143	A
5	BICF2P96458	5109464	A	0	0	G
5	BICF2P96460	5109887	A	0	0.1667	G
5	BICF2P818281	5116564	A	0	0	G
5	BICF2P818283	5117883	G	0	0.2143	A
5	BICF2P1056586	5132750	G	0	0	A
5	BICF2P302891	5141847	A	0	0.2143	G
5	BICF2P1019023	5160498	G	0	0.2143	A
5	BICF2P1209432	5164730	G	0	0	A
5	BICF2P262272	5182010	C	0	0.2143	A
5	BICF2P708831	5183984	G	0	0.2143	A
5	BICF2P520838	5192454	G	0	0.2143	A

5	BICF2P782395	5200504	A	0	0	C
5	BICF2P567634	5220300	G	0	0.2143	A
5	BICF2P201270	5227328	0	0	0	A
5	BICF2P908011	5239342	G	0	0.125	A
5	BICF2P1457733	5245724	G	0	0	A
5	BICF2S23245588	5262874	G	0	0.2143	A
5	BICF2P735121	5265124	A	0	0.2143	G
5	BICF2P527923	5268134	A	0	0.2143	G
5	BICF2P978257	5289248	A	0	0.2143	G
5	BICF2P192233	5291944	0	0	0	A
5	BICF2P443580	5301973	A	0.5	0.5714	G
5	BICF2P1183273	5321680	0	0	0	G
5	BICF2P913682	5323685	A	0.5	0.7857	C
5	BICF2P1171838	5325494	A	0	0	G
5	BICF2S23311911	5337103	A	1	1	G
5	BICF2P270770	5356257	G	0.5	0.7857	A
5	BICF2P29644	5362514	G	0.5	0.5833	A
5	BICF2P617470	5371348	0	0	0	A
5	BICF2P275101	5385868	0	0	0	G
5	BICF2P274313	5395196	G	0.5	0.7857	A
5	BICF2P40584	5410299	A	0.5	0.7857	C
5	BICF2P950563	5419410	T	0.5	0.5714	A
5	BICF2P1146050	5430745	A	0.5	0.5714	G
5	BICF2P663601	5445283	A	0.5	0.7857	G
5	BICF2P962049	5451580	G	0.5	0.7857	A
5	BICF2P450265	5471532	0	0	0	A
5	BICF2P876078	5481390	A	0.5	0.7857	C
5	BICF2P283157	5489168	C	0.5	0.7857	G
5	BICF2P593671	5498902	A	0	0	G
5	BICF2P966369	5520845	A	0	0.1	G
5	BICF2P658310	5535341	G	0	0	A
5	BICF2P1405369	5543739	C	0	0	A
5	BICF2P164675	5556488	G	0	0.2143	A
5	BICF2S23346904	5570196	G	0	0	A
5	BICF2P854713	5583551	A	0	0	G
5	BICF2P1273243	5594800	G	0.5	0.5714	A
5	BICF2P936669	5600355	G	0.5	0.5714	A
5	BICF2P409122	5607836	G	0.5	0.5714	A
5	BICF2P338231	5618069	G	0.5	0.5714	A
5	BICF2P1359807	5628417	G	0.5	0.5714	A
5	BICF2P410802	5636733	G	0.5	0.5714	A
5	BICF2S23729849	5648607	G	0	0.2143	A
5	BICF2P1076530	5665939	A	0.5	0.5714	G
5	BICF2P763240	5673924	C	0.5	0.5714	A
5	BICF2P802518	5681566	A	0.5	0.5714	G
5	BICF2P760534	5692818	G	0.5	0.5714	C
5	BICF2S23217065	5699287	G	0.5	0.5714	A

5	BICF2P813213	5705199	A	0.5	0.5714	G
5	BICF2S2342356	5717604	A	0	0	G
5	BICF2S22951057	5737390	0	0	0	A
5	BICF2P462619	5739133	A	0.5	0.5714	G
5	BICF2P349906	5748505	A	0.5	0.7857	G
5	BICF2P547074	5753687	A	0.5	0.7857	C
5	BICF2P137781	5772954	G	0.5	0.7857	A
5	BICF2S23764501	5783703	A	0.5	0.7857	G
5	BICF2S2331712	5789074	A	0.5	0.7857	G
5	BICF2S23041361	5799854	G	0	0	A
5	BICF2P275085	5810731	G	0	0	A
5	BICF2S23061625	5825871	A	0.5	0.5714	G
5	BICF2P669852	5833032	A	0.5	0.5714	G
5	BICF2P565692	5846112	A	0	0	G
5	BICF2P422273	5854248	A	0	0	G
5	BICF2P1224352	5873621	G	0.5	0.5714	A
5	BICF2P1049948	5879846	A	0	0	C
5	BICF2P27137	5891169	A	0	0	G
5	BICF2S23130444	5900427	G	0.5	0.5714	A
5	BICF2P1267468	5921480	A	0	0	G
5	BICF2S23311427	5932893	G	0.5	0.5714	A
5	BICF2P277249	5937923	0	0	0	G
5	BICF2P94903	5941857	C	0	0	G
5	BICF2S22918910	5952369	G	0.5	0.5714	A
5	BICF2P822990	5960485	A	0	0	G
5	BICF2P574548	5976732	A	0.5	0.5714	G
5	BICF2S23716205	5980372	A	0	0	T
5	BICF2S23317601	5998728	G	0.5	0.5714	A
5	BICF2S23225204	6025573	A	0.5	0.5714	G
5	BICF2P1463618	6031051	A	0	0	G
5	BICF2S2368993	6048559	A	0	0	G
5	BICF2P1489253	6058123	A	NA	0.9	G
5	BICF2S22919400	6060683	A	0.5	0.7857	C
5	BICF2S22951159	6073736	A	0.5	0.7857	G
5	BICF2P458418	6085601	A	0.5	0.7857	G
5	BICF2P946923	6095996	A	0	0	G
5	BICF2P1336010	6110503	G	0	0	A
5	BICF2S23424650	6125853	A	0	0	G
5	BICF2P109254	6132159	A	0	0	G
5	BICF2S22956006	6142871	A	0.5	0.7857	G
5	TIGRP2P73489_rs8781647	6162210	0	0	0	C
5	BICF2P678287	6169061	0	0	0	G
5	BICF2P418082	6181271	0	0	0	G
5	BICF2P328089	6187161	0	0	0	A
5	BICF2S23718810	6221628	A	0.5	0.7857	G
5	BICF2P361729	6234873	G	0.5	0.7857	C
5	BICF2P358450	6247457	A	0	0	G

5	BICF2S23423433	6264155	A	0	0	C
5	BICF2P95925	6277167	G	0.5	0.7857	C
5	BICF2S23044458	6288253	G	0.5	0.7857	A
5	BICF2P249643	6290277	A	0.5	0.2143	T
5	TIGRP2P73491_rs8806909	6295243	0	0	0	G
5	BICF2P998148	6307113	G	0.5	0.7857	A
5	BICF2S23443345	6324948	C	0	0	A
5	BICF2P623280	6346954	G	0	0	A
5	BICF2S2339277	6355278	G	0	0	A
5	BICF2P1201958	6361572	C	0	0	A
5	BICF2P970174	6371504	G	0	0.2143	A
5	BICF2S23621667	6371874	G	0	0.2143	A
5	BICF2S2367248	6376251	G	0	0.2143	A
5	BICF2S23212839	6382805	G	0	0.2143	A
5	BICF2S22948594	6395969	C	0	0.2143	A
5	BICF2P928430	6406245	C	0	0	A
5	BICF2P104550	6422437	A	0	0.2143	G
5	BICF2P905612	6438626	A	0	0.2143	G
5	BICF2P346474	6442368	A	0	0	G
5	BICF2P821272	6462042	G	0.5	0.7857	A
5	BICF2P496900	6468702	A	0.5	0.7857	G
5	BICF2P1161121	6468991	A	0	0	G
5	BICF2S23342198	6484160	A	0.5	0.8333	G
5	BICF2P386891	6489792	A	0	0	G
5	BICF2P327120	6501229	A	0.5	0.2143	G
5	BICF2P1011341	6511850	C	0	0	A
5	BICF2S23014890	6523125	A	0.5	0.2143	G
5	BICF2S23749922	6542442	0	0	0	A
5	BICF2P1220399	6551490	A	0.5	0.2143	T
5	BICF2P988510	6561550	A	0.5	0.7857	G
5	BICF2P988505	6563412	C	0.5	0.1667	A
5	BICF2P298458	6568259	C	0.5	0.2143	A
5	BICF2P660389	6585039	A	0.5	0.2143	G
5	BICF2S23047171	6600112	A	0	0	G
5	TIGRP2P73496_rs8548167	6611464	0	0	0	A
5	BICF2P609378	6632641	A	0.5	0.2143	G
5	BICF2S23733179	6641594	A	0.5	0.2143	G
5	BICF2P289685	6654666	A	0.5	0.7857	G
5	BICF2S23411666	6666105	A	0.5	0.7857	G
5	BICF2S23250848	6672170	A	0.5	0.2143	C
5	BICF2P939030	6681601	G	0.5	0.2143	A
5	BICF2P612888	6708781	A	0.5	0.7857	G
5	BICF2P570076	6726621	A	0	0	G
5	BICF2S23020630	6734516	A	0.5	0.7857	G
5	BICF2P422814	6739940	A	0.5	0.7857	G
5	TIGRP2P73498_rs8813145	6764276	0	0	0	C
5	G323f51S335	6782936	A	0.5	0.2143	G

5	BICF2S2372976	6802372	G	0.5	0.1667	A
5	BICF2P479084	6811163	A	0.5	0.2143	G
5	BICF2P844114	6822191	G	0	0	A
5	BICF2P1196760	6837959	A	0.5	0.2143	C
5	BICF2S23411284	6846068	G	0.5	0.5714	A
5	BICF2S23516808	6854115	C	0.5	0.2143	G
5	BICF2S23028916	6872999	A	0	0	G
5	BICF2P314907	6881047	A	0	0	G
5	TIGRP2P73499_rs8891394	6890614	0	0	0	A
5	BICF2P385491	6900850	0	0	0	C
5	BICF2P396103	6919931	0	0	0	A
5	BICF2P1052934	6923096	G	0	0	A
5	BICF2P983907	6942016	A	0	0	G
5	BICF2P1291712	6953139	A	0	0	G
5	BICF2P763452	6959608	A	0	0	G
5	TIGRP2P73500_rs8658143	6971242	0	0	0	G
5	BICF2S22926218	6989610	C	0	0	A
5	BICF2P893669	6993873	A	0	0	C
5	BICF2P294775	7004964	A	0	0	C
5	BICF2P923672	7027318	0	0	0	A
5	BICF2P993892	7045753	A	0	0	G
5	BICF2P946105	7051426	G	0	0	A
5	BICF2P563333	7067664	A	0.5	0.5714	G
5	TIGRP2P73501_rs8955616	7075374	0	0	0	C
5	BICF2P997516	7093497	G	0	0	A
5	BICF2P328405	7097117	G	0	0	A
5	BICF2P170610	7108476	G	0	0	A
5	BICF2P1000377	7122325	C	0	0	A
5	BICF2P321784	7135905	G	0	0	A
5	BICF2P301624	7141864	A	0	0	C
5	BICF2P904767	7156459	G	1	0.7857	A
5	BICF2P803057	7172513	C	1	0.7857	A
5	BICF2P34472	7180036	A	1	0.7857	C
5	TIGRP2P73504_rs8659642	7194072	0	0	0	G
5	BICF2P1128068	7203176	A	1	0.7857	G
5	BICF2P1228877	7220674	T	1	0.7857	A
5	BICF2P1228880	7223976	G	1	0.7857	A
5	BICF2P601729	7233793	A	1	1	G
5	BICF2P204690	7246149	A	0	0.2143	G
5	BICF2P1454248	7249608	A	0	0.2143	G
5	BICF2S2368607	7263243	G	0	0	A
5	G324f43S107	7299006	G	0.5	0.4286	A
5	BICF2S23354874	7332927	G	0.5	0.4286	A
5	BICF2P1179832	7340425	0	0	0	A
5	BICF2S23716108	7357415	A	0.5	0.4286	G
5	BICF2S2307909	7366369	G	0	0	C
5	BICF2S23048095	7382306	A	0.5	0.4286	T

5	BICF2S23313988	7391060	G	0.5	0.4286	A
5	BICF2P1216277	7397084	0	0	0	A
5	BICF2P1036955	7407297	A	0.5	0.4286	G
5	BICF2P512060	7411215	A	0.5	0.4286	G
5	BICF2P928381	7425061	C	0.5	0.4286	A
5	BICF2P466850	7450719	A	0.5	0.4167	C
5	BICF2P169441	7460558	G	0.5	0.4286	C
5	TIGRP2P73510_rs9106036	7470123	0	0	0	G
5	BICF2P1281485	7482639	0	0	0	G
5	BICF2P27483	7502526	A	0	0	G
5	BICF2P1216802	7517027	A	0.5	0.4286	G
5	BICF2P1075101	7521234	0	0	0	G
5	BICF2P985803	7532778	G	0.5	0.4286	A
5	BICF2P588552	7551712	G	0.5	0.4286	A
5	BICF2P322252	7574500	A	0	0.2143	G
5	BICF2P877535	7587421	A	0.5	0.4286	C
5	BICF2P633894	7608309	G	0.5	0.4286	A
5	BICF2P1444435	7622557	A	0.5	0.4286	G
5	BICF2P760817	7636126	0	0	0	A
5	BICF2S23616325	7657908	A	0	0	G
5	BICF2P293444	7661842	A	0	0.2143	G
5	BICF2P449192	7671826	G	0	0.2143	A
5	BICF2P355426	7684888	A	0	0	G
5	BICF2S2313550	7693481	G	0	0.2143	A
5	BICF2P684237	7704011	A	0	0	G
5	BICF2P892636	7711426	G	0	0.2143	A
5	BICF2P269678	7721397	G	0	0	A
5	BICF2P306752	7729020	C	0.5	0.4286	A
5	BICF2P650448	7749905	A	0	0.2143	G
5	BICF2P1226388	7756159	G	1	0.7857	A
5	BICF2P1108144	7773174	A	1	0.7857	C
5	BICF2P111587	7784639	G	1	0.7857	A
5	BICF2P1022876	7787722	0	0	0	G
5	BICF2P362486	7800486	A	0	0.2143	G
5	BICF2P1083233	7809960	A	0	0.2143	G
5	BICF2P1113875	7826084	A	0	0.2143	C
5	BICF2P990630	7841252	G	0	0	A
5	BICF2P553048	7847373	G	1	0.7857	A
5	BICF2P441206	7860155	A	0	0	G
5	TIGRP2P73517_rs8784336	7875549	0	0	0	G
5	BICF2P1252730	7895618	A	0	0	G
5	BICF2P823483	7903247	A	1	0.7857	G
5	BICF2P1272902	7913607	A	1	0.7857	G
5	BICF2P259784	7927672	0	0	0	A
5	BICF2G63029786	7940183	A	NA	0.625	C
5	BICF2P1143709	7957120	0	0	0	A
5	G325f44S300	7982325	G	0.5	0.4286	A

5	TIGRP2P73519_rs8546790	8002061	0	0	0	G
5	BICF2P453866	8007493	A	0.5	0.4286	G
5	BICF2S23028486	8028303	0	0	0	G
5	BICF2S23617216	8049849	A	0.5	0.4286	G
5	BICF2S23655418	8054776	G	0	0	A
5	TIGRP2P73520_rs8558250	8069590	0	0	0	G
5	BICF2P1453799	8078703	A	0	0	C
5	BICF2S23345084	8086546	A	0	0	G
5	BICF2P367820	8117843	G	0.5	0.4286	A
5	BICF2P1416575	8123860	A	0	0	G
5	BICF2P786776	8133936	G	0.5	0.4286	A
5	BICF2P595992	8145181	A	0	0	G
5	BICF2P100247	8153182	G	0.5	0.2143	A
5	BICF2P679226	8164493	A	0	0	G
5	BICF2P679228	8165567	A	0	0	C
5	BICF2P679235	8167840	C	0	0	G
5	BICF2P161774	8178275	A	0	0	G
5	BICF2P559023	8181132	C	0.5	0.2143	A
5	BICF2S23331443	8198098	A	0.5	0.2143	G
5	BICF2P1270553	8199726	C	0	0	A
5	BICF2P212434	8220709	A	0	0	G
5	BICF2P1460811	8226617	A	0	0	T
5	BICF2P587342	8236608	C	0.5	0.2143	A
5	BICF2P1190362	8246297	G	0	0	A
5	BICF2P199944	8264978	0	0	0	A
5	BICF2P1031709	8270658	0	0	0	A
5	BICF2P727870	8284381	0	0	0	G
5	BICF2S23516940	8309107	G	0.5	0.7857	A
5	BICF2P393947	8317993	A	0	0.2143	G
5	BICF2P1238336	8330532	A	0	0.2143	G
5	BICF2S23764223	8351353	0	0	0	G
5	BICF2P1433032	8360922	A	0	0.2143	C
5	BICF2P514190	8372980	0	0	0	G
5	BICF2S2374670	8386024	G	0.5	0.5714	A
5	BICF2S23251941	8402221	A	0	0	G
5	BICF2S23648905	8413628	A	0.5	0.5714	G
5	BICF2P807873	8428475	A	0.5	0.2143	G
5	BICF2P778319	8431406	A	0.5	0.2143	G
5	BICF2P577422	8441119	C	0	0.2143	A
5	BICF2P941840	8461462	G	0	0.2143	A
5	BICF2P835938	8474798	A	0	0.1667	G
5	BICF2P547394	8487193	A	0.5	0.2143	G
5	BICF2P596163	8499362	A	0	0	G
5	BICF2P113307	8520585	A	0	0	G
5	BICF2P409041	8525720	G	0	0	A
5	BICF2P425559	8534361	G	0	0.2143	A
5	BICF2P459093	8545161	G	0	0	A

5	BICF2P775495	8575669	G	0	0.2143	A
5	BICF2P1145854	8585886	G	1	0.7857	A
5	BICF2P967481	8600959	A	1	0.8333	G
5	BICF2P1216168	8611182	T	1	0.7857	A
5	BICF2P177246	8616904	0	0	0	G
5	BICF2P1252515	8633038	A	0	0	G
5	BICF2P507935	8644109	G	1	0.7857	A
5	BICF2P38116	8658391	C	1	0.7857	A
5	BICF2P186795	8660653	C	0	0	A
5	BICF2S23042567	8671724	A	1	0.7857	C
5	BICF2P433258	8685927	G	1	0.7857	A
5	BICF2P562411	8696240	A	0	0	G
5	BICF2P170762	8718248	A	0	0	G
5	TIGRP2P73530_rs9107162	8730733	0	0	0	C
5	BICF2S23445477	8761953	G	1	0.7857	A
5	BICF2P1162142	8774202	A	0	0	G
5	BICF2P1162141	8777090	A	0	0	C
5	BICF2P1305555	8790991	0	0	0	A
5	BICF2S2373655	8802527	G	0	0	A
5	BICF2S23539848	8817649	A	0	0	C
5	BICF2P511566	8825848	G	0	0.2143	A
5	BICF2S23650339	8849907	C	0	0.2143	A
5	BICF2P648039	8856895	G	0	0.2143	A
5	BICF2P1120663	8871210	G	0	0.2143	A
5	TIGRP2P73532_rs8771680	8878858	0	0	0	A
5	BICF2S22938380	8897619	A	0	0	G
5	BICF2P1347006	8901998	A	0	0	G
5	BICF2S23251377	8913258	A	0	0	G
5	BICF2P798746	8941225	A	0	0	C
5	BICF2P361727	8955468	A	0	0	G
5	BICF2P1052213	8958697	A	0	0	G
5	BICF2P1075940	8970642	0	0	0	G
5	BICF2P1330496	8982583	A	0	0	G
5	BICF2P969459	8993156	C	0	0	A
5	BICF2P1260756	9003478	A	0	0	G
5	BICF2P1260757	9003790	A	0	0	G
5	BICF2P209036	9014826	A	0	0	G
5	BICF2P231169	9016714	0	0	0	A
5	BICF2P1044913	9033125	G	0	0	A
5	BICF2P807093	9043641	0	0	0	G
5	BICF2S23656242	9060221	0	0	0	A
5	BICF2P419806	9081965	G	0	0.2143	A
5	BICF2P6354	9093402	G	0	0.2143	A
5	BICF2P195002	9097360	A	0	0.2143	C
5	BICF2P599920	9112951	A	0	0.2143	G
5	BICF2S23641666	9120531	A	0	0	G
5	BICF2P694044	9144363	G	0	0	A

5	BICF2P671395	9154232	A	0.5	0.5	G
5	BICF2P274462	9166427	A	0.5	0.5	G
5	BICF2P480018	9179033	A	0.5	0.5	G
5	BICF2P1383988	9192686	A	0.5	0.5	G
5	BICF2S24414079	9210602	0	0	0	C
5	BICF2S233180	9213807	A	0	0	C
5	BICF2P122791	9236017	0	0	0	G
5	BICF2P526985	9260074	0	0	0	G
5	BICF2S23311763	9271502	0	0	0	G
5	BICF2S22929451	9303481	0	0	0	A
5	BICF2P1002841	9305559	0	0	0	A
5	BICF2P696751	9322251	0	0	0	A
5	BICF2P1108003	9327073	A	0.5	0.5	G
5	BICF2P901051	9341108	A	0.5	0.5	T
5	BICF2S234771	9381287	T	0	0	A
5	BICF2P323743	9394659	A	0	0	G
5	BICF2P1347656	9397630	A	0	0	T
5	BICF2S2448481	9409914	0	0	0	G
5	BICF2P1223677	9429066	A	0.5	0.5	G
5	BICF2P1223676	9433040	0	0	0	G
5	BICF2P897594	9454814	0	0	0	A
5	BICF2P695937	9475139	C	0	0	G
5	BICF2P238498	9486787	0	0	0	G
5	BICF2P671327	9489918	0	0	0	A
5	BICF2P236473	9500981	A	0	0	G
5	BICF2P928339	9512590	A	0	0	C
5	BICF2P792620	9531655	G	0	0	A
5	BICF2P446931	9544597	T	0	0	A
5	BICF2P142759	9550347	A	0.5	0.5	G
5	BICF2P1045926	9558009	A	0.5	0.5	G
5	BICF2S23136278	9599696	A	0.5	0.5	G
5	BICF2S23139217	9603848	G	0.5	0.5	A
5	BICF2P574481	9614044	0	0	0	G
5	BICF2S22963731	9630785	A	0	0	T
5	BICF2S23648363	9655372	A	0.5	0.5	G
5	BICF2P1027720	9671740	A	0	0.2143	G
5	BICF2P229894	9690103	G	0	0.2143	A
5	BICF2P27987	9696998	C	0	0	G
5	BICF2P23185	9710306	G	0	0	A
5	BICF2P755609	9719169	A	0	0	G
5	BICF2P403938	9725557	G	0	0	A
5	BICF2P740506	9739332	A	0	0	G
5	BICF2P563884	9745992	G	0	0	A
5	BICF2S22931998	9753213	G	0	0	A
5	BICF2P880291	9775791	G	0.5	0.5	A
5	BICF2P591233	9789678	A	0.5	0.5	G
5	BICF2P989392	9798988	G	0.5	0.5	A

5	BICF2P747550	9812910	A	0.5	0.5	G
5	BICF2S23319496	9840309	A	0.5	0.5	G
5	BICF2P1406785	9846907	G	0.5	0.5	A
5	BICF2S22944200	9861068	G	0.5	0.5	A
5	BICF2P1058372	9877020	A	0.5	0.5	C
5	BICF2P262865	9889825	G	0.5	0.5	A
5	BICF2P783651	9901470	A	0.5	0.5	G
5	BICF2P429415	9909125	G	0.5	0.5	A
5	BICF2P108968	9920932	A	0.5	0.5	G
5	BICF2P1334895	9935296	T	0.5	0.5	A
5	BICF2P793888	9944659	G	0.5	0.5	A
5	BICF2P164592	9950297	A	0.5	0.5	G
5	BICF2P1195515	9965683	0	0	0	A
5	BICF2P233219	9974862	0	0	0	A
5	BICF2S23233557	9993114	C	0.5	0.5	A
5	BICF2P1139266	9994525	0	0	0	C
5	BICF2P417629	10011493	G	0.5	0.5	A
5	BICF2P267691	10022427	0	0	0	C
5	BICF2P849588	10033060	0	0	0	A
5	BICF2P425900	10041907	0	0	0	G
5	BICF2P755037	10056554	A	0	0	G
5	BICF2P876498	10073869	G	0	0	A
5	BICF2S23727124	10099391	G	0.5	0.5	A
5	BICF2S23224141	10108534	A	0.5	0.5	G
5	BICF2P238221	10128161	G	0.5	0.5	A
5	BICF2P713312	10135024	G	0.5	0.5	A
5	BICF2S23647820	10150114	A	0	0	G
5	BICF2P1338552	10161015	A	0.5	0.5	G
5	BICF2S23251688	10172032	A	0.5	0.5	G
5	BICF2P566212	10186513	A	0.5	0.5	G
5	BICF2P339373	10191993	G	0.5	0.5	A
5	BICF2P1075451	10218955	A	0.5	0.5	G
5	BICF2P414980	10223783	G	0.5	0.5	A
5	BICF2P918372	10226190	A	0.5	0.5	G
5	BICF2P1271812	10241643	A	0.5	0.5	G
5	BICF2S23120518	10252621	A	0.5	0.5	G
5	BICF2P754020	10259031	G	0.5	0.5	A
5	BICF2S23451221	10271055	G	0.5	0.5	A
5	BICF2P610605	10290006	G	0	0	A
5	BICF2P1245235	10322417	G	0.5	0.5	A
5	BICF2P888461	10346793	A	0	0	G
5	BICF2S23634726	10350567	C	0	0	G
5	BICF2P970472	10365810	A	0	0	C
5	BICF2S23411584	10373683	G	0	0	A
5	BICF2P1018796	10385293	0	0	0	A
5	BICF2P900105	10397310	C	0	0	A
5	BICF2P509022	10407926	A	0	0	G

5	BICF2P1029684	10421446	G	0	0	A
5	BICF2P256064	10441003	A	0	0	T
5	BICF2P508313	10445445	A	0	0	G
5	BICF2P1063131	10461643	G	0	0	A
5	BICF2S23316058	10465026	A	0.5	0.5	G
5	BICF2P820756	10485924	G	0.5	0.5	A
5	BICF2S23631341	10497290	0	0	0	G
5	BICF2P690235	10505237	G	0	0	A
5	BICF2P1471782	10511987	C	0.5	0.5	G
5	BICF2S23518539	10529652	A	0	0	G
5	BICF2P738079	10534379	A	0	0	G
5	BICF2P1401927	10550916	G	0	0	A
5	BICF2P1198876	10565740	G	0	0	A
5	BICF2S23229297	10574896	A	0	0	G
5	BICF2P1409972	10587628	G	0.5	0.5	A
5	BICF2S23312759	10593209	G	0.5	0.5	A
5	BICF2P1126494	10611371	A	0.5	0.5	G
5	BICF2P1298	10618754	A	0.5	0.5	G
5	BICF2P420675	10624696	A	0	0	G
5	BICF2P420676	10624870	A	0	0	G
5	BICF2P420678	10626038	A	0	0	C
5	BICF2P965823	10648948	G	0.5	0.5	A
5	BICF2P965825	10649581	C	0	0	G
5	BICF2S2331073	10667930	A	0.5	0.5	G
5	BICF2P992296	10689794	A	0	0	G
5	BICF2P1153431	10699593	A	0	0	G
5	BICF2S23025903	10709446	A	0.5	0.5	G
5	BICF2S23519930	10728844	G	0.5	0.5	A
5	BICF2P936127	10738903	A	0.5	0.5	G
5	BICF2P1051614	10749378	A	1	1	G
5	BICF2P181763	10753716	A	0	0	G
5	BICF2S23433857	10774160	G	0	0	A
5	BICF2S23121408	10777123	A	0	0	G
5	BICF2S23537652	10803369	G	0	0	A
5	BICF2S2335445	10817786	A	0.5	0.5	G
5	BICF2S23260147	10835304	A	0	0	G
5	BICF2P1056510	10863587	C	0	0	A
5	BICF2P443528	10868132	0	0	0	A
5	BICF2S23336119	10884067	C	0	0	A
5	BICF2S23544923	10897305	G	0	0	A
5	BICF2P321097	10905328	A	0	0	G
5	BICF2P581289	10924159	A	0.5	0.5	G
5	BICF2P1088190	10929591	G	0	0	C
5	BICF2P1252369	10939048	G	0.5	0.5	A
5	BICF2P1304606	10945409	G	0	0	C
5	BICF2P1382751	10958104	A	0	0	G
5	BICF2P861901	10969082	G	0	0	A

5	BICF2P696509	10979208	G	0.5	0.5	A
5	BICF2P127169	10987063	A	0	0	C
5	BICF2P1116413	10999889	A	0	0	T
5	TIGRP2P69237_rs8716033	11015838	0	0	0	A
5	BICF2P1132629	11018174	C	0.5	0.5	G
5	BICF2P1198736	11039782	A	0.5	0.5	G
5	BICF2S23214566	11040870	A	0	0.2143	G
5	BICF2P91381	11054525	C	1	0.7857	G
5	BICF2P1246599	11070616	G	0	0	A
5	BICF2S23036030	11082019	A	0.5	0.5	G
5	BICF2P801692	11098995	A	0.5	0.5	G
5	BICF2P1266439	11107972	A	0	0	G
5	TIGRP2P69239_rs8760907	11110824	0	0	0	G
5	BICF2S23318127	11124145	A	0	0.2143	G
5	BICF2P569504	11143162	A	0	0	G
5	BICF2S23247073	11172089	A	0	0	G
5	BICF2S23648390	11179986	A	0	0	C
5	BICF2S23134295	11185193	A	0	0	G
5	BICF2S23354345	11192338	0	0	0	G
5	BICF2P677197	11201680	A	0	0	G
5	BICF2P27872	11222952	G	0.5	0.7143	A
5	BICF2P27877	11225752	A	1	1	G
5	BICF2S23545738	11240220	A	0.5	0.125	G
5	BICF2P377323	11250501	A	0.5	0.2857	G
5	BICF2P266391	11267932	C	0.5	0.7143	A
5	BICF2P936883	11283458	0	0	0	G
5	BICF2P786311	11300258	0	0	0	G
5	BICF2P1407423	11305816	G	0.5	0.2857	A
5	BICF2P1235803	11316954	A	0	0	G
5	BICF2P978215	11331372	A	0	0	G
5	BICF2P245720	11350367	A	0	0	G
5	BICF2S23722093	11356448	A	0	0.2143	G
5	BICF2P340539	11365120	G	1	1	A
5	BICF2P77482	11376892	G	0	0	A
5	BICF2P1035987	11380134	A	0	0	G
5	BICF2P1324173	11387458	G	0	0	A
5	BICF2P468971	11402056	0	0	0	G
5	BICF2P195501	11415041	A	0	0	G
5	BICF2P90315	11425115	0	0	0	A
5	BICF2P323435	11442115	G	0	0	A
5	BICF2S22957866	11452821	A	0	0	G
5	BICF2S23221365	11459112	0	0	0	G
5	BICF2P631698	11465760	A	0.5	0.2857	G
5	BICF2P526293	11473340	A	0.5	0.7143	G
5	BICF2P288723	11478157	0	0	0	A
5	BICF2P710978	11491221	0	0	0	G
5	BICF2S2321232	11511412	G	0	0	A

5	BICF2P483462	11524556	C	0	0	A
5	BICF2P1175359	11535735	A	0	0	G
5	BICF2S23350669	11548014	C	0.5	0.2857	A
5	BICF2S2314237	11559341	A	0.5	0.7143	G
5	BICF2P756033	11574376	A	0.5	0.2857	G
5	BICF2P1347622	11588444	C	0.5	0.2857	A
5	BICF2P589843	11596807	A	0.5	0.7143	G
5	BICF2S23030813	11605230	0	0	0	G
5	BICF2S22945821	11620549	C	0.5	0.5	A
5	BICF2P80998	11626631	A	0.5	0.2857	G
5	BICF2P1513296	11641915	A	0.5	0.2857	G
5	BICF2S23915973	11665319	G	0.5	0.5	A
5	BICF2S23255824	11680172	T	0.5	0.7143	A
5	BICF2P214985	11703129	A	0	0.2143	G
5	BICF2P1364834	11709571	G	1	1	A
5	BICF2S23760710	11727282	0	0	0	T
5	BICF2S23211707	11749750	0	0	0	G
5	BICF2S23035109	11757453	A	0	0	G
5	BICF2P143005	11782563	G	0	0	A
5	BICF2S23338809	11788973	G	0.5	0.2857	A
5	BICF2P285774	11806996	A	0	0	C
5	BICF2P1413950	11815077	0	0	0	C
5	BICF2S2318369	11849961	0	0	0	A
5	BICF2P1220267	11879801	0	0	0	G
5	BICF2P1073124	11893611	0	0	0	A
5	BICF2S23227133	11912661	G	0	0	A
5	BICF2P1142329	11933903	0	0	0	A
5	BICF2P389247	11938789	0	0	0	G
5	BICF2P1142810	11954313	A	0	0	C
5	BICF2P1142809	11954874	A	0	0	G
5	BICF2P618001	11963111	A	0	0	C
5	BICF2P441330	11990076	A	0.5	0.5	G
5	BICF2P567779	11995050	0	0	0	G
5	BICF2P473338	12013928	G	0.5	0.5	A
5	BICF2P829190	12030443	0	0	0	G
5	BICF2P689724	12035365	A	0.5	0.5	G
5	TIGRP2P69333_rs9126746	12050756	0	0	0	G
5	BICF2S23525452	12058319	T	0	0.2143	A
5	BICF2P284882	12066205	A	1	0.7857	G
5	BICF2P1333034	12078999	A	0	0.2143	G
5	BICF2P313109	12089358	A	0	0.2143	G
5	BICF2S23544899	12108061	A	0	0	G
5	BICF2P291824	12117732	G	0	0	A
5	BICF2P922080	12129573	A	0	0	C
5	BICF2P1329657	12134149	G	0	0	A
5	BICF2P1471836	12144492	A	0	0	G
5	BICF2S23661616	12156834	A	0	0	G

5	BICF2P542783	12173468	G	0.5	0.2857	A
5	BICF2P658339	12184635	G	0.5	0.2857	A
5	BICF2S2311440	12211740	G	0.5	0.7143	C
5	BICF2S23652232	12226926	A	0.5	0.2857	T
5	BICF2P1210760	12234808	A	0	0	G
5	BICF2P917384	12238082	G	0.5	0.75	A
5	BICF2P270689	12257360	A	0.5	0.7143	G
5	BICF2P1359766	12269705	A	0.5	0.7143	G
5	BICF2S23325138	12269826	A	0	0	G
5	BICF2S23744336	12277232	A	0.5	0.7143	G
5	BICF2P791023	12283960	G	0.5	0.7143	A
5	BICF2S23443188	12303349	A	0	0	C
5	BICF2P1308329	12309350	0	0	0	C
5	BICF2P270740	12335265	0	0	0	G
5	BICF2S23626863	12364679	0	0	0	G
5	BICF2S23542205	12379889	A	0	0	G
5	BICF2P649922	12396622	A	0	0	G
5	BICF2S23355971	12398294	A	0	0	G
5	BICF2P1154998	12409186	G	1	1	A
5	BICF2P1154993	12409662	A	0.5	0.5	G
5	BICF2P1018940	12427619	G	1	1	A
5	BICF2P50276	12442952	A	0.5	0.5	G
5	BICF2P22830	12460594	A	0.5	0.5	G
5	BICF2P1393128	12486652	A	0.5	0.5	G
5	BICF2P758349	12492364	0	0	0	G
5	BICF2P822706	12501391	G	0.5	0.5	A
5	BICF2P829027	12519889	A	0.5	0.5	G
5	BICF2P1435673	12526871	A	0.5	0.5	G
5	BICF2P765257	12547442	A	0	0.2143	G
5	BICF2P715466	12561460	A	0.5	0.5	G
5	BICF2P397272	12565887	G	0.5	0.5	A
5	BICF2P217349	12579201	G	0.5	0.7143	A
5	BICF2P377004	12583708	A	0	0	G
5	BICF2P132228	12594076	A	0	0	G
5	TIGRP2P69341_rs8704434	12626418	0	0	0	G
5	BICF2P517389	12629284	G	0.5	0.5	A
5	BICF2P338054	12647234	G	0	0	A
5	BICF2P1139153	12659103	0	0	0	A
5	BICF2P808029	12664450	G	0	0	A
5	BICF2P720245	12669075	G	0	0	A
5	BICF2P181037	12679814	C	0	0.2143	A
5	BICF2P176768	12694561	A	0	0.2143	G
5	BICF2P728600	12716435	A	0.5	0.5	G
5	BICF2P1232283	12719301	G	0.5	0.5	A
5	BICF2P1367589	12740837	G	1	0.7857	C
5	BICF2P1367586	12742216	A	1	0.7857	G
5	BICF2P216035	12763852	A	1	0.7857	G

5	BICF2P866682	12766047	G	1	0.8333	A
5	BICF2P945343	12776878	G	1	0.7857	A
5	BICF2P870437	12796265	G	0	0	A
5	BICF2P915346	12808553	A	1	0.7857	G
5	BICF2P306969	12821552	A	1	0.7857	G
5	BICF2P1496192	12835431	A	0	0	T
5	BICF2P364550	12855516	C	0	0	A
5	BICF2P460930	12867035	A	0.5	0.5	G
5	BICF2S2338469	12887465	A	1	1	G
5	BICF2S23717676	12902613	A	0.5	0.5	G
5	BICF2P1152382	12904633	0	0	0	A
5	BICF2P24839	12917375	0	0	0	G
5	BICF2S23756307	12934988	G	0.5	0.5	A
5	BICF2S23615385	12972537	G	0	0.2143	A
5	BICF2P906919	12990799	A	0.5	0.5	G
5	BICF2P1182162	12996719	G	1	0.9	A
5	BICF2P813287	13008328	C	0	0.2143	A
5	BICF2S23543795	13027174	A	0.5	0.2857	C
5	BICF2P288040	13037107	0	0	0	A
5	BICF2P490491	13057835	C	0.5	0.5	G
5	BICF2S23611054	13131063	C	0	0.2143	G
5	BICF2P1034442	13152546	G	0	0.1	A
5	BICF2P1034441	13152739	G	0	0.2143	A
5	BICF2P253414	13161629	A	0	0.1667	C
5	BICF2P366738	13166847	G	0.5	0.5	A
5	BICF2P288436	13170075	A	0.5	0.5	G
5	BICF2P83127	13187692	G	0.5	0.5	A
5	TIGRP2P69381_rs8583945	13196090	0	0	0	G
5	BICF2S23514750	13203959	A	0.5	0.5	G
5	BICF2S23124045	13218834	A	0.5	0.5	G
5	BICF2P72123	13239645	A	0.5	0.5	G
5	BICF2S24111973	13254970	A	0.5	0.5	G
5	BICF2S23630064	13274263	A	0	0	G
5	BICF2S23010456	13284621	A	0	0	G
5	BICF2P269780	13310902	G	0	0	A
5	BICF2P1269391	13321812	A	0	0	G
5	BICF2P512578	13343383	A	0	0	G
5	BICF2P727149	13351975	0	0	0	G
5	BICF2P950203	13365755	A	0	0	C
5	BICF2P791607	13384368	0	0	0	A
5	BICF2P684744	13402532	0	0	0	A
5	BICF2P943542	13419838	0	0	0	C
5	BICF2P1151846	13497556	C	0	0	A
5	BICF2P413830	13516566	A	0.5	0.2857	G
5	BICF2P850932	13544403	A	0.5	0.7143	G
5	BICF2P444717	13552002	G	0.5	0.5	A
5	BICF2P1024144	13566348	A	0	0	T

5	BICF2P408893	13577694	G	0	0	A
5	BICF2P152160	13591464	G	0.5	0.5	C
5	BICF2P162841	13595727	0	0	0	A
5	BICF2P1138078	13605079	A	0	0	C
5	BICF2S23015885	13637079	A	0.5	0.2857	G
5	BICF2P550709	13647308	0	0	0	A
5	BICF2S23216861	13665979	A	0	0	G
5	BICF2S23648538	13681280	A	0	0	G
5	BICF2P665240	13701845	A	0	0	G
5	BICF2P1185446	13720366	A	0	0	G
5	BICF2P849943	13723482	A	0	0	C
5	BICF2P316822	13740736	A	0	0	G
5	BICF2P457106	13746466	A	0.5	0.5	G
5	BICF2P467965	13755478	A	1	1	G
5	BICF2P869857	13767480	A	0	0	G
5	BICF2P1100948	13779820	G	0.5	0.5	A
5	BICF2P806481	13794715	0	0	0	G
5	BICF2P901531	13801889	G	0	0	A
5	BICF2P596467	13816803	A	0	0	G
5	BICF2P273659	13833281	G	0	0	A
5	BICF2P512691	13836895	G	0	0	A
5	BICF2P868516	13846175	G	0	0	A
5	BICF2P1199176	13868942	G	0	0.2143	A
5	BICF2P1199173	13869376	G	0	0.2143	A
5	BICF2P556777	13890378	G	0.5	0.2857	A
5	BICF2S23218797	13894798	A	0.5	0.2857	G
5	BICF2P1212200	13903817	A	1	0.7857	G
5	BICF2S23429011	13920211	G	0	0.2143	A
5	BICF2S23029677	13929201	C	0	0.2143	A
5	BICF2P964216	13939240	G	0	0.2143	A
5	BICF2P818584	13954764	G	1	0.7857	A
5	BICF2P916601	13966262	A	0	0	G
5	BICF2P99078	13980665	A	0.5	0.2857	T
5	BICF2P2784	13990533	A	0.5	0.2857	C
5	BICF2P619703	13999150	A	0.5	0.2857	C
5	TIGRP2P69477_rs8667274	14025020	0	0	0	C
5	BICF2P137560	14038114	A	0	0	G
5	BICF2P241141	14042889	A	0.5	0.2857	G
5	BICF2P492967	14055101	A	0.5	0.5	G
5	BICF2S23612390	14076313	A	0	0	C
5	BICF2S2329967	14107488	A	1	1	G
5	BICF2P1041476	14121823	A	0	0	G
5	BICF2P1044813	14131105	A	0	0	C
5	BICF2P567274	14133880	G	0.5	0.2857	A
5	BICF2S23230191	14153105	A	0.5	0.5	G
5	BICF2P25397	14159270	A	0.5	0.2857	G
5	BICF2P241038	14171132	A	0.5	0.2857	C

5	BICF2P582449	14184795	C	1	0.7857	A
5	BICF2P1157823	14239736	A	0	0.2143	G
5	BICF2P1157824	14239872	G	1	0.7857	A
5	BICF2P648926	14251181	A	1	0.7857	G
5	BICF2P757684	14260132	G	0.5	0.5	A
5	BICF2P532123	14275831	A	0	0.2143	G
5	BICF2P139494	14287747	T	0	0.2143	A
5	BICF2P589	14340394	A	0	0.2143	G
5	TIGRP2P69493_rs8575791	14354256	0	0	0	G
5	BICF2P592117	14371373	G	0.5	0.2857	A
5	BICF2P496521	14377816	G	1	0.7857	A
5	BICF2P1033376	14389229	C	0	0	A
5	BICF2P962958	14400316	0	0	0	A
5	BICF2S23142293	14416517	G	0.5	0.2857	C
5	BICF2S23259203	14423750	A	0.5	0.5	C
5	BICF2P1031342	14439940	A	0	0.4286	G
5	BICF2S235436	14444900	A	0	0.4286	G
5	BICF2S23524450	14459436	A	1	0.5714	G
5	BICF2P581323	14469835	C	1	0.5714	A
5	BICF2S23624056	14488048	A	0	0	C
5	BICF2P1347753	14503797	G	0.5	0.2857	A
5	BICF2P970354	14514784	G	0	0.2143	A
5	BICF2P1058242	14545095	0	0	0	G
5	BICF2S22918177	14554811	G	0.5	0.5	A
5	BICF2P1003588	14586210	A	0	0.2143	G
5	BICF2P480962	14595370	G	0	0.2143	A
5	BICF2S23030198	14612489	G	1	0.7857	A
5	BICF2S23331160	14619729	A	0	0.4286	G
5	BICF2P1005050	14639021	G	0.5	0.2857	A
5	BICF2P1272546	14642424	A	0	0	G
5	BICF2P1093029	14651301	A	0	0	C
5	BICF2P1327219	14666146	A	0	0	C
5	BICF2S23617243	14689681	G	0.5	0.5	A
5	BICF2S23419564	14699979	A	0.5	0.2857	G
5	BICF2P114010	14720253	0	0	0	C
5	BICF2S23535303	14740098	A	0	0.2143	G
5	BICF2P736188	14748527	0	0	0	T
5	BICF2S23520119	14759439	G	0	0.2143	A
5	G326f32S322	14786078	A	0	0	G
5	G327f27S309	14830219	0	0	0	C
5	BICF2S23642432	14853533	G	0	0	A
5	BICF2P1353635	14876354	0	0	0	G
5	BICF2P924735	14888727	0	0	0	A
5	BICF2S23482	14902056	A	0	0	C
5	BICF2S23533437	14927242	A	1	1	G
5	BICF2P482141	14952768	C	0	0	A
5	BICF2P607717	14967324	A	1	1	G

5	BICF2P1116982	14977997	G	0	0	A
5	BICF2S23238674	14999278	A	0.5	0.2857	G
5	BICF2P645758	15006237	A	0	0.2143	G
5	BICF2P189890	15009903	G	0	0	A
5	BICF2S23619875	15023115	A	0.5	0.2857	G
5	BICF2S23013685	15041043	0	0	0	G
5	BICF2P91661	15051153	A	0	0.2143	G
5	BICF2P819174	15062440	G	0.5	0.2857	A
5	BICF2P9713	15067750	A	0.5	0.7143	G
5	BICF2S23112461	15086259	C	0.5	0.2857	A
5	BICF2P162666	15104598	G	0	0	A
5	BICF2P634673	15118130	C	0	0	A
5	BICF2P1366853	15123506	A	0	0	C
5	BICF2P651107	15148707	G	0	0	C
5	BICF2P783447	15158075	A	0	0	G
5	BICF2P146232	15169211	G	0	0	A
5	BICF2P930440	15181741	C	0	0	A
5	BICF2P1239880	15191694	A	1	1	G
5	BICF2P1014123	15220105	G	1	1	A
5	BICF2P1413236	15233763	A	1	1	C
5	BICF2P1413235	15234054	G	1	1	A
5	BICF2P170792	15243369	A	0	0	C
5	BICF2P775251	15254166	G	0	0	A
5	BICF2S23746532	15264066	G	1	1	A
5	BICF2P764765	15273223	G	0	0	A
5	BICF2P1418275	15292575	C	0.5	0.7143	A
5	BICF2P632662	15303262	G	0	0.2143	A
5	BICF2P183540	15323098	G	0	0	A
5	BICF2P1162557	15333462	G	0.5	0.5	A
5	BICF2P987262	15347498	G	0.5	0.5	A
5	TIGRP2P69505_rs8566039	15356597	0	0	0	G
5	BICF2S23538747	15391207	A	0.5	0.5	G
5	BICF2S23538670	15395179	A	0.5	0.5	C
5	BICF2P179781	15400072	G	0	0	A
5	BICF2P1061302	15413152	A	0	0	G
5	BICF2S23517126	15423382	G	0	0	A
5	BICF2S23218055	15433708	G	0	0	A
5	BICF2P680751	15448667	C	0	0.2143	G
5	BICF2S23125549	15466604	A	0.5	0.5	C
5	BICF2P386284	15468372	0	0	0	C
5	BICF2P452627	15488541	0	0	0	C
5	BICF2S23619618	15491250	G	1	0.5714	A
5	BICF2P886233	15503336	A	0	0.2143	G
5	BICF2P458557	15519936	G	0.5	0.5	A
5	BICF2S23356373	15525552	G	0.5	0.2857	A
5	BICF2S23524220	15543347	G	0	0	A
5	BICF2P928313	15554569	A	0	0	G

5	BICF2P630915	15567990	A	0.5	0.2857	G
5	BICF2S23741230	15572077	A	0	0	G
5	BICF2P723782	15590020	A	0	0	G
5	BICF2P699194	15597996	A	0	0.2143	G
5	BICF2S23721264	15612535	A	1	0.7857	G
5	BICF2P1233780	15627536	A	0	0	G
5	BICF2S23617616	15638016	C	0.5	0.5	A
5	BICF2P185733	15642829	A	0	0.2143	G
5	BICF2P611465	15654192	A	0.5	0.7143	G
5	BICF2P611464	15654379	G	0.5	0.7143	A
5	BICF2S2294672	15664891	A	1	0.7857	G
5	BICF2P798692	15681298	A	0.5	0.5	G
5	BICF2P812671	15688456	A	0.5	0.2857	G
5	BICF2P40856	15698504	A	0	0	G
5	BICF2P158702	15712481	A	0.5	0.7143	G
5	BICF2P473053	15726242	A	1	0.7857	C
5	BICF2S23742785	15740293	G	0	0	A
5	BICF2P581302	15754734	G	0	0.4286	A
5	BICF2S23036420	15755640	A	1	0.5714	G
5	BICF2S23054630	15776734	A	0	0	C
5	BICF2S23030135	15798741	G	0.5	0.5	A
5	BICF2S22933536	15819129	G	0.5	0.5	A
5	BICF2P226495	15831194	G	0	0	A
5	BICF2S2329683	15842557	C	0.5	0.2857	A
5	BICF2S23249465	15877137	A	0	0	C
5	BICF2S23349696	15893558	G	0.5	0.5	A
5	BICF2P714824	15907438	A	0.5	0.5	G
5	BICF2P714822	15907601	A	0	0.2143	G
5	BICF2P1132921	15920920	A	0	0.2143	C
5	BICF2P103563	15936995	A	1	0.7857	G
5	BICF2P535266	15942976	0	0	0	C
5	BICF2S2305764	15971748	C	1	1	G
5	BICF2S2308953	15993903	A	0	0	G
5	BICF2S23218924	16001655	C	0.5	0.5	A
5	BICF2P249942	16009916	0	0	0	T
5	BICF2P1309564	16025307	0	0	0	C
5	BICF2P1085959	16043319	G	0	0.2143	C
5	BICF2P871146	16071170	A	0	0.4286	G
5	BICF2P1194727	16085937	G	0.5	0.7143	A
5	BICF2P381887	16093715	A	0.5	0.5	G
5	BICF2P381179	16108115	G	0	0	A
5	BICF2P968528	16114280	A	0.5	0.5	G
5	BICF2S2312301	16124082	0	0	0	T
5	BICF2P1248827	16145308	A	0.5	0.5	G
5	BICF2S23149838	16152956	G	1	0.7857	A
5	BICF2P1074015	16164136	G	0	0	A
5	BICF2S23519155	16176369	A	0.5	0.2857	G

5	BICF2P217390	16188647	A	0	0	G
5	BICF2P334044	16188961	A	0	0	G
5	BICF2P226781	16207910	G	0	0	A
5	BICF2P759597	16217217	A	0	0.2143	G
5	BICF2P674329	16227231	A	0	0.2143	G
5	BICF2S2456305	16252559	A	0.5	0.2857	G
5	BICF2P316282	16260762	A	0	0	G
5	BICF2P1314557	16270556	G	0.5	0.2857	A
5	BICF2P222913	16279181	G	1	0.7857	A
5	BICF2P956309	16288151	G	0	0	C
5	BICF2P1146563	16306323	G	0	0.2143	A
5	BICF2P1022032	16312040	A	0	0	C
5	BICF2P1464282	16331678	0	0	0	G
5	BICF2P1206671	16341673	A	1	0.5714	G
5	BICF2P747510	16356561	G	0	0.2143	A
5	BICF2S23214056	16370176	G	0.5	0.5	A
5	BICF2P206682	16370289	T	0.5	0.5	A
5	BICF2P1317432	16377676	A	0.5	0.7143	C
5	BICF2S2351279	16392135	G	0.5	0.5	A
5	BICF2S23256937	16406662	A	0	0.1667	G
5	BICF2S23716181	16421253	C	0	0.2143	A
5	BICF2S22924708	16422891	A	0	0.2143	G
5	BICF2P127170	16440753	A	0.5	0.2857	G
5	BICF2P1356169	16459604	0	0	0	G
5	BICF2P305821	16477646	G	0	0	A
5	BICF2P273153	16490832	C	0	0.4286	A
5	BICF2P640510	16497317	G	0.5	0.7143	A
5	BICF2P1223830	16528977	G	0	0	A
5	BICF2P1061824	16544072	G	0	0	A
5	BICF2P151844	16550552	G	0.5	0.2857	A
5	BICF2P315940	16561832	A	0	0	G
5	BICF2P730019	16572475	G	0	0	A
5	BICF2P2606	16589251	A	0	0	G
5	BICF2P1319138	16599165	G	0	0	A
5	BICF2P330178	16606683	T	0	0	A
5	BICF2P359998	16621016	0	0	0	A
5	BICF2P1119709	16630844	G	0	0	A
5	BICF2P1055574	16644635	G	0	0	A
5	BICF2P1239233	16657564	G	0	0	A
5	BICF2S23653740	16669107	G	0.5	0.2857	A
5	BICF2S23545538	16705288	G	0	0	A
5	BICF2P661268	16717807	C	0.5	0.7143	G
5	BICF2S24512323	16720827	A	0.5	0.7143	G
5	TIGRP2P69530_rs8868101	16732753	0	0	0	C
5	BICF2S23146205	16750668	G	0.5	0.7143	A
5	BICF2P1440841	16761504	0	0	0	C
5	BICF2P481230	16775765	C	0.5	0.7143	A

5	BICF2S235419	16783540	A	0	0	G
5	BICF2P194511	16798879	G	0.5	0.7143	A
5	TIGRP2P69533_rs8898361	16807761	0	0	0	C
5	BICF2S23439535	16822849	G	1	1	A
5	BICF2S2346072	16835856	C	0	0	G
5	BICF2S236874	16836574	G	0	0.2143	A
5	BICF2P1313806	16850124	G	0	0.4286	A
5	BICF2S22940824	16864648	A	1	0.7857	G
5	BICF2P455040	16873608	A	0	0.2143	G
5	BICF2P455039	16874290	T	0	0.2143	A
5	BICF2P521691	16885457	G	0.5	0.5	A
5	BICF2P886910	16899293	A	1	1	T
5	BICF2S22960029	16905090	A	0.5	0.2857	G
5	BICF2S23624113	16927071	A	1	0.7857	G
5	BICF2P267777	16932906	A	0.5	0.5	G
5	BICF2P848578	16935132	G	1	1	A
5	BICF2P426639	16945814	G	0.5	0.2857	A
5	TIGRP2P69534_rs9239806	16959852	0	0	0	C
5	BICF2P1200199	16972277	G	0	0	A
5	BICF2P388745	16977086	A	0	0.2143	G
5	BICF2P1281216	16991371	G	0.5	0.7143	A
5	BICF2S23744535	16998129	A	0	0	C
5	BICF2S23636477	17019216	G	0.5	0.2857	A
5	BICF2P1006740	17026256	A	0	0.2143	G
5	BICF2S23048932	17043462	G	1	0.7857	A
5	BICF2P493586	17061931	0	0	0	C
5	BICF2S2351863	17071878	A	0	0	G
5	BICF2P52254	17078854	A	0.5	0.5	G
5	BICF2P197711	17089516	A	0	0	G
5	BICF2P1326141	17103412	A	1	0.7857	G
5	BICF2P1330255	17118795	G	1	0.5714	A
5	BICF2P793761	17129825	A	1	0.7857	C
5	BICF2P621561	17137029	G	1	0.7857	A
5	BICF2P1046359	17141178	G	1	0.7857	A
5	BICF2P1164762	17148019	A	1	0.7857	G
5	BICF2P641829	17167612	A	0	0	G
5	BICF2P473966	17170109	0	0	0	G
5	BICF2S23626390	17188642	G	0.5	0.2857	A
5	BICF2P136548	17224618	G	0	0	A
5	BICF2P1338128	17232356	A	0	0.2143	G
5	BICF2P496647	17238946	0	0	0	C
5	BICF2S23060275	17257609	A	0	0	C
5	BICF2P1278929	17271563	C	0	0	A
5	BICF2P1391941	17276013	A	0	0	G
5	BICF2P580925	17292710	A	0	0	G
5	BICF2P214288	17304555	0	0	0	G
5	BICF2P812846	17311725	A	0	0	G

5	BICF2P672668	17326526	A	0	0	G
5	BICF2P325835	17336263	0	0	0	C
5	BICF2P1200187	17342682	A	0	0	G
5	BICF2P618794	17366997	A	0	0	G
5	BICF2P1205465	17387751	A	0.5	0.7143	C
5	BICF2P1296120	17396330	A	0.5	0.2857	G
5	BICF2P1105129	17402256	G	0.5	0.2857	A
5	BICF2S23721939	17413749	A	1	0.5714	G
5	BICF2P128112	17444526	A	0.5	0.2857	G
5	BICF2S23614209	17454091	A	0.5	0.5	G
5	BICF2P291151	17462975	A	0.5	0.2857	G
5	BICF2S23012014	17468734	A	0	0.2143	G
5	BICF2P979429	17484092	A	0	0.1	G
5	BICF2P1287877	17495189	A	0	0.2143	G
5	BICF2P328139	17508142	A	0.5	0.5	G
5	BICF2P106780	17517856	A	0	0.2143	G
5	BICF2P587621	17535496	A	0	0	G
5	BICF2P629360	17538058	A	0	0.2143	G
5	BICF2S23744802	17553588	A	0.5	0.5	G
5	BICF2P435454	17571483	A	0.5	0.5	G
5	BICF2P334988	17574480	0	0	0	C
5	BICF2P743460	17590740	A	0.5	0.5	C
5	BICF2S23755153	17600443	G	0	0.2143	A
5	BICF2P1317224	17629845	A	0	0.2143	T
5	BICF2P509046	17651465	C	0	0.1667	A
5	BICF2P877357	17663682	A	0	0.4286	G
5	BICF2P1424470	17673656	0	0	0	A
5	BICF2S23427476	17677230	C	0	0.4286	G
5	BICF2P105169	17689790	A	0	0	G
5	BICF2S2313713	17702510	A	0	0.4286	G
5	BICF2P951758	17726057	G	0.5	0.7143	A
5	BICF2P126981	17735795	G	0.5	0.7143	A
5	BICF2P659775	17737965	A	0.5	0.2857	G
5	BICF2P938162	17753602	0	0	0	C
5	BICF2P1310512	17774478	A	1	1	C
5	BICF2P1310511	17775037	A	1	1	G
5	BICF2P646561	17786364	A	0	0	G
5	BICF2P570408	17796496	A	0	0	C
5	TIGRP2P69542_rs8628467	17803774	0	0	0	C
5	BICF2P1077898	17831850	G	0.5	0.7143	A
5	BICF2P1327161	17837490	A	0	0	G
5	BICF2P1327160	17837536	G	0	0	A
5	BICF2P1129415	17842948	0	0	0	G
5	BICF2P213311	17852808	A	0	0.2143	C
5	BICF2P831940	17861402	A	0	0.2143	G
5	BICF2S2329289	17873995	G	0.5	0.5	A
5	BICF2P1342349	17888928	C	0.5	0.5	A

5	BICF2P962078	17903845	0	0	0	G
5	BICF2S23437546	17916971	C	0	0	G
5	BICF2S23326269	17932186	A	0	0	G
5	BICF2P1236535	17947543	G	0	0	A
5	BICF2S2303704	17955031	A	0	0	G
5	BICF2P1117258	17963578	C	0.5	0.5	A
5	BICF2P712774	17978527	A	0.5	0.7143	C
5	BICF2S23732032	18022536	A	0	0	G
5	BICF2P461292	18062682	G	0	0	A
5	BICF2S23047187	18088532	G	0.5	0.2857	A
5	TIGRP2P69577_rs8861600	18096047	A	0.5	0.2857	G
5	BICF2P787100	18109826	A	0	0	G
5	BICF2P145462	18119285	0	0	0	C
5	BICF2P1392000	18132808	A	0.5	0.2857	G
5	BICF2P987667	18145938	G	0	0.4286	A
5	BICF2P1442735	18154271	0	0	0	A
5	BICF2P725569	18166548	A	0.5	0.2857	G
5	BICF2P221416	18173728	G	0	0	A
5	BICF2P1357842	18184109	A	0.5	0.2857	G
5	BICF2P1056057	18196237	G	0	0	A
5	BICF2P331411	18211749	A	0	0	G
5	BICF2P661430	18216685	0	0	0	G
5	BICF2P912454	18247735	0	0	0	G
5	BICF2P178038	18252488	0	0	0	G
5	BICF2P1408010	18266448	A	0	0	C
5	BICF2P1462644	18275054	0	0	0	A
5	BICF2S24413919	18300453	G	0.5	0.2857	A
5	BICF2S23719163	18332836	A	0	0	G
5	BICF2P768656	18363924	0	0	0	G
5	BICF2P1015227	18396335	G	0	0.4286	A
5	BICF2P558375	18399624	G	0	0.4286	A
5	BICF2P120708	18409048	A	0	0	G
5	BICF2P616630	18411649	0	0	0	A
5	BICF2P1035511	18426858	0	0	0	A
5	BICF2S22932080	18437791	G	0.5	0.7143	A
5	BICF2P799501	18450432	G	0	0	A
5	BICF2S23032018	18464789	A	0.5	0.7143	G
5	BICF2P341482	18475103	0	0	0	C
5	BICF2S23346280	18487145	A	1	0.5833	C
5	BICF2S22911048	18502362	G	1	1	A
5	BICF2P709864	18508143	A	0	0	G
5	BICF2S23114666	18517129	A	0.5	0.7143	G
5	BICF2P878980	18524144	G	0	0	A
5	BICF2P811629	18529567	A	0.5	0.2857	C
5	BICF2P566027	18541317	A	0	0	G
5	BICF2P1221960	18566090	G	0.5	0.2857	A
5	BICF2S23543303	18581678	A	0.5	0.7143	G

5	BICF2S23048431	18589742	A	0.5	0.7143	C
5	BICF2S23338227	18600102	A	0.5	0.7143	G
5	BICF2S237373	18615374	A	0.5	0.7143	G
5	BICF2S2305882	18622992	G	0.5	0.25	A
5	BICF2S23162331	18630879	C	0	0	A
5	BICF2P1376065	18634442	A	0	0	G
5	BICF2S23232833	18634495	A	0	0	G
5	BICF2P531811	18644299	C	0.5	0.2857	A
5	BICF2P831765	18656130	0	0	0	G
5	BICF2S23710878	18686327	A	0	0	G
5	BICF2P450511	18692734	A	0	0	G
5	BICF2G63029793	18700754	C	NA	0	A
5	BICF2G63029807	18715747	G	0	0	A
5	BICF2G63029823	18724955	A	0.5	0.7143	G
5	BICF2S23048653	18731576	C	0	0.4286	G
5	BICF2G63029835	18748496	A	0	0	G
5	BICF2P1224199	18761717	A	0	0	G
5	BICF2G63029880	18775291	A	0	0	C
5	BICF2S2355613	18791200	G	0	0	A
5	BICF2G63029892	18808481	G	1	0.5714	A
5	BICF2S2349130	18817112	C	0.5	0.2857	G
5	BICF2G63029903	18825609	C	0.5	0.75	A
5	BICF2G63029913	18843989	A	0	0.4	C
5	BICF2G63029919	18858001	G	1	0.5714	A
5	BICF2G63029920	18863288	G	0.5	0.2857	A
5	BICF2G63029924	18874733	G	0.5	0.2857	A
5	BICF2S23660104	18884697	G	1	1	A
5	BICF2G63029928	18894656	0	0	0	G
5	BICF2G63029934	18926870	A	0	0	G
5	BICF2G63029938	18931795	C	1	1	A
5	BICF2P230310	18947901	G	0.5	0.7143	A
5	BICF2P700065	18959034	G	0.5	0.2857	A
5	BICF2P920767	18964057	A	0.5	0.7143	T
5	BICF2P392914	18967456	G	0.5	0.2857	A
5	BICF2S23029725	18972939	A	0	0	G
5	BICF2P1201179	18978515	G	0.5	0.7143	A
5	BICF2G63029965	18991838	A	0.5	0.2857	C
5	BICF2G63029976	19001066	G	1	1	A
5	BICF2S24116226	19016878	G	0.5	0.7143	A
5	BICF2G63029995	19024283	A	0	0	G
5	BICF2G63029999	19034368	C	0	0.4286	A
5	BICF2G63030014	19047216	A	0.5	0.7143	G
5	BICF2G63030039	19057555	A	0.5	0.7143	G
5	BICF2P346011	19075972	G	0	0	C
5	BICF2P1057771	19078881	A	0.5	0.7143	G
5	BICF2P46619	19090293	G	0	0	A
5	BICF2P803240	19104994	A	0	0	G

5	BICF2G63030080	19125370	A	0.5	0.2857	G
5	BICF2G63030101	19142824	A	0	0	G
5	BICF2P234679	19151059	A	0	0	G
5	BICF2G63030142	19161617	A	0	0	G
5	BICF2G63030163	19170520	G	0.5	0.2857	A
5	BICF2P215337	19184700	G	0.5	0.2857	A
5	BICF2G63030206	19195420	A	0	0	G
5	BICF2G63030222	19206382	C	1	0.5714	G
5	BICF2G63030243	19227253	G	0	0	A
5	BICF2P1408805	19233830	G	1	0.5714	A
5	BICF2P538198	19242989	A	0	0	G
5	BICF2G63030274	19254463	G	1	0.5714	A
5	BICF2P652567	19263931	A	0.5	0.2857	G
5	BICF2S23651202	19266033	G	0.5	0.2857	A
5	BICF2P970872	19277800	A	0.5	0.2857	G
5	BICF2G63030296	19286890	A	0.5	0.2857	C
5	BICF2G63030320	19302058	A	0	0	G
5	BICF2P229870	19310513	0	0	0	G
5	BICF2G63030324	19320078	A	1	0.5714	G
5	BICF2G63030346	19353890	A	1	0.5714	G
5	BICF2G63030354	19369015	G	1	0.5714	C
5	BICF2S2362330	19381324	C	0	0	A
5	BICF2S239345	19398790	0	0	0	G
5	BICF2P810830	19408020	A	1	1	G
5	BICF2P935231	19420165	G	1	1	A
5	BICF2P466134	19433509	G	1	1	A
5	BICF2P796412	19436463	A	0.5	0.7143	G
5	BICF2P145304	19450700	0	0	0	A
5	BICF2P745390	19467336	A	1	0.5714	G
5	BICF2P286394	19470902	G	1	0.5714	A
5	BICF2P871564	19482016	C	0	0	A
5	BICF2P865713	19503337	G	0.5	0.7143	A
5	BICF2S23634546	19504008	G	0.5	0.7143	A
5	BICF2P576720	19526279	0	0	0	G
5	BICF2P1298918	19529311	G	0.5	0.7143	A
5	BICF2P1145558	19553187	A	0.5	0.7143	G
5	BICF2S23739220	19570007	A	0.5	0.7143	G
5	BICF2P1060034	19578019	G	0.5	0.7143	A
5	BICF2P1144998	19596411	A	0.5	0.7143	G
5	BICF2P1239293	19616857	G	0	0	A
5	BICF2P695835	19619277	C	0	0	A
5	BICF2P271204	19630570	A	0	0	G
5	BICF2S23061242	19650941	0	0	0	G
5	BICF2P244029	19660113	A	0	0	G
5	BICF2P234087	19672855	A	0	0	G
5	BICF2P614043	19682969	G	0	0	A
5	BICF2P931931	19690509	G	0	0.4286	A

5	BICF2P555849	19700697	G	0	0	A
5	BICF2P900569	19717430	G	0	0.4286	A
5	BICF2S23149319	19723215	A	0	0.4286	G
5	BICF2P1392272	19734361	A	0.5	0.2857	G
5	BICF2S23234348	19750323	A	0	0	C
5	BICF2P140503	19759159	G	1	1	A
5	BICF2S23546397	19769451	G	0	0	A
5	BICF2P802193	19789089	0	0	0	G
5	G328f32S18	19809379	G	0	0	A
5	BICF2S23057404	19836717	A	0.5	0.2857	G
5	BICF2P357640	19849654	0	0	0	G
5	BICF2S23052900	19870685	G	0.5	0.2857	A
5	BICF2P1029295	19874676	G	0.5	0.2857	A
5	BICF2S23262	19902916	A	0	0	G
5	BICF2S23045280	19917259	A	0	0	G
5	BICF2P1503117	19940409	G	0	0	A
5	BICF2P536811	19944257	A	0	0.4286	G
5	BICF2P235149	19954059	C	0	0.4286	A
5	BICF2S23539512	19966655	A	0	0.4286	G
5	BICF2P883323	19986370	G	0	0	A
5	BICF2S2356054	19989193	A	0	0.4286	C
5	BICF2P231227	20004706	A	0	0	G
5	BICF2P509473	20021892	A	0	0	G
5	BICF2P125401	20035966	G	0	0	A
5	BICF2P256085	20044972	0	0	0	C
5	BICF2P760579	20060359	A	0.5	0.2857	G
5	BICF2P847338	20068718	A	0.5	0.2857	G
5	BICF2P691547	20076176	A	0.5	0.2857	G
5	BICF2P671065	20081789	A	0.5	0.7143	G
5	BICF2P402885	20092146	G	0.5	0.2857	A
5	BICF2P599310	20110578	A	0.5	0.7143	G
5	BICF2S23422587	20118948	A	0.5	0.2857	G
5	BICF2S23146714	20127171	C	0.5	0.2857	A
5	BICF2P811711	20148442	A	0	0	C
5	BICF2P870087	20170698	A	0.5	0.2857	G
5	BICF2S23049108	20173734	G	0.5	0.2857	A
5	BICF2P948210	20193547	0	0	0	A
5	BICF2P1193309	20198556	A	0.5	0.2857	G
5	BICF2P1171720	20216380	A	0.5	0.2857	G
5	BICF2P579011	20224862	A	0	0	G
5	BICF2P70399	20230718	A	0.5	0.7143	G
5	BICF2S2315603	20241615	0	0	0	G
5	BICF2P931796	20256603	A	0.5	0.2857	G
5	BICF2P1152135	20272083	G	1	1	A
5	BICF2P831528	20272545	T	1	0.5714	A
5	BICF2P831529	20272747	A	1	0.5714	G
5	BICF2P545684	20273767	G	1	1	A

5	BICF2P1446277	20280908	A	0	0	G
5	BICF2P259747	20293309	A	0	0	G
5	BICF2P955060	20298211	A	0	0.4167	C
5	BICF2S23145830	20307502	A	0	0.4286	G
5	BICF2P1207666	20310889	G	1	0.5714	A
5	BICF2P1207667	20311521	A	0	0	G
5	BICF2P452427	20323798	A	0	0.4286	G
5	BICF2S23526949	20338601	A	0	0	C
5	TIGRP2P69613_rs8811156	20354869	0	0	0	G
5	BICF2S2418044	20376058	0	0	0	C
5	BICF2S23627287	20381986	0	0	0	C
5	BICF2S23911685	20395797	G	0	0	A
5	BICF2S23658918	20398452	G	0	0	A
5	BICF2S2291675	20408220	A	0.5	0.2857	G
5	BICF2P549810	20415213	A	0.5	0.2857	G
5	BICF2S23646010	20430463	0	0	0	A
5	BICF2P1358887	20440173	0	0	0	A
5	BICF2S22958286	20447166	G	0.5	0.7143	A
5	BICF2P1158142	20481132	0	0	0	C
5	BICF2S23136398	20488146	C	0	0	A
5	BICF2S23056541	20493161	A	0	0	G
5	BICF2P1070127	20505973	0	0	0	G
5	BICF2P1399588	20522614	A	0	0.4286	G
5	BICF2P340918	20536061	A	1	0.5714	G
5	BICF2P362878	20554896	0	0	0	G
5	BICF2S23234452	20564351	A	1	0.5714	G
5	TIGRP2P69615_rs8928655	20579308	0	0	0	C
5	BICF2S2298845	20603363	A	0	0	G
5	BICF2P1211673	20608729	A	0	0	G
5	BICF2S24415510	20611966	G	0	0	A
5	BICF2P1226363	20624402	G	1	0.5714	C
5	BICF2P1226360	20625590	A	1	1	G
5	BICF2P424906	20634364	A	1	1	G
5	BICF2P888612	20644874	C	0	0	A
5	BICF2P127019	20652288	G	0	0	A
5	BICF2S23241671	20655161	A	0	0	G
5	BICF2S23758654	20672720	A	0	0	G
5	BICF2P1378682	20687184	A	0	0	G
5	BICF2P403755	20700018	A	0	0	G
5	BICF2P1210862	20715662	A	0.5	0.7143	G
5	BICF2P1457147	20723156	A	1	1	G
5	BICF2P1402805	20734824	A	0.5	0.7143	G
5	BICF2P1263839	20746366	A	0	0	G
5	BICF2P7050	20764011	T	0	0	A
5	BICF2P292822	20766982	A	0	0	G
5	BICF2P111218	20773014	A	0.5	0.2857	G
5	BICF2P81438	20793085	G	0.5	0.2857	A

5	BICF2P566020	20811822	C	0.5	0.2857	G
5	BICF2P1070674	20815150	G	0.5	0.2857	A
5	BICF2S23715907	20827332	A	0	0	G
5	BICF2P995795	20840652	A	0.5	0.7143	G
5	BICF2P1362489	20854094	A	0	0	G
5	BICF2P90309	20863350	A	0.5	0.7143	G
5	BICF2P1147840	20882278	A	0	0	G
5	BICF2S23615264	20889617	T	1	1	A
5	BICF2S23019675	20901519	T	0	0.4286	A
5	BICF2S23113315	20913544	A	0.5	0.2857	C
5	BICF2S23514695	20945925	G	1	1	A
5	BICF2P822798	20957596	C	0	0.4286	A
5	BICF2P514215	20973407	G	0.5	0.7143	A
5	BICF2P1155828	20980899	A	0.5	0.7143	G
5	BICF2P379525	20988455	A	1	0.5714	G
5	TIGRP2P69624_rs8975891	21003970	0	0	0	A
5	BICF2P1224761	21023580	G	0.5	0.2857	A
5	BICF2P1311837	21052615	A	0	0	G
5	BICF2P37531	21067005	A	0.5	0.2857	G
5	BICF2P998466	21074952	G	1	1	A
5	BICF2S23016360	21088185	A	0.5	0.7143	G
5	BICF2S23319437	21100621	0	0	0	G
5	BICF2P806011	21107905	G	0	0	A
5	BICF2S2379469	21121572	G	0	0.4286	A
5	BICF2S22945217	21125883	G	0	0.4286	A
5	G329f55S54	21153292	0	0	0	C
5	BICF2S23531075	21179968	G	0.5	0.2857	A
5	BICF2P364029	21191040	A	0.5	0.2857	C
5	BICF2S2292315	21202898	G	0	0	A
5	BICF2P1468408	21210013	C	0.5	0.2857	G
5	BICF2S23260377	21225852	A	0	0	G
5	BICF2S23412053	21238306	G	0.5	0.2857	A
5	BICF2S23419975	21241106	C	0.5	0.2857	A
5	BICF2S23419974	21241159	A	0.5	0.2857	G
5	BICF2P3593	21253826	A	0	0	G
5	BICF2P764469	21263779	0	0	0	A
5	BICF2P547456	21294613	A	0	0	T
5	BICF2P93679	21301218	0	0	0	A
5	BICF2P201050	21320672	A	0.5	0.7143	C
5	BICF2S2412221	21321666	A	0	0	G
5	BICF2S23610734	21342784	G	0	0	A
5	BICF2P400390	21385934	A	0	0	G
5	BICF2S23054765	21390237	G	1	0.5714	A
5	BICF2P899740	21419190	A	0	0.4286	G
5	BICF2P250955	21421149	A	0	0	C
5	BICF2P77676	21435425	G	0	0	A
5	BICF2P77684	21436147	A	0	0	G

5	BICF2S23353079	21467072	A	0	0	G
5	BICF2S2326462	21474045	A	1	0.5714	C
5	BICF2P197830	21482096	0	0	0	G
5	BICF2P401528	21496276	A	0	0	G
5	BICF2P557209	21509131	0	0	0	C
5	BICF2S23158625	21516926	A	0.5	0.2857	G
5	BICF2S23214895	21531909	A	0	0	G
5	BICF2P164676	21549788	A	0	0	G
5	BICF2P1445904	21571520	G	0.5	0.2857	C
5	BICF2P592080	21575271	G	0.5	0.2857	A
5	BICF2P722065	21598031	A	0	0	G
5	BICF2P1035807	21611327	A	0	0	C
5	BICF2P552959	21627436	A	0	0.4286	C
5	BICF2P1454022	21651436	A	0.5	0.2857	G
5	BICF2P907281	21654752	G	0	0	A
5	BICF2P464189	21676267	G	0	0	A
5	BICF2P1328959	21677097	A	0	0	G
5	BICF2S2441785	21709596	A	0.5	0.2857	C
5	BICF2P1107438	21712829	0	0	0	A
5	BICF2P1144946	21731042	A	0	0	C
5	BICF2S23138520	21744401	C	0.5	0.7143	A
5	BICF2P263321	21750467	A	0.5	0.2857	G
5	BICF2P133802	21785799	A	0	0	G
5	BICF2P1332073	21793699	G	0	0	A
5	BICF2P646920	21836061	A	0	0	G
5	BICF2P136066	21845647	A	0	0	G
5	BICF2S231948	21891218	A	0.5	0.7143	C
5	BICF2P399317	21910544	A	0	0	G
5	BICF2P1103144	21927210	A	0	0	G
5	BICF2P312496	21931407	G	0	0	A
5	TIGRP2P69639_rs8579595	21973334	0	0	0	A
5	BICF2P615039	21984396	T	0	0	A
5	BICF2P1322636	21996131	A	0	0	C
5	BICF2P757061	22004465	A	0	0	G
5	BICF2P1266478	22012301	A	0	0	G
5	BICF2P689500	22026410	A	0	0	G
5	BICF2S23537650	22034931	A	0	0	G
5	BICF2P801106	22051566	0	0	0	A
5	BICF2S23417578	22063462	0	0	0	A
5	BICF2P147870	22080544	0	0	0	G
5	BICF2P605094	22092076	A	0.5	0.7143	G
5	BICF2P238421	22104028	A	0.5	0.2857	C
5	BICF2P238425	22104441	A	0.5	0.2857	G
5	BICF2P453352	22125698	G	0.5	0.7143	A
5	BICF2P775454	22132125	G	1	1	A
5	BICF2P1284063	22148829	G	0	0	A
5	BICF2P1189797	22149499	G	0	0	A

5	BICF2P1334463	22157157	G	0.5	0.7143	A
5	BICF2P261357	22167407	G	0.5	0.7143	A
5	BICF2S23238779	22174002	A	0	0	G
5	BICF2P747377	22193527	C	0.5	0.2857	A
5	BICF2P308219	22199430	A	0.5	0.2857	G
5	BICF2S23660065	22207512	C	0	0	G
5	TIGRP2P69643_rs8634020	22227616	0	0	0	G
5	BICF2S23019641	22235030	C	1	1	A
5	BICF2P289755	22241449	C	0.5	0.2857	A
5	BICF2P388196	22260501	A	0	0.4286	G
5	BICF2P1074386	22272484	A	0.5	0.2857	G
5	BICF2P803928	22276218	A	0	0	G
5	BICF2P247124	22296366	0	0	0	T
5	BICF2P1316947	22307571	0	0	0	C
5	BICF2S23738231	22314822	A	0	0	C
5	BICF2P568798	22326153	0	0	0	G
5	BICF2P1252566	22333236	0	0	0	G
5	BICF2S23125122	22350016	A	0	0	G
5	BICF2S23125123	22350240	A	0.5	0.2857	G
5	BICF2P1004839	22357941	A	0.5	0.2857	G
5	BICF2P408488	22369773	0	0	0	A
5	BICF2S23535145	22382190	G	0.5	0.2857	A
5	BICF2S23447493	22451834	A	0.5	0.2857	G
5	BICF2S23047309	22480182	G	0	0	A
5	BICF2P1137716	22490850	A	0	0	G
5	BICF2P93323	22511417	A	1	1	G
5	BICF2S22911439	22528127	A	1	1	G
5	BICF2P722352	22529080	A	1	1	G
5	BICF2P1443513	22547405	A	0	0	G
5	BICF2P658693	22551796	0	0	0	C
5	BICF2P378795	22573520	A	0.5	0.7143	G
5	BICF2P231732	22593398	0	0	0	G
5	TIGRP2P69652_rs8819407	22603487	0	0	0	A
5	G331f25S214	22631634	G	0.5	0.2857	A
5	TIGRP2P69653_rs9157692	22642563	0	0	0	C
5	BICF2P1249104	22654780	G	0.5	0.7143	A
5	BICF2P1249103	22655534	G	0.5	0.7143	A
5	BICF2S23659282	22660745	G	0.5	0.7143	A
5	BICF2S23048457	22674913	C	0	0	G
5	BICF2S23153363	22686458	A	1	1	G
5	BICF2S23727595	22699346	A	0	0	G
5	BICF2P532373	22704412	G	0.5	0.2857	A
5	BICF2P1359630	22714569	A	0.5	0.2857	C
5	BICF2S22912536	22731808	A	0	0	C
5	BICF2S23657115	22739424	G	0.5	0.2857	A
5	BICF2P889900	22748703	A	0	0	G
5	BICF2P548123	22759310	A	0	0	C

5	BICF2P905138	22775537	G	0.5	0.2857	C
5	BICF2S22964504	22782671	G	0.5	0.2857	A
5	BICF2S23126854	22798935	G	0	0.4286	A
5	BICF2P406747	22814678	0	0	0	G
5	BICF2S23119566	22821266	A	0	0	G
5	BICF2P251646	22838646	A	0	0	G
5	BICF2S23753081	22840374	0	0	0	G
5	TIGRP2P69655_rs8622836	22854608	0	0	0	G
5	BICF2P783886	22868846	G	0.5	0.2857	A
5	BICF2P573421	22875384	A	0	0	G
5	BICF2S2446098	22888434	A	0	0	C
5	BICF2P12807	22903498	A	0	0.3333	G
5	BICF2P788683	22912298	G	0	0	A
5	BICF2P1330944	22920343	A	0	0	C
5	BICF2P907815	22932032	G	0	0	A
5	BICF2S23054235	22955359	A	0.5	0.2857	G
5	BICF2P356602	22972716	A	0	0.4286	G
5	BICF2P667205	22989124	A	0	0.4286	G
5	BICF2P797660	23003467	G	0.5	0.7143	A
5	BICF2S23643068	23017584	A	0	0	G
5	BICF2S23643069	23017776	A	0	0	G
5	BICF2S23219334	23023588	A	0	0.4286	T
5	BICF2S23127387	23035558	A	0	0	G
5	BICF2P1343483	23049359	G	0.5	0.7143	A
5	BICF2P755652	23059691	0	0	0	C
5	BICF2S2371950	23074197	0	0	0	G
5	BICF2P140611	23081428	G	0	0	A
5	BICF2S23749664	23097271	A	0	0	G
5	BICF2P1357459	23106719	A	0	0	G
5	TIGRP2P69658_rs8826937	23116213	0	0	0	G
5	BICF2P1130497	23127274	0	0	0	G
5	BICF2S22945346	23144612	G	0	0	A
5	BICF2P1083578	23149876	G	1	0.5714	A
5	BICF2P434622	23161131	0	0	0	A
5	BICF2S23248031	23189410	A	0	0	G
5	BICF2P1165354	23195627	G	0	0	A
5	BICF2S23733495	23222971	0	0	0	C
5	BICF2P1385917	23236599	G	0.5	0.2857	A
5	BICF2P940369	23239971	A	0	0	G
5	BICF2P1089638	23245692	A	0	0.4286	G
5	BICF2P860677	23260631	G	1	1	A
5	BICF2P882260	23275068	G	0	0	A
5	BICF2S2314899	23287392	A	0.5	0.2857	G
5	BICF2S23522523	23287767	A	0	0	C
5	BICF2P1098115	23302624	G	1	1	A
5	BICF2S23427480	23317716	A	1	1	G
5	BICF2S22939484	23328594	A	0	0	G

5	BICF2P676493	23332253	G	NA	0.5	A
5	BICF2P112229	23333613	A	0	0	C
5	BICF2P1331295	23345652	A	0	0	G
5	BICF2S24414248	23363696	0	0	0	A
5	BICF2P221897	23372396	G	0	0	A
5	BICF2S23539360	23389930	A	0	0	G
5	BICF2S2305644	23392169	G	0	0	A
5	BICF2S2327544	23413866	A	0	0	G
5	BICF2P1079949	23430337	G	1	1	A
5	BICF2P266869	23469938	A	0	0	G
5	BICF2P1184979	23491323	G	0	0	A
5	BICF2P1063782	23512148	A	0	0	G
5	BICF2P48564	23518238	G	0	0	A
5	BICF2S23312123	23542528	A	1	1	G
5	BICF2S2325423	23568801	C	0	0.4286	A
5	BICF2S23032291	23579995	G	1	0.5714	A
5	BICF2S23516216	23592502	G	0	0.4286	A
5	BICF2S23346643	23613271	G	0	0.4286	A
5	BICF2S23141799	23616891	A	0	0.4286	G
5	BICF2S23144358	23621134	C	1	0.5714	A
5	BICF2S23151652	23658322	G	1	0.5714	A
5	BICF2S23745382	23690199	G	0	0.4286	A
5	BICF2P217420	23722233	G	1	1	A
5	BICF2P881522	23725191	G	1	1	A
5	BICF2P515969	23746514	G	1	1	C
5	BICF2P677536	23749424	G	0	0	A
5	BICF2P433869	23761387	A	1	0.5714	G
5	BICF2S2397392	23779852	A	0	0	C
5	BICF2P793925	23786655	0	0	0	A
5	BICF2P322045	23803089	C	0	0.4286	A
5	BICF2P163929	23816068	G	0	0.4286	A
5	BICF2P1324622	23823986	G	0	0.4286	A
5	BICF2P449445	23834099	A	0	0.4286	G
5	BICF2P1294313	23845902	G	0	0.4286	A
5	BICF2P637301	23853808	A	0	0.4286	G
5	BICF2P1311372	23875790	0	0	0	C
5	BICF2P682783	23902846	0	0	0	G
5	BICF2P703677	23917114	G	0.5	1	A
5	BICF2P150309	23942921	0	0	0	G
5	BICF2P158502	23975094	C	1	1	A
5	BICF2S23551781	23994536	A	0	0	G
5	BICF2P1186540	24013358	0	0	0	A
5	BICF2S2319358	24027799	A	0	0	G
5	BICF2P959468	24064707	C	1	0.5714	G
5	BICF2P1076197	24082687	0	0	0	A
5	BICF2P301328	24108517	A	1	0.5714	T
5	BICF2P787224	24119541	0	0	0	A

5	BICF2P623587	24133747	0	0	0	G
5	BICF2P1380969	24142445	0	0	0	G
5	BICF2P806412	24156174	0	0	0	G
5	BICF2P217461	24178013	G	0	0	C
5	BICF2P1135026	24184498	A	0	0	G
5	BICF2S23240996	24193742	C	0	0	G
5	BICF2P1070838	24212917	G	0	0	C
5	BICF2P586809	24222868	C	0	0.4286	A
5	BICF2P640581	24239983	A	0	0.4286	G
5	TIGRP2P69696_rs9188236	24253081	0	0	0	C
5	TIGRP2P69697_rs9179016	24259078	0	0	0	G
5	BICF2P35066	24267730	G	0	0.4286	A
5	BICF2P274447	24283242	A	0	0	C
5	BICF2S2348307	24294925	G	1	1	C
5	BICF2P607466	24300979	G	0	0	A
5	BICF2P1152227	24318504	A	0	0	C
5	BICF2P1330729	24326362	A	0	0	G
5	BICF2S23439232	24343167	A	0	0	G
5	BICF2P156965	24351399	0	0	0	C
5	BICF2S22910364	24369104	G	0	0	A
5	BICF2P94579	24381098	A	0	0	G
5	BICF2P1316563	24384478	C	0	0	A
5	BICF2P315042	24402486	C	0	0	A
5	BICF2P786205	24405207	C	0	0	A
5	BICF2S23415880	24418048	C	0	0	A
5	BICF2P296022	24448440	A	0	0	G
5	BICF2P154216	24452544	G	0	0	A
5	BICF2P315632	24467432	G	0	0	A
5	BICF2P424727	24489706	0	0	0	C
5	BICF2P129258	24506467	G	0	0	A
5	BICF2P337183	24508094	G	1	1	A
5	BICF2S23153157	24521527	A	0	0	G
5	BICF2S23752222	24537369	A	0	0	G
5	BICF2S23552348	24546493	A	0	0.4286	G
5	BICF2S22963843	24561513	A	0	0.4286	G
5	BICF2P817755	24599967	A	0	0	G
5	BICF2S24510030	24623301	G	1	0.5714	A
5	BICF2S2297318	24649418	G	0	0	A
5	TIGRP2P69702_rs9196017	24665967	0	0	0	C
5	BICF2S23424951	24676880	A	0	0	G
5	BICF2P1406281	24689306	0	0	0	A
5	BICF2S2375030	24695172	A	0	0	G
5	BICF2P516386	24711502	0	0	0	A
5	BICF2S23130350	24718977	G	1	0.5714	A
5	BICF2P1203071	24725184	A	0	0	G
5	BICF2P304568	24739648	G	0	0	A
5	BICF2S23223770	24751732	A	0	0	G

5	BICF2S23610058	24787253	A	0	0	G
5	BICF2S23231858	24797063	A	0	0	G
5	BICF2S23631513	24805953	A	1	1	G
5	BICF2S23014895	24826416	C	0	0	A
5	BICF2S23241688	24846280	A	0	0	G
5	BICF2P319831	24856569	0	0	0	A
5	BICF2P191437	24867191	A	0	0	G
5	BICF2S22914812	24892966	A	1	1	G
5	BICF2P300662	24900060	G	1	1	A
5	BICF2P1498026	24914704	0	0	0	G
5	BICF2P1292805	24923633	A	1	1	G
5	BICF2P669565	24939307	G	0	0	A
5	BICF2S2325576	24990179	G	0	0	A
5	G332f28S218	25011744	A	0	0	G
5	G332f32S99	25014577	A	0	0	G
5	BICF2P713885	25045841	G	0	0	C
5	BICF2P1395185	25050953	0	0	0	A
5	BICF2S22946078	25061526	0	0	0	G
5	BICF2P591807	25068324	0	0	0	G
5	BICF2P1114638	25070596	0	0	0	A
5	BICF2P464081	25088963	G	0	0	A
5	BICF2P1402492	25095918	0	0	0	G
5	BICF2P72616	25121080	0	0	0	G
5	BICF2P1383599	25129280	0	0	0	C
5	BICF2P365050	25140273	0	0	0	C
5	BICF2P169208	25151865	0	0	0	G
5	BICF2P755067	25179615	G	0	0	A
5	BICF2P374521	25188969	G	0	0	A
5	BICF2P830540	25199894	A	0	0	G
5	TIGRP2P69707_rs9164702	25218834	0	NA	0	C
5	BICF2P220753	25229580	0	0	0	A
5	BICF2P904539	25233068	A	0	0	C
5	BICF2S23647271	25254780	A	0	0	T
5	TIGRP2P69710_rs8782058	25272770	0	0	0	G
5	G333f39S78	25284738	G	0	0	A
5	BICF2P1297399	25313838	G	0	0	A
5	BICF2P1422265	25341310	G	0	0	A
5	BICF2P64139	25364046	G	0	0	A
5	BICF2P564200	25379215	A	0	0	G
5	BICF2P771559	25391442	0	0	0	G
5	BICF2P45748	25404785	0	0	0	A
5	BICF2S24319495	25417355	G	0	0	A
5	G334f43S206	25426015	A	0	0.4286	G
5	BICF2P455561	25455890	A	0	0.4286	C
5	BICF2P1106352	25472493	G	0	0.4286	A
5	BICF2S2293365	25481459	A	0	0.4286	G
5	BICF2P102405	25489392	A	0	0.4286	G

5	BICF2P715624	25505776	0	0	0	A
5	BICF2S23625274	25512583	A	0	0.4286	C
5	TIGRP2P69713_rs8911425	25519946	0	0	0	G
5	BICF2P298913	25537005	G	1	1	A
5	BICF2P899783	25552180	0	0	0	G
5	BICF2S23647041	25563084	A	0	0	G
5	BICF2P1350676	25571922	A	0	0.4	G
5	BICF2P262568	25594017	G	0	0.4286	A
5	TIGRP2P69715_rs8875346	25614782	0	0	0	G
5	BICF2P557902	25648909	A	1	0.6	G
5	BICF2P779759	25651551	G	1	0.5714	A
5	BICF2P329571	25675595	C	1	1	G
5	BICF2P1349041	25682894	G	0	0.4286	A
5	BICF2P1021602	25719154	A	0	0.4286	G
5	BICF2P1196946	25727138	A	0	0.4286	G
5	BICF2P801877	25738126	A	0	0.4286	G
5	BICF2P872476	25750704	G	0	0	A
5	BICF2S24412854	25766770	C	0	0	G
5	BICF2S23658554	25783030	G	0	0	A
5	BICF2P390378	25797086	A	0	0.4286	C
5	BICF2P1104518	25808076	G	0	0.4286	A
5	BICF2S23229964	25847184	C	0	0	A
5	BICF2S23630284	25852550	G	0	0.4286	A
5	BICF2P523710	25877691	A	0	0	C
5	BICF2S23014586	25892221	A	0	0	G
5	BICF2S23758350	25927621	G	1	1	A
5	BICF2P241668	25940750	G	0	0	A
5	BICF2P1333682	25944479	G	0	0	A
5	BICF2P418238	25960472	A	0	0.4286	G
5	BICF2P716998	25969285	A	0	0	G
5	BICF2P360913	25981453	C	0	0.4286	A
5	BICF2P966417	25990649	G	1	0.5714	A
5	BICF2P881601	25991661	G	1	0.5714	A
5	BICF2P449137	26008722	G	1	1	A
5	BICF2S22958152	26026918	G	0	0	A
5	BICF2P1294930	26044902	G	0	0	C
5	BICF2S23710390	26057417	C	0	0	A
5	BICF2P82143	26063661	G	1	0.5714	A
5	BICF2P1207080	26080379	A	0	0	G
5	BICF2P1288841	26082184	0	0	0	A
5	BICF2P1502752	26097814	G	0	0	A
5	BICF2P56980	26103232	A	1	1	G
5	BICF2S23120818	26107153	G	0	0	A
5	BICF2P576246	26121840	0	0	0	G
5	BICF2S2311767	26129976	A	0	0	G
5	BICF2P546213	26142906	C	0	0.4286	A
5	BICF2P548780	26161828	G	0	0.4286	A

5	BICF2S2309025	26169039	A	0	0	C
5	BICF2S23412235	26195837	A	1	0.5714	G
5	BICF2S23412236	26195871	A	0	0	C
5	BICF2P542603	26214365	A	0	0	G
5	BICF2P670557	26222619	G	0	0	C
5	BICF2P1414192	26225852	A	0	0	C
5	BICF2P1070834	26248115	T	0	0	A
5	BICF2P417087	26263814	A	0	0	G
5	BICF2P617374	26270475	G	0	0	A
5	BICF2P1415849	26284567	A	1	0.5714	G
5	BICF2P944064	26292160	G	1	0.5714	A
5	BICF2S22942870	26309746	G	0	0.4286	A
5	BICF2P2785	26316708	G	0	0	A
5	BICF2S23311076	26329210	A	0	0	G
5	BICF2P717258	26344003	A	0	0	G
5	BICF2P576778	26356643	G	0	0	A
5	BICF2P304309	26365242	G	1	1	A
5	BICF2S2325371	26378515	G	1	1	A
5	BICF2P224905	26411070	0	0	0	A
5	BICF2P800504	26441089	0	0	0	A
5	BICF2S23239785	26451489	G	0	0.4286	A
5	BICF2S23714456	26487822	G	1	0.5714	A
5	BICF2S23012314	26500899	A	0	0.4286	G
5	BICF2P1427805	26509599	A	0	0	G
5	BICF2P1414531	26523868	C	0	0	A
5	BICF2S2306878	26540825	A	0	0	G
5	BICF2P646477	26552524	G	1	1	A
5	BICF2P646482	26553040	A	1	1	T
5	BICF2P1448677	26559764	A	0	0	C
5	BICF2S23035952	26573126	A	0	0	G
5	BICF2P313469	26578298	G	0	0.4286	A
5	BICF2P426132	26591727	G	0	0	A
5	BICF2P607832	26610933	G	0	0.4286	A
5	TIGRP2P69733_rs9034885	26615914	0	0	0	A
5	BICF2P971848	26628665	G	0	0	A
5	BICF2P252633	26642061	A	1	1	G
5	BICF2P556436	26649847	A	0	0	G
5	BICF2P204076	26664226	A	0	0.4286	G
5	BICF2P833272	26672843	A	0	0	G
5	BICF2P507262	26682308	A	0	0	G
5	BICF2P592586	26717482	A	0	0	G
5	BICF2S23314564	26728845	A	0	0.4286	G
5	BICF2P248028	26742699	A	0	0	G
5	BICF2P580460	26753955	A	0	0	G
5	BICF2S2332821	26765307	A	0	0.4286	G
5	BICF2P464218	26780696	A	0	0	G
5	TIGRP2P69734_rs9224828	26787868	0	0	0	A

5	BICF2P265017	26804535	0	0	0	G
5	BICF2P251582	26845794	T	0	0.4286	A
5	BICF2P1084718	26861262	0	0	0	A
5	BICF2P252615	26885589	0	0	0	A
5	BICF2P628029	26906493	A	0	0	G
5	BICF2P1182505	26921421	0	0	0	G
5	BICF2P1182500	26923050	A	0	0	G
5	BICF2P1331127	26933123	A	0	0	G
5	BICF2P989189	26951071	0	0	0	C
5	BICF2S23454452	26978369	C	0	0.4286	A
5	BICF2S23733931	26987340	C	0	0.4286	A
5	BICF2P1463130	26998505	A	1	0.5714	G
5	BICF2S23350723	27009611	A	0	0.4286	G
5	BICF2P610157	27017517	G	0	0.4286	A
5	BICF2P673330	27030782	A	0	0	G
5	BICF2P1161390	27044231	A	0	0	G
5	BICF2S23249598	27062919	A	0	0	G
5	BICF2S23658762	27092817	A	1	0.5714	G
5	BICF2P1004327	27110391	A	0	0.4286	G
5	BICF2P307070	27141362	A	0	0.3571	C
5	BICF2P594847	27155428	0	0	0	A
5	TIGRP2P69739_rs8820208	27173105	0	0	0	G
5	BICF2P834292	27175796	A	1	0.6429	G
5	BICF2P547035	27191176	G	1	0.6429	A
5	BICF2P28601	27206322	G	1	0.6429	A
5	BICF2P651744	27212539	G	1	0.6429	A
5	BICF2P953991	27227210	A	1	0.6429	G
5	BICF2P1142001	27237911	C	1	0.6429	A
5	BICF2S22928079	27253812	G	1	0.6429	A
5	BICF2P556324	27258964	G	1	0.6667	A
5	BICF2P388698	27270458	T	1	0.6429	A
5	BICF2P924127	27295366	G	1	0.6429	A
5	BICF2P461903	27303909	A	1	0.6429	C
5	BICF2P1085876	27316610	G	1	1	A
5	BICF2P592421	27329336	A	0	0	G
5	TIGRP2P69743_rs8705751	27341885	0	0	0	A
5	BICF2P255319	27349941	G	0	0	A
5	BICF2P612450	27363368	C	0	0	A
5	BICF2P225117	27371841	G	1	1	A
5	BICF2P559446	27384344	A	0	0	G
5	BICF2S23040872	27438864	G	1	0.6429	A
5	BICF2P343989	27442075	C	1	0.6429	G
5	BICF2P1025859	27464519	0	0	0	A
5	BICF2P987822	27480203	G	1	0.6429	A
5	BICF2P1163306	27489154	G	1	0.6667	A
5	BICF2G63030367	27501577	A	0	0	G
5	BICF2S23347835	27511021	G	1	0.6429	A

5	BICF2G63030421	27525766	G	NA	NA	A
5	BICF2G63030434	27533333	A	0	0	C
5	BICF2G63030458	27550040	A	0	0	G
5	BICF2P202847	27554151	A	0	0.3333	G
5	BICF2G63030473	27566072	T	0	0	A
5	BICF2G63030485	27579278	0	0	0	G
5	BICF2P431566	27594610	0	0	0	G
5	BICF2G63030531	27609458	A	0	0	G
5	BICF2S23546786	27615820	G	0	0	A
5	BICF2G63030558	27627477	A	1	0.6429	G
5	BICF2P1354888	27638338	0	0	0	G
5	BICF2G63030602	27647036	G	0	0	A
5	BICF2G63030615	27658051	G	0	0	A
5	BICF2G63030657	27670399	G	0	0	A
5	BICF2G63030677	27684538	G	0	0	A
5	BICF2G63030689	27718185	A	0	0	G
5	BICF2G63030696	27732913	A	0	0	G
5	BICF2P300693	27747565	A	0	0	G
5	BICF2G63030711	27760860	G	0	0.3571	A
5	BICF2G63030716	27764439	G	0	0	A
5	BICF2G63030723	27773825	A	0	0.3571	C
5	BICF2G63030739	27785589	A	0	0.3571	G
5	BICF2G63030748	27798875	A	1	0.6429	G
5	BICF2S23017403	27809810	A	0	0.3571	G
5	TIGRP2P69774_rs9213759	27818519	0	0	0	G
5	BICF2S23628879	27844079	A	0	0	G
5	BICF2S23058247	27879633	A	1	0.6429	G
5	G335f29S227	27901626	A	0	0	G
5	BICF2P382068	27921917	0	0	0	C
5	BICF2S22933716	27932219	C	1	0.6429	A
5	BICF2S23719564	27942118	A	0	0	G
5	BICF2P418753	27946859	G	0	0	A
5	BICF2P440874	27951377	G	1	0.6429	A
5	BICF2P378499	27963748	A	0	0	G
5	BICF2P985188	27979293	A	0	0	G
5	BICF2P1424898	28000821	A	0	0	G
5	BICF2G63030793	28010388	A	0	0	T
5	BICF2P715543	28015612	A	0	0	C
5	BICF2G63030813	28032072	G	0	0	A
5	BICF2P80306	28047509	G	1	0.6429	A
5	BICF2P80300	28048558	A	1	0.6429	G
5	BICF2P869599	28071198	C	1	1	A
5	BICF2P483742	28076927	G	1	1	A
5	BICF2P225518	28088205	C	1	0.6429	G
5	BICF2P1302785	28095773	G	1	1	A
5	BICF2G63030840	28109441	A	0	0	G
5	BICF2P307301	28127099	A	0	0	G

5	BICF2G63030847	28138119	G	0	0.3571	A
5	TIGRP2P69777_rs8972782	28140761	A	0	0	G
5	BICF2P309660	28162679	G	1	0.6429	C
5	BICF2G63030886	28165760	A	0	0	G
5	BICF2G63030897	28176445	C	0	0	A
5	BICF2G63030917	28190325	C	0	0	A
5	BICF2P1091287	28204673	A	0	0	G
5	BICF2G63030940	28211906	0	0	0	A
5	BICF2G63030973	28230423	A	1	0.6429	C
5	BICF2G63030975	28232604	A	1	0.6429	C
5	BICF2G63031012	28254379	T	1	0.6429	A
5	BICF2G63031013	28256023	A	1	0.6429	G
5	BICF2G63031016	28269753	C	0	0	A
5	BICF2G63031055	28290219	0	0	0	A
5	BICF2G63031081	28313544	A	0	0	G
5	BICF2G63031101	28329067	A	1	0.6429	G
5	BICF2P973462	28337108	A	1	0.6429	G
5	BICF2P204790	28343793	A	1	0.6429	G
5	BICF2G63031118	28358571	A	0	0	G
5	BICF2G63031126	28374980	G	0	0	A
5	BICF2G63031142	28386586	A	0	0.3571	G
5	TIGRP2P69815_rs9065337	28399556	A	1	0.6429	G
5	BICF2P677047	28414140	A	1	0.6429	G
5	BICF2G63031176	28417516	G	1	1	A
5	TIGRP2P69832_rs8987158	28432119	G	0	0.3333	A
5	BICF2P348295	28445035	0	0	0	C
5	BICF2P1344746	28457273	G	0	0.3571	A
5	BICF2P586920	28483722	A	1	0.6429	G
5	TIGRP2P69844_rs9142704	28494065	0	0	0	C
5	BICF2P1338555	28502263	G	1	0.6429	A
5	TIGRP2P69849_rs9120834	28514699	A	1	0.6429	G
5	BICF2G63031191	28522460	G	0	0	A
5	BICF2G63031205	28532781	A	0	0	G
5	BICF2G63031220	28545338	A	0	0	G
5	BICF2G63031266	28565024	G	1	0.6667	A
5	BICF2P485462	28575591	A	0	0.3	C
5	TIGRP2P69870_rs9016534	28583308	G	0	0.3571	A
5	BICF2G63031284	28593405	A	1	0.6429	G
5	BICF2G63031294	28606509	A	0	0	C
5	BICF2S23644595	28614825	G	1	0.6429	A
5	BICF2G63031301	28630505	A	0	0	G
5	BICF2G63031305	28635071	A	0	0	C
5	BICF2G63031322	28657373	A	0	0	G
5	BICF2G63031325	28659812	G	0	0.3571	A
5	BICF2G63031337	28675378	A	0	0	G
5	BICF2G63031349	28683028	G	0	0	A
5	BICF2G63031363	28694072	A	0	0	C

5	BICF2G63031369	28704430	C	0	0	A
5	BICF2G63031429	28755240	G	0	0	A
5	BICF2G63031454	28766959	C	0	0	A
5	BICF2G63031474	28776063	A	1	1	T
5	BICF2S23414114	28801341	A	0	0	G
5	BICF2G63031498	28826010	G	0	0	A
5	BICF2G63031507	28835964	A	0	0	G
5	TIGRP2P69908_rs8806827	28843093	0	0	0	G
5	BICF2G63031523	28854093	C	1	1	A
5	TIGRP2P69919_rs9141849	28870199	A	0	0	G
5	BICF2P23629	28878227	C	0	0	A
5	BICF2G63031567	28889867	A	0	0	C
5	BICF2G63031570	28904298	G	0	0	A
5	BICF2G63031573	28911103	A	0	0.3571	C
5	BICF2G63031584	28928626	C	0	0.3571	A
5	BICF2G63031592	28939731	G	0	0.3571	A
5	BICF2G63031611	28946687	C	0	0	A
5	BICF2P869775	28957026	A	0	0	G
5	BICF2P1109424	28978236	A	0	0	G
5	BICF2G63031632	28988342	A	0	0	C
5	BICF2G63031643	28992412	G	0.5	0.3571	A
5	BICF2G63031655	29007870	G	0	0.3571	A
5	BICF2P536829	29023899	G	0	0.3571	A
5	BICF2S23020902	29037489	A	0	0	G
5	BICF2G63031740	29052752	G	0	0.3571	A
5	BICF2P1221170	29061354	C	0	0	A
5	BICF2G63031781	29080423	A	0	0	G
5	BICF2G63031802	29098927	G	0	0	A
5	BICF2G63031830	29115024	A	0.5	0.2857	G
5	BICF2G63031835	29119914	G	0	0	A
5	BICF2P998739	29130431	C	0.5	0.2857	G
5	BICF2G63031871	29148725	A	NA	0.25	G
5	BICF2G63031880	29160733	T	0.5	0.2857	A
5	BICF2G63031894	29168364	A	0	0	G
5	BICF2G63031908	29180619	C	0	0	A
5	BICF2G63031913	29187154	A	0.5	0.2857	G
5	BICF2G63031930	29201876	G	0	0	A
5	BICF2P994296	29219876	A	0	0	G
5	BICF2P90146	29226500	C	0	0	A
5	BICF2G63031937	29240705	A	0	0	G
5	BICF2G63031951	29248704	A	0.5	0.3571	G
5	BICF2G63031958	29257724	C	0	0	A
5	BICF2G63031971	29273188	G	0	0	A
5	BICF2G63032001	29291802	A	0	0	G
5	BICF2S22950399	29304216	0	0	0	C
5	BICF2G63032048	29314132	G	0	0.3571	A
5	BICF2G63032061	29328486	A	0.5	0.7143	G

5	BICF2G63032074	29336643	G	0	0.3571	A
5	BICF2G63032093	29351324	G	0.5	0.7143	A
5	BICF2G63032105	29361503	A	0.5	0.7143	G
5	BICF2S23241306	29376767	C	0.5	0.3571	A
5	BICF2S2328244	29382982	A	1	0.6429	G
5	BICF2P1087959	29396757	G	0.5	0.2857	A
5	BICF2P1087958	29397019	A	0.5	0.2857	G
5	BICF2G63032163	29409871	A	0.5	0.3571	G
5	BICF2G63032189	29421378	A	0	0	G
5	BICF2G63032193	29429230	A	0.5	0.7143	G
5	BICF2G63032207	29441924	A	0	0	G
5	BICF2S23052973	29451949	A	0.5	0.2857	C
5	BICF2S235100	29470704	A	0	0.3571	C
5	BICF2G63032241	29476582	A	1	1	G
5	BICF2S23222066	29488390	G	0	0	A
5	BICF2P844637	29499337	G	1	0.6429	A
5	BICF2G63032274	29499549	A	0	0	G
5	BICF2G63032279	29505059	G	1	1	A
5	BICF2P962297	29513666	A	1	0.6667	G
5	BICF2P1115567	29521381	A	0	0.3571	G
5	BICF2P1115566	29521583	A	1	0.6429	T
5	BICF2G63032318	29533153	G	0	0	A
5	BICF2G63032335	29547532	G	1	1	A
5	BICF2G63032373	29580964	G	0	0	A
5	BICF2G63032378	29592981	A	0	0	G
5	BICF2G63032382	29601500	A	0	0	G
5	TIGRP2P69980_rs8928235	29613582	0	0	0	A
5	BICF2G63032418	29625075	A	0.5	0.7143	G
5	BICF2G63032424	29640672	A	0.5	0.7143	G
5	BICF2S23619646	29652990	A	0	0	G
5	BICF2G63032434	29683178	G	0.5	0.2857	A
5	BICF2G63032466	29698842	C	0	0.3571	A
5	BICF2P129808	29714885	A	0.5	0.7143	G
5	BICF2G63032495	29724894	G	0.5	0.7143	A
5	BICF2G63032501	29727799	A	0.5	0.7143	G
5	BICF2G63032519	29741123	G	0	0	A
5	BICF2G63032546	29753947	G	0	0.3571	A
5	BICF2G63032559	29765922	A	0	0.3571	G
5	BICF2S23440474	29779607	G	0.5	0.7143	A
5	BICF2S23551885	29791715	G	0	0	A
5	BICF2S22929850	29796951	C	0	0	A
5	BICF2G63032572	29836736	G	0.5	0.6429	A
5	BICF2G63032574	29841770	A	0	0.3571	G
5	BICF2G63032576	29842652	G	0	0	A
5	BICF2G63032599	29855214	G	0	0	A
5	BICF2G63032614	29870576	A	0	0.3571	G
5	BICF2G63032671	29905336	A	1	1	C

5	BICF2G63032689	29911810	C	0	0.3571	G
5	BICF2P905524	29932711	A	0	0.3571	T
5	BICF2G63032709	29935731	G	0.5	0.3571	A
5	BICF2G63032714	29949442	A	0	0.3571	C
5	BICF2G63032728	29966471	G	1	1	A
5	BICF2G63032752	29974876	C	1	1	A
5	BICF2G63032767	29984993	C	0	0	A
5	TIGRP2P69985_rs8533119	29994659	0	0	0	A
5	BICF2G63032804	30004141	G	0	0	A
5	BICF2S23215601	30016833	0	0	0	C
5	BICF2G63032841	30028734	G	0.5	0.3571	A
5	BICF2G63032850	30046409	G	0.5	0.6429	A
5	BICF2G63032860	30054115	G	1	1	A
5	BICF2G63032870	30066572	A	0.5	0.6429	G
5	BICF2G63032873	30076577	A	0.5	0.6429	G
5	BICF2G63032887	30084993	A	1	1	G
5	BICF2G63032888	30085018	C	1	1	A
5	BICF2G63032933	30098167	C	0.5	0.6429	A
5	BICF2G63032973	30107018	G	0.5	0.6429	A
5	BICF2S22935388	30137509	G	1	1	A
5	TIGRP2P69988_rs8806469	30157215	0	0	0	A
5	BICF2S23061646	30172188	G	0	0	A
5	BICF2S23060677	30180065	A	0.5	0.6429	G
5	BICF2G63033001	30210371	A	0	0	G
5	BICF2G63033003	30211671	T	0	0	A
5	BICF2S23726420	30229507	A	0	0	G
5	BICF2S23726606	30233609	0	0	0	A
5	BICF2G63033066	30257395	G	0	0	A
5	BICF2S23161211	30279817	C	0.5	0.3571	A
5	BICF2S23711187	30300619	A	0.5	0.3571	G
5	BICF2G63033082	30305812	0	0	0	G
5	BICF2G63033098	30320304	A	0.5	0.3571	G
5	BICF2G63033117	30333342	A	0.5	0.6429	C
5	BICF2G63033153	30342614	G	0	0	A
5	BICF2G63033159	30352452	G	0	0	A
5	BICF2P333449	30362916	A	0	0	G
5	BICF2G63033185	30374962	A	0	0	G
5	BICF2G63033203	30387234	A	0	0	G
5	BICF2G63033210	30396066	G	0	0	A
5	BICF2P1228901	30411139	A	0	0	G
5	BICF2G63033237	30422600	G	0	0	A
5	BICF2G63033265	30438012	G	0.5	0.3333	A
5	BICF2G63033271	30441037	A	0.5	0.3571	G
5	BICF2G63033285	30448114	A	0.5	0.6429	G
5	BICF2G63033299	30452799	G	0.5	0.3571	A
5	BICF2G63033318	30466533	A	0	0	C
5	BICF2G63033326	30475681	0	0	0	A

5	BICF2P1077938	30487079	A	0	0	G
5	BICF2P840637	30498497	A	0	0	C
5	BICF2G63033332	30512832	A	0.5	0.3571	G
5	BICF2G63033342	30522636	C	0.5	0.3571	A
5	BICF2G63033395	30535262	A	0.5	0.6429	G
5	BICF2P1469725	30537437	A	0	0	G
5	BICF2S23449325	30547078	G	0.5	0.3571	A
5	BICF2G63033434	30561665	G	0	0	A
5	BICF2G63033470	30576872	G	0.5	0.3571	A
5	BICF2G63033477	30579521	G	1	1	A
5	BICF2S23136358	30591943	C	0.5	0.3571	A
5	BICF2G63033484	30601530	G	0.5	0.3571	A
5	BICF2G63033497	30621477	G	0.5	0.3571	A
5	BICF2G63033506	30626672	A	0.5	0.6429	G
5	BICF2G63033513	30637027	A	0	0	G
5	BICF2G63033527	30649699	A	0.5	0.6429	G
5	BICF2G63033569	30662803	A	0.5	0.6429	G
5	BICF2S23410489	30678445	G	NA	0.3571	A
5	BICF2P1207789	30685627	A	0.5	0.3571	G
5	BICF2G63033582	30701936	G	1	1	A
5	BICF2G63033603	30716416	G	1	1	A
5	BICF2G63033608	30718517	G	0	0	A
5	BICF2P1210032	30734633	A	0.5	0.7143	G
5	BICF2P158482	30742888	A	0	0	G
5	BICF2P1472590	30754855	G	1	0.6429	A
5	BICF2G63033621	30766517	G	0.5	0.2857	A
5	BICF2S23335615	30781554	A	0.5	0.3571	G
5	BICF2G63033627	30785799	G	0.5	0.2857	A
5	BICF2G63033640	30799553	G	0	0.3571	A
5	BICF2G63033644	30809466	A	0	0	G
5	BICF2G63033657	30822667	G	0	0	A
5	BICF2G63033670	30831938	A	0.5	0.6429	G
5	BICF2S23314481	30860617	A	0.5	0.6429	G
5	BICF2P476422	30867849	A	0	0.3571	G
5	BICF2G63033741	30878045	A	0	0.3571	G
5	BICF2G63033765	30889860	A	0	0	G
5	BICF2P624217	30900943	A	0	0	G
5	BICF2P406499	30913730	A	0	0	T
5	BICF2G63033784	30941202	G	0.5	0.2857	A
5	BICF2G63033789	30948076	A	0.5	0.7	G
5	BICF2G63033803	30951350	G	0.5	0.6429	A
5	BICF2P709220	30956204	G	0	0	A
5	BICF2G63033818	30958172	G	0.5	0.3571	A
5	BICF2G63033849	30977480	G	0	0.3571	A
5	BICF2G63033858	30984516	G	0	0.3571	A
5	BICF2G63033865	30993290	A	0.5	0.3571	G
5	BICF2G63033883	31011976	0	0	0	A

5	BICF2G63033893	31023864	G	0	0	A
5	BICF2G63033902	31028031	G	0	0.3571	A
5	BICF2G63033903	31028244	A	0	0	C
5	BICF2G63033915	31039120	A	0	0	G
5	BICF2S23627253	31058181	A	0	0.3571	G
5	BICF2G63033930	31062046	A	1	0.6667	G
5	BICF2G63033945	31071417	C	1	1	G
5	BICF2G63033951	31072030	A	0.5	0.3571	G
5	BICF2G63033963	31076976	A	0	0	G
5	BICF2G63033971	31086787	G	1	1	A
5	BICF2G63033987	31097057	A	1	1	G
5	BICF2G63034021	31110576	C	0.5	0.2857	A
5	BICF2P1163372	31125282	C	0	0	A
5	BICF2G63034037	31125456	A	1	1	G
5	BICF2G63034086	31138482	G	0.5	0.7143	A
5	BICF2G63034095	31141565	A	0.5	0.7143	G
5	BICF2G63034108	31147442	C	0.5	0.7143	G
5	BICF2G63034127	31153504	A	0.5	0.7143	G
5	BICF2G63034139	31168636	G	0.5	0.7143	A
5	BICF2G63034160	31177061	A	0	0	G
5	BICF2G63034170	31187132	G	0	0	A
5	BICF2S23523475	31194466	G	0.5	0.2857	A
5	BICF2G63034179	31203884	A	1	1	C
5	BICF2S23148482	31213169	G	1	1	A
5	BICF2G63034206	31222884	A	0	0	G
5	BICF2G63034222	31234383	C	0	0	A
5	BICF2G63034231	31246561	C	0	0	A
5	BICF2G63034255	31254300	C	0	0	A
5	BICF2G63034293	31268440	G	0	0	A
5	BICF2G63034299	31273170	0	0	0	G
5	BICF2P1408545	31278639	G	0.5	0.7143	A
5	BICF2G63034336	31280997	A	0.5	0.7143	G
5	BICF2S23517647	31310408	A	0	0	G
5	BICF2S23426087	31318820	A	0	0	G
5	BICF2G63034379	31343141	A	0.5	0.2857	G
5	BICF2G63034383	31351106	0	0	0	G
5	BICF2P623629	31362258	A	0.5	0.2857	G
5	BICF2P781470	31373326	A	0	0	G
5	BICF2G63034395	31385967	A	0	0	G
5	BICF2G63034416	31403921	A	1	1	G
5	BICF2G63034436	31413282	G	1	1	A
5	BICF2G63034460	31423000	G	1	1	A
5	BICF2G63034481	31434120	A	1	1	C
5	BICF2G63034524	31457670	G	1	1	A
5	BICF2G63034526	31464419	C	1	1	A
5	BICF2G63034550	31489084	G	1	1	A
5	BICF2G63034555	31499340	A	0.5	0.7143	G

5	BICF2G63034575	31529995	0	0	0	A
5	BICF2G63034582	31544323	0	0	0	A
5	BICF2P44411	31545314	C	0.5	0.7143	A
5	BICF2G63034594	31561357	A	0.5	0.7143	G
5	BICF2G63034601	31576703	0	0	0	G
5	BICF2G63034605	31588080	A	0.5	0.7143	G
5	BICF2P610496	31595937	A	0.5	0.7143	G
5	BICF2S22936165	31605748	A	0	0	G
5	BICF2P773632	31615209	T	0.5	0.7143	A
5	BICF2P709414	31633381	G	0.5	0.7143	A
5	BICF2G63034628	31636840	0	0	0	A
5	BICF2G63034631	31653501	0	0	0	G
5	BICF2P223818	31660232	A	0.5	0.7143	G
5	BICF2G63034652	31678326	0	0	0	G
5	BICF2G63034660	31689856	A	0	0	G
5	BICF2G63034666	31696524	C	0	0	G
5	BICF2P1144217	31724860	A	0.5	0.7143	C
5	BICF2G63034681	31729509	G	0	0	A
5	BICF2S22961775	31745277	G	0	0	A
5	BICF2S23235080	31754419	G	0	0	A
5	BICF2S22916604	31772797	G	0.5	0.7143	A
5	BICF2S2314539	31777755	A	0.5	0.7143	G
5	G336f39S126	31802515	0	0	0	C
5	BICF2S23713499	31820074	G	0	0	A
5	BICF2P1349419	31821126	0	0	0	A
5	BICF2S23732925	31838235	G	0.5	0.2857	A
5	BICF2G63034707	31851058	A	0.5	0.2857	T
5	BICF2G63034712	31864346	G	1	1	A
5	BICF2G63034715	31867203	G	1	1	A
5	BICF2G63034729	31882133	A	0	0	G
5	BICF2G63034732	31883823	A	1	1	G
5	BICF2G63034742	31891009	G	0	0	A
5	BICF2P142455	31909726	G	0.5	0.2857	C
5	BICF2G63034778	31916573	A	0.5	0.2857	G
5	BICF2P367560	31930804	0	0	0	A
5	BICF2S23057738	31955509	G	0	0	A
5	BICF2G63034795	31972325	G	0.5	0.7143	A
5	BICF2G63034802	31984377	G	1	1	A
5	BICF2G63034824	31994049	A	1	1	G
5	BICF2G63034825	31994119	A	0	0	G
5	BICF2G63034848	32005227	A	0.5	0.7143	C
5	BICF2P644940	32018566	G	0.5	0.7143	A
5	BICF2S23351182	32029995	G	0	0	A
5	BICF2P1255456	32049403	A	0.5	0.6	T
5	BICF2P948952	32054008	G	0.5	0.8	A
5	BICF2P297927	32068916	G	0	0	A
5	BICF2P497913	32075807	G	1	1	A

5	BICF2G63034889	32096001	G	1	1	A
5	BICF2G63034907	32104199	A	0.5	0.7143	G
5	BICF2P893461	32114865	G	0	0	C
5	BICF2G63034943	32125897	A	1	1	G
5	BICF2G63034949	32140044	G	0.5	0.2857	A
5	BICF2G63034955	32143993	A	0	0	C
5	BICF2G63034972	32159024	A	0	0	C
5	BICF2G63034979	32166446	0	0	0	G
5	BICF2G63035005	32182654	G	0.5	0.2857	A
5	BICF2G63035030	32197936	A	0.5	0.7143	G
5	BICF2G63035039	32200727	A	0	0	G
5	BICF2S23034980	32215439	G	0	0	A
5	BICF2G63035069	32227113	G	0	0	A
5	BICF2G63035080	32242056	A	0	0	G
5	BICF2P853011	32246812	A	0.5	0.2857	G
5	BICF2G63035105	32266043	A	0	0	G
5	BICF2G63035108	32270051	A	0	0	G
5	BICF2S23435595	32270135	G	0	0	A
5	BICF2G63035153	32282151	G	0	0	A
5	BICF2G63035174	32289895	G	0	0	A
5	BICF2G63035178	32292911	C	0	0	A
5	BICF2G63035229	32305333	A	0	0	G
5	BICF2S23741834	32308206	A	0	0	G
5	BICF2G63035269	32315955	G	0	0	A
5	BICF2G63035282	32327925	G	0	0	A
5	BICF2G63035298	32338735	G	0	0	A
5	BICF2P209517	32359028	A	0	0	G
5	BICF2G63035338	32367525	A	0	0	C
5	BICF2G63035343	32373488	A	0	0	G
5	BICF2G63035352	32389983	G	0	0	A
5	BICF2G63035363	32397199	G	1	1	A
5	TIGRP2P70116_rs9132150	32436246	0	0	0	G
5	G337f67S283	32457828	G	0	0	A
5	BICF2S23212122	32486180	A	0	0	C
5	BICF2S23049729	32498150	A	0	0	T
5	BICF2P564732	32509030	A	0	0	C
5	BICF2P531159	32513704	0	0	0	A
5	BICF2S23058014	32531966	A	1	1	G
5	BICF2S23056870	32535803	G	1	1	A
5	BICF2P190310	32545774	G	0	0	A
5	BICF2P875162	32557813	A	0	0	G
5	BICF2G63035369	32578583	0	0	0	A
5	BICF2P115675	32580081	A	1	1	G
5	BICF2P1209136	32596236	G	1	1	A
5	BICF2G63035376	32611758	A	0	0	G
5	BICF2G63035383	32622509	A	0	0	T
5	BICF2G63035403	32632285	G	0	0	A

5	BICF2G63035423	32666562	A	0	0	G
5	BICF2P1403452	32674842	A	1	1	G
5	BICF2G63035429	32677016	A	0	0	G
5	BICF2G63035445	32688767	G	0	0	A
5	BICF2S2324177	32691619	G	1	1	A
5	BICF2P1257162	32704081	A	1	1	C
5	BICF2G63035476	32708612	G	0	0	A
5	BICF2S23317145	32725862	G	0	0	A
5	BICF2G63035506	32739976	A	0	0	G
5	BICF2P271455	32743994	G	1	1	A
5	BICF2P1405079	32757545	A	1	1	C
5	BICF2G63035510	32757807	C	1	1	A
5	BICF2G63035542	32771537	G	1	1	A
5	BICF2P363092	32772158	G	1	1	A
5	BICF2G63035558	32781895	G	1	1	A
5	BICF2G63035564	32787898	A	1	1	G
5	BICF2G63035577	32804686	G	1	1	A
5	BICF2G63035590	32812469	A	0	0	G
5	BICF2G63035622	32825677	G	0	0	A
5	BICF2G63035634	32839388	G	0	0	A
5	BICF2G63035639	32845055	G	0	0	A
5	BICF2G63035674	32858771	A	0	0	G
5	BICF2G63035700	32876294	C	0	0	A
5	BICF2G63035705	32879166	C	0	0	A
5	BICF2G63035726	32901346	G	1	1	A
5	BICF2G63035729	32902463	G	1	1	A
5	BICF2G63035735	32907940	A	0	0	C
5	BICF2G63035745	32915280	A	1	1	G
5	BICF2G63035754	32926679	G	0	0	A
5	BICF2P1376728	32938943	0	0	0	A
5	BICF2G63035778	32954927	A	1	1	G
5	BICF2S235971	32959073	A	1	1	G
5	BICF2G63035789	32972070	A	1	1	G
5	BICF2S232422	32983613	A	1	1	G
5	BICF2G63035829	32998418	G	0	0	A
5	BICF2P93507	33009401	A	1	1	G
5	BICF2S23250257	33017317	G	1	1	A
5	BICF2G630182095	33066504	A	0	0	G
5	BICF2G630182101	33087875	0	0	0	G
5	BICF2G630182112	33109469	0	0	0	G
5	BICF2G630182114	33111397	0	0	0	G
5	BICF2G630182116	33112623	G	0	0	A
5	BICF2G630182122	33114868	G	0	0	A
5	BICF2G630182126	33121641	A	0	0	C
5	BICF2G630182130	33132321	A	0	0	G
5	BICF2G630182143	33145023	A	0	0	G
5	BICF2G630182161	33161069	G	0	0	A

5	BICF2G630182163	33172996	A	0	0	C
5	BICF2G630182165	33180027	A	0	0	G
5	BICF2G630182171	33194533	G	0	0	A
5	BICF2G630182175	33200436	A	0	0	G
5	BICF2G630182178	33205153	A	0	0	G
5	BICF2G630182181	33222993	0	0	0	G
5	BICF2G630182182	33223759	0	0	0	G
5	BICF2P244956	33235295	A	0	0	G
5	BICF2G630182187	33249352	T	0	0	A
5	BICF2G630182191	33271775	A	0	0	G
5	BICF2P342766	33306736	A	1	1	G
5	TIGRP2P70256_rs8990724	33336430	0	0	0	C
5	BICF2S23641676	33349773	G	0	0	A
5	BICF2P18343	33382614	0	0	0	A
5	BICF2P860608	33392133	0	0	0	G
5	BICF2G630182200	33404832	A	0	0	C
5	BICF2P1339473	33411030	A	0	0.1667	C
5	BICF2P584586	33464861	0	0	0	G
5	BICF2G630182204	33481797	T	0	0	A
5	BICF2G630182217	33489043	A	0	0	G
5	BICF2G630182219	33505032	A	1	1	G
5	BICF2G630182225	33523633	A	1	1	G
5	BICF2G630182263	33542974	A	0	0	C
5	BICF2S23647232	33555229	A	0	0	C
5	BICF2S233851	33565857	0	0	0	A
5	BICF2S23135707	33576622	A	0	0	G
5	BICF2P113597	33591032	G	0	0	A
5	BICF2P949256	33599543	G	1	1	A
5	BICF2G630182280	33610155	0	0	0	G
5	BICF2P509283	33617005	G	0	0.3571	A
5	BICF2P891223	33626796	A	0	0	C
5	BICF2P475219	33641408	C	0	0.3571	A
5	BICF2G630182288	33656088	A	0	0	G
5	BICF2G630182300	33667537	G	0	0	A
5	BICF2S23722402	33682368	A	1	1	G
5	BICF2G630182317	33694580	A	0	0	G
5	BICF2P711366	33695207	C	0	0	A
5	BICF2S23756179	33706617	A	1	1	G
5	BICF2G630182331	33724420	A	1	1	G
5	BICF2G630182334	33734017	G	1	1	A
5	BICF2G630182339	33744815	G	1	1	A
5	BICF2S23351431	33758285	A	1	1	G
5	BICF2S23712534	33774147	G	0	0	A
5	BICF2P534414	33778368	A	0	0	G
5	BICF2G630182351	33787544	A	1	1	G
5	BICF2G630182360	33802647	G	1	1	A
5	TIGRP2P70281_rs9009889	33815843	0	0	0	C

5	BICF2P387265	33822236	C	0	0	A
5	BICF2G630182371	33841293	G	0	0	A
5	BICF2G630182377	33855235	A	0	0	G
5	BICF2G630182387	33863122	A	0	0	G
5	BICF2G630182394	33872520	G	1	0.6429	A
5	BICF2G630182408	33885310	A	0	0	G
5	BICF2G630182416	33900370	A	0	0	G
5	BICF2G630182417	33902404	A	0	0	C
5	BICF2G630182419	33907693	A	0	0	G
5	BICF2G630182423	33920793	A	1	1	G
5	BICF2S2294378	33932564	A	1	0.6429	G
5	BICF2S23426541	33941015	0	0	0	G
5	BICF2S23034711	33948691	C	1	0.6429	A
5	BICF2P309809	33951606	A	0	0	G
5	BICF2G630182434	33958216	A	0	0.3571	G
5	BICF2P165957	33960444	A	0	0	G
5	BICF2G630182458	33980891	A	0	0	G
5	BICF2S23339487	33983392	A	1	1	C
5	BICF2G630182460	33995804	A	0	0	G
5	BICF2S23036463	34009871	A	1	1	G
5	BICF2G630182484	34023117	A	0	0	G
5	BICF2G630182500	34032870	G	1	1	A
5	BICF2P58417	34042443	G	1	1	A
5	BICF2P849914	34053245	C	1	1	A
5	BICF2P1243852	34066265	C	1	1	A
5	BICF2P1243844	34067927	G	1	1	A
5	BICF2P47085	34082534	0	0	0	G
5	TIGRP2P70316_rs9232068	34090631	A	1	1	G
5	BICF2P961576	34108486	G	1	1	A
5	BICF2P1384061	34110469	G	1	1	A
5	BICF2P1345275	34124126	A	1	1	G
5	BICF2P94109	34157574	A	1	1	G
5	BICF2P328828	34167997	A	1	1	G
5	BICF2P1086169	34181930	A	1	1	G
5	BICF2P1086165	34183023	C	0	0	G
5	BICF2S23431976	34199594	0	0	0	A
5	BICF2P739313	34211721	A	0	0	G
5	BICF2P495815	34213282	A	0	0	G
5	BICF2P1245098	34234918	0	0	0	C
5	BICF2P1298063	34240012	G	0	0	A
5	BICF2P646287	34247685	C	0	0	A
5	BICF2S23353822	34265402	A	0	0	G
5	BICF2P170841	34275412	0	0	0	T
5	TIGRP2P70360_rs8802599	34291290	0	0	0	A
5	BICF2G630182542	34306753	C	1	1	A
5	BICF2P1270366	34336828	A	0	0	G
5	BICF2P1226001	34340133	0	0	0	C

5	BICF2P576634	34364903	0	0	0	G
5	BICF2P449694	34373710	A	0	0	G
5	BICF2S2332602	34390597	A	1	1	G
5	BICF2G630182563	34407893	A	0	0	T
5	BICF2P1217448	34411959	A	1	1	G
5	BICF2P291957	34430217	A	0	0	G
5	BICF2P1115042	34434701	T	1	1	A
5	BICF2S23353896	34453995	C	1	1	A
5	BICF2P791484	34463023	G	0	0	A
5	BICF2P154622	34480550	A	0	0	G
5	BICF2G630182569	34490530	A	0	0	G
5	BICF2G630182583	34512211	T	0	0	A
5	BICF2G630182589	34523422	A	0	0	G
5	BICF2G630182604	34538796	A	0	0	G
5	BICF2G630182609	34548761	A	0	0	G
5	BICF2S23416100	34552051	G	0	0	A
5	BICF2G630182626	34558314	A	0	0	G
5	BICF2G630182632	34569605	0	0	0	C
5	BICF2G630182636	34589470	A	1	1	G
5	BICF2S23544401	34603343	G	0	0	A
5	BICF2G630182650	34607317	A	1	1	G
5	BICF2G630182654	34624101	G	1	1	A
5	BICF2G630182655	34631197	G	1	1	A
5	BICF2G630182658	34647442	G	0	0	A
5	BICF2G630182661	34652148	G	1	1	A
5	TIGRP2P70367_rs9004740	34662362	0	0	0	A
5	BICF2P692633	34675781	A	1	1	G
5	BICF2G630182675	34704328	G	1	0.6429	A
5	BICF2S23443053	34710722	A	0	0.3571	G
5	BICF2G630182679	34720473	G	1	0.6429	A
5	BICF2G630182687	34738198	A	1	0.6429	G
5	BICF2S23443332	34741886	0	0	0	G
5	BICF2P1312293	34760753	A	0	0	G
5	BICF2G630182729	34796613	A	0	0	C
5	BICF2G630182746	34808975	G	0	0	A
5	BICF2G630182755	34814503	A	0	0	G
5	BICF2P254932	34826160	0	0	0	G
5	BICF2P450384	34841789	0	0	0	G
5	BICF2P496810	34851816	0	0	0	C
5	BICF2S23631833	34879011	0	0	0	C
5	BICF2S2349724	34886283	0	0	0	G
5	BICF2G630182762	34892243	A	1	1	G
5	BICF2G630182765	34912496	A	0	0	C
5	BICF2G630182771	34916736	A	0	0	G
5	BICF2G630182801	34933874	C	0	0	A
5	BICF2G630182805	34948327	G	1	1	A
5	BICF2G630182807	34955867	A	0	0	C

5	BICF2G630182810	34960599	G	0	0	A
5	BICF2G630182831	34980333	A	0	0	G
5	BICF2G630182835	34988366	T	0	0	A
5	BICF2G630182839	34998591	C	0	0	A
5	BICF2G630182847	35010844	G	0	0	A
5	BICF2G630182861	35018779	A	0	0	G
5	BICF2G630182874	35029528	G	0	0	A
5	BICF2G630182886	35056533	A	0	0	G
5	BICF2S2361137	35075059	0	0	0	G
5	BICF2P1149896	35092108	0	0	0	C
5	BICF2G630182901	35108429	A	0	0	C
5	BICF2G630182902	35110780	G	0	0	A
5	BICF2G630182934	35139762	C	0	0	G
5	TIGRP2P70373_rs9067743	35148325	0	0	0	C
5	BICF2G630182944	35155584	A	0	0	G
5	BICF2G630182945	35171523	C	0	0	A
5	BICF2G630182947	35186711	A	0	0	T
5	TIGRP2P70375_rs9205113	35197029	0	0	0	G
5	BICF2S23652976	35203942	A	0	0	T
5	BICF2P1133895	35227099	0	0	0	A
5	BICF2G630182952	35267557	A	0	0	G
5	BICF2S23323312	35270649	A	0	0	C
5	BICF2S23630802	35313124	G	0	0	A
5	BICF2P698330	35317283	0	0	0	C
5	BICF2P380586	35329108	0	0	0	G
5	BICF2S23649880	35348610	G	0	0	A
5	BICF2G630182974	35352729	G	0	0	A
5	BICF2P1211313	35370670	G	0	0	C
5	BICF2G630182979	35384970	G	1	1	A
5	BICF2G630182980	35396466	0	0	0	C
5	BICF2P803532	35399480	A	0	0	T
5	BICF2P1099503	35416892	A	0	0	G
5	BICF2P125222	35421800	G	0	0	A
5	BICF2P798838	35439870	G	0	0	A
5	BICF2S23721153	35454577	G	0	0	A
5	BICF2P557550	35468371	A	0	0	C
5	BICF2G630182991	35479814	A	1	1	C
5	BICF2P621135	35496567	A	0	0	G
5	BICF2P734687	35504251	G	0	0	A
5	BICF2P974089	35545353	0	0	0	A
5	BICF2P1023292	35551827	0	0	0	C
5	BICF2P902647	35561689	0	0	0	G
5	BICF2P516942	35589042	0	0	0	G
5	BICF2P232753	35593850	0	0	0	G
5	BICF2S23626288	35609132	A	0	0	G
5	BICF2P84424	35623993	0	0	0	C
5	BICF2P538531	35636542	G	0	0	A

5	BICF2P1256516	35640937	A	0	0	G
5	BICF2P1150834	35681646	G	0	0	A
5	BICF2P963308	35706834	0	0	0	C
5	BICF2P168496	35740097	0	0	0	G
5	BICF2P1051264	35751575	0	0	0	G
5	BICF2P338628	35755815	G	0	0	A
5	BICF2S23656118	35781878	A	0	0	G
5	BICF2G630183016	35795481	A	1	1	C
5	BICF2G630183027	35806461	A	0	0	G
5	BICF2P763491	35818300	A	0	0	G
5	BICF2G630183045	35834085	C	0	0	A
5	BICF2P99750	35855279	C	0	0	G
5	BICF2G630183070	35865449	A	1	1	G
5	BICF2G630183073	35871340	A	0	0	G
5	BICF2G630183083	35889481	T	0	0	A
5	BICF2G630183089	35897353	G	1	1	A
5	BICF2P1231200	35915206	G	1	1	A
5	BICF2S23412476	35920124	A	1	1	G
5	BICF2G630183104	35926254	A	0	0	C
5	BICF2P645423	35940905	A	1	1	C
5	BICF2G630183107	35958915	A	1	1	G
5	BICF2S23449369	35963813	G	1	1	A
5	BICF2G630183111	35985186	A	0	0	G
5	BICF2P639123	35995857	G	0	0	A
5	BICF2G630183119	35995970	C	1	1	A
5	BICF2G630183123	36015910	A	0	0	G
5	BICF2G630183126	36021759	C	0	0	A
5	BICF2P215448	36040214	A	0	0	C
5	BICF2S2325790	36081874	G	1	0.6429	A
5	BICF2P731728	36098191	G	0	0.3571	C
5	BICF2S23242721	36104924	G	1	0.6429	A
5	BICF2G630183134	36115718	A	1	0.6429	G
5	BICF2G630183158	36129586	C	0	0	A
5	BICF2G630183160	36139809	C	0	0.3333	A
5	BICF2S2333678	36149650	A	0	0	G
5	BICF2G630183175	36159293	A	0	0	G
5	BICF2P163422	36167824	A	1	0.6429	G
5	BICF2G630183195	36180581	G	1	1	A
5	BICF2G630183204	36191301	C	0	0.3571	A
5	BICF2G630183230	36220358	G	0	0	A
5	BICF2S2309606	36225969	G	0	0	A
5	BICF2G630183260	36237561	A	0	0.3571	G
5	BICF2G630183278	36253204	A	0	0	G
5	BICF2G630183286	36267981	A	0	0	G
5	BICF2G630183294	36275423	A	0	0.3571	G
5	BICF2G630183307	36286782	A	0	0	G
5	BICF2G630183311	36297512	G	0	0	A

5	BICF2G630183316	36308339	A	0	0.3571	G
5	BICF2S23628022	36323725	0	0	0	G
5	BICF2G630183322	36329420	G	0	0.3571	A
5	BICF2P464907	36347068	A	0	0	G
5	BICF2G630183333	36380114	A	0	0	G
5	BICF2G630183337	36386455	A	0	0.3571	G
5	BICF2G630183354	36417176	A	0	0.3571	G
5	BICF2G630183360	36424277	A	0	0	G
5	BICF2P974193	36432179	G	0	0.3571	A
5	BICF2G630183370	36433846	G	0	0	A
5	BICF2G630183385	36448165	A	0	0	G
5	BICF2G630183412	36455085	A	0	0	G
5	BICF2P908870	36466881	A	1	1	G
5	BICF2P52587	36476657	A	1	0.6429	C
5	BICF2G630183440	36483249	A	0	0	G
5	BICF2G630183462	36493588	A	0	0	G
5	BICF2G630183477	36502769	G	1	0.6429	A
5	BICF2G630183494	36516031	A	0	0.3571	G
5	BICF2G630183502	36526036	G	1	1	A
5	BICF2P300064	36544568	A	1	1	G
5	BICF2G630183510	36551475	G	0	0	A
5	TIGRP2P70406_rs9057888	36569755	0	0	0	G
5	BICF2S22943297	36580694	G	1	0.6429	A
5	BICF2P684828	36581825	0	0	0	C
5	BICF2S23247854	36627467	A	0	0	C
5	BICF2P954487	36629550	G	1	0.6429	A
5	BICF2G630183540	36641857	A	0	0	G
5	BICF2P662247	36680712	0	0	0	C
5	TIGRP2P70409_rs8755759	36688323	0	0	0	G
5	BICF2G630183551	36738109	A	0	0.3571	G
5	BICF2G630183554	36744398	0	0	0	G
5	BICF2G630183575	36758281	A	0	0.3571	G
5	BICF2G630183581	36766013	A	0	0.3571	G
5	BICF2G630183588	36782760	A	0	0	G
5	BICF2G630183598	36795159	A	0	0	T
5	BICF2G630183600	36801419	A	0	0	G
5	BICF2P1396246	36817650	A	0	0	G
5	BICF2G630183615	36829037	G	0	0	A
5	BICF2G630183623	36839546	A	0	0	G
5	BICF2G630183626	36845402	G	0	0	A
5	BICF2G630183630	36848237	G	0	0	A
5	BICF2P153003	36866416	G	0	0	A
5	BICF2G630183638	36866798	0	0	0	T
5	BICF2P65741	36877443	G	0	0	A
5	BICF2G630183652	36882261	0	0	0	G
5	BICF2G630183659	36901882	0	0	0	G
5	BICF2G630183672	36918297	G	0	0	A

5	BICF2G630183701	36942978	0	0	0	G
5	BICF2G630183721	36970791	0	0	0	G
5	BICF2G630183725	36980723	C	0	0	A
5	BICF2P1228354	36987035	G	0	0	A
5	BICF2G630183733	36995725	0	0	0	G
5	BICF2G630183754	37010344	0	0	0	G
5	BICF2G630183765	37023412	0	0	0	C
5	BICF2S2297096	37039243	A	0	0	G
5	BICF2G630183778	37049364	0	0	0	A
5	BICF2G630183781	37055529	A	0	0	G
5	BICF2G630183793	37069801	G	0	0	A
5	BICF2G630183805	37081986	A	0	0	G
5	BICF2S23749414	37090201	A	0	0	G
5	BICF2P267306	37099612	A	0	0	G
5	BICF2P1337948	37111219	C	0	0	A
5	BICF2S23438844	37128092	A	0	0	G
5	BICF2P68050	37134649	A	0	0	G
5	BICF2P1190032	37152828	G	0	0.3571	A
5	BICF2P215349	37160731	A	0	0	G
5	BICF2G630183806	37172005	0	0	0	A
5	BICF2P470453	37198278	A	0	0.3571	C
5	BICF2S22919273	37221857	A	0	0.3571	G
5	BICF2P252322	37225409	A	0	0.3571	G
5	BICF2S23059225	37233291	A	0	0	G
5	TIGRP2P70421_rs8809030	37247015	0	0	0	G
5	BICF2P963999	37248661	0	0	0	A
5	BICF2P957356	37264375	0	0	0	A
5	BICF2P421158	37289012	0	0	0	G
5	TIGRP2P70423_rs9171653	37298295	0	0	0	G
5	BICF2P875538	37315008	G	0	0	A
5	BICF2P875539	37315513	G	0	0	A
5	BICF2P677057	37318220	G	0	0	A
5	BICF2G630183807	37355744	A	1	0.6429	G
5	BICF2S23122235	37366597	A	0	0	G
5	BICF2G630183812	37382169	G	0	0	A
5	BICF2P443615	37397200	A	0	0	G
5	BICF2G630183831	37406702	G	0	0	A
5	BICF2G630183856	37423251	G	0	0	A
5	BICF2G630183881	37436555	A	0	0	C
5	BICF2G630183899	37445259	A	0	0	G
5	BICF2G630183914	37462987	0	0	0	G
5	BICF2S23612088	37470829	A	0	0	G
5	TIGRP2P70429_rs9233234	37479330	0	0	0	C
5	BICF2G630183931	37490895	A	0	0	G
5	BICF2G630183936	37522717	0	0	0	G
5	BICF2P230614	37535456	0	0	0	G
5	BICF2G630183937	37545942	0	0	0	G

5	BICF2P34344	37552003	0	0	0	C
5	G338f35S290	37578368	A	1	1	C
5	G338f42S239	37584702	G	1	1	A
5	BICF2G630183952	37609394	A	0	0	G
5	BICF2G630183953	37621742	G	0	0	A
5	BICF2S2311835	37631936	A	0	0	C
5	BICF2P860094	37654458	0	0	0	A
5	BICF2P974099	37669878	A	0	0	G
5	BICF2G630183956	37677712	T	0	0	A
5	BICF2G630183959	37689838	G	0	0	A
5	BICF2G630183964	37707206	A	0	0	T
5	BICF2G630183965	37709895	C	0	0	A
5	BICF2G630183984	37736985	C	0	0	A
5	BICF2G630183987	37743815	C	0	0	A
5	BICF2G630184002	37756314	C	0	0	A
5	BICF2S235711	37876722	G	0	0	A
5	BICF2P1186960	37907013	0	0	0	A
5	BICF2G630184009	37921975	G	0	0	A
5	BICF2G630184023	37929790	A	0	0	G
5	BICF2G630184036	37949401	A	0	0	G
5	BICF2G630184039	37953528	A	0	0	G
5	BICF2G630184049	37970568	G	0	0	A
5	BICF2G630184051	37973447	G	0	0	A
5	BICF2G630184059	37994917	G	0	0	A
5	BICF2G630184061	37996061	C	0	0	A
5	BICF2G630184094	38013111	A	0	0	G
5	BICF2G630184103	38027373	A	0	0	G
5	BICF2G630184111	38033352	A	1	1	C
5	BICF2G630184122	38044426	G	1	1	A
5	BICF2G630184139	38060129	G	0	0	A
5	BICF2P580706	38066798	G	0	0	A
5	BICF2G630184159	38068065	G	0	0	A
5	BICF2G630184165	38077457	A	0	0	G
5	BICF2G630184189	38091216	A	1	1	G
5	BICF2G630184196	38097112	A	0	0	G
5	BICF2P941188	38102848	A	0	0	G
5	BICF2G630184234	38118038	A	1	1	C
5	BICF2G630184240	38126167	G	1	1	A
5	TIGRP2P70446_rs8969744	38151503	0	0	0	G
5	BICF2G630184243	38157575	A	0	0	G
5	BICF2P781236	38174646	A	0	0	G
5	BICF2G630184245	38186891	G	0	0	C
5	TIGRP2P70448_rs8751283	38198306	0	0	0	G
5	BICF2P927065	38205183	0	0	0	C
5	BICF2G630184248	38216242	A	0	0	G
5	BICF2G630184254	38240684	G	0	0	A
5	BICF2P1062097	38250309	G	0	0	A

5	BICF2G630184285	38271670	A	0	0	G
5	BICF2P233650	38284991	A	0	0	C
5	BICF2G630184334	38298395	G	0	0	A
5	BICF2G630184335	38298615	G	0	0	A
5	BICF2P648451	38309886	A	0	0	C
5	BICF2G630184367	38319693	A	0	0	G
5	BICF2G630184378	38330071	G	0	0	A
5	BICF2G630184401	38345784	A	0	0	G
5	BICF2G630184432	38355077	G	0	0	A
5	BICF2G630184468	38364482	A	0	0	C
5	BICF2G630184477	38370782	A	0	0	G
5	BICF2S2312076	38384585	G	0	0	A
5	BICF2G630184496	38404483	A	1	1	T
5	BICF2S23421104	38426955	G	0	0	A
5	BICF2S22936116	38434139	G	0	0	A
5	BICF2P629205	38467785	A	0	0	G
5	BICF2S22915624	38488629	C	0	0	A
5	BICF2G630184499	38491106	A	0	0	G
5	BICF2G630184505	38500885	G	0	0	A
5	BICF2G630184506	38502485	A	0	0	G
5	BICF2G630184521	38523708	A	0	0	G
5	BICF2G630184528	38531445	A	1	1	G
5	BICF2G630184543	38542626	A	1	1	G
5	BICF2G630184550	38545408	A	1	1	G
5	BICF2G630184566	38555601	A	1	1	G
5	BICF2S236913	38559980	G	0	0	A
5	BICF2S23150704	38571041	A	0	0	C
5	BICF2G630184589	38574354	A	1	1	G
5	BICF2G630184598	38590200	A	0	0	G
5	BICF2P871388	38600756	G	0	0	A
5	BICF2P579191	38607039	G	0	0	A
5	BICF2G630184611	38617168	0	0	0	G
5	BICF2P1212067	38638060	A	0	0	G
5	BICF2P389767	38643147	G	1	1	A
5	BICF2S22958135	38655877	G	1	1	A
5	BICF2P301964	38666847	G	1	1	A
5	BICF2P934960	38675905	G	1	1	A
5	BICF2P887730	38687309	A	0	0	C
5	BICF2P887727	38688057	A	1	1	G
5	BICF2P1001197	38703372	G	0	0	A
5	BICF2P875587	38713660	A	1	1	G
5	BICF2G630184621	38726120	A	0	0	G
5	BICF2G630184625	38732273	G	0	0	A
5	BICF2G630184635	38743889	A	0	0	C
5	BICF2G630184645	38758791	C	1	1	A
5	BICF2G630184683	38799914	A	0	0	G
5	BICF2G630184692	38808874	G	0	0	C

5	BICF2G630184696	38820269	G	0	0	A
5	BICF2G630184702	38834368	A	0	0	G
5	BICF2P1420622	38839053	A	0	0	G
5	BICF2P626493	38856340	A	0	0	G
5	BICF2P219353	38868614	T	0	0	A
5	BICF2P33190	38871634	G	0	0	A
5	BICF2G630184732	38883786	G	0	0	A
5	BICF2G630184745	38896505	A	0	0	C
5	BICF2P887550	38914737	G	1	1	A
5	BICF2G630184760	38916761	A	1	1	G
5	BICF2P875786	38937989	0	0	0	G
5	BICF2G630184774	38940745	C	0	0	G
5	BICF2P273219	38954623	0	0	0	A
5	BICF2G630184776	38963283	A	0	0	G
5	BICF2G630184778	38980340	A	0	0	C
5	BICF2G630184789	38989681	G	0	0	A
5	BICF2G630184802	39000326	A	0	0	G
5	BICF2P909109	39010970	A	0	0	C
5	BICF2S2459689	39025380	G	1	1	A
5	G339f35S50	39039930	0	0	0	C
5	BICF2P485263	39062288	G	0	0	A
5	BICF2G630184824	39067660	A	0	0	G
5	BICF2P527630	39087221	G	0	0	A
5	BICF2S2303153	39094325	G	0	0	A
5	BICF2P1247185	39102104	G	0	0	A
5	BICF2P1085370	39117210	0	0	0	G
5	BICF2G630184848	39136415	A	0	0	G
5	BICF2P1281226	39166404	0	0	0	C
5	BICF2G630184850	39174454	G	0	0	C
5	BICF2S23641943	39181509	0	0	0	G
5	BICF2G630184853	39234378	G	0	0	A
5	BICF2S23639279	39248207	A	0	0	G
5	BICF2G630184865	39280769	A	1	1	G
5	BICF2G630184870	39284390	A	0	0	G
5	BICF2G630184875	39298279	A	0	0	G
5	BICF2G630184881	39308506	G	0	0	A
5	BICF2P937775	39334106	G	0	0	A
5	BICF2P1373995	39341513	G	0	0	A
5	BICF2P354334	39359652	G	0	0	A
5	BICF2G630184900	39370825	G	0	0	A
5	BICF2G630184903	39384667	A	0	0	G
5	BICF2G630184907	39390411	C	0	0	G
5	BICF2P282293	39400165	G	0	0	A
5	BICF2G630184918	39413982	C	0	0	G
5	BICF2P128827	39422818	C	1	1	A
5	BICF2P1268557	39443480	C	0	0	G
5	BICF2G630184924	39447402	A	0	0	G

5	BICF2G630184934	39460520	G	0	0	A
5	BICF2G630184941	39466825	C	0	0	A
5	BICF2G630184955	39487924	G	0	0	A
5	BICF2G630184958	39491134	C	0	0	A
5	BICF2P1072032	39506217	A	0	0	G
5	BICF2P113131	39523653	A	0	0	G
5	BICF2P172730	39542250	A	0	0	G
5	BICF2S23015977	39553565	A	0	0	G
5	BICF2P1246466	39562270	A	0	0	G
5	BICF2P69171	39579110	A	0	0	G
5	BICF2P69174	39579213	G	0	0	A
5	BICF2P707262	39584458	A	0	0	G
5	BICF2G630184973	39596209	A	0	0	C
5	BICF2G630184974	39596323	A	0	0	C
5	BICF2S23017937	39606164	A	0	0	G
5	BICF2G630184995	39619136	G	0	0	A
5	BICF2G630185003	39629615	A	0	0	G
5	BICF2S23730174	39641254	A	0	0	G
5	BICF2G630185015	39661283	A	0	0	G
5	TIGRP2P70472_rs9080288	39678199	0	0	0	G
5	BICF2G630185017	39703097	A	0	0	G
5	BICF2P172591	39718358	0	0	0	G
5	BICF2G630185022	39721987	0	0	0	G
5	BICF2G630185029	39742084	G	0	0	A
5	BICF2G630185036	39748358	C	0	0	A
5	BICF2G630185055	39758138	C	1	1	G
5	BICF2S24116278	39768477	0	0	0	G
5	BICF2G630185060	39787536	G	0	0	A
5	BICF2P900833	39791764	0	0	0	A
5	BICF2G630185062	39837194	G	0	0	A
5	BICF2G630185069	39847118	A	0	0	G
5	BICF2G630185077	39849004	G	0	0	A
5	BICF2G630185078	39849125	A	0	0	G
5	BICF2P24480	39860925	0	0	0	A
5	BICF2P933506	39883763	A	0	0	G
5	BICF2P257525	39894275	A	1	1	G
5	BICF2P174945	39909316	G	1	1	A
5	BICF2G630185097	39922961	0	0	0	G
5	BICF2G630185121	39942730	G	0	0	A
5	BICF2G630185137	39953733	G	0	0	A
5	BICF2S23648024	39965990	G	0	0	A
5	BICF2G630185148	39976342	A	0	0	C
5	BICF2G630185163	39993746	G	1	1	A
5	BICF2G630185166	40000693	A	0	0	G
5	BICF2P75883	40041289	0	0	0	G
5	BICF2S23127907	40045102	A	0	0	G
5	BICF2S23237409	40066370	G	1	1	A

5	BICF2S23341074	40079424	A	1	1	G
5	BICF2P740376	40095131	0	0	0	G
5	BICF2S23347193	40116418	A	0	0	G
5	BICF2G630185169	40123507	0	0	0	T
5	BICF2P440245	40146100	A	1	1	G
5	BICF2P862897	40148742	A	0	0	G
5	BICF2P1402905	40164399	G	1	1	A
5	BICF2P864238	40178240	A	1	1	G
5	BICF2S23057646	40191847	G	1	1	A
5	BICF2P743426	40203559	G	0	0	A
5	BICF2S23440678	40215420	A	0	0	G
5	BICF2P964815	40218819	A	0	0	G
5	BICF2P173306	40227031	A	0	0	C
5	BICF2S23144314	40246039	A	0	0	G
5	BICF2P219390	40250934	G	0	0	A
5	BICF2S23763062	40268848	G	0	0	A
5	BICF2P118278	40269836	A	0	0	G
5	BICF2S24110017	40273932	A	0	0	G
5	BICF2S23634319	40292947	A	0	0	G
5	BICF2S23634320	40293087	A	0	0	G
5	BICF2S23356294	40297123	G	0	0	A
5	BICF2S22923370	40316845	G	0	0	A
5	BICF2S2321955	40325104	A	0	0	G
5	BICF2P976309	40339283	G	0	0	A
5	BICF2G630185190	40352191	A	0	0	G
5	BICF2G630185199	40360278	G	0	0	A
5	BICF2G630185210	40365446	G	0	0	A
5	BICF2G630185224	40386823	A	0	0	G
5	BICF2G630185225	40389950	A	0	0	G
5	BICF2G630185238	40406273	A	0	0	G
5	BICF2G630185241	40411376	0	0	0	C
5	BICF2P948047	40411905	0	0	0	G
5	BICF2P1232840	40431749	A	0	0.3571	G
5	BICF2G630185255	40453665	0	0	0	C
5	BICF2G630185266	40463862	G	0	0	A
5	BICF2G630185277	40474772	A	0	0	G
5	BICF2P548682	40480498	A	0	0	C
5	BICF2G630185294	40492493	A	0	0	G
5	BICF2G630185298	40505686	C	1	0.6429	G
5	BICF2P953223	40525772	A	0	0.3571	C
5	BICF2G630185334	40530268	A	0	0	G
5	BICF2G630185374	40547873	C	0	0	A
5	BICF2G630185381	40552195	A	0	0	G
5	BICF2G630185389	40561273	G	0	0.3571	A
5	BICF2G630185416	40573153	G	1	0.7	A
5	BICF2G630185442	40584164	G	0	0.3571	A
5	BICF2G630185475	40606486	A	0	0	G

5	BICF2G630185490	40616981	C	0	0	A
5	BICF2G630185491	40618237	G	0	0	A
5	BICF2P709783	40640574	T	0	0.3571	A
5	BICF2S23448260	40649273	G	0	0.3571	A
5	BICF2S23448423	40652509	A	0	0.3571	C
5	BICF2P83298	40668224	G	0	0.3571	A
5	BICF2P370830	40678588	0	0	0	G
5	BICF2P151703	40692078	G	1	0.6429	A
5	BICF2G630185562	40708214	G	0	0	A
5	BICF2G630185614	40716559	A	1	0.6429	G
5	BICF2G630185617	40727608	A	0	0	G
5	BICF2G630185621	40733573	A	1	0.6429	G
5	BICF2G630185630	40746782	A	0	0	G
5	BICF2G630185640	40758915	A	0	0	G
5	BICF2P513069	40776571	A	0	0	G
5	BICF2G630185657	40778736	A	0	0	G
5	BICF2G630185666	40781089	A	0	0	G
5	BICF2S236331	40803331	0	0	0	G
5	BICF2P512787	40821224	A	0	0.3571	C
5	BICF2G630185725	40827188	G	0	0.3571	A
5	BICF2G630185754	40853871	G	0	0	C
5	BICF2G630185758	40859918	G	0	0	A
5	TIGRP2P70482_rs9124925	40877377	0	0	0	G
5	BICF2G630185771	40886029	G	0	0	A
5	BICF2G630185774	40894606	C	0	0	A
5	BICF2G630185787	40905773	G	1	0.6429	A
5	BICF2G630185802	40919843	C	1	0.6429	A
5	BICF2S2308493	40935023	C	0	0	A
5	BICF2G630185816	40950354	A	0	0	G
5	BICF2G630185826	40952673	A	0	0	G
5	BICF2P682357	40965077	G	0	0	A
5	BICF2G630185860	40977364	C	0	0	A
5	BICF2G630185885	40994094	G	0	0	A
5	BICF2G630185897	40998333	A	0	0	G
5	BICF2G630185919	41011517	G	0	0	C
5	BICF2G630185948	41028708	A	0	0	C
5	BICF2G630185956	41038084	A	0	0.3333	C
5	BICF2G630185964	41045480	A	1	1	G
5	BICF2G630185974	41055278	A	1	0.6429	G
5	BICF2G630186008	41074007	A	1	1	G
5	BICF2S23444652	41093413	A	0	0	G
5	BICF2G630186024	41107067	G	0	0	A
5	BICF2G630186032	41116843	G	1	1	A
5	BICF2G630186046	41128879	A	1	0.6429	G
5	BICF2S23120140	41146472	A	0	0	G
5	BICF2S23549913	41159832	G	0	0	A
5	BICF2G630186048	41179801	G	0	0.3571	A

5	BICF2S23112756	41191729	A	0	0	G
5	BICF2S23715617	41200003	C	0	0	A
5	BICF2S22920330	41237038	A	0	0	C
5	BICF2S22965002	41242928	A	0	0	G
5	BICF2S23711084	41257313	G	0	0	A
5	BICF2S22963621	41262494	C	0	0	A
5	BICF2P206170	41278284	A	1	0.6429	G
5	BICF2P314107	41289767	0	0	0	G
5	BICF2S244621	41300215	C	0	0	A
5	BICF2P193342	41317118	0	0	0	G
5	BICF2P320722	41320363	0	0	0	A
5	BICF2P806751	41345473	0	0	0	A
5	BICF2P509731	41358055	0	0	0	A
5	BICF2G630186061	41369703	G	0	0	A
5	BICF2G630186064	41385129	A	1	1	G
5	BICF2G630186079	41404963	A	1	0.6429	C
5	BICF2G630186094	41411815	A	1	0.6429	G
5	BICF2G630186106	41425649	G	0	0	A
5	BICF2S23714222	41444144	A	0	0	G
5	BICF2P237050	41462639	A	1	1	G
5	BICF2G630186171	41472065	A	0	0	G
5	BICF2G630186180	41480845	G	0	0	A
5	BICF2G630186182	41481189	A	0	0	G
5	BICF2G630186215	41496464	G	0	0	A
5	BICF2G630186224	41505178	A	0	0	G
5	BICF2G630186239	41524158	A	0	0	C
5	BICF2G630186251	41528401	A	0	0	G
5	BICF2G630186279	41551076	T	0	0	A
5	BICF2P439713	41564157	G	0	0	A
5	BICF2G630186301	41575639	C	0	0	A
5	BICF2P1125615	41586812	A	0	0	G
5	BICF2G630186303	41596934	A	0	0	G
5	BICF2P681858	41610826	G	0	0	C
5	BICF2S23340102	41625388	G	0	0	A
5	TIGRP2P70542_rs9158013	41641465	G	0	0	A
5	BICF2S2441921	41643035	C	0	0	A
5	BICF2P1408286	41663705	G	0	0	A
5	TIGRP2P70555_rs8536328	41665006	C	0	0	A
5	TIGRP2P70559_rs8710169	41678942	G	0	0	A
5	BICF2G630186310	41690223	0	0	0	A
5	BICF2G630186325	41699670	A	0	0	G
5	BICF2P309143	41713520	G	0	0.3571	A
5	BICF2G630186332	41717160	A	0	0	G
5	BICF2G630186351	41730505	A	1	1	G
5	BICF2G630186354	41737933	G	0	0	A
5	BICF2P645920	41749654	A	0	0	G
5	TIGRP2P70595_rs9042416	41766902	A	0	0	G

5	BICF2G630186387	41769809	G	0	0	A
5	BICF2G630186400	41788357	A	0	0	G
5	BICF2P266158	41791042	A	0	0	G
5	BICF2P266162	41791761	G	0	0	A
5	TIGRP2P70630_rs8733196	41806120	A	0	0	G
5	BICF2P182887	41822825	A	0	0	G
5	TIGRP2P70640_rs8831120	41845687	C	1	0.6429	A
5	BICF2P223980	41859458	C	1	0.6429	G
5	BICF2P223979	41859667	A	1	0.6429	G
5	BICF2P211633	41863092	A	1	0.6429	C
5	BICF2P216771	41877056	A	0	0.3571	C
5	BICF2P647721	41885844	G	0	0.3571	A
5	BICF2P1414260	41896366	A	0	0	G
5	BICF2P1079376	41908306	G	0	0	A
5	BICF2P345420	41919294	G	1	0.6667	A
5	BICF2P726422	41934494	A	1	0.6429	G
5	BICF2P621331	41945851	A	0	0	G
5	BICF2P269131	41956356	G	0	0	A
5	TIGRP2P70690_rs8985094	41965452	A	1	1	G
5	BICF2P225206	41978918	G	1	1	A
5	BICF2P363932	41997987	A	0	0	G
5	BICF2G630186413	42000988	G	0	0	A
5	BICF2P555882	42018918	G	0	0	A
5	BICF2P329738	42024607	A	0	0	G
5	TIGRP2P70716_rs8856320	42035734	A	0	0	G
5	TIGRP2P70719_rs9094935	42046057	G	0	0	A
5	TIGRP2P70721_rs9147066	42059750	A	0	0	G
5	BICF2P394905	42079112	G	0	0	A
5	TIGRP2P70736_rs9186315	42094433	A	0	0	G
5	BICF2P271975	42107650	0	0	0	T
5	BICF2P940891	42119094	G	0	0.3571	A
5	BICF2P1023313	42132476	G	0	0.3571	A
5	TIGRP2P70746_rs9194124	42139804	A	0	0.3571	G
5	BICF2S24512133	42155332	G	0	0.3571	A
5	BICF2P441608	42166617	A	0	0	G
5	TIGRP2P70748_rs9204968	42191132	G	0	0	A
5	TIGRP2P70751_rs8741611	42199692	G	0	0.3571	A
5	TIGRP2P70756_rs8657816	42209697	A	0	0	G
5	BICF2P1470873	42224401	G	0	0	A
5	BICF2P434000	42230987	C	0	0.3571	G
5	BICF2P523055	42246436	A	0	0	T
5	BICF2P229103	42273904	0	0	0	A
5	BICF2G630186423	42312538	A	0	0	G
5	BICF2P256438	42352599	0	0	0	A
5	BICF2P1054108	42361791	A	0	0	G
5	BICF2P430858	42391295	0	0	0	G
5	BICF2P1195929	42410907	0	0	0	G

5	BICF2S23129951	42419630	A	0	0.3571	G
5	BICF2S23761118	42426988	A	0	0.3333	G
5	TIGRP2P70766_rs8607439	42447798	C	0	0.3571	G
5	BICF2P777938	42453935	A	0	0.3571	C
5	BICF2P422007	42455159	A	0	0	G
5	BICF2S23237413	42466640	A	0	0.3571	C
5	BICF2S229680	42472747	A	0	0	G
5	BICF2P1078254	42483441	A	0	0	G
5	TIGRP2P70775_rs9120494	42498418	0	0	0	C
5	TIGRP2P70776_rs9130739	42506503	A	0	0	G
5	BICF2G630186424	42571631	A	0	0	T
5	BICF2P37723	42582241	G	0	0	A
5	BICF2S22950038	42614306	G	0	0	A
5	BICF2S23714382	42621129	A	0	0	G
5	BICF2S23022881	42634661	C	0	0	G
5	BICF2P253667	42643469	0	0	0	G
5	BICF2S23732327	42669448	A	0	0	G
5	BICF2P1293791	42687053	0	0	0	C
5	BICF2P257464	42718007	0	0	0	A
5	BICF2S22928611	42735086	A	0	0	G
5	BICF2P343582	42772325	0	0	0	A
5	BICF2P635605	42811058	0	0	0	G
5	BICF2P701721	42824856	0	0	0	C
5	TIGRP2P70795_rs8826446	42858157	0	0	0	A
5	BICF2P768879	42869858	0	0	0	A
5	TIGRP2P70797_rs8708908	42875792	0	0	0	A
5	BICF2S23161529	42892497	A	0	0	G
5	BICF2P429559	42903803	0	0	0	A
5	BICF2P1176440	42907773	C	1	1	A
5	BICF2P683055	42922739	A	NA	NA	G
5	BICF2P1238614	42937434	G	0	0	A
5	TIGRP2P70805_rs8717739	42946941	A	0	0	C
5	BICF2P1119048	42974065	A	0	0	T
5	BICF2P1341365	42976185	A	0	0	G
5	BICF2P1356047	42993653	A	0	0	G
5	BICF2P753299	43020550	A	0	0	G
5	BICF2S23741924	43029020	G	1	1	A
5	BICF2S22952409	43038096	G	1	1	A
5	BICF2P596088	43051311	A	1	0.6429	C
5	BICF2P1120589	43080676	A	0	0	G
5	BICF2P727196	43103765	G	0	0	C
5	TIGRP2P70849_rs8612351	43119082	A	0	0.3571	G
5	BICF2P1427483	43128054	A	0	0	G
5	BICF2P310590	43142761	A	0	0	G
5	BICF2P937003	43150186	0	0	0	G
5	BICF2P338952	43164475	A	0	0	G
5	BICF2G630186426	43176690	0	0	0	A

5	BICF2S23419705	43202747	A	1	0.6429	G
5	BICF2P1356360	43227275	G	0	0	A
5	BICF2P758787	43231868	C	0	0	G
5	TIGRP2P70866_rs8870723	43245970	G	0	0.3571	A
5	BICF2P1000375	43261526	A	0	0.3571	C
5	BICF2P292425	43267073	A	0	0.3571	G
5	TIGRP2P70876_rs9139741	43276128	A	0	0.3571	G
5	TIGRP2P70882_rs8673006	43294294	A	0	0	G
5	BICF2P289423	43297626	G	0	0	A
5	BICF2S23139937	43320461	G	0	0.3571	A
5	BICF2P96875	43333733	G	0	0.3571	A
5	TIGRP2P70903_rs8740143	43347687	G	0	0.3571	A
5	BICF2P1334728	43358922	A	0	0	G
5	TIGRP2P70907_rs8959367	43377265	0	0	0	C
5	G342f53S228	43388939	0	0	0	G
5	TIGRP2P70908_rs8673568	43391456	C	0	0	A
5	TIGRP2P70910_rs9104870	43409523	G	0	0	A
5	BICF2P1039167	43417843	G	0	0	A
5	BICF2P1039165	43419543	G	0	0.3571	A
5	BICF2S24116593	43426969	0	0	0	G
5	BICF2S23162446	43441719	A	0	0.3571	G
5	BICF2P1391141	43452070	A	0	0.3571	C
5	BICF2P194103	43461715	G	0	0.3571	A
5	BICF2P290705	43475080	G	0	0.3571	A
5	TIGRP2P70925_rs8835810	43486480	A	0	0.3571	G
5	BICF2P914791	43486543	A	0	0	G
5	TIGRP2P70926_rs8619729	43489683	A	0	0.3571	G
5	BICF2P1283967	43490133	G	0	0.3571	A
5	TIGRP2P70931_rs8620298	43499913	A	0	0.3333	G
5	BICF2P154619	43509876	G	0	0.3571	C
5	BICF2S2316820	43521313	C	0	0.3333	A
5	BICF2S22946142	43538690	0	0	0	C
5	BICF2S23712651	43574834	A	0	0	G
5	BICF2S23127046	43596112	0	0	0	G
5	BICF2P591505	43612855	A	0	0.3571	G
5	BICF2P1025058	43624678	A	0	0.3571	G
5	BICF2S23719316	43633059	G	0	0.3571	A
5	BICF2S2446554	43647582	0	0	0	G
5	BICF2P78778	43668332	0	0	0	G
5	BICF2S2318341	43677675	G	0	0.3571	A
5	BICF2S23516038	43688883	T	0	0.3571	A
5	BICF2P906984	43704781	0	0	0	G
5	G343f37S21	43731084	G	0	0	A
5	BICF2S23135376	43753642	G	0	0.2857	A
5	BICF2S2362355	43791024	G	0	0.2857	A
5	TIGRP2P70983_rs8602715	43797131	0	0	0	C
5	TIGRP2P70984_rs8618055	43808558	0	0	0	A

5	BICF2P716067	43843342	G	0	0.2857	A
5	BICF2P352386	43864668	0	0	0	C
5	TIGRP2P70993_rs8824848	43887324	A	0	0	G
5	BICF2S2314059	43899193	A	0	0	G
5	BICF2P993275	43914843	A	0	0	C
5	BICF2P760674	43928291	G	0	0.2857	A
5	BICF2P1281968	43933979	G	1	1	A
5	BICF2P871850	43950557	C	0	0	A
5	BICF2G630186433	43956581	0	0	0	C
5	BICF2S23529389	43972174	A	0	0	G
5	BICF2P712925	43981803	A	0	0.2857	G
5	BICF2P592511	44000344	0	0	0	G
5	BICF2P1413613	44020376	G	0	0	A
5	BICF2P1108505	44030449	G	0	0	A
5	BICF2P703663	44047600	A	1	0.7143	G
5	TIGRP2P71030_rs9013592	44057317	C	0	0	A
5	BICF2P58200	44078797	G	0	0	A
5	TIGRP2P71054_rs8629930	44085571	A	0	0	G
5	TIGRP2P71063_rs8965943	44108553	0	0	0	A
5	BICF2P997736	44117925	A	0	0	G
5	BICF2P1070583	44131651	C	1	1	G
5	BICF2P41192	44146070	A	0	0	G
5	BICF2P170755	44149035	G	0	0	A
5	BICF2P1425957	44169481	C	1	0.7143	A
5	TIGRP2P71072_rs9073129	44179705	A	0	0	C
5	BICF2P211464	44183777	C	0	0	A
5	TIGRP2P71085_rs8828691	44205757	A	0	0	T
5	TIGRP2P71086_rs9053099	44223626	A	0	0	G
5	BICF2P862464	44231887	A	0	0.2857	G
5	BICF2P831393	44241926	A	0	0.2857	G
5	BICF2P633912	44257555	A	0	0	G
5	BICF2P1055821	44270437	G	1	0.7143	A
5	BICF2P1055820	44270665	A	1	0.7143	G
5	BICF2P1300835	44276420	G	1	0.7143	A
5	BICF2P543784	44288432	C	1	0.7143	A
5	BICF2P764356	44309705	A	1	0.7143	G
5	BICF2P374997	44321099	A	0	0	G
5	BICF2P1177298	44341037	A	1	0.7143	G
5	BICF2P161242	44354530	0	0	0	A
5	BICF2P161245	44355096	0	0	0	G
5	BICF2P1098323	44360593	G	1	0.7143	A
5	TIGRP2P71121_rs8749800	44369645	C	0	0	A
5	BICF2P1040388	44386425	0	0	0	G
5	TIGRP2P71134_rs8995432	44393739	C	0	0	A
5	BICF2P1246411	44406005	A	0	0	G
5	BICF2P1426462	44416208	A	0	0	G
5	BICF2P1091500	44435870	A	0	0.2857	G

5	TIGRP2P71148_rs9045255	44447167	G	1	0.7143	A
5	BICF2P1065249	44458340	A	0	0.2857	G
5	BICF2P1127037	44461758	A	0	0	G
5	BICF2P140730	44477113	A	1	0.7143	G
5	BICF2P694183	44502381	G	1	1	A
5	BICF2S2304979	44515583	A	0	0.2857	G
5	BICF2P1122683	44518399	G	1	1	A
5	TIGRP2P71160_rs8830132	44531121	A	1	1	G
5	BICF2P1469672	44542628	G	1	0.7143	A
5	BICF2S24114334	44557832	A	1	0.7143	G
5	BICF2P667702	44563520	A	1	0.7143	G
5	BICF2P1224829	44579495	G	1	0.7143	A
5	BICF2S2445944	44585680	G	1	0.7143	A
5	BICF2P252588	44586151	A	1	0.7143	G
5	BICF2P773172	44606853	A	1	0.7143	G
5	BICF2P1173109	44610945	A	1	0.7143	G
5	TIGRP2P71190_rs8688242	44618000	A	1	0.7143	G
5	TIGRP2P71191_rs8963019	44624964	A	1	0.7143	G
5	BICF2P464664	44634112	A	1	0.7143	C
5	BICF2P86247	44644679	0	0	0	G
5	BICF2P764192	44661096	0	0	0	G
5	BICF2P397305	44675224	C	0	0	G
5	BICF2P790965	44681461	A	1	0.7143	G
5	BICF2P564534	44689109	A	1	0.7143	G
5	TIGRP2P71206_rs8740239	44701598	0	0	0	C
5	BICF2P1168121	44733392	A	0	0	G
5	BICF2P473627	44741643	0	0	0	C
5	TIGRP2P71207_rs8548472	44757342	0	0	0	G
5	BICF2P507511	44764263	0	0	0	G
5	BICF2P703930	44788911	A	0	0	C
5	BICF2P426604	44793994	0	0	0	A
5	TIGRP2P71213_rs9156976	44810504	A	0	0	G
5	TIGRP2P71216_rs8563499	44818855	A	0	0	G
5	BICF2P74407	44829938	G	0	0	A
5	BICF2P254361	44847121	0	0	0	G
5	BICF2P374635	44870564	0	0	0	C
5	BICF2P167552	44880153	A	1	0.7143	C
5	BICF2P881350	44894115	A	1	0.7143	G
5	BICF2P341199	44897493	G	1	0.7143	A
5	BICF2P330613	44917551	A	1	0.7143	G
5	BICF2P733704	44939843	A	1	0.7143	G
5	BICF2P1022878	44944533	G	1	0.7143	A
5	BICF2P209879	44958674	A	0	0.2857	G
5	BICF2P1256881	44965608	A	0	0.2857	G
5	BICF2S23646053	44978999	T	1	0.7143	A
5	BICF2P1076079	44982158	A	1	0.7143	C
5	BICF2P264710	44990396	G	1	0.7143	A

5	BICF2S23130606	45007528	A	0	0	G
5	BICF2P769053	45008619	G	1	0.7143	A
5	TIGRP2P71274_rs8563921	45021363	0	0	0	A
5	BICF2P1368808	45022579	A	0	0	G
5	BICF2S2328056	45035865	G	0	0	C
5	BICF2P1135793	45039575	G	0	0	A
5	BICF2P1253115	45047863	A	0	0	C
5	BICF2P1293078	45068348	G	0	0	A
5	BICF2P1293073	45069181	C	0	0	G
5	BICF2P243681	45086157	0	0	0	C
5	TIGRP2P71276_rs8965891	45100786	0	0	0	G
5	BICF2P168367	45118982	C	0	0.2857	A
5	BICF2P599846	45138131	G	0	0.2857	A
5	BICF2P1166160	45151323	A	0	0.2857	G
5	BICF2P1055722	45162144	G	0	0.2857	A
5	BICF2P1099142	45181080	G	0	0.2857	A
5	BICF2P622213	45184489	C	0	0.2857	A
5	BICF2P937318	45196265	G	0	0.125	A
5	BICF2P163563	45217395	G	0	0.2857	A
5	BICF2S233727	45230494	C	0	0.2857	A
5	BICF2P1330462	45242984	0	0	0	A
5	TIGRP2P71279_rs8590046	45243339	0	0	0	G
5	BICF2P967078	45257690	A	0	0	G
5	BICF2S2321350	45272650	A	0	0	G
5	BICF2P1412083	45282726	A	0	0	G
5	BICF2P789003	45290858	A	0	0	G
5	BICF2P998380	45308409	G	0	0.2857	A
5	BICF2S23018364	45312776	A	0	0.2857	G
5	BICF2P917537	45334656	A	0	0	G
5	BICF2P1202106	45370446	A	0	0.2857	C
5	BICF2S2323662	45382916	A	1	0.7143	G
5	BICF2P882771	45395079	A	0	0	G
5	G344f43S206	45434757	A	0	0	G
5	G344f47S123	45439830	A	0	0	G
5	BICF2P1188677	45472932	0	0	0	G
5	BICF2S23443985	45499341	A	0	0	C
5	BICF2P544205	45511883	0	0	0	A
5	BICF2P600330	45534451	G	0	0	A
5	BICF2P527008	45550138	0	0	0	G
5	G345f13S257	45576890	G	0	0.2857	A
5	BICF2P825145	45603573	0	0	0	C
5	BICF2P710637	45636986	0	0	0	G
5	BICF2S2307645	45661442	A	0	0.2857	G
5	G346f51S28	45698126	C	0	0.2857	G
5	BICF2P455389	45723828	0	0	0	C
5	BICF2P7464	45729524	0	0	0	G
5	TIGRP2P71289_rs8899736	45735996	0	0	0	A

5	BICF2P1201551	45757632	G	0	0	A
5	BICF2P116993	45763276	0	0	0	G
5	BICF2P298304	45778362	0	0	0	A
5	TIGRP2P71292_rs8962905	45792882	0	0	0	A
5	BICF2P707836	45795172	A	0	0	G
5	BICF2P518930	45821184	0	0	0	G
5	BICF2S22928795	45839465	G	0	0.2857	A
5	BICF2P476564	45855194	A	1	0.7143	G
5	BICF2P428095	45863257	0	0	0	A
5	BICF2S23331634	45922522	A	1	0.7143	G
5	BICF2S2413183	45935460	G	0	0	A
5	BICF2P309812	45953835	C	1	1	A
5	BICF2S23133529	45980016	G	1	1	A
5	BICF2G630186638	45989240	G	1	1	A
5	BICF2G630186647	46004305	G	1	1	A
5	BICF2G630186657	46015372	G	1	1	A
5	BICF2G630186658	46025287	A	0	0	G
5	BICF2G630186668	46041552	G	1	1	A
5	BICF2G630186696	46062303	A	1	1	G
5	BICF2G630186718	46072535	A	1	1	G
5	BICF2G630186735	46079166	A	1	1	C
5	BICF2G630186743	46083925	G	1	1	A
5	BICF2G630186763	46106356	A	0	0	G
5	BICF2P129904	46128551	0	0	0	A
5	BICF2G630186777	46158109	A	0	0	C
5	BICF2G630186779	46168185	C	0	0	A
5	BICF2P1375880	46181807	A	0	0	C
5	BICF2G630186791	46217490	G	0	0	A
5	BICF2G630186795	46222474	G	0	0	A
5	BICF2G630186833	46245603	A	0	0	C
5	BICF2P274180	46249714	A	0	0	G
5	BICF2P1392393	46250407	A	0	0	G
5	BICF2G630186851	46258320	A	0	0	G
5	BICF2S23524531	46264506	C	0	0	A
5	TIGRP2P71427_rs8866516	46309692	0	0	0	T
5	BICF2G630186869	46315900	0	0	0	G
5	BICF2S2367641	46340032	A	0	0	G
5	G347f36S116	46363445	0	0	0	G
5	BICF2P181027	46383361	G	0	0	A
5	BICF2G630186879	46408387	A	0	0	G
5	BICF2G630186892	46418781	G	0	0	A
5	BICF2G630186927	46435777	A	0	0	G
5	BICF2G630186933	46438890	A	0	0	G
5	BICF2G630186953	46451107	A	0	0	G
5	BICF2G630186964	46463491	G	0	0	A
5	BICF2G630186985	46471727	G	0	0	A
5	BICF2P217445	46489577	0	0	0	A

5	BICF2G630186995	46498069	G	1	1	A
5	BICF2G630187000	46511658	A	0	0	G
5	BICF2S23423959	46524163	T	0	0	A
5	BICF2G630187013	46538636	C	0	0	A
5	BICF2P422578	46548474	0	0	0	G
5	BICF2G630187016	46560470	A	1	1	G
5	BICF2G630187018	46568994	A	0	0	G
5	BICF2G630187020	46583644	A	0	0.2	G
5	BICF2G630187022	46587057	G	0	0.2857	A
5	TIGRP2P71448_rs8939649	46622155	A	0	0.2857	G
5	BICF2P1394519	46643192	A	1	1	G
5	BICF2P283701	46654276	G	0	0.2857	A
5	BICF2P1259970	46674946	A	0	0.2857	G
5	BICF2S2392653	46685227	G	1	0.7143	A
5	BICF2P763805	46692681	A	1	0.8	G
5	BICF2G630187050	46702497	A	1	0.7143	G
5	BICF2G630187066	46716461	A	0	0	G
5	BICF2P56117	46721860	A	0	0.2857	G
5	BICF2G630187070	46729345	A	0	0	C
5	BICF2G630187075	46736938	A	1	1	G
5	BICF2G630187106	46774514	A	0	0.2857	G
5	BICF2G630187117	46785340	A	0	0	G
5	BICF2G630187121	46797455	A	0	0	G
5	BICF2G630187129	46808035	G	0	0.2857	A
5	BICF2G630187144	46817654	C	0	0.2857	G
5	BICF2P97580	46830016	0	0	0	C
5	BICF2G630187147	46844229	0	0	0	G
5	BICF2P1513176	46866466	G	1	1	A
5	BICF2G630187158	46884482	A	0	0	C
5	BICF2G630187160	46886644	G	0	0	A
5	BICF2G630187171	46899140	A	0	0	G
5	BICF2S23111031	46913495	C	0	0	G
5	TIGRP2P71490_rs8492309	46922985	A	0	0	G
5	BICF2G630187198	46937085	T	0	0	A
5	BICF2G630187206	46944163	A	1	1	G
5	BICF2S23511087	46956371	A	0	0	C
5	BICF2G630187227	46966504	A	0	0	C
5	BICF2G630187264	46986065	G	0	0	A
5	BICF2G630187267	46989781	G	0	0	A
5	BICF2G630187268	46990030	A	0	0	G
5	BICF2G630187337	47010908	G	1	1	A
5	BICF2G630187355	47016766	G	0	0	A
5	BICF2G630187387	47036440	G	1	1	C
5	BICF2G630187403	47047616	A	0	0	G
5	BICF2G630187408	47063355	G	0	0	A
5	BICF2P1084320	47070258	A	0	0	C
5	BICF2P1505313	47090810	A	0	0	G

5	TIGRP2P71511_rs8502819	47095461	C	1	1	A
5	BICF2P416741	47107111	A	0	0	G
5	BICF2P272358	47116899	G	1	1	A
5	BICF2P1089498	47128310	G	0	0	A
5	BICF2P232416	47148086	G	0	0	A
5	BICF2G630187455	47150997	G	0	0	A
5	BICF2G630187468	47163453	A	0	0	G
5	BICF2G630187469	47163546	A	0	0	G
5	BICF2G630187475	47173022	G	0	0	A
5	BICF2G630187491	47192258	A	1	0.7143	G
5	BICF2G630187498	47195264	A	1	1	T
5	BICF2G630187503	47214400	G	1	0.7143	A
5	BICF2P1170772	47229455	A	0	0	G
5	BICF2P419745	47232749	T	0	0	A
5	BICF2P209903	47245908	A	0	0	G
5	TIGRP2P71547_rs8844997	47256559	A	0	0	G
5	TIGRP2P71548_rs8644695	47265430	A	0	0	G
5	BICF2P365609	47277620	G	0	0	A
5	BICF2S23234898	47294203	G	0	0	A
5	BICF2P167514	47300476	A	0	0	G
5	BICF2P250380	47317049	G	0	0	A
5	BICF2G630187516	47324118	G	0	0.2857	A
5	TIGRP2P71560_rs8937362	47335318	A	0	0	G
5	BICF2P360563	47350942	A	0	0.2857	G
5	BICF2P1229538	47358271	G	0	0	A
5	TIGRP2P71570_rs9248163	47372989	A	0	0	G
5	BICF2G630187527	47375859	A	0	0	T
5	BICF2G630187536	47386531	A	0	0	G
5	BICF2G630187548	47391598	A	0	0.2857	G
5	BICF2P277933	47408778	A	0	0	C
5	TIGRP2P71577_rs8794018	47414734	A	0	0	C
5	BICF2G630187586	47430380	G	0	0.2857	A
5	BICF2G630187596	47440047	G	0	0.2857	A
5	BICF2P253425	47456234	G	0	0	A
5	BICF2S23647701	47460682	A	0	0	G
5	BICF2S23639621	47476953	A	0	0	G
5	BICF2P446737	47493970	A	0	0	G
5	TIGRP2P71608_rs8802111	47501573	A	0	0	G
5	BICF2P294747	47514597	A	0	0.2857	G
5	TIGRP2P71617_rs9250386	47525882	G	0	0	A
5	BICF2P1122600	47532136	G	0	0	A
5	BICF2P291809	47542477	G	0	0	A
5	BICF2P115332	47558944	G	1	0.7143	A
5	BICF2P661404	47566104	G	0	0.2857	A
5	BICF2P980625	47577747	A	1	0.7143	G
5	BICF2P525607	47587736	0	0	0	G
5	BICF2G630187615	47606221	G	1	0.7143	A

5	BICF2P625366	47617114	G	1	0.7143	A
5	BICF2P1420185	47630122	G	1	0.7143	A
5	BICF2G630187649	47644089	T	1	1	A
5	BICF2G630187658	47652578	A	1	1	G
5	BICF2P981519	47660892	G	0	0.2857	A
5	BICF2G630187682	47668708	C	0	0.2857	A
5	BICF2G630187696	47689361	C	0	0.2857	A
5	BICF2G630187698	47690835	A	0	0	G
5	BICF2G630187705	47707624	A	0	0	G
5	BICF2G630187712	47714308	G	0	0	A
5	BICF2P384664	47727062	A	0	0	G
5	BICF2G630187716	47747458	T	0	0	A
5	BICF2P777829	47752866	A	1	0.7143	C
5	BICF2G630187722	47760526	G	0	0	A
5	BICF2G630187725	47773220	G	0	0	A
5	BICF2P860194	47784696	A	1	0.7143	G
5	TIGRP2P71670_rs8870230	47794633	A	0	0	G
5	BICF2G630187728	47816656	T	0	0	A
5	BICF2S2327772	47828053	A	0	0.2857	G
5	BICF2G630187732	47835346	0	0	0	G
5	BICF2P1335186	47848554	0	0	0	G
5	BICF2G630187745	47862493	C	0	0.2857	A
5	BICF2G630187751	47863106	G	0	0.2857	A
5	BICF2G630187781	47876311	A	0	0.2857	G
5	BICF2G630187783	47886685	A	0	0	C
5	BICF2P583791	47906309	0	0	0	A
5	BICF2S23041992	47931399	G	0	0	C
5	BICF2G630187785	47940263	A	0	0	G
5	BICF2S24415085	47961072	0	0	0	G
5	TIGRP2P71676_rs8990315	47975174	0	0	0	A
5	BICF2S23024764	47980801	A	0	0	G
5	BICF2G630187793	47996518	A	0	0	G
5	TIGRP2P71680_rs9038471	48011761	C	0	0	A
5	BICF2G630187798	48018522	A	0	0	G
5	BICF2G630187804	48041671	G	0	0	A
5	BICF2G630187811	48056873	A	0	0.2857	G
5	BICF2G630187815	48090358	0	0	0	G
5	BICF2P408006	48114518	A	0	0	G
5	BICF2S23147082	48120450	G	0	0	A
5	BICF2P1026929	48130205	A	1	1	G
5	BICF2G630187837	48141422	G	1	1	A
5	BICF2P964054	48151351	C	0	0.2857	A
5	BICF2G630187854	48172024	A	1	1	G
5	BICF2S23757204	48176254	G	0	0	A
5	BICF2G630187856	48200745	A	1	0.7143	G
5	BICF2P212694	48209905	G	0	0.2857	A
5	BICF2P680631	48225037	C	0	0.2857	A

5	BICF2P819073	48234900	A	0	0.2857	G
5	BICF2P334730	48250241	G	0	0.2857	A
5	BICF2P504716	48254824	A	0	0.25	G
5	BICF2P1274127	48271371	G	0	0.2857	A
5	TIGRP2P71719_rs9104422	48277332	A	0	0.2857	G
5	TIGRP2P71729_rs9248555	48290301	0	0	0	G
5	BICF2P1114921	48301502	G	0	0.2857	A
5	BICF2P305241	48322141	0	0	0	G
5	BICF2P953030	48326945	0	0	0	A
5	TIGRP2P71774_rs9240394	48341772	0	0	0	G
5	BICF2P384624	48349170	G	0	0.2857	C
5	TIGRP2P71781_rs8738964	48360935	0	0	0	C
5	BICF2P781415	48370597	0	0	0	G
5	BICF2P781411	48373003	A	0	0	G
5	BICF2P1271569	48378004	0	0	0	A
5	BICF2P414650	48384857	C	0	0.2857	A
5	TIGRP2P71793_rs8764066	48391366	0	0	0	G
5	TIGRP2P71795_rs8819957	48395722	0	0	0	A
5	BICF2P401308	48406766	C	1	1	G
5	BICF2G630187864	48446057	A	1	0.7143	G
5	BICF2G630187869	48453502	G	1	1	A
5	BICF2G630187888	48468139	C	1	1	G
5	TIGRP2P71809_rs8491038	48474779	G	0	0	A
5	BICF2P279275	48485845	A	0	0.2857	C
5	BICF2G630187901	48497433	A	1	1	G
5	BICF2G630187908	48522899	A	1	0.7143	C
5	BICF2P468245	48534047	A	1	0.7143	G
5	BICF2S23529098	48551980	A	0	0.2857	G
5	TIGRP2P71828_rs8550473	48559893	A	0	0	G
5	BICF2P1388567	48560525	A	1	0.7143	G
5	BICF2G630187927	48564869	G	1	1	A
5	BICF2G630187953	48581523	A	1	1	G
5	BICF2P1200980	48588720	A	0	0.2857	G
5	BICF2G630187970	48608611	G	1	1	A
5	BICF2G630187975	48612050	A	1	0.7143	G
5	TIGRP2P71831_rs8638282	48630303	0	0	0	A
5	BICF2G630187995	48637045	G	0	0	A
5	BICF2G630188001	48650051	A	0	0	T
5	BICF2P1423293	48660270	A	0	0	G
5	BICF2G630188042	48685964	A	1	0.7143	G
5	BICF2G630188045	48692940	G	0	0	A
5	BICF2G630188059	48708572	A	1	0.7143	G
5	BICF2G630188062	48714572	G	1	0.7143	A
5	BICF2G630188075	48729406	C	1	1	A
5	BICF2G630188080	48746336	G	1	0.7143	A
5	BICF2P251265	48756520	0	0	0	A
5	BICF2S23620647	48768926	G	1	1	A

5	BICF2P1010252	48773672	A	1	1	G
5	TIGRP2P71853_rs8814509	48801915	C	1	1	A
5	TIGRP2P71861_rs8965568	48816073	G	1	1	A
5	BICF2P1428306	48839040	A	1	1	G
5	BICF2P338180	48848198	A	0	0	T
5	BICF2P219609	48852997	A	0	0.2857	G
5	BICF2P895161	48867973	G	1	0.7143	A
5	BICF2P1257343	48876988	C	0	0	A
5	BICF2G630188091	48886520	A	1	0.7143	G
5	BICF2P866878	48907460	C	1	0.7143	G
5	BICF2G630188123	48920589	A	1	0.7143	G
5	BICF2G630188125	48921462	G	1	0.7143	A
5	BICF2G630188146	48932946	G	0	0.2857	A
5	BICF2G630188149	48944640	G	1	0.7143	A
5	BICF2P742832	48960515	A	0	0	C
5	BICF2S23430932	48971669	0	0	0	A
5	BICF2G630188179	48988920	A	0	0.2857	G
5	BICF2S23730100	48999644	G	0	0	A
5	BICF2G630188212	49010342	G	1	0.7143	A
5	BICF2G630188226	49020553	A	1	0.7143	G
5	BICF2G630188240	49031379	A	1	0.7143	G
5	BICF2G630188253	49040635	A	0	0.2857	G
5	BICF2P1421257	49052338	A	1	0.7143	G
5	BICF2G630188255	49052513	A	0	0.2857	G
5	BICF2P756122	49065505	C	0	0	A
5	BICF2S23737438	49072560	G	0	0	A
5	BICF2G630188283	49082442	A	0	0	G
5	BICF2G630188290	49100246	C	0	0	A
5	BICF2G630188293	49107657	A	1	0.7143	G
5	BICF2G630188303	49116502	A	0	0	G
5	BICF2S24116869	49131711	0	0	0	G
5	BICF2P781275	49148018	T	0	0	A
5	BICF2P100580	49154058	0	0	0	A
5	BICF2G630188323	49164091	G	0	0	A
5	BICF2G630188339	49174135	A	0	0	G
5	BICF2P1371496	49186658	0	0	0	A
5	BICF2G630188374	49207892	G	0	0	A
5	BICF2P1166770	49217076	A	0	0	G
5	BICF2P1166771	49217444	A	0	0	G
5	BICF2G630188388	49230621	G	0	0	A
5	BICF2G630188396	49233686	0	0	0	G
5	BICF2G630188406	49245046	A	0	0	C
5	BICF2G630188417	49261179	G	0	0	A
5	BICF2G630188426	49272644	A	0	0	G
5	BICF2G630188436	49279936	G	0	0	A
5	BICF2G630188459	49290037	G	0	0	A
5	TIGRP2P71922_rs9026470	49302350	C	1	1	A

5	BICF2G630188464	49321757	G	1	1	A
5	BICF2G630188471	49330334	A	1	1	G
5	BICF2P1000909	49333944	A	0	0	G
5	BICF2G630188484	49340493	A	1	1	G
5	BICF2G630188485	49340633	A	1	1	C
5	BICF2G630188491	49354815	A	0	0	G
5	BICF2P1278036	49363038	G	1	1	A
5	BICF2G630188518	49375178	A	0	0	G
5	BICF2G630188530	49383378	G	1	1	A
5	BICF2G630188536	49397019	C	1	1	A
5	BICF2G630188548	49411581	G	0	0	A
5	BICF2G630188553	49416795	A	1	1	G
5	BICF2G630188571	49444519	A	1	1	G
5	BICF2G630188573	49453351	A	1	1	G
5	BICF2S23222404	49478884	A	0	0	G
5	BICF2S23343169	49487828	A	0	0	G
5	BICF2P783410	49505972	0	0	0	C
5	BICF2P855616	49513375	A	0	0	G
5	BICF2P773187	49534633	A	0	0	G
5	BICF2P1106606	49550344	G	0	0	A
5	BICF2G630188596	49554234	A	0	0.2857	G
5	BICF2G630188599	49566681	A	0	0.2857	G
5	BICF2P1384417	49582319	G	0	0	A
5	BICF2P557905	49594917	A	0	0	G
5	BICF2P1211502	49601493	G	0	0	A
5	BICF2P11137	49613791	G	0	0	A
5	BICF2P82260	49622997	A	0	0	G
5	BICF2P998662	49636116	C	0	0	A
5	BICF2G630188609	49648706	C	0	0	A
5	BICF2S23048165	49677964	T	0	0.2857	A
5	BICF2G630188617	49689955	T	0	0.2857	A
5	BICF2P832162	49692954	0	0	0	T
5	BICF2P92384	49715707	0	0	0	A
5	BICF2G630188619	49745678	A	0	0.2857	G
5	BICF2S23018325	49752743	A	0	0.2857	G
5	BICF2P745610	49760643	0	0	0	A
5	BICF2G630188624	49790683	A	0	0.2857	G
5	BICF2G630188633	49802619	C	1	0.7143	A
5	BICF2G630188644	49815138	A	1	0.75	G
5	TIGRP2P72040_rs8637209	49820049	G	1	1	A
5	BICF2P281149	49832980	A	0	0	G
5	BICF2G630188658	49845668	A	0	0	G
5	BICF2G630188673	49854800	G	0	0	A
5	BICF2G630188697	49866374	G	0	0	A
5	BICF2G630188723	49889914	G	0	0	C
5	BICF2G630188727	49893072	G	0	0	A
5	BICF2G630188736	49900354	G	0	0	C

5	BICF2G630188740	49900564	C	0	0	A
5	BICF2S23043187	49919398	A	0	0	G
5	BICF2G630188746	49933380	A	0	0	G
5	BICF2G630188754	49945892	A	0	0	G
5	BICF2G630188772	49965822	A	0	0	G
5	BICF2G630188786	49971811	G	0	0	A
5	BICF2G630188798	49988587	A	0	0	G
5	BICF2G630188802	49992765	G	0	0	A
5	BICF2G630188814	50005191	C	0	0	A
5	BICF2S2357962	50024311	0	0	0	G
5	BICF2G630188822	50028802	A	0	0	G
5	BICF2G630188824	50038163	C	1	0.7143	G
5	BICF2G630188842	50056426	A	1	0.5	C
5	BICF2G630188847	50060252	A	1	0.7143	G
5	BICF2G630188851	50088599	C	0	0	A
5	BICF2G630188869	50102227	A	0	0.2857	G
5	BICF2G630188877	50107004	A	0	0.2857	G
5	BICF2G630188891	50117729	A	0	0	C
5	BICF2G630188914	50130244	A	1	1	G
5	BICF2P838494	50141027	C	0	0	A
5	TIGRP2P72055_rs8623838	50154961	0	0	0	A
5	BICF2P889552	50168752	G	0	0	A
5	BICF2G630188941	50177607	G	0	0	A
5	BICF2G630188963	50187749	G	0	0	A
5	BICF2G630188977	50199759	A	0	0	C
5	BICF2P1188662	50212403	G	0	0	A
5	BICF2P816447	50221989	G	0	0	A
5	BICF2G630188999	50240244	A	0	0	G
5	BICF2G630189008	50245423	A	0	0	G
5	BICF2G630189010	50255576	G	0	0	A
5	BICF2G630189029	50276072	A	0	0	G
5	BICF2G630189031	50278715	C	0	0	A
5	BICF2G630189038	50291416	A	0	0	G
5	BICF2G630189050	50301115	G	0	0	A
5	BICF2G630189061	50312836	A	0	0	C
5	BICF2G630189074	50326798	A	0	0	C
5	BICF2P445723	50348752	G	0	0	A
5	BICF2G630189118	50361806	G	0	0	A
5	BICF2G630189132	50371541	A	0	0	G
5	BICF2G630189148	50381560	A	0	0	G
5	BICF2G630189165	50395816	A	0	0	G
5	BICF2G630189198	50410207	A	0	0	C
5	BICF2G630189206	50417530	A	0	0.125	C
5	TIGRP2P72101_rs9005933	50433232	A	0	0	G
5	BICF2P157110	50445498	A	0	0	G
5	BICF2S2454671	50456371	0	0	0	G
5	TIGRP2P72103_rs8801543	50469550	G	0	0	A

5	TIGRP2P72106_rs8517100	50476903	A	0	0	G
5	BICF2G630189226	50505734	G	0	0	A
5	BICF2P942719	50529646	G	0	0	A
5	TIGRP2P72123_rs8878024	50550799	0	0	0	G
5	BICF2P118660	50558480	0	0	0	G
5	TIGRP2P72131_rs8705801	50565697	0	0	0	G
5	BICF2P1131825	50581624	0	0	0	A
5	BICF2P192244	50589844	0	0	0	G
5	BICF2P183793	50608958	0	0	0	A
5	BICF2G630189232	50626425	C	0	0	G
5	BICF2G630189235	50637458	A	0	0	G
5	BICF2G630189239	50657066	A	0	0	G
5	TIGRP2P72153_rs9163020	50668999	A	0	0	C
5	TIGRP2P72160_rs8508364	50697635	0	0	0	C
5	TIGRP2P72161_rs8575688	50707207	0	0	0	A
5	BICF2P1184636	50724747	A	1	0.7143	G
5	BICF2P385445	50726965	0	0	0	G
5	BICF2P556382	50748165	A	0	0	G
5	BICF2P578956	50764794	0	0	0	A
5	BICF2G630189251	50789784	A	0	0.2857	G
5	BICF2P613646	50796333	0	0	0	A
5	BICF2G630189275	50808029	G	0	0.2857	A
5	BICF2G630189299	50820355	G	0	0.2857	A
5	BICF2S243883	50828648	A	0	0	G
5	BICF2G630189318	50838115	A	0	0.2857	G
5	BICF2G630189330	50841807	A	0	0.2857	G
5	TIGRP2P72223_rs8630596	50854369	G	0	0	A
5	TIGRP2P72227_rs9188337	50864883	A	0	0.2857	G
5	BICF2P1144347	50878379	G	0	0.2857	A
5	BICF2G630189424	50901328	A	1	1	C
5	BICF2P786597	50914929	A	1	1	G
5	BICF2P349668	50924216	G	0	0	A
5	BICF2P1075763	50933064	A	1	0.7143	C
5	BICF2G630189427	50933790	A	0	0	C
5	BICF2P164477	50937430	A	1	0.7143	G
5	BICF2G630189430	50954208	C	0	0	A
5	BICF2P284528	50957856	A	0	0	G
5	BICF2G630189475	50984412	0	0	0	A
5	BICF2P961681	50993251	A	0	0	G
5	BICF2G630189479	51002870	A	0	0.25	G
5	BICF2P108137	51015178	A	0	0	G
5	BICF2S23454421	51025573	A	1	1	C
5	BICF2P1445767	51048754	G	0	0.2857	A
5	TIGRP2P72295_rs8907455	51071313	G	1	0.7143	A
5	TIGRP2P72296_rs8907459	51071979	0	0	0	A
5	BICF2P720600	51087104	A	0	0.2857	C
5	BICF2S23145177	51097644	A	1	1	G

5	BICF2S23713244	51111200	A	0	0.2857	G
5	BICF2P595750	51121559	A	0	0	G
5	BICF2P1326496	51131646	G	1	0.7143	A
5	BICF2P576113	51145440	A	1	0.7143	G
5	BICF2P460246	51180911	G	0	0.2857	A
5	BICF2P550455	51190172	0	0	0	A
5	TIGRP2P72329_rs8945238	51204151	C	0	0	A
5	BICF2S23335908	51215427	G	1	0.7143	A
5	BICF2P153341	51223109	G	0	0	A
5	BICF2P201184	51251794	0	0	0	A
5	BICF2G630189499	51265565	A	0	0.2857	G
5	BICF2G630189510	51282743	G	0	0.2857	A
5	BICF2P815511	51302744	C	0	0	G
5	BICF2G630189519	51322690	A	0	0.2857	G
5	BICF2G630189537	51332238	G	1	1	A
5	BICF2P954643	51345136	A	0	0	G
5	BICF2P1058252	51350026	G	1	1	A
5	BICF2S23524716	51372910	0	0	0	A
5	BICF2P824860	51390174	G	1	1	A
5	TIGRP2P72354_rs8664203	51404087	A	0	0	G
5	BICF2P741610	51412556	0	0	0	A
5	TIGRP2P72356_rs9030426	51431428	A	1	0.7143	G
5	BICF2G630189564	51431551	A	1	0.7143	G
5	BICF2G630189574	51448708	G	0	0.2857	A
5	BICF2S23143370	51456540	C	0	0	A
5	BICF2G630189592	51469589	C	0	0.2857	A
5	BICF2S22952978	51483806	A	0	0	T
5	BICF2G630189614	51488759	A	0	0	G
5	BICF2G630189626	51501381	A	0	0	G
5	BICF2S23519156	51509482	A	0	0.2857	G
5	BICF2G630189633	51520443	A	0	0	C
5	BICF2G630189646	51534992	A	1	0.7143	G
5	BICF2G630189652	51545452	G	0	0	A
5	TIGRP2P72370_rs9072559	51559989	G	1	0.7143	A
5	BICF2G630189667	51579558	A	1	0.7143	G
5	BICF2P1090252	51589627	A	0	0	G
5	BICF2G630189681	51600250	A	0	0	G
5	BICF2G630189684	51604093	G	0	0	A
5	BICF2G630189693	51614598	A	0	0	G
5	BICF2P566885	51624631	0	0	0	C
5	BICF2S23760191	51639046	A	0	0	C
5	BICF2P316670	51656972	A	0	0	T
5	BICF2G630189713	51670566	G	1	0.7143	A
5	BICF2P418608	51683930	A	1	0.7143	G
5	BICF2G630189719	51697464	G	1	0.7143	A
5	BICF2G630189722	51711321	G	0	0	A
5	BICF2P1172297	51721755	0	0	0	G

5	BICF2G630189726	51730260	A	0	0	C
5	BICF2S22916666	51740959	G	0	0	A
5	BICF2G630189735	51753750	A	0	0	G
5	BICF2G630189743	51775244	A	0	0.2857	G
5	BICF2G630189750	51794389	C	0	0.2857	A
5	BICF2P1006725	51798403	C	1	1	A
5	TIGRP2P72397_rs9006225	51807701	T	0	0.2857	A
5	BICF2G630189755	51823234	A	0	0	G
5	BICF2P940216	51831739	0	0	0	C
5	BICF2S23528959	51842046	A	0	0	G
5	BICF2P462792	51863289	A	1	1	C
5	BICF2G630189758	51867621	A	0	0	G
5	BICF2P473972	51872358	A	1	0.7143	G
5	BICF2G630189764	51872563	A	0	0	G
5	BICF2G630189772	51876778	A	0	0	G
5	BICF2S23448999	51891481	G	0	0.2857	A
5	BICF2G630189786	51906170	T	0	0	A
5	BICF2G630189790	51910699	0	0	0	G
5	BICF2G630189796	51915396	A	1	1	C
5	BICF2G630189830	51927276	C	1	1	A
5	BICF2G630189842	51942302	A	1	0.6667	C
5	BICF2P829379	51946093	0	0	0	G
5	BICF2G630189849	51964614	G	0	0.2857	A
5	BICF2P1046481	51970683	0	0	0	A
5	BICF2P1317579	51984575	A	1	0.7143	G
5	TIGRP2P72441_rs8565316	52000305	A	1	0.7143	C
5	TIGRP2P72443_rs8677242	52010416	G	1	0.7143	A
5	TIGRP2P72445_rs8975196	52016911	G	1	0.7143	A
5	BICF2P1243477	52037374	A	0	0	G
5	TIGRP2P72455_rs9232689	52052890	A	1	0.7143	G
5	TIGRP2P72460_rs8843321	52061770	G	1	0.7143	A
5	BICF2P653870	52075285	A	0	0	G
5	BICF2P299041	52100119	A	0	0	G
5	BICF2P644448	52125269	G	1	0.7143	A
5	TIGRP2P72483_rs9127121	52131873	G	0	0	A
5	BICF2S23529704	52149907	A	0	0	T
5	BICF2P977545	52153448	A	1	0.7143	G
5	BICF2P708036	52162303	0	0	0	G
5	BICF2P492365	52166071	G	0	0.2857	A
5	TIGRP2P72504_rs8748895	52178571	A	0	0	G
5	BICF2P460367	52199832	A	0	0	G
5	BICF2P569352	52217778	C	0	0.2857	A
5	BICF2P490173	52244410	G	0	0.2857	C
5	TIGRP2P72534_rs9087606	52252801	G	0	0.2857	A
5	TIGRP2P72538_rs9159219	52265490	0	0	0	A
5	BICF2P284159	52273256	A	0	0	G
5	BICF2S22930616	52280903	G	1	0.7143	A

5	TIGRP2P72550_rs8695048	52298200	G	0	0.2857	A
5	TIGRP2P72551_rs8964711	52306490	0	0	0	A
5	BICF2P1310782	52315333	G	1	0.7143	A
5	BICF2P1328896	52329937	A	1	0.7143	G
5	BICF2P102338	52340239	A	0	0	G
5	BICF2P80151	52349210	A	1	0.7143	G
5	BICF2S23244873	52393976	G	0	0	A
5	TIGRP2P72568_rs8626712	52397194	0	0	0	G
5	BICF2P556386	52406254	G	0	0	A
5	BICF2P1404981	52421872	A	1	0.7143	G
5	TIGRP2P72583_rs8845970	52430947	G	0	0	C
5	BICF2S2336757	52450603	G	0	0	A
5	BICF2P298129	52476846	G	1	1	A
5	TIGRP2P72607_rs8633043	52486063	A	1	1	T
5	BICF2P1300177	52505153	0	0	0	A
5	BICF2P1155240	52509415	G	0	0	A
5	TIGRP2P72628_rs8950654	52526260	A	0	0	G
5	BICF2P499875	52537203	A	0	0	G
5	BICF2P1190821	52544589	G	0	0	A
5	BICF2P1080350	52561183	G	1	1	A
5	BICF2P213671	52570364	A	0	0	C
5	TIGRP2P72674_rs8962319	52594588	0	0	0	C
5	BICF2P913925	52622811	A	0	0	G
5	BICF2P78353	52632803	G	1	1	A
5	TIGRP2P72680_rs8518845	52641903	G	1	1	A
5	BICF2P184221	52650426	A	0	0	G
5	BICF2P906049	52659717	A	0	0	G
5	BICF2P555354	52671293	A	1	1	G
5	BICF2P985597	52683997	G	1	1	A
5	BICF2S23621078	52708058	A	0	0	C
5	TIGRP2P72726_rs8609913	52721205	A	0	0	G
5	BICF2P1020322	52735449	G	0	0	A
5	TIGRP2P72735_rs8731289	52742721	A	0	0	G
5	TIGRP2P72744_rs8562131	52759318	A	1	1	G
5	BICF2P1389814	52765704	A	1	1	G
5	BICF2P283578	52790917	A	0	0	G
5	TIGRP2P72774_rs9157257	52803967	G	0	0	A
5	BICF2S2346153	52828335	A	1	1	C
5	BICF2P77375	52831723	A	0	0	G
5	BICF2P20310	52853287	A	0	0	C
5	BICF2P1471371	52855285	G	0	0	C
5	BICF2P604453	52872378	A	1	1	G
5	BICF2S23442504	52883592	C	1	1	A
5	BICF2P330737	52897341	G	0	0	A
5	TIGRP2P72807_rs8724021	52903077	G	1	1	A
5	BICF2S2323131	52918202	A	0	0	G
5	TIGRP2P72825_rs8502385	52921744	A	0	0	G

5	BICF2P874007	52956607	0	0	0	A
5	BICF2S2293218	52971705	G	0	0	A
5	BICF2S2343736	53002084	G	0	0	A
5	BICF2P1264564	53010789	A	0	0	G
5	BICF2P1396557	53055058	0	0	0	A
5	BICF2P1253543	53067727	G	0	0	A
5	BICF2P1214298	53081185	G	0	0	A
5	BICF2P1282816	53099511	A	1	1	G
5	BICF2S2341189	53108246	A	0	0	G
5	BICF2S23131957	53134151	A	0	0	C
5	BICF2P476978	53150914	G	0	0	A
5	BICF2P337146	53154486	A	0	0	C
5	BICF2P958437	53165188	G	0	0	A
5	BICF2P813727	53187409	C	0	0	A
5	BICF2P114893	53198823	0	0	0	A
5	BICF2S2322246	53204915	0	0	0	A
5	BICF2P1285549	53217906	A	0	0	G
5	BICF2P325845	53224077	C	0	0	A
5	BICF2S23520698	53241675	0	0	0	A
5	BICF2S2334544	53252611	A	0	0	G
5	TIGRP2P72860_rs9147420	53266203	A	0	0	G
5	BICF2S23118328	53272803	G	0	0	A
5	BICF2S2343001	53279732	0	0	0	G
5	BICF2P132698	53292621	G	0	0	A
5	BICF2P720673	53309907	A	0	0	G
5	BICF2P1163368	53318812	A	0	0	G
5	BICF2S23018058	53336490	A	0	0	G
5	BICF2P586593	53344207	G	0	0	C
5	BICF2P1324481	53364577	A	0	0	G
5	TIGRP2P72882_rs8865436	53371173	G	0	0	A
5	BICF2P845796	53380092	A	0	0	G
5	BICF2P291789	53392305	G	0	0	A
5	BICF2P1386757	53396144	A	1	1	G
5	BICF2P1340165	53423246	A	0	0	G
5	BICF2P857994	53435286	A	0	0	T
5	TIGRP2P72914_rs8652483	53448970	G	0	0	A
5	BICF2P1280245	53459506	A	1	1	G
5	BICF2P421194	53461225	A	0	0	G
5	BICF2P590986	53469682	A	0	0	G
5	BICF2P614014	53476408	A	1	1	G
5	TIGRP2P72939_rs8773747	53494666	G	0	0	A
5	BICF2P791083	53498243	C	0	0	A
5	BICF2S23619668	53511523	A	0	0	G
5	TIGRP2P72947_rs9091807	53530171	0	0	0	A
5	BICF2P1091264	53545048	A	1	1	G
5	BICF2P623795	53563492	A	1	1	C
5	BICF2P297774	53570728	A	1	1	G

5	BICF2S23427581	53582312	A	0	0	G
5	BICF2P240089	53597746	A	1	1	G
5	TIGRP2P72958_rs8595976	53609386	G	0	0	A
5	BICF2P1147423	53626894	C	1	1	A
5	BICF2P427240	53636271	G	1	1	A
5	BICF2P289565	53655034	A	0	0	G
5	BICF2P650212	53664074	A	0	0	G
5	BICF2S2298298	53673377	A	0	0	G
5	BICF2P404783	53687444	G	0	0.2857	A
5	BICF2P1287283	53701768	A	1	0.7143	C
5	BICF2P1915	53710448	T	1	0.7143	A
5	BICF2P1022554	53720288	0	0	0	A
5	BICF2P728579	53735814	G	1	1	A
5	BICF2S23044413	53743192	A	0	0	C
5	BICF2P1162831	53751794	A	1	1	C
5	BICF2P1033898	53768573	G	1	0.7143	A
5	TIGRP2P72998_rs8991510	53776936	A	1	1	G
5	BICF2P1289100	53796144	C	1	0.7143	G
5	TIGRP2P73008_rs8616224	53796343	C	1	1	G
5	TIGRP2P73009_rs8616226	53797457	G	1	1	A
5	BICF2P655673	53813197	C	0	0	G
5	TIGRP2P73026_rs8803245	53820695	A	0	0.2857	G
5	TIGRP2P73036_rs9090878	53834903	A	1	0.7143	C
5	BICF2S22930406	53857263	G	1	1	A
5	BICF2P206746	53868645	T	1	0.7143	A
5	BICF2P1187649	53879069	A	1	1	C
5	BICF2P576789	53899318	G	0	0	A
5	BICF2P1307722	53906797	A	0	0.2857	G
5	BICF2P1024075	53922174	A	1	0.7143	G
5	BICF2P571722	53927834	A	1	1	G
5	TIGRP2P73097_rs9216599	53941386	G	0	0.2857	A
5	BICF2S23252373	53965840	G	0	0	A
5	BICF2P988690	53977500	C	1	0.7143	A
5	BICF2P1084522	53984611	G	1	0.7143	A
5	TIGRP2P73111_rs8638496	54002497	A	0	0	G
5	BICF2P1062905	54015148	A	1	1	G
5	TIGRP2P73125_rs8647601	54017712	G	0	0	A
5	BICF2P406736	54031599	G	0	0	A
5	BICF2S2301754	54052233	G	0	0	A
5	BICF2P1041109	54066642	A	0	0	G
5	TIGRP2P73173_rs9192893	54073853	G	0	0	A
5	TIGRP2P73195_rs9075785	54101913	0	0	0	A
5	BICF2P471600	54116203	G	0	0	A
5	BICF2P1242746	54119668	0	0	0	A
5	BICF2P529554	54130772	A	0	0	G
5	BICF2P279210	54142978	C	0	0	A
5	BICF2P425661	54167590	0	0	0	A

5	BICF2P334751	54181238	G	0	0	A
5	BICF2P132146	54209078	A	0	0	G
5	TIGRP2P73235_rs9103464	54215489	0	0	0	G
5	BICF2P378508	54231348	A	0	0	C
5	TIGRP2P73253_rs8764434	54246492	A	0	0	G
5	BICF2S2339970	54265601	G	0	0	A
5	BICF2S23123459	54274262	G	1	1	A
5	TIGRP2P73280_rs8716956	54293460	G	1	1	A
5	TIGRP2P73287_rs8688696	54314200	A	0	0	G
5	BICF2P1336778	54333422	A	0	0	G
5	BICF2S23349827	54338697	A	0	0	C
5	BICF2P1349570	54356315	G	0	0	A
5	BICF2S23332356	54360526	G	0	0	A
5	TIGRP2P73305_rs9166594	54375408	A	0	0	G
5	BICF2P982367	54388000	0	0	0	C
5	TIGRP2P73313_rs8495446	54395396	0	0	0	G
5	BICF2P1361406	54413166	A	1	1	C
5	BICF2S2343004	54422388	C	0	0	A
5	TIGRP2P73335_rs8608516	54438251	G	1	1	A
5	BICF2P1151094	54442717	G	0	0	A
5	BICF2P796192	54452960	A	0	0	G
5	TIGRP2P73348_rs8989397	54474478	A	0	0	G
5	TIGRP2P73350_rs9155015	54475715	A	0	0	G
5	BICF2P595972	54491755	G	0	0	A
5	TIGRP2P73372_rs9062018	54515573	A	0	0	C
5	BICF2P1110259	54521656	A	1	1	C
5	BICF2P926995	54543956	C	1	1	A
5	BICF2P580306	54554829	A	0	0	G
5	BICF2P1124104	54564821	C	0	0	A
5	TIGRP2P73415_rs9136142	54565830	C	0	0	A
5	BICF2P388200	54568597	C	0	0	A
5	BICF2P1395672	54572244	G	0	0	C
5	BICF2P710089	54582216	G	1	1	A
5	BICF2P710090	54582269	G	0	0	A
5	BICF2P914363	54587936	C	1	1	A
5	BICF2P346015	54591596	A	0	0	G
5	BICF2P945533	54606497	A	1	1	G
5	BICF2P626729	54614400	C	0	0	A
5	BICF2P305702	54637609	A	0	0	G
5	BICF2P434707	54650283	A	0	0	G
5	BICF2P1083180	54663649	G	0	0	A
5	BICF2S23427002	54672536	A	1	0.7143	C
5	BICF2S23634774	54689288	A	1	0.7143	C
5	BICF2P641943	54696564	G	1	0.7143	A
5	BICF2P1133974	54699216	A	1	0.7143	G
5	BICF2P10642	54703444	A	1	0.7143	C
5	BICF2P163180	54712984	G	1	0.7143	A

5	BICF2P217209	54728145	A	0	0	C
5	TIGRP2P73441_rs8656989	54734323	0	0	0	A
5	BICF2P93211	54743982	A	0	0	G
5	BICF2P380874	54758429	0	0	0	A
5	BICF2P1145021	54767869	A	0	0	C
5	BICF2P279387	54783511	A	0	0	G
5	BICF2P1114380	54789239	A	0	0	G
5	TIGRP2P73461_rs8691599	54798263	A	0	0	G
5	BICF2S23229423	54816226	A	0	0	G
5	BICF2P642286	54843106	0	0	0	G
5	BICF2P642287	54843838	0	0	0	A
5	TIGRP2P73469_rs8816552	54867315	0	0	0	A
5	TIGRP2P73472_rs8885698	54879375	0	0	0	A
5	BICF2S23547409	54892387	A	0	0	G
5	TIGRP2P73474_rs8857033	54903342	A	0	0	G
5	BICF2P114108	54915282	0	0	0	A
5	BICF2S23658302	54930170	C	0	0	A
5	BICF2S23517150	54944407	T	0	0	A
5	BICF2S23033911	54962431	0	0	0	C
5	BICF2P284000	54991421	C	0	0	A
5	TIGRP2P73550_rs8955501	54995363	A	0	0	C
5	BICF2S23161961	55007296	G	0	0	A
5	BICF2S23660272	55026850	T	0	0	A
5	BICF2S23351020	55043523	G	0	0	A
5	BICF2P1404006	55061717	0	0	0	C
5	TIGRP2P73555_rs8964490	55074181	0	0	0	T
5	TIGRP2P73556_rs8914703	55102818	A	0	0.1429	C
5	BICF2P1153745	55109050	A	0	0.1429	G
5	BICF2S23052465	55121196	C	0	0	A
5	TIGRP2P73571_rs9199026	55136460	A	0	0	G
5	BICF2P1231219	55150726	A	0	0.1429	G
5	BICF2S23122998	55158719	G	0	0	A
5	BICF2S23133037	55166163	G	0	0	A
5	BICF2P1236411	55182542	G	1	1	A
5	BICF2P1236407	55182885	G	0	0	A
5	BICF2P1148990	55212186	G	0	0	A
5	BICF2P1439605	55224932	G	1	0.8571	A
5	TIGRP2P73615_rs8953511	55242154	G	1	0.8571	A
5	BICF2P671511	55253494	G	0	0	A
5	TIGRP2P73625_rs8672880	55270216	A	0	0	G
5	BICF2S2399594	55283003	C	0	0	A
5	BICF2P1140554	55298562	G	0	0	A
5	BICF2P1430426	55303493	T	0	0	A
5	BICF2P612311	55305504	A	0	0	G
5	BICF2S23033955	55317674	G	0	0	A
5	TIGRP2P73637_rs8689653	55341696	A	0	0.1429	C
5	BICF2S23521501	55385542	A	0	0	G

5	TIGRP2P73649_rs8531435	55402922	A	0	0	G
5	BICF2P495439	55415152	A	0	0	G
5	TIGRP2P73652_rs8754614	55419100	G	0	0.1429	A
5	BICF2P215300	55435469	A	0	0.1429	G
5	BICF2P1166272	55451006	G	0	0	A
5	BICF2S23755139	55474311	G	1	0.8571	C
5	BICF2S23750469	55478061	A	0	0.1429	G
5	BICF2P625705	55500310	G	0	0	C
5	TIGRP2P73671_rs9040148	55505982	G	0	0	C
5	BICF2P1185405	55513332	A	1	1	G
5	BICF2P835463	55527379	A	0	0	G
5	BICF2P835464	55528241	T	1	1	A
5	BICF2S23722833	55536121	G	0	0	A
5	BICF2P345316	55547618	G	0	0	A
5	BICF2P1254176	55560906	C	0	0	A
5	BICF2P95061	55575451	G	1	1	A
5	BICF2S23447550	55580160	C	1	0.8571	A
5	BICF2P384981	55595164	C	0	0.1429	A
5	BICF2P561184	55602030	T	0	0	A
5	BICF2S23346221	55608357	0	0	0	A
5	BICF2P442900	55620169	A	0	0.1429	C
5	BICF2S23454554	55660427	A	0	0	G
5	BICF2P478343	55673388	G	1	1	A
5	BICF2S23110842	55726278	T	0	0	A
5	BICF2P865506	55730734	A	0	0.1429	G
5	TIGRP2P73719_rs8796772	55741910	A	0	0	G
5	BICF2P560403	55761999	G	0	0.1429	A
5	BICF2P560412	55763554	A	0	0.1429	G
5	BICF2P170588	55780623	A	1	0.8571	G
5	BICF2P441225	55819622	0	0	0	G
5	BICF2S24112818	55821177	0	0	0	G
5	BICF2P1062625	55835501	0	0	0	A
5	BICF2P668730	55853387	0	0	0	A
5	BICF2P1122012	55882369	G	1	1	A
5	BICF2P577823	55891675	A	1	1	G
5	BICF2P1040203	55909751	G	0	0.1429	A
5	BICF2P655614	55914457	0	0	0	G
5	BICF2P892826	55925426	G	0	0.1429	A
5	BICF2P116815	55942562	A	1	1	G
5	BICF2P942932	55954218	G	1	0.8571	A
5	BICF2P874797	55967186	A	0	0	G
5	TIGRP2P73761_rs9065473	55976235	A	0	0	T
5	BICF2S23426150	55987596	C	1	1	A
5	BICF2P1014265	55991538	G	0	0	A
5	BICF2S23623906	55997198	A	1	0.8571	G
5	BICF2P99989	56015697	G	0	0	A
5	BICF2P1425293	56017287	G	0	0	A

5	BICF2P619042	56048456	G	1	0.8571	A
5	BICF2P735711	56051983	C	1	0.8571	G
5	BICF2P305086	56071527	0	0	0	G
5	BICF2P1171382	56077015	A	1	1	G
5	BICF2P520787	56078029	G	1	1	A
5	BICF2P417250	56093037	A	1	1	G
5	BICF2S23012036	56099182	A	0	0	G
5	BICF2P125723	56109903	C	1	1	A
5	BICF2P66983	56126773	0	0	0	A
5	BICF2P1361849	56147328	G	1	1	A
5	BICF2P1453921	56162384	G	0	0	A
5	BICF2P338915	56167610	G	0	0	A
5	BICF2P103684	56184112	G	0	0.1429	A
5	BICF2P21235	56199928	G	1	1	A
5	BICF2S2374826	56216652	A	0	0	G
5	BICF2P415323	56232363	A	0	0.1429	G
5	BICF2P938692	56235165	C	1	1	A
5	BICF2P123925	56270416	0	0	0	A
5	BICF2P1262146	56326911	A	0	0.1429	G
5	BICF2P115705	56341206	G	0	0	A
5	BICF2P226666	56357691	A	1	0.8571	G
5	BICF2P59539	56364732	0	0	0	A
5	BICF2S22963273	56379568	A	0	0.1429	G
5	BICF2P957897	56393870	A	0	0	G
5	BICF2S23654220	56400446	G	0	0.1429	A
5	BICF2P1419536	56420999	A	1	1	G
5	BICF2P696143	56448301	A	0	0.1429	G
5	TIGRP2P73810_rs8738272	56461302	0	0	0	C
5	BICF2P213318	56478336	A	0	0.1429	G
5	BICF2P599784	56491050	A	0	0	G
5	BICF2P1346770	56509464	0	0	0	G
5	BICF2P460294	56512422	A	0	0.1429	G
5	BICF2S23624472	56534479	G	0	0	A
5	TIGRP2P73813_rs8866094	56547694	0	0	0	C
5	BICF2P287589	56565288	A	1	1	G
5	BICF2P1424794	56568134	C	1	1	A
5	BICF2P1207646	56569000	A	1	1	G
5	BICF2P500586	56571560	A	1	1	G
5	BICF2P669228	56584643	G	0	0.1429	A
5	BICF2P507129	56591899	C	0	0	A
5	BICF2P134523	56606405	0	0	0	A
5	BICF2S23751438	56616662	G	0	0	A
5	BICF2P5275	56648359	G	0	0.08333	A
5	BICF2S23643500	56658005	A	0	0	G
5	BICF2P356200	56661767	A	0	0	C
5	BICF2P514039	56682739	C	0	0	A
5	BICF2S23649940	56687597	0	0	0	A

5	BICF2P296772	56698636	0	0	0	G
5	BICF2P297574	56712768	A	0	0.1429	G
5	BICF2P1425500	56720005	A	1	0.8571	G
5	BICF2P93073	56730511	0	0	0	A
5	BICF2P156295	56751662	G	0	0	A
5	BICF2P1451725	56757988	A	0	0	G
5	BICF2P1331513	56762997	A	1	0.8571	G
5	BICF2P949000	56767647	G	0	0	A
5	BICF2P530111	56785060	A	0	0	G
5	BICF2P1295926	56808156	G	0	0	A
5	BICF2P520189	56813401	0	0	0	G
5	BICF2P296260	56821902	A	0	0	G
5	G348f29S259	56838815	0	NA	0	A
5	TIGRP2P73817_rs9151544	56854895	0	0	0	A
5	BICF2P1311036	56867094	A	0	0.1429	G
5	BICF2P277677	56871806	A	0	0.1429	T
5	BICF2P136419	56901789	0	0	0	G
5	G349f38S161	56927943	A	0	0.1429	G
5	BICF2S22960475	56958729	G	0	0.1429	A
5	BICF2P1438467	56961693	A	0	0	G
5	BICF2P884891	56982084	A	0	0	G
5	BICF2P852466	57014208	0	0	0	G
5	BICF2P1183078	57017031	0	0	0	A
5	BICF2P1135106	57032184	0	0	0	G
5	BICF2P206666	57074141	C	1	1	A
5	BICF2P1008946	57077615	A	0	0	G
5	BICF2P679597	57094031	G	1	1	A
5	BICF2P449153	57101015	A	0	0	G
5	BICF2P728930	57110117	C	0	0	A
5	BICF2S23646198	57128137	A	0	0	C
5	BICF2P1412444	57155207	A	0	0	G
5	BICF2P1037474	57168752	A	0	0	C
5	BICF2P77326	57180161	A	1	1	G
5	TIGRP2P73821_rs9167540	57196387	0	0	0	G
5	BICF2S23449404	57204210	G	0	0.1429	A
5	BICF2S23256201	57217847	G	1	1	A
5	BICF2S23720220	57232502	G	1	0.8571	A
5	BICF2P650512	57241072	A	0	0.1429	G
5	BICF2S23643009	57251901	A	0	0.1429	G
5	BICF2P183298	57264745	A	1	1	G
5	BICF2P1283419	57282425	A	0	0.1429	G
5	BICF2P243218	57301363	0	0	0	A
5	BICF2S23616008	57314907	G	1	0.8571	A
5	BICF2S23120710	57322976	A	1	0.8571	G
5	BICF2S23031741	57338144	A	1	0.8571	G
5	BICF2P184880	57343627	G	1	0.8571	A
5	BICF2P207683	57354035	A	1	0.8571	G

5	BICF2P721170	57366389	A	1	0.8571	G
5	BICF2P828526	57374914	G	1	0.8571	A
5	BICF2P1390528	57392208	G	1	1	C
5	BICF2P853840	57402580	C	1	1	G
5	BICF2S2311289	57417789	A	0	0	G
5	BICF2S23763447	57440117	G	0	0.1429	A
5	BICF2P1033222	57452175	A	1	0.8571	G
5	BICF2S23143997	57454080	A	0	0	C
5	BICF2P590622	57492656	A	0	0.1429	G
5	BICF2P1413095	57501346	A	0	0	G
5	BICF2P1012952	57516231	A	0	0	G
5	BICF2S23754149	57537811	G	0	0	A
5	TIGRP2P73826_rs8614351	57589528	0	0	0	G
5	BICF2S23137199	57593274	G	1	1	A
5	BICF2P560343	57604668	0	0	0	A
5	BICF2P164781	57618197	A	0	0.1429	C
5	BICF2P1079460	57636971	A	0	0.1429	G
5	BICF2P1049308	57643185	G	0	0.1429	A
5	BICF2S23423876	57676511	G	0	0.2857	A
5	BICF2P1363293	57708716	C	0	0.2	A
5	TIGRP2P73828_rs8624577	57728231	0	0	0	G
5	BICF2P637072	57736572	A	0	0.1429	G
5	BICF2P618438	57737874	A	0	0.1429	G
5	BICF2P540679	57744956	A	0	0.1429	G
5	BICF2P1409826	57766215	0	0	0	A
5	BICF2S23134807	57783220	A	0	0	C
5	BICF2P1320133	57804395	A	0	0.1429	G
5	BICF2S23518229	57811123	G	0	0.1429	A
5	BICF2P931809	57843352	A	0	0	C
5	BICF2P262071	57849566	0	0	0	C
5	BICF2P130934	57867594	0	0	0	A
5	BICF2P164739	57878270	0	0	0	A
5	BICF2P891262	57887172	A	0	0	G
5	BICF2S23717988	57893453	A	0	0	G
5	BICF2P1232732	57903612	G	0	0	C
5	BICF2P1158852	57915134	G	0	0	A
5	BICF2P652278	57954486	G	0	0	A
5	BICF2P354429	57968053	A	0	0.1429	G
5	BICF2P176489	57983543	A	0	0.1429	G
5	BICF2P832744	57995875	A	0	0.1429	T
5	BICF2P3419	58008377	0	0	0	A
5	BICF2S23021451	58033215	A	1	0.8571	G
5	BICF2S2364117	58070017	A	0	0.2857	G
5	BICF2P885328	58084565	A	0	0	G
5	BICF2S23216522	58089339	G	0	0	A
5	BICF2P673585	58119200	C	0	0.2857	A
5	BICF2P1431760	58124167	0	0	0	A

5	BICF2P133848	58140219	0	0	0	G
5	BICF2S23647936	58144866	A	0	0	G
5	BICF2S23044774	58157756	A	0	0	G
5	BICF2S23717817	58169929	G	0	0	A
5	BICF2P1111142	58220621	0	0	0	G
5	BICF2P66756	58235967	0	0	0	G
5	BICF2P1025407	58241006	A	1	0.7143	G
5	BICF2P32366	58256046	A	1	1	G
5	BICF2P1165068	58267199	A	0	0	G
5	BICF2S23732228	58274716	G	1	0.8571	A
5	BICF2S23051512	58291053	C	1	0.8571	G
5	BICF2P1455635	58296743	0	0	0	G
5	BICF2S23313815	58308989	A	0	0.1429	G
5	TIGRP2P73846_rs8929884	58327930	0	0	0	G
5	BICF2S2327675	58333238	A	0	0.1429	G
5	BICF2P548766	58344660	0	0	0	G
5	BICF2P496837	58351443	A	1	0.9167	G
5	BICF2P655028	58378311	0	0	0	C
5	BICF2P140412	58394201	0	0	0	G
5	BICF2P180121	58427413	0	0	0	A
5	BICF2S23160788	58448372	0	0	0	G
5	BICF2S2399924	58461937	A	0	0.1429	G
5	BICF2P1127509	58465014	G	0	0.1429	A
5	BICF2S23530901	58469767	C	0	0	A
5	BICF2P859521	58483247	G	0	0.2857	A
5	TIGRP2P73848_rs9164005	58492322	0	0	0	C
5	BICF2P687109	58510047	G	1	1	A
5	BICF2P606241	58523440	A	1	0.7143	G
5	BICF2P1208954	58527562	G	0	0.1429	A
5	BICF2S23758199	58536201	C	0	0	A
5	BICF2S2335536	58549508	A	0	0	G
5	BICF2S23635090	58590938	G	0	0	A
5	BICF2P1034640	58614993	G	0	0	A
5	BICF2P384451	58616905	G	0	0	A
5	BICF2P815877	58630166	G	0	0.1429	A
5	BICF2P1166755	58649092	G	0	0	A
5	BICF2S233824	58657699	A	0	0	G
5	BICF2P973678	58666248	A	0	0.1429	G
5	BICF2P29371	58673493	A	0	0	G
5	BICF2S23644875	58686741	A	0	0	G
5	BICF2P86068	58699817	A	1	0.7143	T
5	BICF2S2337585	58707692	A	0	0.1429	G
5	BICF2P76776	58720278	A	0	0.1429	G
5	BICF2P1037429	58735478	A	0	0.1429	G
5	BICF2S2348206	58750699	G	0	0	A
5	BICF2S2374194	58761977	A	0	0	G
5	BICF2S2374274	58765585	A	0	0	G

5	BICF2S23451135	58790110	G	1	0.7143	A
5	BICF2S2447028	58807639	0	0	0	G
5	BICF2P829769	58812746	0	0	0	C
5	BICF2P1411748	58824097	G	1	0.7143	A
5	BICF2P723475	58861898	A	0	0	G
5	BICF2S22955914	58887081	G	0	0	A
5	BICF2P666334	58897049	T	0	0	A
5	G350f28S66	58934767	A	0	0	G
5	BICF2S23327776	58970656	A	0	0.1429	G
5	BICF2P1246470	58986298	A	0	0	G
5	BICF2P303707	59019257	0	0	0	C
5	BICF2S23133419	59033520	A	0	0	G
5	G351f19S158	59058333	0	0	0	C
5	BICF2S22931227	59089267	C	0	0	A
5	BICF2P1290359	59137606	G	0	0	A
5	BICF2P1013393	59145414	A	0	0	C
5	BICF2P376584	59172521	A	0	0	G
5	BICF2P1399847	59181016	0	0	0	A
5	BICF2P86010	59192287	G	0	0	A
5	BICF2S23755890	59202762	G	0	0.1429	A
5	BICF2P333047	59208734	0	0	0	G
5	BICF2P528235	59226321	G	0	0.1429	A
5	BICF2P1289805	59239424	A	1	0.8571	C
5	BICF2P899650	59250849	G	0	0.1429	A
5	BICF2S23536184	59257466	G	1	0.8571	A
5	BICF2P1301992	59264746	0	0	0	G
5	BICF2P1237403	59275431	A	1	0.8571	G
5	BICF2P1030618	59286429	A	1	0.8571	G
5	BICF2P200733	59296978	G	0	0	A
5	BICF2P1451505	59328765	C	1	0.8571	A
5	BICF2S23216682	59351226	A	0	0	G
5	BICF2S23529355	59361569	G	1	0.8571	A
5	BICF2P1108442	59365149	A	0	0	G
5	TIGRP2P73867_rs9191093	59382844	0	0	0	C
5	BICF2P73892	59411910	A	0	0	G
5	BICF2P514268	59456285	0	0	0	C
5	BICF2S23537627	59486408	G	1	0.8571	A
5	BICF2S23018858	59510797	0	0	0	G
5	BICF2P1171973	59522860	0	0	0	G
5	BICF2S22963543	59554074	A	0	0	G
5	BICF2S23554129	59572227	C	1	0.8571	A
5	BICF2P1041396	59582705	A	0	0	C
5	BICF2S23438281	59593771	G	0	0	A
5	BICF2S2413175	59661467	G	0	0.1429	A
5	BICF2P132009	59670036	0	0	0	G
5	BICF2P1302363	59675059	A	1	0.8571	C
5	BICF2S22955550	59686896	0	0	0	C

5	BICF2S23417359	59705789	G	1	0.8571	A
5	BICF2S2355886	59716831	T	1	0.8571	A
5	BICF2S23636715	59741152	0	0	0	G
5	BICF2P641906	59745328	0	0	0	G
5	BICF2S23250226	59784236	A	1	1	G
5	BICF2S2354351	59791019	C	1	0.8571	G
5	BICF2P324332	59803222	0	0	0	A
5	BICF2P372348	59813421	0	0	0	A
5	BICF2S23158542	59859030	0	0	0	G
5	BICF2P854840	59875568	0	0	0	A
5	BICF2S23533471	59900123	A	0	0	G
5	BICF2P204770	59914675	0	0	0	C
5	BICF2P1245717	59923373	A	0	0	T
5	BICF2S24311425	59927394	G	0	0	A
5	BICF2P24404	59941583	0	0	0	A
5	BICF2S23620705	59951359	0	0	0	G
5	BICF2P7498	59969674	A	0	0	G
5	BICF2P919944	59989288	G	0	0	A
5	BICF2P164246	59995551	0	0	0	C
5	BICF2P659174	60008650	A	1	0.8571	G
5	BICF2P971364	60017973	C	0	0	A
5	BICF2P573035	60029234	A	0	0	G
5	BICF2P878456	60033083	0	0	0	C
5	BICF2S22929699	60049801	G	0	0	A
5	BICF2P322883	60057305	A	0	0.1429	G
5	BICF2P365153	60062123	A	0	0	G
5	BICF2S23655756	60063887	A	0	0	G
5	BICF2P1307859	60065450	A	0	0	G
5	BICF2P1266762	60079673	A	0	0	G
5	BICF2P1460429	60089384	A	1	0.8571	G
5	BICF2S23350610	60100621	A	0	0.1429	C
5	BICF2S22964286	60112714	A	1	0.7143	G
5	BICF2P1426032	60124549	A	0	0.1429	G
5	BICF2P917755	60140618	G	0	0	A
5	BICF2S23518605	60155859	A	1	0.7143	G
5	TIGRP2P73874_rs8907494	60183342	0	0	0	G
5	BICF2P135556	60195435	0	0	0	A
5	BICF2P1168900	60206683	G	1	0.75	A
5	BICF2P227260	60220126	0	0	0	C
5	BICF2P309939	60228223	0	0	0	C
5	BICF2P883839	60246112	C	1	0.7143	A
5	BICF2P496082	60250571	A	1	0.875	G
5	BICF2P1029667	60261977	C	1	0.7143	A
5	BICF2P840319	60276317	A	1	0.7143	G
5	BICF2P568092	60283617	G	1	0.7143	A
5	BICF2S23658639	60302233	A	1	0.7143	G
5	BICF2S23613937	60315043	0	0	0	C

5	BICF2P716130	60325438	A	0	0	C
5	BICF2P1071049	60349288	A	1	0.7143	G
5	BICF2P224611	60352746	0	0	0	G
5	BICF2P601	60372410	A	1	0.8571	G
5	BICF2P649426	60379786	A	1	1	G
5	BICF2P257149	60391081	A	1	0.7143	C
5	BICF2P629229	60406125	0	0	0	C
5	BICF2P1186456	60418721	C	0	0.1429	A
5	BICF2P370959	60435609	C	0	0.1429	G
5	BICF2S23146611	60454123	A	1	0.8571	G
5	BICF2P1260976	60457620	C	1	0.8571	A
5	BICF2P339176	60479042	A	0	0	G
5	BICF2S23739501	60495282	A	1	0.7143	G
5	BICF2S23745508	60502450	A	1	0.7143	G
5	BICF2S23132895	60524086	G	1	0.8571	A
5	BICF2S23042067	60525942	A	1	1	G
5	BICF2P551347	60542162	0	0	0	A
5	BICF2P1296243	60549519	C	0	0	A
5	BICF2P430775	60561888	G	0	0	A
5	BICF2P1000085	60576493	G	0	0	A
5	BICF2S23137920	60582492	G	0	0	A
5	BICF2P492463	60600758	G	0	0.1429	A
5	BICF2P1224372	60605892	A	0	0.1429	C
5	BICF2P1050290	60618077	G	0	0	A
5	BICF2P930967	60637219	A	1	0.8571	G
5	BICF2P306092	60647937	G	1	0.8571	A
5	BICF2P426477	60650658	A	0	0	G
5	BICF2P426491	60651669	A	1	1	G
5	BICF2P1008528	60666131	A	0	0	G
5	BICF2P545482	60693445	A	0	0	G
5	BICF2P55220	60700019	0	0	0	G
5	BICF2S23721356	60708612	A	0	0.1429	C
5	BICF2P807942	60722738	T	0	0	A
5	BICF2P684523	60734432	0	0	0	A
5	BICF2P681320	60735904	0	0	0	G
5	BICF2P224450	60751478	A	0	0	G
5	BICF2P1291561	60760631	G	1	0.7143	A
5	BICF2P1263770	60774189	G	0	0.1429	A
5	BICF2P113158	60779790	A	0	0	G
5	BICF2P275580	60790283	A	1	0.8571	G
5	BICF2S23015528	60804420	A	0	0.1429	G
5	BICF2S23158519	60819480	A	1	0.8571	G
5	BICF2S23420988	60825225	A	0	0	G
5	BICF2P576047	60840651	A	0	0	G
5	BICF2P198906	60855772	A	0	0	G
5	TIGRP2P73881_rs8961452	60862578	0	0	0	C
5	BICF2S23644675	60875079	G	0	0.1429	A

5	BICF2P543708	60882263	0	0	0	C
5	BICF2S2309075	60895991	G	0	0.2857	A
5	BICF2P701197	60902980	0	0	0	C
5	BICF2S2344926	60912194	G	0	0.1429	A
5	BICF2P1062567	60919189	0	0	0	G
5	BICF2S23739122	60931806	G	0	0.1429	A
5	BICF2S23022921	60944228	G	0	0.1429	A
5	BICF2P585831	60959876	0	0	0	C
5	BICF2S23013436	60964286	G	0	0	A
5	BICF2P531176	60980808	A	0	0.1429	G
5	BICF2P261246	60992066	G	0	0	C
5	BICF2S23147246	61003015	A	0	0	G
5	BICF2P926	61009539	A	0	0	G
5	BICF2P615855	61020612	C	1	0.7143	G
5	BICF2P789241	61029262	G	0	0.1429	A
5	BICF2P938745	61033753	A	1	0.8571	G
5	BICF2P308439	61044607	A	0	0.1429	G
5	BICF2S23055692	61057940	A	1	0.7143	G
5	BICF2S23612325	61070004	A	0	0	G
5	BICF2S22951128	61072878	A	0	0.1429	G
5	BICF2S23344066	61084402	A	0	0	G
5	BICF2S23126597	61090881	A	1	0.7143	G
5	BICF2S22964047	61101002	A	1	0.7143	G
5	BICF2P1322256	61116175	A	0	0.1429	G
5	BICF2P734496	61122335	A	1	0.8571	C
5	BICF2S2338996	61130468	G	1	0.8571	A
5	BICF2S22940587	61135750	G	0	0.1429	A
5	BICF2P111279	61148211	C	1	0.7143	G
5	TIGRP2P73884_rs8991562	61165204	0	0	0	C
5	BICF2P838405	61175647	G	0	0	A
5	BICF2S23034181	61184653	G	0	0.2857	A
5	BICF2S23536448	61194707	G	0	0.1429	A
5	BICF2P1323558	61207635	0	0	0	G
5	BICF2P1031795	61221963	A	0	0	G
5	BICF2P1097136	61228962	G	0	0	A
5	BICF2P103106	61242034	G	0	0	A
5	BICF2P502586	61250687	A	0	0	G
5	BICF2P509456	61278980	A	0	0	G
5	BICF2P369361	61288210	0	0	0	G
5	BICF2P620440	61303054	0	0	0	G
5	BICF2S24115746	61331312	G	0	0	A
5	BICF2S23644248	61350080	G	0	0	A
5	BICF2S23651339	61361959	0	0	0	G
5	BICF2P1371030	61388118	0	0	0	C
5	BICF2P787130	61409813	0	0	0	G
5	BICF2P423861	61432360	0	0	0	T
5	TIGRP2P73890_rs9187903	61437994	0	0	0	C

5	BICF2S23629342	61453717	G	0	0.1429	A
5	BICF2S23749773	61457202	G	0.25	0.2857	A
5	BICF2S23759548	61479265	A	0	0.1429	G
5	BICF2P1367053	61497389	A	0.25	0.2857	G
5	BICF2S23629239	61497824	A	0.75	0.7143	G
5	BICF2S23553461	61498887	G	0.75	0.7143	A
5	BICF2P726067	61507357	G	0.25	0.1429	A
5	BICF2S23654628	61524808	A	0	0	C
5	BICF2P316254	61533129	A	0	0.1429	G
5	BICF2P145549	61546076	C	0	0.1429	A
5	BICF2S23320766	61548092	G	0	0.1429	A
5	TIGRP2P73894_rs8875693	61563191	0	0	0	A
5	BICF2P1198823	61575773	0	0	0	G
5	BICF2P1386655	61598979	0	0	0	C
5	BICF2P921696	61607925	0	0	0	A
5	TIGRP2P73895_rs9140236	61628441	0	0	0	G
5	BICF2S23347069	61632724	A	0.75	0.7143	G
5	BICF2S23049013	61647863	G	0.75	0.7143	A
5	BICF2P1025641	61662394	G	0.25	0.1429	A
5	BICF2P1293606	61666432	G	0	0	A
5	BICF2P25010	61671651	A	0	0.1429	C
5	BICF2P578358	61676536	G	0	0.1429	A
5	BICF2P154295	61704410	C	0.75	0.7143	A
5	BICF2P990315	61710781	G	0.75	0.8571	A
5	BICF2P204722	61721289	G	1	1	A
5	BICF2P1364613	61726388	C	1	1	G
5	BICF2S23742197	61726755	C	1	1	G
5	BICF2P792978	61748350	0	0	0	A
5	BICF2P917530	61760513	A	0.25	0.1429	G
5	BICF2P1233480	61786333	A	0	0	G
5	BICF2S24322835	61799697	A	0	0	G
5	BICF2S23640766	61804936	G	0.75	0.8571	C
5	BICF2P295506	61815695	0	0	0	T
5	BICF2S23153845	61833676	A	0.25	0.1429	C
5	BICF2S23410224	61842712	A	0.25	0.1429	G
5	BICF2P771991	61855654	G	0.25	0.1429	A
5	BICF2P1377532	61859810	G	0.25	0.1429	A
5	BICF2P948872	61880881	A	0	0.1429	G
5	BICF2S2323655	61885534	G	0.25	0.2857	A
5	BICF2S23743523	61894381	A	0	0	G
5	BICF2S23148902	61913657	A	0.25	0.2857	G
5	BICF2S23018664	61928153	0	0	0	G
5	BICF2S23450135	61939844	A	0	0.1429	G
5	BICF2P1387692	61953132	A	0	0.1429	C
5	BICF2P1417125	61972499	A	0	0	C
5	BICF2P1249260	61979603	G	0.25	0.1429	A
5	BICF2P652209	61990991	A	0.75	0.7143	G

5	BICF2S23033273	62014826	A	0	0.1429	G
5	BICF2P1056685	62022403	0	0	0	G
5	BICF2P162259	62031503	0	0	0	C
5	BICF2P110863	62050695	A	0	0	C
5	BICF2P628228	62054806	A	0	0	G
5	BICF2P230961	62067802	A	0	0	G
5	BICF2P1385513	62070342	C	0	0	A
5	BICF2S23748248	62088994	A	0	0	G
5	BICF2P952785	62108424	A	0	0	G
5	BICF2S23122797	62113347	A	0.75	0.7143	C
5	BICF2P375889	62128119	A	0.75	0.7143	G
5	BICF2S23114580	62134478	A	0.25	0.08333	G
5	TIGRP2P73915_rs8564849	62144420	0	0	0	A
5	BICF2P828236	62146702	0	0	0	C
5	BICF2S23134813	62158552	G	0.25	0.1429	A
5	BICF2P788297	62178178	0	NA	0	A
5	BICF2S23655820	62184572	G	0.25	0.1429	A
5	BICF2P1302497	62194064	0	0	0	G
5	BICF2P386638	62215937	A	0	0	G
5	BICF2P195246	62243645	C	0	0	A
5	BICF2P345338	62260333	T	0	0	A
5	BICF2P1032	62262473	A	0.25	0.1429	C
5	BICF2P494613	62281854	0	0	0	A
5	BICF2P3735	62294933	0	0	0	G
5	BICF2P395433	62305652	A	1	1	G
5	BICF2S22951187	62312688	G	NA	1	A
5	BICF2P856717	62329417	0	0	0	G
5	BICF2P663814	62331465	0	0	0	G
5	BICF2P1306408	62349167	A	1	1	G
5	BICF2P1129904	62356671	T	1	0.8571	A
5	BICF2P1046219	62358943	C	0.25	0.1429	A
5	BICF2P403862	62363747	A	0	0	G
5	BICF2S23762207	62369677	A	0.75	0.7143	G
5	BICF2P166943	62377852	A	0	0	G
5	BICF2P1030100	62392561	0	0	0	G
5	BICF2P883787	62408156	A	1	0.8571	G
5	BICF2P162925	62412895	G	1	0.8571	A
5	BICF2P941409	62424558	G	1	0.8571	A
5	BICF2P967798	62437093	G	0	0	A
5	BICF2S23757774	62445935	A	0	0	G
5	BICF2P226251	62473659	G	0.25	0.1429	A
5	BICF2P603693	62478402	A	1	1	G
5	BICF2P1185301	62484399	A	0	0	T
5	BICF2P314157	62488337	G	1	1	A
5	BICF2P1218861	62497480	A	0	0	G
5	BICF2S23139721	62497731	A	0.25	0.2857	G
5	BICF2S23529341	62504166	G	0	0	A

5	BICF2S22964157	62516475	A	0.75	0.7143	G
5	BICF2P662028	62528476	A	0.75	0.7143	G
5	BICF2P395661	62551398	A	0.75	0.7143	G
5	BICF2S23742056	62568325	G	0	0	A
5	BICF2S22951983	62572467	G	0	0	A
5	BICF2P547063	62574151	G	0	0	A
5	BICF2P547062	62574345	G	1	0.8571	A
5	BICF2P451858	62587654	0	0	0	C
5	BICF2P982582	62595780	G	0	0.1429	A
5	BICF2S2298492	62607920	A	0	0.1429	G
5	BICF2P1408505	62617296	A	0	0.1429	G
5	BICF2P1134477	62620708	A	0	0	G
5	BICF2P837559	62631864	G	0	0.1429	A
5	BICF2P238860	62632720	G	0	0.1429	A
5	BICF2P523496	62640790	A	0	0.1429	G
5	BICF2S22923497	62655017	A	0	0.1429	G
5	BICF2P314054	62664566	0	0	0	G
5	BICF2P419954	62681603	A	0	0	G
5	BICF2P864419	62697757	G	0	0.1429	A
5	BICF2P43154	62704672	A	0	0.1429	G
5	BICF2P644285	62717698	G	1	0.8571	A
5	BICF2P599779	62721323	A	0.25	0.1429	C
5	BICF2P284536	62737838	C	0.75	0.8571	G
5	BICF2P340616	62739158	A	0.75	0.8571	G
5	BICF2P586796	62748765	G	0	0	A
5	BICF2S23617122	62762892	A	0	0	G
5	BICF2P1065513	62769820	A	0.75	0.7143	G
5	BICF2P109813	62791093	C	0.75	1	A
5	BICF2P425961	62806275	0	0	0	T
5	BICF2S2339876	62814100	A	0.25	0.1429	G
5	BICF2S2305634	62825773	A	0	0	C
5	BICF2P364621	62838076	0	0	0	A
5	BICF2S23649793	62851384	0	0	0	G
5	BICF2S23551305	62865891	A	0.25	0.1429	G
5	BICF2S23551304	62866239	A	0.25	0.1429	G
5	BICF2P381728	62875525	0	0	0	G
5	BICF2P1130383	62881806	A	0.25	0.1429	G
5	BICF2S23251419	62882935	A	0.25	0.1429	G
5	BICF2P1471945	62899060	A	1	0.8571	G
5	BICF2P734582	62906422	A	0.25	0.1429	G
5	BICF2P895865	62920447	G	0.25	0.1429	A
5	BICF2P779271	62928346	A	1	0.8571	G
5	BICF2P779272	62928369	A	1	0.8571	G
5	BICF2P150665	62929254	C	0.75	0.7143	A
5	TIGRP2P73922_rs8706568	62944160	0	0	0	C
5	BICF2P1247515	62960567	0	0	0	G
5	BICF2P379558	62973600	A	0	0.1429	G

5	BICF2S23542432	62977993	A	0	0.1429	G
5	BICF2P58604	62985884	A	0.25	0.1429	G
5	BICF2S23625200	63005709	G	0.75	0.7143	A
5	BICF2P932245	63026179	C	1	0.75	G
5	BICF2P533881	63037190	A	0	0	G
5	BICF2P958242	63057251	A	0	0	C
5	BICF2P398795	63076415	A	0.25	0.1429	G
5	TIGRP2P73923_rs8816621	63088795	0	0	0	G
5	TIGRP2P73925_rs8624162	63091516	0	0	0	A
5	BICF2P203155	63107338	A	0.25	0.1429	G
5	BICF2P420332	63118860	C	0	0	A
5	BICF2P1130664	63126048	G	0.25	0.1429	A
5	BICF2P181378	63136846	A	0	0	C
5	BICF2P555702	63155494	0	0	0	A
5	BICF2P505656	63162250	A	0	0	G
5	BICF2P910269	63177558	0	0	0	G
5	BICF2S23152601	63188144	G	0.75	0.8571	A
5	BICF2P1342085	63200014	A	0	0	G
5	BICF2S23757016	63210215	A	0.25	0.1429	G
5	BICF2P621003	63221479	A	0	0	T
5	BICF2S23731658	63230044	A	0.75	0.8571	G
5	BICF2P1099792	63243480	0	0	0	C
5	BICF2P184948	63281387	A	0	0	G
5	BICF2P903729	63282534	G	0	0	A
5	BICF2P664412	63287197	G	0.75	0.7857	A
5	TIGRP2P73928_rs8675763	63297010	0	0	0	G
5	BICF2S23116616	63309161	0	0	0	G
5	BICF2S2451694	63320612	A	0	0	G
5	TIGRP2P73930_rs8977458	63341459	0	0	0	G
5	BICF2S23642687	63352591	A	0	0	C
5	BICF2P364190	63374771	0	0	0	A
5	BICF2P359197	63403005	C	0	0	A
5	BICF2P538597	63411776	A	0	0	G
5	BICF2P571085	63431677	G	0	0	A
5	BICF2P646842	63435029	A	0.25	0.1429	C
5	BICF2P1207574	63467286	A	0.25	0.2143	G
5	BICF2P286234	63476571	A	0.5	0.6429	G
5	BICF2P1870	63486165	A	0.25	0.2143	G
5	BICF2P1191829	63492385	0	0	0	A
5	BICF2P710738	63524164	A	0.5	0.6429	T
5	BICF2P1002098	63537122	A	0	0	G
5	BICF2P1181199	63544208	A	0.25	0.1429	G
5	BICF2P116463	63549364	A	0.25	0.1429	G
5	BICF2P890916	63568036	C	0	0	G
5	BICF2S2334987	63573484	A	0	0	G
5	BICF2P1238650	63585519	G	0.5	0.3571	A
5	BICF2P459537	63604188	C	0.5	0.3571	A

5	BICF2P590230	63616356	G	0.5	0.6429	A
5	BICF2P1058413	63629122	A	0.5	0.3571	C
5	BICF2P1077109	63633777	0	0	0	G
5	BICF2P1264195	63649662	A	0.5	0.3571	G
5	TIGRP2P73938_rs8652474	63658120	0	0	0	A
5	BICF2P306915	63665707	0	0	0	C
5	BICF2P988353	63676571	0	0	0	G
5	BICF2P954835	63693463	A	0.5	0.6667	G
5	BICF2P117500	63702773	A	0.5	0.6429	G
5	BICF2P700435	63718972	G	0	0	A
5	BICF2S23328017	63727644	A	0.75	0.7857	G
5	BICF2S22941126	63736475	A	0.75	0.7857	G
5	BICF2P1263414	63745775	C	0.75	0.7857	A
5	BICF2P476733	63756253	G	0.25	0.2143	A
5	BICF2P193794	63777135	G	0	0	A
5	BICF2P1230869	63788259	A	0	0	G
5	BICF2P1181614	63794303	A	0.25	0.2143	G
5	BICF2P1259651	63795017	A	0	0.1429	C
5	BICF2P255178	63817785	A	0.25	0.2143	G
5	BICF2P575866	63822540	A	0.25	0.2143	C
5	BICF2P984200	63828640	A	0.25	0.1429	C
5	BICF2P210043	63853602	A	0.25	0.2143	G
5	BICF2P749237	63855578	G	0.25	0.2143	A
5	BICF2P891738	63860162	A	0.25	0.2143	G
5	BICF2P105189	63873964	A	0.25	0.2143	G
5	BICF2P753046	63885172	A	0.75	0.7857	G
5	BICF2P1162252	63910385	C	0.25	0.1429	A
5	TIGRP2P73940_rs8894040	63978994	0	0	0	G
5	BICF2P159532	64024966	0	0	0	A
5	BICF2S23616382	64035047	G	0.25	0.2143	A
5	BICF2S23714189	64038300	A	0.5	0.3571	C
5	BICF2S2329199	64044420	A	0.25	0.2143	G
5	BICF2S22910865	64058057	G	0.25	0.2143	A
5	BICF2S23512139	64069727	G	0.25	0.1429	A
5	BICF2P459900	64082210	0	0	0	A
5	BICF2P492346	64090595	0	0	0	A
5	BICF2S23323363	64109520	A	0	0	T
5	BICF2S23325358	64113324	G	0	0	C
5	BICF2S23339468	64139010	G	0	0	A
5	TIGRP2P73944_rs8855125	64154659	0	0	0	C
5	BICF2S23418861	64162444	G	0.5	0.6429	A
5	BICF2S24113320	64177818	0	0	0	G
5	BICF2P1111296	64187315	0	0	0	A
5	BICF2S23422048	64215092	G	0.75	0.8571	A
5	BICF2S23054888	64225008	G	0.25	0.1429	C
5	BICF2P131865	64248145	0	0	0	A
5	BICF2S23729463	64261843	G	0.5	0.6667	A

5	BICF2P789962	64262973	G	0	0	A
5	BICF2P789964	64263475	A	0	0	G
5	BICF2P393975	64273884	0	0	0	T
5	BICF2P284102	64287286	G	0	0	A
5	BICF2S23251601	64304532	A	0.75	0.8571	G
5	BICF2P904869	64308018	0	0	0	A
5	BICF2P1023180	64326385	0	0	0	C
5	BICF2S23553838	64340765	A	0.5	0.3571	G
5	BICF2S2358214	64347512	A	0	0	G
5	BICF2P1298080	64366209	A	0	0	G
5	BICF2S24116755	64387736	G	0	0	A
5	BICF2P1344258	64405086	C	0	0	G
5	BICF2P198413	64413174	0	0	0	G
5	BICF2P872522	64438437	0	0	0	G
5	TIGRP2P73951_rs8712216	64452060	0	0	0	A
5	BICF2S23626885	64458416	0	0	0	G
5	BICF2P228016	64480380	0	0	0	G
5	BICF2P721056	64504388	0	0	0	G
5	BICF2S23260404	64534918	A	0.75	0.7857	C
5	BICF2P589653	64549832	G	0	0	C
5	BICF2S24110385	64572153	G	0	0	A
5	BICF2P1156229	64578516	A	0	0	C
5	BICF2S23741020	64614327	A	0	0	T
5	BICF2S23042158	64623207	A	0	0	G
5	BICF2P585992	64637884	G	0.25	0.2143	A
5	BICF2P828345	64671412	A	0	0	C
5	BICF2P644972	64676488	G	0.25	0.2143	A
5	BICF2S22920210	64698044	G	0	0	A
5	BICF2P208716	64710056	A	0.5	0.6429	G
5	BICF2P897153	64719235	A	0.5	0.3571	G
5	BICF2P1019000	64725909	G	0	0	A
5	BICF2P592562	64731967	C	0	0	G
5	BICF2S23723037	64735144	A	0.5	0.3571	G
5	TIGRP2P73955_rs9145818	64754756	0	0	0	C
5	BICF2S2296202	64759902	G	0	0	A
5	BICF2P837905	64772353	A	0	0	G
5	BICF2P1167879	64785830	G	0	0	A
5	BICF2S23540037	64793041	G	0	0	A
5	BICF2P770743	64801713	A	0	0	G
5	BICF2P280497	64810545	A	0	0	G
5	BICF2P599131	64816044	A	0	0	G
5	BICF2P1334164	64841941	A	0	0	C
5	BICF2P583160	64850462	A	0	0	G
5	BICF2P76253	64868684	G	0	0	A
5	BICF2P885011	64872269	0	0	0	C
5	BICF2P485524	64887855	A	0	0	G
5	BICF2P77974	64906633	A	0	0	G

5	BICF2P852606	64936309	A	0	0	G
5	BICF2P959210	64947239	A	0	0	G
5	BICF2P591961	64962283	0	0	0	A
5	BICF2P1049057	64968325	C	0	0	A
5	BICF2P1252891	64975201	0	0	0	C
5	BICF2P1255647	65005563	C	0	0	A
5	BICF2P602271	65017673	0	0	0	A
5	BICF2P137180	65039318	0	0	0	C
5	BICF2S23746657	65055122	G	0	0	A
5	BICF2P798233	65058305	A	0	0	G
5	BICF2P1159424	65079821	C	0	0	A
5	BICF2P677921	65092233	G	0	0	A
5	BICF2P559352	65112634	A	0	0	G
5	BICF2P791429	65137373	A	0	0	T
5	BICF2S23748722	65183488	A	0	0	G
5	BICF2P901346	65201391	0	0	0	G
5	BICF2S23425854	65209599	C	0.75	0.7857	A
5	BICF2S22935060	65224353	0	0	0	C
5	BICF2S23335310	65230874	G	0	0	A
5	BICF2S23329871	65253957	C	0	0	A
5	BICF2S23633732	65267999	A	0	0	G
5	TIGRP2P73964_rs8805463	65300431	C	0	0	A
5	BICF2P1055973	65314494	G	0.25	0.2143	A
5	BICF2P1220990	65327351	G	0.25	0.1429	A
5	BICF2P869348	65332305	G	0	0.1429	A
5	BICF2S23652495	65354461	A	0	0	G
5	BICF2P84750	65356597	G	0	0	A
5	BICF2P794732	65374524	A	0.25	0.1429	G
5	BICF2S23646717	65378221	0	0	0	C
5	BICF2S2364368	65394827	G	0	0	A
5	BICF2S23525384	65406128	G	0	0	A
5	BICF2P1152604	65412097	A	0.75	0.7857	C
5	BICF2P373505	65424843	G	0.25	0.2143	A
5	BICF2P1145753	65436228	A	0.75	0.7857	C
5	BICF2P294513	65453232	0	0	0	A
5	BICF2P310097	65458859	A	0.75	0.7857	G
5	BICF2P926490	65475238	A	0	0	C
5	BICF2S23613434	65513339	A	0.25	0.2143	C
5	BICF2P1324303	65516498	0	0	0	G
5	BICF2S23121859	65543776	G	0.5	0.3571	A
5	BICF2S23030250	65554791	A	0.25	0.1429	G
5	BICF2S2298406	65572857	A	0.25	0.1429	G
5	BICF2P590932	65580375	A	0	0	C
5	BICF2P264665	65583805	A	0.5	0.6429	G
5	BICF2P487417	65598717	A	0	0	G
5	BICF2S23213370	65617952	G	0	0	A
5	BICF2P726705	65626903	C	0	0	A

5	BICF2P438923	65638159	A	0	0	C
5	BICF2P566787	65642605	G	0	0	A
5	BICF2P852277	65662615	G	0	0	A
5	BICF2P199241	65667824	A	0.5	0.6429	G
5	BICF2P775376	65678460	G	0.5	0.6429	A
5	BICF2P263118	65690301	A	0	0	G
5	BICF2P117484	65702013	A	0	0	C
5	BICF2P1266081	65732067	G	0	0	A
5	BICF2P885648	65740765	G	0	0	A
5	BICF2S23658549	65750026	A	0.25	0.1429	C
5	BICF2P339558	65768560	G	0	0	A
5	BICF2P631236	65795145	G	0	0	A
5	BICF2P950750	65813169	T	0	0	A
5	BICF2P139786	65824772	A	0	0	G
5	BICF2P4646	65826557	A	0	0	C
5	BICF2P167471	65841311	G	0	0	A
5	BICF2P875594	65856573	A	0	0	C
5	BICF2P1326030	65868568	A	0.5	0.3571	G
5	BICF2S23534689	65880179	A	0	0	G
5	BICF2P227960	65883831	A	0	0	G
5	BICF2P157295	65900638	A	0	0	G
5	BICF2P889074	65906730	G	0	0	C
5	BICF2P1345400	65923061	G	0.25	0.2143	A
5	BICF2S23434355	65929595	G	0	0.2143	A
5	BICF2P837417	65942280	C	0.25	0.2143	A
5	BICF2P280982	65957407	A	0	0	G
5	BICF2P999906	65976465	A	0	0	T
5	BICF2S22937361	66019001	G	0	0	A
5	BICF2P264790	66029693	C	0.25	0.2143	G
5	BICF2P1147056	66038436	G	0	0	C
5	BICF2S22921021	66051131	A	0.25	0.1429	G
5	BICF2P1340443	66067189	G	0	0.1429	A
5	BICF2S2336022	66068054	A	0.25	0.1429	G
5	BICF2P662214	66072118	A	0.25	0.1429	C
5	BICF2P662213	66072337	A	0.25	0.1429	G
5	BICF2P590431	66086380	A	0	0	G
5	BICF2P298506	66097097	G	0.75	0.7857	A
5	BICF2P458734	66107767	G	0.5	0.6429	A
5	BICF2P599460	66114778	0	0	0	G
5	BICF2P987741	66133216	A	0.75	0.9167	G
5	BICF2P137188	66143876	G	0	0	A
5	BICF2P1359421	66157198	G	0	0	A
5	BICF2P967379	66164077	A	0.75	0.8571	C
5	BICF2P85291	66175256	A	0.5	0.6429	G
5	BICF2P1195485	66191185	C	0	0	A
5	BICF2P922937	66194465	T	0.5	0.6429	A
5	BICF2P1040552	66222258	G	0.5	0.6429	A

5	BICF2P1138616	66239640	G	0.5	0.6429	A
5	BICF2S2327433	66261286	A	0	0	G
5	BICF2P1122940	66265898	G	0	0	A
5	BICF2P365667	66276959	A	0.5	0.6429	G
5	BICF2P127478	66289020	G	0.5	0.6429	A
5	BICF2P1410528	66297552	A	0	0	G
5	BICF2P178795	66311263	C	0.5	0.6429	A
5	BICF2S23219015	66323457	A	0	0	G
5	BICF2S23740064	66362971	0	0	0	A
5	BICF2P1027117	66366598	C	1	0.9167	A
5	BICF2P1008770	66381245	A	0.25	0.1429	G
5	BICF2P232151	66394771	0	0	0	C
5	BICF2P808731	66404859	C	0.25	0.1429	A
5	BICF2P173590	66423253	G	0	0	A
5	BICF2P124088	66429400	0	0	0	G
5	BICF2S23537042	66445530	G	0	0	A
5	BICF2S23325812	66456986	A	0	0	G
5	BICF2P945173	66465953	A	0.75	0.8571	G
5	BICF2P731816	66488558	G	0.75	0.8571	A
5	BICF2P244177	66489532	T	0.75	0.8571	A
5	BICF2P857625	66496108	G	0.75	0.8571	A
5	BICF2P1040066	66504537	A	0.75	0.8571	G
5	BICF2P645336	66518848	A	0.25	0.2143	G
5	BICF2P70559	66537234	G	0.75	0.8571	A
5	BICF2P900214	66549569	A	0.25	0.2143	G
5	BICF2S23425198	66559525	G	0	0	A
5	BICF2S23019847	66571130	A	0.25	0.2143	G
5	BICF2S2351224	66576208	A	0.25	0.2143	G
5	BICF2P301683	66586052	0	0	0	G
5	BICF2P153184	66598914	A	0	0	G
5	BICF2P855794	66613665	T	0.25	0.2143	A
5	BICF2P1288638	66620365	A	0.25	0.2143	G
5	BICF2P1107834	66637742	G	0.25	0.2143	A
5	BICF2S23444006	66639366	G	0	0	A
5	BICF2S23426377	66653826	A	0	0	G
5	BICF2S23624769	66664263	A	0	0	G
5	BICF2P260580	66669052	A	0.5	0.3571	C
5	BICF2P343766	66681390	A	0.25	0.1429	G
5	BICF2P854025	66693084	G	0.75	0.7857	A
5	BICF2P854028	66696051	G	0.5	0.6429	C
5	BICF2P601502	66708382	G	0.5	0.3571	A
5	BICF2P575049	66716173	G	0.5	0.3571	A
5	BICF2P831934	66726828	A	0.5	0.6429	G
5	BICF2P733518	66743328	0	0	0	G
5	BICF2S23547384	66768255	G	0.5	0.6667	A
5	BICF2P613327	66792280	G	0.5	0.6429	A
5	BICF2S23244461	66812669	A	0.5	0.6429	G

5	BICF2S23513441	66822663	A	0	0	G
5	BICF2P817031	66829648	G	0.5	0.6429	A
5	BICF2P865778	66840909	G	0.5	0.6429	A
5	BICF2P242469	66851701	A	0.5	0.6429	G
5	BICF2P906690	66863128	A	0.25	0.2143	G
5	BICF2S23510884	66883190	0	0	0	G
5	BICF2P1258819	66900853	G	0.25	0.2143	A
5	BICF2P81075	66909003	G	0	0	A
5	BICF2S22929501	66912576	A	0.75	0.7857	G
5	BICF2P296029	66926250	A	0	0	G
5	BICF2S23257208	66931215	G	0.5	0.6429	A
5	BICF2P302437	66964140	0	0	0	C
5	BICF2S22943205	66968865	G	0.5	0.3571	A
5	BICF2P350901	66978687	0	0	0	G
5	BICF2S22924625	66997931	G	0.5	0.3571	A
5	BICF2P217318	67005003	0	0	0	C
5	BICF2P631510	67022978	A	0	0	G
5	BICF2P1497690	67043806	G	0	0	A
5	BICF2P1352122	67062387	A	0.5	0.3571	C
5	BICF2P569338	67068039	C	0	0	A
5	BICF2P770287	67085884	G	0	0	A
5	BICF2P770286	67087007	A	0	0	G
5	BICF2P142110	67092365	A	0	0	T
5	BICF2P114074	67116098	G	0	0	A
5	BICF2P190824	67142519	G	0.5	0.6429	A
5	BICF2P811405	67149640	C	0	0	A
5	BICF2P501891	67172058	A	0.5	0.3571	G
5	BICF2P501890	67172242	A	0	0	G
5	BICF2P307030	67187803	0	0	0	A
5	BICF2P1393784	67197981	A	0	0	G
5	BICF2P1206786	67212249	A	0	0	G
5	BICF2P931406	67215880	G	0.5	0.6667	A
5	BICF2P779982	67222474	0	0	0	G
5	BICF2P452033	67235346	A	0	0	G
5	BICF2P126843	67260410	C	0.25	0.1429	A
5	BICF2S23241657	67297616	A	0.5	0.6429	G
5	BICF2P995400	67301293	A	0	0	G
5	G354f36S28	67319384	0	0	0	A
5	BICF2S23746792	67342420	G	0	0	A
5	BICF2P582738	67351124	A	0	0	G
5	BICF2S23764120	67356143	C	0	0	A
5	BICF2S24116343	67401378	0	0	0	C
5	BICF2S23252006	67402458	A	0.25	0.1429	G
5	BICF2P383304	67437450	0	0	0	G
5	BICF2P317290	67457321	A	0	0	C
5	BICF2P838630	67461998	A	0.75	0.8571	G
5	BICF2P95564	67497537	0	0	0	G

5	BICF2P549906	67524019	0	0	0	C
5	BICF2P689773	67549949	A	0	0	G
5	BICF2S23045390	67552633	A	0.25	0.1429	G
5	BICF2P1015480	67573954	A	0.25	0.1429	G
5	BICF2P381779	67576960	G	0.75	0.7857	A
5	BICF2P473103	67592407	C	0.5	0.3571	G
5	BICF2P414351	67608137	A	0.5	0.6429	G
5	BICF2P236614	67611755	A	0.5	0.6429	G
5	BICF2P391963	67627875	G	0	0	A
5	BICF2P721420	67652615	A	0.5	0.3571	G
5	BICF2S23123672	67659259	A	0.25	0.1429	G
5	BICF2P402395	67663263	G	0.5	0.3571	A
5	BICF2S23421788	67668216	A	0.5	0.3571	G
5	BICF2P95307	67678182	0	0	0	G
5	BICF2P279427	67707081	0	0	0	G
5	BICF2S23435618	67713262	A	0.25	0.2143	G
5	BICF2P772074	67732568	C	0.25	0.1429	A
5	BICF2P973352	67737551	C	0.25	0.1429	A
5	BICF2S24113801	67740450	G	0.25	0.2143	A
5	BICF2S22935158	67751339	A	0.5	0.6429	G
5	BICF2P483155	67752477	A	0.5	0.6429	G
5	BICF2P1316251	67767329	A	0.25	0.1429	G
5	BICF2P965647	67775316	A	0	0	G
5	BICF2P377442	67791922	A	0	0	G
5	BICF2P512317	67793983	G	0.25	0.1429	A
5	BICF2P512316	67794062	A	0	0	G
5	BICF2P597885	67801380	A	0.25	0.1429	G
5	BICF2P848956	67821641	0	0	0	G
5	BICF2S23046523	67832703	G	0	0	A
5	BICF2P940599	67839436	0	0	0	G
5	BICF2P1387272	67855671	0	0	0	G
5	BICF2P1295354	67867186	A	0	0	C
5	BICF2P1007652	67883709	G	0	0	A
5	BICF2S23740895	67912410	A	0.25	0.2143	G
5	BICF2S2331458	67919382	G	0	0	A
5	BICF2P577927	67952631	C	0	0	G
5	BICF2P577930	67955195	G	0	0	A
5	BICF2P370655	67986806	G	0	0	A
5	BICF2P1355548	67991138	A	0	0	G
5	BICF2P1175699	68004480	A	0	0	G
5	BICF2P742360	68009762	A	0	0	G
5	BICF2P388881	68020259	C	0	0	A
5	BICF2S22942411	68022531	A	0	0	C
5	BICF2S23131499	68044654	G	0	0	A
5	BICF2P416487	68063545	A	0	0	G
5	BICF2S22918688	68071732	G	0	0	A
5	BICF2P796488	68087057	C	0	0	A

5	BICF2P145786	68100867	A	0.25	0.08333	G
5	BICF2P195922	68107472	G	0.25	0.1429	C
5	BICF2P1175409	68116090	A	0.5	0.6429	G
5	BICF2P1425515	68132369	G	0.5	0.6429	A
5	BICF2P226937	68139713	A	0	0	G
5	BICF2P1181570	68156340	G	0.5	0.3571	A
5	BICF2P1269609	68167614	G	0.25	0.1429	A
5	BICF2P296647	68182815	A	0.5	0.6429	G
5	BICF2P519303	68188297	G	0.5	0.6429	A
5	BICF2P1218410	68192123	A	0.5	0.3571	G
5	BICF2P882024	68196577	A	0.5	0.6429	G
5	BICF2P1100899	68200396	A	0.5	0.6429	G
5	BICF2S23745026	68213962	A	0.5	0.3571	G
5	BICF2S23711828	68218539	A	0.5	0.3571	T
5	BICF2P616884	68230836	0	0	0	G
5	BICF2P1193120	68245518	0	0	0	G
5	BICF2S23344809	68252769	A	0.5	0.3571	G
5	BICF2P1225165	68267532	A	0.25	0.1429	G
5	BICF2S23532143	68277744	C	0.5	0.3333	A
5	BICF2S2323030	68287996	C	0.25	0.2143	G
5	BICF2P905700	68303296	G	0.5	0.3571	A
5	BICF2P24608	68314479	A	0.25	0.2143	G
5	BICF2P1270212	68327176	A	0.75	0.8571	G
5	BICF2P103450	68343893	A	0.25	0.2143	G
5	BICF2S237233	68349540	A	0	0	G
5	BICF2P416896	68363809	G	0.25	0.2143	C
5	BICF2S23219168	68372044	A	0.25	0.2143	G
5	BICF2P552323	68393959	A	0.25	0.2143	G
5	BICF2S23436293	68410197	C	0	0	A
5	BICF2S23322721	68435244	C	0	0	A
5	BICF2P94251	68436876	G	0	0	A
5	BICF2P515777	68451424	G	0	0	A
5	BICF2P56458	68466653	A	0	0	T
5	BICF2P542776	68481853	G	0.5	0.3571	A
5	TIGRP2P74042_rs9021762	68482973	0	0	0	C
5	BICF2S23159097	68496148	G	0.5	0.6429	A
5	BICF2P932869	68508811	A	0	0	C
5	BICF2P804974	68520814	G	1	1	A
5	BICF2P847619	68532753	A	0	0	G
5	BICF2P394707	68543290	C	1	1	A
5	BICF2P394706	68543610	C	1	1	A
5	BICF2P1275219	68546633	A	0	0	G
5	BICF2P71168	68555799	G	0.75	0.8571	A
5	BICF2P572765	68558161	A	0.75	0.8571	G
5	BICF2S23530493	68564524	A	0	0	G
5	BICF2P995532	68580063	A	0.5	0.3571	G
5	BICF2P258200	68588263	G	0.25	0.1429	A

5	BICF2P1400616	68597937	A	0.75	0.7857	G
5	BICF2P1394996	68615039	A	0	0	G
5	BICF2P989786	68630173	G	0.5	0.3571	A
5	BICF2P651730	68637766	A	0	0	G
5	BICF2P772897	68650812	G	0	0	A
5	BICF2P1230450	68663691	A	0	0	G
5	BICF2P245940	68673405	A	0	0	C
5	BICF2P699705	68699362	A	0.25	0.2143	G
5	BICF2P991235	68774140	A	0	0	G
5	BICF2P630232	68785060	A	0	0	G
5	BICF2P992773	68793022	A	0	0	G
5	BICF2P216142	68816354	G	0.25	0.1667	A
5	BICF2P1078243	68835481	0	0	0	G
5	BICF2P269901	68849706	A	0.25	0.2143	G
5	BICF2P178168	68857367	A	0	0	C
5	BICF2P126196	68862535	A	0.25	0.2143	C
5	BICF2S23444281	68864953	A	0	0	C
5	BICF2P1003979	68866198	A	0.75	0.7857	G
5	BICF2S23730537	68881020	C	1	1	G
5	BICF2S23520424	68892417	A	0	0	G
5	BICF2P89833	68898234	C	0.25	0.08333	A
5	BICF2P937925	68910417	C	0.5	0.3571	A
5	BICF2S23528026	68927781	T	0.5	0.6667	A
5	BICF2P1240934	68931194	A	0.25	0.2143	G
5	BICF2S23732438	68942719	A	0.5	0.3571	G
5	BICF2P486648	68957677	G	0.75	0.9167	A
5	BICF2S23433094	68966704	T	0.75	0.8571	A
5	BICF2S22947805	68977147	A	0	0	C
5	BICF2P616723	68995000	G	0.75	0.7857	A
5	BICF2P1254407	69032645	0	0	0	A
5	BICF2S23030455	69070220	G	0	0	A
5	BICF2P223987	69076323	A	0	0	G
5	BICF2S23660314	69084953	G	0	0	A
5	TIGRP2P74058_rs9206877	69095815	0	0	0	C
5	BICF2S23210717	69108099	A	0.25	0.1429	G
5	BICF2P481578	69136132	0	0	0	G
5	BICF2P894795	69203988	A	0	0	G
5	BICF2P891928	69215731	A	0	0	G
5	BICF2P1191301	69237278	A	0	0	G
5	BICF2P783831	69245474	0	0	0	G
5	BICF2P1436951	69255084	G	1	1	A
5	BICF2S22933109	69269475	G	0	0	A
5	BICF2P54597	69277174	A	1	1	G
5	BICF2P687902	69291059	A	0	0	G
5	BICF2P962301	69304382	A	0	0	G
5	BICF2S23154273	69306623	A	0	0	G
5	BICF2G63083146	69318755	A	0	0	G

5	BICF2G63083150	69322577	A	0	0	G
5	BICF2G63083170	69334604	A	1	1	G
5	BICF2P590826	69351798	G	0	0	A
5	BICF2G63083204	69365912	A	0	0	G
5	BICF2G63083262	69413843	G	0	0	A
5	BICF2G63083279	69426966	0	0	0	A
5	BICF2P938698	69438492	0	0	0	C
5	BICF2S2363313	69468831	G	0	0	A
5	BICF2P1215452	69512795	A	0	0	G
5	BICF2G630242634	69532100	A	0.25	0.2143	C
5	BICF2G630242607	69542273	G	0.75	0.7857	A
5	BICF2P1070301	69552518	A	0	0	T
5	BICF2P1070294	69553015	A	0	0	G
5	BICF2G630242586	69564102	A	1	1	G
5	BICF2G630242568	69578735	A	0.25	0.2143	G
5	BICF2G630242564	69596522	A	0	0	C
5	BICF2G630242563	69603343	G	1	1	A
5	BICF2G630242560	69609721	C	0	0	A
5	BICF2G630242545	69632081	A	0.25	0.2143	C
5	BICF2S23642280	69634558	G	0	0	A
5	BICF2G630242521	69648780	G	0	0	A
5	BICF2P351043	69663291	A	0.25	0.2143	G
5	BICF2G630242486	69675017	A	0	0	G
5	BICF2G630242477	69684936	A	0	0	G
5	BICF2G630242475	69685016	A	0	0	G
5	BICF2P1194497	69693172	A	1	1	G
5	BICF2G630242432	69705615	G	1	1	A
5	BICF2G630242387	69721784	0	0	0	G
5	BICF2G630242353	69730551	C	0	0	G
5	BICF2G630242342	69735342	A	0	0	G
5	BICF2P1404031	69763906	A	0.25	0.2143	G
5	TIGRP2P74069_rs9141074	69772365	0	0	0	T
5	BICF2G630242321	69793062	A	0	0	G
5	BICF2P788523	69806382	G	0	0	A
5	BICF2S23333761	69879641	A	0.75	0.7857	G
5	BICF2G630242237	69888456	0	0	0	G
5	BICF2G630242223	69897534	A	0.75	0.8333	G
5	BICF2G630242211	69912932	G	0	0	A
5	TIGRP2P74071_rs8877866	69921302	0	0	0	C
5	BICF2P1082888	69941485	A	0	0	G
5	BICF2G630242196	69946103	A	0	0	G
5	BICF2S23210169	69958686	G	0.25	0.2143	A
5	BICF2G630242180	69969778	G	0.75	0.7857	A
5	BICF2G630242175	69980476	0	0	0	G
5	BICF2G630242167	69995168	A	0	0	G
5	BICF2G630242159	70006166	A	0.25	0.2143	G
5	BICF2P1086234	70019083	A	0.75	0.7857	G

5	BICF2G630242119	70031708	A	0	0	G
5	BICF2G630242095	70043414	A	0	0	G
5	BICF2P205133	70050871	G	0.25	0.2143	A
5	BICF2P1316906	70069136	G	0	0	A
5	BICF2G630242089	70079099	A	0	0	G
5	BICF2S22922527	70087091	A	1	1	G
5	BICF2P108567	70105365	0	0	0	C
5	BICF2G630242072	70131616	A	0	0	C
5	BICF2G630242045	70156735	A	0	0	G
5	BICF2G630242040	70161991	G	0	0	A
5	BICF2G630242017	70176438	A	0	0	G
5	BICF2G630242012	70186030	A	0	0	G
5	BICF2G630241995	70200989	0	0	0	G
5	BICF2G630241993	70212750	A	1	1	G
5	BICF2G630241963	70235881	A	0	0	C
5	BICF2P1416176	70248344	0	0	0	G
5	BICF2P712007	70254402	G	0	0	C
5	BICF2G630241926	70255665	A	0	0	G
5	BICF2P655600	70267386	A	0	0	G
5	BICF2P1121745	70277970	A	0.25	0.2143	G
5	BICF2P158937	70279275	A	0.25	0.1667	G
5	BICF2G630241895	70289733	A	0	0	G
5	BICF2G630241884	70304014	0	0	0	G
5	BICF2P1456251	70315073	0	0	0	A
5	BICF2S2443239	70332612	A	0	0	G
5	BICF2P1079771	70335830	0	0	0	A
5	BICF2P1317221	70347104	A	0.75	0.7857	G
5	BICF2G630241882	70358833	G	0.25	0.2143	A
5	BICF2G630241879	70376098	G	0	0	A
5	BICF2G630241874	70381756	C	0	0	A
5	BICF2G630241850	70395337	A	0	0	C
5	BICF2G630241832	70410515	G	0	0	A
5	BICF2S23638002	70425953	A	1	1	G
5	BICF2G630241824	70427145	G	1	1	C
5	BICF2P69983	70443908	A	1	1	G
5	BICF2P173096	70461120	A	0	0	C
5	BICF2P78736	70478483	A	0	0	G
5	BICF2P1313120	70488868	0	0	0	A
5	BICF2P1244340	70504339	0	0	0	G
5	BICF2P851960	70545426	0	0	0	A
5	BICF2P304947	70569141	0	0	0	A
5	BICF2P1137673	70618033	G	0	0	A
5	BICF2G630241796	70632470	A	0	0	G
5	TIGRP2P74085_rs8576204	70639852	0	0	0	G
5	BICF2G630241779	70649120	A	1	1	G
5	BICF2G630241742	70659804	G	1	1	A
5	BICF2G630241723	70673418	G	0	0	C

5	BICF2G630241709	70684629	0	0	0	G
5	BICF2G630241705	70694436	G	0	0	A
5	BICF2G630241695	70702648	A	1	1	G
5	BICF2G630241673	70719755	0	0	0	C
5	BICF2G630241659	70732713	G	0	0	A
5	BICF2P1203151	70743819	A	0	0	G
5	BICF2G630241647	70749900	C	1	1	A
5	BICF2G630241643	70763995	A	0	0	G
5	BICF2G630241631	70778326	0	0	0	A
5	BICF2P339031	70782938	A	1	1	G
5	BICF2G630241626	70785876	G	1	1	A
5	BICF2G630241609	70807935	0	0	0	A
5	BICF2G630241608	70828986	G	0	0	A
5	BICF2G630241596	70840837	A	0	0	G
5	BICF2S23542472	70861378	A	0	0	G
5	BICF2G630241586	70894032	A	0	0	G
5	BICF2G630241579	70901696	A	0	0	G
5	BICF2G630241561	70911384	G	0	0	A
5	BICF2G630241559	70912302	A	0	0	G
5	BICF2G630241531	70921247	G	0	0	A
5	BICF2G630241518	70937238	A	1	1	G
5	BICF2G630241511	70946189	A	0	0	G
5	BICF2P859461	70962625	A	0	0	G
5	BICF2G630241472	70974384	G	1	1	A
5	BICF2G630241436	70988904	A	0	0	G
5	BICF2G630241419	70995182	C	1	1	A
5	BICF2G630241395	71001816	G	0	0	A
5	BICF2G630241393	71002044	G	0	0	A
5	BICF2G630241343	71014465	A	0	0	G
5	BICF2G630241308	71025645	A	0	0	G
5	BICF2G630241274	71045251	C	0	0	A
5	BICF2G630241272	71047816	G	1	1	A
5	BICF2G630241268	71051791	G	1	1	A
5	BICF2G630241250	71064832	A	0	0	G
5	BICF2G630241243	71071472	G	0	0	A
5	BICF2G630241216	71092552	G	0	0	C
5	BICF2G630241201	71098537	A	0	0	G
5	BICF2G630241187	71105309	A	0	0	G
5	BICF2P2846	71114870	A	1	1	G
5	BICF2S2357716	71132085	G	0	0	A
5	BICF2P836205	71137031	G	0	0	A
5	BICF2P1224497	71157114	G	0	0	A
5	BICF2P1297229	71167003	A	1	1	G
5	BICF2G630241089	71180977	A	0	0	G
5	BICF2G630241077	71192310	A	0	0	G
5	BICF2G630241040	71211852	A	0	0	G
5	BICF2G630241031	71224193	A	0	0	G

5	BICF2G630241008	71239919	A	0	0	G
5	BICF2G630240970	71281645	A	0	0	C
5	BICF2G630240954	71289201	G	0	0	A
5	BICF2G630240922	71311210	A	1	1	G
5	BICF2S23317800	71323937	A	0	0	G
5	BICF2G630240912	71337310	A	0	0	G
5	BICF2G630240909	71352216	A	0	0	G
5	BICF2G630240897	71363104	A	0	0	G
5	BICF2G630240872	71379229	A	0	0	G
5	BICF2S23033755	71386889	A	1	1	G
5	BICF2G630240867	71395332	A	0	0	G
5	BICF2P1296033	71404616	A	0	0	G
5	BICF2G630240851	71417317	G	0	0	C
5	BICF2S23045788	71437880	A	1	1	G
5	BICF2G630240845	71439654	A	0	0	T
5	BICF2G630240835	71455365	A	0	0	G
5	BICF2G630240820	71468560	A	0	0	G
5	BICF2S2323475	71477084	C	0	0	A
5	BICF2G630240812	71489926	G	1	1	A
5	BICF2G630240803	71497178	G	1	1	A
5	BICF2P719146	71521509	G	1	1	A
5	TIGRP2P74102_rs8812146	71545960	0	0	0	G
5	BICF2G630240757	71554439	A	0	0	G
5	BICF2G630240744	71564656	A	1	1	G
5	BICF2P407857	71577238	0	0	0	G
5	BICF2P1130435	71604605	G	0	0	C
5	BICF2G630240741	71615495	A	0	0	G
5	BICF2G630240740	71625117	C	1	1	A
5	BICF2P763446	71637761	A	0	0	G
5	BICF2P540963	71647797	A	0	0	G
5	BICF2S2338428	71674323	G	0	0	A
5	BICF2G630240730	71679900	A	0	0	G
5	BICF2P1198706	71699719	0	0	0	G
5	BICF2G630240727	71711226	A	0	0	G
5	BICF2S23254404	71723750	G	0	0	A
5	BICF2G630240718	71729849	C	1	1	G
5	BICF2G630240714	71738675	G	0	0	A
5	TIGRP2P74109_rs8779164	71762722	0	0	0	A
5	BICF2G630240688	71777290	A	0	0	G
5	BICF2G630240674	71785567	A	0	0	C
5	BICF2G630240665	71801145	G	0	0	A
5	BICF2G630240654	71807186	A	0	0	C
5	BICF2P1096540	71820505	G	0	0	A
5	BICF2G630240621	71833727	G	0	0	A
5	BICF2G630240612	71840561	C	0	0	A
5	BICF2G630240583	71865105	A	1	1	G
5	BICF2P262001	71876626	A	0	0	G

5	BICF2P827128	71881868	A	0	0	G
5	BICF2P637002	71884918	G	0	0	A
5	BICF2G630240524	71900303	G	0	0	A
5	BICF2G630240486	71919892	A	0	0	C
5	BICF2G630240481	71923108	G	0	0	A
5	BICF2G630240463	71941670	G	0	0	A
5	BICF2S22914258	71957903	A	0	0	G
5	BICF2G630240434	71970285	C	0	0	G
5	BICF2G630240420	71980370	G	0	0	A
5	BICF2G630240398	71993850	C	0	0	A
5	BICF2G630240391	71999836	A	0	0	G
5	BICF2G630240383	72003307	A	0	0	G
5	BICF2G630240356	72025848	G	1	1	C
5	BICF2S22918836	72026056	A	0	0	C
5	BICF2G630240328	72037218	G	0	0	A
5	BICF2G630240303	72053121	A	0	0	G
5	BICF2G630240278	72072533	A	0	0	G
5	BICF2G630240265	72084740	G	0	0	A
5	BICF2G630240249	72093926	C	0	0	A
5	BICF2G630240235	72105104	C	0	0	A
5	BICF2P472865	72120128	G	1	1	A
5	BICF2P363265	72133520	C	0	0	G
5	BICF2G630240203	72164485	A	0	0	G
5	BICF2G630240171	72171092	G	0	0	A
5	BICF2P1361702	72177047	A	0	0	G
5	BICF2P1462323	72255904	G	0	0	A
5	BICF2G630240149	72266887	A	0	0	G
5	BICF2P159123	72288489	G	0	0	A
5	BICF2S2345833	72311492	A	0	0	C
5	BICF2G630240133	72315642	A	0	0	G
5	BICF2G630240128	72333059	G	0	0	A
5	BICF2G630240118	72337792	A	0	0	G
5	BICF2P644863	72349173	A	0	0	G
5	BICF2G630240057	72364478	A	1	1	G
5	BICF2P1118980	72372253	G	0	0	A
5	BICF2S2299438	72382944	A	1	1	C
5	BICF2G630240049	72402769	G	1	1	A
5	BICF2G630240048	72405063	C	0	0	A
5	BICF2G630240025	72418578	G	0	0	A
5	BICF2G630240000	72433090	A	0	0	G
5	BICF2G630239994	72440696	G	0	0	A
5	BICF2G630239973	72451687	G	0	0	A
5	BICF2G630239952	72463717	C	1	1	A
5	BICF2G630239936	72473429	A	1	1	G
5	BICF2G630239899	72498035	G	0	0	A
5	BICF2G630239892	72504691	G	0	0	A
5	BICF2G630239888	72515660	A	0	0	C

5	BICF2P781770	72519278	C	0	0	A
5	BICF2G630239876	72532335	A	0	0	G
5	BICF2G630239862	72536241	A	0	0	G
5	BICF2G630239850	72543321	A	0	0	C
5	BICF2G630239829	72554750	G	0	0	A
5	BICF2P1162890	72571944	G	1	1	A
5	BICF2P287003	72577782	A	0	0	C
5	BICF2G630239803	72603225	G	0	0	A
5	BICF2G630239794	72607008	A	1	1	G
5	BICF2G630239777	72620574	G	1	1	A
5	BICF2G630239747	72638895	A	0	0	C
5	BICF2G630239731	72647480	A	0	0	G
5	BICF2P633093	72658303	0	0	0	A
5	BICF2S23156606	72670236	G	0	0	A
5	BICF2G630239705	72690257	0	0	0	G
5	BICF2P1267825	72700637	G	0	0	A
5	BICF2P1458515	72708781	G	0	0	A
5	TIGRP2P74125_rs8724700	72716248	0	0	0	G
5	BICF2P467885	72728221	A	0	0	G
5	BICF2P674068	72743180	A	1	1	G
5	BICF2P1214345	72754910	0	0	0	G
5	BICF2S23422605	72761552	A	0	0	G
5	BICF2S23128923	72772399	0	0	0	C
5	BICF2S23132939	72786292	A	0	0	C
5	BICF2P1134831	72805138	G	0	0	A
5	BICF2P1134833	72806954	A	0	0	G
5	BICF2P1134838	72808418	C	0	0	G
5	BICF2P784635	72825942	A	0	0	G
5	BICF2G630239699	72833241	G	0	0	A
5	BICF2P907867	72844614	0	0	0	A
5	TIGRP2P74129_rs8958825	72852658	0	0	0	A
5	BICF2G630239696	72895601	A	0	0	G
5	BICF2G630239687	72909717	A	0	0	G
5	BICF2G630239669	72916528	G	0	0	A
5	BICF2P75332	72930983	G	1	1	A
5	BICF2G630239653	72937144	G	0	0	A
5	BICF2G630239650	72947496	C	0	0	A
5	BICF2G630239639	72956060	C	0	0	A
5	BICF2G630239622	72976310	C	0	0	A
5	BICF2S23324064	72982383	T	0	0	A
5	TIGRP2P74133_rs8672560	72995297	0	0	0	G
5	BICF2G630239620	73005608	0	0	0	G
5	BICF2P221933	73017574	T	0	0	A
5	BICF2G630239617	73038433	A	0	0	G
5	BICF2G630239609	73052398	G	0	0	A
5	BICF2P330751	73065026	A	0	0	G
5	BICF2P330750	73065271	0	0	0	G

5	BICF2G630239602	73074399	C	0	0	A
5	BICF2G630239599	73089574	0	0	0	G
5	BICF2S2302711	73097059	G	0	0	A
5	BICF2G630239581	73109857	0	0	0	A
5	BICF2G630239562	73123006	0	0	0	A
5	BICF2G630239556	73128590	A	0	0	G
5	BICF2G630239541	73144801	G	0	0	A
5	BICF2G630239511	73162587	A	0	0	C
5	BICF2G630239510	73164119	A	0	0	G
5	BICF2G630239487	73185371	A	1	1	G
5	BICF2G630239484	73188256	A	0	0	G
5	BICF2P287436	73214645	A	0	0	G
5	BICF2P469356	73229433	G	0	0	A
5	BICF2G630239446	73232784	A	0	0	G
5	BICF2G630239398	73250624	A	0	0	G
5	BICF2G630239383	73260530	A	0	0	C
5	BICF2P98698	73266556	G	0	0	A
5	BICF2G630239334	73279356	G	1	1	A
5	BICF2G630239319	73295737	G	1	1	A
5	BICF2P747263	73302339	A	0	0	G
5	BICF2G630239316	73318458	A	1	1	G
5	BICF2G630239311	73333993	A	1	1	G
5	BICF2G630239304	73338054	G	0	0	A
5	BICF2G630239286	73351786	A	0	0	G
5	BICF2G630239269	73364115	A	0	0	G
5	BICF2P708903	73370597	G	0	0	C
5	BICF2P1423605	73381969	C	1	1	A
5	BICF2S23156063	73398168	A	0	0	G
5	BICF2P1451281	73424513	A	0	0	G
5	BICF2P768152	73433523	A	1	1	G
5	BICF2G630239258	73448997	A	1	1	G
5	BICF2G630239256	73450794	A	1	1	G
5	BICF2P744722	73462765	G	1	1	A
5	BICF2G630239243	73477770	A	0	0	G
5	BICF2P969806	73496959	0	0	0	G
5	BICF2G630239241	73512474	G	0	0	A
5	BICF2P685871	73520124	A	1	1	G
5	BICF2P685870	73520141	G	1	1	A
5	BICF2S23727888	73545188	G	0	0	A
5	BICF2G630239232	73555562	G	0	0	A
5	BICF2G630239231	73572918	A	0	0	G
5	BICF2G630239225	73584392	G	0	0	A
5	BICF2G630239218	73608789	G	0	0	A
5	BICF2G630239215	73617054	A	0	0	G
5	BICF2P1100409	73623624	0	0	0	G
5	BICF2P172684	73642713	0	0	0	C
5	BICF2S2331542	73660477	C	0	0	A

5	TIGRP2P74140_rs8940327	73673086	0	0	0	A
5	BICF2G630239201	73696067	A	0	0	G
5	BICF2G630239192	73707438	A	0	0	G
5	BICF2P880179	73719510	0	0	0	A
5	BICF2G630239175	73720117	C	0	0	A
5	BICF2G630239170	73736736	0	0	0	G
5	BICF2G630239169	73743668	G	0	0	A
5	BICF2G630239157	73749918	C	0	0	A
5	BICF2G630239145	73761606	C	1	1	A
5	BICF2G630239124	73782818	G	0	0	A
5	BICF2G630239122	73786069	A	0	0	G
5	BICF2G630239120	73798386	A	1	1	G
5	BICF2G630239116	73817830	A	0	0	G
5	BICF2G630239114	73828779	G	0	0	A
5	BICF2P1038631	73839116	G	0	0	A
5	BICF2G630239106	73861873	C	1	1	A
5	BICF2G630239100	73867142	T	0.75	0.8571	A
5	BICF2P1361919	73882633	G	0	0	A
5	BICF2G630239093	73882804	G	0.5	0.6429	C
5	BICF2G630239085	73892013	A	0.75	0.8571	G
5	TIGRP2P74204_rs9151137	73899337	0	0	0	G
5	BICF2G630239073	73914842	G	0	0	A
5	BICF2G630239059	73926046	A	1	1	C
5	BICF2G630239031	73941222	G	0	0	A
5	BICF2S23642308	73954398	A	0	0	G
5	BICF2G630239009	73979320	A	0	0	C
5	BICF2G630239004	73989238	A	0	0	G
5	BICF2P1422777	73990917	G	0	0	A
5	BICF2G630238999	73991514	G	0	0	C
5	TIGRP2P74233_rs9050710	74012803	A	0.25	0.2143	G
5	BICF2P430577	74021212	G	0.25	0.2143	A
5	BICF2S23634538	74028983	C	0.25	0.2143	A
5	BICF2P52155	74037517	G	0.75	0.8333	A
5	BICF2P17924	74050411	A	0.75	0.7857	G
5	BICF2P1236223	74062405	A	1	1	G
5	BICF2P934785	74081558	A	0	0	G
5	BICF2P945138	74085459	C	0	0	A
5	BICF2P474755	74095897	G	0.75	0.7857	A
5	TIGRP2P74263_rs9081839	74122091	G	0	0	A
5	BICF2S23618060	74134132	0	0	0	G
5	BICF2P394406	74144876	A	0	0	G
5	BICF2G630238956	74151225	A	0.75	0.7857	G
5	BICF2G630238943	74166152	G	0.25	0.2143	A
5	TIGRP2P74273_rs9137653	74175266	G	0	0	A
5	BICF2G630238932	74187367	G	0	0	A
5	TIGRP2P74282_rs8944571	74197030	G	0	0	A
5	BICF2P907423	74198240	C	0	0	A

5	BICF2S22918871	74222524	A	0	0	G
5	BICF2G630238907	74253793	A	0	0	G
5	BICF2G630238904	74257324	G	0	0	A
5	BICF2G630238903	74257564	A	0	0	G
5	BICF2P1040804	74278018	A	0	0	C
5	BICF2G630238893	74280046	A	0	0	G
5	BICF2P907696	74291117	A	0	0	G
5	BICF2P424965	74302445	G	0	0	A
5	BICF2P1314195	74303153	G	0	0	A
5	BICF2G630238869	74322869	A	0.25	0.2143	G
5	BICF2G630238863	74331932	0	0	0	G
5	BICF2P541650	74341418	G	0	0	A
5	BICF2G630238849	74347743	A	0	0	G
5	BICF2G630238818	74371706	G	0	0	A
5	BICF2G630238810	74384152	A	0	0	G
5	BICF2G630238803	74398391	A	0.25	0.2143	G
5	BICF2G630238791	74417641	A	0	0	G
5	BICF2G630238787	74432558	0	0	0	G
5	BICF2P807606	74449516	C	0	0	A
5	BICF2G630238771	74471873	A	1	1	G
5	BICF2G630238769	74477201	A	0.75	0.7857	G
5	BICF2G630238764	74488605	A	0	0	G
5	TIGRP2P74329_rs9067706	74502446	A	0.75	0.7857	G
5	BICF2G630238755	74510476	A	0.25	0.2143	G
5	BICF2P708536	74526115	G	0	0	A
5	BICF2G630238736	74531521	G	0	0	A
5	BICF2G630238720	74545250	G	0	0	A
5	BICF2G630238705	74557072	A	0	0	G
5	BICF2G630238703	74567205	A	1	1	G
5	BICF2G630238691	74577509	A	0.75	0.7857	G
5	BICF2G630238648	74597740	G	0	0	A
5	BICF2G630238644	74607138	A	0.75	0.7857	G
5	BICF2G630238638	74612746	A	0.75	0.7857	C
5	BICF2G630238618	74623532	A	0	0	G
5	BICF2G630238593	74635311	A	0.75	0.7857	G
5	BICF2G630238587	74647592	G	0.25	0.2143	A
5	BICF2G630238579	74662492	A	0	0	G
5	BICF2P498356	74685678	G	0.25	0.2143	A
5	BICF2P230747	74693521	G	0.25	0.2143	A
5	BICF2G630238557	74704218	A	0	0	T
5	BICF2G630238553	74707142	G	0.25	0.2143	A
5	BICF2G630238552	74707198	C	0.25	0.2143	A
5	BICF2S23617478	74713345	G	0	0	C
5	BICF2G630238542	74725840	C	0	0	G
5	BICF2S23448033	74730557	G	0.75	0.7857	A
5	BICF2G630238537	74743313	G	0.75	0.7857	A
5	BICF2G630238533	74752237	A	0.25	0.2143	C

5	TIGRP2P74354_rs8652504	74762147	0	0	0	G
5	BICF2S23241256	74776683	A	0	0	G
5	BICF2G630238455	74790147	A	0.75	0.7857	G
5	BICF2P810273	74797420	A	0.75	0.7857	G
5	BICF2G630238449	74798688	C	0.75	0.7857	A
5	BICF2G630238424	74802542	A	0.75	0.7857	G
5	BICF2S2412625	74810286	G	0	0	A
5	TIGRP2P74359_rs8568385	74826769	G	0.25	0.2143	A
5	BICF2G630238417	74841669	G	0.25	0.2143	A
5	BICF2G630238416	74842073	A	0.25	0.2143	G
5	BICF2G630238395	74849595	A	0	0	G
5	BICF2G630238378	74862839	A	1	1	G
5	BICF2G630238371	74871350	G	0.75	0.8333	A
5	BICF2P466503	74880234	G	0	0	A
5	BICF2G630238333	74895820	G	0	0	A
5	BICF2G630238323	74906585	A	0	0	G
5	BICF2P533384	74911480	C	0.75	0.7857	A
5	BICF2P334255	74931343	A	0.75	0.7857	G
5	BICF2P972643	74938052	A	0.75	0.7857	G
5	BICF2S23118963	74950648	A	0	0	G
5	BICF2P1061386	74976017	A	0.75	0.7857	G
5	BICF2P1061989	74983294	G	1	1	A
5	TIGRP2P74383_rs9036670	74991854	A	0	0	G
5	TIGRP2P74385_rs9052551	75006259	C	0	0	A
5	BICF2G630238241	75017261	G	0	0	A
5	BICF2P1336749	75026759	A	0	0	G
5	BICF2G630238178	75048923	C	0.75	0.7857	A
5	BICF2G630238167	75057564	A	1	1	G
5	BICF2G630238162	75062569	A	1	1	G
5	BICF2G630238138	75076210	A	0	0	G
5	BICF2G630238119	75084852	A	0	0	G
5	BICF2G630238112	75095227	A	0	0	G
5	BICF2P235792	75113818	G	0.25	0.2143	A
5	TIGRP2P74426_rs8662754	75125293	0	0	0	A
5	BICF2P168192	75133399	G	0	0	C
5	BICF2S23723329	75147304	A	0	0	G
5	BICF2S2322813	75155106	G	0	0	A
5	BICF2S23641804	75171263	A	0	0	C
5	BICF2P1471313	75182064	A	1	1	G
5	BICF2S2375260	75189050	G	1	1	A
5	BICF2S23235758	75205631	A	0	0	G
5	BICF2S23649042	75211035	A	0	0	G
5	BICF2P525566	75226029	A	0.25	0.2143	C
5	TIGRP2P74428_rs8862114	75235684	A	1	1	T
5	BICF2P1087441	75250969	C	0	0	G
5	BICF2P1170982	75258257	G	0.25	0.2143	A
5	BICF2S23010721	75275821	A	0.25	0.2143	G

5	BICF2S23650410	75289255	A	0	0	G
5	BICF2P764662	75300743	A	0.25	0.2143	G
5	BICF2P1311306	75305676	G	0.25	0.2143	A
5	BICF2P1103586	75319892	C	0	0	A
5	BICF2P366638	75331171	A	0	0	G
5	BICF2P83744	75342986	C	0.75	0.7857	A
5	BICF2P1100611	75354488	G	0.25	0.2143	A
5	BICF2P321788	75359006	G	0	0	A
5	BICF2S23330460	75369784	G	0.75	0.7857	A
5	BICF2S23624819	75380411	G	0	0	A
5	BICF2P961059	75382681	A	0	0	C
5	BICF2P1384661	75394990	A	0	0	G
5	BICF2P1432168	75406902	G	0.25	0.2143	A
5	BICF2S23115128	75425177	G	0.25	0.2143	A
5	BICF2P526616	75429504	A	0	0	G
5	BICF2P526618	75429890	G	0.75	0.7857	A
5	BICF2P595275	75441596	C	0.75	0.7857	A
5	BICF2P781060	75452643	A	0.75	0.7857	G
5	BICF2P675053	75473554	A	0	0	G
5	BICF2P980661	75476911	A	0	0	G
5	BICF2S23334898	75499890	A	0	0	G
5	BICF2P1262096	75510140	A	0.75	0.7857	G
5	BICF2P1215689	75521854	0	0	0	G
5	TIGRP2P74434_rs8780145	75534408	0	0	0	G
5	BICF2P192896	75552241	A	0.25	0.2143	G
5	BICF2P1108131	75565993	G	0	0	A
5	BICF2S23148563	75568887	C	0	0	G
5	BICF2P983531	75588056	0	0	0	A
5	BICF2S23318910	75605465	G	0.25	0.2143	A
5	BICF2P606314	75616507	0	0	0	C
5	BICF2P1326037	75629294	A	0	0	G
5	BICF2P596370	75638383	A	0.75	0.7857	G
5	BICF2P1304755	75648422	A	0.25	0.2143	C
5	BICF2S23648174	75664243	A	0.25	0.2143	G
5	BICF2P277083	75681334	A	0.75	0.7857	G
5	BICF2P744747	75691532	A	0	0	G
5	BICF2S23024731	75701449	A	0	0	G
5	BICF2S23029870	75705312	G	0.75	0.7857	A
5	BICF2P791785	75710339	A	0	0	G
5	BICF2P791787	75710485	A	0.25	0.2143	G
5	BICF2P259433	75722315	A	1	1	G
5	BICF2S2356258	75737267	A	0	0	G
5	BICF2P1237609	75743481	G	0	0	A
5	BICF2P1034274	75749754	A	0.25	0.2143	G
5	BICF2S23641888	75760468	A	0	0	G
5	BICF2P253481	75769665	A	0	0	G
5	BICF2P269251	75774586	0	0	0	A

5	BICF2S22954357	75787288	G	0	0	A
5	BICF2P451975	75800035	G	0	0	A
5	BICF2S23449139	75810700	G	1	1	A
5	BICF2S2371215	75819791	G	1	1	A
5	BICF2P1295312	75833527	A	0.75	0.7857	G
5	BICF2S23154811	75843878	A	1	1	G
5	BICF2P76256	75855667	A	0.25	0.2143	G
5	BICF2P356847	75868081	A	0	0	G
5	BICF2P1085939	75882052	G	0	0	A
5	BICF2P279143	75897610	G	0.25	0.2143	A
5	BICF2P256380	75916767	T	1	1	A
5	BICF2S22943145	75927690	A	0.25	0.2143	C
5	BICF2P294742	75935413	G	0.75	0.7143	A
5	BICF2S23626509	75948857	C	0.75	0.7857	A
5	BICF2S22939223	75952401	A	0.75	0.7857	C
5	BICF2S2335234	75959851	A	0	0	G
5	BICF2S23710745	75973965	A	0	0	C
5	TIGRP2P74441_rs8614804	75987166	0	0	0	G
5	BICF2S2374753	76000298	A	0	0	G
5	BICF2P11341	76013968	G	0	0	A
5	BICF2P479316	76016288	C	0.25	0.2143	A
5	BICF2S23721520	76052349	G	0.5	0.5	A
5	BICF2S23717194	76073934	A	0.5	0.5	C
5	BICF2S23610187	76095548	A	0	0	G
5	BICF2P277454	76116890	0	0	0	A
5	BICF2P256226	76124458	C	0.75	0.7143	A
5	BICF2S23526474	76129534	A	0	0	G
5	BICF2P236475	76134972	0	0	0	C
5	BICF2P305708	76143427	A	0	0	G
5	G355f56S136	76169133	A	1	1	G
5	BICF2S23538757	76193820	A	0.75	0.7857	G
5	BICF2P1425833	76201263	C	0	0	A
5	BICF2P1104560	76212122	G	0	0	A
5	BICF2S23551041	76222312	A	0.5	0.5	G
5	BICF2P789098	76239372	A	0	0	G
5	BICF2P1274553	76248560	A	0.5	0.5	C
5	BICF2P670653	76260181	0	0	0	A
5	BICF2P453940	76278140	A	0.5	0.5	G
5	BICF2S23658742	76278648	A	0	0	G
5	BICF2S23657572	76283036	A	0.5	0.5	C
5	BICF2P551628	76297870	T	0	0	A
5	BICF2S22950408	76308391	C	0	0	A
5	BICF2P1145385	76312633	G	0	0	A
5	BICF2P597433	76321734	A	0.25	0.2857	G
5	BICF2P140966	76346994	G	0	0	A
5	BICF2P517559	76357037	G	0.25	0.2143	A
5	BICF2P520274	76364073	A	1	1	C

5	BICF2P948849	76380142	G	0.25	0.2143	C
5	BICF2P1002496	76386896	C	0.25	0.2143	A
5	BICF2S23613445	76395077	A	0.75	0.7857	C
5	BICF2S23633793	76407885	G	0.25	0.2143	A
5	BICF2P433434	76424969	G	0.25	0.2143	A
5	BICF2P278121	76430571	A	0.75	0.7857	G
5	BICF2S23010712	76448180	A	0.5	0.5	G
5	BICF2S2352278	76456161	G	0.5	0.5	A
5	BICF2P105586	76472161	G	0.25	0.2143	A
5	BICF2S2328460	76476332	G	0.5	0.5	A
5	BICF2S23614965	76492076	A	0.25	0.2143	G
5	BICF2P775279	76501162	A	0.25	0.2143	G
5	BICF2S23735344	76506078	A	0	0	G
5	BICF2P587644	76509555	A	0.25	0.2857	G
5	BICF2P1373154	76523940	A	0	0	G
5	BICF2S22960737	76535768	C	0	0	G
5	BICF2P882089	76553509	A	0	0	G
5	BICF2P40483	76558928	G	0.25	0.2143	A
5	BICF2P877243	76576065	G	0.25	0.2857	A
5	BICF2P575652	76578482	A	0.75	0.7857	G
5	BICF2P321044	76584246	C	0.25	0.2857	G
5	BICF2G630238042	76597554	T	0.75	0.7857	A
5	BICF2G630238037	76599382	A	0.25	0.25	G
5	BICF2G630238027	76607923	G	0.25	0.2857	A
5	BICF2G630238013	76615243	C	0.25	0.2857	A
5	BICF2G630238009	76624812	G	0.75	0.7857	A
5	BICF2G630237990	76643928	G	0.25	0.2857	A
5	BICF2P604278	76652568	A	0	0	C
5	BICF2G630237965	76662168	C	0.25	0.2143	G
5	BICF2P907158	76672593	A	0.25	0.2143	G
5	BICF2G630237959	76687692	A	0	0	T
5	BICF2P910925	76729805	0	0	0	A
5	BICF2G630237944	76745647	A	0.25	0.2143	G
5	BICF2S2366938	76753487	A	0	0	G
5	BICF2G630237908	76774877	G	0.75	0.7857	A
5	BICF2P413559	76797078	C	0.75	0.7143	A
5	BICF2P1002252	76813633	G	0	0	A
5	BICF2G630237900	76820699	A	0.5	0.5	G
5	BICF2P435893	76842075	A	0.5	0.5	G
5	BICF2P358530	76858190	0	0	0	G
5	BICF2P521576	76872361	A	0.5	0.5	G
5	BICF2S23247925	76880262	G	0.5	0.5	A
5	BICF2P624631	76889611	0	0	0	C
5	BICF2P552783	76906234	A	0	0	G
5	BICF2P1015291	76916364	C	0.25	0.2143	A
5	BICF2S24318081	76924201	A	0.75	0.7857	T
5	BICF2P220782	76945349	A	0.25	0.2143	G

5	BICF2S23153978	76949783	G	0.25	0.2857	A
5	BICF2S23641931	76999500	C	0.25	0.2857	A
5	BICF2S2398991	77014360	A	0.25	0.2857	G
5	BICF2P1086565	77024037	C	0.75	0.7143	G
5	BICF2S23327736	77036472	A	0.75	0.7143	G
5	BICF2S23011045	77040668	A	0.75	0.7143	G
5	BICF2P200952	77052373	A	0.75	0.7143	G
5	TIGRP2P74483_rs9035285	77062337	0	0	0	C
5	BICF2S23437454	77080017	G	0.75	0.7143	A
5	BICF2S23650778	77100802	G	0.75	0.7143	A
5	BICF2P529114	77115858	A	0	0	G
5	BICF2P281411	77122164	A	0.25	0.2143	G
5	BICF2P410471	77136150	A	0	0	G
5	BICF2P1460559	77143819	A	0	0	G
5	BICF2S23319208	77161031	G	0.5	0.5	A
5	BICF2P379950	77172629	A	0	0	G
5	BICF2P1211863	77187434	A	0	0	T
5	BICF2S23646549	77189734	A	0.25	0.2143	G
5	BICF2P772267	77207882	G	0.5	0.5	A
5	BICF2S23425529	77216239	A	0.25	0.2143	G
5	BICF2S23120302	77239771	A	0	0	G
5	BICF2P344950	77254283	A	0.25	0.2143	G
5	BICF2P471058	77259372	A	0.5	0.5	G
5	BICF2P192262	77269994	A	0.5	0.5	G
5	BICF2P553400	77280014	A	0	0	G
5	BICF2P306631	77290407	G	0.25	0.2143	C
5	BICF2P1266312	77300255	G	0.25	0.2143	A
5	BICF2P979819	77306504	A	0.25	0.1667	G
5	BICF2P99215	77318448	A	0	0	G
5	BICF2P1188277	77337216	A	0.5	0.5	G
5	BICF2S23249428	77367397	G	0	0	A
5	BICF2S23718407	77380714	A	0.25	0.2857	G
5	BICF2P1221558	77385205	A	0.25	0.1667	G
5	BICF2P1076142	77400770	G	0.25	0.2143	A
5	BICF2P1303114	77417808	A	0.25	0.25	G
5	BICF2P261187	77420806	C	0.75	0.7143	A
5	BICF2P393228	77433333	G	0.25	0.2857	A
5	BICF2S23738370	77458766	A	0.5	0.5	G
5	BICF2S23237461	77470586	G	0	0	A
5	BICF2P112101	77477756	0	0	0	A
5	BICF2S2379709	77491913	0	0	0	G
5	BICF2P792026	77501182	A	0.75	0.7143	G
5	BICF2P457467	77517988	A	0.25	0.2143	G
5	BICF2S23723904	77522606	A	0.5	0.5	G
5	BICF2P295441	77538011	A	0.5	0.5	G
5	BICF2P1053844	77544612	A	0.25	0.2143	C
5	BICF2P1085616	77566277	A	0.5	0.5	C

5	BICF2S23727725	77572280	G	0	0	A
5	BICF2P217620	77584004	A	0.25	0.2143	G
5	BICF2P198477	77603086	A	0.25	0.2143	G
5	BICF2P1337735	77616550	G	0.5	0.4286	A
5	BICF2P492839	77627567	0	0	0	A
5	BICF2S23648659	77653897	0	0	0	G
5	BICF2S23221966	77677531	A	0.25	0.2	G
5	BICF2P341036	77686070	A	0.25	0.2143	G
5	BICF2P771581	77694674	G	0.5	0.5	A
5	BICF2S2346016	77716491	G	0.75	0.7857	A
5	BICF2S22949529	77725781	T	0	0	A
5	BICF2P1402953	77739188	0	0	0	A
5	BICF2S23714922	77745591	A	0.5	0.4286	G
5	BICF2P603658	77751621	A	0	0	G
5	BICF2P1224248	77763140	A	0.25	0.2857	G
5	BICF2P872640	77784887	G	0.5	0.5	A
5	BICF2P833448	77791225	A	0.25	0.2857	G
5	BICF2P833458	77792617	A	0.25	0.25	C
5	BICF2P1234092	77799176	A	0.25	0.2857	G
5	BICF2P364660	77812382	G	0.25	0.2143	A
5	BICF2P632907	77821333	A	0.5	0.5	G
5	BICF2P561672	77834560	G	0.5	0.5	A
5	BICF2P1190411	77850134	C	0.5	0.5	G
5	BICF2P1134467	77856712	A	0	0	C
5	TIGRP2P74510_rs8899522	77874593	0	0	0	A
5	BICF2P1210309	77880825	A	0	0	G
5	BICF2P1335787	77890774	G	0.5	0.5	A
5	BICF2P691586	77915931	A	0.25	0.2143	T
5	BICF2P351817	77941109	0	0	0	A
5	BICF2S23417919	77952599	A	0.5	0.5	G
5	BICF2S23512051	78003804	G	0.25	0.2857	A
5	BICF2P445970	78012253	0	0	0	A
5	BICF2S23343143	78020041	A	0.25	0.2857	G
5	BICF2P664157	78038863	C	0.75	0.7857	A
5	BICF2S23243433	78049590	A	0	0	G
5	BICF2P880002	78060575	C	0.25	0.2857	G
5	BICF2S23220618	78068436	A	0	0	G
5	BICF2S2365872	78081601	G	0.25	0.2143	A
5	BICF2P987661	78091794	G	0.25	0.2143	A
5	BICF2P1342129	78101262	G	0	0	A
5	BICF2P537153	78111890	G	0	0	A
5	BICF2P1169180	78135199	A	0.25	0.2143	G
5	BICF2P575848	78143484	A	0.25	0.2143	G
5	BICF2P757789	78164665	A	0.25	0.2143	G
5	BICF2S23542292	78173984	A	0.25	0.2143	G
5	BICF2S22939955	78178282	G	0.5	0.3333	A
5	BICF2P1185687	78203565	G	0.25	0.2143	A

5	BICF2P746082	78211465	C	0	0	A
5	BICF2P1172338	78222290	A	0.25	0.25	G
5	BICF2P70507	78227032	A	0	0	G
5	BICF2P1096776	78236619	A	0	0	G
5	TIGRP2P74520_rs8986773	78250106	0	0	0	G
5	BICF2P1406685	78258347	G	0	0	A
5	BICF2P478495	78270303	A	0	0	G
5	BICF2P157965	78294237	A	0.25	0.2143	G
5	BICF2P228357	78310223	T	0.25	0.2857	A
5	BICF2P168255	78326806	A	0	0	G
5	BICF2P1038269	78358223	C	0.25	0.2143	A
5	BICF2P695998	78361000	C	0.25	0.2143	A
5	BICF2P324659	78381705	C	0.25	0.2143	A
5	BICF2P994453	78387847	A	0	0	C
5	BICF2P1099772	78416196	A	0	0	G
5	BICF2P255110	78425016	A	0	0	G
5	BICF2P542950	78437368	G	0.25	0.2143	A
5	BICF2P770917	78452288	A	0	0	G
5	BICF2P265273	78453978	A	0	0	G
5	BICF2P1458168	78466356	A	0.25	0.2143	G
5	BICF2P757106	78479172	G	0.25	0.2143	A
5	BICF2P573955	78497024	G	0.25	0.2143	A
5	BICF2P1393995	78505484	0	0	0	A
5	BICF2P1055428	78518547	G	0.5	0.4286	A
5	BICF2P884698	78525890	G	0	0	A
5	BICF2P903598	78534982	A	0	0	G
5	BICF2P1222477	78546290	C	0	0	A
5	BICF2P651457	78561129	A	0	0	G
5	BICF2S23411861	78573283	A	0.25	0.2857	G
5	BICF2P1233816	78584180	G	0.25	0.2857	C
5	BICF2P884310	78593664	A	0.25	0.2857	G
5	BICF2P1060811	78605802	G	1	0.7143	A
5	BICF2P345035	78615242	A	0.75	0.7143	G
5	BICF2P1276357	78617952	A	0	0	G
5	BICF2P406829	78626208	A	0.25	0.2857	G
5	BICF2P110015	78644496	A	0.25	0.2857	G
5	BICF2P650718	78654574	A	0.25	0.2857	C
5	BICF2P596822	78668781	A	0.75	0.7143	G
5	BICF2P594711	78680880	G	0.25	0.2857	A
5	BICF2P1230152	78687243	G	0.75	0.7143	A
5	BICF2P453140	78702445	G	0	0	A
5	BICF2P1146125	78708631	A	0	0	G
5	BICF2P445715	78725207	A	0	0	G
5	BICF2P658211	78729305	G	0	0	A
5	BICF2S23254812	78743384	A	0	0	G
5	BICF2S22957436	78758672	A	0	0	G
5	BICF2P1333692	78769488	G	0.5	0.5714	A

5	BICF2P396145	78780788	A	0	0	C
5	BICF2P276902	78787304	0	0	0	A
5	BICF2P97761	78806696	0	0	0	A
5	BICF2P404578	78814915	G	0	0	C
5	BICF2P216384	78825339	A	0.5	0.5714	G
5	BICF2P1262776	78839054	C	0	0	G
5	BICF2P1374060	78847455	A	0	0	G
5	BICF2P268447	78862849	A	0	0	G
5	BICF2P67693	78867341	G	0	0	A
5	BICF2P1438212	78889949	A	0.5	0.5714	C
5	BICF2S23438427	78890745	A	0	0	T
5	BICF2S23521542	78904012	A	0	0	G
5	BICF2P488596	78915627	A	0.5	0.5714	G
5	BICF2P1439891	78928004	A	0	0	G
5	BICF2P838279	78966981	G	0	0	A
5	BICF2P563345	78971552	G	1	1	A
5	BICF2P1263867	78984517	A	0	0	G
5	BICF2P370990	78998011	A	0	0	T
5	BICF2P523308	79008866	C	0	0	A
5	BICF2P1106483	79024717	A	0	0	C
5	BICF2P223449	79028905	C	0.5	0.5714	A
5	BICF2P120791	79047969	C	0	0	A
5	BICF2P558384	79058634	G	0	0	A
5	BICF2P703501	79079463	A	0	0	G
5	BICF2P1187669	79090831	A	0.5	0.4286	C
5	BICF2P801215	79109322	G	0	0	A
5	BICF2P771372	79142536	G	0	0	A
5	BICF2S23534026	79150374	0	0	0	G
5	BICF2P553061	79165613	A	0	0	C
5	BICF2P676718	79176194	G	0	0	A
5	BICF2P1186166	79191510	C	0	0	G
5	TIGRP2P74548_rs9174853	79226313	0	0	0	G
5	BICF2S23422517	79237910	A	0.5	0.4286	G
5	BICF2S23623158	79269277	A	0.5	0.4286	G
5	BICF2S2366695	79271446	0	0	0	A
5	BICF2P1142258	79283549	G	0.75	0.7857	A
5	BICF2S2339101	79297457	A	0.25	0	C
5	BICF2P904193	79305089	0	0	0	C
5	BICF2P660308	79320498	C	0.25	0.2143	A
5	TIGRP2P74566_rs8798601	79343581	0	0	0	C
5	BICF2P1006569	79382670	0	0	0	A
5	BICF2P479520	79391075	0	0	0	A
5	BICF2S23243790	79407035	G	0.5	0.5714	A
5	BICF2S2359345	79407580	A	0.5	0.5714	C
5	BICF2S23255827	79442427	A	0.5	0.5714	G
5	BICF2P287049	79462093	0	0	0	C
5	TIGRP2P74573_rs8708481	79478150	0	0	0	G

5	BICF2P934033	79492867	0	0	0	G
5	BICF2P309765	79517937	0	0	0	C
5	BICF2S2297212	79522993	A	0	0	G
5	BICF2P1064351	79545777	0	0	0	A
5	BICF2S23761304	79560308	A	0	0	G
5	BICF2P894439	79577467	0	0	0	G
5	BICF2P1067313	79584926	G	0	0	A
5	BICF2P957527	79601154	G	0	0	C
5	BICF2P93284	79612824	A	0	0	G
5	BICF2S23122830	79638040	A	0.5	0.5	G
5	BICF2S23225232	79654073	A	0.75	0.75	G
5	BICF2P641629	79668045	A	0	0	G
5	BICF2P543724	79675876	0	0	0	A
5	BICF2P889549	79687526	0	0	0	C
5	BICF2P983566	79702618	G	0.5	0.5	A
5	BICF2P491431	79708771	A	0	0.2143	G
5	BICF2P691133	79726286	A	0.5	0.5	G
5	BICF2P1280107	79733517	A	0.25	0.2143	G
5	BICF2S23639629	79748306	C	0.25	0.2143	G
5	G356f27S24	79781530	0	0	0	C
5	BICF2P1243400	79804595	A	0.5	0.5	G
5	BICF2P423013	79831289	A	0.5	0.5	G
5	BICF2P643533	79836110	0	0	0	G
5	BICF2S2452507	79864080	0	0	0	G
5	BICF2P684984	79873010	0	0	0	C
5	TIGRP2P74577_rs9113068	79890647	0	0	0	A
5	BICF2S2357479	79911628	G	0	0	A
5	BICF2S23732474	79936072	G	0	0	A
5	BICF2P931049	79955455	0	0	0	A
5	TIGRP2P74578_rs8638767	79970037	0	0	0	G
5	BICF2P184734	79972220	0	0	0	C
5	BICF2P228970	80004238	0	0	0	A
5	BICF2P373634	80027563	A	0	0	G
5	BICF2P1022237	80037053	A	0	0	G
5	BICF2S23427148	80074325	G	0	0	C
5	TIGRP2P74581_rs8603211	80081504	0	0	0	C
5	TIGRP2P74583_rs8569418	80086494	G	0	0	A
5	TIGRP2P74585_rs8882658	80109196	0	0	0	G
5	BICF2P557163	80131029	0	0	0	G
5	BICF2P1041551	80138808	0	0	0	C
5	BICF2P1295263	80150843	0	0	0	G
5	BICF2P1418265	80161222	0	0	0	A
5	BICF2P1142629	80190234	A	0	0	G
5	TIGRP2P74586_rs9121579	80205366	0	0	0	A
5	BICF2P674952	80213154	G	0	0	A
5	BICF2P1329549	80229734	G	0	0	A
5	BICF2P1173815	80251273	0	0	0	C

5	TIGRP2P74588_rs8793470	80261941	0	0	0	C
5	BICF2P771488	80279639	0	0	0	C
5	BICF2P1168127	80292941	G	0	0	C
5	BICF2P597576	80308664	G	0	0	A
5	BICF2P1388620	80322665	G	0	0	A
5	BICF2S23059016	80338819	G	0	0	A
5	BICF2S23059190	80343008	G	0	0	A
5	BICF2S23644334	80377425	G	0.25	0.2857	A
5	BICF2P22256	80386291	T	0	0	A
5	BICF2P1384796	80398693	A	0.25	0.25	G
5	BICF2P482369	80416250	A	0	0	G
5	TIGRP2P74597_rs8674102	80427301	0	0	0	G
5	BICF2P1374371	80432278	G	0.75	0.7143	C
5	BICF2S2415626	80438846	0	0	0	G
5	BICF2P999654	80451336	G	0.75	0.7143	A
5	BICF2P1457235	80457839	A	0.25	0.2857	G
5	BICF2P1212730	80497043	A	0.25	0.25	C
5	BICF2S23043880	80503581	C	0.25	0.2857	G
5	BICF2P171484	80511891	G	0.5	0.5	A
5	BICF2P651953	80525807	G	0.75	0.7143	A
5	BICF2P766758	80537541	A	0.75	0.7143	G
5	G357f51S288	80565233	0	0	0	G
5	BICF2P1331418	80596842	A	0.25	0.2857	C
5	TIGRP2P74601_rs8921964	80615765	0	0	0	G
5	TIGRP2P74602_rs9098555	80635758	0	0	0	A
5	BICF2S2374384	80654029	A	0	0	C
5	BICF2P1001866	80666429	A	0.25	0.2857	C
5	BICF2P643357	80685638	G	0.25	0.2857	A
5	TIGRP2P74603_rs9071261	80699403	0	NA	NA	A
5	BICF2P1186062	80707339	0	0	0	G
5	BICF2P414301	80725735	C	0	0.2857	A
5	BICF2P290286	80731014	G	0.25	0.2857	A
5	BICF2P1353070	80741678	A	0.25	0.2857	G
5	BICF2P412596	80761018	T	0.25	0.2857	A
5	TIGRP2P74607_rs8887358	80769182	0	0	0	G
5	G358f42S122	80784502	0	0	0	C
5	G358f45S20	80796344	G	0.25	0.2857	A
5	BICF2S23411270	80799200	A	0.25	0.25	G
5	BICF2P1227870	80816868	G	0.25	0.2857	A
5	BICF2S23713959	80829485	A	0	0	G
5	BICF2P112508	80835013	0	0	0	A
5	BICF2P1065576	80845504	C	0.5	0.5	A
5	BICF2P649182	80868030	0	0	0	A
5	BICF2P782334	80885805	0	0	0	C
5	BICF2P515274	80892751	0	0	0	G
5	BICF2S23338388	80913805	G	0.5	0.4286	A
5	BICF2S2316269	80920591	A	0.5	0.5714	G

5	BICF2P658100	80954632	0	0	0	A
5	TIGRP2P74609_rs8990510	80969909	0	0	0	G
5	BICF2P1043987	80998455	A	0.25	0.2857	C
5	BICF2P1078805	81008576	A	0	0	G
5	BICF2P682906	81036692	0	0	0	A
5	BICF2P844771	81042705	A	0.25	0.2857	G
5	BICF2P1319778	81083575	G	0	0	A
5	BICF2S23137799	81101445	A	0	0	G
5	BICF2P573137	81133200	A	0	0	C
5	BICF2P515440	81151478	0	0	0	A
5	BICF2S22954464	81196360	G	0	0.125	A
5	BICF2S23258500	81237331	G	0	0.1667	A
5	BICF2S23340614	81252457	G	0.25	0.1429	A
5	BICF2S2336667	81295441	G	0	0.08333	A
5	BICF2S232289	81308624	A	0	0.1429	G
5	BICF2P477114	81323649	A	0.5	0.5	G
5	BICF2S22920880	81365059	G	0	0.2143	A
5	BICF2P572798	81403177	0	0	0	A
5	BICF2S23157170	81427721	C	0.25	0.2143	A
5	BICF2P936230	81481856	A	0	0	G
5	BICF2S23144004	81493523	G	0.5	0.5	A
5	BICF2P99964	81505841	G	0.75	0.7857	A
5	BICF2P1121933	81530370	A	0.75	0.7857	C
5	BICF2P156868	81537949	A	0	0	G
5	BICF2P10853	81547115	G	0.75	0.7143	A
5	BICF2P220299	81566234	A	0.75	0.7143	G
5	BICF2P1022362	81569748	A	0.5	0.5	G
5	BICF2S23214821	81572466	G	0	0	A
5	BICF2P850374	81583586	A	0.25	0.2857	C
5	BICF2P1010737	81600470	0	0	0	C
5	BICF2P685647	81605733	A	0.25	0.2857	G
5	BICF2P698040	81617081	0	0	0	C
5	BICF2P1226027	81633676	A	0	0	G
5	BICF2P1089022	81648941	0	0	0	G
5	BICF2S23319572	81651188	A	0.5	0.5	G
5	BICF2S22936793	81670223	G	0	0	A
5	BICF2S23431121	81681395	A	0	0	C
5	BICF2P258856	81690362	0	0	0	A
5	BICF2P1359501	81697025	A	0	0	G
5	BICF2P740997	81710105	A	0.25	0.2143	C
5	BICF2P1264748	81724690	A	0.5	0.4286	G
5	BICF2P501959	81740735	G	0	0	A
5	BICF2P487199	81750844	T	0.5	0.4286	A
5	BICF2P1096337	81762550	A	0	0	G
5	BICF2P798185	81768370	G	0.5	0.4286	A
5	BICF2P1159106	81776593	C	0.5	0.4286	A
5	BICF2P994428	81789360	A	0	0	G

5	BICF2P451934	81803047	G	0	0	A
5	BICF2S23753903	81808377	A	0	0	G
5	BICF2P1391754	81816418	A	0.25	0.2857	G
5	BICF2S23521397	81825996	G	0.25	0.2143	A
5	BICF2S23649005	81836688	A	0.25	0.2143	G
5	BICF2P639345	81852391	G	0.25	0.2143	A
5	BICF2P318446	81863636	A	0	0	G
5	BICF2P139695	81869371	G	0	0	A
5	BICF2P1431	81885244	A	0	0	G
5	BICF2P856483	81892362	A	0	0	G
5	BICF2P526058	81916847	G	0	0	A
5	BICF2P570236	81927096	A	0	0	G
5	BICF2P1324156	81940163	C	0.5	0.5714	A
5	BICF2P989928	81950340	A	0	0	G
5	BICF2P1037224	81970332	C	0.25	0.2143	A
5	BICF2P1007603	81978565	A	0.25	0.2143	G
5	BICF2P213461	81991205	A	0.5	0.5714	G
5	BICF2P963805	82000595	A	0.25	0.2143	C
5	BICF2P284945	82012036	A	0.25	0.2143	G
5	TIGRP2P74637_rs9220749	82025816	A	0.5	0.4286	T
5	TIGRP2P74638_rs8740799	82033585	A	0	0	G
5	BICF2P479033	82065461	0	0	0	C
5	BICF2S23026966	82089777	A	0.25	0.2857	C
5	BICF2P949209	82109215	A	0.25	0.2857	G
5	BICF2P159065	82120219	A	0	0	G
5	BICF2P1179501	82130025	0	0	0	G
5	BICF2S23528772	82134285	A	0	0	G
5	BICF2P578406	82145629	0	0	0	C
5	BICF2P946635	82172312	G	0.5	0.5	A
5	BICF2P46320	82186830	G	0.5	0.5	A
5	BICF2P1366040	82194248	G	0.75	0.7857	A
5	BICF2S23261251	82208750	A	0.25	0.2857	C
5	BICF2S23238048	82215581	0	0	0	T
5	TIGRP2P74673_rs8884982	82228153	G	0.75	0.8333	A
5	BICF2P240034	82250181	A	0.25	0.3571	G
5	TIGRP2P74677_rs8938896	82263944	C	0	0	A
5	BICF2P611915	82280522	A	0.5	0.5	G
5	BICF2P154169	82283682	G	0.5	0.5	A
5	BICF2P200848	82295594	G	0.25	0.2857	A
5	BICF2S22942614	82314628	A	0.25	0.2857	G
5	BICF2P269011	82317477	A	0	0	G
5	BICF2P1128708	82333144	A	1	0.7143	C
5	BICF2P1284677	82345869	G	0.25	0.2857	A
5	TIGRP2P74703_rs8804659	82363583	A	0	0	G
5	BICF2S23114932	82378344	A	0.25	0.2143	G
5	BICF2P994097	82390923	A	0.5	0.5	G
5	BICF2S23251982	82402458	A	0.5	0.5	G

5	BICF2P924797	82411928	G	0.5	0.5	A
5	BICF2S23329805	82435953	G	0.5	0.5	A
5	BICF2S22947699	82449807	A	0.75	0.7857	G
5	BICF2P1210832	82471114	G	0	0	A
5	BICF2P1403159	82487962	A	0.5	0.5	G
5	BICF2S23447848	82497581	A	0.5	0.5	G
5	BICF2P1412928	82501061	G	0.5	0.5	A
5	BICF2P366887	82522698	G	0	0	A
5	BICF2P1400494	82541687	G	0.75	0.7857	A
5	BICF2S234323	82556817	C	0	0	G
5	TIGRP2P74751_rs8716448	82565945	A	0	0	G
5	BICF2P418463	82577052	G	0.5	0.5	A
5	TIGRP2P74761_rs8753236	82591905	A	0.5	0.5	C
5	BICF2P334344	82594089	A	0	0	G
5	BICF2P189452	82609110	G	0	0	A
5	BICF2S23018372	82618353	G	0	0	A
5	BICF2P740930	82641407	G	0.5	0.5	A
5	TIGRP2P74776_rs9030566	82654371	A	0.5	0.5	G
5	TIGRP2P74782_rs8840929	82671949	G	1	1	A
5	BICF2P232634	82678668	C	0.5	0.5	A
5	BICF2P417412	82692799	A	0.5	0.5	C
5	BICF2P1078185	82703732	A	0.5	0.5	G
5	BICF2S2411401	82716044	A	0.5	0.5	G
5	BICF2S23424207	82719922	C	0.5	0.5	A
5	BICF2P225088	82745908	G	0.5	0.5	A
5	TIGRP2P74809_rs9136292	82754957	G	0.5	0.5	A
5	TIGRP2P74819_rs8950173	82773493	0	0	0	A
5	BICF2P1255862	82799520	G	0	0	A
5	BICF2S23434868	82810047	0	0	0	A
5	BICF2S2325581	82831659	A	0	0	G
5	BICF2P888321	82845297	0	0	0	A
5	BICF2P1441874	82846817	0	0	0	G
5	BICF2S244469	82879867	0	0	0	A
5	BICF2S22945330	82885835	A	0	0	G
5	BICF2P243536	82894662	0	0	0	A
5	BICF2P360755	82956464	0	0	0	G
5	BICF2S23356966	82980875	C	1	0.7143	A
5	BICF2P826188	82989817	T	0	0	A
5	TIGRP2P74832_rs8839296	83006933	0	0	0	G
5	TIGRP2P74834_rs8614371	83014654	A	0.25	0.5	G
5	BICF2P1112465	83038015	G	0.25	0.5	A
5	BICF2P1088349	83044281	G	0	0	A
5	BICF2P581497	83104728	G	0	0	A
5	TIGRP2P74854_rs8641188	83111263	C	0	0	A
5	BICF2P1218176	83127144	G	1	0.7857	A
5	BICF2P146998	83142677	A	0	0	G
5	BICF2P1200967	83146366	G	0	0	A

5	BICF2S23736502	83161073	A	1	0.7143	C
5	BICF2P1364953	83175177	A	1	0.7143	C
5	TIGRP2P74868_rs9012988	83187601	A	0	0	G
5	BICF2P147006	83198499	G	1	0.7143	A
5	TIGRP2P74880_rs9068825	83212645	A	1	0.7143	G
5	BICF2P438252	83215283	C	1	0.7143	A
5	BICF2S23312881	83228334	G	1	0.7143	A
5	BICF2P516628	83244160	G	0.25	0.2143	A
5	BICF2P1284548	83250034	A	0.75	0.5	G
5	BICF2P648260	83260776	G	0.75	0.5	A
5	BICF2S23336730	83271972	A	0.75	0.5	T
5	BICF2P90887	83287730	A	0	0	G
5	TIGRP2P74901_rs9130859	83298723	G	0	0.2857	C
5	BICF2P539971	83335663	A	0	0	G
5	BICF2S23426085	83342648	G	0.25	0.2143	C
5	BICF2P1283151	83357091	T	0	0	A
5	BICF2S23753615	83405665	A	0	0	G
5	BICF2S23354018	83429534	A	0.75	0.7857	G
5	BICF2P1358059	83447231	A	0.25	0.2143	G
5	TIGRP2P74924_rs8520835	83463857	A	0.75	0.7857	G
5	TIGRP2P74928_rs8948895	83474155	A	0	0.2857	G
5	BICF2P1288493	83479553	G	0.25	0.5	A
5	BICF2P1415389	83501491	G	0	0	A
5	TIGRP2P74936_rs8947700	83506314	0	0	0	A
5	BICF2S23318836	83524154	A	0.75	0.5	G
5	BICF2P32514	83530026	G	0	0.25	A
5	BICF2S23057084	83533895	A	0.25	0.2143	G
5	TIGRP2P74956_rs8708324	83546006	A	0.25	0.2143	G
5	BICF2S23228838	83557630	C	0	0.2857	G
5	BICF2P690241	83571520	A	0.25	0.2143	G
5	G359f55S51	83608737	A	0	0	G
5	TIGRP2P74961_rs8791011	83637790	0	0	0	C
5	BICF2P1244948	83642656	0	0	0	A
5	BICF2S2354730	83661091	0	0	0	G
5	G360f30S181	83681168	C	0	0	A
5	BICF2P1168580	83702136	0	0	0	G
5	BICF2S24413382	83711809	G	0.75	0.5	A
5	BICF2S23759391	83725340	A	0.75	0.5	C
5	BICF2S23318828	83743215	G	0.75	0.5	A
5	TIGRP2P74966_rs8498524	83757616	C	0.5	0.5	A
5	TIGRP2P74970_rs8563461	83780084	0	0	0	G
5	BICF2S23312047	83798897	C	0	0	G
5	BICF2P900400	83802030	G	0.5	0.5	C
5	BICF2P860831	83819550	C	0.5	0.5	A
5	BICF2P462052	83844814	C	0.5	0.2143	G
5	TIGRP2P74985_rs8599207	83854941	A	0.5	0.2143	G
5	BICF2P1316090	83855663	C	0.5	0.2143	A

5	BICF2P1311763	83861310	G	0.5	0.2143	A
5	BICF2P1369962	83870485	G	0	0	A
5	BICF2P845317	83887686	A	0	0	G
5	TIGRP2P75014_rs8612167	83903527	G	0.25	0.2857	A
5	BICF2S23651669	83904880	A	0.25	0.2857	G
5	TIGRP2P75015_rs8683878	83920436	A	0.25	0.2857	C
5	TIGRP2P75027_rs8946823	83946125	G	0	0	A
5	BICF2P980227	83958867	A	0	0	G
5	BICF2P704255	83969117	A	0	0	G
5	BICF2P907969	83981525	C	0	0	A
5	TIGRP2P75054_rs8985383	83986492	A	0	0	T
5	BICF2P1144617	84002603	A	0.25	0.2857	C
5	TIGRP2P75056_rs8787542	84017831	A	0	0	G
5	BICF2S2301301	84052008	C	0	0.2857	G
5	TIGRP2P75060_rs8899330	84103388	G	0.25	0.2857	A
5	BICF2S23722882	84116335	A	0	0.2857	G
5	BICF2S23522094	84136154	A	1	0.75	G
5	BICF2S23548152	84145768	G	0	0.2857	A
5	BICF2P452304	84168297	A	1	0.7143	C
5	BICF2P1063473	84171944	A	1	0.7143	G
5	BICF2P70203	84193246	A	1	0.7143	G
5	BICF2P1429054	84234556	A	1	0.75	G
5	BICF2P150113	84245776	A	1	0.7143	G
5	BICF2P729936	84255303	0	0	0	A
5	BICF2P681165	84270220	G	0	0	A
5	BICF2S23540135	84272280	A	0	0.25	G
5	TIGRP2P75066_rs8651427	84290656	C	0	0	A
5	BICF2P134458	84300719	G	0.75	0.4286	A
5	BICF2P585143	84309727	T	0.25	0.2857	A
5	BICF2S23421573	84333873	G	0	0	A
5	BICF2P928831	84347972	A	0.75	0.4286	G
5	BICF2P1108439	84363341	0	0	0	G
5	TIGRP2P75069_rs8875210	84378171	T	0.5	0.2143	A
5	G361f30S240	84392108	G	0	0	A
5	BICF2S23451655	84501740	G	0.25	0.2143	A
5	BICF2S23043454	84504027	A	0.25	0.2143	G
5	TIGRP2P75080_rs8831344	84517548	A	0.25	0.2143	C
5	BICF2P567133	84529873	A	0.5	0.7857	G
5	BICF2P236106	84544752	G	0.5	0.7857	A
5	BICF2S22925174	84559629	A	0.25	0.3	G
5	TIGRP2P75086_rs8882140	84577831	G	0.5	0.7857	A
5	BICF2S23011586	84585399	A	0.25	0.5	G
5	BICF2P994925	84591084	A	0.25	0.5	G
5	BICF2P362347	84601225	G	0.5	0.7857	A
5	BICF2P1236811	84616992	G	0.25	0.5	A
5	BICF2P1236810	84617778	G	0.25	0.5	C
5	BICF2P998860	84646644	0	0	0	A

5	BICF2S23133300	84660246	A	0.25	0.2857	C
5	G362f40S256	84686563	G	0	0	A
5	BICF2P1416416	84720784	0	0	0	T
5	BICF2P1152013	84787093	0	0	0	G
5	BICF2P207053	84794368	0	0	0	G
5	TIGRP2P75096_rs8620505	84801545	A	0	0	C
5	BICF2P1136530	84813306	A	0.75	0.7143	T
5	BICF2P73743	84877466	G	0	0	A
5	BICF2S23530034	84911353	A	0	0	G
5	BICF2S23530033	84911469	C	0	0	A
5	BICF2P978080	84932441	0	0	0	A
5	TIGRP2P75104_rs9114068	84963204	0	0	0	G
5	BICF2P528065	84982784	0	0	0	C
5	BICF2P891553	84989278	0	0	0	G
5	BICF2P258299	85016316	0	0	0	G
5	BICF2P390438	85038436	0	0	0	A
5	BICF2S24412081	85050442	0	0	0	A
5	BICF2P1119161	85070084	0	0	0	A
5	BICF2S2459240	85083653	G	0.25	0.2857	A
5	TIGRP2P75111_rs8806681	85098297	0	0	0	G
5	BICF2P667463	85122962	C	0	0	G
5	BICF2S23739808	85126430	A	0	0.5	C
5	TIGRP2P75113_rs8883161	85149954	0	0	0	C
5	TIGRP2P75114_rs9092503	85168513	0	0	0	C
5	BICF2S233822	85179413	G	0.25	0.5	A
5	BICF2S23334044	85188767	A	0.25	0.5	G
5	TIGRP2P75115_rs9023680	85192871	0	0	0	C
5	BICF2S2348065	85213276	G	0	0.2857	A
5	BICF2S24415334	85215239	A	0.75	0.7143	C
5	TIGRP2P75118_rs8937987	85233671	0	0	0	A
5	BICF2P1391586	85246527	C	0	0	G
5	TIGRP2P75120_rs9048220	85256398	A	0.75	0.5	T
5	BICF2P1508550	85291594	A	0.75	0.5	G
5	BICF2P720051	85308161	G	1	0.7143	A
5	BICF2S23223785	85319943	A	0.5	0.5	G
5	BICF2P1055101	85331025	A	0.75	0.7143	G
5	BICF2P171630	85349946	A	0	0	T
5	BICF2P1327755	85359846	G	0.75	0.7143	A
5	BICF2S23614179	85366466	G	0.5	0.5	A
5	BICF2P4270	85378780	A	NA	0.375	T
5	BICF2P7341	85406339	G	0	0.2857	A
5	TIGRP2P75161_rs8912677	85411324	G	0	0.2857	A
5	BICF2S23349764	85428840	C	1	1	A
5	BICF2P479813	85433993	0	0	0	G
5	BICF2P497236	85452443	A	0.25	0.2143	G
5	TIGRP2P75176_rs8804408	85457175	A	0	0.2143	C
5	BICF2P603053	85468886	G	0.25	0.2143	A

5	BICF2P574012	85486627	G	0.25	0.2143	A
5	BICF2P707625	85495774	G	0.75	0.4286	C
5	BICF2P1039936	85509827	C	0	0.2143	A
5	BICF2P1344770	85516596	G	0.25	0.2143	A
5	BICF2P23488	85534135	G	0.75	0.4286	A
5	BICF2S23716600	85539925	G	0.75	0.4286	A
5	TIGRP2P75204_rs9201036	85552416	C	0.25	0.2143	A
5	BICF2S23133417	85569773	A	0.25	0.2143	G
5	TIGRP2P75210_rs9165148	85580803	G	0.25	0.2143	A
5	TIGRP2P75213_rs9214312	85583049	G	0.75	0.4286	A
5	BICF2P1240467	85610193	0	0	0	C
5	TIGRP2P75219_rs8773625	85663346	0	0	0	A
5	BICF2P736965	85703913	0	0	0	T
5	BICF2P479636	85723327	0	0	0	A
5	TIGRP2P75235_rs8884972	85738342	A	0.5	0.7857	C
5	BICF2P794461	85755354	0	0	0	G
5	TIGRP2P75238_rs8648077	85760256	0	0	0	G
5	BICF2S23344649	85786965	A	0.25	0.2143	G
5	BICF2P398747	85798179	A	0	0	G
5	BICF2P209928	85798703	A	0.5	0.5	G
5	BICF2P643681	85812955	A	0	0	G
5	BICF2P1322089	85817880	0	0	0	A
5	BICF2P193619	85846264	A	0	0	G
5	BICF2P458967	85868614	G	0.25	0.5	A
5	BICF2S23655067	85881846	A	0	0	C
5	BICF2P717934	85888969	G	0.25	0.2857	C
5	BICF2P305431	85919183	C	0.5	0.2143	A
5	BICF2P136820	85929216	0	0	0	A
5	TIGRP2P75257_rs9032815	85948030	A	0.75	0.5	G
5	BICF2P166317	85953882	0	0	0	A
5	BICF2P645065	85970780	G	0.5	0.1667	A
5	BICF2P1425161	85980313	G	0	0	A
5	BICF2P1085916	85994391	A	0.5	0.7857	G
5	BICF2P633960	85998059	G	0.5	0.7857	A
5	TIGRP2P75301_rs9046915	86013360	A	0	0.2857	G
5	BICF2P852719	86028929	A	0.5	0.5	G
5	BICF2S2295560	86031501	A	0.5	0.5	G
5	TIGRP2P75325_rs8949803	86045748	G	0.25	0.2857	A
5	TIGRP2P75327_rs9201589	86064186	A	0	0	C
5	BICF2S23541391	86069201	A	0.75	0.4286	G
5	BICF2S23763367	86110927	A	0.25	0.2143	G
5	BICF2P460301	86112011	0	0	0	C
5	BICF2S23137021	86124860	A	0	0	G
5	BICF2P42863	86146185	A	0.25	0.2857	G
5	BICF2P31552	86158895	A	0	0	C
5	TIGRP2P75397_rs9008342	86172029	C	0.5	0.5	A
5	TIGRP2P75408_rs8974317	86183942	G	0	0	A

5	BICF2S23742672	86192580	G	0	0	A
5	BICF2S23541350	86212701	A	0.25	0.5	G
5	BICF2P840560	86221390	G	0.5	0.5	A
5	BICF2P738173	86232998	0	0	0	A
5	BICF2S2392196	86242496	G	0.5	0.5	A
5	BICF2P1037210	86248084	A	0	0	G
5	BICF2P485005	86251177	C	1	1	A
5	BICF2P925936	86268558	G	1	1	A
5	BICF2S22946128	86277141	A	0	0	G
5	BICF2P489630	86283951	A	1	0.7143	C
5	BICF2S23148421	86284586	A	1	0.7143	G
5	BICF2P1305460	86307504	A	0	0	G
5	BICF2P1087177	86331535	A	0	0.2857	C
5	BICF2P258546	86351283	G	0.5	0.5	A
5	TIGRP2P75532_rs8516428	86368455	G	0.5	0.7143	A
5	BICF2P115041	86386676	A	0	0	G
5	TIGRP2P75567_rs8617189	86404625	G	0.5	0.2857	A
5	BICF2P1087566	86410759	A	0	0	G
5	BICF2S23143270	86420690	A	0.25	0.2857	G
5	BICF2S23656455	86424312	G	0.25	0.5	A
5	BICF2S2367907	86435708	G	0.25	0.2857	A
5	BICF2P483162	86441914	G	0.25	0.2143	A
5	TIGRP2P75599_rs9087741	86448262	A	0.25	0.4286	C
5	TIGRP2P75602_rs9096339	86450459	T	0.5	0.7143	A
5	BICF2P384915	86450846	G	0.5	0.7143	C
5	TIGRP2P75612_rs9146001	86462325	A	0.25	0.2143	G
5	BICF2P863234	86475139	A	0	0	G
5	BICF2P1156675	86495848	A	0	0	G
5	BICF2S23523955	86507967	A	0	0	G
5	TIGRP2P75650_rs8701340	86524326	G	0	0	A
5	TIGRP2P75660_rs8750031	86536219	A	0	0	G
5	BICF2P261920	86562417	G	0.5	0.5	A
5	BICF2P624118	86576991	A	0.5	0.2857	G
5	TIGRP2P75694_rs8595883	86591098	A	0.25	0.2143	G
5	BICF2P1061801	86595707	G	0.25	0.2143	A
5	BICF2P1458217	86602038	A	0.25	0.2143	G
5	BICF2P939474	86610635	G	0.25	0.2143	A
5	BICF2S23215977	86619155	A	0.25	0.2143	G
5	BICF2S23255587	86632065	G	0.75	0.5	A
5	BICF2P422753	86645984	G	0	0	A
5	TIGRP2P75735_rs9121017	86660306	G	0	0	C
5	BICF2P323747	86660476	A	0	0	C
5	BICF2S23653213	86671254	C	0	0	A
5	TIGRP2P75745_rs9145499	86675574	A	0	0	G
5	TIGRP2P75750_rs9137076	86691606	0	0	0	A
5	TIGRP2P75758_rs8645281	86699227	A	0.5	0.5	G
5	BICF2P1246439	86699305	A	0.5	0.5	G

5	BICF2P864300	86713228	A	0.5	0.5	T
5	TIGRP2P75766_rs8863139	86713339	A	0	0	C
5	BICF2P816538	86727703	A	0.5	0.5	G
5	BICF2P1387924	86737059	G	0.25	0.2143	A
5	BICF2P1387923	86737371	G	0.25	0.2143	A
5	BICF2P1415480	86753439	A	0	0	T
5	BICF2S2438527	86757007	A	0	0	T
5	BICF2P1123931	86776998	A	0.5	0.5	G
5	TIGRP2P75806_rs8952823	86779921	G	0.5	0.5	A
5	BICF2P1089267	86800985	C	0.5	0.2857	A
5	BICF2P417824	86819421	A	0.25	0.2143	G
5	BICF2P1290104	86827216	C	0.25	0.5	A
5	TIGRP2P75827_rs8745485	86829380	A	0.25	0.5	G
5	BICF2P1072073	86838640	G	0	0.2143	A
5	BICF2P783496	86858380	C	0.75	0.7857	G
5	BICF2P818669	86868598	C	0.75	0.7857	G
5	BICF2S23742584	86872045	C	0	0.2143	A
5	BICF2P1095202	86889699	A	0	0	G
5	BICF2P1274665	86900422	A	0	0	G
5	TIGRP2P75869_rs9032805	86907986	A	0.25	0.2143	G
5	TIGRP2P75877_rs8968176	86924742	A	0.75	0.7857	G
5	BICF2S23520343	86934701	G	0	0	A
5	BICF2P1116920	86954697	G	0	0	A
5	BICF2P343569	86963846	A	0	0	G
5	BICF2P1399181	86977946	C	0	0	A
5	TIGRP2P75902_rs8767464	86994177	A	0.75	0.7857	G
5	BICF2P89504	87000116	G	0	0	A
5	BICF2S23650081	87019597	A	0.25	0.2143	G
5	BICF2S23416687	87020973	A	0	0	G
5	BICF2P324794	87042175	C	0.25	0.2143	A
5	BICF2P354004	87050035	G	0	0	A
5	TIGRP2P75924_rs8778915	87060899	A	0	0	G
5	BICF2P647562	87069941	A	0.25	0.25	G
5	BICF2S23022328	87078778	G	0.75	0.7857	A
5	BICF2S23212617	87102163	A	0	0	G
5	BICF2S22918307	87122840	A	0	0	G
5	BICF2S23055285	87124780	G	0.75	0.5714	A
5	BICF2S23540792	87144379	A	0.25	0.4286	G
5	BICF2P522936	87150183	G	0.5	0.6	A
5	BICF2S2337403	87184328	G	0	0	A
5	BICF2P693020	87197023	A	0.25	0.4286	C
5	BICF2P537835	87206783	G	0.25	0.4286	A
5	BICF2S23040766	87220748	A	0	0	G
5	BICF2S23141092	87236953	A	0	0	G
5	BICF2P1037886	87247634	G	0	0	A
5	BICF2S23018396	87260069	G	0	0	A
5	BICF2P726333	87262702	A	0	0	C

5	BICF2S229500	87278670	G	0.75	0.5714	A
5	BICF2P784360	87292070	A	0.25	0.4286	C
5	BICF2P68576	87308901	A	1	1	G
5	BICF2S23261228	87340681	A	0	0	G
5	TIGRP2P75939_rs8744537	87343904	0	0	0	G
5	BICF2P957315	87359213	G	0.25	0.4286	A
5	BICF2S2353613	87377340	A	0	0	G
5	BICF2S2296814	87391274	A	0	0	G
5	BICF2P501577	87402296	G	0.75	0.5714	A
5	BICF2S23627302	87418607	A	0	0	T
5	BICF2S23626438	87423344	A	0.25	0.4286	G
5	BICF2P556328	87437551	A	0	0	T
5	BICF2S23416041	87480088	G	0	0.2143	A
5	BICF2S23422111	87495103	G	0	0.1667	A
5	BICF2S23659598	87512139	G	0	0	A
5	BICF2S23658946	87516903	A	0	0	C
5	BICF2P227682	87536170	A	0.25	0.2143	G
5	BICF2P1278635	87543512	A	0.75	0.5714	G
5	TIGRP2P75941_rs8964137	87551918	0	0	0	A
5	BICF2S2457188	87562095	0	0	0	A
5	BICF2P822638	87581787	C	0	0	A
5	BICF2P280791	87584091	A	0	0	C
5	BICF2P600816	87598870	0	0	0	A
5	BICF2S23147066	87613340	A	0	0	G
5	BICF2S23034491	87632274	G	0.5	0.2857	A
5	BICF2P1333910	87644802	G	0.25	0.2143	A
5	BICF2P251004	87654938	G	0.75	0.5833	A
5	BICF2P1242649	87664661	A	0.5	0.5	C
5	BICF2S23549124	87677582	G	0.5	0.5833	A
5	BICF2P1276772	87688322	A	0	0	T
5	BICF2P774451	87702471	A	0	0	G
5	BICF2P1494170	87713738	0	0	0	G
5	BICF2P965216	87725404	0	0	0	A
5	BICF2P1059699	87741405	A	0	0	G
5	BICF2P1310593	87745364	0	0	0	G
5	TIGRP2P75960_rs8738319	87759280	A	0.5	0.2857	C
5	TIGRP2P75970_rs8603400	87777661	A	0.25	0.2857	G
5	BICF2P1310468	87780446	G	0.25	0.2857	A
5	BICF2P195967	87822280	A	0.75	0.7857	G
5	BICF2P819923	87827494	G	0	0	A
5	BICF2P557573	87843405	G	0.25	0.2143	A
5	BICF2S23247013	87862086	A	0.5	0.25	G
5	BICF2P186489	87875616	0	0	0	A
5	BICF2P728701	87890116	A	0.25	0.2143	G
5	BICF2P979227	87897065	G	0.75	0.5	A
5	TIGRP2P76018_rs9160263	87908157	G	0.25	0.5	A
5	TIGRP2P76031_rs8810392	87923699	A	0.75	0.5	G

5	BICF2P119318	87933432	G	0.25	0.2143	A
5	BICF2P420159	87942662	A	0	0	G
5	BICF2P124934	87957290	A	0	0	G
5	BICF2P209565	87964742	A	0.5	0.2857	G
5	BICF2P301512	87975292	G	0.5	0.2857	A
5	TIGRP2P76070_rs9123791	87999968	A	0	0	G
5	BICF2S23451432	88012999	C	0	0	A
5	BICF2P867787	88030262	G	0.75	0.5714	A
5	BICF2P1273720	88040212	G	0	0	A
5	BICF2P942522	88049850	A	0	0	G
5	BICF2P1138003	88067104	A	0.75	0.5	G
5	BICF2P1313425	88074382	G	0	0	A
5	TIGRP2P76110_rs9188353	88087097	A	0	0.2143	G
5	TIGRP2P76127_rs8951329	88101586	A	0	0.2143	G
5	BICF2P567309	88120720	A	0	0	G
5	BICF2P373082	88135665	C	0.75	0.5714	A
5	TIGRP2P76146_rs8893155	88136922	0	0	0	G
5	BICF2P296031	88148138	G	1	0.6	A
5	BICF2P352788	88161794	A	0	0.2143	G
5	BICF2P566038	88181594	0	0	0	G
5	BICF2S2307115	88184606	G	0.5	0.5	A
5	BICF2S23027879	88200141	G	0.25	0.2143	A
5	BICF2P286447	88205032	A	NA	0.25	G
5	BICF2P331153	88216706	A	0	0.2143	G
5	BICF2P445542	88237425	A	0.25	0.2857	G
5	BICF2P201358	88248588	C	0.25	0.5	A
5	BICF2P1389721	88251884	A	0	0	G
5	BICF2P1219736	88264347	A	0.25	0.2857	G
5	BICF2S2333065	88275610	G	0	0.2143	A
5	BICF2P734734	88307320	A	0.25	0.5	G
5	BICF2P281057	88314595	0	0	0	A
5	BICF2P657807	88320389	A	0.5	0.5	G
5	TIGRP2P76184_rs9075053	88335968	A	0.5	0.5	G
5	BICF2P414890	88343876	A	0	0.2143	G
5	BICF2S23440885	88355979	G	0.5	0.7143	A
5	BICF2P624954	88366858	C	0.5	0.2857	A
5	BICF2P832065	88377861	A	0.5	0.2857	G
5	BICF2P1258276	88397692	A	0	0	G
5	TIGRP2P76229_rs9087303	88408221	A	0	0.2143	G
5	BICF2S23256078	88427648	G	NA	NA	A
5	TIGRP2P76281_rs8824292	88444970	A	0	0	G
5	BICF2S23629284	88452146	A	1	0.7857	G
5	BICF2P1346673	88458174	A	1	0.7857	G
5	BICF2S23312942	88480992	A	NA	0.5	G
5	BICF2P611102	88501646	A	0	0.2143	G
5	BICF2S23756076	88521440	A	0	0.2143	C
5	BICF2P1335207	88527467	A	0	0.2143	G

5	TIGRP2P76340_rs8725849	88548832	A	0	0.2143	G
5	BICF2S23019786	88566093	A	0	0	G
5	BICF2P1090240	88566611	G	0	0	A
5	BICF2P293552	88580168	A	0	0	C
5	BICF2S23443354	88587395	A	0	0	G
5	TIGRP2P76367_rs8590070	88606809	A	0	0	G
5	BICF2P106837	88612152	A	1	0.7857	G
5	TIGRP2P76373_rs8981011	88625130	A	0	0	C
5	TIGRP2P76374_rs9109328	88630771	G	0	0.2143	A
5	BICF2S22953455	88643998	A	0	0	G
5	BICF2P378711	88661681	A	0	0	C
5	BICF2P569832	88666178	G	1	0.7857	A
5	BICF2P1329997	88677981	A	1	0.7857	G
5	BICF2P312287	88694403	G	1	0.7857	A
5	BICF2S23755824	88704780	G	1	1	A
5	BICF2S22941255	88727842	A	1	0.7857	G
5	BICF2S22924846	88747513	C	1	0.7857	A
5	BICF2P438108	88770412	A	1	0.7857	C
5	BICF2P173743	88781621	G	0	0.2143	A
5	BICF2P586787	88797730	A	1	0.7857	C
5	BICF2P592280	88806390	G	1	0.7857	A
5	BICF2S23710417	88816634	C	1	1	A
5	BICF2P785182	88831014	A	0	0.2143	G
5	BICF2P953115	88843019	A	0	0.2143	G
5	BICF2P949081	88853335	A	0	0.2143	C
5	BICF2P144978	88861985	G	0	0.2143	A
5	TIGRP2P76420_rs9098097	88879168	A	0	0	G
5	TIGRP2P76425_rs9126422	88885060	A	0	0	G
5	BICF2P906344	88898756	A	1	1	G
5	BICF2G630237833	88914881	G	1	0.7857	A
5	BICF2G630237827	88919869	G	1	0.7857	A
5	BICF2G630237804	88932546	G	1	0.7857	A
5	TIGRP2P76436_rs8741083	88942672	C	0	0	A
5	BICF2P1011813	88957579	G	1	0.7857	A
5	BICF2S23759230	88968141	G	1	0.7857	A
5	TIGRP2P76463_rs9080278	88978932	G	1	0.7857	A
5	BICF2P399478	88996695	A	0	0	G
5	BICF2P399479	88996895	A	1	0.7857	G
5	BICF2P1278113	88999211	C	0	0.2143	A
5	BICF2G630237730	89012083	A	1	0.7857	C
5	BICF2P1236165	89031689	A	0	0	G
5	BICF2P596593	89051311	G	1	1	A
5	BICF2G630237698	89063277	G	0	0	A
5	BICF2G630237686	89068107	A	0	0	C
5	BICF2P1336702	89079980	A	0	0	G
5	BICF2S2336655	89092415	A	0	0	C
5	BICF2P1028369	89104729	G	0	0	A

5	TIGRP2P76520_rs9181607	89124163	A	0	0	G
5	BICF2P779183	89132355	A	1	0.7857	G
5	TIGRP2P76529_rs9133954	89137039	A	0	0	G
5	BICF2G630237639	89159442	G	0	0.2143	A
5	TIGRP2P76540_rs8802141	89161462	A	0	0	G
5	BICF2G630237633	89171520	A	1	1	T
5	BICF2G630237611	89182742	G	1	1	A
5	BICF2G630237608	89205232	A	0	0	G
5	BICF2P439727	89219464	A	0	0	G
5	BICF2G630237568	89219710	A	0	0	G
5	BICF2G630237539	89234894	G	0	0	A
5	BICF2G630237514	89243493	A	0	0	C
5	BICF2G630237513	89243879	A	0	0	T
5	BICF2G630237500	89256535	G	0	0	A
5	BICF2P765879	89265727	G	0	0.2143	A
5	BICF2P1367382	89284578	A	0	0	C
5	BICF2S23331483	89287093	A	0	0	C
5	BICF2G630237460	89303504	A	0	0	C
5	BICF2G630237438	89315218	G	0	0	A
5	BICF2S23347621	89320929	T	0	0	A
5	BICF2P546692	89334048	A	0	0	G
5	BICF2P1035247	89347811	G	0	0	A
5	TIGRP2P76614_rs8873562	89358810	A	1	0.7857	G
5	BICF2P274245	89373546	A	0	0.2143	G
5	BICF2G630237378	89382833	G	1	0.7857	A
5	BICF2G630237373	89392963	A	0	0	G
5	BICF2G630237344	89407629	A	0	0	G
5	BICF2G630237338	89409097	G	0	0	A
5	BICF2S23541564	89412946	0	0	0	G
5	BICF2G630237307	89424540	G	0	0	A
5	BICF2S23738413	89454416	G	0	0	A
5	BICF2G630237292	89459387	0	0	0	G
5	BICF2G630237244	89478560	G	0	0	A
5	BICF2G630237241	89479766	T	0	0	A
5	BICF2S23332793	89489917	C	0	0	A
5	BICF2G630237233	89491611	G	0	0	A
5	BICF2P920412	89493962	0	0	0	G
5	BICF2G630237207	89508041	G	0	0.2143	A
5	BICF2G630237191	89515754	A	0	0	T
5	BICF2G630237188	89525643	A	0	0	G
5	BICF2P850257	89528706	G	0	0	C
5	BICF2P324887	89549303	C	0	0	A
5	BICF2S23621878	89570889	A	0	0	G
5	BICF2G630237153	89577861	G	0	0	A
5	BICF2G630237128	89591708	A	1	1	G
5	BICF2S232285	89596695	A	0	0	G
5	BICF2G630237105	89608146	A	0	0	G

5	BICF2G630237085	89626964	G	0	0	A
5	BICF2G630237046	89645148	A	1	0.7857	G
5	BICF2P521928	89658572	C	1	1	A
5	BICF2P807704	89671556	G	1	1	A
5	BICF2S23344251	89689742	G	0	0	A
5	BICF2G630237037	89712046	0	0	0	A
5	BICF2P1273185	89731752	A	0	0	G
5	BICF2G630237036	89740681	A	0	0	G
5	BICF2G630237034	89746207	G	0	0.2143	A
5	BICF2G630237033	89746787	G	0	0.2143	A
5	BICF2G630237018	89761894	C	0	0	A
5	BICF2G630236994	89772592	A	0	0	G
5	BICF2G630236979	89782003	A	0	0.2143	C
5	TIGRP2P76720_rs8988781	89802931	0	0	0	C
5	BICF2P1491931	89808608	A	1	0.7857	G
5	BICF2G630236945	89817604	A	0	0	G
5	BICF2S23353766	89829408	A	0	0.2143	G
5	BICF2S23720320	89838858	A	1	0.7857	G
5	BICF2S23524513	89850637	G	0	0.2143	A
5	BICF2P863931	89853750	T	1	0.7857	A
5	TIGRP2P76725_rs8624135	89863328	0	0	0	G
5	BICF2G630236901	89875344	A	0	0	G
5	BICF2G630236868	89885180	G	0	0	A
5	BICF2G630236867	89885784	C	0	0	A
5	BICF2G630236819	89904868	G	0	0	A
5	BICF2G630236807	89907161	A	0	0	G
5	BICF2P136844	89926072	A	0	0	G
5	BICF2S23159247	89930397	A	0	0	G
5	BICF2G630236750	89942584	A	1	1	G
5	BICF2P1061199	89957289	A	0	0	G
5	BICF2G630236738	89965070	A	0	0	G
5	BICF2P308118	89977342	A	0	0.2143	G
5	BICF2G630236694	89989747	A	1	1	G
5	BICF2G630236653	90000572	A	0	0	G
5	BICF2P546360	90021344	A	1	0.7857	G
5	BICF2G630236621	90026301	G	0	0	A
5	BICF2G630236596	90035404	G	1	0.7857	A
5	BICF2S23443788	90060772	C	1	0.7857	A
5	BICF2S23760858	90072280	C	1	0.7857	A
5	BICF2P103105	90087577	0	0	0	G
5	BICF2G630236529	90112725	A	0	0	G
5	BICF2G630236519	90122139	C	0	0	A
5	BICF2G630236507	90126984	A	0	0	C
5	BICF2S229290	90139777	G	0	0.2143	A
5	BICF2P808123	90157109	G	0	0	A
5	BICF2G630236470	90163881	G	0	0	A
5	BICF2P1008799	90176516	G	0	0	A

5	BICF2P938020	90200224	A	0	0.2143	G
5	BICF2G630236411	90209170	A	0	0.1667	G
5	BICF2G630236401	90220289	G	0	0	A
5	BICF2G630236389	90236384	G	0	0.2143	A
5	BICF2P1178443	90241480	G	0	0	A
5	BICF2G630236379	90252019	C	0	0	A
5	BICF2P697194	90267315	G	1	0.7857	A
5	BICF2G630236350	90283538	A	0	0	G
5	BICF2G630236310	90293896	A	0	0	G
5	BICF2G630236297	90304012	A	0	0	G
5	BICF2S23410386	90314778	A	0	0	G
5	BICF2G630236279	90327247	G	0	0	A
5	BICF2P1333832	90348856	G	1	0.7857	A
5	BICF2S22914308	90357833	A	0	0	G
5	BICF2P1434787	90369858	A	0	0	C
5	BICF2P187684	90372009	A	1	0.8333	G
5	BICF2G630236215	90379296	G	0	0	A
5	TIGRP2P76791_rs8653865	90392252	A	1	1	G
5	TIGRP2P76804_rs9164669	90412656	A	0	0.2143	G
5	BICF2P1141923	90418770	0	0	0	G
5	TIGRP2P76807_rs8731763	90424910	A	0	0	G
5	TIGRP2P76811_rs8705133	90440698	G	0	0.2143	A
5	BICF2G630236171	90455482	C	0	0.2143	A
5	BICF2G630236161	90462767	G	0	0	A
5	TIGRP2P76827_rs9121943	90470968	0	0	0	G
5	BICF2G630236127	90492472	A	0	0	C
5	BICF2G630236116	90499713	C	0	0	A
5	BICF2P975291	90511325	C	1	1	A
5	BICF2G630236082	90517537	G	0	0	A
5	BICF2P1131625	90534847	G	1	0.7857	C
5	BICF2G630236036	90540185	A	0	0	C
5	BICF2P1036138	90559706	A	0	0	G
5	BICF2G630235980	90574089	G	1	0.7857	A
5	BICF2G630235957	90586872	G	0	0.2143	A
5	BICF2G630235910	90597168	A	0	0.2143	G
5	BICF2G630235890	90609781	C	0	0.2143	A
5	TIGRP2P76894_rs8697907	90623766	A	1	0.7857	G
5	BICF2S23520348	90640246	A	1	1	G
5	BICF2G630235783	90669729	G	0	0	A
5	BICF2S2313329	90679523	0	0	0	G
5	BICF2G630235756	90692217	A	0	0.2143	G
5	BICF2G630235743	90700841	G	0	0.2143	A
5	BICF2P72255	90712702	A	0	0	G
5	BICF2G630235670	90732242	G	0	0	A
5	BICF2G630235669	90739878	A	0	0.2143	G
5	BICF2P1299170	90740011	G	1	0.7857	A
5	BICF2P322652	90754689	G	0	0	A

5	BICF2G630235651	90754943	G	0	0	A
5	BICF2G630235647	90762714	A	0	0	G
5	BICF2G630235604	90783046	A	0	0	G
5	BICF2G630235595	90798208	A	0	0	G
5	BICF2G630235554	90823836	G	0	0	A
5	BICF2G630235524	90832313	G	0	0	A
5	BICF2S2303333	90847234	C	0	0.2143	A
5	BICF2G630235498	90855474	A	1	0.7857	C
5	BICF2G630235488	90865436	A	0	0	T
5	BICF2G630235472	90872715	C	0	0	G
5	BICF2P662460	90874086	G	1	0.7857	A
5	BICF2P151720	90891935	A	0	0.2143	G
5	BICF2S22922517	90894031	G	0	0	A
5	TIGRP2P77011_rs8753364	90898048	A	1	0.7857	C
5	BICF2G630235419	90908018	A	1	0.7857	C
5	BICF2G630235405	90919209	G	1	1	C
5	BICF2S23645691	90932953	A	0	0	G
5	BICF2G630235398	90942205	A	1	0.7857	G
5	BICF2G630235381	90956816	G	0	0	A
5	BICF2G630235341	90976043	G	1	1	A
5	TIGRP2P77032_rs8975548	90986661	G	0	0	A
5	BICF2G630235288	91003652	G	1	0.7857	A
5	BICF2G630235282	91009061	G	0	0.2143	A
5	BICF2G630235264	91012391	C	0	0.2143	A
5	BICF2P1409689	91023273	A	0	0.2143	G
5	BICF2P190084	91038224	G	0	0	A
5	TIGRP2P77071_rs9152979	91046110	G	0.25	0.2857	A
5	BICF2P1165456	91058091	A	0	0.2143	G
5	BICF2P1138622	91071345	A	0	0	G
5	BICF2P597043	91084936	A	0	0.2143	G
5	BICF2P628486	91094861	C	0	0	A
5	TIGRP2P77083_rs9025204	91103068	G	0.25	0.5	A
5	BICF2P171915	91115257	G	0	0	A
5	BICF2P699067	91131687	A	0.75	0.5	G
5	BICF2P1206590	91146127	G	0.75	0.7143	A
5	BICF2S23247077	91149775	G	0.75	0.7143	A
5	BICF2P1115610	91163168	A	0	0	C
5	BICF2P940164	91168960	A	0.25	0.2857	G
5	BICF2P35852	91173383	A	0.75	0.5	G
5	BICF2P1223969	91190854	A	0.25	0.5	G
5	TIGRP2P77132_rs8686166	91213843	A	0	0	G
5	BICF2P159734	91225092	C	0.75	0.5	A
5	BICF2P4386	91230445	0	0	0	G
5	TIGRP2P77153_rs8839493	91241014	A	0	0	G
5	BICF2P705297	91260508	C	0	0.2143	A
5	BICF2P454083	91269384	A	0	0.2143	G
5	BICF2S22931980	91280774	A	0.75	0.5	G

5	BICF2S23250722	91288207	G	0.75	0.5	A
5	BICF2P568220	91302417	A	0	0.2143	G
5	TIGRP2P77169_rs8577749	91319951	A	0	0	G
5	TIGRP2P77170_rs9137174	91321670	G	0	0	A
5	TIGRP2P77173_rs9041258	91333254	A	0.75	0.7143	C
5	BICF2P488860	91341721	G	0.25	0.2857	A
5	BICF2P1094808	91366572	G	0	0	A
5	BICF2P728227	91398593	C	0.25	0.2857	A
5	BICF2P417303	91406200	G	0	0	A
5	BICF2S23519839	91416768	C	0.25	0.5	A
5	BICF2P770863	91434888	A	0	0	G
5	BICF2P1112603	91444017	A	0.25	0.5	G
5	BICF2P745624	91449056	A	0.25	0.5	G
5	BICF2P411375	91462125	A	0	0	G
5	BICF2P692485	91480005	A	0	0	G
5	BICF2P833190	91485997	A	0	0	G
5	BICF2P273921	91503697	C	0	0	G
5	BICF2P1111967	91510734	G	0.25	0.2857	A
5	BICF2S22948100	91528176	G	0	0	A
5	BICF2S22947542	91532350	A	0.75	0.5	G
5	BICF2S23544580	91545451	A	0	0	G
5	BICF2P1143605	91571096	C	0	0.2143	A
5	BICF2P675434	91579355	A	0	0	G
5	BICF2S23421987	91594916	A	0	0.2143	G
5	BICF2P1470416	91597942	A	0.25	0.2857	G
5	BICF2S22925959	91610503	A	NA	NA	G
5	BICF2P1038512	91631297	G	0.25	0.2857	A
5	BICF2P678717	91632586	G	1	1	A
5	BICF2P758545	91644658	A	0	0	G
5	BICF2P690475	91656414	A	0	0	G
5	BICF2S23535576	91675810	A	0	0	G
5	BICF2P265693	91683708	0	0	0	A
5	BICF2P1280324	91704331	A	0.25	0.2857	G
5	BICF2P568401	91719035	A	0	0.2143	G
5	BICF2P587295	91734464	A	0.75	0.7143	G
5	BICF2P827737	91738036	C	0.25	0.2857	A
5	BICF2P739108	91752189	A	0.75	0.7143	G
5	BICF2P307276	91760591	A	0.75	0.7143	G
5	BICF2S24411815	91774080	C	0.25	0.2857	A
5	BICF2S23213551	91784522	A	0.25	0.2857	G
5	BICF2P605427	91811939	G	0.75	0.5	A
5	BICF2P1103859	91819292	A	0.25	0.5	C
5	BICF2S23311810	91838553	C	1	1	A
5	BICF2P616371	91842244	G	0	0	A
5	BICF2P1106607	91850783	A	0	0	G
5	BICF2S23420334	91863559	G	0	0	A
5	BICF2P8576	91893912	A	0	0	G

5	BICF2S23153948	91908518	A	0.75	0.7143	G
5	BICF2S23529696	91928627	A	0	0	C
5	BICF2P731256	91931258	0	0	0	A
5	BICF2P798075	91969480	C	0	0	G
6	BICF2G630807302	3048830	0	0	0	G
6	BICF2S2452969	3084469	0	0	0	G
6	BICF2G630807331	3098913	0	0	0	A
6	TIGRP2P77241_rs9233066	3295041	0	0	0	A
6	BICF2G630807407	3330209	G	1	0.3571	A
6	TIGRP2P77250_rs9255272	3341099	A	0	0.2857	G
6	BICF2P302182	3363625	C	1	0.3571	G
6	G370f5S127	3425487	G	0	0	A
6	G370f11S160	3441866	0	0	0	C
6	BICF2G630807418	3457489	G	1	0.3571	A
6	BICF2P364070	3462052	C	0	0.2857	A
6	BICF2P75630	3487846	A	0	0	G
6	BICF2P186459	3509745	A	0	0.2857	G
6	BICF2P1234367	3575884	0	0	0	C
6	BICF2G630807436	3580953	G	0	0	A
6	BICF2G630807441	3590484	0	0	0	G
6	BICF2P1416917	3601436	A	0	0	G
6	BICF2P686276	3618974	C	0	0	G
6	BICF2P764557	3645062	A	0	0.2857	G
6	BICF2P1368662	3654288	0	0	0	G
6	BICF2G630807461	3687451	A	0	0	G
6	BICF2G630807462	3692623	C	0	0	A
6	BICF2G630807466	3709700	A	0	0.3571	C
6	BICF2G630807470	3728817	A	0	0.2857	G
6	BICF2P1049684	3740764	A	0	0.2857	C
6	BICF2S23221032	3742461	A	0	0.3571	G
6	BICF2G630807476	3749300	G	0	0.3571	A
6	BICF2G630807480	3767273	A	0	0.2857	G
6	BICF2G630807482	3782237	A	0	0.2857	G
6	BICF2G630807484	3807813	A	1	0.3571	G
6	BICF2G630807495	3846545	A	0	0.3571	G
6	TIGRP2P77302_rs8857434	3865999	G	0	0.2857	A
6	BICF2G630807507	3885313	A	0	0.2857	G
6	BICF2G630807509	3887416	G	0	0.2857	A
6	BICF2G630807513	3905289	G	0	0.2857	A
6	BICF2P511890	3911709	0	0	0	A
6	BICF2P1048920	3942650	G	0	0.3571	A
6	BICF2G630807522	3980304	G	0	0.2857	C
6	G371f21S197	4032421	0	0	0	G
6	BICF2P1165143	4056995	0	0	0	A
6	BICF2G630807530	4071100	A	0	0	G
6	BICF2P309937	4092736	G	0	0.3333	A
6	BICF2P346677	4098786	A	0	0.3333	G

6	BICF2G630807538	4105650	0	0	0	G
6	BICF2G630807541	4133786	0	0	0	G
6	BICF2P1005384	4147452	0	0	0	A
6	BICF2G630807542	4155133	A	0	0.3571	G
6	BICF2P1215893	4178961	G	0	0.3571	A
6	BICF2G630807550	4190046	G	0	0.3571	A
6	BICF2P1065106	4198108	C	0	0.3571	A
6	TIGRP2P77316_rs9069415	4215043	0	0	0	G
6	BICF2P1351136	4224712	A	0	0.3571	G
6	BICF2G630807553	4238404	0	0	0	A
6	BICF2P586728	4244796	G	0	0.3333	A
6	BICF2G630807554	4256792	0	0	0	C
6	BICF2P1198480	4275339	G	0	0.6429	A
6	BICF2P869258	4281473	0	0	0	A
6	BICF2G630807565	4290210	G	0	0	A
6	BICF2G630807607	4341749	C	0	0.6429	A
6	BICF2G630807614	4352128	A	0	0.6429	G
6	BICF2G630807623	4362265	A	0	0.6429	G
6	BICF2G630807631	4374013	C	0	0.6429	A
6	BICF2G630807637	4382351	G	0	0.6429	A
6	BICF2G630807650	4394855	C	0	0	A
6	BICF2G630807659	4408303	A	0	0.6429	G
6	BICF2P208106	4420632	C	0	0.6429	A
6	BICF2G630807667	4432094	C	0	0.6429	A
6	BICF2G630807676	4447220	G	0	0	A
6	BICF2G630807688	4466160	A	0	0.6429	G
6	BICF2P700648	4479706	G	0	0.6429	A
6	BICF2G630807707	4485894	G	0	0.6429	A
6	BICF2G630807718	4500852	C	0	0.6429	A
6	BICF2P204085	4516539	0	0	0	A
6	BICF2G630807744	4526933	G	0	0.6429	A
6	BICF2G630807756	4538895	C	0	0	A
6	BICF2S23659611	4547102	A	0	0.6429	C
6	BICF2G630807768	4552881	C	1	1	A
6	BICF2G630807771	4559777	G	0	0	A
6	BICF2G630807774	4584641	0	0	0	C
6	BICF2P160024	4589606	0	0	0	G
6	TIGRP2P77335_rs8717909	4602387	0	0	0	C
6	G372f27S33	4617743	G	1	1	A
6	TIGRP2P77336_rs8620358	4640630	0	0	0	C
6	G373f56S243	4683329	G	0	0.6429	A
6	BICF2S2312684	4707777	A	0	0.6429	G
6	BICF2S2313412	4739725	G	1	1	A
6	BICF2P1304023	4756066	0	0	0	A
6	BICF2S23350957	4770530	A	1	1	G
6	BICF2S23335740	4792065	A	0	0.6429	G
6	BICF2S23337456	4795578	G	1	1	A

6	BICF2S23736139	4811198	A	1	1	G
6	BICF2S23023616	4822328	C	1	1	A
6	TIGRP2P77342_rs9228179	4840409	0	0	0	A
6	BICF2S23133566	4844121	G	1	1	A
6	BICF2P388710	4883081	0	0	0	G
6	TIGRP2P77343_rs8588595	4908449	0	0	0	A
6	TIGRP2P77345_rs8617758	4930165	0	0	0	G
6	BICF2S2335528	4936366	A	1	1	G
6	BICF2S23630642	4951931	C	1	1	G
6	BICF2P234410	4962643	0	0	0	A
6	BICF2S23661934	4970337	G	1	0.3571	A
6	BICF2S23344691	4996791	G	1	1	A
6	BICF2S23012633	5018141	G	0	0.6429	A
6	BICF2S23061237	5041725	C	0	0.6429	A
6	BICF2S23028626	5071386	G	1	1	A
6	BICF2P104379	5075243	0	0	0	G
6	BICF2G630807793	5107541	A	0	0	G
6	BICF2G630807799	5126167	0	0	0	G
6	BICF2P674590	5131197	G	1	1	A
6	BICF2G630807819	5151644	0	0	0	C
6	BICF2S23332716	5165742	0	0	0	A
6	BICF2G630807851	5176582	G	1	1	A
6	BICF2P923427	5191624	0	0	0	A
6	BICF2G630807874	5205381	A	1	1	C
6	BICF2G630807875	5205615	G	1	1	A
6	BICF2P1128572	5211171	0	0	0	G
6	BICF2G630807889	5224091	G	0	0.2857	A
6	BICF2G630807891	5240929	0	0	0	G
6	BICF2P538164	5249293	0	0	0	A
6	BICF2P1399225	5258267	0	0	0	A
6	G374f43S325	5282126	0	0	0	A
6	BICF2G630807896	5311154	A	1	0.7143	C
6	BICF2P879392	5319290	0	0	0	A
6	BICF2G630807897	5327243	0	0	0	A
6	BICF2G630807901	5337960	0	0	0	A
6	TIGRP2P85299_rs8984073	5346888	0	0	0	C
6	BICF2G630807902	5356035	A	1	0.7143	G
6	BICF2G630807903	5369220	0	0	0	A
6	BICF2P169873	5372611	0	0	0	C
6	BICF2G630807904	5386786	0	0	0	G
6	BICF2P1364421	5403969	0	0	0	G
6	BICF2P1222717	5422740	0	0	0	A
6	BICF2G630807909	5444016	G	1	0.7143	A
6	BICF2G630807914	5465923	0	0	0	G
6	BICF2G630807917	5476144	A	0	0.3571	G
6	TIGRP2P85307_rs8662940	5492171	0	0	0	A
6	BICF2S2342680	5508702	A	0	0	G

6	BICF2P1060947	5528289	0	0	0	G
6	TIGRP2P85310_rs9185345	5552948	0	0	0	G
6	BICF2S2302533	5555696	C	0	0.6429	A
6	BICF2S23156663	5572455	G	0	0.6429	A
6	BICF2G630807928	5597988	A	0	0	T
6	BICF2P1381769	5608401	0	0	0	A
6	TIGRP2P85311_rs9195922	5619938	G	0	0	A
6	BICF2P621026	5663568	G	0	0	A
6	BICF2S2335197	5671106	G	0	0	A
6	BICF2P632198	5686380	0	0	0	G
6	TIGRP2P85315_rs8803473	5694610	A	0	0.2857	G
6	BICF2S23224285	5704091	G	0	0	A
6	BICF2P1433712	5721549	G	0	0	A
6	BICF2P658867	5749209	A	0	0	G
6	BICF2P342547	5754903	A	0	0	G
6	BICF2P874933	5758133	A	0	0	G
6	BICF2G630807938	5767336	0	0	0	C
6	BICF2P922787	5770721	G	0	0	A
6	BICF2P1005381	5779207	A	0	0	G
6	BICF2P880946	5786349	A	0	0	G
6	BICF2P1335911	5799020	A	0	0	G
6	BICF2P790802	5810672	A	0	0	G
6	BICF2P795577	5822572	A	0	0.6429	G
6	BICF2P438014	5831638	0	0	0	A
6	BICF2P939836	5843844	T	0	0	A
6	BICF2P695305	5858924	A	0	0.2857	G
6	BICF2P189516	5870138	A	0	0	G
6	BICF2G630807941	5885076	G	1	0.7143	A
6	BICF2P208674	5896276	G	1	0.7143	A
6	BICF2P121934	5901586	0	0	0	A
6	TIGRP2P85325_rs8639285	5911378	G	0	0	A
6	BICF2G630807943	5930654	G	1	0.7143	A
6	BICF2P900559	5943325	G	0	0	A
6	BICF2G630807946	5948170	C	0	0	A
6	BICF2G630807947	5962902	A	0	0	G
6	TIGRP2P85332_rs8678007	5974172	A	1	1	G
6	BICF2G630807951	5980969	A	0	0	G
6	BICF2G630807959	5999698	0	0	0	G
6	BICF2G630807962	6007585	A	0	0	G
6	TIGRP2P85335_rs8968831	6017588	G	1	1	A
6	BICF2G630807965	6030686	A	0	0.2857	C
6	BICF2G630807968	6045609	A	0	0	G
6	BICF2G630807972	6049578	G	0	0	A
6	TIGRP2P85341_rs8897195	6053960	A	0	0	G
6	BICF2G630807977	6061620	G	1	1	A
6	BICF2G630807979	6067508	G	1	1	A
6	BICF2G630807981	6072266	G	1	0.7143	A

6	BICF2P119720	6093526	A	0	0.2857	G
6	BICF2P415895	6099202	C	0	0	A
6	BICF2S23050935	6107984	A	0	0	G
6	BICF2P646823	6119461	G	0	0.2857	A
6	BICF2P389457	6145173	A	0	0	G
6	BICF2P189450	6175268	0	0	0	A
6	TIGRP2P85358_rs9009190	6183638	A	0	0.2857	G
6	BICF2P998045	6193477	A	0	0	G
6	BICF2S2333373	6209899	G	0	0.2857	C
6	BICF2P270925	6216176	A	0	0.2857	G
6	BICF2P458065	6223608	0	0	0	A
6	BICF2P899400	6235345	G	0	0.2857	A
6	BICF2P1150504	6253442	0	0	0	G
6	BICF2P231069	6259931	0	0	0	A
6	BICF2P567461	6269182	G	0	0.2857	C
6	BICF2G630807993	6283197	0	0	0	G
6	BICF2P867435	6291535	A	0	0.2857	C
6	BICF2P785778	6307446	A	0	0	G
6	BICF2P440904	6320310	G	0	0.2857	A
6	BICF2G630807994	6327465	G	0	0	A
6	TIGRP2P85373_rs9017032	6340439	0	0	0	C
6	BICF2P481732	6351099	C	0	0.2857	A
6	BICF2G630807999	6365646	0	0	0	A
6	TIGRP2P85377_rs9069041	6380698	C	0	0	A
6	BICF2P524562	6384649	T	0	0	A
6	BICF2G630808002	6398781	0	0	0	G
6	BICF2P1403221	6406947	G	0	0.25	A
6	BICF2P1367401	6424507	A	0	0	G
6	TIGRP2P85384_rs8956654	6443279	0	0	0	G
6	TIGRP2P85385_rs9028187	6455895	G	0	0.2857	A
6	BICF2P732401	6467443	A	0	0.2857	G
6	BICF2P515380	6482411	0	0	0	G
6	BICF2P310287	6506874	G	0	0.2857	A
6	BICF2G630808007	6521502	0	0	0	G
6	BICF2P1049268	6534913	A	0	0.2857	G
6	BICF2P974170	6554302	A	0	0.2857	G
6	BICF2G630808008	6555729	0	0	0	G
6	TIGRP2P85393_rs9077443	6575350	G	0	0.2857	C
6	TIGRP2P85396_rs9217314	6578790	G	0	0	A
6	TIGRP2P85399_rs9207475	6590390	A	0	0	G
6	BICF2G630808010	6603115	G	0	0.2857	A
6	BICF2P877588	6622270	G	0	0	A
6	BICF2P877587	6624478	A	0	0	G
6	BICF2P138086	6645963	0	0	0	A
6	BICF2P685466	6665132	G	0	0.2857	A
6	BICF2P1276465	6678458	A	0	0	C
6	BICF2P625713	6691308	A	0	0	G

6	BICF2P435896	6702494	G	0	0.2857	A
6	BICF2P776106	6706961	A	0	0.2857	C
6	BICF2G630808020	6716301	A	0	0	T
6	BICF2P746431	6737023	A	0	0.2857	G
6	BICF2P657904	6743737	A	0	0	G
6	BICF2S23753813	6761771	C	0	0.2857	A
6	BICF2P569510	6783122	A	0	0.2857	G
6	BICF2P433079	6787603	G	0	0.2857	A
6	BICF2S23214236	6804191	A	0	0	G
6	BICF2P666773	6819087	T	0	0	A
6	BICF2G630808022	6821211	0	0	0	G
6	TIGRP2P85426_rs9127206	6832400	A	0	0	G
6	BICF2P1018975	6842888	G	0	0	A
6	BICF2P19143	6848071	A	0	0	C
6	BICF2G630808028	6862977	G	0	0	A
6	BICF2G630808031	6868873	A	0	0	T
6	BICF2G630808034	6880175	A	0	0	G
6	BICF2G630808041	6894390	G	0	0.2857	A
6	BICF2G630808048	6910874	0	0	0	A
6	BICF2P1148245	6913635	A	0	0	T
6	BICF2G630808055	6929204	0	0	0	G
6	BICF2G630808058	6934749	A	0	0	C
6	BICF2P425383	6953009	0	0	0	A
6	BICF2G630808073	6971304	G	0	0	A
6	BICF2G630808075	6984659	A	0	0	T
6	BICF2P957230	6998342	A	0	0	G
6	TIGRP2P85444_rs8695680	7009542	A	0	0	G
6	BICF2P864992	7019553	A	0	0	T
6	BICF2P711144	7034558	A	0	0	G
6	BICF2G630808078	7046446	G	0	0	A
6	BICF2S23441278	7052538	G	0	0	A
6	BICF2P427009	7066382	G	0	0	A
6	BICF2P560997	7072609	A	0	0	G
6	TIGRP2P85455_rs8949533	7087246	G	0	0.3571	A
6	TIGRP2P85459_rs8744175	7095917	C	0	0.3571	A
6	BICF2G630808104	7114293	G	0	0.3571	A
6	BICF2G630808120	7128438	C	0	0	A
6	BICF2G630808125	7136835	0	0	0	C
6	BICF2P788615	7148724	C	0	0	A
6	BICF2P965582	7151393	A	0	0	G
6	BICF2G630808134	7151783	A	0	0	G
6	BICF2G630808135	7153065	A	0	0	C
6	BICF2P298753	7166152	G	0	0	A
6	BICF2P1065607	7184264	G	0	0.3571	A
6	BICF2P1065608	7184358	G	0	0.3571	A
6	BICF2P487152	7190211	C	0	0.3571	A
6	BICF2G630808154	7191400	A	0	0	T

6	BICF2G630808161	7195213	T	0	0	A
6	BICF2G630808172	7202222	A	0	0	G
6	BICF2G630808190	7214015	G	0	0	A
6	TIGRP2P85490_rs9242706	7222463	A	0	0	C
6	BICF2P914005	7242740	A	0	0	C
6	BICF2P252126	7246794	C	0	0	A
6	BICF2G630808208	7265846	A	0	0	C
6	BICF2G630808212	7268892	G	0	0	A
6	BICF2G630808220	7281984	A	0	0.3571	G
6	BICF2G630808232	7300950	T	0	0	A
6	BICF2G630808235	7307422	A	0	0	G
6	BICF2G630808241	7317201	G	1	0.7143	A
6	BICF2G630808246	7330203	G	0	0.3571	A
6	BICF2G630808253	7341001	A	0	0.3571	G
6	TIGRP2P85505_rs8537382	7343974	A	0	0.2857	G
6	BICF2P1163743	7350501	G	0	0.2857	A
6	BICF2G630808276	7362514	A	0	0	G
6	BICF2G630808290	7374436	A	0	0.6429	G
6	BICF2G630808297	7387353	A	0	0	C
6	BICF2P200610	7402781	A	1	0.3571	G
6	BICF2P1026609	7403259	A	1	0.3571	T
6	BICF2G630808339	7410716	A	1	0.3571	G
6	BICF2G630808343	7419897	A	1	0.3571	G
6	BICF2G630808360	7435195	A	0	0	G
6	TIGRP2P85563_rs9122879	7443288	C	0	0	A
6	BICF2G630808377	7454888	G	0	0	A
6	BICF2G630808386	7472781	G	0	0.6429	A
6	BICF2G630808387	7476674	A	0	0	G
6	BICF2G630808404	7487908	G	0	0.6429	A
6	BICF2G630808430	7515010	A	0	0	G
6	BICF2S2364813	7524143	G	0	0.6429	A
6	BICF2G630808431	7536473	0	NA	0	G
6	BICF2P413222	7555654	0	0	0	T
6	BICF2S23221602	7588608	G	0	0.3571	A
6	BICF2S23413244	7601735	A	0	0.3571	G
6	G375f38S36	7625642	0	0	0	A
6	BICF2P405533	7654387	0	0	0	A
6	BICF2G630808437	7663934	0	0	0	G
6	BICF2P487203	7675667	0	0	0	A
6	BICF2S2366976	7691403	0	0	0	G
6	BICF2S23113663	7699463	A	1	0.3571	G
6	BICF2P833371	7708590	A	0	0	G
6	G376f41S131	7728673	0	0	0	C
6	BICF2P1179970	7748592	G	0	0	A
6	BICF2P341188	7766292	0	0	0	A
6	BICF2P1045674	7781805	0	0	0	A
6	BICF2P920080	7791557	0	0	0	A

6	BICF2G630808440	7822527	A	0	0	G
6	BICF2P450641	7855826	0	0	0	A
6	BICF2G630808441	7872661	0	0	0	G
6	BICF2P616879	7885305	0	0	0	A
6	BICF2G630808444	7905370	A	0	0	C
6	BICF2G630808446	7957181	G	0	0.3571	A
6	BICF2G630808450	8019902	A	0	0	C
6	BICF2P42048	8030174	A	0	0.3571	G
6	BICF2G630808463	8033272	G	0	0.3571	A
6	BICF2G630808481	8049336	G	0	0.3571	A
6	BICF2G630808483	8051454	A	0	0.3571	G
6	BICF2G630808519	8064625	A	0	0	G
6	BICF2P886253	8073345	A	0	0	G
6	BICF2G630808533	8094239	0	0	0	G
6	BICF2G630808539	8104856	A	0	0	G
6	BICF2G630808541	8109560	A	0	0	G
6	BICF2G630808556	8121763	A	0	0	G
6	BICF2G630808560	8130674	0	0	0	C
6	BICF2P283817	8144107	T	0	0	A
6	BICF2P278591	8154132	A	0	0	G
6	BICF2S2374580	8167051	G	0	0	A
6	BICF2G630808588	8177077	A	0	0	G
6	BICF2G630808592	8182275	A	0	0	C
6	BICF2P530224	8188256	C	0	0	A
6	BICF2G630808600	8221834	0	0	0	A
6	BICF2S23148661	8231439	A	0	0.3571	G
6	BICF2G630808603	8234131	G	0	0	A
6	BICF2G630808607	8249919	A	1	0.3571	G
6	BICF2P93749	8273411	0	0	0	A
6	BICF2P889665	8286341	C	0	0.2857	G
6	TIGRP2P85584_rs9119519	8300910	G	1	0.6429	A
6	BICF2P418360	8307288	A	0	0.2857	G
6	BICF2P1305352	8317816	0	0	0	A
6	BICF2G630808630	8330338	G	1	0.6429	A
6	BICF2G630808634	8334939	0	0	0	G
6	BICF2G630808635	8337821	A	0	0	G
6	BICF2G630808646	8349878	A	0	0	C
6	BICF2G630808666	8362242	A	0	0	G
6	BICF2G630808674	8373481	A	0	0.2857	G
6	BICF2G630808687	8383828	0	0	0	G
6	BICF2G630808690	8395888	0	0	0	A
6	BICF2G630808698	8414432	A	1	1	G
6	BICF2S23046882	8438812	A	1	0.3571	G
6	BICF2G630808719	8444136	0	0	0	G
6	BICF2G630808730	8453008	0	0	0	A
6	BICF2P1200540	8466080	G	0	0	A
6	BICF2G630808760	8480773	A	0	0	G

6	BICF2G630808767	8489299	A	0	0.3571	C
6	BICF2G630808783	8499991	A	0	0.3571	C
6	BICF2P573422	8529188	0	0	0	G
6	BICF2G630808787	8555692	A	0	0.6429	G
6	BICF2P1369869	8556068	A	1	0.3571	G
6	BICF2G630808804	8569675	A	0	0	G
6	BICF2G630808812	8586739	A	0	0.2857	G
6	BICF2P794739	8596608	A	0	0.3571	G
6	BICF2S23133902	8604567	A	0	0	G
6	BICF2G630808834	8611722	0	0	0	G
6	TIGRP2P85630_rs9243498	8614525	A	1	0.3571	G
6	BICF2G630808859	8627632	G	1	0.3	A
6	BICF2S23618658	8636804	0	0	0	A
6	BICF2P112316	8657708	A	1	0.3571	G
6	BICF2P64033	8670477	C	0	0	A
6	BICF2P97144	8675095	T	0	0.6667	A
6	BICF2P952702	8690202	A	1	0.3571	C
6	TIGRP2P85650_rs8622789	8694979	C	0	0.6429	A
6	TIGRP2P85656_rs8866227	8714281	C	0	0.6667	A
6	BICF2G630808907	8723326	A	0	0.2857	G
6	BICF2G630808914	8736232	A	0	0.2857	G
6	BICF2G630808921	8742463	A	0	0.2857	G
6	BICF2G630808932	8752410	A	0	0.3571	G
6	BICF2G630808939	8765891	G	0	0.3571	A
6	BICF2G630808945	8778049	0	0	0	G
6	BICF2G630808956	8795934	A	0	0.3571	G
6	BICF2G630808966	8806510	A	0	0	G
6	TIGRP2P85684_rs9036504	8811028	G	0	0.3571	A
6	BICF2S23012129	8824250	A	0	0.2857	G
6	BICF2P187556	8834992	A	0	0.3571	G
6	BICF2P1195281	8850065	C	1	0.3571	G
6	BICF2S2293092	8857920	0	0	0	A
6	BICF2G630808997	8866867	C	1	0.3571	A
6	BICF2G630809008	8882290	A	1	0.3571	G
6	BICF2P1175380	8891211	A	1	0.6429	G
6	BICF2G630809017	8903364	A	0	0	G
6	BICF2P291644	8913574	A	0	0.3571	G
6	BICF2G630809065	8930360	G	1	0.6429	A
6	BICF2G630809074	8934044	T	1	0.6429	A
6	BICF2P1291646	8939214	A	0	0.2857	G
6	BICF2S23354079	8957416	A	1	0.7143	G
6	BICF2G630809102	8968628	A	0	0.2857	G
6	BICF2G630809112	8972089	A	0	0.2857	G
6	BICF2G630809130	8983376	A	0	0	G
6	BICF2G630809142	8996718	G	0	0	A
6	BICF2G630809161	9014652	A	0	0	G
6	BICF2G630809165	9017867	G	0	0	A

6	BICF2G630809194	9040198	G	0	0	A
6	TIGRP2P85735_rs8612074	9043224	A	0	0	G
6	BICF2G630809202	9052787	A	0	0	G
6	TIGRP2P85743_rs8643382	9066007	A	1	0.6429	G
6	TIGRP2P85748_rs8641594	9074446	A	1	0.6429	G
6	TIGRP2P85754_rs8602612	9095578	T	1	0.6429	A
6	TIGRP2P85758_rs8905305	9098056	A	0	0.2857	G
6	BICF2P894756	9110284	A	0	0	G
6	BICF2G630809296	9117947	G	0	0	A
6	BICF2G630809301	9125232	A	0	0	C
6	BICF2G630809319	9135681	G	0	0	A
6	TIGRP2P85763_rs8693289	9144002	A	0	0.2857	G
6	BICF2G630809337	9161707	A	0	0	G
6	BICF2G630809343	9168664	A	0	0.2857	G
6	BICF2G630809349	9183325	G	1	0.7143	A
6	BICF2G630809350	9183957	C	1	1	A
6	BICF2P206518	9211190	0	0	0	A
6	BICF2P361906	9219378	A	0	0.3571	G
6	BICF2G630809365	9225774	A	0	0.3571	G
6	BICF2G630809373	9254578	A	0	0.6429	G
6	BICF2S23016970	9268507	G	1	1	A
6	BICF2P1413964	9271826	A	0	0.6429	G
6	TIGRP2P85780_rs9028355	9281668	A	0	0	G
6	BICF2G630809385	9300303	G	1	1	A
6	BICF2P1031334	9304579	G	1	1	A
6	BICF2P504081	9317612	0	0	0	A
6	BICF2G630809392	9333490	G	0	0	A
6	BICF2G630809397	9344517	G	0	0	A
6	BICF2G630809405	9352755	A	1	0.7143	C
6	BICF2G630809420	9363690	0	0	0	C
6	BICF2P58503	9379907	A	1	0.6429	G
6	BICF2G630809426	9389502	A	1	0.6667	G
6	BICF2G630809438	9425834	A	0	0	G
6	BICF2G630809443	9430116	A	0	0	G
6	BICF2P784242	9448887	A	0	0	G
6	BICF2G630809468	9454619	0	0	0	A
6	BICF2P573880	9468364	A	0	0.3571	G
6	TIGRP2P85800_rs8665808	9478785	G	0	0.6429	A
6	BICF2G630809489	9493730	G	0	0.6429	A
6	BICF2S23430467	9501976	A	0	0.6429	G
6	BICF2G630809506	9534669	C	0	0.6429	A
6	BICF2P38863	9540366	G	0	0.6429	A
6	TIGRP2P85803_rs8963098	9549074	A	0	0.6429	T
6	BICF2S23740063	9560403	A	0	0	G
6	BICF2G630809515	9573756	A	0	0.6429	C
6	BICF2G630809517	9586001	G	0	0.6429	A
6	BICF2S23623800	9600690	G	0	0	A

6	BICF2G630809522	9633039	A	0	0	G
6	TIGRP2P85806_rs9039902	9637936	0	0	0	G
6	BICF2G630809523	9651466	G	0	0.6429	A
6	BICF2G630809527	9665608	G	0	0.3571	A
6	BICF2G630809531	9674795	T	0	0	A
6	BICF2G630809536	9685064	A	0	0.2857	G
6	BICF2G630809548	9698547	A	0	0.2857	G
6	BICF2G630809552	9711819	G	0	0.2857	A
6	BICF2G630809557	9719984	C	0	0	A
6	BICF2P1373355	9730302	G	0	0	A
6	BICF2P313907	9745700	0	0	0	C
6	BICF2G630809578	9757683	G	0	0.2857	A
6	BICF2G630809584	9764959	A	0	0	G
6	BICF2G630809590	9776467	A	0	0	C
6	BICF2G630809602	9797780	C	0	0	A
6	BICF2G630809617	9816608	G	0	0	A
6	BICF2P756196	9830592	A	0	0.3571	G
6	BICF2G630809636	9836478	G	0	0	A
6	BICF2G630809645	9844100	A	0	0	G
6	BICF2G630809664	9859615	A	0	0	G
6	BICF2G630809679	9867633	A	0	0	G
6	BICF2G630809703	9878732	A	0	0	G
6	BICF2G630809704	9878905	A	0	0	G
6	BICF2G630809733	9898267	A	0	0.6429	G
6	BICF2G630809739	9908416	A	0	0.6429	G
6	BICF2G630809754	9921343	A	0	0.2857	G
6	BICF2G630809759	9926174	G	0	0.6429	A
6	BICF2G630809788	9944620	G	0	0.6429	A
6	BICF2S2373477	9978392	C	0	0	A
6	TIGRP2P85832_rs9184468	9998099	0	0	0	C
6	G378f45S224	10006580	A	0	0.3571	C
6	BICF2S23225290	10026042	A	1	0.7143	G
6	BICF2G630809797	10031286	0	0	0	A
6	BICF2S22927431	10040320	A	1	0.7143	C
6	TIGRP2P85833_rs8822660	10060721	G	0	0.2857	C
6	BICF2S23439135	10065193	C	1	0.7143	G
6	BICF2S23629444	10089941	G	1	0.7143	A
6	BICF2P1221621	10113879	0	0	0	C
6	TIGRP2P85841_rs8598796	10127152	A	0	0.3571	G
6	BICF2P1405819	10139176	A	0	0	G
6	BICF2P384372	10154114	A	0	0	G
6	BICF2S22913951	10156015	G	1	0.3571	A
6	BICF2S23634217	10171062	0	0	0	G
6	TIGRP2P85850_rs9028642	10178625	A	1	0.3571	G
6	BICF2S2363070	10208513	A	1	0.3571	G
6	BICF2G630809810	10234121	A	0.5	0.2143	G
6	BICF2G630809814	10237567	G	1	0.7143	A

6	BICF2G630809819	10254073	A	0	0.2857	G
6	BICF2G630809838	10271748	G	0	0.2857	A
6	TIGRP2P77353_rs8979714	10282579	A	0	0.2857	G
6	BICF2G630809855	10287408	G	0.5	0.2143	A
6	BICF2G630809858	10292711	A	0	0.2857	G
6	BICF2S23121456	10307641	A	0	0	G
6	BICF2G630809889	10317422	A	0	0	G
6	BICF2G630809896	10336273	A	0	0	G
6	BICF2G630809900	10339000	G	0	0	A
6	BICF2P115812	10354346	C	0.5	0.5	G
6	BICF2G630809917	10364926	G	0.5	0.5	A
6	BICF2G630809937	10383477	A	1	1	G
6	BICF2G630809939	10385533	G	0.5	0.5	A
6	BICF2G630809964	10399453	G	0.5	0.5	A
6	BICF2G630809974	10407672	A	0	0	G
6	BICF2G630809997	10429516	A	0.5	0.5	C
6	BICF2G630810005	10438635	A	0.5	0.5	G
6	TIGRP2P77381_rs9090428	10450247	A	0	0	G
6	BICF2G630810029	10456010	A	0.5	0.5	G
6	BICF2P955008	10470181	G	0	0	A
6	BICF2P1023099	10486603	G	0.5	0.5	A
6	BICF2G630810035	10487125	G	0.5	0.5	A
6	BICF2P243201	10489838	A	0	0	G
6	BICF2P764928	10499548	A	0.5	0.5	G
6	BICF2P642986	10516282	C	0	0	A
6	BICF2P1306827	10531823	A	0	0	T
6	BICF2P1076842	10543888	0	0	0	G
6	BICF2S23621423	10553491	0	0	0	A
6	BICF2G630810040	10561492	T	0.5	0.5	A
6	BICF2S24415287	10575221	A	0	0	C
6	TIGRP2P77399_rs8829879	10586075	A	0	0	G
6	BICF2P877858	10588551	G	0	0	A
6	TIGRP2P77405_rs8498134	10592446	A	0	0	G
6	TIGRP2P77409_rs8493131	10603523	0	0	0	G
6	BICF2G630810056	10615480	A	0	0	C
6	BICF2P987030	10635387	G	0	0	A
6	BICF2G630810069	10636139	A	1	1	G
6	BICF2G630810073	10638196	G	1	1	A
6	BICF2G630810079	10650101	A	0	0	G
6	BICF2G630810101	10667040	A	0	0	G
6	BICF2G630810109	10679173	G	0	0	C
6	BICF2G630810120	10694015	G	0	0	A
6	BICF2P697084	10704445	A	0	0	G
6	BICF2P467354	10726039	G	0	0	A
6	BICF2P682297	10735534	G	1	1	A
6	BICF2P682295	10736387	A	0	0	G
6	TIGRP2P77443_rs8790294	10743546	G	0	0	A

6	BICF2S23652505	10762282	G	0	0	A
6	TIGRP2P77447_rs9077638	10766523	A	0.5	0.5	G
6	BICF2P327568	10779815	C	1	1	A
6	BICF2S23248600	10795566	A	0.5	0.1429	G
6	BICF2P342880	10799366	0	0	0	G
6	TIGRP2P77455_rs9078707	10812162	T	0	0	A
6	BICF2P1465616	10815849	0	0	0	G
6	BICF2P300904	10823730	T	0	0	A
6	BICF2G630810130	10839065	A	0	0	C
6	BICF2P1441066	10855464	A	0	0	G
6	TIGRP2P77469_rs9255898	10861512	0	0	0	G
6	BICF2P495282	10876452	T	1	1	A
6	BICF2S23428867	10876861	A	1	1	G
6	BICF2P495280	10877248	0	0	0	A
6	BICF2P1114126	10883206	A	1	1	G
6	BICF2P912520	10899201	A	1	1	G
6	TIGRP2P77484_rs9160374	10905976	A	0	0	G
6	BICF2P1359735	10918203	0	0	0	A
6	TIGRP2P77495_rs9051517	10932750	G	0	0	A
6	BICF2P821586	10940087	C	1	1	A
6	TIGRP2P77502_rs8717555	10951738	G	0	0	A
6	BICF2P1290654	10958588	A	0.5	0.8571	G
6	BICF2P998154	10961992	G	1	1	A
6	BICF2S22934028	10975957	G	0	0	A
6	BICF2P575737	10992901	G	1	1	A
6	BICF2P575733	10994213	C	1	1	A
6	BICF2P669712	11009206	G	1	1	C
6	BICF2P1026297	11020002	A	0	0	G
6	BICF2P704063	11035074	G	0.5	0.1429	A
6	BICF2P559912	11048220	A	0.5	0.1429	C
6	BICF2S2321981	11052200	G	0.5	0.1429	A
6	BICF2P945044	11066613	A	0	0	G
6	TIGRP2P77537_rs8866486	11074638	G	0	0	A
6	BICF2P477684	11096212	G	0.5	0.1429	A
6	BICF2P491986	11106977	A	0.5	0.1429	G
6	TIGRP2P77546_rs8851635	11112698	0	0	0	G
6	TIGRP2P77549_rs8918169	11123290	A	0	0	G
6	BICF2G630810143	11130832	G	0.5	0.1429	A
6	BICF2P740888	11132170	C	0	0	A
6	BICF2G630810149	11154363	G	0	0	A
6	TIGRP2P77558_rs8954907	11163148	G	0	0	C
6	BICF2G630810159	11177149	G	0.5	0.1429	A
6	BICF2G630810173	11181920	A	0.5	0.1429	G
6	BICF2P32896	11213909	G	0	0	A
6	BICF2S22950026	11214576	A	0	0	G
6	TIGRP2P77576_rs9025297	11228500	A	0	0	T
6	BICF2S23310435	11245775	A	0	0	G

6	BICF2P552025	11252689	A	0	0	G
6	BICF2P387089	11263777	0	0	0	G
6	BICF2P945074	11276256	A	0.5	0.8571	G
6	BICF2P529738	11284638	A	0	0	G
6	TIGRP2P77604_rs8593295	11308719	A	0.5	0.1429	G
6	BICF2S22958911	11319148	A	0	0	G
6	BICF2P206053	11333515	G	0.5	0.1429	A
6	TIGRP2P77615_rs8965168	11346649	G	0	0	A
6	TIGRP2P77618_rs8741496	11350761	A	0	0	G
6	BICF2S23729890	11375391	A	0.5	0.8571	T
6	BICF2P254777	11398772	0	0	0	G
6	BICF2P124399	11418616	A	0	0	G
6	BICF2P1332903	11424752	A	0	0	C
6	BICF2P170661	11439931	A	0.5	0.8571	G
6	BICF2P412007	11451540	A	0.5	0.8571	G
6	BICF2P1354767	11462695	G	0.5	0.8571	A
6	BICF2P472346	11474009	A	0.5	0.9167	G
6	BICF2P205255	11484772	A	0.5	0.1429	G
6	BICF2P763423	11497039	C	0.5	0.8571	A
6	BICF2P837893	11506498	G	0	0	A
6	TIGRP2P77646_rs9074967	11514648	0	0	0	A
6	BICF2P925836	11524207	A	0.5	0.1429	G
6	TIGRP2P77652_rs9122981	11535417	0	0	0	C
6	TIGRP2P77653_rs9032601	11569936	A	0.5	0.1429	G
6	BICF2S24111320	11589080	G	0	0	A
6	TIGRP2P77661_rs8793881	11600849	G	1	1	A
6	BICF2P1381179	11605114	C	1	1	G
6	TIGRP2P77677_rs8519218	11608766	G	1	1	A
6	BICF2P1430225	11608900	A	1	1	G
6	BICF2S2296995	11616418	G	1	1	A
6	BICF2P354986	11632379	A	1	1	C
6	TIGRP2P77687_rs8584168	11646154	0	0	0	C
6	BICF2P1231900	11659824	A	0.5	0.1667	G
6	TIGRP2P77693_rs8680618	11663197	G	1	1	A
6	BICF2P642393	11665493	G	1	1	A
6	TIGRP2P77697_rs9073780	11678147	G	1	1	A
6	BICF2S23029673	11690964	A	1	1	G
6	BICF2P686512	11705024	A	1	1	G
6	BICF2P637416	11712666	A	0	0	G
6	TIGRP2P77702_rs8638668	11713666	A	0	0	G
6	BICF2P1375625	11719143	G	0	0	A
6	BICF2P650989	11732013	A	0.5	0.8571	C
6	BICF2S2366502	11742507	A	0	0	G
6	TIGRP2P77740_rs8999913	11753630	G	0	0	A
6	BICF2S23547427	11765750	C	NA	0.08333	A
6	BICF2P379548	11785775	0	0	0	A
6	BICF2S23216925	11794401	A	0	0	C

6	BICF2P179458	11802500	T	0	0	A
6	TIGRP2P77745_rs9176333	11820555	G	0	0	A
6	BICF2S23745316	11824187	A	0	0	G
6	TIGRP2P77772_rs8534320	11833822	0	0	0	A
6	BICF2S2343751	11853430	G	0	0	A
6	TIGRP2P77778_rs8924525	11867729	0	0	0	C
6	TIGRP2P77783_rs8491126	11876794	A	0	0	G
6	TIGRP2P77788_rs8658572	11886329	0	0	0	A
6	BICF2P1405965	11899552	A	0.5	0.1429	C
6	BICF2P1327277	11904372	A	0	0	G
6	TIGRP2P77794_rs8909680	11920326	G	0	0	A
6	BICF2P177358	11933630	A	0	0	G
6	BICF2S23337047	11938181	A	0.5	0.1667	G
6	BICF2P299916	11955836	0	0	0	C
6	TIGRP2P77797_rs8964151	11962340	A	0	0	G
6	TIGRP2P77806_rs9175977	11982455	A	0	0	G
6	TIGRP2P77808_rs8624745	11984193	G	0.5	0.8571	A
6	TIGRP2P77822_rs8918983	12002285	A	0.5	0.8571	G
6	TIGRP2P77827_rs8950242	12012400	C	0	0	A
6	BICF2S23622530	12021986	A	0	0	G
6	BICF2P2926	12032507	0	0	0	G
6	TIGRP2P77831_rs9121910	12050970	A	0.5	0.8571	T
6	BICF2P331957	12059688	A	0	0	G
6	TIGRP2P77836_rs8871869	12063246	G	0.5	0.8571	A
6	BICF2P536041	12072681	0	0	0	G
6	BICF2P835967	12075668	G	0.5	0.1429	A
6	TIGRP2P77845_rs8807963	12091870	G	0.5	0.1429	A
6	TIGRP2P77850_rs8992811	12101971	A	0.5	0.8571	G
6	BICF2P605563	12102033	A	0.5	0.8571	G
6	BICF2P711427	12112784	A	0	0	G
6	BICF2P491557	12126804	G	0.5	0.1429	A
6	BICF2S23211211	12147147	0	0	0	G
6	TIGRP2P77862_rs8495477	12158330	0	0	0	G
6	BICF2P346286	12168542	0	0	0	A
6	BICF2S2323842	12186191	G	0	0	A
6	BICF2P1344319	12201458	A	0.5	0.8571	C
6	TIGRP2P77865_rs9194262	12207719	C	0.5	0.8571	A
6	BICF2P254004	12215067	G	0	0	A
6	BICF2S23228793	12244272	G	0	0	A
6	BICF2P517444	12248027	A	0.5	0.8571	G
6	TIGRP2P77882_rs9236598	12260829	G	0.5	0.8571	A
6	TIGRP2P77887_rs8911366	12276934	G	0.5	0.8571	A
6	BICF2P58871	12286024	G	0.5	0.8571	A
6	TIGRP2P78040_rs8902718	12305364	0	0	0	G
6	TIGRP2P77996_rs8995127	12324662	0	0	0	G
6	BICF2P376084	12325873	A	0.5	0.9167	G
6	BICF2P871275	12334576	G	0	0	A

6	BICF2S2294384	12341209	G	0	0	A
6	BICF2P829738	12361209	A	0.5	0.1429	G
6	BICF2P778011	12373962	A	0.5	0.1429	G
6	BICF2P322746	12381491	T	0.5	0.8571	A
6	BICF2P788382	12388969	G	0.5	0.1429	A
6	BICF2P209679	12401714	G	0	0	A
6	BICF2P1101770	12414216	A	0	0	G
6	TIGRP2P78025_rs9119855	12420938	A	0	0	T
6	BICF2P1262152	12435974	A	0	0	G
6	BICF2P566250	12444605	A	0	0	T
6	BICF2P910338	12469495	C	0.5	0.1429	G
6	TIGRP2P78149_rs8673832	12478945	A	0	0	G
6	BICF2P447231	12500769	G	0	0	A
6	BICF2P631013	12514882	G	0	0	A
6	BICF2P666214	12533559	G	0	0	A
6	BICF2P1266835	12541308	G	NA	NA	A
6	BICF2P125690	12547760	A	0.5	0.8571	G
6	TIGRP2P78173_rs8572320	12560833	0	0	0	A
6	BICF2S23253168	12569758	A	0.5	0.1429	C
6	TIGRP2P78176_rs8869726	12588352	0	0	0	C
6	BICF2P219365	12594267	G	0	0	A
6	BICF2P1266769	12611810	0	0	0	G
6	BICF2P170102	12617301	G	0.5	0.5714	A
6	TIGRP2P78196_rs8743097	12633119	0	0	0	G
6	BICF2P843506	12640124	C	0	0.2857	A
6	BICF2P1037515	12642603	0	0	0	G
6	BICF2P1402144	12655925	A	0	0.2857	G
6	BICF2S23727650	12661827	G	0	0	A
6	BICF2P797313	12678880	A	0	0	G
6	TIGRP2P78223_rs9153087	12690825	A	0	0	G
6	BICF2P456521	12697693	A	0	0	G
6	TIGRP2P78235_rs9174406	12726085	A	0	0	C
6	TIGRP2P78246_rs8833699	12731789	A	0	0.2857	G
6	BICF2S23413415	12748394	G	0.5	0.5833	A
6	TIGRP2P78298_rs8808224	12765968	G	0.5	0.5833	A
6	BICF2P178829	12812822	A	0.5	0.5714	G
6	BICF2S22954901	12842410	A	0	0	G
6	BICF2P949227	12861376	A	0.5	0.5714	G
6	BICF2P515522	12875489	A	0.5	0.5714	C
6	BICF2P515523	12875650	A	0.5	0.5714	G
6	TIGRP2P78377_rs8768163	12886521	0	0	0	A
6	BICF2P382771	12895379	A	0.5	0.5714	C
6	BICF2P384551	12896490	A	0.5	0.5714	G
6	BICF2P1300041	12913285	0	0	0	C
6	BICF2P1172682	12944318	G	0.5	0.5714	A
6	BICF2P1317053	12948831	A	0.5	0.5714	T
6	BICF2P1211237	12949783	G	0.5	0.5714	A

6	BICF2P582779	12968736	T	0.5	0.5714	A
6	BICF2P139555	12982534	A	0.5	0.5714	T
6	BICF2P955019	12984297	A	0.5	0.5714	G
6	BICF2P327162	13003881	G	0	0.2857	A
6	BICF2S23913391	13008697	A	0	0.2857	G
6	BICF2S23229035	13027991	G	0	0.2857	A
6	BICF2P1139791	13040285	A	0	0	G
6	TIGRP2P78388_rs8665983	13047787	C	0	0	A
6	TIGRP2P78394_rs8651698	13058575	G	0.5	0.5714	A
6	BICF2P1235752	13058974	G	0.5	0.5714	A
6	TIGRP2P78405_rs9180048	13069692	A	0.5	0.5714	G
6	BICF2S23551027	13078232	A	0.5	0.5833	G
6	BICF2S23720891	13088563	G	1	0.7143	A
6	BICF2S23024984	13102051	A	0	0.2857	C
6	BICF2S22954260	13113605	A	0	0.2857	G
6	BICF2P1358119	13131171	G	0.5	0.4286	C
6	BICF2P627377	13134801	A	0	0	G
6	BICF2S2445184	13145341	A	0	0	G
6	BICF2P224466	13145950	A	0.5	0.1429	G
6	BICF2S23750152	13158504	G	0.5	0.1429	A
6	BICF2P1363434	13170881	A	0.5	0.1429	C
6	BICF2P935338	13182379	A	0.5	0.5714	G
6	BICF2S23611482	13200874	0	0	0	G
6	BICF2P1135692	13203556	G	0	0	A
6	TIGRP2P78514_rs9046184	13224329	G	0	0	A
6	BICF2P330828	13225503	G	0.5	0.5714	C
6	BICF2P1283462	13255513	A	0	0	G
6	BICF2P537170	13266839	G	0.5	0.4286	A
6	BICF2P1306200	13276027	A	0.5	0.4286	G
6	BICF2P993619	13288042	A	0.5	0.4286	G
6	BICF2P177672	13297697	G	0.5	0.4286	A
6	BICF2S23141804	13309946	0	0	0	G
6	TIGRP2P78530_rs8723919	13325171	A	0.5	0.4286	G
6	BICF2P937973	13334875	C	0	0.25	A
6	TIGRP2P78544_rs9149166	13351357	A	1	1	G
6	TIGRP2P78545_rs8931309	13354259	A	1	1	G
6	BICF2S23217689	13365691	A	0.5	0.1429	G
6	BICF2P1354149	13394775	G	1	1	A
6	BICF2P763992	13400396	G	0	0	A
6	BICF2P486572	13411816	A	0	0	C
6	BICF2P1446011	13424348	C	0	0	G
6	BICF2P23598	13433485	G	0.5	0.8571	A
6	BICF2P227603	13469250	A	0	0	G
6	TIGRP2P78565_rs8621872	13484086	G	0	0	A
6	TIGRP2P78566_rs8769768	13491997	A	0.5	0.1429	T
6	BICF2P1411957	13502749	G	0.5	0.1429	A
6	BICF2P73852	13519271	A	0	0	C

6	TIGRP2P78571_rs8731813	13532232	C	0	0	G
6	BICF2S23125774	13538677	C	0	0	A
6	BICF2P1388279	13543189	C	0.5	0.1429	A
6	BICF2P390830	13549952	G	0	0	A
6	BICF2S22938413	13564349	A	0	0	G
6	BICF2P488011	13572536	A	0.5	0.1429	C
6	BICF2S2295983	13585433	A	0	0	G
6	TIGRP2P78575_rs8688353	13596418	C	0.5	0.1429	A
6	BICF2P917176	13626385	G	0.5	0.1429	A
6	TIGRP2P78579_rs8727064	13631978	0	0	0	C
6	TIGRP2P78581_rs8600057	13640972	A	0.5	0.1429	C
6	BICF2P275833	13662603	0	0	0	C
6	BICF2S2312493	13676226	G	0.5	0.1429	A
6	BICF2S23538908	13689158	A	0	0	C
6	BICF2P1358427	13696788	A	0.5	0.4286	G
6	BICF2P65354	13711624	A	0.5	0.5714	G
6	BICF2S2398855	13722032	0	0	0	G
6	BICF2S2419516	13751048	G	0	0.2857	A
6	BICF2S23449355	13755478	G	0	0.2857	A
6	BICF2P1339750	13776065	A	0	0	G
6	BICF2S23232242	13782401	A	0	0.2857	G
6	BICF2P1425949	13784727	A	1	0.7143	G
6	BICF2P1425947	13784914	A	1	0.7143	G
6	BICF2P190746	13788078	A	0	0	C
6	TIGRP2P78625_rs9121072	13798235	A	0	0	G
6	BICF2P871594	13800609	A	0.5	0.8571	G
6	BICF2P779813	13819695	A	1	1	G
6	BICF2P775123	13825899	A	0	0	G
6	BICF2P997250	13849133	G	1	1	A
6	BICF2P372762	13865166	G	1	1	A
6	BICF2S22958632	13872926	A	0	0.2857	G
6	BICF2P916273	13886763	A	0	0.2857	G
6	BICF2P1248640	13899871	G	0	0.2857	A
6	BICF2P566973	13908168	A	0	0.2857	C
6	TIGRP2P78635_rs9034179	13916115	A	0.5	0.1429	G
6	BICF2P823254	13932909	G	0.5	0.4286	A
6	BICF2P613425	13944836	A	0.5	0.4286	G
6	BICF2P908268	13951377	G	0	0	A
6	BICF2P1378341	13968146	G	1	1	A
6	TIGRP2P78658_rs8655727	13975904	G	0	0.2857	A
6	BICF2P520673	13990813	G	0	0.2857	A
6	BICF2P34631	13997258	G	0	0.25	A
6	BICF2P822850	14010081	C	0	0.25	A
6	BICF2P612603	14027540	G	0	0	A
6	TIGRP2P78673_rs8617682	14036063	C	0	0.2857	G
6	BICF2P1114423	14051057	A	0	0.2857	C
6	BICF2P842351	14055365	C	0	0.2857	G

6	BICF2P692506	14066825	A	0	0	G
6	BICF2S23324965	14077648	C	0.5	0.1429	G
6	BICF2P1409446	14083990	A	1	1	G
6	BICF2S22961650	14092057	A	0.5	0.1429	G
6	BICF2P1272661	14104716	G	0.5	0.8571	A
6	BICF2S23438619	14113527	A	0.5	1	C
6	BICF2P968142	14127897	A	NA	0.6	C
6	TIGRP2P78701_rs8603486	14138393	C	0.5	0.5714	A
6	BICF2P1311300	14160633	G	0	0	A
6	BICF2P732609	14187655	G	0.5	0.5714	A
6	TIGRP2P78730_rs8752893	14195194	0	0	0	A
6	TIGRP2P78731_rs8758837	14204194	0	0	0	C
6	BICF2P343906	14218937	A	0	0	G
6	BICF2S23711465	14236518	G	0	0.2857	A
6	BICF2P813065	14246515	A	0	0	G
6	BICF2S23441251	14249612	A	0	0	C
6	BICF2P398305	14277877	A	0	0	C
6	TIGRP2P78765_rs9034457	14286533	C	0	0.25	A
6	BICF2S22946577	14299458	A	0	0.2857	C
6	BICF2S23617767	14307786	A	1	0.7143	G
6	BICF2P624547	14322156	A	1	1	G
6	BICF2S2326721	14332028	G	1	1	A
6	BICF2P882837	14341802	G	1	1	A
6	BICF2P43109	14359828	G	1	1	A
6	BICF2P180714	14365278	A	1	1	C
6	BICF2P953117	14377909	G	1	1	A
6	BICF2S23329890	14392873	G	1	0.7143	A
6	BICF2P402499	14408543	C	1	1	A
6	BICF2P741731	14409737	0	0	0	G
6	BICF2P232281	14429133	0	0	0	A
6	BICF2P769566	14434709	0	0	0	G
6	BICF2P182583	14448060	A	0	0.2857	C
6	BICF2P1393042	14471843	0	0	0	G
6	BICF2P1053047	14482272	0	0	0	A
6	BICF2P586580	14490107	G	0.5	0.5714	A
6	BICF2P224339	14517105	A	0	0	G
6	BICF2P620438	14556650	A	0.5	0.4286	G
6	TIGRP2P78780_rs8547863	14564011	A	0.5	0.5714	G
6	BICF2P821921	14569078	A	0.5	0.5714	G
6	BICF2P1278810	14573662	G	0.5	0.8571	A
6	BICF2P748383	14584984	G	0.5	0.1429	A
6	BICF2P912731	14589583	A	0.5	0.1429	G
6	BICF2S23112476	14593929	T	0	0.2857	A
6	BICF2S23058317	14614094	A	0	0.2857	C
6	BICF2P1350739	14644724	0	0	0	A
6	BICF2P1438024	14669820	0	0	0	A
6	BICF2S24512436	14674940	A	0	0	C

6	BICF2P528885	14691184	A	0	0.2857	G
6	TIGRP2P78807_rs8577635	14703365	0	0	0	G
6	BICF2P20799	14719405	A	0	0.2857	G
6	BICF2S23156482	14756898	A	0.5	0.5714	T
6	BICF2P248518	14766813	G	0.5	0.5714	A
6	BICF2P788996	14785359	A	0.5	0.5714	C
6	BICF2S23250501	14795185	G	0.5	0.5714	A
6	BICF2P879759	14807396	A	0	0.2857	G
6	TIGRP2P78825_rs9032978	14822434	C	0	0.2857	A
6	BICF2P699862	14845203	G	0	0	A
6	BICF2P334177	14859427	C	0	0	A
6	BICF2P1177973	14869714	A	0.5	0.1429	G
6	TIGRP2P78856_rs8917635	14882956	A	0	0	G
6	TIGRP2P78865_rs9094914	14897240	A	0	0	G
6	BICF2S22939059	14918381	C	0.5	0.1429	A
6	TIGRP2P78868_rs8956526	14932368	A	0	0	G
6	TIGRP2P78877_rs8929874	14940870	A	0.5	0.1429	G
6	BICF2P698143	14960716	0	0	0	G
6	BICF2P217086	14969381	A	0	0	G
6	BICF2S23146736	15004894	A	0	0	G
6	TIGRP2P78882_rs9085196	15015242	G	0	0.2857	A
6	BICF2P1215387	15021300	G	0	0	A
6	BICF2P1426801	15038691	A	0	0	G
6	BICF2P486512	15092668	0	0	0	A
6	BICF2P390512	15156935	0	0	0	G
6	TIGRP2P78967_rs8772007	15162337	C	0	0	G
6	BICF2P741065	15171742	G	0	0	A
6	BICF2P74125	15182037	A	0	0	G
6	BICF2P1313887	15219237	C	0	0	A
6	BICF2S23343413	15238364	C	0	0.2857	G
6	TIGRP2P78987_rs8874853	15253412	A	0	0	G
6	BICF2P1448305	15261699	G	0	0	A
6	TIGRP2P78990_rs8656455	15277922	A	0	0.2857	G
6	BICF2S22938732	15291714	G	0	0.2857	A
6	BICF2S23338576	15300104	C	0	0.25	A
6	TIGRP2P78995_rs8983531	15311085	A	0.5	0.5	T
6	TIGRP2P79002_rs9180140	15331159	G	0	0.2857	C
6	BICF2S24115642	15350474	G	0	0.2857	A
6	TIGRP2P79007_rs8677643	15353985	A	0	0.2857	G
6	BICF2P132251	15368708	G	1	1	A
6	BICF2P504795	15377854	A	0	0	C
6	BICF2P1452398	15391872	A	0	0	G
6	BICF2S23161640	15402224	A	0	0.2857	G
6	BICF2P896636	15413290	A	0.5	0.9167	G
6	TIGRP2P79042_rs8561557	15427813	A	0	0.2857	G
6	BICF2P1372971	15428663	C	0.5	0.5714	A
6	BICF2S23158048	15435399	A	0.5	0.8571	G

6	BICF2P1286441	15453047	A	0	0	G
6	BICF2P1461175	15455294	A	0.5	0.1429	G
6	BICF2P573209	15460624	A	0	0	G
6	BICF2P409215	15477123	A	0.5	0.8571	G
6	BICF2P438684	15480469	G	0.5	0.8571	A
6	BICF2P824836	15485195	G	0.5	0.8571	A
6	BICF2P1267860	15494779	A	0	0	G
6	BICF2S2351545	15511936	A	0.5	0.8571	G
6	BICF2S23028659	15522236	C	0.5	0.1429	G
6	BICF2P1206066	15533002	A	0.5	0.1429	G
6	BICF2S2365739	15539171	T	0.5	0.8571	A
6	BICF2P465503	15551144	C	0.5	0.8571	A
6	BICF2P637863	15561597	A	0	0	G
6	BICF2P1165724	15577093	G	0	0	A
6	TIGRP2P79085_rs8778454	15587070	C	1	1	A
6	BICF2P958223	15597619	A	0.5	0.1429	G
6	BICF2P1181620	15606307	G	0	0	A
6	TIGRP2P79105_rs8782366	15617093	G	0	0	C
6	BICF2S23432256	15640611	A	0	0	C
6	BICF2P969271	15658510	G	0	0.2857	A
6	BICF2P935743	15669194	A	0	0.2857	G
6	BICF2S2346973	15681792	A	0	0.2857	G
6	BICF2P1099159	15686940	A	0	0.2857	G
6	TIGRP2P79115_rs9069087	15698090	C	0	0.25	A
6	BICF2P1131020	15714331	A	0	0	G
6	BICF2P629390	15726088	A	0.5	0.1429	G
6	TIGRP2P79126_rs9138471	15742530	A	0	0.2857	G
6	BICF2P646509	15752646	G	0	0.2857	A
6	BICF2P1186091	15760049	G	0	0	C
6	BICF2S23223735	15775594	A	0	0.2857	G
6	BICF2S23212354	15778814	C	0	0.2857	A
6	BICF2P1087335	15794703	0	0	0	C
6	BICF2P1048320	15802787	A	0	0.2857	G
6	BICF2P510738	15815613	G	0	0.2857	A
6	BICF2P499590	15825019	G	0	0.25	A
6	BICF2P1407106	15845480	A	0	0	G
6	TIGRP2P79148_rs8818736	15849993	A	0.5	0.5714	G
6	BICF2P434712	15868136	A	0	0	G
6	BICF2P1231911	15870354	A	1	0.7143	G
6	BICF2P412093	15876975	G	1	0.7143	A
6	BICF2P1380167	15885731	A	0	0	G
6	BICF2P820521	15890484	A	1	0.7143	G
6	BICF2P1128886	15896957	G	1	0.7143	A
6	TIGRP2P79156_rs8692770	15904681	A	0	0	G
6	BICF2P387801	15925776	A	0	0	C
6	BICF2S23658510	15933385	G	0	0.2857	A
6	TIGRP2P79158_rs8542216	15945105	C	0	0	A

6	BICF2P1399403	15945348	C	0	0	A
6	BICF2P731828	15960639	A	0	0	G
6	BICF2P52793	15967410	A	0	0	G
6	BICF2P272134	15972865	A	0	0	C
6	BICF2P222137	15993923	G	0	0	A
6	BICF2P908334	16002276	G	0	0	A
6	BICF2P217831	16013304	C	0.5	0.1429	A
6	BICF2P1075489	16026571	G	0.5	0.4286	A
6	BICF2P691170	16037996	A	0.5	0.4286	G
6	BICF2P691171	16038448	A	0.5	0.4286	T
6	BICF2P1324968	16043741	C	0.5	0.4286	A
6	BICF2P752628	16065141	A	0.5	0.4286	G
6	BICF2P853453	16067551	G	0	0.2857	A
6	BICF2P628214	16089943	C	0.5	0.5714	A
6	BICF2P780760	16104556	G	0.5	0.5714	A
6	BICF2P199767	16124909	A	0.5	0.5714	G
6	BICF2S23152126	16136331	A	0	0.2857	G
6	TIGRP2P79230_rs8923886	16150143	A	0.5	0.5714	G
6	BICF2P595134	16158469	A	0	0	G
6	BICF2P344658	16174633	G	0	0	A
6	BICF2S23239556	16182604	A	1	1	G
6	TIGRP2P79282_rs9130099	16208598	A	0.5	0.1429	G
6	BICF2S23528492	16212993	A	0	0	G
6	BICF2S22940954	16215859	A	0	0	G
6	BICF2S23756581	16228446	A	0	0.2857	G
6	BICF2P1367032	16241862	A	0	0	G
6	BICF2P827576	16258930	G	0.5	0.4286	A
6	BICF2P165773	16272859	A	0	0.2857	G
6	TIGRP2P79322_rs8867438	16289391	G	0.5	0.4286	A
6	TIGRP2P79333_rs9029093	16301382	A	0	0.2857	G
6	BICF2P400239	16307791	0	0	0	A
6	BICF2P784872	16322416	G	0.5	0.5714	A
6	BICF2S2343695	16324133	A	0.5	0.4286	G
6	TIGRP2P79344_rs8800619	16330641	A	0.5	0.5714	G
6	BICF2P555431	16354148	A	0	0	G
6	BICF2S23317698	16365019	A	0	0	G
6	TIGRP2P79364_rs9150499	16378187	A	0	0	G
6	BICF2S23552717	16390250	A	0.5	0.5714	G
6	TIGRP2P79374_rs8668113	16403085	G	0	0	A
6	BICF2G630165635	16414778	G	0.5	0.4286	A
6	TIGRP2P79384_rs9045956	16431799	A	0	0.2857	G
6	TIGRP2P79385_rs8578665	16444942	A	0	0	G
6	TIGRP2P79391_rs9078044	16448688	G	1	0.7143	A
6	BICF2S23759798	16468281	A	0.5	0.5714	T
6	BICF2G630165643	16490083	A	0	0	G
6	BICF2P97044	16494557	0	0	0	C
6	BICF2S23512744	16505342	A	0.5	0.5714	G

6	BICF2S23763361	16525764	G	0	0	A
6	BICF2S23522180	16551518	A	0.5	0.5714	C
6	BICF2G630165645	16566020	A	0	0	C
6	BICF2G630165647	16613871	G	0.5	0.5714	A
6	BICF2G630165648	16628155	C	0	0	G
6	BICF2P929328	16655852	0	0	0	C
6	BICF2G630165650	16675347	G	0	0	C
6	BICF2S23160764	16691730	G	0.5	0.5714	A
6	BICF2S23226142	16708680	A	0	0	G
6	TIGRP2P79394_rs8798240	16713868	0	0	0	A
6	BICF2S22924787	16752333	G	0	0	A
6	BICF2S22943322	16756215	A	0	0	C
6	BICF2G630165656	16776444	A	0	0	G
6	BICF2S23910889	16799199	0	0	0	C
6	BICF2S2312649	16806629	A	0	0.2857	G
6	G380f50S157	16834845	0	0	0	G
6	BICF2P715806	16865215	0	0	0	G
6	BICF2G630165660	16875303	G	0	0	A
6	BICF2S23521560	16884945	A	0	0.2857	T
6	BICF2P1164778	16908855	0	0	0	G
6	BICF2G630165668	16934422	A	0	0	G
6	BICF2S22944684	16942840	A	0	0.2857	G
6	BICF2G630165669	16953787	G	0	0	A
6	BICF2S2301462	16964247	G	0	0	A
6	BICF2S2354357	16976104	A	0	0.2857	G
6	BICF2P1472860	17012814	0	0	0	A
6	BICF2G630165674	17020287	A	0	0	C
6	BICF2S23635374	17039225	G	0	0.2857	A
6	BICF2G630165678	17054152	A	0	0	G
6	BICF2P318931	17061368	A	0	0.2857	G
6	BICF2P1180369	17068771	A	0	0	C
6	BICF2G630165703	17079014	A	0	0	G
6	BICF2G630165720	17110138	G	0.5	0.4286	A
6	BICF2G630165728	17115716	C	0.5	0.4286	A
6	BICF2G630165737	17122209	G	0.5	0.1429	A
6	BICF2G630165741	17127356	A	0.5	0.5714	G
6	BICF2S23132116	17144593	A	0.5	0.1429	G
6	BICF2G630165762	17150874	C	0.5	0.1429	A
6	TIGRP2P79422_rs9090197	17158066	A	0.5	0.4167	G
6	BICF2G630165780	17170470	A	0.5	0.1429	C
6	BICF2G630165797	17191981	G	0	0	A
6	BICF2G630165801	17193336	A	0.5	0.1429	G
6	BICF2G630165819	17205014	A	0	0	G
6	TIGRP2P79424_rs8626030	17220142	0	0	0	A
6	BICF2G630165821	17228477	C	0	0	G
6	BICF2S23659882	17261356	G	0	0	A
6	BICF2G630165837	17277156	G	0.5	0.3571	A

6	BICF2G630165856	17290324	0	0	0	A
6	BICF2G630165862	17297859	0	0	0	G
6	BICF2G630165886	17312223	C	0	0	G
6	TIGRP2P79437_rs8609967	17319063	G	0.5	0.3571	A
6	BICF2P919938	17340435	A	0	0	C
6	BICF2P312896	17347072	A	0	0	G
6	BICF2P901872	17357797	G	0	0	A
6	BICF2P562790	17367263	A	0.5	0.3571	G
6	BICF2S22956235	17380274	G	0	0	A
6	BICF2P265325	17390573	0	0	0	A
6	BICF2P674111	17403253	0	0	0	G
6	TIGRP2P79459_rs8564038	17422982	0	0	0	C
6	BICF2S2454777	17474264	0	0	0	G
6	BICF2P941394	17484263	A	0	0	G
6	BICF2G630165901	17492311	C	0	0	G
6	TIGRP2P79467_rs8877073	17516247	0	0	0	G
6	BICF2G630165903	17539533	G	0	0.2143	A
6	TIGRP2P79473_rs8518325	17551545	A	0	0.2143	G
6	BICF2P912757	17555554	G	0	0.2143	A
6	BICF2S2313369	17574911	0	0	0	G
6	BICF2G630165911	17587132	A	0	0.2143	G
6	BICF2P564916	17612433	0	0	0	C
6	BICF2P545657	17624764	0	0	0	G
6	BICF2P511818	17648460	0	0	0	G
6	TIGRP2P79484_rs9117238	17665587	0	0	0	C
6	BICF2S22916016	17679529	G	0	0	A
6	TIGRP2P79493_rs9197881	17687738	A	0	0	G
6	TIGRP2P79495_rs9237040	17706161	A	0	0	G
6	BICF2P942186	17719675	A	0.5	0.4286	G
6	BICF2S23443954	17730208	G	0	0	A
6	BICF2S2413592	17733307	0	0	0	C
6	BICF2P1347596	17744160	A	0	0	G
6	TIGRP2P79505_rs8813150	17746922	A	0	0	G
6	BICF2G630165933	17762563	G	0	0.2143	A
6	BICF2S2358041	17776232	0	0	0	G
6	BICF2P527851	17797306	0	0	0	G
6	BICF2S2377546	17807268	0	0	0	G
6	BICF2P985283	17845902	G	0	0	A
6	BICF2G630165939	17848411	A	0	0.2143	G
6	BICF2G630165945	17864553	G	1	0.7857	A
6	BICF2P674567	17874297	A	0	0	G
6	BICF2G630165981	17894209	G	1	0.7857	A
6	TIGRP2P79523_rs8785657	17906042	0	0	0	C
6	BICF2P199049	17923386	G	0	0	A
6	BICF2G630165989	17930754	A	0	0	G
6	BICF2G630165999	17943765	A	0	0	G
6	BICF2G630166002	17951795	C	0	0	A

6	BICF2G630166016	17969354	G	0	0	C
6	BICF2G630166022	17978221	A	0	0	G
6	BICF2G630166034	17989651	A	0	0	G
6	BICF2G630166047	18006680	G	0	0	C
6	BICF2P1044897	18009325	A	0	0	G
6	BICF2G630166051	18029837	G	0	0.2143	A
6	BICF2G630166053	18040666	G	0	0.2143	A
6	BICF2S23632271	18053959	G	0	0	A
6	BICF2G630166056	18057377	G	0	0.2143	A
6	BICF2G630166058	18069332	A	0	0	G
6	BICF2S22957410	18078772	G	0	0	C
6	BICF2G630166094	18100384	G	0	0.2143	A
6	BICF2G630166095	18100509	G	0	0	A
6	BICF2G630166121	18108213	A	0	0	G
6	BICF2G630166128	18118076	A	0	0	G
6	BICF2G630166135	18128222	A	0	0	G
6	BICF2P107821	18140217	0	0	0	C
6	TIGRP2P79533_rs8725946	18166461	0	0	0	C
6	BICF2P916100	18171869	0	0	0	A
6	BICF2G630166141	18191899	A	1	1	G
6	BICF2G630166142	18193815	C	1	1	A
6	BICF2G630166164	18210957	G	1	1	A
6	BICF2G630166174	18214638	G	1	0.7857	A
6	BICF2G630166178	18223109	G	1	1	A
6	BICF2G630166187	18234420	A	1	1	G
6	BICF2G630166193	18240385	C	1	1	A
6	BICF2G630166209	18255445	G	0	0.2143	A
6	BICF2G630166227	18264435	G	0	0	A
6	BICF2P730611	18280695	A	0.5	0.2143	G
6	BICF2S23548789	18292481	A	0	0	G
6	BICF2G630166285	18296716	G	0.5	0.2143	A
6	BICF2G630166298	18308388	A	0	0	G
6	BICF2G630166303	18323456	A	0	0	G
6	BICF2P775622	18339882	G	0.5	0.5714	A
6	BICF2G630166315	18351358	A	0.5	0.2143	G
6	BICF2P641294	18360511	A	0	0	G
6	BICF2G630166353	18375060	A	0	0	G
6	BICF2G630166366	18387605	G	0.5	0.2143	A
6	BICF2G630166370	18396595	G	0.5	0.2143	A
6	BICF2G630166371	18399156	C	0.5	0.2143	G
6	BICF2G630166376	18406220	A	0.5	0.2143	G
6	BICF2G630166380	18413153	A	0.5	0.1667	G
6	TIGRP2P79555_rs8757162	18424319	C	1	0.7857	A
6	TIGRP2P79558_rs8831227	18436038	G	0.5	0.2143	C
6	BICF2P434856	18454226	G	0.5	0.5714	A
6	BICF2P693564	18466722	A	0	0	G
6	BICF2S23330041	18471383	A	0.5	0.2143	G

6	BICF2S23421211	18481025	A	0.5	0.2143	G
6	TIGRP2P79578_rs8553201	18498495	A	0	0	G
6	BICF2P966934	18503927	G	1	0.7857	A
6	BICF2G630166456	18517258	A	0	0	C
6	BICF2G630166462	18526820	A	0	0	G
6	BICF2G630166482	18540236	A	0.5	0.2143	T
6	BICF2G630166509	18553724	A	0.5	0.2143	G
6	BICF2G630166528	18561175	A	0.5	0.4286	G
6	BICF2G630166536	18576119	G	0.5	0.4286	A
6	BICF2G630166569	18590229	A	0	0.2143	G
6	BICF2P497579	18601292	A	0.5	0.2143	G
6	BICF2G630726239	18617019	A	0.5	0.7857	C
6	BICF2P1090035	18620404	G	0.5	0.7857	A
6	BICF2P1284279	18641128	A	0	0.2143	G
6	BICF2P549414	18646599	G	1	1	A
6	BICF2P158390	18657214	A	0	0.2143	G
6	BICF2S23011665	18673032	A	0.5	0.5714	C
6	BICF2S23217442	18682251	T	0	0	A
6	BICF2S23519373	18692017	G	0.5	0.4286	A
6	BICF2P1318132	18701219	A	0	0	G
6	BICF2P31296	18720516	A	0	0	G
6	BICF2P31297	18721121	A	0	0	G
6	BICF2P31299	18722749	A	1	1	G
6	BICF2S23348453	18738136	A	0.5	0.5833	G
6	BICF2P882584	18749183	A	0.5	0.5714	G
6	BICF2S2291996	18760582	A	0.5	0.5714	G
6	BICF2S23213167	18772702	G	0	0.2143	C
6	BICF2P942266	18788832	G	1	0.7857	A
6	BICF2P526875	18800881	A	0.5	0.2143	G
6	BICF2S2346466	18809942	A	0.5	0.5714	G
6	BICF2P490505	18825154	A	0.5	0.5714	G
6	BICF2S2348808	18834342	A	0.5	0.5714	G
6	BICF2P758806	18834832	A	0	0	G
6	BICF2S2348668	18837854	A	0.5	0.5714	G
6	BICF2P516901	18850429	A	0.5	0.2143	G
6	BICF2P183784	18863322	A	0.5	0.2143	G
6	BICF2P603747	18865639	A	0.5	0.2143	G
6	BICF2P1117531	18878983	G	0.5	0.2143	A
6	BICF2S23320553	18887178	0	0	0	G
6	BICF2P1470616	18901385	A	0.5	0.7857	C
6	BICF2P636274	18910212	A	0.5	0.7857	G
6	BICF2P1258050	18926358	G	0.5	0.7857	A
6	BICF2P641063	18929706	C	0.5	0.2143	A
6	BICF2S23445502	18951433	A	1	1	G
6	BICF2P215635	18958337	G	1	1	A
6	BICF2S23746091	18963790	G	0.5	0.5714	A
6	BICF2P1291928	18997571	G	0	0.2143	A

6	BICF2S23027519	19008473	A	0	0	G
6	BICF2P534126	19019631	A	0.5	0.7857	C
6	BICF2P976752	19021368	A	0	0.2143	G
6	BICF2P442186	19029758	A	0	0.2143	G
6	BICF2P1261613	19032658	G	0	0.2143	A
6	BICF2P806453	19044420	A	0	0.2143	G
6	BICF2P306800	19058985	T	0.5	0.7857	A
6	BICF2P516779	19070909	A	0.5	0.7857	G
6	BICF2P1421595	19093841	A	0.5	0.7857	G
6	BICF2G630726231	19105822	G	0.5	0.7857	A
6	BICF2G630726209	19123343	G	0.5	0.7857	A
6	BICF2G630726206	19130145	G	0.5	0.7857	A
6	BICF2P1238222	19140294	A	0.5	0.2143	C
6	BICF2P1256373	19148366	G	0.5	0.2143	A
6	BICF2S23726297	19160610	G	0	0	A
6	BICF2G630726201	19178071	A	0	0.2143	G
6	BICF2P720754	19184163	G	0	0	A
6	BICF2P385680	19194566	G	0	0	A
6	BICF2P150288	19207177	0	0	0	C
6	BICF2P1176185	19224356	A	0	0.2143	G
6	BICF2G630726184	19237408	G	0	0.2143	A
6	BICF2G630726183	19240502	A	0	0.2143	G
6	BICF2G630726171	19255556	A	0.5	0.7857	G
6	BICF2G630726170	19255686	0	0	0	G
6	BICF2P643323	19273069	G	0	0	A
6	BICF2G630726153	19274049	G	0	0	A
6	BICF2P453903	19286116	A	0	0	G
6	BICF2P257899	19297809	C	0	0.2143	A
6	BICF2G630726138	19311714	G	0.5	0.2143	A
6	BICF2P1434901	19329301	A	0.5	0.4286	G
6	BICF2P529830	19334460	G	0.5	0.5714	A
6	BICF2P1442414	19349108	A	0.5	0.2143	T
6	BICF2P807878	19355903	A	0	0	G
6	BICF2P321827	19373311	G	0	0	A
6	BICF2P386169	19382129	C	0	0.2143	A
6	BICF2S2337499	19392703	G	0	0	A
6	BICF2S23729483	19404264	G	0	0	A
6	BICF2P1264689	19439834	A	0	0	G
6	BICF2P1171217	19449922	A	0	0	G
6	BICF2P1270390	19461924	G	1	1	A
6	BICF2S2315404	19480498	G	0	0	A
6	BICF2P310535	19484630	0	0	0	C
6	BICF2P398199	19503467	A	0	0	C
6	BICF2S23628785	19512803	G	0.5	0.2143	A
6	BICF2P699169	19517085	G	0	0	C
6	BICF2P919791	19528152	C	0	0	A
6	BICF2P1241073	19543427	G	0.5	0.2143	A

6	BICF2S23030024	19555140	A	0	0	G
6	BICF2P255639	19559599	A	0	0.2143	C
6	BICF2P583173	19573929	A	0	0	G
6	BICF2P1215116	19588207	A	NA	0.7857	C
6	BICF2P195090	19598328	A	0	0	G
6	BICF2P692076	19608012	A	0	0	G
6	BICF2P1210099	19613971	A	0	0	C
6	BICF2S23146375	19626595	G	0.5	0.2143	A
6	BICF2S23639066	19635916	A	0	0	C
6	BICF2S23434277	19661337	C	1	1	G
6	BICF2S23552358	19667892	G	NA	1	A
6	BICF2P748460	19681602	A	1	1	G
6	BICF2P1168691	19691978	A	0	0	G
6	BICF2P1318500	19702295	G	0	0	A
6	BICF2S22931981	19711056	G	0	0	A
6	BICF2P977673	19749873	T	0	0	A
6	BICF2S23437217	19767947	A	1	1	G
6	TIGRP2P79914_rs9050518	19768501	A	0.5	0.5714	G
6	BICF2P508954	19784746	A	0	0	G
6	BICF2P811960	19792153	A	0	0	G
6	BICF2P811961	19792249	C	0	0	A
6	BICF2P452172	19804263	0	0	0	C
6	BICF2P149504	19837019	G	0.5	0.4286	C
6	BICF2P8975	19850034	0	0	0	T
6	BICF2S23445794	19905378	A	0.5	0.2857	C
6	BICF2P284371	19909554	A	0	0	G
6	BICF2P208890	19934438	A	0	0	G
6	BICF2P1464259	19952437	A	0.5	0.2143	G
6	BICF2P171851	19954823	0	0	0	C
6	BICF2P938684	19964751	0	0	0	A
6	BICF2P586763	19983637	G	0.5	0.7143	A
6	BICF2P640167	19989766	G	0.5	0.5	A
6	BICF2P1022622	20009862	T	0.5	0.2857	A
6	BICF2P843371	20010124	A	0.5	0.2857	G
6	BICF2P538740	20021592	A	0	0	G
6	BICF2P988051	20060523	0	0	0	G
6	BICF2P888273	20070983	0	0	0	C
6	BICF2P23991	20090372	A	0.5	0.7143	G
6	BICF2P920657	20096792	A	0.5	0.2857	G
6	BICF2P276290	20108289	G	0.5	0.4286	A
6	BICF2P1154158	20118516	A	0	0	G
6	BICF2P940453	20126054	G	0	0	A
6	BICF2P576269	20132645	G	0.5	0.7143	A
6	BICF2P1088608	20141344	0	0	0	G
6	TIGRP2P79685_rs8677021	20152777	A	0.5	0.2143	C
6	TIGRP2P79684_rs8926829	20168013	A	0	0.2857	G
6	BICF2P1126805	20174245	A	0.5	0.2857	G

6	BICF2P316204	20192754	A	0	0	G
6	BICF2P404373	20202557	G	0	0.2857	A
6	BICF2P1024392	20215306	0	0	0	G
6	BICF2S23612366	20223548	A	0	0.2857	C
6	BICF2P955749	20228747	0	0	0	C
6	TIGRP2P79670_rs8580717	20249732	C	0	0	A
6	BICF2P676753	20254743	G	0	0	A
6	TIGRP2P79669_rs8732764	20282836	0	0	0	G
6	BICF2P1281363	20308928	G	0	0	A
6	BICF2P1281360	20309555	G	0	0	A
6	BICF2P1420142	20341715	A	0.5	0.2143	G
6	BICF2P469015	20354766	A	0.5	0.2143	G
6	TIGRP2P79666_rs8902600	20383136	C	0.5	0.2143	G
6	BICF2P1410895	20399776	A	0	0	C
6	TIGRP2P79656_rs8723233	20403659	0	0	0	G
6	BICF2S23764035	20427246	A	0	0	G
6	TIGRP2P79654_rs9051450	20435662	0	0	0	C
6	BICF2P1187572	20457244	0	0	0	C
6	BICF2P173328	20462398	0	0	0	C
6	BICF2S23141776	20482746	A	1	0.5	G
6	BICF2P682790	20518153	G	0	0.2857	A
6	BICF2S23418945	20522832	T	0	0.5	A
6	BICF2S23125853	20538663	G	0	0.2857	A
6	BICF2P279522	20544026	G	0	0	A
6	TIGRP2P79641_rs9161090	20556407	0	0	0	G
6	BICF2P488953	20562877	A	0	0.2857	G
6	BICF2S245493	20590990	G	0	0	A
6	BICF2P1214519	20600902	G	1	0.7857	A
6	BICF2P628556	20608801	A	1	0.7857	G
6	BICF2P1122349	20628577	A	0	0.2857	G
6	BICF2P660509	20641069	G	0	0.2857	A
6	BICF2P638782	20649240	A	0	0.2143	G
6	BICF2P959425	20654045	G	0	0.2857	A
6	BICF2P512053	20690326	A	0	0	G
6	BICF2P675080	20700754	A	1	0.5	G
6	BICF2P1271206	20713644	A	0	0.2857	G
6	TIGRP2P79612_rs8876379	20729216	G	0	0.2857	A
6	BICF2P1471715	20741022	0	0	0	A
6	BICF2P990435	20748399	G	0	0.2857	A
6	BICF2P116727	20757772	G	0.5	0.7143	A
6	BICF2P777060	20770313	A	0.5	0.5714	G
6	BICF2P1127288	20790620	A	0	0	G
6	BICF2P986665	20793706	G	0.5	0.2857	A
6	BICF2S2306548	20809704	A	0	0.2857	G
6	TIGRP2P79945_rs8635789	20823419	A	0.5	0.2857	G
6	BICF2S23446482	20827347	G	0	0.2143	A
6	BICF2S23110188	20838176	G	0	0.2143	A

6	TIGRP2P79966_rs8503217	20850614	G	0.5	0.5	A
6	BICF2S2325932	20925889	C	0	0.2857	A
6	BICF2P658082	20934103	A	0	0	G
6	BICF2S23253491	20943640	A	0.5	0.4286	G
6	BICF2P54453	20955191	0	0	0	G
6	G383f22S118	20988801	G	0.5	0.4286	C
6	BICF2S22959118	21012142	A	0.5	0.4286	T
6	BICF2P1092577	21025350	G	0	0	A
6	BICF2S2322474	21032372	A	0.5	0.4286	G
6	BICF2P1070150	21034377	G	0.5	0.4286	A
6	BICF2P765445	21060921	0	0	0	G
6	BICF2P856602	21074771	G	0.5	0.5	A
6	BICF2P1055311	21084056	A	0.5	0.2143	G
6	BICF2P610704	21092861	A	0.5	0.2143	G
6	TIGRP2P80010_rs9224131	21110584	G	0	0.1667	A
6	BICF2P1185671	21119373	A	0	0.5	G
6	BICF2P342724	21136662	0	0	0	A
6	BICF2P231529	21155078	G	0.5	0.7857	A
6	TIGRP2P80029_rs8863419	21169957	G	0.5	0.5	A
6	BICF2P66265	21179934	G	0.5	0.5	A
6	BICF2P1456652	21192353	0	0	0	G
6	BICF2P889408	21210293	A	0	0	G
6	BICF2P905974	21234939	C	0.5	0.75	A
6	BICF2S23058504	21252831	A	0	0	G
6	BICF2S23411791	21266405	G	0.5	0.5	A
6	BICF2P793039	21310983	G	0	0	A
6	BICF2S2455062	21330513	C	0	0	A
6	BICF2S23056199	21353759	G	0	0	A
6	BICF2P439484	21358819	0	0	0	G
6	BICF2P408801	21368837	A	0	0	G
6	BICF2P816669	21383608	C	0	0	A
6	BICF2P1092907	21402143	A	0	0	G
6	BICF2S22953650	21422688	A	0	0	G
6	BICF2P1397621	21430990	G	0	0.2143	A
6	BICF2P510833	21438987	A	1	0.7857	G
6	TIGRP2P80097_rs9061518	21461664	G	0	0.2143	A
6	BICF2P1379427	21471820	0	0	0	G
6	BICF2S23521169	21475826	G	0	0.2857	A
6	BICF2S23059526	21485516	A	0.5	0.2143	G
6	BICF2P356826	21501847	A	0.5	0.5	C
6	BICF2P1335555	21510867	A	0.5	0.2857	G
6	TIGRP2P80110_rs8777403	21531938	0	0	0	A
6	BICF2P396913	21542146	0	0	0	C
6	BICF2S23656452	21561096	C	0	0	A
6	BICF2S23453040	21564761	A	0.5	0.4286	G
6	TIGRP2P80111_rs9084598	21583907	G	0.5	0.4286	A
6	TIGRP2P80114_rs8633526	21588227	A	0.5	0.5714	G

6	TIGRP2P80117_rs8818830	21615290	G	0.5	0.4286	A
6	BICF2P1304979	21625520	A	0.5	0.5714	C
6	TIGRP2P80136_rs8875559	21640693	A	0.5	0.5714	G
6	TIGRP2P80140_rs8631555	21646978	G	0.5	0.5714	A
6	BICF2P1014709	21658584	0	0	0	C
6	BICF2P157697	21670314	A	0.5	0.4286	G
6	TIGRP2P80144_rs9180125	21680750	A	0.5	0.4286	G
6	BICF2S2411183	21691864	A	1	1	G
6	BICF2P225105	21708159	G	0	0.2857	A
6	BICF2P960220	21717456	G	0	0	A
6	TIGRP2P80169_rs8878040	21724010	A	0	0	T
6	BICF2P915764	21737492	C	0	0	A
6	TIGRP2P80181_rs9233714	21757532	A	0	0	T
6	BICF2P730207	21770869	A	0	0	G
6	BICF2P827446	21781937	T	0.5	0.5	A
6	BICF2P1261365	21800213	G	0	0.5	A
6	BICF2P656970	21805666	G	0	0.2143	A
6	BICF2S22945285	21816463	G	0	0.5	A
6	BICF2P327789	21835681	A	0	0	G
6	BICF2P1434054	21844179	A	0.5	0.5	C
6	BICF2P963856	21857090	A	0.5	0.7857	G
6	BICF2P1056692	21868347	A	1	0.7143	G
6	BICF2P1076583	21878549	A	0.5	0.5714	G
6	BICF2P843137	21892665	C	0.5	0.5	A
6	BICF2P468591	21904956	G	0	0	A
6	BICF2P1151032	21913612	A	0	0.2857	G
6	BICF2P591588	21920236	C	0	0.2857	G
6	BICF2P224894	21931008	G	0.5	0.5	A
6	BICF2P1146822	21942766	T	0	0.2857	A
6	BICF2P288167	21959711	A	0	0.5	G
6	BICF2P265335	21973978	0	0	0	G
6	BICF2P177560	21985339	A	0	0.2143	G
6	TIGRP2P80271_rs8840639	21989598	A	1	0.5	G
6	BICF2P1367451	21994033	A	0	0	G
6	TIGRP2P80274_rs8849509	21999319	A	0	0	C
6	BICF2P1252205	22004644	A	1	0.5	G
6	BICF2P567522	22019994	A	0	0	G
6	BICF2P822911	22042815	A	0.5	0.7857	G
6	BICF2P1256117	22054070	A	0	0.5	G
6	BICF2P1198968	22066041	G	1	1	A
6	BICF2P127036	22068945	G	0	0.5	A
6	BICF2S23511117	22089737	G	1	0.5	A
6	BICF2P1508833	22100906	A	0	0	C
6	BICF2P583511	22105558	A	0	0	G
6	BICF2P827773	22121152	A	0	0	C
6	TIGRP2P80294_rs9255675	22128687	C	1	0.5	A
6	BICF2P170182	22143878	A	0.5	0.2857	G

6	BICF2S22943160	22152105	A	0.5	0.2857	G
6	BICF2P287510	22166876	A	0.5	0.5714	C
6	BICF2P1313485	22173911	A	0	0.5	G
6	BICF2P936056	22184017	A	0	0.5	G
6	BICF2S23240806	22208975	G	0	0	A
6	BICF2P182491	22227637	G	0.5	0.2143	A
6	BICF2P1340920	22251314	A	0	0	G
6	BICF2S23615897	22255482	A	0.5	0.7857	G
6	BICF2P1196538	22272366	C	0.5	0.7143	A
6	BICF2S22940669	22274957	A	0	0.1667	G
6	BICF2S23258724	22284618	A	0	0	G
6	BICF2P1217429	22302942	G	0	0.2143	A
6	BICF2P769637	22313239	G	0	0.1667	A
6	BICF2P1415494	22324548	A	0	0.5	G
6	BICF2S23339930	22335735	C	0.5	0.2143	G
6	BICF2P674727	22347967	G	0.5	0.7143	A
6	BICF2S2312894	22376644	A	1	0.7143	G
6	BICF2S23441346	22383702	G	0	0	A
6	BICF2P1231173	22396069	0	0	0	G
6	BICF2P1005607	22402279	A	0.5	0.5	G
6	BICF2S22929532	22413594	A	0.5	0.5	G
6	TIGRP2P80429_rs8779621	22435319	G	0.5	0.7143	A
6	BICF2P447760	22438521	A	0.5	0.7143	G
6	BICF2S23713344	22456706	0	0	0	G
6	BICF2S23710871	22460298	G	0	0	A
6	BICF2P404773	22471857	G	0	0	A
6	BICF2S2297676	22488429	G	0.5	0.5	A
6	BICF2S22915561	22511728	A	0.5	0.2857	G
6	BICF2P961527	22521067	0	0	0	G
6	TIGRP2P80452_rs8956890	22561295	G	0.5	0.5714	A
6	BICF2P1369027	22563604	0	0	0	C
6	BICF2P1349962	22575317	A	1	0.5	G
6	BICF2P229142	22586928	G	0.5	0.7143	A
6	BICF2P1208382	22602963	A	0	0	G
6	BICF2P430934	22619634	T	0	0.2857	A
6	TIGRP2P80478_rs8892064	22625444	A	0	0.1667	G
6	BICF2S2377226	22647516	C	0	0	A
6	BICF2P730879	22663408	T	0	0.5	A
6	TIGRP2P80500_rs8640278	22671288	C	0	0	G
6	TIGRP2P80505_rs8694163	22683048	G	0	0	A
6	BICF2P596603	22688546	A	0	0.2857	G
6	BICF2P1400688	22693213	A	0	0	G
6	BICF2P221871	22708628	A	1	0.5	G
6	BICF2P778479	22712870	A	1	0.5	C
6	BICF2P755544	22730430	A	1	0.5	G
6	BICF2P238948	22737598	C	1	0.5	A
6	BICF2P933473	22751428	A	1	0.5	C

6	BICF2P626198	22767869	G	0.5	0.2857	A
6	BICF2S23748042	22782917	G	0	0	A
6	BICF2P1359282	22796089	G	1	0.7143	C
6	BICF2P855300	22807980	A	0.5	0.5	G
6	BICF2S2442357	22815920	A	0.5	0.7857	G
6	BICF2P1147102	22817054	G	0.5	0.7857	A
6	BICF2P867806	22838199	G	0	0.25	A
6	BICF2S22955543	22844453	A	0.5	0.5714	G
6	BICF2P1165582	22861769	T	0	0.2857	A
6	BICF2P832452	22870775	A	0	0.2857	G
6	TIGRP2P80570_rs9112951	22877954	A	0.5	0.5	T
6	BICF2S23312940	22885510	A	0.5	0.4286	G
6	BICF2P1036894	22891831	C	0.5	0.5	G
6	BICF2P717472	22898363	A	0	0	G
6	BICF2P519620	22916578	A	0	0.2857	G
6	BICF2P1147939	22919033	A	1	1	G
6	BICF2S2349473	22931534	G	1	0.7857	A
6	BICF2P1277678	22946613	G	1	1	A
6	BICF2S2308419	22952282	A	0	0	G
6	BICF2P398154	22955113	G	0	0.2857	A
6	BICF2P1402372	22965757	T	0	0.2857	A
6	BICF2P847578	22998502	G	0	0	A
6	BICF2S23327134	23028375	A	0.5	0.1667	C
6	BICF2S23023203	23029439	A	0.5	0.7857	C
6	BICF2S23730116	23039445	G	1	1	A
6	BICF2P88757	23048450	G	0.5	0.7857	A
6	BICF2P177924	23050430	G	1	1	A
6	BICF2P599836	23052925	A	0.5	0.7857	G
6	BICF2P1040977	23062364	C	0.5	0.5714	A
6	BICF2P1008553	23067362	A	0.5	0.2143	G
6	BICF2P155392	23078751	G	0.5	0.5714	A
6	BICF2P261288	23088441	G	0.5	0.5714	A
6	BICF2S23717478	23090325	T	0	0.2143	A
6	BICF2P713801	23096713	A	0.5	0.6	C
6	BICF2P1186603	23104205	C	0.5	0.7857	A
6	BICF2P337494	23115373	G	0.5	0.2143	A
6	BICF2S22920764	23136875	0	0	0	G
6	BICF2P1385199	23140368	G	0	0.5	A
6	BICF2P92193	23151484	G	0	0	A
6	BICF2P272963	23160353	C	0	0	A
6	BICF2P1383149	23165771	A	0	0	G
6	BICF2P807460	23177930	A	0	0	G
6	BICF2P895074	23184965	G	0	0	A
6	BICF2P430603	23206728	A	0.5	0.2857	G
6	BICF2P248269	23207533	G	0.5	0.2857	A
6	BICF2P164417	23220828	0	0	0	C
6	BICF2P544911	23239069	0	0	0	T

6	BICF2S23120661	23247787	A	1	0.7143	G
6	BICF2P564616	23257649	G	0	0	A
6	BICF2P767435	23276974	A	1	0.5	G
6	BICF2S232109	23289906	A	0.5	0.2857	C
6	BICF2S2455844	23294775	T	0	0	A
6	BICF2P1009202	23299427	A	0.5	0.2857	G
6	BICF2P960195	23303915	G	0	0	A
6	BICF2P799684	23318567	A	0.5	0.5	G
6	BICF2P487163	23321575	A	0.5	0.5	G
6	BICF2P735904	23326052	G	0	0	A
6	BICF2P453669	23340000	A	0.5	0.2857	G
6	BICF2P247749	23354453	0	0	0	C
6	BICF2S23730820	23356530	A	0.5	0.7143	G
6	BICF2P1171190	23359520	A	0	0	G
6	BICF2S23120491	23367194	A	0.5	0.7143	C
6	BICF2S23530716	23373917	A	0	0	G
6	BICF2P699604	23379943	A	0	0	G
6	BICF2P108780	23388976	A	1	0.7857	C
6	BICF2P766339	23392158	A	0	0.2143	C
6	BICF2P220794	23405694	G	1	0.7857	A
6	BICF2P762211	23416271	A	0.5	0.5714	C
6	BICF2P248923	23429457	A	0.5	0.4286	G
6	BICF2S23131259	23437046	G	0.5	0.4286	A
6	BICF2P989945	23452942	0	0	0	C
6	BICF2S2325707	23463622	A	0	0	G
6	BICF2S23654624	23480507	C	0	0.2143	A
6	BICF2P499606	23492059	A	0	0	C
6	BICF2S23133527	23498861	G	0	0	A
6	BICF2S23439352	23510575	G	0.5	0.2143	A
6	BICF2S23322775	23526681	A	0	0	C
6	BICF2P112458	23542574	A	0.5	0.2143	G
6	BICF2P205612	23549300	C	0	0	A
6	BICF2P329014	23555121	A	0	0	G
6	BICF2S2316242	23564913	G	NA	0	A
6	BICF2P1056304	23578055	A	0	0.5	C
6	BICF2S23135724	23604145	T	0.5	0.2857	A
6	BICF2P393069	23629285	A	0	0	G
6	BICF2S23010122	23633365	A	0	0	G
6	TIGRP2P80587_rs9001499	23655061	0	0	0	A
6	BICF2S22959051	23673392	G	0.5	0.2857	A
6	BICF2S23718630	23680809	A	1	0.5	G
6	BICF2P1000422	23691597	A	0.5	0.2857	G
6	BICF2P268704	23702001	A	0	0	G
6	BICF2S23319061	23722084	G	0.5	0.4286	A
6	BICF2P1182130	23730566	G	0.5	0.4286	A
6	BICF2S23531949	23736871	A	0.5	0.5	G
6	BICF2P492430	23745927	G	1	0.5	A

6	BICF2P278457	23750398	G	0.5	0.2857	A
6	BICF2P910004	23775090	G	1	0.7143	A
6	BICF2P1264202	23788358	A	1	0.5	G
6	BICF2S24414619	23798643	G	1	0.5	A
6	BICF2P636189	23815720	A	0	0.25	G
6	BICF2P162837	23824748	C	0	0.2857	A
6	BICF2P393480	23837507	G	0.5	0.7143	A
6	BICF2P492438	23843445	G	0	0.2143	A
6	BICF2S22950355	23856872	T	0.5	0.2857	A
6	BICF2S23162537	23865456	G	0	0	A
6	BICF2S23019381	23880628	G	0.5	0.4286	A
6	BICF2S23617522	23887338	A	0	0	G
6	BICF2P218732	23903407	A	0.5	0.2143	G
6	BICF2P1355245	23918117	A	0.5	0.2143	G
6	BICF2P1127709	23925585	A	0.5	0.2143	C
6	BICF2P1331561	23936306	0	0	0	G
6	BICF2P1062599	23948377	0	0	0	C
6	BICF2S23112533	23959474	A	0.5	0.2143	G
6	BICF2P176655	23979591	A	0.5	0.2143	G
6	BICF2P176652	23979786	A	0	0	G
6	BICF2S2369943	23997058	A	0	0	G
6	BICF2P1035894	24007929	A	0	0	G
6	BICF2P900599	24027137	A	0	0	G
6	BICF2S23621198	24043394	A	0	0	G
6	BICF2S23448364	24057780	A	0	0	G
6	BICF2P911381	24058564	A	0.5	0.2857	G
6	BICF2P799365	24084340	G	0.5	0.2857	A
6	BICF2P310640	24086653	C	0	0	A
6	BICF2P673753	24094484	A	0	0	G
6	BICF2P316451	24104844	G	0	0	A
6	BICF2P761897	24124265	A	0	0	G
6	BICF2S23052895	24127792	A	NA	0.25	G
6	BICF2P353410	24141625	C	0	0	A
6	BICF2S23638052	24151703	A	0.5	0.2857	G
6	BICF2P94760	24164168	A	0	0	G
6	BICF2P1392321	24174549	C	0.5	0.2857	A
6	BICF2S2327858	24184877	A	0.5	0.2857	G
6	BICF2P717608	24197948	G	0.5	0.2857	A
6	BICF2P304357	24209538	0	0	0	G
6	BICF2P965091	24225895	0	0	0	G
6	BICF2S23764437	24238036	C	0.5	0.2143	A
6	BICF2P360986	24244607	G	0	0	A
6	BICF2P701406	24256984	A	1	0.5	G
6	BICF2P195907	24265947	G	0	0	A
6	BICF2P264265	24284825	0	0	0	A
6	BICF2P826082	24326333	A	0	0	G
6	BICF2P575251	24337169	A	0	0	G

6	BICF2P1049614	24347907	C	0	0	G
6	BICF2S23212685	24368835	A	0	0.2143	G
6	BICF2P1241155	24382095	A	0	0	G
6	BICF2P45948	24401532	G	0.5	0.5714	A
6	BICF2P785593	24405526	A	1	0.7857	G
6	BICF2P451245	24419953	A	0	0	C
6	BICF2P1058864	24426787	A	1	0.7857	G
6	BICF2P1275478	24441035	G	0	0	A
6	BICF2P313656	24451753	A	0	0	G
6	BICF2S23053171	24471954	G	0	0	A
6	BICF2P1397387	24475099	G	0	0	A
6	BICF2P961808	24498758	G	0	0	A
6	BICF2P473936	24509646	C	1	0.7857	A
6	BICF2P756559	24522798	C	1	0.7857	A
6	BICF2P1155853	24539988	T	0.5	0.7857	A
6	BICF2P666665	24542422	G	0.5	0.7857	A
6	BICF2P305201	24563276	G	0.5	0.5	C
6	BICF2P640016	24570819	C	0	0.2143	A
6	BICF2S24320536	24577002	G	0.5	0.2857	A
6	BICF2P1416082	24579645	A	0.5	0.2857	T
6	BICF2S2329620	24591869	G	0.5	0.2857	A
6	BICF2P448459	24604540	C	0	0.2857	A
6	BICF2P1343119	24611172	G	0.5	0.2857	A
6	BICF2P982485	24624576	G	0.5	0.75	A
6	BICF2S2378529	24640527	G	0	0	A
6	BICF2P955237	24643742	A	0.5	0.2857	G
6	BICF2S23637231	24650553	A	0	0.2857	G
6	BICF2P667970	24656459	A	0	0	G
6	BICF2S23134394	24675157	G	0.5	0.2143	A
6	BICF2P281266	24687326	A	0.5	0.5714	C
6	BICF2P906612	24701822	A	0	0.5	G
6	BICF2P1426137	24707531	G	0.5	0.7857	A
6	BICF2P661149	24724921	A	0	0.2143	G
6	BICF2S23644398	24735975	A	0	0.2143	G
6	BICF2P969294	24742537	A	0	0	G
6	BICF2S23441551	24758279	C	0	0	A
6	BICF2P63017	24760122	G	0	0.2857	A
6	BICF2P669531	24780252	A	0	0	G
6	BICF2S23055298	24791309	A	0.5	0.4286	G
6	BICF2S23055390	24795227	A	0	0	G
6	BICF2S23416516	24807212	G	0.5	0.4286	A
6	BICF2P763077	24817190	A	1	0.5	G
6	BICF2S23137884	24832147	A	0	0	G
6	BICF2P543675	24846372	G	0	0	A
6	BICF2P1028826	24852118	C	0.5	0.2857	A
6	BICF2S23016168	24866755	A	0	0	G
6	BICF2S23449922	24875516	A	0.5	0.2143	G

6	BICF2P659354	24896834	C	0.5	0.2143	A
6	BICF2P452891	24898670	0	0	0	G
6	BICF2S2329286	24919622	A	0	0.5	G
6	BICF2P603046	24921857	A	0	0	G
6	BICF2P874868	24931768	A	0	0	C
6	BICF2P1202203	24940827	A	0	0	G
6	TIGRP2P80615_rs8796947	24965196	0	0	0	A
6	BICF2P889950	24967981	G	0.5	0.4286	A
6	BICF2P943395	24985533	A	0	0	G
6	BICF2P1152802	25011392	A	0	0	G
6	BICF2S22936820	25017590	G	0.5	0.7143	A
6	BICF2P232298	25025715	G	0.5	0.2143	A
6	BICF2P619569	25041464	0	0	0	G
6	BICF2P896198	25047261	0	0	0	A
6	BICF2P1114903	25063728	G	0	0.5	C
6	BICF2P1278464	25070193	0	0	0	A
6	BICF2S23123703	25083936	A	0	0.2857	G
6	BICF2S23629231	25103286	0	0	0	A
6	BICF2P708967	25146897	A	0	0	G
6	BICF2P299319	25161185	G	0	0.5	A
6	BICF2P709137	25163642	A	0	0.2143	C
6	BICF2P1211155	25174415	G	0.5	0.7857	C
6	BICF2S2305513	25195196	G	0	0	A
6	BICF2S23253635	25196588	G	0	0.5	A
6	BICF2S23014008	25219452	G	0	0.5	A
6	BICF2S23011518	25223408	A	0	0	C
6	BICF2P1256091	25232129	0	0	0	G
6	BICF2P318271	25243730	G	0.5	0.2857	C
6	BICF2P353285	25255725	G	0	0	C
6	BICF2P58303	25268838	0	0	0	C
6	BICF2P585001	25283409	A	0	0.2143	C
6	BICF2S23327463	25291185	A	0	0.2143	C
6	BICF2P526182	25295621	A	0	0	G
6	BICF2P259466	25303566	G	0	0	A
6	BICF2P736504	25317872	A	0	0.2143	G
6	BICF2P806165	25323560	G	0	0	A
6	BICF2P372909	25343732	0	0	0	C
6	BICF2P649580	25347780	G	0	0.2143	A
6	BICF2P253867	25360217	G	0	0	A
6	BICF2S2447033	25380362	G	0	0.2143	A
6	BICF2P302510	25401566	G	0.5	0.5	C
6	BICF2P262801	25413233	A	0.5	0.5	G
6	BICF2S22911256	25417598	C	0.5	0.5	A
6	BICF2P635933	25425933	G	0.5	0.2857	A
6	BICF2P1105226	25437661	G	0.5	0.5	A
6	BICF2P255057	25439489	A	0	0.2143	G
6	BICF2S23656103	25457771	G	0.5	0.5	A

6	BICF2P1014432	25472371	G	0.5	0.5	A
6	BICF2P1212911	25489543	0	0	0	C
6	BICF2P396755	25499073	A	0	0	G
6	BICF2P416030	25501594	A	0	0	G
6	BICF2P1362514	25511520	A	0	0	C
6	BICF2P887368	25521533	A	0	0.2143	G
6	BICF2P716085	25550109	C	0	0	A
6	BICF2P252611	25556007	A	0	0.2857	G
6	BICF2P130385	25564680	0	0	0	C
6	BICF2P1440795	25578013	A	0	0	G
6	BICF2S23637076	25598912	G	0	0	A
6	BICF2S23510273	25608826	G	0	0	A
6	BICF2P524687	25617813	A	0	0	G
6	BICF2P326907	25646074	A	0	0	G
6	BICF2P314717	25666078	A	0	0.2857	G
6	BICF2P1384220	25673357	0	0	0	A
6	BICF2P657290	25685826	A	0	0.2857	T
6	BICF2P1061739	25691386	G	0	0.2857	A
6	BICF2P1280826	25712636	A	0	0.2857	G
6	BICF2S22923866	25715121	A	0	0	C
6	TIGRP2P80630_rs8985200	25731846	0	0	0	G
6	BICF2P301503	25740329	A	0	0	T
6	BICF2S23722765	25754349	A	1	0.7143	G
6	BICF2S23657929	25769618	A	0	0	G
6	BICF2S23540616	25772484	A	0	0	G
6	BICF2S23532804	25785750	G	0	0.2857	A
6	BICF2S23055056	25798400	A	0	0	C
6	BICF2S2357063	25815666	G	0	0.2857	A
6	BICF2P1081726	25819273	C	0.5	0.5714	A
6	BICF2P401516	25826238	G	NA	0.5	A
6	BICF2P1058213	25829411	G	0.5	0.2857	A
6	BICF2P1436428	25841871	A	0	0	G
6	BICF2P1249950	25853399	C	0	0	A
6	BICF2P339474	25858300	A	0	0	G
6	BICF2P1206542	25863855	0	0	0	G
6	BICF2S2343483	25875949	A	0.5	0.4286	G
6	BICF2P187877	25888360	G	0.5	0.4286	A
6	BICF2P372161	25900797	A	0.5	0.4286	G
6	TIGRP2P80632_rs9058647	25910361	0	0	0	G
6	BICF2P186302	25921932	A	0.5	0.4286	G
6	TIGRP2P80634_rs9086288	25935356	0	0	0	C
6	BICF2P168831	25953471	G	0	0.25	A
6	BICF2S22911317	25966319	A	0.5	0.4286	C
6	BICF2S23728625	25977637	A	0	0	G
6	BICF2S23233285	25986523	G	0.5	0.5714	A
6	BICF2S23233792	25990248	A	0	0	G
6	BICF2S23447666	26006339	0	0	0	A

6	BICF2P756867	26027991	0	0	0	G
6	BICF2S22949912	26037154	C	0	0.2857	A
6	BICF2P919545	26061181	A	0.5	0.5714	G
6	BICF2P536806	26082329	0	0	0	G
6	BICF2P1456049	26097103	A	0	0.2857	G
6	BICF2P1283639	26108462	G	0	0.2857	A
6	BICF2P1084186	26125572	G	0.5	0.5714	A
6	BICF2P446708	26128076	A	0.5	0.5714	G
6	BICF2P943894	26141946	A	0.5	0.5714	G
6	BICF2P378451	26151474	A	0.5	0.5714	G
6	BICF2S23637794	26163376	C	0.5	0.5714	A
6	BICF2P764827	26185365	0	0	0	A
6	BICF2S2414709	26201797	A	0	0	G
6	BICF2S23117697	26213732	G	0.5	0.5714	A
6	BICF2S23034247	26230979	A	0	0	G
6	BICF2S23246310	26265530	A	1	1	T
6	BICF2P1035154	26299063	0	0	0	T
6	BICF2P1009396	26319136	0	0	0	A
6	BICF2P1471156	26325315	0	0	0	C
6	BICF2S23037907	26344333	A	0	0	G
6	BICF2S22960526	26359180	G	0	0	A
6	BICF2S23036401	26389387	A	0	0	C
6	BICF2S23212317	26410743	G	0.5	0.5714	A
6	BICF2S23735439	26421756	A	0.5	0.5714	G
6	TIGRP2P80642_rs8538069	26463664	0	0	0	A
6	BICF2S2366962	26506128	A	0.5	0.7143	C
6	BICF2S23321662	26517892	A	0.5	0.2857	G
6	BICF2S23411908	26545462	G	0	0	A
6	BICF2P498378	26560796	0	0	0	G
6	BICF2P866984	26576260	0	0	0	G
6	BICF2S2455331	26582747	0	0	0	G
6	BICF2P1365348	26597820	0	0	0	G
6	BICF2S22959249	26601998	T	0	0	A
6	BICF2S22924180	26624552	G	0	0	A
6	BICF2S23353616	26641496	G	0	0	A
6	BICF2P1117545	26655781	0	0	0	C
6	BICF2P1156472	26663978	0	0	0	A
6	BICF2P83870	26683898	A	0	0	G
6	BICF2P894420	26704079	0	0	0	A
6	G384f30S274	26749367	0	0	0	C
6	BICF2P541014	26787207	0	0	0	C
6	BICF2P274200	26812642	C	0	0	A
6	BICF2P1033202	26819684	0	0	0	A
6	BICF2P127824	26840601	G	0	0	A
6	BICF2P698604	26868447	G	0	0	C
6	BICF2P706527	26881144	A	0	0	G
6	BICF2P187851	26890469	G	0	0	A

6	BICF2P1263694	26917473	C	0	0	A
6	BICF2P1278096	26924547	A	0	0	C
6	BICF2P369796	26938748	G	0	0	A
6	BICF2P1336628	26966586	A	0	0	G
6	BICF2P653938	26971119	G	0	0	A
6	BICF2P285670	26979226	0	0	0	G
6	BICF2P595815	26995207	0	0	0	A
6	BICF2P402302	27004019	0	0	0	A
6	BICF2P1139947	27008081	0	0	0	G
6	BICF2P200611	27025761	0	0	0	A
6	BICF2P266979	27079201	0	0	0	C
6	BICF2P505604	27086523	0	0	0	A
6	BICF2P107406	27124771	0	0	0	A
6	BICF2P1007943	27156668	0	0	0	C
6	BICF2P588284	27173426	0	0	0	A
6	BICF2P754953	27184689	0	0	0	A
6	BICF2P729785	27212714	0	0	0	G
6	BICF2P289004	27233468	0	0	0	C
6	BICF2P334084	27255723	0	0	0	G
6	BICF2S2291174	27267098	0	0	0	A
6	BICF2S23611133	27285067	0	0	0	G
6	BICF2P761076	27290070	A	0.5	0.2857	C
6	BICF2P1088357	27304538	0	0	0	A
6	BICF2P907990	27323357	A	0	0	G
6	BICF2S23911581	27376247	G	0	0	A
6	BICF2P1087458	27385905	0	0	0	G
6	G385f21S257	27406353	0	0	0	C
6	BICF2S230253	27428560	C	0	0	A
6	BICF2P303540	27458682	0	0	0	C
6	BICF2P27420	27511198	C	0.5	0.4286	A
6	BICF2P971245	27520540	A	0.5	0.4286	G
6	BICF2S23014753	27535949	G	0.5	0.4286	A
6	BICF2P508762	27544093	C	0.5	0.4286	G
6	BICF2P414711	27555000	G	0.5	0.2857	A
6	BICF2P257991	27580395	G	0.5	0.5714	A
6	BICF2P437919	27590235	A	0.5	0.5714	G
6	BICF2P718776	27593605	A	0.5	0.5714	G
6	BICF2P759672	27600299	G	0.5	0.5714	A
6	BICF2P1410889	27601481	A	0.5	0.5714	G
6	BICF2P90444	27618262	A	NA	0.5714	G
6	BICF2P716026	27623002	A	0	0	G
6	BICF2P1312040	27635133	A	0	0.25	C
6	BICF2P1182553	27650224	0	0	0	A
6	BICF2S23424899	27667357	C	0	0.2857	A
6	BICF2P174935	27669659	A	0	0.2857	C
6	BICF2S24117176	27723200	0	0	0	C
6	TIGRP2P80717_rs9253449	27727675	0	0	0	C

6	BICF2P1327108	27755153	0	0	0	G
6	BICF2P1331756	27791309	0	0	0	A
6	BICF2S23652912	27825840	G	0	0	A
6	TIGRP2P80803_rs8598256	27860481	0	0	0	G
6	G386f14S161	27880693	A	0	0	G
6	BICF2S23713729	27904452	0	0	0	G
6	BICF2P967289	27921634	G	0	0	A
6	BICF2P114763	27926336	0	0	0	G
6	BICF2P515434	27955410	0	0	0	A
6	BICF2S23335435	27960374	G	0.5	0.5714	A
6	BICF2S23233208	27978635	0	0	0	G
6	BICF2S23054602	27999134	T	0	0	A
6	BICF2P176230	28005160	0	0	0	A
6	BICF2S23617758	28023064	A	0	0	G
6	BICF2S23031846	28070745	A	0	0	G
6	BICF2P811438	28074377	A	0	0	C
6	BICF2P940877	28092014	0	0	0	A
6	BICF2P1468189	28126145	0	0	0	G
6	G387f48S371	28152754	C	0	0	A
6	BICF2P1104199	28181913	0	0	0	G
6	BICF2P348368	28201184	0	0	0	T
6	BICF2P384961	28265045	0	0	0	A
6	BICF2P678912	28279447	0	0	0	A
6	BICF2P1129268	28322447	0	0	0	A
6	BICF2P536946	28325541	0	0	0	C
6	BICF2P638218	28342915	A	0	0	G
6	G388f28S192	28361904	G	0	0.2857	A
6	BICF2P477959	28387382	0	0	0	G
6	BICF2S23224133	28416738	A	0	0	G
6	BICF2S23618820	28436834	G	1	0.7143	A
6	BICF2S23712545	28442168	C	0	0	G
6	BICF2P897154	28471083	0	0	0	A
6	BICF2S23126187	28479939	A	0	0	G
6	BICF2P536394	28495202	0	0	0	G
6	BICF2P280733	28500894	0	0	0	C
6	BICF2P407684	28508637	0	0	0	G
6	BICF2P8597	28530222	A	0	0	G
6	BICF2P1032507	28534122	G	1	0.7143	C
6	BICF2P1032505	28534475	C	1	0.7143	A
6	BICF2P1436009	28548286	A	0	0	G
6	BICF2P907235	28557712	A	1	0.7143	G
6	BICF2P1206103	28578732	G	0	0	A
6	BICF2P954348	28594291	A	0	0	G
6	BICF2P1431752	28608774	A	0	0	G
6	BICF2P1431746	28612163	A	0	0	G
6	BICF2S2294961	28624934	C	0	0.2857	A
6	BICF2P1399691	28640185	G	0	0.2857	A

6	BICF2P1134399	28646855	0	0	0	G
6	BICF2P1069823	28669053	A	1	0.7143	G
6	BICF2S23211337	28676718	A	0	0	G
6	BICF2S23247346	28691044	G	0	0	A
6	BICF2S22942748	28694703	A	0	0	G
6	BICF2S2348227	28711128	A	0	0	G
6	BICF2P682517	28723588	A	0	0	G
6	BICF2P990387	28736395	A	1	0.7143	G
6	BICF2P165280	28741862	G	1	0.7143	A
6	BICF2S2448495	28753894	A	0	0	G
6	BICF2P265761	28767134	G	0	0	C
6	BICF2S23247530	28773876	G	1	1	A
6	BICF2S23434971	28786359	A	1	1	G
6	BICF2P383630	28804181	A	1	1	G
6	BICF2P1120940	28823083	0	0	0	G
6	BICF2P482754	28833933	A	0	0	G
6	BICF2P270340	28848321	0	0	0	G
6	BICF2S23052260	28855681	C	0	0	A
6	BICF2P1146913	28876243	A	0	0	G
6	BICF2S23044577	28884072	A	0	0	G
6	BICF2P716684	28889451	A	0	0	G
6	BICF2S23112733	28902499	A	0	0	G
6	BICF2P272179	28919275	A	0	0	G
6	BICF2P545493	28923498	G	0	0	A
6	BICF2P545494	28923516	A	0	0	G
6	BICF2P288170	28927711	A	0	0	G
6	BICF2P637916	28933513	C	0	0	G
6	BICF2S23121085	28938587	A	0	0	G
6	TIGRP2P80823_rs9105750	28960627	A	0	0	C
6	BICF2P350446	28972984	A	0	0	G
6	TIGRP2P80824_rs8805371	28989075	0	0	0	G
6	BICF2S23439037	29004501	0	0	0	A
6	BICF2P129894	29015864	G	0	0	A
6	BICF2P1327659	29047479	0	0	0	G
6	BICF2S23152130	29078847	A	0	0	G
6	BICF2P649566	29093918	A	0	0.25	C
6	BICF2P1095108	29113290	G	0	0	A
6	BICF2S2378645	29115537	A	0	0	T
6	BICF2P992505	29123215	0	0	0	G
6	BICF2P333430	29130831	A	0	0	G
6	BICF2S22951669	29144756	0	0	0	A
6	BICF2P1417818	29181012	A	1	0.7143	G
6	BICF2P1457574	29194916	G	0	0	A
6	BICF2P1313122	29205729	A	0	0	G
6	BICF2P930421	29211506	A	0	0	C
6	BICF2S22938690	29224882	G	0	0	A
6	BICF2P704885	29236525	A	0	0	G

6	BICF2P423478	29248457	A	0	0	G
6	BICF2P1167348	29258705	C	0	0	A
6	BICF2P254642	29267768	A	0	0	G
6	BICF2P1239210	29286370	C	0	0	A
6	BICF2P1067537	29295101	A	0	0	G
6	BICF2S2341108	29307071	G	0	0	A
6	BICF2P1016121	29323840	C	0	0	A
6	BICF2P605982	29328965	0	0	0	A
6	BICF2P1201594	29343278	0	0	0	A
6	BICF2P365262	29356374	A	0.5	0.2857	G
6	BICF2P576464	29363433	C	0	0	A
6	BICF2P211746	29379193	T	0.5	0.2857	A
6	BICF2P1275401	29384655	G	0.5	0.2857	A
6	BICF2S23328313	29420944	C	0.5	0.25	A
6	BICF2P1100715	29436007	0	0	0	C
6	BICF2P667672	29442655	A	0	0.2857	G
6	BICF2P439682	29459077	A	0	0.2857	G
6	BICF2P484381	29470908	A	0	0.2857	G
6	BICF2P1182563	29481649	A	0	0.2857	G
6	BICF2P37958	29490247	G	0.5	0.5714	A
6	BICF2P1123224	29508015	C	0	0.2857	A
6	BICF2P1015466	29511282	C	0	0.2857	A
6	BICF2S23443978	29541867	A	0	0.2857	G
6	BICF2S23658175	29559055	G	0	0.2857	A
6	BICF2P1335505	29566400	A	0.5	0.2857	G
6	BICF2P621911	29587447	A	0.5	0.4286	C
6	BICF2P678360	29594766	A	0.5	0.2857	C
6	BICF2P1425876	29597429	T	0.5	0.2857	A
6	BICF2S2316921	29604620	A	0	0.2857	G
6	BICF2P918508	29619790	A	0	0	G
6	BICF2P906459	29632974	A	0	0	G
6	BICF2P596865	29645949	0	0	0	G
6	G389f25S329	29668017	C	0	0	A
6	BICF2P920365	29687744	A	0	0	G
6	BICF2P646755	29694430	A	0.5	0.5714	G
6	BICF2P1004250	29708503	A	1	0.7143	G
6	BICF2P168471	29726203	G	0	0	C
6	BICF2P1316323	29728523	A	0	0.2857	G
6	BICF2S23759301	29748033	A	0.5	0.4286	G
6	BICF2P395778	29750867	G	0.5	0.2857	A
6	BICF2P696050	29784277	A	0.5	0.5714	C
6	BICF2S23724885	29788824	A	0	0	G
6	BICF2S22960534	29823320	G	0.5	0.4286	A
6	BICF2P1455640	29832894	A	0	0	G
6	BICF2P1018710	29843115	C	0	0	A
6	BICF2S229607	29862688	C	0.5	0.5714	A
6	BICF2P900547	29875599	C	0	0.2857	A

6	BICF2P94612	29885183	A	0.5	0.2857	G
6	BICF2S23530601	29892061	G	0.5	0.5714	A
6	BICF2P195368	29901293	A	0.5	0.2857	G
6	BICF2P1266054	29922226	G	0	0	A
6	BICF2P622131	29928970	A	0	0	G
6	BICF2P1159271	29940038	A	0	0.2857	G
6	BICF2P180699	29950944	C	0.5	0.2857	G
6	BICF2P1365488	29955696	A	0	0	G
6	BICF2P638752	29966010	G	0.5	0.5714	A
6	BICF2P594608	29979778	A	0.5	0.2857	G
6	BICF2P447605	29986161	A	0	0	G
6	BICF2P872463	29992635	A	0	0	G
6	BICF2P632743	30002668	G	0.5	0.2857	A
6	BICF2P737234	30003722	A	0	0	G
6	BICF2P1211036	30019861	A	1	0.7143	G
6	BICF2S2342707	30032808	A	0	0.2857	G
6	BICF2S22925667	30040622	A	1	0.7143	G
6	BICF2P505905	30057849	A	0	0	G
6	BICF2S23644328	30072136	A	0.25	0.6429	G
6	G390f36S199	30100616	0	0	0	C
6	BICF2S23528738	30125059	A	0.5	0.7143	G
6	BICF2P1408246	30131017	0	0	0	C
6	BICF2P812428	30151745	T	0.5	0.2857	A
6	TIGRP2P80844_rs9030379	30155036	0	0	0	A
6	BICF2S23447005	30168915	G	0	0	A
6	BICF2S22963847	30180325	C	0	0	A
6	BICF2P126725	30201043	0	0	0	G
6	BICF2P424925	30216395	G	1	1	A
6	BICF2P891201	30222205	A	0	0	C
6	BICF2P394949	30234716	G	0	0	A
6	BICF2P796471	30237281	A	0	0	G
6	BICF2P1199652	30245566	0	0	0	A
6	BICF2P49911	30259363	C	1	0.7143	A
6	BICF2P367160	30268808	A	1	0.7143	G
6	BICF2P1410071	30281444	G	0	0.2857	A
6	BICF2P1024427	30320968	G	0.5	0.4286	A
6	BICF2P332405	30329729	A	0	0	G
6	BICF2P402565	30342875	A	1	1	G
6	BICF2P1371467	30355315	G	0	0	A
6	BICF2P534120	30390022	A	0	0	C
6	TIGRP2P80849_rs9025818	30390799	0	0	0	G
6	BICF2P148784	30395314	A	1	1	G
6	BICF2P163535	30414787	0	0	0	G
6	BICF2P481067	30418905	0	0	0	A
6	BICF2P989853	30442390	0	0	0	A
6	BICF2P137751	30464951	G	0	0	A
6	BICF2S23538926	30500492	G	0	0	A

6	BICF2P905484	30515147	C	0.5	0.2857	G
6	BICF2S2303270	30527416	G	0	0.2857	A
6	BICF2S23230434	30547221	G	0	0	A
6	BICF2P1282995	30555767	A	0	0	G
6	BICF2P468703	30570019	A	0	0.2857	C
6	BICF2P1276861	30578724	A	1	0.7143	G
6	BICF2P396597	30590175	C	0.5	0.2857	A
6	BICF2P387883	30604319	A	0.5	0.2857	G
6	BICF2P1245661	30621327	C	0.5	0.2857	A
6	BICF2S22949377	30641636	G	0	0.2857	C
6	BICF2P816740	30669336	A	0.5	0.7143	G
6	BICF2P30466	30675469	A	0.5	0.7143	C
6	BICF2S23244470	30690911	A	0	0	G
6	BICF2P836619	30694717	G	0.5	0.5714	A
6	BICF2P282715	30705314	0	0	0	A
6	BICF2P1397874	30722469	C	0.5	0.5714	G
6	BICF2P917923	30741076	G	0.5	0.2857	A
6	BICF2P917920	30741920	G	0.5	0.2857	A
6	BICF2P1353848	30757081	0	0	0	G
6	BICF2P874646	30771177	A	0.5	0.2857	G
6	BICF2P874654	30774274	A	0.5	0.2857	G
6	BICF2P1180339	30788504	A	0.5	0.2857	G
6	BICF2P514244	30800164	G	0.5	0.2857	A
6	BICF2P856881	30809290	0	0	0	C
6	BICF2S23251131	30823103	G	0.5	0.2857	A
6	BICF2P598167	30835881	A	0	0	G
6	BICF2P198317	30848628	0	0	0	A
6	BICF2S23716269	30855695	G	0.5	0.2857	A
6	BICF2P1386120	30868801	A	0.5	0.2857	G
6	BICF2P204270	30886921	G	0.5	0.4286	A
6	BICF2P873440	30894927	0	0	0	A
6	BICF2P873446	30896499	A	0	0	G
6	BICF2P144559	30900665	0	0	0	C
6	BICF2P1470629	30917487	A	0	0	G
6	BICF2S23226656	30940261	G	0	0	A
6	BICF2P668315	30949244	0	0	0	C
6	BICF2S23014331	30960792	A	0	0	G
6	BICF2P438456	30971665	A	0	0	C
6	BICF2P137455	30984247	A	0.5	0.5714	G
6	BICF2P1440930	30996512	A	0.5	0.4286	G
6	BICF2P1440922	30998693	C	0.5	0.4167	A
6	BICF2P90975	31010975	0	0	0	T
6	BICF2P886957	31023348	0	0	0	C
6	BICF2S23019547	31031645	A	1	1	G
6	BICF2P1106134	31049028	G	1	1	A
6	BICF2P521657	31058982	G	1	1	A
6	BICF2P172351	31067563	A	0	0	G

6	BICF2P445676	31073734	C	0	0.2143	A
6	BICF2P406152	31087763	A	0	0	G
6	BICF2P898398	31099237	G	0	0	A
6	BICF2P1126147	31108747	A	1	1	C
6	BICF2P369864	31120160	A	0	0.2143	G
6	BICF2P589362	31130569	A	1	0.7857	G
6	BICF2P1002330	31136571	C	0	0.2143	A
6	BICF2S23239881	31144892	A	1	0.7857	G
6	BICF2P169514	31158603	G	0	0	A
6	BICF2P978858	31160725	G	0	0	A
6	BICF2P203401	31175232	A	0	0	G
6	BICF2P357604	31187243	C	0	0	A
6	BICF2P304666	31188863	G	0	0.2857	A
6	BICF2G630586560	31221139	G	0	0	A
6	BICF2G630586555	31223490	G	1	0.7857	A
6	BICF2G630586533	31234056	A	0	0	G
6	BICF2G630586490	31266640	C	1	0.7857	A
6	BICF2P68842	31274337	G	1	1	A
6	BICF2G630586474	31282253	G	1	1	A
6	BICF2G630586426	31303150	C	0	0	A
6	BICF2G630586413	31323912	0	0	0	C
6	BICF2P316380	31326899	0	0	0	A
6	BICF2G630586410	31339166	A	0.5	0.2857	G
6	BICF2P889173	31359258	A	0	0	G
6	BICF2P1354575	31370678	G	0.5	0.4286	A
6	BICF2G630586406	31373113	G	0.5	0.2143	A
6	BICF2G630586373	31392244	A	0.5	0.7857	G
6	BICF2G630586361	31403633	C	0	0	A
6	BICF2G630586350	31415653	G	0	0	A
6	BICF2G630586341	31421513	A	0	0	G
6	BICF2P1283892	31439964	C	0.5	0.2143	G
6	BICF2P1283893	31440015	0	0	0	A
6	BICF2G630586305	31495544	A	0	0	G
6	BICF2S22941570	31508401	T	0.5	0.5714	A
6	BICF2G630586304	31516448	A	0	0.2143	G
6	BICF2S23140292	31527771	G	0	0	A
6	BICF2S23229089	31553428	0	0	0	G
6	TIGRP2P80872_rs8782871	31600468	0	0	0	G
6	BICF2G630586300	31603604	A	0.5	0.4286	G
6	BICF2P1320833	31636517	0	0	0	C
6	BICF2S23023929	31684025	G	0	0	A
6	BICF2P934520	31698321	G	0	0	C
6	BICF2P933043	31731949	0	0	0	A
6	BICF2P686007	31746530	0	0	0	A
6	TIGRP2P80880_rs8685059	31761429	0	0	0	C
6	BICF2P1053587	31768381	A	0	0	G
6	BICF2S23032355	31776253	C	0.5	0.5	A

6	BICF2S23122920	31791794	G	0	0	A
6	BICF2P365482	31812759	0	0	0	G
6	BICF2P442357	31820768	0	0	0	G
6	BICF2P515022	31823557	C	0	0.1667	A
6	BICF2P379136	31832571	A	0	0	G
6	BICF2P403395	31844584	0	0	0	G
6	BICF2P167158	31859099	A	0	0.2143	C
6	BICF2P364263	31868891	C	0.5	0.5	A
6	BICF2P1416367	31885889	G	0	0.2143	A
6	BICF2P334562	31894565	C	0.5	0.5	A
6	BICF2S22958858	31914941	A	0.5	0.2857	G
6	BICF2P235383	31926097	A	0.5	0.5	G
6	BICF2S23611000	31943854	A	0.5	0.5	G
6	BICF2P580104	31964007	A	0.5	0.25	C
6	BICF2P676216	31970161	0	0	0	G
6	TIGRP2P80892_rs9239190	31986531	0	0	0	G
6	BICF2S23124815	32001000	G	0	0	A
6	BICF2S23124548	32004983	A	0	0	G
6	BICF2P574946	32026980	0	0	0	A
6	BICF2S23416665	32049690	0	0	0	A
6	BICF2P694019	32064532	0	0	0	A
6	BICF2S2346673	32077176	0	0	0	G
6	BICF2S23526203	32113806	A	0.5	0.5	C
6	BICF2P1033914	32119954	G	0.5	0.2	A
6	BICF2P712476	32153514	0	0	0	G
6	G393f33S95	32178464	G	0	0	A
6	G394f40S328	32223324	G	0	0	A
6	BICF2P107844	32247074	0	0	0	A
6	BICF2G630586289	32262704	A	0.5	0.2857	G
6	BICF2G630586273	32276966	A	0.5	0.2857	G
6	BICF2G630586252	32290577	A	0.5	0.2857	G
6	BICF2G630586251	32294221	G	0.5	0.2857	A
6	BICF2P1049781	32304865	0	0	0	C
6	BICF2G630586247	32320193	C	0.5	0.2857	A
6	BICF2G630586243	32330786	A	0.5	0.2857	G
6	BICF2G630586237	32335990	G	0.5	0.2857	A
6	BICF2G630586232	32339725	A	0.5	0.2857	C
6	BICF2G630586221	32349517	G	0	0	A
6	BICF2G630586204	32362008	A	0	0	G
6	BICF2G630586195	32388206	A	0.5	0.2857	G
6	BICF2G630586186	32395733	A	0.5	0.2857	G
6	BICF2G630586174	32410899	C	0.5	0.2857	G
6	BICF2P766934	32427818	0	0	0	G
6	BICF2G630586173	32433646	A	0	0	C
6	BICF2G630586172	32459136	T	0	0	A
6	TIGRP2P80903_rs9220084	32470293	0	0	0	A
6	BICF2P1048494	32482787	0	0	0	G

6	BICF2P1274393	32510434	0	0	0	A
6	BICF2P554711	32531179	0	0	0	A
6	BICF2S23238114	32550032	G	0	0	A
6	BICF2S23913859	32560872	G	0	0	A
6	BICF2P1082465	32578247	0	0	0	C
6	BICF2G630586164	32611556	A	0.5	0.25	G
6	BICF2S23751231	32614977	0	0	0	G
6	BICF2P940079	32636052	0	0	0	G
6	BICF2S23764469	32677686	G	0	0	A
6	BICF2G630586159	32691102	G	0.5	0.2857	A
6	BICF2S23318850	32701813	G	0	0	A
6	BICF2P395369	32717258	A	0	0	G
6	BICF2P393159	32726110	0	0	0	C
6	BICF2G630586157	32737268	A	0	0	G
6	BICF2G630586155	32751599	A	0	0	C
6	BICF2G630586152	32760070	A	0	0	G
6	BICF2G630586148	32764439	G	0	0	A
6	BICF2G630586141	32776531	A	1	1	G
6	BICF2G630586120	32792009	A	0	0	T
6	BICF2G630586118	32792862	A	0	0	C
6	BICF2G630586094	32804049	A	0.5	0.75	G
6	BICF2G630586089	32816745	A	0.5	0.7143	G
6	BICF2G630586076	32823773	A	0	0.25	G
6	BICF2G630586060	32832568	A	0	0.2	C
6	BICF2G630586047	32845553	A	0	0.2857	G
6	TIGRP2P80934_rs8562242	32858477	A	0.5	0.2143	G
6	BICF2G630586041	32858538	A	0.5	0.5714	G
6	BICF2G630586035	32870834	A	0	0.2143	C
6	BICF2G630586021	32888928	A	0	0.2143	G
6	BICF2G630586020	32892236	G	0	0.2143	A
6	BICF2P1463132	32908844	A	0	0.2857	G
6	BICF2S23519332	32922375	0	0	0	G
6	BICF2G630585990	32934066	A	0	0	G
6	BICF2G630585988	32944553	A	0	0	G
6	BICF2G630585983	32950706	G	1	0.5	A
6	BICF2S22920613	32960859	A	0.5	0.2857	G
6	BICF2G630585970	32979178	A	0.5	0.2143	G
6	BICF2G630585948	32995803	A	1	0.5	G
6	BICF2G630585939	33012983	G	1	0.5	A
6	BICF2P266971	33022613	G	0	0	A
6	BICF2P876678	33029507	0	0	0	G
6	BICF2P159698	33045349	0	0	0	C
6	BICF2S23740638	33056727	A	0	0	G
6	BICF2P779237	33066944	0	0	0	A
6	BICF2G630585924	33080427	G	0	0	A
6	BICF2S23611539	33086696	G	0	0	A
6	BICF2S2361347	33102379	A	0	0	G

6	BICF2S2441636	33109656	A	0	0	G
6	BICF2G630585909	33128416	A	1	0.5	G
6	BICF2G630585884	33136151	A	0	0	G
6	BICF2S2317602	33144178	A	0	0.2143	G
6	BICF2G630585862	33161209	A	1	0.5	G
6	BICF2G630585860	33168480	A	1	0.5	C
6	BICF2G630585845	33184829	G	0	0	A
6	BICF2S23335292	33189108	A	0	0	G
6	BICF2G630585822	33204966	A	0	0	G
6	BICF2G630585816	33212835	A	1	0.5	G
6	BICF2P842066	33224059	A	1	0.5	G
6	BICF2S23327080	33232181	A	0	0.2143	G
6	BICF2G630585790	33237422	A	0	0	C
6	BICF2S22919529	33257269	A	0	0.5	G
6	BICF2G630585768	33260944	G	0	0	A
6	BICF2G630585754	33277659	A	0	0	G
6	TIGRP2P80963_rs8969411	33291381	A	0	0.2857	G
6	TIGRP2P80964_rs8775794	33293874	A	0	0.2857	G
6	BICF2G630585683	33305291	A	0	0	G
6	BICF2P1114240	33317329	A	0	0.2143	G
6	TIGRP2P80972_rs8979745	33320114	G	0.5	0.4286	A
6	BICF2P633167	33320273	A	0.5	0.4286	G
6	TIGRP2P80989_rs8632560	33336139	G	NA	0.6667	A
6	BICF2P500653	33339952	G	0.5	0.2857	A
6	BICF2S2336013	33342213	A	0.5	0.4286	G
6	BICF2P510086	33350226	C	0.5	0.2857	G
6	BICF2P266206	33361996	G	0.5	0.2857	A
6	TIGRP2P81019_rs8968368	33377323	G	0	0.5	A
6	BICF2G630585615	33388716	A	0.5	0.4286	G
6	BICF2G630585581	33396458	C	0.5	0.2857	G
6	BICF2G630585553	33407763	C	0.5	0.5	A
6	BICF2G630585521	33423400	C	0	0.2143	A
6	BICF2G630585516	33427083	A	0.5	0.5	C
6	TIGRP2P81041_rs9013616	33432114	A	0.5	0.5	C
6	BICF2P140675	33443279	A	0.5	0.5	G
6	BICF2S23113728	33455565	A	0.5	0.4	G
6	BICF2S23512763	33468177	G	0.5	0.5	A
6	BICF2G630585504	33481415	A	0.5	0.2143	G
6	BICF2S22943050	33489890	C	0.5	0.2857	A
6	BICF2G630585497	33500464	A	0	0.2857	G
6	BICF2G630585464	33521214	G	0.5	0.5714	A
6	BICF2P932616	33525066	G	0.5	0.2857	A
6	BICF2G630585419	33548297	A	0	0.2857	G
6	BICF2G630585403	33566573	A	0	0	G
6	BICF2G630585392	33570931	A	0	0	C
6	BICF2G630585368	33582449	G	0	0	A
6	BICF2S2327131	33591521	A	0	0	G

6	BICF2G630585306	33613786	G	0	0	A
6	BICF2P1383420	33620695	G	0	0	A
6	BICF2G630585288	33621483	A	0	0	T
6	BICF2G630585283	33625491	G	0	0	A
6	TIGRP2P81106_rs8624553	33627945	A	0	0	G
6	BICF2G630585276	33646931	A	0	0	C
6	BICF2G630585269	33668574	G	0	0	A
6	BICF2G630585267	33677577	C	0	0	A
6	BICF2P1071770	33686123	A	0	0.2143	G
6	BICF2G630585260	33713224	G	0.5	0.2857	A
6	BICF2G630585251	33728186	G	0	0	A
6	BICF2S2432793	33731272	A	0	0	G
6	BICF2G630585247	33731293	A	0	0	G
6	BICF2G630585246	33731696	A	0	0	G
6	BICF2G630585244	33733629	A	0.5	0.5	C
6	BICF2S2293427	33742519	A	0.5	0.5	G
6	BICF2P289952	33753776	A	0.5	0.5	C
6	BICF2P289953	33753798	A	0.5	0.5	G
6	BICF2G630585189	33768975	A	0.5	0.5	G
6	BICF2G630585177	33778623	A	0.5	0.5	G
6	BICF2G630585155	33794401	G	0	0.2143	A
6	BICF2G630585148	33799904	G	0	0.2143	A
6	BICF2S2341729	33812952	C	0	0.2143	A
6	BICF2P872134	33821965	A	0	0.2143	G
6	BICF2G630585128	33835903	C	0	0.2143	A
6	BICF2G630585105	33860261	G	0	0.2143	A
6	BICF2G630585101	33863487	C	0	0.2143	A
6	BICF2G630585090	33871128	A	0	0.2143	G
6	BICF2G630585084	33885598	A	1	0.7857	G
6	BICF2G630585076	33891088	A	0	0	G
6	BICF2G630585064	33903379	G	0	0	A
6	TIGRP2P81125_rs8413452	33915762	C	0	0.2143	A
6	BICF2P195062	33936166	A	0.5	0.7857	C
6	BICF2G630584996	33941504	A	0.5	0.5714	G
6	BICF2G630584989	33952280	A	0	0	G
6	BICF2G630584984	33961302	A	0.5	0.5714	G
6	BICF2G630584967	33977225	A	0.5	0.2143	G
6	BICF2G630584961	33983750	A	0.5	0.1667	G
6	BICF2G630584946	33994334	A	0.5	0.2143	G
6	BICF2G630584929	34012214	G	0.5	0.2143	A
6	BICF2G630584915	34026012	G	0.5	0.7857	A
6	BICF2G630584910	34031035	A	0	0	C
6	BICF2G630584879	34052522	A	0.5	0.2143	G
6	BICF2G630584875	34067028	G	0.5	0.2143	A
6	TIGRP2P81145_rs9092512	34078085	A	0.5	0.5714	T
6	TIGRP2P81148_rs9079944	34088697	A	0.5	0.5714	G
6	TIGRP2P81156_rs9086008	34104789	G	0.5	0.5714	A

6	BICF2G630584866	34119279	G	0.5	0.5	A
6	BICF2G630584860	34127555	G	0.5	0.5	A
6	BICF2G630584850	34138475	G	0.5	0.5	A
6	BICF2G630584847	34145646	G	0.5	0.5	A
6	BICF2G630584843	34150002	A	0.5	0.2143	G
6	BICF2G630584824	34164204	G	0.5	0.5	A
6	BICF2S23319359	34171127	G	0.5	0.5	A
6	BICF2P960979	34179155	A	0.5	0.5	G
6	BICF2P665165	34196845	G	0.5	0.2143	A
6	BICF2G630584798	34198956	A	0	0	G
6	BICF2P1219939	34203582	A	0.5	0.2143	C
6	TIGRP2P81177_rs8680801	34222467	G	0.5	0.2143	A
6	BICF2P62557	34227981	A	0.5	0.2143	G
6	BICF2P1041381	34242682	A	0	0.2857	G
6	BICF2S23513093	34251825	A	0	0	G
6	BICF2G630584731	34255085	A	0	0	G
6	BICF2G630584703	34260936	G	1	0.7143	A
6	BICF2G630584685	34275407	C	1	1	A
6	BICF2G630584662	34282987	A	0	0	G
6	BICF2G630584639	34297408	A	0	0	G
6	BICF2G630584604	34315357	A	0	0	G
6	BICF2P793970	34326625	A	0	0	G
6	BICF2G630584586	34327941	A	0	0	G
6	BICF2P993907	34348364	G	0	0	A
6	BICF2P312113	34361015	A	0	0	G
6	BICF2G630584534	34361331	G	0	0	A
6	BICF2G630584524	34368266	A	0	0	G
6	BICF2G630584509	34382260	A	0	0.2857	G
6	BICF2P1257291	34395175	G	0	0.2857	A
6	BICF2G630584488	34398652	A	0	0	G
6	BICF2P1144792	34409440	A	0	0.2857	G
6	BICF2P923393	34425853	A	0.5	0.5	G
6	BICF2G630584465	34432216	C	0.5	0.1667	A
6	BICF2G630584454	34445435	A	0.5	0.2143	G
6	BICF2P915525	34460412	C	0	0	A
6	BICF2G630584419	34462130	C	0	0	G
6	BICF2G630584417	34462770	A	0	0	C
6	BICF2G630584408	34473683	A	0	0	G
6	BICF2G630584401	34477090	A	0	0	C
6	BICF2S24415381	34491126	0	0	0	C
6	BICF2G630584358	34510554	A	0	0	G
6	TIGRP2P81256_rs9074948	34517257	G	0.5	0.7857	A
6	TIGRP2P81260_rs9032553	34527943	0	0	0	G
6	BICF2P126834	34535955	A	0	0.2857	G
6	BICF2P573476	34551591	A	0	0	G
6	BICF2P412160	34558904	0	0	0	G
6	BICF2G630584326	34569339	A	0	0	G

6	BICF2P879558	34581239	0	0	0	G
6	BICF2G630584311	34595469	A	0	0	G
6	BICF2G630584282	34616995	A	1	1	G
6	BICF2G630584265	34635530	C	0	0	A
6	BICF2G630584254	34643811	C	0	0	A
6	BICF2G630584243	34650065	G	0	0	A
6	BICF2P415461	34670914	A	0.5	0.2143	G
6	BICF2G630584206	34673016	A	0	0	G
6	BICF2P1289134	34690173	A	0	0	G
6	BICF2G630584177	34700841	A	0.5	0.7857	G
6	BICF2G630584171	34705790	A	0.5	0.7857	G
6	BICF2G630584167	34708232	A	0	0	G
6	BICF2G630584155	34719660	A	0.5	0.7857	G
6	BICF2G630584154	34723098	T	0.5	0.2143	A
6	BICF2G630584147	34731480	G	0.5	0.2143	A
6	BICF2P882062	34747657	A	0	0	G
6	BICF2G630584115	34754925	G	0	0	C
6	BICF2G630584097	34760041	A	0	0	C
6	BICF2G630584081	34772603	T	0	0	A
6	BICF2G630584076	34782166	A	0	0	T
6	BICF2P665652	34790892	A	0	0	G
6	BICF2P567160	34794377	G	0.5	0.2143	A
6	BICF2G630584039	34809359	A	0.5	0.7857	G
6	BICF2G630584034	34812095	A	0	0	G
6	BICF2G630584010	34824165	A	0	0	G
6	BICF2G630583996	34828903	A	0	0	G
6	BICF2G630583987	34839464	A	0	0	G
6	BICF2P388604	34846842	A	0	0	G
6	BICF2P91415	34859257	A	0.5	0.2143	G
6	BICF2G630583975	34874368	G	0.5	0.7857	A
6	BICF2G630583949	34881365	G	0.5	0.5	A
6	BICF2S23330281	34894830	A	0.5	0.5	G
6	BICF2G630583937	34897774	G	0.5	0.5	A
6	BICF2G630583924	34911762	A	0	0	G
6	BICF2G630583909	34917587	A	0	0	G
6	BICF2S23252462	34935950	G	0.5	0.5714	A
6	BICF2P305135	34939563	A	0	0	C
6	BICF2P266900	34956002	C	0.5	0.2143	A
6	BICF2P469674	34963301	A	0.5	0.2857	G
6	BICF2G630583872	34977604	G	0.5	0.2857	A
6	BICF2G630583856	34990481	A	0.5	0.4286	C
6	BICF2G630583839	34997726	T	0	0.2143	A
6	BICF2P1325833	35008979	A	0.5	0.2143	C
6	BICF2G630583799	35014520	G	1	0.7857	A
6	BICF2S22928590	35028456	G	0.5	0.7143	C
6	BICF2P528826	35041042	G	0.5	0.5714	A
6	BICF2S2337208	35062793	C	1	0.7857	A

6	BICF2G630583777	35072272	A	0.5	0.2143	G
6	BICF2P591573	35079251	A	0	0	G
6	BICF2G630583767	35097961	0	0	0	G
6	BICF2P1236425	35109329	A	0	0	G
6	TIGRP2P81378_rs8760318	35129440	G	0.5	0.2143	A
6	BICF2P1227098	35136590	A	0.5	0.2143	G
6	BICF2S2339809	35147968	A	0	0.2857	C
6	BICF2P1164130	35160437	G	0.5	0.2143	A
6	TIGRP2P81392_rs8946488	35172216	G	0.5	0.2143	C
6	BICF2S2395270	35179023	T	0	0	A
6	G395f28S166	35192365	C	0.5	0.2143	A
6	BICF2P1070091	35218310	A	0	0.2857	G
6	BICF2G630583761	35245202	0	0	0	C
6	BICF2P984531	35261294	A	0	0	G
6	BICF2P792980	35277569	A	0.5	0.2143	G
6	BICF2P166168	35288882	A	0	0	G
6	BICF2G630583744	35310200	A	0.5	0.2143	G
6	BICF2G630583738	35321312	0	0	0	G
6	BICF2G630583731	35331385	G	0.5	0.2143	A
6	BICF2G630583726	35350092	C	0	0.2857	G
6	BICF2G630583723	35363287	A	0	0.2857	G
6	BICF2G630583720	35377335	C	0	0.2857	G
6	BICF2S23155698	35394361	A	0	0.2857	G
6	BICF2G630583716	35422125	A	0	0.2857	G
6	BICF2G630583698	35449934	A	0.5	0.7857	G
6	BICF2G630583685	35459495	A	0.5	0.7857	G
6	BICF2G630583674	35467492	G	0.5	0.7857	A
6	BICF2G630583670	35480448	A	0.5	0.7857	G
6	BICF2G630583669	35491820	C	0	0	G
6	BICF2P1012737	35504451	0	0	0	A
6	BICF2G630583666	35531234	0	0	0	G
6	BICF2G630583660	35539753	G	0.5	0.5	A
6	BICF2G630583645	35551424	A	0	0	G
6	BICF2G630583639	35557330	A	0.5	0.2143	G
6	BICF2G630583638	35557812	G	0.5	0.2143	A
6	BICF2G630583636	35559772	A	0.5	0.2143	G
6	BICF2G630583614	35572104	C	0.5	0.5	A
6	BICF2G630583587	35581592	G	0	0	A
6	TIGRP2P81425_rs9204625	35596059	A	0.5	0.5	G
6	BICF2G630583536	35610093	C	0	0.2857	G
6	BICF2G630583531	35616569	G	0	0	A
6	BICF2G630583524	35625293	A	0	0	G
6	BICF2G630583521	35628191	A	0	0	G
6	BICF2G630583509	35641555	A	0	0	G
6	BICF2G630583507	35650404	G	0.5	0.5	A
6	BICF2G630583498	35672733	G	0	0	A
6	BICF2G630583492	35679347	A	0.5	0.7857	G

6	BICF2G630583485	35686319	A	0.5	0.7857	G
6	BICF2P1388321	35699879	0	0	0	A
6	BICF2P704998	35713069	0	0	0	A
6	BICF2S23159009	35723291	A	0.5	0.5	G
6	BICF2G630583469	35741588	C	0.5	0.7857	A
6	BICF2G630583465	35742260	G	0.5	0.7857	A
6	BICF2G630583450	35749162	A	0	0	G
6	BICF2G630583431	35756354	A	0.5	0.5	G
6	BICF2G630583408	35774621	A	0	0.2143	C
6	BICF2S23325507	35792470	A	0	0.2857	G
6	BICF2S22962399	35805096	A	0	0.2857	C
6	BICF2S23719622	35833989	A	0	0.1667	C
6	BICF2S23547531	35835271	A	0.5	0.5833	C
6	BICF2S2352468	35854249	G	0.5	0.1667	A
6	TIGRP2P81445_rs8715661	35860314	C	0	0	A
6	G396f39S331	35875031	G	0	0	A
6	BICF2P140480	35897311	A	0	0.2143	G
6	BICF2S23725873	35910533	G	0.5	0.2143	A
6	BICF2P831172	35917836	G	0	0	A
6	BICF2S23715398	35926191	G	0.5	0.2143	A
6	BICF2P1399036	35941835	A	0	0	G
6	BICF2P1405840	35971183	A	0	0.2143	C
6	BICF2P720129	35979542	0	0	0	A
6	BICF2P39527	35988605	0	0	0	G
6	TIGRP2P81457_rs8691370	35999162	G	0.5	0.5	A
6	BICF2P436603	36008867	A	0	0.2143	G
6	TIGRP2P81459_rs8888004	36028163	C	0	0	A
6	BICF2P814810	36028303	G	0.5	0.2143	A
6	BICF2S23049310	36040852	G	1	0.5	A
6	BICF2P1459416	36041946	A	0.5	0.2143	G
6	BICF2P1122110	36053705	C	1	0.5	A
6	BICF2P1413778	36083307	A	0	0	G
6	BICF2P464343	36089768	A	1	0.5	G
6	BICF2P464346	36090140	A	1	0.5	C
6	BICF2S23031597	36099165	A	0	0	G
6	BICF2S23257230	36111870	A	1	0.5	G
6	BICF2P837034	36131825	A	0	0	G
6	BICF2S23751196	36133708	A	0.5	0.2857	G
6	BICF2P678015	36147283	G	1	0.5	A
6	BICF2P504496	36158966	G	1	0.5	A
6	BICF2P10251	36164299	G	1	0.5	A
6	BICF2P851295	36174980	G	1	0.5	A
6	TIGRP2P81491_rs8816063	36180006	A	0	0	G
6	BICF2P708711	36190555	T	1	0.5	A
6	BICF2P92824	36204004	0	0	0	A
6	TIGRP2P81493_rs8881128	36216258	0	0	0	C
6	BICF2S2411755	36241225	G	0	0	A

6	TIGRP2P81496_rs9252277	36252900	0	0	0	G
6	BICF2S2311850	36261227	G	1	0.7857	A
6	TIGRP2P81501_rs8538978	36301763	A	0	0.2143	G
6	BICF2P421833	36310832	G	0	0.2143	A
6	BICF2P1172651	36319056	A	0	0	G
6	BICF2P1393262	36336135	A	0	0.2143	G
6	BICF2P810072	36348436	A	0	0.2143	C
6	BICF2S23760026	36378861	0	0	0	G
6	BICF2P688184	36389377	0	0	0	G
6	TIGRP2P81528_rs8818067	36401127	C	NA	0.7143	A
6	BICF2P146328	36420524	A	0.5	0.7143	G
6	BICF2P969004	36437516	A	0.5	0.7143	G
6	BICF2P838077	36450765	C	0.5	0.7143	G
6	BICF2P721402	36462210	G	0.5	0.7143	A
6	TIGRP2P81549_rs9206834	36474320	G	0.5	0.7143	A
6	BICF2P1468727	36479098	A	0.5	0.7143	G
6	BICF2P719051	36492792	A	0.5	0.7143	G
6	BICF2P1329144	36501164	A	0.5	0.7143	T
6	TIGRP2P81571_rs8755669	36509464	A	0.5	0.7143	G
6	BICF2P969841	36517359	A	0.5	0.7143	G
6	TIGRP2P81578_rs8510967	36527961	A	0.5	0.7143	G
6	TIGRP2P81584_rs8610786	36537648	G	0.5	0.7143	A
6	TIGRP2P81585_rs9000889	36555768	A	1	1	G
6	BICF2P122841	36565320	0	0	0	G
6	BICF2P1060614	36577595	A	0.5	0.7143	G
6	BICF2P1060613	36577634	A	0.5	0.7143	G
6	BICF2P1000501	36590590	A	1	1	G
6	BICF2P1000500	36590724	G	1	1	C
6	TIGRP2P81600_rs8854929	36598530	C	1	1	A
6	BICF2P1456728	36605166	G	1	1	A
6	BICF2S23121175	36618296	A	1	1	G
6	BICF2P24253	36619565	G	1	1	A
6	TIGRP2P81614_rs8946224	36628645	A	1	1	C
6	BICF2P310965	36648557	A	0	0	G
6	BICF2P589524	36653210	A	0	0	G
6	TIGRP2P81640_rs8766358	36664755	C	0	0	A
6	BICF2P1007669	36677836	A	0	0	G
6	BICF2P696643	36685153	G	0.5	0.2857	A
6	BICF2P789497	36703139	A	1	0.7857	G
6	BICF2P995211	36708354	G	1	0.7857	A
6	BICF2P455543	36730206	A	1	0.7857	G
6	BICF2S23124648	36737473	A	0	0	C
6	BICF2P928316	36751564	A	0.5	0.5	C
6	BICF2P385985	36763836	G	0.5	0.5	A
6	BICF2P977588	36770244	G	0.5	0.5	A
6	BICF2P208560	36780134	G	0.5	0.7143	A
6	BICF2P199122	36788668	G	0.5	0.7143	A

6	BICF2P147888	36802109	A	0.5	0.75	T
6	BICF2P1182583	36813933	A	0	0.2143	T
6	BICF2P70172	36824093	A	0	0.2143	G
6	BICF2P477185	36834609	A	0	0.2143	G
6	TIGRP2P81680_rs9173639	36852583	A	0	0.2143	G
6	BICF2S23027798	36862836	G	0.5	0.5	A
6	BICF2P92317	36871868	C	0.5	0.5	A
6	BICF2P57076	36887169	G	1	1	A
6	BICF2P1426882	36895067	G	1	1	A
6	BICF2P931242	36908135	A	0	0	G
6	BICF2P931243	36908594	A	0.5	0.5	C
6	BICF2P87850	36925050	G	0	0	C
6	BICF2S23440954	36930386	G	0.5	0.5	A
6	BICF2P424706	36945055	A	0	0	G
6	BICF2P1262688	36950136	G	0	0	C
6	BICF2P1349649	36965059	0	0	0	G
6	BICF2S2324971	36976209	A	0	0	G
6	BICF2S2349548	36994844	G	0.5	0.2857	A
6	BICF2S2302221	37004077	G	0.5	0.2857	A
6	BICF2P1155481	37007296	G	0.5	0.2857	A
6	TIGRP2P81722_rs8799482	37023514	C	0.5	0.7143	A
6	TIGRP2P81730_rs8983268	37037955	A	0.5	0.7143	G
6	BICF2P796550	37051400	A	0	0	C
6	TIGRP2P81738_rs8537590	37054795	G	0.5	0.7143	A
6	TIGRP2P81753_rs9127078	37072166	A	0	0	G
6	BICF2S2295629	37087955	G	0.5	0.5	A
6	BICF2P674733	37105612	0	0	0	G
6	BICF2P676091	37117106	A	0.5	0.5	G
6	BICF2S23624107	37125345	A	0	0	G
6	TIGRP2P81770_rs8855362	37133625	0	0	0	A
6	BICF2P537803	37148711	G	0	0	A
6	BICF2P777456	37197288	A	0.5	0.5	G
6	TIGRP2P81785_rs9210545	37212020	0	0	0	C
6	BICF2P73908	37214246	A	0.5	0.5	G
6	BICF2P1171961	37227553	A	0.5	0.5	G
6	BICF2P1004554	37248399	0	0	0	C
6	BICF2S23046408	37262295	A	0	0	G
6	BICF2P884959	37288569	A	0	0	G
6	BICF2P877294	37310225	G	0	0	A
6	BICF2P1412416	37318175	C	0	0	A
6	BICF2P1218715	37339703	0	0	0	G
6	BICF2P708610	37344472	0	0	0	C
6	TIGRP2P81813_rs9029862	37355902	G	0.5	0.5	A
6	BICF2P1087066	37365563	0	0	0	G
6	TIGRP2P81821_rs8936346	37379415	A	0	0	C
6	TIGRP2P81822_rs8670150	37389371	A	1	1	C
6	BICF2P1294734	37407903	A	0.5	0.5	G

6	BICF2S22940740	37418777	C	0.5	0.5	A
6	BICF2S22951326	37424610	A	0	0.2143	G
6	BICF2S23660302	37448483	G	0	0	A
6	TIGRP2P70379_rs8591278	37485807	0	0	0	A
6	TIGRP2P81831_rs8854452	37501824	A	0.5	0.2857	T
6	BICF2P979277	37522062	A	0	0	G
6	BICF2P994020	37530241	G	0	0	A
6	BICF2P261941	37536212	A	0	0	G
6	BICF2P389707	37555002	G	0	0	A
6	BICF2P1382455	37559959	C	0	0	A
6	BICF2P551086	37574132	G	0	0	A
6	BICF2P69598	37585228	A	0	0	G
6	BICF2P936784	37597362	A	0	0	G
6	TIGRP2P81839_rs8733735	37623380	0	0	0	G
6	BICF2S23011584	37633483	A	0	0	G
6	BICF2P1269501	37640990	A	0	0	C
6	BICF2P832746	37645048	G	0	0	A
6	BICF2P908459	37661177	G	0.5	0.2857	C
6	BICF2S23722619	37666633	G	0.5	0.5	A
6	BICF2P677176	37675036	G	0.5	0.2857	A
6	BICF2P896800	37677945	C	0	0	A
6	BICF2P29440	37693653	G	0	0	A
6	BICF2P788878	37700624	G	0.5	0.5	A
6	TIGRP2P81848_rs8752056	37709748	G	0.5	0.5	A
6	TIGRP2P81853_rs8771015	37726004	0	0	0	G
6	BICF2S23444248	37726817	G	1	1	A
6	BICF2P616926	37733580	0	0	0	G
6	BICF2P135141	37750233	A	0	0	C
6	BICF2P1099690	37757941	A	0	0	C
6	BICF2S23116907	37769463	A	0	0	G
6	BICF2P1252672	37779907	A	0	0	G
6	TIGRP2P81865_rs8510463	37790693	G	0.5	0.2857	A
6	BICF2P1195961	37814609	A	0	0.2143	G
6	BICF2S22954515	37833543	C	0.5	0.2857	A
6	BICF2S23316582	37835208	0	0	0	G
6	BICF2S23318868	37838524	G	0.5	0.2857	C
6	BICF2P904894	37851775	A	0.5	0.5	G
6	TIGRP2P81880_rs8867532	37866160	G	0.5	0.5	A
6	BICF2P473287	37869573	0	0	0	C
6	BICF2P113284	37883214	A	0.5	0.7143	G
6	BICF2S23710167	37899164	G	0	0	A
6	BICF2P1022445	37906107	A	0.5	0.2857	G
6	BICF2P943403	37916187	C	0	0	A
6	BICF2S22915467	37928500	A	0	0	G
6	BICF2P444831	37952568	A	0	0	G
6	BICF2S24318032	37963463	A	0	0	G
6	BICF2S23651907	37972683	0	0	0	G

6	BICF2S2294691	37980152	A	1	0.7857	C
6	BICF2P767132	37993411	C	0	0.2143	A
6	BICF2P1049668	37994310	T	0	0.2143	A
6	BICF2P407791	37998070	G	0	0.2143	A
6	BICF2S22956008	38015278	A	0	0	G
6	BICF2P210530	38021005	G	0	0.2143	A
6	BICF2P616063	38036956	A	0	0	G
6	BICF2S23226072	38045531	A	0.5	0.5	G
6	BICF2P206253	38062258	G	0.5	0.5	A
6	BICF2S22932178	38073317	A	0.5	0.5	G
6	BICF2P743582	38077439	G	0.5	0.5	A
6	BICF2P276385	38094559	T	0	0	A
6	BICF2S23543539	38106589	A	0	0	G
6	BICF2P742566	38120369	A	0.5	0.2857	T
6	BICF2P87819	38123773	A	0	0	G
6	BICF2P3686	38142918	A	0	0	G
6	BICF2S23628901	38156910	A	0	0	G
6	BICF2P1290830	38164093	C	0.5	0.5	A
6	BICF2P1290835	38166009	G	0.5	0.5	A
6	BICF2S23718338	38172568	A	0	0.2143	G
6	BICF2S23136468	38181100	0	0	0	G
6	BICF2P243531	38197757	A	0	0.1667	C
6	BICF2P2720	38204639	A	0	0	G
6	BICF2P1071489	38223550	A	0	0	G
6	BICF2S23225095	38234286	A	0	0.2143	G
6	BICF2P1346517	38241901	0	0	0	C
6	BICF2P383085	38255360	A	0	0	G
6	BICF2P1456838	38267980	G	0.5	0.7143	A
6	BICF2S23147489	38282584	G	0.5	0.7143	A
6	BICF2P558681	38291428	G	0	0	A
6	BICF2P351744	38295531	0	0	0	A
6	BICF2P424335	38310726	A	0.5	0.7143	G
6	BICF2P690951	38318994	G	0.5	0.7143	A
6	BICF2P141283	38333093	A	0	0	C
6	BICF2P638309	38352295	A	0	0	C
6	BICF2P858494	38363969	A	0.5	0.2857	G
6	BICF2P1042681	38381043	A	0.5	0.7143	G
6	BICF2P640603	38387123	C	0	0	G
6	TIGRP2P81978_rs8491947	38402424	A	0.5	0.7143	C
6	BICF2P1261462	38420806	0	0	0	G
6	BICF2P494616	38432722	A	0	0	G
6	BICF2P7361	38434390	A	0	0	G
6	BICF2P1213815	38443390	0	0	0	G
6	BICF2S2359990	38452688	0	0	0	G
6	BICF2S23014100	38458803	A	0	0	G
6	BICF2P108486	38470951	0	0	0	A
6	TIGRP2P81992_rs9139791	38481317	G	0	0	A

6	BICF2P1344784	38510576	0	0	0	A
6	BICF2P666351	38524965	0	0	0	G
6	BICF2P752788	38534827	C	1	1	A
6	TIGRP2P82001_rs8592454	38550489	0	0	0	C
6	BICF2S23158646	38579980	G	0.5	0.5	A
6	BICF2S23128931	38583025	A	0	0.2143	G
6	BICF2S23150947	38600876	G	0	0	A
6	BICF2P335714	38625124	0	0	0	A
6	BICF2S23629373	38633056	0	0	0	A
6	BICF2P1178849	38662779	A	0	0	T
6	BICF2S23150755	38667061	A	0	0	G
6	BICF2S23522248	38681629	A	0	0	C
6	BICF2P587687	38687902	G	0	0	A
6	BICF2P1124640	38701052	A	0	0	G
6	BICF2P417946	38709287	A	0.5	0.5	G
6	BICF2S23444273	38722929	0	0	0	G
6	BICF2P669211	38754499	A	0	0	G
6	BICF2P1295914	38769259	A	0	0.2143	G
6	BICF2P711852	38780562	G	0.5	0.5	A
6	BICF2P1064392	38790954	G	1	0.7857	C
6	BICF2P471832	38809126	G	NA	0	A
6	BICF2S23233217	38813022	A	0.5	0.5	G
6	BICF2P850764	38826788	0	0	0	G
6	BICF2P125101	38841160	A	1	1	G
6	BICF2S23750116	38847611	G	0	0	A
6	TIGRP2P82060_rs8936920	38866450	G	0.5	0.5	A
6	TIGRP2P82061_rs8831115	38874616	G	1	1	A
6	BICF2S23048443	38882220	A	0.5	0.2857	G
6	TIGRP2P82066_rs9179165	38895107	G	1	1	A
6	BICF2P1374533	38904648	G	0	0	A
6	BICF2P811446	38917497	G	0.5	0.2857	A
6	BICF2S23726560	38937454	A	0.5	0.2857	G
6	BICF2S231825	38946999	G	0.5	0.2857	A
6	BICF2P1125915	38952394	A	0.5	0.2857	T
6	BICF2P931137	38972914	G	0.5	0.2857	A
6	BICF2S23752604	38977150	G	0.5	0.2857	A
6	TIGRP2P82094_rs9213568	38985085	A	0	0	C
6	BICF2S23335303	38998482	G	0.5	0.2857	A
6	BICF2P56247	39016297	A	0	0.2143	C
6	BICF2P145105	39030866	0	0	0	A
6	BICF2S2318200	39041602	A	0	0	G
6	BICF2S23111522	39045439	A	1	0.7857	G
6	BICF2P224708	39059715	A	1	1	G
6	TIGRP2P82118_rs8883275	39071688	C	0	0.1667	A
6	TIGRP2P82128_rs8874885	39082934	A	0	0	G
6	BICF2S23017964	39097167	A	0	0.2143	C
6	BICF2P401835	39102109	A	0	0.2143	G

6	BICF2S23239129	39119333	A	0	0	G
6	BICF2P385097	39130174	0	0	0	A
6	BICF2P126682	39135610	0	0	0	G
6	BICF2P469300	39147439	G	0	0	A
6	G397f45S191	39175400	C	0	0	A
6	G397f51S65	39180736	C	0	0	A
6	BICF2P1113015	39206608	A	0	0	G
6	BICF2P1404105	39227888	0	0	0	A
6	BICF2S2299926	39241175	G	0	0	A
6	BICF2P5483	39258965	C	0	0	A
6	BICF2S22962275	39292315	G	0	0	C
6	BICF2P166449	39305769	0	0	0	G
6	BICF2P314150	39381573	0	0	0	G
6	BICF2P1035881	39388193	G	0	0	A
6	TIGRP2P82142_rs8740405	39399290	A	0	0.2143	G
6	BICF2P596134	39406405	A	0	0	G
6	TIGRP2P82149_rs9232663	39411139	A	0	0.2143	G
6	BICF2P1452402	39430624	A	0	0	G
6	BICF2S23254241	39444791	A	0.5	0.2857	G
6	BICF2P1249700	39455548	G	0.5	0.2857	A
6	BICF2P1047220	39461101	A	0	0	G
6	BICF2P769873	39476843	A	0	0	C
6	BICF2P35354	39489638	G	0	0	A
6	BICF2P930767	39495230	G	0	0	A
6	BICF2P430522	39512453	A	0	0	G
6	BICF2P544718	39514226	A	0.5	0.2857	C
6	BICF2P480388	39534424	A	0	0	G
6	BICF2P1158025	39543827	A	0.5	0.2857	G
6	BICF2S22964848	39549562	A	0.5	0.2857	G
6	BICF2P750001	39567170	C	0.5	0.2857	A
6	BICF2P1270910	39572926	C	0	0	A
6	BICF2S23255200	39588280	A	0	0	G
6	BICF2P1040753	39608196	G	0	0	A
6	BICF2P1277457	39658735	0	0	0	G
6	TIGRP2P82226_rs8596784	39666177	0	0	0	C
6	BICF2P624002	39694886	C	0	0	A
6	TIGRP2P82245_rs9194876	39717597	A	0	0	G
6	BICF2P100083	39724406	0	0	0	C
6	BICF2P631961	39739075	0	0	0	A
6	BICF2S23650477	39744764	0	0	0	G
6	TIGRP2P82257_rs8762873	39763990	A	0	0	G
6	BICF2P116916	39770725	C	0	0	G
6	BICF2P254968	39785457	A	0	0	G
6	BICF2P326205	39790061	A	0	0	G
6	TIGRP2P82295_rs8675598	39803487	C	0	0	G
6	TIGRP2P82304_rs9151866	39815984	C	0	0	A
6	BICF2P475287	39834610	0	0	0	G

6	BICF2P158376	39839267	A	0.5	0.7143	G
6	BICF2P317925	39857636	0	0	0	C
6	BICF2P1338451	39875474	A	1	1	G
6	BICF2P46043	39883104	A	0.5	0.2857	G
6	BICF2P528992	39901942	G	1	1	A
6	BICF2P927925	39906412	G	1	1	A
6	BICF2P1443038	39917821	A	1	1	G
6	BICF2P1230693	39921668	A	0	0	G
6	TIGRP2P82318_rs9176573	39928177	C	0.5	0.7143	A
6	BICF2P972810	39944085	A	0.5	0.2857	G
6	BICF2S2413509	39959210	A	0.5	0.7143	G
6	BICF2P377800	39971169	0	0	0	C
6	TIGRP2P82328_rs8647916	39978030	A	0.5	0.7143	G
6	BICF2P514679	39986503	A	0.5	0.7143	G
6	TIGRP2P82331_rs9204292	40006529	A	0	0	G
6	TIGRP2P82333_rs8820944	40013655	A	0.5	0.7143	G
6	BICF2P1268953	40024815	A	0.5	0.7143	G
6	BICF2P1207827	40060329	0	0	0	C
6	BICF2P1470880	40066220	0	0	0	C
6	BICF2S23240564	40082990	G	0	0	A
6	BICF2P917312	40090406	0	0	0	A
6	TIGRP2P82345_rs9145829	40102423	0	0	0	G
6	BICF2P34730	40121002	0	0	0	A
6	BICF2P1208269	40130382	0	0	0	G
6	BICF2P1244040	40143620	A	0	0	G
6	BICF2P583447	40151360	G	0.5	0.75	A
6	BICF2P577842	40174119	0	0	0	C
6	BICF2P188647	40189715	A	0.5	0.2857	G
6	BICF2P523207	40195865	G	0.5	0.2857	A
6	BICF2P388423	40204594	0	0	0	A
6	BICF2P465621	40216157	0	0	0	A
6	BICF2S23260000	40233163	G	0	0	A
6	TIGRP2P82360_rs8808304	40245591	G	0.5	0.7143	A
6	BICF2P521632	40263177	G	0.5	0.7143	A
6	BICF2P347552	40273002	0	0	0	A
6	BICF2P590590	40291348	A	0.5	0.2857	G
6	TIGRP2P82364_rs8924787	40333250	0	0	0	G
6	BICF2P1002980	40348648	A	0.5	0.7143	G
6	BICF2P798098	40372939	0	0	0	C
6	TIGRP2P82365_rs8825258	40378961	G	0.5	0.2857	A
6	BICF2P107079	40393137	G	1	1	A
6	BICF2P325599	40408680	0	0	0	C
6	BICF2P30207	40413127	0	0	0	C
6	BICF2S23243059	40438670	A	0	0	G
6	TIGRP2P82368_rs8985569	40449811	0	0	0	G
6	G400f26S231	40458049	G	0	0	A
6	BICF2P650722	40479459	0	0	0	G

6	BICF2P747928	40480731	A	0.5	0.7143	G
6	BICF2S232727	40500648	A	0	0	G
6	BICF2S24321342	40511227	0	0	0	G
6	BICF2P377224	40523887	0	0	0	C
6	BICF2P745913	40531719	0	0	0	G
6	BICF2P664527	40565216	0	0	0	G
6	BICF2P1267273	40577287	0	0	0	A
6	BICF2P1309992	40588805	0	0	0	G
6	G401f21S190	40615389	0	0	0	C
6	TIGRP2P82373_rs8584837	40624405	A	0.5	0.7143	G
6	BICF2P380120	40637380	A	0.5	0.2857	G
6	G402f22S83	40660449	C	0	0	A
6	BICF2S24323195	40691228	0	0	0	C
6	BICF2P824568	40695686	A	0.5	0.2857	G
6	BICF2P1208076	40700326	G	0.5	0.2857	A
6	BICF2P1429329	40711639	A	0	0	G
6	BICF2P271071	40728680	A	0.5	0.2857	G
6	TIGRP2P82396_rs8568148	40738969	G	0.5	0.2857	A
6	BICF2S2356725	40753427	G	0.5	0.2857	A
6	BICF2S23514279	40757288	A	0	0	G
6	BICF2P885681	40814424	A	0	0	C
6	BICF2P638257	40825157	0	0	0	C
6	TIGRP2P82407_rs8876991	40843242	A	0	0	C
6	BICF2P1162433	40864044	0	0	0	G
6	BICF2P597754	40874673	A	0.5	0.7143	T
6	TIGRP2P82422_rs9119151	40900427	C	0	0	A
6	BICF2G630583378	40926819	C	0.5	0.75	A
6	BICF2P1212375	40930695	A	0.5	0.7143	G
6	TIGRP2P82438_rs9130780	40950258	A	0	0	T
6	BICF2S23251760	40964426	A	0.5	0.75	G
6	TIGRP2P82484_rs9228380	40974666	A	0.5	0.8	G
6	TIGRP2P82485_rs8670960	40982343	C	0	0	A
6	BICF2P1349009	40988939	G	0.5	0.7143	A
6	BICF2P169561	40998112	0	0	0	G
6	BICF2P1248342	41011763	A	0.5	0.7143	G
6	BICF2P470796	41030604	A	0.5	0.7143	G
6	BICF2P807798	41034460	A	0	0	C
6	BICF2P97573	41050085	G	0	0	A
6	BICF2P486922	41058989	G	0	0	A
6	BICF2S23229527	41071588	G	0.5	0.2857	A
6	BICF2P842137	41082779	A	0.5	0.7143	G
6	BICF2P614427	41094646	G	0.5	0.2857	A
6	BICF2P1229601	41109622	C	NA	0.25	A
6	BICF2P1035522	41118970	C	0.5	0.2857	A
6	BICF2P333846	41131038	A	0.5	0.7143	G
6	BICF2P555024	41136495	A	0.5	0.2857	G
6	BICF2P1470009	41154425	G	0.5	0.2857	A

6	BICF2P105727	41160660	0	0	0	A
6	BICF2P454034	41179557	A	0.5	0.7143	G
6	BICF2P66878	41212458	0	0	0	C
6	BICF2P37868	41224881	A	0.5	0.7143	G
6	BICF2P37870	41227699	0	0	0	G
6	BICF2S22965094	41239432	G	0	0	A
6	TIGRP2P82503_rs9223425	41254421	0	0	0	G
6	BICF2P645561	41265799	0	0	0	G
6	BICF2S23137306	41302126	G	0	0	A
6	G403f31S140	41326727	0	0	0	A
6	BICF2P284636	41350090	0	0	0	C
6	TIGRP2P82508_rs8788083	41371977	0	0	0	G
6	BICF2S2306974	41387448	A	0.5	0.7143	G
6	BICF2P1338820	41394346	0	0	0	G
6	TIGRP2P82510_rs8753459	41409591	0	0	0	C
6	G404f58S144	41420564	A	0.5	0.7143	G
6	BICF2S23047377	41444590	0	0	0	G
6	BICF2P1199587	41450996	0	0	0	A
6	BICF2P683078	41458666	0	0	0	A
6	BICF2P209193	41474386	0	0	0	G
6	BICF2P820069	41481841	A	0.5	0.5	C
6	BICF2P543483	41508915	0	0	0	G
6	BICF2P1050333	41515948	0	0	0	A
6	BICF2P276866	41539118	0	0	0	T
6	BICF2P1145505	41561277	0	0	0	C
6	BICF2P127326	41592520	0	0	0	C
6	BICF2S2329231	41617520	A	0.5	0.2857	G
6	BICF2S23639647	41628581	G	0.5	0.2857	A
6	BICF2S23413953	41640418	G	0.5	0.2857	A
6	BICF2P390703	41669277	0	0	0	A
6	BICF2S22929078	41680668	G	0	0.2143	A
6	BICF2P258497	41696876	0	0	0	C
6	BICF2S23226277	41701574	G	0.5	0.5	A
6	BICF2P710363	41715532	0	0	0	C
6	TIGRP2P82519_rs8740502	41725543	0	0	0	G
6	BICF2S23645268	41741045	A	0.5	0.5	G
6	BICF2P874226	41768297	0	0	0	G
6	BICF2P127018	41782634	0	0	0	C
6	BICF2S23125546	41802735	A	0	0.2143	G
6	BICF2P1422453	41816897	C	0.5	0.5	G
6	BICF2P818983	41838838	0	0	0	G
6	BICF2S23159703	41854286	G	0.5	0.5	A
6	BICF2S23940	41862869	0	0	0	G
6	BICF2P1280729	41874530	0	0	0	C
6	BICF2S23133583	41891064	0	0	0	C
6	BICF2S23138729	41894933	G	0.5	0.5	A
6	BICF2P471036	41907433	0	0	0	G

6	BICF2P1183035	41937272	0	0	0	G
6	BICF2S23330649	41942601	A	0.5	0.2857	G
6	BICF2P1228611	41955258	0	0	0	C
6	BICF2P838452	41971821	0	0	0	G
6	BICF2S23410149	41982712	G	0.5	0.7143	A
6	BICF2S2416476	42020965	A	0.5	0.2857	G
6	TIGRP2P82534_rs8587251	42029289	0	0	0	C
6	BICF2P245285	42042986	0	0	0	C
6	BICF2P1418220	42063400	0	0	0	G
6	BICF2P100332	42083178	0	0	0	G
6	BICF2P838801	42095303	0	0	0	T
6	BICF2S2299574	42133788	G	1	0.7857	A
6	BICF2S2297073	42147204	A	1	0.7857	G
6	BICF2S23149798	42152794	A	0.5	0.5	G
6	TIGRP2P82536_rs9186665	42169128	0	0	0	G
6	BICF2P256717	42175496	0	0	0	G
6	BICF2S2363119	42190315	G	0.5	0.5	A
6	BICF2P774856	42197004	0	0	0	A
6	BICF2P245928	42209098	A	0.5	0.5	G
6	BICF2S23150146	42224350	A	0.5	0.5	G
6	BICF2S23161373	42239048	G	0.5	0.5	A
6	BICF2P253184	42245037	0	0	0	A
6	BICF2S22949706	42254682	A	0.5	0.5	G
6	BICF2P238612	42276739	0	0	0	A
6	BICF2S23622938	42325529	A	0.5	0.5	G
6	BICF2S23522956	42351127	A	0	0	G
6	BICF2S23651995	42358532	A	0.5	0.5	G
6	BICF2P1465567	42375121	0	0	0	G
6	BICF2P1027311	42383629	0	0	0	G
6	BICF2S23355121	42392531	A	0.5	0.5	G
6	BICF2S2325930	42408080	A	0	0	G
6	BICF2S23028404	42421945	G	0.5	0.5	A
6	BICF2S23210218	42434513	A	0	0	G
6	BICF2S23223490	42437711	A	0.5	0.5	G
6	BICF2P345996	42446503	A	0	0	G
6	BICF2P693757	42458262	G	0	0	A
6	BICF2P375468	42469811	C	0	0	A
6	BICF2S23518221	42481317	0	0	0	A
6	TIGRP2P82565_rs9161287	42499929	C	0	0	A
6	TIGRP2P82569_rs8569081	42506456	A	0	0	G
6	TIGRP2P82574_rs8910870	42523498	G	0	0	A
6	BICF2P272231	42533530	G	0	0	A
6	BICF2P108376	42546206	A	0	0.2143	G
6	TIGRP2P82588_rs8808824	42557161	A	0.5	0.5	G
6	BICF2S2299227	42567469	A	0	0	G
6	BICF2P124134	42580374	A	0	0.2143	G
6	TIGRP2P82596_rs9115315	42590620	A	0	0.2143	G

6	BICF2P329910	42604437	A	0	0.2143	G
6	TIGRP2P82606_rs9175159	42611237	A	0	0.1667	G
6	BICF2P460357	42624919	G	0	0.2143	A
6	BICF2P956852	42634754	A	0	0.2143	G
6	TIGRP2P82642_rs8842525	42645713	0	0	0	C
6	BICF2P226765	42653572	G	0	0.2143	A
6	BICF2P1153729	42675942	0	0	0	G
6	BICF2S24112671	42687975	G	0	0.2143	A
6	BICF2S22957863	42697860	A	0	0	G
6	TIGRP2P82657_rs8659325	42709231	A	NA	0	G
6	BICF2P1233123	42719698	0	0	0	C
6	BICF2P734463	42742293	0	0	0	G
6	BICF2P180359	42753398	0	0	0	A
6	BICF2P548213	42762830	0	0	0	G
6	BICF2P413131	42770311	0	NA	0	A
6	BICF2S2346500	42795543	A	0	0	G
6	BICF2P320839	42803624	0	0	0	G
6	BICF2P390918	42822688	0	0	0	A
6	TIGRP2P82658_rs8749998	42837240	0	0	0	G
6	TIGRP2P82659_rs9175507	42846933	G	0.5	0.5	A
6	BICF2P1185090	42868566	0	0	0	G
6	BICF2P1260051	42875674	0	0	0	G
6	BICF2P831408	42905208	0	0	0	A
6	BICF2P860453	42935882	0	0	0	T
6	BICF2P460737	42942330	0	0	0	A
6	BICF2P1053878	42954128	0	0	0	T
6	TIGRP2P82663_rs8851972	42964607	0	0	0	C
6	BICF2S23232169	42982157	A	0	0	G
6	BICF2G630583358	43044867	0	0	0	G
6	TIGRP2P82664_rs9245163	43066870	0	0	0	C
6	BICF2S24411171	43076649	G	0	0.2143	A
6	BICF2S23413040	43086051	A	0.5	0.2857	T
6	BICF2P1291438	43094598	0	0	0	C
6	BICF2S23246875	43109773	A	0	0.2143	C
6	TIGRP2P82667_rs8801624	43145836	0	0	0	G
6	TIGRP2P82669_rs9164792	43160139	A	0.5	0.2857	G
6	BICF2P475423	43179300	0	0	0	G
6	TIGRP2P82670_rs8601122	43185201	A	0.5	0.5	G
6	BICF2S23340246	43204556	A	0.5	0.2857	G
6	BICF2S23221001	43222080	A	0	0	G
6	BICF2P356644	43228633	A	0.5	0.5	G
6	BICF2P544640	43240375	0	0	0	G
6	BICF2P878324	43259247	A	0.5	0.2857	G
6	BICF2P887364	43271887	A	0.5	0.5	G
6	TIGRP2P82676_rs8871439	43276763	A	0.5	0.2857	G
6	BICF2S2364709	43305453	A	0.5	0.25	G
6	BICF2P911070	43316977	G	0.5	0.2857	A

6	BICF2P1327381	43320602	A	1	0.7857	C
6	BICF2P593087	43343372	0	0	0	C
6	BICF2P1426831	43355418	0	0	0	G
6	BICF2P944683	43447406	0	0	0	G
6	BICF2S22946647	43469953	A	0.5	0.5	G
6	TIGRP2P82702_rs8793479	43473515	0	0	0	C
6	BICF2P1432541	43528658	0	0	0	G
6	TIGRP2P82723_rs8533334	43540897	0	0	0	G
6	TIGRP2P82863_rs8563353	43630337	0	NA	0	C
6	BICF2P650324	43674374	0	0	0	G
6	BICF2P824649	43803989	A	0.5	0.5	G
6	G407f28S153	43825320	0	0	0	A
6	BICF2P632566	43845453	0	0	0	C
6	BICF2S23423870	43872797	0	0	0	C
6	TIGRP2P82947_rs9165461	43890484	G	0.5	0.5	C
6	BICF2P656356	43900327	0	0	0	A
6	BICF2P1106924	43913490	C	0	0	A
6	BICF2P562935	43927209	G	0	0	A
6	TIGRP2P82953_rs8825422	43935349	C	0	0	A
6	BICF2P1434203	43955158	A	0	0	C
6	BICF2P1503372	43988357	G	0	0	A
6	BICF2P325299	44019007	0	0	0	G
6	BICF2P38035	44048043	A	0	0	G
6	TIGRP2P83036_rs8807189	44078303	0	0	0	T
6	BICF2P781189	44127692	A	0	0	G
6	BICF2P965097	44137228	C	0	0	A
6	BICF2P232378	44149636	0	0	0	G
6	BICF2P672090	44176909	A	0	0.2143	C
6	BICF2S23633763	44193880	G	0	0.2143	A
6	BICF2P1035658	44206691	G	0	0.2143	A
6	BICF2P693306	44290198	A	1	0.7857	G
6	BICF2S23019666	44319426	A	1	0.7857	G
6	BICF2P744202	44328418	A	0	0.2143	G
6	BICF2P175607	44337390	G	0	0.2143	A
6	BICF2P638850	44352638	G	0	0.2143	A
6	TIGRP2P83058_rs8874745	44362770	C	0	0	A
6	BICF2P753177	44370579	G	0	0.2143	A
6	BICF2P1045010	44380673	G	0	0	A
6	TIGRP2P83076_rs8823878	44398926	0	0	0	G
6	BICF2P768779	44406077	0	0	0	A
6	TIGRP2P83080_rs8934333	44415959	0	0	0	G
6	BICF2S23447042	44426801	T	0	0	A
6	BICF2S23429697	44441933	G	0	0	A
6	BICF2P1001481	44442529	G	0	0	A
6	BICF2P1168545	44447920	G	0	0	A
6	BICF2P639391	44470107	G	0	0	A
6	BICF2P240889	44478783	C	1	0.7857	A

6	BICF2P692331	44486881	G	0	0	A
6	TIGRP2P83100_rs8711827	44489827	C	0	0	A
6	BICF2P1469694	44502797	0	0	0	A
6	BICF2S22953868	44505782	G	0	0	A
6	BICF2S22953873	44506219	A	0	0	G
6	BICF2P1411250	44527433	A	0	0	C
6	BICF2P473843	44532862	G	0	0	A
6	TIGRP2P83118_rs9252268	44538337	A	0	0	T
6	BICF2P312891	44545121	A	0	0	T
6	BICF2P358881	44558651	0	0	0	G
6	BICF2P356356	44567311	A	1	0.7857	G
6	TIGRP2P83144_rs8864515	44576846	0	0	0	C
6	BICF2P965517	44592582	C	0	0.2143	A
6	BICF2P965520	44595201	G	0	0.2143	A
6	TIGRP2P83154_rs8967842	44598417	A	0	0.2143	G
6	TIGRP2P83163_rs8589110	44608978	A	0	0.2143	G
6	BICF2S2308025	44640934	G	0.5	0.25	A
6	BICF2P142921	44644259	A	0.5	0.2857	G
6	BICF2P158240	44673155	G	0	0.2143	A
6	BICF2P589028	44687464	0	0	0	A
6	BICF2P914298	44692097	A	0	0	G
6	BICF2P1251644	44702348	G	0	0.2143	A
6	BICF2P881640	44713623	G	0	0.2143	A
6	BICF2P1201462	44728001	A	0	0	G
6	BICF2P1233304	44744655	A	0	0.2143	C
6	BICF2P950644	44755569	A	0	0.2143	C
6	BICF2S23153204	44758787	A	0	0	C
6	TIGRP2P83206_rs9114311	44780583	A	0	0	C
6	TIGRP2P83214_rs9080268	44796738	G	0.5	0.2857	A
6	BICF2S23547434	44812525	G	0	0	A
6	BICF2S23152700	44818324	G	0.5	0.2857	A
6	BICF2P195837	44835595	A	0	0	G
6	BICF2P855459	44840032	0	0	0	C
6	BICF2P809445	44854371	G	0.5	0.2857	A
6	BICF2S22930362	44864803	G	0.5	0.2857	A
6	BICF2P963441	44881101	0	0	0	G
6	BICF2P435461	44887168	0	0	0	C
6	BICF2S23134832	44906045	G	0	0	C
6	BICF2S23137750	44909456	G	0.5	0.2857	A
6	BICF2S23624618	44932620	G	0.5	0.5	C
6	BICF2P314100	44945317	0	0	0	A
6	BICF2P1240855	44955927	0	0	0	G
6	BICF2P1376856	44966477	A	0	0	C
6	BICF2S245561	44980601	G	0.5	0.7143	A
6	BICF2P295210	44990741	0	0	0	C
6	BICF2S23142184	45009038	G	0.5	0.7143	A
6	BICF2S23438301	45012177	A	0.5	0.7143	G

6	BICF2S2372183	45025251	A	0.5	0.7143	G
6	BICF2S2318329	45034143	G	0.5	0.7143	A
6	BICF2P87264	45047223	C	0.5	0.5	A
6	BICF2P287904	45061492	0	0	0	G
6	BICF2P922649	45073046	G	0.5	0.5	A
6	TIGRP2P83247_rs8685200	45083688	C	0	0	A
6	BICF2P1180537	45084289	A	0	0	G
6	BICF2P884054	45100137	A	0.5	0.5	C
6	BICF2P1056242	45111817	A	0	0	G
6	BICF2P1461305	45117411	A	0.5	0.5	G
6	TIGRP2P83299_rs8595006	45132220	A	0	0	C
6	BICF2S22912808	45145421	G	0.5	0.2857	A
6	BICF2P1260232	45150260	G	0.5	0.2857	A
6	BICF2P394841	45165418	G	0.5	0.7143	A
6	BICF2P478588	45169539	A	0.5	0.7143	G
6	BICF2P478589	45169984	C	0.5	0.2857	A
6	TIGRP2P83315_rs9172750	45172289	A	0.5	0.7143	T
6	TIGRP2P83317_rs8930730	45184033	A	0.5	0.7143	G
6	BICF2P758479	45203340	A	0	0	T
6	TIGRP2P83325_rs9122843	45211932	T	0	0.2143	A
6	BICF2P847827	45221393	A	0	0.2143	G
6	TIGRP2P83338_rs8764544	45238852	G	0.5	0.2857	A
6	BICF2P1044167	45239383	C	0.5	0.2857	A
6	BICF2S23335797	45263678	A	0.5	0.2857	G
6	TIGRP2P83343_rs8934741	45269837	G	1	0.8333	A
6	BICF2P724165	45270247	C	0.5	0.2857	A
6	BICF2P829871	45286606	A	0.5	0.2857	G
6	TIGRP2P83378_rs9061250	45308601	A	0.5	0.2857	G
6	TIGRP2P83381_rs8521667	45318935	G	0.5	0.2857	A
6	BICF2P959003	45326706	C	0.5	0.2857	G
6	BICF2P211351	45333329	G	0.5	0.2857	A
6	TIGRP2P83385_rs8853062	45345813	A	0.5	0.2857	G
6	BICF2S22936827	45371197	0	0	0	A
6	BICF2S23657959	45390026	G	0	0	A
6	BICF2P540128	45395030	A	0	0	G
6	TIGRP2P83398_rs8748884	45405447	A	0.5	0.2857	G
6	BICF2P752394	45418245	C	0.5	0.2857	A
6	TIGRP2P83404_rs9037460	45442278	A	0.5	0.2857	G
6	BICF2S2358335	45451813	G	0.5	0.7143	C
6	BICF2P576655	45481486	G	0.5	0.2857	A
6	TIGRP2P83408_rs9007050	45485982	A	0.5	0.5	C
6	BICF2P1128536	45502090	A	0	0	T
6	BICF2P542453	45514290	G	0	0	A
6	TIGRP2P83415_rs8804615	45523037	G	0.5	0.5	A
6	BICF2P1285401	45537541	G	0.5	0.5	A
6	BICF2P4446	45546506	0	0	0	A
6	BICF2S2356387	45565916	C	0	0	A

6	BICF2P1191784	45584496	0	0	0	G
6	BICF2P1278057	45588807	0	0	0	C
6	BICF2S23522279	45598714	A	0	0	G
6	TIGRP2P83425_rs8584594	45616526	A	0	0	G
6	BICF2P326117	45626014	G	0	0	A
6	BICF2P673707	45639169	A	0	0	G
6	BICF2P79320	45646399	A	0	0	G
6	TIGRP2P83437_rs9203777	45648696	G	0.5	0.5	A
6	BICF2P1245285	45657062	C	0	0	A
6	BICF2P800622	45662210	G	0	0	A
6	BICF2P334641	45681961	A	0.5	0.5	C
6	BICF2S229195	45694092	A	0	0	G
6	BICF2S23746911	45708756	G	0.5	0.5	A
6	BICF2P263269	45717167	A	0.5	0.5	C
6	TIGRP2P83480_rs8947391	45741262	0	0	0	G
6	BICF2P384881	45757001	A	0.5	0.5	T
6	BICF2S22918393	45759310	G	0	0.2143	A
6	BICF2P1016467	45779658	G	0	0	A
6	BICF2P684324	45793691	G	0	0	C
6	BICF2P860611	45809304	A	0	0	G
6	BICF2P545637	45817036	G	0	0	A
6	BICF2P448508	45830328	A	0	0	G
6	BICF2P613899	45842240	A	0	0	C
6	BICF2P1161279	45858168	A	0	0	G
6	BICF2P1242528	45863274	G	0	0	A
6	BICF2P414454	45876941	A	0	0	G
6	BICF2S2369232	45885564	A	0	0	G
6	BICF2P1247084	45920235	G	0	0	A
6	BICF2P124860	45944706	0	0	0	A
6	BICF2S23725012	45960779	A	0	0	G
6	BICF2P571355	45967090	C	0.5	0.7143	A
6	BICF2P1306687	45981789	A	0	0	G
6	BICF2P278088	45988803	0	0	0	G
6	BICF2P392977	46000101	0	0	0	A
6	TIGRP2P83499_rs8742017	46018998	G	0.5	0.7143	A
6	BICF2P1166715	46028473	A	0	0	G
6	BICF2S23123071	46038590	A	0.5	0.7143	C
6	BICF2P82228	46052557	A	0.5	0.7143	G
6	BICF2S2354522	46064414	G	0	0	A
6	BICF2P493305	46086113	0	0	0	G
6	TIGRP2P83513_rs8922998	46102620	C	1	1	G
6	BICF2P1387781	46111441	A	1	1	T
6	BICF2G630583170	46113969	A	0	0	G
6	BICF2P543630	46159294	T	0	0	A
6	BICF2P1055256	46172183	A	0	0	G
6	BICF2P252371	46174960	G	0.5	0.7857	A
6	BICF2S2301470	46189211	A	0.5	0.2143	G

6	BICF2P769363	46196396	G	0.5	0.2143	A
6	G409f20S151	46207632	G	0	0	A
6	BICF2P398823	46237398	0	0	0	G
6	BICF2G630582945	46241784	G	0	0	A
6	BICF2S2411308	46260837	0	0	0	A
6	BICF2S23619636	46286303	A	0	0	G
6	BICF2S23429553	46288605	0	0	0	G
6	BICF2G630582935	46302186	A	1	1	G
6	BICF2G630582896	46324309	G	0	0	A
6	BICF2G630582883	46337424	G	1	1	A
6	BICF2S23744983	46357393	A	0	0	G
6	BICF2G630582862	46377890	A	0	0	G
6	BICF2S2396561	46384614	A	0	0	G
6	BICF2P349207	46409947	0	0	0	A
6	TIGRP2P83587_rs8717585	46417148	G	0	0	A
6	BICF2G630582858	46417330	A	0	0	C
6	BICF2G630582850	46428414	G	0	0	A
6	BICF2P580504	46438333	G	0	0	A
6	BICF2G630582828	46451659	G	0	0	A
6	BICF2G630582813	46462966	C	0	0	A
6	BICF2P1360670	46481714	A	0.5	0.2	G
6	BICF2G630582785	46486559	G	0.5	0.7143	A
6	TIGRP2P83621_rs8670219	46509617	A	0.5	0.2857	C
6	BICF2P179473	46513276	A	0.5	0.25	G
6	BICF2G630582770	46521889	C	1	1	A
6	BICF2G630582765	46529263	A	0.5	0.7143	G
6	BICF2G630582749	46553202	G	0	0	A
6	BICF2S2312826	46573447	G	0.5	0.2857	A
6	BICF2P1208338	46576643	A	0.5	0.7143	G
6	BICF2S23231431	46589392	A	0.5	0.5	C
6	BICF2G630582713	46600521	A	0	0	G
6	BICF2P100296	46612866	A	0	0	G
6	BICF2S23627125	46627286	A	0	0	G
6	BICF2G630582681	46634603	A	0	0.2143	G
6	BICF2G630582674	46644010	A	0.5	0.2857	G
6	BICF2G630582669	46676229	G	0.5	0.5	A
6	TIGRP2P83666_rs9087317	46687254	A	0	0.2143	G
6	BICF2P1346280	46700129	0	0	0	G
6	TIGRP2P83668_rs8720923	46702560	G	0	0	A
6	BICF2P783189	46721318	G	0.5	0.5	A
6	BICF2P989329	46724877	A	0.5	0.5	C
6	BICF2P989331	46725107	G	0.5	0.5	A
6	BICF2S23410961	46727399	A	0.5	0.5	G
6	BICF2S23410964	46727686	G	0.5	0.5	A
6	BICF2S23548750	46736825	A	0.5	0.5	G
6	BICF2P157571	46756392	A	0.5	0.5	T
6	BICF2G630582665	46762916	A	0.5	0.5	G

6	BICF2P190896	46776467	A	0	0	G
6	BICF2P409388	46792939	G	0	0	A
6	TIGRP2P83694_rs9009157	46811136	G	0.5	0.7143	A
6	BICF2P533256	46821059	A	0	0	G
6	TIGRP2P83701_rs8899721	46830649	G	0.5	0.7143	A
6	TIGRP2P83707_rs9059362	46844067	G	0.5	0.7143	A
6	BICF2P1189358	46851035	G	0.5	0.5	A
6	BICF2G630582664	46867218	A	0	0	G
6	BICF2S2346993	46881279	G	0	0.2143	A
6	BICF2P1248138	46890293	0	0	0	A
6	BICF2P355023	46899944	0	0	0	T
6	BICF2P1322179	46909750	0	0	0	A
6	BICF2P865490	46935722	G	0.5	0.7143	A
6	BICF2P1220403	46938206	G	0.5	0.7143	A
6	BICF2P1220419	46943133	A	0.5	0.7143	G
6	BICF2P613887	46959061	A	0	0.2143	G
6	BICF2G630582659	46966648	A	0	0	C
6	TIGRP2P83749_rs8674599	46978926	A	0	0.2143	C
6	BICF2S2316381	46994759	G	0.5	0.7143	A
6	BICF2S2312096	47010192	A	0	0	G
6	BICF2S23510682	47017805	A	0.5	0.7143	G
6	TIGRP2P83763_rs8556585	47027453	A	0	0	G
6	BICF2P1451427	47037718	G	0.5	0.75	A
6	BICF2G630582647	47048879	A	0	0	G
6	BICF2G630582612	47066688	G	0	0.1667	A
6	BICF2G630582600	47070408	G	0.5	0.7143	A
6	BICF2G630582579	47083257	G	0.5	0.7143	A
6	BICF2P653078	47092714	A	1	1	G
6	BICF2P1008950	47112247	A	0.5	0.5	G
6	BICF2P1359976	47119206	G	0.5	0.5	A
6	BICF2P101763	47127895	A	0.5	0.5	G
6	BICF2G630582555	47149732	G	0.5	0.5	A
6	BICF2P1460534	47152278	G	0.5	0.5	A
6	BICF2G630582545	47165932	C	0	0.2143	A
6	BICF2G630582528	47174046	A	0	0	G
6	BICF2G630582513	47186240	A	0	0	G
6	BICF2G630582502	47193832	G	0	0	A
6	BICF2G630582499	47196549	A	0	0.2143	G
6	BICF2P1047812	47209971	0	0	0	G
6	BICF2S23241318	47227461	G	0	0	A
6	BICF2G630582485	47231693	G	0	0	A
6	TIGRP2P83807_rs8725065	47250562	0	0	0	A
6	BICF2G630582483	47275149	G	0.5	0.5	A
6	BICF2G630582474	47285674	A	0	0	G
6	BICF2S23532442	47298074	G	0	0	A
6	BICF2G630582465	47304818	A	0	0	G
6	BICF2G630582461	47323335	A	0	0.2143	T

6	BICF2S23152321	47341662	G	0	0	A
6	BICF2G630582457	47350941	A	0	0.2143	T
6	BICF2G630582447	47358162	A	0	0.2143	G
6	BICF2G630582433	47377782	T	0	0.2143	A
6	BICF2G630582430	47378348	C	0	0	A
6	BICF2G630582423	47380062	G	0	0	A
6	BICF2G630582390	47394370	C	0	0	G
6	BICF2G630582370	47411590	G	1	0.7857	A
6	BICF2G630582352	47425617	A	0.5	0.5	G
6	BICF2S23058224	47428212	G	0.5	0.5	A
6	BICF2G630582336	47445318	G	0.5	0.5	A
6	BICF2G630582334	47451965	A	0	0	G
6	BICF2S23616949	47461051	A	0	0	G
6	BICF2G630582314	47474985	A	1	0.7857	G
6	BICF2G630582297	47484024	G	0.5	0.5	A
6	BICF2G630582287	47499702	A	0.5	0.5	G
6	BICF2G630582267	47513385	A	0.5	0.5	G
6	BICF2G630582253	47518001	A	0	0	G
6	BICF2G630582227	47531282	A	0	0.2143	G
6	BICF2P1333121	47547864	A	0	0	G
6	BICF2P66202	47560062	A	0	0	C
6	BICF2P1007646	47569732	G	0.5	0.5	A
6	BICF2P1118843	47585210	G	0	0	A
6	BICF2P1167508	47588740	C	0	0	A
6	BICF2P1137213	47609802	G	0.5	0.2857	A
6	BICF2S23657099	47610159	A	0.5	0.7143	G
6	BICF2P146897	47621605	G	0	0.2143	A
6	BICF2P127827	47642241	0	0	0	A
6	BICF2P1054196	47645414	G	0.5	0.7143	A
6	BICF2P8422	47657027	A	0.5	0.2857	C
6	BICF2S23325321	47672181	A	0.5	0.2857	G
6	TIGRP2P83838_rs9050267	47686913	C	0.5	0.5	A
6	BICF2G630582189	47703928	G	0	0.25	A
6	BICF2P511193	47719197	A	0	0	G
6	BICF2G630582170	47740449	G	0	0.2143	A
6	BICF2P1406286	47765541	A	0	0	C
6	BICF2G630582163	47772856	A	0.5	0.7143	C
6	BICF2G630582161	47773599	G	0.5	0.7143	A
6	BICF2G630582153	47785482	A	0.5	0.7143	G
6	BICF2G630582143	47798202	A	0	0.2143	G
6	BICF2G630582137	47809260	A	0.5	0.7143	G
6	BICF2G630582127	47822338	G	0	0.2143	A
6	BICF2G630582116	47831580	A	0	0.2143	T
6	BICF2G630582097	47841295	G	0	0.2143	A
6	BICF2G630582077	47852295	A	0	0.2143	G
6	BICF2P567177	47870597	0	0	0	G
6	BICF2G630582055	47883316	G	0	0.2143	A

6	BICF2G630582044	47896705	A	0.5	0.7143	G
6	BICF2S23027501	47904110	G	0	0	A
6	BICF2G630582030	47913040	G	0	0	A
6	BICF2P387874	47915871	G	0.5	0.5	A
6	BICF2G630582015	47920640	A	0.5	0.5	G
6	BICF2G630581994	47940117	A	0	0	G
6	BICF2P544401	47949507	0	0	0	A
6	BICF2G630581984	47964300	A	0	0	C
6	BICF2S23639529	47975941	A	0	0.2143	G
6	BICF2G630581961	47979203	A	0	0	G
6	BICF2S23636696	47996686	A	0	0.2143	G
6	BICF2G630581934	48008750	G	0	0	A
6	BICF2G630581925	48018321	A	0.5	0.7143	G
6	BICF2G630581897	48032471	A	0.5	0.7143	G
6	BICF2P851629	48039192	A	0	0	G
6	BICF2S23258979	48048470	G	0.5	0.7143	A
6	BICF2S23554976	48067262	G	0.5	0.7143	A
6	BICF2S23710284	48080996	A	0	0	G
6	BICF2G630581857	48092675	A	0	0.2143	G
6	BICF2G630581856	48093457	A	0	0.2143	G
6	BICF2P697055	48114045	0	0	0	C
6	BICF2P1003157	48159909	A	0	0.2143	G
6	BICF2P1102956	48171288	A	0.5	0.5	G
6	BICF2S23013662	48177785	A	0	0	G
6	BICF2G630581834	48191766	G	0	0	A
6	BICF2P393964	48204148	A	0	0	G
6	TIGRP2P83859_rs9113657	48229660	0	0	0	C
6	BICF2S2311481	48231136	A	0	0.1	T
6	BICF2G630581819	48245639	C	0	0.2143	A
6	BICF2G630581814	48255905	G	0	0.2143	C
6	BICF2G630581789	48267526	A	0	0.2143	C
6	BICF2G630581772	48279291	G	0	0.2143	A
6	BICF2G630581752	48292569	A	0	0.2143	G
6	BICF2G630581742	48302285	G	0	0.2143	A
6	BICF2G630581720	48313162	G	0	0.2143	A
6	BICF2G630581699	48324366	G	0	0.2143	A
6	BICF2G630581677	48343895	A	1	0.7857	G
6	BICF2P603223	48357138	G	0	0	A
6	BICF2G630581673	48358157	A	0	0	G
6	G410f46S266	48398094	0	0	0	C
6	TIGRP2P83865_rs8855498	48406280	A	0	0	G
6	TIGRP2P83866_rs9140083	48420393	C	0	0	A
6	BICF2G630581661	48426674	G	0	0	A
6	BICF2G630581649	48450847	G	0	0	C
6	TIGRP2P83874_rs8897028	48466647	A	0.5	0.5714	G
6	BICF2G630581581	48479402	A	0	0.2143	C
6	BICF2G630581556	48485914	A	0	0.1667	G

6	BICF2G630581538	48497029	A	0.5	0.2857	G
6	BICF2G630581503	48520737	A	0.5	0.2857	C
6	TIGRP2P83893_rs8732055	48537551	A	0	0	G
6	TIGRP2P83899_rs8574273	48545314	A	0	0	C
6	BICF2G630581461	48558150	A	0	0.2143	G
6	BICF2P1231142	48565734	G	0	0	A
6	BICF2G630581404	48581241	A	1	0.7857	G
6	BICF2G630581361	48605508	G	0	0	A
6	BICF2G630581353	48610629	G	1	0.7857	A
6	BICF2G630581313	48631630	G	1	0.7857	A
6	BICF2G630581308	48635309	A	0	0.2143	G
6	BICF2G630581290	48649281	G	1	0.7857	A
6	BICF2G630581278	48658266	A	0	0	G
6	BICF2G630581256	48676742	A	0	0	G
6	BICF2G630581243	48689171	A	0	0	G
6	BICF2G630581237	48694538	G	0	0	A
6	BICF2P1103323	48708440	G	0	0	A
6	BICF2G630581200	48718465	A	0	0	G
6	BICF2P463689	48731534	0	0	0	G
6	BICF2G630581155	48745323	A	0	0	G
6	BICF2G630581152	48750619	G	0	0	A
6	BICF2G630581124	48763898	A	0	0	T
6	BICF2G630581104	48774446	G	0	0	A
6	BICF2G630581090	48792294	A	0	0.2143	G
6	BICF2G630581074	48803152	C	0	0.2143	G
6	BICF2G630581070	48806498	G	0	0	A
6	BICF2G630581030	48817770	C	0	0	A
6	BICF2G630580994	48834628	G	0	0	A
6	BICF2G630580979	48847143	G	0	0	A
6	BICF2G630580959	48854142	A	0	0	G
6	BICF2G630580924	48863621	G	0	0	A
6	BICF2G630580903	48877877	A	0	0.1429	G
6	BICF2G630580879	48887492	G	0	0.1429	A
6	BICF2G630580873	48907705	G	0	0.1429	A
6	BICF2G630580868	48916088	A	0	0.1429	G
6	BICF2P326433	48924088	G	0	0	C
6	BICF2P1298264	48938931	G	0	0	A
6	BICF2G630580816	48947374	G	0	0.1429	A
6	BICF2G630580796	48958476	C	0	0	A
6	BICF2G630580763	48968287	A	0	0	C
6	BICF2S23313448	48984367	T	1	0.7857	A
6	BICF2G630580739	48992720	G	0	0	A
6	BICF2G630580738	49003572	A	0	0	C
6	G411f35S91	49028457	G	0	0.1429	A
6	TIGRP2P84012_rs9030224	49037573	T	0	0.1429	A
6	BICF2G630580730	49053507	G	1	0.7857	A
6	BICF2G630580692	49065111	A	0	0	G

6	BICF2G630580671	49076407	A	0	0	G
6	BICF2G630580657	49089177	C	0	0	A
6	BICF2G630580644	49096835	G	0	0.1429	A
6	BICF2G630580603	49115442	A	0	0	G
6	BICF2G630580587	49123797	A	0	0	G
6	BICF2G630580560	49134858	G	0	0.1429	A
6	BICF2G630580556	49139715	G	0	0.1429	A
6	BICF2G630580536	49151401	G	0	0.1429	A
6	BICF2G630580494	49166663	A	0	0	G
6	TIGRP2P84030_rs8652786	49177620	G	0	0.1429	A
6	BICF2G630580456	49186053	G	0	0.1429	A
6	BICF2G630580436	49206902	G	0	0.1429	A
6	BICF2G630580426	49210554	A	0	0	G
6	BICF2G630580413	49223347	C	0	0.2143	A
6	BICF2G630580390	49232855	G	0	0.1429	A
6	BICF2G630580358	49265837	A	0.5	0.3571	G
6	TIGRP2P84035_rs8936608	49266772	A	0.5	0.5714	G
6	BICF2P277513	49277708	A	0.5	0.5714	C
6	BICF2G630580319	49292521	G	0.5	0.3571	A
6	BICF2G630580310	49303094	G	0	0.2143	A
6	BICF2P205874	49316258	C	0	0	A
6	TIGRP2P84044_rs8553175	49333257	0	0	0	A
6	BICF2G630580305	49337568	G	0	0	A
6	BICF2G630580278	49352402	G	0	0	A
6	BICF2G630580265	49358113	A	0	0.2143	G
6	BICF2G630580261	49375557	C	0.5	0.5	A
6	BICF2G630580236	49381013	G	0.5	0.5	A
6	BICF2G630580229	49396340	0	0	0	A
6	BICF2G630580209	49408294	A	0.5	0.5	G
6	BICF2G630580164	49429543	A	0.5	0.5	G
6	BICF2G630580126	49441876	A	0.5	0.5	G
6	BICF2G630580110	49450836	C	0.5	0.4167	A
6	BICF2G630580099	49470952	A	1	0.8333	C
6	BICF2S23637916	49476811	A	0	0	G
6	BICF2G630580085	49487556	A	0	0.2143	G
6	BICF2G630580066	49497052	A	0	0	G
6	BICF2G630580036	49508640	A	0.5	0.5714	G
6	TIGRP2P84083_rs8847582	49526827	C	1	0.8571	A
6	BICF2P893515	49527485	A	0.5	0.5714	G
6	BICF2P893514	49527525	C	1	0.8571	A
6	BICF2G630580026	49531469	G	0	0.1429	A
6	TIGRP2P84098_rs8763809	49542664	A	0	0.1429	G
6	BICF2P233539	49575223	A	0	0.2143	C
6	BICF2P897008	49581353	A	0	0.2143	G
6	BICF2G630580017	49589832	G	1	0.7857	A
6	BICF2G630579994	49601671	A	0	0	G
6	BICF2G630579938	49617679	G	0	0.1429	A

6	BICF2G630579880	49631898	G	0	0.2143	A
6	BICF2G630579863	49635652	A	0	0.2143	G
6	BICF2G630579834	49645556	G	0.5	0.5	A
6	BICF2G630579774	49669755	G	0	0	A
6	BICF2G630579755	49680642	G	0	0.1429	A
6	BICF2G630579708	49691970	A	0	0.1429	G
6	BICF2G630579687	49703690	A	0	0.2143	G
6	BICF2G630579655	49718157	G	0	0.1667	A
6	BICF2G630579640	49729171	C	0	0	A
6	BICF2G630579617	49737814	A	0	0.1429	G
6	BICF2G630579604	49749115	G	0	0.2143	A
6	BICF2G630579578	49762025	G	0	0.2143	A
6	BICF2G630579546	49777952	G	0	0.2143	A
6	BICF2G630579521	49791754	G	0	0.2143	A
6	BICF2G630579503	49796598	A	0	0.2143	G
6	BICF2G630579438	49811933	G	0	0.1429	A
6	BICF2G630579427	49819918	G	0	0.1429	A
6	BICF2G630579388	49832241	G	0	0.1429	A
6	BICF2G630579376	49844494	G	0	0.1429	A
6	BICF2S23645696	49862835	0	0	0	A
6	BICF2G630579345	49864519	G	0	0	A
6	BICF2G630579298	49878134	G	0	0.1429	A
6	BICF2G630579239	49897116	G	0	0.1429	A
6	BICF2G630579206	49904862	C	0	0	A
6	BICF2G630579181	49910864	C	0	0	A
6	BICF2G630579147	49929983	G	0	0	A
6	BICF2G630579143	49939993	A	0	0	C
6	BICF2G630579115	49951873	A	0	0	G
6	BICF2S23418149	49963509	0	0	0	A
6	BICF2G630579063	49974800	A	0	0	G
6	BICF2G630579054	49980725	G	0	0	A
6	BICF2G630579022	49994795	C	0	0	G
6	BICF2G630579015	50002986	A	0	0	G
6	BICF2S23128755	50014113	A	0	0	G
6	BICF2G630578878	50038983	G	0	0	A
6	BICF2G630578834	50054324	A	0	0	G
6	BICF2G630578764	50070854	G	0	0	A
6	BICF2G630578757	50072604	A	0	0	G
6	BICF2G630578739	50074785	G	0	0	A
6	BICF2G630578701	50081582	G	0	0	A
6	BICF2G630578688	50083337	G	0	0	C
6	BICF2S2364759	50096774	A	0	0	G
6	BICF2G630578654	50107043	A	0	0.1667	C
6	BICF2G630578645	50117443	G	0	0.2143	A
6	BICF2G630578634	50138041	G	0	0.2143	A
6	BICF2G630578631	50140939	A	0	0.2143	G
6	BICF2G630578618	50151607	A	0	0.2143	G

6	BICF2P364371	50178771	0	0	0	G
6	BICF2S24111128	50207512	0	0	0	T
6	TIGRP2P84170_rs8724995	50224070	0	0	0	C
6	BICF2G630578597	50236868	G	0	0	A
6	TIGRP2P84172_rs8855256	50250458	0	0	0	A
6	BICF2P906181	50275399	0	0	0	A
6	BICF2S24414109	50279603	A	0	0	G
6	BICF2P1414726	50289884	0	0	0	A
6	BICF2S23657635	50305655	A	0	0	G
6	BICF2P435171	50325246	0	0	0	A
6	BICF2P324601	50346779	0	0	0	A
6	BICF2P674423	50355207	0	0	0	A
6	BICF2S2446666	50368346	C	0	0	A
6	BICF2P779463	50392548	0	0	0	A
6	BICF2S23216050	50398634	G	0	0	A
6	BICF2S2419161	50417782	0	0	0	A
6	BICF2P1348390	50434290	G	0	0	A
6	TIGRP2P84183_rs8459244	50442468	A	0	0	G
6	BICF2S23747486	50462501	G	0.5	0.5	A
6	BICF2G630578581	50476532	A	0.5	0.5	C
6	BICF2P1192352	50486970	0	0	0	G
6	TIGRP2P84195_rs8953406	50530784	G	0.5	0.3333	A
6	BICF2P605383	50541379	G	0.5	0.5	A
6	TIGRP2P84204_rs9131713	50545691	A	0.5	0.5	C
6	BICF2P786254	50559320	A	0.5	0.5	C
6	BICF2P1111847	50565561	T	0.5	0.5	A
6	BICF2P1407101	50580649	A	0.5	0.5	T
6	BICF2P341544	50596830	A	0.5	0.5	C
6	BICF2S23512255	50607813	A	0	0	G
6	BICF2P129158	50613518	0	0	0	A
6	BICF2G630578570	50626848	G	0.5	0.3571	A
6	BICF2S2371165	50637655	A	0.5	0.3571	G
6	BICF2P402007	50651168	0	0	0	C
6	BICF2P793490	50657972	G	0.5	0.6429	A
6	BICF2P186757	50669892	0	0	0	G
6	BICF2G630578544	50686527	G	0	0.1429	A
6	BICF2P772149	50695929	A	0	0.1429	G
6	BICF2P1271985	50711296	A	0.5	0.5	G
6	BICF2G630578515	50718190	A	0.5	0.3333	G
6	BICF2P325270	50735161	C	0.5	0.7	A
6	BICF2S2326998	50745973	A	0.5	0.6429	G
6	BICF2S2342454	50754948	0	0	0	A
6	BICF2P271370	50769707	G	0.5	0.6429	A
6	BICF2P759150	50774028	A	0	0	G
6	BICF2G630578492	50788895	A	0.5	0.3571	G
6	BICF2S23419786	50794847	G	0.5	0.25	A
6	BICF2S23439549	50795906	G	0.5	0.3571	A

6	BICF2P1412857	50817118	A	1	1	G
6	TIGRP2P84267_rs8689818	50820138	0	0	0	A
6	BICF2S23543302	50840709	G	0	0	A
6	BICF2G630578460	50850133	G	0.5	0.6429	A
6	BICF2G630578446	50853880	C	0.5	0.6429	A
6	BICF2G630578426	50873401	T	0.5	0.5	A
6	BICF2P217427	50878953	G	0	0	A
6	BICF2G630578396	50896306	A	1	1	G
6	BICF2G630578392	50899201	A	0	0.1429	G
6	BICF2G630578369	50919952	A	0	0.1429	G
6	BICF2G630578361	50925380	A	0	0.1429	G
6	TIGRP2P84294_rs9081125	50937008	A	0	0.1429	G
6	BICF2S23324600	50948226	G	0	0.1429	A
6	BICF2G630578312	50963604	G	0	0.1429	A
6	BICF2G630578299	50970899	A	0	0.1429	G
6	BICF2G630578274	50979981	A	0	0.1429	G
6	TIGRP2P84392_rs8800484	51009395	A	0.5	0.5	G
6	BICF2G630578228	51015592	A	0	0	C
6	BICF2G630578215	51025719	G	0.5	0.5	A
6	BICF2G630578200	51039873	A	0.5	0.5	G
6	BICF2G630578175	51054684	A	0	0	G
6	BICF2G630578162	51062253	A	0	0	C
6	BICF2P1298576	51071891	A	0.5	0.3333	G
6	TIGRP2P84404_rs8702631	51083561	0	0	0	A
6	BICF2G630578121	51102194	G	1	0.8571	A
6	BICF2G630578117	51108192	G	0.5	0.3571	A
6	BICF2P551638	51121317	G	0	0	A
6	BICF2G630578073	51137756	A	0.5	0.3	C
6	BICF2G630578061	51140531	G	0	0.1429	A
6	BICF2P616335	51155248	A	0	0	G
6	BICF2G630578016	51169192	A	0	0.1429	G
6	BICF2P1026090	51184949	G	0	0	A
6	BICF2P554732	51191303	C	0	0	A
6	BICF2P1363905	51206177	0	0	0	C
6	BICF2S23116185	51217907	A	0.5	0.5	G
6	BICF2P566631	51230949	C	0	0	A
6	BICF2S2457791	51234835	A	0	0	C
6	BICF2G630577981	51283942	G	0	0	A
6	BICF2G630577980	51284495	C	0	0	A
6	BICF2P199109	51293708	T	0.5	0.6429	A
6	BICF2G630577966	51303440	A	0.5	0.6429	G
6	BICF2G630577963	51313430	A	0	0	G
6	BICF2G630577952	51335380	A	0	0.1429	G
6	BICF2G630577950	51338730	G	0	0.1429	A
6	BICF2P1368320	51350686	A	0.5	0.5	G
6	BICF2G630577939	51378480	A	0	0	C
6	BICF2P925741	51383225	G	0	0	A

6	BICF2P164492	51402101	A	0	0	G
6	BICF2G630577893	51414407	C	0	0	A
6	BICF2G630577889	51417872	A	0.5	0.5	G
6	BICF2G630577831	51459305	A	0	0.1429	G
6	TIGRP2P84476_rs8955307	51463197	0	0	0	A
6	BICF2G630577827	51476844	A	0	0	C
6	BICF2G630577812	51490240	A	0	0	G
6	TIGRP2P84486_rs8875982	51503579	A	0	0	G
6	BICF2G630577804	51503980	T	0	0	A
6	BICF2G630577797	51511338	A	0	0	G
6	TIGRP2P84488_rs8947193	51521081	0	0	0	G
6	BICF2G630577760	51540241	A	0	0	G
6	BICF2G630577752	51546636	A	0	0	G
6	BICF2G630577737	51555175	A	0.5	0.6429	C
6	BICF2G630577701	51571538	A	0	0	G
6	BICF2G630577693	51578177	A	1	1	G
6	BICF2G630577680	51597706	G	1	1	A
6	BICF2G630577671	51604112	G	0	0	A
6	BICF2G630577644	51627437	T	0	0	A
6	BICF2G630577627	51637213	A	0.5	0.6429	G
6	BICF2G630577620	51643360	A	0	0	G
6	BICF2G630577611	51646503	G	0.5	0.6429	A
6	BICF2G630577568	51660838	G	0	0	C
6	BICF2G630577546	51669719	G	0.5	0.7143	A
6	BICF2G630577532	51683308	A	0.5	0.6429	C
6	BICF2P278281	51695202	G	0.5	0.6429	A
6	TIGRP2P84498_rs8849528	51722661	A	0	0	C
6	TIGRP2P84500_rs8888142	51727338	A	0	0	G
6	BICF2G630577512	51733135	G	1	1	A
6	BICF2G630577506	51738612	C	0	0	A
6	BICF2G630577487	51750565	A	0	0	G
6	BICF2G630577479	51756705	A	0	0	G
6	BICF2G630577462	51770071	C	0	0	A
6	BICF2G630577457	51772780	C	0.5	0.2857	A
6	BICF2G630577451	51775677	A	0.5	0.2857	C
6	BICF2G630577440	51789800	A	0	0.3571	C
6	BICF2G630577432	51806111	A	0.5	0.2857	G
6	BICF2G630577427	51808173	A	0	0	G
6	BICF2S23418290	51820056	A	0	0	G
6	BICF2S23422559	51836672	0	0	0	C
6	BICF2G630577405	51843795	G	0	0	A
6	BICF2G630577398	51854319	A	0	0	G
6	BICF2P1322717	51865767	G	0	0	A
6	BICF2G630577383	51884582	A	0.5	0.3571	G
6	BICF2S23032956	51890820	A	0.5	0.6	G
6	BICF2P915289	51894974	G	0.5	0.6429	A
6	BICF2P691612	51900124	A	0	0	G

6	BICF2G630577352	51909734	A	0.5	0.2857	G
6	BICF2G630577347	51912265	A	1	0.6429	G
6	TIGRP2P84554_rs8975894	51924408	G	0	0	A
6	BICF2G630577295	51939996	A	0	0	G
6	BICF2G630577270	51947882	A	1	0.6429	G
6	BICF2P634885	51957442	A	0	0	C
6	BICF2G630577232	51973593	A	0	0	G
6	BICF2G630577197	51981658	A	0	0	G
6	BICF2G630577173	51995662	G	0	0	A
6	BICF2G630577158	52003113	A	0	0	C
6	BICF2G630577146	52015962	G	0	0	A
6	BICF2P51505	52027328	G	0.5	0.2857	A
6	BICF2G630577114	52044851	A	1	1	G
6	BICF2P12489	52049289	A	0	0	G
6	BICF2P541894	52061435	A	0	0	G
6	BICF2S23529291	52077655	A	0	0	G
6	BICF2P288912	52087988	A	0	0	G
6	BICF2G630577080	52096843	G	0	0	A
6	BICF2G630577075	52117598	A	0	0	G
6	BICF2G630577072	52125165	G	0	0	A
6	BICF2P1112774	52129674	A	0.5	0.2857	C
6	BICF2P929029	52136027	A	0.5	0.7143	G
6	TIGRP2P84636_rs9035902	52143379	C	0.5	0.2857	G
6	BICF2P169102	52153060	0	0	0	G
6	BICF2G630577064	52170536	G	0	0	A
6	BICF2P172903	52181169	A	0	0	G
6	TIGRP2P84644_rs9233592	52197273	C	0	0	G
6	BICF2P1069575	52200607	G	0	0	A
6	BICF2G630577042	52214935	A	0	0	G
6	BICF2P984524	52223776	A	0.5	0.7143	G
6	BICF2G630577029	52234977	C	0	0	A
6	TIGRP2P84654_rs8720084	52252347	0	0	0	A
6	BICF2G630577009	52265062	A	0.5	0.7143	G
6	BICF2G630576972	52278619	A	0.5	0.75	G
6	BICF2P338483	52288436	A	0.5	0.2857	T
6	BICF2P338482	52288657	A	0.5	0.2857	G
6	BICF2G630576960	52291159	A	0.5	0.2857	C
6	BICF2G630576946	52306084	A	0.5	0.7143	G
6	BICF2G630576920	52321768	G	0	0	A
6	BICF2G630576908	52333535	A	0.5	0.2857	C
6	BICF2G630576895	52337365	A	0.5	0.2857	G
6	BICF2G630576890	52350556	G	0.5	0.7143	A
6	BICF2P1212653	52369165	A	0	0	G
6	BICF2G630576854	52382417	A	0	0	G
6	BICF2G630576850	52383371	A	0	0	G
6	BICF2G630576844	52396050	G	0.5	0.2857	A
6	BICF2G630576833	52410661	C	0.5	0.7143	A

6	BICF2S23617567	52422833	A	0	0	G
6	BICF2G630576824	52432892	A	0.5	0.7143	G
6	BICF2P295337	52442125	A	0.5	0.7143	G
6	BICF2P1362974	52453299	A	0	0	G
6	BICF2P1433834	52469228	A	1	1	G
6	TIGRP2P84701_rs8643101	52485199	A	0.5	0.2857	C
6	TIGRP2P84705_rs8643106	52486320	G	0	0	A
6	BICF2P474535	52506822	G	1	1	A
6	BICF2S23545979	52512733	A	0.5	0.2857	G
6	BICF2P1105809	52524835	A	0.5	0.7143	G
6	BICF2G630576803	52535518	G	1	1	A
6	BICF2G630576794	52544158	A	1	1	G
6	BICF2P160418	52562775	A	0	0	G
6	BICF2G630576791	52567747	G	1	1	A
6	BICF2P531913	52580054	A	0	0	C
6	BICF2P214621	52604442	A	0	0	C
6	BICF2P94943	52612624	A	0	0	G
6	BICF2S23655797	52627655	0	0	0	G
6	BICF2P1283940	52638182	0	0	0	G
6	BICF2P1118095	52656749	G	0.5	0.7143	A
6	BICF2P177398	52663707	A	0.5	0.2857	G
6	BICF2P1305621	52674840	A	0.5	0.7143	C
6	BICF2S22941240	52684600	G	0	0	A
6	BICF2P257719	52698333	A	0.5	0.7143	T
6	BICF2P1361899	52715583	A	0.5	0.7143	G
6	TIGRP2P84731_rs8605032	52727454	A	0	0	G
6	BICF2G630576778	52729788	A	0.5	0.2857	G
6	TIGRP2P84739_rs8779684	52742175	A	0.5	0.7143	G
6	BICF2G630576747	52750631	G	0.5	0.2857	A
6	BICF2G630576738	52769768	A	0.5	0.2857	G
6	BICF2G630576734	52779610	G	0.5	0.2857	A
6	BICF2G630576731	52788515	G	0.5	0.2857	A
6	BICF2G630576721	52799638	A	1	1	G
6	BICF2G630576706	52813844	C	0.5	0.2857	A
6	BICF2G630576688	52834998	A	0.5	0.2857	G
6	BICF2G630576682	52836996	G	1	1	A
6	BICF2G630576674	52842531	G	1	1	A
6	BICF2G630576647	52863566	C	1	1	A
6	BICF2G630576629	52871312	C	1	1	G
6	BICF2G630576606	52883209	A	0	0	G
6	BICF2G630576596	52888783	G	0.5	0.2857	A
6	BICF2G630576578	52907341	G	0.5	0.2857	A
6	BICF2G630576559	52912200	G	1	1	A
6	BICF2G630576537	52932940	A	1	1	G
6	BICF2G630576528	52938469	A	0.5	0.2857	G
6	BICF2G630576487	52964535	G	1	1	A
6	BICF2G630576482	52968371	C	0.5	0.2857	G

6	BICF2G630576480	52969027	A	0.5	0.2857	G
6	BICF2G630576452	52988169	C	1	1	A
6	BICF2G630576438	53006326	G	1	1	A
6	BICF2G630576435	53015071	G	0.5	0.2857	A
6	BICF2G630576425	53026592	A	0	0	G
6	BICF2G630576409	53042303	A	0.5	0.7143	G
6	BICF2P333587	53057385	G	0	0	A
6	BICF2P1393573	53067409	A	0	0	T
6	TIGRP2P84804_rs8990326	53081128	A	0	0	G
6	BICF2G630576400	53087342	G	1	1	A
6	BICF2G630576390	53095593	A	1	1	G
6	BICF2P809532	53118464	A	0	0	G
6	BICF2G630576380	53124600	G	1	1	A
6	BICF2G630576375	53131323	A	0	0	C
6	BICF2G630576361	53142559	G	1	1	A
6	BICF2G630576356	53153148	G	1	1	A
6	BICF2P1045560	53169373	G	0	0	A
6	BICF2G630576339	53187184	G	0	0	C
6	BICF2G630576336	53188634	A	1	1	G
6	BICF2G630576274	53215078	G	0	0	A
6	BICF2G630576250	53226445	G	1	1	A
6	BICF2G630576239	53237859	A	0.5	0.2857	G
6	BICF2G630576224	53245032	A	0.5	0.2857	G
6	BICF2S23740454	53259195	G	0	0	A
6	BICF2P3211	53268101	G	0	0	A
6	BICF2G630576208	53290680	A	0	0	G
6	BICF2G630576204	53292047	A	0	0	G
6	BICF2G630576167	53307932	G	0	0	A
6	TIGRP2P84845_rs8982082	53314754	A	0	0	G
6	BICF2G630576137	53329036	A	0	0	G
6	BICF2S22947179	53346387	G	1	1	A
6	BICF2G630576100	53356635	G	1	1	A
6	BICF2S23633861	53360612	G	1	1	A
6	BICF2G630576083	53380948	G	1	1	A
6	TIGRP2P84861_rs8945793	53386080	G	1	1	A
6	BICF2S23761939	53396218	A	0	0	G
6	TIGRP2P84878_rs9003572	53409520	G	0.5	0.7143	A
6	BICF2S23356748	53419205	G	0.5	0.2857	A
6	BICF2G630575998	53430748	G	0	0	A
6	BICF2P258438	53442836	A	1	1	G
6	BICF2P521021	53452628	A	1	1	G
6	BICF2G630575948	53473666	A	1	1	G
6	BICF2S23631820	53475767	A	0	0	G
6	BICF2P485450	53488517	A	0	0	G
6	BICF2P322981	53495082	A	1	1	G
6	BICF2P168632	53522814	0	0	0	A
6	BICF2G630575895	53551549	C	0.5	0.7143	A

6	BICF2G630575885	53555592	A	NA	0.75	G
6	BICF2S23532171	53569235	A	1	1	G
6	TIGRP2P84896_rs8915620	53587281	G	0	0	A
6	BICF2P1281162	53595416	A	0	0	G
6	BICF2G630575831	53619501	A	0.5	0.7143	G
6	BICF2P817950	53635521	A	0	0	G
6	BICF2P1203942	53639316	0	0	0	A
6	BICF2G630575826	53652880	A	0.5	0.7143	G
6	BICF2G630575818	53659642	G	0.5	0.7143	A
6	BICF2G630575763	53673448	A	0	0	G
6	BICF2G630575750	53682675	G	0.5	0.75	A
6	BICF2P185914	53695964	C	0.5	0.7143	A
6	BICF2S2304082	53714602	C	0	0	A
6	TIGRP2P84935_rs8965902	53723110	G	0	0	A
6	BICF2G630575734	53741377	G	0.5	0.2857	A
6	BICF2P953913	53757867	0	0	0	A
6	BICF2S2308	53766240	A	0	0	G
6	BICF2G630575725	53781339	A	0	0	G
6	BICF2G630575716	53785752	G	1	1	A
6	TIGRP2P84946_rs9024454	53801929	A	0	0	G
6	BICF2G630575691	53810433	G	0	0	A
6	BICF2G630575673	53826692	0	0	0	A
6	BICF2G630575665	53833910	C	0	0	A
6	BICF2P213833	53843186	A	0.5	0.2857	G
6	BICF2P1060954	53856926	0	0	0	A
6	BICF2P563608	53871123	0	0	0	C
6	BICF2P1236966	53880125	A	0	0	G
6	BICF2P28105	53900332	A	0	0	G
6	BICF2P158215	53912628	A	0	0	G
6	TIGRP2P84968_rs8957998	53938830	A	0	0	G
6	BICF2P1398108	53950551	A	0.5	0.2857	T
6	BICF2P993965	53969460	0	0	0	G
6	BICF2P619396	53971627	0	0	0	A
6	BICF2G630575652	54009463	C	0	0	A
6	TIGRP2P84984_rs8839568	54016561	A	0	0	G
6	BICF2G630575651	54032967	A	1	1	G
6	BICF2G630575634	54040428	A	0	0	C
6	BICF2G630575598	54059876	G	0.5	0.7143	A
6	BICF2G630575571	54067177	A	0	0	G
6	BICF2G630575558	54074477	G	1	1	A
6	BICF2G630575548	54088717	G	0	0	A
6	BICF2G630575533	54103440	A	0	0	G
6	BICF2P1341132	54115107	A	1	1	G
6	BICF2P728719	54123337	G	1	1	A
6	BICF2P997808	54128126	A	0.5	0.7143	G
6	BICF2P712807	54141175	A	1	1	G
6	BICF2P685260	54144247	A	0	0	G

6	BICF2G630575510	54153757	A	0	0	G
6	BICF2G630575509	54169758	G	0.5	0.7143	A
6	TIGRP2P85003_rs8720203	54179149	A	0	0	G
6	BICF2P225397	54194351	G	0.5	0.7143	A
6	BICF2P448519	54200648	A	0.5	0.7143	G
6	BICF2G630575494	54211643	A	0.5	0.7143	G
6	TIGRP2P85008_rs8734033	54228680	0	0	0	G
6	BICF2G630575466	54240588	A	1	1	G
6	BICF2G630575461	54246881	G	1	1	A
6	BICF2P980696	54261072	A	0.5	0.2857	C
6	BICF2G630575435	54269117	G	0	0	A
6	BICF2P999922	54290005	A	0.5	0.7143	G
6	BICF2G630575421	54299172	A	0	0	G
6	BICF2G630575416	54303398	A	0	0	G
6	BICF2G630575405	54320911	G	0	0	A
6	BICF2G630575388	54336831	A	0	0	G
6	BICF2G630575382	54339284	A	0	0	G
6	BICF2G630575374	54350507	C	0	0	A
6	BICF2G630575360	54363496	A	0	0	C
6	BICF2G630575355	54378284	A	0	0	G
6	TIGRP2P85020_rs9160626	54383769	C	0	0	A
6	BICF2G630575346	54396558	A	0.5	0.2857	C
6	BICF2G630575322	54416685	A	0	0	G
6	BICF2G630575313	54418627	G	0	0	A
6	BICF2P976137	54438203	0	0	0	A
6	BICF2P590161	54444573	0	0	0	A
6	BICF2P288146	54459443	A	0	0	G
6	BICF2P1236294	54464700	C	0.5	0.7143	A
6	TIGRP2P85044_rs8870141	54479316	C	0	0	A
6	BICF2P384039	54498064	A	0.5	0.2857	G
6	BICF2G630575302	54512560	A	0.5	0.7143	G
6	BICF2G630575297	54532293	A	0.5	0.7143	G
6	BICF2G630575290	54537447	A	0.5	0.2857	C
6	BICF2P132138	54544933	A	0	0	T
6	BICF2G630575261	54564052	A	1	1	G
6	BICF2P137010	54574117	A	0	0	C
6	TIGRP2P85082_rs8784431	54588388	0	0	0	C
6	BICF2P771101	54617654	A	0	0	G
6	BICF2P533226	54627247	A	0	0	G
6	TIGRP2P85087_rs8712094	54646441	A	0	0	G
6	BICF2P679664	54649761	A	1	1	C
6	BICF2P745019	54666768	0	0	0	A
6	BICF2P957550	54682442	A	0	0	G
6	TIGRP2P85091_rs8812117	54683066	A	0	0	G
6	BICF2G630575249	54701809	G	1	1	A
6	BICF2G630575244	54710685	A	1	1	G
6	BICF2G630575234	54723409	A	0	0	C

6	BICF2P87065	54728586	A	0	0	C
6	BICF2P422116	54746916	0	0	0	A
6	BICF2G630575217	54751912	A	1	1	G
6	BICF2P280561	54767902	0	0	0	A
6	BICF2G630575201	54793685	A	0	0	G
6	BICF2G630575189	54802519	A	0	0	G
6	BICF2G630575175	54811746	A	0	0	G
6	BICF2G630575152	54827758	G	0	0	A
6	BICF2G630575146	54832958	A	0	0	G
6	BICF2G630575115	54851466	C	0	0	A
6	BICF2P272395	54865227	A	0	0	G
6	BICF2G630575090	54871210	G	0	0	A
6	BICF2G630575086	54879083	A	0	0	C
6	BICF2G630575080	54889824	A	0.5	0.7143	G
6	BICF2P1418227	54904130	A	0	0	C
6	BICF2G630575060	54914460	C	0	0	A
6	BICF2P1010202	54929494	0	0	0	C
6	BICF2G630575042	54938312	A	0	0	C
6	TIGRP2P85128_rs8589584	54957208	0	0	0	T
6	TIGRP2P85129_rs8817889	54971598	A	0	0	G
6	BICF2G630575018	54982002	G	0	0	A
6	BICF2P236916	54994521	A	0	0	G
6	BICF2G630575002	55005755	A	0	0	G
6	BICF2G630574993	55018815	G	0	0	A
6	BICF2G630574973	55028670	A	0	0	G
6	BICF2S23515514	55039162	A	0	0	G
6	BICF2P542195	55079121	0	0	0	A
6	BICF2G630574952	55103756	A	0	0	T
6	BICF2P322771	55112332	0	0	0	T
6	G413f42S82	55134151	0	0	0	C
6	BICF2P939849	55173659	0	0	0	A
6	BICF2S23254257	55182253	A	0	0	G
6	BICF2S22922889	55196403	G	0	0	A
6	BICF2G630574943	55221744	A	0	0	G
6	BICF2G630574937	55232911	C	0	0	A
6	BICF2G630574935	55236200	G	0	0	A
6	BICF2G630574922	55250029	G	0	0	A
6	BICF2G630574909	55279462	A	0	0	C
6	BICF2P1008248	55291810	A	0	0	C
6	BICF2P758431	55293811	A	0	0	G
6	BICF2G630574908	55313825	A	0	0	G
6	BICF2P1443533	55315034	G	1	1	A
6	BICF2G630574877	55337334	G	0	0	A
6	BICF2G630574876	55338097	T	0.5	0.7143	A
6	BICF2G630574874	55353633	T	0	0	A
6	BICF2G630574863	55356446	A	0	0	C
6	BICF2G630574854	55363874	G	0.5	0.7143	A

6	BICF2P624673	55382150	G	0.5	0.7143	A
6	BICF2G630574814	55385318	A	0.5	0.7143	G
6	BICF2P1111663	55397546	G	0	0	A
6	BICF2S23626208	55409481	A	0	0	C
6	BICF2G630574752	55427500	A	1	1	G
6	BICF2S23228667	55434996	A	1	1	G
6	BICF2P1157839	55452633	G	1	1	A
6	BICF2P690499	55457069	C	1	1	A
6	BICF2P607619	55473160	A	0.5	0.7143	G
6	BICF2P1082679	55477370	A	0.5	0.7143	G
6	BICF2P1191886	55489558	C	0.5	0.7143	A
6	BICF2S2291434	55500889	G	0	0	A
6	TIGRP2P85174_rs9244598	55517258	0	0	0	C
6	BICF2P212991	55527616	A	0	0	T
6	BICF2G630574745	55542797	A	0	0	G
6	BICF2P1115353	55552187	A	0	0	G
6	BICF2G630574727	55565015	A	0	0	G
6	BICF2S23015141	55577939	G	0.5	0.7143	A
6	BICF2G630574708	55589989	G	0.5	0.7143	A
6	BICF2P1386020	55598410	A	1	1	G
6	BICF2G630574678	55613641	A	0.5	0.75	G
6	BICF2P1092588	55633527	0	0	0	G
6	BICF2G630574642	55636021	A	0.5	0.2857	G
6	TIGRP2P85192_rs9148232	55643936	C	0.5	0.7143	A
6	BICF2P1255555	55650356	A	0	0	G
6	TIGRP2P85205_rs8914001	55668171	0	0	0	A
6	TIGRP2P85213_rs8938443	55699707	A	0.5	0.2857	G
6	BICF2P522202	55723944	A	0.5	0.7143	G
6	TIGRP2P85225_rs8900931	55730091	G	1	1	A
6	BICF2P234021	55745992	G	0.5	0.2857	A
6	TIGRP2P85240_rs8660915	55777039	G	0	0	A
6	BICF2P923347	55786872	T	0.5	0.7143	A
6	BICF2G630574594	55809462	A	0.5	0.2857	C
6	BICF2P519999	55812384	A	0	0	C
6	BICF2G630574566	55852790	C	0.5	0.25	A
6	BICF2G630574564	55857577	G	0.5	0.2857	A
6	BICF2G630574548	55871605	A	0.5	0.2857	G
6	BICF2G630574534	55888682	C	0.5	0.2857	A
6	BICF2G630574507	55902838	G	0.5	0.2857	A
6	BICF2G630574491	55916759	A	0	0	G
6	BICF2G630574489	55928341	A	0	0	G
6	BICF2G630574479	55954062	A	0	0	T
6	BICF2G630574474	55962485	G	0	0	C
6	BICF2G630574470	55975606	A	0	0	G
6	BICF2G630574461	55983246	C	0	0	A
6	BICF2G630574440	56000700	A	0	0	G
6	BICF2G630574433	56010479	A	0.5	0.2857	T

6	BICF2G630574428	56017035	A	0.5	0.7143	G
6	BICF2G630574423	56032300	G	0.5	0.7143	A
6	BICF2G630574397	56048972	A	0.5	0.7143	T
6	BICF2G630574384	56053913	G	0.5	0.7143	A
6	BICF2G630574380	56056909	A	0.5	0.7143	G
6	BICF2P313760	56057444	A	0.5	0.7143	T
6	BICF2P1140625	56067499	G	0	0	A
6	BICF2P192717	56074671	A	0.5	0.7143	G
6	BICF2P646132	56092182	G	0.5	0.2857	A
6	BICF2P953174	56107661	A	0.5	0.7143	G
6	BICF2P158918	56109611	A	0.5	0.7143	G
6	BICF2G630574369	56122225	T	0	0	A
6	BICF2S23415199	56131701	A	1	1	C
6	BICF2G630574344	56145050	A	0	0	G
6	BICF2G630574307	56162235	0	0	0	G
6	BICF2G630574290	56179031	A	0	0	G
6	BICF2G630574286	56192138	G	0	0	A
6	BICF2P656619	56203277	G	0	0	C
6	BICF2G630574261	56204725	G	1	1	A
6	BICF2P1419328	56232453	0	0	0	G
6	BICF2P1419332	56236944	G	1	1	A
6	BICF2P802024	56255940	0	0	0	C
6	TIGRP2P85291_rs8769420	56262742	G	0	0	A
6	BICF2P1265796	56283857	0	0	0	A
6	BICF2G630574249	56298955	A	0.5	0.6429	G
6	BICF2G630574248	56332565	G	0.5	0.6429	A
6	BICF2P971133	56345181	0	0	0	C
6	BICF2S23915215	56354196	G	0	0	A
6	G414f37S66	56365129	A	0	0	G
6	BICF2G630574244	56436623	A	0.5	0.6429	T
6	TIGRP2P85296_rs9120172	56444728	0	0	0	G
6	BICF2P591648	56461097	0	0	0	A
6	BICF2S23541004	56469738	G	0	0	A
6	BICF2P220988	56485612	0	0	0	A
6	BICF2P642404	56503560	0	0	0	C
6	BICF2S24415326	56543687	G	0	0	A
6	BICF2P594730	56550178	A	0	0	G
6	BICF2P275345	56569500	0	0	0	A
6	BICF2G630574239	56580688	A	0	0	G
6	BICF2G630574203	56633566	G	0	0	A
6	BICF2G630574198	56639262	A	0	0	C
6	BICF2G630574174	56654485	G	0	0	A
6	BICF2G630574168	56657763	G	0	0	A
6	BICF2G630574165	56663610	0	0	0	G
6	BICF2G630574150	56677257	A	0.5	0.3571	G
6	BICF2G630574131	56694352	G	0.5	0.3571	A
6	BICF2G630574125	56695716	G	0.5	0.3571	A

6	TIGRP2P85870_rs9185886	56707545	A	0	0	G
6	BICF2G630574088	56726581	G	0.5	0.3571	A
6	BICF2S23553878	56737854	G	0.5	0.3571	A
6	BICF2G630574084	56743490	G	0	0	A
6	BICF2G630574065	56753117	G	1	1	A
6	BICF2G630574044	56760633	C	1	1	A
6	BICF2G630574028	56765180	A	0	0	G
6	TIGRP2P85873_rs8575950	56782883	0	0	0	G
6	BICF2G630574004	56791784	G	1	1	A
6	BICF2P614196	56804635	T	0	0	A
6	BICF2P368863	56824106	0	0	0	A
6	BICF2G630573990	56838366	A	0	0	G
6	BICF2G630573971	56850108	A	0	0	G
6	BICF2G630573969	56852412	G	0	0	C
6	TIGRP2P85877_rs8918211	56860680	A	0	0	G
6	BICF2G630573959	56872329	A	0.5	0.3571	G
6	BICF2G630573950	56888658	A	0.5	0.6429	G
6	BICF2G630573949	56898474	C	0.5	0.6429	A
6	TIGRP2P85893_rs9127426	56910237	A	0.5	0.6429	G
6	BICF2P745160	56922496	T	0	0	A
6	BICF2G630573904	56932623	G	0.5	0.6429	A
6	BICF2P256315	56945605	A	0.5	0.6429	G
6	BICF2G630573896	56950095	A	0.5	0.6429	G
6	BICF2G630573889	56962171	0	0	0	G
6	BICF2G630573884	56973871	A	0	0	G
6	BICF2G630573877	56983267	G	0	0	A
6	BICF2G630573857	56997177	A	0.5	0.3571	C
6	BICF2G630573839	57007124	A	0	0	G
6	BICF2G630573831	57020866	A	0	0	G
6	BICF2G630573820	57030941	G	0.5	0.6429	A
6	BICF2G630573810	57040448	A	0	0	G
6	BICF2G630573794	57051634	A	0	0	G
6	BICF2G630573776	57064271	A	0	0	G
6	BICF2G630573758	57084501	G	0	0	A
6	BICF2G630573757	57091190	C	0	0	G
6	BICF2S23642046	57112091	0	0	0	G
6	BICF2G630573754	57126391	A	0	0	G
6	BICF2G630573753	57133759	A	0	0	G
6	BICF2P538358	57145776	0	0	0	G
6	BICF2P927234	57169258	0	0	0	A
6	BICF2S2368027	57184384	A	0	0	G
6	BICF2G630573749	57210008	A	0	0	G
6	BICF2G630573744	57222074	A	0	0	G
6	BICF2G630573740	57230665	G	1	1	A
6	BICF2G630573738	57246362	A	0	0	G
6	BICF2G630573728	57258205	C	0.5	0.6429	G
6	BICF2G630573723	57268740	G	0.5	0.6429	A

6	BICF2G630573709	57278526	C	0.5	0.6429	A
6	BICF2P1180040	57291129	A	1	1	G
6	BICF2G630573700	57298598	A	1	1	G
6	BICF2G630573684	57306645	A	0	0	G
6	BICF2S23651052	57316517	C	0	0	G
6	BICF2G630573645	57338843	A	0	0	G
6	BICF2S2348714	57362551	A	0	0	G
6	BICF2G630573629	57382990	G	0	0	A
6	BICF2G630573617	57389486	G	1	1	A
6	BICF2P193228	57404810	A	1	1	C
6	BICF2P1272860	57435775	G	0	0	A
6	BICF2G630573575	57446498	G	0	0	A
6	BICF2G630573566	57454370	G	0	0	A
6	BICF2G630573562	57467248	A	0	0	G
6	BICF2G630573560	57481119	G	0	0	A
6	BICF2G630573552	57494104	G	0	0	A
6	BICF2P56535	57503460	G	0	0	C
6	BICF2P1200119	57512751	A	0	0	G
6	BICF2G630573542	57530689	A	0	0	G
6	BICF2G630573538	57537524	A	0.5	0.6429	G
6	BICF2G630573533	57551414	A	0	0	G
6	BICF2P34422	57558322	A	0	0	C
6	BICF2P868015	57571069	C	0	0	A
6	BICF2P770967	57590389	0	0	0	G
6	BICF2P247119	57593661	G	1	1	A
6	BICF2P690940	57609886	G	1	1	A
6	BICF2G630573522	57627523	G	0	0	A
6	TIGRP2P85973_rs8846515	57640734	A	1	1	G
6	BICF2P83265	57653120	A	1	1	G
6	BICF2P801872	57675371	G	1	1	A
6	BICF2P20812	57684907	A	0	0	G
6	BICF2P20813	57685249	G	1	1	A
6	BICF2G630573477	57701679	A	0.5	0.3571	G
6	BICF2G630573463	57708527	A	0.5	0.3571	C
6	BICF2G630573424	57724276	A	0	0	G
6	BICF2P837280	57740246	A	0.5	0.5714	G
6	BICF2P463039	57748950	A	0.5	0.3571	C
6	BICF2G630573391	57752163	A	0	0	G
6	BICF2S23133221	57762072	G	0	0	A
6	BICF2P1188965	57773441	T	0	0	A
6	BICF2P515341	57784830	A	0	0	G
6	BICF2S2365867	57790878	G	0	0	A
6	BICF2G630573372	57807174	G	0	0	A
6	BICF2G630573368	57819052	A	0	0	G
6	BICF2G630573363	57823733	G	0	0	A
6	BICF2S23029784	57840922	A	0	0	G
6	BICF2G630573350	57847677	C	0	0	A

6	BICF2G630573326	57867687	C	0	0	A
6	BICF2G630573324	57873430	G	0	0	A
6	BICF2G630573316	57887044	A	0	0	C
6	BICF2G630573311	57897701	G	0	0	A
6	BICF2P975176	57903851	A	0	0	G
6	BICF2P899876	57920387	G	1	1	A
6	BICF2P824055	57929723	G	0	0	A
6	BICF2P284466	57956309	A	0	0	G
6	BICF2P1329725	57971066	C	0	0	G
6	BICF2P1085552	57976673	A	0	0	T
6	BICF2S2317296	57985909	A	0.5	0.3571	G
6	BICF2S23554167	57996926	A	0	0	C
6	BICF2P304575	58011790	0	0	0	G
6	BICF2P1222628	58026349	G	0	0	A
6	BICF2S23510808	58029352	A	1	1	C
6	TIGRP2P86035_rs9122704	58036360	C	0	0	A
6	BICF2P934538	58047930	0	0	0	G
6	BICF2P1340532	58060674	A	0	0	G
6	BICF2P847136	58063792	G	0	0	A
6	BICF2P1314017	58083837	A	0	0	G
6	BICF2P599008	58088670	A	1	1	G
6	BICF2P789716	58101237	A	0.5	0.6429	G
6	BICF2P537008	58115213	G	0.5	0.3571	A
6	BICF2P173597	58122596	A	0	0	G
6	BICF2S23350087	58126850	A	0.5	0.6429	G
6	BICF2P837122	58130497	A	0.5	0.3571	G
6	BICF2S23235113	58132824	A	0.5	0.6429	G
6	BICF2P662490	58152040	A	0.5	0.6429	G
6	BICF2P1331022	58163242	G	0	0	A
6	BICF2P823948	58169389	A	0.5	0.6429	G
6	BICF2P1361460	58181441	A	0.5	0.6429	G
6	BICF2S23517261	58196269	G	1	1	A
6	BICF2P1122539	58209614	0	0	0	A
6	BICF2P719032	58216244	A	0.5	0.6429	G
6	BICF2S23223752	58226933	A	0	0	G
6	BICF2P260411	58241389	0	0	0	A
6	BICF2P1268157	58271135	0	0	0	A
6	TIGRP2P86110_rs8521712	58283801	0	0	0	G
6	TIGRP2P86118_rs9084857	58297151	A	0.5	0.6429	T
6	BICF2P871176	58310304	0	0	0	A
6	BICF2P770362	58323865	G	0	0	A
6	BICF2P927829	58334983	0	0	0	A
6	BICF2P672184	58341917	A	0	0	G
6	BICF2P393858	58358978	A	0	0	C
6	BICF2P124875	58363908	A	0.5	0.6429	G
6	BICF2S23213664	58381423	A	0.5	0.6429	G
6	BICF2S23258530	58386025	G	0	0	A

6	BICF2S2373412	58405661	C	0.5	0.6429	G
6	BICF2P1268516	58413227	G	0.5	0.6429	A
6	BICF2S23213970	58426144	A	0	0	G
6	BICF2P1369640	58449338	0	0	0	G
6	BICF2P377907	58459048	A	0	0	G
6	BICF2P773538	58472145	A	0.5	0.3571	G
6	BICF2P511695	58478112	A	0.5	0.3571	G
6	BICF2P546162	58503768	G	0	0	A
6	BICF2S23445434	58521441	A	0	0	T
6	TIGRP2P86140_rs8596660	58531504	0	0	0	G
6	BICF2P1170341	58536872	A	0	0	G
6	BICF2P1084824	58549240	A	1	1	C
6	BICF2P1370106	58580737	A	0	0	G
6	BICF2P871533	58584466	G	0	0	A
6	TIGRP2P86167_rs9008476	58595017	G	0	0	A
6	BICF2S23637190	58611040	A	0	0	G
6	BICF2P642081	58617872	A	0	0	G
6	BICF2P591015	58631844	G	0	0	A
6	BICF2P1300551	58640213	G	0	0	A
6	BICF2P508056	58672414	G	0	0	A
6	BICF2P526435	58674874	G	0	0	A
6	BICF2P1086196	58693745	A	0	0	C
6	BICF2P177326	58704822	G	0	0	A
6	BICF2P518132	58709236	A	0	0	G
6	BICF2P65728	58725956	G	0	0	A
6	BICF2P172163	58732162	A	0	0	G
6	BICF2P813416	58742715	G	0	0	A
6	TIGRP2P86222_rs8645966	58761704	A	0.5	0.3571	G
6	TIGRP2P86223_rs8818592	58767307	G	0	0	A
6	BICF2P1194083	58780475	G	0.5	0.3571	A
6	BICF2P1266666	58791220	G	1	1	A
6	BICF2P754049	58803116	A	0	0	G
6	BICF2P61178	58819370	A	1	1	T
6	TIGRP2P86240_rs9095226	58826541	A	0.5	0.6429	C
6	BICF2S23330596	58838691	A	0.5	0.6429	G
6	BICF2P214796	58848769	A	0.5	0.3571	C
6	BICF2P376167	58866996	C	0.5	0.3571	A
6	TIGRP2P86250_rs9047053	58871090	C	0	0	A
6	BICF2P48903	58885168	G	0	0	A
6	TIGRP2P86264_rs9246408	58901054	A	0	0	G
6	BICF2P1401124	58914189	A	0	0	G
6	TIGRP2P86271_rs9012980	58917384	A	1	1	C
6	BICF2S23319798	58929188	A	1	1	G
6	TIGRP2P86278_rs8945030	58942071	G	0	0	A
6	BICF2P1310865	58953022	C	0	0	A
6	BICF2P744427	58969117	0	0	0	A
6	BICF2P192715	58972835	G	0	0	A

6	BICF2P579807	58989598	G	0	0	A
6	BICF2S23331722	59002214	G	0.5	0.6429	A
6	BICF2P1221000	59007338	A	0	0	G
6	TIGRP2P86286_rs8639931	59019020	0	0	0	G
6	BICF2P939016	59029995	G	0	0	A
6	BICF2P713811	59044806	G	0	0	A
6	BICF2P1258730	59054191	A	0.5	0.3571	G
6	BICF2P672415	59065473	A	0	0	G
6	BICF2S23756724	59079568	C	0.5	0.3571	A
6	BICF2P11210	59087321	A	0.5	0.6429	G
6	BICF2S23158944	59102502	A	0.5	0.6429	G
6	BICF2P1379767	59112027	A	0.5	0.3571	G
6	BICF2P1391612	59128268	A	0.5	0.3571	T
6	BICF2P1286321	59139653	G	0.5	0.6429	A
6	BICF2P721780	59154375	0	0	0	A
6	BICF2S2359076	59165324	A	0.5	0.3571	C
6	BICF2P168335	59197672	0	0	0	G
6	BICF2S23261056	59216393	G	0	0	A
6	BICF2P922768	59226065	0	0	0	C
6	BICF2P137809	59244675	0	0	0	C
6	BICF2P84609	59249778	0	0	0	G
6	BICF2P609143	59267793	0	0	0	A
6	BICF2S23751798	59275032	C	0	0	A
6	BICF2P585026	59308231	0	0	0	A
6	BICF2P417279	59319515	0	0	0	C
6	BICF2P334341	59350744	G	0	0	A
6	TIGRP2P86366_rs8605604	59356441	G	0	0	A
6	BICF2P1118099	59366149	A	0	0	G
6	BICF2P298720	59387874	0	0	0	A
6	BICF2P483434	59404970	A	0	0	G
6	BICF2P730258	59431234	G	0	0	A
6	BICF2P1197340	59442277	0	0	0	A
6	TIGRP2P86378_rs9164291	59447662	A	0	0	C
6	TIGRP2P86382_rs9052357	59484502	C	0	0	A
6	BICF2P1240689	59499400	0	0	0	A
6	TIGRP2P86383_rs8648067	59501097	A	0	0	G
6	BICF2S2376349	59521758	C	0	0	A
6	TIGRP2P86386_rs9032889	59532190	A	0	0	C
6	BICF2P1019125	59536928	A	0.5	0.3571	G
6	BICF2P1374297	59548365	T	0.5	0.6429	A
6	TIGRP2P86390_rs8560151	59548719	A	0.5	0.3571	C
6	BICF2P440946	59569746	A	0.5	0.6429	G
6	BICF2P1108129	59570889	A	0.5	0.3571	C
6	BICF2P511211	59584315	0	0	0	A
6	BICF2S2305741	59603696	G	0.5	0.6429	A
6	TIGRP2P86404_rs9217482	59636945	G	0	0	A
6	BICF2P786672	59639769	A	0.5	0.6429	G

6	BICF2P327146	59650775	G	0.5	0.6429	A
6	BICF2P1091570	59662216	A	0.5	0.6429	G
6	BICF2P177195	59685561	G	0	0	A
6	BICF2P490581	59703980	A	0.5	0.6429	G
6	BICF2P831572	59709288	A	0.5	0.6667	G
6	BICF2P1309575	59726333	A	0.5	0.6429	G
6	BICF2P901170	59738731	A	0.5	0.6429	G
6	BICF2P1297650	59753081	A	0.5	0.6429	G
6	BICF2P1297653	59755412	A	0.5	0.6429	G
6	BICF2S2349350	59784116	A	0.5	0.3571	G
6	BICF2P1064329	59811057	G	1	1	A
6	BICF2P1242308	59821007	A	1	1	G
6	TIGRP2P86438_rs8724929	59827980	G	1	1	A
6	TIGRP2P86445_rs8717250	59844874	G	1	1	A
6	BICF2P341635	59852319	A	1	1	C
6	BICF2P590761	59857894	A	1	1	G
6	BICF2P618381	59874141	A	1	1	G
6	BICF2P1373482	59881266	G	0.5	0.6429	A
6	TIGRP2P86460_rs9093877	59902692	T	1	1	A
6	BICF2P165247	59917378	G	0	0	A
6	BICF2P581390	59932352	A	1	1	G
6	BICF2P1136612	59944539	A	0.5	0.3571	G
6	BICF2P108941	59950392	G	0.5	0.3571	A
6	BICF2P469285	59974394	A	0.5	0.6429	T
6	TIGRP2P86475_rs9073350	59991848	0	0	0	C
6	BICF2P1111222	60003992	0	0	0	A
6	BICF2P500185	60016966	A	0.5	0.6429	G
6	BICF2P133171	60019425	C	0	0	A
6	BICF2P959873	60036150	A	1	1	G
6	BICF2S23716694	60049434	G	0	0	A
6	BICF2S23717110	60053573	G	0	0	A
6	BICF2P1036271	60076530	G	0.5	0.3571	A
6	BICF2P193289	60089576	C	0	0	A
6	BICF2S23259398	60109664	A	0.5	0.6429	G
6	TIGRP2P86489_rs8798631	60119865	A	0.5	0.6429	G
6	BICF2P834932	60128276	A	0.5	0.6429	G
6	BICF2P600577	60141581	G	0.5	0.6429	C
6	BICF2P294291	60143167	A	0.5	0.6429	G
6	BICF2P1092171	60145001	A	0.5	0.3571	G
6	BICF2P789099	60161586	G	0	0	A
6	BICF2P85572	60175473	A	0.5	0.6429	G
6	BICF2P421690	60198427	A	0.5	0.6429	G
6	BICF2S23222591	60206015	C	0	0	A
6	BICF2P291934	60223946	C	0.5	0.6429	A
6	BICF2P414399	60226124	A	0.5	0.6429	G
6	BICF2P489011	60230491	A	0.5	0.6429	G
6	BICF2P1125826	60243203	A	0.5	0.6429	C

6	BICF2S23612881	60248756	G	0.5	0.6429	A
6	TIGRP2P86527_rs8848880	60260049	0	0	0	G
6	TIGRP2P86533_rs8759904	60288244	A	0	0	G
6	BICF2P403390	60296489	C	0	0	A
6	BICF2P950275	60327262	A	0	0	G
6	TIGRP2P86541_rs9136656	60331090	A	0	0	G
6	TIGRP2P86547_rs8842961	60343389	A	0	0	G
6	BICF2S23729461	60368600	A	0.5	0.6429	G
6	BICF2P1065711	60375106	G	0	0	A
6	BICF2S23715702	60380933	C	0.5	0.6429	G
6	BICF2P1042083	60387003	A	0.5	0.6429	G
6	BICF2S2327112	60402126	A	0	0	G
6	BICF2S22947893	60417224	A	0	0	C
6	BICF2P596498	60440867	A	NA	1	C
6	BICF2P327538	60451698	A	0.5	0.6429	G
6	BICF2S23727354	60459798	C	0.5	0.6429	A
6	BICF2P519947	60472562	G	0.5	0.6429	A
6	BICF2P1051937	60480098	A	0.5	0.6429	G
6	BICF2S2329192	60494401	C	0	0	A
6	BICF2P543616	60512783	A	0	0	C
6	TIGRP2P86557_rs8953969	60515067	G	0	0	C
6	BICF2P1073466	60529875	A	0.5	0.6429	T
6	BICF2P1268734	60555451	G	0	0	A
6	BICF2P10462	60560282	A	0	0	C
6	BICF2P1272438	60575002	A	1	1	G
6	BICF2P298105	60613781	G	0	0	A
6	BICF2P1073853	60624648	A	1	1	G
6	TIGRP2P86569_rs9022614	60632695	G	1	1	A
6	BICF2P465509	60638865	G	0	0	A
6	BICF2P611266	60649668	G	0	0	A
6	BICF2P708736	60653369	A	0	0	G
6	BICF2P622317	60669474	0	0	0	A
6	TIGRP2P86584_rs9123621	60674643	A	1	1	G
6	BICF2S2348750	60695309	G	0	0	A
6	BICF2S23536986	60700069	A	0	0	G
6	BICF2P1072993	60709953	A	0	0	G
6	BICF2P194843	60738555	A	0	0	G
6	BICF2P958644	60743319	A	0	0	G
6	BICF2P1113302	60766468	A	0	0	G
6	BICF2P199263	60799491	0	0	0	G
6	BICF2P1267480	60820837	G	0	0	A
6	BICF2P1298139	60833078	A	0	0	G
6	BICF2P970959	60835520	G	0	0	A
6	TIGRP2P86592_rs8657165	60846756	0	0	0	G
6	BICF2S23750524	60862628	G	0	0	A
6	BICF2S23313782	60870177	0	0	0	G
6	BICF2P159724	60889298	A	1	1	G

6	TIGRP2P86594_rs9187647	60898795	A	1	1	G
6	BICF2P77566	60903883	C	1	1	G
6	BICF2P77565	60904209	A	0	0	G
6	BICF2P388639	60916978	A	0	0	C
6	TIGRP2P86606_rs8670319	60961910	G	0	0	A
6	TIGRP2P86617_rs8777731	60979878	G	0	0	A
6	TIGRP2P86619_rs9250982	60993385	0	0	0	G
6	BICF2P398183	61003471	A	0.5	0.3571	G
6	TIGRP2P86623_rs8502807	61015532	0	0	0	G
6	TIGRP2P86626_rs8902527	61030309	C	0.5	0.6429	A
6	BICF2P1408601	61035312	A	0.5	0.6429	G
6	TIGRP2P86629_rs9098882	61049862	0	0	0	C
6	BICF2P750033	61057733	G	0.5	0.3571	A
6	TIGRP2P86631_rs9116915	61067579	A	0.5	0.3571	G
6	BICF2P1169937	61078575	A	0.5	0.3571	G
6	BICF2P1470193	61092413	G	0.5	0.3571	A
6	BICF2P809881	61100363	G	0.5	0.3571	C
6	BICF2P297284	61121673	0	0	0	A
6	BICF2P1349784	61124594	0	0	0	G
6	BICF2P297406	61150443	0	0	0	A
6	BICF2S22913017	61165243	G	0	0	A
6	BICF2P1136975	61170753	0	0	0	C
6	BICF2S23728470	61185149	A	0	0	C
6	BICF2S2435880	61191995	A	0.5	0.6429	G
6	BICF2P211412	61211767	A	0.5	0.3571	G
6	BICF2P1446021	61221190	A	0	0	G
6	BICF2P814778	61226361	G	0	0	A
6	TIGRP2P86696_rs8990176	61238948	A	1	1	C
6	TIGRP2P86701_rs8878571	61251595	A	0	0	C
6	BICF2P1188898	61276296	G	0.5	0.3571	A
6	BICF2S23532366	61283746	A	0	0	G
6	BICF2S235854	61313760	G	0	0	A
6	BICF2S23534949	61325958	G	0	0	A
6	BICF2P190286	61346457	G	0	0	A
6	BICF2S23125592	61356332	G	0	0	A
6	TIGRP2P86740_rs8613765	61365732	G	0.5	0.3571	A
6	TIGRP2P86744_rs9017675	61377115	G	0.5	0.3571	A
6	BICF2S23618321	61398380	A	0	0	G
6	TIGRP2P86749_rs8678811	61402492	A	0	0	G
6	BICF2P834260	61420154	A	0.5	0.3571	T
6	BICF2P904645	61429617	A	0.5	0.3571	G
6	BICF2S2366689	61438237	G	0	0	A
6	BICF2P178065	61454812	G	0	0	A
6	BICF2P142651	61457227	G	0.5	0.3571	A
6	BICF2P1472011	61471708	A	0	0	G
6	TIGRP2P86776_rs8767380	61489709	0	0	0	G
6	BICF2S23610326	61496540	A	0	0	G

6	BICF2S24410264	61509033	0	0	0	A
6	BICF2P783249	61509158	0	0	0	C
6	BICF2S23722731	61530188	0	0	0	G
6	BICF2P1111284	61545595	G	0.5	0.3571	A
6	TIGRP2P86796_rs8906630	61552393	G	0.5	0.3571	A
6	BICF2P541173	61562934	G	0.5	0.3571	A
6	TIGRP2P86801_rs8513365	61571443	G	0	0	A
6	TIGRP2P86810_rs9052320	61582554	G	0	0	A
6	BICF2P619065	61604149	A	0	0	G
6	BICF2P553180	61606021	G	0	0	A
6	BICF2P699136	61625438	A	0.5	0.6429	C
6	BICF2S23010073	61630667	A	0.5	0.6429	G
6	BICF2P131237	61640171	A	0.5	0.6429	G
6	BICF2P312036	61657004	G	0.5	0.6429	A
6	BICF2S22948389	61663313	G	0.5	0.6429	A
6	BICF2P942808	61675004	A	0.5	0.6429	C
6	BICF2S23032540	61693502	G	0	0	A
6	BICF2P1186238	61707171	A	0.5	0.6429	G
6	BICF2P851760	61712834	G	0.5	0.6429	C
6	BICF2P1076861	61722350	C	0	0	A
6	BICF2P1076854	61724158	G	0.5	0.3571	A
6	TIGRP2P86847_rs8817629	61744687	G	1	1	A
6	BICF2S23437451	61757865	C	0	0	A
6	TIGRP2P86864_rs8832487	61767999	A	0.5	0.3571	G
6	BICF2S23151843	61785705	C	0	0	G
6	BICF2S23541636	61791929	G	0	0	C
6	BICF2P655471	61811907	C	0	0	A
6	TIGRP2P86873_rs8968662	61814076	C	0	0	A
6	BICF2P195213	61826945	A	0	0	G
6	TIGRP2P86899_rs8938233	61842480	G	1	1	A
6	BICF2P665648	61847975	A	1	1	G
6	BICF2P885715	61859528	A	0.5	0.3571	G
6	BICF2P380020	61868635	A	0.5	0.6429	G
6	BICF2S23527915	61877002	G	0.5	0.6429	A
6	BICF2P851405	61881882	A	0	0	G
6	BICF2S22928502	61896838	G	0	0	A
6	BICF2S2343856	61908996	C	0.5	0.3571	A
6	BICF2S23764709	61918906	A	0	0	G
6	BICF2P1320957	61931557	0	0	0	G
6	TIGRP2P86949_rs9056450	61941464	A	0	0	G
6	BICF2P952807	61961584	0	0	0	G
6	BICF2S2346224	61968631	C	0.5	0.3571	A
6	BICF2P711900	61976486	C	0.5	0.6429	A
6	BICF2S23734395	61988324	A	0	0	G
6	BICF2S23755221	62002119	0	0	0	A
6	BICF2P547105	62010698	C	0.5	0.6429	A
6	BICF2P558739	62020121	A	0.5	0.6429	G

6	BICF2P351939	62038619	0	0	0	A
6	BICF2S23225831	62044544	G	0.5	0.6429	A
6	BICF2P112157	62074716	A	0	0	C
6	TIGRP2P87028_rs9030393	62080186	C	0.5	0.6429	A
6	BICF2S2304986	62096174	A	0	0	C
6	BICF2P1184386	62106201	0	0	0	G
6	TIGRP2P87038_rs8998035	62117173	A	0	0	G
6	TIGRP2P87042_rs8703254	62132798	0	0	0	A
6	BICF2P253632	62161658	A	0	0	G
6	BICF2P1722	62175962	A	0	0	G
6	BICF2P982011	62186705	0	0	0	A
6	BICF2S23418268	62197170	G	0.5	0.3571	A
6	BICF2P506635	62204324	A	0.5	0.6429	G
6	BICF2P399377	62221889	G	1	1	A
6	BICF2P1072956	62228107	G	1	1	A
6	BICF2P1014719	62253381	G	0	0	A
6	BICF2P1007536	62261947	G	0	0	A
6	BICF2P91160	62276614	A	0.5	0.6429	C
6	BICF2P1081444	62289842	A	0	0	G
6	BICF2S23654938	62296272	A	0.5	0.6429	T
6	BICF2P868518	62313283	A	0	0	G
6	BICF2P810008	62328224	G	0.5	0.3571	A
6	BICF2P235529	62332572	A	0.5	0.3571	G
6	BICF2S23342444	62350448	A	0	0	C
6	BICF2S24411578	62360703	A	0.5	0.3571	G
6	BICF2P736978	62379658	G	0	0	A
6	BICF2S23641288	62392313	G	0	0	A
6	BICF2P229363	62411554	G	0	0	A
6	BICF2P208416	62424307	A	0.5	0.3571	T
6	BICF2P538955	62438493	A	1	1	C
6	BICF2S23349390	62446270	A	0	0	G
6	BICF2S2366078	62465771	A	0	0	G
6	BICF2S23452996	62471154	G	0.5	0.3571	A
6	BICF2P795052	62485900	G	0.5	0.3571	A
6	BICF2P1355088	62497567	A	0.5	0.3571	G
6	BICF2P813370	62503565	A	0.5	0.6429	C
6	BICF2S23452370	62522508	0	0	0	A
6	BICF2P765518	62550832	A	0	0	G
6	BICF2P101339	62561582	0	0	0	C
6	BICF2P932900	62574808	G	1	1	A
6	BICF2P549465	62585050	C	0.5	0.3571	A
6	BICF2S23141911	62604191	G	0	0	A
6	BICF2P1142664	62617408	G	0	0	A
6	BICF2P1268150	62622343	A	0	0	G
6	BICF2P395798	62636995	0	0	0	A
6	BICF2P758308	62645435	C	0	0	A
6	BICF2P224834	62654302	A	0	0	G

6	BICF2P1105484	62674558	C	0	0	G
6	BICF2S23120930	62683267	C	0	0	A
6	BICF2P1332357	62697537	A	0	0	G
6	BICF2P1332356	62698445	A	0	0	G
6	BICF2P1293349	62716943	T	0	0	A
6	BICF2S23222934	62722023	G	0	0	A
6	BICF2P231859	62742278	A	0	0	G
6	BICF2P613412	62746010	A	1	1	G
6	BICF2P1061514	62771627	A	0	0	C
6	BICF2P362674	62789343	A	0	0	C
6	BICF2P1427203	62794719	C	0	0	G
6	BICF2P1427204	62794756	A	0.5	0.3571	G
6	BICF2P428648	62802730	G	0.5	0.3571	A
6	BICF2P434994	62813605	G	0	0	A
6	BICF2P410090	62832993	G	0	0	A
6	BICF2P961798	62836639	G	0.5	0.6429	A
6	BICF2P210780	62848084	G	0	0	A
6	BICF2P497651	62869102	C	0	0	G
6	BICF2P465951	62875173	A	0	0	G
6	BICF2P727450	62888256	G	0	0	A
6	BICF2P333904	62896139	0	0	0	G
6	BICF2S2311545	62910947	A	0.5	0.3571	G
6	TIGRP2P87053_rs8512186	62920028	G	0.5	0.3571	A
6	TIGRP2P87056_rs8630024	62928370	G	0.5	0.6429	A
6	BICF2P79082	62940341	A	0	0	G
6	BICF2P5565	62959180	C	0	0	A
6	BICF2P725144	62970222	G	0.5	0.6429	A
6	BICF2P1270571	62975109	A	0	0	G
6	BICF2P1222463	62987447	A	0	0	G
6	BICF2P888146	63005018	A	0	0	C
6	BICF2P898102	63009003	0	0	0	C
6	BICF2P105841	63023127	0	0	0	A
6	BICF2P110565	63033554	G	0	0	C
6	BICF2P327764	63044393	0	0	0	C
6	BICF2P1112472	63054671	C	0	0	A
6	BICF2P1223812	63066301	A	0	0	G
6	TIGRP2P87061_rs8830962	63095370	0	0	0	G
6	BICF2P634651	63106915	A	0.5	0.6429	G
6	BICF2S23140819	63120812	A	0.5	0.6429	G
6	BICF2P1400510	63130672	A	0	0	G
6	BICF2P1396752	63138611	C	0.5	0.6429	A
6	TIGRP2P87080_rs8880791	63152603	G	0	0	A
6	BICF2P937848	63161209	G	0	0	A
6	BICF2P525857	63174877	G	1	1	A
6	BICF2S23737224	63187224	G	0.5	0.6429	A
6	BICF2S23520828	63195636	A	0.5	0.3571	G
6	BICF2S23752702	63206753	G	0	0	A

6	BICF2P70705	63215925	A	0.5	0.6429	G
6	BICF2S2393706	63228058	A	0	0	T
6	BICF2P1078426	63245275	A	0.5	0.3571	G
6	BICF2P873169	63254591	A	0	0	G
6	BICF2S2325865	63271591	A	0	0	G
6	BICF2P1160944	63278354	0	0	0	A
6	BICF2P281040	63287011	A	0	0	G
6	BICF2P589225	63300576	A	0.5	0.3571	G
6	BICF2P408002	63331864	G	0.5	0.6429	C
6	TIGRP2P87139_rs8709997	63346352	G	0.5	0.3571	A
6	BICF2S23252692	63365446	A	0.5	0.3571	G
6	BICF2P845097	63379903	A	1	1	G
6	BICF2S23121355	63399019	A	1	1	G
6	BICF2P320503	63406115	G	0	0	A
6	BICF2S232143	63416474	A	0	0	G
6	BICF2P1325816	63429860	T	0.5	0.6429	A
6	BICF2S23257026	63437368	A	0	0	G
6	BICF2S2332696	63453924	A	0	0	G
6	BICF2P549484	63460726	G	1	1	A
6	BICF2P147524	63470378	G	0.5	0.6429	A
6	BICF2P463995	63483034	G	0	0	A
6	BICF2P1108960	63493091	C	0	0	G
6	BICF2P231427	63503202	G	0.5	0.3571	A
6	BICF2P75894	63517645	A	0	0	G
6	BICF2S2334287	63527603	A	0	0	G
6	BICF2S23020274	63538006	G	0	0	A
6	BICF2P279040	63554364	0	0	0	G
6	TIGRP2P87158_rs9137663	63567812	A	0.5	0.6429	G
6	BICF2P199411	63570146	0	0	0	C
6	BICF2P671666	63578031	A	0	0	G
6	BICF2P276982	63586443	C	0.5	0.3571	A
6	BICF2P1339845	63597102	G	0	0	A
6	BICF2P1428226	63608217	G	0.5	0.6429	A
6	TIGRP2P87200_rs8734364	63622475	G	0.5	0.6429	A
6	BICF2P560185	63630933	C	0.5	0.3571	A
6	BICF2P1023017	63649862	A	0	0	G
6	TIGRP2P87221_rs8541897	63656734	C	0.5	0.6429	A
6	BICF2S23641542	63667564	G	0.5	0.3571	A
6	BICF2S23423556	63689737	A	0	0	T
6	BICF2P974392	63699628	G	1	1	A
6	TIGRP2P87240_rs8755335	63715938	A	0.5	0.3571	G
6	TIGRP2P87242_rs8689679	63728791	0	0	0	G
6	BICF2P1036926	63735991	0	0	0	A
6	BICF2P827180	63760449	A	1	1	G
6	TIGRP2P87243_rs9054968	63792850	A	0	0	G
6	TIGRP2P87259_rs8975997	63812729	G	0	0	A
6	BICF2S23343696	63837155	A	0	0	G

6	BICF2S23219969	63852029	A	0.5	0.6429	C
6	TIGRP2P87271_rs8623384	63869865	0	0	0	G
6	BICF2P408103	63880017	A	0.5	0.6429	G
6	BICF2P49766	63887224	0	0	0	C
6	BICF2S2311602	63899266	C	0.5	0.3571	A
6	TIGRP2P87274_rs9165509	63913262	0	0	0	G
6	BICF2P481450	63920249	0	0	0	C
6	TIGRP2P87275_rs8895401	63930079	0	0	0	G
6	BICF2P357252	63948192	A	0	0	G
6	BICF2P1347966	63951772	A	0	0	G
6	BICF2S2299083	63965090	C	0	0	A
6	BICF2P1099363	63984905	G	0.5	0.6429	A
6	TIGRP2P87292_rs8524688	63990108	0	0	0	A
6	BICF2S23635177	64013651	A	0	0	G
6	BICF2P576490	64022223	0	0	0	G
6	BICF2P576823	64042403	0	0	0	G
6	BICF2P625531	64068585	G	0	0	A
6	TIGRP2P87309_rs9074708	64082509	A	0.5	0.6429	G
6	BICF2S23211443	64089519	G	0.5	0.6429	A
6	BICF2P716098	64107861	T	0	0	A
6	BICF2P964325	64116450	A	0	0	G
6	TIGRP2P87332_rs9030000	64125594	A	0	0	G
6	BICF2P525185	64125940	G	0	0	C
6	BICF2P495300	64130790	A	0	0	G
6	BICF2P83354	64144029	A	0	0	G
6	BICF2P1319609	64148071	A	0	0	C
6	TIGRP2P87357_rs8952697	64160808	0	0	0	C
6	BICF2S2296188	64178563	C	0	0	A
6	BICF2P588008	64184011	A	0	0	T
6	BICF2S23538323	64200096	C	0	0	A
6	BICF2P634837	64207330	A	0.5	0.6429	C
6	BICF2P1133718	64224120	G	1	1	A
6	BICF2P311492	64227595	C	0.5	0.3571	A
6	BICF2P168629	64259691	A	1	1	G
6	BICF2S23744548	64270223	G	1	1	A
6	BICF2S23454565	64282797	A	0.5	0.6429	G
6	BICF2S2366451	64296182	G	0.5	0.3571	A
6	BICF2P506883	64297568	A	0.5	0.3571	G
6	BICF2S23255626	64311854	A	1	1	G
6	TIGRP2P87432_rs8814663	64328482	G	1	1	A
6	BICF2P757580	64335297	A	1	1	G
6	BICF2P417849	64349507	G	0.5	0.3571	A
6	BICF2S23254043	64361720	G	0.5	0.3571	A
6	BICF2P646224	64366706	G	0.5	0.3571	A
6	BICF2P1001843	64379076	A	0.5	0.3571	G
6	BICF2P137382	64392715	G	0.5	0.3571	A
6	BICF2P801858	64400840	G	0.5	0.3571	A

6	BICF2P247790	64415967	A	0	0	C
6	BICF2P778423	64428848	G	0	0	A
6	TIGRP2P87455_rs9191113	64438120	G	0	0	A
6	BICF2P907347	64452973	A	0.5	0.6429	G
6	BICF2P1048718	64515515	0	0	0	A
6	G417f15S223	64556294	A	0	0	G
6	BICF2P1038491	64572159	T	0	0	A
6	BICF2S23161298	64574764	0	0	0	G
6	BICF2S2341163	64591554	G	0	0	A
6	G418f66S42	64640459	0	0	0	G
6	BICF2S23343477	64669399	G	0	0	A
6	BICF2S23333023	64685011	A	0	0	G
6	BICF2P630785	64702736	0	0	0	A
6	G419f55S239	64739698	G	1	1	A
6	BICF2P1007451	64779045	0	0	0	G
6	BICF2S23350571	64782349	C	1	1	A
6	BICF2S23449711	64796234	G	0.5	0.6429	A
6	BICF2S2377519	64820355	A	0	0	G
6	BICF2S23419123	64880553	A	0	0	G
6	BICF2S23417878	64901431	A	1	1	G
6	BICF2P168671	64910275	0	0	0	A
6	G421f63S108	64950274	G	0.5	0.6429	A
6	G421f67S371	64952101	0	0	0	C
6	BICF2S23416766	65016957	A	0	0	C
6	BICF2P239786	65025117	0	0	0	A
6	BICF2S22918602	65060430	A	0	0	G
6	BICF2S22927854	65070265	G	0.5	0.6429	A
6	BICF2P237353	65102950	0	0	0	A
6	BICF2P1134114	65119926	0	0	0	C
6	BICF2P1136047	65153052	0	0	0	C
6	BICF2P1170841	65166566	A	0	0	C
6	TIGRP2P87477_rs8789635	65181579	A	0.5	0.3571	C
6	BICF2P805311	65190320	G	0	0	A
6	BICF2P576519	65195585	G	0	0	A
6	BICF2P1106762	65214237	A	0	0	G
6	BICF2P1489144	65222795	0	0	0	G
6	BICF2P690837	65233617	A	0.5	0.6429	T
6	BICF2S23133929	65241552	A	0	0	C
6	BICF2P273403	65260150	C	0	0	G
6	BICF2P743911	65263299	G	0	0	A
6	BICF2P1298772	65274605	A	0	0	G
6	BICF2S23024853	65290324	C	1	1	A
6	BICF2P1050727	65302492	A	1	1	G
6	BICF2P99256	65309431	A	0	0	G
6	BICF2P523607	65327456	0	0	0	A
6	BICF2P724279	65341529	A	0	0	G
6	BICF2P86493	65348894	A	0	0	C

6	BICF2S23242919	65355385	G	0.5	0.6429	C
6	BICF2P1214185	65368819	G	0.5	0.6429	A
6	BICF2P1314874	65386653	C	0.5	0.3571	A
6	BICF2P1334599	65396095	G	0.5	0.3571	A
6	BICF2P347440	65406337	C	0.5	0.3571	A
6	BICF2S23740888	65426364	A	0	0	G
6	BICF2S23430299	65444790	G	0.5	0.6429	A
6	BICF2P130550	65451360	0	0	0	G
6	BICF2P1324580	65463450	A	0.5	0.3571	G
6	BICF2P922351	65470499	A	0	0	G
6	BICF2P808229	65486124	G	0.5	0.6429	A
6	BICF2P638578	65494405	0	0	0	C
6	TIGRP2P87510_rs8752591	65512538	A	0.5	0.3571	G
6	TIGRP2P87512_rs8949342	65516471	A	0.5	0.3571	G
6	BICF2P927583	65530539	G	0.5	0.3571	A
6	BICF2P355274	65539191	G	0.5	0.3571	A
6	BICF2S2307368	65554216	A	0.5	0.3571	G
6	BICF2S2299521	65569246	A	0.5	0.3571	G
6	TIGRP2P87521_rs8960506	65580777	A	0	0	G
6	BICF2P421496	65588449	G	0	0	A
6	TIGRP2P87532_rs8686846	65610633	G	0	0	A
6	BICF2P420093	65620601	A	0	0	G
6	BICF2P1391558	65631083	A	0	0	G
6	BICF2S2399245	65644528	C	0	0	A
6	BICF2P240336	65654820	T	0	0	A
6	BICF2S2416720	65678391	G	0	0	A
6	BICF2S2295980	65696985	G	0	0	A
6	BICF2S22934379	65700733	A	0	0	G
6	TIGRP2P87558_rs9065404	65719144	0	0	0	A
6	BICF2S23035505	65739454	A	0.5	0.3571	G
6	BICF2P784528	65745561	A	0	0	G
6	TIGRP2P87565_rs8960420	65759971	A	0.5	0.3571	G
6	BICF2P810090	65779813	0	0	0	G
6	BICF2P1148695	65789615	A	1	1	C
6	BICF2P947551	65801142	T	1	1	A
6	BICF2P769237	65814032	0	0	0	C
6	TIGRP2P87597_rs8702617	65817487	A	0	0	G
6	TIGRP2P87606_rs8703609	65836175	A	0	0	G
6	BICF2P1294493	65845205	0	0	0	G
6	BICF2P776302	65851479	A	0	0	G
6	BICF2P778195	65860590	A	0	0	G
6	TIGRP2P87629_rs8845384	65873983	G	1	1	A
6	BICF2P1184167	65887762	G	0	0	A
6	BICF2P934376	65905415	G	1	1	A
6	BICF2P352195	65908439	A	1	1	G
6	TIGRP2P87665_rs8740729	65923213	0	0	0	C
6	TIGRP2P87669_rs8925116	65937260	A	0	0	G

6	BICF2P670133	65943973	0	0	0	G
6	BICF2P1077929	65957038	G	0	0	A
6	BICF2P536627	65964784	0	0	0	A
6	BICF2S23445312	65986075	A	1	1	G
6	BICF2P1209403	65988610	G	1	1	A
6	BICF2S2292952	65998514	G	0	0	A
6	TIGRP2P87690_rs9203865	66017823	A	1	1	G
6	BICF2P1103956	66032060	A	1	1	G
6	BICF2S23719971	66042050	A	0.5	0.6667	G
6	TIGRP2P87697_rs8816391	66051988	A	0.5	0.3571	C
6	BICF2P537309	66057163	G	0	0	A
6	BICF2P626989	66071231	A	0	0	G
6	BICF2P579635	66090265	A	0	0	C
6	BICF2S23217563	66095723	G	0.5	0.3571	A
6	BICF2S23217560	66096064	T	0	0	A
6	BICF2P245073	66107092	T	0	0	A
6	BICF2S2299683	66118013	A	0	0	C
6	BICF2P853584	66127301	A	0	0	G
6	BICF2S23648200	66132197	A	0.5	0.3333	G
6	BICF2P1027325	66141408	A	0	0	G
6	BICF2P1244225	66153683	C	0.5	0.3571	A
6	BICF2P649454	66165508	G	0.5	0.6429	A
6	BICF2P125612	66174460	A	0.5	0.6429	G
6	BICF2P1053155	66189273	0	0	0	A
6	TIGRP2P87713_rs8938619	66195926	A	0	0	G
6	BICF2P138957	66211769	G	0	0	A
6	TIGRP2P87718_rs8511654	66219907	G	0	0	A
6	BICF2P1099579	66239189	G	0	0	A
6	TIGRP2P87735_rs9067251	66257945	G	0	0	A
6	BICF2P959944	66264250	G	0	0	A
6	BICF2P309509	66282503	G	0	0	C
6	BICF2P118532	66287594	G	0	0	A
6	BICF2P551715	66307928	A	NA	0.3	C
6	BICF2P474877	66317693	0	0	0	A
6	BICF2P92489	66325301	G	0	0	A
6	BICF2P1204050	66335367	A	0	0	G
6	BICF2P852423	66343851	0	0	0	C
6	BICF2P771082	66384831	G	0	0	A
6	BICF2S23650534	66399758	A	0	0	G
6	BICF2P637992	66402482	A	0	0	G
6	BICF2P522126	66415759	G	0	0	A
6	BICF2P579841	66450200	A	1	1	C
6	BICF2P1139196	66459844	A	1	1	C
6	BICF2P364431	66480242	G	0	0	A
6	TIGRP2P87764_rs8522636	66483200	A	0.5	0.6429	G
6	BICF2S2339629	66493892	A	0.5	0.3571	G
6	BICF2P1212948	66511184	A	0	0	T

6	BICF2P386877	66525348	G	0	0	A
6	BICF2S23219258	66530429	A	0	0	G
6	BICF2P450499	66541470	C	0	0	A
6	TIGRP2P87784_rs9196591	66555914	A	0	0	G
6	BICF2P181059	66564268	A	0.5	0.6429	G
6	BICF2S2296614	66584295	A	1	1	C
6	BICF2P595418	66588808	A	1	1	G
6	BICF2S23633900	66603775	C	0.5	0.3571	A
6	TIGRP2P87798_rs9012324	66611382	0	0	0	C
6	BICF2P988961	66625488	A	0	0	C
6	TIGRP2P87805_rs8699624	66632100	G	0	0	A
6	TIGRP2P87811_rs8935167	66650439	A	0	0	G
6	TIGRP2P87816_rs8846908	66656242	C	0	0	A
6	TIGRP2P87828_rs8812556	66668470	G	0.5	0.3571	A
6	TIGRP2P87829_rs8726422	66690141	G	0	0	A
6	BICF2P246114	66704233	A	0	0	C
6	BICF2P1270817	66716336	A	0	0	G
6	TIGRP2P87834_rs9119907	66732806	A	0.5	0.3571	T
6	BICF2S22954879	66759363	A	0	0	G
6	BICF2P1412351	66785195	0	0	0	A
6	BICF2P226511	66797401	A	0	0	G
6	BICF2S22917237	66813551	A	0	0	G
6	TIGRP2P87846_rs8974542	66819964	0	0	0	A
6	BICF2P366596	66828097	A	0	0	G
6	BICF2P259637	66847812	A	1	1	G
6	BICF2P863609	66858570	A	0	0	G
6	BICF2P14501	66862097	A	0	0	G
6	BICF2S23046678	66883624	A	0	0	G
6	BICF2S23041561	66889192	G	0.5	0.6429	A
6	TIGRP2P87870_rs8668245	66902464	0	0	0	G
6	BICF2P1122952	66911325	G	0	0	A
6	BICF2P1117112	66919806	A	0	0	G
6	TIGRP2P87887_rs9006687	66930729	A	0.5	0.3571	G
6	TIGRP2P87898_rs8648936	66966180	G	0	0	A
6	BICF2P632821	66984348	A	0	0	G
6	BICF2P1020096	66996378	G	0	0	A
6	BICF2P1020095	66996422	C	0	0	G
6	TIGRP2P87904_rs9154233	66999450	0	0	0	C
6	TIGRP2P87908_rs8687876	67013727	A	0	0	G
6	TIGRP2P87911_rs8726459	67026653	A	0.5	0.3571	G
6	BICF2S23349193	67033548	G	0	0	A
6	TIGRP2P87922_rs9045499	67054707	A	0	0	G
6	BICF2P775687	67067226	T	0	0	A
6	BICF2P1421363	67074357	0	0	0	A
6	BICF2S2454706	67085485	G	0.5	0.3571	A
6	BICF2S23634245	67105134	A	0.5	0.6429	G
6	BICF2P343289	67114265	G	0.5	0.6429	A

6	TIGRP2P87938_rs8513787	67128016	C	0	0	A
6	TIGRP2P87952_rs8809702	67143919	A	0.5	0.3571	C
6	BICF2P1164286	67155823	A	0	0	G
6	BICF2S2324871	67169821	A	0	0	G
6	BICF2S23647753	67183786	G	0.5	0.3571	A
6	TIGRP2P87959_rs9244341	67204158	A	0	0	G
6	BICF2P27847	67204751	A	0	0	G
6	BICF2P297165	67212203	A	1	1	G
6	BICF2P686463	67226027	A	0.5	0.3571	G
6	BICF2P1282892	67229211	A	0	0	C
6	BICF2P1084225	67240735	G	0	0	A
6	BICF2P430659	67258797	A	0.5	0.6429	G
6	BICF2S23310481	67268734	C	0	0	A
6	TIGRP2P87989_rs8619708	67278471	G	0.5	0.3571	A
6	BICF2P346884	67292148	A	0.5	0.3571	G
6	BICF2P176632	67308118	A	0	0	G
6	BICF2S23749721	67320890	A	0	0	G
6	BICF2S23239841	67321474	C	0	0	A
6	BICF2P1145302	67332752	A	0	0	G
6	BICF2S230177	67354357	C	1	1	A
6	BICF2P61322	67361641	G	1	1	A
6	BICF2P374466	67370352	0	0	0	C
6	BICF2P108249	67397978	G	0	0	A
6	BICF2P383598	67399369	G	0	0	A
6	BICF2S2345174	67405431	A	0	0	G
6	BICF2P262094	67417508	G	1	0.7857	A
6	TIGRP2P88017_rs9084389	67425837	C	0	0	G
6	TIGRP2P88018_rs8882849	67443634	0	0	0	A
6	BICF2P1259755	67454253	A	0	0	G
6	BICF2P46672	67463283	A	1	0.7857	G
6	TIGRP2P88026_rs8545457	67472378	G	1	1	A
6	BICF2P123127	67489915	G	1	0.7857	A
6	BICF2P1391161	67494676	G	1	0.7857	A
6	BICF2P406913	67511458	G	0	0.2143	C
6	BICF2P1089887	67555484	0	0	0	A
6	BICF2S23711715	67565957	A	1	1	G
6	BICF2S23735459	67587892	A	1	1	G
6	BICF2P109297	67610534	A	0	0	G
6	BICF2P1078745	67620294	A	0	0	G
6	BICF2S23522685	67635723	A	0	0	G
6	BICF2S23322911	67648412	G	0	0	A
6	TIGRP2P88050_rs8957070	67655533	0	0	0	C
6	BICF2S22952477	67669535	A	0	0	G
6	BICF2S23634218	67692981	G	0.5	0.8571	A
6	BICF2P543455	67704985	C	0.5	0.8571	A
6	BICF2P239501	67714997	A	0	0	G
6	BICF2P801725	67728215	C	0	0.2143	A

6	BICF2S23162143	67740404	G	0.5	0.6429	A
6	BICF2P155156	67753563	C	0.5	0.8571	A
6	BICF2P324985	67768191	T	0	0	A
6	BICF2P1198835	67770282	A	0.5	0.8571	C
6	BICF2P1110684	67775354	A	0	0	C
6	BICF2P1184416	67783598	G	0.5	0.3571	A
6	BICF2P324620	67793210	A	1	1	G
6	BICF2P299462	67808849	C	0.5	0.1429	A
6	BICF2P1221778	67818628	A	0	0.2143	G
6	BICF2G630573273	67834338	A	0.5	0.3571	G
6	BICF2G630573244	67847074	A	0	0.2143	G
6	BICF2G630573233	67851949	A	0	0	G
6	BICF2P198994	67893838	A	0.5	0.6429	T
6	BICF2G630573191	67897023	G	0.5	0.6429	A
6	BICF2S22910689	67918720	A	0	0	G
6	BICF2G630573177	67923317	G	0	0	A
6	BICF2G630573169	67930904	G	1	1	A
6	BICF2G630573137	67949934	0	0	0	G
6	BICF2G630573118	67964071	C	0.5	0.1429	A
6	BICF2P219331	67966280	A	0.5	0.1429	G
6	BICF2P467171	67977534	G	0	0	A
6	BICF2S23523195	67996689	A	0.5	0.3571	G
6	BICF2P636222	68005585	G	0.5	0.3571	A
6	BICF2P400965	68013544	C	0.5	0.1429	A
6	BICF2P1194903	68023411	G	0.5	0.3571	A
6	BICF2P1001147	68039925	G	0.5	0.6429	A
6	BICF2S23710808	68048150	C	0	0.2143	A
6	BICF2P894688	68065946	A	0.5	0.6429	G
6	BICF2P485436	68078786	0	0	0	A
6	BICF2S23510762	68088453	A	0	0.2143	G
6	TIGRP2P88075_rs9115791	68091983	A	0	0.2143	G
6	BICF2P923338	68107437	A	0.5	0.6429	G
6	BICF2P582670	68116914	G	0.5	0.6429	C
6	BICF2G630573116	68132137	A	0	0	G
6	BICF2G630573115	68142369	A	0.5	0.1429	C
6	BICF2G630573110	68152302	A	0.5	0.6429	G
6	BICF2G630573094	68161399	G	0.5	0.6429	A
6	BICF2G630573073	68172816	A	0.5	0.6429	G
6	BICF2S23255894	68186895	A	1	1	G
6	TIGRP2P88098_rs8728244	68196470	G	0	0	A
6	BICF2G630573048	68213519	A	0	0	G
6	BICF2G630573031	68220557	A	0	0	G
6	BICF2S2369626	68235041	A	0	0	G
6	BICF2G630573016	68241115	A	0	0	G
6	BICF2S23347682	68256055	A	0	0	T
6	BICF2G630573004	68269550	A	1	1	G
6	BICF2G630572983	68282055	A	0	0.2143	G

6	BICF2G630572971	68293289	A	0	0.2143	C
6	BICF2G630572952	68306710	G	0	0	A
6	BICF2G630572926	68320274	A	1	1	G
6	BICF2P1198195	68330390	A	0	0	G
6	BICF2P1198194	68330591	A	0	0	G
6	BICF2G630572917	68336556	T	0	0	A
6	BICF2G630572901	68347721	A	0	0	C
6	BICF2G630572886	68358631	A	1	1	G
6	BICF2G630572879	68370518	A	1	1	G
6	BICF2G630572874	68380212	G	0.5	0.1429	A
6	BICF2G630572873	68383151	G	0.5	0.8571	A
6	BICF2P287138	68392742	G	0.5	0.1429	A
6	BICF2P828538	68404556	A	0.5	0.1429	G
6	BICF2G630572846	68408807	A	0.5	0.8571	G
6	BICF2G630572815	68423132	A	0.5	0.8571	G
6	BICF2P990234	68446744	A	0.5	0.1429	G
6	BICF2G630572784	68449683	A	0	0	G
6	BICF2G630572773	68466572	G	0.5	0.8571	A
6	BICF2G630572763	68478547	A	0	0	C
6	BICF2G630572761	68483989	A	0.5	0.8571	G
6	BICF2P888785	68495522	G	0	0	A
6	BICF2P1238488	68508114	A	0.5	0.1429	G
6	BICF2G630572755	68509298	G	0.5	0.1429	A
6	TIGRP2P88173_rs8612773	68523446	A	0	0	C
6	BICF2G630572750	68531198	A	0	0	G
6	BICF2S23439792	68540728	0	0	0	G
6	BICF2G630572729	68556807	G	0.5	0.1429	A
6	BICF2P1073529	68568342	A	0.5	0.8571	T
6	BICF2P809655	68583646	A	0.5	0.1429	G
6	BICF2P1186343	68586074	C	0.5	0.1429	A
6	BICF2P796344	68601035	0	0	0	A
6	BICF2G630572687	68610084	G	0	0	A
6	BICF2G630572654	68628036	A	0.5	0.1429	G
6	BICF2S23410562	68638546	A	0.5	0.1429	G
6	BICF2P830055	68647443	G	0	0	A
6	BICF2G630572619	68655641	A	0	0	G
6	TIGRP2P88207_rs8995254	68667359	A	1	1	G
6	TIGRP2P88209_rs8749702	68678425	A	0.5	0.1429	G
6	BICF2G630572617	68694011	C	0.5	0.8571	A
6	BICF2G630572607	68705808	C	0.5	0.8571	A
6	BICF2G630572605	68717119	A	0.5	0.8571	G
6	BICF2G630572579	68734996	A	0	0	G
6	BICF2G630572577	68735988	C	1	1	A
6	BICF2G630572556	68747098	A	1	1	G
6	BICF2G630572545	68765481	C	1	1	A
6	BICF2G630572527	68780605	C	0	0	A
6	BICF2G630572525	68783217	G	0	0	A

6	BICF2G630572508	68797504	G	0	0	A
6	BICF2G630572498	68807112	G	0	0	A
6	BICF2G630572494	68816023	0	0	0	G
6	BICF2G630572474	68827684	G	0	0	A
6	BICF2G630572471	68841417	A	0	0	G
6	BICF2G630572467	68851349	A	0	0	G
6	BICF2G630572454	68864798	A	1	1	G
6	BICF2G630572434	68882603	0	0	0	G
6	BICF2G630572433	68887501	0	0	0	G
6	BICF2G630572426	68902359	A	0	0	G
6	BICF2G630572418	68909274	C	0.5	0.1429	A
6	BICF2G630572402	68926540	G	0.5	0.1429	A
6	BICF2G630572392	68931118	A	0.5	0.8571	C
6	BICF2P815584	68942897	G	1	1	A
6	BICF2G630572390	68965078	0	0	0	G
6	BICF2G630572389	68975190	A	0	0	G
6	BICF2G630572381	68977603	A	0	0	G
6	BICF2G630572362	68989053	A	0.5	0.8571	G
6	BICF2G630572361	68989141	G	0.5	0.8571	A
6	BICF2P577782	69006776	0	0	0	A
6	BICF2P968416	69012192	G	0.5	0.1429	A
6	BICF2S23259928	69014755	G	0.5	0.1429	A
6	BICF2P761846	69026590	A	0	0	G
6	BICF2G630572341	69036068	A	0	0	C
6	BICF2G630572329	69054934	A	0.5	0.8571	G
6	BICF2S23413396	69058491	G	0.5	0.1429	A
6	BICF2G630572328	69073696	A	0.5	0.1429	G
6	BICF2G630572304	69084508	G	0	0	A
6	BICF2G630572296	69095972	G	0.5	0.8571	A
6	BICF2G630572278	69108670	A	0.5	0.8571	G
6	BICF2S23030658	69116077	A	0.5	0.8571	G
6	BICF2G630572242	69130298	0	0	0	G
6	BICF2G630572226	69148497	A	0.5	0.1429	C
6	TIGRP2P88217_rs8529227	69159095	0	0	0	G
6	BICF2G630572225	69166870	G	0.5	0.1429	A
6	BICF2G630572214	69177030	A	1	1	G
6	BICF2G630572195	69185202	C	0	0	A
6	BICF2G630572169	69206440	G	0.5	0.1429	A
6	BICF2G630572168	69206590	C	0.5	0.1429	G
6	BICF2G630572158	69215614	0	0	0	C
6	BICF2G630572151	69220759	A	0.5	0.8571	G
6	BICF2G630572121	69234173	G	1	1	A
6	BICF2G630572097	69249185	C	1	1	A
6	BICF2G630572094	69255086	A	1	1	G
6	BICF2G630572077	69268155	A	1	1	G
6	BICF2G630572067	69280162	C	0	0	A
6	BICF2G630572055	69293986	0	0	0	G

6	BICF2P11186	69307867	G	0	0	A
6	BICF2G630572032	69315785	A	0	0	G
6	BICF2G630572026	69322847	A	0	0	G
6	BICF2S23450785	69334609	A	0	0	C
6	BICF2P741779	69352028	A	1	1	C
6	BICF2P1321640	69357154	G	0	0	A
6	BICF2G630571987	69378590	0	0	0	A
6	BICF2G630571976	69383262	A	0	0	G
6	BICF2S23131736	69391301	0	0	0	A
6	BICF2G630571939	69404323	A	1	1	G
6	BICF2G630571938	69404758	A	0	0	C
6	BICF2G630571914	69415116	C	0	0	A
6	BICF2G630571902	69425637	G	1	1	A
6	BICF2G630571891	69446432	A	0	0	G
6	BICF2P534	69450217	0	0	0	A
6	BICF2G630571886	69467752	G	0	0	A
6	BICF2G630571862	69478236	C	0	0	G
6	BICF2G630571851	69485364	A	0	0	G
6	BICF2G630571833	69498341	G	0	0	A
6	BICF2G630571801	69515543	A	0	0	G
6	BICF2G630571791	69521763	A	0	0	G
6	BICF2S23227947	69535271	A	1	1	C
6	BICF2G630571779	69549989	G	0	0	A
6	BICF2G630571756	69554911	A	0	0	G
6	BICF2P978985	69573857	G	0	0	A
6	BICF2P791349	69576459	C	0	0	A
6	TIGRP2P88231_rs8662131	69592177	0	0	0	G
6	BICF2P1102044	69603264	C	0	0	A
6	BICF2P1167880	69612597	G	0	0	A
6	BICF2P552062	69626188	A	1	1	T
6	BICF2S23118026	69640038	G	0	0	A
6	BICF2S23047724	69651912	A	0	0	G
6	BICF2P415988	69655530	A	0	0	C
6	BICF2P238345	69668645	0	0	0	A
6	BICF2P1135739	69689962	A	0	0	G
6	BICF2P557419	69692263	C	1	1	A
6	BICF2S23259242	69705462	0	0	0	G
6	BICF2P514941	69727722	A	1	1	G
6	BICF2P316667	69755370	A	1	1	G
6	BICF2P919927	69763702	G	1	1	A
6	BICF2P1419435	69781135	0	NA	NA	A
6	BICF2P1419434	69782832	A	1	1	G
6	BICF2S23119668	69797565	A	0	0	G
6	BICF2P1150732	69811618	A	1	1	G
6	BICF2S23239619	69819204	A	0	0	G
6	BICF2S2357281	69843812	A	0	0	C
6	BICF2P796501	69856388	G	1	1	A

6	BICF2S23110903	69864585	A	0	0	C
6	BICF2P821432	69876423	A	0	0	G
6	BICF2P1255412	69886959	A	0	0	G
6	BICF2S23059063	69899919	C	0	0	A
6	BICF2P360686	69915204	C	1	1	A
6	BICF2P462186	69920481	A	0	0	G
6	G422f56S108	69960727	A	0	0	G
6	BICF2P370767	69986447	C	NA	0	A
6	BICF2P1203147	69991835	A	0	0	T
6	BICF2P532865	70028101	A	0	0	C
6	BICF2P174770	70038051	0	0	0	A
6	BICF2S23424061	70048676	A	0	0	T
6	BICF2P1126620	70068903	0	0	0	A
6	BICF2P473471	70072014	0	0	0	G
6	BICF2P850133	70099834	A	0	0	G
6	BICF2P1127132	70108507	G	0	0	A
6	BICF2P766236	70123215	A	0	0	G
6	BICF2P237178	70127589	G	1	1	A
6	BICF2P770690	70149497	A	0	0	G
6	BICF2P244833	70157883	A	0	0	C
6	BICF2P718076	70162613	A	0	0	G
6	BICF2S23349262	70174395	G	0	0	A
6	TIGRP2P88246_rs9060796	70190648	0	0	0	A
6	BICF2S23225643	70196875	G	0	0	A
6	BICF2S23322286	70211768	A	0	0	G
6	BICF2P1247585	70228109	G	0	0	A
6	BICF2S2379878	70235760	A	0	0	G
6	BICF2P127246	70244512	G	0	0	A
6	BICF2P564205	70256325	A	1	1	G
6	TIGRP2P88251_rs8976212	70279764	0	0	0	A
6	BICF2P187508	70288387	A	0	0	G
6	BICF2P215991	70299964	G	0	0	A
6	BICF2P160606	70315388	A	0.5	0.1429	G
6	BICF2P769302	70325800	A	0	0	G
6	BICF2P1456868	70334295	A	0	0	G
6	BICF2S229947	70349872	A	0.5	0.1429	C
6	BICF2P510334	70368083	0	0	0	T
6	TIGRP2P88254_rs8899019	70385111	0	0	0	A
6	BICF2S22919682	70389307	G	0	0	A
6	BICF2P109243	70391662	G	0	0	A
6	BICF2S2305825	70410828	A	0	0	G
6	BICF2P318436	70419224	G	0	0	A
6	BICF2P305711	70426047	A	1	1	G
6	BICF2P85610	70441464	0	0	0	A
6	BICF2P777165	70459579	A	1	1	G
6	BICF2P777166	70459789	C	1	1	A
6	BICF2P244126	70463308	A	0	0	G

6	BICF2P371728	70478601	A	1	1	C
6	BICF2P260695	70483594	G	0	0	C
6	BICF2P648327	70495341	A	0	0	C
6	BICF2P1391969	70513837	G	0	0	A
6	BICF2P786906	70531774	A	0	0	G
6	BICF2P241179	70542485	G	0	0	A
6	BICF2P1202719	70557716	G	0	0	A
6	BICF2P430368	70570384	G	1	1	A
6	BICF2P1085638	70577644	A	0.5	0.1429	G
6	BICF2P1432407	70599513	G	0.5	0.1429	A
6	BICF2P43826	70613817	A	0.5	0.8571	G
6	BICF2S23125293	70628977	A	0	0	G
6	BICF2S23653514	70635132	G	0	0	A
6	BICF2P183758	70650473	G	0	0	A
6	BICF2S2366791	70667322	A	0	0	G
6	BICF2P125006	70669774	A	0	0	G
6	BICF2P215252	70686035	C	0	0	A
6	BICF2P944385	70693324	A	0	0	C
6	BICF2P944384	70694273	A	0.5	0.8571	T
6	BICF2P798130	70704499	A	0	0	C
6	BICF2P674314	70715707	A	0	0	G
6	BICF2S23252760	70732303	A	0.5	0.1429	G
6	BICF2P1188110	70745600	A	0	0	G
6	BICF2P1083703	70749705	A	0	0	G
6	BICF2P123652	70760663	G	0	0	A
6	BICF2P1011122	70771225	A	0	0	C
6	BICF2P732377	70782541	A	1	1	G
6	BICF2P1033954	70804053	G	0.5	0.8571	A
6	BICF2P1033960	70805724	G	0.5	0.8571	A
6	BICF2P976247	70817797	A	1	1	G
6	BICF2S23734291	70838530	A	0	0	G
6	BICF2P896461	70842055	A	0	0	G
6	BICF2P202183	70856463	A	0	0	G
6	BICF2P202184	70856545	C	0	0	A
6	BICF2P1171974	70872372	0	0	0	A
6	BICF2P1171978	70878760	A	0	0	G
6	BICF2P1266177	70890863	C	0	0	A
6	BICF2P118334	70904493	G	0	0	A
6	BICF2S23411165	70913743	G	0	0	A
6	BICF2S2459570	70920838	0	0	0	A
6	BICF2S2417676	70935639	G	0	0	A
6	BICF2S24413998	70950569	A	0	0	G
6	BICF2S23526760	70977982	C	0.5	0.8571	A
6	BICF2P125990	70979079	A	0.5	0.1429	C
6	BICF2P379919	70993234	0	0	0	G
6	BICF2P750185	71022423	A	0	0	G
6	BICF2P774830	71024567	A	0	0	G

6	BICF2P178881	71036304	A	0	0	G
6	BICF2S2316778	71055945	A	0	0	G
6	BICF2S23511871	71069399	A	0	0	G
6	BICF2P1090473	71072027	0	0	0	T
6	BICF2P534174	71106176	0	0	0	A
6	BICF2S23054631	71118523	A	0	0	G
6	BICF2S2338152	71128832	C	1	1	A
6	BICF2P1003263	71166179	A	0	0	G
6	BICF2P968020	71178138	A	0	0	G
6	BICF2P194669	71188883	G	0	0	A
6	BICF2P278250	71206938	G	0	0	A
6	BICF2P1092818	71209437	G	0	0	A
6	BICF2P286564	71222563	G	0	0	A
6	BICF2P734218	71235285	A	0	0	G
6	BICF2S23334364	71246449	A	0	0	G
6	BICF2P66349	71272760	C	0	0	A
6	BICF2P217996	71280052	A	1	1	G
6	BICF2S23327348	71289199	G	0	0	A
6	BICF2S2342887	71330375	A	0.5	0.1429	G
6	BICF2S22954263	71355163	C	0.5	0.8571	A
6	BICF2S23021210	71364719	G	0.5	0.8571	A
6	BICF2S22913549	71372515	A	0.5	0.8571	G
6	BICF2S23347134	71382935	G	0.5	0.8571	A
6	BICF2P775657	71395864	A	0	0	G
6	BICF2P379040	71414610	A	0.5	0.8571	G
6	BICF2P379036	71415268	G	0.5	0.8571	A
6	BICF2P165393	71430023	G	0.5	0.8571	A
6	BICF2S2347266	71439666	A	0.5	0.8571	G
6	BICF2P697297	71457203	G	0	0	A
6	BICF2P1457018	71476122	A	0	0	G
6	BICF2P746794	71489443	G	0	0	A
6	BICF2P913072	71497292	G	0.5	0.1429	A
6	BICF2S23157686	71513929	A	0.5	0.8571	G
6	BICF2S23130075	71519314	G	0.5	0.8571	A
6	BICF2S23738230	71535698	G	0.5	0.1429	A
6	BICF2S2345146	71543769	G	0.5	0.8571	A
6	BICF2P425812	71581770	0	0	0	C
6	BICF2P242526	71594911	A	0	0	G
6	BICF2S23510363	71601107	A	0	0	G
6	BICF2P209818	71614946	A	0	0	G
6	BICF2P1370084	71625099	G	0.5	0.1429	A
6	BICF2S2363083	71643631	A	0.5	0.8571	G
6	BICF2S2365111	71648559	A	0.5	0.1429	G
6	BICF2S2331842	71662017	G	0.5	0.1429	A
6	BICF2P1160070	71668806	0	0	0	A
6	BICF2S22935080	71684058	A	0.5	0.1429	G
6	BICF2P331886	71692695	A	0.5	0.8571	G

6	BICF2P33955	71704228	G	0.5	0.8571	A
6	BICF2P1402560	71725438	T	0.5	0.8	A
6	BICF2P822352	71731729	C	0	0	A
6	BICF2P184822	71737269	G	0.5	0.1429	A
6	BICF2P593866	71751153	A	0.5	0.8571	G
6	BICF2S23120846	71762138	G	0.5	0.5714	A
6	BICF2S23535398	71784545	G	0	0	A
6	BICF2S2326272	71797161	G	0.5	0.5714	C
6	BICF2S2332700	71818728	A	1	1	G
6	BICF2S23746914	71831263	A	0.5	0.1429	G
6	G423f44S93	71855698	A	0.5	0.1429	G
6	BICF2S23652830	71903756	A	0.5	0.5714	C
6	BICF2P431072	71920760	A	0	0	G
6	BICF2P589894	71924639	A	0	0	T
6	BICF2P1369667	71933773	A	0	0.25	G
6	BICF2P29925	71945467	0	0	0	A
6	BICF2P497211	71962654	0	0	0	A
6	BICF2P568467	71977180	G	0.5	0.8571	A
6	BICF2P590845	71986567	A	0.5	0.5714	G
6	TIGRP2P88273_rs9057744	71994261	0	0	0	A
6	BICF2P1301244	72005602	G	0.5	0.5714	A
6	BICF2P127255	72016084	G	0.5	0.5714	A
6	BICF2P588863	72025139	G	0.5	0.5714	A
6	BICF2P1132412	72037407	0	0	0	A
6	TIGRP2P88275_rs9200172	72054571	0	0	0	A
6	BICF2S23638221	72068994	C	0	0	A
6	BICF2S22933176	72089371	C	0	0	A
6	BICF2P1323527	72117550	G	1	1	A
6	BICF2P193948	72140488	0	0	0	G
6	BICF2P1328062	72168896	0	0	0	C
6	BICF2P1059840	72181674	T	0.5	0.5714	A
6	BICF2P322288	72185613	A	0	0	C
6	BICF2P1112745	72202530	G	0.5	0.8571	A
6	BICF2P284643	72218483	A	0.5	0.9167	C
6	BICF2P977459	72220582	A	0.5	0.8571	G
6	BICF2S23015345	72234102	G	0.5	0.8571	A
6	BICF2S23228049	72247761	G	0	0	A
6	BICF2P1271261	72265391	A	0.5	0.4286	G
6	BICF2P1271262	72266604	G	0	0	A
6	BICF2P643804	72282176	A	0.5	0.4167	G
6	BICF2P1255497	72291280	A	0	0	G
6	BICF2P181876	72306265	C	0.5	0.4286	A
6	BICF2P878053	72314083	A	0.5	0.4286	G
6	BICF2P362018	72328023	G	0.5	0.4286	A
6	BICF2P554770	72335962	G	0.5	0.4286	A
6	BICF2S22947516	72347457	A	0	0	G
6	BICF2S22937046	72359595	A	1	1	T

6	BICF2P961465	72380556	0	0	0	C
6	BICF2P714132	72384660	A	0.5	0.5714	G
6	BICF2S22964623	72400262	G	0	0	A
6	BICF2S23748940	72401422	G	0	0	A
6	BICF2P1393281	72415023	0	0	0	C
6	TIGRP2P88290_rs8547271	72417795	0	0	0	C
6	BICF2P329796	72435684	G	0.5	0.5833	A
6	BICF2P122000	72438956	G	0.5	0.1429	A
6	BICF2S23332924	72453644	G	1	0.7143	A
6	BICF2P1324811	72467299	G	0	0	A
6	BICF2S23643946	72475149	A	0.5	0.4286	G
6	BICF2S23529473	72490699	G	0	0	A
6	BICF2P334934	72504751	A	0.5	0.5714	G
6	BICF2P506441	72507755	A	0.5	0.5714	G
6	BICF2P439101	72520346	G	0.5	0.5714	A
6	BICF2S23148551	72539418	A	0.5	0.5714	G
6	BICF2P218273	72545779	A	0.5	0.5714	G
6	BICF2P805047	72564345	A	0.5	0.4286	G
6	BICF2S23762778	72573329	G	0.5	0.5714	A
6	BICF2P1311743	72585432	G	0.5	0.8571	A
6	BICF2S23626313	72595118	C	0	0	A
6	BICF2P1166527	72609680	G	0	0	A
6	BICF2P356164	72618779	G	1	1	A
6	BICF2P356165	72619068	A	1	1	G
6	BICF2P312019	72629375	A	1	1	C
6	BICF2P1387978	72641234	G	0	0	A
6	BICF2P1454460	72649835	A	0	0	T
6	BICF2P1022465	72658286	C	0	0.2857	A
6	BICF2P943740	72688795	G	0	0.2857	A
6	BICF2P73013	72702379	G	0	0.2857	A
6	BICF2S23719210	72709784	C	0	0	A
6	BICF2S23714184	72716327	A	0	0	C
6	BICF2P414630	72726284	A	0	0	G
6	BICF2P854480	72738098	A	0	0	G
6	BICF2P404935	72755203	A	0	0.2857	G
6	BICF2P404934	72755336	G	0	0.2857	A
6	BICF2S2375248	72767225	A	0	0.2857	G
6	BICF2P4923	72781031	A	1	0.7143	G
6	BICF2P304243	72794199	0	0	0	C
6	BICF2P897032	72802181	A	1	0.7143	G
6	BICF2S2299076	72809082	A	0	0	G
6	BICF2P1224947	72823662	0	0	0	C
6	BICF2P485619	72830429	G	0.5	0.1429	A
6	BICF2P581006	72854280	G	0.5	0.5714	A
6	BICF2P511140	72868633	A	0	0.2857	G
6	BICF2P1317753	72882790	A	0	0.25	C
6	BICF2P614882	72888034	G	0	0	A

6	BICF2P98157	72903332	T	0.5	0.8571	A
6	BICF2S23338688	72919444	A	0.5	0.8571	G
6	BICF2S23538218	72922882	A	0	0.2857	G
6	BICF2P527430	72926157	0	0	0	A
6	BICF2P963769	72934175	A	0.5	0.5714	G
6	BICF2P402837	72942182	C	0.5	0.8571	G
6	BICF2P133145	72946219	A	0.5	0.5714	G
6	BICF2P686407	72959144	G	0.5	0.8571	A
6	BICF2P668810	72969209	G	0.5	0.5714	A
6	BICF2S23613207	72997775	G	0	0	A
6	BICF2P1305330	73002526	A	0	0	G
6	BICF2S2307686	73019046	A	1	1	C
6	BICF2S23647174	73032432	A	1	0.7143	G
6	BICF2S23649199	73040354	G	0.5	0.4286	A
6	BICF2S23351904	73051392	A	0	0	T
6	BICF2S23148911	73067672	A	0.5	0.4286	G
6	BICF2P1389770	73082222	A	1	1	G
6	BICF2P703868	73085238	A	1	0.7143	G
6	BICF2P830596	73102154	A	0.5	0.5714	G
6	TIGRP2P88313_rs8854004	73109911	A	0.5	0.5714	G
6	BICF2P483996	73119552	A	0	0	G
6	BICF2P798296	73130875	G	0.5	0.8571	A
6	BICF2P85413	73141149	G	0	0	A
6	TIGRP2P88331_rs8709844	73148302	G	0	0.2857	A
6	BICF2S2327266	73154557	0	0	0	C
6	BICF2P707555	73164693	C	0.5	0.5714	A
6	BICF2P881589	73175029	A	0.5	0.5714	C
6	BICF2P267983	73188388	A	0.5	0.5714	G
6	BICF2P775588	73199590	0	0	0	A
6	BICF2S23436694	73210407	C	0.5	0.1429	A
6	BICF2S2447385	73242689	G	0	0	A
6	BICF2P1062513	73249458	G	1	1	A
6	BICF2S22940782	73266318	A	0.5	0.5714	G
6	BICF2S23411634	73271609	G	0.5	0.4286	A
6	TIGRP2P88356_rs8798830	73278079	A	0.5	0.5714	G
6	BICF2P459443	73292541	A	0.5	0.5714	G
6	TIGRP2P88359_rs8906434	73303144	0	0	0	A
6	BICF2P967887	73323337	A	1	0.7143	G
6	BICF2P932829	73326396	A	1	0.7143	G
6	TIGRP2P88360_rs8893746	73337656	A	0.5	0.5714	C
6	BICF2P358688	73347463	A	0.5	0.4286	G
6	TIGRP2P88364_rs8826295	73379544	G	0	0	A
6	BICF2P931853	73382477	0	0	0	C
6	BICF2P417654	73393686	C	0	0.2857	G
6	BICF2P417653	73393741	G	0	0.2857	A
6	BICF2S23416431	73407675	G	0	0	A
6	BICF2S2372532	73419491	0	0	0	G

6	BICF2P1443743	73428695	G	0.5	0.4286	A
6	BICF2P162906	73447401	A	0	0	G
6	BICF2P656888	73455131	G	0	0	A
6	BICF2P875072	73463101	A	0.5	0.4286	C
6	BICF2S23332030	73476971	G	0.5	0.4286	A
6	BICF2S23339408	73494074	G	0.5	0.8571	A
6	BICF2S23350426	73500427	A	0.5	0.1429	G
6	TIGRP2P88395_rs8949591	73508005	G	0	0.2857	A
6	TIGRP2P88405_rs8717789	73520204	A	0	0	G
6	BICF2P625115	73531104	A	0	0	G
6	TIGRP2P88423_rs9068798	73543669	A	0	0	C
6	BICF2P267212	73565008	C	0.5	0.4286	A
6	BICF2P761317	73567831	A	0.5	0.4286	G
6	BICF2P761316	73568134	A	0.5	0.4286	G
6	BICF2P153983	73580426	G	0	0	A
6	TIGRP2P88438_rs8611189	73585102	A	0.5	0.1429	C
6	BICF2P1244996	73592117	A	1	0.7143	G
6	BICF2P1259871	73602618	A	0.5	0.4286	G
6	BICF2P715867	73621476	A	0.5	0.4286	G
6	TIGRP2P88470_rs9110064	73627568	0	0	0	G
6	BICF2S23749769	73641023	A	0	0	G
6	BICF2P1455791	73648849	G	0.5	0.8571	A
6	TIGRP2P88508_rs8796410	73666769	A	0.5	0.8571	G
6	TIGRP2P88511_rs8941260	73671755	A	0	0	G
6	BICF2P1160648	73687495	G	0	0	A
6	BICF2S23160935	73693413	G	0	0	A
6	BICF2P1068113	73709567	G	1	0.7143	A
6	BICF2S23149227	73715151	C	0	0	A
6	BICF2P867290	73728180	A	0	0.2857	G
6	BICF2P275966	73736397	C	0	0.25	A
6	TIGRP2P88532_rs8916025	73745793	G	0	0	A
6	BICF2P1166976	73753438	A	0	0	G
6	TIGRP2P88536_rs9201038	73768022	0	0	0	A
6	BICF2P729715	73773653	0	0	0	A
6	BICF2S2413530	73793071	0	0	0	C
6	BICF2P161127	73799885	A	0	0	G
6	TIGRP2P88538_rs8944227	73807528	0	0	0	G
6	BICF2P329165	73822129	A	0	0	G
6	BICF2P354557	73832185	A	0	0	G
6	TIGRP2P88547_rs8729895	73848882	C	0	0	A
6	TIGRP2P88553_rs8928382	73868957	G	0	0	A
6	BICF2P1438631	73880050	0	0	0	A
6	BICF2P448160	73888184	G	0	0	A
6	BICF2S22963307	73904712	A	0.5	0.1429	C
6	BICF2P335948	73918055	A	0	0	G
6	BICF2P725449	73930386	G	0	0	A
6	BICF2P272660	73941128	A	0	0	G

6	BICF2P586747	73948980	G	0	0	A
6	BICF2S22963558	73962437	A	0	0	G
6	BICF2P375204	73970856	A	0	0.2857	G
6	BICF2P999104	73992053	A	0	0	G
6	BICF2P1287550	74008000	0	0	0	A
6	TIGRP2P88616_rs9178615	74025199	A	1	0.75	G
6	BICF2P185141	74026413	G	1	0.7143	A
6	BICF2P1367521	74040433	A	0.5	0.4286	G
6	BICF2P245414	74055371	0	0	0	G
6	TIGRP2P88639_rs8774656	74061220	A	0	0.2857	G
6	BICF2P301469	74081016	G	0	0	A
6	BICF2P413575	74084206	A	0.5	0.5714	G
6	BICF2S23259394	74096207	A	0	0	G
6	BICF2P28719	74107904	G	0.5	0.1429	A
6	BICF2P1098947	74119767	A	1	0.7143	G
6	TIGRP2P88668_rs9224784	74130488	0	0	0	A
6	TIGRP2P88684_rs9181276	74146072	G	1	0.7143	A
6	BICF2P534486	74154414	G	1	0.7143	A
6	BICF2P744369	74173568	A	0	0	G
6	BICF2P744373	74174190	A	0	0	C
6	BICF2P99866	74177370	A	0	0	G
6	BICF2S23331238	74188800	A	0.5	0.5714	G
6	BICF2S2371518	74199593	A	0	0	G
6	BICF2S23018870	74211364	A	0	0	G
6	TIGRP2P88724_rs8764605	74227398	0	0	0	A
6	BICF2P233668	74240287	0	0	0	A
6	BICF2S23325085	74250113	G	0	0	A
6	BICF2P1408217	74264403	0	0	0	A
6	BICF2S23235888	74270189	G	0	0	A
6	BICF2P890485	74286764	0	0	0	A
6	BICF2P1435525	74295710	G	0	0	A
6	TIGRP2P88762_rs9071430	74307447	A	1	0.7143	G
6	TIGRP2P88766_rs9178817	74320335	0	0	0	G
6	BICF2S2357652	74343315	G	0.5	0.1429	A
6	BICF2S23645642	74361498	A	0.5	0.1429	G
6	BICF2P1115299	74380258	G	0	0	A
6	BICF2P535814	74392041	C	0	0	A
6	BICF2S23624920	74397096	G	0	0.2857	A
6	BICF2P303903	74438117	0	0	0	A
6	BICF2S23452751	74448756	T	0	0.2857	A
6	TIGRP2P88786_rs8685773	74456777	0	0	0	G
6	BICF2P269118	74487114	0	0	0	A
6	BICF2P234047	74498531	0	0	0	G
6	BICF2P1002568	74515773	G	0	0.2857	A
6	BICF2P1306877	74528843	A	0	0.2857	G
6	TIGRP2P88789_rs8734815	74538868	0	0	0	G
6	BICF2S23726126	74546173	G	0.5	0.8571	A

6	BICF2P931589	74563057	G	0	0.2857	A
6	BICF2P836186	74566464	A	0.5	0.5714	G
6	BICF2P1382920	74580586	G	0	0.2857	A
6	BICF2S23033728	74598977	A	0.5	0.1429	G
6	BICF2S22924139	74606314	C	0.5	0.5714	A
6	BICF2P231510	74614492	A	1	0.7143	G
6	BICF2P295274	74628324	G	0.5	0.1429	A
6	BICF2P1315	74651357	G	0	0.2857	A
6	BICF2P492151	74665975	A	0.5	0.8571	G
6	BICF2P1095280	74680907	0	0	0	G
6	BICF2P235839	74695543	0	0	0	A
6	BICF2S23140632	74729156	A	0	0.2857	G
6	BICF2S23646258	74740849	A	0	0	G
6	BICF2S23110412	74758766	A	0	0.2857	G
6	BICF2P564915	74789510	0	0	0	C
6	BICF2S2375156	74797515	G	0	0	A
6	BICF2P1300610	74818548	0	0	0	A
6	BICF2S23741441	74826524	C	0	0	A
6	BICF2S23547299	74832791	G	0.5	0.4167	A
6	TIGRP2P88793_rs9101920	74846143	0	0	0	G
6	BICF2S23614729	74857914	A	0	0	G
6	BICF2S231978	74871163	A	0.5	0.5714	G
6	BICF2S23043091	74886317	A	0	0	G
6	BICF2S23327054	74891663	G	0	0	A
6	BICF2P1096896	74900618	A	0	0	G
6	BICF2P817567	74904566	A	0.5	0.5714	G
6	BICF2P240057	74911504	C	0	0	G
6	BICF2P1158361	74923089	G	0.5	0.5714	A
6	BICF2S23660180	74936403	A	0	0	G
6	TIGRP2P88796_rs9142659	74946706	0	0	0	T
6	BICF2P1294513	74958265	A	0.5	0.4286	G
6	BICF2P33784	74966206	A	0.5	0.5714	C
6	BICF2P33798	74969244	G	0.5	0.8571	A
6	BICF2P1015313	74986920	0	0	0	G
6	BICF2P24013	74994919	A	0	0.2857	G
6	BICF2S23437098	75007081	A	0.5	0.1429	C
6	BICF2P359850	75016030	A	0	0.2857	G
6	BICF2P908029	75029817	A	0.5	0.1429	G
6	BICF2P658600	75037830	G	0	0.2857	A
6	BICF2P1337551	75071338	0	0	0	C
6	BICF2S22952842	75079825	A	0.5	0.8571	G
6	BICF2S23450256	75087788	G	0	0	A
6	BICF2P490777	75095088	0	0	0	A
6	BICF2S23010168	75107991	A	0	0.2857	G
6	BICF2P804916	75127290	G	0	0.25	A
6	BICF2S23248037	75136065	G	1	0.7143	A
6	BICF2S23347800	75144049	A	0	0.2857	G

6	BICF2P1007516	75153399	A	0	0.2857	G
6	BICF2P443955	75165859	C	0.5	0.5714	A
6	BICF2P862086	75175565	G	0.5	0.5714	A
6	BICF2P1047283	75187434	A	0	0	G
6	BICF2P1407953	75200286	A	0.5	0.5833	C
6	BICF2P1307413	75216742	0	0	0	A
6	BICF2P1195736	75238567	A	0	0	T
6	BICF2P109974	75267147	A	0	0	G
6	BICF2P239338	75303383	0	0	0	A
6	BICF2P997868	75325203	G	0.5	0.5714	A
6	BICF2P551964	75358427	A	0	0	G
6	BICF2P385532	75368737	A	0	0	G
6	BICF2P459868	75374197	G	0	0	A
6	BICF2P438177	75388901	G	0	0	A
6	BICF2P1082831	75401445	C	0	0.2857	A
6	BICF2P191111	75407163	C	0.5	0.5714	A
6	BICF2P334097	75420931	G	0	0	A
6	BICF2P521250	75430654	G	0	0	A
6	BICF2P913177	75450633	T	0	0	A
6	BICF2S23127959	75451590	C	0	0.2857	G
6	BICF2P719981	75464905	0	0	0	G
6	BICF2P1405440	75474912	G	0	0	A
6	BICF2P388109	75501828	G	0	0	A
6	BICF2P396989	75504002	A	0	0	C
6	BICF2S23746262	75515635	A	0.5	0.8571	G
6	BICF2S23327375	75546859	G	0	0	A
6	BICF2P687236	75557484	0	0	0	A
6	BICF2P359053	75572581	A	0.5	0.5714	G
6	BICF2P1351716	75589484	0	0	0	G
6	BICF2P179298	75599097	G	0.5	0.5714	A
6	BICF2P1173105	75602447	A	0.5	0.5714	G
6	BICF2P74219	75615262	A	0.5	0.5714	C
6	TIGRP2P88815_rs9106113	75631069	T	0	0	A
6	TIGRP2P88818_rs9009076	75646735	G	0.5	0.8571	A
6	BICF2P658475	75652798	A	0.5	0.1429	G
6	BICF2P869164	75667123	T	0	0.2857	A
6	BICF2P1212194	75670499	A	0	0	G
6	TIGRP2P88824_rs8960153	75682212	A	1	0.7143	G
6	BICF2S23250729	75703728	T	0.5	0.5714	A
6	BICF2P809365	75705067	0	0	0	C
6	TIGRP2P88827_rs8848253	75724521	A	0	0	G
6	BICF2P1037374	75735599	A	0.5	0.8571	C
6	BICF2P633944	75746170	0	0	0	G
6	BICF2P765150	75758702	A	0	0	G
6	TIGRP2P88831_rs9142646	75778360	A	0.5	0.5714	C
6	TIGRP2P88842_rs9157488	75789146	A	0.5	0.8571	G
6	BICF2P317996	75807066	G	0.5	0.5	A

6	TIGRP2P88851_rs8564536	75812055	A	1	0.7143	G
6	BICF2P359785	75824568	A	0	0.2857	C
6	BICF2P665588	75833905	G	0	0	A
6	BICF2P498515	75848537	A	0	0	C
6	BICF2P749141	75856156	G	0.5	0.5714	A
6	BICF2P1504861	75875701	A	0	0	G
6	BICF2P1310492	75885168	A	0	0	G
6	TIGRP2P88896_rs8726223	75905175	A	0.5	0.5714	C
6	TIGRP2P88906_rs8795441	75919926	G	0	0	A
6	BICF2P305076	75933417	G	0	0.2857	A
6	TIGRP2P88910_rs9114987	75935622	G	0.5	0.5714	A
6	TIGRP2P89016_rs8590135	75969209	0	0	0	C
6	BICF2P824783	76001303	G	0	0.2857	A
6	BICF2P809156	76024488	0	0	0	C
6	TIGRP2P89107_rs8622542	76048627	G	0.5	0.5714	A
6	BICF2P1147562	76056462	C	1	1	A
6	BICF2S23442763	76074239	G	1	0.7143	A
6	BICF2P880468	76087601	A	0	0	G
6	BICF2P591256	76091762	G	0	0	A
6	BICF2P591255	76092038	G	0.5	0.4286	A
6	BICF2S2321441	76105875	A	1	1	G
6	BICF2S23152668	76110658	A	0	0	G
6	BICF2P138775	76121128	A	1	0.7143	G
6	BICF2P456049	76136927	0	0	0	A
6	BICF2P1018238	76147740	G	0	0.2857	A
6	TIGRP2P89169_rs9003200	76153157	G	0	0.2857	A
6	BICF2P528316	76174334	A	0.5	0.5714	G
6	BICF2P512618	76176090	G	0	0	A
6	BICF2P397212	76195377	0	0	0	G
6	TIGRP2P89198_rs8699772	76205012	A	0	0	G
6	BICF2P1081	76211675	A	0	0	G
6	BICF2P447416	76224946	0	0	0	G
6	BICF2P1262995	76252577	A	0	0	G
6	BICF2P1233105	76256784	T	1	1	A
6	BICF2P1095952	76269447	A	1	0.7143	G
6	BICF2P114691	76284318	C	0	0.2857	A
6	BICF2P1002858	76295054	A	1	0.7143	G
6	BICF2S23345712	76303108	G	1	1	A
6	TIGRP2P89270_rs8874641	76314122	G	0	0.2857	A
6	TIGRP2P89283_rs9103655	76336556	0	0	0	A
6	BICF2P367142	76337419	A	0	0	G
6	BICF2S2299513	76348906	A	0	0	T
6	BICF2P590386	76360065	G	0	0	A
6	BICF2P1111660	76375871	A	0.5	0.5714	G
6	BICF2P334174	76388202	A	0	0	G
6	BICF2P1401527	76402941	A	0	0.2857	G
6	BICF2P749727	76406695	A	1	1	G

6	TIGRP2P89329_rs8967102	76427215	A	0	0.2857	G
6	TIGRP2P89332_rs8967105	76429622	G	0	0	A
6	TIGRP2P89340_rs8733507	76441764	0	0	0	G
6	BICF2S23762652	76457160	C	0.5	0.1429	A
6	TIGRP2P89361_rs8851626	76464085	A	0	0.2857	G
6	TIGRP2P89367_rs8610218	76485768	A	0	0.2857	G
6	BICF2P976691	76490783	A	0	0	C
6	BICF2P502414	76509464	A	0.5	0.4286	G
6	BICF2P502409	76510285	G	0.5	0.5714	A
6	TIGRP2P89380_rs9117561	76526051	A	0.5	0.5714	G
6	BICF2S23048592	76535885	A	0	0.2857	G
6	BICF2P351581	76555675	G	1	1	A
6	BICF2P507526	76585900	A	0	0	G
6	BICF2P75114	76590856	A	0	0	G
6	BICF2P48318	76602868	G	0	0	A
6	BICF2P365542	76620131	G	0.5	0.8571	A
6	BICF2S2358354	76629477	G	0.5	0.1429	A
6	BICF2P80894	76644031	A	0.5	0.1429	G
6	TIGRP2P89416_rs8843544	76649065	A	0	0	G
6	BICF2P287788	76660083	A	0.5	0.5714	G
6	BICF2S2352907	76672499	G	0	0	A
6	BICF2P588230	76696526	A	0	0	C
6	TIGRP2P89442_rs8576905	76709614	A	0	0.2857	G
6	BICF2P236961	76717545	A	0	0	G
6	TIGRP2P89455_rs8688528	76729583	A	0	0	G
6	BICF2P425963	76742716	A	0	0.2857	G
6	BICF2S2315720	76766559	G	0.5	0.5714	A
6	TIGRP2P89480_rs8809466	76783216	C	0.5	0.5833	A
6	TIGRP2P89486_rs9191021	76787449	A	0.5	0.5714	T
6	TIGRP2P89492_rs8982442	76812893	0	0	0	G
6	BICF2P668278	76820650	A	0.5	0.5714	G
6	BICF2P435111	76837313	A	0.5	0.5714	G
6	BICF2P897972	76848860	A	0.5	0.5714	C
6	BICF2P806375	76855792	A	0	0	G
6	TIGRP2P89530_rs9092484	76871840	A	0.5	0.5714	G
6	BICF2P274333	76894758	A	0	0	G
6	BICF2S23016992	76913741	A	0	0	G
6	BICF2P3921	76925519	0	0	0	G
6	BICF2P1140040	76943397	C	0	0	A
6	G424f52S273	76965059	A	0.5	0.5714	C
6	TIGRP2P89536_rs8541972	76973949	A	0	0	G
6	BICF2S22916589	76986467	G	0.5	0.4286	A
6	BICF2P201575	77025684	A	0.5	0.1429	G
6	BICF2P1121279	77029568	A	0.5	0.1429	C
6	BICF2P1113906	77044962	C	1	1	A
6	TIGRP2P89556_rs9065752	77060773	A	0	0	G
6	TIGRP2P89558_rs9087838	77066102	T	0.5	0.4286	A

6	BICF2P986250	77079207	0	0	0	A
6	TIGRP2P89568_rs8901978	77091639	0	0	0	A
6	TIGRP2P89571_rs9042461	77097156	A	0.5	0.8571	G
6	TIGRP2P89578_rs9177423	77110102	G	0	0.2857	A
6	BICF2G630571722	77131288	G	0	0	A
6	BICF2G630571715	77142537	0	0	0	G
6	BICF2G630571691	77155832	G	0	0	A
6	BICF2G630571675	77165570	A	0.5	0.1429	G
6	BICF2G630571666	77176597	A	0	0	G
6	BICF2G630571645	77194987	A	0	0	G
6	BICF2G630571636	77202068	A	0	0	G
6	BICF2S2328675	77220719	G	0	0.2857	A
6	BICF2G630571622	77224586	A	0.5	0.4286	G
6	BICF2G630571601	77235711	A	1	1	G
6	TIGRP2P89615_rs9130339	77249143	0	0	0	G
6	BICF2G630571589	77258104	T	0	0	A
6	BICF2G630571579	77269226	A	0.5	0.5714	G
6	BICF2P10020	77269616	A	0	0	C
6	BICF2G630571552	77284782	A	0	0	G
6	BICF2G630571535	77294008	A	0	0	G
6	BICF2G630571531	77303829	G	0	0.2857	A
6	BICF2G630571519	77321152	G	0	0	A
6	BICF2G630571505	77327179	G	0	0	A
6	TIGRP2P89636_rs8764239	77338454	G	0	0	A
6	TIGRP2P89640_rs9125050	77350505	G	0	0.2857	A
6	BICF2G630571468	77360944	G	0.5	0.4286	A
6	BICF2S23517874	77375484	G	0.5	0.4286	A
6	BICF2G630571430	77384003	A	0	0	G
6	BICF2S23443545	77399757	G	0.5	0.1429	A
6	BICF2P106731	77411902	G	0.5	0.8571	A
6	BICF2P1036828	77424934	0	0	0	A
6	BICF2G630571381	77433367	C	0.5	0.4286	G
6	BICF2P149661	77434854	G	0	0	A
6	BICF2G630571352	77449496	A	0	0.2857	G
6	BICF2G630571344	77453516	A	0	0	G
6	BICF2G630571301	77472665	A	0.5	0.8571	G
6	BICF2G630571283	77477180	A	0.5	0.1429	C
6	TIGRP2P89666_rs8832339	77489905	A	0	0.2857	C
6	BICF2G630571249	77499995	A	0.5	0.8571	G
6	BICF2G630571231	77511731	G	0	0	A
6	TIGRP2P89672_rs9170619	77530500	0	0	0	A
6	BICF2G630571229	77539214	A	0.5	0.8571	G
6	BICF2G630571215	77550213	C	0	0.25	A
6	BICF2G630571209	77556062	G	0	0	A
6	BICF2G630571199	77575204	A	0	0	C
6	BICF2G630571175	77589000	A	0.5	0.4286	G
6	BICF2G630571171	77593071	A	0	0.2857	C

6	TIGRP2P89685_rs8862852	77610032	C	1	1	A
6	BICF2P1018698	77615431	A	0	0	C
6	BICF2S23543007	77629027	A	0	0	G
6	BICF2P205523	77641341	G	1	1	A
6	TIGRP2P89693_rs8793104	77655200	G	0.5	0.1429	A
6	BICF2P991653	77656215	A	0.5	0.1429	G
6	BICF2P1463422	77665433	G	0.5	0.8571	A
6	BICF2S23216135	77673404	A	0	0	G
6	BICF2P336153	77683562	A	0.5	0.8571	G
6	BICF2P292750	77703258	G	0.5	0.8571	A
6	TIGRP2P89715_rs9194535	77716974	G	0.5	0.8571	A
6	BICF2P355360	77732992	A	0	0	G
6	BICF2P287475	77747380	A	0	0	G
6	BICF2P224656	77755003	A	0.5	0.1429	C
6	BICF2S23215733	77766545	G	0	0	A
6	BICF2S23329792	77780152	A	0.5	0.1429	C
6	TIGRP2P89768_rs9039354	77789143	C	0	0	G
6	BICF2S23554285	77794550	A	0.5	0.1429	G
6	BICF2P488646	77801106	C	0	0	A
6	BICF2P70233	77802733	G	0.5	0.8571	A
6	BICF2P70228	77804150	A	1	1	G
6	BICF2P687932	77820220	A	1	1	G
6	BICF2P687931	77821438	A	1	1	G
6	BICF2P985990	77833526	A	0	0	C
6	BICF2P1121012	77844132	G	0.5	0.8571	A
6	BICF2P267251	77855309	A	0	0	C
6	BICF2P1100851	77868129	G	0.5	0.1429	A
6	BICF2S23647777	77869556	A	0	0	C
6	BICF2P455442	77878219	G	0.5	0.1429	A
6	BICF2P776280	77893541	G	0.5	0.1429	A
6	BICF2P917627	77906343	A	0	0	G
6	BICF2P975802	77919140	A	0.5	0.1429	G
6	BICF2P913284	77925621	A	0.5	0.1429	G
6	BICF2P658322	77937130	G	0	0	A
6	BICF2P312190	77950563	A	0	0	G
6	BICF2P320455	77969303	G	0.5	0.1429	A
6	BICF2P598298	77970718	G	0.5	0.1429	A
6	TIGRP2P89849_rs9088677	77984645	A	0.5	0.8571	G
6	BICF2S2367718	77989425	A	0.5	0.8571	G
6	TIGRP2P89858_rs8895945	78003566	A	0.5	0.8571	G
6	BICF2P347412	78010943	G	0	0	A
6	BICF2P501332	78023343	A	0.5	0.8571	G
6	BICF2P501333	78023438	G	0.5	0.8571	A
6	BICF2P130884	78033560	G	0	0	A
6	TIGRP2P89886_rs9136948	78041316	A	0	0	G
6	BICF2P490359	78053987	G	0	0	A
6	BICF2P1042454	78070732	A	0	0	G

6	TIGRP2P89901_rs8679202	78081387	A	0	0	G
6	TIGRP2P89907_rs8744858	78085058	G	0	0	A
6	BICF2S23329863	78106812	A	0	0	G
6	BICF2S23039484	78113065	A	0.5	0.8571	G
6	BICF2P1349445	78120003	A	0.5	0.08333	G
6	TIGRP2P89953_rs8802163	78133433	A	0	0	G
6	BICF2P944537	78147350	A	0	0	G
6	BICF2S23256926	78163165	C	0	0	A
6	BICF2P1345539	78169276	G	0	0	A
6	BICF2S23052312	78178828	A	0	0	G
6	BICF2P283805	78191162	A	0	0	G
6	BICF2P609201	78224617	A	0.5	0.8571	G
6	TIGRP2P90023_rs8731644	78238858	A	0	0	G
6	BICF2P715457	78246394	A	0	0	G
6	BICF2P384738	78266275	A	0.5	0.8571	G
6	BICF2P1438222	78273639	G	0.5	0.1429	A
6	TIGRP2P90064_rs8803448	78282852	A	0.5	0.8571	C
6	BICF2P253208	78302460	G	0.5	0.1429	A
6	BICF2P1208554	78319174	T	0	0	A
6	TIGRP2P90090_rs9172805	78332403	A	0	0	G
6	BICF2S23754548	78341718	A	NA	0.1429	G
6	TIGRP2P90109_rs8933494	78350150	A	0	0	G
6	BICF2P698981	78369364	A	1	1	G
6	BICF2P1122840	78375810	A	0	0	G
6	BICF2S2378638	78388255	C	0.5	0.8571	A
6	BICF2S23054727	78398255	A	0	0	G
6	BICF2P812176	78410602	A	0.5	0.1429	G
6	BICF2P1107072	78421278	A	0	0	G
6	BICF2P383010	78434106	A	0.5	0.1429	G
6	TIGRP2P90178_rs8568020	78444024	A	1	1	C
6	BICF2P1419826	78458176	A	0	0	G
6	BICF2P322722	78472627	0	0	0	C
6	BICF2P1274052	78476758	C	0	0	A
6	BICF2P596471	78496186	G	0	0	A
6	TIGRP2P90200_rs8931445	78504797	A	0.5	0.1429	T
6	BICF2P82635	78523151	A	0	0	G
6	BICF2P154378	78537705	C	0	0	A
6	BICF2P150932	78545554	A	0	0	G
6	BICF2S2317682	78576602	A	1	1	G
6	BICF2P232749	78586053	A	0	0	G
6	TIGRP2P90223_rs9196765	78598270	A	0.5	0.1429	T
6	BICF2P1087067	78610092	A	0.5	0.1429	G
6	BICF2P677112	78619364	G	0.5	0.1429	A
6	BICF2S2337	78647929	A	0.5	0.1429	G
6	BICF2P1334285	78649878	A	0	0	G
6	BICF2P1364630	78656802	G	0.5	0.1429	A
6	BICF2P1364629	78656873	A	0	0	C

6	BICF2P1067521	78662955	G	0.5	0.8571	A
6	BICF2P749222	78674888	A	0	0	G
6	BICF2P481353	78688713	G	0	0	A
6	BICF2P114509	78701190	A	1	1	G
6	BICF2P635865	78717425	A	0.5	0.1429	G
6	BICF2P1369576	78724374	A	0	0	G
6	BICF2P515745	78729918	C	0	0	A
6	BICF2P946100	78749491	G	1	1	C
6	BICF2P1365605	78759316	A	0.75	0.8571	G
6	BICF2P852676	78773626	A	0.75	0.8571	G
6	BICF2P596466	78780504	A	0.75	0.8571	G
6	BICF2P520012	78807439	A	0.75	0.8571	G
6	BICF2P1186362	78818305	A	0.75	0.8571	C
6	BICF2S2323334	78821808	A	0.25	0.1429	G
6	TIGRP2P90254_rs8899382	78838342	A	0.25	0.1429	G
6	BICF2G630571102	78854967	G	0.75	0.8571	A
6	BICF2G630571098	78859005	A	0.75	0.8571	G
6	BICF2G630571063	78877361	G	0	0	A
6	BICF2G630571052	78882272	A	0	0	G
6	BICF2G630571046	78894075	G	0	0	A
6	BICF2G630571029	78902564	G	0.25	0.1429	A
6	BICF2G630570981	78923730	A	0	0	G
6	BICF2G630570976	78924967	0	0	0	G
6	BICF2G630570942	78938009	A	0.75	0.8571	G
6	BICF2G630570917	78953541	A	1	1	G
6	TIGRP2P90273_rs9219697	78961846	A	0.25	0.1429	G
6	BICF2G630570912	78993367	A	0	0	G
6	BICF2P632554	79025251	G	0.75	0.8571	A
6	BICF2G630570882	79035462	C	1	1	A
6	TIGRP2P90278_rs8673950	79047497	G	0.75	0.8571	A
6	BICF2P1039536	79055321	G	0	0	A
6	BICF2P1070924	79062734	G	0	0	A
6	BICF2G630570876	79078366	G	0.25	0.1429	A
6	BICF2G630570870	79098475	A	0	0	C
6	BICF2G630570828	79131152	C	0	0	A
6	BICF2P462796	79140998	C	0	0	A
6	BICF2G630570799	79152504	A	0	0	G
6	BICF2G630570795	79155022	A	0	0	G
6	TIGRP2P90304_rs9158318	79173845	G	0.75	0.8571	A
6	BICF2G630570756	79178284	C	0	0	A
6	BICF2G630570744	79192747	A	0.75	0.8571	G
6	BICF2G630570715	79204016	A	0.25	0.1429	G
6	TIGRP2P90334_rs8927380	79214019	A	0	0	G
6	BICF2G630570676	79226258	A	1	1	G
6	BICF2P640478	79243487	G	1	1	A
6	BICF2G630570637	79250248	A	0	0	C
6	BICF2P1025924	79262757	G	0.25	0.1429	C

6	BICF2G630570603	79270786	A	0	0	G
6	TIGRP2P90355_rs8719794	79283709	A	0	0	G
6	TIGRP2P90358_rs9189865	79292627	A	0	0	C
6	BICF2G630570590	79317026	C	0	0	A
6	BICF2P1185001	79327939	G	0.25	0.1429	A
6	TIGRP2P90382_rs8812416	79345687	A	0	0	G
6	BICF2P694137	79352702	G	0	0	A
6	BICF2G630570582	79369311	A	0.75	0.8571	G
6	BICF2G630570563	79381705	0	0	0	G
6	BICF2G630570558	79385833	A	0.75	0.8571	G
6	BICF2S23328480	79393593	G	0	0	C
6	BICF2P332851	79402284	A	0.25	0.1429	G
6	TIGRP2P90393_rs8724614	79407506	A	0.25	0.1429	G
6	BICF2S23763215	79426005	G	0	0	A
6	BICF2P202069	79433131	A	1	1	G
6	BICF2P435754	79450340	A	0	0	C
6	BICF2G630570544	79461380	A	0.25	0.1429	G
6	BICF2P641351	79469669	G	0	0	A
6	TIGRP2P90428_rs8762061	79490525	A	0	0	G
6	BICF2S22913845	79501672	G	0.25	0.1429	A
6	BICF2G630570502	79513645	C	0.25	0.1429	A
6	BICF2P175783	79523880	G	0	0	A
6	BICF2P12416	79543521	G	0.75	0.8571	A
6	BICF2G630570484	79548435	A	0.25	0.1429	G
6	BICF2S23752047	79558311	A	0	0	C
6	BICF2P1142550	79570471	G	0	0	A
6	BICF2S23653917	79572769	A	0	0	G
6	BICF2G630570431	79588874	G	0	0	A
6	BICF2G630570417	79593084	A	0	0	G
6	BICF2G630570409	79594656	C	0	0	G
6	BICF2S2324738	79603643	A	0	0	G
6	BICF2P526944	79619818	A	1	1	G
6	BICF2G630570379	79620341	0	0	0	G
6	BICF2S22960558	79627199	A	1	1	G
6	BICF2S23159500	79643019	A	0	0	G
6	BICF2G630570329	79653798	A	0	0	G
6	BICF2G630570319	79661399	G	0	0	A
6	BICF2G630570315	79664152	A	0	0	C
6	BICF2G630570308	79677555	C	1	1	A
6	BICF2P780655	79683554	0	0	0	A
6	BICF2G630570306	79698951	A	0	0	G
6	BICF2P946428	79709984	G	1	1	A
6	BICF2G630570285	79733707	G	1	1	A
6	BICF2G630570263	79748480	G	0	0	A
6	BICF2P1319096	79753433	T	0	0	A
6	BICF2G630570238	79761402	A	1	1	G
6	BICF2G630570229	79765304	A	0	0	G

6	BICF2G630570203	79784818	G	0	0	A
6	BICF2S22919917	79796333	A	1	1	G
6	BICF2G630570184	79798730	G	0	0	A
6	BICF2G630570129	79819593	G	0	0	A
6	BICF2G630570117	79822385	A	0	0	G
6	BICF2G630570094	79838277	A	0	0	G
6	BICF2S23444390	79860449	A	0	0	G
6	BICF2G630570093	79873595	C	1	1	G
6	BICF2P990631	79886377	A	0	0	G
6	BICF2G630570091	79894347	A	0	0	G
6	BICF2S2451868	79905810	A	0	0	G
6	BICF2G630570090	79930175	G	0	0	A
6	BICF2G630570089	79937701	A	0	0	G
6	BICF2S23019861	79959761	G	1	1	A
6	BICF2G630570066	79976113	C	0	0	A
6	BICF2P1437917	79989182	A	0	0	G
6	BICF2G630570063	79998676	0	0	0	G
6	BICF2G630570056	80010327	G	0	0	A
6	BICF2G630570015	80024561	A	0	0	G
6	BICF2G630569973	80034349	G	0	0	A
6	BICF2G630569932	80049347	C	0	0	A
6	BICF2G630569913	80052507	A	1	1	G
6	BICF2G630569896	80064278	A	0	0	G
6	BICF2G630569895	80064431	A	0	0	G
6	BICF2G630569866	80074594	A	0	0	G
6	BICF2G630569848	80086981	C	0	0	G
6	BICF2G630569781	80107118	C	0	0	A
6	BICF2G630569767	80110074	A	0	0	G
6	BICF2G630569762	80125519	A	0	0	C
6	BICF2G630569745	80136261	A	0	0	G
6	TIGRP2P90441_rs9108911	80150708	A	0	0	G
6	BICF2P549043	80163639	A	0	0	G
6	BICF2P954715	80170583	0	0	0	A
6	BICF2G630569630	80181529	C	1	0.7143	G
6	BICF2P809231	80192882	0	0	0	G
6	TIGRP2P90492_rs8889669	80202011	C	0	0.25	A
6	BICF2P1224444	80215967	A	0	0	G
6	BICF2G630569509	80216255	G	0	0.2857	A
6	BICF2S22961359	80235158	G	1	1	A
6	BICF2G630569466	80236663	G	0	0	A
6	BICF2P1337653	80258425	G	0	0	A
6	BICF2P1325660	80263908	G	0	0.2857	A
6	BICF2G630569435	80266066	A	0	0	G
6	TIGRP2P90534_rs8846113	80276383	G	1	1	A
6	BICF2P1469956	80293811	A	1	0.7143	G
6	BICF2P1228141	80306453	A	1	1	G
6	TIGRP2P90570_rs9017915	80322245	A	0	0.2857	G

6	BICF2G630569422	80329910	A	0	0.2857	G
6	TIGRP2P90594_rs9046282	80342637	C	0	0.2857	A
6	BICF2G630569364	80352567	G	0	0	A
6	BICF2G630569331	80360330	A	1	1	G
6	BICF2G630569329	80362700	A	1	1	G
6	BICF2S23146068	80378136	A	0	0	G
6	BICF2G630569299	80390760	G	0	0	A
6	BICF2G630569296	80391056	G	0	0	A
6	BICF2P813737	80428081	0	0	0	A
6	BICF2G630569263	80442009	A	0	0	G
6	TIGRP2P90646_rs9044326	80446432	A	0	0	C
6	BICF2G630569243	80458945	G	0	0	A
6	BICF2G630569238	80465917	G	0	0.2857	A
6	BICF2G630569224	80478466	0	0	0	G
6	BICF2G630569195	80496194	G	0	0	A
6	BICF2G630569149	80510494	G	1	0.7143	A
6	BICF2G630569118	80520221	A	0	0	G
6	BICF2G630569076	80531300	G	1	0.8	A
6	BICF2P329604	80539636	G	1	0.7143	A
6	BICF2P1348382	80546112	0	0	0	C
6	BICF2G630569070	80558053	G	0	0.2857	A
6	BICF2S23726453	80582380	A	0	0.2857	G
6	BICF2P1088577	80601423	G	0	0	A
6	BICF2P1370303	80608513	0	0	0	A
6	BICF2P223115	80621071	C	0	0	A
6	TIGRP2P90680_rs8450920	80639060	0	0	0	G
9	BICF2P309837	3003393	0	NA	NA	NA
9	BICF2P1444473	3013641	G	0	0	A
9	BICF2P132632	3027208	A	0	0	G
9	BICF2S23741434	3043254	A	0	0	C
9	BICF2P1397556	3054369	0	0	0	C
9	BICF2S23733311	3080156	A	0	0	G
9	BICF2P830234	3082799	0	0	0	A
9	TIGRP2P244850_rs8950347	3100030	G	0	0	A
9	TIGRP2P244847_rs9239061	3106843	G	0	0	A
9	TIGRP2P244844_rs9253216	3125432	A	0	0	G
9	BICF2P1237427	3131299	G	0	0	A
9	TIGRP2P244841_rs8989099	3141597	G	0	0	A
9	BICF2S22942334	3154536	G	0	0	A
9	BICF2P554683	3167765	G	0	0	A
9	BICF2S23340023	3183571	G	0	0	C
9	BICF2S23354403	3210246	G	0	0	A
9	BICF2P110834	3225499	A	0	0	G
9	BICF2P794118	3240903	0	0	0	G
9	BICF2P840357	3245548	0	0	0	C
9	BICF2P194139	3275563	A	0	0	G
9	BICF2P72435	3280976	G	0	0	A

9	BICF2P958180	3299230	G	0	0	A
9	BICF2P1060077	3321882	0	0	0	G
9	BICF2P841099	3329060	C	0	0	A
9	TIGRP2P244817_rs8572936	3351660	0	0	0	C
9	BICF2S23030432	3378703	G	0	0	A
9	BICF2P286006	3381125	0	0	0	A
9	TIGRP2P244815_rs8774266	3403240	0	0	0	G
9	BICF2S23244057	3425707	G	0	0	A
9	BICF2P969948	3430398	G	0	0	A
9	BICF2P138811	3445601	G	0	0	A
9	BICF2P701114	3479656	0	0	0	C
9	BICF2P684091	3485210	G	0	0	A
9	BICF2S23441614	3496412	G	0	0	A
9	TIGRP2P244803_rs8968860	3515526	A	0	0	G
9	BICF2P399249	3529583	0	0	0	G
9	BICF2S23638127	3532061	A	0	0	G
9	BICF2S23032400	3560551	A	0	0	G
9	BICF2P442254	3572341	A	0	0	G
9	TIGRP2P244789_rs9150379	3587572	A	0	0	G
9	BICF2P1272565	3605267	G	0	0	A
9	BICF2P347394	3619830	G	0	0	A
9	BICF2P59325	3630867	0	0	0	G
9	BICF2S23232063	3649331	A	0	0	G
9	TIGRP2P244749_rs9186811	3688990	G	0	0	A
9	BICF2S23522744	3696216	G	0	0	A
9	BICF2P1270062	3712133	G	0	0.2857	A
9	TIGRP2P244745_rs9188376	3721827	A	0	0	G
9	BICF2P1347921	3736513	G	0	0.2857	A
9	BICF2P268406	3742838	A	0	0	G
9	BICF2P136294	3757431	G	0	0.2857	A
9	BICF2S2369091	3769127	A	0	0	G
9	BICF2P448454	3778324	G	0	0.2857	A
9	BICF2P1188322	3784861	0	0	0	A
9	BICF2P1224586	3804938	A	0	0.2857	G
9	BICF2P233220	3816341	G	0	0.2857	C
9	BICF2P157033	3823030	A	0	0.2857	C
9	BICF2P268912	3832098	G	0	0.2857	A
9	BICF2P605513	3839816	A	0	0	G
9	BICF2P1320606	3841631	G	0	0.2857	A
9	BICF2P1206077	3873847	G	0	0	A
9	BICF2P616658	3875656	A	0	0	G
9	TIGRP2P244726_rs8678790	3898318	0	0	0	C
9	BICF2P801989	3905549	A	0	0	G
9	BICF2P70804	3915649	A	0	0	G
9	BICF2P1036569	3924413	A	0	0	G
9	BICF2P683447	3938471	0	0	0	G
9	BICF2P295598	3953212	G	0	0	A

9	BICF2P1460864	3966228	A	0	0	G
9	BICF2P173550	3969432	G	0	0	C
9	TIGRP2P244718_rs8924886	3986916	0	0	0	G
9	BICF2P213155	4011385	C	0	0	A
9	BICF2P164422	4014078	A	0	0	C
9	BICF2P95254	4030521	G	0	0	A
9	BICF2S23128734	4059047	A	0	0	C
9	BICF2S2319928	4078769	A	0	0	G
9	TIGRP2P244717_rs8844324	4109330	0	0	0	G
9	BICF2S2301425	4125282	G	0	0	A
9	BICF2P716522	4128865	G	0	0	A
9	BICF2P1194613	4148948	G	0	0	A
9	BICF2P2739	4165832	A	0	0	C
9	BICF2P1055859	4177519	A	0.75	0.3571	G
9	BICF2P256734	4187195	A	0.75	0.3571	G
9	BICF2P627887	4197730	A	0.25	0.6429	G
9	TIGRP2P244711_rs8949157	4213366	0	0	0	C
9	BICF2P520294	4230004	A	0.25	0.6429	G
9	BICF2P259807	4240274	A	0.75	0.3571	C
9	BICF2P103448	4248260	C	0.75	0.3571	A
9	BICF2P1059543	4263260	A	0.25	0.6429	G
9	BICF2P1303684	4272356	A	0.75	0.3571	C
9	BICF2P1199338	4289779	A	0.75	0.3571	G
9	BICF2P831138	4304182	A	0.75	0.3571	G
9	BICF2P234941	4322043	A	0.25	0.3571	C
9	BICF2P899484	4325620	A	1	0.3571	C
9	BICF2P427807	4338705	A	0.75	0.3571	G
9	BICF2P749895	4347640	A	0.75	0.3571	G
9	BICF2P184132	4363516	0	0	0	A
9	BICF2P226103	4390582	A	0.25	0.3571	G
9	BICF2P324692	4402765	A	0.25	0.3571	G
9	BICF2P744273	4436023	A	0.25	0.3571	G
9	BICF2P965193	4444004	A	0.75	0.3571	G
9	BICF2P965192	4444114	A	0.75	0.3571	G
9	BICF2P119329	4462345	A	0.25	0.3571	G
9	BICF2P922847	4473756	A	0.25	0.3571	G
9	BICF2P1317072	4496926	G	0.75	0.6429	A
9	BICF2P1390768	4512376	A	0.25	0.3571	G
9	BICF2P155230	4522141	A	0	0	G
9	BICF2P627209	4536272	G	0	0	A
9	TIGRP2P244704_rs8952541	4553045	0	0	0	G
9	BICF2P691118	4561259	0	0	0	A
9	BICF2P184113	4566379	A	0	0.3571	G
9	BICF2P250163	4588715	0	0	0	C
9	BICF2P820938	4603201	A	0	0	G
9	BICF2P1056783	4636012	A	0.25	0.3571	G
9	BICF2P1179388	4658226	C	1	0.8333	A

9	BICF2P1072538	4674228	A	0	0.3333	G
9	BICF2P1072474	4689128	A	0.25	0.3333	G
9	BICF2P1444921	4705912	A	0	0.3571	G
9	BICF2P735856	4715091	A	0.75	0.6429	G
9	BICF2P1214913	4726767	A	0.25	0.3571	G
9	BICF2P1267051	4768338	0	0	0	A
9	BICF2P169431	4795182	0	0	0	C
9	BICF2P1193661	4798507	C	0.75	0.6429	G
9	BICF2P996727	4807590	G	0.75	0.6429	A
9	BICF2P487612	4822035	A	0.75	0.6667	G
9	BICF2P223820	4836906	0	0	0	G
9	BICF2P224432	4845027	A	0	0	G
9	BICF2P1209975	4858418	G	1	1	A
9	BICF2P1247278	4865944	A	1	1	G
9	BICF2P1300027	4883060	A	1	1	G
9	BICF2P1347247	4899862	G	1	1	A
9	BICF2P852486	4943755	G	1	1	A
9	BICF2P771286	4946089	A	1	1	G
9	BICF2P673827	4977665	A	1	1	G
9	BICF2S23426543	4997475	G	1	1	A
9	BICF2P563223	5024537	A	1	1	G
9	BICF2P1200528	5044124	C	1	1	G
9	BICF2P857977	5062201	G	1	1	A
9	BICF2P96019	5080936	G	1	1	A
9	BICF2S22937780	5112797	A	1	1	G
9	BICF2P864531	5124088	A	1	0.7143	G
9	BICF2P1150673	5130129	A	0	0.25	G
9	BICF2P408847	5141573	A	1	1	G
9	BICF2P869571	5177026	G	1	1	A
9	BICF2P804906	5195258	G	1	1	A
9	BICF2P872141	5200958	A	1	1	G
9	BICF2P1416153	5204985	C	1	1	A
9	BICF2P1132001	5233847	A	1	1	G
9	BICF2P1183694	5248473	A	1	1	G
9	BICF2P463719	5258778	A	0	0	G
9	BICF2P267469	5271171	G	1	1	A
9	BICF2S23645081	5285445	A	1	1	G
9	BICF2P1385899	5299287	G	1	1	A
9	BICF2P1350420	5306932	G	1	1	A
9	BICF2P554565	5323129	G	0	0	A
9	BICF2P195421	5324996	G	0	0	A
9	BICF2P1188376	5343955	G	0	0	A
9	BICF2P656660	5357579	0	0	0	C
9	BICF2P1325828	5360416	A	1	1	G
9	BICF2P528414	5379266	A	0	0	G
9	BICF2S23542688	5418477	A	0	0	C
9	TIGRP2P244687_rs9056937	5433293	0	0	0	G

9	TIGRP2P244686_rs9009094	5440910	0	0	0	C
9	TIGRP2P244684_rs9092387	5459287	0	0	0	G
9	BICF2P494704	5484443	0	0	0	A
9	BICF2P1221332	5492178	0	0	0	T
9	BICF2P852723	5510716	0	0	0	G
9	TIGRP2P244683_rs8522195	5524660	0	0	0	C
9	BICF2P1296339	5533847	A	0	0.2857	G
9	BICF2S2316668	5578646	A	1	0.7143	G
9	BICF2P939527	5595094	A	1	0.7143	G
9	BICF2P31454	5602570	G	1	0.7143	A
9	BICF2S23529960	5629690	G	1	0.7143	A
9	TIGRP2P244677_rs8888207	5636449	0	0	0	C
9	BICF2P728294	5649783	G	0	0	C
9	BICF2P1313720	5658065	G	1	0.7143	A
9	BICF2P1194003	5675117	C	0.25	0.3571	G
9	BICF2P611764	5698719	A	0	0	G
9	BICF2P1260537	5716798	G	1	0.7143	A
9	BICF2P207992	5741115	0	0	0	G
9	BICF2S23544437	5759628	A	0	0	G
9	BICF2P904399	5767271	A	0	0	G
9	BICF2P526583	5782703	C	0	0	A
9	TIGRP2P244671_rs8725181	5816952	0	0	0	C
9	TIGRP2P244670_rs8988033	5825638	0	0	0	T
9	BICF2P456937	5845522	0	0	0	G
9	BICF2P889625	5853780	G	0	0	A
9	BICF2P966162	5901616	C	0	0	A
9	BICF2P304540	5911404	A	0.25	0.3571	G
9	BICF2P836857	5928592	A	0	0	G
9	BICF2P140759	5944905	G	0.25	0.3571	A
9	BICF2P1392759	5959030	A	1	0.7143	G
9	BICF2P622301	5984381	G	1	0.7143	A
9	BICF2P855323	5992385	G	1	0.7143	A
9	BICF2S22910807	6009257	A	1	0.7143	G
9	BICF2P1294857	6022410	G	1	0.7143	A
9	BICF2P945314	6030133	C	1	0.7143	A
9	BICF2P377014	6044667	A	1	0.7143	G
9	BICF2P394340	6052783	A	1	0.7143	G
9	BICF2S22943430	6061889	A	1	0.75	G
9	BICF2S22913460	6072373	G	1	0.7143	A
9	BICF2S23413605	6085552	G	1	0.7143	A
9	BICF2P953611	6099107	G	1	0.7143	A
9	BICF2P186704	6110568	A	1	0.7143	G
9	BICF2P1127873	6132381	0	0	0	G
9	BICF2S23512153	6149205	G	1	0.7143	A
9	BICF2P978767	6161782	A	0.25	0.3571	G
9	BICF2P256046	6185084	G	1	0.7143	A
9	TIGRP2P244661_rs9243072	6217483	0	0	0	C

9	BICF2P71116	6232762	G	1	0.7143	A
9	BICF2P414122	6243813	A	1	0.7143	G
9	BICF2P1228201	6250500	G	1	0.7143	A
9	BICF2P72714	6263917	A	0.75	0.3571	G
9	BICF2P1080530	6276407	G	1	0.7143	A
9	BICF2P1205920	6287948	A	0	0.3333	G
9	BICF2P810872	6295173	C	0.25	0.3571	A
9	BICF2P1277084	6321732	A	0.25	0.3571	G
9	BICF2P393194	6325224	A	1	0.7143	C
9	BICF2P1262435	6347039	G	1	0.7143	A
9	BICF2P1262453	6348724	G	1	0.7143	A
9	BICF2P1249203	6366494	G	1	0.7143	A
9	BICF2P1360950	6376684	A	1	0.7143	G
9	BICF2S22945525	6382596	A	1	0.7143	G
9	BICF2S23626446	6406006	0	0	0	A
9	BICF2P121771	6417009	A	0	0	G
9	BICF2P141355	6446459	A	0.25	0.3571	C
9	BICF2S2371747	6454842	G	0.25	0.3571	A
9	BICF2S2306423	6458145	G	0.25	0.3571	A
9	BICF2P297297	6468770	A	0.25	0.3571	G
9	BICF2S23248780	6487472	G	0	0	C
9	BICF2P470586	6500333	A	0.25	0.3571	G
9	BICF2S22917678	6514707	A	0.25	0.3571	G
9	TIGRP2P244630_rs8860448	6525894	A	0.25	0.3571	G
9	BICF2P935843	6547923	A	0.75	0.3571	C
9	BICF2S2376938	6563089	G	1	0.7143	C
9	BICF2P198850	6567056	0	0	0	G
9	BICF2P439040	6587949	A	0.25	0.3571	G
9	BICF2P896646	6594891	G	0.25	0.3571	A
9	TIGRP2P244614_rs8946181	6614677	G	1	1	A
9	BICF2P1370930	6633689	A	0	0	G
9	BICF2P940041	6653789	G	0	0.6429	A
9	BICF2P382223	6667156	0	0	0	G
9	BICF2S24312404	6677751	G	0	0.2857	A
9	BICF2P116234	6687854	0	0	0	A
9	BICF2P141709	6695493	0	0	0	G
9	BICF2S23541884	6732872	A	0	0.2857	G
9	BICF2S2346403	6789895	A	0	0.2857	G
9	BICF2P368167	6837703	A	0	0	G
9	BICF2P241722	6848680	A	0	0	G
9	BICF2P1354324	6876692	A	0	0	G
9	BICF2P515988	6878982	G	0	0	A
9	BICF2P824003	6908882	G	0	0	A
9	BICF2S2369345	6923651	A	1	0.875	C
9	BICF2P1124396	6941577	0	0	0	C
9	BICF2P669255	6991759	0	0	0	A
9	G536f30S68	7013238	C	1	0.7143	G

9	BICF2P1174471	7040145	0	0	0	G
9	BICF2P157561	7066516	0	0	0	T
9	BICF2P154738	7085971	G	0	0	A
9	BICF2P729876	7103950	0	0	0	G
9	BICF2P1186188	7110867	0	0	0	A
9	BICF2P1219682	7139903	0	0	0	G
9	BICF2P710431	7166748	G	0.25	0.3333	A
9	TIGRP2P244559_rs8950703	7202963	0	0	0	G
9	BICF2P513201	7221808	A	1	0.7143	G
9	BICF2P1221538	7230119	A	0.25	0.3571	G
9	BICF2P538387	7235627	A	0.25	0.3571	G
9	BICF2P668766	7240846	C	0.25	0.3571	A
9	BICF2P928888	7253654	0	0	0	G
9	BICF2P558685	7263326	A	0.25	0.6429	G
9	BICF2P19222	7268964	A	0.25	0.6429	G
9	BICF2P662701	7282996	G	0.25	0.6429	A
9	BICF2P447753	7306156	G	0.25	0.6429	A
9	BICF2P1348152	7318939	A	0.25	0.3571	G
9	BICF2S23138713	7331616	G	0	0.2857	A
9	BICF2P819101	7359959	A	0.25	0.3571	G
9	BICF2P398943	7369340	0	0	0	A
9	BICF2S23257313	7387061	G	0	0.2857	A
9	BICF2P638023	7398271	G	0.75	0.3333	A
9	BICF2P465233	7413021	0	0	0	A
9	BICF2P703333	7428533	A	0.75	0.3	G
9	BICF2P1069150	7432781	G	0.25	0.3571	A
9	BICF2P1071003	7486678	A	0	0	G
9	BICF2P817756	7527606	A	1	0.7143	G
9	BICF2S23231067	7546270	A	0	0	G
9	BICF2P991630	7557388	0	0	0	G
9	BICF2P1259319	7607760	A	0	0	G
9	BICF2P256822	7616531	A	0	0	G
9	BICF2P1363582	7646478	A	0	0	G
9	BICF2P351474	7656919	C	0	0	A
9	BICF2S23228452	7660265	C	0.25	0.3571	A
9	BICF2P502725	7706900	A	0	0.2857	G
9	BICF2P405024	7740690	A	0	0	C
9	BICF2P798232	7749901	A	0	0	G
9	BICF2P1248308	7761269	A	0.25	0.6429	G
9	BICF2P1254128	7771846	0	0	0	G
9	BICF2P400944	7780879	0	0	0	G
9	BICF2P5055	7795848	0	0	0	A
9	BICF2P791305	7797572	0	0	0	G
9	BICF2P1157774	7809290	0	0	0	A
9	TIGRP2P244548_rs9155085	7827755	0	0	0	G
9	BICF2S235394	7842960	G	0	0	A
9	BICF2P92535	7849130	A	0	0	G

9	BICF2P1399341	7856753	G	0	0	A
9	BICF2P421408	7866889	C	0	0	G
9	BICF2P792643	7877238	G	0	0	A
9	BICF2S23237942	7878750	A	0	0	C
9	BICF2P185590	7890288	G	0	0	A
9	BICF2P266426	7910870	A	0	0	C
9	BICF2P811478	7921784	A	0	0	G
9	BICF2S23253303	7933094	A	0	0	G
9	BICF2S23246968	7936804	A	0	0	G
9	BICF2S23749849	7955062	A	0	0	G
9	BICF2P544809	7961259	G	1	1	A
9	BICF2P842963	7977677	A	1	1	G
9	BICF2S23123114	7989771	G	0	0.2857	A
9	BICF2P1146417	7995172	0	0	0	G
9	BICF2S23413192	8025763	A	0	0.2857	G
9	BICF2S23241212	8051619	G	0	0	A
9	BICF2P921631	8116977	0	0	0	A
9	BICF2S23541863	8132931	A	0	0.2857	G
9	BICF2P392700	8169640	G	1	0.7143	A
9	BICF2S23414459	8179290	A	1	0.7143	G
9	BICF2S23113017	8188410	A	0	0	G
9	BICF2P321941	8202822	G	0.75	0.6429	A
9	BICF2P731229	8218637	C	0.75	0.6429	A
9	BICF2P575283	8227319	A	0	0	G
9	BICF2P1183309	8238053	A	0	0	G
9	BICF2P245252	8263008	0	0	0	A
9	BICF2S23316992	8289711	G	0.75	0.6429	A
9	BICF2S23537746	8296622	G	0	0	A
9	BICF2P276094	8309417	0	0	0	G
9	BICF2P856386	8366381	G	0	0	A
9	BICF2P1115290	8387885	A	0	0	T
9	BICF2P813271	8400741	C	0.75	0.3571	A
9	BICF2P375591	8426431	A	0.75	0.3571	G
9	BICF2P979767	8435625	G	0	0	A
9	BICF2P1316318	8456132	G	0	0	A
9	BICF2P45969	8468416	0	0	0	G
9	TIGRP2P244501_rs8911449	8476716	G	1	0.7143	A
9	TIGRP2P244495_rs9188088	8497276	A	1	0.7143	G
9	BICF2P1349021	8502759	A	0.75	0.3571	G
9	BICF2P521103	8519991	A	0	0	G
9	BICF2S2324840	8521556	G	0	0	A
9	BICF2P28358	8541186	A	0.25	0.6429	G
9	BICF2P1337346	8550804	A	0	0.2857	G
9	BICF2P1361804	8562589	G	0.25	0.6429	A
9	BICF2P1160370	8571082	G	0	0	A
9	BICF2P636332	8591522	A	0	0.2857	G
9	BICF2P926324	8633413	0	0	0	G

9	TIGRP2P244432_rs8600181	8641341	A	0	0.2857	G
9	BICF2S2335305	8692157	A	0	0	C
9	BICF2G630514210	8710809	0	0	0	A
9	BICF2S23748085	8717858	A	0	0	G
9	BICF2P929776	8731380	G	0	0.2857	A
9	BICF2S23633834	8750365	A	0	0.2857	G
9	BICF2S23548695	8751749	C	0	0	A
9	TIGRP2P244384_rs8620813	8767407	G	0	0.2857	A
9	BICF2P1173052	8775229	G	0	0.2857	A
9	TIGRP2P244374_rs8830379	8789024	C	1	1	A
9	BICF2S23028001	8789349	A	1	0.7143	G
9	BICF2S2372734	8804096	A	0	0	G
9	BICF2P611733	8821605	G	0	0.2857	A
9	BICF2P218127	8860558	G	1	1	A
9	BICF2P715898	8881793	A	0	0.25	G
9	TIGRP2P244225_rs9071880	8891074	G	1	1	A
9	BICF2P1354705	8916877	G	1	0.7143	A
9	BICF2P352724	8921084	G	1	1	A
9	BICF2S22946443	8928511	G	0	0	A
9	BICF2P437730	8941446	G	0	0	A
9	BICF2P1235993	8954043	A	0.25	0.6429	G
9	BICF2S2347189	8958780	G	0.25	0.6429	A
9	BICF2P1055455	8975617	C	0.75	0.3571	A
9	BICF2P728609	9003244	A	0	0	G
9	BICF2P1032377	9011074	0	0	0	A
9	BICF2S23419353	9017842	A	0.75	0.6429	G
9	BICF2P13314	9037260	A	0.75	0.6429	G
9	BICF2P891181	9051582	0	0	0	C
9	BICF2P626187	9062013	A	0	0	G
9	TIGRP2P244173_rs8752743	9075222	0	0	0	G
9	TIGRP2P244160_rs8529979	9086680	A	0.75	0.3571	G
9	BICF2P777844	9097312	G	0	0	A
9	BICF2P962490	9108563	G	0	0	A
9	BICF2S23643546	9123341	0	0	0	G
9	BICF2S23243300	9133948	A	0	0	G
9	BICF2P782	9146472	A	0.25	0.3571	C
9	BICF2S22933128	9157981	G	1	1	A
9	BICF2P320395	9178052	A	0.75	0.3571	G
9	BICF2S23221624	9196645	C	0.25	0.6429	A
9	BICF2P397747	9207371	G	0.25	0.6429	A
9	BICF2S23621627	9227203	A	0	0	G
9	BICF2P964746	9246801	0	0	0	C
9	BICF2P99329	9258202	A	0	0	G
9	BICF2S23045885	9274047	A	0	0	C
9	BICF2S23146883	9287565	G	0.75	0.3571	A
9	TIGRP2P244114_rs8944033	9309337	C	0.25	0.3571	A
9	BICF2P656088	9321674	A	0.75	0.6429	G

9	BICF2P455637	9335475	G	0	0.2857	A
9	BICF2S2357941	9338667	G	0	0.2857	A
9	TIGRP2P244100_rs8584979	9352952	G	1	0.7143	A
9	BICF2P610379	9362699	A	0	0	G
9	TIGRP2P244070_rs9203066	9372692	A	0.25	0.3571	G
9	BICF2P626535	9395979	G	0.75	0.6429	A
9	TIGRP2P244045_rs8626775	9407065	C	0.75	0.6429	A
9	BICF2P203661	9420705	A	0.75	0.6429	G
9	BICF2P741904	9429200	G	0	0.2857	A
9	BICF2P689752	9433961	A	0	0	G
9	BICF2S23516497	9444922	A	0	0.5	G
9	BICF2P727235	9453323	A	0.25	0.3571	G
9	BICF2P165382	9464159	A	0	0.2857	G
9	BICF2P280964	9467330	A	0	0	C
9	BICF2S22955626	9478807	A	0	0	C
9	BICF2S2319702	9489390	A	0	0	C
9	BICF2P1250900	9511166	0	0	0	G
9	BICF2P24946	9528527	A	0.75	0.6429	G
9	BICF2P1090424	9539490	C	0	0	A
9	TIGRP2P243987_rs8707593	9546502	G	0	0	A
9	BICF2S23547735	9550154	G	0	0	A
9	BICF2S23245057	9556816	A	0	0	G
9	BICF2P423920	9570112	A	0	0	G
9	BICF2P979406	9590495	A	0	0.2857	G
9	BICF2P962122	9635676	G	0	0	A
9	BICF2P668086	9641640	G	0	0	A
9	BICF2P323560	9651202	A	0	0	G
9	BICF2P1048929	9665631	G	0	0.2857	A
9	TIGRP2P243943_rs8984949	9674101	A	0.25	0.3571	G
9	BICF2P1386634	9683637	A	0	0	G
9	BICF2P731720	9698571	0	0	0	G
9	BICF2P818748	9707377	G	0	0.2857	A
9	BICF2P37950	9721569	A	0.75	0.3571	G
9	TIGRP2P243919_rs8829102	9740270	G	0.25	0.6429	A
9	BICF2P635650	9746605	A	1	0.7143	G
9	BICF2S2371174	9767940	A	0	0	G
9	BICF2P279223	9780390	A	0	0.2857	G
9	TIGRP2P243901_rs8638165	9793418	G	0	0.2857	A
9	TIGRP2P243888_rs8839038	9807822	A	0.75	0.3333	G
9	TIGRP2P243877_rs8690482	9813917	A	0.75	0.3571	G
9	BICF2P685191	9821483	A	0.25	0.6429	G
9	TIGRP2P243856_rs9245242	9848647	G	0	0	A
9	BICF2S23414555	9864708	G	0	0.2857	A
9	BICF2P456875	9873881	G	0	0	A
9	TIGRP2P243843_rs8964028	9884753	A	0	0.2857	G
9	BICF2P64870	9896168	0	0	0	G
9	BICF2P887528	9924234	0	0	0	G

9	BICF2P536304	9929430	G	0.75	0.3571	A
9	BICF2P1038107	9946607	0	0	0	C
9	BICF2P701301	9952101	A	0	0	G
9	BICF2S2327894	9967584	A	0	0	G
9	BICF2P221975	9973892	G	0.25	0.3571	A
9	BICF2P855850	10001350	A	0	0	G
9	BICF2P349863	10009854	0	0	0	C
9	BICF2P966011	10020630	A	0.25	0.3571	G
9	BICF2P1239566	10030103	A	0.25	0.3571	G
9	BICF2P1392893	10034878	A	0	0	G
9	BICF2S23712029	10047459	G	0	0	A
9	BICF2P436503	10052928	C	0	0	G
9	BICF2S23741121	10071409	C	0.75	0.3571	A
9	TIGRP2P243791_rs8982854	10081549	A	0.75	0.3571	C
9	BICF2P750687	10093162	A	0.25	0.3571	G
9	BICF2P381480	10097808	A	0	0.2857	G
9	BICF2S23242393	10110199	A	0.75	0.3571	G
9	BICF2P970233	10141068	0	0	0	G
9	BICF2S23052466	10146281	0	0	0	G
9	BICF2S23730374	10170617	A	0.25	0.6429	G
9	BICF2S23719057	10182891	G	0.75	0.3571	A
9	BICF2P925276	10193401	A	0.25	0.6429	G
9	BICF2P879094	10202657	G	0	0	A
9	BICF2S23732437	10218128	A	1	0.8	C
9	BICF2P149841	10224327	A	0	0	G
9	BICF2S2399369	10239350	G	0.75	0.6429	A
9	BICF2S22910017	10257520	A	0	0	G
9	BICF2P711993	10267084	A	0.75	0.6429	G
9	BICF2P277127	10270761	G	1	0.6429	A
9	BICF2P1297148	10312522	0	0	0	A
9	TIGRP2P243747_rs8708523	10326003	0	0	0	C
9	BICF2S23215671	10327677	G	0.75	0.6429	A
9	BICF2S23153415	10343899	A	0	0	G
9	BICF2S23349590	10361250	G	0	0	A
9	BICF2S23134559	10383390	A	0	0	G
9	BICF2P1160161	10385450	A	0.25	0.3571	C
9	BICF2P1012878	10396165	A	0	0	G
9	G538f3S225	10457477	0	0	0	G
9	BICF2P772589	11016676	A	0.25	0.6429	G
9	BICF2S22923671	11017587	A	0.5	0.2143	G
9	BICF2P653614	11035711	G	0.5	0.2143	A
9	BICF2P485955	11042919	G	0	0	A
9	BICF2P180721	11058279	A	0.25	0.6429	G
9	BICF2P546600	11060904	G	0	0	A
9	BICF2S23126858	11073501	A	0.25	0.3571	G
9	BICF2S23149682	11080084	G	0	0	A
9	BICF2P327202	11087739	G	0.75	0.6429	C

9	BICF2P1007553	11096033	G	0.5	0.5	A
9	BICF2P115345	11105909	A	0	0	G
9	BICF2P1203749	11127261	0	0	0	G
9	BICF2P140556	11138110	0	0	0	A
9	BICF2P778472	11163581	0	0	0	A
9	BICF2P1042076	11179357	0	0	0	C
9	BICF2P857354	11185885	G	0.5	0.5	A
9	BICF2P250243	11200004	G	0.5	0.5	A
9	BICF2P253618	11203479	0	0	0	G
9	TIGRP2P243118_rs9185033	11218916	G	0	0	A
9	BICF2P616838	11233808	A	0	0	G
9	BICF2P440293	11245719	A	0.75	0.3571	G
9	BICF2S23763343	11251472	A	0	0.2857	G
9	BICF2P444733	11268256	A	0.75	0.3333	C
9	BICF2P339122	11272935	G	0.75	0.6429	A
9	BICF2P1049068	11282236	A	0	0.3571	G
9	BICF2P1342856	11300669	A	0	0	G
9	TIGRP2P243136_rs9121564	11314912	0	0	0	G
9	BICF2P1145008	11328994	0	0	0	C
9	BICF2P1354703	11343152	G	0.5	0.5	A
9	BICF2P953607	11354174	A	0	0	G
9	BICF2P10798	11367132	G	0	0	A
9	TIGRP2P243147_rs9245286	11377029	A	0	0	G
9	BICF2P512882	11405635	G	0	0.2857	A
9	BICF2P606189	11415150	G	0	0.2857	A
9	BICF2S23538808	11423002	A	0	0	G
9	TIGRP2P243174_rs8966116	11434594	G	1	0.7143	A
9	BICF2P808374	11450001	A	0.75	0.3571	G
9	BICF2P804025	11460670	0	0	0	G
9	BICF2P51571	11474798	A	0	0.2857	G
9	BICF2P1337350	11480159	C	0	0.2857	A
9	BICF2P292699	11498881	A	0.75	0.6429	G
9	BICF2P263280	11502193	A	0	0	G
9	BICF2P1380950	11517483	A	1	0.7143	G
9	BICF2S23511312	11534072	A	0	0.2857	G
9	BICF2S23713967	11536081	A	0	0.2857	G
9	BICF2P1228574	11546155	A	0	0.2857	G
9	BICF2S23328283	11559342	A	0.25	0.3571	T
9	BICF2P863951	11574672	A	0	0.2857	C
9	BICF2P220406	11608558	A	0	0	G
9	TIGRP2P243249_rs9007698	11615060	A	0	0	G
9	BICF2G63024982	11670349	A	0.5	0.3571	C
9	BICF2G63025061	11695602	A	0	0	G
9	BICF2G630828624	12081543	A	0.5	0.7857	T
9	BICF2G630828606	12104387	A	0.25	0.4286	G
9	BICF2G630828600	12113869	A	0.25	0.4286	G
9	BICF2G630828582	12158850	A	0.5	0.2143	G

9	BICF2P1356457	12172779	A	0	0	G
9	BICF2P728553	12190139	G	0	0	A
9	BICF2P96143	12205289	0	0	0	A
9	TIGRP2P123259_rs8861485	12234937	0	0	0	A
9	BICF2S23512001	12236398	G	0.25	0.3571	A
9	BICF2G630828573	12259606	C	0.5	0.2143	G
9	BICF2G630828572	12276518	A	0	0	G
9	BICF2S23231950	12285340	A	0.25	0.3571	C
9	BICF2G630828570	12293676	A	0.25	0.4286	C
9	BICF2G630828563	12308534	A	0.25	0.4286	G
9	BICF2G630828560	12320643	A	0	0	C
9	BICF2S23514060	12333375	A	0.25	0.3571	G
9	BICF2G630828559	12342760	A	0	0	G
9	BICF2P245050	12355242	0	0	0	A
9	BICF2G630828557	12362714	A	0.5	0.5	G
9	BICF2G630828545	12387591	G	0	0.2857	A
9	BICF2P578469	12403067	0	0	0	A
9	TIGRP2P123251_rs9238067	12414354	0	0	0	G
9	BICF2G630828525	12448438	A	0.75	0.3571	C
9	BICF2P354191	12482575	C	0	0	A
9	TIGRP2P123245_rs8651764	12489128	A	0	0	G
9	BICF2P669538	12514538	A	0	0	C
9	BICF2P528397	12526847	G	0	0	A
9	BICF2P370965	12537598	G	0	0	C
9	BICF2P19746	12553807	G	0	0	C
9	BICF2S23649154	12558840	A	0.5	0.2143	G
9	BICF2P368360	12569650	0	0	0	A
9	BICF2P358317	12585309	G	0	0	A
9	BICF2P1195477	12599017	C	0	0	A
9	BICF2G630828515	12614648	G	0	0	A
9	BICF2P168526	12625641	0	0	0	A
9	BICF2S2343967	12638917	A	0	0	G
9	BICF2G630828506	12657770	A	0	0	G
9	BICF2P1208798	12671217	A	1	0.7143	G
9	BICF2P1320995	12674707	A	0.5	0.7857	G
9	BICF2G630828498	12681931	A	0.25	0.1429	G
9	BICF2G630828488	12686853	A	0.5	0.2143	C
9	BICF2G630828482	12702309	A	0	0.2857	T
9	TIGRP2P123217_rs8896353	12724637	C	0.75	0.6429	A
9	BICF2S23511546	12734078	A	0	0	G
9	BICF2G630828467	12744112	G	0	0	A
9	BICF2P314583	12760974	0	0	0	A
9	BICF2P524799	12766452	0	0	0	G
9	BICF2S23750740	12781199	0	0	0	G
9	BICF2G630828431	12809715	A	0.75	0.5714	C
9	BICF2G630828423	12813252	C	0	0	A
9	BICF2P648727	12832265	0	0	0	A

9	BICF2P11418	12835223	G	0.75	0.8571	A
9	BICF2S23247266	12875468	C	0.25	0.3571	G
9	BICF2G630828413	12881946	A	0.25	0.3571	G
9	BICF2G630828406	12893076	G	0.25	0.3571	A
9	BICF2P726363	12909674	A	0	0	G
9	BICF2S23039634	12912580	G	0	0	A
9	BICF2G630828379	12914711	A	0	0	G
9	BICF2G630828367	12926154	0	0	0	G
9	BICF2G630828362	12926703	G	0.25	0.3571	A
9	BICF2G630828334	12945229	G	0	0	A
9	BICF2G630828328	12955171	G	0.25	0.3571	A
9	BICF2S23718395	12971882	G	0	0	A
9	BICF2G630828309	12994392	0	0	0	C
9	BICF2G630828307	12995515	A	0.75	0.6429	C
9	TIGRP2P131962_rs8974461	13010530	G	0	0	A
9	TIGRP2P131961_rs9236335	13021906	A	0.25	0.3571	G
9	TIGRP2P131950_rs8910756	13030246	A	0.75	0.6429	G
9	BICF2P961660	13066662	G	0.75	0.6429	A
9	TIGRP2P131929_rs8973803	13095010	0	0	0	T
9	TIGRP2P131925_rs8813533	13108682	A	0.75	0.6429	C
9	BICF2P186084	13112420	A	0	0	G
9	BICF2P742899	13127108	A	0.75	0.6429	G
9	TIGRP2P131920_rs8580006	13143634	C	0.75	0.6429	A
9	BICF2P1214081	13160299	A	0	0	G
9	TIGRP2P131915_rs9050263	13174761	G	0	0	C
9	TIGRP2P131912_rs8594406	13185078	G	0.25	0.3571	A
9	BICF2G630828274	13191289	G	0.25	0.3571	A
9	TIGRP2P131909_rs8848343	13205733	0	0	0	A
9	BICF2G630828262	13221984	A	0	0.4167	C
9	BICF2G630828258	13225794	A	0.25	0.4286	G
9	BICF2S23034713	13240551	C	0	0	A
9	BICF2P621989	13253767	0	0	0	G
9	BICF2S23748504	13269452	A	0	0	G
9	BICF2S23753880	13273294	A	0	0	G
9	BICF2P187802	13284002	C	0.75	0.5714	A
9	BICF2P365346	13294513	A	0.5	0.2143	G
9	BICF2G630828237	13307440	A	0.25	0.1429	G
9	BICF2G630828219	13320378	A	0.5	0.2143	G
9	BICF2S23764945	13332535	G	0.5	0.2143	A
9	BICF2S23237314	13438336	A	0.75	0.5714	G
9	BICF2G630827883	13454313	G	0	0	A
9	BICF2P1300961	13515707	A	0.25	0.3571	G
9	BICF2G630827759	13567652	G	0.75	0.3333	A
9	BICF2P223643	13571684	0	0	0	A
9	BICF2P1265386	13582670	0	0	0	A
9	BICF2P544093	13613546	0	0	0	A
9	G542f22S110	13633662	0	0	0	A

9	BICF2G630827752	13657473	A	0	0	G
9	BICF2P460813	13674012	0	0	0	G
9	BICF2G630827749	13695034	A	0	0	G
9	BICF2G630827748	13698292	G	0.25	0.3571	A
9	BICF2S23415407	13714015	A	0	0	G
9	BICF2G630827733	13719727	A	0.25	0.3571	T
9	BICF2P1115810	13757511	C	0	0	A
9	BICF2S23244467	13774548	A	0.25	0.3571	G
9	TIGRP2P131828_rs9035352	13806214	A	0	0	C
9	BICF2P1042592	13828479	G	0.25	0.1429	A
9	BICF2P124897	13871033	0	0	0	A
9	G543f33S329	13893710	G	0.25	0.3571	A
9	BICF2G630827725	13916697	A	0.25	0.1429	G
9	BICF2S23523540	13935624	G	0.25	0.3571	A
9	BICF2P853966	13950351	A	0.25	0.3571	C
9	BICF2P863483	13968098	0	0	0	G
9	BICF2P526954	13974999	A	0.5	0.2143	G
9	BICF2G630827709	13986340	A	0.25	0.3571	G
9	BICF2P308563	13998142	G	0.75	0.8571	A
9	TIGRP2P131821_rs8615505	14013136	A	0.25	0.1429	G
9	BICF2P1127726	14022981	A	0.5	0.5	G
9	BICF2P1237669	14032625	G	0.75	0.8571	A
9	BICF2S23512019	14047935	A	0	0.2857	G
9	BICF2P424351	14051925	G	0.5	0.7857	A
9	BICF2P1033073	14059129	A	0.5	0.7857	G
9	BICF2P892842	14069491	0	0	0	G
9	BICF2P644534	14079691	G	0.75	0.6429	A
9	BICF2P1308554	14098787	A	0.75	0.8571	G
9	BICF2P21120	14102917	A	0	0.25	G
9	BICF2P1259934	14110745	A	0	0	G
9	BICF2P1329887	14128139	A	0	0	G
9	BICF2P1231531	14137453	A	0	0	G
9	BICF2P1149155	14163383	G	0.5	0.2143	A
9	BICF2S2355861	14170453	A	0.25	0.3571	G
9	BICF2S23622394	14187950	0	0	0	G
9	BICF2S23259548	14211113	G	0.25	0.3571	A
9	TIGRP2P131789_rs9033253	14222235	G	0.25	0.1429	A
9	TIGRP2P131786_rs8941018	14245058	A	0	0.25	G
9	BICF2S23127082	14266874	G	0.5	0.2143	A
9	TIGRP2P131780_rs8534342	14278408	A	0.25	0.1429	G
9	TIGRP2P131779_rs8694839	14291692	G	0.75	0.3571	A
9	BICF2P689120	14300060	A	0.75	0.3571	G
9	BICF2P688248	14307624	A	0.25	0.1429	G
9	BICF2S23244425	14324095	A	0.25	0.3571	G
9	BICF2P770638	14335247	T	0.25	0.1429	A
9	BICF2P84414	14358471	A	0.75	0.5714	C
9	BICF2P1173655	14373459	0	0	0	A

9	BICF2P527686	14379723	G	0.75	0.5714	A
9	BICF2P403231	14393000	C	0	0	A
9	BICF2P1108446	14457021	A	0.5	0.2143	G
9	BICF2P678798	14473791	0	0	0	A
9	TIGRP2P131771_rs9148885	14488249	A	0.25	0.1429	G
9	BICF2P1119926	14492429	0	0	0	A
9	BICF2P629191	14503687	A	0.25	0.3571	G
9	BICF2P913470	14519413	A	0.25	0.3571	T
9	BICF2P324955	14527438	G	0	0	A
9	BICF2P379867	14537709	0	0	0	A
9	BICF2P601132	14553997	A	0	0	C
9	BICF2P387123	14554856	A	0.25	0.3571	G
9	BICF2P254767	14565576	A	0.5	0.2143	C
9	BICF2P1341435	14580769	C	0.75	0.5714	A
9	BICF2P912862	14588421	A	0.75	0.5714	G
9	BICF2P618781	14602013	A	0.25	0.1429	G
9	BICF2P1086543	14611563	G	0.5	0.2143	A
9	BICF2P1087214	14621075	A	0.25	0.1429	G
9	BICF2P495794	14637573	G	0.25	0.3571	A
9	BICF2P934581	14642149	A	0.5	0.2143	G
9	BICF2P1296472	14653190	G	0	0	A
9	BICF2P369789	14670083	A	0.75	0.6429	G
9	BICF2P1149635	14678086	G	0.75	0.8571	A
9	BICF2S23014061	14690753	A	0.25	0.08333	G
9	BICF2P656194	14698560	A	0.75	0.8571	G
9	BICF2P1007747	14709242	A	0.75	0.8571	G
9	BICF2P854755	14728767	A	0.25	0.1429	G
9	TIGRP2P131711_rs8831018	14736720	G	0.25	0.1429	A
9	BICF2P123287	14753177	G	0.5	0.5	A
9	TIGRP2P131708_rs8381727	14759773	G	0.5	0.5	A
9	TIGRP2P131705_rs9036896	14766492	A	0.25	0.1429	G
9	BICF2P276203	14785588	A	0.25	0.1429	G
9	BICF2P599582	14789437	G	0	0.1429	A
9	BICF2S23914746	14800605	G	0.5	0.5	A
9	BICF2P271566	14814856	G	0.5	0.5	A
9	BICF2S23612829	14827196	A	0	0	G
9	BICF2P1259277	14840296	A	0	0	G
9	BICF2P376749	14850185	G	0	0	A
9	BICF2P659974	14859269	A	0	0	G
9	BICF2P1197821	14874739	A	0	0	G
9	BICF2P859515	14901884	T	0.25	0.3571	A
9	BICF2P713072	14910639	C	0.25	0.3571	A
9	BICF2S23750795	14915740	G	0.25	0.3333	A
9	BICF2P455771	14934740	C	0	0.2857	A
9	TIGRP2P131672_rs9093294	14963127	A	0.25	0.1429	G
9	BICF2S23230471	14978017	G	0.25	0.3571	A
9	BICF2S23238050	14993918	G	0.25	0.3571	A

9	BICF2P1458281	15014208	A	0	0	G
9	BICF2P926316	15022972	A	0	0	G
9	BICF2P865898	15034819	A	0.25	0.3571	C
9	BICF2S23644982	15047240	A	0	0	G
9	TIGRP2P131659_rs8744388	15061419	G	0.75	0.6429	A
9	TIGRP2P131654_rs9177266	15066069	A	0	0	G
9	BICF2P1181220	15081113	0	0	0	G
9	BICF2P1181641	15090714	A	0	0	G
9	BICF2P646505	15104536	G	0	0	A
9	BICF2P1267851	15117102	A	0	0	C
9	BICF2P1267850	15117463	A	0.25	0.1429	G
9	TIGRP2P131639_rs9118783	15132876	G	0	0	A
9	BICF2S23411069	15139234	A	0	0	G
9	BICF2P32704	15153266	G	0.5	0.2143	A
9	BICF2P6438	15157084	A	0.5	0.2143	G
9	BICF2P394191	15172417	G	0	0	A
9	BICF2P1124044	15197640	A	0	0	G
9	BICF2P1159438	15202774	G	0	0	A
9	BICF2P1065371	15216229	0	0	0	A
9	BICF2P985600	15244740	C	0.75	0.5714	A
9	BICF2P993742	15254030	G	0.25	0.3571	A
9	BICF2P883021	15263443	A	0.25	0.4286	G
9	BICF2S23517549	15265713	G	0.25	0.3571	A
9	BICF2P539693	15278060	A	0.25	0.1429	G
9	BICF2P1382224	15287229	A	0.5	0.5	G
9	TIGRP2P131614_rs8555155	15287526	A	0.5	0.5	G
9	BICF2P893694	15291586	A	0.25	0.3571	C
9	BICF2S23743891	15292457	A	0	0	G
9	BICF2P306908	15297801	A	0.25	0.3571	G
9	BICF2P925262	15313327	C	0.25	0.3571	A
9	BICF2P446460	15317966	A	0.75	0.5714	G
9	BICF2P967935	15325764	A	0	0	G
9	BICF2P881346	15338039	A	0.25	0.3571	G
9	BICF2S2322352	15350605	A	0.25	0.3571	G
9	BICF2P835888	15357792	G	1	0.7143	A
9	BICF2P37032	15369632	A	1	0.7143	G
9	BICF2S23346021	15377558	G	0.75	0.5714	A
9	BICF2S24321721	15393031	A	0.25	0.1429	G
9	BICF2S23738913	15398956	A	0	0	G
9	BICF2P709500	15420517	G	0.25	0.1429	A
9	BICF2S23334287	15424096	A	0.5	0.5	G
9	BICF2P143573	15436412	G	0.5	0.2143	A
9	BICF2P1395227	15465264	0	0	0	A
9	BICF2P155745	15492669	A	0	0	G
9	BICF2P445217	15512206	G	0	0	A
9	BICF2S23542057	15531913	G	0.25	0.3571	A
9	TIGRP2P131600_rs8940570	15541602	0	0	0	A

9	BICF2P1219544	15551190	0	0	0	A
9	BICF2S23439872	15568397	A	0	0	G
9	BICF2P102458	15598507	A	0.5	0.2143	G
9	TIGRP2P131596_rs8565189	15626124	0	0	0	C
9	BICF2P1026047	15667471	G	0.75	0.8571	A
9	BICF2S23253915	15674728	0	0	0	G
9	BICF2P37355	15692105	0	0	0	A
9	TIGRP2P131580_rs9033169	15704308	A	0	0.2857	G
9	TIGRP2P131576_rs8802404	15725491	A	0	0	C
9	BICF2P9118	15734386	A	0.5	0.2143	G
9	BICF2P513388	15745898	A	0	0	G
9	BICF2S24110623	15756666	A	0.25	0.3571	C
9	BICF2P1381452	15779169	G	0.25	0.3571	A
9	BICF2S23234883	15811899	C	0.25	0.3571	A
9	BICF2P526229	15819057	0	0	0	A
9	TIGRP2P131561_rs9083262	15827369	T	0.25	0.3571	A
9	BICF2P147614	15839334	G	0.25	0.3571	A
9	BICF2P754719	15868398	G	0.25	0.3571	A
9	BICF2P386788	15881243	G	0	0	A
9	BICF2S23736224	15885731	C	0.25	0.3571	A
9	BICF2P954683	15903068	G	0	0	A
9	BICF2P954689	15905986	G	0	0	C
9	BICF2P954690	15906545	A	0	0	G
9	BICF2P308451	15918817	A	0	0	C
9	BICF2P434306	15933669	C	0	0	G
9	BICF2P112502	15941829	A	1	1	G
9	BICF2P1244067	15960573	A	0	0	G
9	BICF2P1198946	15966161	A	0	0	C
9	BICF2S23135452	15993425	A	0	0	C
9	BICF2P1139135	16007108	A	0.25	0.3571	G
9	BICF2P1301866	16012537	G	0.5	0.5	A
9	BICF2P120022	16020606	A	0	0	G
9	BICF2P232767	16032229	A	0	0	G
9	BICF2S22954942	16063087	A	0.25	0.3571	G
9	BICF2P1256786	16065981	0	0	0	A
9	BICF2S23041512	16077702	G	0.25	0.3571	A
9	TIGRP2P131518_rs9137737	16090815	G	0.75	0.8571	A
9	TIGRP2P131512_rs9197226	16111015	A	0.5	0.5	G
9	BICF2S23269	16119916	A	0.75	0.5714	C
9	BICF2S232192	16123765	A	0.25	0.3571	G
9	TIGRP2P131505_rs9146176	16137266	A	0	0.2857	G
9	BICF2P258474	16137635	0	0	0	G
9	BICF2P960452	16156464	A	0.5	0.5	G
9	BICF2S23331468	16157511	A	0.5	0.5	G
9	BICF2S23744440	16171624	A	0	0	G
9	BICF2P423066	16184548	A	0.5	0.2143	T
9	BICF2P998133	16201592	A	0	0	G

9	BICF2G630827704	16206447	0	0	0	A
9	BICF2P268072	16217336	A	0	0	G
9	BICF2S23211965	16230141	C	0.25	0.3571	A
9	BICF2S2456014	16246753	A	1	0.7	G
9	BICF2P747742	16252859	A	0	0	G
9	BICF2P724540	16267205	A	0.5	0.2143	G
9	BICF2P183501	16273030	G	0.25	0.4286	A
9	BICF2P1017635	16292391	A	0	0	G
9	TIGRP2P131475_rs8615003	16305613	0	0	0	C
9	BICF2P307827	16309893	A	0	0	C
9	BICF2S23336208	16346289	A	0.25	0.4286	G
9	BICF2P861343	16355654	0	0	0	G
9	BICF2P902288	16388439	0	0	0	C
9	BICF2S23258989	16412032	C	0.25	0.3571	A
9	BICF2P814755	16424075	0	0	0	C
9	BICF2P340566	16434252	A	0	0	G
9	BICF2S23610349	16459735	A	0	0	G
9	BICF2P778497	16497952	T	0	0	A
9	BICF2P284902	16507964	T	0.25	0.3571	A
9	BICF2P852282	16522183	A	0.25	0.3571	T
9	BICF2S230712	16525738	A	0	0	C
9	TIGRP2P131450_rs8824940	16547131	G	0.25	0.3571	A
9	BICF2P919957	16553433	G	0.75	0.3571	A
9	BICF2P1190026	16565845	G	0	0.2857	A
9	BICF2P1318954	16590986	G	0.75	0.8571	A
9	BICF2P577039	16603817	G	1	0.7143	A
9	BICF2P220675	16610619	G	1	0.7143	A
9	TIGRP2P131417_rs9250623	16622608	A	0.5	0.5	C
9	BICF2S23214271	16631507	G	0.25	0.6429	A
9	BICF2P1328224	16641333	G	0.75	0.8571	A
9	BICF2P63521	16647149	A	0.25	0.4286	G
9	BICF2P63519	16648647	A	0	0	G
9	BICF2P722870	16657026	G	0.25	0.6429	A
9	BICF2P978505	16666114	A	0.5	0.2143	G
9	TIGRP2P131391_rs8850423	16678142	A	0.5	0.2143	G
9	BICF2P149230	16694305	G	0.5	0.2143	A
9	BICF2P275863	16709435	0	0	0	G
9	BICF2G630827703	16711010	0	0	0	A
9	BICF2S22920941	16734237	G	0.5	0.5	A
9	BICF2P378226	16745160	G	0.25	0.3571	A
9	BICF2P871320	16756351	0	0	0	A
9	BICF2P612600	16773449	G	0.5	0.5	A
9	TIGRP2P131379_rs8990402	16782158	0	0	0	A
9	BICF2S22918640	16792735	G	0.25	0.3571	A
9	BICF2P1362114	16801950	A	0	0.2857	G
9	BICF2P567810	16816940	G	0	0.2857	A
9	BICF2P954258	16826540	A	0	0	G

9	BICF2P410430	16838018	A	0	0.2857	G
9	BICF2P1100728	16852367	A	0.25	0.1429	G
9	BICF2S23023176	16860671	G	0.75	0.3571	A
9	BICF2P229820	16875778	C	0.75	0.5714	A
9	BICF2P819347	16892000	A	0.25	0.1429	G
9	BICF2P1003965	16914513	0	0	0	G
9	TIGRP2P131372_rs9179124	16918718	A	0.25	0.1429	G
9	TIGRP2P131371_rs8611131	16950565	0	0	0	G
9	BICF2G630827702	16968282	0	0	0	A
9	BICF2P1348014	16990729	0	0	0	G
9	BICF2P1283898	17006345	A	0	0	G
9	BICF2P476013	17015576	0	0	0	A
9	BICF2S23148912	17024224	A	0	0	G
9	BICF2P1358518	17038287	A	0	0	G
9	BICF2S24116872	17045666	0	0	0	A
9	G544f58S24	17061852	0	0	0	C
9	TIGRP2P131370_rs8933439	17070882	A	0	0	G
9	BICF2S23136861	17110487	G	0	0.2857	A
9	BICF2P309026	17120698	0	0	0	G
9	TIGRP2P131368_rs8922309	17133915	0	0	0	G
9	BICF2P83519	17148290	A	0	0	G
9	BICF2S23121450	17171595	G	0	0	A
9	BICF2S232490	17203682	A	0.75	0.8571	G
9	BICF2P54232	17207137	G	0.5	0.5	A
9	BICF2P694696	17220618	G	1	1	A
9	BICF2P1398687	17232297	G	0	0	A
9	BICF2S23510298	17248229	A	0.5	0.5	G
9	BICF2P1392188	17276442	A	0	0	C
9	BICF2P997381	17291977	0	0	0	C
9	BICF2P531568	17312815	0	0	0	A
9	TIGRP2P131348_rs8707552	17341543	T	0.75	0.8571	A
9	BICF2P337737	17351069	C	0.5	0.5	G
9	BICF2P1437443	17357886	G	0.75	0.8571	A
9	BICF2P772423	17365800	T	0.75	0.8571	A
9	BICF2P817518	17380923	0	0	0	G
9	BICF2P597343	17388815	A	0.75	0.8571	G
9	BICF2P1113569	17418042	A	0.5	0.5	G
9	BICF2P360409	17432327	A	0.5	0.5	G
9	BICF2P868222	17436389	A	0.75	0.8571	G
9	BICF2P302858	17453208	G	1	1	A
9	BICF2S23641698	17459302	G	0	0.2857	A
9	BICF2S23526433	17475371	C	0.25	0.3571	G
9	G545f29S175	17496942	G	0.25	0.3571	A
9	BICF2S2453276	17510460	0	0	0	C
9	BICF2S23453357	17516336	0	0	0	A
9	BICF2S24110328	17544044	0	0	0	G
9	BICF2P257933	17565412	A	0	0	G

9	BICF2P1470913	17581446	G	0	0	A
9	BICF2P877906	17594202	C	0.75	0.3571	G
9	BICF2P149861	17607535	0	0	0	A
9	BICF2P495004	17619943	A	0	0	G
9	BICF2P1421034	17644284	G	0	0	A
9	BICF2P156242	17668971	C	0	0	A
9	BICF2S2328455	17674514	A	0.25	0.3571	G
9	BICF2P1360458	17676121	A	0.25	0.1429	G
9	BICF2S2329190	17677819	G	0.25	0.3571	A
9	BICF2P35065	17697331	A	0	0	G
9	BICF2S23214249	17699943	A	0.25	0.3571	G
9	BICF2P169593	17715282	A	0.25	0.1429	G
9	BICF2P684563	17727859	C	0	0	A
9	BICF2P202298	17734002	G	0.5	0.5	A
9	TIGRP2P131320_rs8716132	17752619	A	0	0	G
9	TIGRP2P131317_rs9114296	17758090	G	0.25	0.1429	A
9	BICF2P1061810	17774276	A	0.25	0.1429	G
9	TIGRP2P123210_rs8754621	17781657	G	0	0	A
9	BICF2P33519	17796933	G	0.75	0.8571	A
9	BICF2S2337469	17807858	G	0.25	0.1429	A
9	BICF2S23534706	17809271	G	0	0	A
9	BICF2S23522949	17814283	A	0.25	0.3571	G
9	BICF2S23517692	17833175	A	0.25	0.1429	G
9	TIGRP2P123183_rs9187437	17841242	G	0.5	0.5	A
9	BICF2P823592	17853716	A	0.25	0.1429	G
9	BICF2P103127	17861175	0	0	0	C
9	BICF2S23211226	17878285	G	0.25	0.3571	C
9	BICF2P644710	17890277	A	1	0.7143	G
9	BICF2P242077	17899831	0	0	0	G
9	BICF2S23229872	17907286	C	0.25	0.3571	A
9	BICF2P127526	17919104	0	0	0	A
9	BICF2P488623	17935326	0	0	0	C
9	TIGRP2P123165_rs9098280	17949608	A	0	0.2857	G
9	BICF2S2334042	17952343	A	0.5	0.2143	G
9	BICF2P515044	17962389	G	0.25	0.6429	A
9	BICF2S229772	17983069	C	0	0	A
9	TIGRP2P123153_rs9076656	17992766	A	1	1	C
9	BICF2P592894	18004722	G	0.75	0.3571	A
9	TIGRP2P123149_rs8883967	18009623	G	0.25	0.3571	A
9	BICF2P411608	18022770	G	0.25	0.6429	A
9	BICF2P871200	18023940	G	0.25	0.6429	A
9	BICF2P353627	18039088	G	0	0	A
9	BICF2S2357637	18047392	0	0	0	G
9	BICF2P430214	18055714	C	0.25	0.6429	A
9	BICF2S23126382	18099455	0	0	0	A
9	BICF2P98561	18102956	0	0	0	A
9	TIGRP2P123139_rs8986901	18113900	0	0	0	G

9	BICF2P196780	18127435	A	0	0	G
9	BICF2P897099	18136101	G	0	0.2857	A
9	BICF2P1436402	18149434	A	1	0.7143	G
9	BICF2S23416089	18159499	A	0	0	C
9	TIGRP2P123125_rs9074353	18177984	A	0.5	0.5	G
9	TIGRP2P123122_rs8736681	18181850	A	0.5	0.2143	G
9	BICF2P978951	18204550	A	0	0.2857	G
9	BICF2S22937536	18224375	G	0.25	0.3571	A
9	BICF2P322357	18228698	G	0.75	0.5714	A
9	BICF2P881262	18241965	A	0.5	0.7857	G
9	BICF2P83907	18251166	G	0	0.2857	A
9	BICF2S22955731	18253931	A	0.25	0.4286	C
9	BICF2P931374	18268300	G	0.5	0.7857	A
9	BICF2P385032	18278131	0	0	0	A
9	TIGRP2P123096_rs8578541	18293242	A	0.25	0.6429	G
9	BICF2P1371428	18307306	A	0	0.2857	G
9	BICF2P206197	18311761	A	0.75	0.8571	G
9	TIGRP2P123085_rs8592010	18329901	A	0.75	0.8571	T
9	TIGRP2P123081_rs8695428	18342154	G	0.75	0.8571	A
9	BICF2S23748522	18343028	G	0.75	0.8571	A
9	BICF2S23220840	18358373	G	0.75	0.8571	A
9	BICF2P421279	18365893	C	0.5	0.2143	A
9	BICF2S2341377	18377572	C	0.25	0.4286	A
9	BICF2P415777	18399883	G	0	0	A
9	BICF2S2344326	18400861	G	0.25	0.6429	A
9	BICF2P875387	18418646	A	0	0	G
9	BICF2P318017	18423741	G	0	0	A
9	BICF2S23125367	18442784	T	0	0.2857	A
9	TIGRP2P123054_rs8988636	18457045	A	0.5	0.2143	T
9	BICF2S23231596	18463437	G	1	0.7143	A
9	BICF2P214459	18473828	A	0	0	G
9	BICF2S2362140	18491230	C	0.5	0.5	A
9	BICF2S22927103	18493350	A	0.25	0.1429	G
9	BICF2P1262649	18505600	G	0	0	A
9	BICF2P936307	18520414	A	0.75	0.6429	G
9	BICF2P1160954	18537450	G	0	0	A
9	BICF2P600921	18540667	0	0	0	C
9	BICF2P1378969	18552040	G	0	0	C
9	BICF2P1292091	18553621	A	0	0	G
9	BICF2P597796	18564711	C	0	0	A
9	BICF2P280208	18579939	0	0	0	A
9	BICF2P393506	18593667	G	0.75	0.5714	A
9	BICF2P1288768	18597315	G	0.5	0.2143	A
9	TIGRP2P123011_rs8627041	18607426	A	0.25	0.1429	G
9	BICF2S22959351	18622932	A	1	1	C
9	BICF2P946866	18650779	A	0	0	G
9	BICF2P939366	18659769	A	0.25	0.3571	G

9	BICF2P504361	18668090	0	0	0	G
9	BICF2S23435718	18684395	0	0	0	G
9	BICF2P290179	18687570	G	0	0.3333	A
9	BICF2P1437270	18702118	A	0	0.2857	G
9	TIGRP2P122994_rs9180347	18721267	A	0	0.2857	C
9	BICF2P863881	18727519	A	0.75	0.6429	G
9	BICF2S23028523	18734515	A	0.25	0.6429	G
9	BICF2P837568	18749364	G	0	0.2857	A
9	BICF2P158986	18764948	G	0.25	0.4286	A
9	BICF2S2324010	18780707	A	0.25	0.3571	G
9	BICF2P335546	18791284	G	0.25	0.4286	A
9	BICF2P1426353	18805137	A	0.25	0.4286	G
9	BICF2P988600	18825099	G	0	0	A
9	BICF2P1071056	18839603	C	0	0.2857	A
9	BICF2P1039289	18848939	G	0.25	0.6429	A
9	BICF2P1054525	18882376	A	0	0	G
9	TIGRP2P122931_rs8615391	18887205	A	0	0.2857	G
9	BICF2P1137230	18896059	A	0.25	0.6429	G
9	BICF2P718275	18911590	A	0.5	0.5	G
9	BICF2P393326	18926539	C	0.5	0.7857	G
9	BICF2P410707	18929341	C	0	0	A
9	BICF2S2317717	18963499	A	0	0	G
9	BICF2S23223892	18996332	G	0.25	0.3571	A
9	BICF2P328758	18998670	G	0.25	0.3571	A
9	BICF2P158760	19014494	G	0.5	0.2143	A
9	TIGRP2P122906_rs8977328	19028665	A	0.25	0.4286	G
9	BICF2S23055739	19037490	T	1	0.7143	A
9	BICF2P385702	19046619	0	0	0	A
9	BICF2P1161165	19057590	C	0	0.2857	A
9	BICF2S23438262	19070468	C	0	0.2857	A
9	BICF2S23516922	19084362	C	0.5	0.5	A
9	BICF2S2438924	19096254	G	0	0	A
9	BICF2P39549	19112999	A	0	0	C
9	BICF2S2294380	19121052	G	0.5	0.5	A
9	BICF2S2439997	19128539	G	0.25	0.3571	A
9	BICF2P1251030	19129572	G	0.25	0.1429	A
9	BICF2P192682	19140965	G	0.25	0.1429	A
9	BICF2P1227511	19152537	A	0	0	C
9	BICF2P452889	19160959	G	0.25	0.3571	A
9	BICF2S23213488	19175891	A	0.25	0.6429	C
9	BICF2P364366	19183929	G	0	0.2857	A
9	BICF2P47724	19194063	A	1	0.7143	G
9	TIGRP2P122882_rs8697130	19211537	0	0	0	A
9	TIGRP2P122880_rs8730353	19227765	G	0.5	0.5	A
9	BICF2P1103697	19228271	A	0	0	G
9	BICF2P437369	19245568	0	0	0	A
9	BICF2S23147133	19262018	G	0.75	0.3571	A

9	BICF2P277664	19266785	A	1	0.7143	G
9	BICF2P438701	19281859	G	1	0.7143	A
9	BICF2P589810	19289821	A	1	0.75	G
9	BICF2P394578	19312506	A	0	0.25	G
9	BICF2S22940511	19325595	A	0.25	0.3571	C
9	TIGRP2P122861_rs9011246	19338752	A	0.25	0.3571	G
9	BICF2P1079087	19349148	A	0	0	G
9	BICF2S23422364	19359857	T	0	0	A
9	BICF2P464960	19367120	G	0.25	0.6429	A
9	BICF2P579696	19381199	A	0	0	G
9	BICF2P497312	19396139	A	0.25	0.6429	C
9	BICF2P1230700	19418665	A	0.75	0.3571	G
9	BICF2S24322284	19440998	0	0	0	G
9	BICF2P1367090	19448789	A	0	0	G
9	BICF2P1330724	19459162	G	0	0	A
9	BICF2P429213	19487183	G	0.25	0.3571	A
9	TIGRP2P122831_rs8743220	19498086	A	1	0.7143	C
9	BICF2P743446	19517044	A	0	0	G
9	BICF2P664085	19527900	A	0	0	G
9	BICF2S2312267	19545416	G	0	0	C
9	BICF2P674761	19559136	A	0.25	0.1429	G
9	TIGRP2P122814_rs8693201	19563146	0	0	0	G
9	BICF2P439062	19573125	G	0.25	0.3571	A
9	TIGRP2P122813_rs9123115	19585236	A	0.5	0.5	G
9	BICF2P422703	19601839	G	0.25	0.1429	A
9	BICF2P1125643	19623231	A	0.5	0.5	G
9	BICF2P177530	19633156	0	0	0	C
9	TIGRP2P122801_rs9003400	19650590	0	0	0	G
9	BICF2P528580	19662960	0	0	0	A
9	BICF2P518001	19665397	A	0	0	C
9	BICF2S23210280	19676872	A	0	0	G
9	BICF2P1328373	19688653	A	0	0	G
9	BICF2P684875	19704726	0	0	0	C
9	BICF2S23248752	19713259	G	0.25	0.3571	A
9	TIGRP2P122786_rs8677070	19731376	0	0	0	G
9	TIGRP2P122785_rs9047176	19742372	A	0	0	G
9	BICF2P384094	19749596	0	0	0	C
9	BICF2P289055	19762988	A	0	0	C
9	BICF2S2399608	19817149	A	0	0	C
9	BICF2G630827582	19826590	G	0.5	0.7857	A
9	BICF2G630827553	19838442	A	0.5	0.5714	G
9	BICF2G630827487	19859884	A	0.5	0.5714	C
9	BICF2G630827481	19862889	G	0.5	0.2143	A
9	BICF2G630827451	19874171	A	0	0	C
9	TIGRP2P242421_rs8674609	20119692	A	0	0	C
9	BICF2P1437184	20131445	C	0.5	0.4167	G
9	BICF2S22928304	20137705	G	0	0	A

9	TIGRP2P242449_rs8698184	20150433	A	0.25	0.3333	C
9	BICF2G630826890	20160947	A	0.25	0.3571	G
9	BICF2G630826873	20174140	G	0.25	0.5	A
9	BICF2G630826757	20206226	A	0.5	0.4286	G
9	BICF2S23348997	20228913	A	0.5	0.3571	C
9	BICF2G630826601	20253550	A	0.5	0.4286	G
9	BICF2G630826538	20274406	G	0	0	A
9	BICF2P1242571	20287660	A	0	0	G
9	TIGRP2P242544_rs9060129	20299052	A	0.5	0.3571	C
9	BICF2P1381490	20312347	A	0	0	G
9	BICF2S2356115	20324959	G	0.5	0.4167	A
9	TIGRP2P242595_rs9051859	20356754	0	0	0	A
9	BICF2G630826474	20366553	A	0.5	0.4286	G
9	BICF2G630826442	20380295	C	0.5	0.5	A
9	TIGRP2P242635_rs8589686	20412531	A	0	0.2857	G
9	BICF2P1111611	20430110	G	0	0	A
9	BICF2G630826358	20441112	A	0	0	G
9	BICF2G630826342	20447278	G	0.5	0.2143	A
9	BICF2G630826273	20470039	G	0.25	0.1429	A
9	TIGRP2P242653_rs8823067	20484009	C	0	0	A
9	BICF2G630826217	20493330	A	0.75	0.8571	G
9	BICF2G630826205	20505413	G	0.5	0.2143	A
9	BICF2G630826164	20521826	G	0.25	0.6429	A
9	BICF2P51045	20528057	C	0.5	0.2143	A
9	BICF2G630826123	20541124	G	0	0.2857	A
9	BICF2G630826093	20553703	A	0	0	G
9	TIGRP2P242690_rs8796343	20565461	G	0	0.2857	A
9	BICF2G630826078	20574759	A	0.25	0.1429	G
9	BICF2G630826047	20602144	A	0.25	0.1429	G
9	BICF2G630826017	20610786	A	0.75	0.8571	C
9	BICF2G630825929	20644585	G	0.25	0.3571	A
9	BICF2P1359973	20656485	A	0.25	0.1429	G
9	TIGRP2P242759_rs8583903	20666029	C	0.5	0.2143	A
9	BICF2P983534	20677357	G	0.75	0.6429	A
9	BICF2G630825838	20690073	G	0	0	A
9	TIGRP2P242797_rs9239106	20700190	G	0	0.2857	A
9	BICF2G630825760	20714828	G	0.5	0.5	A
9	BICF2G630825718	20723832	A	0	0	G
9	TIGRP2P242819_rs8962883	20735845	G	0.5	0.2143	A
9	BICF2G630825632	20747803	G	0	0.3571	A
9	TIGRP2P242837_rs8524452	20769796	0	0	0	G
9	BICF2G630825531	20787714	G	0	0	A
9	BICF2G630825518	20793073	A	0.5	0.2143	G
9	BICF2S2444016	20807279	G	0	0	A
9	BICF2G630825471	20819536	A	0	0	G
9	BICF2G630825448	20826863	A	0	0	G
9	BICF2S22944095	20850819	A	0.5	0.5	G

9	BICF2G630825326	20875345	G	0.5	0.5	A
9	BICF2P1396747	20884865	G	0.25	0.6429	A
9	BICF2G630825278	20896695	G	0	0	A
9	BICF2G630825255	20912570	G	0	0	A
9	BICF2G630825243	20922135	A	0.25	0.1429	C
9	BICF2G630825050	21045322	G	0	0	A
9	BICF2S22923116	21091441	A	0	0	G
9	BICF2G630824932	21137140	A	0.25	0.2857	G
9	BICF2P269342	21525613	A	0.25	0.1429	C
9	BICF2G630828735	21539215	A	0.25	0.3571	C
9	BICF2G630828744	21543628	A	0.25	0.3571	G
9	BICF2G630828753	21573958	A	0.5	0.2143	G
9	BICF2P1355037	21578312	0	0	0	A
9	BICF2P476836	21647281	0	0	0	G
9	TIGRP2P123761_rs8879961	21661777	0	0	0	T
9	BICF2P618079	21696907	G	0.25	0.1429	A
9	BICF2P1414933	21701189	A	0	0	G
9	TIGRP2P123765_rs9028924	21718690	A	0	0	G
9	BICF2S23552249	21731401	0	0	0	G
9	BICF2P1357714	21737302	A	0.25	0.1429	C
9	BICF2P56992	21750414	A	0.25	0.1429	G
9	BICF2P1468538	21759557	G	0.5	0.5	A
9	BICF2P609918	21793981	A	0.5	0.5	C
9	TIGRP2P123790_rs9215392	21809381	G	0.25	0.1429	A
9	BICF2S23143878	21819076	G	0.25	0.3571	A
9	BICF2P963495	21839275	A	0	0	G
9	BICF2P940856	21852767	G	0.25	0.3571	A
9	BICF2P1304251	21870640	G	0	0	A
9	BICF2P577436	21878369	A	0.25	0.1429	G
9	BICF2P18565	21885345	A	0	0	G
9	BICF2P1105875	21896243	A	0	0	G
9	BICF2P1424692	21917749	A	0.5	0.5	G
9	BICF2P659282	21919855	A	0.5	0.5	G
9	TIGRP2P123815_rs8599056	21933263	A	0.5	0.5	G
9	TIGRP2P123820_rs8992933	21939723	G	0.5	0.5	A
9	BICF2P791911	21946688	G	0.5	0.5	A
9	BICF2P791912	21946767	A	0.5	0.5	C
9	TIGRP2P123841_rs8731442	21950647	G	0.5	0.5	A
9	BICF2P740053	21961172	0	0	0	A
9	BICF2P820321	22000980	0	0	0	A
9	BICF2P186635	22014238	0	0	0	A
9	BICF2P1314088	22037692	C	0.25	0.3571	G
9	TIGRP2P123851_rs8621678	22042123	G	0	0	A
9	TIGRP2P123852_rs8810572	22050265	0	0	0	C
9	BICF2P1297471	22089287	0	0	0	A
9	BICF2P3451	22100598	G	0.25	0.1429	A
9	BICF2S23633704	22105423	C	0.25	0.1429	A

9	BICF2P85786	22131423	A	0	0	G
9	BICF2P441438	22147511	0	0	0	G
9	BICF2P1456283	22161139	0	0	0	A
9	BICF2P497909	22194543	0	0	0	A
9	BICF2P391589	22196559	G	0	0	A
9	BICF2G630828780	22222259	A	0	0	G
9	BICF2P582791	22233163	A	0	0	G
9	BICF2P992151	22246826	G	0	0	A
9	TIGRP2P123872_rs8637978	22257706	G	0	0	A
9	BICF2P1131017	22277662	A	0	0	T
9	BICF2P558323	22287218	0	0	0	T
9	BICF2P1388616	22306903	0	0	0	G
9	TIGRP2P123879_rs9131016	22341741	A	0	0	C
9	BICF2P615176	22351530	0	0	0	A
9	TIGRP2P123881_rs8984665	22360957	G	0	0	C
9	BICF2P849769	22376001	A	0.75	0.5714	G
9	BICF2G630828809	22381705	A	0.25	0.1429	G
9	BICF2G630828813	22398816	A	0	0	G
9	BICF2G630828830	22412658	C	0.75	0.5714	A
9	BICF2S23441603	22420035	A	0	0	G
9	BICF2S23037587	22433027	0	0	0	G
9	BICF2G630828851	22448011	A	1	1	G
9	BICF2G630828858	22460534	A	0.75	0.8571	G
9	BICF2S23051664	22493625	A	0	0.08333	C
9	BICF2P941484	22504342	G	0.25	0.1429	A
9	BICF2S22958957	22508466	G	0	0	A
9	TIGRP2P123899_rs8774420	22518122	C	0.5	0.5	A
9	BICF2P867366	22535218	G	0	0	A
9	TIGRP2P123907_rs8848012	22557059	0	0	0	C
9	TIGRP2P123909_rs9132640	22565788	0	0	0	C
9	TIGRP2P123911_rs8810000	22581460	C	0	0	G
9	BICF2P492809	22586483	A	0.5	0.2143	G
9	BICF2P723418	22601503	A	0.5	0.2143	G
9	BICF2S23158543	22617041	A	0.75	0.3571	G
9	BICF2G630828863	22630713	G	0.75	0.3571	A
9	BICF2G630828866	22640123	G	0.75	0.3571	A
9	BICF2P292223	22662515	A	0	0	G
9	BICF2P714444	22670299	A	0	0	G
9	BICF2G630828883	22685746	A	0.25	0.6429	G
9	BICF2G630828895	22699962	G	0.5	0.2143	A
9	BICF2S24416201	22701428	G	0.5	0.2143	A
9	BICF2P363133	22706912	C	0.25	0.3571	A
9	BICF2P380222	22712583	A	0	0	C
9	BICF2P8441	22724095	A	0.25	0.3333	G
9	BICF2G630828921	22727390	G	0	0	A
9	BICF2P329696	22741546	A	0.5	0.5	G
9	BICF2G630828937	22767770	C	0.5	0.1667	A

9	BICF2G630828944	22777441	G	0.5	0.2143	A
9	BICF2G630828948	22781124	G	0	0	A
9	BICF2G630828965	22785958	G	NA	0.2143	A
9	BICF2G630828984	22797690	A	0	0	C
9	BICF2P821700	22810572	A	1	1	G
9	BICF2G630829005	22818895	C	0	0	A
9	BICF2P1312098	22832737	G	0.5	0.7857	A
9	BICF2G630829021	22850256	G	0.25	0.3571	A
9	BICF2G630829029	22857020	A	0.25	0.3571	G
9	BICF2G630829041	22873028	A	0	0	G
9	BICF2G630829045	22884979	C	0.5	0.2143	A
9	BICF2P1223020	22885889	A	0.5	0.2143	G
9	BICF2G630829068	22912096	A	0.25	0.4286	G
9	BICF2S23029957	22921303	A	0	0.2857	C
9	TIGRP2P123959_rs8806695	22941383	0	0	0	G
9	BICF2G630829086	22942557	G	0.75	0.3571	A
9	BICF2G630829096	22959422	A	0	0	T
9	BICF2G630829101	22963830	A	0	0	G
9	BICF2G630829112	22980345	A	0	0	G
9	BICF2P642964	22995539	A	0	0	G
9	BICF2G630829127	23001093	G	0	0	A
9	BICF2S23451292	23012870	A	0.25	0.7	C
9	BICF2G630829144	23045640	G	0	0	A
9	BICF2P1025331	23070810	0	0	0	G
9	BICF2S23444144	23086835	G	1	0.7143	A
9	BICF2G630829196	23094892	G	1	0.3571	A
9	BICF2G630829204	23113811	G	0.25	0.3571	A
9	BICF2G630829205	23116608	A	0.75	0.3571	G
9	BICF2G630829215	23129335	A	0.75	0.3571	G
9	BICF2G630829234	23138891	C	0.25	0.3571	A
9	BICF2G630829262	23151538	G	0.25	0.3571	A
9	BICF2G630829277	23162898	A	0.75	0.3571	G
9	BICF2G630829300	23173104	A	0.75	0.6429	G
9	BICF2G630829395	23228818	C	0.75	0.3571	A
9	BICF2G630829405	23234904	G	0.75	0.6429	A
9	TIGRP2P124009_rs8740102	23242605	0	0	0	G
9	BICF2G630829424	23254532	G	0.75	0.3571	A
9	BICF2G630829443	23276058	A	0.75	0.6429	G
9	BICF2P325128	23297061	0	0	0	T
9	BICF2G630829453	23306247	A	0.25	0.3571	G
9	BICF2G630829454	23311685	C	0.75	0.3571	G
9	TIGRP2P124011_rs8818058	23328710	0	0	0	C
9	BICF2S23528120	23345924	A	0	0	G
9	G550f59S47	23370058	0	0	0	C
9	BICF2G630829457	23399439	A	0.75	0.3571	G
9	BICF2P133297	23429501	0	0	0	A
9	BICF2P389760	23452689	0	0	0	A

9	BICF2P1019239	23471244	0	0	0	A
9	BICF2P173999	23499012	0	0	0	G
9	BICF2G630829465	23528122	G	0	0	C
9	BICF2G630829467	23534385	G	0	0	C
9	BICF2S22948024	23568615	A	0	0	G
9	BICF2P212537	23589529	C	0.25	0.1429	A
9	BICF2S23727301	23604135	T	0	0	A
9	BICF2P77510	23620883	A	0.75	0.3571	G
9	BICF2S23254549	23622692	A	0	0	G
9	BICF2P1093967	23662144	G	0.75	0.3571	A
9	BICF2S23658597	23689031	A	0	0	G
9	BICF2P303481	23703650	A	0.25	0.3571	G
9	BICF2S23516143	23723202	G	0	0	A
9	BICF2P453612	23734065	G	0	0	A
9	TIGRP2P124013_rs9085715	23746629	0	0	0	G
9	BICF2P697479	23753178	0	0	0	A
9	BICF2P497189	23763978	0	0	0	A
9	BICF2P1252024	23804368	0	0	0	G
9	BICF2S24118055	23805503	G	0	0.2857	A
9	BICF2S23152399	23823551	A	0.25	0.3333	G
9	BICF2G630829471	23859332	G	0.5	0.5	A
9	BICF2G630829486	23888623	A	0.5	0.5	G
9	BICF2G630829491	23898065	G	0.5	0.5	A
9	BICF2G630829506	23917868	G	0.5	0.5	C
9	BICF2G630829511	23926104	G	0.5	0.5	A
9	BICF2G630829536	23945615	A	0.25	0.1429	C
9	BICF2G630829543	23957304	G	0	0	A
9	BICF2G630829545	23959081	T	0	0	A
9	BICF2G630829548	23972204	G	0.25	0.1429	A
9	BICF2G630829567	23988807	G	0.25	0.1429	A
9	BICF2G630829571	23994085	A	0.25	0.1429	C
9	BICF2P879969	24004132	G	0.25	0.1429	A
9	BICF2P1060501	24043213	0	0	0	A
9	BICF2G630829633	24075552	A	0	0.1429	G
9	BICF2P351904	24084144	G	0.25	0.1429	A
9	BICF2G630829650	24084709	A	0.25	0.08333	G
9	BICF2G630829655	24092520	A	0.25	0.1429	G
9	BICF2G630829666	24108127	G	0.25	0.1429	A
9	BICF2G630829677	24120331	A	0	0	G
9	BICF2P428480	24142916	A	1	1	G
9	BICF2P660167	24159667	G	0	0	A
9	BICF2P725553	24162720	G	0.5	0.5	A
9	BICF2G630829731	24182629	G	0	0.2857	A
9	BICF2P402231	24185190	A	0	0	G
9	BICF2G630829749	24196811	C	0	0	A
9	BICF2G630829767	24215414	A	0.25	0.4286	G
9	BICF2G630829769	24216290	G	0.25	0.4286	A

9	BICF2G630829785	24228637	G	0	0.2857	A
9	BICF2G630829791	24231063	A	0	0.2857	G
9	BICF2G630829816	24259926	A	0.5	0.7857	G
9	BICF2G630829823	24268076	G	0.25	0.6429	A
9	BICF2G630829847	24285712	G	0.5	0.7857	A
9	BICF2G630829855	24288729	A	0.5	0.7857	G
9	BICF2G630829860	24303534	A	0.25	0.6429	G
9	BICF2G630829871	24314372	A	0	0	G
9	BICF2P966048	24323235	A	0.75	0.3571	G
9	BICF2G630829890	24336528	A	0.75	0.8571	G
9	BICF2G630829924	24357721	A	0.5	0.7857	G
9	BICF2S23714360	24371654	A	0.25	0.1429	G
9	BICF2G630829929	24387035	G	0.25	0.1429	A
9	BICF2G630829931	24402772	G	0.75	0.8571	A
9	BICF2G630829939	24404993	C	0.75	0.8571	G
9	BICF2G630829972	24420447	C	0.75	0.8571	G
9	BICF2G630829989	24433084	G	0.25	0.1429	A
9	TIGRP2P124059_rs9165827	24442502	A	0	0	G
9	BICF2G630830026	24470605	G	0	0	C
9	BICF2S23610261	24483476	A	0	0	C
9	BICF2G630830079	24504811	A	0.25	0.6429	G
9	BICF2G630830086	24508076	A	0.25	0.6429	G
9	BICF2G630830097	24526676	G	0	0	A
9	BICF2G630830099	24551999	A	0.5	0.8	G
9	BICF2P1289819	24555446	A	0	0	C
9	BICF2G630830103	24565397	G	0.5	0.2143	A
9	BICF2G630830107	24580506	A	0.5	0.2143	G
9	BICF2G630830121	24588355	G	0.5	0.2143	A
9	BICF2G630830173	24617133	A	0.5	0.2143	G
9	BICF2S23646886	24624254	G	0.5	0.2143	A
9	BICF2G630830191	24634142	G	0	0	A
9	BICF2G630830198	24644890	G	0	0	A
9	BICF2G630830207	24685027	0	0	0	A
9	BICF2G630830212	24710869	0	0	0	G
9	BICF2G630830213	24720152	G	0.5	0.7857	C
9	BICF2G630830216	24734483	C	0	0	A
9	BICF2P963953	24740805	A	0.5	0.2143	G
9	BICF2P558193	24792107	0	0	0	A
9	BICF2S23024789	24807179	C	0	0	A
9	BICF2G630830278	24875437	G	0	0	A
9	BICF2G630830300	24904622	G	0	0	A
9	BICF2G630830308	24916897	A	0	0	G
9	BICF2G630830309	24922431	A	0	0	C
9	BICF2G630830340	24953383	T	0	0	A
9	BICF2G630830352	24958282	G	0	0	A
9	BICF2P579865	24967625	0	0	0	A
9	BICF2G630830371	24980440	A	0	0	G

9	BICF2S23628342	25018265	A	0	0	C
9	BICF2G630830408	25024922	A	0	0	G
9	BICF2G630830420	25040286	A	0	0	G
9	BICF2G630830438	25060516	A	1	1	G
9	BICF2G630830449	25071652	A	1	1	G
9	BICF2G630830460	25088625	C	1	1	A
9	BICF2G630830471	25096586	G	1	1	A
9	TIGRP2P124142_rs8626943	25108271	0	0	0	A
9	BICF2G630830491	25121473	A	0	0	G
9	BICF2G630830504	25131473	G	1	1	A
9	BICF2G630830523	25141939	A	1	1	G
9	BICF2G630830534	25150886	A	1	1	G
9	BICF2G630830550	25164722	A	1	1	G
9	BICF2G630830554	25178793	G	1	1	A
9	BICF2P528965	25189885	0	0	0	G
9	TIGRP2P124155_rs9031693	25202544	0	0	0	C
9	TIGRP2P124156_rs8632740	25208636	0	0	0	C
9	BICF2G630830605	25230086	A	0	0	G
9	BICF2G630830616	25251644	A	1	1	C
9	BICF2G630830621	25256521	A	1	1	G
9	BICF2G630830625	25268414	G	0	0	A
9	BICF2G630830641	25290771	C	0	0.2857	A
9	BICF2P264572	25312928	A	0	0	C
9	TIGRP2P124173_rs9164818	25327939	G	0	0	A
9	BICF2G630830696	25335873	G	0	0.2857	A
9	BICF2G630830721	25355058	G	0	0	A
9	BICF2G630830724	25357630	A	0	0	G
9	BICF2G630830740	25369911	A	0	0	G
9	BICF2G630830755	25376656	A	1	0.75	G
9	BICF2G630830760	25384741	A	0	0.2857	G
9	BICF2G630830771	25409162	A	1	0.7143	C
9	BICF2G630830779	25415115	G	1	0.7143	A
9	TIGRP2P124180_rs8909833	25426576	A	0	0	G
9	BICF2G630830815	25440794	G	0.5	0.5	A
9	BICF2G630830832	25455859	A	0	0	G
9	BICF2G630830847	25459799	A	0.5	0.5	G
9	BICF2P699631	25465743	A	1	0.7143	G
9	BICF2G630830858	25490319	A	0.5	0.5	G
9	BICF2G630830862	25497972	G	0.5	0.5	A
9	BICF2G630830864	25507570	G	0.5	0.5	A
9	BICF2G630830882	25518774	G	0.5	0.5	A
9	BICF2G630830893	25528854	A	0.5	0.1667	G
9	TIGRP2P124202_rs8835099	25542291	A	0	0	G
9	BICF2P1125027	25558276	G	0.5	0.5	A
9	TIGRP2P124216_rs8925245	25577947	C	0.5	0.2143	A
9	TIGRP2P124221_rs8939112	25590650	G	0	0	A
9	BICF2S23232639	25615599	A	0.5	0.5	G

9	BICF2G630830906	25624089	C	0	0.2857	A
9	BICF2G630830911	25634815	A	0	0.2857	G
9	BICF2S23652010	25650033	A	0	0	G
9	BICF2P627713	25665005	0	0	0	C
9	TIGRP2P124231_rs8854848	25699993	C	0.5	0.5	A
9	BICF2P506723	25710036	A	0.5	0.5	C
9	BICF2P4949	25744551	G	0	0	C
9	BICF2P455370	25757485	0	0	0	A
9	BICF2G630830925	25764104	0	0	0	G
9	BICF2S23016495	25816551	G	0.25	0.4286	A
9	BICF2S23237535	25823015	G	0.25	0.4286	C
9	BICF2G630830951	25851498	G	0.25	0.4286	A
9	BICF2G630830958	25855647	G	0.25	0.4167	A
9	BICF2G630830963	25864174	A	0.25	0.1429	G
9	BICF2P1251768	25879379	G	0.5	0.5	A
9	BICF2G630830984	25899629	A	0.5	0.7857	G
9	BICF2S22932191	25906138	G	0	0	A
9	BICF2P37637	25910552	G	1	0.7143	C
9	BICF2S2355667	25930358	G	0.5	0.8333	A
9	BICF2P1010945	25937903	G	0.25	0.3571	A
9	BICF2G630830989	25938768	A	0.75	0.3571	G
9	BICF2P864411	25953349	0	0	0	G
9	BICF2P580907	25990782	G	0.25	0.3571	A
9	BICF2P607508	26012915	G	0	0	A
9	BICF2P289987	26016325	A	0	0	G
9	BICF2P705707	26027962	A	0	0	G
9	BICF2S2306419	26040886	G	0.25	0.3333	A
9	BICF2P688692	26051453	T	0.25	0.3571	A
9	TIGRP2P124282_rs8640740	26072168	A	0.25	0.3571	G
9	BICF2G630831000	26093362	A	0.75	0.3571	G
9	BICF2P663036	26106235	0	0	0	C
9	BICF2G630831008	26109694	G	0	0.2857	A
9	BICF2P1205826	26114488	G	0.25	0.3571	A
9	BICF2G630831015	26121963	0	0	0	G
9	BICF2G630831019	26139355	A	1	1	G
9	TIGRP2P124286_rs9091783	26151145	G	0	0	A
9	BICF2G630831046	26181509	A	0	0	G
9	BICF2P746905	26188838	A	0.5	0.2143	G
9	BICF2G630831049	26199912	G	0.5	0.2143	A
9	BICF2G630831058	26209122	G	0.5	0.2143	A
9	BICF2S2315732	26227162	C	0	0.2857	A
9	BICF2P813098	26232743	G	0	0.2857	A
9	BICF2P794912	26244447	0	0	0	C
9	BICF2S22919096	26274409	A	0	0	G
9	BICF2S22946125	26322258	A	1	1	G
9	TIGRP2P124304_rs9042104	26329064	0	0	0	A
9	TIGRP2P124309_rs9015099	26340367	0	0	0	G

9	BICF2P384854	26357653	G	0	0.25	A
9	BICF2G630831107	26360304	G	0	0	A
9	BICF2P278442	26370694	0	0	0	G
9	BICF2P173659	26389328	0	0	0	G
9	BICF2P1412478	26398116	A	0	0.2857	G
9	BICF2G630831119	26405611	G	0	0	A
9	BICF2P1359837	26417028	A	0	0.2857	G
9	TIGRP2P124347_rs8886420	26434299	A	0	0.2857	G
9	BICF2G630831149	26445030	A	0	0	G
9	BICF2G630831152	26450306	G	0	0.2857	A
9	BICF2G630831164	26468685	G	0	0	A
9	BICF2G630831184	26484422	A	0	0	G
9	BICF2G630831187	26486576	C	0	0	A
9	BICF2P621233	26507994	0	0	0	C
9	BICF2P176617	26529113	A	1	0.7143	G
9	BICF2G630831204	26552446	A	0	0.2857	G
9	BICF2G630831212	26576269	A	0	0.2857	G
9	BICF2G630831224	26584969	A	0	0	G
9	BICF2G630831238	26593370	G	0	0	A
9	BICF2S23723946	26614016	A	0	0	G
9	BICF2G630831282	26630902	C	1	0.7143	A
9	BICF2G630831290	26638202	A	0	0.2857	G
9	BICF2P71713	26654410	0	0	0	C
9	BICF2G630831294	26661727	A	0	0	G
9	BICF2G630831297	26679172	A	1	1	G
9	BICF2G630831299	26680259	C	1	1	A
9	BICF2P1248103	26696255	A	0	0	G
9	BICF2P939832	26713984	C	0	0.2857	G
9	BICF2G630831332	26723332	G	0	0	A
9	BICF2G630831334	26735433	G	0	0	A
9	BICF2G630831351	26755116	A	0	0	G
9	BICF2G630831361	26764743	G	1	1	A
9	BICF2G630831376	26788271	A	0	0	G
9	BICF2G630831382	26796538	G	1	1	A
9	BICF2G630831384	26810040	A	0	0	G
9	BICF2G630831400	26824471	A	0	0	C
9	BICF2P390809	26846696	0	0	0	C
9	BICF2P139463	26854709	A	0	0	G
9	BICF2G630831433	26880751	G	0	0	A
9	BICF2P192056	26892870	G	0	0	A
9	BICF2G630831471	26916258	A	0	0	G
9	BICF2P301856	26922036	A	1	1	G
9	BICF2P1364626	26940018	0	0	0	G
9	BICF2G630831487	26955304	G	0	0	A
9	BICF2G630831488	26956032	G	0	0	A
9	BICF2G630831495	26973512	A	0	0	G
9	BICF2P226153	26985628	0	0	0	G

9	BICF2G630831525	26991295	0	0	0	G
9	BICF2P1304179	27003897	A	0	0	G
9	BICF2G630831532	27016091	A	0	0	G
9	TIGRP2P124405_rs9151782	27030574	0	0	0	G
9	BICF2G630831560	27037603	0	0	0	A
9	BICF2P989771	27058611	A	0.5	0.5	G
9	BICF2P94801	27066033	G	0.5	0.5	A
9	BICF2P155824	27077993	0	0	0	C
9	BICF2G630831588	27085898	C	0	0	A
9	BICF2G630831594	27116097	G	0	0	A
9	BICF2G630831601	27137673	0	0	0	G
9	BICF2S22935109	27144527	G	0.25	0.4286	A
9	TIGRP2P124409_rs8770636	27168219	A	0.25	0.4286	G
9	BICF2P1334700	27175995	A	0	0	G
9	BICF2G630831617	27189135	A	0.25	0.4286	G
9	BICF2G630831621	27204161	0	0	0	G
9	BICF2G630831662	27234905	0	0	0	G
9	BICF2G630831679	27262545	0	0	0	G
9	BICF2G630831687	27268119	0	0	0	A
9	BICF2G630831702	27282342	G	0.75	0.5714	A
9	BICF2P585321	27293217	A	0.5	0.2143	G
9	TIGRP2P124416_rs8499045	27302043	A	0	0	G
9	BICF2P688446	27319319	0	0	0	A
9	BICF2G630831733	27328631	0	0	0	A
9	BICF2P1104193	27345349	0	0	0	A
9	BICF2G630831737	27373898	0	0	0	C
9	BICF2G630831739	27384996	A	0.5	0.5	G
9	BICF2G630831746	27402312	C	0.25	0.4286	A
9	BICF2G630831765	27439060	G	0.75	0.5714	A
9	BICF2G630831784	27455660	0	0	0	G
9	BICF2P484513	27469213	A	0.25	0.3571	G
9	BICF2P379734	27478164	G	0.25	0.3571	A
9	TIGRP2P124429_rs9190346	27490260	A	0.75	0.3333	C
9	BICF2P442136	27505131	C	0.75	0.3571	A
9	BICF2S233344	27518829	G	1	1	A
9	BICF2S23134376	27539209	A	0.5	0.7857	G
9	BICF2S23153603	27543222	G	NA	0.9	A
9	BICF2P296384	27573262	G	0.75	0.3571	A
9	BICF2P595725	27587125	0	0	0	C
9	BICF2P594507	27589254	0	0	0	A
9	BICF2P910809	27610112	G	0.75	0.6429	A
9	BICF2G630831825	27615764	G	0.75	0.6429	A
9	BICF2G630831844	27638152	A	0	0	G
9	BICF2P211998	27646669	G	0	0	A
9	BICF2G630831874	27656305	0	0	0	G
9	BICF2G630831882	27658583	0	0	0	G
9	BICF2S22929584	27689381	G	0	0	A

9	BICF2S23624985	27719863	G	1	1	A
9	BICF2S23148301	27727467	A	0.5	0.7857	G
9	BICF2G630831883	27753191	0	0	0	A
9	BICF2G630831885	27795461	A	0	0	G
9	BICF2P827739	27804818	A	1	1	G
9	TIGRP2P124449_rs8851158	27810114	A	0	0	G
9	BICF2P1295818	27822410	A	0.5	0.7857	G
9	BICF2G630831901	27831229	A	0.5	0.1667	G
9	BICF2G630831906	27845204	G	0.5	0.2143	A
9	BICF2G630831913	27857103	A	0	0	G
9	BICF2P994950	27864687	A	0.25	0.6429	G
9	BICF2G630831941	27881079	G	1	0.3333	A
9	BICF2G630831947	27893772	A	0.75	0.8571	G
9	TIGRP2P124465_rs8597761	27914316	A	0	0	C
9	BICF2G630831972	27932294	A	0	0	G
9	BICF2G630831975	27933672	G	0	0	A
9	BICF2P1429507	27945226	A	0	0	G
9	BICF2G630832000	27961574	G	0.5	0.5	C
9	BICF2G630832006	27983423	A	0.5	0.5	G
9	BICF2G630832013	27992917	C	0.5	0.5	A
9	BICF2S2442961	28018440	C	0.25	0.1429	A
9	BICF2G630832039	28053901	A	0	0	C
9	TIGRP2P124467_rs8560403	28066291	0	0	0	G
9	BICF2G630832045	28087731	G	0	0.2857	A
9	BICF2P980814	28097585	A	0.25	0.1429	G
9	BICF2G630832062	28107912	A	0.75	0.8571	C
9	TIGRP2P124471_rs8850785	28119059	C	0.75	0.8571	A
9	BICF2P898428	28121824	A	0.75	0.8571	G
9	TIGRP2P124473_rs9181214	28137126	G	0.5	0.5	A
9	BICF2G630832091	28149593	A	0.25	0.3571	G
9	BICF2G630832097	28172314	C	0.25	0.6429	A
9	BICF2G630832098	28178094	G	0	0.2857	A
9	BICF2G630832108	28192680	G	1	0.7143	A
9	BICF2S2353168	28219010	C	0.25	0.3571	A
9	BICF2P444992	28221397	0	0	0	G
9	TIGRP2P124486_rs9015058	28262169	A	0	0	C
9	BICF2P38700	28280411	0	0	0	G
9	BICF2P633674	28297474	G	0.25	0.1429	A
9	BICF2S22911646	28321622	A	0	0	C
9	BICF2P1324571	28343272	A	0	0	G
9	BICF2P425977	28351844	A	0.25	0.1429	G
9	BICF2P504839	28360578	A	0.75	0.5714	G
9	TIGRP2P124509_rs8705511	28376243	A	0.5	0.7857	G
9	BICF2P1369895	28391933	A	0	0	G
9	BICF2P1383800	28420736	C	0.5	0.5	A
9	TIGRP2P124536_rs9109564	28434500	G	0.75	0.5714	A
9	BICF2S23049628	28456141	G	0	0	A

9	BICF2P511133	28471653	0	0	0	C
9	TIGRP2P124541_rs8751733	28481026	0	0	0	G
9	BICF2P1013176	28491504	A	0.5	0.2143	G
9	TIGRP2P124554_rs8620199	28500348	G	0.75	0.6429	A
9	TIGRP2P124561_rs9140180	28519050	A	0	0	C
9	BICF2P1378613	28520715	A	0.75	0.6429	G
9	BICF2P849393	28543466	A	0.5	0.2143	C
9	BICF2G630832137	28581567	G	0.5	0.5	A
9	BICF2P512664	28606626	0	0	0	A
9	BICF2S23327820	28630068	A	0	0.3571	G
9	BICF2G630832150	28654951	G	0.25	0.1429	A
9	BICF2S23537643	28664802	G	0.25	0.4286	A
9	BICF2P827949	28671115	C	0.25	0.1429	A
9	BICF2G630832189	28700337	G	0.75	0.5714	A
9	BICF2G630832193	28707223	A	0.25	0.3571	G
9	BICF2G630832196	28719420	A	0.5	0.5	G
9	BICF2G630832213	28736700	A	0.25	0.1429	G
9	BICF2G630832220	28749604	G	0.25	0.1429	A
9	BICF2G630832221	28757105	0	0	0	A
9	BICF2G630832223	28762604	G	0.25	0.1429	A
9	BICF2P512239	28792045	0	0	0	A
9	BICF2G630832234	28827137	0	0	0	A
9	BICF2P483186	28839797	A	0.5	0.2143	G
9	BICF2G630832245	28849502	A	0.5	0.2143	G
9	BICF2P622903	28871567	A	0.5	0.2143	G
9	BICF2G630832265	28876937	A	0	0	G
9	BICF2G630832267	28877334	G	0.5	0.5	A
9	BICF2G630832293	28893423	C	0.5	0.2143	A
9	BICF2S23728785	28904905	G	0	0	A
9	BICF2P1256607	28910609	C	0	0.2857	A
9	BICF2P1364986	28912025	A	0	0.2857	C
9	BICF2P715577	28923859	A	0.5	0.2143	G
9	BICF2G630832353	28938596	G	0.5	0.5	A
9	TIGRP2P124602_rs8873349	28954490	0	0	0	G
9	BICF2G630832376	28972155	A	0.5	0.2143	G
9	TIGRP2P124613_rs9113663	28986449	A	0	0.2857	C
9	BICF2S2313426	28991695	G	0.75	0.8571	A
9	BICF2G630832395	29012409	A	0	0	G
9	BICF2G630832397	29031665	G	0.25	0.3571	A
9	BICF2G630832401	29071229	G	0.25	0.3571	A
9	BICF2G630832402	29075374	A	0.25	0.3571	G
9	BICF2G630832408	29089053	A	0.25	0.3571	G
9	BICF2P686487	29099309	A	0	0	G
9	BICF2G630832410	29109787	A	0.25	0.3571	G
9	BICF2S2417582	29120809	0	0	0	G
9	BICF2S2417705	29133245	0	0	0	A
9	BICF2P1253749	29181386	0	0	0	A

9	TIGRP2P124651_rs8559278	29213439	0	0	0	G
9	BICF2P1376654	29214318	0	0	0	G
9	BICF2P1007869	29223694	G	0	0.2857	C
9	BICF2P880741	29237051	0	0	0	C
9	BICF2P175994	29246589	0	0	0	G
9	BICF2P903715	29260874	0	0	0	C
9	BICF2P1044560	29272628	G	0	0	A
9	BICF2S23733240	29280041	0	0	0	G
9	BICF2G630832420	29292538	A	0.25	0.6429	G
9	BICF2G630832428	29312147	A	0.25	0.3571	G
9	BICF2P247080	29317871	A	0.25	0.3571	G
9	BICF2S23724238	29344477	A	0	0	G
9	BICF2P621525	29359128	0	0	0	G
9	BICF2P128506	29363866	0	0	0	A
9	BICF2P703646	29376507	A	0	0	G
9	BICF2P1190838	29383948	A	0	0	C
9	BICF2P153894	29400686	0	0	0	C
9	BICF2P1112605	29419223	A	0.25	0.3571	G
9	BICF2S23448848	29433939	A	0.25	0.3571	G
9	BICF2P133831	29457806	A	0.5	0.2143	G
9	BICF2P1242434	29463998	C	1	0.5833	A
9	TIGRP2P124703_rs8930238	29477612	G	0	0	A
9	TIGRP2P124707_rs9054356	29490602	A	0	0	G
9	BICF2S23221582	29492561	A	0	0	G
9	BICF2P608608	29505562	A	0.25	0.3571	G
9	BICF2S23238306	29515328	C	0.25	0.3571	A
9	TIGRP2P124715_rs9175146	29523270	C	0.25	0.3571	A
9	BICF2S23127944	29533680	C	0	0	A
9	TIGRP2P124721_rs8965077	29544285	A	0	0	G
9	BICF2S23039639	29570819	A	0	0	G
9	TIGRP2P124731_rs9138044	29586941	A	0	0	G
9	BICF2S22931242	29609356	A	0	0	G
9	BICF2S22931930	29613105	G	0.5	0.2143	A
9	TIGRP2P124747_rs8966269	29625229	0	0	0	G
9	BICF2P56923	29643722	0	0	0	G
9	BICF2P1207279	29648722	A	0.25	0.3333	G
9	BICF2S23445364	29679793	G	0	0	A
9	BICF2P564610	29698678	G	0.5	0.7857	A
9	BICF2P823834	29705221	G	0.25	0.3571	A
9	TIGRP2P124782_rs9114659	29748883	G	0.25	0.4286	A
9	BICF2S23617078	29754900	G	0	0	A
9	TIGRP2P124788_rs8495442	29764297	C	0.5	0.5	G
9	BICF2P1460564	29765785	C	0.5	0.5	A
9	BICF2P1283262	29781458	G	0	0	A
9	BICF2P517678	29792987	A	0.25	0.6667	G
9	BICF2P934377	29800710	A	0.25	0.1429	C
9	BICF2P1251983	29814121	A	0.25	0.1429	G

9	BICF2P1097952	29823351	G	0.25	0.1429	A
9	BICF2P465288	29835643	G	0.25	0.08333	A
9	BICF2P1328332	29851726	A	0.25	0.1429	G
9	BICF2P1057164	29862030	C	0	0	A
9	BICF2S2301938	29862746	C	0	0	A
9	BICF2P90380	29870486	A	0	0	G
9	BICF2P1319502	29878004	G	0.75	0.5714	C
9	BICF2P265252	29887773	C	0.25	0.4286	A
9	BICF2P947825	29904024	A	0.75	0.5714	G
9	BICF2S2366542	29916599	G	0	0	A
9	BICF2S23439099	29925533	G	0.5	0.2143	A
9	BICF2P404465	29952052	0	0	0	A
9	BICF2S23440631	29968070	C	0.5	0.7857	A
9	BICF2P278275	29976729	0	0	0	A
9	BICF2P15124	29985725	A	0.25	0.3571	G
9	BICF2P865293	30002348	A	1	0.7143	C
9	BICF2P335760	30018537	A	0.75	0.8571	G
9	BICF2P408899	30037376	A	0	0	G
9	BICF2P408901	30038029	G	0	0	A
9	BICF2S23434833	30050606	A	0.5	0.5	G
9	BICF2P78288	30066211	A	0.5	0.7857	G
9	BICF2P1014337	30073435	G	0.5	0.2143	A
9	BICF2P249497	30087890	G	0	0	A
9	BICF2P271346	30093711	A	0	0	G
9	TIGRP2P124858_rs9005595	30099170	A	0.25	0.1429	G
9	BICF2P835557	30115225	G	0.5	0.2143	A
9	BICF2P664463	30118766	A	0.5	0.7857	G
9	BICF2P11380	30140216	A	0	0	G
9	BICF2P1290874	30145702	G	0	0	A
9	TIGRP2P124881_rs9041767	30153060	G	0.75	0.8571	A
9	BICF2P1047684	30167303	A	0	0	G
9	BICF2P780489	30179511	G	0.75	0.8571	A
9	BICF2P1266509	30188294	A	0.5	0.2143	G
9	BICF2P136137	30203307	A	0	0	G
9	BICF2S23454437	30213435	G	0	0	A
9	TIGRP2P124898_rs8896240	30224874	0	0	0	C
9	BICF2P460526	30235284	A	0.25	0.6429	G
9	BICF2S2443137	30252001	G	0	0	A
9	BICF2P1385209	30258635	C	0	0	A
9	BICF2P7893	30277129	T	0	0	A
9	BICF2S23220057	30296922	A	0	0	C
9	BICF2S23734161	30330162	G	0.75	0.8571	A
9	TIGRP2P124916_rs8517770	30345642	G	0.75	0.8571	A
9	BICF2P612726	30352097	A	0.75	0.8571	C
9	BICF2P411176	30368078	G	0.75	0.8571	A
9	BICF2P31424	30371848	A	0.75	0.8571	G
9	BICF2P461486	30405752	G	0.25	0.1429	A

9	BICF2P1287982	30417877	A	0.25	0.1429	C
9	BICF2P1456913	30436447	C	0.25	0.1429	A
9	BICF2S23130870	30441444	A	0.5	0.2143	G
9	BICF2P965562	30452764	A	0.75	0.3571	G
9	BICF2P930986	30468313	G	0.25	0.6429	A
9	TIGRP2P124950_rs8723618	30475652	A	0.25	0.6429	C
9	BICF2P921874	30487096	A	0.5	0.7857	G
9	BICF2P1063363	30499861	A	0.5	0.2143	G
9	BICF2P646766	30504831	G	0	0	A
9	BICF2P772317	30513015	G	0.5	0.8333	A
9	BICF2P994012	30522669	G	0.5	0.7857	A
9	BICF2P346418	30535071	A	0.5	0.7857	G
9	BICF2S23646726	30546696	C	0.25	0.1429	A
9	BICF2P595751	30566681	G	0	0	A
9	BICF2P859170	30568116	0	0	0	C
9	BICF2P845006	30591080	G	0	0	A
9	BICF2P895077	30610760	A	0	0	G
9	BICF2P904943	30611557	A	1	1	G
9	BICF2S23454318	30631271	A	0.25	0.6429	C
9	BICF2P1425742	30647370	A	0.25	0.6429	C
9	BICF2P524622	30651594	G	0	0	A
9	BICF2S22926273	30663559	A	0.5	0.2143	G
9	BICF2S23446804	30681708	G	0	0	A
9	BICF2P570450	30697279	G	1	1	A
9	BICF2P225354	30701513	G	0.25	0.1429	A
9	BICF2S23913393	30706965	G	0.75	0.3571	A
9	BICF2S23660012	30720191	A	0	0	G
9	BICF2P44044	30745103	G	0.25	0.1429	A
9	BICF2P1225976	30754535	A	0	0	G
9	BICF2P1095566	30774122	C	0	0	A
9	BICF2P405846	30789411	G	0	0	A
9	BICF2S2325490	30795413	C	0	0	A
9	BICF2S23357108	30824740	A	0.25	0.6429	G
9	BICF2P369344	30852878	G	0.25	0.6429	A
9	BICF2P369345	30853111	T	0	0	A
9	TIGRP2P125005_rs8797941	30857596	A	0.25	0.6429	G
9	BICF2P791708	30870027	A	0	0	G
9	BICF2P316925	30883441	0	0	0	A
9	BICF2P1398603	30890432	G	0.75	0.3571	A
9	BICF2P1398601	30890606	G	0.75	0.3571	A
9	TIGRP2P125027_rs8519153	30903733	A	0.5	0.2143	C
9	BICF2S2365146	30918083	G	0.25	0.1429	A
9	BICF2P1008064	30926816	T	0	0	A
9	BICF2S23645786	30956888	G	0	0	A
9	BICF2S23648273	30965162	G	0	0	A
9	BICF2P1420892	30970175	G	0	0.6429	A
9	TIGRP2P125037_rs8777456	30981287	A	0.25	0.6429	G

9	BICF2P1456147	30993821	0	0	0	A
9	BICF2P1036408	31006006	A	0.25	0.6429	G
9	TIGRP2P125055_rs8787315	31016192	A	0.75	0.3571	G
9	BICF2S2337872	31027374	G	0.75	0.3571	A
9	BICF2P864056	31046456	G	0.25	0.6429	A
9	BICF2S23029406	31061297	C	0	0	A
9	BICF2P579558	31067109	G	0.25	0.6429	A
9	BICF2P222398	31074375	A	0.75	0.3571	G
9	BICF2P439781	31091251	A	0.25	0.6429	G
9	BICF2P851059	31094197	G	0.25	0.6429	A
9	BICF2S23258442	31119264	A	0.25	0.6429	G
9	BICF2P13550	31137779	G	0.25	0.6429	A
9	BICF2S23321299	31149117	A	0	0	G
9	TIGRP2P125076_rs9006818	31162799	A	0.75	0.3571	G
9	BICF2P575468	31162999	C	0.25	0.6429	A
9	TIGRP2P125086_rs9169757	31168032	0	0	0	A
9	BICF2P1297013	31180407	G	0.75	0.3571	A
9	BICF2P236403	31190332	A	0	0	G
9	BICF2G630832458	31195063	A	0.5	0.2143	G
9	BICF2G630832463	31199614	A	0.75	0.3571	G
9	BICF2S23311801	31217337	C	0.25	0.6429	A
9	BICF2G630832496	31226717	G	0.75	0.3571	A
9	BICF2G630832509	31236960	A	0	0	G
9	TIGRP2P125117_rs8576756	31251143	A	0	0	G
9	BICF2P1351569	31251493	C	0	0	G
9	BICF2G630832553	31259757	G	0	0	A
9	BICF2G630832571	31270911	A	0	0	C
9	BICF2G630832598	31285649	A	0	0	G
9	BICF2P1467481	31291347	G	0	0	A
9	BICF2P1260647	31297775	G	0.75	0.8571	A
9	BICF2G630832634	31305477	A	0.25	0.1429	C
9	BICF2P904013	31316264	A	0.25	0.1429	G
9	BICF2S23445230	31335044	A	0.75	0.8571	G
9	BICF2G630832689	31338368	0	0	0	A
9	BICF2P1468397	31360047	A	0.25	0.6429	G
9	BICF2P1188773	31367534	0	0	0	A
9	BICF2G630832709	31375287	A	0	0	G
9	BICF2S23037275	31385446	A	0.5	0.7857	G
9	BICF2G630832753	31395897	A	0.5	0.7857	C
9	BICF2G630832768	31407604	A	0.25	0.1429	G
9	BICF2S23212757	31422467	A	0.5	0.2143	G
9	BICF2G630832826	31435434	G	0	0	A
9	BICF2P218365	31442845	A	0	0	G
9	BICF2G630832847	31453097	A	0	0	G
9	BICF2G630832886	31476272	G	0.5	0.2143	A
9	BICF2P445752	31515824	A	0.5	0.2143	T
9	TIGRP2P125193_rs8961396	31522542	C	0.5	0.2143	A

9	BICF2G630832914	31542090	A	0.5	0.7857	G
9	BICF2G630832924	31549819	G	0.5	0.7857	A
9	BICF2G630832982	31571947	A	0	0	G
9	BICF2G630833002	31588332	A	0	0	G
9	BICF2G630833006	31594176	A	0	0	G
9	BICF2P439373	31607014	A	0	0	G
9	BICF2S23657764	31610370	A	0	0	G
9	BICF2G630833086	31625422	0	0	0	C
9	BICF2S231667	31641537	G	0.5	0.2143	A
9	BICF2G630833089	31650441	0	0	0	A
9	BICF2G630833111	31668697	G	0	0	A
9	BICF2P1140871	31676163	0	0	0	A
9	BICF2G630833137	31688563	G	0	0	A
9	TIGRP2P125218_rs9191977	31700038	G	0	0	A
9	BICF2G630833150	31708751	A	0	0	C
9	BICF2G630833193	31727999	G	0.5	0.2143	C
9	BICF2G630833199	31731146	G	0	0	A
9	BICF2S2327014	31741984	G	0.5	0.2143	A
9	BICF2P734473	31757174	0	0	0	A
9	BICF2P343539	31764375	A	0	0	G
9	BICF2P572850	31778462	A	0	0	G
9	BICF2P849794	31793988	A	0	0	G
9	BICF2S23051048	31806338	T	0.5	0.2143	A
9	BICF2P273143	31809310	G	0.5	0.2143	A
9	TIGRP2P125274_rs8923497	31826533	C	0	0	A
9	BICF2P592997	31837101	C	0	0	A
9	BICF2P297901	31844320	C	0	0	A
9	BICF2P165135	31858639	A	0	0	G
9	BICF2P608643	31872270	A	0.5	0.2143	G
9	BICF2P1167028	31894800	G	0.25	0.1429	A
9	TIGRP2P125320_rs8795008	31911559	A	0.5	0.2143	G
9	BICF2P923923	31912890	G	0.25	0.1429	A
9	BICF2P1112691	31917571	A	0.5	0.2143	G
9	BICF2P1112693	31917964	A	0.5	0.2143	C
9	BICF2P782452	31926056	C	0.5	0.2143	A
9	TIGRP2P125338_rs9070968	31938072	A	0.25	0.6429	G
9	BICF2G630833211	31948751	A	0.5	0.2143	G
9	BICF2P648889	31974913	A	0	0	G
9	BICF2G630833244	31985674	A	0.25	0.6429	G
9	BICF2G630833261	31996195	0	0	0	G
9	BICF2G630833273	32016223	A	0	0	G
9	BICF2G630833289	32039127	A	1	1	G
9	BICF2P126723	32053513	A	0.25	0.6429	G
9	BICF2P1156337	32073335	G	0.5	0.2143	A
9	BICF2G630833300	32074586	A	0.75	0.8571	G
9	TIGRP2P125376_rs8924630	32118564	G	0.5	0.2143	C
9	BICF2P741492	32122613	A	0.25	0.1429	C

9	BICF2G630833353	32174379	A	0	0	G
9	BICF2P1006817	32185105	0	0	0	G
9	BICF2G630833361	32194464	A	0.25	0.6429	C
9	BICF2G630833374	32200160	G	0	0	A
9	BICF2P198852	32216181	G	0.75	0.8571	A
9	BICF2G630833380	32223968	A	0.75	0.8571	G
9	BICF2G630833403	32235900	G	1	1	A
9	BICF2G630833414	32246346	A	0	0	G
9	BICF2G630833437	32264461	G	0	0.1429	A
9	BICF2G630833451	32275301	A	0	0	G
9	BICF2P1115752	32276964	A	0.75	0.8571	G
9	BICF2S23526723	32287797	A	0.25	0.6429	G
9	BICF2G630833477	32296152	A	0	0	G
9	BICF2G630833496	32304597	0	0	0	G
9	BICF2G630833504	32317715	0	0	0	G
9	BICF2G630833516	32329065	G	0	0	A
9	BICF2P403525	32345254	C	0.25	0.6667	A
9	BICF2G630833530	32349999	A	0	0	C
9	BICF2S23124692	32366061	A	0.25	0.6429	G
9	BICF2S23616631	32372838	A	0.25	0.6429	G
9	BICF2P86688	32394332	A	0.5	0.1667	G
9	BICF2P400707	32404436	A	0	0	G
9	BICF2P1226555	32417956	G	0.75	0.8571	A
9	BICF2P793963	32431122	A	0.5	0.2143	G
9	BICF2S23730957	32442291	A	0.75	0.3571	C
9	BICF2P771167	32445877	A	0.75	0.3	G
9	BICF2G630833538	32453720	A	0	0	C
9	BICF2P303456	32469420	G	1	1	A
9	BICF2G630833560	32471341	C	0.25	0.6429	A
9	BICF2S23237727	32473750	C	0.25	0.6429	A
9	BICF2G630833581	32478681	C	0.25	0.6429	A
9	BICF2G630833598	32487935	G	0	0	A
9	TIGRP2P125505_rs8755867	32499594	0	0	0	G
9	BICF2P141320	32516668	G	0	0	A
9	TIGRP2P125522_rs9216816	32523488	A	0.75	0.8571	G
9	BICF2P633807	32543933	G	0.25	0.6429	A
9	TIGRP2P125526_rs9186537	32546434	A	0.25	0.6429	G
9	BICF2G630833617	32562381	A	0.25	0.6429	G
9	BICF2G630833623	32570304	A	0	0	G
9	BICF2G630833633	32588392	A	0	0	G
9	BICF2G630833642	32597679	G	0.25	0.1429	C
9	BICF2S23737788	32602678	G	0	0	A
9	BICF2S23441620	32616389	A	0	0	C
9	BICF2G630833644	32635299	A	0	0	G
9	BICF2S2442024	32666562	G	0	0	C
9	BICF2S23416345	32686741	G	0	0	A
9	BICF2P961721	32697596	0	0	0	G

9	BICF2S23528313	32706394	T	0	0	A
9	BICF2S24113908	32720567	0	0	0	C
9	BICF2S23652401	32730723	0	0	0	A
9	BICF2P1137291	32754285	A	0.5	0.1667	C
9	BICF2S23743595	32787377	G	0	0	A
9	BICF2G630833654	32801692	A	0	0	G
9	TIGRP2P125543_rs8576216	32823801	A	0	0	G
9	BICF2G630833680	32824187	A	0	0	G
9	BICF2P1426852	32838630	0	0	0	A
9	BICF2G630833694	32850477	A	0	0	G
9	BICF2P1161648	32870416	G	0.25	0.6429	A
9	BICF2P365782	32880304	A	0.75	0.8571	G
9	BICF2P529809	32890933	A	1	0.8571	G
9	BICF2P731888	32909418	G	0.75	0.8571	A
9	BICF2G630833717	32923794	A	0	0	G
9	BICF2S23412397	32928747	A	0	0	G
9	BICF2P12813	32942031	0	0	0	A
9	BICF2P1171323	32969524	0	0	0	C
9	TIGRP2P125580_rs8598161	32976914	G	0	0	A
9	TIGRP2P125582_rs8650416	32992759	A	0	0	G
9	BICF2S23657953	32994533	A	0	0	G
9	BICF2P1379015	33016084	A	0	0	G
9	TIGRP2P125591_rs9046953	33025118	A	0	0	C
9	BICF2P203331	33033761	A	0	0	T
9	BICF2P1039916	33041000	A	0	0	G
9	BICF2P326994	33053882	A	0	0	G
9	BICF2P532327	33065270	A	0.25	0.6429	C
9	BICF2P174301	33075236	G	0.25	0.6429	A
9	TIGRP2P125634_rs8791892	33096133	G	0	0	A
9	BICF2G630833774	33102532	G	0.25	0.6429	A
9	BICF2P889641	33111844	A	0	0	G
9	TIGRP2P125642_rs9241155	33121838	A	0	0	G
9	BICF2G630833841	33136942	A	0	0	G
9	BICF2G630833888	33152097	A	0	0	G
9	BICF2G630833895	33154676	A	0	0	G
9	BICF2S23225223	33166387	0	0	0	A
9	TIGRP2P125655_rs9075769	33179354	A	0	0	G
9	BICF2P243419	33196426	0	0	0	A
9	BICF2P848241	33206923	0	0	0	A
9	BICF2G630833906	33221088	G	0.25	0.1429	A
9	BICF2G630833912	33226373	A	0.25	0.1429	G
9	BICF2G630833921	33241848	G	0.25	0.1429	A
9	BICF2G630833922	33247670	A	0.25	0.1429	G
9	BICF2G630833937	33258313	G	0.25	0.08333	A
9	BICF2G630833948	33279166	A	0.25	0.1429	G
9	BICF2G630833968	33305226	A	0.5	0.2143	G
9	BICF2S2312436	33327518	A	0	0	G

9	BICF2S22914451	33341090	A	0.5	0.7857	G
9	BICF2P766568	33352698	A	0.5	0.7857	G
9	BICF2G630833993	33363319	G	0.5	0.7857	A
9	BICF2G630834000	33366933	A	0	0	C
9	BICF2G630834017	33375409	A	0.5	0.7857	G
9	BICF2P441965	33389661	G	0.5	0.2143	A
9	BICF2G630834044	33407485	G	0.25	0.1429	A
9	BICF2G630834051	33408680	A	0.25	0.1429	G
9	BICF2G630834074	33426328	A	0.25	0.1429	G
9	BICF2G630834084	33435717	G	0	0	A
9	BICF2G630834099	33442140	0	0	0	A
9	BICF2P352509	33455273	G	0.25	0.1429	A
9	BICF2G630834126	33476545	A	0.5	0.2143	G
9	BICF2G630834148	33497140	A	0.25	0.1429	G
9	BICF2G630834150	33500099	A	0.25	0.1429	G
9	BICF2G630834171	33512035	G	0.25	0.1429	A
9	BICF2P67517	33544469	0	0	0	A
9	BICF2P1053335	33547182	G	0.5	0.7857	A
9	BICF2G630834181	33552225	A	0.5	0.7857	C
9	BICF2P895444	33565590	0	0	0	C
9	TIGRP2P125686_rs8962528	33585130	A	0	0	C
9	BICF2S23126894	33603473	A	1	1	G
9	BICF2S2349702	33622986	A	0	0	G
9	BICF2G630834196	33632784	G	0.25	0.1429	A
9	BICF2G630834200	33638821	G	0.25	0.1429	A
9	BICF2G630834202	33639293	C	0.25	0.1429	G
9	BICF2G630834214	33655712	A	0.75	0.3571	C
9	BICF2G630834221	33667371	A	0.75	0.3571	G
9	BICF2S22942358	33672109	A	0.75	0.3571	G
9	BICF2P1358309	33716392	0	0	0	G
9	BICF2G630834233	33723172	G	1	1	A
9	BICF2P269899	33729770	A	0	0	G
9	BICF2P855951	33735571	G	0.5	0.2143	A
9	BICF2P747243	33743749	A	0.25	0.6429	G
9	BICF2G630834247	33745329	A	0	0	G
9	BICF2P437486	33745408	A	0	0	G
9	BICF2S23433019	33754761	A	0	0	G
9	BICF2G630834251	33767096	A	0.5	0.2143	G
9	BICF2P746247	33784632	G	0.75	0.8571	A
9	BICF2P356973	33788824	G	0.75	0.8571	A
9	BICF2P275732	33803977	A	0	0	G
9	TIGRP2P125736_rs8803563	33814731	A	0	0	G
9	BICF2P616959	33827329	0	0	0	A
9	BICF2G630834263	33853833	A	0	0	G
9	BICF2P190873	33864619	A	0.75	0.3571	G
9	BICF2P1260486	33873555	G	0.75	0.3571	A
9	BICF2P341690	33877060	A	0.75	0.3571	G

9	BICF2G630834275	33879331	G	1	1	A
9	BICF2G630834299	33895347	G	1	1	A
9	BICF2G630834316	33908322	A	0	0	G
9	BICF2G630834343	33918647	A	0	0	G
9	BICF2P678209	33927582	A	0.5	0.2143	G
9	BICF2G630834345	33937906	C	0.5	0.1667	A
9	TIGRP2P125762_rs8635470	33951974	G	1	1	A
9	BICF2P53257	33963114	A	0.5	1	G
9	BICF2G630834364	33972401	0	0	0	G
9	BICF2P191160	33983680	C	0.5	0.2143	A
9	BICF2P76406	34004884	G	0.5	0.2143	A
9	BICF2P76412	34006150	G	0	0	A
9	TIGRP2P125783_rs9027243	34017229	T	0	0	A
9	BICF2P20832	34039241	C	1	1	A
9	TIGRP2P125852_rs9045688	34066967	G	0.75	0.3571	A
9	BICF2P760310	34133373	A	0	0	C
9	BICF2S23011878	34177196	A	0.25	0.1429	G
9	BICF2G630834372	34193946	A	0.25	0.6429	G
9	BICF2G630834393	34207784	C	0.75	0.3571	G
9	BICF2G630834422	34235253	A	0.25	0.6429	G
9	BICF2S2319354	34241589	G	0.25	0.6429	A
9	TIGRP2P125999_rs9085962	34250888	A	0.25	0.6429	T
9	BICF2G630834450	34263517	G	0	0	A
9	TIGRP2P126003_rs8802234	34274903	A	0.75	0.3571	C
9	BICF2G630834483	34282898	A	0	0	G
9	TIGRP2P126010_rs8582718	34302324	A	0.25	0.1429	G
9	BICF2P1067183	34312062	A	0.75	0.8571	G
9	TIGRP2P126044_rs9006420	34318130	G	0	0	A
9	BICF2S2394588	34358974	A	0	0	G
9	BICF2G630834503	34371895	G	0	0	A
9	BICF2G630834518	34374828	G	0	0	A
9	BICF2P1346928	34390137	A	0	0	G
9	BICF2G630834549	34410918	A	0.5	0.7857	G
9	BICF2P61687	34420937	C	0.25	0.1429	A
9	BICF2P706041	34437181	G	0.5	0.7857	A
9	BICF2G630834559	34442607	A	0.25	0.6429	G
9	TIGRP2P126062_rs8704994	34457167	A	0.25	0.1429	C
9	BICF2P1232604	34466551	G	0.75	0.3571	A
9	BICF2G630834566	34479374	A	0.25	0.6429	G
9	BICF2P1333896	34499994	A	0.25	0.1429	T
9	BICF2G630834614	34504984	G	0.25	0.1429	A
9	BICF2G630834651	34521042	G	0.25	0.1429	A
9	BICF2G630834665	34533282	A	0	0	T
9	BICF2P990045	34537812	A	0.5	0.2143	G
9	BICF2G630834681	34552058	A	0.5	0.7857	G
9	BICF2G630834687	34560009	G	0	0	A
9	BICF2G630834692	34576107	C	0	0	A

9	BICF2G630834698	34584807	A	0.25	0.1429	G
9	BICF2G630834701	34597503	A	0.25	0.1429	G
9	BICF2G630834702	34611838	A	0.25	0.1429	G
9	BICF2S23027935	34624817	A	0.5	0.2143	G
9	BICF2G630834711	34631343	A	0.25	0.1429	G
9	BICF2G630834713	34636210	G	0.25	0.1429	A
9	BICF2G630834716	34639093	G	0.75	0.3333	A
9	BICF2G630834731	34650699	A	0.25	0.1429	C
9	BICF2G630834738	34656519	G	0.25	0.1429	A
9	BICF2G630834747	34665747	G	0.25	0.1429	A
9	BICF2G630834761	34678144	A	0.25	0.1429	G
9	BICF2G630834765	34686161	A	0.25	0.1429	G
9	BICF2P460598	34700464	C	0.25	0.1429	A
9	BICF2P742007	34711686	G	0.25	0.6429	A
9	BICF2G630834778	34729755	A	0	0	G
9	BICF2G630834780	34730809	G	0.25	0.1429	A
9	BICF2G630834793	34751362	G	0.25	0.1429	A
9	BICF2P567571	34759291	A	0.75	0.3571	G
9	BICF2G630834810	34769578	A	0.25	0.6429	G
9	BICF2G630834815	34776466	G	0	0	A
9	TIGRP2P126109_rs9108341	34790380	G	0	0	A
9	BICF2G630834826	34801441	A	0	0	G
9	BICF2G630834847	34811317	G	0.5	0.2143	A
9	BICF2G630834875	34828682	A	0.5	0.7857	G
9	BICF2G630834878	34836960	G	0	0	A
9	TIGRP2P126143_rs9175081	34867903	A	0.75	0.8571	C
9	BICF2G630834902	34874150	A	0.75	0.3571	G
9	BICF2G630834918	34880035	A	0.25	0.1429	G
9	BICF2G630834930	34901272	A	0	0	G
9	BICF2G630834937	34904773	0	0	0	C
9	BICF2G630834938	34918580	G	1	1	A
9	BICF2G630834939	34926806	0	0	0	G
9	BICF2G630834943	34939097	G	0.25	0.6429	C
9	BICF2G630834951	34947381	A	0	0	G
9	TIGRP2P126159_rs8812201	34949094	0	0	0	G
9	BICF2G630834969	34963126	A	0	0	G
9	TIGRP2P126164_rs8867691	34998466	0	0	0	A
9	BICF2G630834997	35028337	G	0.5	0.6667	A
9	BICF2G630834998	35043288	A	0.25	0.6429	G
9	BICF2G630835002	35095829	G	0	0	A
9	BICF2G630835005	35098788	A	1	1	G
9	BICF2S23454768	35114509	G	0	0	A
9	BICF2S22938500	35123803	0	0	0	G
9	BICF2P322014	35139005	0	0	0	A
9	BICF2P929240	35150618	0	0	0	G
9	BICF2P262393	35157005	0	0	0	C
9	BICF2S2319639	35172669	A	0.5	0.2143	G

9	BICF2S23535421	35180836	A	1	1	G
9	BICF2G630835029	35197123	A	0.25	0.1429	G
9	BICF2G630835034	35204040	A	0	0	G
9	BICF2P285716	35214288	A	0	0	G
9	BICF2P1409608	35225893	G	1	1	C
9	BICF2S2319072	35251567	G	1	1	A
9	BICF2G630835049	35264352	A	0.75	0.8571	G
9	BICF2P1299747	35280786	G	0.75	0.8571	A
9	BICF2G630835055	35286704	C	0.75	0.9167	A
9	BICF2G630835062	35292186	G	0.25	0.6429	A
9	BICF2G630835075	35298039	G	0	0	A
9	BICF2G630835109	35320954	G	0.25	0.6429	A
9	BICF2G630835127	35329054	A	0.25	0.1429	C
9	BICF2G630835134	35350310	G	0	0	A
9	BICF2G630835140	35352380	A	0	0	G
9	BICF2G630835159	35371920	A	0	0	C
9	BICF2G630835167	35378067	A	0	0	G
9	BICF2P500923	35401486	G	0.5	0.7857	A
9	BICF2P515478	35441401	A	0.25	0.6429	G
9	BICF2G630835179	35453694	G	0.5	0.7857	A
9	BICF2P72186	35455368	G	0.5	0.7857	A
9	BICF2G630835181	35460397	G	0.5	0.7857	A
9	TIGRP2P126171_rs9026734	35468283	0	0	0	C
9	BICF2G630835183	35479269	A	0.5	0.7857	C
9	BICF2G630835188	35489664	A	0.5	0.7857	C
9	BICF2G630835202	35504893	G	0.5	0.7857	A
9	BICF2G630835214	35514332	G	0.5	0.7857	A
9	BICF2G630835218	35524641	G	0.5	0.7857	A
9	BICF2P976417	35556935	0	0	0	G
9	BICF2G630835220	35569993	A	0.5	0.7857	G
9	BICF2G630835221	35581444	G	0.25	0.6429	A
9	BICF2G630835223	35595348	G	0	0	A
9	BICF2S23345425	35606279	A	0.5	0.1	G
9	BICF2P274574	35616538	0	0	0	A
9	BICF2G630835225	35635551	A	0.25	0.6429	G
9	BICF2S2296203	35671597	A	0.25	0.1429	G
9	BICF2P220507	35676099	0	0	0	A
9	TIGRP2P126175_rs9141882	35691218	0	0	0	G
9	BICF2P283506	35706022	A	0	0	G
9	BICF2G630835236	35708735	G	0	0	A
9	BICF2G630835241	35719582	A	0.25	0.6429	C
9	BICF2G630835244	35724781	A	0.25	0.6667	G
9	TIGRP2P126176_rs9191899	35733151	0	0	0	G
9	BICF2G630835259	35743892	A	0.75	0.8571	C
9	BICF2P756517	35760417	A	0.25	0.1429	G
9	BICF2G630835284	35775154	A	0	0	G
9	BICF2G630835295	35778983	A	0	0	G

9	BICF2G630835298	35783414	A	0	0	G
9	BICF2G630835306	35787015	G	0.5	0.2143	A
9	BICF2G630835371	35807871	A	1	1	C
9	BICF2G630835384	35812061	A	1	1	G
9	BICF2G630835400	35825603	A	0.5	0.1	G
9	BICF2G630835408	35836676	A	0.5	0.2143	G
9	BICF2P520511	35856878	G	0.5	0.2143	A
9	BICF2P992513	35865650	A	0.5	0.2143	G
9	BICF2G630835436	35870411	A	0.5	0.2143	G
9	BICF2G630835442	35871080	A	0.5	0.2143	T
9	BICF2G630835465	35883569	G	0	0	A
9	BICF2G630835466	35893015	A	0	0	G
9	BICF2G630835468	35916622	G	0.5	0.2143	A
9	BICF2P7272	35929782	0	0	0	G
9	BICF2S23129241	35942414	G	0.5	0.2143	A
9	BICF2G630835479	35958985	G	0	0	A
9	BICF2P1436081	35970660	A	0	0	G
9	BICF2P1436079	35972308	C	1	1	G
9	BICF2P444707	35984516	G	1	1	A
9	BICF2G630835485	36001950	G	1	1	C
9	BICF2G630835487	36008826	G	1	1	A
9	BICF2G630835514	36047518	A	1	1	G
9	BICF2P161486	36052653	0	0	0	A
9	BICF2S23754553	36069998	A	1	1	G
9	BICF2P231335	36082209	0	0	0	C
9	BICF2G630835521	36109993	A	0	0	G
9	BICF2G630835522	36110703	A	0.25	0.6429	G
9	BICF2S23744057	36124815	A	1	1	G
9	BICF2S23721267	36150732	G	0.5	0.7857	A
9	BICF2P36978	36163517	A	0.5	0.7857	G
9	BICF2S23042367	36170165	A	0.5	0.2143	C
9	BICF2P1352207	36184164	0	0	0	G
9	BICF2P738000	36190831	A	0.5	0.7857	G
9	BICF2G630835536	36209379	A	0.5	0.7857	G
9	BICF2G630835558	36219690	A	0	0	C
9	BICF2P939668	36232996	0	0	0	G
9	BICF2S22912444	36237254	G	0.5	0.7857	A
9	BICF2G630835589	36251709	G	0.5	0.2143	A
9	BICF2G630835599	36261626	G	0	0	A
9	BICF2G630835610	36272945	A	0	0	G
9	BICF2G630835611	36278415	A	0	0	G
9	BICF2P977251	36302389	G	1	1	A
9	BICF2P881745	36314602	A	0	0	G
9	BICF2P563733	36336258	A	1	1	G
9	BICF2S23112305	36344800	G	0	0	C
9	BICF2P1257515	36356781	A	1	1	G
9	BICF2P1428969	36373814	G	0	0	A

9	BICF2S23355768	36375420	A	0	0	G
9	BICF2P791837	36397059	A	0	0	G
9	BICF2P929417	36400807	A	1	1	G
9	BICF2P1381099	36411260	0	0	0	A
9	BICF2G630835629	36430971	A	0	0	G
9	BICF2P214370	36443150	0	0	0	A
9	BICF2P1263641	36457488	G	0.5	0.7857	A
9	BICF2P1263640	36457532	A	0.5	0.2143	G
9	BICF2G630835632	36476472	0	0	0	A
9	BICF2S22955019	36496633	C	0	0	A
9	TIGRP2P126196_rs8672929	36506257	0	0	0	A
9	BICF2P203163	36519050	0	0	0	A
9	BICF2G630835636	36540383	A	0	0	C
9	TIGRP2P126201_rs8645124	36568382	0	0	0	A
9	BICF2P1401890	36580998	G	0.5	0.2143	A
9	BICF2G630835642	36604582	0	0	0	A
9	BICF2P37374	36661134	G	0.5	0.7857	A
9	BICF2G630835648	36677741	G	0	0	A
9	TIGRP2P126206_rs9065709	36754015	0	0	0	A
9	BICF2P1372541	36771372	0	0	0	C
9	BICF2G630835652	36812653	G	0	0	A
9	G554f20S145	36836459	A	0	0	G
9	BICF2P1016594	36866936	0	0	0	G
9	BICF2S23223061	36873235	0	0	0	G
9	BICF2S2327145	36882814	A	0	0	G
9	TIGRP2P126211_rs8697703	36898252	0	0	0	A
9	BICF2P506752	36907558	A	0	0	G
9	BICF2G630835679	36926394	C	0	0	A
9	BICF2G630835683	36928054	A	0	0	G
9	BICF2P1060321	36947882	0	0	0	A
9	BICF2P661018	36953954	0	0	0	A
9	BICF2P1403618	36963437	0	0	0	A
9	BICF2G630835693	36973872	C	0	0	G
9	BICF2G630835696	36990350	A	0	0	G
9	BICF2G630835699	37002989	A	0	0	G
9	BICF2G630835701	37008674	0	0	0	G
9	BICF2G630835711	37020059	T	0	0	A
9	BICF2G630835732	37035203	G	0	0	A
9	BICF2G630835753	37047450	A	0	0	G
9	BICF2G630835762	37055117	G	0	0	A
9	BICF2G630835777	37072140	G	0	0	A
9	BICF2G630835788	37081352	G	0	0	A
9	BICF2S2354885	37091173	G	0	0	A
9	BICF2G630835816	37107410	G	0	0	A
9	BICF2G630835821	37129410	G	0	0	A
9	BICF2G630835824	37136522	A	0	0	G
9	BICF2G630835834	37147898	G	0	0	A

9	BICF2G630835852	37161142	A	0	0	G
9	BICF2G630835868	37176309	G	0	0	A
9	BICF2G630835882	37185046	A	0	0	G
9	BICF2P224043	37213218	A	0.5	0.1667	G
9	BICF2S23349026	37222555	G	0.5	0.2143	A
9	BICF2S23432177	37230849	0	0	0	G
9	BICF2G630835944	37241043	A	0	0	G
9	BICF2G630835960	37259704	C	0.5	0.2143	A
9	BICF2G630835961	37262825	T	0.5	0.2143	A
9	BICF2G630835962	37263005	A	0.5	0.2143	C
9	BICF2G630835964	37264397	A	0.5	0.2143	G
9	BICF2G630835974	37272569	G	0.5	0.2143	A
9	BICF2G630835989	37286196	C	0	0	A
9	BICF2G630836012	37298087	G	0	0	A
9	BICF2P312765	37308929	A	0.5	0.2143	G
9	BICF2P918457	37317951	T	0	0	A
9	BICF2G630836042	37336055	G	0	0	A
9	BICF2P121124	37352533	G	0	0	A
9	BICF2S23423866	37367547	C	0	0	A
9	BICF2G630836073	37378242	A	0	0	G
9	BICF2G630836094	37399884	G	0	0	A
9	BICF2P1225649	37417533	A	0	0	G
9	BICF2G630836099	37432235	G	0	0	A
9	BICF2G630836101	37437915	G	0	0	A
9	BICF2G630836102	37453317	A	0	0	G
9	BICF2P920890	37460114	G	0.5	0.7857	A
9	BICF2P62310	37468526	G	0.5	0.7857	A
9	BICF2P943080	37480293	A	0.5	0.7857	G
9	BICF2P309205	37491968	G	0.5	0.7857	A
9	BICF2G630836108	37504858	0	0	0	A
9	BICF2G630836109	37519916	0	0	0	G
9	BICF2P835256	37533691	G	0.5	0.2143	A
9	BICF2S22940626	37539543	C	0.5	0.2143	A
9	BICF2G630836114	37558924	0	0	0	A
9	BICF2G630836116	37565942	A	0.5	0.2143	G
9	BICF2G630836117	37578479	A	0.5	0.2143	G
9	BICF2P807376	37592042	A	0.5	0.8333	C
9	BICF2G630836121	37597835	0	0	0	C
9	BICF2P763284	37630102	0	0	0	T
9	BICF2G630836132	37649821	A	0.5	0.2143	G
9	BICF2G630836133	37659095	A	0.5	0.1667	C
9	BICF2G630836139	37679430	G	1	1	A
9	BICF2S23325673	37700684	A	1	1	G
9	BICF2G630836146	37710165	0	0	0	C
9	BICF2P751777	37729220	A	1	1	C
9	BICF2P1337755	37734122	A	0.5	0.7857	T
9	BICF2G630836158	37765579	0	0	0	A

9	BICF2P435582	37766183	A	1	1	T
9	BICF2G630836177	37779346	0	0	0	G
9	BICF2P768698	37791568	A	1	1	G
9	BICF2G630836211	37813264	0	0	0	G
9	BICF2G630836219	37825061	0	0	0	A
9	BICF2P739055	37879856	0	0	0	A
9	BICF2G630836255	37895840	0	0	0	C
9	BICF2P395907	37917201	G	0.5	0.7857	A
9	BICF2G630836268	37936717	A	0	0	G
9	BICF2G630836272	37941157	A	0	0	G
9	BICF2G630836278	37951211	G	0	0	A
9	BICF2P1427054	37965807	G	0	0	C
9	BICF2G630836280	37977100	A	0.5	0.8333	C
9	BICF2G630836291	38011817	A	0	0	G
9	BICF2G630836293	38022555	G	0	0	A
9	BICF2P635069	38033201	0	0	0	A
9	BICF2G630836294	38046024	G	0	0	A
9	BICF2G630836300	38071888	A	0.5	0.7857	G
9	BICF2G630836306	38076908	A	0	0	G
9	BICF2G630836318	38099586	A	0	0	G
9	BICF2P785313	38112330	G	0	0	A
9	BICF2G630836329	38133017	G	0	0	A
9	BICF2P1031527	38139354	A	1	1	G
9	BICF2P326735	38169379	0	0	0	A
9	BICF2P1236494	38199395	G	1	1	A
9	BICF2P558058	38246475	A	1	1	G
9	BICF2P1438677	38254985	A	1	1	C
9	BICF2P91650	38315178	A	1	1	G
9	BICF2P1181182	38318828	A	1	1	G
9	BICF2P334463	38333629	A	1	1	G
9	BICF2P1176750	38350203	A	1	1	G
9	BICF2G630836363	38365545	A	1	1	G
9	BICF2G630836389	38401831	G	0	0	A
9	BICF2G630836404	38417716	A	0	0	G
9	BICF2G630836412	38423943	G	0	0	A
9	BICF2G630836421	38443885	G	0	0	A
9	BICF2G630836452	38467001	G	0	0	A
9	BICF2G630836466	38478451	0	0	0	A
9	BICF2P377018	38486274	0	0	0	A
9	BICF2P623039	38503122	G	0.5	0.2143	A
9	BICF2P1186457	38508817	A	0.5	0.1667	G
9	BICF2G630836476	38512050	A	0.5	0.2143	G
9	BICF2G630836487	38527140	G	0	0	A
9	BICF2G630836491	38544278	0	0	0	C
9	BICF2G630836496	38556854	G	0	0	A
9	BICF2S2374339	38577770	G	0	0	A
9	BICF2G630836509	38583692	A	0	0	G

9	BICF2G630836514	38596178	A	0	0	G
9	BICF2G630836526	38610476	0	0	0	C
9	BICF2G630836547	38617962	G	0	0	A
9	BICF2G630836566	38624871	A	0	0	G
9	BICF2P697921	38627593	G	0.5	0.2143	A
9	BICF2G630836570	38627898	A	0	0	G
9	BICF2G630836574	38632075	A	0	0	G
9	BICF2P526484	38648522	A	0.5	0.2143	G
9	BICF2P1376797	38655873	A	0.5	0.7857	G
9	BICF2G630836601	38666161	A	0	0	G
9	BICF2G630836608	38673279	0	0	0	G
9	BICF2P1006689	38675549	A	0.5	0.7857	G
9	BICF2G630836616	38689308	C	0	0	A
9	BICF2G630836630	38706782	A	0	0	G
9	BICF2G630836640	38715967	A	0	0	G
9	BICF2P853239	38723347	G	0	0	A
9	BICF2G630836656	38744036	G	0	0	A
9	BICF2G630836674	38760820	A	0	0	G
9	BICF2G630836695	38778604	A	0.5	0.7857	G
9	BICF2S22937836	38794934	A	0	0	G
9	BICF2S23623508	38805564	A	0.5	0.2143	G
9	BICF2G630836714	38822973	A	0	0	C
9	BICF2G630836723	38844516	A	0	0	G
9	BICF2G630836726	38850859	A	0	0	G
9	BICF2G630836730	38858846	G	0	0	A
9	BICF2G630836734	38871920	0	0	0	G
9	BICF2P853269	38888906	A	0.5	0.7857	C
9	BICF2G630836736	38895898	A	0	0	G
9	BICF2G630836744	38909771	C	0	0	A
9	BICF2P199847	38924810	A	0	0	G
9	BICF2P1422473	38947642	0	0	0	A
9	BICF2G630836769	39003531	A	1	1	G
9	BICF2G630836792	39064200	G	1	1	A
9	BICF2S23345909	39066362	A	0	0	G
9	BICF2P1295921	39087110	C	0	0	G
9	BICF2G630836811	39106375	A	NA	1	G
9	BICF2G630836834	39143487	C	0.5	0.2143	A
9	BICF2G630836847	39161985	G	0	0	A
9	BICF2S2458527	39193696	0	0	0	A
9	BICF2G630836857	39211449	G	0	0	A
9	BICF2G630836862	39259632	0	0	0	G
9	BICF2S22964729	39263097	A	0	0	G
9	BICF2P878673	39288973	0	0	0	A
9	BICF2G630836866	39302475	G	1	1	C
9	BICF2P850542	39308353	A	1	1	G
9	BICF2G630836872	39323762	A	0	0	G
9	BICF2G630836876	39331800	A	1	1	C

9	BICF2P1338965	39347257	G	0	0	A
9	BICF2P1331734	39361603	C	0	0	A
9	BICF2G630836904	39382795	A	0	0	C
9	BICF2G630836919	39409015	G	0.5	0.7857	A
9	BICF2G630836929	39428473	A	NA	NA	G
9	G555f30S162	39457214	0	0	0	G
9	BICF2P1066151	39479097	G	0	0	A
9	BICF2G630836932	39482937	0	0	0	G
9	BICF2G630836939	39501193	0	0	0	C
9	BICF2G630836941	39505853	0	0	0	G
9	BICF2S23440486	39519312	G	0	0	C
9	BICF2G630836945	39541656	T	0	0	A
9	BICF2G630836958	39562421	A	0	0	G
9	TIGRP2P126305_rs8829062	39599430	0	0	0	C
9	BICF2G630836974	39623184	G	0	0	A
9	BICF2S23259973	39665562	G	0	0	A
9	BICF2G630837023	39678839	0	0	0	G
9	BICF2G630837052	39692469	A	0	0	G
9	BICF2S23321769	39765028	A	1	1	C
9	BICF2G630837109	39771733	A	1	1	G
9	BICF2G630837112	39785735	0	0	0	C
9	BICF2P1058910	39798504	C	NA	0	A
9	BICF2P246765	39804122	A	0	0	G
9	BICF2P686074	39813868	G	0.5	0.2143	A
9	BICF2G630837138	39817078	G	0.5	0.2143	A
9	BICF2G630837169	39836274	A	1	1	G
9	BICF2S23430015	39852464	A	0	0	C
9	BICF2G630837209	39864090	A	0	0	G
9	BICF2G630837233	39885922	A	0	0	C
9	BICF2G630837240	39900430	G	0	0	A
9	BICF2G630837260	39926171	G	1	1	A
9	BICF2S23910468	39933066	0	0	0	G
9	BICF2G630837271	39933873	G	0.5	0.2143	A
9	BICF2G630837296	39957193	G	0.5	0.2143	A
9	BICF2G630837298	39959109	A	0.5	0.2143	G
9	BICF2G630837302	39965412	A	0.5	0.2143	G
9	BICF2P827755	39985980	A	0	0	G
9	BICF2G630837307	40014683	A	0.5	0.7857	G
9	BICF2P389979	40030310	A	0.5	0.5	G
9	BICF2G630837347	40032989	G	0	0	C
9	BICF2G630837364	40043645	G	0.5	0.7857	A
9	BICF2G630837373	40084248	A	0.5	0.2143	G
9	BICF2G630837375	40091942	G	0	0	A
9	TIGRP2P126340_rs8998178	40106067	0	0	0	A
9	BICF2G630837390	40117301	G	0	0	A
9	BICF2P331413	40124915	0	0	0	G
9	BICF2G630837405	40157655	A	0.5	0.2143	G

9	BICF2P272135	40163460	G	0.5	0.2143	C
9	BICF2G630837411	40183858	0	0	0	G
9	BICF2G630837416	40194387	A	0	0	G
9	TIGRP2P126345_rs8763412	40207209	G	0.5	0.2143	A
9	BICF2P662113	40224394	A	0.5	0.7857	G
9	BICF2P311014	40228198	G	0.5	0.7857	A
9	BICF2G630837432	40244010	0	0	0	G
9	BICF2S23433032	40261194	G	0.5	0.7857	A
9	BICF2P1055130	40268330	G	0.5	0.7857	A
9	BICF2S23160635	40268515	A	0.5	0.7857	G
9	BICF2P131211	40282823	G	0	0	A
9	BICF2P121708	40291078	G	0.5	0.7857	A
9	TIGRP2P126366_rs8739331	40313799	A	0.5	0.7857	C
9	BICF2G630837435	40325187	0	0	0	C
9	TIGRP2P126369_rs8805380	40339239	G	0.5	0.7857	A
9	TIGRP2P126373_rs9017411	40345777	C	0	0	A
9	BICF2G630837436	40362001	0	0	0	A
9	BICF2S23910388	40366391	A	0.5	0.7857	G
9	BICF2P219872	40389398	G	0	0	A
9	BICF2P467674	40423768	G	0	0	C
9	BICF2G630837437	40439819	0	0	0	G
9	BICF2G630837438	40446015	0	0	0	A
9	BICF2G630837439	40484184	0	0	0	G
9	BICF2P767441	40500880	A	0	0	G
9	BICF2P727496	40551047	0	0	0	A
9	BICF2P859060	40561623	A	0	0	G
9	TIGRP2P126395_rs8905706	40587651	0	0	0	A
9	BICF2P952701	40598113	G	0.5	0.2143	A
9	BICF2G630837449	40619408	G	0.5	0.2143	A
9	BICF2G630837452	40632856	A	0	0	G
9	BICF2P853014	40642007	G	1	1	A
9	BICF2G630837490	40706663	A	0	0	G
9	BICF2S23438813	40732037	A	0.5	0.7857	G
9	BICF2G630837544	40750769	A	0	0	C
9	BICF2P852764	40774215	G	0	0	A
9	BICF2S2393657	40783215	0	0	0	C
9	BICF2S2364431	40793070	C	0	0	A
9	BICF2P750554	40816498	0	0	0	G
9	BICF2S23617077	40825619	G	0	0	C
9	BICF2G630837568	40839715	0	0	0	A
9	BICF2S23454483	40860219	C	0.5	0.7857	G
9	BICF2G630837570	40877332	G	0.5	0.7857	A
9	BICF2G630837620	40897405	0	0	0	G
9	BICF2G630837627	40907489	A	0	0	G
9	BICF2G630837636	40923849	0	0	0	C
9	BICF2G630837662	40934630	G	0.5	0.8333	A
9	BICF2G630837675	40943490	A	0.5	0.7857	G

9	BICF2G630837693	40950922	A	0.5	0.7857	G
9	TIGRP2P126430_rs9087986	40962727	G	0	0	C
9	BICF2P1282614	40971579	G	0	0	A
9	BICF2G630837739	40978211	A	0.5	0.2143	C
9	BICF2G630837761	40996143	G	0.5	0.2143	A
9	BICF2G630837762	41008824	G	0	0	A
9	BICF2G630837764	41013799	0	0	0	C
9	BICF2P233996	41024684	C	0	0	A
9	TIGRP2P126454_rs8973791	41043512	A	0.5	0.2143	G
9	TIGRP2P126468_rs9127184	41070083	A	0.5	0.2143	G
9	BICF2P393208	41079578	G	0.5	0.2143	A
9	BICF2P312237	41129838	A	0.5	0.7857	C
9	BICF2P83286	41139274	A	0.5	0.7857	G
9	BICF2P129144	41171348	G	0.5	0.2143	A
9	BICF2S23130058	41186888	0	0	0	G
9	BICF2G630837790	41198609	A	0.5	0.2143	G
9	BICF2G630837801	41207316	G	0	0	A
9	BICF2G630837818	41226690	A	0.5	0.2143	G
9	BICF2G630837821	41229413	C	0.5	0.2143	G
9	BICF2G630837829	41246540	0	0	0	G
9	BICF2G630837839	41253696	A	0	0	G
9	BICF2G630837855	41262649	G	0	0	A
9	BICF2G630837872	41276849	A	0	0	G
9	BICF2G630837884	41303210	A	0	0	G
9	BICF2G630837892	41314122	A	0	0	C
9	BICF2G630837904	41367726	0	0	0	C
9	BICF2P1281377	41389479	0	0	0	A
9	BICF2S23231243	41405593	A	0.5	0.2143	G
9	BICF2P1216333	41428388	A	0	0	G
9	BICF2G630837942	41428681	A	0	0	G
9	BICF2P1422641	41431713	A	0	0	G
9	BICF2P1422642	41431889	G	0	0	C
9	BICF2G630837953	41440011	C	1	1	A
9	BICF2G630837975	41446712	A	0	0	C
9	BICF2S22928352	41481366	G	1	1	A
9	BICF2S23124256	41492532	A	1	1	G
9	BICF2G630838059	41505381	G	1	1	A
9	BICF2G630838079	41545298	C	0	0	A
9	BICF2G630838083	41547795	A	0	0	G
9	BICF2G630838092	41554428	G	0	0	A
9	BICF2G630838110	41571729	A	0	0	G
9	BICF2P328422	41572615	A	0.5	0.2143	G
9	BICF2P859723	41582281	C	0.5	0.7857	A
9	BICF2P622463	41597515	C	0.5	0.2143	A
9	BICF2P556534	41618018	A	0.5	0.2143	G
9	BICF2G630838172	41639582	G	0	0	A
9	BICF2G630838177	41643223	A	1	1	C

9	BICF2P881331	41658394	G	0.5	0.2143	C
9	BICF2G630838184	41665729	A	0.5	0.2143	G
9	BICF2S23115623	41691942	A	0	0	G
9	BICF2G630838231	41700327	A	0	0	G
9	BICF2G630838240	41712816	A	0	0	G
9	BICF2G630838275	41728462	A	0	0	G
9	BICF2G630838282	41744787	A	0.5	0.2143	G
9	BICF2G630838288	41758248	G	0	0	A
9	BICF2P881974	41788056	A	0	0	C
9	BICF2S23538895	41793590	G	0	0	A
9	BICF2P964411	41824296	G	0.5	0.2143	A
9	BICF2P1220081	41841130	A	0.5	0.2143	T
9	BICF2P161638	41851387	G	0	0	A
9	TIGRP2P126602_rs8585073	41870667	A	0	0	G
9	BICF2P788458	41872475	0	0	0	G
9	BICF2P802073	41885908	C	0.5	0.2143	A
9	BICF2P194893	41890478	A	0	0	C
9	BICF2S23250176	41913693	A	0	0	G
9	BICF2G630838330	41918483	A	0.5	0.2143	C
9	BICF2P644961	41927310	A	0	0	G
9	BICF2G630838360	41932585	0	0	0	G
9	BICF2P576730	41942181	G	0.5	0.7857	A
9	BICF2P1361877	41957487	0	0	0	A
9	BICF2S23553192	41965055	C	0.5	0.7857	A
9	BICF2G630838398	41987315	G	0	0	A
9	BICF2G630838424	42004664	A	0	0	G
9	BICF2P1218221	42011882	A	0	0	G
9	BICF2G630838447	42031159	A	0	0	G
9	BICF2G630838458	42039830	A	0	0	G
9	BICF2G630838470	42053169	A	0.5	0.2143	G
9	BICF2P1274731	42076217	G	1	1	A
9	BICF2S2374017	42094763	G	0.5	0.7857	A
9	BICF2G630838546	42107642	C	0.5	0.2143	G
9	BICF2G630838556	42118031	G	0.5	0.2143	A
9	BICF2G630838576	42130151	G	0.5	0.2143	A
9	BICF2G630838603	42148194	A	0.5	0.2143	G
9	BICF2P1194769	42160470	A	0.5	0.2143	G
9	BICF2P579744	42166652	G	0.5	0.7857	A
9	BICF2P373213	42187768	G	0	0	A
9	TIGRP2P126693_rs8537022	42211427	A	0	0	G
9	BICF2P1186247	42218794	A	0	0	C
9	BICF2G630838622	42229332	G	0	0	A
9	BICF2G630838658	42252678	G	0	0	A
9	BICF2G630838676	42265776	G	1	1	A
9	BICF2G630838689	42277182	C	0.5	0.2143	A
9	BICF2S2335873	42295276	A	0.5	0.7857	G
9	BICF2P693363	42301086	A	0.5	0.5	G

9	BICF2S2376764	42310355	0	0	0	G
9	BICF2G630838716	42342951	G	0.5	0.7857	A
9	BICF2G630838718	42345640	A	0	0	T
9	BICF2G630838781	42369429	A	0	0	G
9	TIGRP2P126728_rs8702024	42380843	A	1	1	C
9	BICF2G630838830	42402742	G	0	0	A
9	BICF2S23260613	42412870	A	0	0	G
9	TIGRP2P126746_rs8710412	42427986	A	0.5	0.2143	G
9	BICF2G630838835	42444227	G	0.5	0.2143	A
9	BICF2G630838837	42446534	A	0.5	0.2143	C
9	BICF2G630838852	42469107	A	0.5	0.7857	G
9	BICF2P517968	42476490	A	0	0	T
9	BICF2P517964	42477901	A	1	1	G
9	BICF2P870540	42484272	G	0	0	A
9	BICF2G630838863	42501399	G	0.5	0.7857	A
9	BICF2G630838882	42512838	G	0.5	0.7857	A
9	BICF2G630838901	42522779	A	0.5	0.7857	G
9	BICF2G630838912	42531518	G	0.5	0.2143	C
9	BICF2G630838941	42538927	G	0.5	0.7857	A
9	BICF2G630838984	42550682	A	0	0	G
9	BICF2P180074	42568517	C	0	0	A
9	BICF2G630839031	42573347	A	0	0	G
9	BICF2G630839100	42606201	C	0	0	A
9	BICF2P1252022	42606957	A	1	1	G
9	BICF2G630839105	42608157	A	1	1	G
9	BICF2G630839159	42639862	G	0	0	C
9	BICF2G630839194	42658261	G	0.5	0.2857	A
9	BICF2G630839218	42671140	A	0.5	0.7143	G
9	BICF2G630839219	42671512	A	0.5	0.7143	G
9	BICF2G630839226	42676805	C	0	0	A
9	BICF2P794852	42688735	G	0	0	C
9	BICF2P1075352	42707716	G	0.5	0.7143	A
9	BICF2P1257658	42716246	T	0	0	A
9	BICF2G630839251	42737473	C	0	0	A
9	BICF2G630839274	42741516	A	0	0	G
9	BICF2G630839327	42758623	A	0	0	G
9	TIGRP2P126829_rs9107732	42770944	A	0.5	0.2857	G
9	BICF2G630839352	42784190	G	0	0	A
9	BICF2G630839389	42812218	A	0	0	G
9	BICF2G630839433	42840299	A	0	0	G
9	BICF2G630839452	42851521	C	1	1	G
9	TIGRP2P126867_rs8989669	42861624	0	0	0	C
9	BICF2P15624	42873333	A	0.5	0.7143	G
9	BICF2G630839516	42903201	A	0.5	0.7143	G
9	BICF2G630839542	42916473	C	0.5	0.2857	A
9	TIGRP2P126881_rs9017672	42927780	A	0	0	G
9	BICF2G630839571	42929679	A	0.5	0.7143	G

9	BICF2G630839587	42937781	C	0	0	A
9	BICF2P265497	42949506	A	0.5	0.2857	G
9	BICF2G630839639	42960027	A	0.5	0.7143	C
9	BICF2G630839652	42964929	A	0.5	0.7143	G
9	TIGRP2P126909_rs9031587	42996586	A	0.5	0.2857	G
9	BICF2G630839689	43020797	A	0	0	G
9	BICF2G630839707	43027145	A	0	0	G
9	BICF2G630839723	43036108	G	0	0	A
9	BICF2G630839742	43054820	A	0.5	0.7143	G
9	BICF2G630839753	43062456	A	0.5	0.7143	G
9	BICF2P900122	43072411	G	0	0	A
9	BICF2G630839764	43072729	A	0	0	C
9	BICF2P450914	43077556	A	0	0	G
9	BICF2P1207780	43083141	A	0	0	G
9	BICF2P797094	43090781	A	0	0	G
9	BICF2G630839818	43111058	G	0	0	A
9	BICF2P554982	43133661	G	0.5	0.7143	C
9	BICF2G630839841	43141432	G	0	0	C
9	BICF2G630839844	43151320	A	0	0	G
9	BICF2G630839863	43166376	A	0	0	G
9	BICF2G630839892	43184511	C	0	0	A
9	BICF2G630839897	43189706	G	0.5	0.2857	A
9	BICF2G630839902	43196615	C	0.5	0.7143	A
9	BICF2G630839920	43207224	A	0.5	0.2857	G
9	TIGRP2P126938_rs8783164	43255503	A	0.5	0.2857	G
9	BICF2G630839950	43266995	A	0	0	G
9	BICF2G630839964	43278584	A	0.5	0.7143	G
9	BICF2G630839972	43289065	A	0.5	0.7143	C
9	BICF2P252311	43292661	A	0	0	G
9	BICF2S23060746	43295035	A	0	0	G
9	BICF2S23741495	43303864	A	0.5	0.7143	G
9	BICF2P224662	43307672	A	0	0	G
9	BICF2P1339649	43340433	G	1	1	A
9	BICF2P689336	43346763	A	0	0	G
9	BICF2S2356413	43364529	C	0	0	A
9	BICF2P435886	43374747	A	0.5	0.2857	G
9	BICF2P414872	43381514	A	0.5	0.7143	G
9	BICF2P191033	43408253	A	0	0	G
9	BICF2P1216677	43417587	G	0	0	A
9	TIGRP2P126995_rs8509709	43428999	C	0	0	G
9	TIGRP2P126998_rs8677925	43441714	G	0	0	A
9	TIGRP2P127000_rs8714608	43457106	C	0.5	0.2857	A
9	BICF2P1129853	43459007	G	0.5	0.2857	A
9	TIGRP2P127005_rs8880175	43473727	0	0	0	C
9	BICF2P242958	43484020	G	0	0	A
9	TIGRP2P127016_rs9016932	43494535	G	0	0	A
9	BICF2P161883	43513695	A	0.5	0.2857	G

9	TIGRP2P127027_rs8669574	43516731	A	0	0	G
9	BICF2P756102	43544947	G	0	0	A
9	BICF2P37943	43546689	A	0	0	G
9	BICF2G630839994	43558215	C	0.5	0.2857	A
9	BICF2G630839997	43560192	A	0.5	0.25	G
9	BICF2G630840002	43564331	G	0.5	0.2857	A
9	BICF2P698732	43576885	G	0	0	A
9	TIGRP2P127058_rs9151421	43592567	0	0	0	A
9	BICF2G630840007	43597173	A	0	0	C
9	TIGRP2P127065_rs8948429	43611224	0	0	0	G
9	TIGRP2P127079_rs8900577	43627716	0	0	0	G
9	TIGRP2P127088_rs9109497	43635732	A	0.5	0.7143	C
9	BICF2P693064	43643712	A	0	0	G
9	BICF2S23120296	43673656	T	0.5	0.7143	A
9	BICF2P1366169	43678284	A	0	0	G
9	BICF2P1450497	43710073	G	0	0	A
9	BICF2P84212	43715415	G	0.5	0.7143	A
9	TIGRP2P127123_rs9019934	43732465	A	0	0	G
9	TIGRP2P127126_rs8869602	43735960	A	0.5	0.7143	G
9	BICF2G630840017	43752988	A	0.5	0.7143	C
9	TIGRP2P127128_rs8521929	43759163	G	0	0	A
9	BICF2G630840029	43772831	A	0.5	0.7143	G
9	BICF2G630840034	43783501	A	0.5	0.7143	C
9	BICF2G630840040	43794598	A	0.5	0.7143	G
9	BICF2G630840052	43804743	G	0	0	A
9	BICF2G630840078	43819094	A	0.5	0.2857	G
9	BICF2G630840081	43826500	G	0.5	0.2857	A
9	TIGRP2P127134_rs9030883	43838072	G	0.5	0.2857	A
9	BICF2S23613504	43887050	A	0	0	C
9	BICF2S2451720	43898593	A	0	0	G
9	BICF2P1366777	43936492	G	0	0	A
9	BICF2P1317284	43940252	G	0	0	A
9	TIGRP2P127139_rs8597996	43944769	A	0	0	G
9	BICF2G630840113	43953421	G	0.5	0.2857	A
9	BICF2G630840139	43986408	G	0	0	A
9	BICF2G630840151	43991642	A	0	0	G
9	BICF2G630840176	44006311	A	0.5	0.7143	G
9	BICF2G630840185	44011151	G	0.5	0.7143	A
9	BICF2G630840198	44022639	A	0.5	0.7143	G
9	BICF2G630840234	44040392	G	0.5	0.2857	A
9	BICF2G630840237	44045206	C	0.5	0.2857	A
9	BICF2G630840256	44059580	C	0.5	0.7143	A
9	BICF2P913662	44071845	0	0	0	A
9	BICF2G630840282	44089313	A	0.5	0.2857	G
9	TIGRP2P127178_rs8909269	44107754	C	0.5	0.5	A
9	BICF2G630840297	44130900	A	0.5	0.7143	G
9	BICF2G630840325	44149022	A	0.5	0.7143	G

9	BICF2G630840334	44164362	A	1	1	G
9	BICF2P569184	44186576	A	0	0	G
9	BICF2P325054	44203449	G	1	1	A
9	TIGRP2P127195_rs9055793	44211454	0	0	0	G
9	BICF2G630840349	44238305	G	0.5	0.2857	A
9	TIGRP2P127198_rs9030833	44241217	G	0	0	A
9	BICF2P1426690	44246638	G	0.5	0.2857	A
9	BICF2S23217	44256095	C	0	0	A
9	BICF2P545967	44266639	0	0	0	A
9	BICF2G630840370	44289750	C	0	0	A
9	BICF2G630840379	44308968	A	0	0	G
9	BICF2G630840389	44314279	G	0.5	0.7143	A
9	BICF2P240339	44340131	0	0	0	A
9	BICF2P660166	44344699	A	0.5	0.7143	G
9	BICF2G630840398	44362686	C	0.5	0.2857	A
9	BICF2G630840400	44378118	A	0.5	0.2857	G
9	BICF2G630840405	44384643	G	1	1	A
9	BICF2G630840429	44397880	A	1	1	C
9	BICF2G630840438	44405219	A	0	0	G
9	BICF2G630840450	44422298	C	0.5	0.7143	G
9	BICF2P915961	44435808	A	0	0	G
9	TIGRP2P127247_rs8785481	44443750	G	0	0	A
9	BICF2G630840468	44447589	A	1	1	C
9	BICF2G630840492	44477569	A	0.5	0.7143	G
9	BICF2G630840514	44489698	G	0.5	0.2857	A
9	BICF2G630840515	44489841	G	0.5	0.2857	C
9	TIGRP2P127266_rs8654074	44499447	A	0	0	G
9	BICF2G630840534	44505568	A	1	1	G
9	BICF2G630840550	44522489	A	0.5	0.2857	G
9	BICF2G630840572	44547106	G	1	1	A
9	BICF2G630840588	44561185	G	1	1	C
9	BICF2G630840604	44568073	C	0.5	0.2857	A
9	BICF2G630840618	44584498	G	1	1	A
9	BICF2G630840621	44587699	A	0.5	0.2857	G
9	BICF2G630840640	44607475	G	0.5	0.2857	A
9	BICF2P1371664	44611697	A	0.5	0.2857	G
9	BICF2G630840645	44622583	G	1	1	A
9	BICF2P1224292	44631774	G	0	0	A
9	BICF2S23255440	44647395	A	0	0	G
9	BICF2P1049044	44666177	A	0.5	0.7143	G
9	BICF2G630840701	44666435	G	0.5	0.7143	A
9	BICF2G630840711	44685451	A	0	0	G
9	BICF2G630840728	44698268	G	0	0	A
9	BICF2G630840734	44701573	A	0	0	G
9	BICF2P1445775	44720207	A	1	1	G
9	TIGRP2P127321_rs8507343	44720833	C	1	1	G
9	BICF2P927393	44734215	G	0.5	0.2857	A

9	BICF2G630840760	44739749	G	0.5	0.2857	A
9	BICF2P946787	44747565	G	0.5	0.2857	A
9	BICF2P855555	44759247	G	0	0	A
9	BICF2G630840809	44773025	G	0	0	C
9	TIGRP2P127362_rs8936418	44782966	0	0	0	G
9	BICF2G630840822	44785341	G	0.5	0.2857	A
9	BICF2G630840832	44793043	A	0	0	G
9	BICF2G630840846	44807889	A	0.5	0.2857	G
9	BICF2G630840851	44816438	A	0	0	G
9	BICF2P692691	44829043	T	0.5	0.2857	A
9	BICF2G630840881	44841179	G	0	0	A
9	BICF2P1282751	44851038	0	0	0	A
9	TIGRP2P127382_rs8963208	44862366	0	0	0	G
9	BICF2G630840915	44874898	G	0	0	A
9	BICF2G630840932	44891878	G	0.5	0.2857	A
9	TIGRP2P127394_rs8839957	44896274	A	0.5	0.2857	G
9	BICF2G630840939	44900430	A	0	0	G
9	BICF2G630840944	44912888	G	0	0	A
9	BICF2G630840947	44927746	A	0	0	G
9	BICF2G630840956	44942663	A	0.5	0.2857	C
9	BICF2G630840985	44962528	G	0	0	A
9	BICF2G630840988	44969122	A	0	0	G
9	BICF2G630840996	44983516	C	0	0	A
9	TIGRP2P127404_rs9161802	44993994	0	0	0	G
9	BICF2G630841000	45006901	G	0.5	0.25	A
9	BICF2G630841011	45021034	C	0	0	A
9	BICF2G630841013	45023238	G	0	0	A
9	BICF2P414366	45043483	G	0	0	A
9	BICF2G630841038	45054204	G	0	0	A
9	BICF2G630841040	45057992	G	0.5	0.2857	A
9	BICF2G630841053	45091259	G	0.5	0.2857	A
9	BICF2P1229877	45095707	G	0	0	C
9	BICF2G630841063	45102924	A	0.5	0.2857	G
9	BICF2G630841070	45111938	A	1	1	G
9	BICF2P825921	45134306	0	0	0	G
9	BICF2P737374	45148023	G	0	0	A
9	TIGRP2P127418_rs9034938	45161899	A	0	0	G
9	TIGRP2P127420_rs8784160	45173685	G	0	0	A
9	TIGRP2P127429_rs8810777	45188905	A	0.5	0.7143	G
9	BICF2P624756	45197605	A	0	0	G
9	BICF2G630841105	45208941	G	0.5	0.7143	A
9	BICF2G630841127	45223896	A	0.5	0.2857	C
9	BICF2P1137227	45231825	0	0	0	G
9	BICF2P120779	45245750	G	0	0	A
9	BICF2P600834	45260720	A	0	0	G
9	BICF2G630841155	45264691	G	0.5	0.7143	C
9	BICF2P1187317	45276074	A	0	0	G

9	TIGRP2P127469_rs9121610	45310669	A	0.5	0.7143	G
9	BICF2S22923141	45324913	G	0.5	0.2857	A
9	BICF2P380718	45336781	A	0.5	0.2857	G
9	BICF2P65976	45344102	A	0.5	0.2857	T
9	BICF2P65972	45345282	A	0	0	G
9	BICF2S23019540	45365293	A	0	0	G
9	BICF2P733656	45368550	G	0	0	A
9	TIGRP2P127490_rs9071362	45376390	G	1	1	A
9	BICF2P13707	45383599	G	0.5	0.2857	A
9	BICF2P94165	45384687	A	0	0	G
9	TIGRP2P127497_rs8831775	45390632	A	0	0	G
9	BICF2P725474	45402585	A	0.5	0.2857	G
9	BICF2P862310	45424747	A	1	1	G
9	BICF2S22946090	45425505	A	0	0	G
9	TIGRP2P127515_rs8712849	45441235	A	0	0	C
9	BICF2S23442356	45451780	G	0.5	0.7143	C
9	TIGRP2P127519_rs9211495	45461737	A	0.5	0.2857	G
9	BICF2P342940	45479647	A	0.5	0.7143	G
9	BICF2P665540	45491057	T	0	0	A
9	BICF2P1124202	45496884	G	0.5	0.7143	A
9	TIGRP2P127531_rs9039390	45505720	0	0	0	G
9	BICF2S22930410	45520019	G	0.5	0.2857	A
9	BICF2S23536916	45533037	A	0	0	G
9	BICF2S22925902	45544335	A	1	1	C
9	BICF2P894953	45554038	A	0	0	G
9	BICF2S23719309	45574296	C	0	0	A
9	BICF2P1106907	45591744	G	0	0	A
9	BICF2P137419	45613861	A	0.5	0.7143	G
9	BICF2P469265	45623036	G	0.5	0.2857	A
9	BICF2P844816	45651555	A	0.5	0.2857	G
9	BICF2S23424205	45668492	G	0	0	A
9	TIGRP2P127602_rs8884419	45706499	G	0	0	A
9	BICF2S23410227	45713167	A	0	0	G
9	BICF2P334155	45730167	G	0.5	0.7143	A
9	BICF2P49040	45739536	A	0.5	0.7143	G
9	BICF2P767195	45748369	G	0.5	0.7143	A
9	BICF2P721433	45759088	A	0.5	0.7143	C
9	BICF2P593523	45778021	A	0.5	0.7143	G
9	BICF2P72804	45792953	G	0.5	0.7143	A
9	BICF2S23148443	45818032	G	0	0	A
9	BICF2P1141278	45838153	A	0.5	0.7143	G
9	BICF2P965484	45852556	A	0.5	0.2857	G
9	BICF2P1253185	45883160	A	0	0	G
9	TIGRP2P127666_rs8637233	45895323	A	0	0	G
9	BICF2P1230497	45901455	A	0.5	0.5	G
9	BICF2S23143335	45904552	T	0.5	0.2857	A
9	BICF2P647646	45919418	G	0.5	0.7143	A

9	BICF2P321869	45922456	G	0.5	0.7143	C
9	BICF2P617533	45925051	G	0.5	0.7143	A
9	BICF2P380084	45931936	A	0	0	G
9	BICF2S23115519	45949273	A	0	0	G
9	BICF2P355682	45961936	G	0	0	A
9	BICF2P978567	45971110	G	0	0	A
9	BICF2P964767	46011581	A	0	0.2143	G
9	BICF2S23450909	46032307	G	0	0	A
9	TIGRP2P127744_rs9023558	46036398	G	1	0.7857	A
9	BICF2P814146	46055233	A	0	0	G
9	TIGRP2P127780_rs8888636	46057128	G	1	0.7857	A
9	BICF2P590225	46067638	G	0	0.2143	A
9	BICF2P590229	46068119	A	0	0.2143	G
9	BICF2P612988	46072993	A	0	0.2143	C
9	BICF2P33514	46100559	A	0	0	G
9	BICF2S23741213	46129353	G	0	0.2143	A
9	BICF2P1375165	46139608	0	0	0	G
9	TIGRP2P127791_rs8547059	46172387	A	1	0.7857	G
9	BICF2P139278	46174723	G	0	0	A
9	TIGRP2P127797_rs8576695	46186132	0	0	0	G
9	TIGRP2P127798_rs9044431	46207594	A	0	0	G
9	BICF2S2396711	46220789	0	0	0	T
9	BICF2P550349	46239022	T	1	0.7857	A
9	BICF2P18846	46249001	0	0	0	C
9	TIGRP2P127807_rs8558394	46281528	0	0	0	C
9	BICF2P188476	46289798	0	0	0	C
9	BICF2P180563	46332742	A	0	0	G
9	BICF2S23334035	46343464	A	1	0.7857	T
9	BICF2P802688	46352551	A	0	0	G
9	BICF2S23316986	46378997	A	1	1	G
9	BICF2S2339560	46381784	G	1	1	A
9	BICF2P1176231	46413781	0	0	0	C
9	BICF2S23153684	46419991	A	0	0	C
9	BICF2S23130171	46429175	A	0	0	G
9	TIGRP2P127819_rs8782478	46444973	G	0	0	A
9	TIGRP2P127820_rs8782477	46446923	A	0	0	C
9	TIGRP2P127822_rs9179880	46451952	0	0	0	C
9	BICF2P579086	46465548	A	0	0	G
9	BICF2P1056746	46481284	A	0	0	G
9	BICF2P368699	46487181	C	0	0	A
9	BICF2S2337002	46498504	G	0	0	A
9	BICF2P157199	46516033	G	0	0	A
9	BICF2P153680	46525644	G	0	0	A
9	BICF2P1110461	46531115	A	0	0	G
9	BICF2P1383032	46551817	A	0	0	G
9	TIGRP2P127863_rs8684086	46560126	A	0	0	G
9	BICF2P890521	46566090	C	0	0	A

9	BICF2P1312330	46587357	G	0	0	A
9	BICF2P967928	46600714	0	0	0	G
9	BICF2S23053902	46615925	C	0	0	A
9	TIGRP2P127871_rs9039784	46630795	A	0	0	G
9	BICF2P62126	46637389	A	0	0	G
9	BICF2P505380	46644882	G	0	0	A
9	BICF2P456138	46655758	A	0	0	G
9	BICF2P36016	46673812	G	0	0	A
9	BICF2P864768	46699066	A	1	1	C
9	TIGRP2P127900_rs9139238	46701646	A	0	0.2143	C
9	BICF2P697377	46727894	A	1	0.7857	G
9	BICF2P128507	46749952	0	0	0	C
9	TIGRP2P127909_rs8789645	46804560	A	0	0.2143	G
9	BICF2G630841159	46814845	0	0	0	A
9	BICF2P31211	46829263	G	0	0	A
9	TIGRP2P127922_rs8723155	46881147	G	0	0	A
9	BICF2P504721	46889704	0	0	0	T
9	BICF2P1340419	46932981	C	0	0.2143	A
9	BICF2P1359703	46943938	A	0	0	G
9	BICF2P1302170	46962268	A	0	0.2143	G
9	BICF2P325180	46966619	G	0	0.2143	A
9	BICF2P541408	46978538	C	0	0	A
9	BICF2P622818	46990511	A	0	0.2143	G
9	BICF2P130367	47001398	0	0	0	G
9	BICF2P1036331	47044682	A	0	0	G
9	BICF2S23536976	47049676	G	1	0.7857	A
9	BICF2S23018060	47058675	A	1	0.7857	G
9	BICF2P475938	47093659	G	0	0	A
9	BICF2P1125476	47109843	G	0	0	A
9	BICF2P197688	47124904	0	0	0	A
9	BICF2S23632135	47132755	0	0	0	A
9	BICF2P1093646	47143151	C	0	0.2143	A
9	TIGRP2P127956_rs8637929	47170815	G	0	0.2143	A
9	BICF2P360616	47175135	0	0	0	A
9	BICF2P1142549	47192696	0	0	0	G
9	BICF2P44507	47204223	C	0	0	A
9	BICF2P718061	47209434	A	0	0	G
9	TIGRP2P127964_rs8536728	47225913	G	0	0.2143	A
9	BICF2P674759	47244696	G	0	0	A
9	BICF2P1311630	47260776	A	0	0.2143	G
9	BICF2P989960	47266914	A	0	0.2143	G
9	TIGRP2P127978_rs8968034	47286743	G	0	0.2143	A
9	BICF2P1079200	47290004	G	0	0.2143	A
9	BICF2P895346	47315276	G	0	0.2143	A
9	BICF2P142242	47343037	A	0	0.1667	G
9	BICF2P587823	47353825	T	0	0.1	A
9	BICF2P266094	47357182	G	0	0	A

9	BICF2P902495	47369293	G	0	0.2143	A
9	BICF2P19842	47381991	A	0	0.1667	C
9	BICF2S23519513	47394623	0	0	0	A
9	BICF2P509129	47429947	A	0	0.1667	G
9	BICF2P1249988	47445878	C	0	0.2143	A
9	BICF2P676084	47460046	G	0	0.2143	A
9	BICF2P1302650	47469883	G	0	0.1667	A
9	BICF2P120798	47497048	0	0	0	G
9	BICF2S2356641	47540847	A	1	1	G
9	BICF2P340934	47542348	G	1	1	A
9	BICF2P614584	47560435	A	1	1	C
9	BICF2P1308700	47565182	0	0	0	A
9	TIGRP2P128006_rs8319569	47582781	G	0	0.2143	A
9	BICF2P1468871	47589418	G	1	0.7857	A
9	TIGRP2P128013_rs8567281	47600720	G	1	0.7857	A
9	BICF2P228007	47620443	G	1	0.7857	A
9	BICF2P1201382	47624597	A	1	0.7857	G
9	BICF2P1229648	47639442	C	1	0.7857	A
9	BICF2P228029	47647011	A	1	0.7857	G
9	BICF2P341331	47659782	A	1	0.7857	C
9	BICF2P1402053	47668055	0	0	0	C
9	BICF2P18608	47682618	G	1	0.7857	A
9	BICF2S23728304	47694098	G	0	0.2143	A
9	TIGRP2P128023_rs8694773	47702918	A	0	0	G
9	BICF2P653362	47715201	C	0	0.2143	G
9	TIGRP2P128027_rs8510124	47725275	G	0	0.2143	A
9	BICF2P330608	47747184	A	0	0.2143	G
9	TIGRP2P128042_rs9168995	47749228	C	0	0.2143	A
9	BICF2P895948	47761049	0	0	0	A
9	BICF2S23116825	47776324	A	0	0	G
9	BICF2S23124809	47793546	A	0	0.2143	G
9	BICF2P435526	47806347	0	0	0	A
9	BICF2P1168261	47831552	A	0	0	G
9	BICF2P690332	47859309	A	0	0	G
9	BICF2P950384	47877758	0	0	0	G
9	BICF2P1440373	47886612	0	0	0	C
9	TIGRP2P128100_rs8990684	47906887	0	0	0	A
9	TIGRP2P128102_rs8982620	47909735	G	0	0	A
9	BICF2S23649207	47927769	G	0	0.1667	A
9	BICF2P794828	47936419	A	1	1	C
9	BICF2S23231135	47948899	A	0	0	G
9	TIGRP2P128115_rs9090434	47958382	G	0	0.2143	A
9	BICF2P1413286	47976591	0	0	0	G
9	BICF2P1392974	47979923	A	0	0	G
9	BICF2P926885	48001409	A	0	0	G
9	BICF2P945643	48014174	0	0	0	C
9	BICF2S2334280	48027810	G	1	0.7857	A

9	BICF2P57249	48056026	G	0	0	A
9	BICF2P867356	48060584	A	0	0	G
9	BICF2P1002363	48080546	G	1	1	A
9	BICF2S2347312	48086539	A	1	0.7857	C
9	TIGRP2P128141_rs9043320	48093469	G	0	0.2143	A
9	TIGRP2P128146_rs8563561	48109024	A	0	0	G
9	BICF2P1249802	48130630	A	0	0	G
9	BICF2S23154457	48133320	A	0	0	G
9	BICF2P1292584	48140428	C	0	0.2143	A
9	TIGRP2P128165_rs8958318	48150758	G	0	0	A
9	TIGRP2P128178_rs9047851	48170387	G	0	0.2143	A
9	BICF2P470855	48181555	A	0	0.2143	G
9	BICF2P156825	48195161	A	1	1	G
9	BICF2S23134023	48197616	G	1	1	A
9	BICF2P264146	48211596	G	0	0.2143	A
9	BICF2P644480	48229641	G	0	0.2143	A
9	BICF2S22941520	48232263	A	0	0	G
9	BICF2S23235526	48251808	G	1	0.7857	A
9	BICF2S23141984	48259329	C	1	1	A
9	BICF2P1456440	48276070	G	0	0.2143	A
9	BICF2P964029	48287228	G	0	0	A
9	BICF2P822569	48294789	A	0	0	C
9	BICF2S23728955	48305105	G	0	0	A
9	BICF2S232939	48316483	A	0	0	G
9	BICF2P693938	48325344	A	0	0.2143	G
9	TIGRP2P128227_rs9001072	48334803	A	0	0.2143	G
9	BICF2P798834	48351258	A	0	0	G
9	BICF2P577580	48369772	G	1	0.7857	A
9	BICF2P244156	48382392	C	1	0.7857	A
9	BICF2P1293972	48393204	A	0	0	C
9	BICF2P552922	48411915	A	1	0.7857	G
9	BICF2S23127077	48418528	A	1	0.7857	G
9	BICF2P932247	48435397	A	0	0.2143	C
9	BICF2P1328404	48452998	A	0	0.2143	G
9	BICF2S22923291	48478119	C	1	0.7857	A
9	BICF2P1202365	48493146	A	0	0.2143	C
9	BICF2S23246952	48507443	0	0	0	A
9	BICF2S23218329	48536412	G	1	0.7857	A
9	BICF2P640660	48542795	C	0	0.2143	G
9	BICF2P542053	48550998	G	0	0.2143	A
9	BICF2P877447	48554219	C	0	0	A
9	BICF2P474924	48580058	G	0.5	0.9286	A
9	BICF2P1109444	48598683	A	0.5	0.9286	G
9	BICF2P1109446	48599760	G	0.5	0.7143	A
9	BICF2P1163507	48623211	G	0.5	0.9286	A
9	BICF2S22942616	48636545	A	0	0	G
9	BICF2P1068851	48646932	A	0	0	G

9	BICF2S23332673	48659367	G	0	0.2143	A
9	BICF2P797392	48671086	A	0	0.2143	C
9	BICF2P225368	48683255	A	0	0.2143	G
9	BICF2P527171	48690382	G	0	0.2143	A
9	BICF2P438859	48701047	G	0	0.2143	A
9	BICF2P893381	48705405	A	0	0.2143	G
9	TIGRP2P128267_rs8519659	48721171	A	0	0	G
9	TIGRP2P128273_rs9236038	48733796	G	0	0.2143	A
9	BICF2P1118525	48737996	A	0	0	G
9	BICF2P837261	48753345	A	1	0.7857	G
9	TIGRP2P128297_rs8917036	48770158	A	1	1	G
9	BICF2P784482	48773668	C	0	0	A
9	BICF2P787226	48783692	A	0	0	G
9	G558f21S142	48808375	G	0	0	A
9	BICF2S2337209	48838821	G	0	0.2143	A
9	BICF2P877383	48845444	A	0	0.2143	G
9	TIGRP2P128318_rs8721087	48886154	0	0	0	A
9	BICF2P276821	48887465	A	0	0.2143	G
9	BICF2P877163	48899018	A	0	0	T
9	G559f29S224	48926954	0	0	0	C
9	BICF2S23344580	48954611	A	0.5	0.07143	C
9	TIGRP2P128326_rs8980333	48962237	0	0	0	C
9	BICF2S23341616	48966803	A	0	0.2143	G
9	BICF2P115287	48977922	C	0.5	0.2857	A
9	BICF2S2378384	48988101	A	0	0.2143	G
9	BICF2S2303613	48993247	G	0	0.2143	A
9	BICF2P308389	49031740	G	0	0	A
9	BICF2P1023003	49035645	A	0	0	G
9	BICF2P658015	49044029	G	0	0.2143	A
9	BICF2P717103	49053754	C	0	0	A
9	TIGRP2P128339_rs8764641	49067990	A	0	0	C
9	BICF2P138084	49091781	G	0.5	0.7143	A
9	BICF2S23550798	49096465	C	0	0	A
9	BICF2P250176	49112809	G	0	0.2143	C
9	BICF2P481590	49128847	0	0	0	A
9	BICF2P1224168	49149842	A	0.5	0.7143	C
9	TIGRP2P128352_rs8884996	49156971	0	0	0	G
9	BICF2S23349985	49171051	A	0	0	G
9	BICF2P744503	49178694	A	0	0	G
9	BICF2P541694	49202776	0	0	0	G
9	BICF2P86867	49239115	0	0	0	A
9	BICF2P7328	49247235	0	0	0	A
9	BICF2P677632	49297354	0	0	0	C
9	BICF2P734518	49302016	0	NA	NA	G
9	BICF2P602822	49318929	0	0	0	G
9	BICF2P668409	49366371	A	0.5	0.07143	G
9	BICF2S23750594	49371822	G	1	1	A

9	BICF2S2344961	49382494	G	0	0	A
9	BICF2S22944553	49393822	A	0.5	0.07143	G
9	BICF2S22930300	49421872	A	1	1	G
9	TIGRP2P128371_rs8770677	49432005	A	0.5	0.9286	G
9	BICF2P1215354	49438586	A	0.5	0.9286	G
9	TIGRP2P128377_rs9102944	49457418	A	1	0.7857	G
9	BICF2S23513129	49470584	G	0.5	0.07143	A
9	BICF2P1128643	49476981	0	0	0	A
9	BICF2P1135331	49489019	C	0.5	0.75	A
9	BICF2P1082893	49496520	G	0.5	1	A
9	BICF2P743857	49511539	G	0.5	0.7143	A
9	BICF2P1019720	49527923	A	0	0	G
9	BICF2S23510795	49563673	A	0.5	0.7143	G
9	BICF2S23245733	49576999	A	0.5	0.9286	G
9	BICF2P118011	49601496	0	0	0	A
9	BICF2P213044	49651659	G	0.5	0.7143	A
9	BICF2S2297178	49677957	G	0.5	0.9286	A
9	BICF2P1157410	49681685	G	0	0	A
9	TIGRP2P128417_rs8794272	49697706	G	0	0	A
9	TIGRP2P128422_rs9167389	49721325	G	0	0	A
9	TIGRP2P128426_rs8798945	49750472	0	0	0	G
9	BICF2P652148	49764978	G	0	0	A
9	BICF2P652147	49765017	A	0	0	G
9	TIGRP2P128430_rs8952663	49812433	G	1	0.7857	A
9	BICF2P813102	49828323	A	0	0.2143	C
9	BICF2P870510	49840790	G	0	0.2143	A
9	BICF2S2458144	49847373	G	0	0.2143	A
9	BICF2P670814	49856011	G	0	0.2143	C
9	TIGRP2P128447_rs9014908	49866890	C	0	0.2143	A
9	TIGRP2P128455_rs8789784	49887606	A	0	0	G
9	BICF2P124563	49902839	G	0	0	A
9	BICF2P328665	49931564	G	0.5	0.2857	A
9	BICF2P231855	49935877	C	0.5	0.2857	G
9	BICF2S23641012	49946865	A	0	0	G
9	BICF2P1917	49967976	A	0.5	0.07143	G
9	BICF2P511906	49985248	C	0	0.2143	G
9	BICF2P556507	50001988	A	0	0.2143	G
9	BICF2P211904	50004144	0	0	0	A
9	BICF2P670409	50017375	C	0	0.2143	A
9	BICF2P766155	50032514	A	0	0.2143	G
9	BICF2S23128512	50043058	A	0	0.2143	G
9	BICF2P489568	50048987	0	0	0	A
9	BICF2P1123039	50059505	A	0	0.2143	T
9	BICF2P1322349	50078526	A	0	0.2143	G
9	BICF2S23620985	50087156	A	0	0.2143	G
9	BICF2P741632	50112934	A	0	0	G
9	BICF2S2378907	50115199	G	0	0.2143	A

9	TIGRP2P128487_rs8813164	50123934	0	0	0	A
9	BICF2P730434	50143851	A	0.5	0.7143	G
9	BICF2P584446	50156097	A	0.5	0.7143	G
9	BICF2P584449	50157353	A	0.5	0.7143	G
9	BICF2P853245	50164666	C	0.5	0.7143	A
9	BICF2P1405000	50184646	A	0	0.2143	T
9	BICF2P827480	50195274	A	0	0	G
9	BICF2P728721	50204714	A	0	0.2143	G
9	BICF2P428944	50212823	A	0.5	0.07143	G
9	BICF2P656033	50226004	A	0	0	G
9	BICF2P1074785	50239506	A	1	0.7857	G
9	BICF2P971316	50246603	G	0	0	A
9	BICF2S23544847	50254249	A	1	0.7857	G
9	BICF2P1328170	50256378	G	0	0	A
9	BICF2S2337129	50264065	A	1	0.7857	G
9	BICF2S23447094	50274178	G	1	0.7857	A
9	BICF2P1039318	50278780	A	0.5	0.07143	C
9	BICF2P722701	50283403	A	0.5	0.07143	G
9	BICF2S23235981	50300808	A	1	1	G
9	BICF2P1402973	50301756	G	1	1	A
9	BICF2P1347419	50314792	C	1	1	A
9	BICF2P193361	50332306	G	0.5	0.9286	A
9	BICF2S23119092	50338990	G	0	0.2143	A
9	BICF2P429200	50349293	A	0.5	0.7143	G
9	BICF2P1431765	50373181	A	0.5	0.07143	G
9	BICF2P987828	50400487	G	0.5	0.7143	A
9	BICF2P745139	50429928	A	0.5	0.9286	G
9	BICF2P1024173	50448874	A	0	0.2143	G
9	TIGRP2P128537_rs9110562	50450699	A	0.5	0.9286	G
9	TIGRP2P128547_rs8533455	50465848	G	0.5	0.9286	A
9	BICF2P1397023	50476119	G	0.5	0.9286	A
9	BICF2P1065566	50486773	G	0.5	0.9286	A
9	TIGRP2P128566_rs9138929	50497241	C	0.5	0.9286	A
9	BICF2S2334024	50521907	A	0	0	C
9	BICF2P1273761	50522350	A	0	0.2143	G
9	BICF2P1219827	50531074	A	0	0	G
9	TIGRP2P128609_rs8410855	50554075	0	0	0	C
9	TIGRP2P128611_rs9127187	50572494	A	0.5	0.07143	C
9	TIGRP2P128621_rs8733943	50583142	G	0.5	0.07143	A
9	BICF2P1087851	50594034	C	0.5	0.07143	A
9	TIGRP2P128662_rs9134557	50622246	A	0.5	0.2857	G
9	BICF2P771876	50623503	A	0.5	0.2857	G
9	BICF2P985889	50650807	A	0.5	0.07143	C
9	TIGRP2P128715_rs8808183	50664855	A	0.5	0.07143	G
9	BICF2P769724	50671173	A	0	0	G
9	BICF2S23335630	50700064	G	0.5	0.9286	A
9	TIGRP2P128729_rs8762603	50703692	A	0.5	0.7143	G

9	BICF2S23516268	50717081	G	0.5	0.7143	A
9	BICF2S23144924	50760148	G	0.5	0.07143	A
9	TIGRP2P128737_rs9246744	50779969	A	1	1	G
9	BICF2P1168224	50783197	A	0.5	0.9286	G
9	BICF2S23243638	50793322	G	0.5	0.07143	A
9	BICF2P338413	50806437	G	0	0.2143	A
9	BICF2P895198	50807463	0	0	0	C
9	BICF2P209349	50820625	C	0	0	G
9	BICF2S2378056	50864181	A	0	0	G
9	TIGRP2P128788_rs8716631	50875302	0	0	0	G
9	BICF2P1244848	50883590	G	0.5	0.2857	A
9	BICF2P1363516	50887362	A	0	0	G
9	BICF2P887545	50895813	A	0	0	G
9	BICF2P193796	50905472	A	0	0	G
9	BICF2S23762723	50919127	G	0.5	0.7143	A
9	BICF2P1067525	50932385	A	0.5	0.7143	G
9	TIGRP2P128813_rs8695731	50941304	A	0.5	0.7143	C
9	BICF2S23741018	50951678	A	0.5	0.7143	C
9	TIGRP2P128822_rs8549589	50958408	A	0.5	0.7143	G
9	BICF2P1201264	50969601	A	0	0	G
9	TIGRP2P128838_rs8916307	50988217	A	0	0	G
9	TIGRP2P128840_rs9192125	50997142	A	0	0.2143	G
9	TIGRP2P128841_rs8947486	51006778	G	0	0.2143	C
9	BICF2P228793	51014545	A	0	0	C
9	BICF2P1334587	51034733	A	0	0.2143	G
9	BICF2P703007	51040585	A	0.5	0.2857	G
9	BICF2P302956	51052545	G	0	0.2143	A
9	BICF2P912680	51060361	A	0	0	C
9	BICF2P488217	51079519	0	0	0	G
9	BICF2P1246065	51087699	A	0	0	G
9	BICF2P909240	51119357	G	0	0	A
9	BICF2P298224	51130082	C	0.5	0.2857	A
9	BICF2P857442	51146546	A	0	0.2143	G
9	BICF2S2323858	51175129	A	0	0	G
9	TIGRP2P128891_rs9209366	51194663	A	0.5	0.7143	G
9	BICF2S23130130	51198789	G	0	0	A
9	BICF2P249174	51201051	G	0	0	A
9	TIGRP2P128900_rs8631906	51221705	C	0	0.2143	A
9	BICF2P1393086	51242373	A	0	0	C
9	TIGRP2P128932_rs8665036	51250936	A	0	0	G
9	BICF2P301757	51263005	G	0	0	A
9	BICF2P1246771	51267810	A	1	0.7857	G
9	TIGRP2P128980_rs8721400	51296665	A	0	0.2143	G
9	BICF2P652670	51312663	A	0	0	C
9	TIGRP2P128991_rs8969837	51313281	A	0	0.2143	G
9	BICF2S23055092	51321528	A	0	0	G
9	BICF2P1102514	51337114	G	0	0.2143	A

9	BICF2S23718138	51348535	A	0.5	0.9286	G
9	BICF2P529428	51360314	A	0	0.2143	G
9	BICF2P1104030	51371569	A	0	0.2143	G
9	BICF2S23337863	51385150	A	0	0.2143	G
9	TIGRP2P129027_rs9099916	51400287	G	0	0.2143	A
9	BICF2P200734	51413134	0	0	0	A
9	BICF2P803441	51419010	G	0	0.1	A
9	BICF2P42147	51433690	C	0	0.2143	A
9	BICF2S23350844	51441375	A	0	0.2143	G
9	BICF2P44150	51442214	G	0	0.2143	A
9	TIGRP2P129044_rs8741436	51462443	G	0	0.2143	A
9	TIGRP2P129049_rs9195111	51467656	G	0	0.2143	A
9	BICF2S2349353	51474094	A	0	0.2143	G
9	BICF2S24318085	51486130	G	0	0.2143	A
9	BICF2S22964348	51507910	G	0.5	0.07143	A
9	BICF2P1254880	51510298	A	0.5	0.9	G
9	BICF2P753594	51531181	C	0.5	0.07143	G
9	BICF2P436494	51541093	G	0.5	0.07143	A
9	BICF2P812982	51544743	G	1	0.7857	A
9	BICF2S23637363	51559722	A	0.5	0.7143	G
9	TIGRP2P129061_rs8664018	51566046	C	0	0	A
9	BICF2P252142	51586633	G	0	0	A
9	BICF2P1411970	51598927	G	0	0	A
9	BICF2P1140358	51604841	G	0.5	0.7143	A
9	BICF2S23250278	51608113	G	0	0	A
9	BICF2P1006218	51612020	A	0.5	0.7143	G
9	BICF2P397161	51627370	C	0	0.2143	G
9	BICF2P890149	51638986	C	0.5	0.7143	A
9	BICF2P792225	51647269	A	0	0.2143	G
9	BICF2P811886	51658185	G	0	0.2143	A
9	TIGRP2P129092_rs8536134	51686265	G	0	0.2143	A
9	BICF2P775027	51700199	G	0	0.2143	A
9	BICF2P1000848	51711557	A	0	0.2143	G
9	BICF2P639277	51718368	G	0	0.2143	A
9	BICF2P319158	51729342	A	0	0	G
9	BICF2P482628	51749460	A	0.5	0.9286	G
9	BICF2P703190	51753587	A	0	0	G
9	BICF2P256008	51771477	A	0	0	G
9	BICF2S22931940	51776397	G	0	0	A
9	BICF2P397932	51790792	A	0	0	G
9	BICF2S22924196	51797485	G	0	0.2143	A
9	BICF2P516508	51801364	C	0	0.1667	A
9	BICF2P637580	51802905	A	0	0	G
9	BICF2P190561	51805265	G	0	0.2143	A
9	TIGRP2P129106_rs8663142	51807706	A	0	0	G
9	BICF2P727531	51830460	0	0	0	C
9	BICF2P984103	51837212	A	0	0	G

9	BICF2P548016	51843521	0	0	0	G
9	TIGRP2P129131_rs8518454	51869219	0	0	0	G
9	TIGRP2P129127_rs8763537	51886593	A	0	0	C
9	BICF2P622646	51901712	0	0	0	G
9	BICF2P644148	51918440	A	0	0	G
9	BICF2P606548	51920369	G	0	0.2857	A
9	TIGRP2P129154_rs8687471	51925096	0	0	0	C
9	BICF2S23212770	51937041	G	0	0.2857	A
9	BICF2P1070652	51946848	0	0	0	G
9	BICF2S23252934	51967363	A	0	0.2857	G
9	BICF2S23020433	51968798	A	0	0	G
9	BICF2P1197297	51995718	A	0	0	G
9	BICF2P974723	52004352	A	0	0.2857	G
9	BICF2P506351	52021997	G	0	0.2857	A
9	BICF2P84548	52031784	G	0	0	A
9	TIGRP2P129190_rs8711268	52039407	0	0	0	A
9	TIGRP2P129192_rs8703617	52054123	0	0	0	G
9	BICF2P1167937	52068696	G	0	0.2857	A
9	BICF2P583133	52079014	0	0	0	A
9	TIGRP2P129194_rs8744612	52084099	A	0.5	0.9286	G
9	BICF2P1291044	52091000	A	0.5	0.6429	C
9	BICF2P873793	52103960	A	0.5	0.9286	G
9	BICF2P1320464	52108416	C	0	0	A
9	TIGRP2P129215_rs9099372	52120272	A	0	0	G
9	BICF2S23126657	52134506	C	0.5	0.6429	A
9	TIGRP2P129233_rs9186861	52151161	A	0.5	0.3333	C
9	BICF2P281284	52168984	G	0.5	0.6429	A
9	BICF2S2347140	52169759	G	0.5	0.6429	A
9	BICF2P306818	52179837	G	0	0.2857	A
9	TIGRP2P129272_rs8641580	52194953	0	0	0	G
9	BICF2P893407	52204925	0	0	0	C
9	BICF2S23647327	52218839	A	0	0	G
9	BICF2S2378118	52224422	G	NA	0.7	A
9	BICF2S2339614	52248874	A	0	0	G
9	BICF2S2328598	52260242	A	0	0	G
9	BICF2S2352066	52278068	A	0	0.2857	G
9	BICF2P303551	52287032	G	0	0.2857	A
9	BICF2P647469	52297856	A	0	0	G
9	BICF2P109365	52301819	G	0	0	A
9	BICF2P844104	52304882	A	0	0	G
9	BICF2P817799	52313765	A	0	0	G
9	TIGRP2P129293_rs8755542	52321840	G	0	0	A
9	BICF2S23744222	52321973	C	0	0	A
9	BICF2P297674	52357101	A	0	0	G
9	BICF2P564059	52369572	G	0	0	A
9	BICF2P629182	52379088	A	0	0	G
9	BICF2P1211075	52396159	G	0.5	0.6429	A

9	BICF2P8703	52407975	A	0	0.2857	G
9	BICF2P122488	52412215	G	0	0	A
9	BICF2P1342526	52419107	A	0	0	G
9	BICF2P238373	52439042	C	0	0	A
9	TIGRP2P129352_rs9162246	52446517	G	0	0	A
9	BICF2P531661	52457710	A	0	0.2857	G
9	BICF2S23432055	52463786	A	0.5	0.3571	G
9	BICF2S23534851	52488483	A	0	0	G
9	TIGRP2P129361_rs8671574	52506731	0	0	0	C
9	BICF2P939212	52515720	A	0.5	0.6667	G
9	BICF2P1074567	52529598	A	0	0	G
9	BICF2P765849	52539409	G	1	0.7143	A
9	BICF2P785316	52543809	G	1	0.7143	A
9	BICF2S24313274	52548772	G	0.5	0.07143	A
9	BICF2P991130	52552788	C	0	0	A
9	BICF2P330625	52566004	0	0	0	G
9	BICF2P1463303	52571959	0	0	0	C
9	BICF2P164035	52580736	G	0.5	0.6429	A
9	BICF2P1044590	52590146	0	0	0	G
9	BICF2S23728647	52622327	A	0	0	G
9	BICF2P548713	52631430	0	0	0	G
9	BICF2P976080	52638438	G	0	0	A
9	TIGRP2P129367_rs9148074	52647113	G	0	0	A
9	TIGRP2P129370_rs8971072	52677766	A	0	0	G
9	BICF2P724217	52685912	0	0	0	A
9	BICF2P1300203	52716974	A	1	1	G
9	BICF2S23442116	52734860	G	0.5	0.9286	A
9	BICF2P1211975	52750232	C	NA	0.07143	A
9	BICF2S23429475	52753363	A	0.5	0.9286	G
9	BICF2P793049	52769832	A	0.5	0.07143	G
9	BICF2P190430	52784985	A	0	0	G
9	BICF2P500532	52787108	A	0	0	G
9	BICF2S23660309	52800493	0	0	0	G
9	TIGRP2P129440_rs8527216	52808839	A	0	0	G
9	BICF2P999649	52837345	A	0	0	G
9	BICF2S22927010	52853393	G	0	0	A
9	BICF2S2364068	52858907	G	0	0	A
9	BICF2P123779	52866013	A	0	0	G
9	BICF2P282196	52890645	A	0	0	G
9	BICF2P1427387	52911441	A	0	0	G
9	TIGRP2P129471_rs8521846	52914591	A	0	0	G
9	BICF2S23526489	52932223	A	0	0	G
9	BICF2S23448869	52943184	A	0	0	G
9	BICF2S23145223	52951579	G	0.5	0.3571	A
9	BICF2S2306795	52959759	A	0	0	G
9	BICF2P1124087	52982519	A	0	0	G
9	BICF2P256562	52993153	A	0.5	0.6429	G

9	BICF2P1162299	53011815	G	0	0.2857	A
9	BICF2P927936	53042416	A	0	0	G
9	TIGRP2P129553_rs8718298	53051476	G	0.5	0.3571	A
9	TIGRP2P129559_rs8636429	53067899	G	0	0	A
9	BICF2P918885	53090966	G	0	0.2857	A
9	BICF2P921172	53092375	G	0	0.2857	A
9	TIGRP2P129599_rs9240916	53104428	A	0	0	C
9	BICF2P312976	53108740	0	0	0	A
9	TIGRP2P129629_rs9186227	53119227	G	1	0.7143	A
9	BICF2P137043	53119291	C	0	0	A
9	BICF2P1458179	53134489	A	1	0.7143	G
9	TIGRP2P129637_rs9240980	53147175	A	0	0	G
9	BICF2P1294495	53164139	A	0.5	0.07143	C
9	BICF2P191114	53168805	G	1	0.7143	A
9	BICF2S23326566	53179985	A	0.5	0.3571	G
9	BICF2P547120	53190570	A	0	0	G
9	BICF2P616809	53200903	G	0	0	A
9	TIGRP2P129681_rs9051446	53219024	G	0.5	0.9286	A
9	BICF2S23210310	53222717	G	1	1	A
9	BICF2P1092146	53236128	G	0	0.2857	A
9	BICF2P883005	53245168	A	0	0.2857	G
9	BICF2P322734	53247168	T	0	0	A
9	BICF2P1435841	53260497	A	0	0	G
9	BICF2P958064	53268923	A	0	0.2857	G
9	TIGRP2P129751_rs9023276	53281469	A	0.5	0.07143	C
9	BICF2P208328	53311286	A	0	0	C
9	TIGRP2P129767_rs8946542	53319697	G	0.5	0.9286	A
9	BICF2P1285681	53344381	A	0	0	G
9	BICF2P1244419	53350831	C	0	0.2857	A
9	BICF2P677185	53358804	A	0.5	0.6429	G
9	TIGRP2P129812_rs9131794	53362465	A	0	0	G
9	TIGRP2P129832_rs8939254	53382144	G	0	0	A
9	BICF2P557640	53390456	A	0.5	0.07143	G
9	TIGRP2P129856_rs8518300	53405653	A	0	0.2857	G
9	BICF2P873768	53416652	A	0	0	G
9	BICF2P890246	53427907	A	0.5	0.6429	C
9	BICF2P139678	53432248	G	0.5	0.6429	A
9	BICF2S22925948	53449693	A	0.5	0.07143	C
9	BICF2S23060805	53454666	A	0	0.2857	G
9	BICF2P1435064	53467382	A	0.5	0.6429	G
9	BICF2P508245	53479879	A	0.5	0.2143	G
9	BICF2S23532268	53488481	0	0	0	A
9	BICF2P279425	53511717	G	0	0.4286	A
9	BICF2G630471369	53524200	A	1	0.5714	G
9	TIGRP2P129898_rs8585914	53540542	A	0	0	G
9	BICF2G630471393	53553642	G	0	0	A
9	BICF2P1240963	53566327	G	0	0	A

9	BICF2G630471426	53576331	G	0	0.7143	A
9	BICF2P125601	53583727	G	0	0	A
9	BICF2S23510528	53583799	A	0	0	G
9	BICF2G630471456	53593365	G	0.5	0.2143	A
9	BICF2G630471474	53604092	A	0.5	0.07143	G
9	BICF2G630471485	53614761	A	0	0	G
9	TIGRP2P129917_rs9100460	53630720	A	0	0	G
9	BICF2P253892	53647141	A	0	0	G
9	BICF2P1151426	53654938	G	0	0	A
9	BICF2S23722590	53661171	A	0	0	G
9	TIGRP2P129923_rs8776947	53677101	0	0	0	G
9	BICF2G630471505	53686935	G	0.5	0.2143	A
9	BICF2S23528651	53694665	G	0	0	A
9	TIGRP2P129930_rs9120744	53712888	G	0.5	0.5	A
9	BICF2S23424188	53723172	A	1	0.5714	G
9	BICF2P896777	53728979	A	0	0	G
9	BICF2P1223940	53737240	A	0	0	G
9	BICF2P479842	53749466	A	0	0	G
9	BICF2P695100	53754603	G	1	0.5714	A
9	BICF2G630471519	53775758	A	0	0	G
9	BICF2G630471540	53786456	C	0.75	0.7143	G
9	BICF2G630471561	53798571	A	0	0	G
9	BICF2P332614	53808540	G	0.75	0.2857	C
9	BICF2P103048	53812050	A	0	0	C
9	BICF2G630471581	53824729	A	0.25	0.2143	G
9	BICF2G630471600	53832977	G	0.25	0.2857	A
9	BICF2G630471619	53844084	A	0	0	G
9	BICF2P284322	53860854	A	0.25	0.2143	G
9	TIGRP2P129993_rs8552460	53867878	A	0.25	0.2857	G
9	BICF2G630471623	53878197	A	0	0	C
9	BICF2P491579	53889120	0	0	0	C
9	BICF2P396584	53908928	0	0	0	A
9	TIGRP2P130010_rs8782369	53911191	G	0	0	A
9	BICF2P1440236	53915832	G	1	1	A
9	BICF2S23612368	53933741	A	0.75	0.7857	G
9	BICF2G630471691	53935027	C	0.75	0.7857	A
9	BICF2G630471710	53949938	G	0.25	0.07143	A
9	BICF2G630471723	53959116	A	0.25	0.07143	G
9	BICF2G630471730	53971544	A	0.25	0.07143	G
9	BICF2S23027868	53993581	G	0.25	0.2857	A
9	BICF2P1089188	54008962	G	0.25	0.2857	A
9	BICF2P1288943	54020234	A	0.25	0.2143	G
9	BICF2G630471776	54021524	A	0.25	0.07143	G
9	BICF2G630471795	54034371	A	0.25	0.07143	G
9	BICF2P486655	54043880	A	0.25	0.07143	G
9	BICF2G630471817	54051733	G	0.5	0.3571	A
9	BICF2P341993	54061884	G	0.75	0.5714	A

9	BICF2G630471847	54073987	A	0.25	0.2143	G
9	BICF2G630471849	54074394	A	0.25	0.2143	C
9	BICF2P375522	54090257	A	0.5	0.2857	G
9	BICF2G630471885	54102643	G	0.75	0.9286	A
9	BICF2S232615	54117001	A	0	0	G
9	BICF2G630471901	54121319	G	0.5	0.3571	A
9	BICF2G630471905	54123105	A	0.5	0.6429	C
9	BICF2G630471924	54133820	G	0.5	0.5	A
9	BICF2G630471955	54142867	C	0.75	0.9286	A
9	BICF2G630471963	54150569	A	0.25	0.2143	G
9	BICF2P1283147	54155327	C	0.75	0.9286	A
9	BICF2P1212798	54166667	C	0.25	0.2857	A
9	BICF2P394159	54180503	A	0.25	0.2857	G
9	BICF2P614388	54195788	0	0	0	G
9	BICF2S23414295	54213641	A	0.75	0.7143	C
9	BICF2P907446	54223642	A	1	1	G
9	BICF2P542856	54234693	G	0	0	A
9	BICF2P683989	54245897	G	0.25	0.2857	A
9	TIGRP2P130130_rs8612259	54258162	A	0.5	0.7143	G
9	TIGRP2P130136_rs8985683	54268774	G	1	1	A
9	BICF2P1065550	54288486	G	0.5	0.2857	A
9	BICF2P389259	54291590	G	0.5	0.2857	A
9	BICF2P1124257	54312611	A	0.5	0.2857	G
9	BICF2P239575	54319975	G	0.5	0.2857	A
9	BICF2S23124778	54332690	A	0.5	0.2857	G
9	BICF2P770237	54340844	G	0.5	0.2857	A
9	BICF2S23762962	54358448	G	0.75	0.5714	A
9	BICF2P526893	54361262	A	0.5	0.2857	G
9	BICF2G630472112	54373687	G	0.75	0.5714	A
9	BICF2G630472165	54388409	A	0.5	0.2857	G
9	TIGRP2P130201_rs9174363	54397820	A	0.75	0.7143	G
9	BICF2P1007163	54408431	G	0.25	0.2857	A
9	TIGRP2P130209_rs8912128	54429433	C	0	0	A
9	BICF2S23323292	54431088	C	0.25	0.2143	A
9	BICF2S22953468	54442883	A	0	0	G
9	BICF2P745330	54445782	A	0.25	0.2143	C
9	BICF2P22244	54461445	A	0.75	0.5714	G
9	BICF2G630472198	54464376	A	0.75	0.5714	G
9	BICF2S23637801	54486138	G	0.25	0.2857	A
9	BICF2S22926196	54496873	G	0.5	0.7143	A
9	BICF2P318602	54505902	G	0.25	0.2857	A
9	TIGRP2P130260_rs8567497	54511647	G	0.5	0.75	A
9	BICF2G630472226	54531979	A	0.25	0.07143	G
9	BICF2G630472234	54534620	A	0.25	0.2857	G
9	TIGRP2P130274_rs9085907	54554627	C	0.75	0.9286	A
9	BICF2P1192833	54576427	G	0.5	0.6429	A
9	BICF2G630472379	54613382	A	0.5	0.3571	G

9	BICF2G630472384	54619276	C	0	0	A
9	BICF2G630472411	54630170	A	0.25	0.2143	G
9	BICF2G630472413	54630529	A	0	0	G
9	BICF2G630472427	54642128	A	0.25	0.07143	G
9	TIGRP2P130442_rs9045528	54664169	A	0	0	G
9	BICF2G630472439	54681918	A	0.25	0.2857	G
9	BICF2G630472444	54688309	A	0.25	0.07143	G
9	BICF2G630472456	54700573	A	0	0	G
9	TIGRP2P130456_rs9093736	54705690	G	0	0	A
9	BICF2P12439	54719853	A	0	0	T
9	BICF2P483579	54737535	A	0.25	0.4167	G
9	TIGRP2P130470_rs8646545	54741094	A	0.25	0.4286	G
9	TIGRP2P130488_rs8978311	54757052	A	0	0.1667	G
9	TIGRP2P130490_rs8798256	54765681	A	0.25	0.2143	G
9	TIGRP2P130497_rs8680413	54793417	G	0.25	0.2143	A
9	BICF2P481093	54799094	0	0	0	C
9	TIGRP2P130503_rs9151713	54811711	G	0.25	0.2143	A
9	BICF2P450113	54825215	C	0.5	0.7143	G
9	TIGRP2P130505_rs9053208	54830267	0	0	0	C
9	BICF2P1158102	54831983	G	0.75	0.7857	A
9	TIGRP2P130516_rs8926411	54835079	0	0	0	A
9	BICF2P363876	54843232	G	0.75	0.7857	A
9	BICF2P248182	54865628	0	0	0	G
9	BICF2P682211	54877462	A	0.75	0.7857	C
9	BICF2P616824	54878823	A	0.5	0.7143	G
9	BICF2P604164	54905965	G	0.5	0.7143	A
9	TIGRP2P130554_rs8779530	54914082	0	0	0	C
9	TIGRP2P130572_rs8797279	54931039	A	0.25	0.2857	G
9	TIGRP2P130576_rs8554526	54940937	G	0.25	0.2857	A
9	BICF2S23335684	54947321	G	0.75	0.7143	A
9	BICF2S23028674	54960776	A	0.75	0.7143	G
9	BICF2S23412537	54977993	G	0	0	A
9	BICF2P180522	54988833	0	0	0	C
9	BICF2P953438	55001289	0	0	0	A
9	BICF2P942910	55010852	0	0	0	C
9	BICF2P791614	55020057	0	0	0	G
9	BICF2S22964777	55034651	G	0	0	A
9	TIGRP2P130587_rs8529768	55039729	0	0	0	G
9	BICF2P498993	55053632	G	0.5	0.5	A
9	BICF2P953835	55063714	C	0.75	0.7143	A
9	BICF2P878354	55079026	G	0.25	0.2857	A
9	BICF2P1213766	55091879	A	0.5	0.5	G
9	BICF2P1154988	55108962	A	0.5	0.5	C
9	BICF2P626451	55125765	A	0.25	0.2857	G
9	TIGRP2P130620_rs8982512	55126652	G	1	1	A
9	BICF2P626445	55126889	A	0.75	0.7143	G
9	TIGRP2P130630_rs9031670	55136902	A	0	0	G

9	BICF2P295436	55147111	A	0	0	G
9	BICF2P894661	55150926	C	0	0	A
9	BICF2S23036930	55151562	G	0	0	A
9	BICF2P1332188	55156151	G	1	1	A
9	TIGRP2P130673_rs9162745	55172832	G	0.5	0.5	A
9	TIGRP2P130677_rs8817121	55182277	0	0	0	C
9	BICF2S23320322	55188785	A	0.5	0.5	G
9	BICF2P673428	55196374	A	0.5	0.5	G
9	BICF2S23151937	55204212	A	0.75	0.5714	G
9	BICF2S23059999	55211825	A	0.75	0.5714	G
9	TIGRP2P130690_rs8984279	55223958	A	0	0	G
9	BICF2P931102	55244311	G	0.25	0.4286	A
9	BICF2P964841	55247180	G	0.25	0.4286	A
9	BICF2P1034906	55262509	A	0.25	0.07143	C
9	BICF2P753263	55269171	A	0.25	0.4167	G
9	BICF2S23412455	55290577	G	0.5	0.5	A
9	BICF2P1222342	55293558	A	0.25	0.07143	G
9	TIGRP2P130713_rs8734558	55304464	G	0.5	0.5	A
9	BICF2S23115489	55325806	A	0.5	0.5	G
9	BICF2P247546	55328597	A	0.25	0.07143	G
9	BICF2P1222181	55342184	A	0	0	G
9	BICF2P1164698	55354587	G	0.25	0.2143	A
9	BICF2G630472510	55363345	A	0	0	G
9	BICF2G630472535	55373115	A	0	0	G
9	BICF2G630472576	55389878	C	0.25	0.4286	G
9	BICF2P794932	55395493	G	0.5	0.5	A
9	BICF2G630472587	55397658	A	0.25	0.07143	G
9	BICF2G630472601	55410253	A	0.25	0.07143	G
9	BICF2G630472603	55424673	G	0	0	A
9	BICF2P923313	55434860	G	0	0.2857	A
9	BICF2G630472614	55450015	T	0.25	0.07143	A
9	BICF2G630472626	55454248	G	0.25	0.07143	A
9	BICF2G630472651	55471508	C	0.75	0.9286	A
9	BICF2P128722	55476833	0	0	0	T
9	BICF2G630472676	55492446	A	0.25	0.07143	G
9	BICF2G630472687	55498890	A	0.25	0.07143	G
9	BICF2G630472698	55504171	G	0	0	C
9	BICF2G630472721	55519178	A	0.25	0.07143	G
9	BICF2G630472726	55528983	A	0.25	0.07143	G
9	BICF2G630472733	55542371	A	0.25	0.07143	T
9	BICF2G630472745	55554206	C	1	1	A
9	BICF2G630472749	55559601	A	1	1	C
9	BICF2G630472761	55573992	G	1	1	A
9	BICF2G630472763	55582299	A	0	0	G
9	BICF2G630472772	55597190	A	1	1	G
9	BICF2G630472775	55612232	A	0	0	G
9	BICF2G630472776	55614162	G	0	0	A

9	BICF2G630472783	55632302	A	1	1	G
9	BICF2G630472789	55640762	T	1	1	A
9	BICF2G630472790	55650085	A	1	1	G
9	BICF2G630472807	55659119	A	0	0	G
9	BICF2G630472811	55662017	C	1	1	A
9	BICF2P503466	55682005	A	0.25	0.07143	G
9	BICF2G630472858	55687396	A	0.25	0.07143	C
9	BICF2G630472860	55696925	A	0	0	G
9	BICF2P935988	55709278	A	0	0	G
9	BICF2G630472906	55722536	A	1	1	G
9	BICF2P75315	55727957	A	0	0	G
9	BICF2S23651341	55733175	G	0.25	0.2857	A
9	BICF2P197561	55745277	A	0	0	G
9	BICF2P567070	55774365	A	0	0	G
9	BICF2G630472983	55781647	A	1	1	G
9	BICF2P755372	55792401	A	0.25	0.07143	G
9	TIGRP2P130855_rs9077374	55804878	G	0.5	0.7143	A
9	BICF2G630472995	55805008	A	0	0	G
9	BICF2G630472997	55813366	G	0.25	0.07143	C
9	BICF2P772510	55826254	A	0.25	0.2143	G
9	BICF2G630473027	55841605	A	0	0	C
9	BICF2G630473035	55847054	C	0	0	A
9	BICF2G630473051	55875536	G	0	0	A
9	BICF2G630473074	55892669	G	0	0	A
9	BICF2G630473095	55905283	A	0	0	G
9	BICF2S23645842	55915302	A	0	0	G
9	BICF2G630473128	55927243	A	1	1	G
9	BICF2G630473166	55954563	A	0.75	0.7143	G
9	BICF2G630473183	55963252	A	0.75	0.7143	G
9	BICF2G630473198	55971811	G	0.75	0.7143	A
9	BICF2P1343066	55987187	G	0.75	0.7143	A
9	BICF2S23235445	55993904	G	0.5	0.5	A
9	BICF2G630473223	56000845	A	0	0	G
9	BICF2G630473230	56005788	A	0	0	G
9	BICF2G630473254	56024181	A	0.5	0.5	G
9	BICF2P846991	56036812	G	0.5	0.5	A
9	BICF2G630473270	56043800	G	0.5	0.5	A
9	TIGRP2P130905_rs8497327	56054272	A	0.25	0.1429	G
9	BICF2G630473286	56061262	G	0.75	0.6429	A
9	BICF2G630473289	56071579	A	NA	0.5	G
9	BICF2P1199801	56094217	G	0	0	A
9	BICF2S2336128	56107479	G	0.25	0.3571	A
9	BICF2S22911164	56109108	A	0	0	G
9	BICF2P224794	56109555	T	0.25	0.1429	A
9	BICF2P198137	56130330	G	0.5	0.4286	A
9	BICF2P877340	56141540	A	0.5	0.5	G
9	TIGRP2P130939_rs9002481	56157845	0	0	0	G

9	BICF2S23328625	56165421	A	0.25	0.2143	C
9	TIGRP2P130942_rs8987843	56169240	G	0.75	0.7857	A
9	BICF2P1412893	56197414	G	0	0	A
9	BICF2P1144818	56203597	G	0	0	A
9	BICF2P83592	56217090	A	0.25	0.2143	G
9	BICF2P1247992	56224332	A	0.25	0.2143	G
9	BICF2P271838	56241939	A	0.25	0.2143	G
9	TIGRP2P130971_rs8559231	56256928	0	0	0	G
9	TIGRP2P130975_rs8988662	56258870	0	0	0	A
9	TIGRP2P130977_rs8653031	56271113	0	0	0	G
9	TIGRP2P130979_rs8817254	56287751	G	0.25	0.1429	A
9	BICF2P1331932	56292817	A	0	0	G
9	BICF2P1355545	56304951	G	0.25	0.1429	A
9	BICF2P375985	56315572	A	0.75	0.6429	G
9	BICF2S23627385	56338879	0	0	0	C
9	BICF2S23416717	56351076	A	0.25	0	G
9	BICF2P425623	56368674	A	0.75	0.7857	G
9	BICF2S23243720	56381402	0	0	0	G
9	BICF2P854739	56404869	A	0.5	0.3571	C
9	BICF2P126838	56408465	A	0.5	0.3571	G
9	BICF2P1341275	56424571	G	0	0	A
9	BICF2P721027	56434134	A	0	0	G
9	BICF2P772268	56434621	A	0.25	0.2857	G
9	BICF2P1337644	56452169	G	0.25	0.1429	A
9	BICF2P252703	56465402	A	0.75	0.8571	C
9	BICF2P1249538	56485020	C	0.25	0.1429	A
9	BICF2P1111197	56491969	G	0.75	0.8571	A
9	BICF2P1372742	56494891	G	0.75	0.8571	A
9	BICF2S23014602	56505873	A	0	0	G
9	BICF2P599379	56517102	A	0.75	0.8571	G
9	BICF2P1205809	56526350	A	0.75	0.8571	C
9	BICF2P1095005	56535611	G	1	0.8571	A
9	BICF2P1111536	56549452	C	0.75	0.8571	G
9	BICF2P1163077	56561650	A	0.75	0.8571	G
9	BICF2P717707	56570254	A	0.75	0.8571	G
9	BICF2P1267047	56578961	G	1	1	A
9	BICF2S2311089	56581605	0	0	0	G
9	BICF2P938441	56598035	C	0.75	0.6429	G
9	BICF2P65993	56631609	A	0.5	0.5	G
9	BICF2P1447068	56639389	A	0.5	0.5	G
9	BICF2S2329700	56641553	A	0	0	G
9	BICF2P1084	56660144	A	0.25	0.3571	G
9	BICF2P163818	56679950	A	0.5	0.6429	C
9	TIGRP2P131016_rs8758223	56698576	0	0	0	G
9	BICF2P1418545	56708849	0	0	0	G
9	BICF2P2433	56758392	0	0	0	C
9	BICF2P321059	56773865	0	0	0	G

9	BICF2P154580	56783385	A	NA	0.7	C
9	BICF2P1265894	56792382	G	1	0.8571	A
9	BICF2P259507	56793936	G	0.75	0.9167	A
9	BICF2S23740147	56799923	C	0.25	0.1429	A
9	BICF2P1244573	56815068	A	0.5	0.3571	G
9	BICF2S23617873	56829350	A	0.25	0.2143	G
9	BICF2P856172	56834230	C	0.25	0.2143	A
9	BICF2P1279633	56855202	A	1	1	C
9	BICF2P639917	56857040	C	0	0	G
9	BICF2P565684	56878470	A	0.25	0.1429	G
9	BICF2P565681	56878671	A	0.25	0.1429	G
9	BICF2P979121	56882346	G	0.25	0.1429	A
9	BICF2P728839	56887902	A	0.75	0.8571	G
9	BICF2P1462669	56896223	G	0	0	A
9	BICF2P184911	56904403	A	0	0	G
9	TIGRP2P131021_rs8701275	56932769	0	0	0	G
9	BICF2P157666	56954383	G	0.25	0.2143	A
9	BICF2S236831	56960842	A	0.25	0.2143	G
9	BICF2S2346755	56981942	G	0.25	0.2143	A
9	BICF2S23736443	56998520	A	0	0	G
9	BICF2P1033788	57007135	A	0.5	0.5714	G
9	BICF2S2327824	57017170	A	0.25	0.2857	G
9	BICF2P1031563	57035400	A	0	0	G
9	BICF2P1031564	57035595	A	0.5	0.3571	T
9	BICF2S2298442	57046184	G	0	0	A
9	BICF2P488147	57057550	A	0.25	0.2143	G
9	BICF2P505461	57085178	G	0.25	0.1429	A
9	TIGRP2P131025_rs8778053	57093074	0	0	0	A
9	BICF2S2373714	57106857	G	0	0	A
9	BICF2P535283	57114581	A	0.25	0.2143	T
9	BICF2S23155810	57123696	A	0	0	G
9	BICF2P1131880	57141686	C	0.25	0.1429	G
9	BICF2P1069837	57153163	A	0.25	0.1429	G
9	BICF2P1436750	57163178	0	0	0	A
9	BICF2P1303462	57231778	C	0.5	0.5714	A
9	BICF2S23138824	57237442	C	0.25	0.2857	A
9	BICF2P311912	57248497	G	0.25	0.2143	A
9	BICF2P175757	57273986	0	0	0	A
9	BICF2P663247	57281932	G	0.75	0.7857	A
9	BICF2S23341467	57311859	G	0.25	0.2143	A
9	BICF2P959637	57317211	C	0.25	0.2143	G
9	BICF2P64569	57338918	A	0.5	0.5714	G
9	BICF2S2344489	57339906	A	0.5	0.5714	G
9	BICF2S23551337	57355604	A	0	0	G
9	BICF2S23340470	57375986	A	0	0	G
9	BICF2P484113	57388959	0	0	0	G
9	BICF2S23355956	57404331	A	0.25	0.3571	G

9	BICF2S23227056	57410511	C	0.5	0.6429	A
9	TIGRP2P131035_rs9084195	57420634	0	0	0	G
9	BICF2S2306823	57436351	G	0	0	A
9	BICF2S23416018	57450341	A	0	0	G
9	BICF2P583320	57464468	A	0.5	0.5	G
9	BICF2P1248244	57467036	G	0.5	0.5	A
9	BICF2G630473300	57489650	G	0.5	0.6429	A
9	BICF2G630473310	57500319	A	0.5	0.4286	G
9	BICF2G630473312	57505233	C	0.25	0.2857	A
9	BICF2P580754	57518654	A	0.75	0.6429	G
9	BICF2G630473329	57535048	A	0	0	G
9	BICF2P766944	57555147	C	0.5	0.4286	A
9	BICF2G630473345	57564211	A	0	0.2143	G
9	BICF2S23639106	57574398	G	0.5	0.5	A
9	BICF2G630473353	57591234	G	0.25	0.2857	A
9	BICF2G630473380	57601169	G	0.5	0.6429	A
9	BICF2G630473388	57607317	G	0.25	0.2857	A
9	BICF2P1284597	57623230	A	0.25	0.1429	G
9	BICF2G630473393	57626311	A	0.25	0.2143	G
9	BICF2G630473394	57630256	G	0.5	0.5	A
9	BICF2S2342742	57647751	A	0.5	0.5	G
9	BICF2G630473404	57655708	A	0	0	G
9	BICF2G630473412	57662062	A	0.75	0.7143	C
9	BICF2G630473430	57668121	A	0.5	0.5	G
9	BICF2G630473441	57672655	A	0.25	0.3571	G
9	BICF2S23658887	57700616	C	0.5	0.5714	G
9	BICF2G630473449	57713051	G	0.25	0.2143	A
9	BICF2G630473454	57725671	A	0.25	0.2143	G
9	BICF2S233562	57739102	C	0.25	0.3571	A
9	BICF2P659692	57752264	A	0.5	0.3571	G
9	BICF2G630473462	57758939	A	0.5	0.3571	G
9	BICF2S23422745	57772294	G	0.25	0.2857	A
9	BICF2G630473486	57784094	G	0.5	0.3571	A
9	BICF2P1359537	57811324	0	0	0	A
9	BICF2S23553126	57823485	T	0	0	A
9	BICF2S23643149	57841697	G	0	0	A
9	BICF2S23244968	57853166	A	0	0	G
9	BICF2G630473518	57866371	C	1	1	A
9	BICF2G630473524	57872609	A	1	1	G
9	BICF2G630473547	57882238	A	0	0	C
9	BICF2G630473563	57891497	A	1	1	C
9	BICF2G630473582	57904969	G	1	1	A
9	BICF2G630473617	57953651	A	0	0	G
9	BICF2S23657763	57967065	0	0	0	G
9	BICF2G630473618	57976560	A	0.25	0.1429	C
9	BICF2G630473641	58001513	A	0.25	0.1429	G
9	BICF2G630473653	58008872	A	0.25	0.1429	G

9	BICF2G630473675	58027552	A	0.5	0.3571	G
9	BICF2P1295530	58031172	0	0	0	C
9	BICF2G630473680	58043176	A	0.5	0.3333	G
9	BICF2G630473683	58054850	C	1	1	G
9	BICF2G630473690	58063839	G	1	1	A
9	BICF2G630473695	58076131	C	1	1	A
9	BICF2G630473702	58081278	G	1	1	A
9	BICF2G630473722	58095179	A	1	1	G
9	BICF2G630473731	58102390	A	0.75	0.6667	C
9	BICF2G630473744	58116208	A	0	0	G
9	BICF2G630473756	58128477	A	0	0	C
9	BICF2G630473766	58138534	A	0	0	G
9	BICF2S2371549	58147917	C	0.75	0.8571	A
9	BICF2P894196	58189768	G	0.75	0.6429	A
9	BICF2G630473811	58201021	A	0.5	0.3571	G
9	BICF2P949228	58212457	A	0	0	G
9	BICF2S23614905	58229061	G	0.5	0.6429	A
9	TIGRP2P131042_rs8812223	58238031	0	0	0	G
9	BICF2G630473845	58268581	A	0	0	G
9	BICF2P1374572	58285925	A	0	0	G
9	BICF2P1117927	58308974	G	0	0	A
9	BICF2S23022035	58324196	A	0	0	G
9	BICF2G630473927	58340052	A	0	0	G
9	BICF2G630473936	58346118	A	0	0	G
9	BICF2G630473940	58354303	C	0	0	A
9	G562f39S144	58381616	G	0	0	A
9	TIGRP2P131059_rs8798282	58396640	0	0	0	A
9	BICF2S24317651	58402907	A	0	0	G
9	BICF2P1359504	58416007	0	0	0	C
9	BICF2P603519	58442837	A	0.75	0.7857	G
9	BICF2G630473948	58451190	G	0	0	A
9	BICF2S23544734	58457706	A	0.5	0.5	G
9	BICF2G630473973	58472551	A	0.25	0.2857	G
9	BICF2G630473981	58487289	A	0.75	0.7143	G
9	BICF2G630473995	58500167	A	0.25	0.2857	C
9	BICF2P727918	58506901	A	0	0	G
9	BICF2P306890	58519763	A	0.5	0.5	G
9	BICF2S23236317	58527653	G	0.25	0.2857	C
9	BICF2P333641	58538700	A	1	1	G
9	BICF2P472772	58556154	G	0	0	A
9	BICF2G630474008	58567410	A	0.25	0.2143	G
9	BICF2S23423930	58573646	C	0.75	0.7857	G
9	BICF2G630474028	58583494	A	0.75	0.7857	G
9	BICF2P180918	58596040	A	0	0	G
9	BICF2G630474039	58597924	A	0	0	G
9	BICF2G630474041	58602557	G	0.25	0.2857	A
9	BICF2G630474054	58612520	C	0.75	0.7143	A

9	BICF2G630474068	58620902	G	1	1	A
9	BICF2G630474074	58628342	G	0	0	A
9	BICF2G630474083	58644271	A	0.25	0.2143	G
9	BICF2P927295	58655878	A	0	0	G
9	BICF2P562918	58664036	0	0	0	G
9	BICF2G630474100	58679972	G	0	0	A
9	BICF2P915257	58692284	G	0	0	A
9	BICF2G630474124	58705911	A	0	0	G
9	BICF2S2311814	58709408	A	0	0	G
9	BICF2G630474135	58719409	A	0	0	G
9	BICF2G630474137	58732622	A	0	0	G
9	BICF2P93403	58748334	A	0	0	G
9	BICF2P521959	58762083	G	0.25	0.2143	A
9	BICF2P966850	58766461	G	0.25	0.2143	A
9	BICF2P874582	58779689	A	0.25	0.2143	G
9	BICF2G630474163	58791653	A	0	0	T
9	BICF2G630474167	58800094	G	0	0	A
9	BICF2G630474183	58813964	A	0	0	G
9	BICF2P1040103	58820717	G	0	0	A
9	BICF2P438595	58844577	A	0	0	G
9	BICF2P274662	58846265	G	0	0	A
9	BICF2G630474227	58860194	G	0.5	0.5	A
9	BICF2G630474236	58866769	G	0.25	0.2143	A
9	BICF2G630474240	58877035	A	0	0	G
9	BICF2S2361054	58888536	A	0	0	G
9	BICF2S23042844	58900272	A	0	0	C
9	BICF2S23427352	58915372	G	0.5	0.5	A
9	BICF2S23450469	58935631	A	0	0	G
9	BICF2S23017379	58938069	A	0.5	0.5	G
9	BICF2P550162	58953028	A	0.25	0.2143	G
9	BICF2P714176	58972961	G	0.25	0.2143	A
9	BICF2G630474276	58990233	G	0	0	A
9	BICF2P799861	59017998	A	0	0	G
9	BICF2P512209	59025120	A	0	0	G
9	BICF2P104318	59029692	A	0.5	0.5	G
9	BICF2P721587	59042656	A	0.5	0.5	C
9	BICF2P76967	59060679	G	0.5	0.5	A
9	BICF2P76963	59061100	A	0.5	0.5	G
9	BICF2S23729828	59070309	A	0.5	0.5	G
9	BICF2P586637	59095558	A	0.5	0.5	G
9	BICF2S23763075	59107808	A	0.5	0.5	G
9	BICF2P513339	59111854	A	0.5	0.3571	C
9	BICF2G630474339	59123218	A	0	0	G
9	BICF2G630474366	59141193	G	0	0	A
9	BICF2G630474382	59152097	A	0	0	G
9	BICF2G630474394	59156413	G	0.25	0.2857	A
9	BICF2P467284	59194294	C	0.25	0.1429	A

9	BICF2G630474427	59208656	A	0	0	G
9	BICF2G630474452	59218056	A	0	0	C
9	BICF2P55775	59232964	C	0.25	0.2857	A
9	BICF2G630474497	59237078	G	0.75	0.6429	A
9	BICF2P150106	59248920	A	0	0	G
9	BICF2G630474505	59262298	G	0.25	0.2143	A
9	BICF2G630474517	59273775	G	0.25	0.2143	A
9	BICF2S24117588	59283628	C	0.25	0.3571	A
9	BICF2S23657684	59300694	0	0	0	G
9	BICF2P118984	59309991	A	0.5	0.6429	G
9	TIGRP2P131087_rs8678473	59317218	0	0	0	A
9	BICF2G630474519	59331052	0	0	0	G
9	BICF2S23764509	59349834	G	0.5	0.6429	A
9	BICF2G630474527	59361628	A	0.75	0.6429	G
9	BICF2G630474528	59365631	G	0.5	0.3571	A
9	BICF2G630474547	59381521	A	0.5	0.3571	G
9	BICF2G630474560	59393447	C	0.5	0.3571	A
9	BICF2G630474576	59406236	A	0.5	0.3571	G
9	BICF2G630474578	59411182	A	0.5	0.3571	G
9	BICF2G630474582	59430150	A	0.25	0.2857	G
9	BICF2G630474587	59436185	G	0.25	0.2857	A
9	BICF2G630474588	59452115	G	0.25	0.2857	C
9	BICF2G630474590	59462126	G	0.25	0.2857	A
9	BICF2G630474606	59473455	G	0	0	A
9	BICF2G630474616	59483554	0	0	0	A
9	BICF2G630474620	59495629	A	0	0	G
9	BICF2G630474628	59510175	A	0	0	G
9	BICF2G630474637	59518447	A	0.25	0.2857	G
9	BICF2P1271166	59542583	0	0	0	G
9	BICF2P119449	59552285	A	0.25	0.3571	G
9	BICF2S23526488	59564592	G	0.25	0.1667	A
9	BICF2G630474650	59580952	A	0.25	0.2857	G
9	BICF2P1303332	59595792	0	0	0	A
9	TIGRP2P131092_rs9039946	59615724	0	0	0	C
9	BICF2S2362679	59634508	A	0.5	0.3333	G
9	BICF2G630474655	59653597	A	0.25	0.3571	G
9	BICF2S2376082	59666049	A	1	1	G
9	BICF2P357957	59707459	G	0.5	0.3571	A
9	BICF2P357961	59709250	C	0.5	0.3571	A
9	BICF2P603881	59727741	G	0.75	0.8	A
9	BICF2P943190	59734028	A	0.5	0.5	G
9	BICF2G630474660	59763646	G	0.5	0.3571	A
9	BICF2G630474664	59771940	A	0.75	0.6429	G
9	BICF2G630474668	59780642	A	0.75	0.6429	G
9	BICF2P608413	59794260	A	0	0	G
9	BICF2G630474680	59805567	C	0.75	0.6429	A
9	BICF2G630474690	59818627	G	0.25	0.2857	A

9	BICF2G630474699	59823085	A	0.25	0.2857	G
9	BICF2G630474713	59841439	A	0	0	G
9	BICF2G630474786	59866132	A	0	0	G
9	BICF2G630474791	59869962	A	0	0	C
9	BICF2S2378654	59881449	G	0.25	0.2857	C
9	BICF2P1143183	59895604	G	0.25	0.2857	A
9	BICF2G630474812	59901027	G	0	0	A
9	TIGRP2P131105_rs9107786	59904745	0	0	0	G
9	BICF2G630474819	59919788	C	0	0	A
9	BICF2P1361507	59936544	0	0	0	G
9	BICF2G630474832	59942998	0	0	0	G
9	BICF2G630474839	59959479	A	0.25	0.2143	G
9	BICF2G630474841	59966111	A	0.25	0.2143	G
9	BICF2G630474842	59973945	G	0.75	0.6429	A
9	BICF2G630474853	59991734	G	0.25	0.2857	A
9	BICF2G630474858	59996893	A	0.25	0.2857	G
9	BICF2G630474874	60012660	A	0.25	0.2857	C
9	BICF2G630474884	60018951	G	0	0	A
9	BICF2G630474896	60035477	G	0	0	A
9	BICF2G630474904	60051591	C	0.25	0.1429	A
9	BICF2G630474911	60075329	A	0.25	0.1429	G
9	BICF2G630474921	60082339	A	0.25	0.1429	G
9	BICF2G630474923	60083905	A	0.25	0.1429	G
9	BICF2G630474981	60109650	A	0.25	0.1429	G
9	BICF2G630474986	60111135	C	0.25	0.1429	A
9	BICF2G630475002	60132468	G	0.25	0	A
9	BICF2P676203	60138150	0	0	0	C
9	BICF2G630475010	60150511	A	0.5	0.1667	G
9	BICF2G630475021	60164515	G	0.5	0.4286	A
9	BICF2G630475035	60173097	A	0.25	0.1429	G
9	BICF2G630475043	60179669	G	0.25	0.1429	A
9	BICF2G630475068	60198661	G	0.25	0.1667	A
9	BICF2G630475078	60204605	G	0	0.1429	A
9	BICF2G630475098	60220055	A	0.25	0.1429	G
9	BICF2G630475115	60230221	A	0.5	0.6429	G
9	BICF2G630475124	60240320	G	0.25	0.1429	A
9	BICF2S23118608	60251307	G	0.75	0.6429	A
9	BICF2G630475164	60269894	G	0.25	0.2857	A
9	BICF2G630475168	60273060	A	0.5	0.6429	G
9	BICF2S23222578	60287933	G	0.25	0.3571	A
9	BICF2P1271033	60297023	G	0.5	0.6429	A
9	BICF2P992597	60313580	A	0	0	C
9	BICF2P674532	60330198	0	0	0	T
9	BICF2P1444168	60352880	A	0.5	0.5	G
9	BICF2P473799	60369052	A	0	0	G
9	BICF2S23213010	60379177	A	0.75	0.7857	G
9	BICF2G630475174	60417635	G	0	0	A

9	BICF2G630475183	60421094	A	0.25	0.2857	G
9	BICF2G630475225	60443003	A	0.25	0.2857	G
9	BICF2G630475232	60446819	A	0.25	0.2857	G
9	BICF2G630475240	60447678	G	0.25	0.2857	A
9	BICF2G630475254	60450997	A	0.5	0.6429	G
9	BICF2G630475267	60455730	G	0.25	0.2857	A
9	BICF2G630475311	60477094	A	0.25	0.2857	C
9	BICF2G630475317	60480041	A	0.25	0.2857	G
9	BICF2G630475333	60492717	A	0.25	0.2857	C
9	BICF2G630475351	60503278	A	0	0	G
9	BICF2G630475372	60526735	A	0	0	G
9	BICF2S23627750	60548128	A	0.5	0.6429	G
9	BICF2S23650465	60576798	A	0	0	G
9	BICF2P1252570	60588760	0	0	0	A
9	BICF2P374221	60598549	A	0.5	0.6429	G
9	BICF2P788102	60609924	G	0.5	0.6429	A
9	BICF2P44267	60630598	G	0.5	0.6429	A
9	BICF2P788975	60644110	A	0.5	0.6429	G
9	BICF2P1033448	60666128	A	0.5	0.6429	G
9	BICF2P223161	60684987	G	0.5	0.6429	A
9	BICF2P819460	60691650	A	0.5	0.6667	G
9	BICF2P744833	60697483	A	0.5	0.6429	G
9	BICF2P236584	60711605	A	0.5	0.6429	G
9	BICF2S23451571	60739837	C	0.5	0.6429	A
9	BICF2S24116623	60750685	0	0	0	A
9	BICF2G630475390	60760574	A	0.25	0.2857	G
9	BICF2G630475403	60774146	A	0.25	0.2857	G
9	BICF2G630475425	60780780	A	0.25	0.2143	G
9	BICF2P1236981	60789325	0	0	0	A
9	BICF2P1394901	60800749	0	0	0	A
9	BICF2P794156	60835896	0	0	0	G
9	BICF2S2296546	60868879	A	0	0	C
9	BICF2P478221	60921856	0	0	0	A
9	TIGRP2P131125_rs8504521	60936664	0	0	0	G
9	BICF2P1110530	60938589	G	0	0	A
9	BICF2S22932540	60957697	A	0	0	G
9	BICF2P843982	60972108	0	0	0	A
9	BICF2P831739	60992525	C	0.25	0.2857	A
9	BICF2G630475462	60996738	A	0.25	0.2857	G
9	BICF2G630475471	61008816	A	0.25	0.2857	G
9	BICF2G630475489	61020685	G	0.25	0.2857	A
9	BICF2G630475504	61033184	A	0.25	0.2857	G
9	BICF2S23427549	61048528	A	0	0.25	G
9	BICF2G630475547	61081601	A	0.75	0.7857	G
9	BICF2G630475569	61090623	A	0	0	G
9	BICF2S22949677	61104205	G	0.25	0.2857	A
9	BICF2G630475587	61120051	G	0.25	0.2857	C

9	BICF2G630475589	61130438	C	0.25	0.2857	A
9	BICF2P219066	61147779	G	0.25	0.2143	A
9	BICF2G630475601	61165936	G	0	0	A
9	BICF2G630475607	61179918	A	0	0	G
9	BICF2G630475612	61184466	A	0.25	0.1429	G
9	BICF2G630475616	61187625	A	0.5	0.5714	C
9	BICF2G630475631	61208532	A	0	0	G
9	BICF2G630475642	61216454	G	0.25	0.2143	A
9	BICF2P401276	61233355	G	0.75	0.7143	A
9	BICF2P860518	61246641	A	0.5	0.5714	G
9	BICF2S22917420	61257868	G	0.75	0.7143	A
9	BICF2P879578	61279690	0	0	0	G
9	BICF2G630475701	61287467	G	0.5	0.6429	A
9	BICF2G630475721	61302357	C	0.5	0.6429	A
9	BICF2G630475754	61318277	G	0.5	0.6667	A
9	BICF2G630475781	61334682	G	0.5	0.6429	A
9	BICF2G630475802	61352236	A	0.5	0.6429	G
9	BICF2G630475813	61363708	G	0.5	0.6429	A
9	BICF2G630475821	61367136	C	1	1	A
9	BICF2G630475828	61380350	0	0	0	G
9	BICF2G630475834	61392293	A	0.5	0.6429	G
9	BICF2G630475843	61403557	C	0.5	0.6429	A
9	BICF2G630475850	61429180	A	0.5	0.6429	G
9	BICF2S23614871	61438740	A	0	0	G
9	BICF2P841367	61458059	0	0	0	G
9	BICF2S24510428	61480722	C	0	0	A
9	BICF2P1288572	61506480	G	0.5	0.3571	A
9	BICF2G630475869	61518474	G	0	0	A
9	BICF2G630475883	61535047	T	0.5	0.6429	A
9	BICF2G630475894	61541351	A	0	0	G
9	BICF2G630475934	61560080	A	0	0	G
9	BICF2G630475940	61572751	G	0	0	A
9	BICF2G630475945	61583211	A	0	0	C
9	BICF2G630475953	61597841	A	0	0	G
9	BICF2G630475958	61626885	A	0	0	C
9	BICF2G630475968	61639071	C	0.5	0.3571	A
9	BICF2G630475972	61644155	G	0	0	A
9	TIGRP2P131182_rs8718582	61672392	0	0	0	A
9	BICF2P554518	61731030	A	0	0	C
9	BICF2G630476029	61747616	G	0.75	0.7857	A
9	BICF2G630476041	61757352	G	0.75	0.7857	A
9	BICF2G630476054	61770161	A	0.75	0.7857	G
9	BICF2S23010931	61780156	A	0.75	0.7857	G
9	BICF2S24512114	61787312	A	0.25	0.2143	G
9	BICF2S2339425	61793521	G	0.25	0.1429	A
9	BICF2P857127	61805790	C	0	0	G
9	BICF2P488658	61822564	G	0	0	A

9	BICF2P526898	61827753	A	0.25	0.2143	C
9	BICF2S23252483	61836459	G	0	0	A
9	BICF2P668937	61859907	G	0.25	0.1429	A
9	BICF2P1034889	61870656	G	0.25	0.1429	A
9	BICF2P1219204	61889785	A	0.25	0.1429	G
9	BICF2P1219205	61890187	A	0	0	C
9	BICF2P1387455	61896425	A	0.25	0.08333	G
9	TIGRP2P131186_rs9134849	61909408	0	0	0	A
9	BICF2P892546	61922935	0	0	0	G
9	BICF2P1007832	61947941	A	0.25	0.1429	C
9	BICF2P1023032	61956409	G	0	0	A
9	BICF2S23751854	61963138	G	0.5	0.3571	A
9	BICF2P942513	61976428	A	0.75	0.8571	C
9	BICF2P442659	61991991	A	0	0	G
9	BICF2S23740010	62000971	A	0	0	G
9	BICF2P172882	62011198	C	0.5	0.3571	A
9	BICF2P577665	62029237	G	1	1	A
9	BICF2P1367414	62035722	G	0.25	0.1429	A
9	BICF2S2318113	62055077	G	1	1	C
9	BICF2P677062	62065607	A	0.25	0.1429	G
9	BICF2P1408713	62080310	A	0.5	0.3571	G
9	BICF2P1125794	62084071	G	0.5	0.3571	A
9	BICF2S2448388	62095482	A	0.25	0.1429	G
9	TIGRP2P131205_rs8581206	62108795	0	0	0	G
9	BICF2P479734	62113864	G	0.5	0.6429	A
9	BICF2P590119	62132027	T	0	0	A
9	BICF2S22947331	62194807	A	1	1	C
9	BICF2P1254422	62221478	A	0.25	0.1429	G
9	TIGRP2P131210_rs8815493	62249943	0	0	0	C
9	BICF2P1184941	62261516	A	0	0	G
9	BICF2P594811	62279072	G	0	0	A
9	BICF2P211557	62296204	A	0.25	0.1429	C
9	BICF2P111143	62316810	0	0	0	A
9	BICF2P1137754	62338264	A	0	0	G
9	BICF2P1465585	62349313	G	1	1	A
9	BICF2P241925	62356379	G	1	1	A
9	BICF2P275124	62366758	A	0.25	0.1429	G
9	BICF2S23151173	62378346	G	0.25	0.1429	A
9	BICF2P804485	62388016	A	0	0	C
9	BICF2P533873	62419496	A	0	0	G
9	BICF2P1354989	62423191	A	0	0	G
9	BICF2P336183	62434222	G	0	0	A
9	BICF2S23214162	62457508	G	0.25	0.08333	A
9	BICF2S23058835	62485333	A	0.25	0.1429	G
9	BICF2S22925190	62496655	A	0	0	G
9	BICF2P1244919	62537443	G	0.5	0.6429	A
9	BICF2S23318442	62550458	A	0.75	0.8571	C

9	BICF2P1035972	62580730	A	0	0	T
9	BICF2P339097	62600155	0	0	0	A
9	BICF2P711670	62607966	G	0.75	0.7857	A
9	BICF2S23241552	62634002	A	0.25	0.1429	G
9	BICF2P173450	62642373	G	0.25	0.1429	A
9	BICF2P834997	62655595	0	0	0	C
9	BICF2S23354605	62664146	G	0.75	0.8571	A
9	BICF2P1169801	62676110	G	0.5	0.3571	A
9	BICF2P444657	62697894	A	0.5	0.6429	G
9	BICF2S23419992	62699241	A	0	0	C
9	BICF2P275657	62699687	A	0	0	G
9	BICF2P391450	62702099	A	0	0	C
9	BICF2P1141392	62708275	G	0.5	0.3571	A
9	BICF2P456015	62718791	A	0	0	G
9	BICF2P8836	62721469	A	0.25	0.1429	G
9	BICF2P138704	62722297	A	0.25	0.1429	G
9	BICF2P773235	62742174	A	0	0	C
9	BICF2P1317490	62749749	A	0.5	0.3333	G
9	BICF2P551570	62766950	G	0	0	A
9	BICF2S22939204	62770600	A	0.5	0.3571	G
9	BICF2P1204317	62787422	A	0.5	0.6429	G
9	BICF2S23140624	62820305	G	0	0	A
9	BICF2P1150053	62833704	A	0	0	G
9	BICF2P208510	62837413	A	0	0	G
9	BICF2P895809	62850710	A	0	0	G
9	BICF2S24411831	62881094	A	0.75	0.7857	T
9	BICF2P570235	62885483	G	0.75	0.7857	A
9	TIGRP2P131232_rs8560276	62897747	0	0	0	G
9	BICF2P1275206	62912412	G	0	0	A
9	BICF2P1036668	62933053	C	0	0	A
9	BICF2P499479	62940251	A	0.75	0.7857	G
9	BICF2S23339686	62952752	G	0.75	0.7857	A
9	BICF2P451572	62963237	G	0.75	0.7857	A
9	BICF2P339627	62975576	0	0	0	A
9	BICF2P306997	62990047	G	0.75	0.7857	A
9	BICF2P571018	63000023	T	0.5	0.75	A
9	BICF2S23544843	63005850	A	0.75	0.7857	G
9	BICF2P1315531	63018557	A	0.75	0.7857	G
9	BICF2P1315529	63020134	A	0.75	0.7857	G
9	BICF2P769927	63036553	G	0.75	0.7857	A
9	TIGRP2P131238_rs8866377	63064417	0	0	0	C
9	BICF2P857121	63066454	C	0.5	0.6429	A
9	BICF2S23018085	63093866	0	0	0	A
9	BICF2S23720049	63110070	G	0	0	A
9	BICF2P516827	63136182	A	0.75	0.7857	G
9	TIGRP2P131239_rs9249613	63176740	0	0	0	A
9	BICF2P496854	63190450	C	0	0	G

9	TIGRP2P131240_rs8705366	63193683	0	0	0	A
9	BICF2P767735	63211751	A	0	0	G
9	BICF2P483675	63217956	G	0	0	A
9	BICF2S23763974	63228598	A	0.75	0.8571	T
9	BICF2P499260	63242849	G	0	0	A
9	BICF2P688771	63252436	A	0	0	G
9	BICF2P5121	63263215	G	0	0	A
9	BICF2S23027638	63275368	C	0	0	A
9	BICF2S2301897	63288552	A	0	0	G
9	BICF2P507534	63305645	A	0	0	G
9	BICF2P254905	63307743	A	0	0	G
9	BICF2P274905	63325723	G	0	0	A
9	BICF2P260055	63330521	A	0	0	G
9	BICF2P858916	63347690	A	0.25	0.1429	G
9	BICF2P745440	63358550	A	0.75	0.7857	G
9	BICF2P289334	63365059	A	0	0	G
9	BICF2S2375842	63391164	G	0.25	0.2143	A
9	BICF2P665182	63399945	G	0.25	0.2143	A
9	BICF2S2375112	63419693	A	0	0	G
9	BICF2P1502710	63444591	A	0	0	G
9	BICF2P1502697	63446795	A	0	0	G
9	BICF2S2314648	63486227	G	0.25	0.2143	A
9	BICF2S23651600	63501302	A	0.25	0.2143	G
9	BICF2S23651869	63506784	C	0	0	A
9	BICF2P336253	63527655	G	0.25	0.2143	A
9	BICF2P164591	63539695	A	0	0	G
9	BICF2P1436914	63552659	A	0	0	G
9	BICF2P664023	63582976	A	0	0	C
9	BICF2P188949	63585508	C	0	0	A
9	BICF2P622477	63595485	G	1	1	A
9	BICF2P280739	63616495	A	0.25	0.2143	C
9	BICF2P273940	63619709	A	0	0	G
9	BICF2P138438	63654209	0	0	0	A
9	BICF2P788470	63679563	A	0.75	0.75	G
9	BICF2S23450786	63694683	G	0.75	0.75	A
9	BICF2P1377975	63707546	T	0	0	A
9	BICF2P1377968	63710346	A	0	0	G
9	BICF2S23729998	63725540	A	0.25	0.2143	T
9	BICF2P781977	63731570	G	1	1	A
9	BICF2S2376623	63742629	A	0	0	G
9	BICF2S23113145	63751107	G	0	0	A
9	BICF2P366822	63764776	0	0	0	A
9	BICF2S22943825	63778763	G	1	1	A
9	BICF2P815027	63783649	C	0	0	A
9	BICF2S23038667	63793640	A	0	0	G
9	BICF2P608382	63807670	A	0	0	G
9	BICF2P230855	63819564	A	0	0	G

9	BICF2P4021	63825860	G	0.25	0.2143	A
9	BICF2P1065919	63841856	A	0.25	0.2143	G
9	BICF2S22955558	63844383	A	0	0	T
9	BICF2P531655	63859709	G	0	0	C
9	BICF2P172267	63861430	G	0	0	A
9	BICF2P157169	63878080	G	0	0	A
9	BICF2P737404	63893397	G	0	0	A
9	BICF2S23351166	63915400	A	0.75	0.7857	G
9	BICF2P1426962	63927263	C	1	1	A
9	BICF2P1215930	63940334	G	0.25	0.2143	A
9	BICF2S23517758	63956195	A	0.25	0.2143	G
9	BICF2P175089	63974132	G	0	0	A
9	BICF2P1388775	63978201	A	0	0	G
9	BICF2P496029	63989178	G	0.75	0.7857	A
9	BICF2S23315396	63996464	A	1	1	G
9	BICF2P1255162	64005213	G	0	0	C
9	BICF2P1308758	64028163	A	0	0	C
9	BICF2P1237291	64036773	A	0	0	C
9	BICF2P75345	64049336	G	0.25	0.2143	A
9	BICF2P318266	64067804	0	0	0	C
9	BICF2S23257502	64079492	A	0.25	0.2143	G
9	BICF2P503630	64085875	A	0.75	0.7857	G
9	BICF2S22951498	64110008	G	1	1	A
9	BICF2P762001	64113986	G	0	0	A
9	BICF2P749766	64114906	A	0	0	G
9	BICF2P1076319	64135387	G	1	1	A
9	BICF2S23013098	64154311	G	1	1	A
9	BICF2S2343839	64159693	A	0	0	G
9	BICF2S23143582	64192318	A	1	1	G
9	BICF2S23313343	64215439	A	1	1	G
9	BICF2S2303872	64222745	A	0	0	G
9	BICF2S2364920	64236590	G	0	0	A
9	BICF2S23033667	64249008	G	0.25	0.3571	A
9	BICF2P500920	64261863	G	0	0	C
9	BICF2P1273502	64263398	C	0.25	0.3571	A
9	BICF2P365656	64280627	C	0.25	0.3571	A
9	BICF2P1056460	64284397	A	0	0	T
9	BICF2P271019	64286178	A	0	0	G
9	BICF2P540492	64298434	A	0	0	C
9	BICF2P406965	64309788	C	0.25	0.3333	A
9	BICF2P1321133	64319694	A	0	0	G
9	BICF2S23655993	64338592	G	0	0	A
9	BICF2P317394	64350642	A	0.25	0.3571	G
9	BICF2P562174	64371122	A	0.25	0.3571	G
9	BICF2P1094627	64381802	G	0.75	0.6429	A
9	BICF2S23420422	64388646	A	0.75	0.6429	G
9	BICF2P1362601	64413380	G	0.5	0.6429	A

10	BICF2P313724	3032598	0	0	0	G
10	BICF2S23551676	3056466	A	0.25	0.5	G
10	BICF2S23216909	3081933	A	0.25	0.5	G
10	BICF2S23122060	3106639	G	0.25	0.5	A
10	BICF2P80152	3124161	G	0	0	A
10	BICF2S23015397	3138788	G	0.25	0.5	A
10	BICF2P414983	3146976	0	0	0	A
10	TIGRP2P137401_rs9172327	3171783	0	0	0	A
10	TIGRP2P137417_rs8746526	3218635	0	0	0	A
10	BICF2P1434658	3231166	0	0	0	A
10	BICF2P1185816	3246030	0	0	0	G
10	BICF2P837966	3281945	0	0	0	C
10	BICF2S23538422	3300301	A	0.25	0.5	G
10	BICF2S23624594	3305479	G	NA	0.6667	A
10	BICF2S23153666	3336514	C	0.25	0.5	A
10	BICF2S23134339	3340566	G	0.25	0.5	A
10	BICF2S2319425	3349524	G	0.25	0.5	A
10	BICF2P946036	3386794	0	0	0	C
10	BICF2S24312362	3458571	0	0	0	A
10	BICF2P1351813	3467311	0	0	0	A
10	BICF2P1106789	3496232	0	0	0	A
10	BICF2P1454205	3508776	A	0	0	G
10	BICF2P1293725	3530354	G	0	0	A
10	BICF2G630668982	3541673	0	0	0	G
10	BICF2P1303102	3605262	0	0	0	A
10	TIGRP2P137429_rs9132915	3628540	0	0	0	G
10	BICF2S23150087	3641119	A	0	0.2857	C
10	BICF2P948311	3696837	0	0	0	A
10	BICF2P201150	3708761	0	0	0	C
10	BICF2P493671	3726987	0	0	0	A
10	BICF2S23332816	3750954	C	0.25	0.5714	A
10	BICF2P708795	3810086	0	0	0	G
10	BICF2P742979	3821336	0	0	0	G
10	BICF2P279756	3856911	0	0	0	G
10	BICF2P330484	3869076	0	0	0	C
10	BICF2P881426	3883833	0	0	0	A
10	BICF2P205401	3931216	0	0	0	G
10	BICF2P1184169	3936815	0	0	0	A
10	BICF2P784053	3992549	0	0	0	A
10	TIGRP2P137445_rs8572249	4011766	0	0	0	A
10	BICF2S23538703	4045506	A	0.25	0.2143	G
10	BICF2P352207	4074917	0	0	0	C
10	BICF2P841430	4098267	A	0	0	G
10	TIGRP2P137447_rs8595942	4105629	0	0	0	C
10	BICF2S23136899	4120842	C	0.25	0.5714	A
10	BICF2P1390665	4161060	A	0.25	0.2143	G
10	BICF2P197443	4184379	0	0	0	A

10	BICF2P168510	4191591	0	0	0	T
10	BICF2P579099	4215158	C	0.25	0.2143	A
10	BICF2P887362	4247584	0	0	0	A
10	BICF2P639742	4255193	0	0	0	G
10	BICF2P442343	4280872	0	0	0	C
10	BICF2P179897	4294415	0	0	0	T
10	BICF2P323402	4305790	A	0.25	0.2143	G
10	BICF2P261885	4316713	G	1	1	A
10	BICF2P1325941	4330293	0	0	0	G
10	BICF2P993521	4343827	G	0.5	0.7857	A
10	BICF2P194336	4352815	A	0	0	G
10	BICF2S23134693	4363262	G	0.25	0.2143	A
10	BICF2P922372	4375664	0	0	0	G
10	BICF2P1272851	4387646	A	0.25	0.1667	G
10	BICF2P1389324	4412270	A	0.25	0.2143	G
10	BICF2P607403	4420879	A	0.5	0.7857	C
10	BICF2P523018	4514991	0	0	0	T
10	BICF2P158858	4522331	A	0	0	G
10	BICF2P574744	4539423	A	0	0	G
10	BICF2P1017367	4553741	A	0.25	0.2143	G
10	BICF2P943825	4561110	G	0.5	0.5	A
10	BICF2S2373644	4574924	C	0.5	0.5	G
10	BICF2P829676	4591597	A	0.5	0.5	C
10	BICF2P1256476	4603245	0	0	0	G
10	BICF2P722117	4641732	G	0.75	0.7143	A
10	BICF2S22951569	4647897	A	0	0	G
10	BICF2P473748	4679395	0	0	0	C
10	BICF2S2317257	4703539	A	0.25	0.2143	G
10	BICF2P119147	4748024	0	0	0	A
10	BICF2P436418	4770034	0	0	0	C
10	BICF2P616702	4783832	0	0	0	C
10	BICF2S23416561	4806236	0	0	0	G
10	BICF2S236415	4814858	C	0.75	0.4286	G
10	BICF2P148333	4823856	0	0	0	C
10	BICF2P38412	4850586	0	0	0	C
10	BICF2P476523	4903176	A	0.25	0.2143	G
10	BICF2P35992	4969402	0	0	0	G
10	TIGRP2P141672_rs9042783	5016842	0	0	0	C
10	BICF2S23738174	5035631	A	0	0	G
10	BICF2S2459053	5041986	T	0.5	0.2143	A
10	BICF2P1364926	5070889	C	0	0	A
10	BICF2S23258301	5076527	G	0.5	0.2143	A
10	BICF2S23041659	5094073	G	0	0	A
10	BICF2P701493	5111375	C	0.5	0.2143	A
10	BICF2P544902	5117529	0	0	0	G
10	BICF2P147652	5149632	G	0	0	A
10	BICF2P1213982	5157275	G	0.5	0.2143	A

10	BICF2P633408	5171364	A	0	0	C
10	BICF2P80262	5187627	G	0	0	A
10	TIGRP2P141678_rs9140022	5206563	0	0	0	G
10	BICF2S23211324	5210568	A	0	0	G
10	BICF2P447413	5221427	G	1	1	A
10	BICF2P623559	5240223	0	0	0	A
10	BICF2S23322314	5245184	A	0.25	0.5833	G
10	TIGRP2P141679_rs8678837	5263947	0	0	0	A
10	G567f76S152	5280491	0	0	0	A
10	BICF2P914978	5317253	0	0	0	A
10	BICF2S23234748	5342778	0	0	0	A
10	BICF2S234970	5356795	A	0.25	0.2857	G
10	BICF2S23642724	5365025	A	0	0	G
10	BICF2P216482	5383501	G	0	0	A
10	BICF2P582948	5400556	0	0	0	C
10	BICF2S2364052	5412951	A	0.5	0.2143	G
10	BICF2S2363180	5417711	C	0.5	0.2143	G
10	G568f47S333	5440236	G	1	1	A
10	BICF2P1400503	5462847	G	1	1	A
10	BICF2S23118257	5479767	G	0.75	0.7143	A
10	BICF2S23212054	5504458	0	0	0	A
10	G569f40S136	5526108	0	0	0	C
10	BICF2S2329781	5547006	G	0	0.25	A
10	BICF2S23244904	5555944	A	0	0.2857	G
10	BICF2P829794	5585060	G	0.5	0.2143	A
10	BICF2S23055939	5594959	G	0.25	0.5	A
10	BICF2P1162538	5626769	A	0	0	G
10	BICF2S23137919	5638637	G	0.25	0.5	A
10	BICF2P950178	5647387	0	0	0	C
10	BICF2P1203829	5679587	A	0	0	G
10	TIGRP2P141684_rs9063255	5767559	0	0	0	C
10	BICF2S23132431	5793484	0	0	0	G
10	BICF2P658083	5810341	0	0	0	G
10	BICF2S2397788	5826381	G	0.25	0.2143	A
10	BICF2S23247158	5849369	A	0	0	C
10	BICF2P845994	5857355	0	0	0	A
10	BICF2P625228	5871628	0	0	0	A
10	G570f26S182	5894741	C	0	0	A
10	BICF2P1429709	5922150	A	0	0.2857	G
10	BICF2P625960	5955346	0	0	0	A
10	BICF2P92143	5968131	G	0.5	0.2143	A
10	BICF2P791915	5979022	A	0	0.2857	G
10	BICF2P116176	5981460	0	0	0	A
10	G571f39S207	6017585	G	0	0	A
10	BICF2P804801	6056190	0	0	0	A
10	G572f16S141	6075468	A	0	0	C
10	BICF2P295619	6097741	0	0	0	A

10	BICF2P1109267	6151102	0	0	0	A
10	BICF2P763613	6193573	A	0.5	0.2143	G
10	BICF2S23147241	6204409	0	0	0	A
10	BICF2P732279	6214322	0	0	0	C
10	BICF2P1151381	6225107	0	0	0	A
10	BICF2S23737954	6238255	G	0	0	A
10	BICF2P695456	6271976	0	0	0	G
10	BICF2P106504	6283096	0	0	0	A
10	BICF2S23011067	6324274	A	1	1	G
10	BICF2S23019630	6328084	A	1	1	C
10	BICF2P815464	6360004	A	0	0.2857	G
10	BICF2P1153895	6376080	0	0	0	A
10	TIGRP2P141690_rs8859765	6386556	0	0	0	T
10	BICF2P966646	6403616	A	0	0.2857	G
10	BICF2P605692	6420775	0	0	0	G
10	BICF2P1402317	6429379	0	0	0	A
10	BICF2P1042608	6451339	0	0	0	G
10	BICF2P745302	6468895	C	0.5	0.2143	A
10	BICF2P471905	6477302	A	0.5	0.2143	G
10	BICF2P792320	6487243	0	0	0	A
10	BICF2P570770	6505892	0	0	0	G
10	BICF2P626487	6514569	A	0	0.2857	G
10	BICF2P310876	6546722	0	0	0	G
10	BICF2S23449565	6569942	A	0	0	G
10	BICF2P230089	6618495	0	0	0	A
10	BICF2P720099	6627986	0	0	0	G
10	BICF2P1153846	6653334	0	0	0	A
10	TIGRP2P141694_rs8941482	6678560	0	0	0	C
10	TIGRP2P141695_rs9058091	6702961	0	0	0	G
10	BICF2P1152234	6733319	A	1	0.5	G
10	BICF2S23512222	6743123	C	0.75	0.7143	A
10	BICF2P405279	6756422	0	0	0	A
10	BICF2P137721	6816827	0	0	0	T
10	BICF2P617378	6840140	0	0	0	G
10	BICF2P457981	6873334	A	0.5	0.2143	G
10	BICF2P1011248	6881097	A	0.5	0.2143	C
10	G576f19S182	6909946	0	0	0	C
10	BICF2P1304549	6929930	0	0	0	A
10	BICF2S23914906	6940773	0	0	0	G
10	BICF2P612307	6979147	0	0	0	A
10	BICF2P57449	7007938	0	0	0	G
10	BICF2P435342	7024301	A	0.75	0.4167	C
10	BICF2S23341290	7033157	A	1	0.4167	G
10	BICF2P1043894	7040245	G	0.75	0.4286	A
10	BICF2P1436032	7053431	C	0.75	0.4286	A
10	BICF2S23753647	7068324	A	0.75	0.4286	G
10	BICF2P730806	7079775	A	0.75	0.4286	G

10	BICF2S23036394	7104919	A	0	0	G
10	BICF2S23035356	7108514	G	0	0	A
10	BICF2S236342	7130361	A	0	0	C
10	BICF2S23251143	7170176	G	1	0.7143	A
10	BICF2P365623	7197759	0	0	0	A
10	BICF2P727174	7200056	0	0	0	C
10	BICF2P338824	7261865	G	0.25	0.5	A
10	BICF2P340141	7273131	A	0.25	0.5	G
10	BICF2P703253	7283733	G	0.25	0.5	A
10	BICF2P1222750	7294685	A	0.25	0.5	G
10	BICF2S2336724	7312004	A	0.5	0.7857	G
10	BICF2P1080176	7315649	G	0.5	0.7857	A
10	BICF2P842214	7326577	0	0	0	G
10	BICF2P592377	7347348	G	0.5	0.7857	A
10	BICF2P132156	7358196	G	0.5	0.7857	A
10	BICF2P1006276	7360093	C	0.5	0.7857	A
10	BICF2P1408492	7372731	C	0.5	0.7857	A
10	BICF2P918645	7390289	G	0.5	0.7857	A
10	BICF2P1389331	7399630	A	0.5	0.2143	G
10	BICF2P1341850	7409512	A	0.5	0.2143	G
10	TIGRP2P141701_rs8992111	7427439	0	0	0	A
10	BICF2S23047160	7446537	G	0.5	0.2143	A
10	BICF2P1376145	7454554	G	0.5	0.2143	C
10	BICF2P1092036	7467901	A	0.5	0.2143	T
10	BICF2S23050064	7477509	C	0.5	0.2143	A
10	BICF2S23423449	7505950	0	0	0	G
10	BICF2P535024	7512753	G	0.5	0.2143	A
10	BICF2P181213	7528471	A	0.5	0.2143	G
10	BICF2P49374	7558555	G	0.5	0.2143	A
10	BICF2P304506	7572784	C	0.5	0.2143	A
10	BICF2P1093470	7621497	G	0.5	0.2143	A
10	BICF2P925150	7625680	A	0.5	0.2143	G
10	BICF2P1105131	7650089	G	0.25	0.5	A
10	BICF2P778791	7665221	A	0.25	0.5	G
10	BICF2S23355174	7676100	A	0.5	0.1667	G
10	BICF2P5108	7685308	A	0.5	0.2143	G
10	BICF2P1270711	7694400	G	0.25	0.5	A
10	BICF2P256839	7716926	C	0.25	0.5	A
10	BICF2P653041	7735333	G	0.25	0.5	A
10	BICF2P813570	7744387	G	0.25	0.5	A
10	BICF2S23624813	7759002	A	0.25	0.5	G
10	BICF2P488258	7768560	G	0	0	A
10	BICF2P86991	7777208	A	0	0	C
10	BICF2P190315	7792968	G	0	0	A
10	BICF2S2444424	7805503	G	0.25	0.5	A
10	BICF2P295148	7813138	A	0	0	G
10	BICF2P197953	7822004	G	0	0	A

10	BICF2P373329	7845331	A	0	0	G
10	G577f35S36	7866732	0	0	0	C
10	BICF2P721345	7898160	0	0	0	A
10	TIGRP2P141709_rs8684254	7905937	0	0	0	A
10	BICF2P1412359	7927964	G	0.75	0.5	A
10	BICF2P1104685	7938435	0	0	0	A
10	BICF2P1171081	7949390	G	0.25	0.5	A
10	BICF2S23129706	7966915	A	0.5	0.7857	G
10	BICF2P185665	7971079	A	0.5	0.7857	G
10	BICF2P484831	7987200	A	1	1	G
10	BICF2P900588	7996352	A	1	1	G
10	BICF2S23444133	8009531	A	1	1	C
10	BICF2P395308	8019967	G	1	1	C
10	BICF2P599664	8028239	A	1	1	G
10	BICF2P896379	8070007	0	0	0	A
10	BICF2P635077	8082416	G	1	1	A
10	TIGRP2P141711_rs8543433	8090316	0	0	0	G
10	BICF2P768240	8109634	0	0	0	G
10	BICF2P1112049	8145514	0	0	0	A
10	BICF2P36174	8176609	A	0	0	G
10	BICF2P1424159	8226957	0	0	0	A
10	BICF2P1042684	8257992	0	0	0	A
10	G578f71S155	8289828	0	0	0	G
10	BICF2P748308	8324159	0	0	0	A
10	BICF2P1390597	8341246	C	0.25	0.5	A
10	BICF2P753483	8365103	G	0.75	0.7143	A
10	BICF2P753481	8365396	A	0.75	0.7143	G
10	BICF2P1144330	8376408	A	0.25	0.5	C
10	BICF2S22957387	8399169	G	0.75	0.7143	A
10	BICF2S23052387	8424072	G	0	0	A
10	BICF2P1153020	8439966	G	0.25	0.5	A
10	BICF2P661940	8441871	G	0.25	0.5	A
10	BICF2P661941	8442370	A	0.25	0.5	T
10	BICF2P344383	8479592	C	0.5	0.7857	A
10	BICF2P1433333	8514903	T	0.5	0.7857	A
10	BICF2P527987	8552413	G	0.5	0.7857	A
10	BICF2P1393451	8586698	0	0	0	A
10	BICF2P318489	8602431	G	0.25	0.5	A
10	BICF2P1127836	8620616	C	0.5	0.2143	A
10	BICF2S234906	8625145	G	0	0	A
10	BICF2P1213939	8653746	G	0.5	0.7857	C
10	BICF2P1119320	8697455	0	0	0	A
10	BICF2P606582	8722311	0	0	0	A
10	BICF2S23642337	8731792	A	0	0	G
10	TIGRP2P141723_rs8788080	8760845	0	0	0	A
10	BICF2P550486	8797787	0	0	0	G
10	BICF2S23145890	8810412	A	0	0	G

10	BICF2P919122	8821729	A	0.25	0.5	G
10	BICF2S23760446	8834134	G	0.5	0.2143	A
10	BICF2P346061	8862961	G	0.5	0.2143	A
10	BICF2P1401556	8869043	C	0.5	0.7857	G
10	BICF2P1167620	8879364	G	0.5	0.7857	A
10	BICF2P520735	8909524	G	0.5	0.7857	A
10	BICF2P592315	8931274	G	0.5	0.7857	A
10	BICF2P918283	8947418	G	0.5	0.7857	A
10	BICF2P492925	8965567	G	0.25	0.5	C
10	BICF2P440827	8974471	A	0.5	0.7857	T
10	BICF2P194965	8990289	G	0.5	0.7857	A
10	BICF2P1053820	8999303	C	0.5	0.7857	G
10	BICF2P110204	9011292	0	0	0	G
10	BICF2P1170295	9017398	G	0.5	0.7857	A
10	BICF2P169855	9030861	A	0.5	0.7857	G
10	BICF2P169854	9031116	A	0.5	0.7857	T
10	BICF2P248110	9040255	A	0.25	0.7143	G
10	BICF2P1433877	9050992	G	0.5	0.7857	A
10	BICF2S24110485	9075132	0	0	0	G
10	BICF2P130531	9089913	0	0	0	A
10	BICF2S23654391	9117729	A	0.5	0.2143	G
10	BICF2P1359538	9136638	G	0.25	0.5	A
10	BICF2P895944	9158211	A	0.5	0.2143	G
10	BICF2P916070	9189039	G	0.25	0.5	A
10	BICF2P594450	9207359	C	0.25	0.5	A
10	BICF2P571004	9212170	G	0.25	0.5	A
10	BICF2P460467	9223107	G	0.25	0.5	A
10	BICF2P1058753	9242091	G	0.75	0.7143	A
10	BICF2P1058754	9242137	G	0.75	0.7143	A
10	BICF2P255973	9251468	A	0.25	0.5	C
10	BICF2P422653	9260727	A	0.25	0.5	G
10	BICF2P509361	9271106	A	0.25	0.5	G
10	BICF2P891226	9290612	0	0	0	A
10	BICF2P730497	9314870	G	0	0.2857	A
10	BICF2P1166761	9354049	0	0	0	A
10	BICF2P790614	9377816	A	0.5	0.2143	G
10	BICF2P1419982	9410031	0	0	0	A
10	TIGRP2P141734_rs8917883	9426800	0	0	0	A
10	BICF2S2441418	9445119	0	0	0	C
10	BICF2P196568	9467560	0	0	0	A
10	BICF2P45452	9506695	A	0.75	0.4286	G
10	BICF2P161973	9541186	0	0	0	A
10	BICF2P555215	9547807	A	1	0.7143	G
10	BICF2P484456	9577391	A	0	0.2857	G
10	BICF2P947771	9585333	G	1	0.75	A
10	BICF2P976577	9592333	A	1	0.75	C
10	BICF2P1056634	9628651	A	0	0.2857	G

10	BICF2P945408	9663107	A	0	0.2857	G
10	BICF2S23762663	9698893	A	1	1	C
10	BICF2P1040109	9713960	C	0.75	0.4286	A
10	BICF2P475438	9726873	A	0	0	G
10	BICF2P1154532	9738187	A	1	1	G
10	BICF2P605914	9744554	A	1	1	G
10	BICF2S23046151	9748801	A	1	1	G
10	BICF2P145764	9761041	G	1	1	A
10	BICF2P120884	9785815	0	0	0	G
10	BICF2S23121107	9790066	G	0	0	A
10	BICF2P571652	9802000	A	0.75	0.4286	G
10	BICF2S22913956	9818874	A	0.75	0.4286	G
10	BICF2P532643	9836009	G	0.5	0.7857	A
10	BICF2P367457	9848655	0	0	0	A
10	BICF2P927336	9859738	0	0	0	G
10	BICF2S23715357	9880899	A	0.5	0.7857	C
10	BICF2P1459668	9904595	A	0	0	G
10	BICF2P1318411	9933314	A	0.5	0.2143	G
10	BICF2P193161	9945670	0	0	0	C
10	BICF2P1456428	9969503	0	0	0	A
10	BICF2P464350	9972656	A	0.5	0.2143	G
10	BICF2P233183	10007965	0	0	0	G
10	G579f41S199	10039130	A	0	0	C
10	G579f42S265	10040438	A	0.25	0.5714	G
10	BICF2P251568	10060618	A	0.25	0.2143	G
10	BICF2P586886	10097539	0	0	0	A
10	BICF2P533704	10155349	G	0.25	0.2143	A
10	BICF2P5518	10211706	G	0.5	0.7857	A
10	BICF2P891420	10216377	0	0	0	C
10	BICF2P391159	10245655	0	0	0	C
10	BICF2P1225262	10265925	G	0.5	0.2143	A
10	BICF2P809657	10279174	G	0.25	0.2143	A
10	BICF2P776045	10281069	A	0.5	0.9	G
10	BICF2P699421	10302292	G	0.5	0.7857	A
10	BICF2P1164988	10322338	G	0.5	0.7857	A
10	BICF2P1254712	10333372	C	0.5	0.7857	G
10	BICF2P1064035	10340230	A	0.5	0.7857	G
10	BICF2P164833	10380958	0	0	0	A
10	BICF2P53899	10393318	A	0.5	0.7857	G
10	BICF2P154098	10403688	A	0.5	0.7857	G
10	BICF2P408346	10412216	A	0.5	0.7857	G
10	TIGRP2P137479_rs9137591	10424137	0	0	0	A
10	BICF2P1121174	10446660	0	0	0	C
10	BICF2P540844	10462369	C	0	0	A
10	BICF2P163800	10466997	0	0	0	C
10	BICF2S23712785	10485510	A	0	0	G
10	BICF2S22951426	10491306	A	0.5	0.2143	G

10	BICF2S22912683	10500857	G	0.25	0.2143	A
10	BICF2P1371294	10506272	A	0.25	0.5714	G
10	BICF2P446404	10511720	C	0.5	0.2143	A
10	BICF2P1219487	10526832	A	0.5	0.1667	C
10	BICF2P1370648	10539991	G	0.5	0.2143	A
10	BICF2P1081679	10549505	G	0.5	0.2143	A
10	BICF2P895698	10566659	A	0.5	0.2143	G
10	BICF2S23239961	10576266	G	0.5	0.2143	A
10	BICF2P1392440	10582816	G	0.5	0.2143	A
10	BICF2P1139734	10609565	C	0.5	0.2143	A
10	TIGRP2P137482_rs8651763	10636416	0	0	0	G
10	BICF2P822147	10644758	A	0.75	0.7857	C
10	BICF2P949229	10655233	A	0.75	0.7857	C
10	BICF2P491870	10677936	G	0.75	0.7857	C
10	BICF2P1433273	10692535	A	0.5	0.3	G
10	BICF2P619179	10703666	C	0.5	0.2143	G
10	BICF2P330949	10707193	C	0.25	0.5714	A
10	BICF2P816919	10758379	0	0	0	G
10	BICF2S22960809	10769841	G	0	0	A
10	TIGRP2P137491_rs9078549	10780970	0	0	0	A
10	BICF2S23631212	10791303	0	0	0	A
10	BICF2P797775	10804969	G	0.5	0.2143	A
10	BICF2P556483	10832919	C	0.5	0.2143	G
10	BICF2P592005	10836411	G	0.5	0.2143	A
10	BICF2P956151	10844974	A	0.25	0.5714	G
10	BICF2S23446932	10859628	G	0	0	A
10	BICF2P712340	10871535	G	0.25	0.5714	A
10	BICF2S24410053	10879097	A	0	0	C
10	BICF2P261508	10899997	0	0	0	A
10	BICF2P86524	10907439	A	0.25	0.5714	G
10	BICF2P567173	10916707	0	0	0	A
10	BICF2P1017211	10934167	0	0	0	A
10	BICF2P1118840	10944541	G	0.5	0.2143	A
10	TIGRP2P137492_rs8775117	10948489	0	0	0	A
10	BICF2S23211087	10979685	G	0.5	0.2143	A
10	BICF2P283272	10983308	A	0.5	0.2143	G
10	BICF2S24414279	11000274	G	0.75	0.7857	A
10	BICF2S23426676	11006041	A	0.75	0.7857	G
10	BICF2S23053016	11017208	G	0.75	0.7857	A
10	BICF2S23012887	11045711	A	0.75	0.7857	C
10	BICF2P718692	11056641	A	0.25	0.5714	G
10	BICF2S23633421	11072007	G	0.75	0.7857	A
10	BICF2P753942	11081762	A	0.25	0.5714	G
10	BICF2P1167464	11086490	A	0.25	0.5714	G
10	BICF2S23444568	11100691	A	0.75	0.8333	G
10	BICF2P668742	11113579	C	0.5	0.2143	A
10	BICF2P884833	11121003	A	0.25	0.5714	G

10	BICF2P723131	11137439	A	0.5	0.2143	G
10	BICF2P263265	11148822	G	0.5	0.2143	A
10	G580f46S240	11169956	A	0	0	G
10	TIGRP2P137497_rs8915750	11190468	0	0	0	C
10	BICF2P466106	11221610	0	0	0	A
10	BICF2P795952	11231464	T	0.5	0.2143	A
10	BICF2P738959	11241077	G	0.5	0.2143	A
10	BICF2P926761	11256796	G	0.75	0.7857	C
10	BICF2P774655	11260040	C	0.75	0.7857	A
10	BICF2P344081	11275742	0	0	0	C
10	BICF2P675135	11286497	G	0.75	0.7857	A
10	TIGRP2P137499_rs9037069	11302747	0	0	0	C
10	BICF2P1210313	11312838	A	0.75	0.7857	G
10	BICF2S23611160	11324248	A	0.75	0.8333	G
10	BICF2P1016611	11334108	A	0.75	0.7857	G
10	BICF2P709352	11384057	A	0.75	0.7857	G
10	BICF2P484070	11389378	A	0.75	0.7857	G
10	BICF2P797505	11405132	C	0.75	0.7857	G
10	BICF2P776298	11440860	G	0.5	0.2143	A
10	BICF2P747041	11461551	0	0	0	G
10	BICF2S22943767	11470246	C	0.25	0.5714	A
10	BICF2S23752626	11532898	A	0.25	0.5714	C
10	TIGRP2P137504_rs8940561	11542831	0	0	0	A
10	BICF2S23342115	11549828	A	0.25	0.5714	G
10	BICF2P1304125	11559700	G	0.25	0.5714	A
10	TIGRP2P137505_rs8943212	11570287	0	0	0	A
10	BICF2P1307818	11611583	0	0	0	G
10	BICF2S23651627	11616330	C	0	0	A
10	BICF2P1340585	11637768	0	0	0	A
10	BICF2P332589	11644756	C	0.25	0.5714	G
10	BICF2P483509	11656441	0	0	0	A
10	BICF2P673774	11677772	0	0	0	A
10	TIGRP2P137507_rs8589300	11698336	0	0	0	A
10	BICF2P1069518	11722019	0	0	0	G
10	BICF2P462764	11758427	G	0	0	A
10	BICF2S23128717	11765012	G	0	0	A
10	BICF2P1260553	11792711	A	0.25	0.5714	G
10	BICF2S23740399	11834786	G	0	0	A
10	BICF2P1034564	11850352	A	0	0	G
10	BICF2P380539	11865459	C	0	0	A
10	BICF2S23435011	11871355	A	0	0	G
10	BICF2P1203203	11906661	0	0	0	A
10	BICF2S2297387	11915602	A	0.25	0.5714	G
10	BICF2P915634	11932455	0	0	0	T
10	TIGRP2P137511_rs8564763	11946384	0	0	0	C
10	BICF2P504911	11951126	G	0.25	0.5714	A
10	BICF2P229118	11961276	G	0.25	0.5714	A

10	BICF2P381746	11976157	0	0	0	G
10	BICF2S23537038	11998853	A	0	0	G
10	BICF2P477614	12017097	A	0.25	0.5714	G
10	BICF2S23012002	12018727	0	0	0	A
10	BICF2S23428022	12029052	A	0.25	0.5714	C
10	BICF2P1179759	12043785	G	0	0	A
10	BICF2P307384	12062017	0	0	0	G
10	BICF2S22930988	12062548	0	0	0	C
10	BICF2P1319955	12063648	G	0	0	A
10	BICF2P582642	12082601	G	0	0	A
10	BICF2P1191449	12087526	A	0	0	G
10	BICF2S23117066	12108015	G	0.25	0.5714	A
10	BICF2P405361	12109168	G	0.25	0.5714	A
10	BICF2P654832	12110791	G	0.25	0.5714	A
10	BICF2P1174414	12134276	A	0	0	G
10	BICF2P842186	12145374	A	0	0	G
10	BICF2P1411885	12146657	G	0.25	0.5714	A
10	BICF2P1194766	12156426	G	0.25	0.5714	A
10	BICF2S23037045	12172500	0	0	0	G
10	BICF2P800331	12186630	A	0	0	G
10	BICF2P216551	12192088	G	0	0	A
10	BICF2P1095774	12211334	A	0.25	0.5714	G
10	BICF2S23037623	12227519	G	0.25	0.5714	A
10	BICF2S23019926	12243693	G	0.25	0.5714	A
10	BICF2S23032891	12248197	A	0.25	0.5714	G
10	BICF2P750514	12263875	A	0	0	C
10	BICF2S22912847	12275358	G	0	0	A
10	BICF2P415301	12296389	G	0	0	A
10	BICF2P808618	12322353	A	0	0	C
10	BICF2S23347592	12335478	G	0	0	A
10	BICF2P622438	12345185	G	0	0	A
10	BICF2P1131455	12360627	A	0	0	G
10	BICF2P1331753	12368783	A	0	0	G
10	BICF2P803293	12378450	G	0	0	A
10	BICF2P989649	12386289	G	0	0	A
10	BICF2P761543	12399179	G	0	0	A
10	BICF2P1125271	12410052	A	0	0	G
10	BICF2P1289476	12438271	A	0.25	0.5714	G
10	BICF2P553784	12446649	C	0	0	A
10	BICF2S2317448	12459480	G	0	0	A
10	BICF2S23113007	12470425	A	0	0	G
10	BICF2G630668957	12477857	T	0.25	0.5714	A
10	TIGRP2P137521_rs9035849	12494770	0	0	0	G
10	BICF2S23554577	12504362	T	0	0	A
10	BICF2G630668947	12520146	G	0.25	0.5714	A
10	BICF2G630668931	12534243	A	0.25	0.5714	C
10	BICF2G630668929	12536977	G	0.75	0.4286	A

10	BICF2S23611584	12552002	A	0	0	G
10	BICF2G630668917	12558537	A	0.75	0.4286	G
10	BICF2P723333	12609082	0	0	0	C
10	TIGRP2P137525_rs8823700	12625366	0	0	0	A
10	BICF2G630668912	12631189	0	0	0	G
10	BICF2G630668908	12647562	0	0	0	G
10	BICF2G630668906	12652002	0	0	0	C
10	BICF2G630668898	12675486	0	0	0	G
10	BICF2G630668880	12690886	G	0	0	A
10	BICF2G630668851	12696221	0	0	0	C
10	BICF2G630668816	12710344	A	0	0	G
10	BICF2P1097262	12723077	A	0	0	G
10	BICF2S23731463	12731854	G	0	0	A
10	BICF2P231676	12751511	0	0	0	A
10	TIGRP2P137531_rs9193334	12763175	0	0	0	C
10	BICF2P757479	12783975	T	0	0	A
10	BICF2S22954174	12801633	G	0.25	0.5714	A
10	BICF2G630668802	12815982	G	0.75	0.4286	A
10	BICF2P1183775	12841620	G	0	0	A
10	BICF2G630668799	12841949	T	0.25	0.5714	A
10	BICF2G630668784	12848333	G	0.25	0.5714	A
10	BICF2G630668780	12858119	G	0.25	0.5714	A
10	BICF2G630668758	12877349	G	0.25	0.5714	A
10	BICF2G630668753	12883536	A	0.75	0.4286	G
10	BICF2G630668751	12894618	A	0.25	0.5714	G
10	BICF2G630668746	12904183	G	0	0	A
10	BICF2S23030011	12927170	C	0	0	A
10	BICF2S23346844	12937928	A	0.25	0.5714	G
10	BICF2G630668682	12952585	G	0.25	0.5714	A
10	BICF2P1006461	12963860	A	0.25	0.5714	C
10	BICF2G630668680	12976221	G	0.25	0.5714	A
10	BICF2G630668656	12993795	A	0	0	G
10	BICF2G630668653	12998401	C	0.75	0.4286	A
10	BICF2G630668640	13011828	A	0	0	G
10	BICF2G630668626	13020500	G	0.25	0.5714	A
10	BICF2G630668607	13039127	G	0.25	0.5714	A
10	BICF2G630668599	13042160	A	0.25	0.5714	G
10	BICF2G630668564	13063513	0	0	0	G
10	BICF2S23159482	13065026	A	0	0	G
10	BICF2G630668562	13084794	0	0	0	C
10	BICF2G630668557	13090517	G	0.75	0.4286	A
10	BICF2G630668542	13098500	A	0.75	0.3333	G
10	BICF2G630668532	13120588	G	0.25	0.5714	A
10	BICF2P950102	13144787	0	0	0	G
10	BICF2P1361615	13165751	0	0	0	G
10	BICF2G630668529	13174412	G	0.75	0.4286	A
10	BICF2G630668517	13182072	0	0	0	G

10	BICF2P331954	13205131	0	0	0	A
10	BICF2S23229133	13223901	A	0.25	0.5833	G
10	BICF2S23411	13226026	G	0.25	0.5714	A
10	TIGRP2P137537_rs9091258	13242211	0	0	0	A
10	BICF2P34370	13259282	G	0.25	0.5714	A
10	BICF2P1019601	13268339	A	0.25	0.5714	G
10	BICF2G630668495	13279482	A	0.25	0.5714	T
10	BICF2G630668488	13285695	A	0.25	0.5714	G
10	BICF2G630668472	13299435	G	0.75	0.4286	A
10	BICF2G630668466	13308894	A	0.25	0.5714	G
10	BICF2G630668441	13329297	G	0.25	0.5714	A
10	BICF2G630668432	13340991	C	0.25	0.5714	A
10	BICF2G630668424	13362953	A	0.75	0.4167	G
10	BICF2P423122	13365218	0	0	0	A
10	BICF2G630668422	13393452	A	0.25	0.5714	G
10	BICF2G630668412	13407526	A	0	0	G
10	BICF2P674965	13414808	A	0.75	0.4286	G
10	BICF2S23326440	13422512	A	0.75	0.4286	G
10	BICF2P1237568	13435580	A	0.75	0.4286	C
10	BICF2P1439928	13439502	A	0.75	0.4286	G
10	BICF2G630668401	13447215	A	1	1	C
10	BICF2G630668397	13460870	G	0	0	A
10	BICF2G630668391	13467963	A	0.75	0.4286	G
10	BICF2G630668366	13478082	G	0.25	0.5714	A
10	BICF2S23441254	13491773	G	0	0	A
10	BICF2G630668349	13502579	G	0.25	0.5714	A
10	BICF2G630668347	13520959	C	0.25	0.5714	G
10	BICF2G630668346	13525238	C	0	0	A
10	BICF2S23144055	13542709	A	0	0	G
10	BICF2G630668338	13547701	G	1	1	A
10	BICF2G630668330	13553752	G	1	1	A
10	BICF2G630668329	13559026	G	0	0	A
10	BICF2G630668296	13579391	A	1	1	G
10	BICF2G630668289	13582865	C	1	1	A
10	BICF2G630668270	13620103	A	1	1	G
10	BICF2P237994	13637244	G	0.25	0.5714	A
10	BICF2S22910944	13639100	A	0.75	0.4286	C
10	BICF2G630668267	13660508	0	0	0	A
10	BICF2P1122201	13666858	A	1	1	G
10	BICF2P678176	13674441	A	0	0	G
10	BICF2G630668240	13704607	A	0	0	G
10	BICF2G630668224	13723824	G	0	0	A
10	BICF2G630668213	13738599	G	0	0	A
10	BICF2G630668210	13743536	A	0	0	G
10	BICF2P1024825	13796955	0	0	0	A
10	BICF2S23650439	13818508	C	0	0	A
10	BICF2G630668204	13824615	0	0	0	G

10	BICF2P276510	13836709	C	0	0	A
10	TIGRP2P137554_rs8940107	13866660	0	0	0	G
10	BICF2P225410	13881530	A	0.75	0.4286	G
10	BICF2G630668174	13894577	A	0.25	0.5714	G
10	BICF2S23539555	13917465	T	0	0	A
10	BICF2P1345080	13927879	A	0.75	0.4286	C
10	BICF2P42472	13942585	A	0.25	0.5714	G
10	BICF2P908302	13949772	A	0.75	0.4286	G
10	BICF2P626862	13968079	A	0.75	0.4286	G
10	BICF2P371545	13976454	G	0.75	0.4286	A
10	BICF2S23243159	13987009	A	0.25	0.5714	G
10	BICF2G630668130	13998098	G	0.25	0.5714	A
10	BICF2P1032483	14009725	A	0.75	0.4286	G
10	BICF2G630668104	14020175	G	1	1	A
10	BICF2G630668095	14039967	A	0.25	0.5714	G
10	BICF2G630668089	14046241	A	0.25	0.5714	G
10	BICF2P436672	14053580	A	1	1	T
10	BICF2G630668076	14070997	G	1	1	A
10	BICF2G630668052	14087517	A	0.25	0.5714	G
10	BICF2P1197066	14098348	A	0	0	C
10	BICF2G630668040	14099859	G	0	0	A
10	BICF2P1096888	14118731	G	0.25	0.5714	A
10	BICF2G630667991	14135199	A	0	0	G
10	BICF2P862409	14155756	C	0	0	A
10	BICF2P35801	14158514	0	0	0	A
10	TIGRP2P137566_rs8762764	14170748	0	0	0	G
10	BICF2P925611	14206472	G	0.25	0.5714	A
10	BICF2P905176	14222517	G	0	0.5714	A
10	BICF2P733198	14231693	A	0.75	0.4286	G
10	BICF2P433765	14244786	A	0.75	0.4286	G
10	BICF2S22945419	14252981	A	0.25	0.5714	G
10	BICF2S23040335	14262537	G	0	0	A
10	BICF2G630667968	14286174	A	1	1	G
10	BICF2G630667949	14295681	A	1	1	G
10	BICF2G630667934	14316840	A	0.75	0.4286	G
10	BICF2G630667923	14322936	G	0.75	0.4286	A
10	BICF2S23134379	14335427	A	0	0	G
10	BICF2S23045534	14345241	G	1	0.4167	A
10	BICF2S22918652	14355368	G	1	1	A
10	BICF2S2317283	14394798	A	0	0	G
10	BICF2S23158988	14419170	C	0	0	G
10	BICF2P330466	14426510	0	0	0	A
10	TIGRP2P137572_rs8566283	14442848	0	0	0	C
10	BICF2P550031	14463429	0	0	0	A
10	BICF2S23154039	14500275	G	0	0	A
10	BICF2P570400	14530023	0	0	0	A
10	BICF2P1003918	14549034	0	0	0	A

10	BICF2S2317764	14561346	A	0.25	0.5714	G
10	BICF2P324173	14577265	C	0.75	0.4167	A
10	BICF2S23620390	14587412	G	1	1	A
10	BICF2G630667894	14592309	A	0	0	G
10	BICF2G630667877	14598429	G	0.25	0.2143	A
10	BICF2G630667842	14609443	A	0	0	G
10	BICF2G630667834	14619808	A	0.75	0.7857	G
10	BICF2G630667810	14635274	A	0.75	0.7857	G
10	BICF2G630667800	14647541	G	0.25	0.2143	A
10	BICF2P1029753	14652828	C	0	0	A
10	BICF2P1354152	14663415	G	0.25	0.2143	A
10	BICF2G630667793	14687389	0	0	0	G
10	BICF2G630667787	14704641	A	0	0	G
10	BICF2G630667780	14710804	A	0.25	0.2143	G
10	BICF2G630667763	14723990	A	0	0	G
10	BICF2P877782	14730825	G	0	0	C
10	BICF2P877783	14730844	C	0.25	0.5714	A
10	BICF2G630667760	14733437	A	0	0	G
10	BICF2P908662	14744518	A	0	0	G
10	BICF2G630667758	14757231	G	0.75	0.4167	A
10	BICF2G630667757	14771007	A	0.25	0.2143	G
10	BICF2G630667742	14783513	A	0.25	0.2143	G
10	BICF2G630667740	14786216	A	0.25	0.2143	G
10	BICF2G630667726	14793883	A	0.25	0.2143	G
10	BICF2G630667700	14801680	G	0.25	0.2143	A
10	BICF2P942975	14814155	G	0.25	0.2143	A
10	BICF2P540345	14825638	G	0	0	A
10	BICF2P842328	14836058	A	0.25	0.2143	G
10	BICF2P747070	14855306	C	0.25	0.2143	A
10	BICF2G630667682	14866792	G	0	0	A
10	BICF2P652856	14879058	A	0.25	0.2143	G
10	BICF2P670634	14887963	G	0	0	A
10	BICF2P1270684	14898479	G	0	0	A
10	BICF2P805802	14909147	A	0	0	G
10	BICF2P676270	14916418	C	0.25	0.2143	A
10	BICF2P1256108	14928083	0	0	0	A
10	BICF2P485070	14946366	G	0.25	0.2143	A
10	BICF2P572633	14951709	A	0	0	G
10	BICF2P112319	14966622	0	0	0	A
10	BICF2P111969	14974756	G	0	0	A
10	BICF2P943137	14990547	0	0	0	C
10	BICF2G630667678	14999307	A	0	0	G
10	BICF2P1199199	15011326	0	0	0	G
10	BICF2P286584	15024451	0	0	0	C
10	BICF2P194220	15039777	A	0	0	G
10	TIGRP2P137578_rs8693500	15050376	0	0	0	A
10	BICF2P1011013	15061712	A	0	0	G

10	BICF2P138871	15065505	A	0	0	G
10	BICF2P704528	15082379	C	0	0	G
10	BICF2P806163	15105763	G	0	0	A
10	BICF2P838659	15119421	0	0	0	A
10	BICF2P1188502	15139583	A	0	0	C
10	BICF2P1029924	15148009	A	0.25	0.2143	G
10	BICF2G630667674	15159529	0	0	0	C
10	BICF2P597618	15169258	G	0	0	A
10	BICF2G630667669	15181707	A	0	0	G
10	BICF2G630667632	15193131	A	0	0	G
10	BICF2G630667624	15196945	A	0	0	G
10	BICF2P431692	15214424	C	0	0	G
10	BICF2G630667599	15224543	A	0	0	G
10	BICF2G630667590	15233406	A	1	1	G
10	BICF2G630667586	15237930	G	0	0	A
10	BICF2G630667573	15245622	A	1	1	G
10	BICF2G630667553	15257061	A	1	1	G
10	BICF2G630667541	15267767	C	1	1	A
10	BICF2G630667526	15274350	A	1	1	G
10	BICF2G630667484	15292459	A	0.75	0.7857	G
10	BICF2P774079	15296179	A	0.75	0.7857	G
10	BICF2P343272	15315750	C	0	0	A
10	BICF2P698767	15318065	G	0	0	A
10	BICF2P679740	15332669	A	0	0	G
10	BICF2S23022684	15349046	G	1	1	A
10	BICF2S23334975	15354718	G	1	1	A
10	BICF2S23246912	15365121	G	0.25	0.2143	A
10	BICF2P1128052	15384112	A	0	0	G
10	BICF2P1128045	15385859	A	0	0	C
10	BICF2G630667469	15395831	G	0.25	0.2143	A
10	BICF2G630667450	15404277	A	0.25	0.2143	C
10	BICF2G630667436	15415392	C	0.25	0.2143	A
10	BICF2G630667430	15436471	A	0.25	0.2143	G
10	BICF2G630667420	15445695	A	0.25	0.2143	G
10	BICF2P1096891	15458495	A	0.25	0.2143	G
10	BICF2P444343	15469812	A	0.25	0.2143	G
10	BICF2S22938867	15479419	A	1	1	G
10	BICF2P800963	15500806	A	0.75	0.7857	G
10	BICF2G630667403	15503081	A	1	1	G
10	BICF2S23418846	15514095	A	0	0	G
10	BICF2G630667354	15534933	A	0.25	0.2143	G
10	BICF2G630667349	15541329	C	0.75	0.7857	A
10	BICF2G630667333	15551793	C	0.25	0.2143	A
10	BICF2G630667328	15560171	A	0	0.2143	G
10	BICF2G630667320	15577089	G	0	0	A
10	BICF2P948334	15597114	0	0	0	G
10	BICF2G630667318	15612355	G	0.25	0.2143	A

10	BICF2G630667309	15619329	A	0	0	T
10	BICF2P229848	15632262	G	0	0	A
10	BICF2G630667295	15640791	A	0	0	G
10	BICF2G630667282	15654324	G	0	0	A
10	BICF2S23027779	15673428	0	0	0	G
10	G582f57S210	15703080	0	0	0	C
10	BICF2S23540228	15728316	A	0	0	T
10	BICF2S23540229	15728385	A	0	0	G
10	BICF2G630667271	15732144	A	0	0	G
10	BICF2G630667270	15745043	G	0	0	A
10	BICF2G630667267	15764876	A	0	0.1	C
10	BICF2G630667258	15768624	G	0.25	0.2143	A
10	BICF2S22928326	15783294	G	0.75	0.7857	A
10	BICF2P1153772	15793705	0	0	0	A
10	BICF2S23332874	15802720	A	0	0	G
10	BICF2S23449675	15845592	C	0.75	0.7857	A
10	BICF2S23121948	15850511	A	0.75	0.7857	G
10	BICF2S23137905	15871322	A	0.75	0.7857	G
10	BICF2S23327520	15885341	G	0.75	0.7857	A
10	BICF2S23914543	15898074	A	0.75	0.7857	G
10	BICF2S2352970	15937763	A	0.25	0.2143	G
10	BICF2P193782	15942188	0	0	0	G
10	BICF2S23758184	15959375	0	0	0	G
10	BICF2P298929	15976246	0	0	0	A
10	BICF2S22926474	15999486	A	0.75	0.9	C
10	BICF2S22957237	16018920	0	0	0	G
10	BICF2G630667248	16023583	G	1	1	A
10	BICF2G630667230	16037535	G	0	0	A
10	BICF2G630667217	16043582	0	0	0	G
10	BICF2G630667211	16064170	C	0.25	0.2143	A
10	BICF2G630667209	16066544	A	0	0	G
10	BICF2S23015473	16106234	A	0.25	0.2143	G
10	BICF2P980513	16112361	0	0	0	C
10	TIGRP2P137591_rs9085346	16127011	0	0	0	C
10	BICF2S23761887	16144389	G	0.25	0.2143	A
10	BICF2S2333447	16148959	G	0.5	0.2143	A
10	BICF2S2366112	16160953	0	0	0	A
10	BICF2P1432014	16170068	0	0	0	A
10	BICF2S23121173	16223936	A	0	0	G
10	BICF2G630667201	16246762	A	0	0	G
10	BICF2G630667190	16259823	G	0	0	A
10	BICF2P624218	16285421	0	0	0	G
10	BICF2G630667187	16300467	A	0.25	0.2143	G
10	BICF2G630667186	16316212	A	0	0	T
10	BICF2G630667184	16319313	G	0	0	A
10	BICF2G630667175	16331014	T	0.25	0.2143	A
10	BICF2S23717644	16355566	A	0.75	0.7857	T

10	BICF2S23653696	16380544	G	0.75	0.7857	A
10	BICF2S22954730	16398359	G	1	0.7857	A
10	BICF2S23524544	16432202	C	0	0	A
10	BICF2S24321914	16441352	0	0	0	G
10	BICF2G630667158	16451919	A	0.5	0.2143	G
10	BICF2G630667148	16462260	A	1	1	G
10	BICF2G630667147	16462406	C	0	0	A
10	BICF2G630667138	16471769	A	1	1	C
10	BICF2G630667127	16483223	A	0	0	G
10	BICF2S2318921	16497053	T	0	0	A
10	BICF2G630667097	16504746	G	0	0	A
10	BICF2G630667084	16519195	G	0	0	A
10	BICF2G630667069	16526194	A	0	0	G
10	BICF2P700052	16540001	G	0	0	A
10	BICF2G630667045	16556165	A	0	0	G
10	BICF2G630667044	16565405	G	0	0	A
10	BICF2P943690	16599217	0	0	0	A
10	BICF2S24116722	16612931	A	0	0	C
10	BICF2S22958804	16623424	A	0	0	G
10	BICF2S23228113	16674345	A	0	0	G
10	BICF2G630667039	16678898	C	0	0	A
10	BICF2G630667020	16693407	A	0.25	0.2143	C
10	BICF2P430809	16712932	A	0.25	0.2143	G
10	BICF2G630666990	16722796	G	0.25	0.2143	A
10	BICF2G630666978	16740741	A	0.25	0.5714	G
10	BICF2S23754748	16749953	A	0.25	0.2143	G
10	BICF2G630666959	16759520	G	0.25	0.2143	A
10	BICF2G630666936	16767377	A	0.25	0.2143	G
10	BICF2G630666924	16786588	A	0.25	0.2143	C
10	BICF2G630666923	16811309	A	0	0	G
10	BICF2G630666921	16826764	G	0	0	A
10	BICF2G630666905	16885083	A	0	0	G
10	BICF2G630666879	16905510	A	0	0	G
10	BICF2G630666850	16935660	G	0.5	0.7857	A
10	BICF2G630666821	16946158	G	0.5	0.7857	A
10	BICF2G630666809	16951620	G	0.5	0.7857	A
10	BICF2G630666784	16970379	A	0.25	0.2143	G
10	BICF2P1171453	16974417	G	0	0	A
10	BICF2P1002669	16993092	A	0.25	0.5714	G
10	BICF2P480785	16998762	0	0	0	A
10	BICF2P1312747	17008951	A	0.25	0.2143	G
10	BICF2P319736	17013332	A	0.25	0.2143	G
10	BICF2S23026580	17031330	A	0	0	G
10	BICF2S23427952	17041649	A	0	0	C
10	BICF2P1203806	17054023	G	0	0	A
10	BICF2P682501	17064643	A	0	0	C
10	BICF2S23550470	17072470	A	0	0	G

10	BICF2P1130065	17083696	A	0	0	G
10	BICF2G630666775	17083986	A	0	0	G
10	BICF2P814692	17092363	G	0.25	0.1	A
10	BICF2G630666763	17092525	A	0	0	G
10	BICF2P370304	17104743	G	0.5	0.7857	A
10	BICF2P830613	17116744	0	0	0	G
10	BICF2G630666734	17131554	G	0	0	A
10	BICF2G630666708	17139501	A	0	0	G
10	BICF2G630666694	17146552	C	0	0	A
10	BICF2P636946	17161543	A	0	0	G
10	BICF2S23232930	17165696	A	0	0	G
10	BICF2S23229186	17169611	A	0	0	G
10	BICF2G630666617	17210241	A	0	0	G
10	BICF2G630666613	17215776	A	0	0	G
10	BICF2S23550198	17231288	A	0.5	0.2143	G
10	BICF2S2315987	17242209	A	0	0	G
10	BICF2S22962514	17256141	A	0.5	0.7857	G
10	BICF2G630666603	17266999	A	0	0	G
10	BICF2G630666597	17270177	C	0.25	0.2143	A
10	BICF2G630666589	17273336	A	0.5	0.2143	G
10	BICF2G630666581	17303422	A	0.25	0.2143	G
10	BICF2G630666576	17323502	A	0.25	0.5714	C
10	BICF2G630666574	17331902	G	0	0	A
10	BICF2G630666565	17352396	C	0	0	A
10	BICF2G630666561	17355738	A	0	0	G
10	BICF2G630666560	17379124	A	0.25	0.2143	T
10	BICF2G630666550	17409201	A	0	0	G
10	BICF2P885725	17414241	T	0.25	0.5714	A
10	BICF2G630666532	17422812	G	0.25	0.5833	A
10	BICF2P663407	17435056	A	0.5	0.2143	G
10	BICF2G630666504	17446261	G	0.75	0.7857	A
10	BICF2G630666490	17460628	C	0	0	G
10	BICF2G630666477	17476560	G	0.5	0.2143	A
10	BICF2G630666471	17480744	G	0.5	0.2143	A
10	BICF2S23625861	17497583	C	0.75	0.7857	A
10	BICF2S23041688	17503071	A	0.5	0.2143	G
10	BICF2S23728697	17520668	A	0.75	0.7857	G
10	BICF2S23157783	17533939	G	0.25	0.5714	A
10	BICF2S23737487	17539889	G	0.25	0.5714	A
10	BICF2G630666460	17559638	A	0	0.2143	G
10	BICF2P1367712	17580409	0	0	0	G
10	BICF2P437553	17594295	0	0	0	A
10	BICF2P663111	17604837	A	0.25	0.5714	C
10	BICF2P179049	17607430	G	0.25	0.5714	A
10	BICF2G630666420	17627841	T	0.5	0.2143	A
10	BICF2G630666416	17630102	A	0.5	0.2143	G
10	BICF2G630666383	17650587	G	0.5	0.2143	A

10	BICF2G630666382	17650691	A	0.5	0.2143	G
10	BICF2G630666374	17658530	G	0.5	0.2143	A
10	BICF2G630666362	17672724	G	0.5	0.2143	A
10	BICF2G630666354	17677149	G	0.5	0.2143	A
10	BICF2G630666350	17692480	G	0.5	0.2143	A
10	BICF2S23740201	17702587	A	0.25	0.5714	C
10	BICF2P660001	17723309	0	0	0	T
10	BICF2G630666346	17739364	A	0.25	0.2143	G
10	BICF2P199657	17768086	0	0	0	G
10	TIGRP2P137613_rs9009665	17780502	0	0	0	G
10	BICF2S23629669	17791566	G	0	0	A
10	BICF2S23024485	17820998	G	0.5	0.2143	C
10	BICF2G630666341	17852072	G	0	0	A
10	BICF2P1148357	17861375	A	0	0	G
10	BICF2S23444542	17880581	G	0	0	A
10	BICF2P991910	17882879	A	0	0	G
10	BICF2G630666331	17894824	G	0.25	0.5714	A
10	BICF2P674656	17911769	A	0	0	G
10	BICF2P13254	17925536	0	0	0	A
10	BICF2P1309651	17930054	G	0	0	A
10	BICF2G630666323	17950308	0	0	0	A
10	BICF2P1136930	17955545	G	0	0	A
10	BICF2P1023950	17970776	A	0	0	G
10	BICF2P808639	17975003	T	0	0	A
10	BICF2P460601	17987522	A	0.5	0.2143	G
10	BICF2P137153	18007211	G	0	0	A
10	BICF2P502550	18015762	A	0	0	G
10	BICF2G630666316	18026427	A	0	0	G
10	BICF2G630666314	18035201	A	0	0	G
10	BICF2G630666307	18047809	A	0.5	0.2143	G
10	BICF2G630666291	18057413	A	0.5	0.2143	G
10	BICF2G630666272	18067610	G	0.5	0.2143	A
10	BICF2G630666261	18078970	G	0	0	A
10	BICF2G630666249	18093228	C	0	0	A
10	BICF2P11721	18102432	A	0.5	0.2143	C
10	TIGRP2P137635_rs8614929	18116461	A	0.5	0.2143	G
10	BICF2P1292368	18124216	A	0	0	G
10	BICF2G630666217	18141270	A	0.5	0.2143	G
10	BICF2G630666199	18147298	C	0	0	A
10	BICF2P917004	18162839	G	0.5	0.2143	A
10	BICF2G630666183	18178040	A	0	0	G
10	BICF2S2335347	18192975	A	0.5	0.7857	C
10	BICF2G630666158	18195616	A	0	0	G
10	BICF2G630666147	18215462	A	0.5	0.2143	G
10	BICF2G630666146	18216075	G	0.5	0.7857	A
10	BICF2P786503	18234270	0	0	0	A
10	BICF2G630666129	18239835	A	0.5	0.7857	G

10	BICF2G630666109	18252782	A	0.5	0.2143	G
10	BICF2G630666082	18264781	G	0.5	0.2143	A
10	BICF2S23642204	18279051	A	0	0	C
10	BICF2P731432	18291026	A	0	0	C
10	BICF2G630666065	18291749	G	0.5	0.2143	A
10	BICF2G630666051	18293942	A	0.5	0.2143	G
10	BICF2P826040	18306317	G	0.5	0.2143	A
10	BICF2G630666017	18320326	G	0.5	0.7857	A
10	BICF2G630666007	18336207	A	0.5	0.7857	G
10	BICF2G630666005	18344084	A	0	0	C
10	BICF2S2309084	18361522	C	0	0	A
10	BICF2G630665999	18375253	A	0	0	G
10	BICF2S2328886	18380480	G	0.5	0.2143	A
10	BICF2G630665985	18389766	A	0.5	0.7857	C
10	BICF2G630665970	18401415	A	0.5	0.7857	G
10	BICF2G630665959	18421071	G	0	0	A
10	BICF2G630665950	18428031	G	0	0	A
10	BICF2G630665944	18432109	A	0.5	0.7857	G
10	BICF2S23340292	18439038	A	0.5	0.8333	G
10	BICF2G630665925	18448668	A	0	0	C
10	BICF2G630665899	18457689	A	0	0	G
10	BICF2G630665865	18480319	A	0.5	0.7857	C
10	BICF2S23222684	18484499	A	0	0	G
10	BICF2G630665860	18498511	T	0.5	0.7857	A
10	BICF2G630665853	18532922	G	0.5	0.7857	A
10	BICF2G630665845	18546343	A	0.5	0.7857	G
10	BICF2G630665832	18553547	A	0.5	0.7857	G
10	BICF2P725595	18567472	0	0	0	C
10	BICF2P1022253	18581523	0	0	0	A
10	BICF2P231798	18586898	0	0	0	C
10	BICF2G630665824	18603448	A	0.5	0.2143	G
10	BICF2G630665816	18613158	T	0.5	0.2143	A
10	BICF2G630665810	18621559	C	0.5	0.2143	A
10	BICF2G630665797	18634408	G	0.5	0.1667	A
10	BICF2G630665795	18645753	G	0.5	0.7857	A
10	G583f55S106	18670967	C	0.5	0.7857	A
10	BICF2P346324	18727191	0	0	0	C
10	BICF2P1034991	18735057	G	0	0	A
10	BICF2S2342163	18755730	A	0	0	G
10	TIGRP2P137654_rs8704941	18767228	0	0	0	A
10	TIGRP2P137655_rs8855366	18780812	0	0	0	G
10	BICF2G630665793	18825019	A	0	0	G
10	BICF2P554488	18832269	0	0	0	A
10	BICF2P739798	18848828	0	0	0	A
10	BICF2G630665791	18865245	A	0	0	T
10	BICF2P384885	18905016	0	0	0	C
10	BICF2P145394	18912256	0	0	0	A

10	BICF2S23633219	18956435	A	0.5	0.7857	G
10	BICF2G630665788	18970933	A	0	0	G
10	BICF2P129067	18976228	0	0	0	A
10	TIGRP2P137865_rs8655603	19066039	0	0	0	A
10	TIGRP2P138053_rs8699336	19108435	0	0	0	A
10	BICF2P1142106	19251815	0	0	0	A
10	BICF2P1081130	19296074	0	0	0	G
10	BICF2G630665516	19314747	A	0	0	G
10	TIGRP2P138510_rs9185689	19329009	0	0	0	C
10	TIGRP2P138525_rs8752959	19340473	0	0	0	G
10	TIGRP2P138549_rs8758853	19350605	0	0	0	C
10	BICF2P981880	19360486	0	0	0	G
10	TIGRP2P138558_rs8415316	19374850	0	0	0	A
10	BICF2G630665434	19401408	0	0	0	A
10	BICF2S23642383	19412316	A	1	1	G
10	BICF2P367563	19453514	0	0	0	A
10	BICF2P165847	19547095	A	0	0	G
10	BICF2G630476105	19554997	A	0	0	G
10	BICF2S23661386	19568635	A	0	0	G
10	BICF2G630476114	19603863	A	0	0	C
10	BICF2P27466	19608952	0	0	0	G
10	BICF2P899132	19627237	0	0	0	G
10	BICF2G630476115	19632084	A	0	0	G
10	BICF2G630476118	19690960	A	0	0	G
10	BICF2P550469	19732537	0	0	0	G
10	BICF2G630476119	19749018	A	0	0	G
10	BICF2P271043	19773318	A	0	0	G
10	BICF2S2372888	19781210	G	1	1	A
10	BICF2P365143	19791824	A	0	0	T
10	BICF2S23043071	19815401	A	0.5	0	G
10	TIGRP2P138791_rs8654162	19827631	0	0	0	C
10	TIGRP2P138792_rs9079833	19841914	0	0	0	C
10	G585f41S202	19860072	0	0	0	G
10	BICF2G630476123	19885370	A	0	0	G
10	G586f25S4	19902950	0	0	0	C
10	G586f28S199	19906689	A	1	1	C
10	BICF2P1204119	19931662	A	0	0	G
10	BICF2S23215334	19950451	A	1	1	C
10	TIGRP2P138807_rs8629138	19957464	0	0	0	C
10	TIGRP2P138808_rs9013281	19970295	0	0	0	G
10	BICF2S23639100	19981268	A	0	0	G
10	BICF2P1090644	19995790	G	0	0	A
10	BICF2P1212855	20007598	0	0	0	G
10	BICF2G630476131	20014765	A	0	0	G
10	BICF2G630476132	20039109	A	1	1	C
10	BICF2P804757	20045961	0	0	0	A
10	BICF2G630476134	20075150	A	1	1	G

10	TIGRP2P138810_rs9111077	20097325	0	0	0	C
10	BICF2G630476136	20105887	A	0	0	G
10	BICF2S23645267	20123248	0	0	0	G
10	TIGRP2P138812_rs8921108	20128451	0	0	0	G
10	BICF2P499870	20147895	0	0	0	A
10	BICF2P1405215	20154780	0	0	0	A
10	BICF2P647101	20163274	A	1	1	C
10	G587f34S290	20184188	A	0	0	G
10	BICF2G630476139	20203658	A	0	0	C
10	BICF2S2367328	20231672	C	0.5	0.7857	A
10	BICF2P376281	20271278	A	1	1	G
10	BICF2G630476142	20286558	A	0	0	G
10	BICF2G630476156	20303993	G	1	1	A
10	BICF2G630476167	20320072	G	1	1	A
10	BICF2G630476170	20323047	G	1	1	A
10	BICF2P701741	20352017	A	1	1	G
10	BICF2G630476178	20355685	G	0	0	A
10	BICF2P400229	20357446	A	0	0	G
10	BICF2P760594	20367034	0	0	0	C
10	BICF2S22910335	20376337	A	1	1	G
10	BICF2G630476217	20382900	G	1	1	A
10	BICF2G630476218	20383008	A	0	0	G
10	BICF2P1157786	20405433	G	0	0	A
10	BICF2G630476268	20421439	A	0	0	G
10	BICF2G630476296	20426969	A	0	0	G
10	BICF2G630476338	20441780	G	0	0	A
10	BICF2S23136386	20455293	0	0	0	G
10	BICF2G630476365	20467377	G	1	1	A
10	BICF2G630476380	20474964	A	1	1	G
10	BICF2P887404	20488919	0	0	0	C
10	BICF2G630476391	20503665	0	0	0	C
10	BICF2S23431554	20521179	G	0	0	A
10	BICF2S2323343	20535790	A	1	1	G
10	BICF2S2293422	20539936	G	1	1	A
10	BICF2S22917215	20556374	C	1	1	A
10	BICF2S22917368	20571502	G	1	1	A
10	BICF2S2299144	20575604	G	1	1	A
10	BICF2G630476398	20594829	0	0	0	A
10	BICF2S2312648	20597292	A	0	0	G
10	BICF2S23753253	20615770	G	1	1	A
10	BICF2S22964470	20625101	A	1	1	G
10	BICF2S23633590	20632378	G	0	0	A
10	BICF2G630476414	20647231	A	1	1	G
10	BICF2G630476461	20663647	G	1	1	A
10	BICF2G630476474	20674545	A	1	1	G
10	BICF2P206086	20682248	G	0	0	A
10	BICF2G630476537	20695147	C	0	0	A

10	BICF2P408249	20711058	G	0	0	A
10	BICF2G630476615	20717890	0	0	0	G
10	BICF2G630476696	20732911	C	0	0	A
10	BICF2G630476734	20737727	A	0	0	G
10	BICF2S23762716	20746370	A	0	0	G
10	BICF2S23440926	20760367	A	0	0	G
10	BICF2G630476757	20769085	A	0	0	G
10	BICF2G630476786	20785314	A	1	1	G
10	BICF2S23539950	20809193	A	1	1	G
10	BICF2P1189167	20815061	0	0	0	C
10	BICF2P1186475	20835533	0	0	0	G
10	BICF2S23646761	20856615	G	0	0	A
10	BICF2P145315	20862472	0	0	0	G
10	BICF2S23552428	20879998	A	0	0	G
10	BICF2P215163	20900857	0	0	0	C
10	BICF2S23535897	20907566	A	0	0	G
10	BICF2S23711700	20924642	0	0	0	G
10	BICF2S23248178	20938553	A	0	0	G
10	BICF2S23653707	20953528	G	0	0	A
10	BICF2G630476819	20969912	G	0	0	A
10	BICF2G630476847	20977836	A	0	0	G
10	BICF2S23763624	21003653	G	0.25	0.2143	A
10	BICF2G630476863	21015546	A	0	0.1	C
10	BICF2G630476883	21032209	A	0	0	G
10	BICF2G630476894	21038174	A	0	0	G
10	BICF2S23057546	21048848	A	0.5	0.4167	G
10	BICF2G630476924	21054725	C	0	0	A
10	BICF2G630476932	21059241	A	0.5	0.4286	C
10	BICF2S22931870	21063615	G	0	0	A
10	BICF2S22914679	21074310	A	0	0	G
10	BICF2P212964	21088011	G	0.5	0.5714	A
10	BICF2S2398845	21101134	A	0	0	G
10	BICF2P397711	21120656	C	0	0	G
10	BICF2G630477021	21135938	A	0	0	C
10	TIGRP2P138832_rs8665665	21139835	0	0	0	A
10	BICF2P949774	21149675	0	0	0	C
10	BICF2S22963358	21168069	A	0.5	0.5714	G
10	BICF2G630477022	21182592	A	0.25	0.2143	G
10	BICF2S23548656	21186445	A	0.25	0.2143	G
10	TIGRP2P138833_rs9196182	21199037	0	0	0	C
10	BICF2G630477032	21208404	A	0.75	0.7857	G
10	BICF2G630477053	21224211	G	0.75	0.7857	A
10	BICF2G630477070	21230860	G	0.5	0.5714	A
10	BICF2G630477074	21250016	G	0	0	A
10	BICF2G630477080	21258702	G	0	0	C
10	BICF2G630477108	21274843	G	0.75	0.7857	A
10	BICF2P379354	21277028	G	0.75	0.7857	A

10	BICF2G630477117	21288528	A	0.75	0.7857	G
10	BICF2G630477121	21301586	A	0.5	0.4286	C
10	TIGRP2P138835_rs8967021	21314541	0	0	0	G
10	BICF2G630477137	21327079	A	0	0.1667	C
10	BICF2G630477156	21333717	A	0.75	0.7857	G
10	BICF2G630477187	21345466	C	0.25	0.2143	A
10	BICF2G630477216	21356215	G	0.75	0.7857	A
10	BICF2P770135	21367723	A	0.25	0.2143	G
10	BICF2G630477234	21388067	A	1	1	G
10	BICF2G630477242	21392104	A	0	0	C
10	BICF2G630477266	21399975	A	0	0	G
10	BICF2G630477274	21401730	C	0.25	0.2143	A
10	BICF2G630477307	21414509	0	0	0	C
10	BICF2S23747195	21437588	A	0	0	G
10	BICF2P72900	21466901	A	0.25	0.2143	G
10	BICF2S22946687	21472308	A	0	0	G
10	BICF2S2324854	21490775	A	0	0	G
10	BICF2P803082	21498876	A	0.25	0.2143	G
10	TIGRP2P138837_rs8681520	21514450	0	0	0	C
10	TIGRP2P138839_rs9088156	21534921	0	0	0	C
10	BICF2G630477316	21541594	G	0.75	0.7857	A
10	BICF2G630477319	21582032	A	0	0	G
10	BICF2S23251757	21595461	A	0	0	C
10	BICF2G630477321	21606691	A	0.25	0.2143	G
10	BICF2G630477322	21609208	A	0	0	G
10	BICF2P858340	21622170	0	0	0	G
10	BICF2P1290657	21634100	0	0	0	A
10	BICF2P1434136	21645722	A	0	0	G
10	BICF2S23347379	21662278	A	0	0	G
10	BICF2S23229875	21668563	A	0	0	G
10	BICF2P1204435	21680347	0	0	0	A
10	BICF2S23620835	21689634	G	0.75	0.7857	A
10	BICF2S23551774	21707591	A	0	0	G
10	BICF2S22949031	21712139	G	0	0	A
10	BICF2S2366144	21723906	A	0	0	G
10	G588f45S168	21755499	0	0	0	C
10	BICF2S2417932	21760238	A	1	1	G
10	TIGRP2P138843_rs9089525	21792224	0	0	0	C
10	BICF2P1138892	21799255	A	0	0	G
10	BICF2G630477325	21835914	A	0.25	0.2143	G
10	BICF2G630477326	21852307	A	0	0	G
10	BICF2P1035132	21865018	0	0	0	G
10	BICF2S23252280	21882118	G	0.5	0.4286	A
10	BICF2P1113285	21891186	0	0	0	G
10	BICF2P1083878	21899705	G	0	0	A
10	BICF2P1093156	21908933	0	0	0	G
10	BICF2P1330629	21924129	0	0	0	G

10	BICF2G630477338	21935313	A	0.25	0.2143	G
10	BICF2G630477358	21946554	A	0.25	0.2143	G
10	BICF2G630477394	21956429	G	0.25	0.2143	C
10	BICF2G630477421	21964082	G	0.5	0.4286	A
10	BICF2P826827	21975190	0	0	0	G
10	BICF2G630477444	21975242	A	0.25	0.2143	G
10	BICF2P948947	21980085	A	0.25	0.2143	G
10	BICF2P1207446	21992574	G	0.25	0.2143	A
10	BICF2G630477457	22008650	C	0.5	0.4286	A
10	BICF2G630477461	22012123	G	0.5	0.4286	A
10	BICF2G630477504	22027741	G	0.25	0.2143	A
10	BICF2G630477520	22035692	G	0	0	A
10	BICF2G630477564	22055807	G	0.25	0.1667	A
10	BICF2G630477571	22057419	A	0.25	0.2143	G
10	BICF2S23627211	22082398	G	0	0	A
10	BICF2G630477576	22094309	C	0	0	A
10	BICF2S23731451	22108980	A	0	0	G
10	BICF2P488106	22122019	C	0.75	0.7857	A
10	BICF2G630477592	22135454	G	0.5	0.4286	A
10	BICF2P985162	22217941	A	0.5	0.4286	G
10	BICF2P203260	22218825	G	0.5	0.4286	A
10	BICF2G630477616	22230481	A	0	0	G
10	BICF2G630477627	22247102	A	0.5	0.4286	G
10	BICF2P1171767	22257558	G	0.5	0.4286	A
10	BICF2G630477655	22266843	A	0.25	0.2143	G
10	BICF2G630477665	22277669	A	0.5	0.4286	G
10	BICF2G630477687	22295036	G	0.5	0.4286	A
10	BICF2P864547	22303498	A	0.5	0.4286	G
10	BICF2G630477709	22311210	G	0.5	0.4286	A
10	BICF2G630477729	22321916	A	0.5	0.4286	G
10	BICF2G630477747	22341555	A	0.5	0.4286	G
10	BICF2G630477755	22345647	A	0.5	0.4286	G
10	BICF2G630477771	22357421	G	0.5	0.4286	A
10	BICF2G630477785	22373594	C	0.5	0.4286	A
10	BICF2G630477791	22385267	A	0.5	0.4286	G
10	BICF2G630477799	22412123	A	0.5	0.4286	T
10	BICF2G630477805	22423701	G	0.5	0.4286	A
10	BICF2G630477816	22431549	A	0.5	0.4286	G
10	BICF2G630477848	22441887	A	0.5	0.4286	G
10	BICF2P1501613	22457894	A	0.5	0.4286	G
10	BICF2G630477888	22469396	C	0.5	0.4286	A
10	BICF2G630477916	22478140	A	0.5	0.4286	G
10	BICF2G630477918	22478756	G	0.5	0.4286	A
10	BICF2S23051244	22484452	A	0.5	0.4286	G
10	BICF2G630477949	22496690	A	0.5	0.4286	G
10	BICF2S23440743	22512341	A	0.5	0.4286	G
10	BICF2P234188	22517579	A	0.5	0.4286	C

10	BICF2G630477974	22530300	A	0.5	0.4286	G
10	BICF2G630477988	22540539	A	0.5	0.4286	G
10	BICF2G630477998	22553244	G	0.5	0.4286	A
10	BICF2P1457618	22569225	0	0	0	A
10	BICF2G630478017	22583034	G	0.5	0.4286	A
10	BICF2G630478037	22599889	G	0.5	0.4286	C
10	BICF2G630478046	22603202	A	0.5	0.4286	G
10	BICF2P391908	22609480	G	0.5	0.4286	A
10	BICF2G630478052	22625838	A	0.5	0.4286	G
10	BICF2S23656207	22639172	G	1	1	A
10	BICF2G630478117	22645059	A	1	1	G
10	BICF2G630478126	22662728	G	1	1	A
10	TIGRP2P138853_rs8954626	22668351	0	0	0	C
10	BICF2G630478142	22682034	G	1	1	A
10	BICF2G630478145	22689195	G	1	1	A
10	BICF2P1421647	22690682	A	1	1	G
10	BICF2G630478157	22706146	G	0.75	0.7857	A
10	BICF2G630478160	22717729	G	0.75	0.7857	A
10	BICF2G630478167	22729079	G	0.75	0.7857	A
10	BICF2P665689	22754002	A	0	0	G
10	BICF2S23912298	22764074	A	0	0	G
10	BICF2P469421	22778943	A	0	0	G
10	BICF2G630478171	22782488	A	0	0	G
10	BICF2G630478172	22804156	G	1	1	A
10	BICF2G630478197	22809184	A	0.5	0.5833	C
10	BICF2S22955508	22813536	G	1	1	A
10	BICF2P1268219	22821451	A	0	0	C
10	BICF2S22921836	22835461	C	0	0	A
10	BICF2G630478219	22853806	0	0	0	G
10	BICF2G630478225	22863274	A	0	0	G
10	BICF2G630478261	22875760	A	0.25	0.2143	G
10	BICF2S23627912	22885243	A	0	0	C
10	BICF2G630478301	22899045	G	0.25	0.2143	A
10	BICF2G630478327	22915081	A	0.25	0.2143	G
10	BICF2G630478352	22920571	A	0.25	0.2143	G
10	BICF2G630478365	22940260	0	0	0	G
10	BICF2G630478366	22962566	A	0	0	C
10	BICF2G630478368	22975403	G	0	0	A
10	BICF2S23312766	22986131	G	0.75	0.7857	A
10	TIGRP2P138855_rs8781050	22996271	0	0	0	A
10	BICF2P1297054	23002173	A	0	0	G
10	BICF2G630478371	23017473	A	0	0	G
10	BICF2G630478374	23031325	G	0	0	A
10	BICF2G630478398	23036389	G	0	0	A
10	BICF2G630478414	23055530	G	0.25	0.2143	A
10	BICF2G630478418	23060041	G	0.25	0.2143	A
10	BICF2P235837	23083559	A	0	0	G

10	BICF2S2309770	23097706	A	0	0	G
10	BICF2P417548	23110264	0	0	0	C
10	TIGRP2P138857_rs8560213	23122673	0	0	0	G
10	BICF2P681558	23134200	0	0	0	G
10	BICF2S2297011	23147314	A	0.25	0.1667	G
10	BICF2S23660477	23149171	A	0.75	0.7857	G
10	BICF2S22922710	23151631	A	0.25	0.2143	G
10	BICF2S22961798	23165150	A	0.25	0.2143	G
10	BICF2S23418007	23174965	G	0.75	0.7857	A
10	BICF2S23039142	23191906	A	0.75	0.7857	T
10	TIGRP2P138884_rs9238972	23218570	0	0	0	C
10	BICF2S23254840	23238155	A	0.5	0.5714	G
10	BICF2P1254506	23252830	0	NA	0	A
10	BICF2S22961647	23254590	A	0	0	G
10	BICF2P1266921	23274862	0	0	0	C
10	BICF2S23443816	23276597	A	0	0	G
10	BICF2S23225723	23296810	A	0	0	G
10	BICF2S2321625	23301594	A	0	0	G
10	TIGRP2P138885_rs8811851	23313995	0	0	0	C
10	BICF2S23763011	23329591	A	0.75	0.7857	G
10	BICF2P520027	23337289	0	0	0	C
10	BICF2S22958360	23368805	A	0	0	G
10	BICF2S22912122	23395551	A	0	0	G
10	BICF2S23354597	23403871	A	0	0	G
10	BICF2P843320	23421506	0	0	0	A
10	BICF2S23754811	23436418	G	0.75	0.7857	A
10	BICF2P1349718	23475067	0	0	0	C
10	BICF2P1299326	23516595	A	0	0	T
10	BICF2G630478423	23519257	A	0.25	0.2143	G
10	BICF2S2375146	23530983	G	0.75	0.7857	A
10	BICF2G630478460	23542730	A	0	0	G
10	BICF2G630478479	23569729	G	0.25	0.2143	A
10	BICF2G630478497	23577755	A	0	0	G
10	BICF2G630478507	23586597	A	0	0	G
10	BICF2G630478534	23604396	A	0.25	0.2143	G
10	BICF2G630478542	23619067	A	0.25	0.2143	G
10	BICF2G630478549	23628582	A	0.5	0.5714	C
10	BICF2G630478568	23638368	G	0.25	0.2143	A
10	BICF2G630478575	23644730	G	0	0	A
10	BICF2G630478620	23662622	C	0	0	A
10	BICF2G630478624	23666778	C	0	0	A
10	BICF2G630478635	23673487	A	0	0	G
10	BICF2G630478646	23684117	A	0.75	0.7857	C
10	BICF2P970656	23700037	0	0	0	A
10	BICF2P371631	23717704	0	0	0	C
10	BICF2G630478662	23733491	G	0	0	A
10	BICF2G630478665	23750626	G	0.25	0.2143	A

10	BICF2G630478667	23768657	G	0.25	0.2143	A
10	BICF2G630478669	23772829	G	0.25	0.2143	A
10	BICF2G630478675	23785661	A	0.25	0.2143	G
10	BICF2G630478678	23802783	0	0	0	G
10	BICF2G630478691	23813676	A	0	0	G
10	BICF2G630478726	23819454	0	0	0	C
10	BICF2P99091	23838094	0	0	0	G
10	BICF2G630478746	23848926	G	0	0	A
10	BICF2G630478760	23861679	G	0	0	A
10	BICF2G630478761	23862706	T	0	0	A
10	BICF2G630478775	23883228	0	0	0	A
10	BICF2P300290	23885989	A	0.5	0.5714	G
10	BICF2S23412813	23906828	C	0.75	0.7857	A
10	BICF2S23324988	23909334	C	0.5	0.5714	A
10	BICF2P500453	23928809	0	0	0	G
10	BICF2G630478794	23933962	0	0	0	C
10	BICF2G630478797	23943275	G	0.5	0.5714	A
10	BICF2P762174	23945382	A	0.5	0.5714	T
10	BICF2P1120337	23951327	A	0.25	0.2143	G
10	BICF2G630478807	23955054	A	0.25	0.2143	G
10	BICF2G630478814	23967571	G	0	0	A
10	BICF2G630478824	23969439	A	0.25	0.2143	G
10	BICF2P999052	23981154	0	0	0	A
10	BICF2G630478866	23994370	A	0	0	G
10	BICF2P720284	24010374	0	0	0	A
10	BICF2S23731603	24018745	A	0	0	G
10	BICF2P224668	24030903	T	0.5	0.5714	A
10	BICF2G630478870	24035894	G	0.5	0.5714	A
10	BICF2S23355356	24048734	G	0.25	0.2143	A
10	BICF2P568651	24062877	A	0	0	G
10	BICF2G630478886	24076577	G	0.75	0.7857	A
10	BICF2G630478893	24081845	A	0.75	0.7857	G
10	BICF2S23217631	24092628	A	0	0	G
10	BICF2G630478914	24106972	G	0.5	0.4286	A
10	BICF2G630478924	24118823	G	0.25	0.1667	A
10	BICF2P390535	24127290	A	0.5	0.2143	G
10	BICF2P573979	24145612	A	0.25	0.2143	G
10	BICF2P801304	24159608	C	0.25	0.2143	A
10	BICF2S23632430	24165797	G	0.5	0.5714	A
10	BICF2P277444	24175614	A	0.25	0.2143	G
10	BICF2G630478959	24186541	A	0.5	0.5714	G
10	BICF2G630478966	24197264	G	0	0	A
10	BICF2P155864	24207867	A	1	1	C
10	BICF2G630478981	24230443	G	0.75	0.7857	A
10	BICF2G630478986	24235029	A	0.5	0.5714	G
10	BICF2G630479000	24252341	A	0.25	0.2143	G
10	BICF2P675624	24263476	0	0	0	A

10	BICF2P1363701	24265623	A	0.25	0.2143	C
10	BICF2G630479010	24282070	A	0.25	0.2143	G
10	BICF2G630479036	24297917	G	1	1	A
10	BICF2G630479054	24310750	A	0.75	0.7857	G
10	BICF2G630479089	24318035	A	0.25	0.2143	G
10	BICF2G630479099	24322630	A	0.25	0.2143	G
10	BICF2G630479139	24341512	C	1	1	A
10	BICF2G630479147	24348989	A	0	0	C
10	BICF2G630479159	24383697	A	0	0	C
10	BICF2G630479194	24413575	A	0.75	0.8333	C
10	BICF2G630479195	24414977	G	1	1	C
10	BICF2S23145758	24430781	G	0	0	A
10	BICF2G630479205	24443590	G	0	0	A
10	BICF2P1097418	24456246	A	0.75	0.7857	G
10	BICF2G630479220	24462125	A	0	0	G
10	BICF2G630479260	24476882	A	0.75	0.7857	G
10	BICF2S23614659	24490444	0	0	0	G
10	BICF2G630479266	24495249	G	0.25	0.2143	A
10	BICF2G630479272	24511245	A	0	0	G
10	BICF2G630479294	24526332	A	0	0	G
10	BICF2P644899	24530545	G	0.25	0.2143	A
10	BICF2G630479321	24542865	A	0.25	0.2143	G
10	BICF2G630479352	24556493	A	0.25	0.2143	G
10	BICF2G630479376	24565784	A	0.25	0.2143	C
10	BICF2G630479409	24580963	A	0.25	0.2143	G
10	BICF2G630479422	24591132	G	0.25	0.2143	A
10	BICF2G630479452	24607900	C	0	0	A
10	BICF2G630479466	24614208	G	0.25	0.2143	A
10	TIGRP2P138899_rs8919447	24630568	0	0	0	C
10	BICF2G630479468	24643919	A	0	0	G
10	BICF2G630479469	24664738	A	0	0	G
10	BICF2P1405852	24670857	0	0	0	C
10	BICF2S23023369	24684607	0	0	0	G
10	BICF2P701195	24701967	A	0	0	G
10	TIGRP2P138901_rs8855903	24708228	0	0	0	C
10	BICF2P724328	24731582	A	0	0	G
10	BICF2P449997	24737343	G	1	1	A
10	BICF2P423374	24745907	A	0	0	G
10	BICF2P505563	24750145	A	0	0	G
10	BICF2P1238038	24759819	A	0.25	0.2143	G
10	BICF2P658421	24780598	A	0	0	G
10	BICF2S23614505	24784231	C	0	0	A
10	TIGRP2P138902_rs8756618	24803017	0	0	0	G
10	BICF2S23417685	24811844	A	0	0	C
10	BICF2P187940	24819649	G	0	0	A
10	BICF2P498253	24859477	A	0	0	G
10	BICF2P935185	24864948	A	0	0	G

10	BICF2P845591	24882859	A	0.25	0.2143	G
10	BICF2P860441	24886154	A	0	0	G
10	BICF2P266140	24901701	C	0	0	G
10	BICF2P1000590	24916401	A	0.25	0.2143	G
10	BICF2S2441307	24948056	A	0.25	0.2143	T
10	BICF2S23225309	24982440	A	0.25	0.2143	G
10	BICF2S23541070	25001305	G	0.25	0.2143	A
10	BICF2S23240926	25015047	A	1	1	G
10	BICF2S23620136	25024181	A	0.75	0.7857	C
10	BICF2P293757	25044348	0	0	0	C
10	BICF2P120597	25053733	0	0	0	A
10	BICF2P1122245	25058906	0	0	0	A
10	BICF2P794060	25072419	G	1	1	A
10	BICF2P293118	25086591	A	0	0	T
10	BICF2S2291091	25102339	G	0.25	0.2143	A
10	BICF2P1405561	25111013	0	0	0	A
10	BICF2P1103002	25117596	G	0.25	0.2143	A
10	BICF2P255600	25128691	G	0.25	0.2143	A
10	BICF2P7902	25142880	A	0.25	0.2143	G
10	BICF2P28615	25161525	A	0.25	0.2143	G
10	BICF2P621546	25163665	G	0.25	0.2143	A
10	BICF2P1195488	25178947	G	0	0	A
10	BICF2P633946	25192688	A	0	0	G
10	BICF2P1343887	25203052	0	0	0	C
10	BICF2P395862	25210626	A	0	0	G
10	BICF2P686473	25221307	G	0	0	A
10	BICF2P38160	25233363	0	0	0	C
10	BICF2P1379452	25260851	0	0	0	G
10	BICF2S23047093	25268125	0	0	0	T
10	BICF2P626698	25286050	A	0	0	G
10	BICF2P96245	25292525	0	0	0	G
10	BICF2P326837	25303662	G	0.75	0.7857	A
10	BICF2P921727	25339740	A	0	0	G
10	BICF2P921796	25346215	A	1	1	G
10	BICF2P1150585	25359914	G	0	0	A
10	BICF2P1369320	25369165	G	1	1	A
10	BICF2S22942696	25382756	C	0.25	0.2143	A
10	BICF2S23731219	25413986	0	0	0	G
10	BICF2P34890	25416202	A	0	0	G
10	BICF2S23141456	25437245	C	0.25	0.2143	G
10	BICF2S22922635	25440578	A	0.25	0.2143	G
10	BICF2S24416376	25449712	A	0.75	0.8333	G
10	BICF2P578928	25464137	A	0	0	G
10	BICF2P680237	25473368	G	0.25	0.2143	A
10	BICF2P500368	25486667	G	0	0	A
10	BICF2S22920265	25496024	G	0.25	0.2143	A
10	BICF2P467953	25511288	0	0	0	G

10	BICF2P107435	25523549	0	0	0	T
10	BICF2P97539	25540933	A	0	0	G
10	BICF2P225222	25543712	0	0	0	C
10	BICF2P858747	25556556	A	0.25	0.2143	G
10	BICF2P253015	25574436	A	0	0	G
10	BICF2P1191743	25587259	0	0	0	G
10	BICF2P611768	25612161	0	0	0	G
10	BICF2P549512	25635053	G	0.75	0.7857	A
10	BICF2S23741999	25649991	0	0	0	A
10	BICF2P1383491	25657039	C	0	0	G
10	BICF2P1383492	25657545	G	0.75	0.7857	C
10	BICF2P418144	25663694	A	0.75	0.7857	G
10	BICF2P1221052	25673093	A	0	0	C
10	BICF2S2411339	25687611	A	0	0	G
10	BICF2P106912	25691673	G	0	0	A
10	BICF2P722107	25703920	A	0.75	0.7857	G
10	BICF2S23760981	25722314	A	0	0.2143	G
10	BICF2P1392199	25730052	C	0	0	A
10	BICF2P1096676	25745303	A	0.25	0.2143	G
10	BICF2P1446201	25752826	G	0	0	A
10	BICF2P1406081	25767830	A	0.75	0.7857	G
10	BICF2P1406080	25767881	G	0.75	0.7857	A
10	BICF2P591	25774163	C	1	1	A
10	BICF2S23134002	25792024	A	1	1	C
10	TIGRP2P138927_rs9178835	25804310	0	0	0	A
10	BICF2P365292	25817157	0	0	0	C
10	BICF2P716505	25820311	G	0	0	A
10	BICF2P373368	25838030	A	0.25	0.2143	G
10	BICF2P384880	25843638	A	0	0	G
10	BICF2P1110640	25853208	A	0	0	G
10	BICF2P1329068	25870135	A	0	0	G
10	BICF2P1405476	25877921	A	0	0	G
10	BICF2P710967	25895601	G	0	0	A
10	BICF2P143905	25902790	A	0	0	G
10	BICF2P436474	25915896	0	0	0	G
10	BICF2S23049369	25929812	0	0	0	A
10	BICF2P302853	25936817	0	0	0	G
10	BICF2S23453542	25952701	G	0	0	A
10	BICF2P458474	25957370	A	0	0	G
10	BICF2S23341051	25969044	A	0	0	G
10	BICF2P915238	26006066	A	0	0	G
10	BICF2P451067	26022690	G	0	0	A
10	BICF2P1421854	26034926	A	0	0	G
10	BICF2P1068149	26043959	A	0	0	C
10	BICF2P273115	26063353	G	0	0	A
10	BICF2P1153978	26076455	G	0	0	A
10	BICF2P937538	26097105	A	0	0	G

10	BICF2S2338701	26109233	A	0	0	G
10	BICF2P993573	26119086	0	0	0	G
10	BICF2S23254187	26139321	A	0	0	G
10	BICF2S2352701	26159399	A	1	1	G
10	BICF2S23620859	26172348	A	1	1	G
10	BICF2S23316193	26177251	C	1	1	G
10	BICF2S23419855	26185551	G	1	1	C
10	BICF2P373029	26216419	0	0	0	A
10	BICF2P614390	26234731	0	0	0	A
10	BICF2S24413320	26255452	A	0	0	G
10	BICF2S23553130	26267162	A	0	0	G
10	BICF2P514912	26283193	0	0	0	G
10	BICF2S237390	26305822	G	1	1	A
10	BICF2S23416847	26369162	C	1	1	A
10	BICF2S23348046	26376714	C	0	0	G
10	BICF2S23549137	26418638	0	0	0	C
10	BICF2P526856	26435525	0	0	0	C
10	BICF2S23612887	26440690	A	0	0	G
10	BICF2S23548114	26465365	A	0	0	G
10	BICF2G630479488	26474395	A	0	0	G
10	BICF2S24116484	26489495	C	0	0	A
10	BICF2P740631	26496871	A	0	0	C
10	BICF2G630479505	26515070	G	0	0	A
10	BICF2P613853	26519542	0	0	0	G
10	G591f53S23	26544854	A	0	0	G
10	BICF2G630479506	26568383	A	1	1	C
10	BICF2P511905	26605626	0	0	0	A
10	BICF2P193245	26616318	0	0	0	C
10	BICF2G630479515	26625528	A	0	0	G
10	BICF2G630479516	26640723	G	0	0	A
10	BICF2G630479522	26649403	G	0	0	A
10	BICF2G630479527	26685677	G	1	1	A
10	BICF2G630479530	26705650	A	0	0	C
10	BICF2G630479531	26725806	A	1	1	C
10	BICF2P1022170	26737348	0	0	0	A
10	BICF2P147609	26739207	0	0	0	G
10	BICF2S22919932	26760275	A	0	0	G
10	BICF2S2297831	26764039	G	0	0	A
10	TIGRP2P138950_rs8688784	26774397	0	0	0	G
10	BICF2P1074346	26788124	G	0	0	A
10	BICF2P302477	26798069	0	0	0	G
10	BICF2P899683	26811503	A	0	0	G
10	BICF2S24415766	26825274	G	0	0	A
10	BICF2P139518	26859907	G	0	0	A
10	BICF2S2393307	26878650	G	0	0	A
10	BICF2G630479539	26887899	A	0	0	G
10	TIGRP2P138951_rs8710659	26902510	0	0	0	G

10	BICF2G630479553	26913515	A	1	1	G
10	BICF2G630479564	26930101	C	1	1	A
10	BICF2P437724	26986510	G	0	0	A
10	BICF2P595974	26995373	0	0	0	C
10	BICF2G630479566	27008667	G	0	0	A
10	BICF2P861764	27017542	0	0	0	C
10	BICF2G630479567	27035698	A	0	0	G
10	BICF2S2343111	27051691	T	0	0	A
10	BICF2P770847	27068594	0	0	0	C
10	BICF2G630479569	27091084	A	0	0	G
10	BICF2S2343115	27119242	C	0	0	G
10	BICF2P124410	27144297	0	0	0	A
10	BICF2P467422	27153536	0	0	0	A
10	BICF2S2437318	27163464	T	0	0	A
10	BICF2P508091	27180081	0	0	0	A
10	BICF2P735960	27197266	0	0	0	A
10	BICF2P846210	27216107	0	0	0	G
10	BICF2G630479574	27249094	A	0	0	C
10	BICF2G630479576	27286453	A	1	1	G
10	BICF2G630479593	27290027	A	1	1	G
10	BICF2P214331	27302496	0	0	0	A
10	BICF2G630479641	27333038	A	0.25	0.2143	C
10	BICF2G630479645	27336610	A	1	1	G
10	BICF2G630479656	27362056	G	0	0	A
10	BICF2G630479660	27372914	A	0	0	G
10	BICF2G630479661	27382951	G	0	0	A
10	BICF2P429596	27406026	0	0	0	G
10	BICF2S2345856	27430553	A	0	0	G
10	BICF2G630479687	27447367	G	0	0	A
10	BICF2G630479692	27450838	A	0	0	T
10	BICF2G630479722	27469289	A	0	0	G
10	BICF2G630479756	27496654	A	0	0	C
10	BICF2G630479770	27510776	G	0	0	A
10	BICF2S23212868	27520501	A	0	0	G
10	BICF2G630479796	27531056	A	0	0	G
10	BICF2P63501	27551176	T	0	0	A
10	BICF2P1258837	27556175	G	0	0	A
10	BICF2G630479858	27592716	A	0	0	G
10	BICF2P1322925	27608856	G	0	0	A
10	BICF2G630479872	27617766	T	0	0	A
10	BICF2G630479874	27624226	A	0	0	G
10	BICF2P546574	27644959	0	0	0	A
10	BICF2S23150971	27652075	G	0	0	A
10	BICF2G630479879	27659686	C	0	0	A
10	BICF2G630479883	27675650	G	0	0	A
10	BICF2P501728	27695725	A	0	0	G
10	BICF2P858171	27706507	A	0	0	G

10	BICF2S2444969	27726241	0	0	0	A
10	BICF2G630479896	27728559	A	0	0	G
10	BICF2S23223941	27745162	0	0	0	G
10	BICF2G630479899	27754718	C	0	0	A
10	BICF2G630479905	27787936	0	0	0	G
10	BICF2G630479908	27805633	A	0	0	G
10	BICF2P539900	27807343	A	0	0	G
10	BICF2P539899	27808738	0	0	0	A
10	BICF2G630479915	27822055	0	0	0	G
10	BICF2G630479918	27837113	0	0	0	G
10	BICF2S23343127	27866141	A	0	0	G
10	BICF2S2313423	27885444	0	0	0	G
10	BICF2G630479932	27896307	0	0	0	G
10	BICF2P1267216	27909117	A	0	0	G
10	BICF2G630479934	27915422	0	0	0	G
10	TIGRP2P138999_rs9019897	27956797	0	0	0	G
10	BICF2P157476	27974681	0	0	0	A
10	BICF2G630479937	27989652	A	0	0	G
10	BICF2P526343	27999824	0	0	0	G
10	BICF2P627139	28004122	0	0	0	A
10	BICF2S22914364	28022932	C	0	0	G
10	BICF2P411485	28028909	0	0	0	G
10	BICF2G630479938	28038280	0	0	0	G
10	BICF2G630479941	28052916	C	0	0	A
10	BICF2G630479947	28061484	A	0	0	C
10	BICF2G630479959	28088975	A	0	0	G
10	BICF2G630479971	28101803	C	1	1	G
10	BICF2G630479978	28114588	A	1	1	G
10	BICF2G630479987	28127871	G	1	1	A
10	BICF2G630479989	28134023	G	1	1	A
10	BICF2G630480006	28148299	A	0	0	G
10	BICF2G630480010	28155698	G	0	0	A
10	BICF2G630480016	28168522	C	0	0	A
10	BICF2G630480031	28179883	A	0	0	G
10	BICF2G630480053	28193737	G	1	1	A
10	BICF2G630480060	28198028	A	1	1	G
10	BICF2P533951	28213655	0	0	0	C
10	BICF2G630480080	28231373	G	0	0	A
10	BICF2P1359589	28242551	G	0	0	A
10	BICF2P599991	28254423	0	0	0	C
10	BICF2G630480106	28265348	A	1	1	G
10	BICF2S23031835	28271671	A	1	1	G
10	BICF2P690393	28281504	0	0	0	T
10	BICF2G630480120	28299320	A	1	1	G
10	BICF2S23241272	28307578	A	0	0	G
10	BICF2G630480137	28319763	G	0	0	A
10	BICF2G630480148	28330929	G	0	0	A

10	BICF2G630480153	28343168	A	1	1	G
10	BICF2G630480157	28351202	A	1	1	G
10	BICF2G630480176	28369144	A	0	0	G
10	BICF2G630480182	28376137	A	1	1	G
10	BICF2G630480196	28392403	A	0	0	G
10	BICF2G630480198	28395898	C	0	0	A
10	TIGRP2P139006_rs8858819	28414175	0	0	0	C
10	BICF2G630480205	28418845	A	1	1	G
10	BICF2G630480215	28424937	A	1	1	G
10	BICF2G630480227	28428303	A	1	1	G
10	BICF2G630480253	28441191	A	1	1	G
10	BICF2S2303469	28455265	A	0	0	G
10	BICF2G630480267	28466658	A	0	0	G
10	BICF2G630480288	28477259	C	0	0	A
10	BICF2G630480301	28485598	T	0	0	A
10	BICF2P858138	28497264	A	0	0	G
10	BICF2P352626	28517955	G	0	0	A
10	BICF2S22955637	28525628	A	0	0.125	G
10	BICF2G630480335	28536849	A	0	0	G
10	BICF2P1346434	28545838	0	0	0	G
10	BICF2G630480347	28559396	A	1	1	G
10	BICF2S2356319	28571342	A	1	1	G
10	BICF2S23424933	28580298	A	1	1	G
10	BICF2G630480354	28589111	C	0	0	A
10	BICF2P429659	28602493	A	0	0	T
10	BICF2P873939	28611525	A	0	0	G
10	BICF2P287803	28618127	A	0	0	G
10	BICF2P1250813	28623870	A	0	0	G
10	BICF2P803201	28634185	A	1	1	C
10	BICF2S23427091	28653741	G	0	0	A
10	BICF2S23454756	28669053	A	0	0	G
10	BICF2G630480363	28679071	G	0	0	A
10	BICF2G630480370	28683599	A	1	1	G
10	BICF2S23210932	28703870	A	1	1	G
10	BICF2G630480381	28704923	A	0	0	G
10	BICF2G630480390	28730144	A	1	1	G
10	BICF2P1026927	28739263	A	0	0	G
10	BICF2S23617771	28757040	A	0	0	G
10	BICF2G630480418	28767979	A	0	0	G
10	BICF2G630480420	28780641	G	0	0	A
10	BICF2S23521427	28785076	G	0	0	A
10	BICF2P1225894	28799474	G	0	0	A
10	BICF2P1327063	28807405	G	1	1	A
10	BICF2P1260545	28811570	G	0	0	A
10	BICF2P1002095	28822740	A	0	0	G
10	BICF2G630480475	28831926	G	1	1	A
10	BICF2P1258797	28845807	A	0	0	C

10	BICF2G630480494	28856014	G	0	0	C
10	BICF2G630480499	28870183	G	0	0	C
10	BICF2G630480510	28887899	A	0	0	G
10	BICF2G630480527	28897956	G	0	0	C
10	BICF2G630480539	28915781	A	0	0	G
10	BICF2G630480548	28924870	A	0	0	G
10	BICF2P863133	28945319	0	0	0	A
10	BICF2S23751063	28947049	G	1	1	A
10	BICF2P499640	28958264	A	0	0	G
10	BICF2G630480574	28978764	C	0	0	A
10	BICF2G630480575	28982590	G	0	0	A
10	BICF2S23430126	29002046	G	0	0	A
10	BICF2P1096973	29036575	0	0	0	C
10	BICF2P457782	29047934	A	1	1	G
10	BICF2P627080	29060173	G	0	0	C
10	BICF2P122978	29065144	A	0	0	C
10	BICF2G630480580	29083141	A	0	0	G
10	BICF2G630480586	29094330	A	0	0	G
10	BICF2G630480588	29095396	A	0	0	G
10	BICF2G630480614	29108951	A	0	0	G
10	BICF2G630480618	29121420	G	0	0	A
10	BICF2G630480625	29131162	A	0	0	G
10	BICF2P636949	29142653	A	1	1	G
10	BICF2G630480641	29144137	A	0	0	G
10	BICF2G630480647	29147943	A	0	0	G
10	BICF2G630480648	29148511	A	0	0	G
10	BICF2G630480656	29153068	A	0	0	G
10	BICF2G630480666	29166027	A	0	0	G
10	BICF2G630480671	29170470	C	0	0	A
10	BICF2G630480680	29184257	A	0	0	T
10	BICF2P743549	29200609	0	0	0	A
10	BICF2G630480698	29211789	A	0	0	G
10	BICF2G630480710	29221656	A	0	0	G
10	BICF2P1464569	29238272	G	1	1	C
10	BICF2P699387	29246912	A	1	1	G
10	BICF2G630480756	29256503	G	0	0	A
10	BICF2G630480778	29291533	A	0	0	C
10	BICF2G630480790	29309276	A	0	0	G
10	BICF2G630480793	29313604	T	1	1	A
10	BICF2P177725	29328954	A	1	1	G
10	BICF2P291764	29333941	G	1	1	A
10	BICF2P965381	29339598	G	1	1	A
10	BICF2G630480810	29357970	G	1	1	A
10	TIGRP2P139030_rs9048754	29360072	0	0	0	C
10	BICF2P484531	29360412	G	1	1	A
10	BICF2P422982	29375782	A	0	0	G
10	BICF2P1070918	29387001	G	0	0	A

10	BICF2P1087828	29397063	C	1	1	A
10	BICF2G630480826	29411685	A	0	0	G
10	BICF2P434453	29421522	0	0	0	C
10	BICF2P955401	29428913	C	1	1	G
10	BICF2P240795	29454610	0	0	0	G
10	BICF2S23048635	29484499	A	1	1	G
10	BICF2S22950227	29502228	C	0	0	A
10	TIGRP2P139031_rs9046711	29509997	0	0	0	C
10	BICF2P298144	29549256	0	0	0	C
10	BICF2S2331376	29559969	C	0	0	G
10	BICF2G630480849	29569581	G	0	0	A
10	BICF2G630480855	29581019	A	0	0	G
10	BICF2P824888	29583609	A	0	0	G
10	BICF2G630480862	29610414	A	0	0	G
10	TIGRP2P139033_rs8969533	29617159	0	0	0	G
10	BICF2P375792	29628142	0	0	0	G
10	BICF2G630480874	29646273	A	1	1	C
10	BICF2G630480878	29656570	A	0	0	G
10	BICF2G630480881	29661047	A	0	0	G
10	BICF2P567674	29679320	G	0	0	A
10	BICF2S23745558	29682963	C	0	0	G
10	BICF2G630480887	29693403	G	0	0	A
10	BICF2S23715660	29723874	C	0	0	A
10	BICF2S23518284	29735350	A	0	0	G
10	BICF2P178140	29746894	A	0	0	G
10	BICF2G630480954	29756061	G	1	1	A
10	BICF2G630480963	29765847	A	1	1	G
10	BICF2G630480983	29783274	G	1	1	A
10	BICF2G630480987	29786485	G	1	1	A
10	BICF2G630481003	29798765	G	1	1	A
10	BICF2G630481028	29812523	A	1	1	G
10	BICF2G630481045	29825115	A	0	0	G
10	BICF2G630481055	29842662	C	0	0	A
10	BICF2G630481078	29860593	A	1	1	G
10	BICF2G630481087	29869233	A	0	0	G
10	BICF2P1128224	29882451	C	1	1	G
10	BICF2G630481096	29893305	G	0	0	C
10	BICF2G630481097	29900366	A	0	0	G
10	BICF2G630481115	29929504	A	1	1	G
10	TIGRP2P139048_rs8547396	29938312	0	0	0	G
10	BICF2P1033341	29948702	A	0	0	G
10	BICF2G630481131	29961010	C	0	0	A
10	BICF2P328126	29989763	A	0	0	G
10	BICF2G630481139	29995659	A	0	0	G
10	BICF2G630481140	29996199	G	1	1	A
10	BICF2P1283413	30011809	A	0	0	G
10	BICF2P782194	30019215	A	1	1	G

10	BICF2P631135	30028099	A	0	0	T
10	BICF2G630481154	30045156	A	0	0	G
10	BICF2P652681	30050768	0	0	0	G
10	BICF2P633255	30062226	A	0	0	G
10	TIGRP2P139052_rs8924301	30076310	0	0	0	G
10	BICF2P22069	30087743	0	0	0	G
10	BICF2P906018	30118992	0	0	0	C
10	BICF2S23515819	30138371	A	0	0	G
10	BICF2P683135	30145984	0	0	0	C
10	BICF2P1048342	30164176	A	0	0	G
10	BICF2S2307644	30170795	A	0	0	G
10	BICF2P694040	30183338	G	0	0	A
10	BICF2P803749	30197617	0	0	0	G
10	BICF2P585845	30203858	A	0	0	G
10	BICF2P675081	30215055	G	0	0	C
10	BICF2P284563	30224871	0	0	0	T
10	BICF2G630481165	30254753	G	0.25	0.2143	A
10	BICF2S2366419	30256994	G	0.25	0.2143	A
10	BICF2S23126999	30289594	C	0.25	0.2143	G
10	BICF2S23126865	30293283	T	0.25	0.2143	A
10	BICF2G630481173	30306341	G	0	0	A
10	BICF2P1223854	30328432	A	0.25	0.2143	G
10	BICF2P948046	30341380	A	0	0	G
10	BICF2P804111	30348711	A	0.75	0.8333	G
10	BICF2P1360603	30383943	G	0.75	0.7857	A
10	BICF2P1227714	30401094	G	0.75	0.7857	A
10	BICF2P85217	30407615	A	0.75	0.7857	C
10	BICF2S23511874	30417661	A	0.75	0.7857	G
10	BICF2G630481210	30442159	A	0	0	G
10	BICF2P503104	30465695	0	0	0	C
10	BICF2P261919	30475844	A	0	0	G
10	BICF2G630481229	30487275	A	0	0	G
10	BICF2G630481235	30506754	A	0.25	0.1667	C
10	BICF2G630481242	30510101	A	0.75	0.7857	G
10	BICF2G630481246	30531921	A	0	0	G
10	BICF2P901008	30556612	A	0.75	0.7857	G
10	BICF2P196644	30568894	A	0	0	G
10	BICF2P160827	30587517	G	0.75	0.7857	A
10	BICF2P953818	30591042	A	0.75	0.7857	G
10	BICF2S2291658	30619221	A	0.75	0.7857	G
10	BICF2G630481282	30634450	A	0	0	C
10	BICF2G630481302	30645633	G	0	0	A
10	BICF2G630481304	30646249	G	0	0	A
10	BICF2G630481321	30654523	A	0	0	C
10	BICF2G630481327	30659863	A	0	0	C
10	BICF2G630481331	30671763	A	0	0	G
10	BICF2G630481341	30686326	A	0	0	G

10	BICF2P659334	30703917	C	0	0	A
10	BICF2G630481362	30718289	A	0.25	0.2143	G
10	BICF2P813464	30728672	A	0.25	0.2143	G
10	BICF2S23763790	30753175	A	0.25	0.2143	G
10	BICF2P254803	30756285	C	0	0	A
10	BICF2G630481401	30768736	G	0	0	A
10	BICF2G630481415	30785330	A	0	0	G
10	BICF2G630481417	30786024	G	0.25	0.2143	A
10	BICF2G630481447	30797637	A	0.25	0.2143	C
10	BICF2G630481458	30810041	C	0.25	0.2143	A
10	BICF2G630481465	30820492	G	0	0	A
10	BICF2P989091	30833589	A	0.25	0.2143	G
10	BICF2P781865	30847094	G	0.25	0.2143	A
10	BICF2G630481479	30858274	G	0	0	A
10	BICF2G630481484	30870742	C	0	0	A
10	BICF2G630481501	30889828	A	0	0	G
10	BICF2G630481508	30906324	G	0.25	0.2143	A
10	BICF2G630481517	30915373	A	0.25	0.2143	T
10	BICF2G630481530	30927164	G	0	0	C
10	BICF2G630481540	30951911	C	1	1	A
10	BICF2G630481564	30964709	G	0.5	0.5	A
10	BICF2P470923	30976227	A	1	1	G
10	BICF2P870186	30991821	C	1	1	A
10	BICF2G630481596	30995122	A	1	1	C
10	BICF2G630481637	31008291	G	1	1	A
10	BICF2P238969	31021388	C	0.75	0.7143	A
10	BICF2P1461580	31044184	A	0.75	0.7143	G
10	BICF2P1175008	31058053	A	0.5	0.5	G
10	BICF2P1091213	31067467	G	0	0	A
10	BICF2G630481664	31074678	A	0.75	0.7143	G
10	BICF2P1234798	31087984	A	0	0	G
10	BICF2S23550073	31089847	C	0.25	0.2143	A
10	BICF2G630481716	31098988	C	0	0	A
10	BICF2G630481734	31111495	A	0	0	G
10	BICF2G630481738	31115476	A	0.75	0.75	G
10	BICF2G630481751	31128725	A	0.75	0.7143	G
10	BICF2G630481758	31134023	A	0.75	0.7143	C
10	BICF2G630481762	31145292	A	0.25	0.2143	G
10	BICF2G630481767	31154636	A	0	0	G
10	BICF2G630481773	31161067	A	0.25	0.2143	G
10	BICF2G630481777	31167560	A	0.25	0.2143	T
10	BICF2P693194	31178952	0	0	0	G
10	BICF2G630481785	31188654	A	0.25	0.2143	G
10	BICF2G630481793	31204463	A	0.25	0	C
10	BICF2P1272248	31220644	A	0.5	0.5	C
10	BICF2P1130920	31224224	A	0.5	0.5	G
10	BICF2P705560	31234860	A	1	1	G

10	BICF2P1403831	31247405	A	0.75	0.7143	G
10	BICF2G630481843	31276530	A	0.5	0.5	G
10	BICF2G630481850	31311505	A	0.5	0.5	G
10	BICF2S23031816	31320947	A	0.5	0.5	G
10	BICF2P131327	31341375	0	0	0	C
10	BICF2G630481853	31380951	G	0.75	0.7143	C
10	BICF2S23538928	31389876	A	0.5	0.5	G
10	BICF2G630481854	31398282	G	0	0	A
10	BICF2P264101	31440483	0	0	0	A
10	BICF2G630481856	31453416	A	0	0	G
10	BICF2P558841	31471268	0	0	0	G
10	BICF2P607205	31481248	A	0	0	C
10	BICF2S23655938	31494278	G	0.75	0.7143	A
10	BICF2S23021687	31512564	A	0.5	0.5	G
10	BICF2P499816	31529957	A	0	0	G
10	BICF2S23323501	31546731	A	0.75	0.7143	G
10	BICF2P984626	31557095	0	0	0	G
10	BICF2G630481869	31588776	G	0.25	0.2857	A
10	BICF2G630481882	31595217	G	0.25	0.2143	A
10	BICF2P643538	31603131	A	0	0	G
10	BICF2G630481933	31618974	G	0	0	A
10	BICF2G630481980	31637315	C	0.5	0.5	A
10	BICF2P25510	31648690	A	0	0	G
10	BICF2P419905	31659642	A	0	0	G
10	BICF2S23511731	31677742	A	0.25	0.2143	G
10	BICF2G630482010	31685353	G	0.25	0.2143	A
10	BICF2G630482027	31694344	G	0.25	0.2143	A
10	BICF2G630482037	31714912	A	1	0.8333	G
10	BICF2P645219	31725189	A	0.25	0.2143	T
10	BICF2S23228865	31737530	0	0	0	G
10	BICF2P664731	31759009	C	0.25	0.2857	A
10	BICF2G630482063	31764736	C	0.25	0.2857	A
10	BICF2G630482113	31781049	A	0.25	0.2857	G
10	BICF2G630482135	31794383	A	0.25	0.2857	G
10	BICF2G630482138	31799140	A	0.25	0.2857	G
10	BICF2S22932828	31816674	A	0.5	0.5	G
10	BICF2G630482158	31828872	A	0	0	G
10	BICF2S23749083	31835290	A	0	0	G
10	BICF2P1440469	31852506	0	0	0	G
10	BICF2P1412373	31859130	A	0	0	G
10	BICF2P228913	31869902	0	0	0	A
10	BICF2G630482171	31894441	A	0	0	G
10	BICF2G630482174	31903230	A	0	0	G
10	BICF2G630482180	31915896	0	0	0	G
10	BICF2G630482183	31925613	G	0	0	A
10	BICF2G630482201	31942130	G	0.25	0.2857	A
10	BICF2G630482212	31951428	A	0	0	G

10	BICF2P801503	31962025	A	0	0	G
10	BICF2P1231353	31971455	A	0	0	G
10	BICF2P526424	31984573	A	0	0	G
10	BICF2G630482245	31998236	A	0.75	0.7143	G
10	BICF2G630482255	32004905	G	0.75	0.7143	A
10	BICF2G630482257	32017218	G	0.75	0.7143	A
10	BICF2P1052419	32050372	C	0	0	G
10	BICF2G630482303	32055294	A	0.25	0.2857	G
10	BICF2G630482308	32069848	0	0	0	C
10	BICF2P569451	32074999	G	0	0	A
10	BICF2G630482320	32087195	G	0.25	0.2857	A
10	BICF2G630482328	32096502	A	0.5	0.5	G
10	BICF2P846592	32130810	G	0.25	0.2857	A
10	BICF2G630482347	32135313	A	0	0	G
10	BICF2G630482361	32142710	A	0	0	G
10	BICF2G630482381	32160246	A	0.5	0.5	G
10	BICF2G630482386	32167224	G	0.5	0.5	A
10	BICF2G630482397	32181570	A	0	0	G
10	BICF2P1499453	32204394	G	0	0	A
10	BICF2G630482460	32217199	G	0	0	A
10	BICF2G630482472	32228192	A	0.75	0.7857	G
10	BICF2G630482473	32258739	C	0.75	0.7857	G
10	BICF2G630482474	32272203	A	0	0	T
10	BICF2G630482475	32296059	G	0.5	0.5	A
10	BICF2S23634087	32310484	A	0	0	G
10	BICF2G630482478	32318202	A	0	0	G
10	BICF2G630482484	32326764	G	0	0	A
10	BICF2G630482494	32347459	A	0	0	G
10	BICF2G630482510	32358578	A	0.25	0.2143	T
10	BICF2G630482514	32371738	G	0	0	A
10	BICF2G630482525	32377908	G	0	0	A
10	BICF2G630482532	32386892	A	0	0	G
10	BICF2G630482537	32397486	A	0	0	G
10	BICF2G630482551	32417208	A	0	0	G
10	BICF2G630482561	32422346	A	0	0	G
10	BICF2G630482625	32438437	C	0	0	A
10	BICF2G630482639	32443065	G	0.25	0.2143	A
10	BICF2G630482650	32453140	A	0.25	0.2143	C
10	BICF2G630482676	32475297	T	0.25	0.25	A
10	BICF2G630482686	32481184	A	0	0	G
10	BICF2G630482699	32495782	A	0.25	0.2857	G
10	BICF2P592822	32530279	0	0	0	C
10	BICF2P116460	32536946	0	0	0	A
10	BICF2P720363	32548482	A	0	0	G
10	BICF2G630482708	32566255	G	0.5	0.5	A
10	BICF2P945657	32574281	A	0	0	C
10	BICF2G630482722	32582792	A	0.25	0.2143	G

10	BICF2P622311	32598504	A	0	0	G
10	BICF2S23254641	32601327	G	0.25	0.2143	A
10	BICF2P1026925	32610453	G	0.5	0.5	A
10	BICF2G630482731	32610626	0	0	0	G
10	BICF2G630482736	32617406	A	0.25	0.2143	G
10	BICF2G630482749	32636451	A	0.25	0.2143	C
10	BICF2G630482753	32641910	A	0.25	0.2857	G
10	BICF2G630482757	32649952	A	0.25	0.2857	G
10	BICF2G630482772	32661807	A	0.5	0.5	C
10	BICF2G630482793	32672328	A	0.5	0.5	G
10	BICF2P76987	32683899	A	0.5	0.3333	G
10	BICF2S23115820	32692017	A	0.25	0.2143	G
10	BICF2P692040	32708941	G	0.5	0.5	A
10	BICF2P1027088	32727030	A	0.5	0.5	G
10	BICF2P458155	32729129	A	0.25	0.2857	G
10	BICF2G630482803	32745020	0	0	0	A
10	BICF2P1442573	32753732	0	0	0	C
10	BICF2S2342652	32764278	G	0.25	0.2857	A
10	BICF2P224297	32765834	G	0.25	0.2857	A
10	BICF2G630482828	32776929	A	0.5	0.5	G
10	BICF2G630482849	32792764	C	0	0	A
10	BICF2G630482873	32809636	A	0.25	0.2857	G
10	BICF2G630482883	32821367	G	0.25	0.2857	C
10	BICF2P485069	32833140	0	0	0	A
10	BICF2S24117345	32846852	A	0.25	0.2857	G
10	BICF2P1376911	32857479	G	0	0	A
10	BICF2P1356547	32868354	0	0	0	G
10	BICF2P1115174	32898129	C	1	0.75	A
10	BICF2G630482891	32917906	G	0	0	A
10	BICF2G630482894	32926024	A	0	0	G
10	BICF2S23646033	32938690	G	0	0	A
10	BICF2G630482908	32947939	A	0.5	0.5	G
10	BICF2G630482928	32966040	A	0	0	G
10	BICF2G630482936	32969930	G	0.25	0.2143	C
10	BICF2P519982	32975151	G	0.25	0.2143	A
10	BICF2G630482949	32986306	G	0.25	0.2857	A
10	BICF2G630482966	33004123	G	0.25	0.2143	A
10	BICF2G630482967	33006592	A	0.5	0.5	G
10	BICF2S23727941	33021142	G	0.5	0.5	A
10	BICF2G630482978	33071063	A	0	0	C
10	BICF2P215267	33108030	0	0	0	C
10	BICF2G630482980	33116849	A	0.5	0.5	G
10	BICF2G630482990	33123810	G	0.5	0.5	A
10	BICF2S22946995	33134376	G	0.5	0.5	A
10	BICF2S23046311	33147353	A	0	0	G
10	BICF2P119369	33155249	A	0.25	0.2857	G
10	BICF2S23452942	33171776	A	0	0	G

10	BICF2P673602	33188495	G	0	0	A
10	TIGRP2P139102_rs9032917	33192025	0	0	0	G
10	BICF2P340937	33204931	G	0	0	A
10	BICF2S23122996	33220018	C	0.25	0.2143	A
10	BICF2P203687	33230819	0	0	0	G
10	BICF2S22930557	33268557	A	0.25	0.2857	G
10	BICF2S22934653	33272839	C	0.75	0.7143	A
10	BICF2S23257104	33283422	A	0.25	0.2857	G
10	BICF2P1118630	33304322	G	0.25	0.2143	A
10	BICF2G630483055	33337475	A	0.25	0.2857	G
10	BICF2G630483060	33346874	G	0.25	0.2857	A
10	BICF2S23626371	33360149	G	0.5	0.5	C
10	BICF2G630483082	33362950	A	0	0	G
10	BICF2G630483090	33377776	A	0.25	0.2857	G
10	BICF2G630483116	33384701	G	0.25	0.2857	A
10	BICF2G630483134	33398943	G	0.25	0.2857	A
10	BICF2G630483149	33410478	A	0.5	0.5	G
10	BICF2G630483171	33422749	G	0.25	0.2857	A
10	BICF2P916298	33439357	C	0	0	A
10	BICF2P195845	33440968	G	0.25	0.2143	A
10	BICF2G630483204	33443017	A	0	0	G
10	BICF2P882366	33459715	A	0	0.2857	G
10	BICF2G630483226	33465211	A	0	0	C
10	BICF2G630483265	33476717	G	0.25	0.2857	A
10	BICF2G630483294	33498510	C	0	0	A
10	BICF2G630483295	33501973	A	0	0	G
10	BICF2G630483311	33511036	A	0.5	0.5	C
10	BICF2G630483342	33526072	A	0	0	G
10	BICF2G630483348	33534843	G	0.5	0.5	A
10	BICF2G630483372	33551299	A	0.75	0.7857	G
10	BICF2G630483382	33558165	G	0.25	0.2857	A
10	BICF2G630483413	33577571	A	0	0	G
10	BICF2G630483416	33580749	A	0.5	0.5	G
10	BICF2G630483425	33598752	G	0	0	A
10	BICF2P458513	33609701	G	0.5	0.5	A
10	BICF2P219712	33623489	G	0.5	0.5	A
10	BICF2G630483436	33631802	A	0	0	G
10	BICF2P260189	33638313	G	0.5	0.5	A
10	BICF2P1402360	33650412	A	0	0	G
10	BICF2P444140	33663758	A	0	0	G
10	BICF2P640968	33678806	G	0.5	0.5	A
10	BICF2P1304032	33680432	A	0	0	G
10	TIGRP2P139112_rs8782588	33717317	0	0	0	A
10	BICF2S2354515	33728178	C	0	0	A
10	BICF2G630483452	33732477	G	0.5	0.5	A
10	BICF2G630483458	33746398	G	0	0	A
10	BICF2G630483465	33754327	A	0	0	C

10	BICF2G630483473	33768320	A	0.5	0.5	G
10	BICF2G630483488	33777044	G	0	0	A
10	BICF2P977031	33780809	A	0	0	G
10	BICF2G630483500	33793166	A	0.5	0.5	G
10	BICF2G630483503	33798889	A	0	0	G
10	BICF2G630483513	33811923	C	0.5	0.5	A
10	BICF2G630483519	33822923	G	0.5	0.5	A
10	BICF2G630483529	33836691	A	0.5	0.5	G
10	BICF2G630483538	33848217	A	0	0	G
10	BICF2G630483544	33857332	G	0	0	A
10	BICF2P797002	33858548	A	0	0	T
10	BICF2G630483556	33871772	A	0	0	C
10	BICF2P1075313	33881765	A	0	0	G
10	BICF2P754606	33892205	A	0	0	G
10	BICF2G630483565	33913149	A	0.5	0.5	G
10	BICF2P966926	33914574	0	0	0	A
10	BICF2G630483573	33934660	G	0.5	0.5	A
10	BICF2G630483581	33947018	G	0.5	0.5	A
10	BICF2G630483585	33951415	G	0.5	0.5	A
10	BICF2S23517561	33969545	A	0	0	G
10	BICF2G630483608	33977607	A	0.5	0.5	G
10	BICF2G630483622	33983939	A	0.5	0.5	G
10	BICF2G630483642	33999026	A	0.5	0.5	G
10	BICF2G630483656	34010482	G	0.5	0.5	A
10	BICF2G630483667	34024241	A	0	0	G
10	BICF2G630483685	34036743	A	0.25	0.2143	G
10	BICF2G630483716	34047734	G	0	0	A
10	BICF2P297904	34048614	A	0.25	0.2143	T
10	BICF2P925977	34061886	A	0	0	G
10	BICF2G630483733	34071996	G	0	0	A
10	BICF2P1316652	34076957	G	0	0	A
10	BICF2P729577	34087715	A	0.5	0.5	G
10	BICF2P368223	34098518	G	0.25	0.2143	A
10	BICF2S23513180	34113768	G	0.25	0.25	A
10	BICF2P1222947	34128377	A	0.75	0.7143	C
10	BICF2G630483753	34135370	A	0.75	0.7143	G
10	BICF2G630483760	34161079	G	0	0	A
10	BICF2G630483763	34170229	0	0	0	A
10	BICF2G630483768	34179508	A	0.25	0.2143	G
10	BICF2P857077	34197469	C	0.25	0.2143	A
10	BICF2P481783	34213148	A	0.25	0.2143	G
10	BICF2P253977	34242259	G	0	0	A
10	BICF2S23043263	34256915	A	0	0	G
10	BICF2P244935	34261081	A	0	0	C
10	BICF2G630483809	34280381	A	0.75	0.7857	G
10	BICF2G630483812	34281786	A	0.75	0.7857	G
10	BICF2G630483825	34289308	C	0	0	A

10	BICF2G630483828	34299844	A	0.75	0.7857	G
10	BICF2G630483844	34308385	A	0	0	C
10	BICF2G630483876	34331296	G	0.75	0.7857	A
10	BICF2G630483888	34338472	C	0	0	A
10	BICF2G630483891	34344700	A	0.25	0.2143	G
10	BICF2G630483905	34353223	G	0.5	0.5	A
10	BICF2G630483921	34363842	A	0	0	G
10	BICF2P6692	34380250	G	0.25	0.2143	A
10	BICF2P641734	34391228	G	0.25	0.2857	A
10	BICF2S23414016	34403931	A	0.25	0.2143	G
10	BICF2P1226077	34412330	A	0	0	G
10	BICF2S23351406	34449453	A	0	0	G
10	BICF2P1411955	34465237	A	0.25	0.2143	C
10	BICF2S23111326	34479725	G	0.75	0.7857	A
10	BICF2G630483950	34490429	A	0	0	G
10	BICF2P1415713	34501647	A	0.75	0.7857	G
10	BICF2G630483985	34510032	A	0.75	0.7857	G
10	BICF2G630483993	34514153	A	0.75	0.7857	G
10	BICF2G630484003	34522929	G	0.25	0.2143	A
10	BICF2G630484007	34524769	A	0.75	0.7857	G
10	BICF2G630484041	34540022	C	0	0	A
10	BICF2G630484046	34547281	G	0.25	0.2143	A
10	BICF2G630484059	34560548	G	1	1	A
10	BICF2G630484074	34569271	G	1	1	A
10	BICF2G630484099	34586842	C	1	1	A
10	BICF2G630484104	34595242	A	0	0	C
10	BICF2P1163786	34608495	G	0.25	0.2857	A
10	BICF2G630484132	34616873	A	0.5	0.5	C
10	BICF2G630484139	34626927	C	0.75	0.7857	A
10	BICF2P1295719	34645237	0	0	0	A
10	BICF2P676418	34688775	0	0	0	G
10	BICF2G630484155	34704840	A	0.5	0.5	G
10	BICF2G630484171	34760402	C	0	0	A
10	BICF2G630484212	34794875	A	0.25	0.2857	G
10	BICF2G630484214	34803510	G	0.25	0.25	A
10	BICF2S22944440	34828003	A	0	0	G
10	BICF2P472900	34848953	0	0	0	A
10	BICF2G630484243	34869983	G	0.75	0.7857	A
10	BICF2G630484251	34889887	A	0.75	0.7857	C
10	BICF2G630484252	34898482	G	0.25	0.2143	A
10	BICF2G630484255	34902618	C	0.75	0.7857	A
10	BICF2G630484268	34914088	A	0.75	0.7857	G
10	BICF2G630484270	34928992	G	0.75	0.7857	A
10	BICF2G630484277	34944072	A	0.75	0.7857	G
10	BICF2G630484279	34955000	A	0.75	0.7857	G
10	BICF2G630484290	34968682	A	0.75	0.7857	G
10	BICF2S23044409	34980697	G	0.5	0.4	A

10	BICF2G630484319	34992238	G	0	0	A
10	BICF2G630484335	35004815	A	0.75	0.7143	G
10	BICF2G630484341	35006165	A	0.75	0.7143	G
10	BICF2G630484364	35030555	G	0.75	0.7143	A
10	BICF2G630484383	35044752	A	0.75	0.7143	G
10	BICF2G630484389	35053576	C	0.75	0.7143	A
10	BICF2S23331971	35080572	A	0.25	0.2143	G
10	BICF2G630484432	35099037	0	0	0	A
10	BICF2P816220	35128425	G	0	0	A
10	BICF2P1031524	35153426	C	0	0	A
10	BICF2S2294307	35159595	A	0	0	G
10	BICF2P1224117	35167369	A	0.25	0.2857	G
10	BICF2P850545	35185828	C	0	0	A
10	BICF2P408985	35190000	A	0	0	G
10	BICF2P1001790	35201146	G	0.25	0.2857	A
10	BICF2P357488	35206534	A	0	0	G
10	BICF2P750894	35220509	G	NA	0	C
10	BICF2P750895	35220658	A	0.5	0.5	G
10	BICF2S23340539	35232472	A	0	0	G
10	BICF2P185453	35241357	A	0.5	0.5	C
10	BICF2G630484462	35272428	G	0.25	0.2857	A
10	BICF2G630484469	35278281	A	0	0	G
10	G593f26S145	35299424	G	0	0.25	A
10	BICF2S23737992	35324850	A	0	0	G
10	BICF2S22938822	35340366	G	0.5	0.5	A
10	BICF2P226117	35364798	0	0	0	A
10	BICF2S23029368	35381802	A	0	0	G
10	BICF2S23547957	35414752	A	0	0	G
10	BICF2P1254781	35434916	0	0	0	A
10	BICF2G630484478	35457194	A	0.5	0.5	G
10	BICF2G630484489	35473379	A	0	0	G
10	BICF2G630484492	35476192	G	0.25	0.2143	A
10	BICF2G630484496	35482081	G	0.75	0.7143	A
10	BICF2G630484505	35496374	T	0.75	0.7143	A
10	BICF2G630484516	35504902	A	0	0	G
10	BICF2G630484524	35518182	A	0.25	0.2857	G
10	BICF2G630484546	35540046	G	1	1	C
10	BICF2G630484554	35550159	G	0.5	0.5	A
10	BICF2G630484583	35569350	G	1	1	A
10	BICF2S22957362	35579758	A	0.25	0.2857	G
10	BICF2G630484604	35581061	G	0.5	0.5	A
10	BICF2G630484618	35592796	A	0.25	0.2857	G
10	BICF2G630484630	35607143	G	0	0	A
10	BICF2G630484635	35619078	A	0	0	G
10	BICF2G630484650	35636842	A	1	1	G
10	BICF2S23239859	35642060	A	0	0	G
10	BICF2G630484676	35654092	A	0	0	G

10	BICF2P758541	35667447	0	0	0	A
10	BICF2G630484694	35674195	C	0.75	0.7857	A
10	BICF2G630484713	35685617	G	0.5	0.5	A
10	BICF2G630484733	35696731	A	0.5	0.5	G
10	TIGRP2P139161_rs8677769	35721335	0	0	0	A
10	BICF2G630484771	35739669	A	0.25	0.2857	G
10	BICF2G630484791	35755614	C	0	0	A
10	BICF2G630484800	35765777	G	0.75	0.7143	A
10	BICF2P168087	35769412	C	0	0	G
10	BICF2S23610938	35769490	A	0	0	G
10	BICF2P983899	35778878	G	0.75	0.7143	A
10	BICF2G630484842	35791106	G	0	0	A
10	BICF2G630484859	35799855	A	0	0	G
10	BICF2P172107	35821405	A	0	0	G
10	BICF2G630484882	35827747	A	0	0	G
10	BICF2P641309	35835352	G	0.5	0.5	A
10	BICF2P1068469	35841236	G	0	0	A
10	BICF2G630484888	35846621	A	0	0	G
10	BICF2P947256	35862062	G	1	1	A
10	BICF2P1272878	35867390	G	0.5	0.5	A
10	BICF2S23223668	35869398	A	0.5	0.5	G
10	BICF2P958865	35880528	0	0	0	A
10	BICF2P1308431	35899962	G	0.5	0.5	A
10	BICF2G630484921	35904855	A	1	1	G
10	BICF2G630484943	35914869	G	1	1	A
10	BICF2G630484957	35933728	A	0	0	G
10	BICF2G630484960	35938737	A	0.25	0.2143	G
10	BICF2G630484973	35950340	G	0.25	0.2143	A
10	BICF2G630484988	35972484	0	0	0	G
10	BICF2P633275	35976301	A	0.25	0.25	G
10	BICF2G630484989	35983885	A	0	0	G
10	BICF2P1001580	35996540	G	0.25	0.2857	A
10	BICF2G630484994	36010673	A	0.75	0.7143	G
10	BICF2G630485019	36029330	A	0	0	C
10	BICF2G630485020	36029382	A	0	0	G
10	BICF2G630485074	36048450	G	0	0	A
10	BICF2P390372	36050874	A	0	0	C
10	BICF2G630485082	36054599	G	1	1	A
10	BICF2P1029702	36065030	C	0.75	0.8333	A
10	BICF2P468087	36085590	G	1	1	A
10	BICF2P534526	36094752	A	0	0	G
10	BICF2P385231	36103342	G	0	0	A
10	BICF2P613676	36117781	C	0.75	0.7857	A
10	BICF2P613678	36117960	G	0	0	A
10	BICF2P146712	36123523	C	0.25	0.2143	A
10	BICF2G630485114	36133793	A	0	0	G
10	BICF2G630485132	36151785	A	0.75	0.7857	G

10	BICF2G630485158	36163718	G	0.25	0.2857	A
10	BICF2G630485166	36171602	A	0.5	0.5	G
10	BICF2G630485187	36187500	A	0.25	0.2857	C
10	BICF2S2374903	36191239	0	0	0	G
10	BICF2P765696	36212359	G	0	0	A
10	BICF2G630485198	36227153	A	0.25	0.2143	G
10	BICF2P1139618	36236963	A	0.5	0.5	C
10	BICF2G630485212	36254448	G	0.25	0.2857	C
10	BICF2P464497	36264085	G	0	0	A
10	BICF2G630485221	36272016	A	0	0	G
10	BICF2S23421042	36285230	A	0.25	0.2143	T
10	BICF2P72997	36303081	A	0	0	G
10	BICF2P315835	36309755	A	0	0	G
10	BICF2G630485250	36318564	A	0	0	G
10	BICF2S23729041	36330798	A	0	0	G
10	BICF2S23644288	36338091	0	0	0	A
10	BICF2S23019562	36348924	G	0.5	0.5	A
10	BICF2G630485264	36358268	G	0.5	0.5	A
10	BICF2G630485272	36370981	A	0.25	0.2857	T
10	BICF2G630485275	36385873	G	0	0.2857	A
10	BICF2G630485281	36394293	G	0	0	A
10	BICF2G630485296	36415357	A	0.5	0.5	G
10	BICF2G630485302	36426227	G	0.25	0.2143	A
10	BICF2G630485313	36435273	C	0.25	0.2143	G
10	BICF2G630485317	36455053	A	0.25	0.2857	G
10	BICF2S2446212	36496240	A	0.25	0.2143	G
10	BICF2G630485322	36501357	A	0	0	C
10	BICF2G630485339	36512740	G	0.75	0.7143	A
10	BICF2G630485358	36539165	A	0.25	0.2857	G
10	BICF2P69729	36548655	G	1	1	A
10	BICF2P901724	36562792	G	1	1	A
10	BICF2G630485397	36571549	T	0	0	A
10	BICF2G630485404	36583771	A	0	0	G
10	BICF2G630485414	36595189	A	0.75	0.7143	G
10	BICF2G630485432	36604679	A	0.75	0.7143	G
10	BICF2G630485439	36618801	A	0.25	0.2857	G
10	BICF2G630485455	36630724	A	0.75	0.7143	G
10	BICF2G630485476	36635539	T	0.75	0.7143	A
10	BICF2G630485495	36640641	A	0.75	0.7143	G
10	BICF2S23249420	36652031	G	0	0	A
10	BICF2P225158	36664946	A	0	0	G
10	BICF2P1050395	36676410	C	0	0	G
10	BICF2P1050396	36676868	A	0	0	G
10	BICF2P313700	36690495	A	0	0	G
10	BICF2G630485528	36697246	G	1	1	A
10	BICF2G630485534	36707610	G	0	0	C
10	BICF2G630485535	36708257	A	0	0	G

10	BICF2G630485552	36722348	G	0.25	0.2857	A
10	BICF2G630485557	36739145	G	0.75	0.7143	A
10	BICF2G630485558	36746896	T	0	0	A
10	BICF2G630485572	36756464	G	1	1	A
10	BICF2P515967	36773989	A	1	1	G
10	BICF2G630485595	36787230	A	0	0	G
10	BICF2P884049	36790501	G	1	1	A
10	TIGRP2P139177_rs8843170	36802957	0	0	0	G
10	BICF2G630485620	36813234	A	0	0	G
10	BICF2G630485638	36825374	A	0	0	G
10	BICF2G630485650	36840456	G	0	0	A
10	BICF2G630485655	36850489	G	1	1	A
10	BICF2G630485667	36864810	G	1	1	A
10	BICF2G630485675	36870957	C	0	0	A
10	BICF2S2313581	36884042	A	0	0	G
10	BICF2G630485706	36895004	G	0	0	A
10	BICF2G630485762	36911677	A	1	1	G
10	BICF2G630485768	36913486	A	1	1	G
10	BICF2G630485773	36918678	A	1	1	G
10	BICF2P1384152	36928192	A	1	1	C
10	BICF2P1384151	36929023	A	1	1	G
10	BICF2S23258698	36939685	C	1	1	G
10	BICF2P768416	36964111	A	1	1	C
10	BICF2P1075514	36993575	A	0.75	0.7143	G
10	BICF2S2329886	37006910	T	1	1	A
10	BICF2S23146524	37009227	C	0	0	A
10	BICF2P959863	37020464	A	0.75	0.7143	G
10	BICF2P1372903	37027295	A	0.75	0.7143	C
10	BICF2P897774	37040713	G	0.75	0.7143	A
10	TIGRP2P139182_rs8992083	37052947	0	0	0	C
10	BICF2P606577	37053018	G	0.75	0.7143	A
10	BICF2P919741	37071036	G	0.75	0.7143	A
10	BICF2P64311	37084539	A	1	1	G
10	BICF2P1150171	37094655	G	0.25	0.2857	A
10	BICF2P1214424	37107029	A	0.25	0.2857	G
10	BICF2P1162810	37110847	C	0.25	0.2857	G
10	BICF2P42717	37131708	0	0	0	A
10	BICF2S2343707	37143412	C	0.25	0.2857	A
10	G594f11S246	37149625	G	0	0	A
10	G594f17S29	37179771	G	0.25	0.2857	C
10	TIGRP2P139191_rs8568966	37205872	0	0	0	G
10	BICF2P286426	37223324	G	0.25	0.2857	A
10	BICF2P382404	37225797	A	0.75	0.7143	G
10	BICF2S23246356	37250415	C	0	0	A
10	BICF2P763246	37283540	C	0	0	A
10	BICF2P1465180	37299733	A	0	0	C
10	BICF2P1440689	37324816	G	0.25	0.2857	A

10	BICF2P1024485	37336606	G	1	1	A
10	BICF2P425578	37345910	0	0	0	G
10	BICF2P219667	37352459	G	1	1	A
10	BICF2P468594	37368056	A	0	0	G
10	TIGRP2P139195_rs8828572	37377429	0	0	0	G
10	BICF2P239529	37389769	0	0	0	A
10	BICF2P736516	37399207	0	0	0	G
10	BICF2S23148212	37412358	G	0.75	0.7857	A
10	BICF2P1406287	37422188	G	0.75	0.7857	A
10	BICF2P771630	37435268	0	0	0	G
10	G595f15S150	37459379	A	0	0	G
10	BICF2P1278074	37477990	T	0.75	0.7857	A
10	BICF2P520325	37481223	A	0.25	0.2857	G
10	BICF2P1391279	37492411	A	0	0	G
10	BICF2P1404951	37505647	A	0.75	0.7857	G
10	BICF2P405508	37517209	G	1	1	A
10	BICF2S22936758	37554220	A	0.5	0.5	G
10	BICF2P242040	37559961	G	0	0	A
10	BICF2S2367757	37587626	C	0	0	A
10	BICF2S23713782	37594337	C	0	0	G
10	BICF2S23060073	37605651	A	0	0	G
10	BICF2P254600	37620538	A	0	0	G
10	TIGRP2P139200_rs8818017	37630079	0	0	0	A
10	BICF2S23022363	37647571	G	0	0	A
10	BICF2S23619213	37652895	G	0	0	C
10	BICF2S23410471	37667988	A	0.25	0.2143	G
10	BICF2S23439635	37675509	A	0.25	0.2143	G
10	BICF2P159936	37682574	A	0	0	G
10	BICF2P164006	37687688	A	0	0	G
10	BICF2P1378112	37712100	G	0	0	A
10	BICF2P979124	37716238	G	1	1	C
10	BICF2P629340	37717246	A	0	0	G
10	BICF2S23213996	37730090	T	1	1	A
10	BICF2S23124381	37755253	G	0.5	0.5	A
10	BICF2S23319672	37776039	C	0.5	0.5	A
10	BICF2P1238518	37778220	A	0	0	T
10	BICF2P1374437	37786454	A	0	0	G
10	BICF2P541826	37791609	A	0	0	G
10	BICF2S23714804	37801706	T	1	1	A
10	BICF2P795958	37809914	T	0	0	A
10	BICF2P711598	37818834	G	0.25	0.2857	A
10	BICF2S22919745	37823788	A	0.25	0.2857	C
10	BICF2P396998	37835322	A	0	0	T
10	BICF2P721675	37846943	G	0.75	1	A
10	BICF2P1314217	37864196	A	0.5	0.5	G
10	BICF2P478892	37877299	A	0.5	0.5	G
10	BICF2P119336	37883448	G	0.75	0.7857	A

10	BICF2P771063	37911827	A	0	0	C
10	BICF2P1044300	37926345	G	0.75	0.7143	A
10	BICF2P282943	37928801	T	0.75	0.7143	A
10	TIGRP2P139206_rs9105364	37942810	0	0	0	A
10	BICF2S23634322	37953471	G	0.25	0.2143	A
10	BICF2P1231523	37963579	0	0	0	G
10	BICF2P290500	37982890	0	0	0	G
10	BICF2P461544	37989904	G	0	0	A
10	BICF2P656484	37996908	A	0.5	0.5	G
10	BICF2P868755	38017794	G	0	0	A
10	BICF2P632945	38025066	A	0	0	T
10	BICF2S23256222	38035059	A	0	0	G
10	BICF2P497921	38049725	A	0	0	G
10	BICF2P777345	38065028	A	0	0	G
10	BICF2G630485797	38087206	G	0	0	A
10	BICF2G630485826	38102328	A	0	0	G
10	BICF2P271475	38113644	0	0	0	G
10	BICF2G630485847	38122987	G	0	0	A
10	BICF2P735686	38146776	0	0	0	G
10	BICF2G630485873	38163826	G	0	0	A
10	BICF2G630485878	38175728	G	0	0	A
10	BICF2P1118910	38190033	0	0	0	G
10	TIGRP2P139214_rs9115464	38199920	0	0	0	A
10	BICF2P1194143	38214637	A	0	0	C
10	BICF2G630485921	38233148	0	0	0	G
10	BICF2P1448673	38238248	0	0	0	G
10	BICF2P317616	38250495	C	0	0	A
10	BICF2P944326	38272861	0	0	0	G
10	BICF2P1146446	38284969	A	0	0	C
10	BICF2P485230	38304191	C	0.25	0.2143	G
10	BICF2P516380	38319800	0	0	0	A
10	BICF2S23431289	38333156	A	0.5	0.5	G
10	TIGRP2P139216_rs8542476	38345804	0	0	0	C
10	BICF2S23226305	38356815	A	0	0	G
10	BICF2G630485926	38368136	0	0	0	G
10	BICF2G630485966	38387719	0	0	0	G
10	BICF2G630485982	38393602	C	0.5	0.5	G
10	BICF2S22932100	38399056	A	0.5	0.5	C
10	BICF2G630486003	38419922	G	0	0	A
10	BICF2G630486011	38424180	C	0	0	A
10	BICF2G630486023	38439553	A	0	0	G
10	BICF2G630486028	38449331	0	0	0	G
10	BICF2G630486037	38458112	A	0	0	G
10	BICF2G630486059	38474301	A	0	0.25	G
10	BICF2G630486061	38487153	A	0.25	0.2857	G
10	BICF2G630486075	38497735	A	1	1	G
10	BICF2G630486084	38505347	A	1	1	G

10	BICF2G630486096	38514454	A	1	1	G
10	BICF2G630486124	38534770	0	0	0	G
10	BICF2G630486134	38543105	G	1	1	A
10	BICF2G630486143	38551602	A	1	1	G
10	BICF2P775928	38580411	A	0.25	0.2857	G
10	BICF2P1340850	38602054	A	0.75	0.7143	G
10	BICF2S2318225	38607019	A	0	0	G
10	BICF2S23152609	38621402	A	0	0	G
10	BICF2P139769	38639092	A	0.75	0.7143	C
10	BICF2P1332866	38646573	A	1	0.7143	C
10	BICF2P1058648	38660698	A	0.75	0.7143	G
10	BICF2G630486196	38686708	G	0	0	A
10	BICF2G630486208	38698465	G	0.5	0.5	A
10	BICF2S23010067	38730284	0	0	0	G
10	BICF2G630486231	38750810	G	0.75	0.7143	A
10	BICF2P100100	38758578	0	0	0	A
10	BICF2P1180602	38767211	0	0	0	G
10	BICF2S2307147	38786406	0	0	0	A
10	BICF2P1178659	38798721	A	0.5	0.5	G
10	TIGRP2P139242_rs8652605	38838964	0	0	0	G
10	TIGRP2P139247_rs8901726	38864204	0	0	0	C
10	BICF2P1150929	38887016	G	0.75	0.7857	A
10	BICF2P1151282	38888423	A	0.75	0.7857	G
10	BICF2P934261	38893195	C	0.75	0.7857	G
10	BICF2P201153	38912725	T	0.75	0.7857	A
10	BICF2P1030862	38926325	G	0.75	0.7857	A
10	BICF2P276528	38932670	A	0.5	0.5	G
10	BICF2P1423566	38940452	A	0.75	0.7857	C
10	BICF2S23037662	38974517	0	0	0	G
10	BICF2P1015233	39004837	G	0.5	0.5	A
10	TIGRP2P139250_rs8573464	39015928	0	0	0	A
10	BICF2P5886	39020983	A	0	0	G
10	BICF2P998895	39034021	G	1	1	A
10	BICF2S23035215	39049802	G	0.5	0.5	A
10	BICF2P679919	39054709	G	1	1	A
10	BICF2P452541	39066689	A	0	0.2143	G
10	BICF2P1165311	39080280	A	0	0	G
10	BICF2P806038	39092265	0	0	0	A
10	BICF2P879518	39109056	A	0	0	T
10	BICF2P646200	39111891	0	0	0	A
10	BICF2S2379147	39139431	A	0.25	0.2143	G
10	BICF2P132436	39156349	A	0.75	0.7143	G
10	BICF2P242655	39158620	A	0.5	0.4286	G
10	BICF2P203790	39170781	G	0.75	0.7143	A
10	TIGRP2P139254_rs8621736	39189073	0	0	0	A
10	BICF2S23758989	39202650	A	0	0	G
10	BICF2P675419	39209627	0	0	0	G

10	BICF2S23327479	39211422	A	0	0	G
10	BICF2P762723	39215389	A	0.25	0.2143	G
10	BICF2P586564	39233694	G	0.75	0.7143	A
10	BICF2P1410286	39240626	A	0.75	0.7143	G
10	BICF2S23244019	39250606	0	0	0	G
10	BICF2S2353527	39264825	G	0	0	A
10	BICF2S23547220	39275393	C	0.75	0.7857	A
10	BICF2P1230023	39297941	0	0	0	G
10	BICF2P112768	39307473	G	0.5	0.5	A
10	TIGRP2P139257_rs8958236	39321634	0	0	0	A
10	TIGRP2P139258_rs8644196	39333127	0	0	0	G
10	G597f45S25	39357064	0	0	0	C
10	BICF2S23531659	39384638	A	0	0	G
10	BICF2P697825	39402706	G	0.5	0.5	A
10	BICF2P240158	39414760	0	0	0	A
10	BICF2P1092538	39446216	G	0.5	0.5	A
10	BICF2P1318151	39464685	G	0.5	0.5	A
10	BICF2P971898	39472600	A	0.5	0.5	G
10	BICF2P762453	39479839	A	0.5	0.5	G
10	BICF2P482825	39499305	A	0.75	0.7857	G
10	BICF2P1418691	39503251	G	0.5	0.5	A
10	BICF2P474746	39516025	A	0.5	0.5	G
10	BICF2P1211090	39535053	G	0.5	0.5	A
10	BICF2P1192904	39538389	C	0	0	G
10	BICF2P1333410	39539071	G	0.5	0.5	A
10	BICF2S23331156	39561079	C	0.75	0.7143	A
10	BICF2S23331155	39561108	A	0.75	0.7143	C
10	BICF2P541381	39564789	G	0.75	0.7857	A
10	BICF2P608090	39621228	A	0.25	0.2143	C
10	BICF2P438721	39627024	A	0	0	G
10	BICF2S23532407	39631709	G	0.25	0.2143	A
10	G598f14S99	39659863	A	0.75	0.7857	G
10	BICF2S23913669	39664974	T	0.75	0.7857	A
10	BICF2P1332823	39685477	0	0	0	C
10	BICF2P584384	39697379	A	0	0	C
10	BICF2P584383	39698732	0	0	0	A
10	BICF2S24415854	39756079	A	0.75	0.8333	G
10	BICF2S23612256	39768855	G	0.75	0.7857	A
10	BICF2P1295510	39779228	0	0	0	A
10	BICF2P1163832	39791660	G	0.75	0.7857	A
10	BICF2S23144149	39814392	A	0	0	G
10	BICF2P1400778	39826155	A	0	0	C
10	BICF2S2308374	39836839	A	0.5	0.5	G
10	BICF2P171223	39850356	A	0.25	0.2857	G
10	BICF2S23720259	39861289	T	0	0	A
10	BICF2P1123078	39871680	G	0.5	0.5	A
10	BICF2P493654	39886776	G	0.5	0.5	A

10	BICF2P1165238	39894054	G	0.5	0.5	A
10	BICF2S23636873	39926531	0	0	0	G
10	BICF2P493862	39928680	G	0.5	0.5	A
10	BICF2P155531	39940255	G	0.5	0.5	A
10	BICF2P115855	39959669	A	0.5	0.5	G
10	BICF2S23516482	39964845	A	0.5	0.5	G
10	BICF2P1001877	39980538	A	0.5	0.5	G
10	BICF2P1391629	39987136	G	0.5	0.5	A
10	BICF2P651358	40028984	G	0.5	0.5	A
10	BICF2P67903	40036990	C	0	0	A
10	BICF2S23646068	40054208	C	0.75	0.7143	A
10	BICF2S235950	40055403	G	0.75	0.7143	A
10	BICF2S23314483	40069982	A	0.5	0.5	G
10	BICF2S23532899	40096229	G	0.25	0.2143	A
10	BICF2S23034702	40107135	A	0	0	G
10	BICF2P1309037	40120294	0	0	0	G
10	BICF2P877803	40127484	A	0.25	0.2857	G
10	BICF2P977782	40135983	C	0	0	A
10	BICF2P953599	40148271	G	0	0	A
10	BICF2P158638	40173832	G	0.25	0.2143	A
10	BICF2P1144761	40182227	A	0	0	G
10	BICF2P1081558	40196030	A	0	0	G
10	BICF2S23637559	40205095	A	0.75	0.7143	G
10	BICF2S23021735	40226244	G	0	0	A
10	BICF2P53099	40233562	A	0.5	0.5	G
10	BICF2P648792	40241992	G	0	0	A
10	BICF2P316980	40250825	G	0	0	A
10	BICF2P949491	40252453	A	0.25	0.1667	C
10	BICF2P455217	40261786	A	0	0	G
10	BICF2P753822	40284316	A	1	1	G
10	BICF2S2376126	40293391	G	0.25	0.2143	A
10	BICF2P277947	40300716	G	0.75	0.7857	A
10	BICF2P399349	40317495	A	0	0	G
10	BICF2P1196416	40330047	C	0	0	A
10	BICF2P539231	40332760	A	0	0	G
10	BICF2P334890	40348826	G	0	0	A
10	BICF2P490200	40362602	G	0	0	A
10	BICF2P1033785	40378190	G	0	0	A
10	BICF2S234558	40381822	C	0	0	A
10	BICF2P411307	40391968	G	0	0	A
10	BICF2S23520134	40405692	A	0.5	0.5	G
10	BICF2S23416045	40418321	A	0	0	G
10	BICF2P553041	40431178	G	0.25	0.2857	A
10	BICF2P755454	40443972	G	1	1	A
10	BICF2S23231161	40448172	G	1	1	C
10	BICF2P1085572	40462235	A	1	1	G
10	BICF2P1234996	40469119	0	0	0	A

10	BICF2P335737	40482362	A	0	0	C
10	BICF2P288281	40499654	G	0	0	A
10	BICF2P124797	40503251	G	0	0	A
10	BICF2P1122712	40522994	A	0	0	G
10	BICF2P749529	40526205	G	0.25	0.2857	A
10	BICF2P994038	40528000	G	0.25	0.2857	C
10	BICF2P809771	40548689	G	0.5	0.5	A
10	BICF2P284753	40553335	A	0	0	C
10	BICF2S23239448	40570875	C	0.25	0.2857	A
10	BICF2P657631	40578559	A	0	0	G
10	BICF2P528896	40589268	G	0.25	0.2143	A
10	BICF2P439279	40615900	G	0	0	A
10	BICF2P1336929	40624271	C	0	0	A
10	BICF2P981498	40643088	A	0.5	0.5	G
10	BICF2P680482	40663239	C	0	0	G
10	BICF2P44428	40668862	G	0	0	A
10	BICF2P721822	40678528	C	0.5	0.5	G
10	BICF2P747092	40693573	G	0.5	0.5	A
10	BICF2G630486234	40709654	A	0.5	0.5	G
10	BICF2G630486243	40719396	A	0.5	0.5	G
10	BICF2P785828	40721965	G	0.5	0.5	A
10	BICF2P1251880	40746976	G	0.5	0.5	A
10	BICF2P976837	40764963	A	0.5	0.5	G
10	BICF2G630486258	40769299	A	0.5	0.5	G
10	BICF2G630486281	40783687	A	0.5	0.5	G
10	BICF2G630486311	40795060	C	0.5	0.5	A
10	BICF2G630486323	40801773	A	0.5	0.5	C
10	BICF2G630486325	40803942	A	0.75	0.7857	G
10	BICF2G630486328	40807823	G	0.25	0.2143	A
10	BICF2G630486332	40823185	G	0.25	0.2857	A
10	BICF2G630486335	40834409	A	0	0	G
10	BICF2G630486337	40837645	A	0	0	G
10	BICF2G630486360	40858466	G	0.25	0.2857	A
10	BICF2G630486386	40870911	C	0.25	0.2857	A
10	BICF2G630486388	40872298	C	0	0	A
10	BICF2G630486407	40884706	A	0	0	G
10	BICF2G630486415	40897092	A	0	0	G
10	BICF2G630486457	40912675	G	0.25	0.2143	A
10	BICF2P1242755	40918256	C	0.75	0.7857	A
10	BICF2P266904	40922793	A	0.5	0.5	G
10	BICF2G630486468	40938268	0	0	0	G
10	BICF2P1250824	40942742	G	0.75	0.7857	A
10	BICF2P1206027	40951573	A	0.75	0.7857	G
10	BICF2P1017262	40963594	A	0.75	0.7857	G
10	BICF2G630486514	40983876	G	0.5	0.5	A
10	BICF2G630486518	40996630	C	0.5	0.5	A
10	BICF2G630486519	41004933	0	0	0	G

10	BICF2G630486537	41010330	A	0	0	G
10	BICF2P534789	41020537	0	0	0	A
10	BICF2G630486552	41050419	A	0.25	0.25	G
10	BICF2G630486584	41069760	G	0.25	0.2857	A
10	BICF2G630486595	41078220	A	0.25	0.2857	G
10	BICF2G630486613	41091174	C	0.5	0.5	A
10	BICF2G630486618	41102099	A	0.5	0.5	G
10	BICF2G630486642	41118734	G	0.5	0.5	A
10	BICF2G630486664	41134702	G	0.25	0.2	A
10	BICF2P278325	41135571	A	0.25	0.2857	C
10	BICF2S2327113	41148764	G	0.25	0.2143	A
10	BICF2G630486685	41169424	A	0.75	0.7143	G
10	BICF2P1196668	41175122	A	0	0	C
10	BICF2G630486726	41189782	G	0	0	A
10	BICF2G630486752	41194625	A	0	0	G
10	BICF2G630486786	41205411	A	0.25	0.2143	G
10	BICF2G630486807	41217714	G	0	0.2143	A
10	BICF2P759245	41235650	G	0.75	0.7857	A
10	BICF2G630486827	41241214	G	0.75	0.7857	A
10	BICF2P315296	41262433	0	0	0	A
10	BICF2G630486842	41274006	G	0.75	0.7143	A
10	BICF2G630486859	41287603	A	0.75	0.7143	G
10	BICF2G630486902	41304517	A	1	1	G
10	BICF2G630486931	41314853	G	0	0	A
10	BICF2S23150428	41315878	A	0	0	G
10	BICF2G630486932	41316282	C	0	0	G
10	TIGRP2P139292_rs8645545	41320981	0	0	0	A
10	BICF2S23036712	41334486	C	0.25	0.2857	A
10	BICF2G630486981	41349536	A	0	0	G
10	BICF2G630486989	41355323	0	0	0	G
10	BICF2S2452880	41368598	A	0	0	G
10	BICF2G630487001	41382537	G	0.25	0.2143	A
10	BICF2G630487027	41391590	A	0.25	0.2143	C
10	BICF2S23534231	41403191	G	0.25	0.2143	A
10	BICF2G630487060	41413756	A	0	0	G
10	BICF2G630487086	41423627	G	0.5	0.5	A
10	BICF2P1056358	41445578	G	0	0	A
10	BICF2G630487110	41448783	G	0.25	0.2143	C
10	BICF2G630487114	41458367	A	0.25	0.2143	G
10	BICF2G630487131	41479701	T	0.25	0.2143	A
10	BICF2P350599	41488778	A	0	0	G
10	BICF2G630487152	41498001	A	0.5	0.5	G
10	BICF2G630487168	41509962	C	0.25	0.2143	A
10	BICF2G630487186	41524201	G	0.5	0.5	A
10	BICF2S2313417	41534976	A	0	0	C
10	BICF2S23445002	41554369	G	1	1	A
10	BICF2G630487209	41563084	C	0.25	0.2143	A

10	BICF2G630487234	41574056	A	0.5	0.5	G
10	BICF2G630487265	41585138	G	0.25	0.2143	A
10	BICF2G630487276	41595813	C	0.5	0.5	A
10	BICF2G630487303	41612062	G	0.25	0.2857	A
10	BICF2G630487308	41616437	A	0	0	G
10	BICF2S23640227	41622898	C	0.5	0.5	A
10	BICF2P1038271	41632884	A	0.5	0.5	G
10	BICF2S23630352	41646068	G	0	0	A
10	BICF2G630487316	41655003	G	0.25	0.2143	A
10	BICF2G630487329	41672851	G	0.25	0.2143	A
10	BICF2G630487354	41700858	G	0.5	0.5	A
10	BICF2G630487365	41710557	A	0.25	0.2143	G
10	BICF2S23537585	41723154	A	0	0	G
10	BICF2G630487409	41739604	G	0	0	A
10	BICF2G630487416	41743616	A	1	1	G
10	BICF2G630487418	41748482	A	0.75	0.7857	G
10	BICF2G630487423	41760905	G	0.75	0.7857	A
10	BICF2G630487458	41771467	A	0.5	0.5	G
10	BICF2S23710954	41781328	A	0.5	0.5	G
10	BICF2G630487463	41794835	G	0.75	0.7143	A
10	BICF2G630487492	41813783	G	1	0.7857	A
10	BICF2G630487514	41827080	A	0	0	G
10	BICF2G630487523	41843692	A	0	0	G
10	BICF2G630487543	41858027	A	1	1	G
10	BICF2P951190	41870230	G	1	1	A
10	BICF2P770348	41876193	G	0	0	A
10	BICF2S23713363	41889811	A	0	0	G
10	BICF2G630487551	41902162	A	1	1	G
10	BICF2G630487560	41906821	A	1	1	G
10	BICF2G630487609	41920424	A	0	0	G
10	BICF2G630487626	41935370	A	0	0	G
10	BICF2G630487650	41945115	A	0	0	C
10	BICF2G630487656	41956497	A	0	0	G
10	BICF2G630487668	41972539	A	0	0	G
10	BICF2G630487671	41977602	A	0	0	G
10	BICF2G630487684	41987215	0	0	0	G
10	BICF2G630487686	42004838	G	0	0	A
10	BICF2G630487692	42012571	G	0	0	A
10	BICF2P71818	42032076	G	0	0	A
10	BICF2G630487706	42034048	A	0	0	G
10	BICF2G630487719	42046052	A	0	0	G
10	BICF2G630487725	42060335	A	0	0	G
10	BICF2G630487753	42075134	G	0.75	0.7857	A
10	BICF2G630487760	42080058	C	0	0	A
10	BICF2P1313444	42095524	A	0.25	0.2143	G
10	BICF2G630487802	42095657	G	0	0	A
10	BICF2G630487816	42103909	A	0	0	G

10	BICF2G630487819	42104976	C	0.75	0.8333	A
10	BICF2P556763	42121675	A	0.5	0.5	G
10	BICF2G630487838	42128012	C	0.25	0.2143	A
10	BICF2G630487870	42138655	G	0	0	A
10	BICF2G630487873	42144645	A	0	0	G
10	BICF2G630487881	42147529	C	0	0	A
10	BICF2G630487914	42162747	C	0	0	A
10	BICF2P290114	42176459	G	1	1	A
10	BICF2G630487951	42187969	A	0	0	T
10	BICF2P97279	42193068	C	0	0	A
10	BICF2G630487956	42193330	A	0	0	G
10	BICF2S23762350	42194691	G	0	0	A
10	BICF2G630487971	42207366	A	0.75	0.7857	G
10	BICF2P980691	42217595	G	0	0	A
10	BICF2S23251491	42230146	A	0	0	C
10	BICF2G630487984	42257296	A	0.25	0.2857	G
10	BICF2G630488002	42269506	G	0	0	A
10	BICF2G630488020	42275984	A	0	0	G
10	BICF2P1381759	42288759	G	0.25	0.2857	A
10	BICF2G630488053	42307807	0	0	0	A
10	BICF2G630488063	42311266	0	0	0	A
10	BICF2G630488077	42327531	G	0	0	A
10	BICF2G630488100	42340854	A	0	0	G
10	BICF2S23317551	42343179	G	0	0	A
10	BICF2P1201124	42360745	G	0	0	A
10	BICF2P657241	42366072	A	0.75	0.7857	G
10	BICF2P501801	42377786	A	1	1	C
10	BICF2P696493	42399133	G	0.5	0.5	A
10	BICF2G630488122	42405207	A	0	0	G
10	BICF2P534596	42419656	G	0	0	A
10	BICF2P207352	42424398	G	0	0	A
10	BICF2G630488130	42445958	G	0	0	A
10	BICF2G630488132	42448422	G	0	0	A
10	BICF2S23032313	42458010	C	0	0	A
10	BICF2G630488149	42477947	G	0.5	0.5	A
10	BICF2G630488155	42483781	G	0.75	0.7143	A
10	BICF2G630488168	42499215	A	0	0	G
10	BICF2G630488177	42504590	G	0	0	A
10	BICF2G630488189	42516459	G	0	0	A
10	BICF2P256103	42535916	G	0.5	0.5	A
10	BICF2P1039179	42538568	A	0.5	0.5	C
10	BICF2P1046961	42574502	A	0.25	0.2857	T
10	BICF2P465094	42585302	A	0.25	0.2857	G
10	BICF2G630488196	42597561	G	0.25	0.2143	A
10	BICF2P1139678	42608803	A	0.5	0.5	G
10	BICF2G630488207	42619343	A	0.25	0.2857	G
10	BICF2G630488228	42633438	G	0	0	A

10	BICF2G630488242	42642755	G	0.5	0.5	A
10	BICF2G630488256	42660173	A	0	0	G
10	BICF2G630488267	42670586	G	1	1	A
10	BICF2G630488287	42688707	G	1	1	A
10	BICF2G630488316	42701533	A	1	1	T
10	BICF2G630488324	42719319	A	0	0	C
10	BICF2P26604	42724040	G	0.75	0.7143	C
10	BICF2S23656498	42743628	A	0	0	G
10	TIGRP2P139348_rs8838088	42745856	0	0	0	A
10	BICF2S23344938	42762105	G	0.25	0.2857	A
10	BICF2P1071570	42768655	0	0	0	A
10	BICF2P1437716	42786589	C	0.5	0.5	G
10	BICF2G630488333	42800192	G	0	0	A
10	BICF2G630488363	42809479	G	0.5	0.5	A
10	BICF2G630488396	42820509	A	0.5	0.5	T
10	BICF2G630488427	42842254	A	0.25	0.2857	G
10	BICF2P487007	42855331	A	0.5	0.5	G
10	BICF2S23154094	42861305	G	0.5	0.5	A
10	BICF2G630488445	42875580	A	0.25	0.2857	G
10	BICF2G630488488	42892313	A	0.25	0.2857	G
10	TIGRP2P139406_rs8837845	42901314	A	0.25	0.2857	G
10	BICF2G630488498	42914491	A	0	0	G
10	BICF2G630488547	42936059	A	0.25	0.2143	G
10	BICF2G630488558	42946371	C	0.5	0.5	A
10	BICF2G630488572	42960780	A	0.25	0.2143	G
10	BICF2G630488585	42973724	0	0	0	G
10	BICF2G630488587	42976090	G	0.25	0.2143	A
10	BICF2G630488596	42992307	A	0	0	G
10	BICF2G630488599	43001543	A	0.25	0.2143	C
10	BICF2G630488610	43010007	A	0	0	G
10	BICF2G630488624	43026483	A	0.75	0.7143	G
10	TIGRP2P139421_rs8765949	43033813	G	0.75	0.7143	A
10	BICF2G630488651	43058076	G	0.75	0.7143	A
10	BICF2P1464923	43072409	A	0.25	0.2857	G
10	BICF2G630488675	43081325	A	0	0	G
10	BICF2G630488685	43086455	A	0	0	G
10	BICF2G630488699	43101695	A	0	0	C
10	BICF2S23125191	43111855	A	0.75	0.7143	G
10	TIGRP2P139438_rs8700438	43115081	A	0.75	0.7143	G
10	BICF2P838661	43131052	A	0.5	0.5	G
10	BICF2P355650	43139339	A	0.5	0.5	C
10	BICF2G630488721	43164610	G	0.25	0.2857	A
10	BICF2P1063186	43171556	A	0.5	0.5	T
10	BICF2S23142951	43186188	A	0.5	0.5	G
10	TIGRP2P139476_rs8608933	43197179	G	0.5	0.5	A
10	BICF2G630488729	43213813	C	0.5	0.5	G
10	BICF2G630488736	43217122	A	0	0	G

10	BICF2G630488751	43236140	G	0.5	0.5	A
10	BICF2P621985	43250702	A	0.5	0.5	G
10	BICF2G630488756	43251656	A	0.75	0.7143	G
10	BICF2G630488773	43264862	G	0	0	A
10	BICF2G630488774	43264881	G	0	0	A
10	BICF2P578227	43275298	G	0	0	A
10	BICF2P1380227	43283630	A	0.5	0.5	G
10	BICF2P811103	43292033	A	0	0	G
10	BICF2G630488815	43304460	A	0.75	0.7143	G
10	TIGRP2P139516_rs8946217	43309606	A	0.25	0.2143	G
10	BICF2G630488880	43329282	G	0	0	A
10	BICF2S2306463	43332534	A	0	0	G
10	BICF2G630488907	43351052	G	0	0	A
10	BICF2G630488915	43355087	G	0	0	A
10	BICF2G630488926	43370347	A	0.75	0.7857	G
10	BICF2P513762	43376946	A	0.25	0.2143	C
10	BICF2G630488941	43378636	G	0	0	A
10	BICF2G630488966	43397991	G	0	0	A
10	BICF2P640829	43406486	G	0.25	0.2143	A
10	TIGRP2P139536_rs8906929	43413233	A	0.25	0.2143	C
10	BICF2P1436499	43440766	G	0.25	0.2143	A
10	BICF2P606850	43447427	G	0.25	0.1667	A
10	BICF2G630489020	43464010	A	0	0	G
10	BICF2P1192070	43471863	G	0.25	0.2143	A
10	TIGRP2P139550_rs8583806	43482931	C	0.25	0.2143	G
10	BICF2P1073900	43498330	A	0.25	0.2143	G
10	BICF2P504478	43505277	C	0.25	0.2143	A
10	TIGRP2P139560_rs9061560	43511921	A	1	1	G
10	BICF2P145412	43520457	0	0	0	G
10	BICF2P853585	43537884	A	0	0	G
10	BICF2P939876	43541219	G	0	0.2143	A
10	BICF2P525121	43557747	G	0	0.2143	A
10	BICF2G630489040	43565519	A	0	0	G
10	BICF2G630489041	43565551	C	0	0	A
10	BICF2P45549	43578888	G	0	0	A
10	BICF2G630489072	43586023	G	0	0	A
10	BICF2G630489091	43599116	A	1	0.7857	G
10	BICF2G630489102	43608598	G	0	0	A
10	BICF2G630489134	43628071	G	0	0	A
10	BICF2G630489146	43631913	A	0	0	G
10	BICF2G630489160	43648675	A	1	0.7857	G
10	BICF2G630489163	43654414	A	0	0	C
10	BICF2G630489164	43666516	A	1	0.7857	G
10	BICF2S23430322	43710058	G	0	0	A
10	BICF2S2326696	43764481	A	0	0	G
10	BICF2G630489186	43772450	A	0.5	0.5	G
10	BICF2S23439828	43788543	G	0	0	A

10	BICF2P1461473	43799631	0	0	0	T
10	BICF2S23049060	43804231	G	0	0	A
10	BICF2S23437528	43818085	A	0	0	G
10	BICF2S2361940	43883008	A	0.5	0.5	G
10	BICF2S2361383	43887311	G	0	0	A
10	BICF2S23624421	43907941	A	0	0	G
10	BICF2S23431593	43941433	G	0.5	0.5	A
10	BICF2S2343305	43946585	A	0.5	0.5	G
10	BICF2G630489204	43974459	A	0.5	0.4167	C
10	BICF2G630489205	43977659	A	0.5	0.5	G
10	BICF2S22945324	43990510	G	0	0	A
10	BICF2G630489215	44000702	A	0	0	G
10	BICF2S2342675	44025503	0	0	0	A
10	BICF2P1017693	44036519	0	0	0	A
10	BICF2P1137656	44052513	A	0.5	0.5	G
10	BICF2S2358325	44065828	A	0	0	G
10	BICF2S2414384	44073077	A	0	0	G
10	BICF2G630489227	44085137	C	0.5	0.5	A
10	BICF2P765908	44091583	A	0.5	0.5	G
10	BICF2G630489259	44111061	G	0	0	A
10	BICF2G630489270	44118130	A	0	0.2143	G
10	BICF2G630489282	44128145	A	0	0.2143	G
10	BICF2G630489298	44138969	G	0	0.2143	A
10	BICF2G630489335	44164207	A	0	0	C
10	BICF2S2444832	44166499	G	0	0	A
10	BICF2G630489356	44182803	G	0	0	A
10	BICF2G630489358	44184206	G	0	0.2143	A
10	BICF2G630489364	44201655	G	0	0	A
10	TIGRP2P139652_rs8732220	44228690	G	0	0	A
10	TIGRP2P139653_rs8542630	44232198	0	0	0	A
10	BICF2P276951	44243684	A	0	0	T
10	TIGRP2P139657_rs8578244	44255730	A	0	0	G
10	TIGRP2P139659_rs8593112	44264087	G	0	0	A
10	BICF2G630489373	44285234	A	0.5	0.5	G
10	BICF2G630489378	44290193	G	0.5	0.5	A
10	BICF2G630489384	44302212	A	0	0.2143	G
10	TIGRP2P139666_rs8739623	44317016	0	0	0	A
10	BICF2G630489393	44327168	A	0.5	0.5	G
10	BICF2G630489395	44341615	G	0	0	A
10	BICF2G630489396	44349474	G	0	0.2143	A
10	BICF2G630489399	44355645	A	0	0	G
10	BICF2G630489401	44368645	A	0	0	G
10	BICF2P810265	44388352	A	0	0	C
10	BICF2G630489408	44398279	A	0.5	0.5	G
10	BICF2S22911059	44402496	G	0.5	0.5	A
10	BICF2S2374545	44427894	G	0.5	0.2857	A
10	BICF2S2298579	44439993	A	0	0.2143	G

10	TIGRP2P139688_rs9049951	44448909	A	0.5	0.5	G
10	TIGRP2P139697_rs9177090	44460451	A	0.5	0.5	G
10	BICF2P1183622	44474127	A	0	0.2143	G
10	BICF2S23059613	44499914	A	1	0.7857	C
10	TIGRP2P139722_rs8888545	44523289	A	0	0.2143	G
10	BICF2S23655231	44536897	G	0	0.2143	A
10	BICF2P581061	44550732	A	0	0.2143	G
10	BICF2P1377460	44561414	G	0.5	0.5	A
10	BICF2P334949	44574658	G	0	0.2143	A
10	TIGRP2P139737_rs9201446	44595080	A	0	0.2143	C
10	BICF2S24414849	44602357	0	0	0	G
10	BICF2P480840	44616856	A	0	0.2143	C
10	BICF2P1333074	44627065	A	0	0.2143	C
10	BICF2P1036734	44634142	A	1	0.7857	G
10	BICF2S23724955	44644266	A	0	0	G
10	BICF2P254286	44665280	A	0	0.2143	G
10	BICF2P1424160	44669036	0	0	0	G
10	BICF2S2449958	44717055	G	0.5	0.2857	A
10	BICF2P1404240	44732290	0	0	0	T
10	BICF2S23152821	44760475	G	0	0.1667	A
10	BICF2P913643	44802054	A	0	0	G
10	BICF2S22939332	44814574	C	0	0	G
10	BICF2S2315074	44815554	A	0.5	0.5	G
10	BICF2S23257139	44831771	A	0.5	0.5	G
10	TIGRP2P139770_rs8714789	44861065	A	0.5	0.5	G
10	TIGRP2P139772_rs9064513	44865134	G	0.5	0.5	A
10	BICF2P765325	44873297	G	0.5	0.5	A
10	BICF2P224361	44891997	A	0.5	0.5	G
10	BICF2S23053174	44903609	G	0	0	A
10	BICF2P843694	44915968	G	0	0	A
10	TIGRP2P139789_rs8977415	44935714	G	1	1	A
10	TIGRP2P139810_rs8974037	44952578	A	1	1	G
10	BICF2P400957	44955958	G	1	1	A
10	BICF2P214108	44972905	G	0	0	A
10	TIGRP2P139821_rs8723298	44979138	A	0	0	G
10	BICF2P605282	44997040	A	0	0	G
10	BICF2P701358	45016763	A	0	0	G
10	TIGRP2P139832_rs8979293	45019270	A	0	0	G
10	BICF2P568243	45024878	A	0	0	G
10	BICF2P585664	45036352	C	0	0	A
10	BICF2P704927	45049375	A	0	0	G
10	TIGRP2P139844_rs9240558	45064366	A	0	0	G
10	BICF2P1301571	45078145	A	0	0	G
10	BICF2P835369	45083267	G	0	0	A
10	BICF2P1133346	45096357	C	0	0	A
10	BICF2P562703	45102863	G	0	0	A
10	BICF2P1223150	45105941	G	0	0	A

10	BICF2S23647462	45117963	T	1	1	A
10	BICF2P1243631	45135020	G	0	0	A
10	BICF2P1243622	45137099	T	0	0	A
10	BICF2P63884	45143831	A	1	1	G
10	BICF2P933910	45149929	A	0	0	G
10	BICF2S2449082	45176158	G	0	0.2143	A
10	BICF2P181997	45184159	G	1	0.7857	A
10	TIGRP2P139866_rs8736612	45210778	A	0	0	G
10	BICF2P335557	45219705	G	1	0.7857	A
10	BICF2S2343485	45230538	A	0	0.2143	C
10	BICF2P1154868	45243460	A	1	0.7857	G
10	BICF2P1379781	45256166	G	1	0.7857	A
10	TIGRP2P139884_rs8991316	45271169	G	0	0	A
10	BICF2P183346	45296138	G	0	0	A
10	BICF2P522140	45318838	A	0	0	G
10	BICF2P1234018	45325281	G	1	1	A
10	BICF2S2325649	45327219	G	1	1	A
10	BICF2P423149	45336273	G	0	0	A
10	BICF2P423148	45337227	G	0	0	A
10	BICF2S23252418	45348002	A	0	0	C
10	BICF2P1023825	45365077	A	0	0	G
10	BICF2P3733	45370356	A	0	0	G
10	BICF2P1001809	45382452	A	1	1	G
10	BICF2P1001810	45382527	T	0	0	A
10	BICF2P1283185	45391299	C	0	0	A
10	BICF2P712695	45403748	A	0	0	G
10	BICF2P185678	45414962	A	0.5	0.7143	G
10	BICF2P497520	45432592	A	1	1	G
10	BICF2P391820	45438015	G	1	1	A
10	BICF2P1214286	45461118	G	0.5	0.2857	A
10	BICF2P102572	45503048	G	0.5	0.2857	A
10	BICF2P433551	45516118	C	0.5	0.7143	A
10	BICF2P468559	45519391	0	0	0	A
10	BICF2P274981	45529021	G	0	0	A
10	BICF2P950169	45547512	G	0.5	0.2857	A
10	TIGRP2P139922_rs8698287	45558489	G	0.5	0.2857	A
10	BICF2P1059934	45565736	G	0	0	A
10	BICF2P969615	45589457	A	0	0	G
10	BICF2P861550	45604813	A	0	0	G
10	BICF2P960330	45611859	0	0	0	C
10	BICF2P431058	45631037	G	0	0	A
10	TIGRP2P139950_rs9072316	45632213	0	0	0	A
10	BICF2P886693	45643689	0	0	0	A
10	BICF2P1201228	45660146	G	0	0	A
10	BICF2S24114004	45670337	G	0	0	A
10	BICF2P1025931	45682635	0	0	0	A
10	TIGRP2P139956_rs9068843	45690207	0	0	0	G

10	BICF2P1368046	45705116	0	0	0	A
10	BICF2P1189462	45720420	A	0.5	0.5	G
10	BICF2P667480	45726040	G	0	0	A
10	BICF2P450212	45736998	G	0.5	0.5	A
10	BICF2P1332371	45749555	C	0	0	A
10	BICF2S23510683	45760742	C	1	0.7857	G
10	BICF2P1290796	45775969	G	0.5	0.5	A
10	TIGRP2P139963_rs8692653	45790760	A	0	0	G
10	BICF2P1169011	45790854	A	0.5	0.5	G
10	BICF2S23013987	45800309	G	0	0	A
10	BICF2S23013988	45800384	A	0	0	G
10	BICF2P1415214	45806605	A	0	0	G
10	BICF2P767733	45816029	A	0	0	G
10	BICF2P1134152	45836042	A	0	0	C
10	TIGRP2P139977_rs9008250	45845729	G	0	0	A
10	TIGRP2P139978_rs8969291	45865615	A	0	0	G
10	G601f49S234	45875098	G	0	0	A
10	BICF2S23720057	45897329	A	0	0	G
10	BICF2P493191	45911493	0	0	0	C
10	BICF2S23040801	45925642	C	1	0.7857	A
10	BICF2S2448757	45952674	A	0	0	G
10	BICF2S23162594	45954997	G	0	0	A
10	BICF2P953887	45973325	C	0.5	0.5	A
10	BICF2P1137443	45980053	G	0.5	0.2857	A
10	BICF2P1035869	45992727	A	0.5	0.5	T
10	BICF2P746591	46009270	0	0	0	A
10	BICF2S22917457	46018089	A	0	0	G
10	BICF2P368233	46025909	0	0	0	G
10	TIGRP2P139993_rs8611738	46053943	A	0	0	G
10	BICF2S23728079	46068971	A	0	0	T
10	BICF2P490171	46090107	G	0	0	A
10	BICF2P538238	46119710	A	0.5	0.5	G
10	BICF2P883157	46133097	A	0	0	G
10	BICF2S23725864	46142053	G	0.5	0.5	A
10	BICF2S23248987	46159904	C	0.5	0.5	A
10	BICF2P904777	46162557	A	0	0	G
10	BICF2P516844	46165181	G	0.5	0.5	A
10	BICF2P719245	46172615	A	0.5	0.5	G
10	BICF2P491531	46188597	C	0.5	0.5	A
10	BICF2P491532	46189138	C	0.5	0.5	A
10	BICF2P1398946	46192454	A	0.5	0.5	G
10	BICF2P975062	46202123	G	0.5	0.2857	A
10	BICF2P1010653	46213908	G	0.5	0.5	A
10	BICF2P621532	46228328	G	0.5	0.2857	A
10	BICF2P892190	46233715	G	0	0	A
10	BICF2S23113199	46246942	A	0.5	0.2857	G
10	BICF2P759701	46255012	A	0	0	C

10	BICF2P310568	46271817	A	0.5	0.2857	T
10	BICF2S2346831	46282707	A	0	0	G
10	BICF2P480197	46288578	0	0	0	A
10	BICF2P453093	46317142	G	0	0	A
10	BICF2S23216408	46338238	A	0.5	0.5	G
10	TIGRP2P140009_rs8729527	46350461	0	0	0	A
10	BICF2S23433663	46356582	G	0	0	A
10	TIGRP2P140015_rs8854829	46368121	C	0	0	A
10	TIGRP2P140020_rs8669028	46386701	A	0.5	0.5	G
10	BICF2P193369	46400237	A	1	1	C
10	BICF2P777318	46412804	C	0	0	A
10	BICF2P1265867	46419917	A	0	0	G
10	TIGRP2P140032_rs8813807	46434749	C	0	0	A
10	BICF2P175859	46439004	0	0	0	A
10	BICF2P1007870	46458607	A	1	1	G
10	BICF2P824541	46461792	0	0	0	G
10	BICF2P64662	46473282	A	0	0	G
10	BICF2P1415306	46496773	A	0	0	G
10	BICF2P1111589	46516468	G	0	0	A
10	BICF2P1113556	46521178	G	0	0	A
10	BICF2S23635132	46535350	G	0	0	A
10	BICF2P676636	46544502	A	0	0	G
10	BICF2P431557	46554064	A	0	0	G
10	BICF2S2294616	46571139	G	0	0	A
10	BICF2P599505	46580732	G	0	0	A
10	BICF2S22942867	46592080	A	0	0	C
10	BICF2S23554214	46601724	A	0	0	G
10	BICF2P194025	46617231	A	0	0	G
10	BICF2P929545	46631044	G	0	0	A
10	BICF2P117210	46641861	0	0	0	A
10	BICF2P1225214	46663039	G	0.5	0.2857	A
10	TIGRP2P140041_rs9096548	46680527	G	1	1	A
10	BICF2P336294	46698137	C	0.5	0.7143	A
10	BICF2P800683	46701575	0	0	0	A
10	BICF2P695666	46718324	G	0.5	0.7143	A
10	BICF2P269282	46729702	A	0.5	0.7143	G
10	BICF2P334671	46738918	0	0	0	A
10	BICF2P92787	46802461	G	0.5	0.7143	C
10	BICF2P76950	46814829	A	0.5	0.7143	C
10	BICF2P666500	46835010	0	0	0	G
10	BICF2P1060082	46848298	A	0.5	0.7143	G
10	BICF2S22922842	46859412	A	0	0	T
10	BICF2P515506	46865964	0	0	0	G
10	BICF2S22930022	46877388	G	0	0	A
10	TIGRP2P140098_rs8747466	46891415	0	0	0	A
10	BICF2P199429	46899038	0	0	0	G
10	BICF2S245243	46911528	T	0	0	A

10	BICF2P1272856	46961231	G	1	1	A
10	TIGRP2P140102_rs8686598	46969605	A	1	1	G
10	TIGRP2P140108_rs8831631	46979398	A	1	1	C
10	BICF2S23112136	46996672	0	0	0	G
10	TIGRP2P140123_rs9039152	47011584	A	0	0	G
10	BICF2P765310	47012961	A	0	0	C
10	BICF2P1423434	47034725	C	0	0	A
10	TIGRP2P140131_rs8524939	47038721	G	0	0	A
10	BICF2P1055222	47049629	C	0	0	A
10	BICF2P604806	47058957	A	0	0	T
10	BICF2P975750	47078351	A	0	0	G
10	BICF2P430768	47084456	A	0	0	G
10	BICF2P1167660	47096763	G	0	0	A
10	BICF2P1161631	47105569	G	1	1	C
10	BICF2P978820	47132553	0	0	0	A
10	BICF2P1052375	47147239	0	0	0	A
10	BICF2P1369747	47154045	0	0	0	A
10	BICF2S23712415	47162314	0	0	0	A
10	BICF2P131546	47174270	0	0	0	G
10	BICF2P657987	47218092	0	0	0	C
10	BICF2S23727804	47237475	A	0	0	G
10	BICF2S2412201	47254227	G	0	0	A
10	G602f57S143	47268009	G	0	0	A
10	BICF2P510591	47296613	0	NA	0	G
10	BICF2P737332	47300591	0	0	0	A
10	BICF2P1185080	47311801	0	0	0	A
10	BICF2S23238847	47338487	G	0	0	A
10	BICF2S23238846	47338727	C	0	0	A
10	BICF2P1042520	47356309	0	0	0	A
10	BICF2P1042521	47357591	A	0	0	G
10	BICF2P605501	47373081	A	0	0	G
10	BICF2S23045738	47383468	0	0	0	A
10	TIGRP2P140159_rs8731160	47403195	C	0	0	A
10	BICF2P499465	47427981	A	0	0	G
10	BICF2P642641	47439047	0	0	0	A
10	BICF2P218081	47449467	G	0	0	A
10	BICF2P1265786	47464232	A	0	0	G
10	BICF2S23119583	47471291	G	0	0	A
10	TIGRP2P140186_rs9014262	47476963	A	0	0	G
10	BICF2S23249786	47486547	A	0	0	G
10	BICF2P26578	47495987	A	0	0	G
10	BICF2P1325377	47512353	A	0	0	G
10	TIGRP2P140244_rs8909636	47524260	C	0	0	A
10	BICF2S2296279	47548376	G	0	0	A
10	BICF2P441145	47557787	0	0	0	A
10	BICF2P514757	47585417	A	0	0	G
10	TIGRP2P140252_rs9192317	47597928	0	0	0	G

10	G603f51S284	47609870	0	0	0	C
10	BICF2P1301151	47632376	A	0	0	C
10	TIGRP2P140253_rs8827627	47643611	C	0	0	A
10	BICF2P1330339	47654742	0	0	0	C
10	BICF2P1330338	47660038	G	0	0	A
10	BICF2P631582	47668766	A	0	0	G
10	BICF2P751397	47688095	A	0	0	G
10	BICF2P1212053	47701080	G	0	0	A
10	BICF2P264175	47707894	G	0	0	A
10	BICF2P837939	47713981	G	0	0	A
10	TIGRP2P140282_rs8581088	47737234	A	0	0	G
10	BICF2P173604	47752745	G	0	0	A
10	BICF2P249010	47772095	G	0	0	A
10	BICF2P423562	47783793	A	0	0	C
10	BICF2P303476	47813250	A	0	0	G
10	BICF2P552450	47819114	A	0	0	C
10	BICF2P1164247	47828720	A	0	0	G
10	BICF2P1013873	47842307	C	0	0	G
10	BICF2P1013871	47843323	G	0	0	A
10	BICF2P354371	47856083	A	0	0	G
10	BICF2P795320	47866916	G	0	0	A
10	BICF2P582256	47878552	0	0	0	A
10	BICF2P1274358	47887520	0	0	0	A
10	BICF2P320070	47900208	A	0	0	G
10	BICF2P1032271	47909444	A	0	0	C
10	BICF2P879265	47924184	A	0	0	G
10	BICF2S23044711	47932496	G	0	0	A
10	BICF2P640502	47949621	A	0	0	G
10	BICF2P579019	47959122	A	0	0	G
10	BICF2P382989	47977206	G	0	0	A
10	BICF2P410787	47980584	C	0	0	A
10	BICF2P320635	47989661	A	0	0	T
10	BICF2S22939177	48004279	0	0	0	G
10	TIGRP2P140337_rs9008188	48021549	A	0	0	G
10	BICF2P576184	48028764	0	0	0	A
10	BICF2S23135825	48045077	G	0.5	0.2857	A
10	BICF2S23431884	48055479	A	0.5	0.2857	C
10	BICF2P844381	48067447	C	0.5	0.2857	G
10	TIGRP2P140341_rs9138355	48089405	G	0	0	C
10	BICF2P797500	48096023	G	0	0	A
10	BICF2P190591	48106191	A	0	0	G
10	BICF2P662426	48116743	A	0	0	C
10	TIGRP2P140359_rs9202217	48139032	0	0	0	C
10	BICF2P839270	48153911	A	0.5	0.2857	G
10	BICF2P1245418	48162209	A	0.5	0.2857	G
10	BICF2P351285	48174656	G	0.5	0.2857	A
10	BICF2P473593	48188141	A	0.5	0.7143	G

10	BICF2P436022	48202682	G	0.5	0.2857	A
10	BICF2P230142	48215980	0	0	0	C
10	BICF2P1306137	48229894	G	0.5	0.7143	A
10	BICF2P637575	48236520	G	0	0	A
10	BICF2P699235	48261183	G	0	0	A
10	BICF2S23233393	48267951	G	0	0	A
10	BICF2S22945304	48284503	A	0.5	0.2857	G
10	BICF2S23434367	48293365	A	0	0	G
10	BICF2P55149	48301085	A	0	0	G
10	BICF2S23426470	48312738	A	0.5	0.7143	C
10	TIGRP2P140368_rs9064797	48326484	A	0	0	G
10	BICF2S23415217	48334976	C	0	0	A
10	BICF2P737166	48347204	G	0	0	A
10	BICF2P1137388	48358942	C	0	0	A
10	BICF2P344756	48373684	A	0	0	G
10	BICF2P1107602	48385624	G	0.5	0.7143	A
10	BICF2P1173568	48394140	0	0	0	A
10	BICF2P1435551	48411303	A	0	0	G
10	BICF2S23418599	48426030	A	0	0	G
10	BICF2S23741895	48429328	G	0	0	A
10	BICF2P399575	48444897	C	0	0	A
10	BICF2P797208	48449898	G	0.5	0.7143	A
10	BICF2P178143	48467464	A	0	0	C
10	TIGRP2P140388_rs9190295	48473026	A	0.5	0.7143	G
10	BICF2P1060335	48486356	G	0.5	0.75	A
10	BICF2P140940	48496503	A	0.5	0.7143	C
10	BICF2P135433	48525079	A	0	0	G
10	TIGRP2P140413_rs9038337	48530062	C	0.5	0.7143	G
10	TIGRP2P140421_rs9246415	48542279	A	1	1	C
10	TIGRP2P140422_rs9246416	48542479	A	0.5	0.7143	G
10	BICF2P170971	48558502	G	0	0	A
10	BICF2P1198312	48570589	A	0	0	C
10	BICF2S2344451	48582562	A	0.25	0.3571	G
10	BICF2S23327324	48594997	A	0.25	0.3571	G
10	TIGRP2P140443_rs8563943	48604921	A	0.5	0.7143	G
10	TIGRP2P140445_rs9175617	48615487	T	0	0	A
10	BICF2S23111064	48627995	A	0	0	G
10	TIGRP2P140464_rs8948715	48656355	A	0	0	G
10	BICF2P813747	48672173	A	NA	0	C
10	BICF2P1201439	48685647	A	0	0	C
10	BICF2P527115	48694059	G	0	0	A
10	BICF2P777362	48703125	G	0	0	A
10	BICF2P160200	48717626	A	0.5	0.2857	G
10	BICF2P158891	48726542	G	0.5	0.7143	A
10	BICF2P538817	48747235	C	0.5	0.7143	A
10	TIGRP2P140479_rs9194695	48758362	A	0	0	G
10	BICF2P519537	48781928	0	0	0	A

10	BICF2P417813	48783951	A	0	0	G
10	BICF2P919131	48795460	A	0	0	G
10	BICF2P895044	48802826	G	0	0	A
10	TIGRP2P140494_rs8891607	48806189	C	0	0	G
10	BICF2P1215157	48824301	A	0	0	G
10	TIGRP2P140509_rs9125003	48836095	0	0	0	A
10	BICF2P815806	48844290	0	0	0	G
10	BICF2S23737472	48852437	A	0.5	0.7143	G
10	BICF2P895715	48873222	G	0	0	A
10	BICF2P1375311	48881546	0	0	0	G
10	BICF2S2312577	48896281	A	0	0	G
10	BICF2P469762	48902006	C	0	0	G
10	BICF2P1058389	48922960	0	0	0	G
10	BICF2P556028	48939909	0	0	0	G
10	TIGRP2P140530_rs8684144	48948980	C	0	0	A
10	BICF2P405980	48963402	0	0	0	G
10	BICF2P823791	48976608	0	0	0	A
10	BICF2P884191	48988278	A	0	0	G
10	TIGRP2P140531_rs8593773	49005496	G	0	0	C
10	TIGRP2P140533_rs8830620	49018112	G	0	0	A
10	BICF2P1142650	49035909	G	0	0	A
10	BICF2P1142649	49036517	A	0	0	G
10	BICF2P1251048	49051740	C	0	0	A
10	TIGRP2P140547_rs8942444	49062328	C	0	0	A
10	BICF2S23251816	49081773	A	0	0	G
10	BICF2P1174613	49092792	A	0	0	T
10	BICF2P613390	49111244	0	0	0	A
10	TIGRP2P140552_rs8980453	49147523	A	0	0	G
10	TIGRP2P140554_rs8674357	49162133	G	0	0	A
10	BICF2S23210774	49177648	0	0	0	G
10	BICF2P715511	49190173	0	0	0	G
10	BICF2P1090099	49223755	A	0	0	G
10	BICF2P364534	49241973	A	0	0	G
10	BICF2P563295	49247767	G	0	0	A
10	BICF2P1125295	49261464	A	0.5	0.7143	G
10	BICF2P475960	49267100	A	0.5	0.7143	G
10	BICF2P743042	49278236	A	0.5	0.7143	G
10	BICF2P324205	49315986	G	0.5	0.75	A
10	BICF2P231168	49323883	G	0.5	0.7143	A
10	BICF2P750403	49357690	G	0	0	A
10	TIGRP2P140598_rs8921159	49359295	G	0	0	A
10	BICF2P1325940	49378606	0	NA	0	C
10	BICF2P638205	49386335	A	0	0	T
10	BICF2S23114872	49393762	A	0	0	G
10	TIGRP2P140615_rs8835746	49408320	G	0	0	A
10	BICF2P915373	49408463	A	0	0	G
10	BICF2P1167552	49415564	G	0	0	A

10	BICF2P159927	49420291	A	0	0	G
10	BICF2P1018381	49424827	A	0	0	C
10	BICF2P868981	49431717	A	0	0	G
10	BICF2P580773	49441321	G	0.5	0.2857	A
10	BICF2P309612	49450123	A	0.5	0.2857	G
10	TIGRP2P140627_rs8986483	49470182	C	0.5	0.7143	A
10	BICF2S23157851	49479603	G	0.5	0.75	A
10	BICF2S23028302	49486311	A	0.5	0.7143	G
10	BICF2P729369	49502113	A	0.5	0.7143	G
10	BICF2P285574	49512150	G	0.5	0.7143	A
10	TIGRP2P140634_rs8550304	49523070	A	0.5	0.7143	C
10	TIGRP2P140638_rs8848702	49533942	G	0.5	0.7143	A
10	TIGRP2P140640_rs9200059	49548921	G	0.5	0.7143	A
10	TIGRP2P140646_rs8651520	49563710	G	0.25	0.3571	A
10	BICF2P1103247	49572877	A	0.25	0.3571	C
10	BICF2P1056332	49584101	A	0.75	0.6429	G
10	BICF2P601639	49593364	A	0.25	0.3571	C
10	TIGRP2P140656_rs9033492	49602733	G	0.5	0.7143	A
10	TIGRP2P140659_rs8653000	49618598	A	0.25	0.3571	G
10	BICF2P371832	49635540	A	0.25	0.3571	G
10	BICF2S23911776	49652314	G	0	0	A
10	BICF2P98311	49688292	G	0	0	A
10	BICF2P378903	49691606	G	0	0	A
10	BICF2P1073054	49703702	G	0	0	A
10	BICF2P316680	49715500	G	0.25	0.3571	A
10	BICF2P1328944	49745955	A	0.25	0.3571	G
10	BICF2P550910	49761427	A	0.25	0.3571	C
10	BICF2P475378	49773186	A	0	0	G
10	BICF2P1371746	49787246	A	0.5	0.2857	G
10	BICF2P1002982	49797389	A	0.25	0.3571	G
10	BICF2P1338749	49810449	A	0.25	0.3571	C
10	BICF2P1083924	49819838	C	0.25	0.3571	A
10	BICF2S2329136	49830222	G	0.25	0.3571	A
10	BICF2P1394262	49843900	C	0.25	0.3571	A
10	BICF2P583269	49853194	G	0	0	C
10	BICF2P1305225	49866589	A	0.25	0.3571	G
10	BICF2P513683	49883282	G	0.25	0.3571	A
10	BICF2P365225	49897267	T	0.25	0.3571	A
10	BICF2P512821	49903482	G	0.25	0.3571	A
10	BICF2S23041026	49961459	C	0	0	A
10	BICF2S23514637	49968155	G	0	0	A
10	BICF2S23719690	49990618	G	0	0	A
10	BICF2P104632	50017784	0	0	0	A
10	BICF2S2294875	50048089	A	0.25	0.3571	G
10	TIGRP2P140746_rs8514365	50070417	0	0	0	A
10	G605f45S73	50106985	0	0	0	C
10	BICF2S23719213	50132283	A	0.5	0.75	G

10	BICF2S239504	50168821	A	0	0	G
10	BICF2P735527	50191317	0	0	0	A
10	TIGRP2P140753_rs9087643	50215599	0	0	0	G
10	TIGRP2P140754_rs8959982	50221671	0	0	0	C
10	G607f36S111	50232652	A	0.25	0.3571	G
10	BICF2P734255	50253444	0	0	0	C
10	BICF2P971107	50275862	0	0	0	G
10	BICF2P1549	50287047	0	0	0	G
10	BICF2S23519584	50295069	A	0	0	G
10	BICF2P1396858	50308719	G	0	0	A
10	BICF2P1396854	50311980	C	0	0	A
10	BICF2P310475	50312580	0	0	0	A
10	BICF2P306713	50324698	A	0.25	0.3571	G
10	BICF2P575763	50350387	A	0.75	0.6429	G
10	BICF2P1295202	50361732	A	0	0	C
10	BICF2S23319924	50379748	A	0	0	G
10	BICF2P1284430	50388961	G	0.75	0.6429	A
10	BICF2S23025171	50402063	G	0.75	0.6429	A
10	BICF2S23014216	50405977	A	0.75	0.6429	G
10	BICF2S23550619	50425538	T	0.75	0.6429	A
10	BICF2P1172998	50431903	G	0.75	0.6429	A
10	TIGRP2P140794_rs8553661	50435520	A	0.75	0.6429	G
10	TIGRP2P140798_rs8946486	50441059	0	0	0	C
10	BICF2P625002	50469408	0	0	0	G
10	BICF2P963328	50482360	A	0.25	0.3571	G
10	BICF2P652022	50495458	A	0.75	0.6429	G
10	BICF2P652397	50499295	G	0.75	0.6429	A
10	TIGRP2P140815_rs8868057	50508920	G	0	0	A
10	BICF2S23638822	50530312	C	0	0	A
10	TIGRP2P140824_rs8834559	50531990	A	0	0	G
10	TIGRP2P140833_rs9046989	50543709	A	0	0	G
10	BICF2S23036635	50558977	A	0	0	G
10	BICF2S23760334	50568595	A	0.25	0.3571	G
10	BICF2S23036428	50585703	G	0.25	0.3571	A
10	BICF2P879346	50601178	A	0.25	0.3571	G
10	BICF2P370816	50614162	A	0.25	0.3571	G
10	BICF2S23337089	50618364	A	0.25	0.3571	G
10	BICF2S234632	50627370	A	0	0	G
10	TIGRP2P140864_rs8561246	50637973	C	0	0	A
10	BICF2P685406	50654077	A	0.25	0.3571	G
10	BICF2P1413307	50667609	A	0.25	0.3	G
10	BICF2P1413306	50667939	A	0	0	G
10	BICF2P236853	50676680	0	0	0	G
10	BICF2S22911780	50689352	A	0	0	T
10	TIGRP2P140870_rs8594984	50699600	C	0	0	G
10	BICF2P1396250	50707576	G	0	0	A
10	BICF2P1276314	50712625	A	0	0	T

10	BICF2P1416883	50720841	A	0	0	G
10	BICF2S23426994	50729667	A	0	0	G
10	BICF2S23412302	50733918	A	0	0	G
10	BICF2P706009	50748819	A	0	0	G
10	BICF2P1174831	50757085	G	0	0	A
10	TIGRP2P140889_rs8627994	50767357	A	0	0	G
10	BICF2P1271347	50767454	G	0	0	A
10	BICF2P1421263	50779600	G	0	0	A
10	TIGRP2P140898_rs8847984	50784215	A	0	0	G
10	TIGRP2P140899_rs8880524	50801676	A	0	0	G
10	BICF2P1429720	50805618	A	0	0	G
10	TIGRP2P140903_rs8931164	50809044	A	0	0	T
10	BICF2P471586	50819428	A	0	0	C
10	BICF2P727502	50856455	A	0	0	G
10	BICF2P817476	50876352	G	0.75	0.6429	A
10	BICF2P280956	50889458	A	0	0	G
10	BICF2S23430723	50904244	G	0	0	A
10	BICF2P1179211	50915861	G	0.75	0.6429	A
10	BICF2P563108	50923309	A	0	0	G
10	BICF2P950087	50934157	G	0	0	A
10	TIGRP2P140909_rs8911642	50952878	T	0	0	A
10	BICF2P797997	50967110	C	0	0	A
10	BICF2S2333017	50973375	G	0	0	A
10	BICF2P1229357	50989016	A	0	0	C
10	BICF2P138204	50999225	A	0	0	G
10	TIGRP2P140920_rs8563734	51003990	G	0	0	A
10	BICF2P1324352	51027235	A	0	0	G
10	TIGRP2P140942_rs8957933	51047274	A	0	0	G
10	BICF2S2452559	51049130	A	0	0	G
10	BICF2P1371660	51067926	A	0	0	C
10	BICF2S2357364	51073299	C	0	0	A
10	BICF2S2335025	51091105	C	0	0	A
10	TIGRP2P140954_rs8835950	51104711	A	0	0	G
10	BICF2P1281970	51111495	A	0	0	G
10	BICF2P212239	51120198	G	0	0	A
10	BICF2P697079	51160281	A	0	0	C
10	BICF2P910465	51180552	A	0	0	G
10	BICF2P1095909	51187023	A	0	0	G
10	BICF2P1346869	51205222	A	0.5	0.2857	G
10	BICF2P409457	51211022	C	0.5	0.2857	A
10	BICF2P175559	51225872	A	0.75	0.6429	T
10	BICF2P43725	51238416	G	0	0	A
10	BICF2P1355694	51254374	A	0.5	0.2857	G
10	BICF2S23043872	51256560	G	0.5	0.2857	A
10	BICF2P1296086	51284373	A	0.25	0.3571	G
10	BICF2S22947221	51291943	A	0.25	0.3571	G
10	TIGRP2P141001_rs8509255	51302709	A	0	0	G

10	BICF2P916076	51322765	A	0	0	G
10	BICF2P559995	51326866	G	0	0	A
10	BICF2P135325	51339239	A	0	0	G
10	BICF2P969034	51358165	0	0	0	T
10	TIGRP2P141014_rs9146482	51370493	0	0	0	G
10	BICF2P1263933	51371186	0	0	0	A
10	BICF2S2345939	51384418	A	0	0	G
10	BICF2S23438101	51396993	A	0.5	0.7143	G
10	BICF2S232399	51409615	A	0.25	0.3571	C
10	BICF2P792386	51418120	A	0.5	0.2857	G
10	BICF2P604767	51433223	G	0	0	A
10	BICF2P630147	51440869	A	0	0	G
10	BICF2P72878	51452644	G	0	0	A
10	BICF2S23635809	51471125	A	0.75	0.6429	G
10	BICF2P671728	51484860	A	0.5	0.7143	G
10	BICF2S23722746	51492917	A	0	0	G
10	BICF2S23421028	51497234	G	0	0	A
10	BICF2S23046875	51540995	A	0	0	G
10	TIGRP2P141038_rs8671023	51560110	A	0.75	0.6429	G
10	BICF2P916178	51583544	G	0	0	A
10	TIGRP2P141065_rs8714074	51594381	A	0	0	G
10	BICF2S2348758	51600870	A	0.75	0.6429	G
10	TIGRP2P141080_rs8724112	51630559	A	0	0	G
10	BICF2S23012179	51655799	A	0.25	0.3571	G
10	BICF2P1437391	51667277	0	0	0	G
10	TIGRP2P141086_rs8744982	51673000	A	0.5	0.2857	G
10	BICF2P403250	51688753	C	0	0	G
10	BICF2P403249	51688876	A	0	0	G
10	BICF2S24321816	51691482	T	0	0	A
10	BICF2P123121	51691502	G	0.25	0.3571	A
10	BICF2S23662004	51700843	A	0.5	0.7143	G
10	TIGRP2P141098_rs8690011	51707292	A	0.5	0.7143	G
10	BICF2P432142	51715584	A	0.5	0.2857	G
10	BICF2P1413978	51730747	A	0	0	G
10	TIGRP2P141115_rs9147834	51749083	A	0.25	0.3571	G
10	TIGRP2P141117_rs8798593	51752770	G	0.75	0.6429	A
10	TIGRP2P141130_rs8883070	51766399	G	0.5	0.2857	A
10	BICF2P777540	51779247	G	0.25	0.3571	A
10	BICF2P801124	51796759	A	0.75	0.6429	G
10	BICF2S23022854	51809103	A	0	0	G
10	BICF2P384680	51831584	A	0.25	0.3571	G
10	BICF2S23619301	51847978	G	0	0	A
10	TIGRP2P141147_rs8653133	51856780	A	0.25	0.3571	G
10	BICF2P516731	51871909	0	0	0	C
10	BICF2P1083276	51886583	G	0	0	A
10	BICF2P1250183	51890513	G	0.5	0.25	A
10	BICF2P936798	51900962	A	0	0	G

10	BICF2P113417	51919900	A	0	0	G
10	BICF2P879099	51947930	0	0	0	A
10	BICF2S234375	51963915	A	0	0	G
10	BICF2P858306	51971059	A	0.5	0.7143	G
10	BICF2P1027073	51987736	0	0	0	G
10	BICF2P1438362	52002653	G	0.5	0.7143	A
10	BICF2P889845	52005493	A	0.5	0.7143	C
10	BICF2P1378030	52026673	G	0.5	0.7143	A
10	BICF2S23327861	52043182	A	0.25	0.1429	G
10	BICF2P99528	52071772	0	0	0	A
10	BICF2P278253	52073435	0	0	0	G
10	BICF2P1472164	52099240	A	0	0	G
10	BICF2S2315239	52106940	G	0.25	0.1429	A
10	BICF2S22938852	52140094	A	0.25	0.1429	G
10	BICF2P210415	52143774	0	0	0	G
10	TIGRP2P141181_rs8913846	52155145	A	0	0	G
10	BICF2S2337290	52174626	A	0.25	0.5714	G
10	BICF2P576093	52179009	G	0	0	A
10	TIGRP2P141197_rs8856744	52187674	G	0	0	A
10	BICF2S23610713	52206929	G	0.5	0.7143	A
10	BICF2S23642467	52218830	0	0	0	A
10	BICF2P969667	52225835	A	0	0	G
10	BICF2P1135520	52230246	0	0	0	A
10	TIGRP2P141213_rs9201461	52242593	G	0.75	0.4286	A
10	BICF2P1243207	52247453	G	0.5	0.2857	A
10	BICF2S24416625	52251713	G	0	0	A
10	BICF2P223992	52258724	G	0	0	A
10	BICF2P117402	52275976	A	0	0	G
10	BICF2P726753	52289586	A	0	0	G
10	BICF2P949124	52294880	A	0.5	0.2857	G
10	BICF2P776916	52306228	A	0	0	G
10	TIGRP2P141235_rs8861572	52313512	A	0	0	G
10	BICF2P118402	52327600	A	0.5	0.2857	G
10	BICF2P1269903	52346603	G	0	0	A
10	BICF2P473982	52349453	A	0.5	0.2857	G
10	BICF2P852525	52358185	0	0	0	G
10	BICF2P852522	52359864	G	0.5	0.2857	A
10	BICF2S23526628	52380430	T	0.5	0.2857	A
10	BICF2P776953	52390719	A	0.5	0.2857	G
10	BICF2S22963842	52401144	A	0.5	0.2857	C
10	BICF2P225634	52415430	A	1	1	G
10	BICF2P901643	52419068	G	0.5	0.2857	A
10	BICF2P1257131	52467095	0	0	0	G
10	BICF2P619795	52490239	0	0	0	A
10	BICF2P312132	52502803	0	0	0	A
10	TIGRP2P141251_rs8490278	52519495	0	0	0	A
10	TIGRP2P141252_rs8770301	52527867	G	0.5	0.7143	C

10	BICF2P270912	52536158	G	0.5	0.7143	A
10	BICF2P855892	52544922	G	0.5	0.7143	A
10	BICF2P199862	52562583	C	0.5	0.2857	A
10	BICF2P640245	52563503	G	0.5	0.7143	A
10	BICF2P261423	52572534	A	0.5	0.7143	G
10	TIGRP2P141287_rs9178819	52581028	A	0	0	G
10	TIGRP2P141289_rs8666824	52595963	A	0.5	0.25	T
10	BICF2P421203	52612128	A	0	0	G
10	BICF2S2372233	52613170	G	0	0	A
10	TIGRP2P141312_rs8577289	52641925	A	0	0	G
10	BICF2S2337451	52648945	A	0	0	T
10	BICF2P462328	52667964	G	0	0	A
10	TIGRP2P141316_rs9252244	52671288	C	0	0	A
10	TIGRP2P141321_rs8733429	52691574	A	0	0	G
10	BICF2S2458721	52722466	C	1	1	A
10	BICF2P773296	52729290	G	0	0	A
10	BICF2P338997	52740720	A	0.5	0.7143	G
10	BICF2S23261214	52767157	0	0	0	A
10	BICF2S22920071	52790284	G	0.5	0.2857	A
10	TIGRP2P141333_rs8824566	52799805	G	0.75	0.8571	A
10	BICF2P247761	52818394	G	0	0	A
10	BICF2P476459	52822892	G	0	0	A
10	BICF2P988929	52825936	A	0.25	0.5714	G
10	BICF2S23725116	52834551	A	0.25	0.1429	C
10	BICF2P285314	52851819	A	0	0	G
10	BICF2P895418	52855982	A	0.5	0.2857	G
10	BICF2S23741968	52869812	A	0.5	0.7143	G
10	BICF2S23128603	52880552	A	0.25	0.1429	G
10	BICF2P1199470	52892205	C	0.5	0.2857	G
10	BICF2S23611798	52907993	0	0	0	G
10	BICF2P556213	52916168	G	0	0	A
10	BICF2P1182788	52929340	G	0	0	A
10	BICF2P1425381	52936780	G	0	0	A
10	TIGRP2P141391_rs9017127	52947309	G	0	0	A
10	TIGRP2P141392_rs8718435	52967986	A	0	0	C
10	BICF2P594170	52971020	A	0	0	G
10	BICF2S22915649	52988337	A	0	0	G
10	TIGRP2P141397_rs8736971	53007392	A	0	0	C
10	BICF2S23255435	53038303	G	0	0	A
10	BICF2P583697	53054313	C	0	0	A
10	BICF2P583698	53054485	A	0.5	0.7143	G
10	BICF2S23653869	53069534	C	0.5	0.7143	A
10	BICF2P609442	53077871	A	0	0	C
10	BICF2P565985	53086080	A	0.25	0.5714	G
10	TIGRP2P141401_rs8977842	53098324	G	0.5	0.5	A
10	BICF2P1083827	53109869	G	0	0	A
10	BICF2S2331161	53126983	A	0.5	0.2857	G

10	BICF2S23530525	53133635	A	0	0	G
10	BICF2S236855	53150946	A	0.75	0.8571	G
10	BICF2P1215764	53160425	0	0	0	A
10	BICF2P207488	53175794	G	0	0	A
10	BICF2P440151	53179016	C	0	0	A
10	BICF2S23226724	53184134	A	0	0	G
10	BICF2P1253324	53189812	A	0	0	G
10	BICF2P997099	53203987	A	0	0	G
10	BICF2P597204	53215531	A	0.5	0.2857	T
10	BICF2P1500119	53229477	G	0	0	A
10	BICF2S23639091	53240541	A	0	0	G
10	BICF2P907372	53246768	A	0	0.1667	G
10	TIGRP2P141414_rs9122801	53260097	G	0.5	0.2857	A
10	BICF2P566165	53270920	A	0	0	G
10	TIGRP2P141419_rs9011909	53271395	A	0.25	0.3571	G
10	TIGRP2P141422_rs8489190	53289056	C	0	0	G
10	BICF2P918072	53293025	G	0	0.2143	A
10	BICF2P438704	53309115	C	0.5	0.5	G
10	BICF2P941220	53317902	A	0	0	G
10	TIGRP2P141437_rs8575951	53328196	0	0	0	C
10	BICF2P258880	53341606	G	0	0	A
10	BICF2P604872	53348622	A	0	0	G
10	BICF2P512486	53360036	C	0.5	1	A
10	BICF2S2354530	53379657	A	0.5	0.7143	G
10	BICF2S23547210	53414958	A	0	0	G
10	BICF2P169463	53429129	0	0	0	A
10	BICF2P683637	53442186	A	1	0.7857	G
10	BICF2S22922588	53482187	G	0	0	A
10	TIGRP2P141453_rs9237505	53488738	0	0	0	G
10	TIGRP2P141459_rs9019273	53499997	A	0	0	G
10	BICF2S23426213	53515855	G	1	1	A
10	BICF2P615803	53523113	A	1	1	T
10	BICF2P366221	53536757	G	0.25	0.1429	A
10	BICF2P730219	53550004	G	0.25	0.1429	A
10	BICF2S22936632	53566333	A	0.25	0.3571	C
10	BICF2S23425996	53569757	A	0.25	0.3571	G
10	BICF2P809230	53587816	G	0.5	0.2857	A
10	BICF2P776381	53590127	G	0.75	0.4286	A
10	BICF2P620211	53606766	0	0	0	A
10	BICF2P1254411	53623805	C	0	0.2143	A
10	BICF2P36524	53628530	A	0	0.2143	G
10	BICF2P1208400	53637289	0	0	0	A
10	TIGRP2P141483_rs8749265	53653670	0	0	0	A
10	BICF2S23327132	53659571	A	0	0.2143	G
10	BICF2P1371813	53681435	A	0	0	G
10	BICF2P393453	53691846	C	0	0	A
10	BICF2P631129	53697159	G	0.5	0.7143	A

10	BICF2S22960598	53713692	A	0	0.2143	T
10	TIGRP2P141497_rs8647868	53718723	G	0.5	0.7143	A
10	BICF2P15564	53733617	A	0	0.2143	G
10	BICF2P13519	53740736	G	0	0.2143	A
10	BICF2S23539816	53753961	A	0	0	C
10	TIGRP2P141512_rs8697528	53769678	A	0	0	G
10	TIGRP2P141514_rs9015008	53781284	A	0	0	G
10	BICF2P655769	53796392	G	0	0.2143	A
10	BICF2P331380	53804944	A	1	0.7857	G
10	TIGRP2P141524_rs8872031	53812774	G	1	0.7857	A
10	BICF2S23339049	53823210	A	1	0.7857	G
10	BICF2S23037799	53835151	G	1	0.7857	A
10	BICF2P399883	53847224	0	0	0	G
10	BICF2P1342617	53852362	0	0	0	G
10	BICF2P806477	53862483	C	0	0	A
10	BICF2P1144339	53869265	A	0	0	C
10	TIGRP2P141543_rs8530373	53878970	0	0	0	A
10	TIGRP2P141544_rs9078878	53894505	A	0	0	C
10	TIGRP2P141549_rs9218744	53901665	A	0	0	G
10	BICF2P168885	53919391	0	0	0	A
10	BICF2S22934123	53934166	0	0	0	A
10	BICF2S23247259	53935111	G	0	0	A
10	TIGRP2P141551_rs9157479	53957273	G	0	0	A
10	BICF2S23641669	53964665	C	1	1	A
10	TIGRP2P141558_rs8713380	53970806	0	0	0	C
10	TIGRP2P141566_rs8490935	53985526	C	1	1	A
10	BICF2P241830	53996368	G	0	0	A
10	BICF2P486473	54005203	G	0	0	A
10	BICF2P358902	54016167	0	0	0	A
10	BICF2P710769	54029095	A	0	0	C
10	BICF2P949640	54047996	0	0	0	G
10	BICF2P1369939	54053769	A	0	0	C
10	BICF2P949653	54062780	A	0.5	0.5	G
10	BICF2P1183997	54079736	0	0	0	A
10	TIGRP2P141575_rs8840931	54085169	0	0	0	T
10	BICF2P138769	54100058	A	0.25	0.1429	G
10	TIGRP2P141576_rs9157074	54107505	A	0.5	0.5	G
10	TIGRP2P141585_rs8882756	54149029	G	0	0	A
10	BICF2P1446832	54156493	G	0	0	A
10	BICF2P92360	54168822	0	0	0	A
10	BICF2P679352	54181596	A	0.25	0.1429	G
10	BICF2P752473	54213138	0	0	0	G
10	BICF2S23156246	54233423	G	0.5	0.5	A
10	BICF2P414147	54239950	A	0.25	0.1429	G
10	TIGRP2P141611_rs9055807	54256328	A	0.25	0.3571	G
10	BICF2P1456613	54262907	A	0	0	G
10	TIGRP2P141615_rs8841212	54275746	0	0	0	A

10	BICF2P786592	54284449	G	0	0	A
10	BICF2P330900	54285965	C	0	0	G
10	TIGRP2P141634_rs9061158	54299722	G	0.25	0.1429	A
10	BICF2P874151	54303244	C	0	0	A
10	BICF2P271616	54317265	C	0	0	A
10	BICF2P268758	54331950	A	0	0	G
10	BICF2S23146467	54341958	G	0.25	0.3571	A
10	BICF2P1004176	54350897	G	0	0	A
10	BICF2P351039	54362689	A	0	0	G
10	BICF2P970247	54375389	G	0	0	A
10	BICF2S23539723	54387081	A	0.25	0.3571	G
10	BICF2S2456524	54398893	0	0	0	G
10	BICF2P1024077	54416040	A	0.25	0.3571	C
10	BICF2P1144849	54418886	G	0	0.2143	A
10	BICF2S22944962	54434306	G	0	0.2143	A
10	BICF2S23230436	54442440	A	0.25	0.3571	G
10	BICF2S23451275	54458338	G	0	0.2143	A
10	BICF2S22944619	54464388	A	0.25	0.5714	G
10	BICF2P948366	54482093	C	0	0	G
10	BICF2P594975	54492506	G	0.25	0.3571	A
10	BICF2S23147693	54498644	G	0	0.2143	A
10	BICF2P310091	54520415	G	0.25	0.5714	A
10	BICF2P1290697	54546217	G	0	0.2143	A
10	TIGRP2P141665_rs8874709	54559962	A	0	0.2143	G
10	BICF2S23625084	54591875	A	0	0	G
10	BICF2P1393909	54614024	0	0	0	A
10	BICF2P418182	54631767	A	0	0	G
10	BICF2P535563	54668066	G	0.5	0.2857	A
10	BICF2S23311001	54675333	A	0	0	T
10	BICF2P129238	54687638	0	0	0	C
10	BICF2S22964147	54696119	0	0	0	G
10	BICF2P1231957	54711568	0	0	0	G
10	BICF2S2304187	54718352	A	0.75	0.4286	G
10	BICF2S23739188	54739366	A	0.25	0.5714	G
10	BICF2P934388	54741156	G	0.5	0.2857	A
10	BICF2S23651604	54758054	0	0	0	A
10	BICF2S23651997	54765475	A	0.25	0.3571	G
10	BICF2P1424112	54774755	A	0	0	G
10	BICF2S23659897	54790549	G	0.25	0.3333	A
10	BICF2S2334910	54804471	C	0.75	0.6429	A
10	BICF2S22925151	54814743	A	0.75	0.6429	G
10	BICF2S23741793	54830075	A	0	0	G
10	BICF2P562769	54853186	0	0	0	C
10	BICF2P1064109	54866221	0	0	0	A
10	BICF2P322794	54873889	A	0.25	0.3571	G
10	BICF2P444457	54885400	A	0	0	G
10	BICF2P992493	54897772	0	0	0	A

10	BICF2P617053	54918608	A	0.75	0.4286	C
10	BICF2S23520061	54928137	G	0	0.2143	A
10	BICF2P1180057	54951455	A	0	0.2143	G
10	BICF2P843879	54959358	A	0.5	0.5	T
10	BICF2P248346	54977421	0	0	0	A
10	BICF2P312885	54982828	A	0.5	0.5	G
10	BICF2P126909	54999142	A	0.5	0.5	G
10	BICF2S2345735	55013214	C	0.25	0.3571	A
10	BICF2S2329978	55029633	A	0.5	0.2857	G
10	BICF2S23056529	55040660	A	0.75	0.6429	G
10	BICF2P627212	55057207	A	0	0	G
10	BICF2S23537948	55070221	A	0.5	0.2857	G
10	BICF2P1305538	55077111	G	0.25	0.1429	A
10	BICF2P1260951	55086171	A	0.25	0.1429	G
10	BICF2P1078815	55100976	A	0	0.2143	G
10	BICF2G630489428	55108298	0	0	0	A
10	BICF2S23150541	55126488	G	0	0	A
10	BICF2P1366149	55135411	0	0	0	A
10	BICF2G630489437	55156673	A	0.25	0.5714	G
10	BICF2G630489468	55166937	A	0.75	0.6429	G
10	BICF2G630489496	55178979	C	0.75	0.8571	A
10	BICF2G630489526	55195769	A	0.5	0.5	C
10	BICF2G630489531	55204569	G	0.5	0.5	A
10	BICF2G630489551	55213314	A	0.5	0.5	G
10	BICF2G630489561	55225138	G	0.25	0.1429	A
10	BICF2P1389763	55238601	C	0.25	0.3571	A
10	BICF2G630489586	55250139	A	0.25	0.1429	G
10	BICF2G630489599	55258103	A	0.25	0.1429	G
10	BICF2G630489616	55275843	G	0.5	0.5	A
10	BICF2G630489641	55284700	A	0.5	0.5	G
10	BICF2G630489662	55300001	C	0.25	0.3571	A
10	BICF2S23243529	55309315	A	0	0	G
10	BICF2G630489693	55325813	A	0	0	G
10	BICF2G630489723	55331745	A	1	0.7857	T
10	BICF2G630489744	55339145	G	0.5	0.7143	A
10	BICF2G630489767	55354396	A	0.5	0.7143	C
10	BICF2G630489776	55362432	A	0	0	G
10	BICF2G630489805	55372960	A	0	0	G
10	BICF2S23158563	55393510	A	0	0	G
10	BICF2G630489848	55404374	A	0	0.2143	G
10	BICF2G630489865	55414758	A	0.25	0.5714	G
10	BICF2G630489877	55418737	G	0	0.2143	A
10	BICF2G630489884	55432019	A	0.25	0.3571	G
10	BICF2G630489897	55441981	A	NA	0.5	C
10	BICF2G630489920	55453689	A	0.25	0.3571	G
10	BICF2G630489934	55476870	T	0	0.2143	A
10	BICF2P890505	55501297	0	0	0	G

10	BICF2P116818	55521115	0	0	0	A
10	BICF2S23129312	55524241	A	0	0	G
10	BICF2G630489939	55555638	G	0.5	0.2857	A
10	BICF2G630489945	55560490	G	0.5	0.7143	A
10	BICF2P230843	55598563	0	0	0	A
10	BICF2P441979	55604300	0	0	0	A
10	BICF2S23129695	55614199	G	0.5	0.2857	A
10	BICF2G630489971	55631669	A	0.25	0.5714	T
10	BICF2G630489978	55642268	G	0.75	0.4286	A
10	BICF2G630489984	55650369	G	0.75	0.4286	A
10	BICF2G630490019	55669499	C	0	0	A
10	BICF2G630490028	55678470	A	0.25	0.1429	G
10	BICF2S2361874	55684457	0	0	0	A
10	BICF2S23144879	55695814	C	0.75	0.4286	A
10	BICF2G630490046	55712404	C	0	0	A
10	BICF2G630490049	55730747	A	0	0.2143	G
10	BICF2G630490069	55743289	A	0	0.2143	C
10	BICF2G630490085	55755220	A	0	0.2143	G
10	BICF2G630490102	55763840	G	0	0	A
10	BICF2G630490131	55781064	C	0	0.2143	A
10	BICF2G630490139	55789283	G	0	0.2143	A
10	BICF2G630490144	55800066	G	0	0.2143	A
10	BICF2G630490164	55818821	A	0.75	0.6429	G
10	BICF2G630490170	55826474	C	0.25	0.3571	A
10	BICF2G630490177	55835023	A	0.5	0.7143	G
10	BICF2S24115443	55852795	0	0	0	A
10	BICF2S23244106	55873465	0	0	0	G
10	BICF2G630490185	55892034	A	0.25	0.3571	C
10	BICF2P436325	55906721	A	0.75	0.7	C
10	BICF2P983598	55922709	G	0.25	0.3571	A
10	BICF2S23048692	55927376	A	1	1	C
10	BICF2P1419702	55946874	0	0	0	A
10	BICF2S2339273	55949062	G	0	0	A
10	BICF2S2445823	55969467	G	0	0	A
10	BICF2S23912706	55990432	0	0	0	G
10	BICF2S23436453	56006615	A	0	0	T
10	TIGRP2P141781_rs8864441	56061161	0	0	0	A
10	BICF2S23334627	56074492	G	0	0	A
10	BICF2P779624	56086010	0	0	0	G
10	BICF2P1214376	56104322	0	0	0	G
10	BICF2P201206	56112760	A	0	0	G
10	BICF2P602500	56150177	0	0	0	A
10	BICF2S22954384	56156269	A	0.75	0.4286	G
10	BICF2P1186737	56174907	A	0.75	0.6429	G
10	BICF2G630490188	56181921	G	0	0	A
10	BICF2G630490189	56199633	G	0	0.2143	A
10	BICF2P632875	56203108	G	0.25	0.3571	A

10	BICF2G630490204	56218936	A	1	0.7857	G
10	BICF2G630490214	56225847	A	0	0	C
10	BICF2P236193	56243575	A	0	0	G
10	BICF2G630490226	56254773	G	0	0	A
10	BICF2P499017	56260782	G	0	0	A
10	BICF2P255683	56270146	A	0	0	C
10	BICF2G630490230	56288518	G	0	0	A
10	BICF2G630490236	56295051	G	1	0.7857	A
10	BICF2G630490248	56308123	C	0	0	A
10	BICF2S23217554	56316157	A	0	0	G
10	BICF2S2305762	56330674	A	0	0	G
10	BICF2P810530	56342333	C	0	0.2143	A
10	BICF2P773324	56356384	A	0	0	G
10	BICF2G630490256	56358035	G	0	0.2143	A
10	BICF2P58807	56365773	G	0	0.2143	A
10	BICF2G630490265	56375834	G	0	0	A
10	BICF2P441971	56386309	A	0	0.2143	G
10	BICF2P1144785	56397557	A	0	0.2143	G
10	BICF2P117098	56408472	A	0	0	G
10	BICF2P400792	56421983	C	0	0	A
10	BICF2P722164	56439007	G	0	0.2143	A
10	BICF2S2337109	56457443	A	0	0.2143	G
10	BICF2S23726210	56470739	A	0	0	C
10	BICF2S23660298	56483902	G	0	0.2143	A
10	BICF2P901500	56493323	A	0	0	G
10	TIGRP2P141790_rs8973440	56505548	0	0	0	A
10	BICF2G630490272	56517009	A	0	0.2143	G
10	BICF2G630490273	56522514	G	0	0	A
10	BICF2G630490277	56541729	A	0.75	0.6429	G
10	BICF2G630490294	56555388	G	0.75	0.6429	A
10	BICF2G630490306	56563857	A	0	0	G
10	BICF2G630490316	56595025	A	0	0	G
10	BICF2G630490333	56606475	A	0	0	C
10	BICF2S23355436	56622532	C	0	0.2143	A
10	BICF2G630490347	56634077	A	0	0	G
10	BICF2G630490382	56644590	A	0	0	G
10	BICF2G630490391	56650002	C	0	0	A
10	BICF2P499786	56681588	C	1	0.7857	A
10	BICF2G630490426	56685220	A	0	0	G
10	BICF2P426476	56697025	G	0	0	A
10	BICF2P506172	56706726	C	0.25	0.3571	A
10	BICF2G630490482	56719966	A	0	0	C
10	BICF2G630490514	56733658	A	1	0.7857	G
10	BICF2G630490528	56744735	G	0.75	0.6429	A
10	BICF2S23630218	56765708	G	0.75	1	A
10	BICF2P1309422	56801037	A	0.25	0.3571	G
10	BICF2P1399597	56820658	A	0	0	C

10	BICF2S23412822	56825111	A	0	0.2143	C
10	BICF2G630490559	56841869	A	0	0	G
10	BICF2G630490570	56851003	G	0	0	A
10	BICF2S23332046	56867117	G	0	0	A
10	BICF2G630490602	56874515	A	0	0	G
10	BICF2G630490622	56881401	A	0	0	G
10	BICF2G630490649	56891092	A	0	0	G
10	BICF2G630490676	56903014	G	0	0.2143	A
10	BICF2G630490682	56919323	A	0	0	G
10	BICF2G630490693	56925614	A	0	0	G
10	BICF2G630490720	56942501	G	0	0	A
10	BICF2G630490734	56951503	A	0	0.2143	G
10	BICF2G630490757	56959705	G	0	0	A
10	BICF2G630490761	56978845	A	0.25	0.3571	G
10	BICF2P939888	56982895	0	0	0	G
10	BICF2P647892	57005139	A	0.25	0.5714	G
10	BICF2P118721	57019027	G	0.75	0.4286	A
10	BICF2G630490770	57033042	G	0	0.1667	A
10	BICF2G630490783	57046139	A	0.75	0.4286	G
10	BICF2S23413557	57059175	A	0	0	C
10	BICF2G630490814	57065610	G	0	0	A
10	BICF2G630490844	57080281	A	0.25	0.3571	G
10	BICF2G630490864	57089240	A	0.25	0.5714	G
10	BICF2G630490877	57103657	A	0.25	0.5714	C
10	BICF2G630490885	57110786	G	0.25	0.3571	A
10	BICF2G630490893	57121914	A	0	0	G
10	BICF2P1009145	57133688	A	0	0	C
10	BICF2P269981	57144580	A	0	0	G
10	BICF2S23140478	57165095	A	0.25	0.5714	G
10	TIGRP2P141811_rs8579010	57176643	0	0	0	A
10	BICF2G630490901	57181947	A	0	0	C
10	BICF2P823316	57199100	A	0	0	G
10	BICF2G630490912	57204668	C	0	0	A
10	BICF2P716479	57222825	A	0	0	G
10	BICF2G630490923	57230313	G	0	0	A
10	BICF2G630490933	57236177	A	0	0	T
10	BICF2G630490942	57240610	A	0	0	G
10	BICF2G630490968	57266436	C	0	0	A
10	BICF2G630490974	57271096	G	0	0	A
10	BICF2S23048750	57287021	C	0	0	A
10	BICF2P481424	57288133	A	0	0	G
10	BICF2G630490981	57293516	G	0	0	A
10	BICF2G630491024	57314998	C	0	0	A
10	BICF2G630491062	57348154	G	0	0.2143	A
10	BICF2G630491066	57353121	A	0.25	0.1429	G
10	BICF2G630491092	57362025	A	0.25	0.1429	G
10	BICF2G630491119	57383571	A	0.75	0.6429	G

10	BICF2G630491120	57384581	G	0.75	0.6429	C
10	BICF2G630491136	57395358	G	0	0	A
10	BICF2G630491191	57414624	A	1	1	G
10	BICF2G630491222	57436628	A	0	0	G
10	BICF2G630491251	57448166	A	0	0	G
10	BICF2P664339	57461690	G	0	0.2143	A
10	TIGRP2P141822_rs9107462	57475098	0	0	0	G
10	BICF2G630491270	57477164	G	1	0.7857	A
10	BICF2G630491296	57488609	A	0.5	0.5	G
10	BICF2G630491311	57501889	A	0.5	0.5	C
10	BICF2G630491325	57512624	A	0.5	0.5	G
10	BICF2G630491343	57526725	A	0	0	G
10	BICF2G630491354	57534994	A	0.5	0.5	C
10	BICF2G630491388	57568919	G	1	0.7857	A
10	BICF2G630491396	57577664	G	0	0.2143	A
10	BICF2P896395	57586880	G	1	1	A
10	BICF2G630491416	57594268	A	0	0	G
10	BICF2P1118128	57604532	G	0	0	A
10	BICF2G630491467	57624496	G	1	0.7857	A
10	BICF2G630491473	57630035	A	0	0	G
10	BICF2S23112761	57640816	A	0	0	G
10	BICF2P406490	57650543	A	1	1	G
10	BICF2G630491516	57677062	G	0	0.2143	A
10	BICF2G630491543	57685226	G	0	0	A
10	BICF2G630491612	57706780	G	0.5	0.5	A
10	BICF2G630491624	57729486	A	0.5	0.5	C
10	BICF2G630491634	57740971	G	1	0.7857	A
10	BICF2G630491652	57749843	A	0	0.2143	G
10	BICF2P1249817	57759756	A	0.5	0.5	G
10	BICF2G630491658	57772884	G	0.5	0.5	A
10	BICF2G630491688	57800641	C	0.5	0.5	A
10	BICF2G630491701	57811335	G	0.5	0.5	A
10	BICF2P270945	57825605	A	0.5	0.5	G
10	BICF2G630491721	57835151	G	0.5	0.5	A
10	BICF2G630491727	57850227	A	0.5	0.5	G
10	BICF2G630491731	57865340	A	0.5	0.5	G
10	BICF2G630491736	57879171	A	0.5	0.5	G
10	BICF2G630491741	57895083	A	0.5	0.5	G
10	BICF2G630491744	57906064	G	0.75	0.7143	A
10	BICF2G630491746	57923830	A	0.5	0.5	T
10	BICF2P784462	57936636	A	0	0	G
10	BICF2S22922039	57947997	G	0.75	0.75	A
10	BICF2G630491757	57950063	A	0.25	0.2143	G
10	BICF2G630491762	57988897	G	0.25	0.2857	A
10	BICF2G630491764	57996937	G	0	0	A
10	BICF2G630491770	58010316	G	0.25	0.2857	A
10	BICF2G630491778	58018214	A	0	0	G

10	BICF2G630491784	58029905	G	0	0	A
10	BICF2G630491788	58041409	C	0	0	A
10	BICF2G630491796	58071141	A	0	0	G
10	BICF2S23550301	58075689	A	0.25	0.2857	G
10	BICF2G630491801	58088621	G	0	0	A
10	BICF2G630491805	58099816	A	0.5	0.5714	G
10	BICF2G630491811	58110655	T	0	0	A
10	BICF2P1438819	58123912	0	0	0	A
10	BICF2P817400	58138781	0	0	0	A
10	BICF2P491965	58144352	0	0	0	G
10	BICF2S23537914	58173780	C	0.25	0.2857	A
10	BICF2P771477	58186740	0	0	0	A
10	BICF2G630491819	58199458	T	0.5	0.6429	A
10	BICF2G630491824	58202438	G	0.25	0.3571	A
10	BICF2G630491835	58217070	G	0.25	0.2143	A
10	BICF2S23044988	58225158	G	0.25	0.2857	A
10	BICF2G630491871	58238177	A	0.5	0.6429	G
10	BICF2G630491886	58250922	G	0.25	0.3571	A
10	BICF2S23412215	58261160	A	0.25	0.2857	C
10	BICF2G630491908	58275016	A	0.25	0.3571	C
10	BICF2G630491921	58287041	G	0.5	0.3571	A
10	BICF2P664664	58301281	G	0.5	0.3571	C
10	BICF2S23342157	58310092	A	0.25	0.2857	G
10	BICF2G630491939	58317254	A	0.25	0.3571	G
10	BICF2G630491956	58328326	A	0.5	0.6667	G
10	BICF2S23355711	58362341	G	0.25	0.2857	A
10	BICF2P98601	58376546	A	0.5	0.3571	G
10	BICF2P335070	58390182	G	0.5	0.3571	A
10	BICF2G630491964	58406036	G	0	0	A
10	BICF2P750361	58418297	A	0	0	G
10	BICF2P1384236	58424564	C	0	0	A
10	BICF2G630491982	58442674	G	0.5	0.6429	A
10	BICF2G630491987	58443278	G	0.5	0.3571	A
10	BICF2G630492003	58456666	G	0.25	0.2857	A
10	BICF2P593837	58469044	0	0	0	A
10	BICF2G630492004	58484252	A	0.75	0.7143	G
10	BICF2G630492022	58500421	A	0.75	0.7143	G
10	BICF2G630492024	58501054	C	0.25	0.2857	A
10	BICF2G630492039	58520322	A	0.25	0.2857	C
10	BICF2G630492047	58537735	A	0.25	0.2857	G
10	BICF2P1243874	58556734	A	0.75	0.7143	G
10	BICF2P593069	58559157	G	0	0	A
10	BICF2G630492081	58570751	G	0.25	0.2857	A
10	BICF2P1180852	58588953	G	0.25	0.2857	A
10	BICF2G630492103	58589196	A	0	0	C
10	BICF2G630492106	58598270	G	0	0	A
10	BICF2G630492108	58604495	G	0	0	A

10	BICF2G630492116	58620835	G	0.25	0.2857	A
10	BICF2G630492135	58630360	G	0.25	0.2857	A
10	BICF2G630492151	58635986	A	0.25	0.2857	G
10	BICF2G630492157	58641713	A	0	0	G
10	BICF2G630492166	58650185	A	0.25	0.3333	G
10	BICF2G630492187	58667130	A	0.25	0.2857	G
10	BICF2P1429399	58673713	A	0.25	0.2857	G
10	BICF2G630492206	58680393	A	0.25	0.2857	G
10	BICF2G630492217	58688936	C	0	0	A
10	BICF2G630492218	58690740	C	0	0	A
10	BICF2G630492221	58697114	A	0	0	G
10	BICF2G630492235	58709607	A	0	0	G
10	BICF2G630492278	58731785	A	0	0	G
10	BICF2G630492301	58744606	A	0.25	0.2857	G
10	BICF2P342565	58769300	0	0	0	A
10	TIGRP2P141867_rs9062868	58783898	0	0	0	G
10	BICF2G630492307	58833108	A	0	0	G
10	BICF2P929637	58841564	0	0	0	A
10	BICF2G630492310	58871018	G	0.25	0.2857	A
10	BICF2G630492313	58883680	A	0.25	0.2857	G
10	BICF2P6814	58900211	A	0.75	0.7143	T
10	BICF2S23328929	58905045	G	0.75	0.7143	A
10	BICF2S23521975	58923672	A	0.25	0.2857	G
10	BICF2P817210	58927703	G	0	0	A
10	BICF2P451531	58938521	A	0	0	G
10	BICF2P30031	58950540	A	0	0	G
10	BICF2P1237253	58963489	C	0	0	G
10	BICF2P734829	58988272	0	0	0	A
10	BICF2P1121326	58999172	A	0.75	0.7143	G
10	BICF2G630492348	59009283	A	1	1	G
10	BICF2G630492379	59034638	A	0.5	0.5714	G
10	BICF2G630492383	59045568	G	0	0	A
10	BICF2G630492393	59060586	G	0	0	A
10	BICF2S23552704	59068238	0	0	0	C
10	BICF2G630492403	59081099	G	0.5	0.5714	A
10	BICF2G630492424	59096143	G	0.5	0.5714	A
10	BICF2G630492428	59099312	A	0.5	0.5714	G
10	BICF2G630492451	59117214	A	0.5	0.5714	G
10	BICF2G630492463	59129371	G	0.5	0.5714	A
10	BICF2G630492481	59139779	G	0	0	A
10	BICF2G630492493	59145612	C	0	0	A
10	BICF2P1103044	59156964	G	0.25	0.2857	A
10	BICF2G630492526	59176850	A	0	0	G
10	BICF2G630492527	59180359	A	0	0	C
10	BICF2G630492535	59193460	G	0	0	A
10	BICF2P302806	59210480	A	0.5	0.5714	C
10	BICF2S22942869	59217472	A	0.5	0.5714	G

10	BICF2P1443299	59225274	A	0.5	0.5714	G
10	BICF2P871346	59247563	0	0	0	A
10	BICF2P937713	59265374	A	0.5	0.4286	G
10	BICF2P1133063	59280328	A	0.25	0.2857	C
10	BICF2S23441191	59282920	A	0.5	0.5714	G
10	BICF2P44198	59305963	A	0.25	0.1429	C
10	BICF2P1113481	59329957	A	0	0	G
10	BICF2P1049158	59340953	A	0	0	C
10	BICF2P249846	59355412	G	0.5	0.5714	A
10	BICF2P1418961	59368167	A	0	0	G
10	BICF2P118130	59376505	G	0	0	A
10	BICF2S22920458	59391859	A	0	0	G
10	BICF2P1219271	59400315	A	0.25	0.2857	G
10	BICF2P1059030	59413430	A	0.25	0.2857	C
10	BICF2P703500	59420631	G	0.25	0.2857	A
10	BICF2P191987	59426694	A	0.25	0.2857	G
10	BICF2P510199	59434783	G	0	0	A
10	BICF2P1032819	59448449	G	0	0	A
10	BICF2P559445	59455583	A	0	0	C
10	BICF2S23343825	59476642	A	0	0	G
10	BICF2P1265074	59524313	G	0.5	0.4286	A
10	BICF2P1115469	59536935	0	0	0	G
10	BICF2S23140067	59547687	A	0.5	0.4286	C
10	BICF2S23229873	59568369	G	0.5	0.4286	A
10	BICF2P271194	59571633	A	0.5	0.4286	G
10	BICF2P284540	59592516	0	0	0	A
10	BICF2P632499	59600853	0	0	0	A
10	BICF2P388799	59614818	A	0.5	0.4286	G
10	BICF2P1124607	59617984	G	0	0	A
10	BICF2P308051	59628196	A	0	0	G
10	BICF2P488444	59643503	A	0	0	G
10	BICF2P177374	59653166	A	0	0	G
10	BICF2S23130185	59670840	T	0.5	0.4286	A
10	BICF2P971531	59681831	C	0.5	0.4286	A
10	BICF2P1267670	59691412	G	0.5	0.5	A
10	BICF2P194647	59725550	A	0.25	0.1429	G
10	BICF2P924544	59731609	A	0	0	G
10	BICF2P465084	59740614	A	0	0	G
10	BICF2P1310973	59749107	G	NA	0.5	A
10	BICF2S23148142	59769073	A	0.5	0.5714	G
10	BICF2P233551	59778126	A	0.25	0.1429	G
10	BICF2P788947	59796849	A	0	0	G
10	BICF2P184479	59801268	A	0.25	0.1429	G
10	BICF2S23052804	59817490	A	0	0	G
10	BICF2P566462	59846011	A	0.5	0.4286	G
10	BICF2P1096664	59853625	G	0.25	0.2857	A
10	BICF2S23220227	59870049	C	0.25	0.2857	A

10	BICF2S23155665	59891933	A	0.75	0.7143	G
10	BICF2P249878	59903312	A	0.5	0.4286	C
10	BICF2P569714	59912518	G	0.25	0.2857	A
10	BICF2P285843	59915184	A	0	0	G
10	BICF2P356295	59935591	C	0	0	G
10	BICF2P1411839	59948159	A	0.75	0.7143	G
10	BICF2P1411833	59950793	A	0	0	G
10	BICF2P1058095	59969396	G	0.75	0.8	A
10	BICF2P1445407	59982992	G	0.75	0.75	A
10	BICF2P1445405	59983859	T	0.75	0.7143	A
10	BICF2P640724	59984676	A	0.75	0.7143	G
10	BICF2S23326734	60001269	A	0.25	0.1429	G
10	BICF2P705846	60013840	G	0.75	0.8571	A
10	BICF2P1329172	60028156	A	0.5	0.5714	G
10	BICF2S235965	60034691	0	0	0	G
10	TIGRP2P141890_rs8784107	60052438	0	0	0	G
10	BICF2P368947	60070786	0	0	0	A
10	BICF2S23026439	60079872	A	0.5	0.5714	T
10	BICF2P209236	60089886	0	0	0	C
10	BICF2P338235	60101001	A	0.75	0.8571	C
10	BICF2P824387	60113642	A	0.75	0.8571	G
10	BICF2P939795	60123682	G	0	0	A
10	BICF2P347211	60157738	0	0	0	A
10	BICF2S23547546	60184457	G	0	0	A
10	BICF2S23513579	60206370	A	1	1	G
10	BICF2P1015852	60232142	G	1	1	A
10	BICF2S237368	60240301	G	0.25	0.1429	A
10	BICF2P949743	60257668	0	0	0	A
10	BICF2P1125692	60271487	0	0	0	A
10	BICF2P903947	60304159	C	0	0	A
10	BICF2P1424415	60333397	C	0.25	0.1429	A
10	BICF2P493344	60349763	A	0	0	G
10	BICF2P76247	60355103	C	0.25	0.1429	A
10	BICF2P269358	60371049	C	0	0	A
10	BICF2S23432259	60386377	G	0	0	A
10	BICF2P216368	60387242	C	0	0	G
10	BICF2S23438608	60399808	G	0.25	0.1429	A
10	BICF2P342852	60415501	G	0.25	0.1429	A
10	BICF2P1368107	60421330	G	0	0	A
10	BICF2S23315433	60456532	A	0	0	G
10	BICF2S23426508	60476168	G	0	0	C
10	TIGRP2P141895_rs8715676	60488079	0	0	0	A
10	BICF2P955954	60504906	0	0	0	A
10	BICF2S22941762	60513054	G	0	0	A
10	BICF2S22962407	60530455	G	0	0	A
10	BICF2S23110665	60567960	A	0	0	G
10	BICF2P383458	60574693	0	0	0	C

10	BICF2P323975	60621515	A	0	0	C
10	BICF2S2346750	60674403	G	0.75	0.8571	A
10	BICF2P798823	60692450	0	0	0	A
10	BICF2P1032425	60705018	A	0	0	T
10	BICF2P673189	60739437	A	1	1	G
10	BICF2S22936876	60763302	G	0	0	A
10	BICF2P1384039	60770352	G	0.75	0.8571	A
10	BICF2P195903	60789727	A	0.75	0.8571	G
10	BICF2P839127	60808346	G	0.75	0.8571	A
10	BICF2P304794	60816151	A	0.75	0.8571	C
10	BICF2S22957331	60827926	0	0	0	C
10	BICF2P1180115	60843906	0	0	0	G
10	BICF2S23512754	60850636	G	0.25	0.1429	A
10	BICF2S23322059	60861972	G	0.25	0.1429	C
10	BICF2S23333629	60879410	A	0.25	0.1429	G
10	BICF2P114700	60892466	0	0	0	A
10	BICF2S2364198	60923087	A	1	1	G
10	BICF2P822629	60928007	G	1	1	A
10	BICF2P1180307	60939742	G	1	1	A
10	BICF2P1135023	60951568	A	0	0	G
10	BICF2S23452001	60963119	G	0	0	A
10	BICF2S23315519	60974590	G	0	0	A
10	BICF2P322412	60990872	0	0	0	A
10	BICF2S2366163	61000220	A	0.75	0.8571	G
10	BICF2P1092634	61009458	G	0	0	A
10	BICF2S23137618	61029461	G	1	1	A
10	BICF2P1370043	61040745	A	0	0	G
10	BICF2P200400	61054834	A	0	0	G
10	BICF2P667208	61067454	G	1	1	A
10	BICF2P217147	61087064	0	0	0	A
10	BICF2S23038456	61089680	A	0	0	G
10	BICF2P963969	61102510	A	0.25	0.1429	G
10	BICF2P1315790	61114427	A	0.25	0.1429	G
10	BICF2P780308	61133677	G	1	1	A
10	BICF2P1287702	61138682	A	0	0	G
10	BICF2P602338	61146139	G	1	1	A
10	BICF2P989259	61166355	A	1	1	G
10	BICF2P885601	61176672	0	0	0	A
10	BICF2P620024	61182320	A	0	0	C
10	BICF2P689842	61213918	A	0	0	G
10	BICF2P447041	61217475	A	0.75	0.8571	G
10	BICF2P676575	61247087	C	1	0.8571	A
10	BICF2P157574	61254217	G	1	1	A
10	BICF2P1132919	61270872	G	1	1	A
10	BICF2P877345	61280357	0	0	0	A
10	BICF2P409743	61288767	G	0.25	0.1429	A
10	BICF2P56217	61297572	A	0.25	0.1429	G

10	BICF2S23752799	61318742	G	0	0	A
10	BICF2P1206583	61331614	A	0	0	G
10	BICF2P114573	61337953	G	0	0	A
10	BICF2P257923	61341437	G	0	0	A
10	BICF2S22942051	61344927	G	0.75	0.9286	A
10	BICF2P884502	61355044	A	1	0.9286	C
10	BICF2P1067630	61376821	A	0	0	G
10	BICF2S23325450	61392681	G	0.75	0.9286	A
10	BICF2P1274933	61402302	A	0	0	G
10	BICF2P281193	61416573	G	0.75	0.9286	A
10	BICF2P1390376	61423338	G	1	0.9286	A
10	BICF2P1244041	61438911	A	0.75	0.9286	G
10	BICF2P1350421	61445633	G	0.75	0.9286	A
10	BICF2P277574	61459238	G	0.75	0.9286	A
10	BICF2P1310110	61469898	G	0	0	A
10	BICF2P77977	61486199	0	0	0	A
10	BICF2P871681	61498214	A	0.25	0.07143	G
10	BICF2P825934	61502746	C	0.75	0.9286	A
10	BICF2P556582	61520100	G	0	0	A
10	BICF2P180182	61534567	G	0	0	A
10	BICF2P1456154	61541606	A	0.75	0.9286	C
10	BICF2P1373615	61566611	A	0.75	0.9286	G
10	BICF2P864923	61574601	G	0.75	0.9286	A
10	BICF2P202673	61586449	A	1	1	C
10	BICF2S23554378	61594335	G	0.25	0.07143	A
10	BICF2P901889	61622375	C	0.25	0.07143	A
10	BICF2P486834	61642076	G	0.25	0.07143	A
10	BICF2S23520070	61655861	G	0	0	A
10	BICF2P826929	61666519	C	0.75	0.9286	A
10	BICF2P190979	61695262	0	0	0	G
10	BICF2S23536384	61701014	G	0.75	0.9286	A
10	BICF2P564567	61715028	A	0.75	0.9286	G
10	BICF2S22954327	61722313	T	0.75	0.9286	A
10	BICF2P344630	61735666	0	0	0	A
10	BICF2P154999	61759033	G	0.75	0.9286	A
10	BICF2P3673	61780896	G	0	0	A
10	BICF2P763826	61794980	G	0.25	0.07143	A
10	BICF2S237142	61803966	0	0	0	A
10	BICF2S23119242	61814336	G	0.25	0.07143	A
10	BICF2S23545618	61830526	A	0	0	C
10	BICF2S23022674	61838885	G	0.25	0.07143	A
10	BICF2P419288	61858412	A	0	0	G
10	BICF2S23060690	61866956	A	1	1	G
10	BICF2P538011	61878046	G	0	0	A
10	BICF2P775688	61887367	0	0	0	A
10	BICF2S23517127	61914795	G	1	1	A
10	BICF2S2314027	61927759	A	0	0	G

10	BICF2S23426365	61941743	A	0.75	0.9286	C
10	BICF2P1242609	61954703	A	0	0	G
10	BICF2P602846	61962487	A	0	0	G
10	BICF2P1215154	61977066	A	0	0	G
10	BICF2P729624	61995287	G	0	0	A
10	BICF2P1094001	62001973	A	0	0	G
10	BICF2P989551	62008407	G	0	0	A
10	BICF2P471410	62019745	A	0	0	G
10	BICF2P1005445	62038950	A	0	0	G
10	BICF2P669988	62052622	A	0	0	G
10	BICF2P293287	62061877	A	0.25	0.07143	G
10	BICF2P854199	62076872	A	0	0	G
10	BICF2P812977	62094182	0	0	0	C
10	BICF2P56391	62103540	A	0.25	0.07143	G
10	BICF2P171592	62115508	0	0	0	A
10	BICF2P528031	62123233	G	0.25	0.07143	A
10	BICF2P1061263	62140575	0	0	0	C
10	BICF2S23157935	62149220	G	0.25	0.07143	A
10	BICF2P1183525	62158483	0	0	0	A
10	BICF2S2312442	62172392	G	0.25	0.07143	A
10	BICF2P449768	62185256	0	0	0	G
10	BICF2S22945216	62197260	A	0.25	0.07143	G
10	BICF2P704267	62206038	0	0	0	A
10	BICF2S23525023	62229693	0	0	0	G
10	BICF2P499864	62238131	G	0.25	0.07143	A
10	BICF2P1114492	62254692	A	1	1	G
10	BICF2P710267	62266375	G	1	1	A
10	BICF2P536324	62280321	A	0.25	0.07143	G
10	BICF2P11606	62288672	G	1	1	A
10	BICF2P709954	62297315	A	0	0	G
10	BICF2S23259009	62312419	A	0	0	G
10	BICF2S23737269	62329284	G	0.75	0.9286	A
10	BICF2P1195405	62335167	A	0	0	G
10	BICF2P103551	62350808	G	0	0	C
10	BICF2S23017965	62361817	A	0.75	0.9286	G
10	BICF2P552585	62381168	A	0.25	0.07143	G
10	BICF2P576356	62387870	A	0.25	0.07143	G
10	BICF2S2339076	62400139	0	0	0	A
10	BICF2S23714390	62430040	0	0	0	A
10	BICF2P13034	62435990	A	0.75	0.9286	G
10	BICF2S23752183	62455536	A	0.75	0.9286	G
10	BICF2P788627	62465701	G	0.25	0.07143	A
10	BICF2P528613	62472085	G	1	1	A
10	BICF2S23550325	62488946	G	0.75	0.9286	A
10	BICF2S23538026	62501024	G	1	1	A
10	BICF2S23050968	62511561	G	0	0	A
10	BICF2P1342964	62520032	A	0	0	G

10	BICF2P509550	62526738	A	1	1	G
10	BICF2S23349937	62542700	0	0	0	G
10	BICF2P266654	62553016	A	0	0	G
10	BICF2S23058165	62557307	A	0	0	G
10	BICF2P974373	62562585	A	1	1	T
10	BICF2P670604	62572884	0	0	0	C
10	BICF2P689210	62587856	A	0	0	G
10	BICF2S2416924	62594639	G	0.5	0.5	A
10	BICF2P805358	62594881	A	0	0	G
10	BICF2P805344	62597593	A	0	0	G
10	BICF2P725442	62613031	A	0.25	0.07143	G
10	BICF2P243374	62619695	A	0.5	0.5	G
10	BICF2S23534053	62629612	A	0	0	G
10	BICF2P288085	62645935	A	0	0	G
10	TIGRP2P141924_rs9172925	62656688	0	0	0	G
10	BICF2P749405	62686051	0	0	0	T
10	BICF2P833588	62689442	0	0	0	G
10	BICF2S2309846	62708929	C	0.5	0.3571	A
10	BICF2S22933661	62726601	A	0	0	C
10	BICF2P1324538	62734011	T	0	0	A
10	G613f38S204	62799364	G	0	0	A
10	TIGRP2P141929_rs8992288	62819575	0	0	0	C
10	BICF2S23146473	62825949	G	0	0	A
10	BICF2P448896	62838193	0	0	0	A
10	BICF2P450001	62867448	0	0	0	C
10	BICF2P580757	62884482	A	0	0	C
10	BICF2P263991	62887772	A	0	0	G
10	BICF2S23622289	62905694	G	0	0	A
10	BICF2P175788	62916778	A	0	0	G
10	BICF2S2304896	62934137	G	0	0	A
10	BICF2P505540	62941836	A	0.25	0.2143	G
10	BICF2P803196	62951390	A	0	0.2143	G
10	BICF2P1177857	62964153	A	0.5	0.3571	C
10	BICF2P409830	62985184	C	0.5	0.3333	A
10	BICF2P1208844	62996439	C	0.5	0.3571	A
10	BICF2P1433825	62999439	0	0	0	A
10	BICF2P190659	63015292	0	0	0	G
10	BICF2P126731	63028470	A	0	0	G
10	BICF2S23130129	63033467	A	0	0	G
10	BICF2P1155464	63046301	A	0.5	0.3571	G
10	BICF2P1045625	63057138	G	0	0	A
10	BICF2P1045624	63057561	A	0.5	0.3571	G
10	BICF2S22939962	63070785	G	0	0	A
10	BICF2S2312153	63079450	G	0.25	0.4286	A
10	BICF2P362736	63093057	G	0	0	A
10	BICF2P1321141	63108981	A	0	0	G
10	BICF2P1075652	63114167	G	0.75	0.5714	A

10	BICF2P873686	63126598	A	1	0.5714	G
10	BICF2P1377892	63145599	G	0.25	0.2143	A
10	BICF2S2356471	63153499	G	0	0	A
10	BICF2P1349687	63170807	G	0	0	A
10	BICF2P1239039	63190406	A	0	0	G
10	BICF2P246143	63192697	A	0	0	G
10	BICF2P839523	63206763	A	0.5	0.6429	G
10	BICF2P651097	63222315	G	0	0	A
10	BICF2P1106052	63228480	A	0	0	G
10	BICF2P366020	63266214	A	0	0	G
10	BICF2S23523758	63277450	A	0.5	0.3571	C
10	BICF2P969746	63288343	A	0.5	0.6429	G
10	BICF2P303962	63300105	A	0.5	0.3571	G
10	BICF2P693725	63309616	A	0.75	0.7857	G
10	BICF2P245367	63337957	A	0	0	C
10	BICF2P516553	63352884	0	0	0	C
10	BICF2P1236841	63359165	0	0	0	A
10	BICF2P838826	63374847	0	0	0	C
10	TIGRP2P141934_rs9137749	63387667	0	0	0	C
10	BICF2P281100	63388227	0	0	0	A
10	BICF2S23243834	63410384	0	0	0	G
10	BICF2P708667	63416225	G	0.5	0.6429	A
10	TIGRP2P141935_rs8859222	63428044	0	0	0	A
10	BICF2S23027006	63451235	A	0.5	0.3571	T
10	BICF2P313811	63457980	A	0.25	0.2143	G
10	BICF2P884203	63502904	0	0	0	G
10	BICF2P954365	63514320	0	0	0	G
10	TIGRP2P141937_rs8883973	63541244	0	0	0	G
10	BICF2P826311	63556420	A	0.25	0.2143	G
10	BICF2P1412890	63561112	0	0	0	G
10	BICF2P102214	63575940	0	0	0	A
10	TIGRP2P141938_rs8715718	63606940	0	0	0	A
10	BICF2P1204793	63681449	0	0	0	C
10	TIGRP2P141942_rs9095634	63701784	0	0	0	C
10	BICF2S23147404	63731738	G	0.25	0.4286	C
10	BICF2S23352020	63740369	G	0	0	A
10	BICF2P406665	63755697	A	0.25	0.4286	G
10	BICF2S23759973	63762414	A	0.25	0.2143	T
10	BICF2P883114	63767706	G	0	0	A
10	BICF2P871442	63786358	G	0.25	0.2143	A
10	BICF2P1290902	63811522	A	0.5	0.3571	C
10	BICF2P233006	63814404	G	0.25	0.2143	A
10	BICF2S23751386	63828906	A	0	0	G
10	TIGRP2P141945_rs9121589	63842961	0	0	0	C
10	BICF2P1057357	63851597	0	0	0	A
10	BICF2S23250878	63863630	A	0	0	G
10	BICF2P609264	63881490	A	0	0	G

10	BICF2S23140895	63891522	A	0	0	G
10	BICF2S23430811	63896578	A	0	0	G
10	BICF2S23332231	63912428	A	0.25	0.2143	G
10	TIGRP2P141946_rs9220412	63927682	0	0	0	A
10	BICF2S23319032	63928850	G	0.5	0.3571	A
10	BICF2S23036697	63940162	A	0	0	G
10	BICF2P667276	63958043	A	0	0	C
10	BICF2P298235	63964924	A	0	0	G
10	BICF2S23354787	63989984	A	0	0	T
10	BICF2S23126279	63992813	A	0	0	C
10	BICF2P233343	64005842	A	0.75	0.5714	G
10	BICF2P572902	64023984	0	0	0	G
10	BICF2P1163634	64047187	G	0	0	A
10	BICF2P1014884	64070127	G	0.75	0.5714	A
10	BICF2P993724	64089210	0	0	0	G
10	BICF2S22953011	64092374	A	0.5	0.3571	G
10	BICF2P240405	64102544	A	0	0	C
10	BICF2P1140675	64127179	G	0	0	A
10	BICF2P46466	64135761	A	0	0	C
10	BICF2S2419687	64155748	G	0.25	0.4286	A
10	BICF2P831347	64164572	G	0.25	0.4286	A
10	BICF2P831346	64164763	0	0	0	G
10	BICF2P1263316	64180437	A	0.5	0.6429	G
10	BICF2P903213	64191057	G	0.25	0.2143	A
10	BICF2P740157	64197051	0	0	0	G
10	BICF2P1322426	64219309	A	0	0	G
10	BICF2P720310	64228751	A	0	0	G
10	BICF2S23417780	64246141	A	0.5	0.3571	G
10	BICF2P1031533	64251818	C	0.25	0.4286	A
10	BICF2S23520126	64288769	A	0	0	G
10	BICF2P1071598	64302966	0	0	0	A
10	BICF2P1085256	64312701	0	0	0	A
10	BICF2P793868	64338938	0	0	0	A
10	BICF2P396608	64345708	0	0	0	A
10	BICF2S2357810	64374696	C	0	0	A
10	BICF2S23629952	64380099	A	0	0	G
10	BICF2P407581	64392762	0	0	0	A
10	BICF2S23344347	64406684	A	0	0	C
10	BICF2P1412124	64413467	A	0.25	0.4286	G
10	BICF2P1140670	64426203	C	0.5	0.6429	A
10	BICF2P113421	64466685	G	0.5	0.6429	A
10	BICF2P149407	64477123	0	0	0	A
10	BICF2P172081	64484378	A	0	0	C
10	BICF2P492593	64512298	C	0.5	0.6429	A
10	BICF2S23144987	64524584	G	0.25	0.4286	A
10	BICF2P1310267	64532235	A	0.5	0.6429	G
10	BICF2P156225	64539642	A	0.25	0.4286	G

10	BICF2P1464859	64550628	A	0.25	0.4286	G
10	BICF2P147162	64561411	0	0	0	G
10	BICF2P1235499	64573625	A	0	0.4167	G
10	BICF2P1350758	64605215	0	0	0	G
10	BICF2P576559	64629526	A	0.5	0.6429	C
10	BICF2P115480	64630259	C	0.25	0.4286	A
10	BICF2P836617	64649074	C	0.5	0.6429	A
10	BICF2P140529	64660895	G	0.25	0.4167	A
10	TIGRP2P141960_rs8647390	64665794	0	0	0	A
10	BICF2P20411	64693020	A	0	0.2143	G
10	BICF2P249414	64708495	G	0.25	0.2143	A
10	BICF2P1237960	64715653	G	0.25	0.2143	A
10	BICF2P950139	64735502	0	0	0	C
10	BICF2P154539	64749485	G	0.25	0.2143	A
10	BICF2P282216	64758538	A	0.25	0.2143	G
10	BICF2S22933323	64774204	A	0	0	G
10	BICF2P86686	64781455	A	0.5	0.3571	G
10	BICF2P1205980	64795710	A	0.25	0.4286	G
10	BICF2P81653	64803636	A	0	0	G
10	BICF2P67520	64843647	0	0	0	G
10	TIGRP2P141963_rs8714161	64864576	0	0	0	G
10	BICF2S23348561	64892535	C	0	0	G
10	BICF2S24310295	64931987	A	0	0	G
10	BICF2P920271	64942771	A	0	0	G
10	BICF2P1278784	64956435	A	0.75	0.7857	G
10	BICF2P203418	64968237	G	0	0	A
10	BICF2P562624	64980485	0	0	0	G
10	BICF2P753850	64990335	T	0	0	A
10	BICF2S23717810	65006612	C	0	0	A
10	BICF2P783256	65023208	A	0	0	G
10	BICF2P977085	65042098	G	0	0	A
10	BICF2P625967	65047487	G	0	0	A
10	BICF2P202302	65059277	G	0	0	A
10	BICF2P1410418	65064896	A	0	0	G
10	BICF2P424885	65068693	A	0	0	T
10	BICF2P496371	65089416	A	0	0	G
10	BICF2S23529008	65100396	A	0	0	G
10	BICF2P910926	65142039	C	0	0	A
10	BICF2S2376500	65161142	A	0	0	G
10	BICF2P836133	65186944	0	NA	0	G
10	BICF2S23642683	65209901	G	0	0	A
10	BICF2S23255126	65225959	G	0	0	A
10	BICF2S23624241	65230563	A	0	0	C
10	BICF2S235304	65243918	A	0	0	G
10	BICF2P107806	65251772	A	0	0	G
10	BICF2P1097881	65262845	C	0	0	G
10	BICF2P864182	65282831	A	0	0	G

10	BICF2P132840	65286567	A	0	0	G
10	BICF2P666262	65297204	0	0	0	A
10	BICF2P99974	65318848	A	0.75	0.5714	G
10	BICF2P945349	65327276	G	0	0	A
10	BICF2P1306344	65334245	A	0	0	G
10	BICF2P401973	65344772	A	0.25	0.4286	G
10	BICF2P1005657	65359322	A	0.25	0.4286	G
10	BICF2P1420240	65366635	G	0.25	0.3333	A
10	BICF2S23623302	65393869	A	0.75	0.7857	G
10	BICF2S23623994	65418869	A	0	0	G
10	BICF2P885286	65431516	A	0.5	0.3571	G
10	BICF2P725846	65436189	0	0	0	A
10	BICF2S2412927	65447999	G	0	0	A
10	BICF2P365368	65458754	C	1	1	A
10	BICF2P1269878	65461608	A	0	0	C
10	BICF2P491944	65480823	A	0.25	0.2143	G
10	BICF2S23729762	65491085	G	0	0	A
10	BICF2S23763177	65493060	A	0.25	0.4286	G
10	BICF2P923590	65510914	A	0.5	0.6429	C
10	BICF2P298375	65535575	0	0	0	G
10	BICF2S23147444	65549779	A	0	0	G
10	BICF2S23648387	65569279	A	0.25	0.2143	C
10	BICF2S23534797	65578773	A	0.5	0.3571	G
10	BICF2S22936981	65615556	C	0.25	0.4286	A
10	BICF2S23649701	65620990	A	0	0	G
10	BICF2P1143451	65638950	0	0	0	A
10	BICF2P671940	65654565	A	0	0	G
10	BICF2P1257167	65668705	G	0.25	0.4286	A
10	BICF2P1029670	65695950	G	0.25	0.4286	A
10	BICF2P1259294	65708418	A	0.25	0.2143	C
10	BICF2P904177	65752166	A	0.25	0.4286	G
10	BICF2P893025	65788849	A	0.25	0.4286	G
10	BICF2P791777	65794514	G	0.25	0.4286	A
10	BICF2P1144616	65813397	0	0	0	A
10	BICF2P350455	65827319	0	0	0	C
10	BICF2P1238698	65846335	G	0	0.4286	A
10	BICF2P1015704	65868000	G	0.75	0.7857	A
10	BICF2P237731	65878116	A	0.25	0.4286	G
10	BICF2P293836	65897633	0	0	0	A
10	BICF2P1021241	65908818	0	0	0	A
10	BICF2P1363949	65932134	G	0.25	0.2143	A
10	BICF2P136377	65943806	A	0.75	0.7857	G
10	BICF2P497221	65955312	G	0.75	0.7857	A
10	BICF2P221274	65970655	A	0.75	0.9	G
10	BICF2S23252447	65997715	G	0.75	0.7857	A
10	BICF2P538838	66014522	A	0.5	0.6429	G
10	BICF2P858466	66055746	A	1	1	G

10	BICF2P772937	66062942	A	0	0	C
10	BICF2P145395	66079289	G	0.25	0.4286	A
10	BICF2P328179	66108993	A	0.25	0.4167	G
10	BICF2P1222884	66127687	A	0.75	0.7857	G
10	BICF2S2393496	66145428	0	0	0	A
10	BICF2P284083	66148745	T	0.75	0.7857	A
10	BICF2P960444	66164148	G	0.75	0.7857	A
10	BICF2S23125279	66247778	A	0.5	0.6429	G
10	BICF2P716590	66282150	0	0	0	G
10	BICF2S2316011	66308890	C	0.5	0.3571	A
10	BICF2S23237843	66312758	A	0.5	0.3571	T
10	BICF2P863351	66323026	G	0.25	0.4286	A
10	BICF2P37110	66337249	A	0.5	0.6429	G
10	BICF2P253659	66346803	A	0.5	0.6429	G
10	BICF2S23045651	66368522	C	0.5	0.3571	A
10	BICF2P465304	66381184	A	0.75	0.5714	G
10	BICF2S23117831	66397820	A	0.5	0.3571	G
10	BICF2S2459921	66406558	0	0	0	A
10	BICF2P803329	66422090	0	0	0	A
10	BICF2P428105	66437636	C	0.25	0.4286	A
10	BICF2S23047668	66468306	A	0	0	G
10	BICF2P300478	66470296	A	0.5	0.6667	C
10	BICF2P484572	66491789	A	0	0	G
10	BICF2P1323493	66507839	A	0	0	C
10	BICF2S2307824	66518743	A	0	0	T
10	BICF2P1364780	66528809	G	0.5	0.6429	A
10	BICF2P1202	66549437	A	0.5	0.6429	G
10	BICF2S23048617	66551874	A	1	1	C
10	BICF2S2303703	66562182	A	0.5	0.3571	G
10	BICF2S22934298	66574366	C	0.5	0.3571	A
10	BICF2P657834	66586397	0	0	0	C
10	BICF2S23146267	66606742	A	0.5	0.6429	C
10	BICF2S22931532	66608939	A	0.5	0.6667	C
10	BICF2P373390	66637311	A	0.5	0.6429	C
10	BICF2P1367936	66652397	A	0.5	0.6429	C
10	BICF2P711413	66656760	G	0.5	0.6429	C
10	BICF2P538827	66672305	A	0.5	0.6667	G
10	BICF2P92588	66678866	A	0.5	0.6429	G
10	BICF2P410774	66691114	G	0.25	0.2143	A
10	BICF2P242695	66703272	G	0.25	0.2143	A
10	BICF2P1083795	66718590	G	0.25	0.2143	A
10	BICF2P919415	66731359	A	0.25	0.2143	G
10	BICF2S23422025	66738024	G	0.25	0.2143	A
10	BICF2P260073	66752360	A	0.75	0.7857	G
10	BICF2P1201684	66762626	G	0.25	0.2143	A
10	BICF2P1232144	66770958	G	0	0.1667	A
10	BICF2P926226	66787521	G	0.25	0.2143	A

10	BICF2P1149839	66794420	G	0.25	0.1667	A
10	BICF2P264719	66804956	A	0.25	0.2143	G
10	BICF2P215505	66851050	G	0	0	A
10	BICF2P1362589	66865552	G	0	0	A
10	BICF2P758662	66873121	0	0	0	A
10	TIGRP2P141994_rs9192630	66893387	0	0	0	A
10	BICF2P799375	66902872	A	0	0	C
10	BICF2P159108	66907478	A	0	0	T
10	BICF2P1458360	66928516	G	0	0	A
10	BICF2P1210926	66945341	G	0.25	0.3571	A
10	BICF2P952681	66960719	A	0.5	0.5714	G
10	BICF2P307939	66973417	A	0.25	0.3571	G
10	BICF2P1334283	66988434	0	0	0	C
10	BICF2P760297	67009699	A	0.25	0.3571	G
10	BICF2P1039039	67011247	A	0.25	0.3333	G
10	BICF2P1088514	67022843	A	0.25	0.3571	G
10	BICF2P540685	67033712	A	0.25	0.3571	C
10	BICF2P64524	67055226	C	0.25	0.3571	A
10	BICF2P111414	67061245	A	0.25	0.3571	G
10	BICF2P985197	67064223	G	0.25	0.3571	A
10	BICF2S23754732	67069144	0	0	0	G
10	BICF2P742809	67084837	0	0	0	A
10	BICF2S23428862	67094853	A	0.25	0.3571	C
10	BICF2P693277	67103993	C	0	0	A
10	BICF2P1197181	67113618	A	0	0	G
10	BICF2P15376	67114562	A	0.5	0.5714	G
10	BICF2P318406	67129157	C	0	0	A
10	BICF2S23416849	67144682	C	0	0	A
10	BICF2S23032971	67170094	A	0	0	G
10	BICF2P168751	67179972	G	0.25	0.3571	A
10	BICF2P321202	67198208	A	0.5	0.4286	G
10	BICF2S23349532	67209545	C	0.25	0.3571	A
10	BICF2S23053109	67219189	A	0.25	0.1	G
10	BICF2S23238533	67239149	A	0.25	0.2143	G
10	BICF2P1410377	67240594	G	0	0	A
10	BICF2S2373178	67256449	C	0.75	0.6429	A
10	BICF2S23342136	67274729	0	0	0	C
10	BICF2P569042	67281924	0	0	0	A
10	BICF2P1258359	67296982	0	0	0	G
10	BICF2P818558	67322388	A	0.25	0.3571	G
10	BICF2S23252904	67340116	A	0.5	0.4286	G
10	BICF2P1339694	67350696	A	0	0	G
10	BICF2P607913	67358179	A	0	0	G
10	BICF2S23741741	67369458	A	0.25	0.3571	G
10	BICF2S23522374	67387024	A	0	0	G
10	BICF2P227986	67402622	0	0	0	G
10	BICF2S23336950	67435887	G	0	0	A

10	BICF2P1049132	67442175	A	0	0	G
10	BICF2P1050346	67450546	A	0	0	C
10	BICF2P233161	67463699	G	1	0.6667	A
10	BICF2P1298279	67474760	G	0.75	0.6429	A
10	BICF2P1413241	67483518	A	0	0	G
10	BICF2P512899	67497312	G	0	0	A
10	BICF2P268930	67514763	0	0	0	C
10	BICF2S23315090	67550123	G	0	0	A
10	BICF2P1351728	67561930	0	0	0	G
10	BICF2S23622913	67563585	A	0.25	0.25	C
10	BICF2P359014	67574241	G	0.75	0.7857	A
10	BICF2P800213	67591260	G	0	0	A
10	BICF2S23550668	67599867	A	0	0	C
10	BICF2P1146600	67608673	A	0	0	G
10	BICF2P1321313	67618207	A	0	0	G
10	BICF2P993582	67627254	A	0	0	G
10	BICF2P278101	67637494	G	0.5	0.5	A
10	BICF2S23251761	67647447	A	0.25	0.2857	G
10	BICF2P526962	67660908	G	0.5	0.5	A
10	BICF2P773747	67676047	G	0.5	0.5	A
10	BICF2P1456818	67686923	A	0	0	C
10	BICF2S23326718	67696570	A	0.25	0.2857	G
10	BICF2P989136	67730683	0	0	0	G
10	BICF2P1406628	67750033	A	0	0	G
10	BICF2P400502	67768061	C	1	1	A
10	BICF2P823840	67803124	G	0.5	0.5	A
10	BICF2S22960468	67807496	G	0.5	0.5	A
10	BICF2S23538986	67828815	0	0	0	G
10	BICF2P29743	67844432	G	0.5	0.5	A
10	BICF2S23647325	67858787	A	0.25	0.2143	G
10	BICF2S2294962	67865659	A	0.25	0.2143	G
10	BICF2P886496	67893625	G	0.75	0.7857	A
10	BICF2S23214734	67897365	A	0	0	G
10	BICF2P1336575	67922240	A	0.25	0.2143	C
10	BICF2P511761	67935493	A	0.25	0.2143	G
10	BICF2S231670	67938032	A	0.5	0.5	G
10	BICF2P541019	67947533	A	0.5	0.5	G
10	BICF2S2374310	67956932	A	0.5	0.5	G
10	TIGRP2P142016_rs8680133	67973781	0	0	0	G
10	BICF2P1113786	67979635	A	0	0	G
10	BICF2P923352	67988744	C	1	1	A
10	BICF2P341259	68001089	A	0	0	G
10	BICF2S23758497	68015910	C	0	0	A
10	BICF2P887094	68034360	0	0	0	T
10	TIGRP2P142019_rs8543649	68059667	0	0	0	A
10	BICF2P291770	68075263	0	0	0	G
10	BICF2S23030401	68100090	A	0	0	G

10	BICF2P474839	68108759	G	0	0	C
10	BICF2P862338	68122903	C	0.5	0.5	A
10	BICF2P1241044	68129711	G	0.5	0.5	A
10	BICF2P282379	68141665	A	0	0	G
10	BICF2S23130745	68149157	A	0	0	G
10	BICF2P638141	68168692	G	0.25	0.2143	A
10	BICF2P869152	68173197	0	0	0	G
10	BICF2P401145	68184234	0	0	0	G
10	BICF2P1451979	68195242	G	0.75	0.7143	A
10	BICF2P1336425	68210492	A	0.75	0.75	G
10	BICF2P406567	68226116	G	0.75	0.7143	A
10	BICF2P110742	68234268	A	0.25	0.2143	G
10	BICF2P1188307	68244692	A	0.5	0.5	G
10	BICF2P819940	68263833	A	0.25	0.2143	G
10	BICF2P1332669	68271876	A	0	0	G
10	BICF2P1186682	68277894	A	0	0	G
10	BICF2P449526	68290145	G	0.25	0.2857	A
10	BICF2S23725285	68301618	0	0	0	G
10	BICF2P660961	68320226	A	0.5	0.5	G
10	BICF2P663129	68323860	C	0	0	A
10	BICF2P628262	68326970	A	0	0	C
10	BICF2P808237	68340072	A	0	0	T
10	BICF2S23631554	68348260	A	0.25	0.2143	C
10	BICF2P112859	68365696	0	0	0	C
10	BICF2P330467	68369705	A	0.25	0.2143	G
10	BICF2P481574	68399873	0	0	0	G
10	BICF2S23052105	68403365	A	0	0	G
10	BICF2S23141371	68406177	A	0.5	0.5	G
10	BICF2P725803	68416972	G	0	0	A
10	BICF2P203681	68431740	0	0	0	A
10	BICF2P419450	68442452	A	0.25	0.2857	G
10	BICF2P642104	68457842	A	0.5	0.2	G
10	BICF2P1090002	68462189	A	0	0	G
10	BICF2P381674	68476348	T	0	0	A
10	BICF2S23541283	68483976	A	0	0	G
10	BICF2P192539	68494938	0	0	0	G
10	BICF2P445764	68533519	0	0	0	A
10	BICF2P236497	68540328	0	0	0	G
10	BICF2P200409	68557366	0	0	0	G
10	BICF2P1067306	68564897	G	1	1	A
10	BICF2P1097534	68583455	G	0.75	0.7857	A
10	BICF2P1452928	68601498	A	0.5	0.5	G
10	BICF2P1036499	68614890	A	0.25	0.2143	G
10	BICF2S23740996	68625641	A	0	0	G
10	BICF2P1317379	68632054	G	0.25	0.2143	A
10	BICF2S23540461	68646224	A	0	0	G
10	BICF2S2395155	68648839	0	0	0	A

10	BICF2P1190703	68651457	G	0.5	0.5	A
10	TIGRP2P142024_rs8737244	68655822	0	0	0	G
10	BICF2P290605	68675666	G	0.5	0.5	A
10	BICF2P481925	68683673	G	0.25	0.2143	A
10	BICF2P1231633	68685371	A	0.5	0.5	G
10	BICF2P1231632	68685389	A	0.25	0.2857	C
10	BICF2S23550231	68696367	A	0.5	0.5	G
10	BICF2S23150753	68702444	A	0	0	G
10	BICF2P627923	68715900	G	0.25	0.2857	A
10	BICF2S23039709	68727213	G	0	0	C
10	BICF2P1083139	68741290	A	0	0	G
10	BICF2P837623	68752093	A	0	0	C
10	BICF2P1177811	68765532	A	0.25	0.2143	G
10	BICF2P1298571	68775993	A	0.75	0.7143	G
10	BICF2P261701	68788746	A	0.75	0.7143	G
10	BICF2P1118533	68795974	A	0	0	G
10	BICF2P963750	68800740	A	0	0	G
10	BICF2P963752	68806148	0	0	0	A
10	BICF2P1289527	68825065	G	0.25	0.2143	A
10	BICF2P1237947	68827570	A	0.25	0.2143	G
10	BICF2P1289286	68848527	A	0.25	0.2857	G
10	BICF2S23129807	68853106	A	0.25	0.2857	G
10	BICF2P1014113	68873109	G	0	0	A
10	BICF2P118612	68875405	A	0.25	0.2857	G
10	BICF2P335835	68892966	G	1	1	A
10	BICF2P242482	68901659	A	0.25	0.2143	G
10	BICF2P1433134	68913104	0	0	0	C
10	BICF2P325728	68922078	T	0.25	0.2857	A
10	BICF2P1014812	68940432	A	0	0	G
10	BICF2P590306	68942918	G	0.5	0.5	A
10	BICF2P987543	68956423	A	0	0	G
10	BICF2S23652479	68957803	A	0	0	G
10	BICF2S23248674	68968385	A	0	0	G
10	BICF2P240586	68980418	A	0	0	G
10	BICF2S23015044	68992887	G	0.5	0.5	A
10	BICF2S23130895	69000703	G	0.5	0.5	A
10	BICF2S23323647	69011854	A	0	0	G
10	BICF2S23513088	69026432	A	0	0	G
10	BICF2S23413646	69037983	A	0	0	G
10	BICF2P1023418	69056877	A	0.5	0.5	G
10	BICF2S23040035	69057940	C	0.5	0.5	A
10	BICF2P844284	69071007	A	0.5	0.5	T
10	BICF2P385526	69081207	0	0	0	G
10	BICF2S23546794	69092374	G	0	0	A
10	TIGRP2P142033_rs8863262	69106233	0	0	0	A
10	TIGRP2P142034_rs8730701	69122501	0	0	0	C
10	BICF2P45601	69131727	G	0.5	0.5	A

10	BICF2S23511226	69146098	0	0	0	G
10	TIGRP2P142036_rs8756957	69153964	0	0	0	T
10	BICF2S23134189	69166290	A	1	1	G
10	BICF2P973408	69176424	0	0	0	A
10	BICF2S23658063	69197918	G	0	0	A
10	BICF2P287842	69213078	G	0.5	0.5	A
10	BICF2S23041814	69223646	G	0	0	A
10	BICF2S23024921	69234533	C	1	1	A
10	BICF2S23720065	69243872	0	0	0	A
10	BICF2P672858	69254945	0	0	0	C
10	BICF2P675768	69270613	A	0	0	C
10	BICF2S23713268	69271106	G	0.5	0.5	A
10	BICF2P1408081	69275427	A	0.5	0.5	G
10	BICF2S237995	69279008	0	0	0	G
10	BICF2S23034611	69287581	C	0.5	0.5	A
10	BICF2S23253529	69306839	A	0.5	0.5	G
10	BICF2S23544398	69313908	G	0.5	0.5	A
10	BICF2S2321174	69328666	0	0	0	A
10	BICF2P1143461	69336652	0	0	0	G
10	TIGRP2P142038_rs9137793	69358593	0	0	0	G
10	BICF2P1285490	69375178	A	0	0	G
10	BICF2P1285489	69375267	A	0	0	T
10	BICF2P848951	69383672	C	0	0	G
10	BICF2S23145796	69402905	A	0.75	0.7857	T
10	BICF2S23449391	69421172	A	0	0	C
10	BICF2P1013880	69432399	A	0	0	G
10	BICF2P1010001	69462365	A	0	0	G
10	BICF2P364709	69478444	A	0	0	G
10	BICF2P855072	69490311	A	0	0	G
10	BICF2P926636	69504215	A	0	0	G
10	BICF2P926635	69504264	A	0	0	G
10	BICF2P1011611	69509130	A	0	0	G
10	BICF2P1335642	69517985	0	0	0	A
10	TIGRP2P142041_rs8706658	69547509	0	0	0	G
10	BICF2S22940632	69579748	A	0	0	G
10	BICF2P550765	69592463	0	0	0	A
10	BICF2S23134141	69606529	A	0.75	0.7857	C
10	BICF2S23521795	69617786	C	0	0	A
10	BICF2P1317829	69622838	0	0	0	C
10	BICF2P585249	69639086	C	0	0	A
10	BICF2P795076	69651592	A	0.25	0.2143	G
10	BICF2S23160233	69661048	A	0	0	G
10	BICF2P1141093	69678109	G	0.75	0.7857	A
10	BICF2S23117081	69685941	A	0.5	0.5	G
10	BICF2P1037725	69698991	A	0.25	0.2857	G
10	BICF2P1023012	69708323	A	0.25	0.2857	G
10	BICF2P1458300	69719382	A	0.5	0.5	G

10	BICF2S23129820	69726334	A	0	0	G
10	BICF2S23323392	69756965	G	0.75	0.7857	A
10	BICF2P125172	69772764	0	0	0	A
10	BICF2P701368	69785246	C	0.25	0.2143	A
10	BICF2P755791	69789549	A	0.75	0.7857	G
10	BICF2S23414100	69797135	A	0	0	G
10	BICF2P1100165	69806670	A	1	0.7857	G
10	BICF2P738930	69816891	A	1	1	G
10	BICF2P162872	69828186	A	0	0	G
10	BICF2P89098	69840354	G	0.75	0.7857	A
10	BICF2P196546	69856863	A	0.75	0.7857	G
10	BICF2P1356466	69870142	A	0.25	0.2143	G
10	BICF2P32638	69883562	G	0.25	0.2143	A
10	BICF2P1134765	69885507	G	0	0	A
10	BICF2P1278253	69899616	A	0.75	0.7857	G
10	BICF2P1154999	69909154	G	0	0	A
10	BICF2P358578	69910726	G	0	0	C
10	BICF2P771960	69921096	A	0.25	0.2143	G
10	BICF2P77420	69927082	T	0	0	A
10	BICF2P1172901	69936392	A	1	1	C
10	BICF2P1109223	69944055	G	0.75	0.7857	A
10	BICF2P777787	69964533	G	1	1	A
10	BICF2P1171096	69966151	A	0.25	0.2857	C
10	BICF2P333722	69981159	A	0.75	0.7143	G
10	BICF2P1221173	69989382	C	0	0	A
10	TIGRP2P142044_rs9233700	70008406	0	0	0	A
10	BICF2S2298653	70013832	A	0.25	0.2857	G
10	BICF2P764487	70033389	A	0	0	G
10	BICF2P764485	70034259	A	0	0	G
10	BICF2P764482	70035931	A	0	0	C
10	BICF2P1456274	70057001	A	1	1	G
10	BICF2P402451	70068417	A	0	0	C
10	BICF2S23049416	70075614	A	0	0	G
10	BICF2P224963	70082068	A	0.25	0.2143	C
10	BICF2P1235056	70095678	A	0.75	0.7143	G
10	BICF2P794522	70099413	A	0	0	C
10	BICF2P1435011	70104690	A	0	0	G
10	BICF2P892153	70123467	A	0.75	0.7857	G
10	BICF2P528249	70127651	A	0.25	0.2143	G
10	BICF2P1086379	70147501	G	0.25	0.2857	A
10	BICF2P717209	70150073	A	0.5	0.5	G
10	BICF2P547355	70167598	T	0	0	A
10	BICF2P258761	70175632	A	0	0	G
10	BICF2P989049	70184743	C	0.25	0.2857	A
10	BICF2P121051	70202846	A	0	0	G
10	BICF2P121050	70202953	G	0	0	A
10	BICF2P383148	70211548	C	0.25	0.2143	A

10	BICF2S23423946	70215620	G	0.5	0.5	A
10	BICF2S23716167	70226695	G	0.25	0.2857	C
10	BICF2S23326688	70234300	A	0	0	G
10	BICF2S23638251	70247328	A	0	0	C
10	BICF2P1072013	70264173	A	0	0	G
10	BICF2P1072015	70265330	A	0	0	G
10	BICF2P14998	70280672	A	0	0	G
10	BICF2P1205673	70290497	A	0.75	0.7857	T
10	BICF2P1150913	70301243	0	0	0	A
10	BICF2P911270	70319982	A	0.5	0.5	G
10	BICF2S23432760	70326872	G	0.5	0.5	A
10	BICF2P104299	70334793	0	0	0	C
10	BICF2S23232095	70347403	A	0.5	0.5	G
10	BICF2S23762545	70365074	G	0	0	A
10	BICF2S23761795	70368997	A	0	0	G
10	BICF2S23653520	70382013	A	0	0	G
10	BICF2P1392963	70390190	A	0.5	0.5	C
10	BICF2P968718	70431098	G	0.25	0.2143	A
10	BICF2P112091	70438598	A	0	0	G
10	BICF2P598228	70451076	A	0	0	T
10	BICF2S23330379	70460506	A	0.5	0.5	G
10	BICF2S23636815	70473545	0	0	0	G
10	BICF2S23056939	70494601	A	0.75	0.7143	G
10	BICF2P440252	70519661	G	0.25	0.2143	C
10	BICF2P713355	70547644	G	1	1	A
10	BICF2P1303845	70555546	G	0	0	A
10	TIGRP2P142089_rs8676694	70564410	C	0	0	A
10	TIGRP2P142096_rs8767181	70578262	G	0.25	0.2143	A
10	BICF2P592936	70592903	A	0.5	0.5	C
10	TIGRP2P142105_rs9053078	70610564	G	0	0	A
10	BICF2S23452165	70630270	A	0.75	0.7857	G
10	BICF2P286088	70640884	A	0	0	G
10	BICF2S23649210	70659586	A	0.75	0.7857	C
10	BICF2P885832	70668595	0	0	0	C
10	BICF2P663203	70696871	A	0.5	0.5	G
10	BICF2P41585	70705300	G	0.75	0.7857	A
10	BICF2S23425562	70724149	A	0	0	G
10	BICF2S23013811	70741972	A	0	0	G
10	BICF2P406706	70751375	A	0.25	0.2143	G
10	TIGRP2P142169_rs8803154	70770416	A	0.5	0.5	G
10	BICF2S23435360	70772181	A	0	0	G
10	TIGRP2P142194_rs8968570	70788699	G	0.25	0.2857	A
10	BICF2S23544332	70802022	G	0.5	0.5	A
10	TIGRP2P142199_rs9190244	70815369	G	0	0	A
10	BICF2P930620	70820196	A	0	0	G
10	BICF2P549709	70836821	A	0	0	G
10	BICF2P1380678	70843373	A	0.75	0.7143	G

10	BICF2P38356	70870913	A	0.25	0.2857	G
10	BICF2S23548142	70879700	G	0.25	0.2857	A
10	TIGRP2P142246_rs9132132	70886781	A	0.25	0.2143	G
10	BICF2S23215125	70897713	A	0	0	G
10	BICF2P1051161	70920161	A	0.25	0.2143	G
10	BICF2P339814	70925841	A	0	0	G
10	BICF2S23220915	70935630	A	0.75	0.7143	G
10	BICF2P2915	70943703	A	0	0	G
10	BICF2P1220758	70965502	A	0.25	0.2857	C
10	BICF2P721092	70977442	A	0	0	G
10	TIGRP2P142322_rs8819312	70987501	A	0	0	G
10	TIGRP2P142332_rs8870965	70996944	A	0	0	G
10	TIGRP2P142342_rs8527080	71008842	A	0	0	G
10	TIGRP2P142346_rs8697788	71018609	A	0	0	G
10	BICF2S23045376	71024738	G	0.25	0.2857	A
10	BICF2P1335408	71027400	A	0	0	G
10	BICF2P709640	71038731	A	0.25	0.2857	C
10	BICF2P867718	71054115	G	1	1	A
10	BICF2P1147579	71063768	G	0.75	0.7143	A
10	BICF2P606142	71074074	A	0.25	0.2143	G
10	BICF2P893147	71082800	A	0.25	0.2857	G
10	BICF2S23141787	71094481	G	0.25	0.2143	A
10	BICF2P1347037	71110517	A	0	0	G
10	BICF2P856970	71114695	A	0.25	0.2857	G
10	BICF2P611615	71126246	A	1	1	G
10	BICF2S23752819	71131667	A	0.25	0.2857	G
10	BICF2G630492620	71149708	G	0.75	0.7857	A
10	BICF2G630492623	71150964	A	0.25	0.2857	G
10	BICF2G630492634	71165893	A	1	0.7143	G
10	BICF2G630492647	71174903	G	1	1	A
10	BICF2G630492648	71175503	C	1	1	A
10	BICF2P1072005	71194606	G	0.5	0.5	A
10	BICF2P156879	71202258	A	0.5	0.5	G
10	BICF2G630492672	71211573	A	0	0	C
10	BICF2G630492681	71222698	A	0.25	0.2857	G
10	BICF2G630492691	71232445	A	0.25	0.2857	G
10	BICF2S23421837	71243059	G	0.25	0.2857	A
10	BICF2G630492706	71261011	C	0.25	0.2857	A
10	BICF2G630492714	71265882	A	0	0	G
10	BICF2S23619361	71279601	0	0	0	G
10	BICF2G630492729	71293030	G	NA	0.6667	A
10	BICF2G630492745	71309903	A	0.25	0.2857	G
10	BICF2G630492752	71313757	G	0.25	0.2857	A
10	BICF2G630492775	71327343	A	0	0	G
10	BICF2G630492788	71338031	0	0	0	G
10	TIGRP2P142511_rs9033005	71346290	G	0.25	0.2143	A
10	BICF2G630492831	71357594	A	0.5	0.5	G

10	BICF2G630492844	71360359	G	0.5	0.5	A
10	BICF2S23419942	71371811	A	0.25	0.1667	G
10	BICF2S23348910	71384164	A	0.5	0.5	G
10	BICF2P668598	71392546	A	0.25	0.2143	C
10	BICF2G630492894	71405356	A	0.5	0.5833	G
10	BICF2G630492915	71416577	A	0.25	0.2857	G
10	BICF2G630492920	71434651	G	0.75	0.7143	A
10	BICF2G630492931	71440956	A	0	0.25	G
10	BICF2G630492949	71446224	A	0.25	0.2857	G
10	BICF2G630492956	71451050	G	0.75	0.7143	A
10	BICF2S23213424	71465347	G	1	1	A
10	BICF2G630493007	71487832	A	0.75	0.7857	G
10	BICF2G630493025	71504391	A	1	1	G
10	BICF2P1167486	71507070	A	1	1	G
10	TIGRP2P142578_rs8876627	71527552	0	0	0	A
10	BICF2G630493059	71527667	C	0.5	0.5	A
10	BICF2S23435141	71530212	A	0.5	0.5	G
10	BICF2G630493067	71550140	A	0.25	0.2857	G
10	BICF2G630493088	71559113	A	0.5	0.5	G
10	BICF2G630493152	71593982	G	0	0	A
10	BICF2G630493155	71604232	G	0.5	0.5	A
10	BICF2S2334722	71611932	A	0	0	G
10	BICF2G630493156	71623396	G	0	0	A
10	TIGRP2P142619_rs8837356	71637716	G	0	0	A
10	BICF2P572537	71650258	G	0	0	A
10	BICF2P855401	71661013	0	0	0	A
10	BICF2P187729	71678031	G	0	0	A
10	BICF2G630493171	71686805	A	0.25	0.2143	C
10	BICF2G630493180	71695374	G	0.25	0.2143	A
10	BICF2G630493200	71713717	A	0	0	G
10	BICF2G630493203	71715688	A	0	0	G
10	BICF2G630493257	71747594	G	0	0	A
10	BICF2P1282251	71755255	A	0.5	0.5714	G
10	BICF2G630493290	71763448	T	0	0	A
10	BICF2P668033	71775550	A	0	0	C
10	BICF2G630493321	71785831	A	1	1	C
10	BICF2G630493342	71794972	A	1	1	G
10	BICF2P490104	71810355	G	0.75	0.7143	A
10	TIGRP2P142684_rs9043843	71857002	A	0	0	G
10	BICF2P945003	71874923	C	0	0	G
10	BICF2P635140	71882669	G	0	0	A
10	BICF2P1381513	71893850	G	0.25	0.2143	A
10	BICF2P626018	71901708	G	0.75	0.7857	A
10	BICF2G630493392	71920115	A	0.25	0.2857	G
10	BICF2G630493397	71927508	A	0.5	0.5	G
10	BICF2G630493407	71934858	G	0.5	0.5	A
10	BICF2G630493418	71945135	A	0.5	0.5	G

10	BICF2G630493435	71959156	G	0.5	0.5	A
10	BICF2G630493442	71967991	A	0	0	G
10	BICF2G630493464	71981077	G	0.25	0.2143	C
10	BICF2G630493479	71991029	G	0.25	0.2857	A
10	BICF2G630493487	72002528	C	0.25	0.2857	A
10	BICF2G630493531	72025606	A	0.25	0.2857	G
10	BICF2P59705	72043358	A	0	0	C
10	TIGRP2P142749_rs8522915	72050580	A	0.75	0.7143	G
10	TIGRP2P142755_rs9118059	72057722	A	0	0	G
10	BICF2P900013	72063697	G	0	0	A
10	BICF2P164304	72073677	G	0	0	A
10	TIGRP2P142790_rs9081297	72091041	A	0	0	G
10	BICF2G630493584	72099249	A	0	0	G
10	BICF2P808338	72101235	A	0	0	G
10	BICF2P808339	72101415	A	0	0	G
10	BICF2G630493609	72107750	G	0.5	0.5	A
10	BICF2G630493637	72117078	A	0	0	G
10	BICF2G630493672	72130512	G	0.75	0.7143	A
10	BICF2P206486	72143627	G	0	0	A
10	BICF2G630493701	72157638	G	0.75	0.7857	A
10	BICF2P504908	72162931	A	0	0	C
10	BICF2G630493744	72177241	G	0.25	0.2143	A
10	BICF2G630493755	72192602	A	1	1	G
10	BICF2G630493785	72213776	A	1	1	G
10	TIGRP2P142851_rs8880694	72223171	C	0.75	0.7857	A
10	TIGRP2P142853_rs8787903	72231723	A	0.75	0.7857	G
10	BICF2G630493877	72248770	A	0	0	G
10	BICF2G630493904	72256098	A	0.5	0.5714	G
10	BICF2G630493961	72273643	C	0	0	A
10	BICF2G630493964	72274570	G	0.25	0.2143	A
10	BICF2S23435776	72277420	C	0.25	0.2857	A
10	BICF2S23441702	72279612	G	0.75	0.7143	A
10	BICF2G630493999	72290075	A	0.25	0.2857	G
10	BICF2G630494028	72305748	A	0.25	0.2857	G
10	BICF2P1298014	72309352	G	0.5	0.5	A
10	BICF2G630494052	72316902	A	0.75	0.7143	G
10	TIGRP2P142914_rs8679424	72344455	G	0	0	A
10	BICF2P253037	72378836	0	0	0	C
10	BICF2G630494064	72391908	A	0	0	C
10	BICF2P1180652	72397458	A	0	0	G
10	BICF2S2307054	72405963	A	0	0	G
10	BICF2S23124788	72419055	G	0	0	A
10	BICF2S23658552	72438602	A	0	0	G
10	BICF2P1409561	72450954	T	0	0	A
10	BICF2G630494083	72471775	G	0	0	A
10	BICF2G630494087	72474083	A	0	0	C
10	BICF2G630494111	72486446	0	0	0	G

11	BICF2P668751	3047852	A	0.5	0	G
11	BICF2P319972	3123609	A	0.5	0.08333	C
11	BICF2P270425	3160933	A	0.75	0.7857	T
11	BICF2P222415	3180398	0	0	0	G
11	BICF2P1092406	3250471	C	0.5	0.1429	A
11	BICF2P41395	3273054	0	0	0	A
11	G618f8S209	3302821	0	0	0	C
11	G619f8S149	3329375	0	0	0	C
11	G620f20S181	3374543	0	0	0	C
11	BICF2S2306756	3393149	A	0.5	0.1429	C
11	BICF2P757289	3406727	A	0.25	0.2143	G
11	BICF2P109854	3418692	G	0.5	0.1429	A
11	BICF2P368048	3432890	A	0.25	0.2143	G
11	BICF2P771981	3445531	0	0	0	G
11	BICF2P1143274	3461111	0	0	0	A
11	BICF2S22950773	3476047	A	0.25	0.6429	G
11	BICF2S2341930	3524160	C	0.25	0.6429	G
11	BICF2P1287750	3575010	0	0	0	C
11	BICF2P214336	3712613	G	1	0.4	A
11	BICF2G63027441	3751303	G	0	0	A
11	BICF2P1069149	3775338	0	0	0	A
11	G623f23S172	3797509	0	0	0	C
11	BICF2S23056694	3835514	C	0	0	G
11	BICF2P331249	3860994	T	0.25	0.2143	A
11	BICF2P974524	3895775	0	0	0	C
11	BICF2P800917	3907891	0	0	0	G
11	BICF2P41060	4022660	A	0.5	0.1429	G
11	BICF2P349652	4048797	0	0	0	G
11	TIGRP2P126405_rs8617006	4088072	0	0	0	G
11	BICF2P620164	4094927	0	0	0	A
11	BICF2P1211297	4131782	0	0	0	A
11	BICF2P1472330	4148150	0	0	0	C
11	BICF2S23514671	4170651	C	0	0	A
11	BICF2S23135525	4177209	A	0.25	0.2143	G
11	BICF2S24116836	4187710	G	0.25	0.6429	A
11	TIGRP2P144249_rs9038479	4199830	0	0	0	C
11	TIGRP2P144246_rs8943046	4218505	0	0	0	G
11	BICF2P1317760	4243005	0	0	0	G
11	BICF2P1317759	4243932	A	0.5	0.1429	G
11	BICF2P89993	4276515	0	0	0	G
11	BICF2P85357	4300151	0	0	0	G
11	BICF2P1195278	4304846	A	0.5	0.1429	G
11	BICF2P1211034	4308755	A	0.5	0.1429	C
11	BICF2P899486	4357941	0	0	0	A
11	TIGRP2P144235_rs8852329	4387938	0	0	0	A
11	BICF2P199814	4401983	0	0	0	G
11	BICF2P216409	4407092	0	0	0	A

11	BICF2S23530585	4433287	G	0	0	A
11	BICF2P208262	4441945	0	0	0	C
11	BICF2P382716	4458354	0	0	0	A
11	BICF2P187306	4474591	0	0	0	C
11	BICF2P169599	4492213	0	0	0	G
11	BICF2P645088	4550792	0	0	0	C
11	BICF2P844001	4566956	0	0	0	A
11	G630f25S79	4580164	0	0	0	A
11	TIGRP2P143192_rs8550018	4614844	G	NA	0	A
11	G631f34S254	4650742	0	0	0	A
11	BICF2P491002	4681784	0	0	0	A
11	G633f51S270	4763383	0	0	0	C
11	BICF2S23911110	4782291	0	0	0	G
11	BICF2P450538	4793964	0	0	0	A
11	BICF2P1348959	4806461	A	0.5	0.1429	G
11	BICF2P184813	4810966	0	0	0	C
11	BICF2P692941	4838627	0	0	0	C
11	BICF2P734487	4851446	A	0.25	0.2143	G
11	TIGRP2P144055_rs8951880	4864196	0	0	0	G
11	BICF2P94556	4869391	0	0	0	G
11	BICF2P54557	4875195	A	0.25	0.2143	G
11	BICF2P450874	4932136	0	0	0	G
11	BICF2S2448146	4937172	A	0.25	0.6429	G
11	TIGRP2P144054_rs9015725	4953998	0	0	0	C
11	TIGRP2P144053_rs8920213	4966424	A	0.25	0.2143	C
11	BICF2S23655207	4968512	0	0	0	G
11	G634f26S181	5024116	0	0	0	C
11	BICF2S23038144	5047109	C	0.25	0.6429	A
11	BICF2P20201	5166275	A	0.5	0.1429	C
11	BICF2P605713	5183553	C	0.25	0.2143	G
11	BICF2P1108445	5215099	G	0.5	0.1429	A
11	BICF2P1123767	5223500	A	0.25	0.2143	G
11	BICF2P685844	5254236	G	0.75	0.7857	A
11	BICF2P164344	5263222	G	0.75	0.7857	A
11	BICF2S2296204	5269181	G	0.5	0.1429	A
11	BICF2P1447864	5277832	G	0.5	0.1429	A
11	BICF2P214896	5289066	A	0.5	0.1429	G
11	BICF2P706947	5294577	A	0.5	0.1429	G
11	BICF2P409939	5309861	A	0.25	0.2143	G
11	BICF2P844586	5314961	0	0	0	G
11	TIGRP2P150135_rs8512833	5327776	0	0	0	C
11	BICF2S2459404	5332791	C	0.5	0.1429	G
11	BICF2P390247	5355019	A	0.5	0.1429	T
11	BICF2P577619	5365397	A	0.5	0.1429	T
11	BICF2P1428057	5378849	G	0.75	0.7857	A
11	BICF2P156101	5387883	0	0	0	G
11	BICF2P401711	5396693	A	0.5	0.1429	C

11	BICF2P974873	5408404	A	0.5	0.1429	C
11	BICF2P178197	5416597	A	0.5	0.1429	G
11	BICF2P851010	5430708	G	0.5	0.1429	A
11	BICF2S2301364	5444596	G	0.5	0.1429	A
11	BICF2P1315481	5459294	A	0.5	0.1429	C
11	BICF2P332173	5465032	G	0.5	0.1429	A
11	BICF2P978958	5479765	0	0	0	G
11	TIGRP2P150124_rs9190374	5496803	A	0.25	0.6429	G
11	BICF2P200797	5497416	A	0.5	0.1429	G
11	BICF2P884769	5525910	A	0.5	0.08333	G
11	TIGRP2P150122_rs8885002	5550265	0	0	0	A
11	BICF2P239808	5582160	0	0	0	G
11	TIGRP2P150120_rs8971374	5592738	A	0.25	0.2143	G
11	BICF2S2393608	5604031	0	0	0	A
11	BICF2S23439277	5614265	A	0	0	C
11	BICF2P786038	5642298	0	0	0	C
11	BICF2P771704	5662400	0	0	0	A
11	BICF2P1098772	5675223	A	0.75	0.7857	G
11	BICF2P1288573	5710859	G	0.5	0.1429	A
11	BICF2P700503	5750941	A	0.5	0.1429	G
11	TIGRP2P150109_rs8767282	5764105	0	0	0	C
11	BICF2P1406089	5774732	0	0	0	G
11	BICF2S2451624	5813010	C	0	0	A
11	TIGRP2P150106_rs8996680	5830661	A	0.75	0.7857	G
11	BICF2P1173378	5848248	A	0.5	0.625	G
11	BICF2P108034	5867323	A	0.25	0.2143	G
11	BICF2S23425079	5915508	G	0	0	A
11	BICF2P571357	5922506	0	0	0	A
11	TIGRP2P150098_rs9131527	5937199	A	0.25	0.2143	G
11	BICF2P702331	5966274	A	0.75	0.7857	G
11	BICF2P1183058	5988821	A	0.75	0.7857	G
11	BICF2P1309764	6010076	A	0.75	0.7857	G
11	BICF2P438566	6026012	A	0.5	0.1429	G
11	BICF2P1272347	6044781	G	0.5	0.1429	A
11	BICF2P1004406	6056722	G	0.25	0.2143	A
11	BICF2P1315236	6066515	0	0	0	A
11	BICF2P1192114	6094766	A	0.5	0.1429	C
11	BICF2P1431816	6101124	0	0	0	G
11	BICF2P355627	6124244	C	0.25	0.2143	A
11	BICF2P309441	6133138	G	0.25	0.2143	A
11	BICF2P329896	6142749	A	0.25	0.2143	G
11	BICF2S23060166	6170518	G	0.75	0.3571	A
11	BICF2P392930	6186787	A	0.25	0.2143	C
11	BICF2S22955679	6189018	T	0.75	0.3571	A
11	BICF2P1362530	6201031	0	0	0	A
11	BICF2P337855	6214953	A	0.5	0.1429	T
11	BICF2P814387	6222754	A	0.25	0.2143	G

11	BICF2S23647308	6234780	G	0.75	0.3571	A
11	TIGRP2P150060_rs8971641	6246243	A	0.5	0.1429	C
11	BICF2P970573	6256609	G	0.5	0.1429	A
11	BICF2S2436185	6274337	G	0	0	A
11	BICF2P1086529	6283243	G	0.5	0.1429	A
11	BICF2P129298	6323126	0	0	0	G
11	G639f28S293	6363434	0	0	0	A
11	BICF2S23117426	6417074	A	0	0	G
11	BICF2P1130215	6458144	A	0.5	0.1429	G
11	BICF2S2328939	6482506	A	0.25	0.6429	C
11	BICF2S2367390	6490115	G	0.25	0.6429	A
11	BICF2P1061328	6506732	0	0	0	G
11	BICF2P1278249	6517414	0	0	0	A
11	BICF2P1194233	6525275	A	0.5	0.1429	G
11	BICF2S2352244	6550553	A	0	0	G
11	TIGRP2P150039_rs8663792	6591291	G	0.5	0.1429	A
11	BICF2S2321420	6615948	T	0.25	0.6429	A
11	G641f37S153	6643367	A	0.25	0.6429	G
11	BICF2P107439	6686422	0	0	0	G
11	BICF2S2454483	6712664	0	0	0	G
11	BICF2P1032951	6722328	0	0	0	A
11	BICF2S23056179	6779648	C	0	0	A
11	BICF2S23212641	6809360	A	0	0.6667	G
11	BICF2S23255043	6833167	A	0	0	G
11	BICF2S23522651	6847619	C	0	0	A
11	BICF2P932987	6854917	0	0	0	C
11	BICF2S23256864	6878884	G	0	0	A
11	TIGRP2P150033_rs8781834	6918331	G	0.75	0.7857	A
11	TIGRP2P150032_rs8834298	6927849	0	0	0	G
11	BICF2S23535295	6975739	0	0	0	A
11	BICF2S2442173	6995549	0	0	0	A
11	BICF2S23037464	7003769	A	0	0	C
11	BICF2S2323535	7023853	C	0	0	A
11	TIGRP2P150029_rs8677473	7092018	A	0.75	0.7857	C
11	BICF2P1061554	7100426	G	0	0.1667	A
11	BICF2P229728	7112476	G	0.25	0.1667	A
11	BICF2P574398	7125137	G	0.25	0.2143	A
11	BICF2P114041	7141158	G	0.25	0.2143	A
11	TIGRP2P150019_rs8881402	7158381	A	0.25	0.2143	G
11	BICF2P997679	7171601	G	0.25	0.2143	A
11	BICF2P1321472	7201372	G	0.25	0.2143	A
11	TIGRP2P150006_rs8966189	7211434	G	0.25	0.2143	A
11	TIGRP2P150001_rs8940057	7223302	G	0.25	0.2143	A
11	BICF2P428466	7236992	A	0.25	0.2143	G
11	BICF2P79517	7245589	G	0	0	A
11	BICF2S23445920	7261843	C	0.25	0.6429	A
11	BICF2P944027	7278626	0	0	0	T

11	G643f57S97	7302180	0	0	0	A
11	G643f60S183	7303349	A	0	0	G
11	BICF2P44194	7324217	0	0	0	G
11	BICF2S23914471	7326005	C	0	0	A
11	G644f48S19	7348768	0	0	0	A
11	BICF2P623647	7374170	0	0	0	A
11	BICF2P102045	7390910	0	0	0	A
11	TIGRP2P149977_rs8821762	7399029	G	0.25	0.1667	A
11	BICF2P562545	7421268	0	0	0	G
11	G646f23S228	7504714	A	0	0	C
11	BICF2S2366868	7525415	A	0.75	0.3571	G
11	BICF2P953472	7534034	0	0	0	T
11	BICF2P212986	7550478	0	0	0	A
11	BICF2S2395925	7563649	0	0	0	A
11	BICF2P151876	7569487	A	0.25	0.2143	C
11	BICF2P748969	7605116	A	0	0	G
11	BICF2P311414	7622205	0	0	0	A
11	TIGRP2P149969_rs9120182	7640067	A	0.75	0.3571	G
11	TIGRP2P149965_rs8557225	7649375	A	0.75	0.3571	G
11	BICF2P604103	7672232	G	0.75	0.3571	A
11	BICF2S23256530	7682107	A	0.75	0.3571	G
11	BICF2P1414918	7693729	A	0.75	0.3571	G
11	TIGRP2P149951_rs8705639	7712979	A	0.75	0.3	G
11	BICF2S2345369	7756845	A	0	0	G
11	BICF2S23053077	7773796	A	0	0	T
11	BICF2P443478	7788629	0	0	0	A
11	G647f13S81	7816251	A	0	0	G
11	BICF2P10021	7847861	A	0.25	0.2143	G
11	BICF2P263686	7855584	G	0.25	0.2143	A
11	BICF2S23347594	7869247	A	0	0	G
11	BICF2P312478	7877510	G	0	0	A
11	TIGRP2P149908_rs8879977	7895438	C	0	0	A
11	TIGRP2P149900_rs8769825	7903826	G	0	0	A
11	BICF2P132705	7916051	0	0	0	A
11	BICF2P863101	7965751	0	0	0	A
11	TIGRP2P149894_rs8643029	7989129	A	0.25	0.6429	G
11	BICF2P1053067	8000821	0	0	0	A
11	BICF2S23049486	8011417	G	0	0	A
11	TIGRP2P149892_rs9090105	8024904	A	0.25	0.6429	C
11	BICF2P969729	8035512	C	0.25	0.6429	A
11	BICF2P1019749	8072685	0	0	0	G
11	BICF2S23037144	8084949	A	0.25	0.6429	G
11	TIGRP2P149887_rs8989157	8127675	0	0	0	G
11	BICF2S23236581	8156137	A	0	0	G
11	BICF2P905805	8166624	C	0.25	0.6429	A
11	G649f34S300	8188049	0	0	0	C
11	BICF2P296142	8220673	0	0	0	C

11	BICF2S23641373	8230525	0	0	0	A
11	TIGRP2P149885_rs8912893	8236757	A	0	0	G
11	BICF2P545154	8263015	0	0	0	A
11	BICF2S237605	8289691	A	0.25	0.6429	G
11	BICF2P276348	8296752	0	0	0	A
11	BICF2S23151669	8317669	A	0.25	0.6429	G
11	BICF2S2338108	8322404	A	0.75	0.8333	G
11	TIGRP2P149879_rs8619106	8326881	0	0	0	A
11	BICF2P937814	8357855	0	0	0	G
11	TIGRP2P149873_rs8525406	8371974	A	0	0	C
11	TIGRP2P149872_rs9232182	8379018	A	0.25	0.6429	G
11	BICF2P1312272	8399868	A	0	0	C
11	BICF2P1287143	8421192	0	0	0	G
11	BICF2P1045378	8430769	0	0	0	C
11	BICF2S22931044	8447576	C	0.75	0.7857	A
11	TIGRP2P149866_rs9009656	8511195	G	0	0	A
11	BICF2P1137579	8524515	0	0	0	A
11	BICF2P371490	8538588	A	0	0	G
11	BICF2P337100	8573167	0	0	0	A
11	TIGRP2P149861_rs8633543	8597183	0	0	0	G
11	BICF2P625025	8602927	0	0	0	A
11	TIGRP2P149860_rs8970013	8614734	0	0	0	G
11	G651f11S91	8637907	A	0	0	C
11	BICF2P97412	8659922	0	0	0	G
11	BICF2P982266	8706562	A	0	0	G
11	BICF2P1291203	8725066	G	0	0	A
11	BICF2P875466	8738955	G	0	0	A
11	BICF2P708760	8742389	A	0	0	G
11	BICF2P708761	8743127	A	0	0	T
11	BICF2P666438	8782440	G	0	0	A
11	TIGRP2P149835_rs8919112	8795752	0	0	0	G
11	BICF2P404366	8822742	G	0.25	0.2143	A
11	BICF2S2299721	8842658	G	0	0.2143	A
11	BICF2P329587	8922437	0	0	0	A
11	BICF2P476049	8924568	0	0	0	G
11	BICF2P361106	8978130	0	0	0	G
11	BICF2P1125455	9015515	C	0.25	0.2143	A
11	TIGRP2P149822_rs8823669	9036650	G	0.25	0.1667	A
11	TIGRP2P149820_rs8856077	9039657	A	0.25	0.2143	G
11	BICF2P653018	9063908	0	0	0	C
11	BICF2S22939919	9080450	G	0	0	A
11	BICF2P399524	9110062	A	0	0	G
11	BICF2P967591	9120094	G	0	0	A
11	BICF2P1373234	9140828	G	0.75	0.3571	A
11	BICF2P1304130	9163839	A	0.75	0.3571	G
11	BICF2P647240	9173664	A	0.75	0.3571	G
11	BICF2S23614480	9183810	A	0	0	G

11	TIGRP2P149796_rs9178510	9188808	0	0	0	G
11	TIGRP2P149793_rs8653478	9222032	0	0	0	A
11	BICF2P745563	9272260	A	0	0	G
11	BICF2P202293	9290409	0	0	0	A
11	G653f34S339	9313611	A	0	0	G
11	G653f38S70	9315900	0	0	0	C
11	BICF2P1092835	9335666	0	0	0	A
11	BICF2S23331719	9340841	A	0.5	0.1429	G
11	BICF2P1109603	9354547	A	0.75	0.3571	G
11	BICF2P104187	9365462	C	0.5	0.8571	A
11	BICF2P1300227	9375896	A	0	0	C
11	TIGRP2P149748_rs8759820	9419550	A	0	0	G
11	BICF2S23044928	9448725	G	0	0	A
11	BICF2P1445173	9464756	A	0	0	G
11	G654f30S200	9494037	G	0.75	0.7857	A
11	G654f31S297	9499940	0	0	0	C
11	TIGRP2P149702_rs9055620	9527454	G	0	0	A
11	TIGRP2P149690_rs8723656	9537155	A	0	0	C
11	BICF2P205369	9542583	G	0	0	A
11	TIGRP2P149688_rs8913494	9554324	0	0	0	A
11	BICF2P387069	9570129	A	0.25	0.2143	C
11	BICF2P689972	9584338	G	0.25	0.2143	A
11	BICF2P90635	9593705	A	0.75	0.3571	G
11	TIGRP2P149664_rs8806551	9608074	G	0.25	0.6429	A
11	BICF2P12726	9624107	G	0.25	0.6429	A
11	BICF2P1250672	9628450	A	0.25	0.6429	G
11	BICF2P56356	9639528	0	0	0	C
11	BICF2P1371973	9659205	G	0	0	A
11	BICF2P353096	9666216	A	0	0	G
11	BICF2P765640	9685108	C	0	0	A
11	BICF2P1210106	9747749	A	0	0	G
11	BICF2P559474	9773895	G	0	0	A
11	BICF2P761931	9792764	A	0.25	0.2143	C
11	TIGRP2P149623_rs8498194	9812792	0	0	0	A
11	BICF2P1171484	9829119	G	0.75	0.7857	A
11	BICF2P757584	9846991	A	0	0	G
11	BICF2P1180450	9852928	A	0	0	G
11	BICF2S2367260	9857170	A	0	0	C
11	TIGRP2P149596_rs9171257	9888366	A	0	0	G
11	BICF2P784281	9891485	G	0	0	C
11	BICF2S2378221	9911530	A	0.5	0.1429	G
11	BICF2P595504	9919070	A	0	0	G
11	BICF2S24310553	9941804	A	0.5	0.1429	G
11	BICF2P424379	9958112	0	0	0	G
11	BICF2S23441547	9969034	C	0.5	0.1429	A
11	BICF2P1095229	9982729	A	0.25	0.2143	G
11	BICF2S23530639	9995856	0	0	0	A

11	BICF2P252905	10015516	C	0	0	A
11	BICF2S23132776	10044032	C	0.5	0.1429	A
11	BICF2P303449	10094258	0	0	0	G
11	BICF2P1393769	10157966	A	0	0	G
11	TIGRP2P143997_rs8810529	10180994	C	0	0	A
11	BICF2P331549	10214643	A	0	0	T
11	BICF2P478016	10224870	0	0	0	G
11	BICF2P478017	10224894	G	0	0	A
11	TIGRP2P143955_rs8808275	10238971	0	0	0	A
11	BICF2P457963	10247114	G	0	0	A
11	BICF2S22957047	10265125	0	0	0	G
11	BICF2P103961	10273230	0	0	0	A
11	BICF2P946175	10286106	G	0	0	A
11	BICF2P1209896	10308488	A	0.75	0.7857	G
11	BICF2P483570	10316412	G	1	1	A
11	BICF2P1116363	10328691	G	1	1	A
11	BICF2P1451292	10338656	0	0	0	A
11	BICF2P269647	10366855	A	0	0	G
11	BICF2P330520	10376576	A	0	0	G
11	BICF2S22926483	10398067	A	0	0	G
11	BICF2P857336	10429289	G	0	0	A
11	BICF2P813843	10493498	0	0	0	A
11	BICF2P1168021	10522306	G	0	0	A
11	BICF2P1388743	10537956	A	0	0	G
11	BICF2P517621	10556010	G	0.25	0.2143	A
11	TIGRP2P143862_rs9236609	10578183	0	0	0	A
11	TIGRP2P143860_rs8928376	10587742	0	0	0	G
11	BICF2P652643	10606231	G	0	0	A
11	BICF2S2346933	10628495	A	0.5	0.1429	G
11	BICF2P603521	10639269	A	0.5	0.1429	G
11	BICF2S23311946	10650040	A	0.5	0.1429	G
11	BICF2S23725005	10660728	G	0.5	0.1429	A
11	BICF2P907243	10676988	A	0	0	G
11	BICF2P950535	10685349	G	0	0	A
11	BICF2P943776	10698810	G	0	0	A
11	BICF2P896857	10712715	A	0	0	T
11	BICF2P813269	10714654	A	0	0	C
11	BICF2P434950	10720693	C	0	0	A
11	TIGRP2P143775_rs8983798	10733186	0	0	0	G
11	TIGRP2P143773_rs8686387	10741443	0	0	0	A
11	BICF2P1179027	10764657	0	0	0	A
11	TIGRP2P143770_rs9075470	10807238	0	0	0	C
11	BICF2P240708	10831053	G	0.5	0.8571	A
11	BICF2S2375692	10847813	0	0	0	A
11	TIGRP2P143740_rs8682444	10873092	A	0	0.2143	C
11	BICF2P1032952	10917608	0	0	0	G
11	TIGRP2P143719_rs9063853	10937466	A	1	0.8333	C

11	BICF2P537892	10956970	0	0	0	A
11	BICF2P905801	10970107	A	0	0	G
11	BICF2P1316073	10980119	G	0	0	A
11	TIGRP2P143710_rs9094560	10992177	0	0	0	G
11	BICF2P1361525	11004366	G	0.75	0.7857	A
11	TIGRP2P143705_rs8955063	11024616	0	0	0	G
11	BICF2P908877	11031152	A	1	1	G
11	BICF2S23427144	11043187	G	0.5	0.8571	C
11	BICF2P554507	11062708	A	0.5	0.8571	G
11	BICF2P600260	11069741	A	0.5	0.8571	G
11	BICF2P189495	11071523	A	0.5	0.8571	G
11	BICF2P189496	11071686	C	0.5	0.8571	G
11	TIGRP2P143682_rs9141144	11084586	A	0.5	0.8571	C
11	BICF2S22929038	11087996	G	0.5	0.8571	A
11	BICF2P161849	11115293	G	0.5	0.8571	A
11	TIGRP2P143667_rs9156200	11139211	A	1	1	G
11	TIGRP2P143664_rs8778490	11155240	G	1	1	A
11	BICF2P172144	11194638	0	0	0	G
11	BICF2P879853	11213398	0	0	0	G
11	BICF2P1246721	11236925	0	0	0	G
11	BICF2S2356511	11266047	A	0.5	0.8571	G
11	BICF2P27672	11403968	G	0.25	0.2143	A
11	TIGRP2P143307_rs8760159	11413161	A	0.5	0.1429	G
11	BICF2P902821	11420641	G	0	0	A
11	BICF2S23659394	11445458	A	0	0	C
11	BICF2S23649057	11454591	G	0.25	0.6429	A
11	BICF2P1406502	11501003	0	0	0	G
11	BICF2P648670	11516895	A	0	0	T
11	BICF2P561488	11540379	A	0	0	C
11	BICF2P251123	11574970	A	0.5	0.1429	G
11	BICF2P736413	11583869	G	0.25	0.6429	A
11	BICF2P53018	11594538	A	0	0	G
11	BICF2P358794	11605521	0	0	0	A
11	BICF2P1236789	11626254	C	0.75	0.3571	A
11	BICF2P1236783	11627455	G	0.75	0.3571	A
11	BICF2P492568	11640230	A	0.5	0.1429	G
11	TIGRP2P143259_rs8523491	11661513	G	0.75	0.7857	A
11	TIGRP2P143254_rs9069524	11686594	G	0.25	0.2143	A
11	BICF2P1035249	11718405	0	0	0	G
11	BICF2P1232126	11735084	A	0.25	0.2143	G
11	BICF2P787687	11759492	A	0.75	0.7857	G
11	BICF2P622412	11776896	0	0	0	A
11	BICF2P461273	11804524	A	1	0.7857	G
11	BICF2P1008862	11852704	0	0	0	A
11	BICF2P572679	11867703	G	0	0	A
11	BICF2P572680	11867758	G	0	0	C
11	BICF2P493988	11875309	A	0	0	G

11	TIGRP2P143232_rs8708294	11887350	A	0	0	T
11	BICF2S2439215	11900186	A	0	0	G
11	BICF2P1272321	11921698	G	0	0	A
11	BICF2G63027171	11983646	A	0.5	0.6429	G
11	BICF2G630287100	12172736	G	0.25	0.1667	A
11	BICF2G630287171	12182796	A	0.25	0.2143	G
11	BICF2G630287225	12190892	G	0	0	A
11	BICF2G630287518	12275750	0	0	0	G
11	BICF2G630288477	12650626	G	0.25	0.6429	A
11	TIGRP2P145392_rs9253708	12673055	G	0.25	0.6429	A
11	TIGRP2P145068_rs9144596	12805414	0	0	0	A
11	BICF2P487538	12843841	G	0	0	A
11	BICF2P499114	12845857	A	0.5	0.6429	C
11	BICF2P1000382	12867969	A	0	0	G
11	BICF2P1000379	12868874	A	0	0	G
11	TIGRP2P145061_rs9212679	12885246	A	0	0	G
11	BICF2P1363201	12894256	G	0.5	0.6429	A
11	TIGRP2P145042_rs8996851	12903743	G	0.5	0.6429	A
11	BICF2S23037520	12924948	A	0.5	0.8571	C
11	BICF2P1121645	12950208	A	0	0	T
11	BICF2P382427	12959928	G	0.5	0.8571	A
11	BICF2P286807	12970950	A	0.5	0.8571	G
11	BICF2P1439522	12975783	0	0	0	G
11	BICF2P116362	12993360	0	0	0	A
11	BICF2P302412	13008065	C	0.5	0.8571	A
11	BICF2P1204535	13043617	A	0	0	G
11	BICF2P941128	13065274	G	0	0	A
11	BICF2P1291842	13091486	G	0	0	A
11	TIGRP2P144985_rs9185164	13121296	G	0	0.2143	A
11	BICF2P1250067	13157772	C	0.5	0.1429	A
11	TIGRP2P144969_rs8940467	13182706	G	0.5	0.1429	A
11	BICF2S23038530	13226024	G	0.5	0.1429	A
11	BICF2P283198	13241795	0	0	0	C
11	TIGRP2P144966_rs8873137	13259038	A	0	0	G
11	BICF2G630288899	13272603	A	0.5	0.1429	G
11	BICF2G630288912	13282918	C	0.5	0.1429	A
11	BICF2P44050	13338976	A	0.5	0.1429	G
11	BICF2S22917577	13356349	G	0.25	0.2143	A
11	BICF2P1219809	13370898	G	0.25	0.2143	A
11	BICF2P984488	13375253	G	0.25	0.2143	A
11	BICF2S2362334	13386695	G	0.25	0.2143	A
11	BICF2P164587	13411399	G	0.25	0.2143	A
11	TIGRP2P144898_rs9036606	13479763	A	0	0	G
11	BICF2G630288995	13504153	A	0	0	G
11	BICF2G630288999	13544944	A	0.5	0.1429	G
11	BICF2P574977	13559613	0	0	0	G
11	BICF2P830989	13811903	A	0.75	0.75	G

11	BICF2P51314	14181013	G	0	0	A
11	BICF2G630290157	14192846	A	0.25	0.1667	G
11	TIGRP2P146310_rs9227582	14205136	A	0.5	0.875	C
11	BICF2G630290317	14228128	G	0	0	A
11	BICF2G630290384	14237040	G	0.75	0.8333	A
11	BICF2G630290672	14306016	G	0.25	0.2143	A
11	BICF2G630291135	14449165	A	0	0	G
11	BICF2G630291663	14589052	A	0	0.1	G
11	BICF2G630291770	14605982	A	0	0.1667	C
11	BICF2G630291870	14631071	G	0	0.25	A
11	BICF2G630291939	14651422	G	0	0.25	A
11	BICF2S2459118	14732716	A	0	0	G
11	BICF2P1412705	14848716	A	0	0	G
11	BICF2G630292303	14885852	C	0.5	0.1429	A
11	BICF2G630292309	14901405	C	0.5	0.1429	A
11	BICF2G630292311	14918364	C	0	0	A
11	BICF2G630292315	14946104	G	0.5	0.3571	A
11	BICF2G630292318	14952108	G	0.5	0.3571	A
11	BICF2G630292322	14966785	A	0.5	0.3571	G
11	BICF2P1212252	14979188	A	NA	0.3571	G
11	BICF2G630292332	14999125	A	0.5	0.3571	G
11	BICF2S23215906	15029087	A	0	0	C
11	BICF2G630292347	15041235	A	0.5	0.3571	G
11	BICF2G630292355	15050026	A	1	1	G
11	BICF2G630292358	15059651	G	0	0	A
11	BICF2G630292367	15076531	A	0	0	G
11	BICF2S22962891	15100262	G	0.5	0.6429	A
11	BICF2G630292394	15129159	C	0	0	A
11	BICF2P426661	15150958	A	0	0	G
11	BICF2G630292470	15188946	G	0	0.2143	A
11	BICF2G630292533	15226802	A	0	0	G
11	BICF2G630292538	15237204	A	0	0	G
11	BICF2G630292544	15240338	A	0	0.2143	G
11	BICF2G630292551	15250595	A	1	0.7857	G
11	TIGRP2P147455_rs8602758	15261663	G	0	0	A
11	BICF2G630292561	15273294	G	0	0.2143	A
11	BICF2G630292569	15293588	G	0	0.2143	A
11	BICF2G630292579	15307687	A	0	0	G
11	BICF2P362572	15319790	G	0	0.2143	A
11	BICF2G630292587	15336849	G	0.5	0.1429	A
11	TIGRP2P147482_rs9247961	15342194	0	0	0	G
11	BICF2G630292602	15359104	A	0.5	0.8571	G
11	BICF2G630292603	15359617	G	0.5	0.8571	A
11	BICF2G630292617	15364172	G	0.5	0.8571	A
11	BICF2G630292648	15380298	A	1	1	G
11	BICF2G630292693	15408162	A	0	0	G
11	TIGRP2P147505_rs8652842	15426348	A	0	0	G

11	BICF2G630292734	15444884	A	0.5	0.9167	C
11	BICF2P1375262	15467534	A	0	0	C
11	BICF2P1025064	15484240	A	0	0	G
11	BICF2G630292744	15503040	A	0	0	G
11	BICF2G630292750	15540323	G	0	0	A
11	BICF2G630292756	15554530	A	0	0	G
11	BICF2G630292757	15560503	G	0	0	A
11	TIGRP2P147556_rs8710700	15578748	A	1	1	C
11	BICF2P293317	15582569	0	0	0	A
11	BICF2G630292760	15595122	A	0	0	G
11	BICF2S2335596	15610842	A	0.5	0.1429	G
11	BICF2P172793	15623513	C	0	0.2143	A
11	BICF2P622231	15633045	A	0.5	0.1429	G
11	TIGRP2P147568_rs8557609	15643171	A	0	0	G
11	BICF2P28000	15670912	0	0	0	G
11	BICF2P834421	15682912	0	0	0	A
11	BICF2P438825	15699798	0	0	0	A
11	TIGRP2P147603_rs9118677	15721818	A	0	0	G
11	BICF2P1130863	15733388	T	0	0	A
11	TIGRP2P147617_rs8711990	15751021	G	0	0	A
11	TIGRP2P147619_rs8823613	15755384	G	0	0	A
11	BICF2P57863	15773336	0	0	0	A
11	TIGRP2P147692_rs8730816	15796447	G	0.5	0.3571	A
11	TIGRP2P147693_rs8590186	15801312	0	0	0	A
11	BICF2P365517	15814465	G	0.5	0.3571	A
11	BICF2P759341	15836086	0	0	0	G
11	BICF2P675972	15847620	0	0	0	C
11	BICF2P1245754	15882374	G	0.5	0.6429	A
11	BICF2S23325890	15895610	A	0.5	0.1429	G
11	BICF2G630292798	15908515	A	0.5	0.6429	G
11	BICF2G630292801	15909365	A	0.5	0.6429	G
11	BICF2G630292831	15926136	0	0	0	G
11	BICF2P869707	15930625	G	NA	0	A
11	BICF2G630292847	15939865	G	0	0	A
11	BICF2P451372	15965158	G	0.5	0.1429	A
11	TIGRP2P147756_rs8549028	15989322	0	0	0	G
11	TIGRP2P147759_rs8689965	15998249	A	0.5	0.8571	G
11	BICF2P551233	16008616	A	0	0	C
11	BICF2P868646	16027694	A	1	1	C
11	BICF2S23726024	16043293	A	0.5	0.1429	G
11	BICF2G630292874	16060048	0	0	0	G
11	BICF2P1128038	16060458	G	0	0	A
11	TIGRP2P147802_rs8850336	16066614	G	0	0	A
11	BICF2G630292879	16079218	0	0	0	G
11	BICF2G630292889	16093502	C	0	0	A
11	BICF2G630292892	16093654	A	0	0	G
11	BICF2P1161507	16107113	0	0	0	A

11	BICF2G630292922	16110176	A	0	0	G
11	BICF2G630292923	16110198	C	0	0	A
11	BICF2G630292934	16118044	A	1	1	G
11	BICF2G630292953	16131639	A	1	1	G
11	TIGRP2P147810_rs8558234	16139699	A	1	1	G
11	BICF2P1069469	16161309	0	0	0	A
11	BICF2G630292963	16180778	A	0	0.2143	G
11	BICF2P1276082	16203584	0	0	0	A
11	BICF2S23511471	16215647	A	0	0	G
11	BICF2P1020536	16254290	A	0	0.2143	G
11	BICF2G630292968	16268212	A	0	0	T
11	BICF2G630292972	16276040	G	0	0	A
11	BICF2G630293049	16319080	A	0	0	G
11	BICF2G630293076	16332547	G	0.5	0.8571	A
11	BICF2G630293078	16339940	A	0.5	0.6429	G
11	BICF2G630293144	16376561	G	1	0.7	A
11	TIGRP2P147940_rs8864445	16387559	A	0	0.2143	G
11	BICF2G630293191	16402832	C	0.5	0.6429	A
11	BICF2P766408	16413910	C	0.5	0.6667	A
11	TIGRP2P147942_rs9157470	16423361	0	0	0	G
11	BICF2P953808	16440917	G	0	0.2143	A
11	BICF2P388825	16445027	0	0	0	A
11	BICF2G630293222	16461381	0	0	0	G
11	BICF2P173554	16470076	0	0	0	A
11	BICF2S23346640	16486976	G	0.5	0.6429	A
11	BICF2G630293237	16497743	G	0.5	0.6429	A
11	BICF2G630293242	16505277	A	0.5	0.6429	G
11	BICF2G630293244	16515263	A	0.5	0.6429	C
11	BICF2P202248	16533044	G	0.5	0.6429	A
11	BICF2S2358377	16537019	G	0	0	A
11	BICF2G630293247	16550620	A	0	0	G
11	BICF2S23549178	16565912	T	0	0	A
11	BICF2G630293254	16596154	0	0	0	C
11	BICF2P693130	16614363	G	0	0	A
11	BICF2S2302994	16620400	A	0	0	G
11	BICF2G630293271	16632868	G	0	0	A
11	BICF2G630293272	16633047	G	0	0	A
11	TIGRP2P147982_rs9133980	16645501	A	0	0.2143	G
11	BICF2S23116542	16654514	C	1	0.7857	G
11	BICF2P205003	16679868	A	1	0.7857	G
11	BICF2P665812	16702376	0	0	0	G
11	G661f44S349	16728018	0	0	0	G
11	BICF2P35573	16752388	A	0.5	0.6429	G
11	TIGRP2P148004_rs9065573	16774219	0	0	0	G
11	BICF2G630293293	16781748	G	0	0	A
11	BICF2P1246998	16791824	G	0.5	0.1429	A
11	BICF2P55319	16813521	A	0	0	G

11	BICF2P953255	16829245	0	0	0	G
11	BICF2P919808	16847411	A	1	1	G
11	BICF2S23032402	16860218	G	0	0	A
11	BICF2P818158	16872074	0	0	0	A
11	BICF2P989944	16887154	C	0.5	0.8571	A
11	BICF2P463587	16900858	G	0	0	A
11	BICF2S23660185	16912872	A	0	0	G
11	BICF2S23630769	16920361	A	0	0	G
11	BICF2S23043047	16935031	G	0	0	A
11	BICF2S23431826	16959263	A	0	0	G
11	BICF2G630293306	16968579	G	0	0	A
11	BICF2P895870	16984343	A	0	0	G
11	BICF2S23334793	16992660	A	0.5	0.8571	G
11	BICF2S23911017	17017467	G	0	0	A
11	BICF2P402337	17020222	0	0	0	A
11	BICF2S23252938	17038703	C	0	0	A
11	BICF2S23119597	17054271	A	0	0	G
11	BICF2P1419595	17058442	0	0	0	G
11	BICF2S22910870	17074749	A	0	0	G
11	BICF2P509495	17081729	G	0	0	A
11	BICF2P421357	17096037	G	0	0	A
11	BICF2P90493	17107032	A	0	0	G
11	BICF2P445669	17118788	G	0	0	A
11	BICF2G630293309	17126826	0	0	0	C
11	BICF2G630293330	17136139	A	0.5	0.8571	G
11	BICF2G630293338	17148215	A	0.5	0.8571	G
11	BICF2G630293354	17162525	A	0.5	0.1429	C
11	BICF2G630293366	17179950	A	0.5	0.1429	C
11	BICF2G630293370	17188897	A	0.5	0.8571	G
11	BICF2G630293381	17193700	C	0	0	A
11	BICF2S2306554	17199715	T	0	0	A
11	BICF2S24410883	17206790	T	1	1	A
11	BICF2G630293407	17217768	T	0.5	0.1429	A
11	BICF2S23527290	17229150	G	0	0	A
11	BICF2S23234577	17250223	A	0	0	G
11	BICF2S24511779	17269747	G	0	0	A
11	BICF2P401162	17272602	A	0	0	G
11	BICF2P401161	17273091	A	0.5	0.8571	G
11	BICF2P1390942	17293069	A	0.5	0.8571	G
11	BICF2P1390945	17293199	G	0.5	0.8571	A
11	BICF2G630293426	17300978	A	0	0	C
11	BICF2S23336786	17301569	A	0	0	G
11	BICF2G630293438	17308924	A	0.5	0.1429	G
11	BICF2P784489	17325124	A	0	0	G
11	BICF2G630293468	17334238	A	0.5	0.8571	G
11	BICF2G630293478	17344955	A	0	0	C
11	BICF2S23256177	17353593	G	0.5	0.8571	A

11	BICF2P334123	17366477	A	0	0	T
11	BICF2P588281	17398670	A	0.5	0.1429	G
11	BICF2S23312504	17420880	0	0	0	C
11	BICF2G630293497	17427187	C	0	0	A
11	BICF2G630293525	17441707	A	1	0.7857	G
11	BICF2G630293540	17456510	A	0.5	0.1429	G
11	BICF2G630293556	17475378	A	0	0.2143	G
11	BICF2S23713485	17483707	G	0.5	0.08333	A
11	BICF2G630293576	17495391	G	0.5	0.1429	A
11	BICF2G630293594	17508042	A	0	0	G
11	BICF2G630293604	17523652	A	0	0	C
11	BICF2P1070138	17536102	A	0.5	0.6429	G
11	BICF2P1070141	17538032	A	0.5	0.6429	G
11	BICF2G630293634	17556139	A	0.5	0.6429	G
11	BICF2P81696	17570982	0	0	0	G
11	BICF2P518357	17581211	G	1	1	A
11	TIGRP2P148033_rs8884260	17619273	0	0	0	C
11	BICF2P375840	17649550	0	0	0	G
11	BICF2P110857	17669692	A	0.5	0.1429	G
11	BICF2P730985	17678808	G	0	0	A
11	BICF2G630293644	17694347	A	0	0.2143	G
11	BICF2P1187113	17702881	A	0	0	G
11	BICF2S23416435	17713343	G	0.5	0.7	A
11	BICF2S22946719	17716730	A	0.5	0.6429	G
11	BICF2S23617070	17730295	A	0	0	G
11	BICF2P328115	17753652	0	0	0	A
11	BICF2G630293675	17762076	A	0	0.2143	G
11	BICF2P1138073	17772069	0	0	0	G
11	BICF2G630293676	17789329	G	0	0	C
11	BICF2G630293684	17808565	A	0.5	0.6429	G
11	BICF2G630293705	17823791	G	0	0	A
11	BICF2G630293708	17825506	A	0.5	0.3	G
11	BICF2G630293714	17839474	G	0.5	0.6429	A
11	BICF2G630293731	17848467	G	0	0	A
11	BICF2G630293753	17863756	G	0.5	0.1429	A
11	BICF2G630293768	17875726	G	0.5	0.1429	A
11	BICF2G630293780	17884525	C	0.5	0.1429	A
11	BICF2G630293799	17895762	C	0.5	0.1429	A
11	BICF2G630293818	17908969	A	0.5	0.1429	G
11	BICF2G630293849	17929862	G	0	0	A
11	BICF2P110934	17947152	0	0	0	A
11	BICF2G630293850	17962661	A	0	0	G
11	BICF2P266130	17970006	G	0.5	0.1429	A
11	BICF2G630293857	17981647	A	0.5	0.1429	G
11	BICF2S23036178	17994846	C	0	0	G
11	BICF2G630293859	17999413	G	0.5	0.5	A
11	BICF2G630293875	18013440	G	0.5	0.1429	A

11	BICF2S23045649	18035367	0	0	0	G
11	BICF2P351614	18043650	A	1	1	G
11	BICF2P19258	18061101	A	1	1	G
11	BICF2G630293889	18086068	G	0.5	0.5	A
11	BICF2S23534753	18110454	0	0	0	A
11	BICF2P843027	18117079	0	0	0	A
11	BICF2G630293902	18148835	G	1	1	A
11	BICF2G630293909	18167986	A	0.5	0.5	G
11	BICF2G630293914	18185175	0	0	0	A
11	BICF2G630293927	18202467	0	0	0	G
11	BICF2G630293933	18213642	A	0	0	G
11	BICF2G630293934	18213948	0	0	0	G
11	BICF2G630293939	18219097	G	1	1	A
11	BICF2G630293965	18236239	A	1	1	C
11	BICF2G630293968	18240332	G	1	1	A
11	BICF2G630293982	18251938	A	1	1	G
11	BICF2G630293990	18269911	0	0	0	A
11	BICF2P956108	18273917	0	0	0	A
11	BICF2G630293995	18290528	A	0	0	G
11	BICF2G630294031	18316724	C	1	1	A
11	BICF2G630294051	18339255	C	0	0	A
11	BICF2G630294055	18343083	A	0	0	G
11	BICF2P1140371	18358355	A	1	1	T
11	BICF2G630294082	18381333	A	1	1	G
11	BICF2G630294096	18389176	A	1	1	G
11	BICF2G630294130	18405775	G	0.5	0.5	A
11	BICF2G630294145	18413721	0	0	0	T
11	BICF2G630294149	18430151	A	0.5	0.5	G
11	BICF2G630294152	18435148	G	0.5	0.5	A
11	BICF2G630294163	18456618	A	0.5	0.5	G
11	BICF2G630294203	18482294	G	0.5	0.1429	C
11	BICF2G630294211	18495327	C	0.5	0.8571	G
11	BICF2G630294216	18503760	G	0.5	0.1429	A
11	BICF2G630294224	18516891	A	0.5	0.5	G
11	BICF2S2453933	18537529	G	1	0.6429	A
11	BICF2G630294236	18538438	A	1	1	G
11	BICF2G630294243	18551716	A	0	0	G
11	BICF2G630294248	18555184	A	0	0	G
11	BICF2P1362816	18579634	A	0	0	G
11	BICF2G630294284	18586488	G	0	0	A
11	BICF2G630294297	18600776	A	0.5	0.8571	C
11	BICF2G630294329	18620309	C	0.5	0.1429	A
11	BICF2G630294344	18634034	G	0	0	A
11	BICF2G630294349	18643939	A	0.5	0.1429	G
11	BICF2G630294375	18654005	G	0	0	A
11	BICF2P199564	18673669	C	0	0	A
11	BICF2G630294406	18679940	A	0	0	G

11	BICF2G630294409	18689494	G	0.5	0.1429	A
11	BICF2G630294430	18703872	A	0.5	0.1429	C
11	BICF2G630294452	18721003	A	0	0	G
11	BICF2G630294459	18727147	A	1	0.6429	C
11	BICF2S22917717	18740733	G	0	0.3571	A
11	BICF2S22927034	18752132	A	0	0	T
11	BICF2P24975	18752560	A	0	0	G
11	BICF2S23726677	18758035	0	0	0	G
11	BICF2G630294517	18771556	G	0.5	0.1429	A
11	BICF2P709432	18786354	G	0.5	0.5	A
11	BICF2S23436979	18788347	G	0.5	0.5	A
11	BICF2S23436978	18788529	A	0.5	0.5	C
11	BICF2P560627	18794064	0	0	0	A
11	BICF2G630294542	18803152	G	1	1	A
11	BICF2G630294559	18824284	A	0.5	0.1429	C
11	BICF2G630294567	18833712	A	0	0	G
11	BICF2G630294577	18843807	A	0	0	G
11	BICF2G630294603	18862390	A	0	0	G
11	BICF2G630294618	18873551	G	0	0	A
11	BICF2G630294632	18887070	A	0	0	G
11	BICF2G630294651	18903531	G	0.5	0.2143	A
11	BICF2G630294653	18913755	A	0	0	T
11	BICF2G630294659	18930831	A	0.5	0.2143	G
11	BICF2G630294675	18945047	G	0	0	A
11	BICF2G630294686	18953123	G	0.5	0.2143	A
11	BICF2G630294724	18965806	G	0	0	A
11	BICF2P428495	18988414	A	0.5	0.1429	C
11	BICF2G630294741	18999416	G	0.5	0.8571	A
11	BICF2G630294746	19010423	A	0.5	0.4286	G
11	BICF2G630294750	19025463	A	0.5	0.4286	C
11	BICF2G630294756	19034571	A	0.5	0.4286	C
11	BICF2G630294770	19055170	A	0.5	0.4286	C
11	BICF2G630294787	19071837	T	1	0.7143	A
11	BICF2P270204	19080791	A	0.5	0.5714	T
11	BICF2G630294814	19091032	A	0.5	0.4286	G
11	BICF2S236113	19101800	G	0.5	0.1429	A
11	BICF2G630294836	19114139	A	0	0.2857	G
11	BICF2G630294854	19132047	T	0	0	A
11	BICF2G630294857	19137967	G	0.5	0.4286	A
11	BICF2G630294874	19147686	G	0	0	A
11	BICF2S23423737	19161486	A	0.5	0.4286	G
11	BICF2P735901	19173685	A	0	0	C
11	BICF2P1057901	19177849	A	0	0	G
11	BICF2P151331	19191743	G	0.5	0.4286	A
11	BICF2P151332	19191965	A	0.5	0.4286	G
11	BICF2G630294922	19212509	A	0	0	G
11	BICF2G630294928	19219236	A	0.5	0.5	G

11	BICF2P1025070	19236367	A	1	0.6429	C
11	BICF2P371803	19244268	G	0	0	A
11	BICF2P649308	19257565	A	0	0	G
11	BICF2G630294943	19272876	A	0.5	0.5	G
11	BICF2G630294952	19275110	G	0.5	0.5	A
11	BICF2G630294965	19290128	A	0.5	0.5	C
11	BICF2G630294984	19303580	A	0	0.2857	C
11	BICF2P1283469	19309826	A	0	0	G
11	BICF2P996832	19330631	G	0	0	A
11	BICF2P996836	19331696	A	0.5	0.5	G
11	BICF2P773359	19354018	A	0	0	G
11	BICF2P966539	19371628	A	0	0	G
11	BICF2G630295011	19377552	A	0.5	0.5	C
11	BICF2S23120899	19391467	G	0.5	0.2143	A
11	BICF2G630295034	19423728	A	0.5	0.1429	T
11	BICF2G630295039	19428887	C	0	0.3571	G
11	BICF2P365017	19435848	A	0.5	0.5	C
11	BICF2G630295055	19435915	A	0.5	0.1429	C
11	BICF2G630295070	19453591	A	0.5	0.1429	G
11	BICF2S23639422	19478632	A	0	0	G
11	BICF2P395372	19488082	G	0.5	0.2143	A
11	BICF2G630295111	19494299	A	0	0.6429	G
11	BICF2G630295122	19504479	A	0.5	0.1429	C
11	BICF2G630295131	19513517	A	0	0.2857	C
11	BICF2G630295137	19517500	G	0	0	A
11	BICF2G630295152	19527377	A	0	0	G
11	BICF2G630295168	19543196	G	0	0	A
11	BICF2S2344073	19551846	G	0.5	0.25	C
11	BICF2G630295198	19565561	A	0.5	0.3571	G
11	BICF2G630295220	19575460	A	0.5	0.2143	G
11	BICF2G630295233	19590464	A	0	0	G
11	BICF2G630295236	19595751	G	0	0.6429	A
11	BICF2G630295242	19606558	A	0	0	G
11	BICF2G630295247	19610187	A	1	0.3571	C
11	BICF2G630295267	19621547	G	0.5	0.7857	A
11	BICF2S23523600	19663755	A	0	0	C
11	BICF2S23338718	19667872	G	0	0.3571	A
11	BICF2G630295272	19680649	0	0	0	G
11	BICF2P539905	19695834	A	0	0.6429	G
11	BICF2P1310910	19699968	A	0	0.6429	G
11	BICF2P327835	19711469	G	0	0.3571	A
11	BICF2G630295282	19732403	G	0.5	0.8571	A
11	BICF2G630295301	19763127	C	0	0.6429	G
11	BICF2G630295303	19768918	A	0.5	0.1429	C
11	BICF2G630295322	19791585	A	1	0.3571	G
11	BICF2G630295335	19805514	0	0	0	A
11	BICF2G630295339	19830104	A	0	0.3571	G

11	BICF2P316322	19857005	A	1	0.7143	G
11	BICF2G630295378	19861239	T	0	0	A
11	BICF2P1115743	19871765	G	1	0.3571	A
11	BICF2G630295396	19874541	A	0	0	G
11	BICF2P931039	19880988	A	0	0.6429	C
11	BICF2G630295406	19882857	A	0	0	G
11	BICF2G630295410	19885164	C	1	0.3571	A
11	BICF2G630295432	19897678	G	1	0.3571	C
11	BICF2P544957	19913744	A	0	0.2857	G
11	BICF2G630295453	19919173	A	0.5	0.5714	G
11	BICF2G630295482	19932009	A	1	0.7143	G
11	BICF2G630295494	19941563	A	0.5	0.1429	G
11	BICF2P928741	19969802	G	0.5	0.5	A
11	BICF2P1162901	19971880	G	1	0.7143	A
11	BICF2G630295523	19980914	G	1	0.6429	A
11	BICF2G630295527	19992941	G	0	0	A
11	BICF2G630295546	20000789	G	0	0	A
11	BICF2P1248023	20014381	A	0	0	G
11	BICF2G630295561	20038791	C	0.5	0.1429	A
11	BICF2G630295566	20047237	A	0.5	0.5	C
11	BICF2S23033111	20056580	C	0.5	0.1429	A
11	BICF2G630295582	20068179	G	0.5	0.1429	A
11	BICF2G630295597	20089514	G	0	0.3571	A
11	BICF2G630295607	20104244	A	1	0.7143	G
11	BICF2G630295619	20115707	A	1	0.75	G
11	BICF2G630295621	20132853	A	0.5	0.1429	C
11	BICF2G630295626	20138489	G	0.5	0.1429	A
11	BICF2G630295657	20158990	G	0	0	A
11	BICF2G630295663	20164785	G	0	0	A
11	BICF2G630295685	20174069	A	0	0	G
11	BICF2G630295703	20197034	A	0	0	C
11	BICF2P913824	20210746	G	0.5	0.4286	A
11	BICF2G630295718	20226024	A	0	0	C
11	BICF2G630295725	20235606	G	0	0	A
11	BICF2P926015	20237216	0	0	0	A
11	BICF2S23220673	20241197	A	0.5	0.2143	C
11	BICF2G630295747	20262748	G	0.5	0.7857	C
11	BICF2P937396	20276093	A	0.5	0.2143	G
11	BICF2G630295769	20288891	A	0	0.2857	G
11	BICF2P673357	20292116	0	0	0	G
11	BICF2G630295787	20298013	A	0.5	0.5	G
11	BICF2P315052	20312127	G	1	1	A
11	BICF2G630295824	20323443	A	0.5	0.5714	G
11	TIGRP2P148087_rs8611965	20333613	0	0	0	G
11	BICF2P1451901	20345147	0	0	0	G
11	BICF2G630295849	20345294	G	0.5	0.1429	A
11	BICF2G630295857	20354945	A	0.5	0.1429	T

11	BICF2G630295864	20360350	G	0.5	0.1429	A
11	BICF2G630295866	20361043	G	0.5	0.2143	A
11	BICF2G630295868	20371379	A	0.5	0.1667	G
11	BICF2G630295873	20387161	G	0.5	0.2143	A
11	BICF2G630295882	20394351	A	0.5	0.2143	G
11	BICF2P293356	20411470	A	1	0.3571	C
11	BICF2S23423639	20426647	A	0.5	0.2143	G
11	BICF2G630295918	20436742	A	0.5	0.1429	G
11	BICF2G630295925	20451977	G	0.5	0.2143	A
11	BICF2G630295930	20464259	G	0.5	0.1429	A
11	BICF2G630295932	20472644	G	0.5	0.1429	A
11	BICF2P185213	20488368	G	0	0.6429	A
11	BICF2G630295961	20488525	G	0.5	0.1429	A
11	BICF2G630295972	20497962	C	0.5	0.1429	G
11	BICF2P776428	20505580	A	0.5	0.2143	G
11	BICF2S23235200	20516034	A	0.5	0.2143	G
11	BICF2G630296021	20533360	G	0.5	0.1429	A
11	BICF2G630296037	20539934	A	0.5	0.08333	G
11	BICF2G630296043	20554714	A	0.5	0.2143	G
11	BICF2P192213	20573071	G	0.5	0.5	A
11	BICF2P209873	20577123	A	0.5	0.2143	G
11	BICF2P528224	20586616	C	0.5	0.1667	A
11	BICF2S2368242	20601796	A	1	0.7143	G
11	BICF2S23548912	20629209	G	0.5	0.08333	A
11	BICF2S23141428	20631719	A	0.5	0.1429	G
11	BICF2P1349400	20643054	C	0.5	0.08333	A
11	BICF2S2303223	20661188	A	0.5	0.1429	G
11	BICF2P330067	20666455	A	0	0	C
11	BICF2P508985	20679551	G	0.5	0.1429	A
11	BICF2P71271	20692673	A	0.5	0.5	G
11	BICF2P871369	20700625	G	0.5	0.5	A
11	BICF2S23730380	20715584	C	0	0.6429	G
11	BICF2P1342439	20740244	A	0	0.2857	C
11	BICF2P686280	20748799	A	0.5	0.1429	G
11	BICF2P124153	20763075	G	0	0.3571	A
11	BICF2P701	20770006	C	0.5	0.4286	A
11	BICF2S24415651	20796364	G	0	0.2857	A
11	BICF2P620196	20806549	C	0.5	0.5714	A
11	BICF2S23018347	20816393	A	0.5	0.4286	G
11	BICF2S23054915	20830359	G	0.5	0.5714	A
11	BICF2P640630	20840849	0	0	0	G
11	BICF2P979350	20850097	0	0	0	A
11	BICF2S23329555	20871523	A	0.5	0.1429	C
11	BICF2P55387	20876796	A	0	0	G
11	BICF2P461569	20884876	A	0.5	0.1429	G
11	BICF2P718382	20887666	G	0.5	0.1429	A
11	BICF2S23410102	20905473	A	0	0	G

11	BICF2S23410100	20905591	G	0	0	A
11	BICF2S22926672	20916974	A	0	0	G
11	BICF2S22926024	20920934	C	0.5	0.1429	A
11	BICF2P321292	20931783	G	0.5	0.1429	A
11	BICF2P286516	20943171	A	0.5	0.1429	G
11	BICF2P878837	20962099	G	0.5	0.7857	A
11	BICF2P296900	20974727	G	0.5	0.1429	A
11	BICF2P691930	21002974	G	0.5	0.5	A
11	BICF2P888442	21013506	A	0.5	0.5	G
11	BICF2S237332	21037442	A	0.5	0.5	G
11	BICF2P1320088	21046559	A	0.5	0.5	G
11	BICF2S23036108	21057588	G	0	0.2857	A
11	BICF2P813776	21068122	G	0	0	A
11	BICF2P146129	21090648	G	0.5	0.2143	C
11	BICF2P588166	21106176	A	0.5	0.5	G
11	BICF2S23436060	21115548	A	0.5	0.5	C
11	BICF2P1184037	21145171	A	0.5	0.5	G
11	BICF2P155321	21164474	0	0	0	G
11	BICF2S2369315	21172501	G	0.5	0.5	A
11	BICF2S2314915	21187619	A	0.5	0.5	G
11	BICF2S22957845	21204022	G	0.5	0.5	A
11	BICF2P780026	21217980	0	0	0	T
11	BICF2S2449908	21229344	A	0.5	0.5	C
11	BICF2P1045595	21231365	A	0.5	0.7857	G
11	BICF2P218661	21242536	G	0	0.2857	A
11	BICF2P893687	21262623	C	0.5	0.5	A
11	BICF2P1018750	21273655	A	0	0.2857	G
11	BICF2P256262	21275205	A	0.5	0.2143	G
11	BICF2P551986	21289090	G	0.5	0.2143	A
11	BICF2P1088502	21293746	A	0.5	0.7857	G
11	BICF2G630296074	21305424	G	0.5	0.7857	A
11	BICF2G630296084	21311139	C	0.5	0.7857	G
11	BICF2G630296097	21323724	G	0	0	A
11	BICF2S23643044	21334121	A	0	0	G
11	BICF2G630296151	21349033	A	1	0.7143	G
11	BICF2S23424668	21362051	A	0	0	T
11	BICF2G630296161	21378912	G	0	0	A
11	BICF2G630296171	21390635	A	0	0	G
11	BICF2P976361	21414114	0	0	0	G
11	BICF2G630296192	21427157	G	0	0	A
11	BICF2P1170256	21446033	A	0	0	G
11	BICF2S22934385	21457814	C	0	0	A
11	BICF2P36004	21461927	A	0	0	C
11	TIGRP2P148105_rs8937602	21476758	0	0	0	A
11	BICF2P247877	21488182	G	0	0	A
11	BICF2P1291028	21504981	0	0	0	A
11	BICF2P889224	21513372	A	0.5	0.2143	G

11	BICF2P788111	21524299	0	0	0	G
11	BICF2P163083	21544158	G	0.5	0.5	A
11	BICF2P787554	21556020	A	0.5	0.5	G
11	BICF2P1066394	21562728	A	0.5	0.5	G
11	BICF2P735269	21583354	G	0	0	A
11	BICF2S23418452	21596161	A	0	0	G
11	BICF2P962745	21606854	A	0.5	0.5	G
11	BICF2P298471	21612389	G	1	1	A
11	BICF2P1255374	21622125	C	0	0	A
11	BICF2P1423859	21650406	A	0	0	G
11	BICF2S23553865	21667664	A	0	0	C
11	BICF2P912457	21677563	A	1	1	G
11	BICF2P1037814	21690213	C	0.5	0.5	A
11	BICF2S23519359	21707109	A	0	0	G
11	BICF2P1047236	21721244	A	0	0	G
11	BICF2S23158677	21728173	0	0	0	G
11	BICF2P137284	21737513	0	0	0	A
11	BICF2P1289	21748807	A	0	0	G
11	BICF2P467771	21758196	A	0.5	0.8571	G
11	BICF2P131909	21775911	A	0	0	G
11	BICF2P1049611	21781376	G	0	0	A
11	BICF2S23151308	21796136	A	0	0.2857	G
11	BICF2P981606	21818957	A	0	0.2857	C
11	BICF2P682281	21828687	A	0.5	0.4286	G
11	BICF2P683713	21843115	A	0	0	G
11	BICF2P377574	21857531	G	1	0.7143	A
11	BICF2P1222161	21867246	G	0	0.2857	A
11	BICF2P820002	21875250	A	0	0.2857	G
11	BICF2P71513	21879707	G	1	0.7143	A
11	BICF2P178507	21902450	A	0	0	G
11	BICF2P389783	21914982	A	0	0.2857	C
11	BICF2P1232948	21929517	G	0	0.2857	A
11	BICF2S23321729	21945763	A	0	0	G
11	BICF2P1171430	21954146	A	0	0.2857	G
11	BICF2P112299	21969781	A	0	0	G
11	BICF2P960409	21976857	A	0	0	G
11	BICF2P1128958	21997285	A	0	0	G
11	BICF2P125473	22007281	0	0	0	A
11	BICF2P682791	22014370	A	0	0.2857	G
11	TIGRP2P148111_rs9155029	22033882	0	0	0	G
11	BICF2P257354	22048998	0	0	0	A
11	BICF2P1439645	22067669	0	0	0	A
11	BICF2P932618	22074092	0	0	0	A
11	BICF2P462498	22101053	A	0	0.3333	G
11	BICF2P235304	22103422	G	0	0.3571	A
11	BICF2P651853	22127861	A	0	0	G
11	BICF2S23642197	22143363	A	0	0	G

11	BICF2S23057081	22155476	G	0	0	A
11	BICF2P405315	22166214	0	0	0	G
11	BICF2P353359	22184358	C	0	0	G
11	BICF2S23237996	22202112	A	0	0.3571	G
11	BICF2P84561	22215994	C	0.5	0.1429	A
11	BICF2P84578	22220226	G	0.5	0.5	A
11	BICF2P1324705	22235388	A	0.5	0.08333	C
11	BICF2P1409031	22251020	G	0.5	0.1429	A
11	BICF2P1376392	22257970	A	0.5	0.1429	C
11	BICF2P1068480	22266055	A	0.5	0.1429	G
11	BICF2P1456492	22278174	G	0.5	0.5	A
11	BICF2P375721	22288701	G	0.5	0.5	A
11	BICF2P980779	22299358	G	0	0	A
11	BICF2P1441250	22317022	G	0	0	A
11	BICF2S2357368	22331950	A	0	0	G
11	TIGRP2P148118_rs8832368	22355444	A	0	0	C
11	BICF2P1180025	22381904	A	0	0	G
11	BICF2P1179939	22391348	0	0	0	A
11	BICF2P613909	22405643	G	0	0	A
11	BICF2S24320279	22424240	G	1	0.7143	A
11	BICF2P1183899	22443228	0	0	0	A
11	TIGRP2P148145_rs9250226	22472195	A	0	0.2857	G
11	BICF2P772375	22507136	A	1	0.7143	G
11	BICF2P1104748	22519160	A	0	0	C
11	BICF2P1268241	22542119	A	1	0.7143	G
11	BICF2P1263191	22560590	C	0	0	A
11	BICF2P637875	22564459	G	0	0	A
11	BICF2S22950239	22577681	C	0	0	A
11	TIGRP2P148182_rs9049494	22604189	A	1	0.7143	G
11	BICF2P753231	22618504	A	0	0	G
11	BICF2P1317858	22622253	G	0	0	A
11	BICF2P83580	22633444	G	0	0	A
11	BICF2P1132579	22643683	G	1	0.7143	C
11	BICF2P620184	22672249	A	0	0	C
11	BICF2P896811	22697291	C	0	0	G
11	BICF2P286061	22715952	G	1	0.7143	A
11	BICF2P619871	22726133	G	0	0	A
11	BICF2P162373	22745515	A	0	0	G
11	BICF2P66074	22751071	A	1	0.7143	G
11	BICF2P1301563	22771903	G	1	0.7143	A
11	BICF2P1139513	22785692	G	1	0.7143	A
11	BICF2P1214132	22795378	G	1	0.7143	C
11	BICF2P935660	22808919	0	0	0	G
11	BICF2P632351	22818510	A	0	0.25	G
11	BICF2P160571	22851866	G	1	0.7143	A
11	BICF2P531315	22865853	A	0	0	C
11	BICF2P903604	22905143	G	1	0.7143	A

11	BICF2P701019	22916411	T	1	0.7143	A
11	BICF2P834413	22925544	A	0	0.2857	G
11	TIGRP2P148238_rs8934988	22941347	C	1	0.7143	A
11	BICF2P801078	22943743	G	1	0.7143	A
11	BICF2P1071742	22955945	G	0	0	A
11	TIGRP2P148250_rs8703911	22967784	A	1	0.7143	G
11	BICF2P799121	22982788	C	0.5	0.4286	A
11	BICF2S22935039	22999604	A	0.5	0.5	G
11	BICF2P868123	23004789	0	0	0	G
11	BICF2S23059899	23020722	A	0.5	0.5	G
11	BICF2P767539	23053879	G	0.5	0.5	A
11	BICF2P776057	23070251	0	0	0	A
11	TIGRP2P148266_rs9195575	23117401	A	0.5	0.5	C
11	BICF2P722746	23138532	A	0	0	G
11	BICF2P513165	23149614	0	0	0	G
11	BICF2P591209	23167247	G	0.5	0.5	A
11	BICF2P147167	23193051	A	0.5	0.5	G
11	BICF2P362081	23198720	G	0	0	A
11	BICF2S22960816	23215357	G	0.5	0.5	A
11	BICF2P128937	23234061	G	0.5	0.5	A
11	BICF2G630296198	23259837	0	0	0	C
11	TIGRP2P148296_rs8746180	23272064	A	0	0	G
11	TIGRP2P148308_rs9222896	23285367	G	0.5	0.5	A
11	TIGRP2P148309_rs8723273	23287673	A	0.5	0.5	G
11	BICF2P697774	23300144	G	0	0	A
11	BICF2P245757	23333404	A	0	0	T
11	BICF2P294204	23342212	G	0	0	A
11	BICF2P635200	23354347	G	1	1	A
11	BICF2P405530	23361620	G	1	1	A
11	BICF2P1256504	23373578	G	1	1	A
11	BICF2P231200	23392842	0	0	0	C
11	TIGRP2P148343_rs8874372	23411913	G	1	1	A
11	BICF2P679809	23414531	A	1	1	G
11	BICF2P186232	23441780	0	0	0	A
11	BICF2P1246544	23451746	0	0	0	G
11	TIGRP2P148357_rs8941434	23480401	G	1	1	A
11	BICF2P936273	23484330	G	1	1	A
11	BICF2P808834	23501911	A	1	1	C
11	BICF2P1018187	23510424	A	1	1	G
11	BICF2G630296200	23524137	0	0	0	A
11	BICF2P1095814	23539463	G	1	1	A
11	BICF2P1095813	23540643	G	0.5	0.5	A
11	BICF2P914727	23551115	A	1	1	C
11	BICF2P1143791	23556438	A	0	0	G
11	BICF2P495885	23574951	A	0	0	G
11	BICF2P504097	23577921	C	0.5	0.5	G
11	BICF2S23140790	23584745	C	0.5	0.5	A

11	TIGRP2P148394_rs8946632	23590605	0	0	0	G
11	BICF2S23232745	23620291	G	0.5	0.8571	A
11	TIGRP2P148398_rs9234794	23623075	0	0	0	A
11	TIGRP2P148404_rs9019982	23665422	C	0	0.3571	A
11	BICF2P716331	23670422	A	0	0.3333	C
11	TIGRP2P148409_rs9112137	23679953	G	0	0	A
11	BICF2P545015	23701171	G	0.5	0.4286	A
11	BICF2P545013	23702328	A	0	0.2857	C
11	BICF2P851474	23723216	A	0.5	0.4286	G
11	BICF2P31451	23726980	A	0	0	G
11	BICF2S23545592	23740673	A	0.5	0.4167	C
11	BICF2S2419777	23754712	C	0	0	G
11	TIGRP2P148433_rs9223548	23768094	0	0	0	G
11	TIGRP2P148435_rs8575135	23777146	A	0.5	0.5	C
11	TIGRP2P148436_rs9107108	23784437	G	0.5	0.5	A
11	BICF2P456741	23811264	G	0	0.3571	A
11	BICF2S23051925	23823002	G	0	0	A
11	TIGRP2P148451_rs9213240	23829466	A	0.5	0.4286	G
11	BICF2S23249318	23859108	G	0	0.3571	A
11	BICF2P132548	23872841	A	0	0.3571	C
11	BICF2P609850	23874973	G	0	0	A
11	BICF2P903006	23892861	C	0	0.3571	A
11	TIGRP2P148464_rs8802465	23917490	G	0	0.3571	A
11	BICF2P69513	23920402	A	0	0	G
11	BICF2P1226088	23971071	A	0	0	G
11	BICF2P850476	23978180	A	0	0	G
11	BICF2P393436	23998672	G	0.5	0.5	A
11	TIGRP2P148485_rs8825379	24010686	A	0.5	0.5	G
11	BICF2S23045162	24027591	G	0.5	0.5	A
11	BICF2P846551	24075603	G	0	0.6667	A
11	BICF2S22941556	24081567	G	0	0.6429	A
11	BICF2P596320	24090787	G	0.5	0.4286	A
11	BICF2P1395717	24105860	A	0.5	0.4286	G
11	BICF2P97854	24118410	0	0	0	C
11	BICF2P134729	24129832	0	0	0	G
11	BICF2P1064149	24158720	A	0	0	C
11	BICF2P245558	24173685	G	0	0	A
11	BICF2G630296230	24184830	A	0	0	G
11	BICF2G630296247	24199975	A	0	0	G
11	BICF2G630296257	24218547	C	0	0	A
11	BICF2G630296267	24238732	G	0	0	A
11	BICF2G630296274	24248584	A	0	0	G
11	BICF2G630296285	24276028	A	0	0	G
11	BICF2G630296286	24282836	G	0	0	A
11	BICF2S23052880	24325837	G	0	0	C
11	BICF2S23052495	24339470	A	0	0.6429	G
11	BICF2P728492	24355939	G	1	1	A

11	BICF2G630296326	24357586	A	0	0	G
11	BICF2G630296336	24378992	C	0	0	A
11	BICF2G630296343	24380673	A	0	0	G
11	BICF2G630296369	24396615	G	0	0	A
11	BICF2S23310692	24403630	0	0	0	G
11	BICF2G630296430	24434245	G	0.5	0.8571	A
11	BICF2S2334228	24457819	G	0	0	A
11	BICF2P351697	24470396	A	0	0	G
11	BICF2G630296486	24470485	C	0	0	G
11	BICF2G630296520	24484419	G	0	0	A
11	BICF2G630296551	24498328	G	0	0	A
11	BICF2P1183915	24517274	A	0	0.2857	G
11	BICF2S23644722	24519194	0	0	0	G
11	BICF2P614788	24548562	A	0	0.2857	G
11	BICF2G630296566	24558851	A	0	0	G
11	BICF2P636063	24566381	0	0	0	G
11	BICF2G630296568	24576819	0	0	0	G
11	BICF2P1122151	24597873	A	0.5	0.7857	G
11	BICF2G630296576	24607183	0	0	0	G
11	BICF2G630296581	24611572	C	0	0	A
11	TIGRP2P148511_rs8680143	24626538	0	0	0	G
11	BICF2G630296622	24634760	A	1	0.3571	G
11	BICF2G630296638	24652556	C	0	0	G
11	BICF2G630296639	24657822	G	0.5	0.1429	A
11	BICF2G630296647	24669458	G	0.5	0.1429	A
11	BICF2G630296669	24686919	G	0	0	A
11	BICF2P1445042	24693691	C	0.5	0.8571	A
11	BICF2S23023183	24694613	A	0.5	0.8571	G
11	BICF2S23759717	24703980	A	0	0	G
11	BICF2P1382218	24719635	G	0	0.6429	A
11	BICF2G630296690	24732205	G	0	0.6667	A
11	BICF2G630296698	24734012	A	0	0.6429	G
11	BICF2G630296731	24743173	A	0.5	0.8571	G
11	BICF2G630296745	24751209	A	0.5	0.1429	G
11	BICF2G630296750	24770055	G	0.5	0.8571	A
11	BICF2G630296752	24780654	G	0	0	A
11	BICF2P479608	24788637	G	0.5	0.8571	A
11	BICF2G630296768	24813153	A	0	0	C
11	BICF2S22923056	24820481	C	1	0.3571	A
11	BICF2P1304051	24841623	A	0.5	0.8571	G
11	BICF2P370338	24861858	A	0	0	G
11	BICF2G630296772	24891056	0	0	0	G
11	BICF2G630296774	24905573	0	0	0	T
11	BICF2P1264007	24912956	0	0	0	T
11	BICF2G630296778	24928929	A	0	0	T
11	BICF2G630296790	24933939	A	0.5	0.4286	G
11	BICF2P662094	24943622	A	0.5	0.5	G

11	BICF2G630296793	24961266	A	0	0.25	C
11	BICF2G630296804	24970373	0	0	0	A
11	BICF2G630296809	24979068	0	0	0	G
11	BICF2P496829	24991882	A	0	0.2857	G
11	BICF2G630296829	24997766	C	0	0	A
11	BICF2G630296845	25001074	G	0	0	A
11	BICF2G630296869	25018436	C	0.5	0.7857	A
11	BICF2G630296877	25030529	A	0	0	T
11	BICF2G630296908	25046640	A	0	0	G
11	BICF2P725682	25048477	A	0	0	G
11	BICF2G630296920	25059021	A	0	0	G
11	BICF2G630296925	25064966	A	0	0	G
11	BICF2G630296933	25078190	C	0	0	A
11	BICF2G630296936	25081603	A	0	0	G
11	BICF2G630296947	25086727	G	0	0	A
11	BICF2G630296958	25097275	A	0	0	G
11	BICF2G630296975	25105818	G	1	1	A
11	BICF2G630296982	25121723	A	1	1	G
11	BICF2G630296983	25125112	A	1	1	G
11	BICF2G630297001	25131381	G	0	0	A
11	BICF2G630297015	25140485	C	1	1	A
11	BICF2G630297036	25153145	C	1	1	A
11	BICF2P37395	25185645	A	0	0	G
11	BICF2P127301	25206568	G	1	0.7143	A
11	BICF2G630297086	25209601	A	0	0.2857	G
11	BICF2G630297106	25220179	A	1	1	G
11	BICF2G630297123	25240522	T	0	0	A
11	BICF2P1339679	25244580	G	0	0	A
11	BICF2G630297126	25257315	G	0.5	0.7857	A
11	BICF2G630297140	25266339	G	0.5	0.7857	A
11	BICF2P1197232	25267896	G	0	0.6667	A
11	BICF2G630297175	25299438	C	0	0.2857	A
11	BICF2G630297179	25300528	A	0	0	G
11	BICF2P1291816	25315340	G	0	0.2857	A
11	BICF2S23414290	25327483	0	0	0	A
11	BICF2G630297216	25379633	G	0	0	A
11	BICF2G630297221	25386335	C	0.5	0.5	A
11	BICF2P895958	25392093	G	0	0.2857	A
11	BICF2G630297229	25405929	C	0	0	G
11	BICF2G630297240	25418914	G	0	0	A
11	BICF2G630297245	25426877	A	0	0.2857	G
11	BICF2G630297264	25463498	A	0.5	0.5	G
11	BICF2G630297267	25468062	A	0	0.2857	G
11	BICF2G630297270	25475413	A	0.5	0.5	G
11	BICF2G630297272	25486064	G	0.5	0.5	C
11	BICF2G630297299	25518918	A	0	0.2857	G
11	BICF2G630297306	25531123	A	0	0	G

11	BICF2G630297314	25550667	C	0	0	A
11	BICF2G630297316	25558273	G	0	0	A
11	BICF2S23716451	25599398	A	1	0.7143	C
11	BICF2G630297325	25615313	G	1	0.3571	C
11	BICF2G630297338	25632611	G	0	0.6429	A
11	BICF2G630297344	25642624	A	0	0	G
11	BICF2G630297350	25645608	G	0	0.6429	A
11	BICF2P1032572	25672268	A	0	0	G
11	BICF2S23610823	25690067	A	1	0.3571	G
11	BICF2G630297366	25702434	A	0	0	G
11	BICF2G630297368	25706808	G	1	0.3571	A
11	BICF2S23329243	25727796	A	1	0.7143	G
11	BICF2S23353200	25744875	A	1	0.7143	G
11	BICF2G630297399	25778877	A	0	0	G
11	BICF2G630297411	25793959	A	0	0	C
11	BICF2P101144	25804529	0	0	0	A
11	BICF2G630297428	25810179	A	0	0	G
11	BICF2G630297447	25819421	A	0	0	G
11	BICF2G630297466	25828804	T	0	0	A
11	BICF2G630297492	25852185	G	0	0	A
11	BICF2P875880	25865078	A	0	0	G
11	BICF2G630297504	25879693	G	0	0	A
11	BICF2G630297520	25899899	A	0	0	G
11	BICF2G630297541	25926297	A	1	0.7143	G
11	BICF2S23521165	25941449	G	0	0.3571	A
11	BICF2G630297543	25966667	G	0.5	0.5	A
11	BICF2S2375173	25972581	A	0	0.3571	G
11	BICF2P1226927	25982652	0	0	0	G
11	BICF2P65307	26013333	0	0	0	G
11	BICF2S2414118	26037471	0	0	0	A
11	TIGRP2P148546_rs8528869	26060182	0	0	0	A
11	BICF2P876961	26079913	0	0	0	C
11	BICF2P1148389	26084731	A	0.5	0.1429	G
11	BICF2G630297548	26098724	0	0	0	A
11	BICF2G630297554	26115342	A	0	0	C
11	BICF2S23258056	26120879	A	0	0	G
11	BICF2G630297560	26145931	A	0.5	0.7857	C
11	BICF2G630297573	26158152	G	0	0.3571	C
11	BICF2G630297580	26165198	G	1	0.7143	A
11	BICF2G630297591	26180398	G	0.5	0.5	A
11	BICF2P1377315	26193267	A	0	0.2857	G
11	BICF2G630297603	26197240	G	0	0.6429	A
11	BICF2S23417045	26211330	C	0	0.2857	A
11	BICF2G630297618	26229451	A	0	0.6429	G
11	BICF2G630297621	26242406	A	0.5	0.5714	G
11	BICF2G630297624	26244338	G	0.5	0.5714	A
11	BICF2P1321302	26266110	G	0	0	A

11	BICF2S23136522	26298311	A	0	0.6429	G
11	BICF2S22940429	26301963	G	0.5	0.1429	A
11	BICF2P181297	26316215	0	0	0	G
11	BICF2S23312875	26332457	A	0.5	0.8571	G
11	BICF2S23226633	26352105	G	0.5	0.1429	A
11	BICF2G630297665	26378815	G	0	0.2	A
11	BICF2G630297667	26387396	A	0	0.2857	C
11	BICF2G630297673	26393873	G	0	0.2857	A
11	BICF2G630297680	26406412	A	0	0	G
11	BICF2G630297694	26423705	A	0	0.2	G
11	BICF2G630297714	26439355	A	0	0.2857	G
11	BICF2G630297723	26457516	G	0	0.2857	A
11	BICF2G630297725	26498904	A	0	0	G
11	BICF2P1444826	26522158	A	0	0	G
11	BICF2G630297726	26553130	C	0	0	G
11	BICF2G630297750	26587399	A	0	0.3571	G
11	BICF2S23621496	26590814	0	0	0	A
11	BICF2P207137	26601801	0	0	0	C
11	BICF2P1310551	26660532	0	0	0	A
11	BICF2S23218774	26712280	G	0.5	0.7857	A
11	BICF2S23012779	26720864	A	0.5	0.5	G
11	BICF2P1167163	26727300	G	0.5	0.1429	A
11	BICF2S23259395	26740651	C	0.5	0.1429	G
11	BICF2P1410148	26741343	G	0.5	0.1429	A
11	BICF2P1115719	26750935	G	0	0.3571	A
11	BICF2P1168708	26776005	A	0.5	0.1429	G
11	BICF2P607826	26784373	G	0.5	0.1429	A
11	BICF2P933659	26799040	A	0	0.3571	G
11	BICF2S2297605	26809269	A	0	0	G
11	BICF2P75449	26818111	A	0	0.3571	G
11	BICF2P994476	26835076	0	0	0	G
11	BICF2S23231526	26870141	A	0	0.7	G
11	BICF2P958706	26881208	C	0	0	A
11	BICF2P1267606	26896436	G	0	0	A
11	BICF2P1267604	26898595	G	0	0	C
11	BICF2P1308802	26918900	A	0	0.6429	C
11	BICF2S23040875	26949427	A	1	0.6429	G
11	BICF2P844136	26963026	A	0	0.6429	G
11	BICF2P950903	26977423	A	1	0.6429	G
11	BICF2S22940836	26983919	G	0	0.3571	A
11	BICF2P201440	26992470	G	0	0.3571	A
11	BICF2S2309633	27003230	T	0	0.3571	A
11	BICF2S23038563	27014247	A	0	0.2857	C
11	BICF2P174860	27050246	0	0	0	C
11	BICF2P95128	27060092	0	0	0	A
11	BICF2G630297756	27078999	A	0	0.3571	G
11	BICF2S23050947	27098600	G	1	0.7143	A

11	BICF2G630297757	27109037	G	0	0.6429	A
11	BICF2G630297770	27118428	A	0	0.6429	G
11	BICF2G630297805	27132847	A	1	0.3571	G
11	BICF2G630297820	27140083	G	0	0.3571	A
11	BICF2G630297832	27156667	C	0	0.6429	G
11	BICF2G630297841	27172565	A	0	0.3571	G
11	BICF2P825425	27178549	C	0	0.3571	A
11	BICF2P569995	27197653	0	0	0	A
11	BICF2G630297868	27214445	G	0	0.3571	A
11	BICF2S23420306	27238471	C	0	0	A
11	BICF2G630297870	27245906	A	1	0.3571	G
11	BICF2G630297875	27263499	G	0	0.6429	A
11	BICF2G630297880	27277762	C	1	0.3571	A
11	BICF2G630297881	27293720	G	0	0.6429	A
11	BICF2G630297906	27306996	G	0	0.6429	A
11	BICF2G630297911	27315445	C	0	0.6429	A
11	BICF2G630297933	27356229	A	1	0.6429	G
11	BICF2G630297939	27361028	A	1	0.3571	G
11	BICF2G630297965	27378150	A	0	0.3571	G
11	BICF2G630297979	27384781	G	1	0.3571	A
11	BICF2G630297998	27397738	A	0	0.3571	C
11	BICF2G630298000	27407655	A	0	0.6429	G
11	BICF2G630298014	27416124	C	0	0.5	A
11	BICF2G630298086	27472640	A	0	0.6429	T
11	BICF2G630298088	27476204	C	0	0.3571	G
11	BICF2P475432	27482607	A	0	0.2857	G
11	BICF2G630298105	27484355	G	0	0.2857	A
11	BICF2S2353548	27486294	A	0	0	C
11	BICF2P640804	27505796	0	0	0	A
11	BICF2P954878	27513097	0	0	0	G
11	BICF2G630298108	27522872	A	0	0	G
11	BICF2S2456129	27547013	G	1	0.3571	C
11	G662f34S142	27554348	C	1	0.3333	A
11	BICF2P1473023	27579198	A	1	0.6429	C
11	BICF2G630298113	27591579	A	0	0.6429	G
11	BICF2P983334	27601172	C	0	0.3571	A
11	BICF2G630298133	27618732	A	0	0.6429	C
11	BICF2G630298155	27625629	A	0	0	G
11	BICF2G630298201	27658868	G	0	0	A
11	BICF2G630298210	27673411	G	1	0.6429	A
11	BICF2G630298249	27691581	A	0	0	G
11	BICF2G630298252	27692549	G	0	0	A
11	BICF2G630298288	27706944	G	0	0.125	A
11	BICF2G630298294	27731303	A	0	0	C
11	BICF2S22946260	27741478	A	0	0	G
11	BICF2G630298303	27757207	A	0	0	G
11	BICF2G630298305	27771034	G	0	0	A

11	BICF2S2354103	27780358	G	1	1	A
11	BICF2G630298308	27803452	A	0.5	0.1429	G
11	BICF2G630298330	27839302	A	0	0	G
11	BICF2P1216930	27849026	A	0.5	0.1429	G
11	BICF2G630298346	27859064	G	0.5	0.1429	A
11	BICF2G630298351	27861509	G	NA	0	A
11	BICF2G630298354	27866895	A	0	0	G
11	BICF2P167768	27899467	0	0	0	C
11	BICF2G630298355	27914311	A	0.5	0.5833	C
11	BICF2P250950	27922188	0	0	0	C
11	BICF2G630298356	27939754	A	0	0	G
11	BICF2G630298358	27950424	A	1	1	G
11	BICF2P281218	27952276	G	1	1	A
11	BICF2G630298374	27959165	G	0	0	A
11	BICF2P1090053	27971570	A	1	0.7143	G
11	BICF2P1404198	27979701	A	0	0	T
11	BICF2P171927	28009650	G	0	0.2857	A
11	BICF2S23033910	28018338	A	0.5	0.1429	G
11	BICF2P258967	28029058	A	0.5	0.4286	G
11	BICF2P898775	28038434	G	0	0	A
11	BICF2P478326	28056385	G	0	0	A
11	G663f37S177	28076157	G	0	0	A
11	BICF2S23751313	28096820	G	0	0	A
11	BICF2G630298391	28111135	C	0	0	A
11	BICF2G630298411	28119964	G	0	0	A
11	BICF2G630298425	28132595	A	0.5	0.4286	G
11	BICF2G630298432	28140922	A	0	0.2857	G
11	BICF2G630298466	28166384	G	0	0.2857	A
11	BICF2P423109	28185376	A	0.5	0.1429	G
11	BICF2S23641753	28188784	A	0.5	0.1429	G
11	BICF2G630298473	28193452	A	0	0	G
11	BICF2G630298479	28200871	G	0.5	0.8571	A
11	BICF2G630298492	28212318	G	0.5	0.8571	A
11	BICF2G630298499	28216603	G	0	0	A
11	BICF2G630298517	28226462	G	0	0	C
11	BICF2P746164	28240808	G	0	0	A
11	BICF2P1224532	28248203	A	0	0	G
11	BICF2G630298548	28261043	G	0	0	A
11	BICF2G630298570	28276024	G	0	0	A
11	BICF2G630298573	28282214	A	0	0	G
11	BICF2P929252	28307924	0	0	0	G
11	BICF2G630298576	28335184	0	0	0	A
11	BICF2S23233885	28342874	C	0.5	0.5714	G
11	BICF2G630298577	28356276	A	0	0	C
11	BICF2G630298578	28361012	C	0.5	0.1429	A
11	BICF2G630298579	28388366	A	0.5	0.1429	G
11	BICF2P1124809	28399353	0	0	0	C

11	BICF2G630298580	28410096	A	0.5	0.1429	G
11	BICF2G630298581	28430501	A	0	0	G
11	BICF2G630298583	28455287	A	0	0	G
11	BICF2G630298597	28496564	G	1	1	A
11	BICF2G630298666	28517301	T	0.5	0.4286	A
11	BICF2G630298685	28539761	G	0	0	A
11	BICF2G630298745	28559300	A	NA	0	C
11	BICF2G630298762	28566339	G	0.5	0.5714	A
11	BICF2G630298789	28577556	A	0	0	G
11	TIGRP2P148661_rs9006547	28599121	0	0	0	C
11	BICF2G630298793	28607134	G	0.5	0.5714	A
11	BICF2P393214	28615434	0	0	0	A
11	BICF2G630298794	28651487	A	0.5	0.1429	T
11	BICF2G630298799	28716419	G	0.5	0.1429	A
11	BICF2P345763	28728371	0	0	0	A
11	BICF2G630298800	28760284	A	0	0	G
11	BICF2P849339	28763004	0	0	0	G
11	BICF2G630298803	28787491	T	0	0	A
11	BICF2P171735	28817771	0	0	0	A
11	BICF2P1030319	28833129	0	0	0	A
11	BICF2G630298808	28844990	G	0	0	A
11	BICF2P556718	28861743	0	0	0	G
11	BICF2G630298816	28873806	C	0	0	A
11	BICF2G630298827	28879387	A	0	0.3571	G
11	BICF2S23319762	28893102	A	0.5	0.5	G
11	BICF2G630298853	28901872	A	0.5	0.5	G
11	BICF2G630298866	28915373	G	0.5	0.5	A
11	BICF2G630298870	28927434	A	0.5	0.5	G
11	BICF2S2332622	28945193	A	0	0	G
11	BICF2G630298878	28961279	G	0	0.2857	A
11	BICF2S2443329	28983662	A	0.5	0.4286	C
11	BICF2G630298885	28992936	A	0.5	0.2143	C
11	BICF2P428253	29025093	0	0	0	A
11	BICF2G630298888	29032242	A	0	0.2857	G
11	BICF2G630298893	29052790	A	0	0	G
11	BICF2G630298905	29067632	C	1	0.3571	A
11	BICF2G630298908	29078383	A	1	0.3571	G
11	BICF2G630298911	29084163	G	1	1	A
11	BICF2G630298921	29095243	A	0	0	G
11	BICF2G630298932	29112972	A	0.5	0.2143	G
11	BICF2G630298939	29126554	G	0.5	0.2143	A
11	BICF2P149301	29138165	G	0.5	0.5	A
11	BICF2G630298944	29141967	A	0	0.2857	G
11	BICF2G630298954	29157734	A	0	0.2857	G
11	BICF2G630298998	29170379	G	0	0.2857	A
11	BICF2G630299009	29184586	C	0.5	0.2143	A
11	BICF2G630299021	29196397	A	0	0	G

11	BICF2G630299027	29214251	A	0	0.3571	G
11	BICF2G630299037	29228815	G	0	0	A
11	BICF2G630299043	29234329	A	0	0	G
11	BICF2G630299060	29251608	G	0	0.3571	A
11	BICF2G630299067	29256521	A	0	0.3571	G
11	BICF2G630299090	29288989	A	0	0.3571	G
11	BICF2P228229	29315825	0	0	0	A
11	BICF2P491485	29338197	0	0	0	C
11	TIGRP2P148678_rs9184266	29360374	0	0	0	A
11	BICF2G630299096	29388941	G	0	0	A
11	BICF2P1184335	29408620	0	0	0	A
11	BICF2S23115602	29420578	A	0	0.2857	G
11	BICF2P761182	29432661	0	0	0	A
11	BICF2P319178	29444514	0	0	0	A
11	BICF2P1365549	29456749	0	0	0	A
11	BICF2G630299099	29470043	A	0.5	0.2143	G
11	BICF2G630299100	29492186	C	0	0	A
11	BICF2P824932	29504143	0	0	0	A
11	BICF2P766591	29512692	0	0	0	G
11	BICF2P620360	29523647	G	0.5	0.2143	A
11	G664f19S194	29548643	0	0	0	C
11	BICF2P689261	29565114	A	0.5	0.4286	G
11	BICF2G630299103	29570581	A	0	0	G
11	BICF2P701388	29579089	0	0	0	C
11	BICF2P663715	29633998	G	0.5	0.4286	A
11	BICF2S23434630	29654371	G	0.5	0.5714	A
11	BICF2P520021	29676429	A	0.5	0.5714	G
11	BICF2P714250	29683230	C	0.5	0.5714	G
11	BICF2G630299110	29694574	A	0.5	0.2143	G
11	BICF2G630299111	29705315	A	0	0	G
11	BICF2S23160325	29734366	G	0.5	0.2143	A
11	BICF2S23150627	29747289	A	0.5	0.2143	G
11	BICF2P1142593	29755085	G	0.5	0.4286	C
11	BICF2G630299125	29766811	A	0	0	G
11	BICF2G630299132	29775704	G	0	0.2857	A
11	BICF2G630299139	29813989	A	0	0	G
11	BICF2G630299145	29828468	A	0	0	G
11	BICF2G630299160	29837082	G	NA	0	A
11	BICF2P578001	29837633	A	0	0.2857	G
11	BICF2G630299180	29858752	G	0	0	A
11	BICF2S2308967	29874625	G	0	0.3571	A
11	BICF2P1047482	29877367	A	0	0	C
11	BICF2G630299241	29900158	G	1	0.7143	A
11	BICF2G630299277	29919965	A	0.5	0.1429	G
11	BICF2G630299281	29923010	A	0.5	0.1429	G
11	BICF2G630299297	29941783	G	0.5	0.1429	A
11	BICF2G630299301	29948566	G	0	0	A

11	BICF2G630299309	29970738	G	0	0.3571	A
11	BICF2P205773	29988601	A	0.5	0.2143	G
11	BICF2P453030	30002381	A	0.5	0.5	C
11	BICF2P386951	30034722	A	0.5	0.1429	T
11	BICF2P413264	30042053	A	0.5	0.2143	T
11	BICF2G630299334	30049507	A	0.5	0.1429	G
11	BICF2S23248430	30064091	C	0.5	0.2143	A
11	BICF2G630299341	30073823	A	0	0.3571	T
11	BICF2G630299358	30090955	A	0	0.2857	G
11	BICF2G630299365	30097935	G	0	0	A
11	BICF2G630299385	30114710	A	0	0.3571	C
11	BICF2G630299391	30124945	C	NA	0.5	A
11	BICF2G630299434	30158383	A	0.5	0.5	C
11	BICF2G630299444	30166071	A	0	0.3571	C
11	BICF2P118018	30199184	A	1	0.7143	G
11	BICF2P27204	30221624	A	0	0	G
11	BICF2P694211	30228435	G	0	0	A
11	BICF2G630299584	30241448	G	0	0	A
11	BICF2G630299587	30245842	0	0	0	G
11	BICF2G630299599	30252648	C	0	0	A
11	BICF2P197587	30271820	A	0.5	0.2143	G
11	BICF2G630299629	30280602	G	0.5	0.2143	A
11	BICF2G630299644	30292837	G	0.5	0.2143	A
11	BICF2G630299668	30323727	A	0.5	0.5	C
11	BICF2G630299669	30323849	G	0.5	0.5	A
11	BICF2G630299705	30351070	A	0.5	0.5	G
11	BICF2G630299746	30366466	A	0	0	G
11	BICF2G630299754	30372942	A	0	0	G
11	BICF2G630299795	30392231	G	0	0	A
11	BICF2G630299803	30398092	G	0.5	0.5	A
11	BICF2P1291045	30411265	A	0.5	0.5	G
11	BICF2G630299830	30420453	A	1	0.6429	G
11	BICF2P1116154	30440286	0	0	0	A
11	TIGRP2P148773_rs8675875	30454705	0	0	0	G
11	BICF2S23749357	30466990	0	0	0	G
11	TIGRP2P148775_rs8835522	30491774	0	0	0	A
11	G665f49S129	30503115	A	0	0	C
11	BICF2S23649890	30534383	A	1	1	G
11	BICF2S23410619	30559902	A	1	1	G
11	BICF2S22941236	30575254	C	1	1	A
11	BICF2P1159169	30604687	A	0	0.3571	C
11	BICF2P1075328	30616829	0	0	0	A
11	BICF2S23719313	30634806	A	0	0	G
11	BICF2S23722377	30638025	G	0.5	0.2143	A
11	BICF2G630299842	30653884	A	0	0.6429	T
11	BICF2G630299855	30663037	A	0.5	0.8571	G
11	BICF2G630299878	30679046	G	0	0.6429	A

11	BICF2P438060	30682186	C	0	0.2857	A
11	BICF2S2301099	30700717	0	0	0	G
11	BICF2P1103444	30709456	A	0	0	G
11	BICF2G630299892	30725940	A	0.5	0.5714	G
11	BICF2G630299893	30730092	C	0.5	0.5833	A
11	BICF2G630299896	30743666	A	0.5	0.5714	G
11	BICF2S22911094	30771655	A	0.5	0.4286	G
11	BICF2P344959	30778083	A	0.5	0.4286	G
11	BICF2G630299908	30785969	A	0	0	T
11	BICF2G630299915	30798206	G	0	0	A
11	BICF2P110183	30817164	A	0.5	0.4286	G
11	BICF2P826162	30821035	G	0.5	0.4286	A
11	BICF2G630299923	30831530	G	0.5	0.5714	A
11	TIGRP2P148781_rs8825996	30872930	0	0	0	C
11	BICF2P506092	30882200	0	0	0	T
11	BICF2G630299964	30890968	G	0.5	0.5714	A
11	BICF2G630299989	30938113	G	0.5	0.5714	A
11	BICF2G630300004	30952230	G	0.5	0.5714	A
11	BICF2G630300011	30967754	A	0.5	0.5714	G
11	BICF2G630300017	30981982	A	0.5	0.5714	G
11	BICF2P1280996	31000020	C	0.5	0.5833	A
11	BICF2G630300026	31006780	A	0	0	G
11	BICF2P1083256	31019918	G	0.5	0.4286	A
11	BICF2G630300032	31036888	A	0	0	G
11	BICF2G630300034	31046373	C	0	0	A
11	BICF2G630300035	31055308	A	0	0	G
11	BICF2P117069	31072309	T	0.5	0.4286	A
11	BICF2G630300036	31075391	A	0.5	0.5714	G
11	BICF2S23429844	31095097	A	0.5	0.5	G
11	BICF2G630300044	31096841	G	0.5	0.5	A
11	BICF2G630300049	31114436	G	0	0	A
11	BICF2G630300053	31122308	A	0	0	G
11	BICF2G630300058	31134293	A	0	0	G
11	BICF2G630300073	31146053	G	0.5	0.2143	A
11	BICF2P1421775	31160215	C	0.5	0.7857	A
11	BICF2G630300089	31173993	A	0	0	G
11	BICF2P1077885	31193949	0	0	0	G
11	BICF2G630300092	31211122	G	0	0	A
11	BICF2G630300123	31227157	A	0	0.2857	G
11	TIGRP2P148797_rs8659542	31236607	0	0	0	G
11	BICF2G630300145	31265353	A	0	0.2857	G
11	BICF2G630300159	31281993	C	0	0	G
11	BICF2G630300171	31298644	A	0	0	G
11	BICF2S22943906	31325382	G	0	0	A
11	BICF2G630300189	31336671	G	0	0	A
11	BICF2G630300194	31340973	A	0	0	G
11	BICF2G630300209	31350118	A	0	0	C

11	BICF2G630300219	31361591	A	0	0	G
11	BICF2G630300229	31378579	G	0	0	A
11	BICF2G630300252	31392418	G	0	0	A
11	BICF2G630300261	31395067	G	0	0	A
11	BICF2G630300277	31412024	A	0	0	G
11	BICF2G630300288	31418816	G	0	0	A
11	BICF2G630300329	31440525	A	0	0	G
11	BICF2G630300331	31442913	G	0.5	0.2143	A
11	BICF2G630300353	31457345	G	0	0	A
11	BICF2G630300400	31468002	C	0	0	A
11	BICF2G630300427	31476643	G	0	0	A
11	BICF2P850139	31493818	G	0	0	A
11	BICF2G630300464	31504657	G	0.5	0.2143	A
11	BICF2P369907	31510215	A	0	0.2857	G
11	BICF2G630300493	31543485	G	0.5	0.5	A
11	BICF2G630300525	31552521	A	0	0	G
11	BICF2G630300537	31569508	A	0	0	G
11	BICF2G630300545	31578686	T	1	0.7143	A
11	BICF2G630300547	31580822	G	0.5	0.5	A
11	BICF2P988995	31590905	G	0.5	0.7857	A
11	BICF2P632834	31604573	A	0.5	0.7857	G
11	BICF2S23029239	31614729	A	0	0	G
11	TIGRP2P148799_rs9207592	31639262	0	0	0	A
11	BICF2G630300573	31653181	A	0.5	0.5	G
11	BICF2P929963	31670818	G	0	0.2857	A
11	BICF2P836237	31679018	G	0	0.2857	A
11	BICF2P1317494	31688749	A	0.5	0.5	T
11	BICF2G630300588	31694583	A	0.5	0.5	C
11	BICF2G630300623	31709653	G	0.5	0.2143	A
11	BICF2G630300626	31735040	G	0	0.25	A
11	BICF2G630300632	31747010	A	0	0	G
11	BICF2P1426766	31757647	0	0	0	A
11	BICF2G630300633	31769800	A	0	0.2857	G
11	BICF2S23231865	31779374	A	0	0.25	G
11	BICF2G630300646	31791235	C	0.5	0.5	A
11	BICF2G630300662	31797835	G	0.5	0.5	A
11	BICF2G630300683	31819146	A	0.5	0.5	G
11	BICF2G630300689	31831896	G	0	0	A
11	BICF2G630300692	31841509	G	0	0.2857	A
11	BICF2G630300697	31843624	G	0	0.2857	A
11	BICF2G630300746	31856494	C	0	0	A
11	BICF2G630300749	31869786	G	0	0.2857	A
11	BICF2P775801	31891444	A	0	0	G
11	BICF2S23057473	31903227	C	0	0	A
11	BICF2P281857	31932220	A	0	0	G
11	BICF2S2345606	31939564	G	0	0	A
11	BICF2P216288	31947169	A	0	0	G

11	BICF2P239310	31959950	A	0	0	C
11	BICF2P254861	31975811	G	0	0	A
11	BICF2S231270	31988752	A	0.5	0.2143	G
11	BICF2S2359799	32003801	0	0	0	G
11	BICF2S23517990	32007662	0	0	0	G
11	BICF2P457249	32033525	C	0	0.125	A
11	BICF2P967438	32043400	A	0.5	0.2143	T
11	BICF2P1361908	32058462	A	0.5	0.7857	G
11	BICF2P379994	32069518	A	0.5	0.2143	G
11	BICF2P1266709	32077561	C	0.5	0.2143	A
11	BICF2P222288	32093156	T	0.5	0.7857	A
11	BICF2S23517020	32099444	A	0.5	0.7857	G
11	BICF2S23047210	32115719	A	1	0.7143	G
11	BICF2P695900	32126031	A	0	0	G
11	BICF2P1033624	32142140	G	0	0.2857	C
11	BICF2P454456	32175491	A	0	0	C
11	BICF2S23434478	32205124	0	0	0	G
11	BICF2P1003706	32231884	G	0	0	A
11	BICF2P496658	32234990	C	0	0	A
11	BICF2P1217773	32257923	A	0	0	G
11	BICF2P50610	32270617	A	0	0	T
11	TIGRP2P148821_rs8589376	32283217	0	0	0	C
11	BICF2P155743	32295280	A	0.5	0.2143	G
11	BICF2P952461	32304815	0	0	0	A
11	BICF2S23426023	32321461	A	0.5	0.2143	G
11	BICF2S2346546	32329573	G	0.5	0.2143	A
11	BICF2S23517839	32339188	G	0.5	0.2143	A
11	BICF2P1365122	32363758	G	0.5	0.2143	A
11	BICF2P1365131	32366453	G	0.5	0.2143	A
11	BICF2P493958	32405620	A	0	0.2857	G
11	BICF2S22953867	32449706	A	0	0	C
11	BICF2S23727561	32455639	G	0.5	0.5	A
11	BICF2P363781	32466322	G	1	0.7143	A
11	BICF2P1468476	32483896	A	0.5	0.5	G
11	BICF2P955174	32496343	A	0.5	0.5	G
11	BICF2P1329855	32503214	G	0	0	A
11	BICF2P804663	32508839	A	0	0	G
11	BICF2P809829	32511633	A	0.5	0.7857	G
11	BICF2S23530335	32527913	C	0.5	0.5	A
11	TIGRP2P148825_rs9197535	32548998	0	0	0	A
11	BICF2S23255975	32559501	A	0.5	0.5	G
11	BICF2P867682	32569849	G	0.5	0.2143	A
11	BICF2P368366	32581541	C	0.5	0.2143	A
11	BICF2P1467388	32597763	A	0	0.2857	C
11	BICF2P1378120	32632797	G	0	0.2857	A
11	BICF2P855505	32640665	A	0.5	0.2143	G
11	BICF2P1336251	32658311	0	0	0	G

11	BICF2P378996	32669668	0	0	0	A
11	BICF2P205065	32672958	0	0	0	A
11	BICF2S2413397	32690796	C	0	0	A
11	G666f37S132	32700125	0	0	0	A
11	BICF2S23232442	32722536	0	0	0	G
11	BICF2P157878	32739496	0	0	0	A
11	BICF2P1398049	32780836	0	0	0	A
11	BICF2S2436574	32799092	0	0	0	G
11	BICF2P56279	32813505	A	0.5	0.7857	G
11	BICF2P1325239	32824323	A	0	0	T
11	BICF2P522203	32836686	T	0.5	0.7	A
11	BICF2P1429396	32853115	G	0	0	A
11	TIGRP2P148830_rs8974133	32860574	0	0	0	A
11	BICF2P217984	32871269	0	0	0	A
11	BICF2P548063	32887744	0	0	0	A
11	BICF2P1302782	32891174	C	0	0	A
11	BICF2P531097	32908558	C	0.5	0.7857	A
11	BICF2P1290820	32922914	A	0.5	0.7857	G
11	BICF2P550340	32935770	T	0.5	0.2143	A
11	BICF2S23260615	32968071	A	0.5	0.2143	G
11	BICF2S23339423	32975713	G	0	0.2857	A
11	BICF2S23341116	32995046	G	0.5	0.5	A
11	BICF2P1031491	33027731	G	0	0.2857	A
11	BICF2P1031492	33028837	G	0	0.2857	A
11	BICF2P812943	33048789	A	0.5	0.2143	G
11	BICF2P216928	33067902	A	0.5	0.2143	G
11	BICF2P524836	33104002	A	0.5	0.2143	C
11	BICF2S23654212	33138697	A	0	0	C
11	BICF2P230882	33148870	A	0	0	G
11	BICF2P608417	33159023	A	0	0	C
11	BICF2P633536	33173437	A	0.5	0.2143	G
11	BICF2P384490	33181506	G	0.5	0.7857	A
11	BICF2P392327	33196301	G	0.5	0.7857	A
11	BICF2S23550361	33212834	A	0.5	0.2143	C
11	BICF2P990247	33237785	G	0.5	0.7857	A
11	BICF2S23112023	33247837	G	0.5	0.7857	A
11	BICF2P955410	33305204	0	0	0	A
11	BICF2S22918349	33328562	A	0	0	C
11	BICF2P1426297	33371657	G	0	0	A
11	BICF2S23118050	33389565	A	0	0.2857	C
11	BICF2P605886	33412035	0	0	0	A
11	BICF2S2328043	33425537	G	0.5	0.7857	A
11	BICF2S23631053	33455834	G	0.5	0.7857	A
11	BICF2S22914838	33485591	A	1	1	G
11	BICF2S2317338	33490088	C	1	1	A
11	BICF2P143501	33503603	0	0	0	A
11	BICF2S23159652	33522200	C	1	0.7143	A

11	BICF2P886238	33528677	A	1	0.7143	G
11	BICF2P1310603	33547323	C	1	0.7143	G
11	BICF2P357830	33559159	A	1	0.7143	G
11	BICF2P707879	33574929	0	0	0	A
11	BICF2P369872	33587601	T	0	0	A
11	BICF2P1226314	33595884	A	0.5	0.7857	G
11	BICF2S23021829	33605667	G	0	0	A
11	BICF2P1396275	33619057	A	0	0	G
11	BICF2P186836	33637558	G	1	1	A
11	BICF2P964147	33651331	G	0	0.2857	A
11	BICF2P299341	33662370	A	0	0.2857	G
11	BICF2P578992	33683432	G	0.5	0.5	A
11	BICF2P859534	33695190	A	0.5	0.5	G
11	BICF2P1191626	33708767	A	0	0.2857	G
11	BICF2P1011536	33718085	G	0.5	0.5	A
11	BICF2P1054463	33737891	C	0.5	0.7857	A
11	BICF2P914752	33742887	A	0.5	0.7857	G
11	BICF2P574984	33755767	G	0.5	0.7857	A
11	BICF2P365956	33765721	0	0	0	A
11	BICF2P414306	33781985	G	1	1	A
11	BICF2S23329510	33791316	A	1	1	G
11	BICF2P1135619	33806530	A	1	1	G
11	BICF2S23751452	33811404	A	0.5	0.2143	C
11	BICF2S2362148	33828603	A	0	0	G
11	BICF2P1353520	33837143	A	0	0	G
11	BICF2P1425588	33847533	A	0	0	G
11	BICF2S23657655	33861082	G	0	0	C
11	BICF2P666228	33873353	A	0	0.2857	G
11	BICF2P853493	33884943	0	0	0	A
11	BICF2P1174922	33924830	A	0	0	T
11	BICF2S2451861	33942981	G	0	0	A
11	BICF2P1120035	33964419	0	0	0	A
11	BICF2P280125	33993467	A	1	1	G
11	BICF2P86237	33994661	A	1	1	G
11	BICF2S23044477	34022888	A	1	1	G
11	BICF2P148402	34032997	A	0	0.2857	G
11	BICF2P670707	34044525	A	1	1	G
11	BICF2S23446410	34059314	A	0	0	G
11	BICF2P610556	34069446	G	1	1	A
11	BICF2S23334009	34080733	T	0	0	A
11	BICF2P990485	34092039	G	0	0	A
11	BICF2P661307	34108550	A	0.5	0.7857	G
11	BICF2P538485	34111675	A	0	0	C
11	BICF2S23252598	34144515	A	0.5	0.2143	G
11	BICF2S2341332	34172742	G	0.5	0.7857	A
11	BICF2S23729958	34193349	C	0.5	0.2143	A
11	BICF2S2321607	34201464	G	0.5	0.2143	A

11	BICF2S23615280	34218888	0	0	0	A
11	BICF2P842917	34226972	0	0	0	G
11	BICF2S23521281	34245463	G	0	0	A
11	BICF2S23047340	34249259	C	0	0	A
11	BICF2P298512	34259099	A	0	0	G
11	BICF2P757467	34292102	A	0.5	0.7857	C
11	BICF2P1164880	34296082	A	0.5	0.7857	G
11	BICF2S23011025	34299001	C	0	0	G
11	BICF2P196716	34302752	A	0.5	0.8333	C
11	TIGRP2P148897_rs8999430	34316715	0	0	0	A
11	BICF2P435382	34338074	G	0	0	A
11	BICF2P1279642	34341408	G	1	1	A
11	BICF2P1213998	34364632	A	0	0	G
11	BICF2P212917	34376386	G	0	0	A
11	BICF2P960433	34390554	A	1	1	G
11	BICF2P1221043	34397373	0	0	0	A
11	BICF2P553157	34411060	G	0	0	A
11	BICF2P534546	34432490	A	1	1	G
11	BICF2P1396247	34453534	0	0	0	G
11	BICF2P1395269	34477169	A	1	1	C
11	BICF2P1145239	34510264	G	0	0	A
11	BICF2P532323	34516636	A	0	0	G
11	BICF2P86650	34547708	G	0	0	A
11	BICF2P45825	34592984	G	0	0	A
11	BICF2P61438	34605734	G	0	0	A
11	BICF2P1252243	34644184	A	0	0	G
11	BICF2P332582	34664793	G	0	0	A
11	BICF2P1027048	34701927	T	0	0	A
11	BICF2S2319080	34723330	G	0	0	A
11	BICF2S23319292	34741189	A	0	0	G
11	TIGRP2P148902_rs8661616	34744333	0	0	0	A
11	BICF2P745082	34788633	G	0	0	A
11	BICF2P932357	34813303	G	0	0	A
11	BICF2P250902	34847251	0	0	0	A
11	BICF2P996778	34870569	0	0	0	G
11	BICF2P1041384	34883225	0	0	0	A
11	BICF2P938867	34919023	0	0	0	A
11	BICF2P597272	34931203	C	0	0	A
11	BICF2S2445284	34963653	0	0	0	G
11	BICF2S23150444	34981472	G	0	0	A
11	BICF2P374651	35022362	G	0	0	A
11	BICF2S2456012	35072830	A	0	0	G
11	BICF2S23233743	35075473	G	0	0	A
11	G667f23S183	35111671	A	0	0	G
11	BICF2S2334417	35132197	G	0	0	C
11	G668f35S129	35157341	A	0	0	C
11	BICF2P1189606	35182734	0	0	0	A

11	BICF2S23424151	35191713	G	0	0	A
11	BICF2S22916873	35207633	A	0	0	G
11	BICF2S23161151	35214889	A	0	0	C
11	BICF2S2301825	35228662	A	0	0	C
11	BICF2P179452	35237304	0	0	0	A
11	G669f13S175	35263745	A	0	0	C
11	G670f34S196	35318966	G	0	0	A
11	BICF2S23027422	35353553	G	0	0	A
11	G671f20S35	35381005	G	0	0	A
11	BICF2S23145330	35394549	A	0	0	G
11	BICF2P707727	35398583	0	0	0	A
11	BICF2S23136941	35428978	G	0	0	A
11	G672f38S107	35453715	A	0	0	G
11	BICF2S23430617	35484736	G	0	0	A
11	BICF2P200637	35495439	0	0	0	G
11	G673f47S112	35519119	G	0	0	A
11	BICF2P1226574	35546785	0	0	0	A
11	BICF2P1214963	35572339	0	0	0	G
11	BICF2S23150328	35587763	0	0	0	A
11	BICF2P736464	35595450	A	0.5	0.7857	G
11	BICF2S22919345	35606033	G	0	0	A
11	BICF2P1331669	35618541	G	0.5	0.7857	A
11	BICF2S23150034	35647715	G	0	0	A
11	BICF2P1273994	35672050	G	0.5	0.7857	A
11	BICF2S23714676	35674225	C	0.5	0.7857	G
11	BICF2P661574	35692648	A	0.5	0.8333	C
11	BICF2S2375951	35706515	A	0.5	0.2143	G
11	BICF2P267425	35708301	A	0.5	0.7857	G
11	BICF2S23429593	35727614	A	0	0	G
11	BICF2P551897	35736309	A	0.5	0.7857	G
11	BICF2P974754	35742421	G	0.5	0.7857	A
11	BICF2P751088	35761590	G	0.5	0.7857	A
11	BICF2P1423876	35765370	G	0.5	0.7857	A
11	BICF2P559015	35796050	C	0.5	0.7857	G
11	BICF2P1169284	35810870	A	0.5	0.7857	G
11	BICF2P511897	35829192	A	0.5	0.7857	G
11	BICF2S2316045	35836142	A	0.5	0.7857	G
11	BICF2P1104029	35845667	G	0.5	0.7857	A
11	BICF2S23613918	35867357	G	0	0	A
11	BICF2P674626	35875844	G	0.5	0.7857	A
11	BICF2P1067726	35890234	A	0.5	0.8333	G
11	BICF2P1067727	35890320	G	0.5	0.7857	A
11	BICF2P1067730	35891601	0	0	0	A
11	BICF2S24310803	35911874	A	0.5	0.7857	G
11	BICF2S23331281	35918500	A	0.5	0.7857	G
11	BICF2P959626	35927911	A	0.5	0.7857	C
11	BICF2P1164801	35963288	G	0.5	0.8333	A

11	BICF2P345169	35977355	A	0.5	0.2143	G
11	BICF2P1162988	35994453	G	0.5	0.7857	A
11	BICF2P700072	36006528	C	0.5	0.7857	A
11	BICF2P995290	36030887	G	0.5	0.7857	A
11	BICF2S23038947	36048630	A	0.5	0.7857	G
11	BICF2P494620	36058670	G	0.5	0.7857	A
11	BICF2P1453750	36065269	G	0.5	0.7857	A
11	BICF2S23536724	36079284	A	0.5	0.7857	G
11	BICF2P100162	36097340	G	0.5	0.7857	A
11	BICF2S23646868	36120525	T	0	0	A
11	BICF2S23355661	36134341	A	0.5	0.2143	G
11	BICF2S23021448	36146790	G	0	0	C
11	BICF2P696343	36162211	G	0.5	0.7857	A
11	BICF2P535628	36176896	A	0.5	0.7857	G
11	BICF2P138348	36180497	A	0.5	0.7857	C
11	BICF2P443517	36201798	C	0.5	0.7857	A
11	BICF2P525110	36208110	0	0	0	A
11	BICF2P990488	36221976	A	0.5	0.7857	G
11	BICF2P681606	36226618	G	0.5	0.7857	A
11	BICF2P98398	36242601	0	0	0	A
11	BICF2P1046131	36255483	T	0.5	0.2143	A
11	BICF2S23259230	36263235	G	0.5	0.2143	A
11	BICF2S22918785	36282384	C	0	0	A
11	BICF2S23159756	36283882	G	0.5	0.7857	A
11	BICF2S22963829	36305350	G	0.5	0.7857	A
11	BICF2S2379845	36321967	A	0.5	0.2143	G
11	BICF2P93273	36328834	A	0.5	0.7857	C
11	BICF2P1398773	36343543	A	0.5	0.2143	G
11	BICF2P1101477	36353483	G	0.5	0.7857	A
11	BICF2P203206	36364803	A	0.5	0.2143	G
11	BICF2P1132820	36374631	A	0.5	0.7857	C
11	BICF2S23122525	36377013	C	0	0	A
11	BICF2P1384693	36393162	G	0.5	0.2143	A
11	BICF2P1020121	36405430	G	0.5	0.7857	A
11	BICF2P610779	36409478	G	0.5	0.2143	A
11	BICF2P824858	36422457	A	0.5	0.2143	G
11	BICF2P7507	36436733	A	0.5	0.2143	G
11	BICF2P1428719	36443607	G	0.5	0.2143	A
11	BICF2P1191640	36458248	C	0.5	0.2143	G
11	BICF2P264106	36488874	A	0.5	0.2143	T
11	BICF2S23762564	36490075	A	0.5	0.2143	C
11	TIGRP2P148931_rs8906800	36506230	0	0	0	A
11	BICF2P704790	36513689	G	0.5	0.2143	A
11	BICF2P239482	36531129	C	0.5	0.2143	A
11	BICF2P547497	36552467	G	0	0	A
11	BICF2P544850	36558106	A	0	0	G
11	BICF2P680396	36561357	A	0	0	G

11	BICF2S22954856	36581190	A	0.5	0.7857	G
11	BICF2P73334	36585418	A	0	0	G
11	BICF2P1292918	36603811	A	0	0	G
11	BICF2S23759444	36605628	A	0	0	G
11	BICF2P1095405	36628500	G	0.5	0.7857	A
11	BICF2P279945	36639200	0	0	0	A
11	BICF2P1187341	36659974	A	0	0	G
11	BICF2S22940466	36669815	A	0.5	0.7857	C
11	BICF2P689546	36677461	A	0	0	C
11	BICF2S23238303	36680635	G	0.5	0.7857	A
11	BICF2P910034	36690738	A	0.5	0.7857	G
11	TIGRP2P148936_rs8651078	36705761	0	0	0	G
11	BICF2P143775	36714627	0	0	0	A
11	BICF2P534915	36728560	A	0	0	G
11	BICF2P564405	36733449	A	0	0	C
11	BICF2P101336	36752218	G	0.5	0.2143	A
11	BICF2P1308315	36759212	G	0	0	A
11	BICF2P565838	36777218	G	0	0	A
11	BICF2P1412181	36805090	G	0.5	0.8333	A
11	BICF2S23414228	36822456	G	0	0	A
11	BICF2P1379001	36829058	A	0	0	C
11	BICF2P548212	36842545	A	0	0	G
11	TIGRP2P148939_rs8945530	36846559	0	0	0	G
11	BICF2P755580	36861293	G	0	0	A
11	BICF2P1415909	36872713	C	0	0	A
11	BICF2S23728995	36885429	A	0	0	G
11	BICF2P105000	36893151	0	0	0	A
11	BICF2P466957	36904484	G	0	0	A
11	TIGRP2P148940_rs8892537	36916179	0	0	0	A
11	BICF2S23516471	36928683	A	0.5	0.9	G
11	BICF2P271217	36939360	G	0.5	0.2143	A
11	BICF2P915368	36951544	G	1	1	A
11	BICF2P97206	36961469	A	0.5	0.2143	C
11	BICF2P570440	36975665	C	0	0	A
11	BICF2P349347	36992006	G	0.5	0.2143	A
11	BICF2P349348	36992186	T	0	0	A
11	BICF2S23511333	36996546	A	0	0	G
11	BICF2P573986	37017872	C	0	0	G
11	BICF2S23054942	37021172	A	0	0	G
11	BICF2P1093441	37027914	A	0	0	G
11	BICF2P1034521	37037147	A	0	0	G
11	BICF2S23519002	37046634	G	0	0	A
11	BICF2S23016739	37053548	A	0	0	G
11	BICF2P711110	37066058	C	0	0	A
11	BICF2P1381716	37081693	C	0	0	A
11	BICF2P1381720	37083899	A	0	0	G
11	BICF2P820118	37088306	A	0	0	G

11	BICF2P1411700	37115610	G	0.5	0.2143	A
11	BICF2S23346186	37128421	A	0	0	G
11	BICF2P355130	37138903	A	0.5	0.2143	G
11	BICF2P1351518	37177482	A	0	0	G
11	BICF2P1164912	37180164	A	0	0	G
11	BICF2P1077362	37194625	G	0.5	0.2143	A
11	BICF2P1109847	37211405	G	0	0	A
11	BICF2P781952	37225492	0	0	0	G
11	BICF2P1400709	37230231	A	0	0	G
11	BICF2P402538	37247527	G	0	0	A
11	BICF2S23223694	37252333	G	0	0	A
11	BICF2P485701	37267238	A	0	0	T
11	BICF2P665711	37271998	A	0.5	0.5	C
11	BICF2S23752439	37289973	C	0	0	A
11	BICF2S23050380	37295576	C	0.5	0.5	A
11	BICF2P802836	37309297	A	0	0	G
11	BICF2P757119	37318349	A	0	0	G
11	BICF2P1000671	37333212	A	0	0.2857	G
11	TIGRP2P148945_rs8823371	37340743	0	0	0	A
11	BICF2P798208	37341657	T	0	0	A
11	BICF2P407518	37355229	A	0	0	G
11	BICF2P102071	37368439	G	0.5	0.2143	A
11	BICF2P135361	37375948	A	0.5	0.5	G
11	BICF2P1014651	37394998	A	0	0	G
11	BICF2P1107075	37406024	A	0.5	0.7857	G
11	BICF2P1099081	37432667	C	0	0	A
11	BICF2S2358332	37452062	A	0.5	0.2143	G
11	BICF2P7710	37465834	0	0	0	A
11	BICF2S23020838	37477227	A	0.5	0.2143	C
11	BICF2P302698	37483772	T	0	0.2857	A
11	BICF2P1045771	37491835	G	0	0.2857	A
11	BICF2P218562	37511188	G	0	0.2857	A
11	BICF2P277661	37523322	A	0	0.2857	G
11	BICF2S2355733	37545779	G	0	0	A
11	BICF2P932787	37549480	A	0.5	0.5	C
11	BICF2P808894	37566219	C	0	0	A
11	BICF2P834176	37577762	A	0	0	G
11	BICF2P1027338	37585065	A	0.5	0.7857	G
11	BICF2S23025025	37595375	G	0	0	A
11	BICF2S23218151	37612611	0	0	0	A
11	BICF2P827048	37617451	G	0	0	A
11	BICF2P338927	37639413	A	0	0	G
11	BICF2P4243	37645975	0	0	0	T
11	BICF2P662451	37658392	A	0	0	G
11	BICF2P643317	37664170	G	1	1	A
11	BICF2P399138	37685179	0	0	0	G
11	BICF2S23452792	37706390	A	0	0	G

11	BICF2P1105732	37718733	C	0	0	A
11	BICF2P1004779	37721858	0	0	0	C
11	BICF2P279702	37741405	G	0	0	A
11	BICF2P1400326	37750909	G	0	0	A
11	BICF2P429542	37771538	G	0.5	0.2143	A
11	BICF2S23010069	37784531	G	0.5	0.5	A
11	BICF2P380106	37790351	C	0	0	G
11	BICF2P132685	37802625	C	0.5	0.2143	A
11	BICF2P1470253	37814647	A	0	0.2857	T
11	BICF2P1347592	37830178	G	0	0.25	A
11	BICF2P746621	37840524	0	0	0	G
11	BICF2P95013	37850797	A	0	0.2857	G
11	BICF2P380973	37858084	A	0	0.2857	C
11	BICF2P1108798	37869446	G	0	0	A
11	BICF2P1127843	37881087	G	0	0	A
11	BICF2P525927	37909655	A	0	0	C
11	BICF2P1188474	37920746	A	0	0	G
11	BICF2P955933	37934980	G	0	0	A
11	BICF2P914677	37943800	G	0	0	A
11	BICF2P981804	37951325	G	0	0	A
11	BICF2P429725	37962058	G	0.5	0.2143	A
11	BICF2P1141531	37973070	A	0.5	0.7857	G
11	BICF2P809203	38005674	C	0.5	0.2143	A
11	BICF2P1211478	38007750	A	0.5	0.2143	G
11	BICF2P403296	38023239	G	0	0	C
11	BICF2P734228	38034327	A	0	0	G
11	BICF2S23413064	38052634	A	0.5	0.2143	G
11	BICF2P1429576	38062295	0	0	0	A
11	BICF2P1109069	38072199	G	0.5	0.2143	A
11	BICF2S23033585	38085049	G	0	0	A
11	BICF2S23331601	38098577	A	0.5	0.2143	G
11	BICF2S23412162	38099570	G	0.5	0.7857	A
11	BICF2S23148013	38115443	G	0	0	A
11	BICF2S23454861	38133150	G	0.5	0.2143	A
11	BICF2S23056896	38134082	G	0.5	0.2143	A
11	BICF2S2343203	38151947	T	0.5	0.1667	A
11	BICF2P1205745	38167571	G	0	0	C
11	BICF2P1031081	38174465	C	0	0	G
11	BICF2S22916123	38186960	A	0	0	G
11	BICF2P150139	38193521	A	0	0	G
11	BICF2P696062	38205431	G	0	0	A
11	BICF2S2341738	38217259	G	0.5	0.7857	A
11	BICF2P990064	38229235	G	0.5	0.7857	A
11	BICF2P706228	38253054	A	0.5	0.2143	G
11	BICF2P706227	38253092	A	0.5	0.2143	G
11	BICF2S23756520	38262588	G	0	0	A
11	BICF2P508643	38275399	A	0.5	0.2143	G

11	BICF2P87462	38286056	A	0.5	0.2143	C
11	BICF2P1178787	38297056	A	0.5	0.2143	G
11	BICF2P1248372	38314538	A	0.5	0.2143	C
11	BICF2P210015	38321112	A	0.5	0.1667	G
11	BICF2S22923332	38334763	G	0	0	A
11	BICF2P742919	38343008	0	0	0	A
11	TIGRP2P148956_rs8948919	38363212	0	0	0	A
11	BICF2S237620	38367465	A	0	0	G
11	G674f58S213	38406442	A	0	0	G
11	BICF2S22913009	38442534	A	0	0	G
11	BICF2P690434	38452005	A	0.5	0.5	G
11	BICF2P1134479	38457203	G	0.5	0.5	A
11	BICF2S23160697	38470102	C	0	0	A
11	TIGRP2P148959_rs8983706	38479993	0	0	0	G
11	BICF2P522986	38499212	C	0.5	0.5	A
11	BICF2P279938	38502177	G	0.5	0.5	A
11	BICF2P181451	38523312	G	0.5	0.5	A
11	BICF2P1332119	38536984	G	0.5	0.5	A
11	BICF2P935694	38550019	G	1	1	A
11	BICF2P1276408	38564800	A	1	1	G
11	BICF2P518644	38576675	A	0.5	0.5	C
11	BICF2P110721	38594237	A	0.5	0.5	G
11	BICF2P1145788	38616119	A	0.5	0.5	G
11	BICF2P449142	38619967	C	0.5	0.5	A
11	TIGRP2P148964_rs8852418	38634833	0	0	0	G
11	BICF2S23410270	38667551	G	0	0	A
11	BICF2P1105439	38676856	A	0	0.2857	G
11	BICF2P293051	38686888	G	1	0.6429	A
11	BICF2P317662	38717717	G	NA	NA	A
11	BICF2P939275	38727000	A	1	0.7143	G
11	BICF2P377511	38742358	0	0	0	G
11	BICF2P120637	38753223	A	0	0	T
11	BICF2P30576	38765549	A	0.5	0.2143	G
11	BICF2P1284061	38774811	G	0.5	0.5	A
11	BICF2P451731	38791349	0	0	0	A
11	BICF2P989422	38830067	G	1	0.7143	A
11	BICF2P1003196	38850161	A	1	0.7143	G
11	BICF2P288262	38859158	G	0	0	A
11	BICF2S22918197	38880554	A	0	0	G
11	BICF2P1261686	38890409	C	0	0	A
11	BICF2P226528	38893777	A	0	0	G
11	BICF2S23416726	38936961	G	0	0	A
11	BICF2S23436988	38942405	A	0	0	G
11	BICF2P644413	38961710	A	0	0	T
11	BICF2P855190	38964264	A	0	0	G
11	BICF2P212417	38979412	G	0	0	A
11	BICF2P779676	38991608	C	0	0	A

11	BICF2S23051580	39004359	A	0	0	G
11	BICF2S22960865	39010430	G	0	0	A
11	BICF2P1246533	39026880	A	0	0	G
11	BICF2P1027826	39036169	A	0	0	C
11	BICF2P1035311	39051228	A	0	0	G
11	BICF2P898353	39060009	G	0	0	A
11	BICF2P137763	39072460	A	0	0	C
11	BICF2P982187	39091985	A	0	0	G
11	BICF2P853078	39101990	A	0	0	G
11	BICF2P188020	39114436	0	0	0	C
11	BICF2P785569	39126445	G	0.5	0.2143	A
11	BICF2P445200	39154979	0	0	0	A
11	BICF2P901547	39176896	G	0	0	A
11	BICF2P677302	39183236	C	0	0	A
11	BICF2P1080107	39195910	C	0	0	A
11	BICF2P687903	39208719	A	0	0	G
11	BICF2P324220	39226378	A	0	0	C
11	BICF2P878475	39227560	A	0	0	G
11	BICF2P1415516	39239315	A	0	0	G
11	BICF2P1038522	39253715	A	0	0	G
11	BICF2P593315	39264044	A	0	0	G
11	BICF2P121911	39277520	A	0	0	G
11	BICF2P749888	39286761	A	0	0	G
11	BICF2P1413906	39293909	A	0	0	G
11	BICF2S23329688	39298225	0	0	0	G
11	BICF2P345952	39310116	G	0.5	0.7857	A
11	BICF2P412404	39325482	G	0	0	A
11	BICF2S2297995	39337125	A	0	0	G
11	BICF2P267148	39347230	A	0	0	C
11	BICF2S23632377	39360423	A	1	0.7143	C
11	BICF2P1093279	39375957	A	0	0.2857	G
11	BICF2S2326699	39408512	0	0	0	G
11	BICF2P1258076	39418367	G	0.5	0.5	A
11	BICF2P719913	39426776	A	0.5	0.5	G
11	BICF2P459103	39454083	G	0	0	A
11	BICF2S23036705	39457724	A	0	0	G
11	BICF2S23032773	39470819	A	0	0.2857	G
11	BICF2P1255810	39479501	A	0.5	0.5	G
11	BICF2P912292	39497006	A	0	0.2857	G
11	BICF2P311377	39510069	A	0.5	0.2143	G
11	BICF2S23440424	39523057	G	0	0.25	A
11	BICF2S23511120	39531811	A	0.5	0.5	T
11	TIGRP2P148976_rs9058191	39540307	0	0	0	A
11	TIGRP2P148977_rs8677243	39556313	0	0	0	A
11	BICF2P324041	39563585	0	0	0	G
11	BICF2P1415080	39575221	0	0	0	A
11	BICF2P338484	39607069	A	0	0	G

11	BICF2P1432449	39623485	A	0	0	C
11	G675f57S208	39646734	0	0	0	A
11	BICF2S23258982	39668643	G	0	0.2857	A
11	BICF2S23758223	39680075	A	0	0	G
11	BICF2S2343886	39690160	G	0	0	A
11	TIGRP2P148981_rs9211160	39705228	0	0	0	A
11	BICF2P705964	39714648	A	0	0	T
11	BICF2P780735	39721593	0	0	0	A
11	BICF2P422571	39739403	0	0	0	C
11	BICF2P215123	39745962	A	0	0	G
11	TIGRP2P148982_rs8985694	39764953	0	0	0	G
11	BICF2P1268238	39786703	G	0	0	A
11	BICF2P825657	39795123	G	0	0	A
11	BICF2P796490	39803170	A	0	0	C
11	BICF2P482675	39819919	A	0	0	G
11	BICF2P1282932	39828031	A	0	0	G
11	BICF2P708415	39839969	A	0.5	0.5	G
11	BICF2P306388	39856907	A	0.5	0.5	G
11	TIGRP2P148984_rs8717210	39861600	0	0	0	C
11	BICF2P1197260	39890362	A	0.5	0.5	G
11	BICF2P1022226	39899773	G	0.5	0.5	A
11	BICF2P43664	39908709	G	0.5	0.5	A
11	BICF2P117270	39922635	0	0	0	A
11	BICF2P517629	39930544	A	0.5	0.7857	G
11	BICF2P802184	39973384	A	0.5	0.5	G
11	BICF2P579873	39977543	C	0.5	0.5	A
11	BICF2P568118	39992720	A	1	0.7143	G
11	BICF2P98575	40004597	A	1	0.7143	G
11	BICF2P448274	40011315	A	1	0.7143	G
11	BICF2P1498580	40037533	G	0	0	A
11	BICF2P618197	40050550	G	0	0	A
11	BICF2P915487	40061429	G	0	0	C
11	BICF2S23247915	40067106	A	1	0.7143	G
11	BICF2S23623205	40078080	A	0.5	0.5	G
11	TIGRP2P148987_rs9179293	40091793	0	0	0	G
11	BICF2P415178	40129145	A	0.5	0.5	G
11	BICF2P294458	40150323	0	0	0	G
11	BICF2P508545	40162561	0	0	0	A
11	G676f29S119	40194115	0	0	0	C
11	BICF2S23617071	40211491	0	0	0	A
11	BICF2S23763376	40257619	G	0.5	0.5	A
11	BICF2P339376	40286874	0	0	0	G
11	BICF2P566472	40303294	0	0	0	A
11	G677f18S269	40328179	G	0	0.25	A
11	G677f23S268	40338861	G	0.5	0.5	A
11	BICF2P216741	40376398	0	0	0	A
11	TIGRP2P148992_rs8697339	40382938	0	0	0	C

11	BICF2S23215858	40404810	A	0.5	0.7857	G
11	BICF2S23045596	40431947	C	0	0.2857	A
11	BICF2P980632	40442512	0	0	0	A
11	G678f21S211	40464493	0	0	0	A
11	BICF2S23149944	40484049	G	0.5	0.7857	A
11	BICF2S23424821	40494704	A	0.5	0.5	G
11	BICF2S23547823	40514142	G	0.5	0.5	A
11	BICF2S23027546	40515300	A	0.5	0.5	G
11	BICF2S23737631	40559234	G	1	0.7143	A
11	BICF2S23624846	40568863	G	1	0.7143	A
11	BICF2S23217051	40579875	G	1	0.7143	A
11	BICF2S23454417	40584702	0	0	0	G
11	BICF2P688747	40602357	0	0	0	T
11	BICF2P792140	40613017	A	0	0	C
11	BICF2P22260	40632694	T	0.5	0.7857	A
11	G679f43S271	40658792	0	0	0	C
11	G680f42S273	40717567	G	0	0	A
11	BICF2P551558	40742592	G	0.5	0.5	A
11	BICF2S23912554	40752480	0	0	0	G
11	BICF2S23115728	40758228	0	0	0	A
11	BICF2P760133	40788965	G	0	0	A
11	BICF2P858820	40794422	G	0.5	0.2143	A
11	BICF2P377619	40819312	0	0	0	A
11	BICF2P969229	40831894	0	0	0	C
11	BICF2P171609	40843504	0	0	0	C
11	BICF2P504777	40853616	A	0	0	G
11	BICF2P1054989	40876201	A	0	0	G
11	BICF2P211495	40884075	0	0	0	A
11	BICF2P659484	40895414	0	0	0	G
11	BICF2P19563	40908461	A	0	0	C
11	BICF2P1171006	40967934	0	0	0	G
11	BICF2P387118	40976548	0	0	0	A
11	BICF2P821297	40993712	0	0	0	A
11	BICF2P781636	41018756	0	0	0	A
11	BICF2P190343	41030256	0	0	0	G
11	BICF2P583349	41040891	0	0	0	A
11	TIGRP2P149000_rs8979945	41058942	0	0	0	C
11	BICF2P388623	41075926	0	0	0	C
11	BICF2P119617	41107383	A	0	0	G
11	BICF2P1192319	41115369	A	0.5	0.1667	G
11	BICF2S23139506	41125075	A	0.5	0.2143	G
11	BICF2P117244	41137627	G	0.5	0.2143	A
11	BICF2S23018735	41156571	A	0.5	0.5	G
11	BICF2P574934	41160280	A	0.5	0.5	G
11	BICF2P634142	41167262	A	0.5	0.5	T
11	BICF2P1180552	41175539	G	0	0.5714	A
11	BICF2P449512	41192156	G	0.5	0.5	A

11	BICF2P592259	41195424	G	0.5	0.5	A
11	BICF2S22962694	41210783	A	0.5	0.2143	G
11	BICF2P615149	41234336	C	0.5	0.2143	A
11	BICF2P151855	41244556	G	0	0.5714	A
11	BICF2P37053	41256421	A	1	0.4286	G
11	BICF2P493036	41267274	A	1	0.7143	G
11	BICF2P783576	41277060	G	0.5	0.2143	A
11	BICF2P253325	41291692	A	0.5	0.2143	G
11	BICF2P753682	41302166	C	0.5	0.2143	A
11	BICF2P876598	41311497	G	0.5	0.2143	A
11	BICF2P634956	41322535	0	0	0	A
11	BICF2P417574	41336945	A	0.5	0.2143	G
11	BICF2P793030	41358412	A	1	1	G
11	BICF2P676056	41359032	A	1	0.7143	G
11	BICF2P332330	41367058	A	0	0	G
11	BICF2S24511149	41383617	G	0	0	A
11	BICF2P965533	41407884	0	0	0	G
11	BICF2P1374981	41417853	G	0	0	A
11	BICF2P1078565	41429543	0	0	0	A
11	BICF2P501043	41443725	A	1	0.7143	G
11	BICF2P224420	41451682	G	1	1	A
11	BICF2P1245581	41459716	G	1	1	A
11	BICF2P734629	41471051	A	0.5	0.5	G
11	BICF2P724946	41488843	A	1	0.7143	G
11	BICF2P1068706	41497418	A	1	0.7143	C
11	BICF2S22931678	41509953	A	1	0.7143	G
11	BICF2P1288166	41519641	G	1	0.7143	A
11	BICF2P1241249	41529323	A	0	0.2857	G
11	BICF2P292734	41543568	A	0	0.2857	G
11	BICF2P803662	41551984	A	0	0	G
11	BICF2P707095	41565944	0	0	0	A
11	BICF2S23742995	41576487	G	0.5	0.5	A
11	BICF2P255308	41590702	G	0	0	A
11	BICF2P857492	41596363	A	0	0.2857	G
11	BICF2S23428769	41612291	A	0	0.2857	G
11	BICF2P890783	41625398	A	0	0.2857	G
11	BICF2P798316	41637663	A	0	0.2857	G
11	BICF2P706423	41652199	C	0.5	0.2143	A
11	BICF2P1296622	41655328	C	0	0	G
11	BICF2P329450	41674500	A	0	0	G
11	BICF2S23623027	41677998	A	0	0	G
11	BICF2S23611257	41698184	A	0.5	0.5	G
11	BICF2P712872	41702602	G	1	0.7143	A
11	BICF2P569499	41706463	C	0.5	0.5	A
11	BICF2P462376	41721248	G	0.5	0.2143	A
11	BICF2P277323	41730117	G	0.5	0.5	A
11	BICF2P86952	41740372	G	0.5	0.2143	A

11	BICF2P737121	41753208	G	0.5	0.2143	A
11	BICF2P1270783	41766562	A	0.5	0.2143	G
11	BICF2P461051	41768769	A	0.5	0.2143	G
11	BICF2P751419	41781244	A	0	0	G
11	BICF2P703048	41791566	A	0.5	0.2143	G
11	BICF2P978383	41815760	G	1	0.4286	A
11	BICF2P260258	41830089	A	0	0	G
11	BICF2P971774	41843962	0	0	0	A
11	TIGRP2P149013_rs8968962	41852746	0	0	0	G
11	BICF2P1020079	41864823	A	0.5	0.2143	G
11	BICF2P152841	41877241	C	0	0	G
11	BICF2P1194593	41884540	C	0	0.25	A
11	BICF2P989114	41902363	C	0.5	0.2143	A
11	BICF2P952704	41915820	A	0	0	C
11	BICF2S23641563	41918391	C	1	0.3333	A
11	BICF2S23752402	41942290	A	1	0.4286	G
11	BICF2S23725001	42029569	G	0	0.2857	A
11	G682f16S254	42101273	0	0	0	C
11	BICF2S23028535	42122334	T	0.5	0.2143	A
11	BICF2P611886	42143138	0	0	0	A
11	BICF2S2371542	42150324	A	0	0	G
11	BICF2G630300759	42170065	0	0	0	G
11	BICF2P690918	42184060	C	0.5	0.5	A
11	BICF2P1307129	42194021	C	0	0	G
11	BICF2P348554	42215732	G	0	0	A
11	BICF2P626376	42227616	G	1	1	A
11	BICF2P617862	42234608	A	0	0	G
11	BICF2P281068	42241641	A	0	0	G
11	BICF2P353831	42251680	G	0	0	A
11	BICF2P71569	42270501	G	0	0	A
11	BICF2P496690	42278575	G	0	0.2857	A
11	BICF2P923218	42291635	C	0	0.2857	A
11	BICF2P502254	42298690	0	0	0	A
11	BICF2P32989	42315086	G	0	0	A
11	BICF2S23216574	42329340	A	0	0	C
11	BICF2P768117	42339654	G	0	0	C
11	BICF2S23525492	42348500	G	0	0	A
11	BICF2P595164	42386351	0	0	0	C
11	BICF2P833220	42390728	G	1	1	A
11	BICF2P107934	42409265	A	1	0.4286	G
11	BICF2P135759	42418351	A	1	0.4286	G
11	BICF2S23318165	42432976	A	1	0.7143	C
11	BICF2P626005	42437268	G	1	0.7143	A
11	BICF2S23027739	42437443	A	1	0.7143	T
11	BICF2P940135	42451164	C	1	0.7143	A
11	BICF2P1097410	42461433	A	0	0.2857	G
11	BICF2S23213489	42467682	G	0	0	A

11	BICF2S23431932	42475127	G	1	0.7143	C
11	BICF2P77814	42488457	G	0	0	A
11	BICF2P789480	42492830	0	0	0	A
11	BICF2P1228508	42523226	A	0	0	C
11	BICF2P885466	42536338	A	0	0.2857	G
11	BICF2P1170042	42543751	A	0	0.2857	G
11	TIGRP2P149030_rs8804497	42547191	0	0	0	G
11	BICF2P1328205	42551620	0	0	0	A
11	BICF2S2306848	42580732	A	0	0	G
11	BICF2S23238383	42592504	G	0	0.2857	A
11	BICF2P1417650	42599858	A	0	0.5714	G
11	BICF2P627039	42611363	G	0	0	A
11	BICF2P917915	42625996	G	0	0.5714	A
11	BICF2S2351449	42635936	C	0.5	0.5	A
11	BICF2P349840	42671695	A	0.5	0.8333	G
11	BICF2P1248432	42689934	A	0.5	0.7857	C
11	BICF2P228511	42700578	C	0.5	0.7857	A
11	BICF2P1121573	42713103	G	0.5	0.2143	A
11	BICF2P559217	42726364	C	0	0	A
11	BICF2P1111059	42735613	G	0.5	0.2143	A
11	BICF2P152209	42749008	A	0.5	0.5	G
11	BICF2P299625	42760832	A	0.5	0.2143	G
11	BICF2P401174	42770635	C	0.5	0.2143	A
11	BICF2P634065	42782857	G	0.5	0.7857	A
11	BICF2P367028	42797017	C	0.5	0.5	A
11	BICF2P302332	42807434	A	0.5	0.5	G
11	BICF2P1045986	42813471	C	0.5	0.5	G
11	BICF2P491872	42816114	G	0.5	0.5	A
11	BICF2P885481	42829703	A	0.5	0.5	G
11	BICF2S2361531	42854583	G	0	0.2857	A
11	BICF2S23033936	42865309	G	0	0	A
11	BICF2P1209645	42873634	A	0	0	G
11	BICF2P544059	42880163	T	0	0	A
11	BICF2P1113751	42883529	G	0	0	A
11	BICF2P1006475	42916888	A	0.5	0.7857	G
11	BICF2S23434809	42927196	C	0.5	0.5	A
11	BICF2P1311714	42931397	A	0.5	0.2143	G
11	BICF2P402862	42944081	A	0	0.2857	C
11	BICF2P1142163	42953569	G	0.5	0.1667	A
11	BICF2P141970	42964235	A	1	0.4286	G
11	BICF2P383167	42973349	A	1	0.7143	C
11	BICF2P109877	42982553	A	1	0.7143	C
11	BICF2P1139818	42991796	A	0.5	0.5	G
11	BICF2P1001799	43008412	C	0	0	A
11	BICF2P762746	43023084	A	0.5	0.5	G
11	BICF2P927915	43034057	G	0.5	0.2143	A
11	BICF2P1427537	43049365	A	0	0	G

11	BICF2P775125	43059842	C	0	0	G
11	BICF2P879936	43085147	A	0.5	0.2143	G
11	BICF2P285010	43093962	0	0	0	A
11	BICF2P972272	43107530	A	0.5	0.2143	G
11	BICF2S23353659	43116707	A	0.5	0.5	G
11	BICF2P463228	43126008	G	0.5	0.5	A
11	BICF2S23125917	43138650	C	0	0.2857	A
11	BICF2P1151956	43148591	0	0	0	A
11	BICF2P488675	43163116	A	0.5	0.5	C
11	BICF2P526897	43173486	G	0	0	A
11	BICF2P1058563	43196225	A	0.5	0.5	G
11	BICF2P266183	43209126	G	0	0	A
11	BICF2S23521430	43223978	G	0	0	C
11	BICF2S2315810	43234522	A	0	0	G
11	BICF2P206788	43262247	C	0	0	A
11	BICF2P889650	43267480	G	0	0	A
11	BICF2P205101	43283612	G	0.5	0.2143	A
11	BICF2P25846	43287519	0	0	0	G
11	BICF2P865493	43320303	0	0	0	A
11	BICF2P759476	43326235	A	0.5	0.2143	G
11	BICF2P1246274	43341999	0	0	0	G
11	BICF2S22942162	43351918	0	0	0	C
11	BICF2S23326210	43358752	G	0.5	0.7857	A
11	BICF2P6181	43367886	0	0	0	A
11	BICF2S23318417	43395822	A	0	0	G
11	BICF2S23323662	43419908	C	0	0	A
11	BICF2S23252584	43442653	A	0.5	0.7857	C
11	BICF2P608801	43449805	0	0	0	G
11	BICF2S23535379	43476907	0	0	0	G
11	BICF2P160112	43524948	0	0	0	G
11	BICF2S2293731	43528571	0	0	0	G
11	BICF2P668127	43544043	A	0.5	0.7857	T
11	BICF2P547806	43551229	A	0.5	0.2143	C
11	BICF2P1266331	43609449	A	0.5	0.2143	G
11	BICF2P215214	43626880	0	0	0	G
11	BICF2S23313347	43647669	C	0.5	0.7857	A
11	BICF2P632777	43670362	0	0	0	G
11	BICF2S23620222	43710504	0	0	0	C
11	BICF2P695620	43728686	C	0.5	0.2143	A
11	BICF2P637197	43807665	A	0	0	G
11	BICF2P603602	43815333	G	0	0	A
11	BICF2P915227	43827375	G	0	0	A
11	BICF2P1226185	43854063	A	0	0	G
11	BICF2P1051347	43862256	C	0	0	G
11	BICF2P1404562	43888582	G	0.25	0	A
11	BICF2P1363955	43899661	A	1	1	G
11	BICF2P244647	43918928	0	0	0	A

11	BICF2P303156	43933047	G	0	0	A
11	BICF2S23642349	43950585	C	0	0	A
11	BICF2S23728110	43963941	G	0	0	A
11	BICF2P700215	43967847	A	1	1	G
11	BICF2P909399	43976267	A	1	1	C
11	BICF2P241904	43994647	G	0	0	A
11	BICF2P542767	43998544	A	1	1	C
11	BICF2S2361823	44001369	C	0	0	A
11	BICF2P449143	44010634	A	0	0	G
11	BICF2P871058	44022289	G	0	0	A
11	BICF2P379107	44033776	A	1	1	G
11	BICF2P1215191	44047337	T	0	0	A
11	BICF2P1393057	44067363	A	0	0	T
11	BICF2P645624	44075438	A	0	0	G
11	BICF2P851464	44090309	A	0	0	G
11	BICF2P1042612	44097438	A	0	0	T
11	BICF2S2396873	44123665	G	0	0	A
11	BICF2P44510	44128911	G	0	0	A
11	BICF2P432191	44142760	G	0	0	A
11	BICF2P302625	44167160	A	0	0	G
11	BICF2P283052	44177978	A	1	1	G
11	BICF2S23134137	44191398	A	0	0	G
11	BICF2P122855	44206776	A	0	0	G
11	BICF2S23352137	44226544	A	0	0	G
11	BICF2S23737391	44231128	G	0	0	A
11	BICF2P595918	44246983	G	0	0	A
11	BICF2P951175	44267162	G	0.5	0.7857	A
11	BICF2P820577	44287242	0	0	0	A
11	BICF2S2347185	44306872	0	0	0	G
11	BICF2P469379	44312390	0	0	0	G
11	BICF2P296145	44333849	0	0	0	C
11	BICF2S2393232	44344664	0	0	0	A
11	BICF2S2325952	44370985	A	0	0	G
11	BICF2P19798	44389570	0	0	0	A
11	BICF2P1214772	44392733	0	0	0	G
11	BICF2P133066	44405676	A	0.5	0.7857	C
11	BICF2P118799	44414986	0	0	0	A
11	BICF2S23538466	44426498	0	0	0	A
11	BICF2P744682	44445082	A	0	0.2857	G
11	BICF2P424384	44453132	0	0	0	G
11	BICF2P904461	44466294	G	1	1	A
11	BICF2P534098	44480366	0	0	0	A
11	BICF2S2299761	44493914	0	0	0	G
11	BICF2P808103	44510154	0	0	0	A
11	BICF2P1049940	44524302	0	0	0	G
11	BICF2P724359	44547704	G	1	1	A
11	BICF2P50797	44554913	G	0.5	0.7857	A

11	BICF2P927085	44577022	A	0.5	0.1667	G
11	BICF2P579523	44591529	A	0.5	0.5	G
11	BICF2S22915698	44604579	A	0.5	0.2143	G
11	BICF2P1175025	44612973	A	0	0.2857	G
11	BICF2P328680	44630596	A	0.5	0.7857	G
11	BICF2P184297	44660715	C	0.5	0.5	A
11	BICF2P543656	44672572	A	0	0	G
11	BICF2P420381	44677565	A	0.5	0.5	G
11	BICF2S23038599	44693793	A	0	0	G
11	BICF2S23059475	44705090	A	0	0	G
11	BICF2S2418062	44713481	T	0	0	A
11	BICF2P549513	44729939	0	0	0	G
11	BICF2S22947334	44756627	G	0	0	A
11	BICF2P467548	44769205	G	1	0.7143	A
11	BICF2P209774	44774510	A	0	0	G
11	BICF2P280953	44783863	C	1	0.7143	A
11	BICF2P1320206	44793361	C	1	0.7143	A
11	BICF2P631922	44806547	A	0	0	T
11	BICF2S23727905	44818850	G	1	0.7143	A
11	BICF2S23211994	44835515	A	1	0.75	G
11	BICF2S2333483	44864295	A	1	0.7143	G
11	BICF2P581065	44880612	G	0	0	A
11	BICF2P915963	44886441	0	0	0	A
11	BICF2S23161967	44923660	G	0	0	A
11	BICF2P696637	44958279	C	0.5	0.2143	A
11	BICF2P772811	44965936	A	0	0	G
11	BICF2P358942	44984474	A	0	0	G
11	BICF2P252135	44999032	G	0	0	A
11	BICF2P1205646	45004762	A	0	0	G
11	BICF2P1094047	45029982	G	0.5	0.5	A
11	BICF2P1148640	45037509	C	0.5	0.5	A
11	BICF2P357595	45066620	A	0	0	T
11	BICF2P1353884	45094192	A	0	0	T
11	BICF2P883670	45110041	0	0	0	C
11	BICF2P253677	45147623	A	0.5	0.5	G
11	BICF2S23612092	45155802	0	0	0	G
11	BICF2P130936	45179756	0	0	0	A
11	BICF2P747033	45191870	G	0	0	A
11	BICF2P1092990	45202756	C	0	0	A
11	BICF2P1310326	45212163	A	0	0	G
11	BICF2P553101	45219630	A	0	0	G
11	BICF2P945263	45233825	G	1	1	A
11	BICF2P897045	45268401	A	0	0	G
11	BICF2S23035903	45282089	C	0.5	0.5	A
11	BICF2S23313216	45294278	G	0.5	0.5	A
11	BICF2S22940978	45308680	G	0	0	C
11	BICF2P326067	45313163	C	0	0	A

11	BICF2S2307483	45323239	A	0	0	G
11	BICF2P1106520	45339750	0	0	0	A
11	BICF2P779797	45347171	0	0	0	A
11	BICF2P592088	45367388	0	0	0	A
11	BICF2P1464790	45386307	A	0.5	0.5	C
11	BICF2P252840	45398379	0	0	0	G
11	BICF2P1208897	45409701	C	0	0.25	A
11	BICF2S23539613	45429938	A	0	0	G
11	BICF2P1004921	45458427	0	0	0	A
11	BICF2P376164	45468192	0	0	0	A
11	BICF2P94610	45477045	0	0	0	A
11	BICF2P793037	45510046	A	0	0	G
11	BICF2P698482	45517155	G	0.5	0.7857	A
11	BICF2P2085	45539910	0	0	0	A
11	BICF2P565575	45548346	0	0	0	A
11	BICF2P1284853	45555892	0	0	0	A
11	BICF2P608520	45564379	A	0.5	0.1667	C
11	BICF2P514718	45587628	0	0	0	A
11	BICF2P1162939	45600471	C	0.5	0.2143	A
11	BICF2P354483	45601462	A	0	0	G
11	BICF2P390581	45611645	A	0.5	0.2143	T
11	BICF2S2321514	45621132	G	0.5	0.2143	A
11	BICF2S22946559	45633151	A	0.5	0.2143	G
11	BICF2P639825	45640016	G	0.5	0.2143	A
11	BICF2P508867	45652639	T	0	0	A
11	BICF2P445109	45660985	G	0.5	0.2143	A
11	BICF2S23646251	45670551	C	0.5	0.2143	A
11	BICF2P935930	45678285	0	0	0	G
11	BICF2P417220	45699251	G	1	1	A
11	BICF2P1214187	45706407	C	1	1	A
11	BICF2S23036750	45721981	A	0.5	0.2143	G
11	BICF2S23036835	45725791	A	0.5	0.2143	G
11	BICF2S23129084	45746678	A	0.5	0.7857	G
11	TIGRP2P149142_rs9066282	45747349	0	0	0	G
11	BICF2P931370	45765479	A	0	0	C
11	BICF2S23149094	45771049	A	1	1	G
11	BICF2P225162	45785355	A	1	1	G
11	BICF2P829767	45811146	0	0	0	A
11	BICF2S23125400	45839978	A	1	1	G
11	BICF2S22939358	45860080	T	0	0	A
11	BICF2S22958726	45863993	G	0	0	A
11	BICF2P1062037	45874607	A	0	0	G
11	BICF2P1035641	45892929	A	0	0	G
11	BICF2P895488	45902704	A	0	0	G
11	BICF2P751277	45909153	G	0	0	A
11	BICF2P1402008	45922322	T	0	0	A
11	BICF2P563135	45935233	A	0	0	G

11	BICF2P447098	45944793	A	0	0	G
11	BICF2P182562	45960245	T	0	0	A
11	BICF2P127226	45970800	0	0	0	A
11	BICF2S23426602	45994328	0	0	0	C
11	BICF2P793084	46001562	0	0	0	G
11	BICF2P1364544	46020241	G	0	0	A
11	BICF2S23146872	46028156	0	0	0	G
11	BICF2P971958	46042054	G	0	0	A
11	BICF2P182717	46048540	G	0	0	A
11	BICF2P138114	46071634	A	0	0	G
11	BICF2P566079	46090962	G	0	0	A
11	BICF2P543167	46094426	A	0	0	G
11	BICF2P831782	46107219	A	0	0	G
11	BICF2P610065	46123230	A	0	0	T
11	BICF2P554214	46126703	A	0	0	G
11	TIGRP2P149151_rs8655834	46142264	0	0	0	A
11	BICF2P336320	46158528	A	0	0	G
11	BICF2S23714821	46162597	A	0	0	G
11	BICF2S23139736	46173907	G	1	1	A
11	BICF2S23018084	46189651	G	1	1	A
11	TIGRP2P149152_rs8930858	46203385	0	0	0	A
11	BICF2P1293369	46216701	A	0	0	G
11	BICF2P522887	46227068	A	0	0	G
11	BICF2P587033	46238346	C	0	0	A
11	BICF2P206180	46253068	A	0.5	0.7857	G
11	BICF2P1279371	46270211	G	0	0	A
11	BICF2P190879	46276994	G	0	0	A
11	BICF2S22960301	46288733	A	0	0	C
11	BICF2P913178	46304934	A	0.5	0.7857	C
11	BICF2P1030340	46328447	C	0.5	0.7857	A
11	BICF2P857657	46341162	G	0.5	0.7857	A
11	BICF2P526533	46345585	A	0.5	0.7857	G
11	BICF2P1209884	46379460	0	0	0	G
11	BICF2P1364243	46401753	0	0	0	A
11	BICF2P979113	46410436	G	0	0	A
11	BICF2P833942	46421959	0	0	0	G
11	BICF2P815662	46431451	0	0	0	C
11	BICF2P358190	46437062	0	0	0	G
11	BICF2P408669	46466693	0	0	0	G
11	BICF2P577736	46473402	G	0	0	A
11	BICF2P800096	46485740	0	0	0	G
11	BICF2P1412427	46494943	A	0	0	G
11	BICF2P760495	46508814	A	0	0	G
11	BICF2P1292455	46519965	A	0	0	G
11	BICF2P399065	46529033	A	0.5	0.7857	C
11	BICF2S23116582	46549153	0	0	0	G
11	BICF2P312548	46560701	A	0	0	G

11	BICF2P619844	46563995	0	0	0	G
11	BICF2S23010316	46582960	A	0.5	0.5	G
11	BICF2P1210347	46588154	C	0.5	0.5	A
11	BICF2P95006	46599028	A	0	0	G
11	BICF2P1168820	46611227	A	0.5	0.5	T
11	BICF2P1421907	46621466	G	0	0	A
11	BICF2P775411	46643381	A	0.5	0.5	G
11	BICF2P850766	46650757	G	0.5	0.5	A
11	BICF2P187007	46655617	A	0.5	0.5	G
11	BICF2P712983	46674014	A	0.5	0.5	C
11	BICF2S23747665	46680802	A	0.5	0.5	G
11	BICF2P1293563	46696799	A	0	0	G
11	BICF2S23659890	46708501	A	0	0	C
11	BICF2S23631060	46719416	G	0.5	0.5	A
11	BICF2P274858	46732880	0	0	0	G
11	BICF2P860484	46738459	A	0	0	G
11	BICF2P222103	46750486	A	0	0	G
11	BICF2P532512	46761510	A	0	0	G
11	BICF2P292206	46771966	T	0	0	A
11	BICF2P292207	46772068	A	0	0	G
11	BICF2P1210652	46785901	G	0	0	A
11	BICF2P604118	46799066	A	0.5	0.7857	C
11	BICF2P186535	46806996	G	0.5	0.7857	A
11	BICF2S23229447	46819643	A	0	0.25	G
11	BICF2S23336218	46838606	G	0	0	A
11	BICF2P1363557	46841479	A	0	0	G
11	BICF2P1363558	46841751	0	0	0	C
11	BICF2P292180	46860855	A	0.5	0.7857	G
11	BICF2P510357	46864844	G	0	0	A
11	BICF2P822019	46880198	G	0	0	A
11	BICF2P90662	46885511	A	0	0	G
11	BICF2P598169	46903396	T	0	0	A
11	BICF2S2378697	46919696	0	0	0	G
11	BICF2P108638	46930830	A	0	0	G
11	BICF2P108640	46931538	A	0	0	G
11	BICF2P1389991	46948403	G	0.5	0.7857	A
11	BICF2P704983	46979129	0	0	0	G
11	TIGRP2P149163_rs9128109	46998036	0	0	0	A
11	BICF2P744905	47006398	0	0	0	A
11	BICF2P703163	47020382	G	0	0	A
11	BICF2P1045820	47032463	0	0	0	C
11	BICF2P1159630	47036618	G	0	0	A
11	BICF2P910477	47047925	A	0.5	0.2143	G
11	BICF2P190322	47072725	0	0	0	G
11	BICF2P623925	47083631	0	NA	0	A
11	BICF2P910619	47093017	0	0	0	G
11	BICF2P480161	47113117	0	0	0	G

11	BICF2P168227	47126350	A	0.5	0.7857	G
11	BICF2P1376345	47127398	A	0.5	0.7857	C
11	BICF2P221070	47153895	G	0.5	0.2143	A
11	BICF2P545628	47162145	G	1	1	A
11	BICF2P890348	47179346	A	1	1	G
11	BICF2P417618	47185339	A	0	0	G
11	BICF2P102722	47206060	A	1	1	G
11	BICF2P1241047	47210057	A	0	0	C
11	BICF2P200135	47229295	G	0	0	A
11	BICF2P245467	47238300	A	0	0	C
11	BICF2P1333221	47243760	A	0	0	G
11	BICF2P109422	47267166	A	0	0	G
11	BICF2P596562	47282503	A	0	0	G
11	BICF2S23526247	47291685	G	1	1	A
11	BICF2P880621	47306190	0	0	0	A
11	BICF2S2355443	47320717	C	1	1	A
11	BICF2P147773	47339845	0	0	0	C
11	BICF2S2351143	47347854	A	1	1	G
11	BICF2P584000	47360318	A	1	1	G
11	BICF2P1073463	47369670	A	1	1	C
11	TIGRP2P149166_rs9161523	47382199	0	0	0	G
11	BICF2P411826	47395809	A	0	0	G
11	BICF2P423300	47404632	T	0	0	A
11	BICF2S22915918	47415122	C	0	0	A
11	BICF2P1262381	47426808	G	0	0	A
11	BICF2P574732	47448928	A	0	0	G
11	BICF2P890469	47452095	C	0	0	A
11	BICF2P425442	47465571	A	0	0	G
11	BICF2P1128413	47480462	C	0	0	A
11	BICF2S23738121	47489929	G	1	1	A
11	BICF2P300868	47499377	A	0	0	C
11	BICF2P306059	47507236	G	0	0	A
11	BICF2P911285	47522122	A	0	0	G
11	BICF2S23024768	47533645	G	0	0	C
11	BICF2P21492	47544247	A	0	0	G
11	BICF2S23531268	47559434	G	1	1	A
11	BICF2P1113472	47571559	0	0	0	G
11	BICF2S2444783	47592902	G	0	0	A
11	BICF2P883844	47605292	0	0	0	G
11	BICF2S23057394	47610237	G	0	0	A
11	TIGRP2P149168_rs8620472	47629857	0	0	0	A
11	BICF2S22915631	47653194	G	0	0	A
11	BICF2S22915802	47657116	A	0	0	G
11	TIGRP2P149170_rs8548665	47676413	0	0	0	A
11	BICF2S23723265	47679803	A	0	0	G
11	BICF2P888170	47693240	0	0	0	G
11	BICF2P1356220	47703252	G	1	1	A

11	BICF2P1151055	47722385	G	1	1	A
11	BICF2P149929	47726389	G	1	1	A
11	BICF2S23119494	47742815	A	0	0	C
11	BICF2P434988	47754806	A	0	0	C
11	BICF2P305045	47756974	G	0	0	A
11	BICF2P285352	47764404	A	0	0	G
11	BICF2S23149053	47773990	G	0	0	A
11	BICF2S23038964	47790432	G	0	0	A
11	BICF2S23048374	47795516	A	0	0	G
11	BICF2S23111189	47806096	A	0	0	G
11	BICF2S23635468	47825025	A	0	0	G
11	BICF2P145719	47837755	A	0	0	G
11	BICF2S23237106	47849866	A	0	0	G
11	BICF2P461854	47853491	A	0	0	C
11	BICF2S23352615	47854582	G	0	0	A
11	BICF2P1007947	47864097	C	0	0	A
11	BICF2S23247196	47877076	G	0	0	A
11	BICF2P782460	47906150	G	0	0	A
11	BICF2P267090	47913773	C	0	0	A
11	BICF2S23452166	47941909	G	1	1	A
11	BICF2P196834	47952894	0	0	0	G
11	BICF2P263515	47964185	0	0	0	C
11	BICF2P1460834	47973733	A	0	0.2857	G
11	BICF2P215738	47994938	G	0	0	A
11	BICF2P1276143	48003285	G	0	0	A
11	BICF2P408301	48013268	A	1	0.7143	G
11	BICF2S22954179	48028132	G	0	0.2857	A
11	BICF2P1124119	48054058	A	0	0	G
11	BICF2P620828	48066065	A	0	0	G
11	BICF2P245636	48073094	G	0	0	A
11	BICF2P185221	48095421	0	0	0	A
11	BICF2P199426	48151459	A	0	0	C
11	BICF2P198758	48168009	A	0	0	C
11	BICF2P1011221	48176358	A	0	0	G
11	BICF2P1239524	48190690	A	0	0	G
11	TIGRP2P149176_rs8719295	48204730	0	0	0	A
11	BICF2P1131285	48213981	G	0	0	A
11	BICF2P370999	48226822	G	0	0	A
11	BICF2P168729	48238283	C	0	0	A
11	BICF2P332889	48246252	A	0	0	G
11	BICF2P1408493	48256069	0	0	0	A
11	BICF2P345345	48274353	A	0	0	C
11	BICF2S23334802	48286285	A	1	1	G
11	BICF2P363481	48290755	A	0	0	G
11	BICF2P1403442	48303749	G	0	0	A
11	BICF2P286827	48309492	A	0	0	G
11	BICF2P1212859	48313925	0	0	0	A

11	BICF2P20196	48326811	A	0	0	G
11	BICF2P20195	48327705	A	0	0	G
11	BICF2P836935	48342641	0	0	0	A
11	BICF2S23330216	48348348	A	1	1	G
11	BICF2P1012015	48364185	A	0	0	G
11	BICF2S23439605	48370677	A	0	0	C
11	BICF2P983355	48387121	A	0	0	G
11	BICF2P114915	48393374	C	1	1	A
11	BICF2P696959	48407696	C	0	0	A
11	BICF2P157092	48417764	T	1	1	A
11	BICF2S23723400	48443301	A	0	0	G
11	G684f27S145	48462030	C	1	1	A
11	G684f32S303	48479123	G	1	1	A
11	BICF2S2376049	48492069	G	0	0	A
11	BICF2S23256992	48497088	G	1	1	A
11	BICF2P971011	48515683	A	1	1	G
11	BICF2S23637205	48519624	G	0	0	A
11	BICF2S23412031	48534559	A	0	0	G
11	BICF2S23246713	48538625	G	0	0	A
11	BICF2S23249373	48542441	G	0	0	A
11	G685f66S210	48579395	A	0	0	G
11	BICF2S23158872	48609921	C	0	0	A
11	BICF2S2347183	48633069	A	0	0	G
11	BICF2P866279	48636170	0	0	0	G
11	BICF2P997633	48657023	C	1	1	A
11	BICF2S23421745	48671095	A	0	0	G
11	BICF2S23152100	48682052	C	1	1	A
11	BICF2S23636714	48722459	G	0	0	C
11	BICF2P774750	48742287	0	0	0	A
11	BICF2S2458592	48755161	C	0	0	A
11	G686f47S141	48767819	G	0	0	A
11	BICF2P1285907	48791338	C	0	0	A
11	TIGRP2P149180_rs8500443	48799152	0	0	0	C
11	BICF2S22929901	48815832	A	0	0	G
11	BICF2S22922797	48819805	A	0	0	G
11	BICF2P186776	48830616	G	1	1	A
11	TIGRP2P149184_rs8815557	48849024	0	0	0	A
11	BICF2P1168652	48915662	A	0	0	G
11	BICF2P946206	48931026	A	1	1	C
11	BICF2P261204	48935254	A	1	1	G
11	BICF2S23748784	48952831	A	1	1	G
11	BICF2P199888	48958782	G	1	1	A
11	BICF2P269909	48968756	G	1	1	A
11	BICF2P415136	48979449	T	0	0	A
11	BICF2S23440391	49000764	A	0	0	G
11	BICF2S23439591	49003850	G	0	0	A
11	BICF2P1246346	49020679	A	0	0	G

11	BICF2P662744	49026739	A	0	0	G
11	BICF2P318936	49038004	A	0	0	G
11	G688f44S208	49064757	A	0	0	G
11	BICF2S23413012	49087166	A	0	0	G
11	BICF2S22959536	49094775	G	0	0	A
11	BICF2P625740	49106712	0	0	0	G
11	BICF2S23763532	49135869	A	0	0	G
11	BICF2P457820	49149748	C	0	0	A
11	BICF2P136387	49161446	0	0	0	G
11	BICF2S23753930	49173150	A	0	0	T
11	BICF2S22960066	49175109	G	0	0	A
11	BICF2S23724168	49188329	C	0	0	A
11	BICF2P904521	49205171	0	0	0	A
11	BICF2S23235169	49210100	G	0	0	A
11	BICF2P226287	49223791	0	0	0	A
11	BICF2S22955634	49237846	A	0	0	G
11	BICF2P878120	49319964	G	0	0	A
11	BICF2P1201772	49333697	G	0	0	A
11	BICF2S23546902	49341935	A	0	0	G
11	BICF2P872591	49347739	G	0	0	A
11	BICF2P1132957	49361096	A	0	0	G
11	BICF2S23324524	49373643	C	0	0	A
11	TIGRP2P149198_rs8919213	49383334	0	0	0	G
11	BICF2P1167623	49392903	G	0	0	A
11	BICF2P1298028	49405021	A	0	0	G
11	BICF2S23630135	49418630	G	0	0	A
11	BICF2S2358039	49436130	0	0	0	G
11	BICF2P15341	49444497	A	0	0	C
11	BICF2P683678	49455180	G	0	0	A
11	BICF2S23553072	49468345	C	0	0	A
11	BICF2P660943	49479434	A	0	0	G
11	BICF2P466013	49487649	G	0	0	A
11	BICF2S23642993	49496191	A	0	0	G
11	BICF2S2357535	49523469	C	0	0	A
11	BICF2P460649	49532805	G	1	1	A
11	BICF2P1286850	49545201	G	1	1	A
11	BICF2P918943	49564075	G	0	0	A
11	BICF2P1084043	49568709	A	0	0	G
11	BICF2P300308	49576821	A	0	0	G
11	BICF2S2357632	49591546	G	0	0	A
11	BICF2P622926	49606265	A	0	0	G
11	BICF2S23414050	49611899	A	0	0	G
11	BICF2P1402499	49623516	G	0	0	A
11	BICF2P1334387	49640323	G	0	0	C
11	BICF2S23553541	49654740	G	0	0	A
11	G689f53S248	49713719	0	0	0	C
11	G689f55S356	49714575	A	0	0	G

11	TIGRP2P149205_rs9011150	49764932	0	0	0	G
11	TIGRP2P149207_rs9016053	49785027	0	0	0	A
11	BICF2P1171040	49810962	0	0	0	A
11	BICF2S23012319	49821797	G	0	0	A
11	BICF2S23230771	49830159	G	0	0	C
11	BICF2P241004	49883010	0	0	0	A
11	BICF2P280562	49902204	0	0	0	C
11	BICF2S2457817	49919852	A	0	0	G
11	TIGRP2P149208_rs9063708	49931496	0	0	0	A
11	BICF2P1438244	49933148	0	0	0	C
11	BICF2P419374	49978689	0	0	0	A
11	BICF2P631295	49995022	0	0	0	C
11	G692f43S188	50016413	0	0	0	C
11	BICF2P769410	50044609	0	0	0	A
11	BICF2P1266357	50063676	0	0	0	T
11	BICF2S2335461	50075186	T	0	0	A
11	BICF2S22942684	50092549	A	1	1	C
11	BICF2P1139597	50101267	G	1	1	A
11	BICF2S2332607	50116295	A	1	1	C
11	BICF2S23110673	50148285	G	1	1	A
11	BICF2S23731607	50159325	G	1	1	A
11	BICF2S23149698	50164266	A	1	1	G
11	BICF2S23711217	50191006	A	1	1	G
11	BICF2S23522286	50200990	A	1	1	G
11	G693f51S238	50231293	A	1	1	T
11	G693f56S286	50233462	A	1	1	C
11	BICF2S2323965	50286560	C	1	1	A
11	G694f25S287	50302742	0	0	0	C
11	G695f35S43	50348134	0	0	0	C
11	G697f11S121	50431081	G	0	0	A
11	G698f26S218	50467690	0	0	0	A
11	BICF2P469758	50534693	A	1	0.7143	G
11	G700f32S293	50552051	0	0	0	C
11	BICF2P142249	50570805	0	0	0	A
11	BICF2P343058	50577659	0	0	0	G
11	BICF2P516156	50604853	A	0	0	C
11	BICF2P781699	50621367	A	0	0	G
11	BICF2S23258104	50630537	A	0	0	C
11	BICF2P284868	50634856	A	0	0	G
11	BICF2P123144	50647414	G	0	0	A
11	BICF2S23340038	50658370	C	0	0	A
11	BICF2S23340638	50661636	G	0	0	A
11	BICF2S23011640	50681958	A	0	0	C
11	BICF2P564568	50713638	A	0	0	G
11	BICF2P1205845	50720767	G	0	0	A
11	BICF2P350292	50726954	G	0	0	A
11	BICF2P1233736	50747224	A	0	0	G

11	BICF2S23310034	50754199	A	1	1	G
11	BICF2S23013343	50771804	A	0	0	T
11	BICF2S23741633	50775357	G	0	0	A
11	BICF2S2341185	50788702	A	1	1	G
11	TIGRP2P149252_rs8981591	50802510	0	0	0	G
11	BICF2S22924597	50810715	A	0	0	G
11	BICF2S22948407	50841199	0	0	0	G
11	BICF2P1067075	50875676	A	1	1	G
11	BICF2P1067077	50876879	A	1	0.7143	G
11	BICF2P491478	50893136	G	1	0.7143	A
11	BICF2S23018721	50899073	G	0	0	A
11	BICF2P333673	50916140	C	0	0	A
11	BICF2P589083	50926276	A	1	0.7143	G
11	BICF2P725977	50934783	0	0	0	A
11	BICF2P68068	50958778	G	1	0.7143	A
11	BICF2P1057482	50968784	A	0	0	G
11	BICF2P984935	51002324	0	0	0	A
11	BICF2P1052415	51034009	G	0	0	A
11	G701f37S366	51059403	0	0	0	A
11	BICF2P899459	51090553	A	0	0.2857	C
11	BICF2P748229	51103203	C	0	0	A
11	BICF2S24410135	51108662	A	0	0	G
11	BICF2S2292087	51134161	A	0	0	G
11	BICF2P765018	51157654	0	0	0	C
11	BICF2P369578	51174879	A	0	0	T
11	BICF2P1251910	51227832	A	0	0	G
11	BICF2P256245	51245507	0	0	0	A
11	BICF2P424899	51261797	A	0	0	G
11	BICF2S2395920	51290403	C	0	0	G
11	BICF2S23634061	51310429	G	0	0	A
11	BICF2S23635002	51315154	G	0	0	A
11	BICF2P128592	51327022	C	0	0	A
11	BICF2P835237	51360575	C	0	0	A
11	BICF2P218210	51403971	G	0	0	A
11	BICF2P156310	51416357	0	0	0	A
11	BICF2P851370	51418830	A	0	0	G
11	BICF2P385563	51430905	A	0	0	G
11	BICF2P1502842	51444831	A	0	0	G
11	BICF2P519098	51451491	A	0	0	G
11	BICF2P609895	51465004	G	0	0	A
11	BICF2P1274852	51478375	C	0	0	A
11	BICF2P553369	51486975	A	0	0	G
11	BICF2P502784	51504406	A	1	0.7143	G
11	BICF2P1167388	51510678	A	0	0	G
11	BICF2P278819	51530419	C	0	0	A
11	BICF2P140694	51537278	G	0	0	A
11	BICF2P885474	51551327	C	0	0	A

11	BICF2P1239981	51567073	T	0	0	A
11	BICF2P265255	51584388	G	0	0	A
11	BICF2P823532	51614562	0	0	0	A
11	BICF2P765592	51658169	0	0	0	A
11	BICF2P1141977	51678726	A	0	0	C
11	BICF2P1331674	51736287	0	0	0	A
11	BICF2P314314	51742735	A	0	0	G
11	BICF2P399180	51764224	0	0	0	G
11	BICF2P1114911	51797025	0	0	0	A
11	BICF2P346937	51820329	0	0	0	A
11	BICF2P1168788	51832858	A	0	0.2857	G
11	BICF2P1008365	51842314	A	0	0.2857	G
11	BICF2P400078	51854682	C	0	0.2857	A
11	BICF2P157813	51871483	A	0	0.2857	G
11	BICF2P1143084	51892236	A	0	0.2857	C
11	BICF2P555426	51945109	0	0	0	A
11	BICF2P429850	51970430	A	0	0	C
11	TIGRP2P149274_rs8956398	51989051	0	0	0	A
11	BICF2P1008617	51997211	C	0	0.2857	A
11	BICF2S23434356	52019219	A	0	0	G
11	BICF2P558943	52046833	G	0	0	A
11	BICF2P49966	52066646	G	0	0	A
11	BICF2P1054145	52091533	C	0	0	A
11	BICF2S23620648	52123518	A	0	0	G
11	BICF2P390026	52151231	A	0	0	G
11	BICF2P251746	52153625	C	0	0	A
11	BICF2P111729	52165512	A	0	0	G
11	TIGRP2P149276_rs8556027	52180881	0	0	0	G
11	BICF2P662445	52195504	G	0	0	A
11	BICF2P403329	52213439	A	0	0	G
11	BICF2S23321113	52226546	A	0	0	C
11	BICF2P199206	52255898	C	0	0	G
11	TIGRP2P149279_rs9120141	52275063	0	0	0	G
11	BICF2S23525663	52288596	0	0	0	G
11	TIGRP2P149280_rs9200645	52303999	0	0	0	G
11	BICF2P197885	52314827	0	0	0	A
11	BICF2P891943	52357324	0	0	0	A
11	BICF2P641224	52361149	0	0	0	A
11	BICF2P1362875	52371013	0	0	0	T
11	BICF2S22964956	52402938	G	0	0	A
11	BICF2S22965117	52406330	G	0	0	A
11	BICF2S22960419	52422105	G	0	0	A
11	BICF2P369690	52439434	0	0	0	A
11	BICF2S23542870	52453969	G	0	0	A
11	BICF2S23912251	52505214	0	0	0	G
11	BICF2S23628382	52518078	A	1	1	G
11	BICF2S23754121	52523033	G	1	1	A

11	BICF2P998323	52533862	A	0	0	G
11	BICF2S23416181	52559641	A	0	0	T
11	BICF2P1328573	52575241	G	0	0	A
11	BICF2P273617	52585575	C	0	0	A
11	BICF2P263856	52591612	C	1	1	A
11	BICF2P777546	52603478	0	0	0	A
11	BICF2P645865	52617559	0	0	0	A
11	BICF2S23658614	52634317	A	0	0	C
11	BICF2P1383556	52661978	G	0	0	A
11	TIGRP2P149283_rs8905001	52672296	0	0	0	C
11	BICF2P561069	52695482	0	0	0	C
11	BICF2P1051344	52714610	0	0	0	A
11	BICF2P983486	52727191	A	0	0	G
11	BICF2P596905	52732484	G	0	0	A
11	BICF2P179605	52746395	A	0	0	G
11	BICF2P596766	52761808	A	0	0	G
11	BICF2S2324173	52785198	A	0	0	G
11	BICF2P24288	52796840	A	0	0	G
11	TIGRP2P149290_rs8869838	52817916	0	0	0	G
11	G704f59S102	52832730	A	0	0	G
11	BICF2S245400	52856584	0	0	0	A
11	BICF2P506000	52882871	0	0	0	G
11	BICF2S23153864	52902595	A	0	0	G
11	BICF2P779489	52905383	G	0	0	A
11	BICF2P441340	52910666	G	0	0	C
11	BICF2P1141045	52916016	A	0	0	G
11	BICF2S23038853	52927078	G	0	0	A
11	BICF2P75740	52935880	C	0	0	G
11	BICF2S23039982	52946058	C	0	0	A
11	BICF2S23141820	52964908	G	0	0	A
11	BICF2S23144375	52968737	G	0	0	A
11	BICF2S23159413	52975685	A	0	0	G
11	BICF2S23042286	52991450	A	0	0	G
11	BICF2S23213250	52993465	G	0	0	A
11	BICF2S23032888	53011721	G	0	0	A
11	BICF2S23024657	53015531	A	0	0	C
11	BICF2P615496	53031868	0	0	0	A
11	BICF2S23017165	53056893	A	0	0	G
11	BICF2S23712261	53066506	G	0	0	A
11	BICF2P175815	53072613	A	0	0	G
11	BICF2P655613	53094144	0	0	0	C
11	BICF2P530380	53096202	A	0	0	C
11	BICF2P422536	53111219	A	0	0	G
11	TIGRP2P149302_rs9148735	53131087	A	0	0	C
11	BICF2S2417273	53160478	G	0	0	A
11	BICF2P611830	53166991	0	0	0	A
11	BICF2P1394904	53185265	G	0	0	A

11	BICF2P1179462	53207495	C	0	0	A
11	BICF2P659280	53212107	G	0	0	A
11	BICF2P995565	53234287	A	0	0	G
11	BICF2P903048	53254172	G	1	1	C
11	BICF2P1414651	53267394	A	1	1	G
11	BICF2S23640119	53272068	0	0	0	G
11	BICF2S23657443	53284293	C	0	0	A
11	BICF2S23041623	53286128	G	1	1	A
11	BICF2P730051	53297234	C	0	0	A
11	BICF2S23016136	53303452	A	0	0	G
11	BICF2S2418998	53324258	G	0	0	C
11	BICF2P790785	53326163	0	0	0	A
11	BICF2S23650669	53373174	T	0	0	A
11	BICF2P297619	53388014	0	0	0	G
11	BICF2P422853	53419943	A	0	0	C
11	BICF2S23158671	53500808	G	0	0	A
11	BICF2P781269	53518976	A	0	0	C
11	BICF2S23047912	53531188	0	0	0	C
11	BICF2S2322372	53533049	G	0	0	A
11	TIGRP2P149313_rs9115351	53544307	0	0	0	C
11	BICF2P715691	53561038	0	0	0	A
11	BICF2P616094	53578488	A	0	0	C
11	BICF2P661427	53581964	C	0	0	G
11	BICF2P1432532	53604701	A	0	0	C
11	BICF2P447228	53652718	A	0	0	G
11	BICF2P432272	53685583	0	0	0	A
11	BICF2S23242561	53702860	G	0	0	A
11	BICF2P23070	53710017	G	0	0	A
11	BICF2S23417549	53744710	0	0	0	A
11	BICF2P1228701	53807070	A	0	0	C
11	BICF2P214021	53818531	A	0	0	C
11	BICF2P477381	53835549	0	0	0	G
11	BICF2P1009749	53849600	C	0	0	A
11	TIGRP2P149316_rs8773237	53864266	0	0	0	C
11	BICF2S23642572	53880623	G	0	0	A
11	BICF2P1183453	53906459	0	0	0	G
11	BICF2P892793	53941778	A	0	0	T
11	BICF2P772508	53955286	G	1	1	A
11	BICF2P1384954	54002917	A	0	0	G
11	BICF2P637175	54006139	A	0	0	G
11	BICF2P392585	54016161	0	0	0	T
11	BICF2S23330011	54029961	A	0	0	G
11	BICF2P1413245	54046914	A	0	0	G
11	BICF2P1261843	54058129	A	0	0	G
11	BICF2P667916	54070816	A	0	0	G
11	BICF2P424128	54079329	G	0	0	A
11	BICF2S23759970	54096923	G	0	0	A

11	BICF2P738206	54127569	0	0	0	G
11	BICF2P72527	54135065	A	1	1	C
11	BICF2P1199185	54142507	A	0	0	G
11	BICF2P471735	54175569	0	0	0	G
11	BICF2S2399044	54190742	G	0	0	A
11	BICF2S23258874	54199763	G	0	0	A
11	BICF2S2447356	54221408	A	0	0	G
11	BICF2P280123	54247307	A	0	0	G
11	BICF2P435595	54268141	A	0	0	G
11	BICF2P974065	54274069	A	1	1	G
11	BICF2P1187820	54296922	G	0	0	C
11	BICF2P388304	54316796	G	0	0	A
11	BICF2P1442643	54325492	A	0	0	G
11	BICF2S23325489	54329668	A	1	1	G
11	BICF2S23652573	54361352	0	0	0	A
11	BICF2P856402	54377092	0	0	0	A
11	BICF2P1239567	54389479	G	0	0	A
11	BICF2P1125071	54399857	A	0	0	G
11	BICF2P660094	54414146	C	0	0	A
11	BICF2P1212277	54438877	C	0	0	G
11	BICF2G630300873	54451587	A	0	0	G
11	BICF2P1100325	54534211	C	0	0	A
11	BICF2P1145382	54566102	A	0	0	G
11	BICF2G630301298	54576752	G	0	0	A
11	BICF2S22955487	54600395	C	0	0	G
11	BICF2S23746787	54607280	G	0	0	A
11	TIGRP2P149492_rs8845383	54618327	0	0	0	G
11	BICF2P962086	54644824	0	0	0	A
11	BICF2P826164	54656398	G	0	0	A
11	TIGRP2P149493_rs8956241	54668548	0	0	0	A
11	TIGRP2P149494_rs9174881	54693101	0	0	0	C
11	BICF2P1243559	54695405	0	0	0	G
11	BICF2P1354233	54707790	A	0	0	G
11	BICF2S23114409	54722306	G	0	0	A
11	BICF2P534588	54737372	C	1	1	G
11	BICF2S23332143	54746673	A	0	0	T
11	TIGRP2P149505_rs8689729	54873099	0	0	0	A
11	BICF2P949134	54893363	0	0	0	G
11	BICF2S23249316	54918685	G	0	0	A
11	BICF2P806172	54930293	A	0	0	G
11	BICF2P1008860	54938559	0	0	0	C
11	BICF2S23436883	54955407	G	0	0	A
11	BICF2S23413929	54965895	A	0	0	C
11	BICF2S23711094	54979130	G	1	1	A
11	BICF2P595597	54990154	A	0	0	G
11	BICF2S2351389	55026247	A	1	1	C
11	BICF2P91280	55053314	0	0	0	A

11	BICF2S23649300	55066365	G	1	1	A
11	BICF2S23453392	55077974	G	1	1	A
11	BICF2S23445061	55086854	A	0	0	G
11	G709f51S301	55129797	A	0	0	G
11	G709f54S163	55131056	0	0	0	C
11	BICF2S23042457	55167659	0	0	0	C
11	BICF2P126079	55183954	A	0	0	C
11	TIGRP2P149509_rs8974124	55192874	0	0	0	G
11	BICF2P1395759	55207131	A	1	1	G
11	BICF2P1364813	55209473	A	1	1	G
11	BICF2P931289	55214835	A	1	1	C
11	BICF2P627520	55228797	G	0	0	A
11	BICF2P1015744	55241525	A	1	1	C
11	BICF2P1257182	55243793	0	0	0	A
11	BICF2P1253535	55248320	G	1	1	A
11	BICF2P302290	55258284	A	0	0	G
11	BICF2S23651990	55273236	0	0	0	A
11	BICF2P466438	55300155	0	0	0	C
11	BICF2P483889	55312784	0	0	0	G
11	BICF2P1114804	55315592	0	0	0	G
11	BICF2S23450293	55335346	0	0	0	A
11	BICF2S23643704	55341281	0	0	0	A
11	BICF2P1256445	55368518	A	0	0	G
11	BICF2S22918376	55387728	G	1	1	A
11	BICF2P1093773	55399184	A	0	0	C
11	TIGRP2P149512_rs8880209	55407594	0	0	0	C
11	BICF2S23243782	55432513	G	1	1	A
11	BICF2P1218201	55452657	G	1	1	A
11	BICF2P581909	55460129	0	0	0	C
11	BICF2P1134445	55471474	A	0	0	G
11	BICF2G630301313	55477564	A	0	0	G
11	BICF2P386071	55497603	A	0	0	C
11	BICF2S23247414	55500542	G	0	0	C
11	BICF2P783032	55528487	C	0	0	A
11	BICF2P95084	55536949	C	0	0	A
11	BICF2S2316320	55571399	A	0	0	G
11	BICF2P912477	55585627	G	0	0	A
11	G710f35S261	55604036	A	0	0	G
11	BICF2P358280	55630796	0	0	0	A
11	BICF2P119727	55667844	G	0	0	A
11	BICF2P218090	55672611	G	1	1	A
11	BICF2P482720	55691973	G	1	1	A
11	BICF2S23118240	55700341	A	0	0	G
11	BICF2P870304	55704360	G	1	1	A
11	BICF2P870305	55706395	G	1	1	A
11	BICF2P477633	55719287	A	1	1	G
11	BICF2P687630	55747598	A	1	1	C

11	BICF2S22963793	55752508	G	1	1	A
11	BICF2S23660598	55765644	G	0	0	A
11	G711f45S76	55809049	A	1	1	G
11	BICF2S23154509	55833730	A	0	0	G
11	BICF2P546761	55940453	0	0	0	T
11	G713f30S184	55980131	G	0	0	A
11	BICF2P715936	56008800	A	0	0	G
11	BICF2P1295013	56030477	A	0	0	G
11	BICF2P862694	56045996	A	0	0	G
11	BICF2P982804	56061180	C	0	0	A
11	BICF2P155141	56063695	A	0	0	C
11	BICF2P1146396	56080120	G	0	0	A
11	BICF2P387961	56094569	C	0	0	A
11	BICF2P268806	56107179	G	0	0	A
11	BICF2P103819	56116672	A	0	0	G
11	BICF2P795225	56120789	A	0	0	G
11	BICF2P32044	56137876	T	0	0	A
11	BICF2P32049	56138994	A	0	0	G
11	BICF2P1154270	56163280	G	0	0	A
11	BICF2P1248770	56166597	G	0	0	A
11	BICF2P592493	56178951	G	0	0	A
11	BICF2P868938	56207077	0	0	0	C
11	TIGRP2P149576_rs8992846	56226392	0	0	0	G
11	BICF2S2348309	56244446	A	0	0	G
11	BICF2P1271639	56246551	G	0	0	A
11	BICF2S22963047	56263450	A	1	1	G
11	BICF2S23356536	56291317	G	0	0	A
11	BICF2S24512143	56299400	0	0	0	T
11	BICF2P1337317	56316714	G	0	0	A
11	BICF2P740918	56329893	A	0	0	G
11	BICF2P381953	56349348	A	0	0	G
11	BICF2P149554	56356001	G	0	0	A
11	BICF2P152356	56362544	A	1	1	G
11	BICF2S23118223	56377400	G	0	0	A
11	BICF2S24115875	56386440	C	0	0	A
11	TIGRP2P149581_rs8908933	56398495	0	0	0	A
11	BICF2S2361917	56413396	C	1	1	A
11	BICF2S23260716	56427282	G	0	0	A
11	BICF2P844655	56461991	0	0	0	A
11	BICF2S23220226	56480308	G	0	0	A
11	BICF2P693837	56506042	0	0	0	C
11	BICF2P381074	56519561	0	0	0	G
11	BICF2S23019795	56529480	C	0	0	A
11	BICF2S23129791	56535777	C	0	0	A
11	BICF2S2365240	56559320	0	0	0	G
11	BICF2S2341209	56591149	A	0	0	G
11	BICF2P505824	56605414	0	0	0	C

11	BICF2S2305829	56619845	A	0	0	G
11	BICF2S23234959	56631125	C	0	0	G
11	BICF2P1042761	56671495	0	0	0	G
11	BICF2S23019225	56687671	T	0	0	A
11	TIGRP2P150167_rs8726474	56698433	0	0	0	G
11	BICF2S23547355	56710322	A	0	0	C
11	BICF2P920645	56724853	C	0	0	G
11	BICF2S2414003	56732345	C	1	1	A
11	BICF2S23438390	56761908	G	1	1	A
11	BICF2S23438525	56765064	G	1	1	A
11	TIGRP2P150168_rs8820303	56792885	0	0	0	C
11	BICF2P36149	56827680	A	1	1	G
11	BICF2S23642526	56839514	A	0	0	G
11	BICF2P1140002	56883534	A	0	0	G
11	BICF2P1309525	56945751	0	0	0	A
11	BICF2S23528533	56953968	C	0	0	A
11	BICF2P473195	57032893	G	0	0	A
11	BICF2S23633992	57045584	A	0	0	C
11	BICF2S2442659	57096892	0	0	0	A
11	BICF2P553050	57102348	0	0	0	A
11	BICF2P201651	57115404	A	0	0	G
11	BICF2P1215923	57129227	A	0	0	G
11	BICF2S23057454	57132554	A	0	0	C
11	BICF2S22934314	57147508	G	0	0	A
11	BICF2P531926	57160450	0	0	0	A
11	BICF2P194734	57184707	A	0	0	G
11	BICF2P150808	57201677	A	0	0	C
11	BICF2P756578	57215661	0	0	0	G
11	BICF2P1241588	57224260	A	0	0	G
11	BICF2P1415334	57244368	C	0	0	A
11	BICF2P381660	57249000	G	0	0	A
11	BICF2P818075	57260953	C	0	0	A
11	BICF2S235577	57279127	G	0	0	A
11	BICF2P752610	57290378	G	0	0	A
11	BICF2P1040099	57297351	A	0	0	G
11	BICF2P1014840	57311961	A	0	0	G
11	BICF2P836542	57321889	A	0	0	G
11	BICF2P31046	57329046	G	0	0	A
11	BICF2P436434	57346227	A	0	0	G
11	BICF2P1041176	57353092	A	0	0	G
11	BICF2S23337897	57376306	A	0	0	G
11	BICF2P1084386	57389398	0	0	0	C
11	BICF2S22915080	57397026	G	0	0	A
11	BICF2P683041	57410147	A	0	0	C
11	BICF2S2309166	57418068	G	0	0	A
11	BICF2P352755	57419846	G	0	0	A
11	BICF2P1252126	57424170	C	0	0	G

11	BICF2P995730	57437770	G	0	0	A
11	BICF2P661028	57444886	T	0	0	A
11	BICF2S22946523	57450192	G	0	0	A
11	BICF2P630124	57458186	G	1	0.7143	A
11	BICF2P832137	57475353	G	0	0	A
11	BICF2P761921	57480804	A	0	0	G
11	BICF2P4731	57491760	C	0	0	A
11	BICF2P631700	57500057	G	0	0	A
11	BICF2S23432141	57517455	G	0	0	A
11	BICF2P892696	57529881	0	0	0	G
11	BICF2P1323603	57541047	A	0	0	G
11	TIGRP2P150231_rs8498546	57549648	0	0	0	A
11	TIGRP2P150232_rs8848490	57561267	0	0	0	G
11	BICF2S23019665	57579230	G	0	0.2857	A
11	BICF2P1248668	57598272	0	0	0	A
11	BICF2S23257510	57620315	A	1	0.7143	G
11	BICF2P267484	57650778	0	0	0	A
11	BICF2P1212187	57675169	0	0	0	A
11	BICF2S22956720	57691567	C	1	0.7143	A
11	BICF2S22956721	57691659	A	1	0.7143	C
11	BICF2S241610	57699911	A	0	0.2857	G
11	BICF2S23246736	57709630	A	1	1	G
11	BICF2P332937	57724943	G	0	0	A
11	BICF2S22964973	57749340	A	1	1	G
11	BICF2S22960086	57764338	G	0	0	A
11	BICF2S23756393	57765281	C	0	0	A
11	BICF2S23756395	57765589	A	0	0	G
11	BICF2S22943912	57809689	C	0	0	A
11	BICF2S232456	57811925	0	0	0	G
11	BICF2P999995	57836375	A	1	0.7143	G
11	BICF2P703710	57864621	0	0	0	G
11	BICF2S23435826	57869540	G	0	0	A
11	BICF2P879418	57879514	0	0	0	C
11	BICF2P275615	57895041	0	0	0	G
11	BICF2S23118925	57913651	A	0	0	G
11	BICF2S23517705	57921078	A	0	0	G
11	BICF2S23018057	57950406	0	0	0	A
11	BICF2S23141869	57973544	G	0	0	A
11	BICF2P760556	58002809	0	0	0	C
11	BICF2S23113984	58006310	C	0	0	G
11	BICF2S22950933	58026036	G	0	0	A
11	TIGRP2P150242_rs9211208	58030654	0	0	0	G
11	BICF2S23222414	58044219	G	1	1	A
11	BICF2S2339072	58059159	A	1	1	C
11	BICF2P1053788	58065598	G	0	0	A
11	BICF2P628645	58077495	A	1	0.7143	G
11	BICF2P1375586	58096422	0	0	0	A

11	BICF2P1172380	58104024	A	1	0.7143	G
11	BICF2P78414	58113328	A	1	0.7143	C
11	BICF2P1117272	58136553	A	1	0.7143	G
11	BICF2S23734200	58149118	G	1	0.7143	A
11	BICF2P1384665	58160390	G	1	0.7143	A
11	BICF2P1047879	58166816	C	1	0.7143	G
11	BICF2P471203	58170361	C	0	0.2857	A
11	BICF2P278568	58179902	G	1	0.7143	A
11	BICF2S23435499	58195009	A	1	1	C
11	BICF2P6649	58204638	0	0	0	G
11	TIGRP2P150248_rs8652846	58214452	0	0	0	G
11	BICF2S22960253	58224558	G	0	0	A
11	BICF2P453433	58245601	A	0	0	T
11	BICF2P862026	58254418	A	0	0	G
11	BICF2P376563	58260706	A	0	0	G
11	BICF2P350522	58274682	G	0	0	A
11	BICF2P1351119	58285752	A	0	0	G
11	BICF2P551015	58309160	0	0	0	G
11	BICF2S23648925	58333175	A	0	0	T
11	BICF2P1343522	58347609	A	1	1	G
11	BICF2P1049455	58359006	C	1	0.7143	G
11	BICF2P625420	58371682	0	0	0	G
11	BICF2S22921925	58377984	A	0.5	0.2857	C
11	BICF2S23118349	58389087	C	0	0	A
11	BICF2P1219469	58418047	0	0	0	C
11	BICF2P637856	58424851	0	0	0	C
11	BICF2S23336725	58449835	A	0.5	0.4286	G
11	BICF2S23647328	58460552	T	0	0	A
11	BICF2P175878	58476103	A	1	0.7143	G
11	BICF2P1310827	58477636	G	1	0.7143	A
11	BICF2P1045956	58479716	G	1	0.7143	A
11	BICF2P736708	58492825	A	0	0	G
11	BICF2P981077	58502606	C	0.5	0.4286	A
11	BICF2P133599	58525342	A	1	0.7143	G
11	BICF2S23654079	58537517	A	1	0.7143	G
11	BICF2S23025910	58553223	A	1	1	G
11	BICF2S23651434	58561049	A	1	1	G
11	BICF2P161687	58591484	G	0	0	C
11	BICF2S23634646	58604294	A	0	0	G
11	BICF2S22963386	58666558	A	0	0	G
11	BICF2P730850	58678470	A	0	0	G
11	BICF2S23145467	58695144	A	0.5	0.2857	G
11	BICF2S23112224	58704432	G	0.5	0.5714	A
11	BICF2P1299647	58707790	A	0	0	G
11	BICF2P542346	58724919	A	1	1	G
11	BICF2P567976	58730592	G	1	1	A
11	BICF2S23655668	58753036	A	1	1	T

11	BICF2S23656408	58757722	G	0	0.2857	A
11	BICF2S2448390	58765347	G	0	0	A
11	BICF2S2335024	58784386	G	1	1	A
11	BICF2P828383	58793046	A	0	0	G
11	BICF2P443971	58801630	A	0.5	0.7143	G
11	BICF2P881809	58811467	A	0.5	0.2857	G
11	BICF2P518727	58823928	G	0.5	0.5714	A
11	BICF2P453053	58841542	C	0	0	A
11	BICF2P35873	58846330	A	0	0	G
11	BICF2P314191	58867916	0	0	0	G
11	BICF2S2345871	58870622	A	0	0.2857	C
11	BICF2S23655961	58883702	G	0	0.2857	A
11	BICF2P486449	58900877	A	0	0.2857	G
11	BICF2P622643	58915024	G	1	0.7143	A
11	BICF2P305527	58926648	A	1	1	G
11	BICF2P517086	58943615	A	0	0.2857	G
11	BICF2P51913	58968416	C	0.5	0.5714	A
11	BICF2P314199	58978311	A	0.5	0.2857	G
11	BICF2P1353304	58992466	C	0.5	0.5714	A
11	BICF2P1353308	58993804	G	0.5	0.2857	A
11	BICF2S23316102	59004863	A	1	1	G
11	BICF2S23316103	59005051	A	1	1	G
11	BICF2S23318835	59008611	A	1	1	G
11	BICF2P945931	59022041	A	0.5	0.5714	G
11	BICF2S23618096	59029843	G	0	0.2857	A
11	BICF2P1252366	59043779	A	0	0	G
11	BICF2S23218356	59054858	C	0	0	A
11	BICF2S24413049	59068399	0	0	0	C
11	BICF2P1111366	59080665	G	0	0.2857	A
11	BICF2S23527635	59095909	A	0	0.2857	G
11	BICF2P236811	59104369	G	0	0.2857	A
11	BICF2S23052810	59110805	A	0	0.2857	C
11	BICF2P201769	59135266	A	0	0	G
11	BICF2P899267	59152993	A	1	0.7143	G
11	BICF2P949706	59158957	0	0	0	G
11	BICF2P211675	59169119	A	0	0	G
11	BICF2P7371	59179546	G	1	1	A
11	BICF2P1277100	59194378	A	0.5	0.2857	C
11	BICF2S23650067	59202654	G	0	0.2857	A
11	BICF2P197455	59215219	A	0	0.2857	G
11	BICF2P1135816	59227045	A	0	0.2857	G
11	BICF2P611845	59239945	A	0	0.2857	G
11	BICF2P1272336	59252317	G	0	0.2857	A
11	BICF2P129432	59268112	C	0	0	G
11	BICF2P1348969	59277805	G	0.5	0.2857	A
11	BICF2P1313850	59286507	C	0	0	A
11	BICF2P174156	59300071	A	0	0	C

11	BICF2S2336642	59316903	G	1	1	A
11	BICF2P339463	59325717	G	0	0	A
11	BICF2S22963749	59337139	A	0	0	G
11	BICF2P1385469	59345873	A	0	0	G
11	BICF2P1289121	59356295	G	0	0	A
11	BICF2P345495	59363648	G	0.5	0.4286	A
11	BICF2P447533	59381572	G	0.5	0.7143	A
11	BICF2S23739951	59393395	A	0	0	G
11	BICF2P1053202	59400580	A	1	1	G
11	BICF2P1070708	59410645	A	0	0	G
11	BICF2P1070700	59411402	G	0	0	A
11	BICF2G630301374	59423600	G	0	0.2857	A
11	BICF2G630301378	59436022	G	0.5	0.4286	A
11	BICF2S23612332	59450485	A	0.5	0.25	G
11	BICF2G630301384	59466043	A	0.5	0.2857	G
11	BICF2G630301394	59474365	G	0.5	0.5	A
11	BICF2G630301427	59488115	A	0.5	0.4286	G
11	BICF2G630301433	59490726	A	0.5	0.2857	G
11	BICF2G630301445	59501622	G	0	0.2857	A
11	BICF2G630301454	59516189	A	0	0.2857	G
11	BICF2G630301462	59530464	G	0.5	0.7143	A
11	BICF2G630301466	59536678	A	0.5	0.25	C
11	BICF2P462051	59550120	G	0	0	A
11	BICF2G630301476	59559396	A	0	0	C
11	BICF2P1416724	59581150	T	0.5	0.7143	A
11	BICF2P198572	59588708	0	0	0	G
11	BICF2G630301499	59595781	A	0	0	G
11	BICF2G630301540	59618531	G	0.5	0.2857	A
11	BICF2G630301565	59630312	G	0	0	A
11	BICF2G630301592	59645562	G	0	0	A
11	G716f42S197	59682151	G	0	0	A
11	BICF2G630301604	59737797	A	0.5	0.5714	G
11	BICF2G630301605	59745218	A	0	0.2857	G
11	BICF2P1148879	59775576	G	0.5	0.5714	A
11	BICF2P800182	59782358	0	0	0	A
11	BICF2P1090078	59793408	G	0	0	A
11	BICF2P1355101	59801688	T	0	0	A
11	BICF2P359835	59812326	G	0	0	A
11	BICF2P866429	59832234	A	0	0	G
11	BICF2G630301611	59856338	G	1	0.7143	A
11	BICF2G630301614	59862348	A	0.5	0.2857	G
11	BICF2G630301620	59874134	A	0	0	G
11	BICF2G630301624	59883903	A	0	0.2857	C
11	BICF2G630301636	59889682	C	0	0.2857	A
11	BICF2S23035805	59897924	G	0.5	0.4286	A
11	BICF2G630301639	59906022	A	0	0	G
11	BICF2G630301655	59926261	A	0	0	G

11	BICF2P171803	59958535	0	0	0	A
11	BICF2P858178	59966128	0	0	0	A
11	BICF2S23044578	59976585	C	0.5	0.5714	A
11	BICF2S23549996	59985654	G	0.5	0.5714	A
11	BICF2S23447977	60001828	T	0.5	0.4286	A
11	BICF2G630301658	60025764	G	0	0	A
11	BICF2G630301659	60036664	0	0	0	G
11	BICF2G630301661	60058140	0	0	0	A
11	BICF2G630301663	60072472	A	0.5	0.7143	G
11	BICF2G630301668	60082577	A	0	0.2857	G
11	BICF2S23415362	60090196	A	0	0.2857	G
11	BICF2G630301690	60105602	A	0	0.2857	G
11	BICF2G630301703	60113728	C	0	0.2857	A
11	BICF2G630301719	60122803	A	0	0.2857	G
11	BICF2P24585	60144974	G	0.5	0.7143	A
11	BICF2G630301744	60156181	A	0	0	G
11	BICF2G630301746	60156813	A	0	0	G
11	BICF2G630301762	60176894	A	0	0.2857	T
11	BICF2P757041	60180557	C	0	0.2857	G
11	BICF2P369385	60199341	G	0	0.2857	A
11	BICF2P1302920	60203554	A	0	0.25	G
11	BICF2G630301766	60223036	C	0	0	A
11	BICF2P723618	60229783	G	0	0.2857	A
11	BICF2P796456	60239028	G	0	0.2857	A
11	BICF2P654458	60251003	C	0	0.2857	A
11	BICF2P249655	60265851	A	0	0.2857	G
11	BICF2P468149	60288293	G	0	0.2857	A
11	BICF2P28354	60299180	A	0	0.2857	G
11	BICF2P419681	60309060	A	0	0.2857	G
11	BICF2G630301773	60319951	G	0	0	A
11	BICF2G630301790	60334530	G	0	0	A
11	BICF2P263166	60342920	A	0	0.2857	G
11	BICF2P745670	60355709	G	0	0.2857	A
11	BICF2S23022653	60366483	A	0	0	G
11	BICF2P564441	60380107	C	0	0	A
11	BICF2P838085	60395167	A	0	0	G
11	BICF2P1228584	60401767	A	0	0.2857	G
11	BICF2P898674	60407705	A	0	0	G
11	BICF2P200118	60414304	A	0	0.2857	G
11	BICF2P451518	60421740	0	0	0	G
11	BICF2G630301823	60439699	A	0	0	G
11	BICF2S24317378	60453035	G	1	0.7143	A
11	BICF2G630301833	60457262	G	1	0.7143	A
11	BICF2G630301838	60469038	A	0.5	0.2857	C
11	BICF2S23722734	60486995	A	0.5	0.4167	C
11	BICF2G630301850	60494409	G	0.5	0.2857	A
11	BICF2P615340	60495499	G	0	0.2857	C

11	BICF2G630301856	60501590	A	0.5	0.2857	T
11	BICF2P128276	60516305	A	0	0.2857	G
11	BICF2P628690	60516868	A	0	0.2857	G
11	BICF2G630301872	60524366	T	0	0	A
11	BICF2G630301874	60530836	A	0.5	0.2857	G
11	BICF2G630301882	60537529	A	0.5	0.2857	G
11	BICF2G630301901	60565859	G	0	0	A
11	BICF2P1168374	60576781	0	0	0	A
11	BICF2G630301903	60590873	0	0	0	G
11	G717f33S96	60616005	0	0	0	C
11	BICF2P871046	60619334	0	0	0	A
11	TIGRP2P150279_rs8906774	60695570	0	0	0	T
11	BICF2G630301917	60702455	C	0.5	0.2857	A
11	BICF2G630301919	60711085	G	0	0	A
11	BICF2G630301923	60725161	A	0.5	0.2857	G
11	BICF2G630301931	60732704	A	0	0	C
11	BICF2G630301939	60751895	C	0	0	A
11	BICF2G630301945	60760668	C	0	0	A
11	BICF2G630301953	60766981	A	0	0	G
11	BICF2G630301968	60779477	A	0	0	G
11	BICF2G630301972	60789299	A	0	0	G
11	BICF2G630301986	60810683	C	0	0	A
11	BICF2G630301987	60813549	A	0	0	C
11	BICF2P145002	60829735	G	0.5	0.2857	A
11	BICF2G630302000	60838978	G	0	0	A
11	BICF2G630302013	60851577	G	0	0	A
11	BICF2G630302024	60860349	G	0.5	0.2857	C
11	BICF2G630302047	60870117	A	0	0	G
11	BICF2S23720072	60889263	G	0.5	0.2857	A
11	BICF2P493492	60895875	A	0.5	0.2857	G
11	BICF2P1293660	60904819	A	0	0	G
11	BICF2G630302123	60925814	G	0	0	A
11	BICF2G630302126	60928888	G	0	0	A
11	BICF2G630302147	60939848	A	0.5	0.2857	C
11	BICF2P1140301	60957813	G	0	0	A
11	BICF2G630302182	60971704	C	0.5	0.2857	G
11	BICF2G630302183	60971760	G	0.5	0.2857	A
11	BICF2P405127	60981048	A	1	0.75	C
11	BICF2G630302202	60986605	A	0	0	G
11	BICF2P603789	61005055	G	0	0.2857	A
11	BICF2G630302242	61021140	A	0	0.2857	G
11	BICF2S23754200	61033448	A	0	0	G
11	BICF2G630302263	61043928	A	0	0	G
11	BICF2G630302283	61057312	G	0	0	A
11	BICF2G630302285	61071397	G	0	0	A
11	BICF2G630302290	61079229	G	0	0.2857	A
11	BICF2P873622	61095104	G	0	0	A

11	BICF2S23446084	61106521	G	0	0	A
11	BICF2G630302306	61113255	0	0	0	A
11	BICF2G630302307	61129159	0	0	0	C
11	BICF2S23223545	61156541	G	0.5	0.2857	A
11	BICF2G630302331	61160402	C	0	0.2857	A
11	BICF2G630302351	61169939	A	0	0.2857	C
11	BICF2G630302352	61174233	G	0	0.2857	A
11	BICF2G630302356	61190060	G	0	0.2857	A
11	BICF2G630302368	61194786	A	0	0	G
11	BICF2P1063190	61205073	A	0	0.2857	G
11	BICF2P940896	61215654	G	1	0.7143	A
11	BICF2P684919	61227435	G	0	0	A
11	BICF2G630302393	61242502	A	0	0	G
11	BICF2G630302407	61265602	G	0.5	0.4286	A
11	BICF2P359467	61273637	A	0.5	0.7143	G
11	BICF2G630302417	61299959	G	0.5	0.7143	A
11	BICF2S2317919	61307474	A	0	0	G
11	BICF2S23110572	61321902	A	0	0	G
11	BICF2G630302458	61330267	A	0	0	G
11	BICF2G630302488	61341588	A	0	0	C
11	BICF2P1350151	61363681	A	0	0	T
11	BICF2G630302548	61372973	A	0	0	G
11	BICF2G630302559	61381491	G	0	0	A
11	BICF2S23141993	61391301	A	0	0	T
11	BICF2G630302605	61397029	0	0	0	T
11	BICF2G630302609	61401079	A	0	0	C
11	BICF2P815744	61413940	G	1	1	A
11	BICF2P815753	61414660	G	1	1	A
11	BICF2G630302622	61422996	C	1	1	A
11	BICF2S23134972	61435254	G	1	1	A
11	BICF2G630302628	61449765	G	0	0	A
11	BICF2G630302636	61456504	G	0	0.2857	A
11	BICF2G630302651	61469468	A	0	0	C
11	BICF2P378373	61479835	0	0	0	G
11	BICF2P1378754	61492902	0	0	0	G
11	BICF2S2311442	61517461	0	0	0	G
11	BICF2S2362555	61552912	A	1	0.7143	G
11	BICF2S22959009	61576119	G	0	0	A
11	BICF2S22958866	61580558	A	1	0.7143	G
11	BICF2S23617550	61585254	C	0	0	A
11	BICF2P382756	61597148	0	0	0	A
11	BICF2P456166	61605878	0	0	0	C
11	BICF2P1293007	61625024	0	0	0	A
11	BICF2G630302673	61638067	A	0	0.2857	G
11	BICF2P820985	61644238	0	0	0	A
11	BICF2G630302693	61654134	C	0	0	A
11	BICF2S2449675	61666729	G	0	0	A

11	BICF2S23619325	61684787	G	0	0	A
11	BICF2S2452007	61695096	C	0	0	A
11	G719f34S63	61706285	A	0	0	G
11	BICF2S2345788	61728110	G	0	0	A
11	BICF2P707152	61734001	0	0	0	A
11	BICF2G630302696	61751566	A	0	0	C
11	BICF2G630302701	61755547	A	0	0	G
11	BICF2G630302739	61784806	A	0	0	G
11	BICF2G630302768	61800824	A	1	1	G
11	BICF2G630302780	61809373	G	0	0	A
11	BICF2G630302791	61819774	A	0	0	G
11	BICF2G630302810	61829964	C	1	1	A
11	BICF2G630302849	61842302	G	0	0	A
11	BICF2G630302860	61850975	A	1	1	G
11	BICF2G630302870	61863776	G	1	0.7143	A
11	BICF2G630302878	61870770	A	1	1	G
11	BICF2G630302897	61884590	A	0.75	0.7143	G
11	BICF2G630302938	61896781	A	0.25	0.2857	G
11	BICF2G630302966	61909796	G	0.75	0.7143	A
11	BICF2G630302993	61924979	A	1	1	G
11	BICF2G630302999	61935768	A	0	0	G
11	BICF2G630303003	61944413	A	0	0	T
11	BICF2G630303021	61951273	G	0	0	A
11	BICF2G630303036	61964686	A	0	0	G
11	TIGRP2P150311_rs9128512	61981164	0	0	0	G
11	BICF2G630303060	61985844	A	0	0	G
11	BICF2P320982	62003133	G	0	0	A
11	BICF2P320983	62003230	A	0.75	0.7143	G
11	BICF2S23733325	62014096	G	0.75	0.7143	A
11	BICF2G630303091	62023857	A	0	0	C
11	BICF2G630303100	62034660	C	0	0	A
11	BICF2S23033811	62047784	0	0	0	G
11	BICF2P226645	62061556	A	0	0	G
11	BICF2P1120063	62066999	A	0	0	G
11	BICF2P287175	62077189	C	0	0	A
11	BICF2G630303121	62095338	A	0	0	C
11	BICF2G630303137	62103093	A	0.25	0.2857	G
11	TIGRP2P150313_rs8726316	62114592	0	0	0	A
11	BICF2G630303172	62129767	A	0	0	C
11	BICF2G630303183	62145430	C	1	1	A
11	BICF2G630303195	62153151	G	0	0	A
11	BICF2G630303208	62165189	A	0.75	0.7143	G
11	BICF2S23552105	62170805	A	0.75	0.75	G
11	BICF2P389896	62184833	G	0.25	0.2857	A
11	BICF2P607507	62201758	A	0	0	C
11	BICF2G630303245	62204904	A	0	0	G
11	BICF2G630303275	62224599	A	0	0	G

11	BICF2G630303276	62231523	A	0	0	G
11	BICF2G630303279	62244418	T	0	0	A
11	BICF2P516371	62261665	G	0.75	0.7143	A
11	BICF2G630303326	62272931	G	0.75	0.7143	A
11	BICF2P129881	62289825	0	0	0	A
11	BICF2G630303351	62297080	A	0	0	G
11	BICF2G630303358	62311447	A	0	0	G
11	BICF2G630303387	62329626	A	0	0	G
11	BICF2G630303397	62333730	G	0	0	C
11	BICF2P723934	62342549	0	0	0	A
11	TIGRP2P150318_rs8850547	62355227	0	0	0	A
11	BICF2G630303436	62373218	A	0.25	0.2857	G
11	BICF2G630303444	62379796	G	1	1	A
11	BICF2G630303463	62392822	A	1	1	C
11	BICF2S23056947	62401369	A	0.25	0	G
11	BICF2P690138	62411208	A	0.75	0.7143	G
11	BICF2G630303492	62430877	G	0.75	0.7143	A
11	BICF2G630303504	62433806	A	0.25	0.2857	G
11	BICF2G630303521	62445709	A	1	1	G
11	BICF2G630303533	62457025	A	0	0	C
11	BICF2P510077	62470938	A	0	0	G
11	BICF2G630303576	62486971	A	0	0	G
11	BICF2G630303582	62491192	A	0.75	0.7143	G
11	BICF2G630303629	62512306	G	0	0	A
11	BICF2G630303641	62515328	A	0.75	0.7143	G
11	BICF2G630303663	62529830	G	0	0	A
11	BICF2G630303674	62548284	C	1	1	A
11	BICF2G630303688	62565277	A	0.75	0.7143	C
11	BICF2G630303691	62571559	G	0.75	0.7143	A
11	BICF2G630303715	62589531	A	0.75	0.7143	G
11	BICF2G630303729	62599777	T	0.75	0.7143	A
11	BICF2G630303741	62606632	G	1	1	A
11	TIGRP2P150349_rs8862823	62623903	G	0	0	A
11	BICF2G630303801	62638018	G	1	0.7143	A
11	BICF2S23758955	62656471	0	0	0	G
11	BICF2G630303823	62676412	G	0.5	0.5	A
11	BICF2G630303849	62689049	A	0.75	0.6429	C
11	TIGRP2P150359_rs9124819	62705736	0	0	0	A
11	BICF2S23045180	62714684	A	0.25	0.1429	G
11	BICF2P1079031	62735071	A	0	0	G
11	BICF2P1039769	62744282	0	0	0	G
11	BICF2S2454169	62766998	T	0.5	0.5	A
11	BICF2G630303869	62768016	G	0.75	0.8571	A
11	BICF2G630303896	62779287	C	1	1	A
11	BICF2G630303932	62796323	A	0.5	0.5	G
11	BICF2G630303955	62814008	C	0.25	0.1429	A
11	BICF2S23150123	62832863	G	0.5	0.5	A

11	BICF2G630303991	62854009	A	0	0	G
11	BICF2G630304005	62859028	A	0.25	0.3571	G
11	BICF2G630304017	62873651	G	0	0	A
11	BICF2G630304027	62885469	G	0.25	0.3571	A
11	TIGRP2P150401_rs9227835	62887203	A	0	0	G
11	BICF2G630304031	62895516	A	0	0	G
11	BICF2G630304048	62909620	A	0.75	0.6429	G
11	BICF2P1416533	62916961	G	0.75	0.6429	A
11	BICF2G630304062	62938445	A	1	1	G
11	BICF2G630304063	62940639	C	1	1	A
11	BICF2P619824	62951256	0	0	0	G
11	BICF2P1043792	62968971	A	0.75	0.6667	G
11	BICF2P799466	62976214	A	0	0	G
11	BICF2G630304074	62994823	A	0	0	G
11	BICF2G630304088	62999311	A	0	0	G
11	BICF2G630304102	63008509	A	0	0	G
11	BICF2G630304127	63021269	A	0	0	G
11	BICF2P77022	63038471	G	1	1	A
11	TIGRP2P150456_rs8510809	63051157	A	0.75	0.6429	G
11	BICF2G630304149	63055040	A	1	1	G
11	BICF2P987945	63073086	T	0.75	0.6429	A
11	BICF2P1154795	63086456	A	0	0	G
11	BICF2G630304165	63090563	G	0	0	A
11	BICF2G630304188	63100645	A	0.75	0.6429	G
11	BICF2G630304205	63112385	C	0.25	0.3571	A
11	BICF2G630304237	63134839	G	0	0	A
11	BICF2G630304238	63135608	G	0	0	A
11	BICF2G630304255	63156122	G	0	0	A
11	BICF2G630304259	63161165	A	0	0	G
11	BICF2G630304268	63171840	C	0	0	A
11	BICF2G630304278	63178674	C	0	0	A
11	BICF2S23224640	63187316	A	0	0	G
11	BICF2P601071	63203031	A	0.25	0.1429	G
11	BICF2G630304307	63208720	A	0	0	G
11	BICF2G630304315	63222249	A	0	0	G
11	BICF2G630304317	63222505	G	0	0	A
11	TIGRP2P150512_rs9227757	63227649	A	0.5	0.5	G
11	BICF2G630304342	63239337	A	0.25	0.1429	G
11	BICF2G630304371	63254350	A	0	0	G
11	BICF2G630304392	63267171	G	0.75	0.8571	A
11	BICF2G630304397	63281032	G	0.75	0.6429	A
11	BICF2S22915349	63291827	A	0	0	G
11	BICF2G630304414	63296041	A	0.25	0.1429	G
11	BICF2G630304427	63308804	A	0.75	0.9167	G
11	BICF2G630304461	63324822	A	0	0	G
11	BICF2G630304473	63330766	G	0.5	0.5	A
11	BICF2G630304485	63342964	A	0.5	0.5	C

11	BICF2G630304493	63353813	A	0.75	0.8571	G
11	BICF2G630304514	63370571	G	0.25	0.3571	A
11	BICF2P71332	63386092	A	0.25	0.1429	G
11	BICF2P655275	63398977	G	0	0	A
11	BICF2G630304531	63408987	A	0.25	0.3571	G
11	BICF2G630304545	63412631	C	1	0.8571	A
11	BICF2P1296749	63428655	G	0.5	0.5	A
11	BICF2G630304552	63439672	A	0.5	0.5	G
11	BICF2G630304569	63452171	A	0.5	0.5	G
11	BICF2G630304582	63457082	A	0.5	0.5	C
11	BICF2P1464203	63476736	G	0.5	0.5	A
11	BICF2G630304616	63484861	G	0.5	0.5	A
11	BICF2G630304617	63485768	G	0.5	0.5	A
11	BICF2P75649	63494609	G	0.5	0.5	A
11	TIGRP2P150555_rs8911094	63505424	C	0	0	G
11	BICF2P653552	63523750	A	0.5	0.5	G
11	BICF2G630304628	63526738	A	0	0	C
11	BICF2G630304641	63538599	A	0	0	G
11	TIGRP2P150583_rs8556503	63555199	G	0.5	0.5	A
11	BICF2S23327909	63564611	G	0.25	0.1429	A
11	BICF2G630304689	63565539	A	0.75	0.6429	C
11	BICF2P608918	63572018	G	0.5	0.5	A
11	BICF2G630304729	63592214	G	0.75	0.6429	A
11	BICF2P756174	63595221	0	0	0	G
11	BICF2S23521071	63609277	G	0	0	A
11	BICF2P792561	63636896	A	0.5	0.5	G
11	BICF2G630304834	63651511	A	0.5	0.5	G
11	BICF2G630304877	63673388	C	1	0.4286	A
11	BICF2P638133	63677888	G	0.75	0.4286	A
11	BICF2G630304893	63688285	0	0	0	A
11	BICF2G630304897	63701667	A	0.75	0.6429	G
11	BICF2S23253534	63717585	A	0	0.2143	G
11	BICF2S23253533	63717683	G	0.75	0.5	A
11	BICF2G630304899	63723961	G	0	0	A
11	BICF2G630304924	63733812	A	0	0	G
11	TIGRP2P150662_rs8683998	63748183	A	0	0.2143	G
11	BICF2S2299269	63758308	A	0	0.1667	C
11	TIGRP2P150664_rs8686128	63767673	G	0.75	0.5	A
11	BICF2P6990	63779917	C	0	0	G
11	BICF2P649773	63792519	A	0	0	C
11	TIGRP2P150674_rs8975862	63808511	G	0	0	A
11	BICF2G630304967	63825355	A	0	0	C
11	BICF2P1016233	63826992	G	0	0.2143	A
11	BICF2P534215	63829330	A	0	0	G
11	BICF2P1224560	63842271	A	0	0	G
11	BICF2G630305022	63842404	G	0.75	0.4286	A
11	BICF2G630305042	63849019	A	0.75	0.4286	G

11	BICF2G630305061	63864170	A	0.75	0.6429	C
11	BICF2S23634862	63875357	A	0	0	G
11	BICF2P255340	63881525	A	0	0	G
11	BICF2P630411	63884676	G	0	0.2143	A
11	BICF2G630305069	63899785	A	0	0	G
11	BICF2G630305082	63906858	A	0	0	G
11	BICF2G630305097	63921524	A	0.75	0.6429	G
11	BICF2P1154484	63935340	0	0	0	G
11	BICF2G630305111	63945866	G	0.25	0.5714	A
11	BICF2P596978	63953391	G	0.25	0.5714	A
11	BICF2G630305152	63974213	G	0	0.2143	A
11	BICF2G630305154	63975612	G	0	0	A
11	BICF2G630305177	63996430	G	0	0.2143	A
11	BICF2G630305189	64004944	A	0	0.2143	G
11	BICF2G630305195	64015538	G	0	0.2143	A
11	BICF2G630305202	64028058	G	0	0	C
11	BICF2G630305206	64033514	G	0	0.2143	A
11	BICF2P347885	64051691	A	0	0	C
11	BICF2P1335387	64059133	G	0	0.2143	A
11	BICF2P59926	64066066	C	0	0	A
11	BICF2P929914	64070376	G	0	0.2143	C
11	BICF2P1216468	64083959	A	0	0	G
11	BICF2P1258852	64093109	0	0	0	G
11	BICF2P859274	64107784	G	0.25	0.3571	A
11	BICF2P529447	64118510	A	1	0.7857	G
11	BICF2P880805	64125511	G	0.75	0.4286	A
11	BICF2P668575	64142980	C	0.25	0.5714	A
11	BICF2G630305215	64152345	A	1	1	G
11	BICF2G630305223	64165909	C	0.75	0.4286	G
11	BICF2G630305231	64176755	A	1	0.7857	C
11	BICF2G630305244	64181892	A	0	0.2143	G
11	BICF2G630305264	64197376	A	0.75	0.4286	C
11	TIGRP2P150740_rs8774481	64204807	A	0.25	0.3571	G
11	BICF2G630305298	64218692	G	0	0.2143	A
11	BICF2G630305332	64233902	G	1	0.7857	A
11	BICF2S22915645	64246382	A	0.75	0.4286	G
11	BICF2G630305353	64255913	C	0	0	A
11	BICF2G630305359	64268431	0	0	0	A
11	BICF2P1201942	64281512	A	0.75	0.4286	G
11	BICF2G630305380	64290919	A	0	0	G
11	BICF2S230802	64300683	G	0	0	A
11	BICF2G630305395	64309916	G	0	0.2143	A
11	BICF2P141370	64321624	A	0.75	0.4286	G
11	BICF2G630305429	64338710	0	0	0	G
11	BICF2G630305432	64339174	0	0	0	C
11	BICF2G630305444	64345280	G	0	0	A
11	BICF2P1069611	64364460	G	0.75	0.4286	A

11	BICF2G630305469	64365471	G	0.25	0.3571	A
11	BICF2P576198	64373695	G	0	0.25	A
11	BICF2S2363126	64373733	0	0	0	A
11	BICF2G630305485	64379055	A	0.25	0.3571	G
11	BICF2G630305503	64389063	A	0.25	0.3571	C
11	BICF2G630305527	64400331	G	0	0	A
11	TIGRP2P150772_rs9159767	64412468	A	0.25	0.3571	G
11	BICF2G630305558	64424389	G	0	0	A
11	BICF2G630305589	64438933	A	0	0	G
11	BICF2G630305613	64450597	C	0	0	A
11	BICF2G630305662	64467221	A	0.75	0.6429	G
11	BICF2G630305680	64471225	G	0	0	A
11	BICF2G630305684	64471818	0	0	0	A
11	TIGRP2P150779_rs8716667	64481776	A	0.75	0.4286	G
11	TIGRP2P150781_rs8978630	64497241	A	0.75	0.6429	G
11	BICF2G630305760	64508719	C	0.75	0.6429	A
11	BICF2P698765	64535685	A	0	0	G
11	BICF2G630305782	64538201	A	0.75	0.4286	G
11	BICF2G630305798	64560815	G	0.25	0.5714	A
11	BICF2G630305802	64567477	G	0.75	0.4286	A
11	BICF2G630305811	64576063	G	0.25	0.5714	A
11	BICF2G630305817	64584612	0	0	0	C
11	BICF2P1049359	64612701	A	1	1	G
11	BICF2G630305835	64613064	0	0	0	G
11	BICF2G630305855	64629430	A	1	0.7857	G
11	BICF2P1159772	64630901	A	0	0.2143	G
11	BICF2G630305857	64631922	A	0	0.2143	G
11	BICF2G630305890	64651122	G	1	0.7857	A
11	BICF2G630305898	64658767	A	1	1	G
11	BICF2G630305903	64664045	G	1	1	A
11	BICF2G630305915	64672286	A	0.75	0.6429	G
11	BICF2G630305936	64686565	A	0.25	0.3571	G
11	BICF2G630305949	64691766	G	0.75	0.6429	A
11	BICF2G630305955	64708870	A	0	0	G
11	BICF2G630305956	64710582	G	0	0	C
11	TIGRP2P150821_rs9087728	64721490	0	0	0	C
11	BICF2P973203	64729055	A	0	0	G
11	BICF2P544714	64734549	A	0	0	G
11	BICF2P1438249	64745955	A	0	0	C
11	BICF2G630305972	64765032	A	1	1	C
11	BICF2G630305980	64771969	0	0	0	G
11	BICF2P518386	64776544	A	0	0.2143	G
11	TIGRP2P150826_rs8902009	64779892	G	0	0	A
11	BICF2P459833	64793226	A	1	0.7857	G
11	BICF2P159715	64807090	A	0.75	0.4286	G
11	BICF2G630306006	64824549	A	0.75	0.6429	G
11	BICF2G630306009	64826159	G	0	0.2143	A

11	TIGRP2P150867_rs9214892	64845580	A	0	0.2143	T
11	TIGRP2P150869_rs9026029	64848668	C	0.75	0.4286	A
11	BICF2S2378804	64860512	A	0	0.2143	G
11	BICF2G630306025	64875802	G	0	0	A
11	BICF2G630306036	64881408	G	0	0	A
11	BICF2P1008523	64900291	A	0	0	G
11	BICF2G630306066	64917395	A	0.75	0.6429	G
11	BICF2G630306070	64918862	A	0	0.2143	C
11	BICF2G630306082	64929231	A	0.75	0.6429	G
11	BICF2P1430918	64946239	0	0	0	C
11	BICF2S23162394	64974337	A	0.25	0.3571	G
11	BICF2G630306098	64979427	C	0.25	0.3571	A
11	BICF2G630306108	64990802	G	0.25	0.3571	A
11	BICF2G630306127	64998179	A	1	1	G
11	BICF2G630306160	65012483	G	0.25	0.3571	A
11	BICF2G630306179	65033949	A	0.25	0.3571	G
11	BICF2G630306188	65052218	A	0	0	G
11	BICF2P164696	65062705	A	0	0	G
11	BICF2G630306203	65074142	A	0.75	0.6429	G
11	BICF2G630306217	65079897	A	0.75	0.6429	G
11	TIGRP2P150914_rs8720892	65110218	G	0.75	0.6429	A
11	BICF2P462179	65117162	0	0	0	G
11	BICF2P407128	65129081	G	0	0	A
11	BICF2G630306223	65137013	C	0	0	A
11	BICF2G630306228	65156811	G	0.75	0.4286	A
11	BICF2G630306243	65164732	A	0.25	0.5714	G
11	BICF2S23357159	65178089	G	0.75	0.6429	A
11	BICF2G630306265	65185124	A	0.25	0.5714	G
11	BICF2P1260288	65196123	A	0	0	G
11	BICF2G630306294	65212614	A	0.25	0.5714	G
11	BICF2G630306296	65225542	A	0.25	0.3571	G
11	BICF2G630306307	65230058	A	0	0	G
11	BICF2G630306330	65240746	A	0.25	0.3571	G
11	BICF2G630306343	65251764	A	0	0	G
11	BICF2P847123	65262559	G	0.75	0.4286	A
11	TIGRP2P150962_rs8714153	65284211	G	0	0	A
11	BICF2G630306353	65290598	A	0	0	G
11	BICF2G630306359	65298007	G	0.75	0.4286	A
11	BICF2G630306371	65317800	G	1	0.7857	C
11	BICF2G630306372	65325393	A	0	0	G
11	TIGRP2P150975_rs9223243	65335653	G	0	0.2143	A
11	BICF2S2378518	65347020	G	0	0	A
11	BICF2G630306390	65355002	G	0	0	A
11	BICF2G630306422	65377171	G	1	0.7857	A
11	TIGRP2P150982_rs8612765	65380491	A	0	0	G
11	BICF2G630306442	65396407	G	1	1	A
11	BICF2G630306446	65402148	G	0	0	A

11	BICF2G630306470	65412782	G	0	0	A
11	BICF2G630306489	65424668	G	0	0	A
11	BICF2S23250186	65445274	G	0	0.2143	A
11	BICF2G630306495	65454554	C	0	0.2143	A
11	BICF2P1056047	65456099	A	0	0	G
11	BICF2P1056046	65456117	G	0.75	0.6429	A
11	BICF2P732289	65458427	0	0	0	A
11	BICF2S23653829	65475303	A	0	0	C
11	BICF2G630306507	65481545	C	0.75	0.6429	A
11	BICF2G630306520	65499634	T	0.25	0.3571	A
11	BICF2G630306531	65510202	0	0	0	G
11	BICF2P816294	65512556	0	0	0	G
11	BICF2P1203509	65526683	C	0.25	0.3571	A
11	TIGRP2P151012_rs8670936	65527231	G	0.25	0.3571	A
11	BICF2G630306538	65540569	A	0.25	0.3571	G
11	BICF2P1221256	65559504	A	0	0	G
11	BICF2P1111641	65566861	A	0.5	0.5	G
11	BICF2G630306627	65583033	A	0.25	0.3571	G
11	BICF2G630306634	65586288	A	0.25	0.3571	G
11	BICF2G630306650	65597370	A	0	0	G
11	TIGRP2P151026_rs8833944	65597448	C	0.5	0.5	A
11	BICF2S2294641	65614664	0	0	0	A
11	BICF2S23417740	65619773	A	0.75	0.8571	C
11	BICF2G630306660	65637779	A	0	0	G
11	BICF2P355410	65644741	A	0	0	G
11	TIGRP2P151035_rs9108334	65644798	A	0	0	G
11	BICF2P382666	65658943	A	0	0	G
11	BICF2P267385	65672014	A	0	0	G
11	BICF2P271156	65683492	G	0	0	A
11	BICF2P135938	65688711	A	1	1	C
11	BICF2P560930	65692717	A	1	1	G
11	BICF2P291597	65704333	G	0	0	A
11	BICF2S23430976	65715184	C	0.25	0.1429	G
11	BICF2P910107	65728646	A	0	0	G
11	BICF2P685856	65735727	A	0	0	G
11	BICF2S2451655	65779216	A	0	0	G
11	BICF2P17417	65781725	0	0	0	A
11	BICF2P1053653	65807187	A	0.25	0.1429	G
11	BICF2P1255487	65823803	A	0.25	0.1429	G
11	BICF2P1213067	65831544	A	0.75	0.8571	G
11	BICF2P366242	65842935	A	0	0	G
11	BICF2P497190	65852664	A	1	1	G
11	BICF2P164671	65867230	C	0	0	A
11	BICF2P778401	65882762	A	0	0	G
11	BICF2P1133252	65893175	G	0	0	A
11	BICF2P446224	65899466	G	0.25	0.1429	A
11	BICF2P1049071	65902838	G	0	0	C

11	BICF2P356029	65917384	A	1	1	G
11	BICF2P1049115	65921304	C	1	1	A
11	TIGRP2P151124_rs9136957	65937570	A	0	0	T
11	BICF2P725134	65943073	G	0	0	C
11	BICF2P668672	65953401	A	0.25	0.1429	G
11	BICF2P285924	65983015	G	0.25	0.1429	A
11	BICF2P942478	65994569	A	0.25	0.1429	G
11	BICF2P1431742	66006607	A	0.25	0.1429	G
11	BICF2S22950959	66015056	A	0.25	0.1429	G
11	BICF2S2376155	66022829	A	0.25	0.1429	G
11	BICF2P628650	66041556	A	0	0.1429	C
11	BICF2G630306661	66060255	0	0	0	G
11	TIGRP2P151144_rs8842762	66088763	G	0.75	0.8571	A
11	BICF2P901631	66090771	C	0.5	0.2857	A
11	BICF2P1332321	66102349	G	0.75	0.4286	A
11	BICF2P295006	66127005	A	0.5	0.2857	G
11	TIGRP2P151180_rs8780724	66142816	A	0.25	0.1429	G
11	BICF2P134150	66146590	G	0.25	0.1429	A
11	TIGRP2P151184_rs9042196	66151479	G	0.75	0.4286	A
11	TIGRP2P151191_rs9148372	66159839	T	0.5	0.2857	A
11	BICF2S23661156	66177173	G	0	0	A
11	TIGRP2P151198_rs9002591	66187010	T	0	0	A
11	TIGRP2P151202_rs9106116	66204542	A	0	0	G
11	BICF2P548090	66211421	A	0	0	G
11	TIGRP2P151206_rs8639023	66217098	0	0	0	C
11	BICF2P159450	66231342	A	0.25	0.5714	G
11	TIGRP2P151214_rs9111395	66248964	A	0.5	0.2857	C
11	BICF2S23514492	66250178	G	0.5	0.2857	A
11	TIGRP2P151217_rs8854872	66255756	A	0	0	G
11	BICF2P608709	66265223	C	0	0	G
11	BICF2P606216	66276642	A	0	0.6	G
11	TIGRP2P151225_rs8915449	66287644	G	0	0	A
11	BICF2P582842	66297489	C	0.25	0.1429	A
11	BICF2P511058	66298939	A	0.25	0.1429	G
11	TIGRP2P151259_rs8759812	66309405	G	0	0	A
11	TIGRP2P151265_rs8880592	66321829	C	0.5	0.2857	G
11	BICF2P1248030	66332944	G	0.5	0.2857	A
11	BICF2P313034	66354291	G	0	0	A
11	BICF2P921745	66360773	A	0	0	G
11	BICF2S23714179	66374340	A	NA	1	G
11	BICF2P467055	66385640	A	0.75	0.8571	C
11	TIGRP2P151289_rs8938002	66394413	A	0	0	C
11	TIGRP2P151292_rs8961371	66406622	C	0.25	0.1429	A
11	BICF2P650191	66416062	A	0.75	0.8571	G
11	TIGRP2P151298_rs9078019	66431526	T	0.25	0.1429	A
11	BICF2P231400	66434185	G	0.25	0.1429	A
11	TIGRP2P151300_rs9151522	66445709	C	0.5	0.2857	A

11	BICF2P59033	66453044	G	0.5	0.2857	A
11	BICF2P213097	66458564	G	0.5	0.2857	A
11	BICF2S23147047	66473482	A	0.5	0.2857	G
11	BICF2S2369607	66485142	G	0.5	0.2857	A
11	TIGRP2P151338_rs9118242	66494827	A	0	0	T
11	BICF2P1218225	66520052	0	0	0	T
11	BICF2S23612357	66534100	0	0	0	G
11	BICF2P698063	66549462	0	0	0	A
11	BICF2P101748	66558964	A	0	0	G
11	BICF2S23550329	66568114	A	0	0	G
11	BICF2S23026015	66580750	G	0.5	0.2857	A
11	BICF2P1323621	66592767	C	0	0	A
11	BICF2P39301	66604869	C	0.5	0.7143	G
11	BICF2P39300	66605111	T	0.5	0.2857	A
11	BICF2S23627420	66615788	A	0	0	G
11	BICF2P1264244	66622108	A	0.5	0.2857	C
11	BICF2P1264256	66623554	A	0.5	0.2857	G
11	BICF2P356517	66638206	C	0.5	0.2857	A
11	BICF2P736646	66644674	A	0.5	0.25	G
11	BICF2S22949030	66664213	A	0	0	C
11	BICF2P358059	66666314	G	0.5	0.2857	A
11	BICF2P1191148	66678357	A	0.5	0.2857	G
11	BICF2S23640675	66696449	A	0.5	0.2857	G
11	TIGRP2P151405_rs8645467	66700742	A	0.5	0.2857	G
11	BICF2P1266743	66718636	A	0	0	C
11	BICF2P430991	66733117	0	0	0	A
11	BICF2P1086291	66736486	A	0	0	G
11	BICF2P676830	66752855	0	0	0	G
11	BICF2S23651430	66769689	G	0	0	A
11	BICF2P775461	66787435	G	0	0	A
11	BICF2P1009348	66796900	G	0	0	A
11	TIGRP2P151440_rs8807113	66808976	A	0.75	0.4286	G
11	BICF2P526164	66815565	0	0	0	G
11	BICF2P1063109	66834499	A	0	0	G
11	TIGRP2P151450_rs8903640	66841755	A	0	0	G
11	BICF2P1504956	66857506	G	0	0	A
11	TIGRP2P151461_rs8946304	66863044	C	0.25	0.1429	A
11	BICF2P1214828	66875923	A	0.25	0.08333	C
11	BICF2P609876	66885201	A	0.25	0.1429	G
11	BICF2S23613350	66898813	G	0.25	0.1429	A
11	TIGRP2P151468_rs9161785	66902955	A	0.25	0.1429	G
11	TIGRP2P151475_rs9045442	66912771	A	0.25	0.1429	C
11	BICF2P455975	66919057	G	0.25	0.1429	A
11	BICF2S23542922	66936666	A	0.75	0.8571	G
11	TIGRP2P151499_rs9123243	66948444	C	0.5	0.2857	G
11	BICF2P1090138	66957718	A	0.25	0.1429	G
11	BICF2P1090139	66957769	C	0.25	0.5714	A

11	BICF2S22963580	66970903	A	0	0	G
11	BICF2P985486	66982047	T	0.5	0.2857	A
11	BICF2P1307269	66997214	A	0	0.5714	T
11	BICF2P696089	67000941	T	0.25	0.5714	A
11	BICF2P524010	67015486	A	0.25	0.1429	G
11	BICF2P392168	67022883	G	0.25	0.1429	A
11	TIGRP2P151526_rs8619688	67029549	A	0	0	G
11	BICF2P794654	67036809	A	0	0	G
11	BICF2P484274	67046029	A	0	0	G
11	BICF2P374956	67064442	G	0	0	A
11	BICF2P602679	67076729	G	0	0	A
11	BICF2P607387	67086948	0	0	0	G
11	BICF2P607383	67087356	A	0	0	G
11	BICF2P230759	67092370	C	0	0	A
11	BICF2P220175	67097291	G	0	0	C
11	BICF2P642936	67105515	A	0	0	G
11	BICF2P65732	67115647	A	0	0	G
11	BICF2P645847	67132529	0	0	0	A
11	BICF2S24116327	67146903	C	0	0	A
11	BICF2P1458243	67158068	0	0	0	C
11	BICF2P750958	67183373	C	0.25	0.5714	A
11	TIGRP2P151568_rs9219501	67195951	A	0.25	0.5714	G
11	BICF2P856107	67209640	A	0.25	0.5714	G
11	BICF2P249476	67223002	0	0	0	G
11	TIGRP2P151578_rs8771960	67232254	A	0.25	0.5714	C
11	BICF2P1141680	67240592	A	0.25	0.5714	G
11	BICF2P1008050	67255223	C	0.25	0.5714	A
11	BICF2P1308827	67266490	C	0.25	0.5714	A
11	BICF2P301587	67292655	A	0.75	0.8571	G
11	BICF2P6230	67305494	A	0.5	0.2857	G
11	BICF2P1502717	67310263	A	0.5	0.2857	G
11	BICF2P453915	67321131	A	0	0	G
11	BICF2P601294	67337742	0	0	0	G
11	TIGRP2P151608_rs8872526	67345453	A	0	0	G
11	BICF2P813350	67351706	A	0	0	G
11	TIGRP2P151618_rs8585865	67366395	A	0	0	G
11	TIGRP2P151621_rs9064907	67374587	A	0	0	G
11	BICF2P686580	67379717	A	0	0	G
11	BICF2S23322467	67401073	A	0.75	0.4286	G
11	BICF2S2306377	67401892	C	0.75	0.4286	G
11	BICF2P1159069	67404663	A	0.75	0.4167	G
11	BICF2S23136783	67418353	A	0	0	G
11	BICF2P71382	67428387	G	0.25	0.5714	A
11	BICF2P518199	67436900	A	0.25	0.5714	G
11	BICF2P453543	67448798	G	0	0	A
11	BICF2S23764623	67460408	A	0	0	G
11	BICF2P142714	67470819	G	0.75	0.4286	A

11	BICF2P171344	67511671	G	0	0	A
11	BICF2P171345	67511723	A	0	0	T
11	BICF2P426964	67523073	A	0	0	G
11	BICF2S23550990	67532832	G	0	0	A
11	BICF2S23633907	67539780	A	0	0	G
11	BICF2P1220131	67563313	0	0	0	A
11	BICF2P730894	67589135	A	0.25	0.5714	G
11	BICF2P678136	67598727	A	0	0	G
11	BICF2P619935	67613316	A	0	0	G
11	BICF2S23215860	67628984	A	0	0	C
11	BICF2P1027980	67639812	A	0.5	0.2857	T
11	BICF2P689703	67657770	A	0.5	0.2857	G
11	BICF2S23215504	67668754	C	0.5	0.7143	G
11	BICF2P1245828	67693464	A	0	0	G
11	BICF2P1208757	67695323	A	0	0	G
11	BICF2P935272	67705667	G	0	0	A
11	BICF2P57881	67713410	A	0	0	G
11	BICF2P364555	67724228	G	0.25	0.1429	A
11	TIGRP2P151727_rs8878126	67736019	G	0.75	0.4286	A
11	BICF2P928014	67749458	A	0	0	G
11	BICF2P945830	67767019	C	0	0	G
11	BICF2P307995	67777001	G	0.75	0.4286	A
11	BICF2P1132749	67787626	G	0.75	0.4286	A
11	BICF2P159316	67797360	G	0	0	A
11	TIGRP2P151757_rs9240270	67805656	G	0.75	0.4286	A
11	BICF2P747593	67823674	A	0	0	G
11	BICF2P905900	67834789	A	0	0	C
11	BICF2P638293	67846177	C	0	0	A
11	BICF2S23153200	67848069	A	0	0	T
11	BICF2P1034448	67853048	T	0	0	A
11	BICF2P890187	67865078	G	0	0	A
11	BICF2P230975	67873386	G	0.5	0.2857	A
11	BICF2P1423470	67887265	A	0.5	0.7143	G
11	BICF2P543366	67906300	A	0.25	0.5714	G
11	BICF2P583439	67908341	A	0	0	G
11	BICF2P1410507	67920857	A	0	0	G
11	BICF2P641102	67932473	C	0	0	A
11	BICF2P615869	67950854	A	0.25	0.1429	G
11	TIGRP2P151808_rs8865846	67957614	A	0.75	0.4286	C
11	BICF2P672512	67968129	G	0.25	0.5714	A
11	TIGRP2P151818_rs9152799	67981214	C	0.25	0.1429	A
11	BICF2P1067772	67998124	A	0	0	G
11	TIGRP2P151830_rs9012235	68003809	A	0.25	0.1429	T
11	TIGRP2P151834_rs9081885	68010400	A	0	0	C
11	BICF2S23219954	68011825	A	0	0.5714	G
11	BICF2P978132	68019119	A	0	0	G
11	TIGRP2P151840_rs8674188	68029045	A	1	1	G

11	BICF2S23527869	68038058	A	0.5	0.2857	G
11	BICF2P913835	68048441	A	0.25	0.5714	G
11	BICF2P913834	68048458	C	0	0	G
11	BICF2P982854	68059913	G	0.75	0.4286	A
11	BICF2P646805	68069292	0	0	0	A
11	BICF2P1191963	68083541	A	0.25	0.1429	G
11	BICF2S23546211	68100184	A	0.75	0.8571	G
11	TIGRP2P151863_rs9037870	68102478	A	0.75	0.8571	G
11	BICF2P955753	68110523	0	0	0	G
11	TIGRP2P151864_rs8825084	68121455	0	0	0	A
11	BICF2P1389530	68136288	0	0	0	G
11	BICF2P405982	68140207	G	0.25	0.5714	A
11	BICF2P216307	68167587	A	0.5	0.2857	G
11	BICF2P545730	68179082	A	0.75	0.8571	G
11	BICF2S2301819	68186010	A	0.25	0.5714	C
11	BICF2P1180331	68197498	A	0.25	0.5714	G
11	BICF2P1244574	68202701	A	0.25	0.5714	C
11	TIGRP2P151895_rs8699556	68203625	A	0	0	G
11	BICF2P702867	68211418	A	0.5	0.2857	T
11	BICF2P703561	68212569	A	0.5	0.2857	G
11	BICF2P479048	68218701	A	0.5	0.7143	G
11	TIGRP2P151911_rs8547412	68234713	G	0	0	A
11	TIGRP2P151912_rs8769132	68257151	0	NA	0	A
11	BICF2P816697	68272829	G	0	0	A
11	BICF2P458131	68278967	A	0	0	G
11	BICF2P668629	68294720	A	0	0	G
11	BICF2P290025	68308397	0	0	0	A
11	BICF2P1185732	68321219	0	0	0	G
11	TIGRP2P151922_rs8796080	68323083	G	0.25	0.5833	A
11	BICF2S23451102	68334688	A	0	0	C
11	TIGRP2P151930_rs8935371	68342632	G	0.75	0.4286	A
11	BICF2S23417202	68348593	G	0	0	A
11	BICF2P658454	68378899	A	0	0	G
11	TIGRP2P151947_rs9092882	68386797	A	0	0	G
11	BICF2P1084948	68390843	G	0	0	A
11	BICF2P672133	68410365	0	0	0	A
11	BICF2P965663	68417490	G	0.75	0.4286	A
11	TIGRP2P151959_rs8746399	68430619	G	0.75	0.4286	A
11	BICF2P298491	68447291	C	0	0	A
11	BICF2P769101	68453579	G	0	0	A
11	TIGRP2P151967_rs9197170	68464811	0	0	0	A
11	BICF2P908679	68474017	G	0	0	A
11	BICF2P482826	68493923	C	1	1	G
11	BICF2P381414	68496037	A	0.75	0.4286	G
11	BICF2P812539	68508022	A	0.75	0.4286	G
11	TIGRP2P151983_rs9205904	68528184	G	0	0	A
11	BICF2P82123	68529280	A	0.75	0.4286	G

11	BICF2P339079	68540682	A	0.75	0.4286	G
11	BICF2P898899	68560580	A	0	0	G
11	BICF2P238727	68567564	A	0	0	G
11	BICF2P914364	68577663	C	0.5	0.2857	A
11	BICF2P393090	68590810	A	0	0	G
11	TIGRP2P152008_rs8546293	68602205	G	1	1	A
11	BICF2P1020504	68611984	A	0.25	0.1429	G
11	BICF2P433053	68621040	C	0.75	0.8571	A
11	BICF2P1371147	68634324	C	0.75	0.8571	G
11	BICF2P551426	68646699	A	0.75	0.8571	G
11	BICF2P1044993	68662067	G	0.5	0.2857	A
11	BICF2P225138	68668694	0	0	0	C
11	BICF2P455671	68678100	A	0	0	G
11	BICF2P262922	68711012	G	0	0	A
11	BICF2P735583	68719993	A	0.5	0.2857	G
11	TIGRP2P152024_rs8906228	68729137	A	0.5	0.7143	C
11	BICF2P1206877	68739480	0	0	0	A
11	BICF2S23052549	68755540	C	0.5	0.2857	G
11	TIGRP2P152041_rs9164713	68764856	A	0.25	0.5714	G
11	BICF2P997143	68772909	A	0.5	0.2857	G
11	BICF2P551732	68783296	A	0	0	G
11	BICF2P495340	68792463	A	0	0	G
11	BICF2P739187	68794837	G	0.25	0.5714	A
11	BICF2P977078	68809917	0	0	0	A
11	BICF2P1503169	68821125	G	0.75	0.8571	A
11	BICF2P1178200	68827677	G	0	0	A
11	BICF2P22279	68845092	G	0	0	A
11	BICF2P789889	68857154	A	0	0	G
11	BICF2P825137	68869732	A	0	0	G
11	BICF2P187394	68876404	A	0.75	0.8333	G
11	BICF2S23018767	68891921	A	0	0	G
11	BICF2P422105	68900294	C	0.25	0.1429	A
11	BICF2P456208	68908950	A	0.25	0.1429	G
11	BICF2P769671	68927240	A	0.5	0.2857	G
11	BICF2P86563	68931781	A	0	0	G
11	TIGRP2P152111_rs8649116	68947319	0	0	0	G
11	TIGRP2P152114_rs9116675	68956769	A	0.5	0.2857	G
11	BICF2P46315	68965740	A	0.5	0.2857	G
11	BICF2P648141	68986058	G	0	0	A
11	BICF2P332921	68996365	A	0.25	0.1429	G
11	BICF2P1164922	69006417	A	0.75	0.4286	G
11	BICF2S23112069	69020607	C	0	0	A
11	BICF2P644955	69024395	G	0.5	0.7143	A
11	BICF2P591919	69041120	0	0	0	G
11	BICF2P785198	69049353	G	0.5	0.7143	A
11	BICF2S233972	69063845	A	0.5	0.7143	G
11	TIGRP2P152148_rs8943064	69080348	A	0	0	G

11	BICF2P102529	69101460	A	0.25	0.1429	C
11	BICF2P1470720	69115094	A	0.25	0.5714	C
11	BICF2P1127381	69133359	G	0	0	A
11	BICF2S23553117	69143819	G	0.5	0.2857	A
11	BICF2P789189	69158038	G	0.25	0.5714	A
11	BICF2P176253	69162210	T	0.25	0.1429	A
11	BICF2S23248959	69178554	G	0.5	0.7143	A
11	TIGRP2P152154_rs9185072	69190078	G	0.25	0.1429	A
11	BICF2S23130480	69199517	C	0.75	0.4286	A
11	BICF2P314662	69208240	A	0	0	G
11	BICF2P852480	69220043	C	0	0	A
11	BICF2S23315293	69234566	G	0.25	0.5714	A
11	TIGRP2P152157_rs8838781	69243260	A	0.75	0.4286	G
11	TIGRP2P152161_rs9158250	69254198	A	0.75	0.4286	G
11	BICF2S232633	69267129	0	0	0	C
11	BICF2S23715971	69281719	A	0	0	C
11	BICF2S2295359	69291133	A	0	0	G
11	BICF2S2298815	69310574	A	0.5	0.2857	G
11	BICF2P177178	69334949	A	0	0	G
11	BICF2S23655353	69345277	A	0.5	0.2857	G
11	BICF2P429532	69366141	0	0	0	G
11	BICF2P1356658	69368931	A	0	0	G
11	TIGRP2P152172_rs8926356	69399345	A	0	0	G
11	BICF2P1317888	69422681	G	1	1	A
11	BICF2P104981	69430544	A	0	0	T
11	BICF2S23637805	69445240	0	0	0	A
11	BICF2P694106	69460832	G	0	0	C
11	BICF2S23040831	69480428	T	0.25	0.1429	A
11	TIGRP2P152190_rs9248757	69483649	A	0.25	0.1429	G
11	TIGRP2P152203_rs9147233	69501200	G	0.25	0.1429	A
11	TIGRP2P152208_rs8501565	69518881	A	0.75	0.4286	G
11	BICF2S23023483	69538636	A	1	1	G
11	BICF2S23742772	69544422	A	0	0	G
11	TIGRP2P152212_rs9108430	69552138	0	0	0	C
11	BICF2P969119	69569670	A	0.25	0.5	G
11	BICF2P617861	69575728	0	0	0	C
11	TIGRP2P152219_rs8562028	69587963	0	0	0	G
11	BICF2P963447	69603661	0	0	0	A
11	BICF2G630306667	69621954	G	0.75	0.8571	A
11	BICF2G630306679	69643458	A	0.25	0.1429	G
11	BICF2G630306682	69647830	G	0	0	A
11	BICF2S23247472	69677124	A	0.25	0.1429	G
11	BICF2P290073	69679692	G	0.75	0.5	A
11	BICF2P290887	69685474	G	0.75	0.5	A
11	BICF2G630306730	69701205	0	0	0	G
11	BICF2G630306745	69712146	G	0.75	0.5	A
11	BICF2G630306750	69714435	A	0.25	0.5	G

11	BICF2G630306751	69724967	A	NA	0	G
11	BICF2P390207	69737803	A	0	0	G
11	BICF2P1178914	69753641	G	0	0	A
11	BICF2G630306787	69762993	A	0.5	0.3571	G
11	BICF2P676003	69775055	C	0.5	0.3571	A
11	BICF2G630306824	69804919	A	0.5	0.3571	G
11	BICF2G630306825	69807186	G	0.5	0.3571	A
11	BICF2G630306829	69822644	G	0.5	0.3571	A
11	BICF2G630306834	69833410	C	0.5	0.3571	A
11	BICF2G630306841	69844027	A	0.5	0.3571	G
11	BICF2P244460	69861386	G	0	0	A
11	TIGRP2P152270_rs9175629	69877426	A	0.5	0.3571	G
11	BICF2P861555	69886109	C	0	0	A
11	TIGRP2P152285_rs8721801	69922826	A	0.25	0.2143	G
11	BICF2P70321	69937223	G	0.25	0.2143	C
11	BICF2P333944	69953253	C	0.25	0.2143	A
11	BICF2P83331	69956855	A	0.75	0.7857	G
11	TIGRP2P152311_rs8932181	69975843	C	0.25	0.2143	A
11	BICF2P950554	69985196	0	0	0	A
11	TIGRP2P152318_rs9159712	69993188	G	0.25	0.2143	A
11	BICF2P1196122	70010750	0	0	0	A
11	BICF2S23443440	70045362	G	0	0	A
11	BICF2P150532	70059163	0	0	0	G
11	BICF2P933889	70073543	A	0.75	0.7857	G
11	BICF2P413232	70091862	A	0.5	0.6429	G
11	BICF2P582760	70099804	A	0.5	0.3571	G
11	BICF2P63799	70104869	G	0.5	0.3571	A
11	TIGRP2P152338_rs8581549	70124611	A	0	0	G
11	BICF2P1320271	70127954	G	0	0	A
11	TIGRP2P152343_rs8597595	70128615	G	0	0	A
11	BICF2P1222317	70147162	G	0	0	A
11	BICF2P1239507	70153334	A	0	0	C
11	BICF2P89315	70163549	G	0	0	A
11	TIGRP2P152356_rs8808184	70173684	G	0	0	A
11	BICF2S23713898	70185413	C	0	0	A
11	BICF2P176923	70200716	A	0.5	0.3571	C
11	TIGRP2P152377_rs9076368	70218965	G	0	0	A
11	BICF2P125920	70233927	C	0.25	0.2143	A
11	BICF2S23151782	70253263	A	0	0	G
11	BICF2P1406886	70256494	A	0	0	G
11	BICF2P616706	70268434	A	0.5	0.3571	G
11	BICF2P216975	70279675	G	0.25	0.4286	A
11	BICF2P132860	70288934	A	0	0	G
11	BICF2S23342162	70304641	G	0.5	0.3571	A
11	BICF2P1022913	70319662	A	0.25	0.2143	G
11	BICF2P1022922	70322734	A	0	0	G
11	BICF2P209184	70343868	A	0.25	0.2143	C

11	BICF2P767635	70385348	A	0	0	G
11	BICF2S23728970	70401578	G	0.25	0.2143	A
11	BICF2P981089	70413324	G	0.25	0.2143	A
11	BICF2P1111923	70419090	G	0	0	A
11	BICF2P65250	70427546	G	0.25	0.2143	A
11	BICF2P734672	70438792	A	0.25	0.4286	G
11	BICF2P1225697	70460476	C	0.25	0.4286	G
11	BICF2P1225707	70462489	A	0.25	0.4286	G
11	TIGRP2P152435_rs8629120	70487655	0	0	0	G
11	BICF2P293463	70495965	A	0	0	G
11	BICF2S2447270	70503443	A	0	0	G
11	G720f22S62	70519160	0	0	0	A
11	BICF2P1162516	70549528	A	0	0	G
11	TIGRP2P152450_rs8971311	70562584	G	0.25	0.2143	A
11	BICF2P1414858	70578183	A	0.75	0.7857	G
11	BICF2P693692	70588982	A	0.75	0.7857	C
11	TIGRP2P152459_rs9079012	70609068	G	0	0	A
11	BICF2P85936	70621129	G	0	0	A
11	TIGRP2P152465_rs9201419	70623318	G	0	0	A
11	BICF2P866615	70641581	T	0	0	A
11	BICF2P474647	70650732	G	0.25	0.2143	A
11	TIGRP2P152473_rs8565826	70656971	A	0	0	G
11	BICF2P424850	70668123	G	0	0	A
11	BICF2P976394	70681118	G	0	0	A
11	BICF2P1247677	70700222	A	0.75	0.7857	G
11	BICF2S23656403	70702225	A	0.75	0.7857	G
11	TIGRP2P152492_rs9200277	70714393	A	0	0	G
11	BICF2S22939906	70726642	A	0.25	0.2143	G
11	BICF2S23161679	70758906	A	0	0	G
11	BICF2S23532960	70760338	G	0	0	A
11	BICF2P1439354	70799679	A	0.5	0.6429	G
11	BICF2P798524	70807803	A	0.25	0.2143	G
11	BICF2S22961403	70824281	A	0.25	0.2143	G
11	BICF2P1440369	70829086	A	0.5	0.6667	G
11	BICF2P1403054	70841634	G	0	0	A
11	BICF2P405890	70862378	A	0	0	G
11	TIGRP2P152528_rs8622499	70872927	A	0	0	G
11	BICF2P1121055	70883109	0	0	0	C
11	BICF2P327891	70890572	0	0	0	A
11	BICF2P85632	70907305	C	0	0	A
11	BICF2P433893	70913592	A	0	0	G
11	BICF2P471239	70942781	C	0.25	0.2143	A
11	BICF2S23538318	70950276	G	0.75	0.5714	A
11	TIGRP2P152570_rs9168295	70969609	G	0.75	0.5714	A
11	BICF2S22944567	70985695	G	1	0.5714	A
11	TIGRP2P152593_rs8812362	70991680	A	0.25	0.4286	C
11	BICF2P29017	71009460	G	0.25	0.2143	A

11	BICF2S23417809	71013919	G	1	1	A
11	BICF2P1152059	71025991	A	0	0	G
11	BICF2P1444199	71037783	G	0.25	0.2143	A
11	BICF2P759784	71047337	A	0.25	0.2143	C
11	BICF2S23344766	71068045	G	0.25	0.2143	A
11	BICF2S22924871	71074453	A	0	0	G
11	BICF2P1049438	71091329	A	1	1	G
11	BICF2P670069	71094355	A	0	0	G
11	BICF2S2368077	71108400	G	0.5	0.6429	A
11	BICF2P520176	71117514	G	0.5	0.6429	A
11	BICF2P1221112	71128848	G	0.5	0.3333	A
11	BICF2S23549389	71166278	T	0.5	0.5	A
11	BICF2S2336914	71181435	G	0.25	0.2143	A
11	BICF2P1106001	71192456	A	0.25	0.2857	G
11	TIGRP2P152666_rs8763240	71202237	A	0.5	0.5	G
11	BICF2P156702	71210103	G	0	0.1429	A
11	BICF2P420164	71213676	G	0	0.1429	A
11	BICF2S23658613	71227505	A	0	0	G
11	BICF2P401410	71260488	G	0	0	A
11	BICF2S23025283	71267856	G	0.5	0.6429	A
11	BICF2P1383108	71281673	C	0.5	0.5	G
11	BICF2P852326	71308647	A	0.25	0.2143	G
11	BICF2P1149744	71319056	C	0	0	A
11	BICF2P516167	71328179	A	0.5	0.3571	G
11	BICF2P1013392	71339307	0	0	0	C
11	BICF2S23044709	71349862	G	0.5	0.3571	A
11	BICF2P759856	71360535	0	0	0	G
11	BICF2P878630	71378025	G	0.75	0.7857	A
11	BICF2S22957696	71388156	A	0	0	C
11	BICF2P559200	71402160	C	1	0.8571	A
11	BICF2P528844	71405890	G	0	0.1429	A
11	BICF2P1369165	71426928	G	0.25	0.4286	A
11	BICF2P646953	71446660	A	0	0	G
11	BICF2P646950	71447358	A	0	0	G
11	BICF2P1041447	71452258	A	0.25	0.4286	G
11	BICF2P1362373	71464784	A	0.25	0.2857	G
11	BICF2P190296	71472607	A	0.75	0.5714	G
11	BICF2P1317801	71485985	A	0	0	G
11	BICF2P782106	71501451	A	0.25	0.4286	G
11	BICF2S23353375	71517224	A	0.75	0.5714	C
11	BICF2P482199	71524550	G	0.25	0.3571	C
11	BICF2P581766	71536142	A	0	0	G
11	BICF2P1284131	71543367	A	0	0.1429	G
11	BICF2P625812	71555966	0	0	0	C
11	BICF2P773417	71568013	A	0.25	0.3571	G
11	BICF2P592354	71577551	0	0	0	G
11	BICF2P427368	71590374	A	0	0	G

11	BICF2P1225523	71600056	A	0	0	G
11	TIGRP2P152855_rs8964945	71619756	A	0	0	G
11	BICF2P877426	71626695	C	0	0.1429	A
11	BICF2P1090889	71645119	C	0	0	A
11	BICF2P187019	71660790	C	0.25	0.2143	A
11	BICF2P480550	71676384	A	0.25	0.4286	G
11	BICF2P214429	71682108	A	0	0	G
11	BICF2S23256628	71695361	A	0.75	0.6429	G
11	BICF2S22923722	71706875	G	0.25	0.4286	A
11	BICF2P955934	71720598	0	0	0	A
11	BICF2P967357	71732636	T	0	0.1429	A
11	BICF2S23351625	71737352	A	0	0.1429	G
11	BICF2P682006	71751765	0	0	0	G
11	BICF2P999859	71765184	C	0	0	A
11	BICF2S23421088	71777952	C	1	1	A
11	BICF2S2321945	71795046	A	0.25	0.2857	G
11	BICF2S23211160	71815282	A	0.25	0.2857	T
11	BICF2S2367637	71818030	C	0.5	0.5	A
11	BICF2S23618225	71821424	A	0.25	0.3571	G
11	TIGRP2P153002_rs8702595	71833208	0	0	0	G
11	BICF2P1249136	71843346	A	0.25	0.2143	G
11	BICF2P968910	71855058	A	0	0	G
11	BICF2S23632411	71864087	A	0.25	0.2143	G
11	BICF2P268233	71896237	G	0	0	A
11	TIGRP2P153022_rs8608014	71904914	A	0	0	G
11	TIGRP2P153024_rs8885168	71913764	A	0.25	0.4286	G
11	BICF2P342417	71920751	G	0.25	0.4286	A
11	BICF2P947235	71922006	C	0.25	0.4286	A
11	BICF2P1230943	71937333	A	0	0	G
11	BICF2S23228939	71952258	A	0.75	0.6429	G
11	BICF2P857756	71958801	A	0.25	0.2143	G
11	BICF2P156051	71974638	A	0.25	0.2143	G
11	BICF2P286943	71980149	G	0.75	0.7857	A
11	TIGRP2P153063_rs8600422	71996144	0	0	0	A
11	BICF2P550138	72014353	G	0	0	A
11	BICF2S23324760	72030862	A	0.5	0.3571	G
11	TIGRP2P153086_rs8903754	72038895	0	0	0	A
11	TIGRP2P153092_rs9229977	72049036	G	0	0.1429	A
11	BICF2P321374	72059674	A	0	0.4286	G
11	BICF2P889820	72070527	A	0	0	C
11	BICF2S22921927	72091943	A	0.25	0.3571	C
11	BICF2P341803	72100367	A	0.25	0.3571	G
11	BICF2S2302305	72108416	G	0.25	0.2143	A
11	BICF2P313487	72115263	A	0.5	0.3571	G
11	BICF2P987381	72127504	G	0.25	0.4286	A
11	BICF2P196323	72134469	A	0	0	G
11	TIGRP2P153210_rs8848930	72141083	A	0.75	0.7857	G

11	BICF2G630306865	72164282	A	0	0	G
11	BICF2P894582	72180839	A	0.25	0.2857	G
11	TIGRP2P153236_rs8798086	72188966	G	0.25	0.2857	A
11	TIGRP2P153239_rs9202108	72197836	A	0.5	0.6429	G
11	BICF2S23446917	72212959	A	0	0	G
11	BICF2P539594	72227517	A	0	0	C
11	TIGRP2P153246_rs8906130	72236071	A	0.5	0.3333	G
11	BICF2P560672	72244409	A	0	0	G
11	BICF2P30914	72256650	A	0	0	G
11	BICF2P1088170	72263159	A	0	0	G
11	TIGRP2P153268_rs8514443	72267374	A	0	0	C
11	BICF2P1167407	72278361	A	0	0	G
11	BICF2P51703	72289644	C	0	0	A
11	BICF2P956000	72293400	G	0.25	0.4286	A
11	TIGRP2P153295_rs9164620	72302465	G	0	0	A
11	BICF2P1396193	72318315	A	0	0	C
11	TIGRP2P153309_rs8755961	72327662	G	0	0	A
11	BICF2P1413358	72340680	A	0	0	G
11	TIGRP2P153317_rs8632754	72352471	A	0	0.1429	G
11	BICF2P1136768	72371760	A	1	0.8571	G
11	BICF2P590188	72381093	0	0	0	A
11	BICF2S23016228	72392904	C	0	0	A
11	BICF2P514142	72407491	C	1	0.8571	G
11	BICF2P801750	72428260	A	0	0	G
11	TIGRP2P153358_rs9172165	72438807	0	0	0	A
11	BICF2S23518067	72452598	A	1	0.8571	G
11	BICF2S2304129	72471087	A	0	0	G
11	BICF2P142016	72480774	G	0.25	0.2143	A
11	BICF2P736609	72488829	G	0	0.1429	A
11	BICF2P970535	72514090	A	0.5	0.5	G
11	BICF2P1444698	72533824	G	0.5	0.3571	A
11	BICF2P1175838	72552740	A	0	0	G
11	BICF2P719729	72561873	G	0.25	0.2143	A
11	BICF2P1247543	72571358	A	0	0.2143	G
11	BICF2P73783	72579556	C	0.25	0.2143	A
11	BICF2P495533	72588965	0	0	0	G
11	BICF2P855172	72605032	G	0	0.1429	A
11	BICF2P871149	72605864	0	0	0	A
11	BICF2S23344916	72614822	G	0.5	0.2857	A
11	TIGRP2P153419_rs8588049	72627876	G	0.5	0.2857	A
11	BICF2P312771	72644118	0	0	0	A
11	TIGRP2P153429_rs8599395	72650707	A	0.75	0.5714	G
11	BICF2P423734	72666193	G	0.75	0.5714	A
11	BICF2P1335372	72668866	A	0	0	G
11	BICF2P249317	72681282	G	0.75	0.5714	A
11	TIGRP2P153440_rs9185586	72696540	A	0.75	0.5714	G
11	BICF2S22931094	72703860	G	0	0	A

11	BICF2P469598	72723108	A	0	0	G
11	BICF2P197224	72731967	C	0.75	0.5714	G
11	BICF2S23642328	72748114	G	0.5	0.5	A
11	BICF2P1291088	72752083	A	0	0	G
11	BICF2S2365774	72766409	A	0	0	G
11	BICF2S23550747	72780324	A	0.5	0.5	G
11	BICF2P864943	72787111	G	0.25	0.2857	C
11	BICF2P908442	72797055	A	0	0	G
11	BICF2P156463	72810903	G	0	0	A
11	BICF2P302327	72826126	0	0	0	A
11	BICF2S2398685	72832472	A	0	0	C
11	TIGRP2P153477_rs9054496	72844876	C	0	0.2143	A
11	BICF2S2355132	72852967	A	0.25	0.2143	G
11	BICF2P552413	72865692	G	0.5	0.5	A
11	BICF2P433582	72879058	C	0.5	0.5	A
11	BICF2S22955499	72891577	G	0	0	A
11	BICF2S23237061	72903965	A	0	0	G
11	TIGRP2P153512_rs9236093	72921490	A	0	0	G
11	BICF2P1143254	72943336	A	0	0	G
11	BICF2P276238	72945483	A	0.75	0.5714	C
11	BICF2P210691	72957735	0	0	0	G
11	BICF2P337239	73004100	0	0	0	C
11	BICF2P1342363	73018654	G	0.25	0.4286	A
11	BICF2S23357023	73030487	G	0.5	0.7143	A
11	TIGRP2P153531_rs9159611	73047517	G	0.75	0.5714	A
11	BICF2P842107	73058126	A	0.75	0.5714	C
11	TIGRP2P153551_rs8874226	73059861	G	0	0	A
11	BICF2P58150	73074272	G	0.25	0.2857	A
11	BICF2P214733	73090192	T	0	0	A
11	BICF2P779255	73097995	A	0	0	T
11	BICF2P317288	73106027	G	0	0	A
11	BICF2S23530197	73117674	G	0	0	A
11	BICF2P955386	73156395	A	0	0	G
11	BICF2P456137	73160444	A	0	0	G
11	BICF2P459464	73165556	0	0	0	G
11	BICF2P356708	73180889	A	0	0	G
11	BICF2P1274751	73188942	A	0	0	G
11	TIGRP2P153590_rs8566089	73204100	A	0.25	0.4286	G
11	BICF2P902124	73216265	A	0	0	G
11	BICF2P963063	73231284	A	0	0	G
11	BICF2P911828	73233408	G	0	0	A
11	BICF2P518909	73249670	0	0	0	A
11	BICF2P1032688	73260838	0	0	0	G
11	BICF2P1455890	73268283	G	1	0.7857	A
11	TIGRP2P153614_rs8892228	73300572	A	0.5	0.2857	G
11	BICF2P627725	73324757	G	0.75	0.5	A
11	BICF2S2451353	73341140	G	0.75	0.5	A

11	BICF2S23743965	73350755	C	0.75	0.5	A
11	BICF2P1084064	73369833	A	0.75	0.8333	G
11	BICF2P373967	73371391	A	0.5	0.2857	G
11	BICF2P651659	73394890	A	0.25	0.2143	G
11	BICF2P690908	73427344	G	0	0	A
11	BICF2P615607	73433111	A	0.5	0.2857	G
11	TIGRP2P153660_rs9068386	73447381	G	0	0	A
11	BICF2S23353756	73457377	G	0.5	0.2857	A
11	BICF2P1173832	73462161	G	0.5	0.2857	A
11	TIGRP2P153677_rs8695711	73466518	A	0.5	0.2857	G
11	BICF2P963961	73492308	0	0	0	G
11	BICF2P1061566	73497691	A	0.5	0.7143	G
11	BICF2P222114	73501917	A	0	0	G
11	TIGRP2P153712_rs8600410	73510281	G	0.25	0.5	A
11	BICF2P657251	73530520	G	0	0	A
11	BICF2S24413727	73531562	G	0.5	0.2857	A
11	BICF2P1253108	73543219	A	0.75	0.7857	G
11	BICF2G630306887	73570433	G	0	0	A
11	BICF2G630306896	73584905	0	0	0	A
11	BICF2G630306909	73591010	G	0.75	0.7857	C
11	BICF2G630306942	73609011	A	0	0	G
11	BICF2G630306962	73621493	G	0	0	A
11	BICF2G630306972	73625447	A	0.25	0.5	C
11	BICF2S2351966	73627258	C	1	1	A
11	BICF2G630307001	73638372	G	0.5	0.2857	A
11	BICF2P152084	73647126	A	0.25	0.5	C
11	BICF2G630307068	73661151	A	0.25	0.5	G
11	BICF2G630307095	73675343	A	0.25	0.5	G
11	BICF2G630307116	73686443	T	0.5	0.2857	A
11	BICF2P635181	73695223	A	0	0	G
11	BICF2G630307161	73714104	A	0	0	G
11	BICF2G630307173	73719724	C	NA	0	A
11	BICF2G630307199	73728220	C	0.5	0.2857	A
11	BICF2G630307228	73747457	A	0.5	0.7143	C
11	BICF2G630307231	73751405	A	0.5	0.2857	G
11	BICF2G630307273	73769615	G	0.5	0.7143	A
11	BICF2P693398	73772058	A	1	1	G
11	BICF2P693403	73772860	A	1	1	G
11	TIGRP2P153773_rs8799996	73790247	0	0	0	C
11	BICF2P964273	73798309	G	0	0	A
11	BICF2P902726	73807772	C	0	0	A
11	BICF2G630307319	73829868	C	0	0.2143	G
11	TIGRP2P153782_rs9091564	73837102	A	0	0.2143	G
11	BICF2P929094	73843256	G	0	0	A
11	BICF2S23036466	73866070	A	0	0	G
11	BICF2P469475	73880045	A	0	0.2143	G
11	BICF2G630307364	73886141	G	0.75	0.5714	A

11	BICF2G630307384	73894170	A	0.75	0.5714	G
11	BICF2G630307410	73902866	G	0	0.2143	A
11	BICF2G630307423	73918063	A	0	0.2143	G
11	BICF2G630307429	73932576	G	0	0	A
11	BICF2P1402695	73950502	A	0	0.2143	G
11	BICF2G630307430	73960307	C	1	0.7857	A
11	BICF2G630307459	73974228	A	0	0	G
11	BICF2G630307473	73981377	G	0.5	0.2857	A
11	BICF2G630307490	73997153	G	0.75	0.7857	A
11	BICF2G630307499	74011172	A	0	0	G
11	BICF2P271514	74014802	G	0.25	0.2143	A
11	TIGRP2P153825_rs9220227	74028283	T	0.75	0.5714	A
11	BICF2S22949291	74029868	A	0.25	0.2143	G
11	BICF2P60853	74030530	A	0	0.2143	G
11	BICF2G630307539	74038341	G	0.75	0.7857	A
11	BICF2S236655	74057155	A	0	0.2143	G
11	BICF2G630307599	74063355	G	1	0.7857	A
11	BICF2S2307595	74064546	G	0	0	A
11	BICF2P953363	74073955	A	0.75	0.7857	G
11	TIGRP2P153849_rs9157404	74083881	A	0	0	G
11	BICF2P1220826	74102636	A	0.25	0.4286	G
11	BICF2P910441	74108591	A	0	0	G
11	BICF2G630307645	74120184	C	0	0	A
11	BICF2G630307659	74137989	G	0.75	0.5714	C
11	BICF2S22942017	74140178	A	0	0	C
11	BICF2P1441939	74141207	A	0.75	0.5714	G
11	BICF2G630307678	74152475	A	0.5	0.5	C
11	BICF2P634771	74157073	G	0	0	A
11	TIGRP2P153888_rs9205702	74164330	G	0	0	A
11	BICF2P296806	74180812	G	0.25	0.4286	A
11	TIGRP2P153896_rs8574672	74190655	A	0	0	G
11	BICF2S2317580	74201157	A	0	0	G
11	BICF2G630307727	74213361	A	0	0	G
11	BICF2G630307764	74224229	A	1	1	G
11	BICF2G630307797	74236584	A	0	0	G
11	BICF2G630307811	74244699	A	0.25	0.4286	G
11	BICF2G630307853	74258966	A	0	0	G
11	BICF2G630307883	74273053	A	1	1	T
11	TIGRP2P153912_rs8552878	74280974	G	1	1	A
11	BICF2S2344488	74294747	A	0.75	0.5714	G
11	BICF2G630307884	74308058	A	0.25	0.4286	G
11	BICF2P292855	74314615	0	0	0	C
11	BICF2P798307	74332784	A	0	0	G
11	BICF2G630307911	74335015	A	1	1	G
11	BICF2P276107	74340734	A	0.75	0.7857	G
11	BICF2G630307924	74352962	C	0	0	A
11	BICF2G630307930	74355973	A	0	0	G

11	TIGRP2P153940_rs8972812	74364417	G	0	0	A
11	BICF2P1431977	74377021	A	0	0	G
11	BICF2P1431974	74377567	G	0	0	A
11	BICF2P1254122	74385009	G	1	0.7857	A
11	BICF2G630307966	74393560	G	0	0.1667	A
11	BICF2P642727	74405691	0	0	0	G
11	BICF2P643111	74408632	A	1	0.7857	G
11	BICF2S23111508	74410476	C	0	0	G
11	TIGRP2P153976_rs9046209	74423262	G	1	0.7857	A
11	BICF2S23334345	74436970	A	0.25	0.2143	T
11	BICF2S23334346	74437232	C	0.25	0.2143	A
11	BICF2G630307972	74440105	G	0.25	0.2143	A
11	BICF2P275941	74454348	A	0	0	C
11	TIGRP2P153986_rs9217231	74465644	A	0.25	0.2143	G
11	BICF2G630307993	74475570	A	0.25	0.2143	G
11	BICF2P731554	74485768	A	0.75	0.5714	G
11	TIGRP2P153995_rs8849553	74503374	0	0	0	A
11	BICF2G630308042	74515351	A	1	1	G
11	BICF2G630308065	74520132	G	0	0.2143	A
11	BICF2G630308106	74542838	A	0	0	G
11	BICF2G630308141	74559182	C	0.75	0.5714	A
11	BICF2P1327720	74567568	G	0	0	C
11	BICF2G630308175	74575227	A	0.25	0.2143	G
11	BICF2G630308194	74586691	C	0	0	A
11	BICF2G630308196	74589290	A	0	0.2143	G
11	BICF2P264945	74604833	0	0	0	G
11	BICF2S2298714	74614662	C	0	0	A
11	BICF2G630308228	74632402	C	0	0	G
11	BICF2G630308236	74636256	0	0	0	G
11	BICF2G630308268	74646875	G	0.75	0.5714	A
11	BICF2G630308301	74658707	A	0.25	0.4286	C
11	TIGRP2P154065_rs8642031	74672619	T	0.25	0.2857	A
11	BICF2G630308352	74682811	A	0.75	0.5714	G
11	BICF2G630308390	74693181	C	0	0	G
11	BICF2G630308422	74713349	C	0	0	A
11	BICF2P1225513	74720219	G	0	0	A
11	BICF2G630308434	74730233	G	0	0	A
11	BICF2G630308447	74738862	G	0	0	A
11	TIGRP2P154075_rs8618839	74751831	0	0	0	A
11	BICF2G630308481	74772589	G	0	0	A
11	BICF2G630308483	74775738	A	0	0	C
11	BICF2G630308502	74784860	G	0	0	A
11	BICF2G630308538	74802423	G	0	0	A
11	BICF2G630308560	74810795	G	0	0	A
11	BICF2P241985	74821270	A	0.25	0.4286	G
11	BICF2P269278	74834179	A	0	0	G
11	BICF2S23121788	74864568	0	0	0	G

11	BICF2P847648	74891631	0	0	0	G
11	BICF2S22929936	74911463	G	0	0	A
11	BICF2G630308581	74929562	G	0.25	0.4286	A
11	BICF2G630308585	74938075	G	0.25	0.2857	A
11	BICF2G630308598	74948326	G	0.25	0.2857	A
11	BICF2P1372005	74962392	A	0.5	0.2857	C
11	BICF2S24111676	74976432	A	0.5	0.2857	G
11	BICF2G630308606	74981626	A	0.75	0.7143	G
11	BICF2P552813	74994236	G	0.25	0.4286	A
11	BICF2P1267841	75010057	A	0.75	0.5714	G
11	BICF2P281460	75017326	G	0.25	0.4286	A
11	TIGRP2P154099_rs9136774	75030322	0	0	0	A
11	BICF2S23330335	75050575	G	0	0	A
11	BICF2S23249964	75071514	G	0	0	A
11	BICF2P644919	75078376	A	0.25	0.4286	G
11	TIGRP2P154102_rs8941833	75091196	0	0	0	G
11	BICF2S23421031	75095302	A	0.5	0.75	C
11	BICF2P1055231	75110282	G	1	1	A
11	BICF2P251025	75126025	0	0	0	C
11	BICF2P486290	75138621	A	0.75	0.5714	G
11	BICF2P253948	75147593	A	0.25	0.4286	G
11	TIGRP2P154131_rs8728484	75152722	0	0	0	A
11	BICF2S22912258	75175363	A	0.25	0.2857	G
11	BICF2P383001	75194287	G	0.5	0.75	A
11	BICF2P312044	75199181	G	0.5	0.7143	A
11	TIGRP2P154134_rs8937238	75216521	G	0.25	0.2857	A
11	BICF2S2375539	75224726	A	0.25	0.2857	G
11	BICF2P973781	75237548	G	0.5	0.2857	A
11	BICF2G630308612	75243852	0	0	0	G
11	BICF2P956046	75245839	A	0.5	0.2857	G
11	BICF2P1365115	75257993	T	0.5	0.2857	A
11	BICF2P414186	75274903	G	0.5	0.7143	A
11	BICF2G630308623	75282118	G	0	0	A
11	BICF2G630308646	75292032	A	0	0	G
11	TIGRP2P154161_rs9183378	75307502	T	0.5	0.2857	A
11	TIGRP2P154165_rs9144510	75314284	A	0.5	0.2857	G
11	BICF2G630308704	75324064	G	0.5	0.7143	A
11	BICF2P718394	75325792	A	0.5	0.2857	G
11	BICF2S23020876	75338861	G	0	0	A
11	BICF2G630308756	75357343	A	0	0	G
11	TIGRP2P154185_rs9218496	75359530	C	0	0	A
11	BICF2G630308773	75373635	A	0	0	G
11	BICF2P887817	75382516	0	0	0	A
11	BICF2P738091	75400011	C	0.5	0.7143	A
11	BICF2P16466	75405674	A	0.5	0.7143	C
11	BICF2P1315253	75422384	A	0.5	0.2857	G
11	BICF2P338310	75429264	A	0.5	0.2857	G

11	BICF2G630308819	75447435	G	0.5	0.7143	A
11	BICF2G630308829	75456735	A	0	0	T
11	BICF2G630308830	75456823	A	0	0	G
11	BICF2G630308847	75468391	A	0	0	C
11	BICF2G630308863	75475208	G	0	0	A
11	BICF2G630308881	75486671	G	0	0	C
11	BICF2S23353290	75514571	C	0.5	0.75	A
11	BICF2P646781	75527922	A	0.5	0.2857	G
11	TIGRP2P154224_rs8819696	75534476	A	0	0	G
11	TIGRP2P154227_rs8775243	75544030	0	0	0	G
11	BICF2G630308933	75555631	G	0.5	0.2857	A
11	BICF2G630308964	75566801	A	0	0	C
11	BICF2G630308996	75581417	A	0.25	0.4167	G
11	TIGRP2P154233_rs8663077	75597467	G	0.25	0.4286	A
11	BICF2G630309052	75611015	A	0.25	0.2857	G
11	BICF2G630309085	75619361	A	0.25	0.2857	G
11	BICF2P1314357	75625067	G	0	0	A
11	BICF2P645580	75639715	A	0	0	G
11	BICF2G630309138	75647811	A	0.5	0.7143	G
11	BICF2G630309173	75663767	A	0	0	G
11	BICF2S23333010	75671929	A	0.25	0.4286	G
11	BICF2G630309229	75683349	G	0	0	A
11	TIGRP2P154287_rs8927470	75694780	G	0.25	0.2857	A
11	TIGRP2P154297_rs8917558	75713939	A	0.25	0.4286	G
11	BICF2P757655	75714100	G	0.25	0.4286	A
11	BICF2S23228375	75717527	A	0.75	0.7143	C
11	BICF2P1189485	75728358	A	0.5	0.7143	G
11	TIGRP2P154318_rs8963127	75739317	A	0	0	G
11	BICF2G630309367	75752480	G	0.5	0.2857	A
11	BICF2G630309440	75784443	G	0.75	0.5714	A
11	BICF2G630309455	75787023	A	0	0	G
11	BICF2G630309463	75803035	A	0	0	G
11	BICF2G630309469	75810998	A	0	0	C
11	BICF2P185250	75822314	A	0	0	G
11	BICF2G630309518	75831392	G	0.25	0.4286	A
11	BICF2P757296	75851582	G	0.5	0.2857	A
11	BICF2G630309556	75855093	A	0.25	0.2857	G
11	BICF2G630309582	75871031	A	0.25	0.2857	G
11	BICF2G630309587	75877028	G	1	1	A
11	BICF2G630309613	75888772	A	0.75	0.5714	G
11	BICF2G630309670	75920799	A	1	1	G
11	BICF2G630309689	75930341	A	0.25	0.2857	G
11	BICF2G630309703	75937612	G	1	1	A
11	BICF2G630309732	75950361	G	0.75	0.5714	A
11	BICF2G630309752	75957817	C	0	0	G
11	BICF2P254158	75977221	A	0.25	0.2857	G
11	BICF2G630309801	75982320	A	0.25	0.4286	G

11	BICF2G630309822	75992204	A	0.5	0.2857	C
11	BICF2S23721293	76006663	0	0	0	G
11	BICF2G630309873	76020894	A	0	0	G
11	BICF2G630309874	76027564	A	0	0	G
11	TIGRP2P154447_rs9139933	76038759	A	0	0	G
11	BICF2P274611	76044336	C	0	0	A
11	TIGRP2P154455_rs8546275	76050141	A	0	0	G
11	BICF2P1018502	76065337	A	0.5	0.3333	G
11	BICF2G630309896	76082107	G	0	0	A
11	BICF2P1432698	76085159	G	0	0	A
11	BICF2G630309920	76095573	A	0	0	C
11	BICF2G630309987	76108096	G	0.25	0.4286	A
11	BICF2G630310056	76129471	A	0.25	0.4286	T
11	BICF2G630310071	76130174	A	0.75	0.5714	G
11	BICF2S23055455	76142515	A	1	1	G
11	BICF2P780247	76161686	0	0	0	G
11	BICF2G630310208	76162635	0	0	0	A
11	BICF2G630310222	76169962	G	0.5	0.2857	A
11	BICF2G630310252	76179813	A	0	0	G
11	BICF2G630310293	76187575	G	0	0	A
11	BICF2G630310393	76209592	A	0	0	G
11	BICF2G630310414	76213997	A	0	0	G
11	BICF2G630310439	76224410	C	0.75	0.7143	A
11	BICF2G630310471	76242516	0	0	0	G
11	BICF2G630310484	76248914	C	0.25	0.2857	G
11	TIGRP2P154546_rs8821425	76258148	T	0	0	A
11	BICF2G630310525	76258880	G	0	0	A
11	TIGRP2P154567_rs8941149	76273234	A	0	0	G
11	BICF2G630310566	76280840	A	0	0	G
11	BICF2G630310584	76300709	G	0.75	0.7143	A
11	BICF2G630310585	76300751	A	0.75	0.7143	G
11	BICF2G630310586	76300936	A	0	0	G
11	BICF2G630310588	76301887	A	0	0	G
11	BICF2P1011646	76306333	G	1	1	A
11	BICF2G630310626	76319251	G	0.75	0.7143	A
11	BICF2S2328662	76326556	A	0	0	G
11	TIGRP2P154626_rs8733714	76338801	C	0	0.2857	A
11	BICF2G630310705	76350670	A	0.75	0.7143	G
11	TIGRP2P154649_rs8524795	76360134	A	0	0	G
11	BICF2G630310741	76376968	A	0.75	0.7143	G
11	BICF2S23718925	76389653	A	0.75	0.7143	G
11	TIGRP2P154662_rs8638003	76397822	C	0.25	0.2857	A
11	TIGRP2P154678_rs9073538	76409979	A	0	0	G
11	TIGRP2P154704_rs9225621	76422928	G	0	0	A
11	BICF2P417451	76435438	G	0	0	A
11	BICF2P1470535	76443504	G	0.75	0.7143	A
11	BICF2P1270897	76455987	A	0.25	0.2857	G

11	BICF2S23761729	76459948	G	1	1	A
11	BICF2P1214230	76474445	A	0	0	G
11	BICF2P774595	76480189	G	0.25	0.2857	A
11	BICF2G630310803	76489247	A	0.25	0.2857	G
11	TIGRP2P154786_rs9187761	76498881	G	0	0	A
11	BICF2G630310823	76518062	A	0	0	G
11	BICF2G630310831	76521159	C	0	0	A
11	BICF2G630310854	76541325	A	0.25	0.2857	G
11	BICF2G630310861	76544944	A	0.25	0.2857	C
11	BICF2P1315316	76562032	A	0	0	G
11	BICF2P899350	76576467	G	0.5	0.7143	A
11	BICF2G630310900	76587107	A	0.25	0.2857	G
11	BICF2P199408	76592482	A	0.25	0.4286	G
11	BICF2P1318396	76603094	A	0.25	0.4286	G
11	BICF2G630310930	76616906	G	0.75	0.5714	A
11	BICF2G630310945	76627806	A	0.25	0.2857	G
11	BICF2G630310971	76637023	A	0	0	G
11	BICF2G630311005	76653648	G	0	0	A
11	BICF2G630311021	76666267	A	0.25	0.2857	G
11	TIGRP2P154892_rs9194091	76671895	0	0	0	A
11	TIGRP2P154913_rs8883545	76692190	A	0.25	0.2857	C
11	BICF2P675488	76694087	A	0	0	G
11	BICF2G630311035	76713221	A	0.25	0.2857	G
11	TIGRP2P154939_rs8660596	76717884	A	0	0	G
11	BICF2G630311038	76717979	G	0	0	A
11	BICF2P714755	76724621	G	0.5	0.2857	A
11	BICF2P714744	76728163	C	0	0	A
11	BICF2G630311065	76744489	G	0.25	0.2857	A
11	BICF2S23114812	76751555	A	0	0	G
11	BICF2G630311107	76769655	C	1	1	A
11	BICF2G630311116	76775187	G	1	1	A
11	TIGRP2P154961_rs8822389	76794048	0	0	0	A
11	BICF2P371634	76798855	G	1	1	A
11	BICF2P798870	76809416	A	0.5	0.2857	G
11	BICF2G630311139	76828564	A	1	1	G
11	BICF2G630311155	76839440	A	1	1	G
11	BICF2G630311170	76845620	A	1	1	G
11	BICF2G630311181	76856173	G	1	1	A
11	BICF2G630311204	76868380	G	1	1	A
11	BICF2G630311225	76880316	A	0	0	G
11	BICF2S23439162	76889134	A	0.25	0.4286	G
11	BICF2P467357	76908698	A	0	0	G
11	BICF2G630311228	76921018	A	0	0	G
11	BICF2P752268	76930031	A	0.25	0.4286	G
11	BICF2G630311235	76942663	G	0.5	0.2857	A
11	BICF2G630311268	76951141	G	0.25	0.2143	C
11	BICF2P1244981	76961417	A	0	0	G

11	TIGRP2P155024_rs8910015	76969876	A	0	0	G
11	TIGRP2P155032_rs8765199	76981274	C	0.75	0.7857	A
11	BICF2G630311324	76992656	G	0.25	0.2857	A
11	BICF2G630311353	77007055	G	0.75	0.5714	A
11	BICF2G630311376	77018883	G	1	0.7857	A
11	BICF2G630311398	77027155	G	0	0	A
11	BICF2G630311427	77040102	G	0.25	0.4286	A
11	BICF2G630311429	77042104	G	0.75	0.7143	A
11	BICF2S23737751	77048593	C	0	0	A
11	BICF2G630311454	77050804	G	0.75	0.7143	A
11	BICF2G630311490	77061694	C	0.5	0.2857	G
11	BICF2G630311517	77077960	G	1	1	A
11	BICF2S22911225	77086060	G	0	0	A
11	BICF2G630311560	77105046	C	0	0	A
11	BICF2G630311561	77113843	A	1	1	G
11	BICF2G630311564	77134956	A	0	0	G
11	BICF2G630311573	77154998	G	1	1	A
11	BICF2S23432948	77193861	G	0	0	A
11	BICF2G630311588	77205524	A	1	1	G
11	BICF2G630311601	77211236	A	NA	0	C
11	BICF2G630311657	77233569	G	0	0	A
11	BICF2G630311662	77234659	A	0.5	0.5	G
11	BICF2S23521294	77249637	A	0	0	G
11	BICF2G630311715	77260005	A	0	0	G
11	BICF2P282343	77269758	G	0.5	0.5	A
11	BICF2G630311729	77279554	A	0.5	0.5	G
11	BICF2G630311735	77280708	A	0	0	C
11	BICF2G630311757	77295798	A	0.5	0.5	G
11	BICF2P3948	77305354	A	0.5	0.5	G
11	BICF2P670280	77315053	A	0.5	0.5	G
11	BICF2P1201469	77331701	G	0	0	A
11	BICF2P1031307	77344714	C	0.5	0.5	A
11	BICF2P159098	77356418	0	0	0	A
11	BICF2P398385	77371693	G	0.5	0.5	A
11	BICF2P83514	77381645	T	0.5	0.5	A
11	BICF2S23338871	77394920	A	0.5	0.5	G
11	BICF2S23322669	77398130	A	0.5	0.5	G
11	BICF2P1039227	77408959	A	0	0	G
11	BICF2P1089090	77415697	G	0.5	0.5	A
13	BICF2G630602134	3036181	A	0	0.2857	G
13	BICF2G630602144	3059745	C	0	0.2857	A
13	BICF2P1398043	3070571	0	NA	0	A
13	BICF2G630602161	3091358	C	0	0.2857	A
13	BICF2G630602166	3096116	G	0	0.2857	A
13	BICF2P905576	3108639	A	0	0.2857	G
13	BICF2P283616	3127761	A	0.25	0.2857	C
13	BICF2G630602185	3128888	A	0	0.2857	G

13	BICF2G630602202	3140444	A	0	0.2857	G
13	BICF2G630602211	3157151	A	0	0.2857	G
13	BICF2G630602213	3166236	A	0	0.2857	G
13	BICF2G630602219	3174563	A	0	0	G
13	BICF2G630602228	3186137	A	0	0.2857	G
13	BICF2G630602240	3207298	A	0	0.2857	G
13	TIGRP2P174027_rs8910829	3216282	0	0	0	G
13	BICF2S23118753	3220153	G	0	0.2857	A
13	BICF2P1405373	3241781	0	0	0	G
13	BICF2G630602264	3243042	C	0	0.2857	A
13	BICF2G630602279	3256712	A	0	0.2857	G
13	BICF2P610078	3266021	A	0.25	0.2857	C
13	BICF2G630602316	3278970	A	0	0.25	C
13	BICF2G630602317	3290838	A	0.25	0.2857	G
13	BICF2P788594	3302919	C	0.25	0.2857	A
13	BICF2G630602323	3327456	G	0.25	0.2857	A
13	BICF2G630602327	3336134	G	0.25	0.2857	A
13	BICF2G630602334	3352265	A	0	0.2857	G
13	BICF2G630602340	3364535	A	0.25	0.2857	G
13	BICF2G630602345	3379702	C	0.25	0.2857	A
13	BICF2G630602347	3382975	A	0.25	0.2857	G
13	BICF2G630602358	3403281	G	0	0	A
13	BICF2G630602361	3407199	G	0	0	A
13	BICF2G630602382	3424543	G	0	0	A
13	BICF2G630602385	3429392	G	0	0	A
13	BICF2G630602390	3459897	A	0	0	G
13	BICF2P435750	3462140	C	0	0.2857	G
13	BICF2G630602398	3476377	A	0	0	T
13	BICF2G630602408	3494613	A	0	0	C
13	BICF2G630602412	3496797	G	0	0	A
13	BICF2G630602420	3508769	G	0	0	A
13	BICF2G630602425	3528610	A	0	0	G
13	BICF2P125003	3536218	A	0	0	G
13	BICF2G630602435	3547351	G	0	0	A
13	BICF2G630602441	3560062	G	0	0	A
13	BICF2G630602449	3572749	G	0	0	A
13	BICF2G630602450	3578743	A	0	0	G
13	BICF2G630602458	3600562	A	0	0	G
13	BICF2G630602462	3611148	A	0	0	T
13	BICF2G630602471	3627320	A	0	0	C
13	BICF2P1325541	3636946	A	0	0	G
13	BICF2G630602486	3650134	G	0	0	C
13	BICF2G630602491	3657083	A	0	0.2857	G
13	BICF2G630602503	3670615	0	0	0	C
13	BICF2G630602511	3681562	A	0	0	G
13	BICF2G630602515	3691928	G	0	0	A
13	BICF2G630602523	3712451	C	0	0	A

13	BICF2G630602525	3714783	C	0	0	A
13	BICF2S23755243	3726649	A	0	0	C
13	BICF2G630602532	3737858	A	0	0	C
13	BICF2G630602547	3756458	A	0	0	G
13	BICF2G630602554	3760837	G	0	0	A
13	BICF2G630602563	3778724	G	0	0	A
13	BICF2G630602566	3786814	A	0	0	G
13	BICF2P1493587	3797137	G	0	0	A
13	BICF2G630602580	3810612	T	0	0	A
13	BICF2G630602584	3824116	A	0	0	G
13	BICF2G630602592	3833120	A	0	0	G
13	BICF2P622589	3848371	G	0	0	A
13	BICF2G630602605	3857350	G	0	0	A
13	BICF2G630602606	3866503	A	0	0	G
13	BICF2P150235	3886169	G	0	0	A
13	BICF2G630602616	3895672	A	0	0	G
13	BICF2G630602622	3903669	A	0	0	G
13	BICF2G630602624	3910336	A	0	0	C
13	BICF2P1044527	3928789	A	0	0	G
13	BICF2G630602636	3938457	A	0	0	G
13	BICF2G630602641	3945246	A	0	0	G
13	BICF2G630602646	3964960	A	0	0	G
13	BICF2G630602649	3977297	A	0	0	G
13	BICF2G630602652	3988820	G	0	0	A
13	BICF2G630602653	3990959	C	0	0	A
13	BICF2G630602673	4011324	A	0	0.2857	G
13	BICF2G630602679	4022456	G	0	0.2857	A
13	BICF2G630602681	4025406	G	0	0	A
13	BICF2G630602693	4042410	A	0	0	G
13	BICF2G630602706	4054152	A	0.25	0.5714	C
13	BICF2G630602713	4062631	G	0	0	A
13	BICF2G630602747	4098861	A	0	0	C
13	BICF2G630602752	4114923	G	0	0	A
13	BICF2G630602762	4128055	G	0	0	A
13	BICF2G630602771	4133574	C	0	0	A
13	BICF2G630602784	4142723	A	0.25	0.5714	G
13	TIGRP2P174082_rs9025829	4156306	0	0	0	C
13	BICF2G630602798	4176521	G	0	0	A
13	BICF2G630602815	4205861	A	0.25	0.5714	C
13	BICF2P852106	4218309	A	0	0	C
13	BICF2P557599	4265009	0	0	0	C
13	BICF2P933613	4272003	G	0.25	0.5714	A
13	BICF2G630602836	4286785	A	0.25	0.5714	G
13	BICF2G630602837	4291351	A	0.25	0.5714	C
13	BICF2P6104	4304867	0	0	0	A
13	BICF2P922869	4317608	A	0	0	G
13	BICF2G630602845	4333227	G	0	0	A

13	BICF2S23254242	4336760	C	0.75	0.4286	A
13	BICF2G630602851	4350125	A	0	0	G
13	BICF2G630602853	4359308	G	0	0	A
13	BICF2G630602856	4371999	C	0	0	G
13	BICF2P246233	4385618	0	0	0	G
13	BICF2P444644	4405146	G	0	0	A
13	BICF2G630602865	4416306	G	0	0	A
13	BICF2P1358635	4427788	0	0	0	A
13	BICF2G630602874	4451696	A	0	0	C
13	BICF2G630602876	4466720	A	0	0	C
13	BICF2P957335	4477964	C	0.25	0.5714	A
13	BICF2P403346	4485990	0	0	0	A
13	TIGRP2P174093_rs8705065	4511639	0	0	0	A
13	BICF2G630602893	4523040	G	0	0	A
13	BICF2P350778	4539623	0	0	0	G
13	BICF2G630602899	4548379	T	0	0	A
13	BICF2G630602905	4560582	C	0	0	A
13	BICF2P841532	4570420	A	0.25	0.5714	C
13	BICF2G630602913	4584574	G	0.25	0.5714	A
13	BICF2P1326570	4601826	A	0.25	0.5714	T
13	BICF2G630602922	4616501	A	0.25	0.5714	T
13	BICF2G630602925	4623932	G	0.25	0.5714	A
13	BICF2G630602926	4637282	G	0.75	0.4286	A
13	BICF2G630602931	4654445	A	0.25	0.5714	G
13	BICF2G630602935	4668935	A	0	0	G
13	BICF2P756435	4673412	0	0	0	G
13	BICF2S23411122	4681168	A	0.25	0.5714	G
13	BICF2S23733704	4697198	G	0.75	0.4286	C
13	BICF2P474181	4712609	0	0	0	G
13	BICF2G630602944	4722932	A	0.25	0.5714	G
13	BICF2G630602946	4730009	A	0	0	G
13	BICF2G630602950	4740978	G	0.25	0.5714	A
13	BICF2G630602952	4752645	0	0	0	C
13	BICF2G630602954	4769538	0	0	0	A
13	BICF2G630602962	4785534	A	0	0	G
13	BICF2G630602965	4797957	A	0	0	C
13	BICF2G630602969	4807000	G	0.25	0.5714	A
13	BICF2G630602973	4823169	A	0	0	G
13	BICF2G630602978	4831336	C	0.25	0.5714	A
13	BICF2G630602992	4844876	A	0	0	G
13	BICF2G630603007	4871620	G	0	0	A
13	BICF2G630603022	4912351	C	0	0	A
13	BICF2G630603030	4926649	A	0	0	G
13	BICF2G630603039	4940922	G	0	0	A
13	BICF2G630603044	4953303	C	0	0	A
13	BICF2G630603047	4956858	A	0	0	G
13	BICF2G630603051	4971031	A	0	0	G

13	BICF2G630603058	4989652	T	0	0	A
13	BICF2P1359888	4991492	0	0	0	A
13	BICF2G630603065	5004947	A	0	0	G
13	BICF2G630603074	5014634	G	0.25	0.5714	A
13	BICF2G630603095	5032439	A	0	0	G
13	BICF2G630603101	5038848	0	0	0	G
13	BICF2G630603118	5052198	T	0	0	A
13	BICF2G630603122	5061915	A	0	0	G
13	BICF2G630603141	5079942	C	0	0	A
13	BICF2P542422	5084814	G	1	0.4286	A
13	BICF2G630603152	5102985	A	0.75	0.4286	G
13	BICF2P1300931	5119338	A	0.75	0.4286	G
13	BICF2P712462	5132602	0	0	0	A
13	BICF2G630603160	5151804	G	0.25	0.5714	A
13	BICF2G630603161	5152585	A	0.25	0.5714	G
13	BICF2P795808	5164030	0	0	0	C
13	TIGRP2P180738_rs8846789	5177216	0	0	0	G
13	TIGRP2P180742_rs8891552	5192482	0	0	0	A
13	BICF2P983357	5201827	A	0	0	G
13	BICF2P3284	5220185	0	0	0	G
13	BICF2G630603189	5228516	A	0.75	0.4286	G
13	BICF2G630603194	5261274	A	0	0	G
13	BICF2P1174867	5264386	A	0.25	0.2857	G
13	BICF2G630603198	5270622	G	0.75	0.4286	A
13	BICF2G630603203	5290386	A	0	0	G
13	BICF2G630603205	5303959	A	0	0	G
13	BICF2P365003	5323044	G	0	0	A
13	BICF2S23753227	5332931	C	1	0.4	A
13	BICF2G630603211	5338690	A	0	0	C
13	BICF2G630603216	5348491	A	0	0	G
13	TIGRP2P180753_rs8927746	5373486	0	0	0	A
13	BICF2G630603222	5389511	A	0	0	G
13	BICF2P1083826	5394956	0	0	0	A
13	BICF2P1270204	5412504	0	0	0	G
13	BICF2P486678	5422919	G	0.75	0.4286	A
13	BICF2P1070641	5434105	0	0	0	C
13	BICF2S23231402	5444520	A	0.75	0.4167	G
13	BICF2G630603235	5452619	T	0.25	0.5714	A
13	BICF2P359548	5470260	0	0	0	C
13	BICF2G630603240	5478165	G	NA	NA	A
13	BICF2S23611117	5492055	0	0	0	A
13	BICF2P632664	5512124	G	0.25	0.2143	A
13	BICF2G630603262	5526971	G	0.5	0.2143	A
13	BICF2P286166	5543491	A	0.5	0.2143	C
13	BICF2G630603275	5555513	G	0.5	0.2143	A
13	BICF2G630603302	5575779	A	0.5	0.2143	G
13	TIGRP2P180792_rs8857877	5576204	A	0.25	0.2857	G

13	BICF2G630603303	5576301	A	0	0	G
13	BICF2G630603307	5577552	G	0.5	0.2143	A
13	BICF2G630603321	5591954	C	0.5	0.2	A
13	BICF2G630603322	5602578	A	0.5	0.2143	G
13	BICF2G630603325	5615915	A	0.5	0.2143	G
13	BICF2G630603329	5627970	G	0.5	0.2143	A
13	BICF2G630603335	5645379	A	0.5	0.2143	G
13	TIGRP2P180799_rs9124029	5660097	0	0	0	G
13	BICF2P936693	5671030	0	0	0	A
13	BICF2G630603346	5684200	C	0	0	G
13	BICF2P1270319	5693305	0	0	0	A
13	BICF2P1053953	5707116	A	0	0	G
13	BICF2G630603359	5716715	G	0	0	A
13	BICF2P875194	5721979	0	0	0	A
13	BICF2P1089381	5734118	0	0	0	G
13	BICF2G630603376	5744993	A	0	0	G
13	BICF2P1076453	5767757	0	0	0	G
13	BICF2G630603389	5788503	C	0	0	G
13	BICF2G630603398	5797455	A	0	0	C
13	BICF2P1233246	5810712	G	0	0	A
13	BICF2G630603417	5821919	G	0	0	C
13	BICF2G630603422	5832846	A	0	0	G
13	BICF2G630603433	5846823	G	0	0	A
13	BICF2G630603450	5860625	G	0	0	A
13	BICF2G630603460	5863333	A	0	0	T
13	BICF2G630603463	5865630	A	NA	0	T
13	BICF2P1169505	5878269	G	0	0	A
13	BICF2P399302	5896895	G	0.25	0.2143	C
13	BICF2P595104	5903746	0	0	0	G
13	TIGRP2P180901_rs8994138	5911608	A	0	0	G
13	BICF2G630603464	5935764	G	0	0	A
13	BICF2G630603466	5950742	0	0	0	G
13	BICF2P1009944	5957807	C	0	0	A
13	BICF2P358091	5977309	0	0	0	G
13	BICF2S23616262	5980254	G	0	0	C
13	BICF2G630603469	5996086	G	0	0	A
13	BICF2P1213669	6007042	G	0.5	0.2143	A
13	TIGRP2P180908_rs8730508	6020996	G	0.5	0.2143	A
13	BICF2P825645	6030059	A	0.5	0.2143	G
13	BICF2P359215	6038790	G	0.5	0.2143	A
13	BICF2P233875	6050149	0	0	0	C
13	BICF2G630603470	6068158	T	0.25	0.2857	A
13	BICF2G630603472	6075807	A	0	0	G
13	BICF2P1144319	6094486	G	0.5	0.2143	C
13	BICF2P580776	6102029	A	0.5	0.2143	G
13	BICF2P564654	6108101	G	0.5	0.2143	C
13	BICF2P653113	6118131	G	0.5	0.2143	A

13	BICF2P904228	6133020	A	0.5	0.2143	G
13	BICF2P40776	6167215	G	0.5	0.2143	A
13	BICF2P1270755	6179541	C	0.5	0.2143	A
13	BICF2P41815	6188023	A	0.5	0.5	C
13	BICF2P850402	6200154	G	0.5	0.2143	A
13	TIGRP2P180938_rs8741896	6218232	A	0.5	0.2143	G
13	BICF2P1102608	6228373	A	0.5	0.2143	G
13	TIGRP2P180948_rs9242607	6237740	A	0.5	0.2143	G
13	BICF2P1072189	6244563	G	0.5	0.2143	A
13	BICF2P497394	6260595	A	0.5	0.2143	G
13	BICF2P451548	6278522	0	0	0	A
13	BICF2P977971	6288737	G	0.5	0.2143	C
13	BICF2S2392193	6305486	A	0.5	0.2143	C
13	TIGRP2P180970_rs9026100	6317768	G	0.5	0.2143	A
13	BICF2P227491	6325026	0	0	0	A
13	BICF2P690213	6337263	A	0.5	0.2143	G
13	BICF2P1182174	6348310	0	0	0	A
13	BICF2G630603483	6365262	A	0	0.2857	G
13	BICF2G630603484	6375022	A	0	0.2857	G
13	BICF2P212177	6383843	G	0.5	0.2143	A
13	BICF2P137477	6410096	0	0	0	C
13	BICF2P809484	6418765	A	NA	0.2143	G
13	BICF2P1270259	6432929	A	0	0.2857	G
13	BICF2P324609	6449787	T	0.5	0.2143	A
13	BICF2P1346598	6456324	G	0.5	0.2143	A
13	TIGRP2P181002_rs9250723	6466156	A	0.5	0.2143	G
13	BICF2P353421	6477098	A	0.5	0.2143	G
13	BICF2P1445804	6487848	G	0.5	0.2143	A
13	BICF2P987559	6507623	G	0.5	0.2143	A
13	BICF2P987561	6509346	0	0	0	A
13	BICF2S2352768	6522377	0	0	0	G
13	BICF2P30752	6532185	A	0.5	0.2143	T
13	BICF2P787659	6544213	A	0.5	0.2143	G
13	TIGRP2P181018_rs8724645	6558190	A	0.5	0.2143	G
13	BICF2P177179	6568025	G	0.5	0.2143	A
13	BICF2G630603492	6578418	C	0.25	0.2143	A
13	BICF2P1073234	6604546	G	0.5	0.2143	A
13	BICF2P1460590	6623992	A	0.5	0.1667	C
13	BICF2G630603493	6633953	A	0.25	0.2143	G
13	TIGRP2P181032_rs9232035	6655568	A	0.5	0.2143	C
13	TIGRP2P181033_rs8931682	6664373	A	0.5	0.2143	C
13	TIGRP2P181034_rs9135796	6684479	G	0.5	0.2143	A
13	BICF2G630603497	6698034	A	0.25	0.2143	T
13	BICF2P1210979	6708802	0	0	0	A
13	BICF2G630603503	6742985	A	0	0.25	G
13	G774f30S24	6767750	A	NA	NA	G
13	TIGRP2P181042_rs8752974	6777361	A	0	0	G

13	BICF2P643476	6796109	G	0	0	A
13	BICF2P643473	6797044	G	0	0	A
13	TIGRP2P181056_rs9040793	6814813	A	0	0	G
13	TIGRP2P181067_rs8730125	6825855	A	0	0	G
13	TIGRP2P181073_rs9064035	6836797	G	0	0	A
13	TIGRP2P181083_rs8813898	6843178	A	0	0	G
13	TIGRP2P181093_rs9082989	6859868	0	0	0	A
13	BICF2G630603513	6873170	A	0	0.2857	T
13	BICF2P1293961	6910792	A	0	0	T
13	BICF2P747561	6913672	0	0	0	A
13	BICF2P573407	6928170	0	0	0	A
13	TIGRP2P181107_rs8762427	6936371	0	0	0	A
13	BICF2P723539	6950323	0	0	0	A
13	TIGRP2P181109_rs9187529	6962949	0	0	0	G
13	TIGRP2P181111_rs8703803	6969517	A	0.5	0.2143	G
13	BICF2P1148756	6985203	A	0	0	G
13	TIGRP2P181116_rs8607445	6999661	A	0	0	G
13	BICF2P86662	7003570	0	0	0	A
13	BICF2S23524989	7021972	0	0	0	G
13	BICF2G630603518	7035021	C	0	0.2857	A
13	BICF2G630603519	7039420	A	0.25	0.2857	G
13	BICF2P164787	7058508	0	0	0	A
13	G775f32S135	7061632	0	0	0	C
13	BICF2P103050	7086495	A	0	0	C
13	BICF2P652420	7098829	A	0	0	G
13	TIGRP2P181128_rs8520426	7107136	0	0	0	G
13	TIGRP2P181136_rs8613781	7127019	0	0	0	G
13	BICF2P765162	7139916	0	0	0	G
13	TIGRP2P181139_rs8982906	7146188	A	0	0.2857	G
13	BICF2P1438364	7156582	0	0	0	G
13	BICF2P867078	7166088	A	0	0.2857	G
13	BICF2P1224076	7182266	G	0	0.25	A
13	BICF2P319605	7199010	G	0	0.2857	A
13	BICF2P539973	7218790	G	0	0.2857	A
13	BICF2P775727	7233003	A	0	0.2857	C
13	BICF2S2378899	7237691	A	0	0.2857	G
13	TIGRP2P181167_rs8671465	7246599	A	0	0.2857	G
13	BICF2P474464	7257604	0	0	0	A
13	BICF2P665153	7279145	G	0	0.2857	A
13	TIGRP2P181170_rs8670334	7290491	C	0	0.2857	G
13	BICF2P782371	7306027	A	0	0.2857	T
13	BICF2P245526	7323036	G	0.25	0.5	A
13	BICF2P889350	7331416	G	0.25	0.5	A
13	BICF2P1263141	7344665	G	0.25	0.5	A
13	BICF2S23144512	7353018	A	0.25	0.5	G
13	BICF2P990678	7363678	A	0.25	0.5	G
13	BICF2P1135671	7374283	C	0.25	0.2143	A

13	BICF2G630603527	7376238	C	0.25	0.5	A
13	BICF2S23122792	7384059	A	0.25	0.5	G
13	TIGRP2P181203_rs8844102	7401047	C	0	0.2857	G
13	TIGRP2P181208_rs8609594	7410513	G	0.25	0.5	A
13	BICF2P839832	7423360	A	0.75	0.4286	G
13	BICF2P212924	7432836	0	0	0	A
13	TIGRP2P181210_rs9040063	7442429	0	0	0	C
13	BICF2P1028734	7455499	A	0	0.2857	G
13	TIGRP2P181213_rs8647242	7469784	A	0	0.2857	C
13	BICF2P906491	7485254	A	0	0.25	G
13	BICF2P1237530	7486596	C	0	0.2857	A
13	BICF2P529071	7498752	A	0	0.2857	G
13	BICF2P871825	7513787	G	0	0.2857	A
13	BICF2P536955	7527445	A	0.5	0.5	T
13	BICF2G630603538	7532622	A	0.25	0.5714	G
13	TIGRP2P181236_rs9196016	7562538	0	0	0	G
13	BICF2P651696	7565502	A	0.5	0.2143	G
13	BICF2G630603541	7575251	A	0	0	G
13	BICF2G630603542	7580033	G	0.25	0.2857	A
13	BICF2P900970	7591804	0	0	0	A
13	BICF2P334420	7601652	0	0	0	A
13	BICF2G630603545	7618781	A	0.25	0.2143	G
13	BICF2P727971	7626816	A	0.5	0.6	G
13	BICF2P535075	7636202	G	0.5	0.5	A
13	BICF2P955005	7649483	G	0	0.2857	A
13	TIGRP2P181245_rs8969472	7672985	0	0	0	C
13	BICF2G630603552	7709145	G	0.25	0.5	A
13	BICF2P120057	7730253	0	0	0	A
13	BICF2S23613832	7748971	G	0.5	0.5	A
13	BICF2P298945	7754512	G	0	0	A
13	BICF2P202705	7763093	0	0	0	A
13	BICF2P266270	7782960	0	0	0	C
13	BICF2S23261183	7788337	A	0.5	0.5	G
13	BICF2P126049	7807220	A	0	0	G
13	BICF2P823099	7815787	G	0	0	A
13	TIGRP2P181257_rs8955981	7823506	G	0	0.2857	A
13	BICF2P746996	7833097	G	NA	0	A
13	BICF2P4310	7846208	0	0	0	G
13	TIGRP2P181265_rs8685944	7860357	A	0.75	0.5	G
13	TIGRP2P181270_rs9134711	7868035	A	0.75	0.5	G
13	BICF2G630603557	7879989	0	0	0	C
13	BICF2S23020894	7889527	G	0.75	0.5	A
13	BICF2S23132551	7908654	A	0	0	G
13	BICF2P734842	7915781	A	0.75	0.5	G
13	TIGRP2P181290_rs9239018	7934222	A	0.75	0.5	G
13	TIGRP2P181293_rs9239013	7935439	G	0.75	0.5	A
13	BICF2P31344	7957327	G	1	0.5	A

13	BICF2G630603558	7967301	A	0	0	C
13	TIGRP2P181303_rs8735812	7991865	G	0.75	0.5	A
13	TIGRP2P181304_rs8449981	7999498	A	0.75	0.5	G
13	BICF2P665811	8016403	G	0.75	0.5	A
13	BICF2P847641	8035999	C	0.75	0.5	G
13	BICF2P483673	8052539	C	0.75	0.5	G
13	BICF2S23012118	8061858	A	0.75	0.5	G
13	BICF2P1439231	8079481	A	0.75	0.5	G
13	BICF2P1006529	8089391	A	0.75	0.5	G
13	BICF2P997955	8103334	G	0.75	0.5	A
13	BICF2P80259	8110936	A	0.75	0.5	G
13	BICF2P638114	8138903	A	0.75	0.5	G
13	BICF2P3449	8156093	0	0	0	G
13	TIGRP2P181339_rs8933719	8167754	G	0.75	0.5	C
13	BICF2P235546	8180230	G	0.75	0.5	A
13	BICF2P1028658	8197127	G	0.75	0.5	A
13	BICF2P1158183	8203378	0	0	0	A
13	BICF2P1129143	8214295	0	0	0	C
13	BICF2S23139507	8233847	A	0	0	G
13	TIGRP2P181348_rs8997745	8234655	A	0.75	0.5	G
13	BICF2P1135301	8264251	0	0	0	A
13	BICF2P543364	8286652	0	0	0	A
13	BICF2S23334507	8316233	0	0	0	A
13	BICF2P813984	8338711	0	0	0	A
13	BICF2S23210168	8350429	C	0.75	0.5	G
13	BICF2S23719582	8361957	G	0.75	0.5	A
13	BICF2S2342531	8379797	G	0.75	0.5	A
13	BICF2P188790	8384816	0	0	0	G
13	BICF2S23256495	8414539	A	0.75	0.5	G
13	BICF2S23732530	8420838	A	0.75	0.5	G
13	BICF2S241540	8431437	G	0.75	0.5	A
13	BICF2P384765	8445044	G	0.25	0.5	A
13	TIGRP2P181364_rs8883537	8458731	G	0.75	0.5	A
13	BICF2P231397	8466836	G	0.75	0.5	A
13	BICF2P852903	8486489	A	0.75	0.5	G
13	TIGRP2P181373_rs8868942	8487331	A	0.75	0.5	C
13	BICF2P1407441	8500213	G	0	0	A
13	BICF2P497897	8516231	A	0.75	0.5	C
13	BICF2P1292661	8523291	0	0	0	A
13	BICF2P778851	8541665	0	0	0	C
13	TIGRP2P181379_rs9030370	8555877	A	0.75	0.5	C
13	BICF2G630603571	8559700	G	0	0	A
13	BICF2G630603587	8570574	G	0	0	A
13	BICF2P494104	8597098	G	0.25	0.2143	A
13	BICF2G630603616	8609513	G	0	0	A
13	BICF2G630603637	8622277	G	0	0	A
13	BICF2P890617	8628066	C	0	0	A

13	BICF2G630603662	8643743	C	0	0	A
13	BICF2G630603677	8664419	G	0	0	A
13	BICF2G630603698	8673599	A	0	0	G
13	BICF2G630603719	8682536	A	0	0	C
13	BICF2P586094	8705560	0	0	0	A
13	BICF2G630603723	8726417	A	0	0.2857	C
13	BICF2G630603728	8731297	A	0	0.2857	C
13	BICF2G630603731	8748568	G	0	0.2857	A
13	BICF2G630603732	8753528	G	0	0.25	A
13	BICF2G630603735	8768409	A	0	0.2857	G
13	BICF2S2456735	8776401	C	0	0	A
13	BICF2P1038537	8790128	0	0	0	C
13	BICF2G630603736	8809291	0	0	0	A
13	BICF2G630603739	8828643	G	0	0.2857	A
13	BICF2G630603749	8843093	0	0	0	G
13	BICF2P1414212	8853634	C	0	0	A
13	BICF2P347710	8866156	C	0	0	G
13	BICF2G630603771	8874163	G	0	0.2857	A
13	BICF2G630603779	8880869	A	0	0.2857	G
13	BICF2G630603792	8889804	A	0	0.2857	G
13	BICF2P33744	8909924	A	0.25	0.2143	C
13	BICF2G630603806	8913536	G	0	0.2857	A
13	BICF2G630603817	8927987	G	0	0.2857	A
13	BICF2G630603823	8939926	A	0	0.2857	C
13	BICF2G630603827	8949554	G	0.25	0.5	A
13	BICF2S23143168	8962280	G	0.25	0.2143	A
13	BICF2S23355180	8970080	G	0.25	0.2143	A
13	BICF2G630603839	8990142	G	0	0	A
13	BICF2P235011	8999700	0	0	0	A
13	BICF2G630603845	9015640	A	0	0	G
13	BICF2S23640649	9024539	G	0.25	0.2143	A
13	BICF2G630603862	9035438	A	0	0	G
13	BICF2G630603865	9039021	G	0	0	A
13	BICF2P872271	9058565	A	0	0	G
13	BICF2P451803	9065696	A	0	0	G
13	BICF2G630603880	9077287	G	1	0.5	A
13	BICF2G630603885	9093802	G	0.75	0.7857	A
13	BICF2G630603890	9100021	A	0.75	0.7857	G
13	BICF2G630603902	9112226	A	0.75	0.7857	G
13	BICF2G630603915	9120371	A	0.25	0.2143	G
13	BICF2G630603929	9136758	C	0.25	0.2143	A
13	BICF2G630603937	9143429	A	0.75	0.7857	G
13	BICF2P787831	9158719	0	0	0	A
13	BICF2G630603951	9175770	G	0.75	0.4286	A
13	BICF2G630603952	9177584	C	0.75	0.4286	A
13	BICF2P264618	9190458	0	0	0	A
13	BICF2G630603957	9201177	0	0	0	G

13	BICF2P1306594	9212814	A	0	0	G
13	BICF2G630603961	9232063	C	0.75	0.4286	A
13	BICF2G630603964	9236734	G	0.75	0.4286	A
13	BICF2P1373914	9249895	0	0	0	A
13	BICF2G630603967	9260105	A	0.75	0.4286	G
13	BICF2S23913925	9276040	A	0	0	G
13	TIGRP2P181410_rs8823670	9284847	0	0	0	C
13	BICF2G630603982	9294240	A	0.75	0.4286	G
13	BICF2G630603988	9312669	A	0.25	0.5714	T
13	BICF2G630603989	9315602	G	0.25	0.5714	A
13	BICF2P480953	9327957	A	0.75	0.4286	G
13	BICF2G630604010	9338939	A	0.75	0.4286	C
13	BICF2G630604036	9366796	A	0.5	0.7857	G
13	BICF2G630604041	9376051	A	0.25	0.5714	G
13	BICF2G630604056	9391939	G	0.25	0.2857	A
13	BICF2G630604061	9396244	G	0.25	0.2857	A
13	BICF2G630604069	9412241	G	0.25	0.2857	A
13	BICF2G630604073	9425427	G	0.5	0.5	C
13	BICF2G630604075	9436758	A	0.5	0.5	G
13	BICF2G630604084	9446019	0	0	0	G
13	BICF2G630604089	9460241	A	0.25	0.2857	G
13	BICF2G630604094	9471791	G	0.25	0.2857	A
13	BICF2G630604098	9478177	A	0.25	0.2857	G
13	BICF2P656090	9489053	A	0.5	0.1667	T
13	BICF2P533597	9500401	0	0	0	A
13	BICF2G630604107	9511191	A	0.25	0.2857	G
13	BICF2G630604126	9530387	A	0.75	0.5	C
13	BICF2P413570	9542317	C	0.5	0.2143	A
13	BICF2P413568	9542434	A	0.75	0.5	G
13	BICF2G630604144	9547275	0	0	0	A
13	BICF2G630604149	9556551	A	0.75	0.5	G
13	BICF2P243755	9569071	A	0.5	0.2143	G
13	BICF2P696406	9579735	A	0.25	0.2143	G
13	TIGRP2P181413_rs9073163	9608084	0	0	0	G
13	BICF2G630604154	9631366	C	0	0	A
13	BICF2G630604180	9641925	G	0	0	A
13	BICF2G630604186	9648877	T	0	0	A
13	BICF2G630604187	9655009	A	0	0	G
13	BICF2G630604193	9667029	A	0	0	G
13	BICF2G630604202	9671587	A	0.25	0.2143	G
13	BICF2S23112860	9693482	G	1	0.7143	A
13	BICF2G630604211	9700593	A	0.75	0.7857	G
13	BICF2G630604220	9713561	A	0.5	0.5	C
13	BICF2P1001146	9718234	A	0.5	0.5	G
13	BICF2G630604228	9736204	A	0.5	0.2143	G
13	BICF2P249712	9741199	A	0.5	0.5	C
13	BICF2G630604233	9752655	G	0	0	A

13	BICF2S23736314	9766620	A	1	1	C
13	BICF2P231533	9776710	0	0	0	A
13	BICF2S23515572	9792541	G	0.5	0.7857	A
13	BICF2G630604235	9809028	A	0	0	G
13	BICF2P95340	9810571	A	0.75	0.5	G
13	BICF2G630604236	9823464	C	0	0	A
13	BICF2G630604241	9841710	C	0.75	0.7857	A
13	BICF2P278090	9846943	A	0.75	0.7857	G
13	BICF2G630604267	9862864	G	0.75	0.7857	A
13	BICF2G630604277	9876033	A	0.75	0.7857	G
13	BICF2G630604281	9882443	G	0.75	0.7857	A
13	BICF2G630604307	9899740	C	0.5	0.2143	A
13	BICF2G630604322	9906277	A	0.75	0.4286	T
13	BICF2G630604327	9917267	T	0.75	0.4286	A
13	BICF2P865483	9933429	G	0	0	A
13	BICF2P1265506	9938868	G	0	0	A
13	BICF2G630604348	9953636	C	0.5	0.2143	A
13	BICF2P172330	9964262	0	0	0	A
13	BICF2P1129070	9971940	0	0	0	A
13	BICF2G630604368	9997980	A	0	0	C
13	BICF2P550591	10007299	C	0	0	A
13	BICF2P935905	10035670	A	1	1	G
13	BICF2P265073	10044470	A	0	0	C
13	BICF2S23155674	10055619	C	1	1	A
13	BICF2P869769	10070026	G	0.75	0.7857	A
13	BICF2P1275808	10084566	A	0.25	0.2143	G
13	BICF2G630604383	10086210	A	0	0	T
13	BICF2P925145	10101417	C	0.75	0.7857	A
13	BICF2P989582	10102141	A	0.75	0.7857	G
13	BICF2P1429591	10115775	A	0	0	G
13	BICF2P1429586	10117059	A	0.75	0.7857	T
13	BICF2P437114	10120443	G	0	0	A
13	BICF2P437112	10120596	G	0	0	A
13	BICF2G630604386	10135029	G	0	0	C
13	BICF2S24321767	10152493	A	0	0	G
13	BICF2S23512657	10157151	G	0	0	A
13	BICF2G630604391	10175062	A	0	0	G
13	BICF2G630604411	10182231	A	0	0	G
13	BICF2G630604415	10184498	G	0.25	0.5714	A
13	BICF2G630604417	10192590	A	0.25	0.5714	G
13	BICF2P1231504	10210459	G	0	0	A
13	BICF2S23727080	10214746	A	0	0	G
13	BICF2P1130785	10225305	A	0	0	G
13	BICF2G630604436	10236846	A	0.75	0.7857	G
13	BICF2G630604442	10250132	G	0.75	0.7857	A
13	BICF2P634248	10266126	C	0.75	0.7857	G
13	BICF2G630604443	10270128	G	0.75	0.7857	A

13	BICF2G630604463	10299977	A	0.75	0.7857	G
13	BICF2P330444	10312827	A	0.75	0.5	G
13	BICF2G630604479	10324590	G	0.75	0.5	A
13	BICF2G630604494	10329416	A	0	0	G
13	BICF2G630604496	10329710	A	0	0	C
13	BICF2G630604512	10339055	A	0	0	G
13	BICF2G630604541	10355725	G	0	0	A
13	BICF2G630604551	10362349	A	0	0	G
13	BICF2G630604590	10380255	A	0	0	G
13	BICF2G630604608	10384632	A	0	0	G
13	BICF2G630604629	10399296	A	0	0	G
13	BICF2G630604655	10408414	A	0	0	G
13	BICF2G630604676	10423744	G	0	0	A
13	BICF2P310560	10436318	A	0.75	0.7857	G
13	BICF2P975682	10444670	A	0.75	0.5	G
13	BICF2G630604698	10457808	A	0	0	G
13	BICF2G630604710	10468017	A	0	0.2857	G
13	BICF2G630604723	10479954	G	0.75	0.5	A
13	BICF2G630604725	10509063	A	0.75	0.5	T
13	BICF2G630604727	10514481	A	0	0	C
13	BICF2G630604729	10526648	G	0	0	A
13	BICF2P526099	10537947	A	0.75	0.5	G
13	BICF2P606262	10546978	0	0	0	G
13	BICF2S22953483	10562921	G	0.25	0.5	A
13	BICF2P1384135	10573452	C	0	0	G
13	BICF2P583203	10584164	G	0	0	A
13	BICF2P501999	10612476	A	0	0	G
13	BICF2S22937164	10623213	A	0.25	0.5	G
13	BICF2S2296327	10629032	G	0.25	0.2143	A
13	BICF2S23223177	10644572	A	0	0.2857	G
13	BICF2S23763039	10656321	G	0.25	0.5	A
13	BICF2S23749989	10673760	G	0	0	A
13	BICF2P903017	10686666	0	0	0	A
13	BICF2G630604787	10719282	0	0	0	A
13	BICF2P1337526	10733876	0	0	0	G
13	BICF2G630604789	10745334	G	1	1	C
13	BICF2S23056118	10755023	G	1	0.7143	A
13	BICF2S22920359	10775477	G	0.75	0.5	A
13	BICF2P85832	10801155	A	0	0	G
13	BICF2G630604796	10819921	G	0.75	0.7857	A
13	BICF2G630604797	10821593	C	0.75	0.7857	A
13	BICF2P845571	10837111	A	0	0	G
13	BICF2G630604828	10852921	G	0.75	0.7857	A
13	BICF2G630604837	10857955	A	0.75	0.7857	C
13	BICF2G630604851	10877143	A	0	0	G
13	BICF2G630604855	10881117	G	0	0	A
13	BICF2G630604865	10893926	C	0	0	G

13	BICF2S23043043	10904747	A	0.75	0.5	G
13	BICF2G630604902	10923168	A	0	0	G
13	BICF2G630604915	10931249	A	0	0	G
13	BICF2G630604934	10946917	A	0	0	G
13	BICF2P144523	10949249	A	0	0	G
13	BICF2P61075	10960844	G	0.25	0.2143	A
13	BICF2P927359	10987746	A	0.75	0.5	G
13	BICF2P1437856	10999826	A	0.75	0.7857	G
13	BICF2G630604948	11007041	A	0.75	0.7857	C
13	BICF2G630604951	11017292	G	0	0	A
13	BICF2G630604960	11047731	A	0	0	G
13	BICF2G630604966	11061820	A	0.25	0.2143	G
13	BICF2G630604969	11074395	A	0.25	0.2143	G
13	BICF2S23051794	11079568	G	0.25	0.2143	A
13	BICF2P1055154	11095120	A	0	0	G
13	BICF2G630604984	11097671	G	0.75	0.7857	A
13	BICF2G630604996	11118383	A	0	0	G
13	BICF2G630604997	11121755	C	0	0	A
13	BICF2G630605021	11132643	G	0.75	0.7857	A
13	BICF2G630605045	11150514	A	0.25	0.2143	G
13	BICF2P909231	11155020	A	0	0	G
13	BICF2G630605058	11166724	A	0.75	0.7857	G
13	BICF2P419989	11188617	A	0	0.2857	G
13	BICF2G630605068	11197372	0	0	0	G
13	BICF2G630605079	11202610	A	0.75	0.5	G
13	BICF2P293816	11220582	0	0	0	A
13	BICF2S23135424	11224697	A	0	0	C
13	BICF2P604375	11238628	A	0	0	G
13	BICF2G630605092	11256817	C	0	0.1	A
13	BICF2P1423563	11261655	C	0	0	G
13	BICF2P282164	11270787	0	0	0	A
13	BICF2P677087	11288082	A	0.25	0.2143	G
13	BICF2P579064	11295844	0	0	0	A
13	BICF2P946181	11308705	0	0	0	A
13	BICF2S23141275	11318222	A	0.25	0.2143	G
13	BICF2P497347	11344124	0	0	0	A
13	BICF2S2297323	11360713	A	1	0.7143	G
13	BICF2P795344	11366694	G	0.25	0.2143	A
13	BICF2S23121336	11381435	A	0	0	G
13	BICF2P787045	11385898	A	1	1	G
13	BICF2P1510386	11401675	A	0	0	G
13	BICF2P1389702	11415999	A	0	0	G
13	BICF2P1371290	11425908	A	0	0	G
13	BICF2S23054512	11434354	A	0	0	G
13	BICF2S23012774	11448697	G	0	0	A
13	BICF2G630605109	11466979	A	0.25	0.2143	C
13	BICF2G630605129	11496408	G	1	0.7143	A

13	BICF2G630605143	11509194	A	0	0	G
13	BICF2G630605148	11513549	0	0	0	G
13	BICF2G630605160	11528745	G	0	0	A
13	BICF2G630605193	11542152	G	0	0	A
13	BICF2P32064	11547853	G	0	0	A
13	BICF2G630605222	11560350	A	0	0	G
13	BICF2G630605234	11570084	A	0	0	G
13	BICF2G630605270	11593074	A	0	0	C
13	BICF2G630605297	11610207	A	0	0	G
13	BICF2S23527822	11616021	C	0.75	0.7857	A
13	BICF2G630605313	11636793	T	0.75	0.7857	A
13	BICF2G630605317	11643024	C	0	0	A
13	BICF2G630605333	11655718	A	0	0	G
13	BICF2G630605339	11659792	A	0	0	G
13	BICF2G630605340	11660194	G	0	0	A
13	BICF2G630605341	11661960	A	0	0	G
13	BICF2P112526	11678731	A	0	0	G
13	BICF2G630605342	11686241	0	0	0	A
13	BICF2G630605344	11695899	C	0.75	0.5	A
13	BICF2P182473	11728542	G	0	0.2857	A
13	BICF2G630605349	11733448	G	0	0	A
13	BICF2G630605354	11742565	A	0.25	0.5	G
13	BICF2G630605356	11777117	G	0.25	0.2143	A
13	BICF2G630605357	11790465	C	0.25	0.2143	G
13	BICF2G630605367	11810072	A	0	0	G
13	BICF2G630605369	11811625	G	0.25	0.5	A
13	BICF2S23340769	11811850	A	0.25	0.5	G
13	BICF2G630605374	11833265	A	0	0	G
13	BICF2G630605379	11834689	A	0.25	0.5	G
13	BICF2S2447904	11847620	A	0	0	T
13	BICF2P1251877	11862371	0	0	0	A
13	BICF2G630605401	11870370	G	0	0	A
13	BICF2S23654432	11885247	A	0.5	0.5	C
13	TIGRP2P174168_rs9094905	11906944	0	0	0	C
13	BICF2G630605405	11920014	G	0	0	A
13	BICF2G630605407	11926079	A	0.5	0.2143	C
13	BICF2G630605411	11938203	T	0	0.2857	A
13	BICF2S2331377	11958909	G	0.25	0.2143	A
13	BICF2G630605426	11972718	A	0.25	0.2857	G
13	BICF2G630605438	11989716	G	0.25	0.2857	C
13	BICF2G630605442	11994854	G	0	0	A
13	BICF2G630605449	12023598	G	0.25	0.2857	A
13	BICF2G630605454	12035109	A	0.25	0.2857	G
13	BICF2G630605460	12043708	G	0.25	0.2857	A
13	BICF2P983123	12052105	G	0.25	0.2857	A
13	BICF2P1144501	12064248	T	0.25	0.2857	A
13	BICF2G630605491	12075858	C	0.25	0.2857	G

13	BICF2G630605500	12086712	A	0.25	0.2857	C
13	BICF2G630605522	12106892	A	0	0	G
13	BICF2P343967	12116369	G	0	0	A
13	BICF2G630605526	12129387	A	0	0	G
13	BICF2S22937486	12132815	G	0.75	0.7143	A
13	BICF2G630605529	12144248	A	0	0	G
13	BICF2G630605532	12161770	A	0	0	G
13	BICF2G630605535	12174294	A	0.25	0.2857	G
13	BICF2G630605541	12204152	G	0	0	A
13	BICF2S22921708	12231229	A	0.75	0.7143	G
13	BICF2G630605548	12242584	A	0	0	C
13	BICF2G630605552	12251646	A	0.25	0.2857	G
13	BICF2P435809	12260714	C	0	0	A
13	BICF2S23130605	12275607	C	0	0	A
13	BICF2P1270678	12283002	A	0	0	G
13	BICF2G630605567	12296356	A	0	0	G
13	BICF2G630605598	12315643	A	0	0	T
13	BICF2G630605618	12323569	G	1	1	A
13	BICF2G630605635	12331436	0	0	0	A
13	BICF2P734712	12347011	C	0	0	A
13	BICF2S22913351	12357456	A	1	1	G
13	BICF2S22938994	12367011	A	1	1	G
13	BICF2P1189575	12404823	G	0	0	A
13	BICF2G630605644	12408947	0	0	0	A
13	BICF2G630605650	12427550	G	0	0	A
13	BICF2G630605663	12433017	A	0.5	0.7857	G
13	BICF2G630605675	12451615	A	0.5	0.7857	G
13	BICF2P456971	12473376	A	0.25	0.2143	G
13	BICF2P616206	12479844	G	0.25	0.2143	A
13	BICF2G630605680	12483674	G	0.25	0.2143	C
13	BICF2P731314	12493666	A	0	0	C
13	BICF2P96572	12509494	A	0.5	0.2143	G
13	BICF2P375201	12513114	G	0	0	A
13	BICF2S2337542	12534382	A	0	0	G
13	BICF2P1372167	12539831	G	0	0	A
13	BICF2S23642149	12549497	A	0	0	G
13	BICF2G630605713	12561842	A	0	0	G
13	BICF2G630605714	12562038	G	0	0	A
13	BICF2G630605736	12579394	A	0	0	G
13	BICF2G630605745	12583708	G	0	0	A
13	BICF2G630605760	12601634	A	0	0	G
13	BICF2G630605764	12605118	A	0	0	G
13	BICF2G630605769	12618614	A	0	0	G
13	BICF2G630605786	12628723	A	0	0	G
13	BICF2G630605794	12644674	C	0	0	A
13	BICF2G630605804	12653353	G	0	0	A
13	BICF2P495552	12661694	A	0.5	0.7857	G

13	BICF2G630605829	12685677	A	0	0	G
13	BICF2G630605855	12697436	A	0	0	C
13	BICF2G630605866	12707657	A	0.25	0.2143	G
13	BICF2S23649766	12728215	G	0	0	A
13	BICF2S23537943	12741556	G	0.25	0.5714	A
13	BICF2S23029501	12748018	A	0.25	0.5714	G
13	BICF2G630605874	12758804	C	0.25	0.2143	G
13	BICF2G630605909	12783991	A	0.75	0.7857	G
13	BICF2G630605914	12790841	A	0.75	0.7857	G
13	BICF2G630605923	12804567	C	0.75	0.7857	A
13	BICF2G630605935	12818267	A	0.25	0.2143	C
13	BICF2G630605936	12831900	A	0	0	C
13	BICF2G630605940	12837128	A	0	0	G
13	BICF2P755556	12854536	0	0	0	G
13	BICF2G630605965	12858496	G	0	0	A
13	BICF2G630605982	12877115	C	0.25	0.2143	A
13	BICF2G630605984	12880163	C	0	0	A
13	BICF2S23350136	12897466	G	0.25	0.2143	A
13	BICF2G630606011	12914215	A	0	0.1667	G
13	BICF2P179492	12923537	A	0	0	C
13	BICF2S23419869	12929609	G	0	0	A
13	BICF2P977887	12938337	C	0	0	A
13	BICF2P473110	12956551	G	0.75	0.7857	A
13	BICF2P1268921	12964124	G	0	0	C
13	BICF2G630606044	12984746	G	0	0	A
13	BICF2S2418953	13005012	G	0.75	0.7857	A
13	BICF2S23549758	13015954	A	1	0.7857	C
13	BICF2P1141408	13020400	A	0.25	0.2143	G
13	BICF2G630606061	13029923	G	0.75	0.7857	C
13	BICF2G630606068	13035008	G	0	0	A
13	BICF2G630606073	13042853	A	1	1	G
13	BICF2P1094638	13061559	0	0	0	G
13	BICF2P784470	13065667	A	0	0	C
13	BICF2G630606101	13083847	C	0	0	A
13	BICF2G630606105	13090256	A	0	0	G
13	BICF2G630606109	13102322	G	0	0	A
13	BICF2G630606112	13106665	G	0	0	A
13	BICF2G630606119	13113451	A	0	0	C
13	BICF2P238217	13122130	A	0.75	0.7857	G
13	BICF2G630606150	13136320	G	0	0	A
13	BICF2G630606164	13148662	A	0	0	G
13	BICF2G630606173	13160159	A	0	0	G
13	BICF2G630606178	13168740	G	0	0	A
13	BICF2G630606199	13189273	C	0	0	A
13	BICF2G630606204	13191679	A	0	0	G
13	BICF2G630606215	13210254	G	0	0	A
13	BICF2P79898	13228453	A	0.25	0.2143	G

13	BICF2G630606253	13242525	A	1	1	C
13	BICF2G630606260	13249181	A	0.75	0.7857	C
13	BICF2G630606264	13251143	A	0.75	0.7857	G
13	BICF2G630606276	13260412	A	0.5	0.2143	G
13	BICF2G630606287	13276331	A	0.75	0.4286	G
13	BICF2P424681	13287200	A	0	0	G
13	BICF2G630606320	13294754	G	0.25	0.2143	A
13	BICF2G630606336	13307814	A	0.25	0.2143	G
13	BICF2G630606347	13324714	A	0.25	0.5714	G
13	BICF2G630606359	13352804	G	0.5	0.8333	A
13	BICF2P1236779	13377903	A	0.5	0.7857	G
13	BICF2S23020055	13389306	A	0	0	G
13	BICF2P1383332	13400345	C	0.25	0.5714	A
13	BICF2P334779	13415450	A	0	0	G
13	TIGRP2P174225_rs8579944	13435687	0	0	0	A
13	BICF2P1363793	13452641	A	0	0	C
13	BICF2P956276	13456639	A	0	0	G
13	BICF2G630606372	13489424	A	0	0	G
13	BICF2S22958961	13503950	A	0.5	0.2143	G
13	BICF2G630606394	13512854	A	0.5	0.5	G
13	BICF2G630606409	13524718	A	0	0.25	G
13	BICF2G630606422	13536438	G	0	0.2857	A
13	BICF2G630606441	13550478	C	0.25	0.5714	A
13	BICF2G630606469	13566203	G	0	0.2857	A
13	BICF2G630606472	13571358	G	0.5	0.5	A
13	TIGRP2P174239_rs9116345	13586198	T	0	0	A
13	TIGRP2P174243_rs8843400	13599964	0	0	0	G
13	BICF2S23136936	13605387	G	0.5	0.5	A
13	BICF2P1020075	13618345	G	0.5	0.5	A
13	TIGRP2P174250_rs8633052	13631864	A	0	0	G
13	TIGRP2P174264_rs9206823	13641989	G	0	0	A
13	G776f29S35	13678709	A	0	0	G
13	BICF2P1442923	13697463	0	0	0	A
13	TIGRP2P174297_rs9142603	13709367	0	0	0	A
13	BICF2S2355934	13728275	A	0	0	C
13	BICF2G630606490	13731432	A	0	0	C
13	BICF2G630606492	13752108	G	0.5	0.8333	A
13	BICF2G630606498	13756985	A	0.5	0.2143	C
13	BICF2P326286	13775407	A	0	0.2857	G
13	BICF2G630606511	13779935	G	0	0.2857	A
13	BICF2P676226	13798535	A	0	0.2857	G
13	BICF2S23529237	13810080	G	1	0.7143	C
13	BICF2G630606542	13818618	A	0	0.2857	G
13	BICF2G630606547	13824221	C	0	0.2857	G
13	BICF2G630606594	13837358	A	0	0.2857	G
13	BICF2G630606616	13863692	C	0	0.2857	A
13	BICF2P325539	13874328	0	0	0	A

13	BICF2G630606622	13891852	A	0.5	0.7857	G
13	BICF2G630606628	13900155	G	0	0	A
13	BICF2G630606636	13909066	A	0	0.2857	G
13	BICF2G630606651	13931753	A	0	0.2	G
13	BICF2P368108	13938384	G	0	0.2857	A
13	BICF2G630606664	13960971	A	0.5	0.5	T
13	BICF2P858215	13961864	G	0.5	0.5	C
13	BICF2G630606687	13973404	A	0.5	0.2143	G
13	BICF2G630606704	13988738	A	0	0.2857	G
13	BICF2G630606729	14015331	G	0.5	0.5	A
13	BICF2G630606737	14024771	A	0	0	G
13	BICF2G630606770	14049234	A	0	0	G
13	BICF2G630606785	14059619	0	0	0	A
13	BICF2G630606796	14064512	0	0	0	A
13	BICF2G630606810	14081168	0	0	0	A
13	BICF2P1514025	14099317	0	0	0	G
13	BICF2P1247499	14113322	0	0	0	A
13	BICF2G630606859	14128789	G	0	0	A
13	BICF2G630606863	14135020	0	0	0	G
13	BICF2G630606876	14151994	0	0	0	G
13	BICF2P230895	14167176	0	0	0	A
13	BICF2G630606904	14179191	G	0.5	0.5	A
13	BICF2G630606908	14179958	0	0	0	G
13	BICF2G630606944	14191249	0	0	0	A
13	BICF2G630606982	14209388	G	0.5	0.7857	A
13	BICF2P898605	14223345	A	0	0	G
13	BICF2G630606996	14226394	A	0	0	G
13	BICF2G630607005	14238645	A	0.5	0.2143	G
13	BICF2G630607017	14250439	G	0.5	0.2143	A
13	BICF2P1148982	14253835	A	0.5	0.1667	G
13	BICF2G630607050	14266777	A	0	0	G
13	BICF2G630607069	14277606	0	0	0	G
13	BICF2G630607086	14288091	0	0	0	T
13	BICF2G630607091	14299706	A	0.25	0.2857	G
13	BICF2G630607116	14311380	C	0.75	0.5	A
13	BICF2G630607142	14323318	G	0.75	0.5	A
13	BICF2G630607151	14329290	G	0.75	0.5	A
13	BICF2G630607194	14347057	A	0.25	0.2857	G
13	BICF2G630607200	14354781	G	0	0	A
13	BICF2G630607218	14369108	A	0.25	0.2857	G
13	BICF2G630607231	14376918	A	0.25	0.2857	C
13	BICF2P228043	14393771	G	0.25	0.2857	A
13	BICF2G630607271	14398386	G	0.75	0.5	A
13	BICF2G630607287	14411209	G	0	0.25	A
13	BICF2S2369397	14435575	A	0.5	0.2143	C
13	BICF2P1355194	14446907	A	0	0	C
13	BICF2P4530	14459237	A	0.5	0.7857	G

13	BICF2G630607304	14467359	G	0.5	0.7857	A
13	BICF2G630607354	14489502	A	0.5	0.7857	G
13	BICF2G630607357	14493610	G	0.5	0.7857	A
13	BICF2G630607374	14511705	G	0	0	A
13	BICF2G630607375	14513030	A	0.5	0.7857	C
13	BICF2G630607398	14534944	A	0	0	G
13	TIGRP2P174316_rs8961644	14549972	0	0	0	A
13	BICF2G630607415	14566531	G	0	0	A
13	BICF2S23118341	14588716	G	0.5	0.5	A
13	BICF2P73506	14596590	0	0	0	G
13	BICF2G630607426	14615683	A	0.25	0.2143	G
13	BICF2G630607427	14616577	G	0	0	A
13	BICF2G630607436	14628625	T	0.5	0.2143	A
13	BICF2G630607458	14645635	A	0.25	0.2143	C
13	BICF2S23056670	14662142	A	0.25	0.2143	G
13	BICF2P400464	14671959	A	0.25	0.2857	C
13	BICF2G630607518	14696748	A	0	0	C
13	BICF2G630607527	14709529	A	0.5	0.2143	G
13	BICF2G630607552	14720274	C	0	0.2857	A
13	BICF2G630607609	14736978	A	0.5	0.5	G
13	BICF2G630607626	14743952	A	0	0.2857	G
13	BICF2G630607662	14760373	A	0	0.2857	G
13	BICF2G630607693	14772700	G	0	0.2857	A
13	BICF2P642361	14778759	A	0	0.2857	G
13	BICF2G630607744	14794951	T	0	0.4	A
13	BICF2G630607750	14800659	A	0	0.25	G
13	BICF2G630607769	14815946	G	0	0.2857	A
13	BICF2G630607789	14827317	G	0	0	A
13	BICF2G630607798	14835520	T	0	0	A
13	TIGRP2P174336_rs9031519	14869761	0	0	0	G
13	BICF2G630607852	14906091	A	0	0	G
13	BICF2G630607881	14924690	T	0	0.2857	A
13	BICF2P258392	14929284	G	0.5	0.5	A
13	BICF2G630607911	14956874	A	0	0.2857	C
13	BICF2G630607915	14962441	A	0	0.2857	G
13	BICF2G630607935	14981614	G	0.75	0.7143	A
13	BICF2G630607941	14988044	G	0.75	0.7143	A
13	BICF2G630607957	15000685	A	0.25	0.5	G
13	TIGRP2P174343_rs9009012	15037033	0	0	0	A
13	TIGRP2P174344_rs9090504	15042799	0	0	0	A
13	BICF2P1290979	15058768	0	0	0	G
13	BICF2G630607990	15086018	A	0.5	0.5	G
13	BICF2G630607998	15092956	G	0	0.2857	A
13	BICF2G630608012	15099980	G	0.5	0.1667	A
13	BICF2G630608040	15114335	A	0	0	G
13	BICF2G630608052	15123308	G	0.5	0.2143	A
13	BICF2G630608084	15141777	G	0	0	A

13	TIGRP2P174348_rs9129973	15168356	0	0	0	A
13	BICF2G630608157	15191159	G	0.75	0.4286	A
13	BICF2P23129	15211017	G	0	0	A
13	BICF2P1416809	15234045	G	0	0	A
13	BICF2P559373	15237521	G	0	0	A
13	BICF2G630608186	15258797	G	0.5	0.2143	A
13	BICF2P427754	15269896	A	0.5	0.2143	G
13	BICF2G630608208	15272122	G	0	0	A
13	BICF2G630608219	15290837	0	0	0	G
13	BICF2P118227	15296286	C	0.25	0.2857	A
13	BICF2G630608229	15311125	0	0	0	G
13	BICF2P600658	15318280	A	0.75	0.4286	T
13	BICF2G630608259	15337568	G	0.25	0.2857	A
13	BICF2P107209	15347141	G	0.25	0.2857	A
13	BICF2G630608310	15357590	0	0	0	C
13	BICF2P682956	15395071	A	0	0	G
13	BICF2G630608384	15398985	A	0	0.2857	G
13	BICF2G630608421	15430893	C	0.5	0.2143	A
13	BICF2G630608425	15439408	G	0.5	0.2143	A
13	BICF2G630608449	15455111	G	0.5	0.2143	C
13	BICF2G630608453	15459715	G	0	0	A
13	BICF2P332251	15468430	G	1	0.75	A
13	BICF2G630608469	15478348	A	0	0	C
13	BICF2G630608470	15480799	A	0	0	G
13	BICF2G630608482	15491010	G	0	0	A
13	BICF2P569888	15505823	G	0.25	0.2143	A
13	BICF2P391669	15524588	G	0.25	0.5	A
13	BICF2S23429332	15525394	G	0.25	0.2143	A
13	BICF2G630608494	15543531	G	0.5	0.2143	A
13	BICF2G630608509	15552601	A	0.25	0.2857	G
13	BICF2G630608520	15562532	G	0.5	0.7857	A
13	BICF2G630608529	15578427	G	0.5	0.7857	A
13	BICF2G630608539	15585076	A	0	0.2857	C
13	BICF2G630608576	15618350	A	0.5	0.1667	G
13	BICF2S23229653	15630289	A	0.25	0.5	G
13	BICF2P1020953	15644885	A	0.75	0.5	G
13	BICF2P444802	15661056	G	0.75	0.5	A
13	BICF2P697433	15671311	A	0.5	0.7857	G
13	BICF2P706462	15674648	G	0.5	0.7857	A
13	BICF2P367260	15687598	A	0.5	0.2143	G
13	BICF2S23351493	15704100	G	0.25	0.5	A
13	BICF2S23314761	15716180	G	0.75	0.5	A
13	BICF2G630608587	15730678	C	0.5	0.2143	A
13	BICF2G630608590	15735480	A	0	0	G
13	BICF2P282286	15754838	C	0	0	A
13	BICF2P937959	15757670	A	0	0	G
13	BICF2P959871	15771226	A	0	0	G

13	BICF2G630608621	15791207	0	0	0	G
13	TIGRP2P174365_rs8992332	15801524	0	0	0	A
13	BICF2G630608635	15818506	0	0	0	G
13	BICF2G630608655	15828687	G	0.25	0.2857	A
13	BICF2G630608672	15838488	0	0	0	G
13	BICF2G630608681	15856528	A	0	0	G
13	BICF2S23426142	15874911	A	0	0	G
13	BICF2G630608775	15910189	G	0.25	0.2857	A
13	BICF2P1010731	15916307	C	0.25	0.2857	A
13	BICF2G630608803	15931300	A	0	0.2857	G
13	BICF2G630608825	15940101	C	0	0.2857	A
13	BICF2S23243248	15952032	A	0	0.2857	C
13	BICF2P1218213	15962115	C	0	0.2857	A
13	BICF2S23117025	15977923	A	0	0.2857	G
13	BICF2P1338587	15990756	G	0	0.2857	A
13	BICF2P1281143	15998218	G	0	0	A
13	BICF2P724747	16013330	C	0	0	A
13	BICF2S23447349	16028699	C	0	0	A
13	TIGRP2P174372_rs9146977	16041739	0	0	0	G
13	BICF2P458545	16053832	C	0	0.2857	A
13	BICF2P458542	16054696	A	0	0.2857	G
13	BICF2P927955	16072110	A	0	0.2857	G
13	BICF2P253177	16078813	C	0	0.25	A
13	BICF2G630608900	16104035	A	0	0	G
13	BICF2G630608907	16112969	A	0	0.2	C
13	BICF2P1137239	16127731	C	0.25	0.2143	A
13	BICF2P443346	16134695	A	0.5	0.5	G
13	BICF2G630608922	16147863	G	0	0	A
13	BICF2P1096460	16178746	A	1	1	G
13	BICF2P1034847	16184801	G	0	0	A
13	BICF2P1342383	16196451	G	0	0	A
13	BICF2G630608962	16212235	A	1	0.7143	G
13	BICF2G630608964	16216325	A	0	0	G
13	BICF2G630609005	16232640	A	0.25	0.2857	G
13	BICF2P691115	16241176	G	0.75	0.7143	A
13	BICF2G630609023	16250736	G	0.75	0.75	A
13	BICF2G630609041	16264888	C	0	0	A
13	BICF2P1156115	16281369	A	0	0	G
13	BICF2G630609048	16291310	G	0	0	A
13	BICF2P527722	16303034	A	0	0	G
13	BICF2G630609054	16308332	G	0	0	A
13	BICF2G630609069	16324952	A	0	0	C
13	BICF2G630609072	16334230	A	0	0	G
13	BICF2G630609083	16343990	A	0	0	G
13	BICF2G630609095	16357891	0	0	0	G
13	BICF2G630609098	16365807	A	0	0	G
13	BICF2P418166	16383882	A	0.5	0.2143	G

13	BICF2G630609105	16387682	A	0.5	0.2143	C
13	BICF2G630609117	16407916	C	0	0	A
13	BICF2G630609128	16418786	C	0	0	A
13	BICF2G630609131	16423609	A	0	0	G
13	BICF2P122442	16440965	G	0.5	0.2143	A
13	BICF2P1374319	16447547	T	0.5	0.2143	A
13	BICF2P324148	16470506	A	0.5	0.7857	G
13	BICF2P691752	16479993	A	0.5	0.5	C
13	BICF2P37931	16498449	C	1	0.7143	A
13	BICF2P592164	16504816	G	0.5	0.5	A
13	BICF2P1190101	16515963	A	1	0.7143	G
13	BICF2P576736	16528543	A	1	0.7143	C
13	BICF2P702755	16540980	A	1	0.7143	G
13	BICF2P659539	16550468	A	0.5	0.5	G
13	BICF2P201866	16570304	0	0	0	G
13	BICF2S23024538	16573431	A	0.5	0.5	G
13	BICF2P65609	16587028	0	0	0	G
13	BICF2G630609185	16604204	A	0	0	C
13	BICF2G630609187	16616126	A	0	0	G
13	BICF2G630609188	16632070	G	0.5	0.5	A
13	BICF2G630609206	16646750	A	0	0	G
13	BICF2G630609210	16653879	G	1	0.75	A
13	BICF2G630609218	16665562	G	0.5	0.5	A
13	BICF2G630609230	16678254	A	0	0	G
13	BICF2G630609238	16687463	G	0.5	0.2143	A
13	BICF2G630609247	16702300	A	0	0	G
13	BICF2G630609252	16713354	C	0.5	0.5	G
13	BICF2P916087	16727996	G	0	0	A
13	BICF2P1368819	16742602	A	0.5	0.2143	G
13	BICF2S23746346	16745557	A	0.5	0.2143	G
13	BICF2G630609294	16755517	A	0.5	0.2143	G
13	BICF2G630609308	16767183	A	0	0	G
13	BICF2G630609317	16780994	A	0.5	0.5	G
13	BICF2G630609336	16798626	A	0	0	G
13	BICF2G630609340	16807766	A	0	0	G
13	BICF2G630609347	16822501	A	0.5	0.7857	G
13	BICF2S23252574	16835923	A	0.5	0.7857	G
13	BICF2P1303880	16858375	G	0	0.2857	A
13	BICF2S2304146	16859084	A	0	0.2857	G
13	BICF2P709645	16893476	A	1	1	G
13	BICF2P566325	16911034	G	0	0.2857	A
13	BICF2G630609471	16924503	A	0.5	0.7857	C
13	BICF2G630609484	16930813	G	1	0.7143	A
13	BICF2G630609499	16948056	C	1	1	A
13	BICF2P177257	16956506	A	0.5	0.7857	G
13	BICF2S23310924	16968473	A	0	0	G
13	BICF2G630609534	16977695	G	0.5	0.7857	A

13	BICF2G630609547	16986654	G	0.5	0.2143	A
13	BICF2G630609566	17004861	A	0	0	G
13	BICF2G630609583	17009253	G	0	0	A
13	BICF2G630609616	17022988	A	0	0	C
13	BICF2G630609618	17031824	A	0	0	G
13	BICF2G630609629	17050439	A	0	0	G
13	BICF2P126512	17057745	A	0	0.2857	C
13	BICF2P199121	17069242	C	0	0.2857	A
13	BICF2P1394023	17079223	A	0	0	G
13	BICF2P592467	17098799	C	0.5	0.5	A
13	BICF2G630609638	17107860	A	0.5	0.5	G
13	BICF2G630609650	17119907	G	1	0.7143	C
13	BICF2G630609661	17126829	G	0.5	0.2143	A
13	BICF2G630609680	17135617	A	0	0.2857	G
13	BICF2G630609693	17152115	G	0.5	0.7857	A
13	BICF2G630609704	17169345	G	0.5	0.5	A
13	BICF2G630609705	17177648	A	0.5	0.5	C
13	BICF2G630609714	17183233	A	0.5	0.5	G
13	BICF2G630609720	17192865	A	1	0.7143	T
13	BICF2P1278590	17206716	A	0.5	0.5	G
13	BICF2P674390	17217545	A	0	0.2857	G
13	BICF2S23628247	17228680	T	0.5	0.5	A
13	BICF2P440772	17238503	C	0.5	0.5	A
13	BICF2S2451146	17254397	G	0.5	0.2143	A
13	BICF2G630609725	17281819	G	0	0	A
13	BICF2S23434059	17287625	A	0.5	0.1	G
13	BICF2S23728188	17320269	G	0.5	0.2143	A
13	BICF2G630609728	17331809	0	0	0	G
13	BICF2P221691	17375197	G	1	1	A
13	BICF2G630609738	17382563	A	1	1	C
13	BICF2G630609742	17390354	C	0	0	A
13	BICF2G630609767	17406396	G	0.5	0.2143	A
13	BICF2G630609771	17413915	G	0.5	0.2143	A
13	BICF2G630609780	17431857	A	0.5	0.2143	G
13	BICF2P573695	17441598	A	0.5	0.2143	G
13	TIGRP2P174400_rs8882081	17452625	0	0	0	A
13	BICF2G630609796	17460489	C	0	0	A
13	BICF2G630609803	17472480	G	0	0	A
13	BICF2G630609838	17482304	A	0	0	G
13	BICF2G630609840	17499527	A	0	0	G
13	BICF2P1374610	17511274	T	0	0	A
13	BICF2P137534	17517516	0	0	0	A
13	BICF2S23761384	17541770	C	0.5	0.2143	A
13	BICF2P760434	17554950	A	0.5	0.7857	G
13	BICF2P1159742	17562554	A	0	0	G
13	BICF2P693726	17575194	T	0	0.2857	A
13	BICF2P487470	17587486	A	0	0.2857	T

13	BICF2S2447015	17606355	0	0	0	T
13	BICF2G630609849	17616813	A	0	0.2857	G
13	BICF2G630609859	17628548	A	0.5	0.5	G
13	BICF2G630609888	17636233	A	0.5	0.5	G
13	BICF2P109462	17645457	0	0	0	A
13	BICF2G630609928	17661686	A	0	0.2857	T
13	BICF2S2432564	17666338	C	0.5	0.5	A
13	BICF2G630609937	17679064	A	0	0.2857	G
13	BICF2G630609968	17692957	A	0	0.2857	G
13	BICF2G630609980	17698811	C	0	0	A
13	BICF2G630610018	17711775	G	0	0	A
13	BICF2P659763	17724702	A	0	0.2857	G
13	BICF2G630610034	17736261	A	0.5	0.2143	G
13	BICF2S2414989	17763039	0	0	0	A
13	TIGRP2P174402_rs8542477	17768859	0	0	0	G
13	BICF2P347506	17795829	0	0	0	G
13	BICF2P221270	17804651	G	0.5	0.5	A
13	BICF2P1468218	17819592	A	0.5	0.5	G
13	BICF2P1468219	17819909	A	0.5	0.5	G
13	BICF2P795796	17827695	G	0.5	0.5	A
13	BICF2P795795	17827925	T	0.5	0.5	A
13	BICF2S23320535	17868449	G	0.5	0.5	A
13	BICF2P440915	17875681	G	0.5	0.5	A
13	BICF2P1306822	17883835	G	0.5	0.5	A
13	BICF2G630610050	17901965	A	0	0	G
13	BICF2P436849	17910801	G	0.5	0.5	A
13	BICF2G630610053	17918792	0	0	0	A
13	BICF2P1001247	17930038	A	0.5	0.5	G
13	BICF2P336900	17946556	G	0.5	0.5	A
13	BICF2P1247872	17959985	C	0.5	0.5	A
13	BICF2P721572	17965606	A	0.5	0.5	G
13	BICF2P95378	17966017	G	0	0.2857	A
13	BICF2G630610055	17976673	0	0	0	G
13	BICF2P1308303	17992322	A	0.5	0.5	G
13	BICF2G630610057	18002076	0	0	0	G
13	BICF2P535652	18011429	G	0.5	0.5	A
13	BICF2P1145308	18020832	G	0.5	0.5	C
13	BICF2P274251	18038511	A	0.5	0.5	G
13	BICF2P737500	18047801	G	0	0	A
13	TIGRP2P174408_rs9097400	18056193	0	0	0	C
13	BICF2G630610094	18074753	A	0	0	G
13	BICF2G630610097	18079579	A	0.5	0.5	G
13	BICF2G630610101	18093370	A	0	0	G
13	BICF2P570629	18104940	A	1	0.7143	C
13	BICF2G630610103	18118401	G	0	0	A
13	BICF2G630610114	18126197	C	0	0	A
13	BICF2P681073	18137327	A	0.5	0.2143	G

13	BICF2S22933732	18151469	G	0	0	A
13	BICF2G630610130	18162128	A	0	0	C
13	BICF2G630610143	18171797	G	0.5	0.7857	A
13	BICF2S23740057	18183940	G	0.5	0.7857	A
13	BICF2P585973	18198633	A	0.5	0.2143	G
13	BICF2P999805	18204568	A	0.5	0.2143	G
13	BICF2P1124215	18220505	A	0.5	0.2143	G
13	BICF2P1156779	18229058	A	0.5	0.2143	G
13	BICF2P1100337	18250593	A	1	0.7143	G
13	BICF2S22947617	18263993	A	0	0	C
13	BICF2S23513111	18284485	A	0	0	G
13	BICF2P140672	18295953	0	0	0	A
13	BICF2S23728635	18304264	G	0.5	0.5	A
13	BICF2P560437	18317514	A	0.25	0.3571	G
13	BICF2P761116	18327967	A	0.25	0.3571	G
13	BICF2P1044750	18333447	A	0.25	0.1429	C
13	BICF2P378783	18344146	G	0.25	0.3571	A
13	BICF2P496834	18367880	A	0.25	0.3571	G
13	BICF2S22934651	18376372	A	0	0.2857	G
13	BICF2P666259	18384664	A	0.75	0.3571	G
13	BICF2P1343519	18389026	G	0.75	0.3571	A
13	BICF2P987185	18408979	A	0.25	0.3571	G
13	BICF2P1157596	18414326	C	0.25	0.3571	A
13	BICF2P197597	18427769	G	0.5	0.5	A
13	BICF2P579556	18435845	0	0	0	G
13	BICF2P413719	18451783	A	0.25	0.5	G
13	BICF2P404671	18462961	A	0.5	0.5	G
13	BICF2S23613764	18471300	G	0.5	0.2143	A
13	BICF2S2362259	18480894	A	0.25	0.1429	G
13	BICF2P682740	18499006	A	0.75	0.8571	G
13	BICF2P1311798	18507470	G	0	0	A
13	BICF2P1313740	18518467	A	0	0	C
13	BICF2P39358	18529754	C	0.75	0.8571	G
13	BICF2P177786	18544009	A	0.25	0.1429	G
13	BICF2P437154	18553964	G	0.25	0.6429	A
13	BICF2P765861	18562825	A	0.25	0.1429	C
13	BICF2P459552	18576007	G	0	0	A
13	BICF2P457238	18584059	C	0	0	A
13	BICF2P346815	18617321	A	0.25	0.3571	G
13	BICF2P180346	18625655	0	0	0	A
13	BICF2S23131872	18636565	0	0	0	G
13	BICF2P307408	18650571	G	0.25	0.1429	C
13	BICF2P915111	18664748	A	0.25	0.1429	C
13	BICF2P499296	18694473	G	0.5	0.5	A
13	BICF2P1204033	18710127	A	0.25	0.1429	G
13	BICF2P1069978	18717836	A	0	0	G
13	BICF2S23130494	18732796	G	1	1	A

13	BICF2P396107	18739269	G	0	0	A
13	BICF2P556893	18766767	A	0.5	0	G
13	BICF2P679956	18772740	G	0	0	A
13	BICF2P53570	18780043	A	0	0	C
13	BICF2P609661	18793283	A	0	0	G
13	BICF2P261248	18808381	G	0.75	0.3571	A
13	BICF2P450200	18815623	A	0	0.2857	G
13	BICF2P69056	18836740	G	0	0.2857	A
13	BICF2P83527	18845562	G	0.5	0.5	A
13	BICF2P848521	18851825	G	0	0.2857	A
13	BICF2P853971	18864071	A	0	0.2857	G
13	BICF2P322024	18876653	0	0	0	A
13	BICF2P395257	18883643	G	0.75	0.3571	A
13	BICF2P1118531	18896561	A	0.25	0.6429	G
13	BICF2P1084236	18913036	G	0.75	0.3571	A
13	BICF2P928946	18919846	G	1	0.7143	A
13	BICF2P350561	18932436	G	1	0.6429	A
13	TIGRP2P174420_rs8637308	18955358	0	0	0	G
13	BICF2S23246911	18968333	A	0	0	G
13	BICF2S23621394	18976617	G	0.25	0.4286	A
13	BICF2S23219249	18987345	G	0	0	A
13	BICF2S23230380	19006877	A	0.5	0.5	G
13	BICF2S22932096	19012270	T	0.25	0.1429	A
13	BICF2P527059	19029069	0	0	0	G
13	BICF2S23055854	19043322	A	0	0.6429	G
13	BICF2S23256346	19059178	G	0.25	0.6429	A
13	BICF2S23752412	19070524	G	0	0	A
13	BICF2S23554801	19087860	G	0	0	A
13	BICF2S23749132	19097141	A	0	0	G
13	BICF2P274129	19139368	A	0	0	G
13	BICF2P1320052	19151262	0	0	0	G
13	BICF2S2332757	19161015	0	0	0	A
13	G777f43S170	19190861	A	0	0	T
13	BICF2S2443419	19194684	A	0	0	T
13	BICF2P126305	19214438	A	0	0	C
13	BICF2P273547	19223333	A	0	0	G
13	BICF2S22913717	19237875	A	0	0	G
13	BICF2P1177586	19267685	0	0	0	C
13	BICF2S23059689	19274344	A	0	0.2857	G
13	BICF2S23129016	19303382	A	0	0.2857	C
13	BICF2P576358	19312371	A	0.5	0.5	G
13	BICF2P207238	19326783	A	0.25	0.1429	T
13	BICF2P585538	19333729	A	0.25	0.3571	G
13	BICF2P585537	19333746	C	0.25	0.3571	A
13	BICF2P1500968	19343569	A	0	0	G
13	BICF2P741530	19374933	C	0	0	A
13	BICF2P1379336	19384104	G	0.5	0.5	A

13	BICF2P506219	19391817	G	0	0	A
13	BICF2P1315123	19401356	A	0	0	G
13	BICF2P627166	19420092	G	0.75	0.5714	A
13	BICF2S23736283	19434513	G	0.75	0.5714	A
13	BICF2P496480	19439903	G	0.75	0.5714	C
13	BICF2P913233	19455021	G	0.25	0.3571	A
13	BICF2P778772	19467108	0	0	0	G
13	BICF2S24113536	19480977	G	0.75	0.3571	A
13	BICF2P1251882	19486500	A	0	0	G
13	TIGRP2P174425_rs8616030	19500318	0	0	0	C
13	BICF2S23911667	19509224	0	0	0	A
13	BICF2S23230209	19518846	G	0	0	A
13	BICF2S23315732	19535045	A	0	0.2857	G
13	BICF2S23150337	19545796	0	0	0	C
13	BICF2S23715908	19556533	A	0.25	0.3571	G
13	BICF2P890114	19565866	A	0.25	0.3571	G
13	BICF2P175098	19571985	A	0	0	G
13	BICF2P1435582	19575507	A	0.25	0.3571	C
13	BICF2S23210158	19606225	A	0	0	G
13	BICF2P427938	19609707	A	0.25	0.3571	G
13	BICF2S23531023	19620065	G	0	0	A
13	BICF2S23345034	19643114	G	0	0	A
13	BICF2G630610200	19654218	A	1	1	G
13	BICF2S23546371	19673746	0	0	0	C
13	BICF2S23546372	19674054	A	0.25	0.3571	C
13	BICF2S23545451	19677764	G	0	0	A
13	BICF2G630610240	19692694	A	0.5	0.5	G
13	BICF2G630610253	19700476	G	0.5	0.5	A
13	BICF2G630610280	19711127	A	0.5	0.5	G
13	BICF2G630610289	19725707	A	0.25	0.1429	G
13	BICF2G630610292	19741416	A	0	0	G
13	BICF2G630610300	19748127	G	0.25	0.1429	A
13	BICF2G630610317	19761440	G	0.75	0.5714	A
13	BICF2G630610329	19779490	C	0	0	A
13	BICF2G630610333	19780378	A	0.75	0.6429	G
13	BICF2P398473	19799701	A	0	0	G
13	BICF2P18222	19813045	A	0.25	0.1429	G
13	BICF2P1069482	19824023	G	0	0	A
13	BICF2P1252994	19828942	G	0.5	0.5	A
13	BICF2G630610354	19848981	A	0.25	0.6429	G
13	BICF2G630610359	19853216	A	0	0.2857	G
13	BICF2G630610364	19856825	A	0	0	G
13	BICF2G630610381	19860629	G	0.25	0.1429	A
13	BICF2S229644	19878437	A	0.75	0.8571	G
13	BICF2G630610424	19891764	G	0.5	0.5	A
13	BICF2P151837	19904793	A	0.25	0.3571	G
13	BICF2G630610441	19913472	C	0	0	A

13	BICF2G630610450	19918920	A	0	0	G
13	BICF2G630610460	19929672	G	0	0	A
13	BICF2G630610477	19943254	A	0.25	0.1429	G
13	BICF2G630610500	19953027	G	0	0	A
13	BICF2G630610518	19969551	C	0.25	0.4286	A
13	BICF2G630610531	19983557	A	0.25	0.6429	C
13	BICF2G630610536	19987796	A	0.25	0.3571	G
13	BICF2P482834	19994508	A	0.75	0.8571	G
13	BICF2G630610553	19998930	A	0	0	G
13	BICF2G630610560	20000638	A	0.25	0.6429	G
13	BICF2G630610575	20009395	G	0	0.25	A
13	BICF2G630610590	20015218	A	0.25	0.3571	G
13	BICF2S22955808	20022396	A	1	0.7143	G
13	BICF2S22939671	20033108	G	0	0	A
13	BICF2G630610623	20048615	A	0	0	G
13	BICF2G630610629	20058553	A	0	0	C
13	BICF2P703448	20078748	G	0.75	0.5714	A
13	BICF2P1359045	20083268	A	0	0.2857	G
13	BICF2G630610665	20092304	0	0	0	A
13	BICF2P691005	20110405	A	0	0	G
13	BICF2P273182	20117734	G	0.5	0.5	A
13	BICF2P377210	20128222	A	0.5	0.7857	G
13	BICF2G630610679	20145323	G	0.5	0.5	A
13	BICF2G630610683	20148373	G	0	0	A
13	BICF2G630610708	20163351	A	0	0	G
13	BICF2G630610720	20171291	A	0.5	0.5	T
13	BICF2G630610768	20192157	A	0.5	0.2143	G
13	BICF2P224700	20206060	0	0	0	A
13	BICF2G630610809	20221481	A	0	0.2857	G
13	BICF2G630610812	20228650	A	0	0	G
13	BICF2P1365514	20251246	A	0.5	0.2143	G
13	BICF2P1365515	20251329	A	0.5	0.25	G
13	BICF2P565100	20260365	C	1	0.7143	G
13	BICF2P243383	20262832	A	0	0	G
13	BICF2G630610813	20269668	G	0.5	0.5	A
13	BICF2G630610832	20279974	G	0.5	0.5	A
13	BICF2P554457	20296318	0	0	0	G
13	BICF2G630610849	20301899	A	0	0	G
13	BICF2G630610856	20309614	A	0	0	T
13	BICF2S23251777	20320963	A	0.5	0.25	G
13	BICF2G630610897	20329366	T	0	0.2857	A
13	TIGRP2P174437_rs8650744	20335265	0	0	0	G
13	BICF2P1359240	20348960	A	0	0	G
13	BICF2G630610951	20363145	T	0	0.2857	A
13	BICF2P593689	20368945	G	0	0.2857	A
13	BICF2G630610959	20378488	G	0	0.2857	A
13	BICF2G630610975	20391078	A	0	0	G

13	BICF2G630610992	20401311	A	0.5	0.5	G
13	BICF2P1395484	20420742	0	0	0	G
13	BICF2P827483	20432240	A	0	0	C
13	BICF2G630611010	20444003	0	0	0	G
13	BICF2G630611011	20447630	A	0	0.2857	G
13	BICF2G630611029	20462315	G	0	0	A
13	BICF2G630611045	20470689	A	1	1	G
13	BICF2G630611050	20483279	C	0	0	A
13	BICF2G630611062	20503588	G	0.5	0.5	A
13	BICF2G630611069	20510871	A	0.5	0.5	G
13	BICF2G630611082	20516687	C	0.5	0.5	A
13	BICF2G630611091	20529588	A	0.5	0.5	G
13	BICF2G630611109	20544719	T	0	0	A
13	BICF2G630611119	20554900	A	0.25	0.1429	G
13	BICF2G630611127	20620567	T	0	0	A
13	BICF2S23351295	20635148	G	0	0	A
13	BICF2S22964966	20658362	A	0	0.2857	G
13	TIGRP2P174439_rs9084035	20681921	0	0	0	A
13	BICF2G630611131	20695492	G	0	0.2857	A
13	BICF2G630611144	20700683	G	0	0	A
13	BICF2P1266698	20730558	0	0	0	C
13	BICF2G630611147	20738953	G	0	0	A
13	BICF2P844990	20747865	0	0	0	A
13	TIGRP2P174440_rs8766889	20767630	0	0	0	G
13	BICF2P901437	20775311	0	0	0	G
13	BICF2S22915548	20780848	T	0.5	0.5	A
13	BICF2S23520084	20815660	G	1	0.7143	A
13	BICF2P704668	20829572	G	0.5	0.5	A
13	BICF2P144958	20839511	0	0	0	G
13	BICF2G630611151	20855959	A	0	0	C
13	BICF2P143191	20862993	0	0	0	C
13	BICF2G630611162	20903036	A	0	0	C
13	BICF2G630611171	20912185	A	0	0	G
13	BICF2G630611184	20918701	A	0	0	G
13	BICF2G630611196	20939967	C	0	0	A
13	BICF2G630611201	20948752	A	0	0	C
13	BICF2G630611209	20958481	A	0	0	T
13	BICF2G630611231	20977731	G	0	0	A
13	BICF2G630611238	20990338	A	0	0.2857	G
13	BICF2G630611243	21009137	C	0	0.2857	A
13	BICF2G630611254	21020433	A	0	0.25	G
13	BICF2G630611260	21024629	G	0	0.2857	A
13	BICF2G630611262	21025686	A	0	0.2857	G
13	BICF2G630611283	21034089	A	0	0	C
13	BICF2P351176	21046665	A	0	0	G
13	BICF2P1269511	21058930	A	0	0	G
13	BICF2P648415	21069059	A	0	0	G

13	TIGRP2P174442_rs8885629	21073466	0	0	0	G
13	BICF2G630611294	21086465	C	0.5	0.5	G
13	BICF2G630611295	21086488	C	0.5	0.5	G
13	BICF2P922758	21098347	G	0	0	A
13	BICF2G630611308	21103428	G	0.5	0.5	A
13	BICF2G630611327	21116818	C	0.5	0.5	A
13	BICF2G630611365	21129734	A	0.5	0.5	G
13	BICF2G630611388	21147259	A	0.5	0.5	C
13	BICF2P710746	21151217	G	0	0	C
13	BICF2G630611412	21170459	A	0	0	G
13	BICF2G630611413	21171455	A	0.5	0.7857	G
13	BICF2G630611415	21175614	A	0	0	G
13	BICF2G630611429	21183942	G	0.5	0.5	A
13	BICF2S2318817	21196203	0	0	0	T
13	BICF2G630611433	21209024	C	0.5	0.2143	G
13	BICF2G630611454	21235491	A	0.5	0.5	T
13	BICF2G630611474	21262596	A	0	0	G
13	BICF2P603082	21267883	G	0	0	A
13	BICF2G630611493	21277016	A	0.5	0.7857	G
13	BICF2G630611512	21291323	A	0	0.2857	G
13	BICF2G630611520	21300363	0	0	0	A
13	BICF2G630611546	21315435	G	1	1	C
13	BICF2G630611562	21323793	A	0	0	G
13	BICF2S23627016	21333261	G	0.5	0.5	A
13	BICF2G630611570	21349013	A	0.5	0.1667	C
13	BICF2G630611599	21358202	C	0.5	0.8333	A
13	BICF2G630611610	21363293	G	0.5	0.2143	A
13	BICF2G630611616	21371599	A	0.5	0.2143	G
13	BICF2G630611629	21379696	A	0.5	0.2143	C
13	BICF2G630611642	21394112	0	0	0	G
13	BICF2G630611681	21408285	0	0	0	G
13	BICF2G630611709	21415002	0	0	0	G
13	BICF2G630611730	21428820	A	0.5	0.2143	G
13	BICF2G630611739	21445990	A	0	0.2857	G
13	BICF2S2323116	21468228	C	0.5	0.7857	A
13	BICF2G630611756	21480231	A	0	0	C
13	BICF2P188824	21485653	A	0.5	0.5	T
13	BICF2G630611781	21496018	C	0.5	0.5	A
13	BICF2P823456	21512109	A	0	0	G
13	BICF2G630611822	21517868	A	0	0	G
13	BICF2G630611833	21529064	A	0	0	G
13	BICF2G630611849	21544303	G	0	0	A
13	BICF2G630611872	21552883	A	0	0	G
13	BICF2P353630	21564309	A	0.5	0.5	G
13	BICF2P569328	21582897	0	0	0	A
13	BICF2G630611906	21592894	0	0	0	A
13	BICF2G630611925	21600902	A	0.5	0.5	G

13	BICF2G630611940	21610806	C	0.5	0.5	A
13	BICF2P918734	21623410	0	0	0	G
13	BICF2P722085	21633777	0	0	0	G
13	BICF2G630611947	21658633	G	0.5	0.5	A
13	BICF2G630611955	21669509	G	0.5	0.5	A
13	BICF2S23724919	21682776	G	0	0	A
13	BICF2G630611964	21690023	A	0.5	0.2143	G
13	BICF2G630611973	21701920	A	0.5	0.5	G
13	BICF2P1124540	21720405	0	0	0	A
13	BICF2G630611985	21731588	C	0	0	G
13	BICF2G630611986	21735982	C	0	0	A
13	BICF2G630611996	21746782	A	0	0	G
13	BICF2G630612004	21761195	G	0	0	A
13	BICF2S23617374	21774917	A	0	0	G
13	BICF2G630612008	21787930	G	0	0	C
13	BICF2G630612009	21793415	A	0	0	G
13	BICF2S23247830	21835678	0	0	0	A
13	BICF2G630612018	21860775	G	0.5	0.5	A
13	BICF2G630612023	21865955	0	0	0	A
13	BICF2P216637	21874537	A	0	0	G
13	BICF2G630612032	21888871	G	0.5	0.5	A
13	TIGRP2P174461_rs8766024	21900817	0	0	0	G
13	BICF2G630612038	21918933	G	0.5	0.5	A
13	BICF2P250140	21931733	G	0.5	0.5	A
13	BICF2P650729	21944848	A	0.5	0.5	G
13	BICF2P875338	21948337	A	0.5	0.5	G
13	BICF2P433902	21978155	G	1	1	A
13	BICF2P930468	21990473	A	0	0	G
13	BICF2P315011	22006912	A	0.5	0.5	G
13	BICF2G630612057	22012935	G	1	0.7143	A
13	BICF2P249331	22033792	T	0.5	0.2143	A
13	BICF2S2304131	22051720	G	0	0	A
13	BICF2S23132005	22084397	A	0	0	G
13	BICF2P605779	22099644	G	0	0	A
13	BICF2P1159074	22104302	A	0.5	0.5	G
13	BICF2S22939888	22122823	A	0	0	G
13	BICF2P824782	22126307	C	0	0.2857	G
13	BICF2G630612150	22141614	G	1	1	A
13	BICF2P673369	22149713	G	0	0	A
13	BICF2S22917229	22161430	G	0.5	0.2143	A
13	BICF2S23652089	22178767	0	0	0	G
13	BICF2S23652088	22178823	A	0	0	G
13	BICF2G630612153	22215591	G	0	0	C
13	BICF2G630612158	22222851	A	0.5	0.2143	G
13	BICF2G630612189	22235859	G	NA	0.1667	A
13	BICF2P179778	22237963	C	0.5	0.2143	G
13	BICF2G630612204	22243767	A	0.5	0.2143	G

13	BICF2G630612205	22254964	A	0	0	C
13	BICF2G630612206	22268402	A	0	0.2857	G
13	BICF2G630612228	22283991	A	0.5	0.5	T
13	BICF2G630612234	22287835	A	0.5	0.5	G
13	BICF2P300805	22301274	A	0.5	0.5	G
13	BICF2P190854	22316554	0	0	0	A
13	BICF2S23726680	22326464	G	0	0.2857	A
13	BICF2S23243046	22335015	0	0	0	G
13	BICF2G630612263	22349569	0	0	0	A
13	BICF2G630612284	22360484	G	0.5	0.2143	A
13	BICF2G630612298	22370643	G	0	0	A
13	BICF2G630612317	22384679	G	0	0	A
13	BICF2G630612329	22392251	A	0	0	G
13	BICF2G630612374	22405594	A	0	0	G
13	BICF2P124157	22419744	G	0.5	0.1	A
13	BICF2G630612396	22433373	A	0	0.2857	G
13	BICF2G630612403	22444739	G	0.5	0.2143	A
13	BICF2P118144	22455824	G	0	0	A
13	BICF2P1012495	22467136	C	0	0.2857	A
13	BICF2G630612428	22467697	G	1	0.7143	A
13	BICF2G630612478	22493428	A	0	0	G
13	BICF2G630612482	22501891	A	0.5	0.2143	G
13	BICF2P1023987	22510310	G	0	0	A
13	BICF2G630612500	22518351	A	0.5	0.7857	G
13	BICF2G630612508	22534019	A	0	0	C
13	BICF2G630612519	22540553	A	0.5	0.2143	C
13	BICF2G630612544	22557228	G	0	0	A
13	BICF2G630612554	22570265	A	0	0	G
13	BICF2G630612561	22574997	G	0	0	A
13	BICF2G630612583	22586357	A	0	0.2857	G
13	BICF2G630612603	22605693	A	0	0	G
13	BICF2P951355	22626376	A	0	0	C
13	BICF2G630612615	22626839	G	0.5	0.5	A
13	BICF2P1313620	22640389	G	0	0.2857	C
13	BICF2G630612627	22644943	G	0.5	0.5	A
13	BICF2P497447	22664633	A	0	0	C
13	BICF2S23259132	22666702	A	0	0.2857	C
13	BICF2G630612637	22683322	G	0	0	A
13	BICF2G630612645	22688041	A	0	0.2857	G
13	BICF2P1052982	22691061	A	0	0.2857	G
13	BICF2G630612667	22708136	G	0	0	A
13	BICF2G630612690	22714370	A	0	0	G
13	BICF2G630612711	22731044	G	0	0	A
13	BICF2G630612713	22739650	C	0	0	A
13	BICF2S23054517	22748362	C	0	0	A
13	BICF2G630612744	22765682	G	1	0.7143	A
13	BICF2G630612759	22772085	G	0	0	A

13	BICF2G630612807	22792867	A	0.5	0.2143	G
13	BICF2P532039	22804395	A	0	0	G
13	BICF2G630612827	22807331	A	0	0	G
13	BICF2G630612840	22821977	T	0	0	A
13	BICF2G630612848	22834937	G	0	0	A
13	BICF2G630612850	22839394	A	0	0	G
13	BICF2G630612866	22854585	G	0	0.2857	A
13	TIGRP2P174470_rs9054086	22866145	0	0	0	G
13	BICF2P362172	22881338	A	0	0	C
13	BICF2G630612923	22892752	A	0	0	G
13	BICF2S23353450	22902533	G	0	0	A
13	BICF2G630612931	22916452	G	0	0	A
13	BICF2S23121112	22937592	A	0.5	0.2143	C
13	BICF2G630612971	22944477	A	0	0	G
13	BICF2G630613003	22958946	A	0	0	C
13	BICF2G630613004	22959881	A	0.5	0.2143	G
13	BICF2G630613015	22966236	G	0.5	0.5	A
13	BICF2P1145781	22987780	A	0	0.2857	G
13	BICF2S2318454	22991514	A	0.5	0.7857	G
13	BICF2P240664	23004428	G	0.5	0.2143	A
13	BICF2P525040	23022021	A	0.5	0.2143	G
13	BICF2P648379	23023126	G	0	0	A
13	BICF2S23444219	23039341	A	0.5	0.2143	G
13	BICF2P1434455	23047599	C	0.5	0.1667	A
13	BICF2P345543	23064990	0	0	0	G
13	BICF2P1092492	23077277	A	0.5	0.5	T
13	BICF2G630613029	23083271	0	0	0	G
13	BICF2P1430436	23099879	G	0	0	A
13	BICF2G630613069	23112844	A	0	0	G
13	BICF2G630613079	23117377	A	0	0	G
13	BICF2G630613095	23127535	C	0.5	0.5	A
13	BICF2S23314230	23139331	A	0	0	G
13	BICF2G630613136	23155124	A	0	0	G
13	BICF2G630613174	23180227	A	0.5	0.5	C
13	BICF2P426728	23189049	G	0	0	A
13	BICF2G630613185	23199800	G	0	0	A
13	BICF2G630613189	23207208	A	0	0.2857	G
13	BICF2G630613220	23222643	G	0	0.2857	A
13	BICF2G630613224	23226727	C	0.5	0.2143	G
13	BICF2G630613237	23229736	A	0.5	0.5	G
13	BICF2G630613246	23235401	G	0	0	A
13	BICF2G630613258	23243268	A	0	0	G
13	BICF2G630613264	23253081	A	0	0.2857	G
13	BICF2S23036194	23273326	A	0	0	G
13	BICF2G630613289	23280036	G	0	0	A
13	BICF2G630613309	23288808	A	0.5	0.2143	C
13	BICF2P919738	23307300	A	0	0	G

13	BICF2G630613333	23309553	A	0.5	0.5	T
13	BICF2G630613336	23310527	A	0.5	0.5	G
13	BICF2P157512	23335059	T	0.5	0.5	A
13	BICF2G630613406	23347050	A	1	0.7143	G
13	BICF2G630613414	23359042	A	0.5	0.5	G
13	BICF2G630613421	23369395	A	0.5	0.7857	G
13	BICF2P704096	23383166	0	0	0	G
13	TIGRP2P174484_rs8599758	23403586	0	0	0	G
13	BICF2P797319	23424206	A	0	0.2857	G
13	BICF2P942108	23430873	0	0	0	G
13	BICF2P1054927	23444057	A	0	0	G
13	BICF2G630613435	23454048	0	0	0	G
13	BICF2G630613447	23468605	A	0	0	C
13	BICF2P468535	23472088	0	0	0	G
13	BICF2G630613461	23474680	A	0	0	G
13	BICF2P920416	23476966	A	0	0	G
13	BICF2G630613491	23487992	G	0.5	0.1667	A
13	BICF2S23713956	23506853	G	0.5	0.2143	A
13	BICF2S23157428	23522295	A	0.5	0.5	G
13	BICF2G630613555	23530710	A	0.5	0.2143	G
13	BICF2G630613573	23546872	A	0	0	G
13	BICF2G630613587	23554454	A	0	0	C
13	BICF2G630613592	23564925	A	0.5	0.5	G
13	BICF2G630613631	23583164	G	0.5	0.7857	C
13	BICF2G630613639	23587294	A	0.5	0.7857	G
13	BICF2G630613668	23601222	A	1	0.7143	G
13	BICF2P388351	23611291	G	0	0.2857	A
13	BICF2P772226	23623431	A	0.5	0.7857	G
13	BICF2S23117687	23643526	A	0	0	G
13	BICF2G630613686	23645755	A	0.5	0.5	C
13	BICF2G630613700	23656564	0	0	0	G
13	BICF2G630613717	23668972	G	0	0	A
13	BICF2G630613734	23680032	A	0	0.2857	G
13	BICF2G630613749	23700368	C	0	0	A
13	BICF2G630613761	23706001	G	0	0	C
13	BICF2G630613763	23706280	A	0	0	T
13	BICF2S23659924	23715468	A	0	0	G
13	BICF2S23657300	23730713	G	0.5	0.5	A
13	BICF2G630613794	23739360	A	0.5	0.2143	T
13	BICF2G630613797	23750516	A	0	0.2857	G
13	BICF2G630613807	23764514	A	0.5	0.5	C
13	BICF2G630613812	23771542	A	0	0	G
13	BICF2G630613823	23789037	G	0	0	A
13	BICF2G630613846	23799573	A	0.5	0.5	G
13	BICF2G630613851	23806062	0	0	0	G
13	BICF2P257816	23827729	0	0	0	G
13	BICF2G630613852	23833687	0	0	0	G

13	BICF2S22924915	23845380	A	0.5	0.5	G
13	BICF2P492898	23852256	G	0.5	0.5	A
13	BICF2P932753	23855195	G	0.5	0.2143	A
13	BICF2P932755	23855429	A	0.5	0.2143	G
13	BICF2P656947	23875332	A	0.5	0.5	C
13	BICF2S2294688	23893661	A	0.5	0.2143	G
13	BICF2S2393998	23929632	G	0	0	A
13	BICF2S23240444	23932746	A	0.5	0.7857	G
13	BICF2S244264	23946082	A	0	0	G
13	BICF2P251097	23966385	C	0.5	0.5	A
13	BICF2P115217	23971099	A	0.5	0.5	C
13	BICF2G630613871	23979488	G	0.5	0.2143	A
13	BICF2P5930	23997731	G	0	0	A
13	BICF2G630613900	24002346	G	0.5	0.5	A
13	BICF2G630613914	24012946	G	0.5	0.5	A
13	BICF2P139829	24032178	A	0	0	C
13	BICF2P1202222	24035518	C	0	0	A
13	BICF2P976002	24054567	G	0.5	0.5	C
13	BICF2G630613951	24065031	A	0.5	0.5	G
13	BICF2G630613963	24069849	C	0.5	0.5	A
13	BICF2G630613973	24081275	A	0.5	0.5	G
13	BICF2P299136	24099313	G	0	0	A
13	BICF2P1179347	24112364	G	0.5	0.5	A
13	BICF2P1253901	24118124	G	0	0	A
13	BICF2P1209414	24134521	A	0	0.2	C
13	BICF2S22925645	24141747	A	0	0	G
13	BICF2P545026	24150791	A	0	0	C
13	BICF2P1355985	24167569	A	0.5	0.5	G
13	BICF2P1355983	24167948	C	0	0	A
13	BICF2S23036062	24177097	A	0	0.2857	G
13	BICF2S23115583	24192864	A	0	0	G
13	BICF2S23658277	24194764	A	0.5	0.5	C
13	BICF2S2347915	24200752	G	0	0.2857	A
13	BICF2P1188454	24210601	A	0	0	G
13	BICF2P259985	24225970	C	0	0	A
13	BICF2P1259600	24239199	G	0	0	A
13	BICF2S2346590	24248892	G	0	0	A
13	BICF2S23719999	24258336	A	0	0.2857	G
13	BICF2P974023	24266877	G	0.5	0.2143	A
13	BICF2G630614003	24272585	G	0	0	A
13	BICF2G630614004	24278279	A	0	0	G
13	BICF2G630614021	24286245	A	0	0.2857	G
13	BICF2G630614028	24295558	A	0	0	G
13	BICF2G630614032	24303336	G	0	0	A
13	BICF2P702513	24318022	A	0.5	0.7857	C
13	BICF2G630614053	24324297	A	0.5	0.7857	G
13	BICF2G630614063	24335426	A	0	0	G

13	BICF2P1115931	24356200	G	0.5	0.7857	A
13	BICF2G630614072	24363484	G	0.5	0.2143	A
13	BICF2G630614079	24369167	A	0.5	0.2143	G
13	BICF2G630614099	24380019	G	0	0	A
13	BICF2G630614108	24393149	A	0.5	0.2143	G
13	BICF2G630614121	24409046	C	0	0	A
13	BICF2G630614135	24414650	A	0	0	G
13	BICF2G630614146	24425691	G	0.5	0.2143	A
13	BICF2G630614165	24433840	G	0	0	A
13	BICF2G630614192	24441690	A	0.5	0.2143	G
13	BICF2G630614211	24454282	A	0	0	T
13	BICF2G630614225	24459598	G	0	0	A
13	BICF2G630614227	24460643	A	1	1	G
13	BICF2G630614231	24485536	G	0	0	A
13	BICF2G630614261	24506154	G	0.5	0.2143	A
13	BICF2P183094	24511197	G	0	0	A
13	BICF2S23345340	24523360	G	0.5	0.7857	A
13	BICF2G630614296	24529607	C	0	0	A
13	BICF2G630614306	24543301	C	0	0	A
13	BICF2S23710419	24563123	G	0.5	0.2143	A
13	BICF2G630614312	24574714	A	0	0	G
13	BICF2P700830	24581284	A	0.5	0.7857	G
13	BICF2G630614316	24593830	A	0.5	0.2143	G
13	BICF2G630614334	24601616	G	0	0	A
13	BICF2P231372	24610128	0	0	0	G
13	BICF2S23516162	24631458	A	0	0	G
13	BICF2G630614359	24635727	C	0.5	0.2143	A
13	BICF2S23111129	24645346	A	0	0	G
13	BICF2G630614372	24657133	G	0.5	0.2143	A
13	BICF2G630614387	24668735	G	0.5	0.2143	A
13	BICF2G630614410	24685597	G	0.5	0.2143	A
13	BICF2P1231598	24699160	G	0.5	0.7857	A
13	BICF2P997148	24718428	G	0.5	0.5	A
13	BICF2P631048	24733845	A	0.5	0.5	G
13	BICF2P1149249	24757101	A	0.5	0.7857	G
13	BICF2P1404531	24766508	A	0.5	0.5	C
13	BICF2G630614416	24777905	A	1	0.7143	C
13	BICF2P698619	24788438	C	0	0	A
13	BICF2G630614418	24794972	0	0	0	A
13	BICF2S2324706	24828133	0	0	0	C
13	BICF2S2326094	24831851	A	0	0	G
13	BICF2S23256538	24844838	A	0.5	0.7857	G
13	BICF2P230727	24858741	C	0	0.2857	G
13	BICF2G630614422	24871078	0	0	0	A
13	BICF2S23620436	24879304	A	0.5	0.2143	G
13	BICF2P345231	24894311	A	0.5	0.2143	G
13	BICF2S23243733	24917997	G	0.5	0.5	A

13	BICF2S23624143	24920731	G	0	0	A
13	BICF2G630614443	24934136	G	0.5	0.2143	A
13	BICF2G630614476	24947413	A	0.5	0.2143	C
13	BICF2G630614496	24956151	A	0.5	0.2143	G
13	BICF2G630614505	24968863	G	0.5	0.2143	A
13	BICF2G630614534	24994072	C	0	0	A
13	BICF2G630614547	24998584	A	0.5	0.2143	G
13	BICF2G630614558	25005413	C	0	0.2857	G
13	BICF2G630614565	25008775	G	0	0.2857	A
13	BICF2G630614585	25013556	A	0	0	G
13	BICF2G630614605	25029140	A	0	0	G
13	BICF2G630614606	25029779	A	0	0	G
13	BICF2G630614620	25034459	A	0.5	0.5	G
13	BICF2G630614627	25036340	A	0.5	0.5	G
13	BICF2G630614670	25053029	A	0	0	G
13	BICF2G630614691	25059538	A	0	0	G
13	BICF2G630614728	25071007	A	0	0	G
13	BICF2G630614744	25090233	C	0.5	0.5	A
13	BICF2G630614746	25096000	C	0.5	0.2143	G
13	BICF2G630614764	25125673	A	0	0	G
13	BICF2G630614770	25135960	G	0.5	0.2143	A
13	BICF2G630614783	25154542	A	0	0	G
13	BICF2S23714554	25165808	G	0	0	A
13	BICF2G630614832	25186282	A	0	0	G
13	BICF2G630614849	25198444	A	0.5	0.7857	G
13	BICF2G630614875	25208958	A	0.5	0.5	C
13	BICF2G630614905	25220673	A	0.5	0.7857	G
13	BICF2G630614922	25231507	A	0	0.2857	G
13	BICF2G630614925	25233012	A	0	0.2857	C
13	BICF2G630614944	25249849	A	1	0.7143	G
13	BICF2G630614945	25254560	A	0.5	0.2143	G
13	BICF2G630614987	25269874	G	0	0.2857	A
13	BICF2G630615000	25277595	A	0	0	C
13	BICF2G630615044	25289212	G	0	0	A
13	BICF2G630615064	25301745	A	0.5	0.2143	G
13	BICF2G630615102	25324929	A	0	0	G
13	BICF2G630615121	25343441	A	0	0	G
13	TIGRP2P174503_rs9151817	25358958	0	0	0	G
13	BICF2S23250133	25378959	A	0	0	G
13	BICF2P763706	25391642	0	0	0	G
13	BICF2S22911889	25410671	G	0	0	C
13	BICF2P227428	25416407	A	0	0.2857	G
13	BICF2G630615155	25419951	G	0	0.2857	A
13	BICF2G630615170	25428455	G	0	0	A
13	BICF2G630615186	25441325	A	0	0	G
13	BICF2S2448882	25457547	A	0	0	C
13	BICF2G630615207	25470547	G	0	0	A

13	BICF2P129093	25477618	0	0	0	A
13	BICF2G630615212	25497611	A	0	0	G
13	BICF2S22928267	25522982	G	0	0	A
13	BICF2G630615221	25531993	A	0.5	0.5	G
13	BICF2S23442124	25543285	A	0.5	0.5	G
13	BICF2G630615250	25564467	A	0.5	0.5	C
13	BICF2G630615252	25566955	A	0.5	0.5	G
13	BICF2G630615262	25581981	A	0.5	0.5	G
13	BICF2G630615279	25597672	A	0.5	0.5	C
13	BICF2G630615298	25613978	C	0	0	A
13	BICF2G630615320	25622993	A	0	0	G
13	BICF2G630615344	25633933	A	0.5	0.5	G
13	BICF2G630615376	25654506	G	0.5	0.5	A
13	BICF2G630615390	25659960	A	0	0	G
13	BICF2G630615411	25674079	A	0	0	G
13	BICF2G630615416	25680506	C	0	0	A
13	BICF2G630615434	25691106	A	0	0	G
13	BICF2G630615451	25701164	A	0	0.2857	G
13	BICF2G630615463	25705558	A	1	0.7143	G
13	BICF2S2317998	25715700	A	0.5	0.2143	G
13	BICF2G630615487	25731744	G	0	0	A
13	BICF2G630615506	25740030	C	0	0	A
13	BICF2G630615520	25750507	A	0	0	G
13	BICF2G630615547	25764429	A	0	0	C
13	BICF2S23543403	25798926	C	0.5	0.5	A
13	BICF2S23654058	25820335	G	0	0.2857	A
13	BICF2P140059	25843825	0	0	0	G
13	BICF2P454243	25860295	A	0	0	G
13	BICF2P527892	25874529	0	0	0	C
13	BICF2S22928705	25875822	G	0.5	0.5	A
13	BICF2S22929120	25893472	A	0.5	0.7857	G
13	BICF2G630615593	25906886	A	0	0	G
13	BICF2G630615604	25915740	G	0.5	0.5	A
13	BICF2G630615623	25924408	A	0.5	0.2143	G
13	BICF2G630615630	25939284	G	0	0	A
13	BICF2G630615640	25944543	A	0.5	0.5	C
13	BICF2G630615656	25964826	G	0.5	0.2143	A
13	BICF2S2309128	25974300	0	0	0	T
13	BICF2P898358	25986381	G	0.5	0.5	A
13	BICF2G630615675	25990983	A	0.5	0.2143	G
13	BICF2G630615686	26002382	G	0.5	0.2143	A
13	BICF2G630615709	26022133	G	0.5	0.1667	A
13	BICF2G630615710	26022828	A	0.5	0.2143	G
13	BICF2G630615715	26031870	A	0	0	G
13	BICF2G630615722	26042607	G	0.5	0.5	A
13	BICF2P1416007	26059954	A	0	0	G
13	BICF2G630615746	26061087	A	1	1	G

13	BICF2G630615774	26089097	A	0.5	0.5	G
13	BICF2G630615796	26102639	A	0.5	0.5	G
13	BICF2P644878	26126278	0	0	0	A
13	BICF2P1469927	26137933	0	0	0	C
13	BICF2G630615815	26146231	A	0.5	0.5	G
13	BICF2G630615822	26151369	A	1	0.7143	G
13	BICF2S23336082	26165209	A	0.5	0.2143	G
13	BICF2P469248	26181282	A	0	0	G
13	BICF2P504931	26191381	0	0	0	A
13	BICF2S23539097	26202515	G	0.5	0.5	A
13	BICF2G630615845	26230670	A	0	0	G
13	BICF2G630615846	26231625	G	0.5	0.5	A
13	BICF2P1506764	26247013	A	0	0	G
13	BICF2G630615865	26256881	G	0	0	A
13	BICF2G630615879	26266454	A	0	0	C
13	BICF2P1275283	26278054	G	0.5	0.2143	A
13	BICF2G630615889	26294158	A	0	0	G
13	BICF2G630615901	26301402	C	0	0	A
13	BICF2G630615912	26315885	A	0	0	T
13	BICF2G630615922	26319007	G	0	0	A
13	BICF2G630615929	26323479	G	0	0	A
13	BICF2G630615936	26330877	A	0	0	G
13	BICF2G630615942	26337339	G	0.5	0.7857	A
13	BICF2G630615966	26351000	A	0	0	G
13	BICF2S23039693	26352325	G	0.5	0.2143	A
13	BICF2S23227687	26364342	A	0.5	0.2143	G
13	BICF2G630616004	26379341	A	0	0	G
13	BICF2G630616021	26397269	A	0	0	G
13	BICF2G630616027	26407673	G	0.5	0.2143	A
13	BICF2P1434110	26417251	G	1	0.7143	A
13	BICF2S22918116	26430585	A	0.5	0.5	C
13	BICF2P1318675	26440885	C	0	0.2857	G
13	BICF2P631138	26460553	A	0	0	G
13	BICF2G630616071	26470743	C	0	0	A
13	BICF2S23534972	26479266	A	0	0	C
13	BICF2G630616079	26485563	G	0	0	A
13	BICF2G630616099	26506017	G	0	0	A
13	BICF2G630616101	26509434	A	0	0	G
13	BICF2G630616120	26527418	A	0	0	G
13	BICF2G630616131	26532326	G	1	0.7143	A
13	BICF2P1371406	26546518	0	0	0	G
13	BICF2P438675	26556929	A	0	0	C
13	BICF2G630616160	26569595	G	0	0	A
13	BICF2G630616162	26584733	G	0	0	A
13	BICF2G630616199	26596943	A	0	0	G
13	BICF2G630616201	26600552	A	1	0.7143	C
13	BICF2P1224625	26621147	A	0	0	C

13	BICF2G630616234	26623710	A	0	0.2857	G
13	BICF2G630616243	26653152	A	0	0	T
13	BICF2G630616257	26657485	A	0	0	G
13	BICF2G630616268	26669790	A	0	0	G
13	BICF2S2373761	26686582	C	0	0	A
13	BICF2S23139708	26700745	C	0	0.2857	G
13	BICF2G630616285	26708913	A	0	0	T
13	BICF2P1207814	26717374	G	0	0	A
13	BICF2G630616306	26731749	G	0	0	A
13	BICF2G630616325	26742935	A	0	0	G
13	BICF2G630616358	26758659	G	0	0	A
13	TIGRP2P174517_rs9048992	26762796	0	0	0	G
13	BICF2S23450127	26779281	G	0	0.2857	A
13	BICF2G630616395	26783892	G	0	0	A
13	BICF2P790332	26801998	G	0.5	0.2143	A
13	BICF2P1428283	26807341	A	0.5	0.2143	G
13	BICF2P474021	26828478	G	0.5	0.2143	A
13	BICF2S23260848	26837055	G	0.5	0.2143	A
13	BICF2S23631203	26841309	A	0.5	0.2143	G
13	BICF2S23157464	26854125	A	0.5	0.2143	G
13	BICF2S23340302	26868889	G	0.5	0.2143	A
13	BICF2G630616421	26896592	A	0	0	G
13	BICF2G630616422	26903052	G	0	0	A
13	BICF2G630616424	26911739	A	0	0	G
13	BICF2G630616434	26922036	A	0	0	G
13	BICF2P287849	26934504	G	0	0	A
13	BICF2G630616465	26951845	C	0	0	A
13	BICF2G630616472	26959221	G	0	0	A
13	BICF2S23720960	26979577	A	0	0	G
13	BICF2G630616486	27000044	C	0.5	0.2143	A
13	BICF2P102491	27004521	A	1	1	G
13	BICF2P1078108	27014183	A	0	0	G
13	BICF2P844798	27026973	A	0	0.2857	G
13	BICF2P16542	27065279	0	0	0	C
13	BICF2P265320	27079420	A	1	0.7143	G
13	BICF2G630616502	27090843	A	1	0.7143	G
13	BICF2G630616510	27094574	A	1	0.7143	G
13	BICF2G630616516	27097125	G	1	0.7143	A
13	BICF2G630616530	27113882	G	1	0.7143	A
13	BICF2G630616540	27120636	A	1	0.7143	G
13	BICF2G630616547	27137121	G	0.5	0.2143	A
13	TIGRP2P174522_rs8713397	27141374	0	0	0	A
13	BICF2G630616563	27152646	G	0	0	A
13	BICF2G630616577	27158955	A	0	0	G
13	BICF2G630616583	27159342	A	0	0	G
13	BICF2S23129171	27165724	A	0	0.2857	G
13	BICF2P334623	27182215	A	0.5	0.5	G

13	BICF2P915421	27190475	A	0	0	G
13	BICF2G630616592	27205103	A	0	0.2857	G
13	BICF2G630616598	27209027	A	0	0	C
13	BICF2G630616615	27227623	G	0	0.2857	A
13	BICF2S23618778	27233316	A	0	0	G
13	BICF2P19680	27246752	0	0	0	G
13	BICF2G630616622	27260533	A	0	0.2857	G
13	BICF2G630616642	27269237	A	0	0.2857	G
13	BICF2G630616660	27281292	A	0.5	0.7857	G
13	BICF2S23145005	27294613	G	1	0.7143	A
13	BICF2S23259014	27318760	A	0	0	G
13	BICF2G630616673	27342825	A	0	0	C
13	BICF2G630616688	27351560	G	0	0	A
13	BICF2G630616697	27360384	A	0.5	0.2143	G
13	BICF2G630616702	27371905	A	0	0	G
13	BICF2P1298613	27383736	A	0.5	0.2143	G
13	BICF2P262498	27392532	A	0.5	0.2143	G
13	BICF2P612593	27397292	0	0	0	A
13	BICF2P360830	27404911	G	0.5	0.2143	A
13	BICF2G630616710	27418698	A	0	0	G
13	BICF2P1278289	27433863	A	0.5	0.2143	G
13	BICF2P565928	27440062	G	0.5	0.2143	A
13	BICF2P1417679	27453462	C	1	0.75	A
13	BICF2G630616713	27470034	G	0	0	A
13	BICF2S2348813	27473947	G	0	0	A
13	BICF2G630616730	27489928	A	0	0	G
13	BICF2G630616740	27505380	A	1	1	G
13	BICF2G630616742	27509632	G	0	0	A
13	BICF2G630616746	27522299	A	0	0	G
13	BICF2G630616769	27541599	C	1	1	A
13	BICF2G630616774	27543706	A	0	0	G
13	BICF2G630616786	27558889	A	0	0	G
13	BICF2G630616789	27575055	A	0	0.2857	G
13	BICF2G630616800	27579271	A	0	0	G
13	BICF2G630616805	27599073	A	0	0	G
13	BICF2P949802	27608956	0	0	0	A
13	BICF2G630616806	27613738	C	0	0	A
13	BICF2G630616818	27623769	C	0	0	A
13	BICF2P773144	27645931	A	0	0	G
13	BICF2P1396319	27663429	A	0	0	G
13	BICF2G630616844	27672147	A	0	0	C
13	BICF2G630616847	27686410	A	0	0	G
13	BICF2G630616867	27701447	C	0	0	A
13	BICF2G630616878	27710747	A	0	0	G
13	BICF2G630616907	27722729	A	0.5	0.7857	G
13	BICF2G630616914	27731984	G	0	0	A
13	BICF2S23212644	27749393	G	0.5	0.5	A

13	BICF2G630616933	27752446	A	0	0.2857	G
13	BICF2G630616957	27764299	G	0.5	0.7857	A
13	BICF2G630616971	27776372	A	0.5	0.5	G
13	BICF2G630617004	27801151	G	0	0	A
13	BICF2G630617014	27807592	A	0.5	0.5	C
13	BICF2G630617037	27822412	A	0	0	G
13	BICF2G630617043	27831739	G	1	1	A
13	BICF2G630617067	27841901	A	0	0	G
13	BICF2P988582	27856384	G	0.5	0.2143	A
13	BICF2G630617082	27864633	C	0.5	0.5	A
13	BICF2G630617102	27879107	A	0	0	G
13	BICF2G630617121	27892373	A	0	0	G
13	BICF2G630617126	27899721	G	0	0.2857	A
13	BICF2P449473	27913803	A	0	0	C
13	BICF2S23151194	27922577	A	0.5	0.2143	G
13	TIGRP2P174526_rs8682962	27956630	0	0	0	A
13	BICF2G630617152	27968255	T	0	0	A
13	BICF2P924973	27988085	0	0	0	A
13	BICF2P1342663	28001468	A	0.5	0.7857	G
13	BICF2G630617157	28006015	A	0	0	G
13	BICF2G630617162	28018160	0	0	0	A
13	BICF2G630617170	28029935	G	0	0	A
13	BICF2P1205132	28030395	0	0	0	A
13	BICF2G630617177	28033662	0	0	0	C
13	BICF2G630617188	28045668	A	0	0	G
13	BICF2G630617207	28047934	G	0	0	A
13	BICF2G630617215	28048619	A	0	0	G
13	BICF2G630617245	28076481	A	0	0	G
13	BICF2G630617251	28091596	0	0	0	G
13	BICF2G630617258	28103948	A	0	0	C
13	BICF2P898929	28110441	A	0	0	G
13	BICF2G630617264	28133854	A	0	0	G
13	BICF2P105843	28148379	C	0.5	0.2143	A
13	BICF2S23029185	28162597	A	0.5	0.7857	G
13	BICF2S23033902	28166664	A	0.5	0.2143	G
13	BICF2S23029487	28178693	A	0	0	G
13	BICF2P166682	28204495	A	1	1	G
13	BICF2P726805	28208279	A	0	0	G
13	BICF2G630617270	28210311	G	0	0	A
13	BICF2G630617282	28230429	A	0.5	0.5	C
13	BICF2G630617285	28233305	A	0	0	G
13	BICF2G630617296	28245780	A	0.5	0.5	C
13	BICF2G630617306	28255693	A	0	0	G
13	BICF2G630617319	28273467	A	0.5	0.5	C
13	BICF2G630617323	28279396	A	0	0	G
13	BICF2P564681	28291984	A	0	0	G
13	BICF2S23051140	28301987	G	0	0	A

13	BICF2P1458071	28316062	0	0	0	A
13	BICF2G630617344	28324572	A	0	0	G
13	BICF2G630617348	28337118	A	0.5	0.2143	G
13	BICF2G630617377	28360294	G	0	0	A
13	BICF2G630617391	28379210	G	0.5	0.2143	A
13	BICF2G630617394	28383512	G	1	1	A
13	BICF2P928845	28404340	A	0	0	G
13	BICF2P504166	28410252	G	0.5	0.2143	A
13	BICF2P300209	28423773	A	0	0	G
13	BICF2P1282193	28431021	0	0	0	G
13	TIGRP2P174531_rs9023113	28445035	0	0	0	A
13	BICF2P539993	28460402	G	0.5	0.5	C
13	BICF2P539992	28460490	G	0.5	0.5	A
13	BICF2P539986	28460869	T	0.5	0.5	A
13	BICF2G630617417	28467529	A	0	0.2	G
13	BICF2P871887	28474850	G	0.5	0.5	A
13	BICF2P17941	28491108	G	0.5	0.2143	C
13	BICF2P1258740	28497528	G	0	0	A
13	BICF2G630617430	28512533	G	0.5	0.2143	A
13	BICF2S22922677	28539727	G	0.5	0.2143	A
13	BICF2S22927798	28544020	A	0	0	G
13	BICF2S2332280	28556714	G	0	0	A
13	BICF2G630617441	28567941	A	0	0.2857	G
13	G779f35S83	28588428	A	0	0	G
13	TIGRP2P174532_rs9083243	28595921	0	0	0	A
13	BICF2S23648815	28611224	G	0	0	A
13	BICF2P1415651	28619636	G	0	0.2857	A
13	BICF2P1133123	28623847	A	0	0.2857	C
13	BICF2G630617453	28668400	G	0.5	0.5	A
13	BICF2G630617454	28671394	G	0.5	0.5	A
13	BICF2P1120423	28675649	T	0	0.2857	A
13	BICF2G630617457	28689330	A	0	0	G
13	BICF2S23542932	28712411	C	0	0	G
13	BICF2G630617478	28717015	T	0	0	A
13	BICF2G630617488	28728032	A	0	0.2857	G
13	BICF2G630617496	28748263	C	0	0.2857	A
13	BICF2S2336017	28752034	G	1	0.7143	A
13	BICF2G630617516	28762194	A	0	0.2857	G
13	BICF2G630617533	28775680	G	0	0.2857	A
13	BICF2G630617535	28786140	A	0	0.2857	C
13	BICF2P864645	28808894	G	0	0	A
13	BICF2P841449	28824892	A	0	0	G
13	BICF2P942548	28838183	G	0	0	C
13	BICF2P460279	28847214	G	0	0	A
13	BICF2P513813	28858637	G	0	0	A
13	BICF2P1292716	28866815	A	0	0	G
13	BICF2P318652	28879708	A	0	0	T

13	BICF2G630617550	28892953	A	1	0.7143	T
13	BICF2S236936	28907475	0	0	0	G
13	BICF2S23624844	28914409	0	0	0	G
13	BICF2G630617579	28928605	G	0	0.2857	A
13	BICF2S23542607	28946535	G	0	0	A
13	TIGRP2P174536_rs9119936	28975711	0	0	0	A
13	BICF2G630617580	28992435	A	1	0.6667	T
13	BICF2P1197081	29012602	0	0	0	C
13	TIGRP2P174537_rs8935986	29016068	0	0	0	C
13	BICF2G630617583	29033271	A	0	0	G
13	BICF2S23610364	29038597	G	0	0	A
13	BICF2S23524284	29062901	0	0	0	A
13	BICF2P1258944	29084575	0	0	0	C
13	BICF2G630617589	29116209	0	0	0	G
13	BICF2P492152	29118911	A	0	0	G
13	BICF2P1438401	29157652	0	0	0	A
13	BICF2G630617591	29181680	G	0.5	0.7857	A
13	BICF2S2332496	29189548	G	0.5	0.7857	A
13	BICF2P645412	29199455	0	0	0	C
13	BICF2P869867	29216469	A	0	0	G
13	BICF2S2332598	29224454	G	0	0	A
13	BICF2G630617594	29233318	G	0.5	0.2143	A
13	BICF2P1394049	29246279	G	0	0	A
13	BICF2G630617607	29267079	A	0.5	0.2143	G
13	BICF2G630617646	29285137	A	0.5	0.7857	G
13	BICF2G630617662	29298418	G	0.5	0.1667	A
13	BICF2G630617688	29312301	A	0.5	0.2143	G
13	BICF2P647598	29320668	A	0.5	0.2143	G
13	BICF2G630617705	29327568	C	0.5	0.2143	A
13	BICF2G630617708	29353608	A	0.5	0.7857	G
13	BICF2P593161	29367722	0	0	0	A
13	BICF2G630617718	29380216	A	0.5	0.2143	T
13	BICF2G630617733	29391604	A	0.5	0.7857	G
13	BICF2G630617741	29403043	C	0	0.2857	A
13	BICF2P978920	29412347	0	0	0	C
13	BICF2S22949846	29454952	A	0	0.2857	G
13	BICF2G630617745	29463660	G	0	0	A
13	BICF2G630617748	29477724	C	0.5	0.2143	A
13	BICF2G630617757	29506356	A	0	0	G
13	BICF2G630617761	29509757	G	0	0	A
13	BICF2P343725	29536625	A	0	0	G
13	BICF2G630617784	29543774	A	0	0	T
13	BICF2P104086	29562879	0	0	0	A
13	BICF2P450349	29576890	A	0	0	G
13	BICF2P323658	29580501	0	0	0	A
13	G781f43S142	29604221	0	0	0	C
13	BICF2S24317234	29629329	C	0.5	0.5	A

13	BICF2P1217348	29663362	0	0	0	G
13	BICF2P349782	29678678	0	0	0	G
13	BICF2P1377548	29721185	A	0	0.2857	G
13	BICF2P1013261	29730125	A	0	0.2857	G
13	BICF2P767342	29745709	A	0	0.2857	G
13	BICF2P585151	29761306	0	0	0	G
13	G782f50S151	29785418	G	0	0	A
13	BICF2S23520159	29805477	A	0.5	0.5	G
13	BICF2G630617790	29844662	G	0	0	A
13	BICF2G630617812	29857339	G	0.5	0.7857	A
13	BICF2P1265977	29870739	A	0	0	G
13	BICF2G630617829	29877568	A	0	0.2857	G
13	BICF2G630617837	29891687	A	0	0	G
13	BICF2G630617841	29904110	C	0	0.2857	A
13	BICF2S23043108	29921884	G	0	0.2857	A
13	BICF2S23037374	29926042	C	0	0.2857	A
13	BICF2G630617855	29935531	G	0	0.2857	A
13	BICF2G630617860	29952327	A	0	0	G
13	BICF2G630617864	29963871	A	0	0.2857	T
13	BICF2S2343470	29972104	C	0.5	0.5	A
13	BICF2G630617871	29983514	G	0.5	0.2143	A
13	BICF2G630617879	29992102	G	0	0	A
13	BICF2S23029238	30004936	C	0	0	A
13	BICF2G630617896	30034012	C	0	0	A
13	BICF2G630617906	30042064	G	0	0	A
13	BICF2P769650	30060610	A	0	0.2857	G
13	BICF2S2328722	30067856	G	0	0.2857	A
13	BICF2G630617922	30079107	A	0.5	0.5	G
13	BICF2G630617940	30097354	A	0.5	0.5	G
13	TIGRP2P174555_rs8590846	30114300	0	0	0	C
13	BICF2G630617959	30121346	0	0	0	C
13	BICF2G630617967	30130122	A	0	0	G
13	BICF2G630617997	30149191	C	1	0.7143	G
13	BICF2S23326599	30155201	A	0	0.2857	G
13	BICF2G630618014	30168629	G	0	0.2857	A
13	BICF2G630618021	30183351	A	0	0	G
13	BICF2G630618024	30194357	G	0	0.2857	A
13	BICF2G630618026	30199287	G	0	0.2857	A
13	BICF2G630618039	30212758	A	0.5	0.5	C
13	BICF2G630618072	30260273	C	0.5	0.7857	G
13	BICF2G630618081	30271940	A	0.5	0.5	G
13	BICF2G630618100	30283476	G	0	0.2857	A
13	BICF2G630618116	30290531	A	0	0	G
13	BICF2G630618130	30301323	A	0	0	G
13	BICF2G630618133	30306681	G	0.5	0.5	A
13	BICF2S23763401	30314958	0	0	0	G
13	BICF2S23624348	30327134	A	0.5	0.2143	G

13	BICF2G630618146	30337930	G	0.5	0.2143	C
13	BICF2G630618174	30350594	G	0.5	0.2143	A
13	BICF2P1238954	30367943	G	0.5	0.2143	A
13	BICF2G630618176	30379059	0	0	0	A
13	BICF2G630618183	30385360	A	0	0	G
13	BICF2G630618202	30394845	A	0	0	C
13	BICF2S229669	30409698	G	0	0	A
13	BICF2P227356	30418286	A	0	0	G
13	BICF2S23545388	30429615	G	0.5	0.2143	A
13	BICF2P1004600	30443706	A	0	0.2857	G
13	BICF2S2302035	30457922	G	0	0	A
13	BICF2G630618246	30467417	A	0	0.2857	G
13	BICF2P337745	30480156	A	0	0.2857	T
13	BICF2P337742	30480616	A	0	0.2857	G
13	BICF2P1351100	30488934	G	0.5	0.1667	A
13	BICF2S23260214	30503135	A	0.5	0.5	G
13	BICF2G630618288	30519654	G	0	0	A
13	BICF2G630618294	30522428	A	0	0	G
13	BICF2G630618310	30534729	A	0	0	G
13	BICF2G630618331	30547590	A	0	0	G
13	BICF2G630618360	30563093	G	0	0	A
13	BICF2G630618363	30564517	G	0	0	A
13	BICF2P775070	30568105	0	0	0	G
13	BICF2P1155894	30584539	A	0	0	G
13	BICF2P807152	30623033	0	0	0	G
13	BICF2P1158869	30629492	A	0	0	G
13	TIGRP2P174559_rs8556593	30640949	0	0	0	C
13	BICF2S22916159	30650656	G	0	0	A
13	BICF2G630618369	30674764	A	0.5	0.5	G
13	BICF2S23660645	30710676	A	0	0	C
13	BICF2G630618373	30713723	A	0	0	G
13	BICF2G630618379	30717162	A	0	0	G
13	BICF2G630618416	30733081	A	0	0	G
13	BICF2G630618444	30743867	A	0	0	C
13	BICF2G630618449	30745644	A	0	0	C
13	BICF2G630618462	30754643	C	0	0	A
13	BICF2G630618481	30764157	C	0	0	A
13	BICF2G630618492	30769347	G	0	0	A
13	BICF2G630618495	30775049	A	0	0	G
13	BICF2G630618507	30787669	A	0	0	G
13	BICF2G630618522	30798149	A	0	0	G
13	BICF2G630618542	30813061	A	0	0	G
13	BICF2G630618554	30825306	A	0	0	G
13	BICF2G630618559	30838820	A	0	0	G
13	BICF2G630618591	30856225	G	0	0	A
13	BICF2G630618605	30868506	G	0.5	0.5	A
13	BICF2G630618654	30900380	A	0	0.2857	G

13	BICF2G630618655	30902818	G	0	0.2857	A
13	BICF2G630618681	30916232	G	0	0.2857	A
13	BICF2G630618710	30940681	A	0	0.2857	G
13	BICF2G630618769	30955769	G	0	0.2857	A
13	BICF2G630618781	30961098	A	0	0	G
13	BICF2G630618789	30977560	A	0	0	G
13	BICF2P281287	30986415	A	0	0.2857	C
13	BICF2S23420168	31004409	A	0.5	0.2143	G
13	BICF2G630618796	31025649	A	0	0	G
13	BICF2G630618810	31036508	A	0	0	G
13	BICF2G630618831	31042416	C	0	0	A
13	BICF2S23034036	31059708	0	0	0	G
13	BICF2G630618870	31066269	A	0	0.2857	G
13	BICF2S23513256	31080202	A	0	0	G
13	BICF2G630618897	31086130	A	0	0	G
13	BICF2G630618907	31102033	C	0	0	A
13	BICF2G630618909	31112212	A	0	0.2857	G
13	BICF2P1395518	31130087	C	0	0	G
13	BICF2G630618919	31130656	C	0	0	G
13	BICF2G630618921	31147170	A	0	0	G
13	BICF2G630618928	31159041	A	0	0	G
13	BICF2G630618936	31185854	G	0	0	A
13	BICF2G630618942	31188819	C	0	0	A
13	BICF2G630618983	31199619	C	0	0	A
13	BICF2G630618994	31202831	G	0	0	A
13	BICF2P918987	31213047	A	0.5	0.5	G
13	BICF2G630619014	31228015	A	0	0	G
13	BICF2G630619017	31234474	G	0	0.2857	A
13	BICF2G630619030	31237887	G	0	0	C
13	BICF2G630619060	31253599	A	0	0	G
13	BICF2P618235	31260294	G	0	0	A
13	BICF2G630619073	31270807	A	0	0	G
13	BICF2G630619098	31294801	0	0	0	G
13	TIGRP2P174579_rs8761632	31304072	0	0	0	C
13	BICF2P464553	31319053	A	0.5	0.5	G
13	BICF2G630619123	31331224	A	0	0	G
13	BICF2G630619126	31337522	G	0.5	0.2143	A
13	BICF2G630619141	31349384	C	0.5	0.2143	A
13	BICF2G630619159	31367148	G	0	0	A
13	BICF2G630619181	31382249	A	0	0	C
13	BICF2P781264	31394313	G	0	0	A
13	BICF2G630619194	31401399	G	0	0	A
13	BICF2G630619197	31407823	G	0	0.2857	A
13	BICF2G630619215	31426742	G	0	0.2857	A
13	BICF2G630619222	31435535	A	0	0	G
13	BICF2G630619230	31444694	A	0.5	0.5	C
13	BICF2G630619237	31452777	G	1	0.7143	A

13	BICF2G630619304	31471598	A	0.5	0.2143	G
13	BICF2P1317475	31477041	G	0.5	0.2143	A
13	BICF2G630619325	31489459	A	0.5	0.5	G
13	BICF2P1031440	31500024	G	0.5	0.5	A
13	BICF2G630619331	31505160	A	0	0	G
13	BICF2P1060740	31510185	A	0.5	0.5	G
13	BICF2G630619343	31524738	G	0.5	0.2143	C
13	BICF2G630619371	31542154	G	0.5	0.5	A
13	BICF2G630619384	31549297	G	0.5	0.5	A
13	BICF2G630619413	31567107	A	0.5	0.5	G
13	BICF2G630619414	31567400	G	0.5	0.5	A
13	BICF2G630619420	31579792	G	0.5	0.5	A
13	BICF2S23635996	31600104	A	0.5	0.5	G
13	BICF2P1410751	31605252	0	0	0	G
13	BICF2G630619428	31619956	A	0	0	G
13	BICF2G630619437	31627168	A	0	0	G
13	BICF2G630619458	31657756	A	0	0	G
13	BICF2G630619462	31662809	A	0	0	G
13	BICF2G630619472	31672886	A	0	0	G
13	BICF2G630619489	31684733	A	0	0	G
13	BICF2G630619494	31698223	A	0	0	C
13	BICF2G630619504	31706359	G	0	0	A
13	BICF2P1365035	31717717	A	0	0	G
13	BICF2G630619537	31729720	C	0	0.2857	A
13	BICF2P760583	31740738	G	0	0	A
13	BICF2G630619544	31752060	A	0	0	G
13	BICF2P551759	31774019	A	0.5	0.5	C
13	BICF2P271806	31776337	A	0	0	G
13	BICF2P1426653	31796381	A	0	0	G
13	BICF2P1236335	31797159	G	0.5	0.5	A
13	BICF2P737823	31807391	0	0	0	G
13	BICF2G630619557	31823857	A	0	0.2857	G
13	BICF2P670307	31834905	A	0	0.2857	C
13	BICF2G630619573	31835050	A	0	0	G
13	TIGRP2P174591_rs8766041	31847855	0	0	0	A
13	BICF2S23646910	31852778	A	0.5	0.5	G
13	BICF2G630619591	31858091	G	0	0.2857	C
13	BICF2G630619594	31859679	A	0.5	0.5	G
13	TIGRP2P174595_rs8696382	31874374	A	0	0	T
13	BICF2G630619611	31879690	A	0	0	G
13	BICF2G630619620	31897305	C	0.5	0.2143	A
13	BICF2G630619621	31898933	A	0.5	0.2143	C
13	BICF2G630619627	31902812	G	0.5	0.2143	A
13	BICF2G630619660	31917724	G	0.5	0.2143	A
13	BICF2G630619679	31930357	G	0.5	0.2143	A
13	BICF2P811347	31937956	A	0.5	0.2143	G
13	BICF2G630619691	31940728	G	0.5	0.1	A

13	BICF2G630619706	31952473	G	0	0	A
13	BICF2G630619719	31960674	C	1	0.7143	A
13	BICF2G630619721	31963403	A	0	0.2857	G
13	BICF2G630619742	31974248	A	0.5	0.2143	G
13	BICF2G630619751	31983218	A	0.5	0.2143	G
13	BICF2P742818	31996501	A	0	0	C
13	BICF2P308924	32011274	C	0	0	G
13	TIGRP2P174646_rs8764384	32026484	G	0	0.2857	A
13	BICF2P479619	32028538	A	0	0.2857	G
13	BICF2P1170281	32049259	A	0.5	0.5	G
13	BICF2S23717009	32055774	A	0	0	G
13	BICF2P841192	32073174	A	0	0	G
13	BICF2G630619767	32079877	G	0.5	0.2143	A
13	BICF2P579970	32085507	C	0	0	A
13	BICF2P1252582	32106652	G	0	0	A
13	BICF2P254088	32111293	0	0	0	A
13	BICF2P309798	32120963	A	0.5	0.7857	G
13	BICF2P493163	32133725	A	0	0	G
13	BICF2P380260	32153302	G	0	0	A
13	BICF2P1369411	32162822	A	0.5	0.5	G
13	BICF2P63458	32169650	A	0	0.2857	G
13	BICF2G630619819	32193067	G	0.5	0.5	A
13	BICF2P1054760	32204972	A	0	0	C
13	BICF2P1266641	32216115	G	0.5	0.7857	A
13	BICF2P528434	32231330	A	0.5	0.5	G
13	BICF2G630619833	32240707	A	0.5	0.5	C
13	BICF2S24416187	32246860	0	0	0	A
13	BICF2P789038	32262468	0	0	0	A
13	BICF2P864619	32270195	A	0	0	G
13	BICF2G630619836	32285206	A	0.5	0.5	G
13	BICF2G630619848	32292493	G	0.5	0.5	A
13	BICF2G630619855	32307487	A	0	0	G
13	BICF2G630619863	32323671	C	0.5	0.5	G
13	BICF2G630619864	32327745	A	0	0	G
13	BICF2G630619869	32338163	A	0.5	0.7857	G
13	BICF2G630619872	32356894	C	0.5	0.5	A
13	BICF2G630619881	32373318	A	0.5	0.7857	T
13	BICF2S24116911	32393951	0	0	0	G
13	BICF2G630619891	32397260	A	0	0	C
13	BICF2S22963794	32412417	A	0	0.2857	G
13	BICF2S23247226	32417138	C	0	0	G
13	BICF2P1092778	32427111	A	0	0.2857	G
13	BICF2P172781	32430032	G	0	0	A
13	TIGRP2P174743_rs8965161	32431486	G	0	0	A
13	BICF2P662621	32431662	G	0	0	A
13	BICF2G630619902	32440773	A	0	0	G
13	BICF2G630619907	32446681	A	0	0	G

13	BICF2S23050814	32459850	G	0	0	A
13	BICF2G630619929	32471156	A	0.5	0.7857	G
13	BICF2P876000	32481102	C	0	0	G
13	TIGRP2P174758_rs8810161	32493472	A	0	0	G
13	BICF2P804701	32508577	G	0	0	A
13	BICF2P804700	32508723	C	0	0	A
13	TIGRP2P174777_rs9078915	32514995	G	0.5	0.5	A
13	BICF2P1469768	32532493	A	0	0	G
13	BICF2G630619982	32540535	A	0	0	G
13	BICF2P1419957	32554764	T	0	0	A
13	BICF2P1419958	32554838	A	0	0	G
13	BICF2P343147	32557602	A	0	0	G
13	BICF2G630620049	32583084	G	0	0	A
13	BICF2G630620073	32595455	G	0.5	0.2143	A
13	BICF2G630620075	32595570	G	0	0	A
13	BICF2G630620095	32604315	A	0	0	G
13	TIGRP2P174827_rs8702804	32614520	A	0	0	G
13	BICF2G630620124	32617578	G	0.5	0.2143	A
13	BICF2G630620126	32621614	A	0.5	0.2143	G
13	BICF2G630620154	32631744	A	0.5	0.2143	G
13	BICF2S23629964	32637330	G	0.5	0.2143	A
13	BICF2P1259579	32643673	A	0	0	G
13	BICF2G630620188	32653660	A	0.5	0.2143	G
13	BICF2G630620210	32663298	A	0.5	0.2143	G
13	BICF2P514080	32676178	A	0	0	G
13	BICF2S23755948	32687252	0	0	0	G
13	BICF2G630664951	32715604	G	0.5	0.5	A
13	BICF2G630664929	32725064	A	0	0	G
13	BICF2G630664921	32729909	A	1	1	G
13	BICF2G630664911	32743001	G	0	0	A
13	BICF2G630664905	32753971	A	0.5	0.7857	G
13	BICF2G630664904	32754029	G	0.5	0.7857	A
13	BICF2G630664849	32766629	A	0.5	0.2143	G
13	BICF2S23347189	32780097	A	0.5	0.5	G
13	BICF2G630664827	32797842	A	0.5	0.2143	G
13	BICF2P1104782	32802571	A	0	0.2857	G
13	BICF2P1104781	32802694	G	0	0.2857	A
13	BICF2G630664803	32813009	A	0	0	G
13	BICF2G630664768	32828017	G	1	0.7143	A
13	TIGRP2P174928_rs8643422	32834126	A	0	0	G
13	BICF2P311078	32845973	A	1	0.7143	G
13	BICF2G630664759	32862264	A	0	0	G
13	BICF2G630664743	32870000	A	0	0	G
13	BICF2S23356286	32887856	0	0	0	G
13	BICF2G630664739	32891431	G	0	0	A
13	BICF2G630664738	32892124	A	0	0	G
13	BICF2G630664706	32905664	G	0	0.2857	A

13	BICF2P33640	32922977	A	0	0.2857	G
13	BICF2P33651	32925027	A	0	0.2857	G
13	BICF2P304340	32936958	C	0	0.2857	A
13	BICF2G630664702	32958695	G	0.5	0.5	A
13	BICF2G630664694	32961385	A	0.5	0.5	G
13	BICF2S23334469	32974577	A	0.5	0.5	G
13	BICF2S23346437	32983541	C	1	1	G
13	BICF2G630664672	32998675	A	0	0.2857	G
13	BICF2G630664654	33011623	G	0.5	0.7857	A
13	BICF2P87576	33027153	0	0	0	T
13	BICF2S23636378	33030373	0	0	0	G
13	BICF2S2349077	33047146	A	0	0.2857	G
13	BICF2S23624795	33060622	G	0.5	0.7857	A
13	BICF2S23414571	33095524	G	0.5	0.5	A
13	BICF2S2436191	33107633	A	0	0	G
13	BICF2S23515353	33108681	G	0	0	A
13	BICF2G630664626	33129115	A	0	0	G
13	BICF2P668759	33163701	0	0	0	G
13	BICF2S2438003	33174721	A	0	0	G
13	BICF2S23155021	33185517	G	0.5	0.5	A
13	BICF2S23154314	33189171	A	0.5	0.5	G
13	BICF2G630664615	33201590	G	1	1	A
13	BICF2G630664592	33216557	G	0.5	0.7857	A
13	BICF2G630664577	33225859	G	0.5	0.7857	A
13	BICF2G630664527	33235278	G	0	0	A
13	BICF2G630664507	33248415	A	0	0	G
13	BICF2P155431	33255848	A	0.5	0.5	G
13	BICF2G630664494	33267703	A	0.5	0.5	G
13	BICF2G630664487	33272472	A	0.5	0.5	C
13	BICF2P1420208	33288968	G	0	0	A
13	BICF2G630664476	33292885	C	1	1	G
13	BICF2P420841	33301796	A	0	0	G
13	BICF2G630664460	33304027	G	0.5	0.5	A
13	BICF2S23433590	33316733	G	0.5	0.2143	A
13	BICF2G630664436	33337260	A	0	0.2857	G
13	BICF2G630664431	33339673	A	1	0.7143	G
13	BICF2G630664399	33355796	A	0.5	0.5	G
13	BICF2G630664366	33364734	G	0.5	0.5	A
13	BICF2G630664365	33365195	G	0.5	0.5	A
13	BICF2G630664345	33374965	G	0	0	A
13	BICF2G630664319	33391890	A	0	0	C
13	BICF2P233085	33405999	C	1	0.7143	A
13	BICF2G630664295	33411520	A	1	0.7143	G
13	BICF2G630664281	33422374	G	1	0.7143	A
13	BICF2G630664277	33431913	C	0	0	G
13	BICF2G630664252	33441918	C	0.5	0.2143	G
13	BICF2G630664245	33445434	G	0.5	0.2143	A

13	BICF2G630664222	33456458	A	0.5	0.2143	G
13	BICF2G630664218	33475730	A	0.5	0.7857	T
13	BICF2G630664207	33479028	A	0	0	G
13	BICF2G630664197	33485500	G	1	1	A
13	BICF2G630664186	33488679	A	0	0	G
13	BICF2G630664162	33507948	A	0	0	G
13	BICF2G630664157	33511619	G	0	0.2857	A
13	BICF2G630664124	33524051	A	0	0.25	C
13	BICF2P152154	33544668	A	0	0	G
13	BICF2G630664091	33548438	G	0	0.2857	A
13	BICF2G630664086	33560281	A	0	0.2857	G
13	TIGRP2P174981_rs9028701	33587003	A	0.5	0.2143	G
13	BICF2P1158405	33593869	G	0	0	A
13	BICF2G630664075	33610279	A	0	0	G
13	BICF2G630664074	33620086	A	0	0	G
13	BICF2G630664063	33626067	A	0	0	G
13	TIGRP2P174998_rs8875768	33638383	A	0	0	G
13	BICF2P92516	33663009	A	0	0	G
13	TIGRP2P175007_rs8810558	33679052	0	0	0	C
13	BICF2S2338045	33683948	A	0.5	0.5	G
13	BICF2P1108805	33696770	G	0	0	A
13	TIGRP2P175009_rs8968851	33714869	0	0	0	G
13	BICF2P79343	33729903	0	0	0	A
13	BICF2S2331550	33741195	G	0.5	0.7857	A
13	BICF2S2398333	33779389	A	0	0	G
13	BICF2P1207620	33791669	0	0	0	G
13	BICF2G630663968	33806471	G	1	1	A
13	BICF2G630663962	33811919	G	1	1	A
13	BICF2G630663956	33821781	A	0	0	G
13	BICF2G630663951	33836437	C	0	0.2857	A
13	BICF2G630663950	33846603	A	0	0.2857	G
13	TIGRP2P175014_rs8847359	33863440	A	1	0.7143	G
13	BICF2P1100069	33878576	A	1	0.7143	G
13	BICF2P160815	33913025	C	0.5	0.2143	A
13	BICF2G630663920	33922162	G	0.5	0.2143	A
13	BICF2G630663916	33944462	A	0	0	G
13	BICF2P137526	33960030	0	0	0	C
13	TIGRP2P175032_rs8549684	33972579	A	0	0	G
13	BICF2P1368861	33984345	A	0	0	G
13	BICF2G630663892	33998013	A	0.5	0.2143	G
13	BICF2G630663890	34006486	A	0	0	G
13	BICF2G630663865	34038763	G	0	0	A
13	BICF2G630663858	34040034	G	0	0	A
13	BICF2G630663811	34062176	A	0.5	0.5	G
13	BICF2G630663804	34066429	A	0.5	0.5	G
13	BICF2P1341235	34068733	G	0	0	A
13	BICF2G630663800	34068978	A	0.5	0.5	C

13	BICF2P250189	34075018	0	0	0	G
13	BICF2G630663782	34095411	A	0.5	0.5	G
13	BICF2P301161	34099305	A	0.5	0.5	C
13	BICF2G630663762	34111516	A	0.5	0.5	C
13	BICF2P942442	34127507	G	0.5	0.5	A
13	BICF2P696842	34133087	G	0.5	0.7857	A
13	BICF2G630663739	34163909	C	0.5	0.5	A
13	BICF2P597825	34170610	T	0	0.2857	A
13	BICF2P597824	34171465	G	0.5	0.5	A
13	BICF2P147542	34180268	A	0	0.2857	G
13	BICF2P225956	34194767	A	0	0	G
13	BICF2S23527835	34205573	G	0	0	A
13	BICF2S23910481	34209485	0	0	0	A
13	BICF2P315725	34219712	A	0	0.2857	G
13	BICF2G630663736	34233640	G	0	0	A
13	BICF2P713780	34239690	A	0	0	G
13	BICF2G630663707	34250283	A	0	0	C
13	BICF2G630663681	34262717	A	0	0.2857	G
13	BICF2G630663676	34265913	A	0	0	G
13	BICF2P1282363	34285855	A	0.5	0.2143	C
13	BICF2G630663629	34295908	G	0	0	A
13	BICF2S22921821	34305275	A	0.5	0.2143	G
13	BICF2G630663590	34316528	A	0.5	0.7857	G
13	BICF2P550813	34327425	G	0.5	0.2143	A
13	BICF2G630663579	34333933	C	0	0	G
13	BICF2G630663578	34333951	G	0	0	C
13	BICF2P43663	34339142	A	0	0	G
13	BICF2S2296705	34353224	A	1	1	G
13	BICF2G630663527	34363019	A	0	0	T
13	BICF2G630663515	34372439	A	0	0	G
13	BICF2G630663507	34376027	A	0	0	G
13	BICF2G630663500	34385888	A	0	0	G
13	BICF2G630663498	34404195	G	0	0	A
13	BICF2S23125522	34408086	G	0.5	0.2143	A
13	BICF2G630663452	34420764	G	0.5	0.2143	A
13	BICF2P551915	34435892	A	0.5	0.5	G
13	BICF2G630663433	34446174	G	1	1	A
13	BICF2S2347604	34460617	G	0	0	A
13	TIGRP2P175164_rs9015357	34467889	G	0	0	A
13	BICF2G630663387	34483867	G	0	0	A
13	BICF2G630663381	34486969	A	1	0.7143	G
13	BICF2G630663379	34489821	A	0.5	0.2143	G
13	BICF2S23234741	34503247	C	1	0.7143	A
13	BICF2G630663340	34514909	G	1	1	A
13	BICF2P327350	34529752	A	0	0	G
13	BICF2G630663321	34535600	A	1	1	G
13	BICF2G630663300	34549442	A	0.5	0.2143	G

13	BICF2G630663291	34570727	0	0	0	G
13	TIGRP2P175203_rs8645196	34584163	G	0	0	A
13	BICF2G630663279	34594944	A	0	0	C
13	BICF2G630663274	34603556	G	0.5	0.7857	A
13	BICF2G630663264	34608119	G	0.5	0.7857	A
13	BICF2G630663248	34617466	A	0.5	0.7857	G
13	BICF2P460641	34632001	A	0.5	0.2143	C
13	BICF2G630663240	34647025	G	0	0.2857	A
13	BICF2G630663227	34652635	G	0	0.2857	A
13	BICF2G630663203	34668551	A	0	0	G
13	BICF2G630663197	34676956	A	0.5	0.7857	G
13	BICF2S23152711	34677019	A	0.5	0.2143	G
13	BICF2G630663188	34687268	A	0.5	0.2143	G
13	BICF2G630663129	34715634	G	0	0.2857	A
13	BICF2G630663123	34724578	C	0.5	0.7857	A
13	BICF2G630663107	34731056	A	0	0	C
13	BICF2G630663091	34746846	A	0.5	0.5	G
13	BICF2G630663089	34760117	C	0	0	A
13	BICF2G630663084	34766737	A	0	0	G
13	TIGRP2P175235_rs9217634	34788436	0	0	0	G
13	BICF2G630663056	34809758	G	0.5	0.7857	C
13	BICF2G630663051	34816534	G	0.5	0.5	A
13	BICF2G630663047	34818194	A	0.5	0.5	G
13	BICF2G630663040	34822334	A	0.5	0.5	G
13	BICF2S23449422	34838676	G	0	0	A
13	BICF2S23134024	34853291	A	0	0	G
13	BICF2S2349330	34860784	A	0	0.2857	G
13	BICF2G630663036	34869755	G	0	0	A
13	BICF2S2309419	34873488	A	0	0	G
13	BICF2P995789	34880846	A	0.5	0.5	G
13	BICF2P1468336	34889109	G	0	0	A
13	BICF2G630662988	34900468	A	0.5	0.7857	G
13	BICF2G630662972	34902706	G	0.5	0.7857	A
13	BICF2G630662954	34917697	A	0	0.2857	C
13	BICF2G630662940	34926901	C	0	0.2857	A
13	BICF2G630662915	34940879	A	0.5	0.2143	G
13	BICF2G630662904	34949982	A	0	0	G
13	BICF2P559136	34960359	0	0	0	C
13	BICF2P1177996	34982148	A	0.5	0.7857	G
13	BICF2G630662879	34985585	G	0.5	0.7857	A
13	BICF2S24113451	35001500	A	0	0.2857	G
13	G783f41S170	35011526	0	0	0	A
13	BICF2P1148532	35033493	A	0	0	G
13	G784f33S88	35056804	0	0	0	G
13	BICF2P1039403	35080492	0	0	0	G
13	BICF2P1020848	35095408	G	0	0	A
13	TIGRP2P175253_rs8999560	35104852	A	0	0	G

13	BICF2G630662855	35109670	A	0	0	G
13	BICF2G630662824	35121837	G	0	0	A
13	BICF2G630662767	35140745	C	0	0	A
13	BICF2G630662749	35146152	A	0	0	G
13	BICF2G630662729	35161773	A	0	0.2857	G
13	BICF2P440002	35169645	A	0	0	G
13	BICF2P1100047	35172957	A	0	0	G
13	BICF2G630662694	35179641	G	0	0.2857	A
13	BICF2P21135	35196146	A	0	0	G
13	BICF2S23055676	35203774	C	0	0	A
13	BICF2G630662670	35220183	A	0	0	G
13	TIGRP2P175312_rs8598888	35231705	C	0.5	0.5	A
13	BICF2G630662646	35243532	T	0	0	A
13	BICF2G630662635	35251437	A	0.5	0.5	G
13	BICF2G630662588	35280329	G	0.5	0.5	A
13	BICF2P39618	35287833	A	0.5	0.5	G
13	BICF2S23038487	35287960	A	0.5	0.5	G
13	BICF2G630662543	35295785	A	0.5	0.5	G
13	BICF2G630662528	35306185	G	0	0	A
13	BICF2G630662502	35321168	A	0	0.2857	G
13	BICF2S231378	35330651	G	0.5	0.5	C
13	TIGRP2P175335_rs9125886	35349970	A	0.5	0.5	G
13	BICF2G630662498	35354989	C	0.5	0.5	A
13	BICF2S23131445	35364883	A	0	0	G
13	BICF2G630662456	35377104	A	0.5	0.5	G
13	BICF2G630662439	35391502	A	0	0	G
13	BICF2G630662432	35397676	G	0.5	0.5	A
13	TIGRP2P175337_rs8992944	35409275	0	0	0	G
13	BICF2G630662393	35422376	A	0	0.2857	G
13	BICF2P967691	35436453	G	0.5	0.5	A
13	BICF2P525480	35444639	G	0	0	A
13	BICF2S23258137	35457532	0	0	0	G
13	BICF2G630662356	35468959	A	0	0	G
13	BICF2G630662349	35480312	G	0	0	A
13	BICF2G630662339	35497407	G	0	0	C
13	BICF2G630662334	35503607	A	0	0	C
13	BICF2G630662319	35512197	G	0.5	0.5	A
13	BICF2G630662287	35533229	A	0	0	G
13	BICF2G630662279	35544858	A	0	0	G
13	BICF2G630662248	35558602	C	0.5	0.5	G
13	BICF2G630662215	35574059	G	0.5	0.5	A
13	BICF2G630662210	35582786	C	1	0.7143	G
13	BICF2G630662195	35598158	G	0	0	A
13	BICF2G630662178	35606276	A	0	0	G
13	BICF2G630662161	35618615	G	0	0.2857	A
13	BICF2P255873	35632103	0	0	0	C
13	BICF2S22921830	35649101	A	0	0.2857	C

13	BICF2S23447021	35652816	A	1	0.7143	G
13	BICF2G630662154	35692506	A	0	0.2857	G
13	BICF2G630662153	35702004	A	0.5	0.5	G
13	BICF2G630662141	35707965	A	0	0	G
13	BICF2P1306367	35723854	G	1	1	A
13	BICF2G630662104	35731309	C	0.5	0.5	A
13	BICF2P609231	35749318	G	0.5	0.5	A
13	BICF2G630662092	35760043	G	0.5	0.5	A
13	BICF2G630662090	35769715	A	0	0	C
13	BICF2S23513096	35776912	A	0	0	C
13	BICF2G630662079	35788226	A	0	0	G
13	BICF2G630662049	35804493	C	0	0	A
13	BICF2G630662041	35811476	G	0	0	A
13	BICF2P440683	35824594	G	0	0	A
13	BICF2G630662003	35837969	G	0	0.2857	A
13	BICF2G630662002	35838008	C	0	0.2857	A
13	BICF2G630661962	35868275	A	0.5	0.5	G
13	BICF2G630661960	35871175	A	0.5	0.5	G
13	BICF2P879840	35883430	G	0.5	0.5	A
13	BICF2P1367785	35891679	A	0.5	0.5	G
13	BICF2G630661912	35904840	A	0	0	C
13	BICF2G630661910	35917225	A	0	0	G
13	BICF2P296831	35966843	G	0.5	0.7857	A
13	BICF2P728019	35979311	0	0	0	A
13	BICF2G630661904	35987986	G	0	0.2857	A
13	BICF2G630661867	36006308	A	0.5	0.5	C
13	BICF2G630661864	36008436	A	0.5	0.5	G
13	BICF2G630661852	36021194	C	1	1	G
13	BICF2P295107	36034328	C	0.5	0.5	G
13	BICF2G630661804	36042761	G	0.5	0.7857	A
13	BICF2P442240	36074552	G	0.5	0.2143	A
13	BICF2P883484	36084406	A	0	0	G
13	BICF2P1228979	36090993	0	0	0	A
13	BICF2P290462	36105682	G	0	0	A
13	BICF2P228373	36111764	G	0	0	A
13	BICF2P833565	36125777	G	0	0	A
13	BICF2G630661793	36135980	A	0.5	0.2143	G
13	BICF2G630661779	36146604	A	0.5	0.2143	G
13	BICF2G630661758	36175772	0	0	0	A
13	BICF2G630661743	36185570	A	0.5	0.2143	G
13	BICF2P897231	36195720	G	0.5	0.7857	A
13	BICF2P195478	36204683	A	0	0.2857	G
13	BICF2P226053	36233682	G	0.5	0.2143	A
13	BICF2P274273	36237360	G	0.5	0.2143	A
13	BICF2P1193484	36250566	A	0.5	0.2143	G
13	BICF2G630661722	36265969	A	0.5	0.7857	C
13	BICF2S232722	36274563	A	0	0	G

13	BICF2G630661687	36280547	0	0	0	C
13	BICF2P303573	36287320	T	0.5	0.7857	A
13	BICF2G630661675	36295134	G	0.5	0.2143	A
13	BICF2G630661658	36305562	A	0.5	0.7857	G
13	BICF2G630661629	36317116	A	0.5	0.7857	C
13	BICF2G630661623	36328548	A	1	1	C
13	BICF2P1087868	36350761	0	0	0	G
13	BICF2G630661615	36351723	A	0	0	G
13	BICF2G630661611	36364067	G	0.5	0.2143	A
13	BICF2S2412368	36395489	0	0	0	G
13	BICF2S22952347	36409223	G	0	0	A
13	BICF2S23617423	36424219	A	0	0	G
13	BICF2P236412	36454003	0	0	0	A
13	BICF2G630661603	36459764	A	0.5	0.2143	G
13	BICF2G630661600	36460927	A	0.5	0.2143	G
13	BICF2G630661573	36471804	G	0.5	0.2143	A
13	BICF2G630661562	36477966	A	0.5	0.2143	G
13	BICF2G630661547	36484636	A	0.5	0.2143	G
13	BICF2G630661544	36486080	A	0.5	0.7857	G
13	BICF2P1314451	36495331	G	0.5	0.8333	A
13	BICF2G630661513	36495894	A	0.5	0.2143	G
13	BICF2G630661494	36504965	A	0.5	0.2143	G
13	BICF2P183301	36516145	A	0.5	0.8333	G
13	BICF2S23224021	36525333	A	0	0	G
13	BICF2G630661441	36538471	A	0	0	G
13	BICF2G630661436	36540261	T	0	0	A
13	BICF2P821881	36557919	G	0.5	0.7857	A
13	BICF2P678186	36564029	C	0.5	0.7857	A
13	BICF2P1391842	36568861	A	0.5	0.7857	G
13	TIGRP2P175493_rs8796102	36571689	G	0	0	A
13	BICF2G630661410	36589236	T	0	0	A
13	BICF2G630661387	36604097	C	0	0	G
13	BICF2P1076212	36605766	A	0	0	G
13	BICF2P610951	36626568	A	0	0	G
13	BICF2P610957	36628017	A	0	0	T
13	BICF2S23115607	36638416	T	0	0	A
13	TIGRP2P175505_rs8841289	36643754	A	0	0	C
13	BICF2P587027	36658305	A	0	0	G
13	BICF2P591645	36669119	A	0	0	G
13	BICF2S22912678	36682866	G	0	0	A
13	TIGRP2P175538_rs8610013	36704018	A	0	0	G
13	BICF2P707278	36718259	G	0.5	0.7857	A
13	BICF2P707280	36718848	A	0.5	0.7857	G
13	BICF2P584495	36721024	A	0.5	0.7857	G
13	BICF2P278261	36739928	G	1	1	A
13	BICF2P1036879	36740681	A	1	1	G
13	BICF2P750280	36744716	A	1	1	G

13	BICF2S23320902	36745997	G	0	0	A
13	BICF2P169606	36756880	0	0	0	A
13	BICF2P899584	36767823	A	0	0	G
13	BICF2P94124	36785376	G	0.5	0.7857	A
13	BICF2P1046287	36789344	A	0.5	0.2143	C
13	BICF2P257297	36791189	G	0	0	A
13	TIGRP2P175574_rs9100767	36806148	G	0.5	0.7857	A
13	BICF2P483867	36809937	A	0	0	G
13	TIGRP2P175579_rs9160073	36818969	A	0.5	0.7857	G
13	BICF2P984683	36822948	G	0.5	0.7857	A
13	BICF2P27839	36833452	A	1	1	G
13	BICF2S23139010	36837202	A	0	0	G
13	BICF2S23444009	36861970	A	0.5	0.7857	G
13	BICF2S23444008	36861995	A	0	0	G
13	BICF2S23153546	36875646	G	0.5	0.7857	A
13	BICF2G630661377	36884560	G	0.5	0.7857	A
13	BICF2S23254204	36895625	A	0	0	G
13	BICF2G630661376	36910080	0	0	0	A
13	BICF2S23616468	36919279	A	0	0	G
13	TIGRP2P175612_rs8946140	36931977	G	0.5	0.7857	A
13	TIGRP2P175618_rs9223433	36938098	G	0.5	0.7857	A
13	BICF2S23013138	36951495	A	0	0	G
13	BICF2P292633	36968445	A	0.5	0.7857	C
13	BICF2P945029	36983595	G	0.5	0.7857	A
13	BICF2S23613461	36987144	0	0	0	A
13	BICF2P351987	36998441	A	0.5	0.5	G
13	BICF2P1205758	37008721	G	0	0	A
13	BICF2P1365771	37020413	A	0	0.2857	G
13	BICF2P448317	37034530	G	0	0.2857	A
13	TIGRP2P175651_rs9000784	37043193	G	0	0	A
13	TIGRP2P175657_rs9203624	37054931	G	0	0.2857	A
13	BICF2P513213	37074905	G	0	0.2857	A
13	BICF2P1050635	37086961	G	0.5	0.8333	A
13	BICF2P1389335	37094234	C	0	0	A
13	BICF2P1389342	37099592	G	0	0.2857	A
13	BICF2G630661374	37121002	A	1	1	C
13	BICF2G630661350	37131760	0	0	0	G
13	BICF2G630661346	37134346	C	1	1	A
13	BICF2G630661330	37155827	A	0	0	G
13	BICF2P1022948	37165132	A	0	0	G
13	TIGRP2P175706_rs8776238	37169637	A	0	0	G
13	BICF2P1022924	37169994	C	0	0	A
13	BICF2G630661293	37180519	A	1	1	G
13	BICF2G630661292	37180570	A	1	1	G
13	BICF2G630661232	37194215	C	0	0	A
13	BICF2P1218173	37196297	G	0	0	A
13	BICF2G630661200	37206165	G	1	1	A

13	BICF2P456452	37222648	G	1	0.7143	A
13	BICF2G630661166	37225658	G	1	1	A
13	BICF2G630661155	37241373	A	1	0.7143	G
13	BICF2G630661149	37254180	C	0	0	A
13	BICF2S23323724	37266393	A	0	0	G
13	BICF2G630661131	37277358	A	0	0	G
13	BICF2P192527	37289290	G	0	0	C
13	BICF2P213659	37327156	A	0	0	G
13	BICF2P644845	37339875	A	0	0	G
13	BICF2P1435078	37348342	G	0	0	C
13	BICF2G630661123	37352436	G	0	0.2857	A
[truncated: 713,266 more chars]
